# Mycobacteriaceae Phenome Atlas (MPA): A Standardized Atlas for the Mycobacteriaceae Phenome Based on Heterogeneous Sources

**DOI:** 10.1007/s43657-023-00101-5

**Published:** 2023-06-13

**Authors:** Wan Liu, Hui Cen, Zhile Wu, Haokui Zhou, Shuo Chen, Xilan Yang, Guoping Zhao, Guoqing Zhang

**Affiliations:** 1 National Genomics Data Center & Bio-Med Big Data Center, CAS Key Laboratory of Computational Biology, Shanghai Institute of Nutrition and Health, University of Chinese Academy of Sciences, Chinese Academy of Sciences, Shanghai, 200031 China; 2 Shanghai Southgene Technology Co., Ltd., Shanghai, 201210 China; 3 Institute of Synthetic Biology, Shenzhen Institutes of Advanced Technology, Chinese Academy of Sciences, Shenzhen, 518055 China; 4 Hangzhou Institute for Advanced Study, University of Chinese Academy of Sciences, Hangzhou, 310024 China

**Keywords:** Mycobacteriaceae phenome, Data elements, Co-evolution, Pathogenicity

## Abstract

The bacterial family Mycobacteriaceae includes pathogenic and nonpathogenic bacteria, and systematic research on their genome and phenome can give comprehensive perspectives for exploring their disease mechanism. In this study, the phenotypes of Mycobacteriaceae were inferred from available phenomic data, and 82 microbial phenotypic traits were recruited as data elements of the microbial phenome. This Mycobacteriaceae phenome contains five categories and 20 subcategories of polyphasic phenotypes, and three categories and eight subcategories of functional phenotypes, all of which are complementary to the existing data standards of microbial phenotypes. The phenomic data of Mycobacteriaceae strains were compiled by literature mining, third-party database integration, and bioinformatics annotation. The phenotypes were searchable and comparable from the website of the Mycobacteriaceae Phenome Atlas (MPA, https://www.biosino.org/mpa/). A topological data analysis of MPA revealed the co-evolution between *Mycobacterium tuberculosis* and virulence factors, and uncovered potential pathogenicity-associated phenotypes. Two hundred and sixty potential pathogen-enriched pathways were found by Fisher's exact test. The application of MPA may provide novel insights into the pathogenicity mechanism and antimicrobial targets of Mycobacteriaceae.

**Supplementary Information:**

The online version contains supplementary material available at 10.1007/s43657-023-00101-5.

## Introduction

Phenotypes are the observable characteristics of an organism resulting from the expression of a particular genotype in a specific environment (Chibucos et al. 2014). "The phenome is a set of measurable traits, including the physical, chemical, and biological traits of individuals and populations, which result from the complex interactions of genes, epigenetics, symbiotic microorganisms, diet, and environmental exposures" (Jin 2021). Microbial phenotypes—such as physiological, biochemical, and culture phenotypes—are widely used in the taxonomy and identification of microorganisms (Rastogi et al. 2001) and are currently used as data elements in polyphasic taxonomy or ontology (Vandamme et al. 1996; Uilenberg and Goff 2006; Siegele et al. 2019).

Polyphasic taxonomy is widely used to identify microbes according to the perspective of taxonomic classification (Colwell 1970). With the rapid development of genome sequencing techniques and bioinformatics tools, the microbial characteristics were beyond the data elements in polyphasic taxonomy. In recent years, more and more microbial molecular traits have been used to characterize microbes, such as virulence factors (VFs), antimicrobial resistance (AMR), and pathogenicity (Beceiro et al. 2013; Nüesch-Inderbinen et al. 2019). Moreover, the reconstruction of bacterial and archaeal genomes from the metagenomes of environmental and host-associated samples became another source of new microbes (Nayfach et al. 2021), which can be described by predicting molecular function information and sampling information. It is difficult to use polyphasic taxonomy to describe increasing molecular-level phenotypes. Going beyond partial phenotypes involved in polyphasic taxonomy, the Ontology of Microbial Phenotypes (OMP) database includes additional phenotypes surrounding microbe–host interactions and molecular-level regulation, and the terms in the OMP are continuously updated according to the OMP GitHub repository (Chibucos et al. 2014). However, we found that some common phenotypic data elements in "polyphasic taxonomy", such as isolation information, sampling information, environmental information, culture and growth information, safety information, and physiology and metabolism information, are currently missing in the OMP. Therefore, there are no applicable methods for depicting microbial phenotypes, and it is necessary to integrate and design more suitable data elements of microbial phenomes.

Mycobacteriaceae consists of numerous pathogenic mycobacteria, such as *Mycobacterium tuberculosis* (*M. tuberculosis*) and *Mycobacterium leprae*, and its taxonomy is controversial. *Mycobacterium* were re-divided into five genera in 2018 (Gupta et al. 2018). *Hoyosella* is classified as being in the Mycobacteriaceae family in the National Center for Biotechnology Information (NCBI) taxonomy (Federhen 2012), while it is classified as being in the Nocardiaceae family (Nouioui et al. 2018; Oren and Garrity 2019). *Bactoderma* and *Stibiobacter* are classified as Mycobacteriaceae in the List of Prokaryotic names with Standing in Nomenclature (LPSN) (Parte et al. 2020), and as Bacteria incertae sedis in the NCBI Taxonomy, and *Bactoderma* was classified into the Patulibacteraceae family by Salam et al. (2020). The proportion of drug-resistant strains in Mycobacteriaceae has gradually increased, which has become a major public health problem. The collection of Mycobacteriaceae phenotypes is helpful in the diagnosis and treatment of Mycobacteriaceae clinical infections. For example, "Fastness", an acid–alcohol fast staining phenotype, can be used as an auxiliary diagnosis of tuberculosis caused by *M. tuberculosis*, and the drug resistance phenotype can help physicians select appropriate treatment (Chevalier et al. 2014; Vilchèze et al. 2014). To gather the phenotypes of Mycobacteriaceae and help find potential pathogenicity-related risk factors, five genera classified into Mycobacteriaceae by both the NCBI Taxonomy and the LPSN were included in this study.

The currently available Mycobacteriaceae phenotypes are widely distributed, and there is only limited information about them (Supplementary Table 1). A few Mycobacteriaceae databases are species based and contain only partial phenotypes. Mycobrowser is a comprehensive genomic and proteomic data repository for pathogenic mycobacteria that provides manually curated annotations and other appropriate tools to facilitate the genomic and proteomic study of these organisms, but only includes 10 strains from nine *Mycobacterium* species, and does not include polyphasic phenotypes (Kapopoulou et al. 2011). MycoperonDB provides the operons and transcription units of five strains from four *Mycobacterium* species, but has not been updated since the first version released in 2006 (Ranjan et al. 2006). The *Mycobacterium tuberculosis* Pathway/Genome Database contains 51 genomes, their associated metabolic pathways, and predictions of missing enzymes and transcription units in these metabolic pathways, but only contains one species and does not provide polyphasic phenotypes (Midford et al. 2019). Furthermore, some databases provide polyphasic phenotypes, but not functional phenotypes. The Bacterial Diversity Metadatabase (BacDive) is a comprehensive repository of structured data on prokaryotic taxonomy, morphology, physiology, culture, and isolation, but only contains 196 species and seven subspecies of Mycobacteriaceae, and does not include functional phenotypes (Reimer et al. 2019, 2022). PathoSystems Resource Integration Center (PATRIC), maintained by the bacterial Bioinformatics Resource Center, provides a biological information analysis platform for all bacteria, and includes basic metadata regarding organism, host, motility, and cell shape, but contains only limited functional phenotypes, such as AMR, Gene Ontology (GO) terms, and VFs (Davis et al. 2020).

Considering the above points, multiscale phenotypes were gathered from macroscopic and microscopic traits, which were organized as the data elements of the Mycobacteriaceae phenome. Then, the Mycobacteriaceae Phenome Atlas (MPA) was developed based on these data, which included the curated and annotated phenotypic data of 10,755 strains from 236 species and 18 subspecies in Mycobacteriaceae. In addition, MPA's web server provides a user-friendly interface to search and compare the integrated phenome of all Mycobacteriaceae strains, and it is freely accessible at https://www.biosino.org/mpa/. The co-evolution of *M. tuberculosis* with VFs and the investigation of pathogen-enriched pathways might provide clues to the molecular mechanism of Mycobacteriaceae pathogenicity and aid in the study of the potential targets for antimicrobial drugs.

## Materials and Methods

**Fig. 1:**
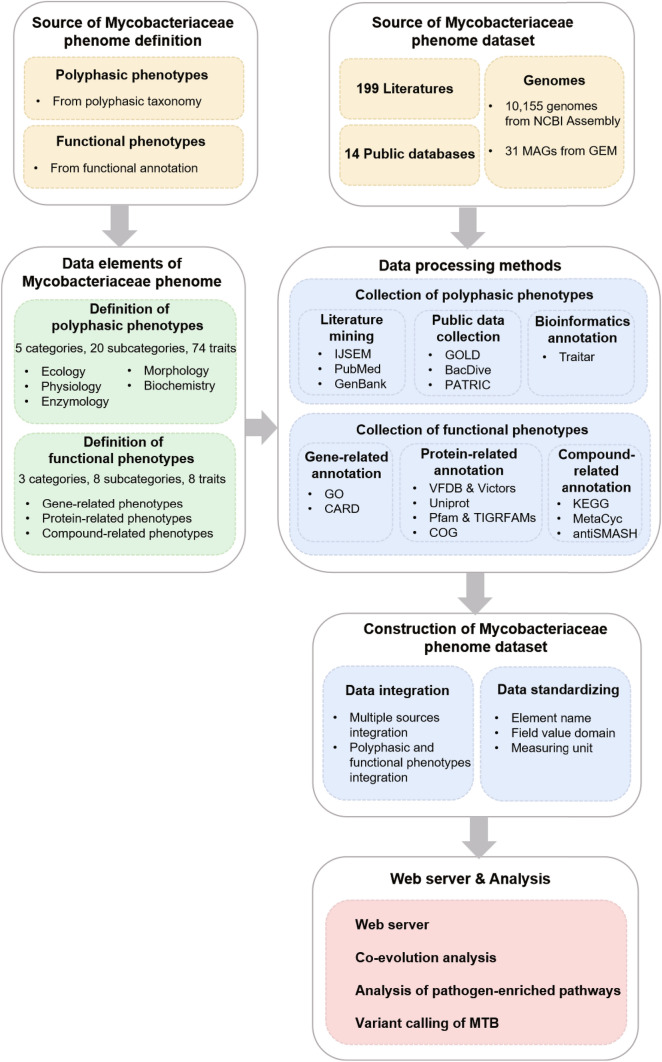
Pictorial methodology for developing the MPA. Data sources for Mycobacteriaceae phenome definition and dataset construction are indicated in yellow. Data elements of Mycobacteriaceae phenome are in green. How the phenotypic dataset was processed and integrated is depicted in blue. Mycobacteriaceae dataset was accessed through the web interface and used for correlated analysis, which is shown in red

Pictorial methodology for developing the MPA is displayed in Fig. 1.

### Genome Investigation

A total of 10,595 Mycobacteriaceae genome records were downloaded from the NCBI Assembly database (Kitts et al. 2016) (up to November 9, 2021). From these, 287 records missing strain names were discarded, and 144 records were de-duplicated (when there was more than one genome in one strain), thus retaining 10,164 genome records after our quality control. If a strain had more than one genome record in the NCBI Assembly database, then redundant genome records were discarded according to the following standards. First, genomes with the highest level of assembly were selected, and the order of assembly level from highest to lowest is complete genome, chromosome, scaffold, and contig according to NCBI Assembly (https://www.ncbi.nlm.nih.gov/assembly/help/). Second, if more than one genome for a strain in the highest assembly level was available, then the genome with the earliest released date was selected. Species and genus names were standardized based on the 2018 Mycobacteriaceae classification (Gupta et al. 2018).

In total, 380 Mycobacteriaceae metagenome-assembled genomes (MAGs) were collected from the 52,515 MAGs of Genomes from Earth's Microbiomes (GEM) (Nayfach et al. 2021), and 86 MAGs were retained after de-replication with dRep (version 3.2.0, parameters: dereplicate -p 12 -comp 50 -con 5 -pa 0.9 -sa 0.95) (Olm et al. 2017).

Keeping in mind Mycobacteriaceae's taxonomic controversy, five genera of Mycobacteriaceae were involved in this study, including *Mycobacterium*, *Mycobacteroides*, *Mycolicibacillus*, *Mycolicibacter*, and *Mycolicibacterium* (Gupta et al. 2018; Gupta 2019). Seven of the assembled genomes and 55 MAGs in the nonredundant MAGs that did not belong to these five genera were removed, and thus 10,158 genomes and 31 MAGs were used in the following procedures (Supplementary Table 2).

### Variant Calling of *M. tuberculosis* in MPA

Six thousand nine hundred and eighty-two *M. tuberculosis* strains were obtained from the MPA database. Minimap2 (v2.24-r1122, default parameters) was used to compare the above strains by using *M. tuberculosis* H37Rv (GenBank assembly accession: GCA_000195955.2) as a reference genome (Li 2018). SAMtools (v1.9) and BCFtools (v1.8) were used to call single-nucleotide polymorphisms and small indels (Danecek et al. 2021).

### Polyphasic Phenotypes Curation and Prediction

In this study, microbial characteristics derived from "polyphasic taxonomy" are curated as polyphasic phenotypes, including qualitative or even quantitative observations, measurements, or experimental test results. The polyphasic phenotypes were extracted by literature mining, third-party database integration, and bioinformatics annotation.

**Fig. 2:**
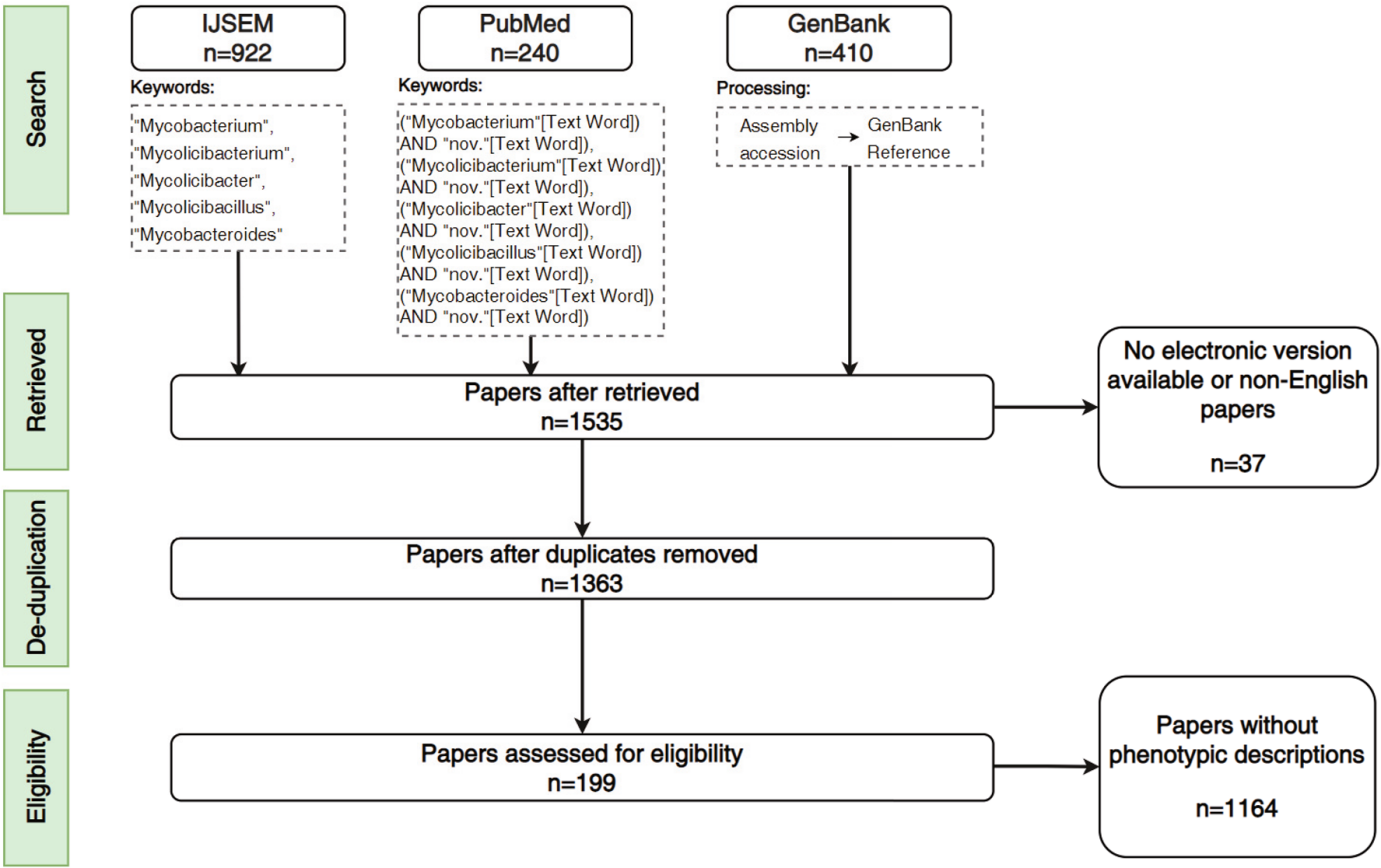
The workflow of the literature recruitment for manual curation. A total of 922, 240, and 410 records were obtained from IJSEM, PubMed, and GenBank, respectively. After excluding 37 non-English papers and those without electronic versions, 1535 papers were retrieved and 1363 papers were retained for review after de-duplication, of which 199 papers had phenotypic descriptions and were used for further manual curation

The literature sources included PubMed, *International Journal of Systematic and Evolutionary Microbiology* (IJSEM), and the references of Genetic Sequence Data Bank (GenBank) records (Sayers et al. 2021). The recruitment procedure is shown in Fig. 2. According to International Code of Nomenclature of Prokaryotes, a new taxon should be published with the "nov." abbreviation in its name, along with a description of the taxon (i.e., the polyphasic phenotypes we defined) (Parker et al. 2019). A total of 223 articles were retrieved from PubMed (up to November 2021) with the query "(((((Mycobacterium [Text Word]) OR (Mycobacteroides [Text Word])) OR (Mycolicibacillus [Text Word])) OR (Mycolicibacter [Text Word])) OR (Mycolicibacterium [Text Word])) AND (nov. [Text Word])", which was aimed to obtain relevant literature related to the type strain of each species in Mycobacteriaceae. IJSEM recorded the publications of novel microbial taxa, and 910 papers were retrieved from it (up to November 2021) by searching the name of the five Mycobacteriaceae genera. In addition, 402 papers were retrieved from the references of Mycobacteriaceae genome records in GenBank (up to November 2021). In total, 199 papers with phenotypic descriptions from a total of 1363 nonredundant papers were used, and 13,667 phenotypes from 799 strains were mined by manual curation.

The second source of polyphasic phenotypes was integrated from Genomes OnLine Database (GOLD) of the Joint Genome Institute (JGI), BacDive, and PATRIC (Davis et al. 2020; Mukherjee et al. 2021; Reimer et al. 2019, 2022). Biome information (such as ecosystem, ecosystem category, ecosystem type, ecosystem subtype, and specific ecosystem), which is otherwise known as isolation information of microbes, was integrated into the MPA from the GOLD database. Pathogenicity information of Mycobacteriaceae was integrated into the MPA from GOLD, BacDive, and PRTRIC.

Polyphasic phenotypes can be predicted by Traitar (V1.1.2) software, which is a bioinformatics tool for characterizing microbial phenotypes based on nucleotide or protein sequences (Weimann et al. 2016). All retained genomes from the NCBI Assembly database, as well as the generated MAGs from GEM, were used as the input to Traitar prediction. Three genomes (GenBank assembly accession: GCA_001318645.1, GCA_001199935.1, and GCA_001144025.1) did not have results by Traitar prediction.

### Functional Phenotypes Annotation

In this study, microbial molecular characteristics derived from genome functional annotation are denoted as functional phenotypes, which tend to be functional descriptions with qualitative high-throughput prediction. Functional phenotypes were divided into three categories: gene-related phenotypes, protein-related phenotypes, and compound-related phenotypes. The functional phenotypes, which were different from the phenotypes obtained from experimental tests, such as physiological, biochemical, and enzymatic phenotypes, were annotated using bioinformatics tools.

Gene-related phenotypes included GO annotations and AMR. The GO annotations were predicted by Blast2GO (V2.2.28+) (Conesa and Götz 2008; Carbon et al. 2019), with an *e* value of 1 × 10^–3^. The AMR genes were predicted using the resistance gene identifier (RGI, V5.0.0) of the Comprehensive Antibiotic Resistance Database (CARD) with default parameters (Alcock et al. 2020).

Protein-related phenotypes contained VFs, amino acid mutations, and orthologous groups. We integrated Virulence Factor Database (VFDB) (Liu et al. 2019) and Victors database (Sayers et al. 2019) to generate a new reference dataset of VFs, which included 6,313 nonredundant sequences. VFs were predicted based on the generated reference dataset of VFs using Blast2GO (V2.2.28+), with an *e* value of 1 × 10^–3^. Amino acid mutations were annotated by the UniProt Swiss-Prot database using double index alignment of next-generation sequencing data (DIAMOND) (V0.9.9) (Buchfink et al. 2014; Bateman et al. 2021), with an *e* value of 1 × 10^–3^. Orthologous groups were annotated by clusters of orthologous groups of proteins (COG) using DIAMOND, with an *e* value of 1 × 10^–3^, and the protein names from UniProt Swiss-Prot in orthologous groups were further annotated by DIAMOND using the same parameters (Buchfink et al. 2014; Bateman et al. 2021; Galperin et al. 2021). Protein domains were annotated by protein family (Pfam) and the Institute for Genomic Research (TIGR) Functional Analysis of Genomes (TIGRFAMs) using HMMER (V3.1b2) (Haft et al. 2013; Mistry et al. 2013; El-Gebali et al. 2019), with an *e* value of 1 × 10^–5^.

Compound-related phenotypes included Kyoto Encyclopedia of Genes and Genomes (KEGG) metabolites, MetaCyc metabolites, and secondary metabolite biosynthetic gene clusters (smBGCs). Protein sequences from each genome were annotated by DIAMOND, with an *e* value of 1 × 10^–3^ using all protein sequences from KEGG GENES database and MetaCyc as the reference database, respectively. KEGG pathways were further retrieved by the KEGG API (http://rest.kegg.jp/link/pathway/ko), and the relationship between the protein sequence from MetaCyc and MetaCyc pathway was constructed by using proteins.dat and reactions.dat downloaded from MetaCyc (Buchfink et al. 2014; Kanehisa et al. 2019; Caspi et al. 2020). The metabolites involved in these pathways were collected as either KEGG metabolites or MetaCyc metabolites. AntiSMASH (V5.1.2 with default parameters) was used to annotate and predict smBGCs (Blin et al. 2019).

### Data Integration and Database Construction

Polyphasic phenotypes curated from the scientific literatures were standardized and integrated, and the DOIs of the curated literature was provided for their fast tracking. If a polyphasic phenotype was from multiple sources, such as the scientific literature and Traitar prediction, then the phenotypic data with a higher confidence source was retained, with literature preferred over the prediction. Polyphasic and functional phenotypes were linked by the strain name in MPA.

The MPA's data were deposited in the MongoDB database using Extract–Transform–Load scripts. We constructed a user-friendly web interface with HTML, CSS, and JavaScript, and JQuery (https://jquery.com/) was implemented to achieve front-end user interaction with SpringBoot framework (https://projects.spring.io/spring-boot/) for the server backend development. The charts were drawn with Apache ECharts (https://echarts.apache.org), and the web server was hosted on an in-house server.

### Co-evolution Analysis of Microbial Phenomes Using Topological Data Analysis

The co-evolution between phenotypes and microbial genomes can be studied by topological data analysis (TDA) using *tmap,* which is an integrative framework for population-scale microbiome stratification and association (Liao et al. 2019). Microbial phenotypes of VFs and genomes of Mycobacteriaceae species with > 90% completeness and < 5% contamination calculated by CheckM (v1.1.2) via the lineage-specific workflow (Parks et al. 2015) were extracted from MPA for subsequent tmap analysis. The genomes of 9979 strains among all records of Mycobacteriaceae qualified for use in this study. As some strains are missing VFs phenotypes, only 9854 strains could be used to establish the pairwise genome distance matrix by Mash (v1.1 with default parameters) analysis (Ondov et al. 2016).

The distance matrix was used to generate a TDA network by *tmap* (parameters: min_samples = 3, resolution = 35, overlap = 1, eps threshold = 98, filter = UMAP), which consists of 9524 strains, 1069 nodes, and 5411 edges (Liao et al. 2019; McInnes et al. 2020). The nodes with no significant strains of each feature were filtered out by requiring a spatial analysis of functional enrichment (SAFE) score ≥ 0.5, which corresponds to a negative log-transformed *p* value of 0.05, after 1000 iterations by the SAFE algorithm.

The co-evolution was detected by the similarity of TDA network enrichment patterns with SAFE co-enriched scores > 0 between two target traits. As *M. tuberculosis* is one of the most well-studied pathogenic Mycobacteriaceae (Chevalier et al. 2014; Gagneux 2018; Koch and Mizrahi 2018), co-evolution between VFs and *M. tuberculosis* was analyzed in depth.

### Analysis of Pathogen-Enriched Pathways by Fisher's Exact Test

Fisher's exact test was carried out to investigate pathogen-enriched pathways in 533 strains with pathogenicity information. We counted pathways in each pathogen and non-pathogen Mycobacteriaceae strain and used a one-sided (greater) Fisher's test with Benjamini–Hochberg correction to determine pathogen-enriched pathways (adjusted *p*-value < 0.05).

## Results

### Data Elements of the Mycobacteriaceae Phenome

**Fig. 3:**
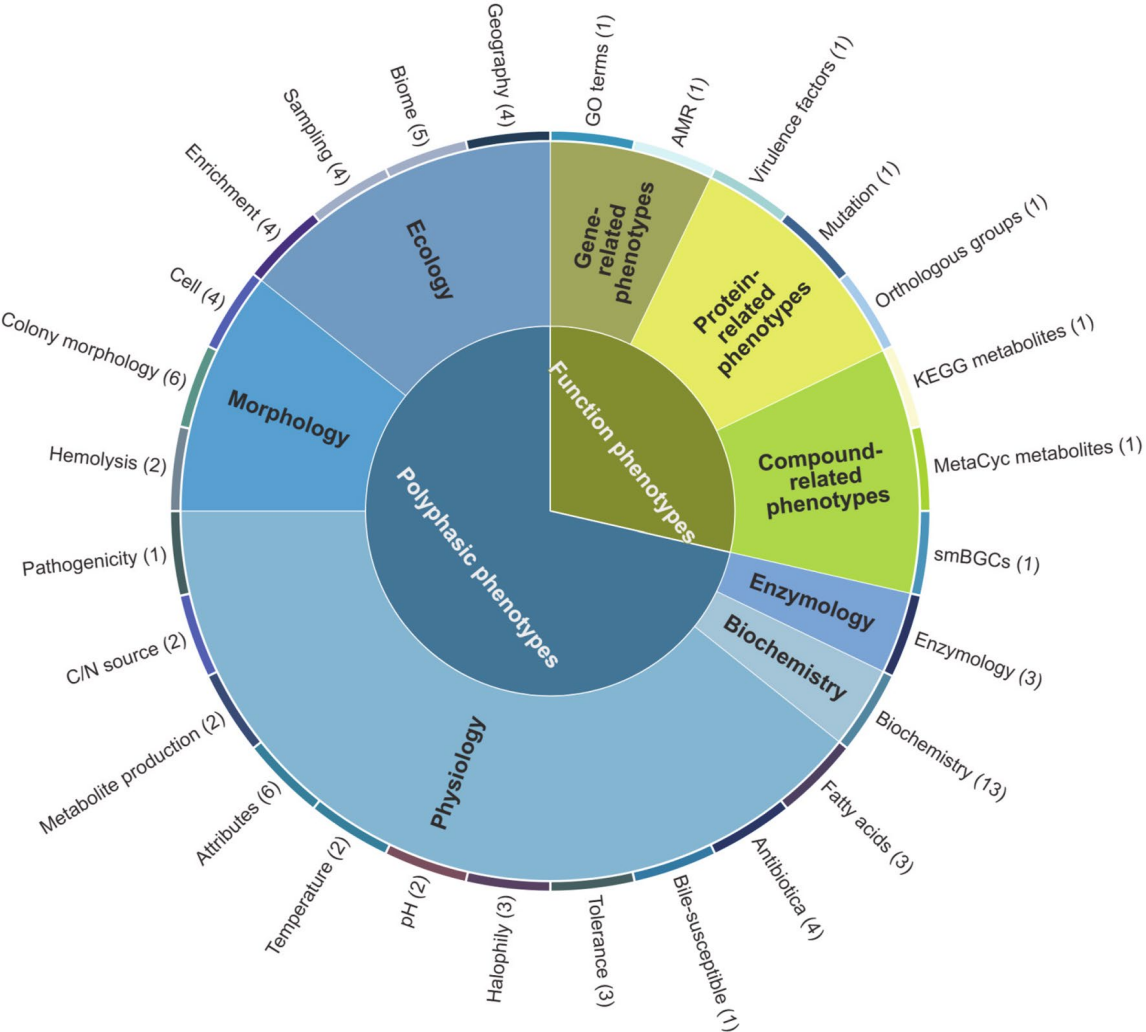
Summary of data elements in MPA. Levels I and II indicate the name of categories, and Level III refers to the name of 28 subcategories and the number of embodied phenotypes. Level I includes "Polyphasic phenotypes" and "Functional phenotypes". Level II contains "Ecology", "Morphology", "Physiology", "Biochemistry", "Enzymology", "Gene-related phenotypes", "Protein-related phenotypes", and "Compound-related phenotypes". Level III consists of "Geography", "Biome", "Sampling", "Enrichment", "Cell", "Colony morphology", "Hemolysis", "Pathogenicity", "C/N source", "Metabolite Production", "Attributes", "Temperature", "pH", "Halophily", "Tolerance", "Bile-susceptible", "Antibiotica", "Fatty acids", "Biochemistry", "Enzymology", "GO terms", "AMR", "Virulence factors", "Amino acid mutations", "Orthologous groups", "KEGG metabolites", "MetaCyc metabolites", and "smBGCs"

In all, 82 microbial phenotypic traits were recruited as data elements for the Mycobacteriaceae phenome, which are shown in Fig. 3, and a detailed list can be found in Supplementary Table 3. The polyphasic phenotypes included 74 traits that can be summarized into five categories and 20 subcategories, which are shown in Fig. 3. The functional phenotypes included eight traits and can be summarized into three categories: gene related, protein related, and compound related. Every data element can be uniquely identified by name and field value domain. The element name, field value domain, and measuring unit were standardized.

We compared all 82 phenotypic traits in the MPA with microbial phenotypes in BacDive and OMP, which are listed in Supplementary Table 3 and 4. There are 151 phenotypic fields in BacDive, and a total of 65 phenotypic names in MPA matched 72 traits in BacDive. For example, the two fields of "Cell length" and "Cell length unit" in BacDive both match MPA's "Cell length" field, because we combined "Cell length" and "Cell length unit" as one field in MPA. Some phenotypes that were only present in BacDive were not applied to Mycobacteriaceae, such as flagella phenotypes. Some embodied phenotypes in BacDive were raw experimental results and could be cross-linked by the MPA. As OMP is a typical ontology designed for computational science with lots of conceptual terms, there are 2059 terms in 24 subcategories in OMP (up to May 7th, 2021), and some terms are very detailed and in the value domains of MPA. For example, the phenotypes of cell shape could be mapped to many terms in OMP, such as OMP:0000086 spiral cell shape, OMP:0000123 coccobacillus cell shape, and OMP:0000128 stalked cell shape. Thus, 22 traits and related value domains of MPA are compatible with 905 terms and 51.3% of 14 subcategories (1762 terms) in the OMP. The other phenotypes described in OMP were in much more detail compared with the data elements in MPA. Worth mentioning in this study are 11 unique phenotypes collected by the MPA and included neither in BacDive or OMP: "Biome" (including "Ecosystem", "Ecosystem category", "Ecosystem type", "Ecosystem subtype", and "Specific ecosystem"), "Minimum inhibitory concentration", "Pyrolysis esters", "Virulence factors", "Amino acid mutations", "Orthologous groups", and "smBGCs". "Biome" is in a hierarchical structure for the structure and functioning of the ecosystem where the microbe was sampled. "Minimum inhibitory concentration" is an indicator for the correlation between susceptibility testing and clinical outcomes for drugs (Schön and Chryssanthou 2017). "Pyrolysis esters" is an important element for mycobacterium classification (Kazda et al. 1993). "Virulence factors" is used to indicate the potential virulence of the microbe. "Amino acid mutations" is used to describe amino acid variations, and may be helpful to the study of microbe evolution and the biosynthetic reconstruction of the microbe. "Orthologous groups" is a phylogenetic classification of proteins encoded by complete genomes, and could be used to find the proteins with similar functions from diverse genomes. "smBGCs" is used to describe the potential natural products produced by the microbe. These newly microbial phenotypes are valuable complements to the existing microbial phenotypes in BacDive and OMP.

The field value domains of phenotypes were standardized and structuralized by third-party vocabularies or by MPA. Some phenotypic values were standardized by third-party vocabularies; for example, the values of "Host disease", "Enzyme", "Compound", and "Country" were standardized by the Disease Ontology (Bello et al. 2018), ENZYME (Bairoch 2000), Public Chemical Database (PubChem) (Kim et al. 2021), and M49 standards (https://unstats.un.org/unsd/methodology/m49/), respectively, and the first three can be cross-linked to related databases. Some phenotypes were standardized by ordinal or unordered categorical variables. For example, "Growth rate" was described as "fast growing", "grow rapidly", "rapidly growing", "slowly growing", "grow slowly", "grows slowly", or "slow growing"; these were standardized in the MPA as "rapidly growing" or "slowly growing". Some field value units of measurable phenotypes were also unified, such as "Cell length", "Cell width", "Cell diameter", "Colony size", "Tolerance concentration", and "Optimum temperature". For example, "Tolerance concentration" is the concentration of compound that the microorganism can withstand or endure; the unit was "μg/mL", "mg/L", or "mg/mL" in the original text, and was standardized as "mg/mL" in the MPA for better comparison between Mycobacteriaceae strains.

### MPA Phenomic Data

**Fig. 4:**
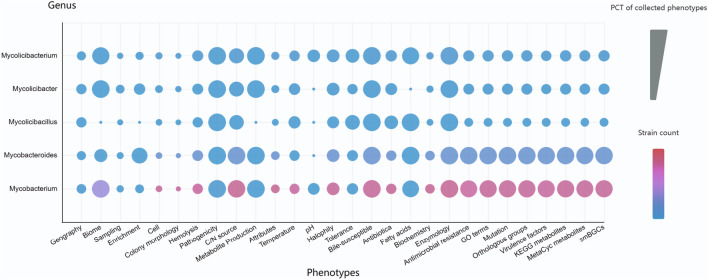
Phenomic distribution in five Mycobacteriaceae genera. The bubble size indicates the number of strains in the selected genus, and the shade indicates the number of phenotypes in the selected genus and phenotypic subcategory. Percentage (PCT) of collected phenotypes is the count of phenotypes collected by each category in a genus divided by the number of phenotypes expected to be collected in this genus. For example, in the category of Geography, each strain is expected to collect four phenotypes, including Continent, Country/Region, Geographic location, and Geographic coordinate. The number of Mycobacterium strains in the category of Geography is 107, and the count of phenotypes collected by these 107 strains is 194; thus, PCT of phenotypes collected by Geography in *Mycobacterium* is 0.45 (194/4*107)

The MPA collected more than 90 million phenotypes from 82 phenomic data elements in collected Mycobacteriaceae strains. Among the five Mycobacteriaceae genera, *Mycobacterium* includes the most strains (Fig. 4), having 8083 of them, of which 6990 belong to *M. tuberculosis.* This might be because *M. tuberculosis* is a pathogenic Mycobacteriaceae that causes an estimated 10.4 million new cases and 1.7 million deaths per year (Gagneux 2018), and it has been widely isolated and sequenced. *Mycobacteroides* includes the second most common strains, which is due to the fact that *Mycobacteroides* is associated with lung, skin, and soft tissue infections (Gupta et al. 2018).

**Fig. 5:**
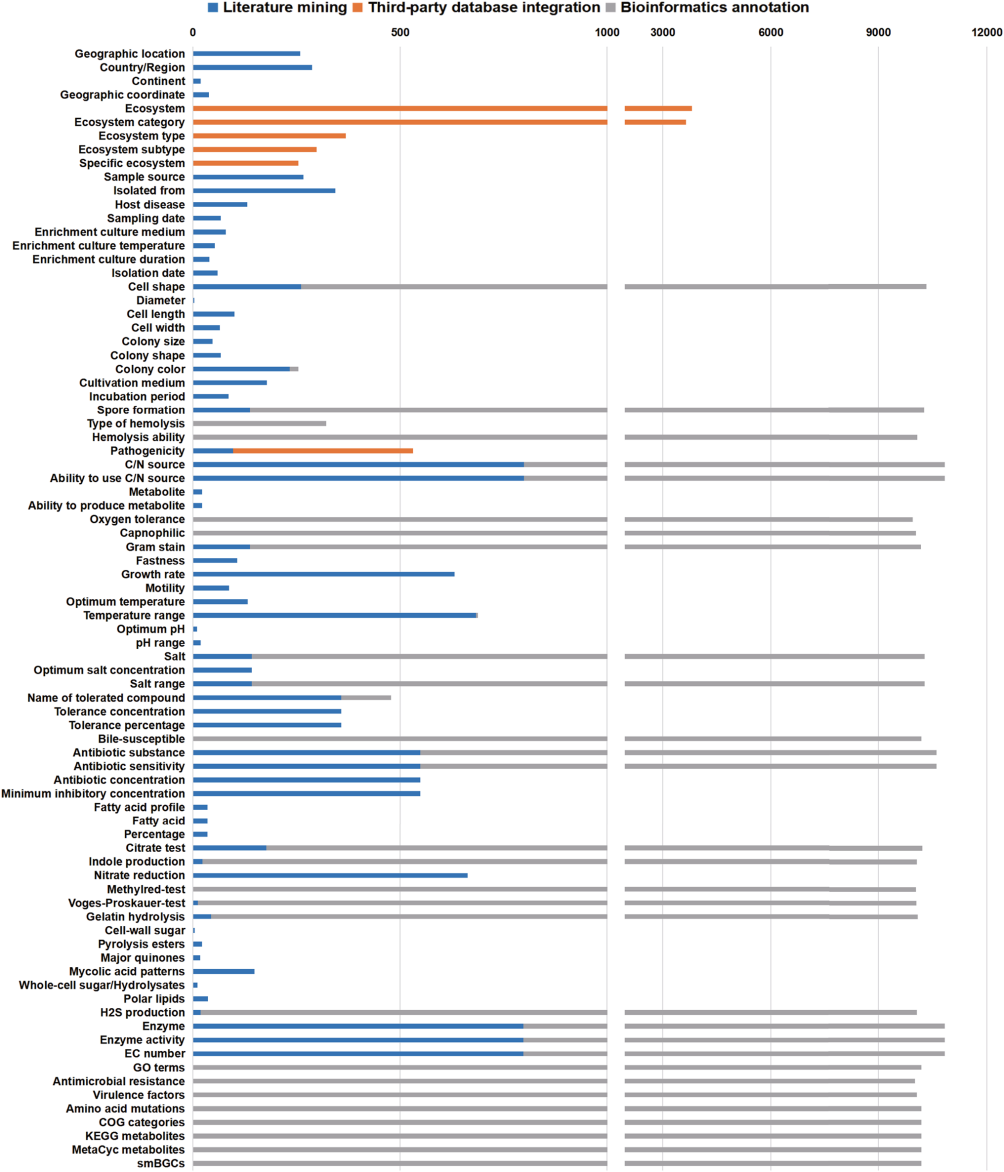
Contributions of heterogeneous data sources to traits in MPA. The *y*-axis indicates 82 phenotypic names of the Mycobacteriaceae phenome. Blue, orange, and gray columns refer to the number of strains from literature mining, third-party database integration, and bioinformatics annotation, respectively

The polyphasic phenotypes in the MPA were collected using three methods (Fig. 5). First, 64 polyphasic phenotypes from 799 strains were collected from the literature, although the curated traits were limited. For example, for "Growth rate", 323 (3.0%) strains were rapidly growing, while 227 (2.1%) strains were slowly growing. However, some pathogeny-related phenotypes were exclusively curated from articles. For example, mycolic acid is a component of the cell wall, which increases the infection potential for the host (Ghazaei 2018), and 123 (1.1%) strains contain the mycolic acid. Fastness phenotype is one of the auxiliary diagnosis of tuberculosis (Chevalier et al. 2014; Vilchèze et al. 2014), and 113 (1.0%) strains are acid fast or acid–alcohol fast, a finding consistent with previous studies (Goodfellow et al. 2012). Phenotypes coverage by literature mining are listed in Supplementary Table 5. Second, as the GOLD is a resource regarding genome and metagenome sequencing projects and their associated metadata, 8362 biome phenotypes from 3801 (35.3%) strains were retrieved from GOLD to help understand the isolation information of the microbes. The pathogenicity information of 436 strains was integrated into the MPA from GOLD, BacDive, and PRTRIC. Finally, 26 polyphasic phenotypes from 10,186 strains were annotated by Traitar and are greatly complementary to the curated phenotypes. For example, most Mycobacteriaceae are Gram positive (Goodfellow et al. 2012); among 10,153 (94.4%) Gram-positive strains, only 129 were collected by literature mining, while 10,024 were identified by Traitar imputation. Moreover, some phenotypes were not curated from the scientific literature and can be imputed by tools. For example, Hemolysis is the phenomenon of lysing red blood cells in the blood, which is an important indicator in Mycobacteriaceae identification, and 10,074 (93.7%) strains in Mycobacteriaceae are imputed with hemolytic phenotypes by Traitar.

The functional phenotypes were predicted by bioinformatics tools, and eight phenotypes of 10,186 strains were annotated in MPA, including numerous pathogeny-related molecules. A total of 139 classes of VFs were obtained from 10,060 strains, while glycopeptidolipids were annotated in 9755 (97.0%) strains and is the most prevalent VF in *Mycobacterium*, which are major surface glycolipids that could contribute to waterborne Mycobacteriaceae infections (Freeman et al. 2006; Ripoll et al. 2007). The top amino acid mutation in *Mycobacterium* is C38A (existing in 10,174 strains), which is associated with Mycobacteriaceae pathogenicity (Shi et al. 2014). The largest number of smBGCs are non-ribosomal peptide synthases (including 10,172 strains), which are involved in the synthesis of important natural products with biological activities, such as cyclosporine, bleomycin, and vancomycin.

In MPA, polyphasic phenotypes prefer to be qualitatively or even quantitatively detailed experimental test results, while functional phenotypes tend to be functional descriptions with qualitative high-throughput prediction. Although some phenotypes seem duplicated in polyphasic phenotypes and functional phenotypes literally, but they are unique in terms of phenotypic data. For instance, antibiotic substance in polyphasic phenotypes refer to the results of antimicrobial susceptibility test, in which antibiotic concentration, antibiotic sensitivity, and minimum inhibitory concentration are also included correspondingly. AMR in functional phenotypes is based on the functional annotation of sequence to obtain the name of potentially resistant drugs, and the AMR genes are embodied accordingly. In addition, metabolites of polyphasic phenotypes are derived from experimental results to test whether the bacterium is able to produce some metabolites. KEGG metabolites and MetaCyc metabolites of functional phenotypes illustrate the metabolites involved in the pathways that were predicted from the genome of Mycobacteriaceae strains. There are certain correlations between some phenotypes in polyphasic phenotype and functional phenotypes, but they are unique and complementary to each other, and their combination can help user find more comprehensive information.

**Fig. 6:**
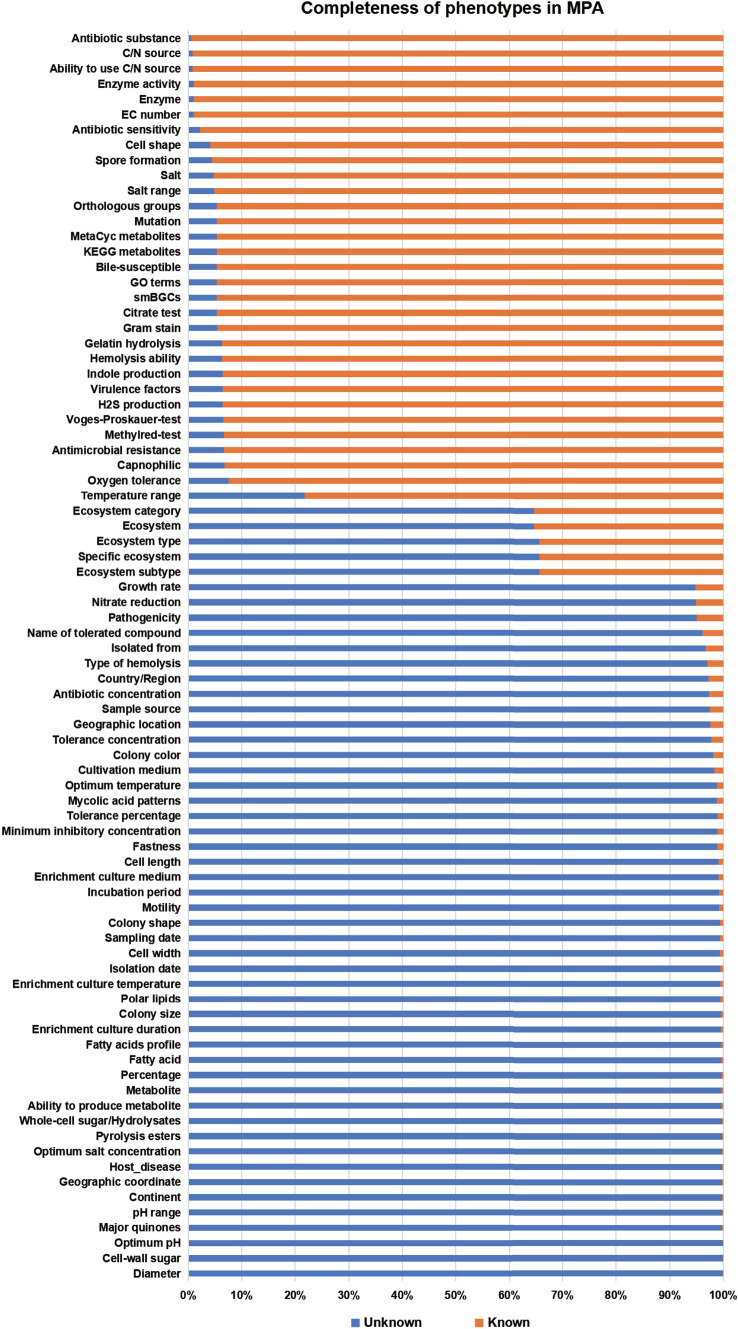
Completeness of phenotypes in MPA. The *y*-axis indicates 82 phenotypic elements of the Mycobacteriaceae phenome. The orange bar indicates the proportion of phenotypes recorded in the MPA, while the blue bar represents the proportion of phenotypes that are missing

Finally, we count the completeness/missingness of the phenotypes in MPA, and the detailed results can be seen in Fig. 6. The completeness of different phenotypes varied a lot. For example, the completeness of virulence factors phenotypes is very high, which can reach 93.54%, while the missingness of mycolic acid patterns is obvious, which is a pathogeny-related phenotype, only 1.14% of strains has records. Combining with Fig. 5, it can be seen that the completeness of the polyphasic phenotype predicted based on strain sequences is better, such as, Antibiotic substance, Ability to use C/N source and C/N source. However, more than two thirds of the polyphasic phenotype have less than 50% completeness in MPA, and Traitar cannot compensate for this lack of phenotypic information. Most of this polyphasic phenotypic information needs to be obtained through relevant experimental studies. This also shows that the current laboratory experiments to Mycobacteriaceae phenotypes are still insufficient. For functional phenotypes, not all phenotypes can achieve 100% phenotypic integrity due to the lack of genome completeness and the limited of existed reference database of functional phenotypes annotation for Mycobacteriaceae.

### MPA Web Server

The MPA web server is developed to facilitate search and comparison of phenomic data, the usage can be found on the help page (https://www.biosino.org/mpa/help).

**Fig. 7:**
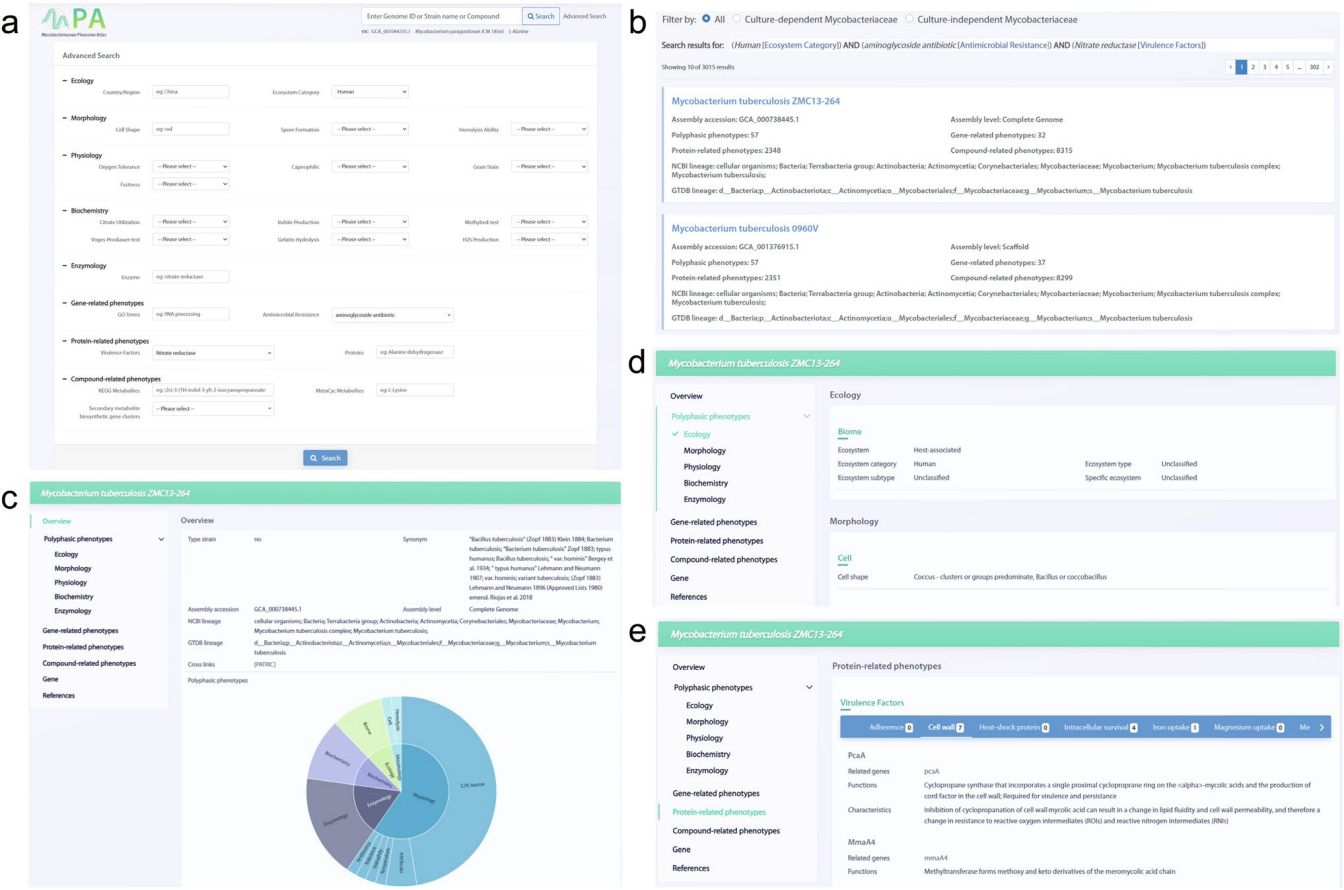
An example of advanced search in MPA. **a** Typed-in queries set "Human" for "Ecosystem Category" in Ecology module, "aminoglycoside antibiotic" for "Antimicrobial Resistance" in the Gene-related phenotypes module, and "Nitrate reductase" for "Virulence factors" in the Protein-related phenotypes module. **b** Subsequently, there were a total of 3,015 returned entries in the MPA. Users can select any of them to view their details. **c** An example of the "Overview" section in the strain detail page. **d** An example of the "Polyphasic phenotypes" section in the strain detail page. **e** An example of the "Functional phenotypes" section in the strain detail page

The user can retrieve the Mycobacteriaceae of interest and related phenotypic information by simple or advanced search (Fig. 7). The simple search allows for fuzzy queries by species name, genome ID or compound name, while the advanced search offers large-scale sophisticated queries, containing 23 terms from five polyphasic modules (ecology, morphology, physiology, biochemistry, enzymology) and three functional modules (gene-related phenotypes, protein-related phenotypes, compound-related phenotypes). The basic information of strains which match the query conditions are listed in the result page, and the comprehensive phenotypes can be visualized in the detail page of each strain.

**Fig. 8:**
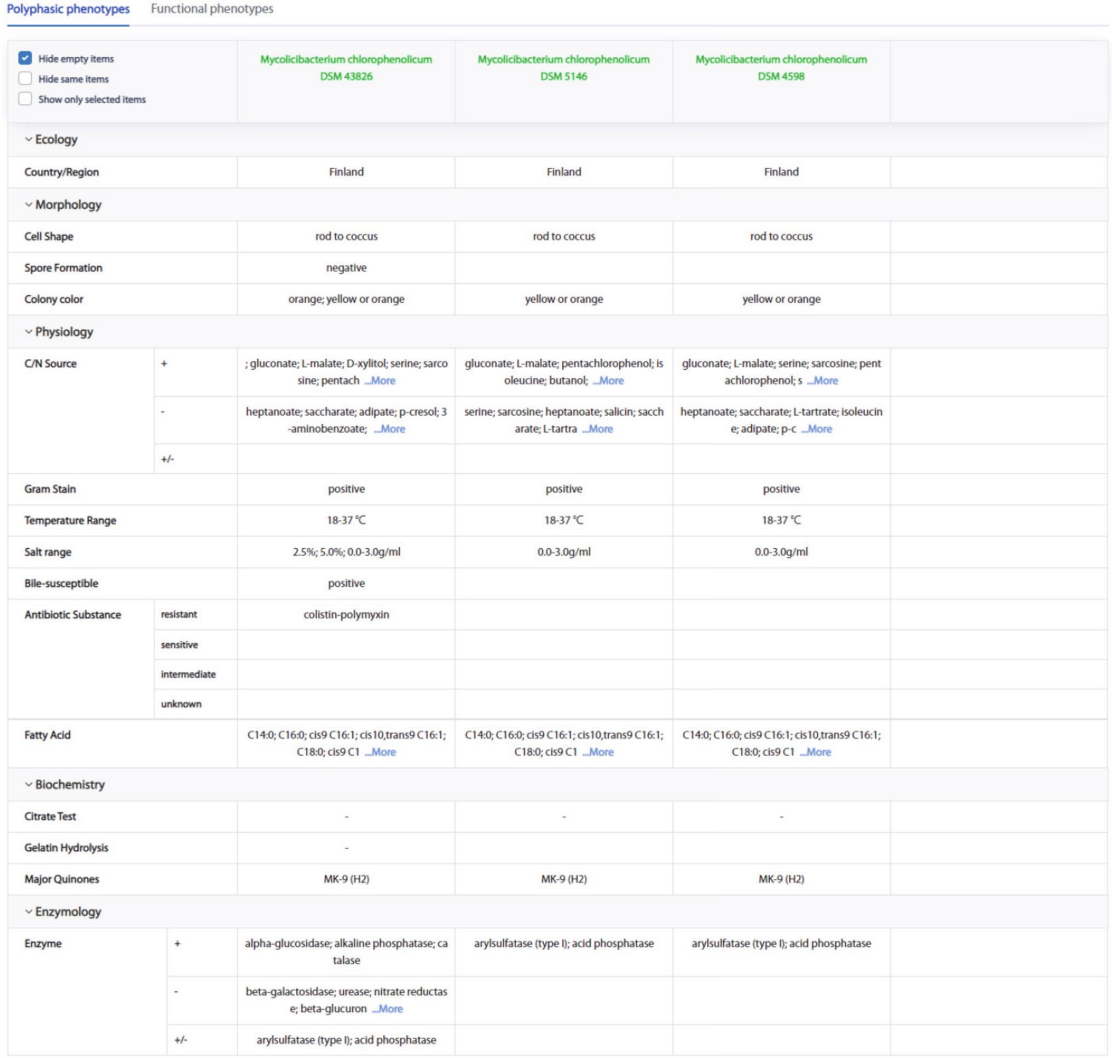
The result of the phenotype comparison for the reclassification of *Mycolicibacterium chlorophenolicum* DSM 43826. Phenotypes such as "Whole-cell fatty acid compositions", "Cell shape", "Major quinones", "Temperature range", "Nitrate reduction", "Acid phosphatase activity", "Arylsulfatase activity", "Salt range", and "C/N Source" were consistent between the MPA web server and the scientific literature

The user can compare up to 41 phenotypes and four strains at a time by using phenotype comparison function, with the options of only view phenotypes with different values, only view phenotypes without null values, or only view phenotypes of interest. To demonstrate this function, we compared the phenotypes between *Mycolicibacterium vanbaalenii* DSM 7251 and *Mycolicibacterium mageritense* DSM 44476. We reproduced the phenotypic differences between the two strains described in literature and found differential traits from the molecular perspective that was not collected in literature, such as VFs, AMR, smBGCs and so on, which may aid researchers to find key clues for research topics (Garcia 1997; Khan et al. 2002). On the other hand, phenotype comparison function may be a useful tool to make preliminary classifications and identifications of strain. For example, phenotype comparison could be used for *Mycolicibacterium chlorophenolicum* DSM 43826, which was classified from *Rhodococcus chlorophenolicus* to *M. chlorophenolicum* in 1994 (Hagglblom et al. 1994). The phenotypes used for comparison among DSM 43826, DSM 5146, and DSM 4598, can be illustrated by the MPA web server (Fig. 8), such as "Whole-cell fatty acid compositions", "Cell shape", "Major quinones", "Temperature range", "Nitrate reduction", "Acid phosphatase activity", "Arylsulfatase activity", "Salt range", and "C/N Source".

Tuberculosis caused by *M. tuberculosis* infection is a fatal infectious disease. *M. tuberculosis* can adapt to various antibiotics and invade the host's immune system. To facilitate the relevant researchers to *M. tuberculosis* drug resistance research, we obtained 20 genes/intergenic regions related to drug resistance from the previous *M. tuberculosis*-GWAS research results, involving 78 mutation types (57 genetic mutation types and 21 mutation of intergenic region types) (Coll et al. 2018). Totally, 74 of 78 mutation types existed in MPA (54 genetic mutation types and 20 mutation of intergenic region types), and the user can access this data from the MPA download page (https://www.biosino.org/mpa/download).

### Co-evolution of *M. tuberculosis* Genome with Virulence Phenotypes

**Fig. 9:**
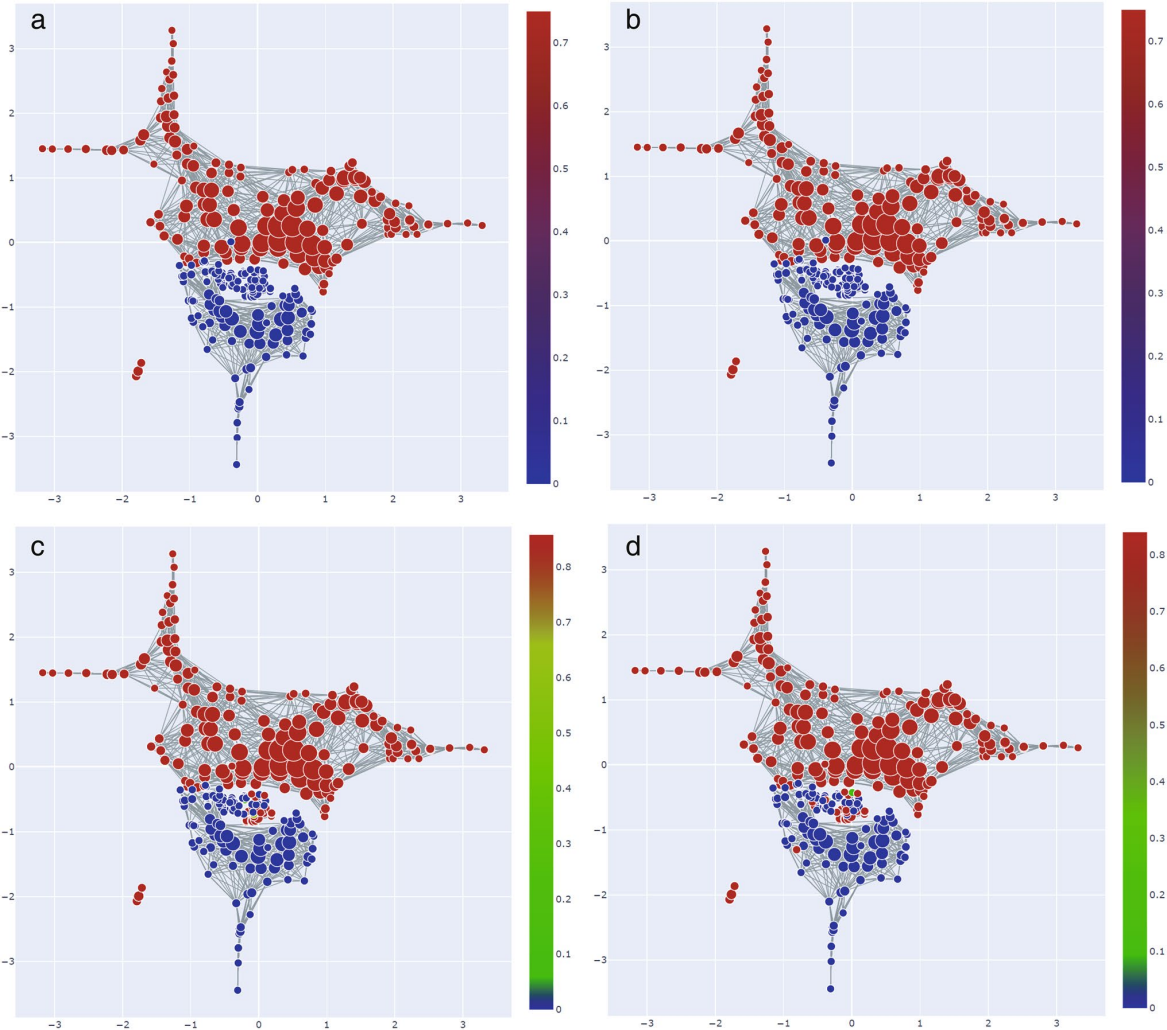
TDA network enrichment patterns of pathogenicity-related phenotypes of *M. tuberculosis*. The TDA network enrichment patterns and their SAFE scores of **a** *M. tuberculosis*, **b** ESX-3, **c** ESX-5 (ESAT-like secretion system), and **d** Nitrate reductase. Node color is based on the SAFE scores of corresponding covariates, from red (large values) to blue (small values). The TDA network enrichment patterns of ESX-3, ESX-5, and nitrate reductase appear to all be comparable to that of *M. tuberculosis*

VFs genes are essential for virulence or pathogenicity (Siegrist et al. 2009; Shah et al. 2015; Chen 2016; Sayes et al. 2016; Ly and Liu 2020) and might be the targets of antimicrobial drugs or aid in the selection of antimicrobial drugs. *M. tuberculosis* is a pathogen that has been widely spread and is not completely controlled. Among a total of 151 virulence factors whose SAFE-enriched scores are greater than 0, 36 VFs were found to be co-enriched with the genomes of *M. tuberculosis*, suggesting the co-evolution of these VFs with the virulence of *M. tuberculosis*. For instance, the co-enriched ESX-3 T7SS secretes certain effectors that are essential for iron uptake, while the other secreted effectors modulate virulence in an iron-independent fashion (Fig. 9a, b) (Tufariello et al. 2016). The co-enriched ESX-5 secretion system of *M. tuberculosis* is vital for bacterial virulence and for the secretion of the large PE/PPE protein family (Fig. 9a, c) (Shah et al. 2015). The co-enriched nitrate reductase helps *M. tuberculosis* to survive in O_2_-depleted areas of inflammatory or necrotic tissue (Fig. 9a, d) (Smith 2003). Topological data analysis of pathogenicity-related phenotypes in MPA could be potentially used as an effective tool for studying the evolution of pathogenesis, their molecular mechanism and antimicrobial targets of *M. tuberculosis*.

### Investigation of Pathogen-Enriched Pathways

In further analysis of pathogen-enriched pathways for Mycobacteriaceae strains by Fisher's exact test, 260 of 537 KEGG pathways were found to be pathogen-enriched pathways of Mycobacteriaceae (Supplementary Table 6). These pathways might aid in the study of pathogenicity mechanism for Mycobacteriaceae and be the potential targets for antibiotics. For example, sulfur metabolism pathway, a pathogen-enriched pathway by our analysis, is essential for the survival and virulence of many pathogens (including *M. tuberculosis*), and many genes involved in this pathway could be used as drug targets (Bhave et al. 2008; Zeng et al. 2013). In addition, protein export pathway in the pathogen-enriched pathways is essential for pathogenesis (Miller et al. 2017). Biosynthesis of amino acid has been evolved as a mechanism to evade starvation attack in *M. tuberculosis*, which is also a pathogen-enriched pathway by our analysis (Mishra and Surolia 2018).

## Discussion

In this study, a data-driven approach was used to establish the data elements of the Mycobacteriaceae phenome. Through literature review, third-party database integration, and bioinformatics annotation, 82 microbial phenotypic traits were developed. The MPA covers the most collectable and computable phenotypes, has almost half of its phenotypes as compatible with BacDive and OMP, and is a valuable complement to the existing microbial phenotype-related databases. Furthermore, the name and value of each element in MPA is standardized, which will greatly help potential phenomic comparison and analysis. In general, the standardized data elements of MPA are not only suitable for the data governance process of Mycobacteriaceae, but can also be helpful for understanding the phenomes of other pathogens.

Most polyphasic phenotypes of strain are well described and compared in the literature on the discovery of novel microbial species, and the subsequent study of these strains focus on the study of limited specific phenotypes. In this study, we adopted a phenome method by curating the literature of microbial species discovery. For instance, we manually curated 63 phenotypes from 199 papers with some phenotypes such as Enzyme activity, Enzyme, Ability to use C/N source, and C/N source, having up to 90% coverage in the literature (Supplementary Table 5). By contract, the phenotype curation efficiency and coverage is quite low by the traditional method of studying individual phenotype. For example, we tried other search method when we found that infection rates were not available through our current search method, but the search results were poor. Of the 4,084 papers in the search results, only 71 were related to infection rate. After curating the top 20 relevant papers, we found that only one is about the relationship between infection rate and phenotypes at the strain level. We used about one week to curate these  phenotypes, and it is not realistic to curate all 13,667 phenotypes by using this method. Compared to the traditional method of studying individual phenotype, we use a phenome approach to study Mycobacteriaceae phenome, which efficiently curated a better coverage of phenotypes.

The MPA gathered the phenomic data of Mycobacteriaceae strains. Compared to the existing Mycobacteriaceae datasets, MPA is not species based and contains almost all phenotypes (Ranjan et al. 2006; Kapopoulou et al. 2011; Midford et al. 2019; Reimer et al. 2019, 2022; Davis et al. 2020), which make MPA the largest and most complete Mycobacteriaceae dataset. It can also be cross-linked to typical microbial phenotype databases such as BacDive and PATRIC. The dataset in the MPA can be analyzed by *tmap* to explore important phenotypes for virulence and pathogenicity. The co-evolution of *M. tuberculosi*s with VFs and the investigation of pathogen-enriched pathways might provide clues to the molecular mechanism of Mycobacteriaceae pathogenicity, and aid in the study of the potential targets for antimicrobial drugs. Due to that infection rate data is limited, it is hard for us to discuss the differential phenomic characters in terms of pathogenicity and infection rate basing on the current research strategy. However, our data could potentially be used for epidemiologists to study the differential phenomic characters in terms of pathogenicity and infection rate by combing the infection rate collected in the pandemic study.

## Conclusion

This study used a data-driven approach to establish the data elements of the Mycobacteriaceae phenome, which is a valuable complement to the existing microbial phenotype-related databases. The largest and most complete Mycobacteriaceae dataset was constructed by using the Mycobacteriaceae phenome. A topological data analysis of MPA revealed the co-evolution between *M. tuberculosis* and virulence factors, and uncovered potential pathogenicity-associated phenotypes. Two hundred and sixty potential pathogen-enriched pathways were found by Fisher's exact test. The application of MPA may provide novel insights into the pathogenicity mechanism and antimicrobial targets of Mycobacteriaceae. For the sake of the limited time and materials, a phenome approach was used to study Mycobacteriaceae phenome. Further efforts can be done to apply the traditional method of studying individual phenotype to study the individual phenotype of interest.

### Supplementary Information

Below is the link to the electronic supplementary material.

Supplementary file1 (PDF 4469 KB)

## Data Availability

All data generated or analyzed during this study are included in the Mycobacteriaceae Phenome Atlas (MPA, https://www.biosino.org/mpa/). Key data resources and tools used in this study are listed in Supplementary Table 7.

## Abbreviations

MPA: Mycobacteriaceae Phenome Atlas
VFs: Virulence factors
AMR: Antimicrobial resistance
OMP: Ontology of Microbial Phenotypes
NCBI: National Center for Biotechnology Information
LPSN: List of prokaryotic names with standing in nomenclature
PATRIC: PathoSystems Resource Integration Center
MAGs: Metagenome-assembled genomes
IJSEM: International Journal of Systematic and Evolutionary Microbiology
GOLD: Genomes OnLine Database
JGI: Joint Genome Institute
GO: Gene Ontology
RGI: Resistance gene identifier
CARD: Comprehensive Antibiotic Resistance Database
COG: Clusters of orthologous groups
TDA: Topological data analysis
*M. tuberculosis*: *Mycobacterium tuberculosis*
BacDive: Bacterial Diversity Metadatabase
VFDB: Virulence Factor Database
DIAMOND: Double index alignment of next-generation sequencing data
KEGG: Kyoto Encyclopedia of Genes and Genomes
SAFE: Spatial analysis of functional enrichment
TIGRFAM: The Institute for Genomic Research (TIGR) Functional Analysis of Genomes
GEM: Genomes from Earth's microbiomes
Pfam: Protein family
GenBank: Genetic Sequence Data Bank
smBGCs: Secondary metabolite biosynthetic gene clusters
PubChem: Public Chemical Database
GTDB: Genome Taxonomy Database
UniProt: Universal Protein Resource
PCT: Percentage
