# Supplementary material for: Mycobacteriaceae Phenome Atlas (MPA): A Standardized Atlas for the Mycobacteriaceae Phenome Based on Heterogeneous Sources
Source: Phenomics. 2023 Jun 13;3(5):439–56. doi: 10.1007/s43657-023-00101-5 (PMC10593683; doi:10.1007/s43657-023-00101-5)
Supplement: Supplementary file 1 — Supplementary file1 (PDF 4469 KB) [file 43657_2023_101_MOESM1_ESM.pdf]

Supplementary Table 1 The overview of Mycobacteriaceae datasets

Database name	Institute	Genus number	Species number	Strain number	Subspecies number	Genome number	MAGs number	Desription
MPA (Mycobacteriaceae Phenome Atlas) https://www.biosino.org/mpa/	Shanghai Institute of Nutrition and Health (SINH), China	5 (*Mycobacterium* , *Mycobacteroides* , *Mycolicibacillus* , *Mycolicibacter* , and *Mycolicibacterium*)	236	10,755	18	10,155	31	MPA is a Mycobacteriaceae phenome database, which integrates the phenotypes of strains in Mycobacteriaceae family by literature mining, third-party database integration, and bioinformatics annotation. The phenotypes of Mycobacteriaceae are inferred from available phenomic data, and 82 microbial phenotypic traits were recruited as data elements of the microbial phenome, containing 5 categories and 20 subcategories of polyphasic phenotypes, and 3 categories and 8 subcategories of functional phenotypes, all of which are complementary to the existing data standards of microbial phenotypes. The phenotypes were searchable and comparable from the website of MPA. A topological data analysis of MPA revealed the co-evolution between Mycobacterium tuberculosis and virulence factors, and uncovered potential pathogenicity-associated phenotypes. 260 potential pathogen-enriched pathways were found by Fisher's exact test. The application of MPA may provide novel insights into the pathogenicity mechanism and antimicrobial targets of Mycobacteriaceae.
Mycobrowser https://mycobrowser.epfl.ch/	Ecole Polytechnique Féd érale de Lausanne, Switzerland	1 (*Mycobacterium*)	9	10	0	10	0	Mycobrowser is a comprehensive genomic and proteomic data repository for pathogenic mycobacteria that provides manually curated annotations and other appropriate tools to facilitate the genomic and proteomic study of these organisms, but only includes 10 strains from 9 Mycobacterium species, and does not include polyphasic phenotypes.
MycoperonDB http://cdfd.org.in/mycoperondb/	Centre for DNA Fingerprinting and Diagnostics, India	1 (*Mycobacterium*)	4	5	0	0	0	MycoperonDB provides the operons and transcription units of five strains from four Mycobacterium species, but has not been updated since the first version released in 2006.
Mycobacterium tuberculosis Pathway/GenomeDatabases https://mycobacterium.biocyc.org/	SRI International, USA	1 (*Mycobacterium*)	1	51	N/A	51	0	The Mycobacterium tuberculosis Pathway/Genome Database contains 51 genomes, their associated metabolic pathways, and predictions of missing enzymes and transcription units in these metabolic pathways, but only contains one species and does not provide polyphasic phenotypes.
BacDive http://bacdive. dsmz.de/	Leibniz Institute DSMZ-German Collection of Microorganisms and Cell Cultures, Germany	2 (*Mycobacterium* and *Mycolicibacterium*)	196	747	7	2747	0	BacDive is a comprehensive repository of structured data on prokaryotic taxonomy, morphology, physiology, culture, and isolation, but only contains 196 species and 7 subspecies of Mycobacteriaceae, and does not include functional phenotypes.
PATRIC https://www.patricbrc.org/	National Institute of Allergy and Infectious Diseases, USA	6 (*Hoyosella* , *Mycobacterium* , *Mycobacteroides* , *Mycolicibacillus* , *Mycolicibacter* , and *Mycolicibacterium*)	213	10000+	1804	32936	0	PATRIC maintained by the bacterial Bioinformatics Resource Center, provides a biological information analysis platform for all bacteria, and includes basic metadata regarding organism, host, motility, and cell shape, but contains very few functional phenotypes, such as antimicrobial resistance, GO terms, and virulence factors.

Data collection upto 19th, April, 2021

Supplementary Table 2 Mycobacteriaceae genomes used in MPA

source	genome_id	taxonomy	taxon_id	genome_length	completeness	contamination	whether used for TMAP
GEM	3300001422_1	GTDB: d__Bacteria; p__Actinobacteriota; c__Actinobacteria; o__Mycobacteriales; f__Mycobacteriaceae; g__Mycobacterium; s__	IMG: 3300001422	2797424	62.13	0.55	No
GEM	3300009503_23	GTDB: d__Bacteria; p__Actinobacteriota; c__Actinobacteria; o__Mycobacteriales; f__Mycobacteriaceae; g__Mycobacterium; s__	IMG: 3300009503	4364775	92.93	0.90	Yes
GEM	3300009503_66	GTDB: d__Bacteria; p__Actinobacteriota; c__Actinobacteria; o__Mycobacteriales; f__Mycobacteriaceae; g__Mycobacterium; s__	IMG: 3300009503	2836500	65.19	2.28	No
GEM	3300010157_10	GTDB: d__Bacteria; p__Actinobacteriota; c__Actinobacteria; o__Mycobacteriales; f__Mycobacteriaceae; g__Mycolicibacterium; s__	IMG: 3300010157	2620373	75.67	2.41	No
GEM	3300010373_34	GTDB: d__Bacteria; p__Actinobacteriota; c__Actinobacteria; o__Mycobacteriales; f__Mycobacteriaceae; g__Mycobacterium; s__	IMG: 3300010373	4345273	71.18	3.58	No
GEM	3300011118_36	GTDB: d__Bacteria; p__Actinobacteriota; c__Actinobacteria; o__Mycobacteriales; f__Mycobacteriaceae; g__Mycolicibacterium; s__	IMG: 3300011118	2997865	92.28	1.10	Yes
GEM	3300011418_6	GTDB: d__Bacteria; p__Actinobacteriota; c__Actinobacteria; o__Mycobacteriales; f__Mycobacteriaceae; g__Mycolicibacter; s__	IMG: 3300011418	4532678	97.73	0.61	Yes
GEM	3300012177_4	GTDB: d__Bacteria; p__Actinobacteriota; c__Actinobacteria; o__Mycobacteriales; f__Mycobacteriaceae; g__Mycobacterium; s__	IMG: 3300012177	4701679	68.58	1.47	No
GEM	3300013004_22	GTDB: d__Bacteria; p__Actinobacteriota; c__Actinobacteria; o__Mycobacteriales; f__Mycobacteriaceae; g__Mycolicibacterium; s__	IMG: 3300013004	3164719	93.94	0.66	Yes
GEM	3300013093_17	GTDB: d__Bacteria; p__Actinobacteriota; c__Actinobacteria; o__Mycobacteriales; f__Mycobacteriaceae; g__Mycobacterium; s__	IMG: 3300013093	3038802	83.25	1.83	No
GEM	3300015206_8	GTDB: d__Bacteria; p__Actinobacteriota; c__Actinobacteria; o__Mycobacteriales; f__Mycobacteriaceae; g__Mycolicibacterium; s__	IMG: 3300015206	5572061	74.70	3.43	No
GEM	3300015206_9	GTDB: d__Bacteria; p__Actinobacteriota; c__Actinobacteria; o__Mycobacteriales; f__Mycobacteriaceae; g__Mycobacterium; s__	IMG: 3300015206	5450430	96.67	4.23	Yes
GEM	3300017650_11	GTDB: d__Bacteria; p__Actinobacteriota; c__Actinobacteria; o__Mycobacteriales; f__Mycobacteriaceae; g__Mycolicibacter; s__Mycolicibacter kumamotonensis	IMG: 3300017650	3616136	83.71	0.61	No
GEM	3300017816_9	GTDB: d__Bacteria; p__Actinobacteriota; c__Actinobacteria; o__Mycobacteriales; f__Mycobacteriaceae; g__Mycolicibacterium; s__	IMG: 3300017816	4368534	78.86	2.91	No
GEM	3300023088_8	GTDB: d__Bacteria; p__Actinobacteriota; c__Actinobacteria; o__Mycobacteriales; f__Mycobacteriaceae; g__Mycobacterium; s__	IMG: 3300023088	3006706	62.81	1.74	No
GEM	3300024429_5	GTDB: d__Bacteria; p__Actinobacteriota; c__Actinobacteria; o__Mycobacteriales; f__Mycobacteriaceae; g__Mycolicibacterium; s__	IMG: 3300024429	3789932	95.40	1.91	Yes

GEM	3300024433_18	GTDB: d__Bacteria; p__Actinobacteriota; c__Actinobacteria; o__Mycobacteriales; f__Mycobacteriaceae; g__Mycolicibacterium; s__Mycolicibacterium sp001510415	IMG: 3300024433	3394879	84.59	0.31	No
GEM	3300025202_2	GTDB: d__Bacteria; p__Actinobacteriota; c__Actinobacteria; o__Mycobacteriales; f__Mycobacteriaceae; g__Mycolicibacterium; s__	IMG: 3300025202	5883197	95.71	2.70	Yes
GEM	3300025503_14	GTDB: d__Bacteria; p__Actinobacteriota; c__Actinobacteria; o__Mycobacteriales; f__Mycobacteriaceae; g__Mycobacterium; s__	IMG: 3300025503	3524065	65.00	0.06	No
GEM	3300025503_23	GTDB: d__Bacteria; p__Actinobacteriota; c__Actinobacteria; o__Mycobacteriales; f__Mycobacteriaceae; g__Mycobacterium; s__	IMG: 3300025503	2481188	54.24	0.38	No
GEM	3300025528_11	GTDB: d__Bacteria; p__Actinobacteriota; c__Actinobacteria; o__Mycobacteriales; f__Mycobacteriaceae; g__Mycolicibacterium; s__Mycolicibacterium hassiacum	IMG: 3300025528	3573366	82.61	0.23	No
GEM	3300025534_6	GTDB: d__Bacteria; p__Actinobacteriota; c__Actinobacteria; o__Mycobacteriales; f__Mycobacteriaceae; g__Mycolicibacterium; s__	IMG: 3300025534	4089664	100.00	1.14	Yes
GEM	3300025691_5	GTDB: d__Bacteria; p__Actinobacteriota; c__Actinobacteria; o__Mycobacteriales; f__Mycobacteriaceae; g__Mycolicibacterium; s__	IMG: 3300025691	3945965	79.82	0.67	No
GEM	3300025713_6	GTDB: d__Bacteria; p__Actinobacteriota; c__Actinobacteria; o__Mycobacteriales; f__Mycobacteriaceae; g__Mycolicibacterium; s__	IMG: 3300025713	4073597	97.03	1.12	Yes
GEM	3300027496_1	GTDB: d__Bacteria; p__Actinobacteriota; c__Actinobacteria; o__Mycobacteriales; f__Mycobacteriaceae; g__Mycobacterium; s__	IMG: 3300027496	4745287	74.30	3.41	No
GEM	3300027692_5	GTDB: d__Bacteria; p__Actinobacteriota; c__Actinobacteria; o__Mycobacteriales; f__Mycobacteriaceae; g__Mycobacterium; s__	IMG: 3300027692	6565537	97.44	2.22	Yes
GEM	3300027708_25	GTDB: d__Bacteria; p__Actinobacteriota; c__Actinobacteria; o__Mycobacteriales; f__Mycobacteriaceae; g__Mycolicibacterium; s__	IMG: 3300027708	2063682	60.81	0.98	No
GEM	3300027760_22	GTDB: d__Bacteria; p__Actinobacteriota; c__Actinobacteria; o__Mycobacteriales; f__Mycobacteriaceae; g__Mycolicibacterium; s__	IMG: 3300027760	2866696	90.94	0.91	Yes
GEM	3300027870_35	GTDB: d__Bacteria; p__Actinobacteriota; c__Actinobacteria; o__Mycobacteriales; f__Mycobacteriaceae; g__Mycolicibacterium; s__	IMG: 3300027870	3678803	75.92	0.38	No
GEM	3300027894_8	GTDB: d__Bacteria; p__Actinobacteriota; c__Actinobacteria; o__Mycobacteriales; f__Mycobacteriaceae; g__Mycobacterium; s__	IMG: 3300027894	5152414	74.46	2.37	No
GEM	3300028569_30	GTDB: d__Bacteria; p__Actinobacteriota; c__Actinobacteria; o__Mycobacteriales; f__Mycobacteriaceae; g__Mycolicibacterium; s__	IMG: 3300028569	2922698	91.74	2.36	Yes
NCBI Assembly	GCA_000651795.1	NCBI: cellular organisms; Bacteria; Terrabacteria group; Actinobacteria; Actinomycetia; Corynebacteriales; Mycobacteriaceae; Mycobacterium; Mycobacterium tuberculosis complex; Mycobacterium tuberculosis;	NCBI: 1448421	4405924	99.94	0.00	Yes
NCBI Assembly	GCA_000662585.1	NCBI: cellular organisms; Bacteria; Terrabacteria group; Actinobacteria; Actinomycetia; Corynebacteriales; Mycobacteriaceae; Mycobacterium; Mycobacterium tuberculosis complex; Mycobacterium tuberculosis;	NCBI: 1448662	4434386	99.94	0.00	Yes
NCBI Assembly	GCA_000389905.1	NCBI: cellular organisms; Bacteria; Terrabacteria group; Actinobacteria; Actinomycetia; Corynebacteriales; Mycobacteriaceae; Mycobacterium; Mycobacterium tuberculosis complex; Mycobacterium tuberculosis;	NCBI: 1304279	4404786	90.35	0.33	Yes

NCBI Assembly	GCA_000523595.1	NCBI: cellular organisms; Bacteria; Terrabacteria group; Actinobacteria; Actinomycetia; Corynebacteriales; Mycobacteriaceae; Mycobacterium; Mycobacterium avium complex (MAC); Mycobacterium intracellulare;	NCBI: 1767	5517312	96.41	0.36	Yes
NCBI Assembly	GCA_000660765.1	NCBI: cellular organisms; Bacteria; Terrabacteria group; Actinobacteria; Actinomycetia; Corynebacteriales; Mycobacteriaceae; Mycobacterium; Mycobacterium tuberculosis complex; Mycobacterium tuberculosis;	NCBI: 1324244	4403926	99.94	0.00	Yes
NCBI Assembly	GCA_000655875.1	NCBI: cellular organisms; Bacteria; Terrabacteria group; Actinobacteria; Actinomycetia; Corynebacteriales; Mycobacteriaceae; Mycobacterium; Mycobacterium tuberculosis complex; Mycobacterium tuberculosis;	NCBI: 1427302	4437660	99.94	0.00	Yes
NCBI Assembly	GCA_000658535.1	NCBI: cellular organisms; Bacteria; Terrabacteria group; Actinobacteria; Actinomycetia; Corynebacteriales; Mycobacteriaceae; Mycobacterium; Mycobacterium tuberculosis complex; Mycobacterium tuberculosis;	NCBI: 1438824	4392926	99.61	0.00	Yes
NCBI Assembly	GCA_000659505.1	NCBI: cellular organisms; Bacteria; Terrabacteria group; Actinobacteria; Actinomycetia; Corynebacteriales; Mycobacteriaceae; Mycobacterium; Mycobacterium tuberculosis complex; Mycobacterium tuberculosis;	NCBI: 1295753	4389202	99.28	0.00	Yes
NCBI Assembly	GCA_000662905.1	NCBI: cellular organisms; Bacteria; Terrabacteria group; Actinobacteria; Actinomycetia; Corynebacteriales; Mycobacteriaceae; Mycobacterium; Mycobacterium tuberculosis complex; Mycobacterium tuberculosis;	NCBI: 1448678	4422567	99.94	0.00	Yes
NCBI Assembly	GCA_000257745.1	NCBI: cellular organisms; Bacteria; Terrabacteria group; Actinobacteria; Actinomycetia; Corynebacteriales; Mycobacteriaceae; Mycobacterium; Mycobacterium xenopi;	NCBI: 1150591	4434836	99.09	1.14	Yes
NCBI Assembly	GCA_000649295.1	NCBI: cellular organisms; Bacteria; Terrabacteria group; Actinobacteria; Actinomycetia; Corynebacteriales; Mycobacteriaceae; Mycobacterium; Mycobacterium tuberculosis complex; Mycobacterium tuberculosis;	NCBI: 1438858	4419479	99.94	0.00	Yes
NCBI Assembly	GCA_000661085.1	NCBI: cellular organisms; Bacteria; Terrabacteria group; Actinobacteria; Actinomycetia; Corynebacteriales; Mycobacteriaceae; Mycobacterium; Mycobacterium avium complex (MAC); Mycobacterium colombiense;	NCBI: 1324261	6044235	99.94	0.50	Yes
NCBI Assembly	GCA_000389925.1	NCBI: cellular organisms; Bacteria; Terrabacteria group; Actinobacteria; Actinomycetia; Corynebacteriales; Mycobacteriaceae; Mycobacterium; Mycobacterium tuberculosis complex; Mycobacterium tuberculosis;	NCBI: 1310114	4392876	89.66	0.67	No
NCBI Assembly	GCA_000523615.1	NCBI: cellular organisms; Bacteria; Terrabacteria group; Actinobacteria; Actinomycetia; Corynebacteriales; Mycobacteriaceae; Mycobacterium; Mycobacterium kansasii;	NCBI: 1299326	6896162	99.54	3.54	Yes
NCBI Assembly	GCA_000655895.1	NCBI: cellular organisms; Bacteria; Terrabacteria group; Actinobacteria; Actinomycetia; Corynebacteriales; Mycobacteriaceae; Mycobacterium; Mycobacterium tuberculosis complex; Mycobacterium tuberculosis;	NCBI: 1427303	4448271	99.78	0.67	Yes
NCBI Assembly	GCA_000662925.1	NCBI: cellular organisms; Bacteria; Terrabacteria group; Actinobacteria; Actinomycetia; Corynebacteriales; Mycobacteriaceae; Mycobacterium; Mycobacterium tuberculosis complex; Mycobacterium tuberculosis;	NCBI: 1448679	4423857	99.94	0.00	Yes
NCBI Assembly	GCA_000660785.1	NCBI: cellular organisms; Bacteria; Terrabacteria group; Actinobacteria; Actinomycetia; Corynebacteriales; Mycobacteriaceae; Mycobacterium; Mycobacterium tuberculosis complex; Mycobacterium tuberculosis;	NCBI: 1324245	4416147	99.94	0.00	Yes
NCBI Assembly	GCA_000651815.1	NCBI: cellular organisms; Bacteria; Terrabacteria group; Actinobacteria; Actinomycetia; Corynebacteriales; Mycobacteriaceae; Mycobacterium; Mycobacterium tuberculosis complex; Mycobacterium tuberculosis;	NCBI: 1448426	4369162	99.94	0.00	Yes
NCBI Assembly	GCA_000649315.1	NCBI: cellular organisms; Bacteria; Terrabacteria group; Actinobacteria; Actinomycetia; Corynebacteriales; Mycobacteriaceae; Mycobacterium; Mycobacterium tuberculosis complex; Mycobacterium tuberculosis;	NCBI: 1438872	4417036	99.94	0.00	Yes
NCBI Assembly	GCA_000661105.1	NCBI: cellular organisms; Bacteria; Terrabacteria group; Actinobacteria; Actinomycetia; Corynebacteriales; Mycobacteriaceae; Mycobacterium; Mycobacterium tuberculosis complex; Mycobacterium tuberculosis;	NCBI: 1324262	4398871	99.94	0.33	Yes
NCBI Assembly	GCA_000662605.1	NCBI: cellular organisms; Bacteria; Terrabacteria group; Actinobacteria; Actinomycetia; Corynebacteriales; Mycobacteriaceae; Mycobacterium; Mycobacterium tuberculosis complex; Mycobacterium tuberculosis;	NCBI: 1448663	4405588	99.94	0.00	Yes

NCBI Assembly	GCA_000260575.2	NCBI: cellular organisms; Bacteria; Terrabacteria group; Actinobacteria; Actinomycetia; Corynebacteriales; Mycobacteriaceae; Mycobacteroides; Mycobacteroides abscessus;	NCBI: 1162288	4794504	100.00	0.00	Yes
NCBI Assembly	GCA_000658555.1	NCBI: cellular organisms; Bacteria; Terrabacteria group; Actinobacteria; Actinomycetia; Corynebacteriales; Mycobacteriaceae; Mycobacterium; Mycobacterium tuberculosis complex; Mycobacterium tuberculosis;	NCBI: 1438825	4387431	99.94	0.23	Yes
NCBI Assembly	GCA_000659525.1	NCBI: cellular organisms; Bacteria; Terrabacteria group; Actinobacteria; Actinomycetia; Corynebacteriales; Mycobacteriaceae; Mycobacterium; Mycobacterium tuberculosis complex; Mycobacterium tuberculosis;	NCBI: 1295756	4401637	99.94	0.00	Yes
NCBI Assembly	GCA_000389945.1	NCBI: cellular organisms; Bacteria; Terrabacteria group; Actinobacteria; Actinomycetia; Corynebacteriales; Mycobacteriaceae; Mycobacterium; Mycobacterium tuberculosis complex; Mycobacterium tuberculosis;	NCBI: 1310115	4390306	99.61	0.00	Yes
NCBI Assembly	GCA_000523635.1	NCBI: cellular organisms; Bacteria; Terrabacteria group; Actinobacteria; Actinomycetia; Corynebacteriales; Mycobacteriaceae; Mycobacterium; Mycobacterium avium complex (MAC); unclassified Mycobacterium avium complex (MAC);	NCBI: 1335321	5393830	99.61	1.44	Yes
NCBI Assembly	GCA_000662945.1	NCBI: cellular organisms; Bacteria; Terrabacteria group; Actinobacteria; Actinomycetia; Corynebacteriales; Mycobacteriaceae; Mycobacterium; Mycobacterium tuberculosis complex; Mycobacterium tuberculosis;	NCBI: 1448680	4424508	99.94	0.00	Yes
NCBI Assembly	GCA_000655915.1	NCBI: cellular organisms; Bacteria; Terrabacteria group; Actinobacteria; Actinomycetia; Corynebacteriales; Mycobacteriaceae; Mycobacterium; Mycobacterium tuberculosis complex; Mycobacterium tuberculosis;	NCBI: 1427304	4392894	99.94	0.00	Yes
NCBI Assembly	GCA_000651835.1	NCBI: cellular organisms; Bacteria; Terrabacteria group; Actinobacteria; Actinomycetia; Corynebacteriales; Mycobacteriaceae; Mycobacterium; Mycobacterium tuberculosis complex; Mycobacterium tuberculosis;	NCBI: 1448427	4423523	99.94	0.00	Yes
NCBI Assembly	GCA_000649335.1	NCBI: cellular organisms; Bacteria; Terrabacteria group; Actinobacteria; Actinomycetia; Corynebacteriales; Mycobacteriaceae; Mycobacterium; Mycobacterium tuberculosis complex; Mycobacterium tuberculosis;	NCBI: 1438852	4534850	99.78	2.93	Yes
NCBI Assembly	GCA_000662625.1	NCBI: cellular organisms; Bacteria; Terrabacteria group; Actinobacteria; Actinomycetia; Corynebacteriales; Mycobacteriaceae; Mycobacterium; Mycobacterium tuberculosis complex; Mycobacterium tuberculosis;	NCBI: 1448664	4445479	99.94	0.33	Yes
NCBI Assembly	GCA_000260595.1	NCBI: cellular organisms; Bacteria; Terrabacteria group; Actinobacteria; Actinomycetia; Corynebacteriales; Mycobacteriaceae; Mycobacteroides; Mycobacteroides abscessus;	NCBI: 1162287	4975940	100.00	0.00	Yes
NCBI Assembly	GCA_000658575.1	NCBI: cellular organisms; Bacteria; Terrabacteria group; Actinobacteria; Actinomycetia; Corynebacteriales; Mycobacteriaceae; Mycobacterium; Mycobacterium tuberculosis complex; Mycobacterium tuberculosis;	NCBI: 1448467	4397369	99.94	0.00	Yes
NCBI Assembly	GCA_000659545.1	NCBI: cellular organisms; Bacteria; Terrabacteria group; Actinobacteria; Actinomycetia; Corynebacteriales; Mycobacteriaceae; Mycobacterium; Mycobacterium tuberculosis complex; Mycobacterium tuberculosis;	NCBI: 1295757	4403881	99.94	0.00	Yes
NCBI Assembly	GCA_000661125.1	NCBI: cellular organisms; Bacteria; Terrabacteria group; Actinobacteria; Actinomycetia; Corynebacteriales; Mycobacteriaceae; Mycobacterium; Mycobacterium tuberculosis complex; Mycobacterium tuberculosis;	NCBI: 1324263	4394277	99.94	0.00	Yes
NCBI Assembly	GCA_000660805.1	NCBI: cellular organisms; Bacteria; Terrabacteria group; Actinobacteria; Actinomycetia; Corynebacteriales; Mycobacteriaceae; Mycobacterium; Mycobacterium tuberculosis complex; Mycobacterium tuberculosis;	NCBI: 1324247	4418769	99.94	0.00	Yes
NCBI Assembly	GCA_000411895.1	NCBI: cellular organisms; Bacteria; Terrabacteria group; Actinobacteria; Actinomycetia; Corynebacteriales; Mycobacteriaceae; Mycobacterium; Mycobacterium tuberculosis complex; Mycobacterium tuberculosis;	NCBI: 1322343	4169405	96.26	0.17	Yes
NCBI Assembly	GCA_000523655.1	NCBI: cellular organisms; Bacteria; Terrabacteria group; Actinobacteria; Actinomycetia; Corynebacteriales; Mycobacteriaceae; Mycobacterium; Mycobacterium avium complex (MAC); Mycobacterium avium;	NCBI: 1335418	5320946	99.90	1.00	Yes
NCBI Assembly	GCA_000651855.1	NCBI: cellular organisms; Bacteria; Terrabacteria group; Actinobacteria; Actinomycetia; Corynebacteriales; Mycobacteriaceae; Mycobacterium; Mycobacterium tuberculosis complex; Mycobacterium tuberculosis;	NCBI: 1448428	4419169	99.94	0.00	Yes

NCBI Assembly	GCA_000662965.1	NCBI: cellular organisms; Bacteria; Terrabacteria group; Actinobacteria; Actinomycetia; Corynebacteriales; Mycobacteriaceae; Mycobacterium; Mycobacterium tuberculosis complex; Mycobacterium tuberculosis;	NCBI: 1448681	4420167	99.94	0.00	Yes
NCBI Assembly	GCA_000260615.2	NCBI: cellular organisms; Bacteria; Terrabacteria group; Actinobacteria; Actinomycetia; Corynebacteriales; Mycobacteriaceae; Mycobacteroides; Mycobacteroides abscessus;	NCBI: 1162284	5488620	100.00	0.25	Yes
NCBI Assembly	GCA_000655935.1	NCBI: cellular organisms; Bacteria; Terrabacteria group; Actinobacteria; Actinomycetia; Corynebacteriales; Mycobacteriaceae; Mycobacterium; Mycobacterium tuberculosis complex; Mycobacterium tuberculosis;	NCBI: 1427305	4395657	99.94	0.00	Yes
NCBI Assembly	GCA_000649355.1	NCBI: cellular organisms; Bacteria; Terrabacteria group; Actinobacteria; Actinomycetia; Corynebacteriales; Mycobacteriaceae; Mycobacterium; Mycobacterium tuberculosis complex; Mycobacterium tuberculosis;	NCBI: 1438851	4621583	99.94	8.64	No
NCBI Assembly	GCA_000662645.1	NCBI: cellular organisms; Bacteria; Terrabacteria group; Actinobacteria; Actinomycetia; Corynebacteriales; Mycobacteriaceae; Mycobacterium; Mycobacterium tuberculosis complex; Mycobacterium tuberculosis;	NCBI: 1448665	4414215	99.94	0.00	Yes
NCBI Assembly	GCA_000661145.1	NCBI: cellular organisms; Bacteria; Terrabacteria group; Actinobacteria; Actinomycetia; Corynebacteriales; Mycobacteriaceae; Mycobacterium; Mycobacterium tuberculosis complex; Mycobacterium tuberculosis;	NCBI: 1324264	4415580	99.94	0.00	Yes
NCBI Assembly	GCA_000658595.1	NCBI: cellular organisms; Bacteria; Terrabacteria group; Actinobacteria; Actinomycetia; Corynebacteriales; Mycobacteriaceae; Mycobacterium; Mycobacterium tuberculosis complex; Mycobacterium tuberculosis;	NCBI: 1448490	4404560	99.94	0.00	Yes
NCBI Assembly	GCA_000660825.1	NCBI: cellular organisms; Bacteria; Terrabacteria group; Actinobacteria; Actinomycetia; Corynebacteriales; Mycobacteriaceae; Mycobacterium; Mycobacterium tuberculosis complex; Mycobacterium tuberculosis;	NCBI: 1324248	4402320	99.94	0.00	Yes
NCBI Assembly	GCA_000659565.1	NCBI: cellular organisms; Bacteria; Terrabacteria group; Actinobacteria; Actinomycetia; Corynebacteriales; Mycobacteriaceae; Mycobacterium; Mycobacterium tuberculosis complex; Mycobacterium tuberculosis;	NCBI: 1295758	4405634	99.94	0.00	Yes
NCBI Assembly	GCA_000416365.2	NCBI: cellular organisms; Bacteria; Terrabacteria group; Actinobacteria; Actinomycetia; Corynebacteriales; Mycobacteriaceae; Mycobacterium; unclassified Mycobacterium;	NCBI: 1273687	6324222	99.81	0.50	Yes
NCBI Assembly	GCA_000523675.1	NCBI: cellular organisms; Bacteria; Terrabacteria group; Actinobacteria; Actinomycetia; Corynebacteriales; Mycobacteriaceae; Mycobacterium; Mycobacterium avium complex (MAC); Mycobacterium avium; Mycobacterium avium subsp. avium;	NCBI: 1299330	5363719	93.83	0.33	Yes
NCBI Assembly	GCA_000651875.1	NCBI: cellular organisms; Bacteria; Terrabacteria group; Actinobacteria; Actinomycetia; Corynebacteriales; Mycobacteriaceae; Mycobacterium; Mycobacterium tuberculosis complex; Mycobacterium tuberculosis;	NCBI: 1448430	4411408	99.94	0.00	Yes
NCBI Assembly	GCA_000662985.1	NCBI: cellular organisms; Bacteria; Terrabacteria group; Actinobacteria; Actinomycetia; Corynebacteriales; Mycobacteriaceae; Mycobacterium; Mycobacterium tuberculosis complex; Mycobacterium tuberculosis;	NCBI: 1448682	4416995	99.94	0.00	Yes
NCBI Assembly	GCA_000662665.1	NCBI: cellular organisms; Bacteria; Terrabacteria group; Actinobacteria; Actinomycetia; Corynebacteriales; Mycobacteriaceae; Mycobacterium; Mycobacterium tuberculosis complex; Mycobacterium tuberculosis;	NCBI: 1448666	4424307	99.94	0.00	Yes
NCBI Assembly	GCA_000649375.1	NCBI: cellular organisms; Bacteria; Terrabacteria group; Actinobacteria; Actinomycetia; Corynebacteriales; Mycobacteriaceae; Mycobacterium; Mycobacterium tuberculosis complex; Mycobacterium tuberculosis;	NCBI: 1438849	4451087	99.88	3.22	Yes
NCBI Assembly	GCA_000655955.1	NCBI: cellular organisms; Bacteria; Terrabacteria group; Actinobacteria; Actinomycetia; Corynebacteriales; Mycobacteriaceae; Mycobacterium; Mycobacterium tuberculosis complex; Mycobacterium tuberculosis;	NCBI: 1427306	4417257	99.94	0.00	Yes
NCBI Assembly	GCA_000261085.1	NCBI: cellular organisms; Bacteria; Terrabacteria group; Actinobacteria; Actinomycetia; Corynebacteriales; Mycobacteriaceae; Mycobacteroides; Mycobacteroides abscessus; Mycobacteroides abscessus subsp. bolletii;	NCBI: 1162283	4886939	100.00	0.00	Yes
NCBI Assembly	GCA_000661165.1	NCBI: cellular organisms; Bacteria; Terrabacteria group; Actinobacteria; Actinomycetia; Corynebacteriales; Mycobacteriaceae; Mycobacterium; Mycobacterium tuberculosis complex; Mycobacterium tuberculosis;	NCBI: 1324265	4420588	99.94	0.00	Yes

NCBI Assembly	GCA_000658615.1	NCBI: cellular organisms; Bacteria; Terrabacteria group; Actinobacteria; Actinomycetia; Corynebacteriales; Mycobacteriaceae; Mycobacterium; Mycobacterium tuberculosis complex; Mycobacterium tuberculosis;	NCBI: 1448516	4438623	99.94	0.00	Yes
NCBI Assembly	GCA_000660845.1	NCBI: cellular organisms; Bacteria; Terrabacteria group; Actinobacteria; Actinomycetia; Corynebacteriales; Mycobacteriaceae; Mycobacterium; Mycobacterium tuberculosis complex; Mycobacterium tuberculosis;	NCBI: 1324249	4441236	99.94	0.00	Yes
NCBI Assembly	GCA_000659585.1	NCBI: cellular organisms; Bacteria; Terrabacteria group; Actinobacteria; Actinomycetia; Corynebacteriales; Mycobacteriaceae; Mycobacterium; Mycobacterium tuberculosis complex; Mycobacterium tuberculosis;	NCBI: 1295759	4411263	99.94	0.17	Yes
NCBI Assembly	GCA_000416385.1	NCBI: cellular organisms; Bacteria; Terrabacteria group; Actinobacteria; Actinomycetia; Corynebacteriales; Mycobacteriaceae; Mycobacterium; unclassified Mycobacterium;	NCBI: 1273686	5413599	100.00	1.44	Yes
NCBI Assembly	GCA_000523695.1	NCBI: cellular organisms; Bacteria; Terrabacteria group; Actinobacteria; Actinomycetia; Corynebacteriales; Mycobacteriaceae; Mycobacterium; Mycobacterium xenopi;	NCBI: 1299334	5477373	77.75	6.70	No
NCBI Assembly	GCA_000663005.1	NCBI: cellular organisms; Bacteria; Terrabacteria group; Actinobacteria; Actinomycetia; Corynebacteriales; Mycobacteriaceae; Mycobacterium; Mycobacterium tuberculosis complex; Mycobacterium tuberculosis;	NCBI: 1448683	4426952	99.94	0.50	Yes
NCBI Assembly	GCA_000651895.1	NCBI: cellular organisms; Bacteria; Terrabacteria group; Actinobacteria; Actinomycetia; Corynebacteriales; Mycobacteriaceae; Mycobacterium; Mycobacterium tuberculosis complex; Mycobacterium tuberculosis;	NCBI: 1448431	4457490	99.94	0.06	Yes
NCBI Assembly	GCA_000655975.1	NCBI: cellular organisms; Bacteria; Terrabacteria group; Actinobacteria; Actinomycetia; Corynebacteriales; Mycobacteriaceae; Mycobacterium; Mycobacterium tuberculosis complex; Mycobacterium tuberculosis;	NCBI: 1427307	4394807	99.61	0.00	Yes
NCBI Assembly	GCA_000662685.1	NCBI: cellular organisms; Bacteria; Terrabacteria group; Actinobacteria; Actinomycetia; Corynebacteriales; Mycobacteriaceae; Mycobacterium; Mycobacterium tuberculosis complex; Mycobacterium tuberculosis;	NCBI: 1448667	4422335	99.61	0.33	Yes
NCBI Assembly	GCA_000661185.1	NCBI: cellular organisms; Bacteria; Terrabacteria group; Actinobacteria; Actinomycetia; Corynebacteriales; Mycobacteriaceae; Mycobacterium; Mycobacterium tuberculosis complex; Mycobacterium tuberculosis;	NCBI: 1324266	4400475	99.94	0.00	Yes
NCBI Assembly	GCA_000649395.1	NCBI: cellular organisms; Bacteria; Terrabacteria group; Actinobacteria; Actinomycetia; Corynebacteriales; Mycobacteriaceae; Mycobacterium; Mycobacterium tuberculosis complex; Mycobacterium tuberculosis;	NCBI: 1438848	4382585	99.94	0.33	Yes
NCBI Assembly	GCA_000261105.1	NCBI: cellular organisms; Bacteria; Terrabacteria group; Actinobacteria; Actinomycetia; Corynebacteriales; Mycobacteriaceae; Mycobacteroides; Mycobacteroides abscessus;	NCBI: 1162285	4935864	100.00	0.00	Yes
NCBI Assembly	GCA_000658635.1	NCBI: cellular organisms; Bacteria; Terrabacteria group; Actinobacteria; Actinomycetia; Corynebacteriales; Mycobacteriaceae; Mycobacterium; Mycobacterium tuberculosis complex; Mycobacterium tuberculosis;	NCBI: 1448527	4423731	99.94	1.11	Yes
NCBI Assembly	GCA_000659605.1	NCBI: cellular organisms; Bacteria; Terrabacteria group; Actinobacteria; Actinomycetia; Corynebacteriales; Mycobacteriaceae; Mycobacterium; Mycobacterium tuberculosis complex; Mycobacterium tuberculosis;	NCBI: 1295760	4397990	99.94	0.00	Yes
NCBI Assembly	GCA_000660865.1	NCBI: cellular organisms; Bacteria; Terrabacteria group; Actinobacteria; Actinomycetia; Corynebacteriales; Mycobacteriaceae; Mycobacterium; Mycobacterium tuberculosis complex; Mycobacterium tuberculosis;	NCBI: 1324250	4415970	99.94	0.00	Yes
NCBI Assembly	GCA_000417925.1	NCBI: cellular organisms; Bacteria; Terrabacteria group; Actinobacteria; Actinomycetia; Corynebacteriales; Mycobacteriaceae; Mycobacterium; Mycobacterium tuberculosis complex; Mycobacterium tuberculosis;	NCBI: 1341711	4391869	99.94	0.00	Yes
NCBI Assembly	GCA_000523715.1	NCBI: cellular organisms; Bacteria; Terrabacteria group; Actinobacteria; Actinomycetia; Corynebacteriales; Mycobacteriaceae; Mycobacterium; Mycobacterium xenopi;	NCBI: 1299333	5287678	78.35	2.42	No
NCBI Assembly	GCA_000651915.1	NCBI: cellular organisms; Bacteria; Terrabacteria group; Actinobacteria; Actinomycetia; Corynebacteriales; Mycobacteriaceae; Mycobacterium; Mycobacterium tuberculosis complex; Mycobacterium tuberculosis;	NCBI: 1448432	4401123	99.94	0.00	Yes

NCBI Assembly	GCA_000655995.1	NCBI: cellular organisms; Bacteria; Terrabacteria group; Actinobacteria; Actinomycetia; Corynebacteriales; Mycobacteriaceae; Mycobacterium; Mycobacterium tuberculosis complex; Mycobacterium tuberculosis;	NCBI: 1427308	4414450	99.94	0.00	Yes
NCBI Assembly	GCA_000663025.1	NCBI: cellular organisms; Bacteria; Terrabacteria group; Actinobacteria; Actinomycetia; Corynebacteriales; Mycobacteriaceae; Mycobacterium; Mycobacterium tuberculosis complex; Mycobacterium tuberculosis;	NCBI: 1448684	4428589	99.61	0.00	Yes
NCBI Assembly	GCA_000649415.1	NCBI: cellular organisms; Bacteria; Terrabacteria group; Actinobacteria; Actinomycetia; Corynebacteriales; Mycobacteriaceae; Mycobacterium; Mycobacterium tuberculosis complex; Mycobacterium tuberculosis;	NCBI: 1438846	4418377	99.61	0.00	Yes
NCBI Assembly	GCA_000661205.1	NCBI: cellular organisms; Bacteria; Terrabacteria group; Actinobacteria; Actinomycetia; Corynebacteriales; Mycobacteriaceae; Mycobacterium; Mycobacterium tuberculosis complex; Mycobacterium tuberculosis;	NCBI: 1324267	4443529	99.61	0.00	Yes
NCBI Assembly	GCA_000261125.1	NCBI: cellular organisms; Bacteria; Terrabacteria group; Actinobacteria; Actinomycetia; Corynebacteriales; Mycobacteriaceae; Mycobacteroides; Mycobacteroides abscessus;	NCBI: 1162286	5204460	100.00	0.00	Yes
NCBI Assembly	GCA_000662705.1	NCBI: cellular organisms; Bacteria; Terrabacteria group; Actinobacteria; Actinomycetia; Corynebacteriales; Mycobacteriaceae; Mycobacterium; Mycobacterium tuberculosis complex; Mycobacterium tuberculosis;	NCBI: 1448668	4445683	99.94	0.00	Yes
NCBI Assembly	GCA_000658655.1	NCBI: cellular organisms; Bacteria; Terrabacteria group; Actinobacteria; Actinomycetia; Corynebacteriales; Mycobacteriaceae; Mycobacterium; Mycobacterium tuberculosis complex; Mycobacterium tuberculosis;	NCBI: 1448534	4406298	99.94	0.33	Yes
NCBI Assembly	GCA_000660885.1	NCBI: cellular organisms; Bacteria; Terrabacteria group; Actinobacteria; Actinomycetia; Corynebacteriales; Mycobacteriaceae; Mycobacterium; Mycobacterium tuberculosis complex; Mycobacterium tuberculosis;	NCBI: 1324251	4404469	99.94	0.00	Yes
NCBI Assembly	GCA_000659625.1	NCBI: cellular organisms; Bacteria; Terrabacteria group; Actinobacteria; Actinomycetia; Corynebacteriales; Mycobacteriaceae; Mycobacterium; Mycobacterium tuberculosis complex; Mycobacterium tuberculosis;	NCBI: 1295763	4404458	99.94	0.00	Yes
NCBI Assembly	GCA_000418575.1	NCBI: cellular organisms; Bacteria; Terrabacteria group; Actinobacteria; Actinomycetia; Corynebacteriales; Mycobacteriaceae; Mycobacteroides; Mycobacteroides abscessus; Mycobacteroides abscessus subsp. bolletii;	NCBI: 1306401	4840000	99.89	0.17	Yes
NCBI Assembly	GCA_000656015.1	NCBI: cellular organisms; Bacteria; Terrabacteria group; Actinobacteria; Actinomycetia; Corynebacteriales; Mycobacteriaceae; Mycobacterium; Mycobacterium tuberculosis complex; Mycobacterium tuberculosis;	NCBI: 1427309	4426747	99.94	0.00	Yes
NCBI Assembly	GCA_000651935.1	NCBI: cellular organisms; Bacteria; Terrabacteria group; Actinobacteria; Actinomycetia; Corynebacteriales; Mycobacteriaceae; Mycobacterium; Mycobacterium tuberculosis complex; Mycobacterium tuberculosis;	NCBI: 1448433	4400853	99.94	0.00	Yes
NCBI Assembly	GCA_000523735.1	NCBI: cellular organisms; Bacteria; Terrabacteria group; Actinobacteria; Actinomycetia; Corynebacteriales; Mycobacteriaceae; Mycobacteroides; Mycobacteroides abscessus;	NCBI: 1335412	4877071	99.97	1.78	Yes
NCBI Assembly	GCA_000663045.1	NCBI: cellular organisms; Bacteria; Terrabacteria group; Actinobacteria; Actinomycetia; Corynebacteriales; Mycobacteriaceae; Mycobacterium; Mycobacterium tuberculosis complex; Mycobacterium tuberculosis;	NCBI: 1448685	4425718	99.94	0.00	Yes
NCBI Assembly	GCA_000649435.1	NCBI: cellular organisms; Bacteria; Terrabacteria group; Actinobacteria; Actinomycetia; Corynebacteriales; Mycobacteriaceae; Mycobacterium; Mycobacterium tuberculosis complex; Mycobacterium tuberculosis;	NCBI: 1438870	4409718	99.94	0.89	Yes
NCBI Assembly	GCA_000661225.1	NCBI: cellular organisms; Bacteria; Terrabacteria group; Actinobacteria; Actinomycetia; Corynebacteriales; Mycobacteriaceae; Mycobacterium; Mycobacterium tuberculosis complex; Mycobacterium tuberculosis;	NCBI: 1324268	4432102	99.94	0.00	Yes
NCBI Assembly	GCA_000263135.1	NCBI: cellular organisms; Bacteria; Terrabacteria group; Actinobacteria; Actinomycetia; Corynebacteriales; Mycobacteriaceae; Mycobacterium; Mycobacterium tuberculosis complex; Mycobacterium tuberculosis;	NCBI: 757419	4412883	99.28	0.00	Yes
NCBI Assembly	GCA_000662725.1	NCBI: cellular organisms; Bacteria; Terrabacteria group; Actinobacteria; Actinomycetia; Corynebacteriales; Mycobacteriaceae; Mycobacterium; Mycobacterium tuberculosis complex; Mycobacterium tuberculosis;	NCBI: 1448669	4433460	99.94	0.00	Yes

NCBI Assembly	GCA_000658675.1	NCBI: cellular organisms; Bacteria; Terrabacteria group; Actinobacteria; Actinomycetia; Corynebacteriales; Mycobacteriaceae; Mycobacterium; Mycobacterium tuberculosis complex; Mycobacterium tuberculosis;	NCBI: 1448539	4419686	99.94	0.00	Yes
NCBI Assembly	GCA_000659645.1	NCBI: cellular organisms; Bacteria; Terrabacteria group; Actinobacteria; Actinomycetia; Corynebacteriales; Mycobacteriaceae; Mycobacterium; Mycobacterium tuberculosis complex; Mycobacterium tuberculosis;	NCBI: 1295764	4399592	99.94	0.00	Yes
NCBI Assembly	GCA_000419295.1	NCBI: cellular organisms; Bacteria; Terrabacteria group; Actinobacteria; Actinomycetia; Corynebacteriales; Mycobacteriaceae; Mycobacterium; unclassified Mycobacterium;	NCBI: 1187065	5759365	99.54	0.40	Yes
NCBI Assembly	GCA_000660905.1	NCBI: cellular organisms; Bacteria; Terrabacteria group; Actinobacteria; Actinomycetia; Corynebacteriales; Mycobacteriaceae; Mycobacterium; Mycobacterium tuberculosis complex; Mycobacterium tuberculosis;	NCBI: 1324252	4436783	99.94	0.00	Yes
NCBI Assembly	GCA_000656035.1	NCBI: cellular organisms; Bacteria; Terrabacteria group; Actinobacteria; Actinomycetia; Corynebacteriales; Mycobacteriaceae; Mycobacterium; Mycobacterium tuberculosis complex; Mycobacterium tuberculosis;	NCBI: 1427310	4397501	99.94	0.00	Yes
NCBI Assembly	GCA_000523755.1	NCBI: cellular organisms; Bacteria; Terrabacteria group; Actinobacteria; Actinomycetia; Corynebacteriales; Mycobacteriaceae; Mycobacterium; Mycobacterium avium complex (MAC); Mycobacterium avium;	NCBI: 1335419	5628496	99.94	1.09	Yes
NCBI Assembly	GCA_000651955.1	NCBI: cellular organisms; Bacteria; Terrabacteria group; Actinobacteria; Actinomycetia; Corynebacteriales; Mycobacteriaceae; Mycobacterium; Mycobacterium tuberculosis complex; Mycobacterium tuberculosis;	NCBI: 1448434	4416255	99.94	0.00	Yes
NCBI Assembly	GCA_000663065.1	NCBI: cellular organisms; Bacteria; Terrabacteria group; Actinobacteria; Actinomycetia; Corynebacteriales; Mycobacteriaceae; Mycobacterium; Mycobacterium tuberculosis complex; Mycobacterium tuberculosis;	NCBI: 1448686	4430569	99.94	0.00	Yes
NCBI Assembly	GCA_000649455.1	NCBI: cellular organisms; Bacteria; Terrabacteria group; Actinobacteria; Actinomycetia; Corynebacteriales; Mycobacteriaceae; Mycobacterium; Mycobacterium tuberculosis complex; Mycobacterium tuberculosis;	NCBI: 1438868	4411655	99.94	0.00	Yes
NCBI Assembly	GCA_000661245.1	NCBI: cellular organisms; Bacteria; Terrabacteria group; Actinobacteria; Actinomycetia; Corynebacteriales; Mycobacteriaceae; Mycobacterium; Mycobacterium tuberculosis complex; Mycobacterium tuberculosis;	NCBI: 1324270	4417077	99.94	0.00	Yes
NCBI Assembly	GCA_000662745.1	NCBI: cellular organisms; Bacteria; Terrabacteria group; Actinobacteria; Actinomycetia; Corynebacteriales; Mycobacteriaceae; Mycobacterium; Mycobacterium tuberculosis complex; Mycobacterium tuberculosis;	NCBI: 1448670	4433609	99.94	0.00	Yes
NCBI Assembly	GCA_000658695.1	NCBI: cellular organisms; Bacteria; Terrabacteria group; Actinobacteria; Actinomycetia; Corynebacteriales; Mycobacteriaceae; Mycobacterium; Mycobacterium tuberculosis complex; Mycobacterium tuberculosis;	NCBI: 1448564	4444263	99.94	1.78	Yes
NCBI Assembly	GCA_000660925.1	NCBI: cellular organisms; Bacteria; Terrabacteria group; Actinobacteria; Actinomycetia; Corynebacteriales; Mycobacteriaceae; Mycobacterium; Mycobacterium tuberculosis complex; Mycobacterium tuberculosis;	NCBI: 1324253	4400834	99.94	0.00	Yes
NCBI Assembly	GCA_000419315.1	NCBI: cellular organisms; Bacteria; Terrabacteria group; Actinobacteria; Actinomycetia; Corynebacteriales; Mycobacteriaceae; Mycobacterium; Mycobacterium marinum;	NCBI: 1229516	6029340	99.88	0.07	Yes
NCBI Assembly	GCA_000659665.1	NCBI: cellular organisms; Bacteria; Terrabacteria group; Actinobacteria; Actinomycetia; Corynebacteriales; Mycobacteriaceae; Mycobacterium; Mycobacterium tuberculosis complex; Mycobacterium tuberculosis;	NCBI: 1295765	4401928	99.94	0.00	Yes
NCBI Assembly	GCA_000656055.1	NCBI: cellular organisms; Bacteria; Terrabacteria group; Actinobacteria; Actinomycetia; Corynebacteriales; Mycobacteriaceae; Mycobacterium; Mycobacterium tuberculosis complex; Mycobacterium tuberculosis;	NCBI: 1427311	4430445	99.94	0.00	Yes
NCBI Assembly	GCA_000651975.1	NCBI: cellular organisms; Bacteria; Terrabacteria group; Actinobacteria; Actinomycetia; Corynebacteriales; Mycobacteriaceae; Mycobacterium; Mycobacterium tuberculosis complex; Mycobacterium tuberculosis;	NCBI: 1448435	4442427	99.94	0.00	Yes
NCBI Assembly	GCA_000663085.1	NCBI: cellular organisms; Bacteria; Terrabacteria group; Actinobacteria; Actinomycetia; Corynebacteriales; Mycobacteriaceae; Mycobacterium; Mycobacterium tuberculosis complex; Mycobacterium tuberculosis;	NCBI: 1448687	4436263	99.94	0.33	Yes

NCBI Assembly	GCA_000523775.1	NCBI: cellular organisms; Bacteria; Terrabacteria group; Actinobacteria; Actinomycetia; Corynebacteriales; Mycobacteriaceae; Mycobacteroides; Mycobacteroides abscessus;	NCBI: 1335409	4759632	98.65	0.00	Yes
NCBI Assembly	GCA_000649475.1	NCBI: cellular organisms; Bacteria; Terrabacteria group; Actinobacteria; Actinomycetia; Corynebacteriales; Mycobacteriaceae; Mycobacterium; Mycobacterium tuberculosis complex; Mycobacterium tuberculosis;	NCBI: 1438845	4401032	99.94	0.00	Yes
NCBI Assembly	GCA_000661265.1	NCBI: cellular organisms; Bacteria; Terrabacteria group; Actinobacteria; Actinomycetia; Corynebacteriales; Mycobacteriaceae; Mycobacterium; Mycobacterium tuberculosis complex; Mycobacterium tuberculosis;	NCBI: 1324271	4418114	99.94	0.00	Yes
NCBI Assembly	GCA_000662765.1	NCBI: cellular organisms; Bacteria; Terrabacteria group; Actinobacteria; Actinomycetia; Corynebacteriales; Mycobacteriaceae; Mycobacterium; Mycobacterium tuberculosis complex; Mycobacterium tuberculosis;	NCBI: 1448671	4382710	99.94	0.07	Yes
NCBI Assembly	GCA_000270345.1	NCBI: cellular organisms; Bacteria; Terrabacteria group; Actinobacteria; Actinomycetia; Corynebacteriales; Mycobacteriaceae; Mycobacterium; Mycobacterium tuberculosis complex; Mycobacterium tuberculosis;	NCBI: 443149	4398812	98.45	0.00	Yes
NCBI Assembly	GCA_000658715.1	NCBI: cellular organisms; Bacteria; Terrabacteria group; Actinobacteria; Actinomycetia; Corynebacteriales; Mycobacteriaceae; Mycobacterium; Mycobacterium tuberculosis complex; Mycobacterium tuberculosis;	NCBI: 1448566	4425907	99.78	0.84	Yes
NCBI Assembly	GCA_000419335.1	NCBI: cellular organisms; Bacteria; Terrabacteria group; Actinobacteria; Actinomycetia; Corynebacteriales; Mycobacteriaceae; Mycobacterium; Mycobacterium marinum;	NCBI: 1288036	6134390	99.54	0.40	Yes
NCBI Assembly	GCA_000660945.1	NCBI: cellular organisms; Bacteria; Terrabacteria group; Actinobacteria; Actinomycetia; Corynebacteriales; Mycobacteriaceae; Mycobacterium; Mycobacterium tuberculosis complex; Mycobacterium tuberculosis;	NCBI: 1324254	4414945	99.94	0.42	Yes
NCBI Assembly	GCA_000659685.1	NCBI: cellular organisms; Bacteria; Terrabacteria group; Actinobacteria; Actinomycetia; Corynebacteriales; Mycobacteriaceae; Mycobacterium; Mycobacterium tuberculosis complex; Mycobacterium tuberculosis;	NCBI: 1295766	4402430	99.94	0.00	Yes
NCBI Assembly	GCA_000651995.1	NCBI: cellular organisms; Bacteria; Terrabacteria group; Actinobacteria; Actinomycetia; Corynebacteriales; Mycobacteriaceae; Mycobacterium; Mycobacterium tuberculosis complex; Mycobacterium tuberculosis;	NCBI: 1448437	4418999	99.61	0.00	Yes
NCBI Assembly	GCA_000656075.1	NCBI: cellular organisms; Bacteria; Terrabacteria group; Actinobacteria; Actinomycetia; Corynebacteriales; Mycobacteriaceae; Mycobacterium; Mycobacterium tuberculosis complex; Mycobacterium tuberculosis;	NCBI: 1427312	4425556	99.94	0.00	Yes
NCBI Assembly	GCA_000523795.1	NCBI: cellular organisms; Bacteria; Terrabacteria group; Actinobacteria; Actinomycetia; Corynebacteriales; Mycobacteriaceae; Mycobacterium; Mycobacterium avium complex (MAC); Mycobacterium intracellulare;	NCBI: 1335422	5728455	99.72	0.75	Yes
NCBI Assembly	GCA_000663105.1	NCBI: cellular organisms; Bacteria; Terrabacteria group; Actinobacteria; Actinomycetia; Corynebacteriales; Mycobacteriaceae; Mycobacterium; Mycobacterium tuberculosis complex; Mycobacterium tuberculosis;	NCBI: 1448688	4422977	99.94	0.00	Yes
NCBI Assembly	GCA_000649495.1	NCBI: cellular organisms; Bacteria; Terrabacteria group; Actinobacteria; Actinomycetia; Corynebacteriales; Mycobacteriaceae; Mycobacterium; Mycobacterium tuberculosis complex; Mycobacterium tuberculosis;	NCBI: 1438844	4397667	99.94	0.00	Yes
NCBI Assembly	GCA_000661285.1	NCBI: cellular organisms; Bacteria; Terrabacteria group; Actinobacteria; Actinomycetia; Corynebacteriales; Mycobacteriaceae; Mycobacterium; Mycobacterium tuberculosis complex; Mycobacterium tuberculosis;	NCBI: 1324272	4412080	99.94	0.00	Yes
NCBI Assembly	GCA_000270565.1	NCBI: cellular organisms; Bacteria; Terrabacteria group; Actinobacteria; Actinomycetia; Corynebacteriales; Mycobacteriaceae; Mycobacteroides; Mycobacteroides abscessus;	NCBI: 1001720	5273535	99.78	0.00	Yes
NCBI Assembly	GCA_000422125.1	NCBI: cellular organisms; Bacteria; Terrabacteria group; Actinobacteria; Actinomycetia; Corynebacteriales; Mycobacteriaceae; Mycobacterium; Mycobacterium tuberculosis complex; Mycobacterium tuberculosis;	NCBI: 1306414	4391174	99.94	0.00	Yes
NCBI Assembly	GCA_000662785.1	NCBI: cellular organisms; Bacteria; Terrabacteria group; Actinobacteria; Actinomycetia; Corynebacteriales; Mycobacteriaceae; Mycobacterium; Mycobacterium tuberculosis complex; Mycobacterium tuberculosis;	NCBI: 1448672	4415128	99.94	0.00	Yes

NCBI Assembly	GCA_000658735.1	NCBI: cellular organisms; Bacteria; Terrabacteria group; Actinobacteria; Actinomycetia; Corynebacteriales; Mycobacteriaceae; Mycobacterium; Mycobacterium tuberculosis complex; Mycobacterium tuberculosis;	NCBI: 1448573	4421391	99.89	1.10	Yes
NCBI Assembly	GCA_000660965.1	NCBI: cellular organisms; Bacteria; Terrabacteria group; Actinobacteria; Actinomycetia; Corynebacteriales; Mycobacteriaceae; Mycobacterium; Mycobacterium tuberculosis complex; Mycobacterium tuberculosis;	NCBI: 1324255	4404295	99.94	0.00	Yes
NCBI Assembly	GCA_000659705.1	NCBI: cellular organisms; Bacteria; Terrabacteria group; Actinobacteria; Actinomycetia; Corynebacteriales; Mycobacteriaceae; Mycobacterium; Mycobacterium tuberculosis complex; Mycobacterium tuberculosis;	NCBI: 1295767	4407234	99.94	0.00	Yes
NCBI Assembly	GCA_000656095.1	NCBI: cellular organisms; Bacteria; Terrabacteria group; Actinobacteria; Actinomycetia; Corynebacteriales; Mycobacteriaceae; Mycobacterium; Mycobacterium tuberculosis complex; Mycobacterium tuberculosis;	NCBI: 1427313	4394931	99.94	0.00	Yes
NCBI Assembly	GCA_000523815.1	NCBI: cellular organisms; Bacteria; Terrabacteria group; Actinobacteria; Actinomycetia; Corynebacteriales; Mycobacteriaceae; Mycobacterium; Mycobacterium avium complex (MAC); Mycobacterium intracellulare;	NCBI: 1299331	5489059	94.63	0.08	Yes
NCBI Assembly	GCA_000652015.1	NCBI: cellular organisms; Bacteria; Terrabacteria group; Actinobacteria; Actinomycetia; Corynebacteriales; Mycobacteriaceae; Mycobacterium; Mycobacterium tuberculosis complex; Mycobacterium tuberculosis;	NCBI: 1448439	4410151	99.94	0.00	Yes
NCBI Assembly	GCA_000663125.1	NCBI: cellular organisms; Bacteria; Terrabacteria group; Actinobacteria; Actinomycetia; Corynebacteriales; Mycobacteriaceae; Mycobacterium; Mycobacterium tuberculosis complex; Mycobacterium tuberculosis;	NCBI: 1448689	4420606	99.94	0.00	Yes
NCBI Assembly	GCA_000649515.1	NCBI: cellular organisms; Bacteria; Terrabacteria group; Actinobacteria; Actinomycetia; Corynebacteriales; Mycobacteriaceae; Mycobacterium; Mycobacterium tuberculosis complex; Mycobacterium tuberculosis;	NCBI: 1438874	4397376	99.94	1.33	Yes
NCBI Assembly	GCA_000661305.1	NCBI: cellular organisms; Bacteria; Terrabacteria group; Actinobacteria; Actinomycetia; Corynebacteriales; Mycobacteriaceae; Mycobacterium; Mycobacterium tuberculosis complex; Mycobacterium tuberculosis;	NCBI: 1324273	4411062	99.94	0.00	Yes
NCBI Assembly	GCA_000270585.1	NCBI: cellular organisms; Bacteria; Terrabacteria group; Actinobacteria; Actinomycetia; Corynebacteriales; Mycobacteriaceae; Mycobacteroides; Mycobacteroides abscessus;	NCBI: 1001716	5233665	100.00	0.00	Yes
NCBI Assembly	GCA_000426065.1	NCBI: cellular organisms; Bacteria; Terrabacteria group; Actinobacteria; Actinomycetia; Corynebacteriales; Mycobacteriaceae; Mycobacterium; unclassified Mycobacterium;	NCBI: 1298864	6657122	99.43	1.13	Yes
NCBI Assembly	GCA_000662805.1	NCBI: cellular organisms; Bacteria; Terrabacteria group; Actinobacteria; Actinomycetia; Corynebacteriales; Mycobacteriaceae; Mycobacterium; Mycobacterium tuberculosis complex; Mycobacterium tuberculosis;	NCBI: 1448673	4422271	99.94	0.00	Yes
NCBI Assembly	GCA_000658755.1	NCBI: cellular organisms; Bacteria; Terrabacteria group; Actinobacteria; Actinomycetia; Corynebacteriales; Mycobacteriaceae; Mycobacterium; Mycobacterium tuberculosis complex; Mycobacterium tuberculosis;	NCBI: 1448575	4425475	99.94	0.82	Yes
NCBI Assembly	GCA_000660985.1	NCBI: cellular organisms; Bacteria; Terrabacteria group; Actinobacteria; Actinomycetia; Corynebacteriales; Mycobacteriaceae; Mycobacterium; Mycobacterium tuberculosis complex; Mycobacterium tuberculosis;	NCBI: 1324256	4403376	99.94	0.00	Yes
NCBI Assembly	GCA_000659725.1	NCBI: cellular organisms; Bacteria; Terrabacteria group; Actinobacteria; Actinomycetia; Corynebacteriales; Mycobacteriaceae; Mycobacterium; Mycobacterium tuberculosis complex; Mycobacterium tuberculosis;	NCBI: 1295769	4401623	99.94	0.00	Yes
NCBI Assembly	GCA_000652035.1	NCBI: cellular organisms; Bacteria; Terrabacteria group; Actinobacteria; Actinomycetia; Corynebacteriales; Mycobacteriaceae; Mycobacterium; Mycobacterium tuberculosis complex; Mycobacterium tuberculosis;	NCBI: 1448440	4427024	99.94	0.00	Yes
NCBI Assembly	GCA_000656115.1	NCBI: cellular organisms; Bacteria; Terrabacteria group; Actinobacteria; Actinomycetia; Corynebacteriales; Mycobacteriaceae; Mycobacterium; Mycobacterium tuberculosis complex; Mycobacterium tuberculosis;	NCBI: 1427314	4436257	99.94	0.00	Yes
NCBI Assembly	GCA_000523835.1	NCBI: cellular organisms; Bacteria; Terrabacteria group; Actinobacteria; Actinomycetia; Corynebacteriales; Mycobacteriaceae; Mycobacteroides; Mycobacteroides abscessus;	NCBI: 1299324	5030544	98.21	0.44	Yes

NCBI Assembly	GCA_000663145.1	NCBI: cellular organisms; Bacteria; Terrabacteria group; Actinobacteria; Actinomycetia; Corynebacteriales; Mycobacteriaceae; Mycobacterium; Mycobacterium tuberculosis complex; Mycobacterium tuberculosis;	NCBI: 1448690	4429181	99.94	0.00	Yes
NCBI Assembly	GCA_000649535.1	NCBI: cellular organisms; Bacteria; Terrabacteria group; Actinobacteria; Actinomycetia; Corynebacteriales; Mycobacteriaceae; Mycobacterium; Mycobacterium tuberculosis complex; Mycobacterium tuberculosis;	NCBI: 1438842	4394500	99.94	0.00	Yes
NCBI Assembly	GCA_000661325.1	NCBI: cellular organisms; Bacteria; Terrabacteria group; Actinobacteria; Actinomycetia; Corynebacteriales; Mycobacteriaceae; Mycobacterium; Mycobacterium tuberculosis complex; Mycobacterium tuberculosis;	NCBI: 1324274	4415203	99.94	0.00	Yes
NCBI Assembly	GCA_000270605.1	NCBI: cellular organisms; Bacteria; Terrabacteria group; Actinobacteria; Actinomycetia; Corynebacteriales; Mycobacteriaceae; Mycobacteroides; Mycobacteroides abscessus;	NCBI: 1001737	5341825	100.00	0.00	Yes
NCBI Assembly	GCA_000662825.1	NCBI: cellular organisms; Bacteria; Terrabacteria group; Actinobacteria; Actinomycetia; Corynebacteriales; Mycobacteriaceae; Mycobacterium; Mycobacterium tuberculosis complex; Mycobacterium tuberculosis;	NCBI: 1448674	4414576	99.94	0.00	Yes
NCBI Assembly	GCA_000658775.1	NCBI: cellular organisms; Bacteria; Terrabacteria group; Actinobacteria; Actinomycetia; Corynebacteriales; Mycobacteriaceae; Mycobacterium; Mycobacterium tuberculosis complex; Mycobacterium tuberculosis;	NCBI: 1448580	4422221	99.94	0.00	Yes
NCBI Assembly	GCA_000661005.1	NCBI: cellular organisms; Bacteria; Terrabacteria group; Actinobacteria; Actinomycetia; Corynebacteriales; Mycobacteriaceae; Mycobacterium; Mycobacterium tuberculosis complex; Mycobacterium tuberculosis;	NCBI: 1324257	4416226	99.94	0.00	Yes
NCBI Assembly	GCA_000442795.1	NCBI: cellular organisms; Bacteria; Terrabacteria group; Actinobacteria; Actinomycetia; Corynebacteriales; Mycobacteriaceae; Mycobacterium; Mycobacterium tuberculosis complex; Mycobacterium tuberculosis;	NCBI: 1266446	4398504	99.94	0.00	Yes
NCBI Assembly	GCA_000659745.1	NCBI: cellular organisms; Bacteria; Terrabacteria group; Actinobacteria; Actinomycetia; Corynebacteriales; Mycobacteriaceae; Mycobacterium; Mycobacterium tuberculosis complex; Mycobacterium tuberculosis;	NCBI: 1295771	4400813	99.94	0.00	Yes
NCBI Assembly	GCA_000656135.1	NCBI: cellular organisms; Bacteria; Terrabacteria group; Actinobacteria; Actinomycetia; Corynebacteriales; Mycobacteriaceae; Mycobacterium; Mycobacterium tuberculosis complex; Mycobacterium tuberculosis;	NCBI: 1427315	4404265	99.94	0.00	Yes
NCBI Assembly	GCA_000652055.1	NCBI: cellular organisms; Bacteria; Terrabacteria group; Actinobacteria; Actinomycetia; Corynebacteriales; Mycobacteriaceae; Mycobacterium; Mycobacterium tuberculosis complex; Mycobacterium tuberculosis;	NCBI: 1448442	4396993	99.94	0.26	Yes
NCBI Assembly	GCA_000663165.1	NCBI: cellular organisms; Bacteria; Terrabacteria group; Actinobacteria; Actinomycetia; Corynebacteriales; Mycobacteriaceae; Mycobacterium; Mycobacterium tuberculosis complex; Mycobacterium tuberculosis;	NCBI: 1448691	4401819	99.94	0.00	Yes
NCBI Assembly	GCA_000523855.1	NCBI: cellular organisms; Bacteria; Terrabacteria group; Actinobacteria; Actinomycetia; Corynebacteriales; Mycobacteriaceae; Mycobacteroides; Mycobacteroides abscessus;	NCBI: 1299323	5152492	97.89	0.13	Yes
NCBI Assembly	GCA_000649555.1	NCBI: cellular organisms; Bacteria; Terrabacteria group; Actinobacteria; Actinomycetia; Corynebacteriales; Mycobacteriaceae; Mycobacterium; Mycobacterium tuberculosis complex; Mycobacterium tuberculosis;	NCBI: 1438838	4419070	99.94	0.00	Yes
NCBI Assembly	GCA_000661345.1	NCBI: cellular organisms; Bacteria; Terrabacteria group; Actinobacteria; Actinomycetia; Corynebacteriales; Mycobacteriaceae; Mycobacterium; Mycobacterium tuberculosis complex; Mycobacterium tuberculosis;	NCBI: 1324275	4408941	99.94	0.00	Yes
NCBI Assembly	GCA_000270625.1	NCBI: cellular organisms; Bacteria; Terrabacteria group; Actinobacteria; Actinomycetia; Corynebacteriales; Mycobacteriaceae; Mycobacteroides; Mycobacteroides abscessus;	NCBI: 1001724	4841923	100.00	0.00	Yes
NCBI Assembly	GCA_000658795.1	NCBI: cellular organisms; Bacteria; Terrabacteria group; Actinobacteria; Actinomycetia; Corynebacteriales; Mycobacteriaceae; Mycobacterium; Mycobacterium tuberculosis complex; Mycobacterium tuberculosis;	NCBI: 1448598	4448770	99.94	1.90	Yes
NCBI Assembly	GCA_000442945.1	NCBI: cellular organisms; Bacteria; Terrabacteria group; Actinobacteria; Actinomycetia; Corynebacteriales; Mycobacteriaceae; Mycobacterium; Mycobacterium tuberculosis complex; Mycobacterium tuberculosis;	NCBI: 1262525	4402571	99.94	0.00	Yes

NCBI Assembly	GCA_000662845.1	NCBI: cellular organisms; Bacteria; Terrabacteria group; Actinobacteria; Actinomycetia; Corynebacteriales; Mycobacteriaceae; Mycobacterium; Mycobacterium tuberculosis complex; Mycobacterium tuberculosis;	NCBI: 1448675	4427864	99.94	0.00	Yes
NCBI Assembly	GCA_000661025.1	NCBI: cellular organisms; Bacteria; Terrabacteria group; Actinobacteria; Actinomycetia; Corynebacteriales; Mycobacteriaceae; Mycobacterium; Mycobacterium tuberculosis complex; Mycobacterium tuberculosis;	NCBI: 1324258	4421774	99.94	0.00	Yes
NCBI Assembly	GCA_000659765.1	NCBI: cellular organisms; Bacteria; Terrabacteria group; Actinobacteria; Actinomycetia; Corynebacteriales; Mycobacteriaceae; Mycobacterium; Mycobacterium tuberculosis complex; Mycobacterium tuberculosis;	NCBI: 1295772	4398883	99.94	0.00	Yes
NCBI Assembly	GCA_000652075.1	NCBI: cellular organisms; Bacteria; Terrabacteria group; Actinobacteria; Actinomycetia; Corynebacteriales; Mycobacteriaceae; Mycobacterium; Mycobacterium tuberculosis complex; Mycobacterium tuberculosis;	NCBI: 1448443	4404952	99.94	0.00	Yes
NCBI Assembly	GCA_000656155.1	NCBI: cellular organisms; Bacteria; Terrabacteria group; Actinobacteria; Actinomycetia; Corynebacteriales; Mycobacteriaceae; Mycobacterium; Mycobacterium tuberculosis complex; Mycobacterium tuberculosis;	NCBI: 1427316	4386234	99.94	0.00	Yes
NCBI Assembly	GCA_000523875.1	NCBI: cellular organisms; Bacteria; Terrabacteria group; Actinobacteria; Actinomycetia; Corynebacteriales; Mycobacteriaceae; Mycobacteroides; Mycobacteroides abscessus; Mycobacteroides abscessus subsp. bolletii;	NCBI: 1299321	5258802	96.89	0.00	Yes
NCBI Assembly	GCA_000649575.1	NCBI: cellular organisms; Bacteria; Terrabacteria group; Actinobacteria; Actinomycetia; Corynebacteriales; Mycobacteriaceae; Mycobacterium; Mycobacterium tuberculosis complex; Mycobacterium tuberculosis;	NCBI: 1438837	4417676	99.94	0.00	Yes
NCBI Assembly	GCA_000663185.1	NCBI: cellular organisms; Bacteria; Terrabacteria group; Actinobacteria; Actinomycetia; Corynebacteriales; Mycobacteriaceae; Mycobacterium; Mycobacterium tuberculosis complex; Mycobacterium tuberculosis;	NCBI: 1448692	4412154	99.94	0.00	Yes
NCBI Assembly	GCA_000270645.1	NCBI: cellular organisms; Bacteria; Terrabacteria group; Actinobacteria; Actinomycetia; Corynebacteriales; Mycobacteriaceae; Mycobacteroides; Mycobacteroides abscessus;	NCBI: 1001739	5144692	100.00	0.00	Yes
NCBI Assembly	GCA_000661365.1	NCBI: cellular organisms; Bacteria; Terrabacteria group; Actinobacteria; Actinomycetia; Corynebacteriales; Mycobacteriaceae; Mycobacterium; Mycobacterium tuberculosis complex; Mycobacterium tuberculosis;	NCBI: 1324276	4413078	99.94	0.00	Yes
NCBI Assembly	GCA_000658815.1	NCBI: cellular organisms; Bacteria; Terrabacteria group; Actinobacteria; Actinomycetia; Corynebacteriales; Mycobacteriaceae; Mycobacterium; Mycobacterium tuberculosis complex; Mycobacterium tuberculosis;	NCBI: 1448606	4416037	99.94	0.00	Yes
NCBI Assembly	GCA_000442965.1	NCBI: cellular organisms; Bacteria; Terrabacteria group; Actinobacteria; Actinomycetia; Corynebacteriales; Mycobacteriaceae; Mycobacterium; Mycobacterium tuberculosis complex; Mycobacterium tuberculosis;	NCBI: 1262526	4408178	99.94	0.00	Yes
NCBI Assembly	GCA_000661045.1	NCBI: cellular organisms; Bacteria; Terrabacteria group; Actinobacteria; Actinomycetia; Corynebacteriales; Mycobacteriaceae; Mycobacterium; Mycobacterium tuberculosis complex; Mycobacterium tuberculosis;	NCBI: 1324259	4426198	99.94	0.00	Yes
NCBI Assembly	GCA_000652095.1	NCBI: cellular organisms; Bacteria; Terrabacteria group; Actinobacteria; Actinomycetia; Corynebacteriales; Mycobacteriaceae; Mycobacterium; Mycobacterium tuberculosis complex; Mycobacterium tuberculosis;	NCBI: 1448445	4408737	99.94	0.00	Yes
NCBI Assembly	GCA_000662865.1	NCBI: cellular organisms; Bacteria; Terrabacteria group; Actinobacteria; Actinomycetia; Corynebacteriales; Mycobacteriaceae; Mycobacterium; Mycobacterium tuberculosis complex; Mycobacterium tuberculosis;	NCBI: 1448676	4426728	99.94	0.00	Yes
NCBI Assembly	GCA_000659785.1	NCBI: cellular organisms; Bacteria; Terrabacteria group; Actinobacteria; Actinomycetia; Corynebacteriales; Mycobacteriaceae; Mycobacterium; Mycobacterium tuberculosis complex; Mycobacterium tuberculosis;	NCBI: 1295773	4407771	99.94	0.00	Yes
NCBI Assembly	GCA_000656175.1	NCBI: cellular organisms; Bacteria; Terrabacteria group; Actinobacteria; Actinomycetia; Corynebacteriales; Mycobacteriaceae; Mycobacterium; Mycobacterium tuberculosis complex; Mycobacterium tuberculosis;	NCBI: 1427317	4412479	99.94	0.00	Yes
NCBI Assembly	GCA_000523915.1	NCBI: cellular organisms; Bacteria; Terrabacteria group; Actinobacteria; Actinomycetia; Corynebacteriales; Mycobacteriaceae; Mycobacteroides; Mycobacteroides abscessus;	NCBI: 1335415	4829246	100.00	0.67	Yes

NCBI Assembly	GCA_000649595.1	NCBI: cellular organisms; Bacteria; Terrabacteria group; Actinobacteria; Actinomycetia; Corynebacteriales; Mycobacteriaceae; Mycobacterium; Mycobacterium tuberculosis complex; Mycobacterium tuberculosis;	NCBI: 1438835	4385582	99.94	0.00	Yes
NCBI Assembly	GCA_000663205.1	NCBI: cellular organisms; Bacteria; Terrabacteria group; Actinobacteria; Actinomycetia; Corynebacteriales; Mycobacteriaceae; Mycobacterium; Mycobacterium tuberculosis complex; Mycobacterium tuberculosis;	NCBI: 1448693	4410420	99.94	0.00	Yes
NCBI Assembly	GCA_000443005.1	NCBI: cellular organisms; Bacteria; Terrabacteria group; Actinobacteria; Actinomycetia; Corynebacteriales; Mycobacteriaceae; Mycobacterium; Mycobacterium tuberculosis complex; Mycobacterium tuberculosis;	NCBI: 1262529	4408147	99.67	0.00	Yes
NCBI Assembly	GCA_000270685.2	NCBI: cellular organisms; Bacteria; Terrabacteria group; Actinobacteria; Actinomycetia; Corynebacteriales; Mycobacteriaceae; Mycobacteroides; Mycobacteroides abscessus;	NCBI: 1001725	4839534	100.00	0.00	Yes
NCBI Assembly	GCA_000661385.1	NCBI: cellular organisms; Bacteria; Terrabacteria group; Actinobacteria; Actinomycetia; Corynebacteriales; Mycobacteriaceae; Mycobacterium; Mycobacterium tuberculosis complex; Mycobacterium tuberculosis;	NCBI: 1324277	4436449	99.94	0.00	Yes
NCBI Assembly	GCA_000658835.1	NCBI: cellular organisms; Bacteria; Terrabacteria group; Actinobacteria; Actinomycetia; Corynebacteriales; Mycobacteriaceae; Mycobacterium; Mycobacterium tuberculosis complex; Mycobacterium tuberculosis;	NCBI: 1421993	4422640	99.94	0.00	Yes
NCBI Assembly	GCA_000662885.1	NCBI: cellular organisms; Bacteria; Terrabacteria group; Actinobacteria; Actinomycetia; Corynebacteriales; Mycobacteriaceae; Mycobacterium; Mycobacterium tuberculosis complex; Mycobacterium tuberculosis;	NCBI: 1448677	4427636	99.94	0.00	Yes
NCBI Assembly	GCA_000651495.1	NCBI: cellular organisms; Bacteria; Terrabacteria group; Actinobacteria; Actinomycetia; Corynebacteriales; Mycobacteriaceae; Mycobacterium; Mycobacterium tuberculosis complex; Mycobacterium tuberculosis;	NCBI: 1448399	4430496	99.94	0.00	Yes
NCBI Assembly	GCA_000661065.1	NCBI: cellular organisms; Bacteria; Terrabacteria group; Actinobacteria; Actinomycetia; Corynebacteriales; Mycobacteriaceae; Mycobacterium; Mycobacterium tuberculosis complex; Mycobacterium tuberculosis;	NCBI: 1324260	4427181	99.94	0.00	Yes
NCBI Assembly	GCA_000656195.1	NCBI: cellular organisms; Bacteria; Terrabacteria group; Actinobacteria; Actinomycetia; Corynebacteriales; Mycobacteriaceae; Mycobacterium; Mycobacterium tuberculosis complex; Mycobacterium tuberculosis;	NCBI: 1427318	4424546	99.94	0.00	Yes
NCBI Assembly	GCA_000659805.1	NCBI: cellular organisms; Bacteria; Terrabacteria group; Actinobacteria; Actinomycetia; Corynebacteriales; Mycobacteriaceae; Mycobacterium; Mycobacterium tuberculosis complex; Mycobacterium tuberculosis;	NCBI: 1295774	4415099	99.94	0.00	Yes
NCBI Assembly	GCA_000663225.1	NCBI: cellular organisms; Bacteria; Terrabacteria group; Actinobacteria; Actinomycetia; Corynebacteriales; Mycobacteriaceae; Mycobacterium; Mycobacterium tuberculosis complex; Mycobacterium tuberculosis;	NCBI: 1448694	4443406	99.94	0.00	Yes
NCBI Assembly	GCA_000572125.1	NCBI: cellular organisms; Bacteria; Terrabacteria group; Actinobacteria; Actinomycetia; Corynebacteriales; Mycobacteriaceae; Mycobacterium; Mycobacterium tuberculosis complex; Mycobacterium tuberculosis;	NCBI: 1010834	4407929	99.94	0.00	Yes
NCBI Assembly	GCA_000523935.1	NCBI: cellular organisms; Bacteria; Terrabacteria group; Actinobacteria; Actinomycetia; Corynebacteriales; Mycobacteriaceae; Mycobacteroides; Mycobacteroides abscessus;	NCBI: 1335417	5308379	100.00	0.17	Yes
NCBI Assembly	GCA_000661405.1	NCBI: cellular organisms; Bacteria; Terrabacteria group; Actinobacteria; Actinomycetia; Corynebacteriales; Mycobacteriaceae; Mycobacterium; Mycobacterium tuberculosis complex; Mycobacterium tuberculosis;	NCBI: 1324278	4397966	99.94	0.00	Yes
NCBI Assembly	GCA_000240345.2	NCBI: cellular organisms; Bacteria; Terrabacteria group; Actinobacteria; Actinomycetia; Corynebacteriales; Mycobacteriaceae; Mycobacterium; Mycobacterium avium complex (MAC); Mycobacterium avium; Mycobacterium avium subsp. paratuberculosis;	NCBI: 1051629	4735471	91.92	0.06	Yes
NCBI Assembly	GCA_000661715.1	NCBI: cellular organisms; Bacteria; Terrabacteria group; Actinobacteria; Actinomycetia; Corynebacteriales; Mycobacteriaceae; Mycobacterium; Mycobacterium tuberculosis complex; Mycobacterium tuberculosis;	NCBI: 1324294	4432887	99.94	0.00	Yes
NCBI Assembly	GCA_000651515.1	NCBI: cellular organisms; Bacteria; Terrabacteria group; Actinobacteria; Actinomycetia; Corynebacteriales; Mycobacteriaceae; Mycobacterium; Mycobacterium tuberculosis complex; Mycobacterium tuberculosis;	NCBI: 1448400	4409564	99.94	0.00	Yes

NCBI Assembly	GCA_000660465.1	NCBI: cellular organisms; Bacteria; Terrabacteria group; Actinobacteria; Actinomycetia; Corynebacteriales; Mycobacteriaceae; Mycobacterium; Mycobacterium tuberculosis complex; Mycobacterium tuberculosis;	NCBI: 1324229	4410300	99.94	0.00	Yes
NCBI Assembly	GCA_000658855.1	NCBI: cellular organisms; Bacteria; Terrabacteria group; Actinobacteria; Actinomycetia; Corynebacteriales; Mycobacteriaceae; Mycobacterium; Mycobacterium tuberculosis complex; Mycobacterium tuberculosis;	NCBI: 1423506	4384298	99.94	0.00	Yes
NCBI Assembly	GCA_000443145.1	NCBI: cellular organisms; Bacteria; Terrabacteria group; Actinobacteria; Actinomycetia; Corynebacteriales; Mycobacteriaceae; Mycobacteroides; Mycobacteroides abscessus;	NCBI: 1353275	5268718	99.83	0.00	Yes
NCBI Assembly	GCA_000656215.1	NCBI: cellular organisms; Bacteria; Terrabacteria group; Actinobacteria; Actinomycetia; Corynebacteriales; Mycobacteriaceae; Mycobacterium; Mycobacterium tuberculosis complex; Mycobacterium tuberculosis;	NCBI: 1427319	4423648	99.94	0.00	Yes
NCBI Assembly	GCA_000659205.1	NCBI: cellular organisms; Bacteria; Terrabacteria group; Actinobacteria; Actinomycetia; Corynebacteriales; Mycobacteriaceae; Mycobacterium; Mycobacterium tuberculosis complex; Mycobacterium tuberculosis;	NCBI: 1295720	4405189	99.94	0.00	Yes
NCBI Assembly	GCA_000663245.1	NCBI: cellular organisms; Bacteria; Terrabacteria group; Actinobacteria; Actinomycetia; Corynebacteriales; Mycobacteriaceae; Mycobacterium; Mycobacterium tuberculosis complex; Mycobacterium tuberculosis;	NCBI: 1448695	4433017	99.94	0.00	Yes
NCBI Assembly	GCA_000523955.1	NCBI: cellular organisms; Bacteria; Terrabacteria group; Actinobacteria; Actinomycetia; Corynebacteriales; Mycobacteriaceae; Mycobacteroides; Mycobacteroides abscessus;	NCBI: 1335416	4840778	99.67	1.22	Yes
NCBI Assembly	GCA_000572155.1	NCBI: cellular organisms; Bacteria; Terrabacteria group; Actinobacteria; Actinomycetia; Corynebacteriales; Mycobacteriaceae; Mycobacterium; Mycobacterium tuberculosis complex; Mycobacterium tuberculosis;	NCBI: 1010835	4401899	99.94	0.00	Yes
NCBI Assembly	GCA_000240365.2	NCBI: cellular organisms; Bacteria; Terrabacteria group; Actinobacteria; Actinomycetia; Corynebacteriales; Mycobacteriaceae; Mycobacterium; Mycobacterium avium complex (MAC); Mycobacterium avium; Mycobacterium avium subsp. paratuberculosis;	NCBI: 1112208	4697010	87.19	0.06	No
NCBI Assembly	GCA_000661425.1	NCBI: cellular organisms; Bacteria; Terrabacteria group; Actinobacteria; Actinomycetia; Corynebacteriales; Mycobacteriaceae; Mycobacterium; Mycobacterium tuberculosis complex; Mycobacterium tuberculosis;	NCBI: 1324279	4437254	99.94	0.00	Yes
NCBI Assembly	GCA_000443305.1	NCBI: cellular organisms; Bacteria; Terrabacteria group; Actinobacteria; Actinomycetia; Corynebacteriales; Mycobacteriaceae; Mycobacterium; Mycobacterium tuberculosis complex; Mycobacterium tuberculosis;	NCBI: 1344043	4397671	99.94	0.00	Yes
NCBI Assembly	GCA_000651535.1	NCBI: cellular organisms; Bacteria; Terrabacteria group; Actinobacteria; Actinomycetia; Corynebacteriales; Mycobacteriaceae; Mycobacterium; Mycobacterium tuberculosis complex; Mycobacterium tuberculosis;	NCBI: 1448401	4421561	99.94	0.00	Yes
NCBI Assembly	GCA_000660485.1	NCBI: cellular organisms; Bacteria; Terrabacteria group; Actinobacteria; Actinomycetia; Corynebacteriales; Mycobacteriaceae; Mycobacterium; Mycobacterium tuberculosis complex; Mycobacterium tuberculosis;	NCBI: 1324230	4417299	99.94	0.00	Yes
NCBI Assembly	GCA_000658875.1	NCBI: cellular organisms; Bacteria; Terrabacteria group; Actinobacteria; Actinomycetia; Corynebacteriales; Mycobacteriaceae; Mycobacterium; Mycobacterium tuberculosis complex; Mycobacterium tuberculosis;	NCBI: 1423512	4411321	99.94	0.00	Yes
NCBI Assembly	GCA_000661735.1	NCBI: cellular organisms; Bacteria; Terrabacteria group; Actinobacteria; Actinomycetia; Corynebacteriales; Mycobacteriaceae; Mycobacterium; Mycobacterium tuberculosis complex; Mycobacterium tuberculosis;	NCBI: 1324295	4418087	99.94	0.00	Yes
NCBI Assembly	GCA_000656235.1	NCBI: cellular organisms; Bacteria; Terrabacteria group; Actinobacteria; Actinomycetia; Corynebacteriales; Mycobacteriaceae; Mycobacterium; Mycobacterium tuberculosis complex; Mycobacterium tuberculosis;	NCBI: 1427320	4436518	99.94	0.00	Yes
NCBI Assembly	GCA_000659225.1	NCBI: cellular organisms; Bacteria; Terrabacteria group; Actinobacteria; Actinomycetia; Corynebacteriales; Mycobacteriaceae; Mycobacterium; Mycobacterium tuberculosis complex; Mycobacterium tuberculosis;	NCBI: 1295721	4407496	99.94	0.00	Yes
NCBI Assembly	GCA_000663265.1	NCBI: cellular organisms; Bacteria; Terrabacteria group; Actinobacteria; Actinomycetia; Corynebacteriales; Mycobacteriaceae; Mycobacterium; Mycobacterium tuberculosis complex; Mycobacterium tuberculosis;	NCBI: 1448696	4449801	99.94	0.00	Yes

NCBI Assembly	GCA_000523975.1	NCBI: cellular organisms; Bacteria; Terrabacteria group; Actinobacteria; Actinomycetia; Corynebacteriales; Mycobacteriaceae; Mycobacterium; Mycobacterium avium complex (MAC); unclassified Mycobacterium avium complex (MAC);	NCBI: 1335322	5571449	99.26	0.71	Yes
NCBI Assembly	GCA_000572175.1	NCBI: cellular organisms; Bacteria; Terrabacteria group; Actinobacteria; Actinomycetia; Corynebacteriales; Mycobacteriaceae; Mycobacterium; Mycobacterium tuberculosis complex; Mycobacterium tuberculosis;	NCBI: 1010836	4399405	99.94	0.00	Yes
NCBI Assembly	GCA_000661445.1	NCBI: cellular organisms; Bacteria; Terrabacteria group; Actinobacteria; Actinomycetia; Corynebacteriales; Mycobacteriaceae; Mycobacterium; Mycobacterium tuberculosis complex; Mycobacterium tuberculosis;	NCBI: 1324280	4399625	99.94	0.00	Yes
NCBI Assembly	GCA_000651555.1	NCBI: cellular organisms; Bacteria; Terrabacteria group; Actinobacteria; Actinomycetia; Corynebacteriales; Mycobacteriaceae; Mycobacterium; Mycobacterium tuberculosis complex; Mycobacterium tuberculosis;	NCBI: 1448402	4409041	99.94	0.00	Yes
NCBI Assembly	GCA_000660505.1	NCBI: cellular organisms; Bacteria; Terrabacteria group; Actinobacteria; Actinomycetia; Corynebacteriales; Mycobacteriaceae; Mycobacterium; Mycobacterium tuberculosis complex; Mycobacterium tuberculosis;	NCBI: 1324231	4408283	99.61	0.00	Yes
NCBI Assembly	GCA_000240385.2	NCBI: cellular organisms; Bacteria; Terrabacteria group; Actinobacteria; Actinomycetia; Corynebacteriales; Mycobacteriaceae; Mycobacterium; Mycobacterium avium complex (MAC); Mycobacterium avium; Mycobacterium avium subsp. paratuberculosis;	NCBI: 1078001	4755085	98.77	3.25	Yes
NCBI Assembly	GCA_000443325.1	NCBI: cellular organisms; Bacteria; Terrabacteria group; Actinobacteria; Actinomycetia; Corynebacteriales; Mycobacteriaceae; Mycobacterium; Mycobacterium tuberculosis complex; Mycobacterium tuberculosis;	NCBI: 1344044	4408813	99.94	0.00	Yes
NCBI Assembly	GCA_000658905.1	NCBI: cellular organisms; Bacteria; Terrabacteria group; Actinobacteria; Actinomycetia; Corynebacteriales; Mycobacteriaceae; Mycobacterium; Mycobacterium tuberculosis complex; Mycobacterium tuberculosis;	NCBI: 1423519	4396922	99.94	0.00	Yes
NCBI Assembly	GCA_000661755.1	NCBI: cellular organisms; Bacteria; Terrabacteria group; Actinobacteria; Actinomycetia; Corynebacteriales; Mycobacteriaceae; Mycobacterium; Mycobacterium tuberculosis complex; Mycobacterium tuberculosis;	NCBI: 1324296	4446393	99.94	0.00	Yes
NCBI Assembly	GCA_000656255.1	NCBI: cellular organisms; Bacteria; Terrabacteria group; Actinobacteria; Actinomycetia; Corynebacteriales; Mycobacteriaceae; Mycobacterium; Mycobacterium tuberculosis complex; Mycobacterium tuberculosis;	NCBI: 1427321	4408797	99.94	0.00	Yes
NCBI Assembly	GCA_000659245.1	NCBI: cellular organisms; Bacteria; Terrabacteria group; Actinobacteria; Actinomycetia; Corynebacteriales; Mycobacteriaceae; Mycobacterium; Mycobacterium tuberculosis complex; Mycobacterium tuberculosis;	NCBI: 1295723	4407215	99.94	0.00	Yes
NCBI Assembly	GCA_000663285.1	NCBI: cellular organisms; Bacteria; Terrabacteria group; Actinobacteria; Actinomycetia; Corynebacteriales; Mycobacteriaceae; Mycobacterium; Mycobacterium tuberculosis complex; Mycobacterium tuberculosis;	NCBI: 1448697	4423638	99.94	0.00	Yes
NCBI Assembly	GCA_000523995.1	NCBI: cellular organisms; Bacteria; Terrabacteria group; Actinobacteria; Actinomycetia; Corynebacteriales; Mycobacteriaceae; Mycobacterium; Mycobacterium avium complex (MAC); Mycobacterium avium;	NCBI: 1335420	5712139	99.61	2.48	Yes
NCBI Assembly	GCA_000611915.1	NCBI: cellular organisms; Bacteria; Terrabacteria group; Actinobacteria; Actinomycetia; Corynebacteriales; Mycobacteriaceae; Mycobacteroides; Mycobacteroides abscessus; Mycobacteroides abscessus subsp. abscessus;	NCBI: 1185650	4841074	100.00	0.17	Yes
NCBI Assembly	GCA_000443345.1	NCBI: cellular organisms; Bacteria; Terrabacteria group; Actinobacteria; Actinomycetia; Corynebacteriales; Mycobacteriaceae; Mycobacterium; Mycobacterium tuberculosis complex; Mycobacterium tuberculosis;	NCBI: 1344045	4404319	99.94	0.00	Yes
NCBI Assembly	GCA_000651575.1	NCBI: cellular organisms; Bacteria; Terrabacteria group; Actinobacteria; Actinomycetia; Corynebacteriales; Mycobacteriaceae; Mycobacterium; Mycobacterium tuberculosis complex; Mycobacterium tuberculosis;	NCBI: 1448403	4394481	99.94	0.00	Yes
NCBI Assembly	GCA_000661465.1	NCBI: cellular organisms; Bacteria; Terrabacteria group; Actinobacteria; Actinomycetia; Corynebacteriales; Mycobacteriaceae; Mycobacterium; Mycobacterium tuberculosis complex; Mycobacterium tuberculosis;	NCBI: 1324281	4411747	99.94	0.00	Yes
NCBI Assembly	GCA_000240405.2	NCBI: cellular organisms; Bacteria; Terrabacteria group; Actinobacteria; Actinomycetia; Corynebacteriales; Mycobacteriaceae; Mycobacterium; Mycobacterium avium complex (MAC); Mycobacterium avium; Mycobacterium avium subsp. paratuberculosis;	NCBI: 1078002	4745157	98.69	3.90	Yes

NCBI Assembly	GCA_000660525.1	NCBI: cellular organisms; Bacteria; Terrabacteria group; Actinobacteria; Actinomycetia; Corynebacteriales; Mycobacteriaceae; Mycobacterium; Mycobacterium tuberculosis complex; Mycobacterium tuberculosis;	NCBI: 1324232	4398557	99.94	1.00	Yes
NCBI Assembly	GCA_000658925.1	NCBI: cellular organisms; Bacteria; Terrabacteria group; Actinobacteria; Actinomycetia; Corynebacteriales; Mycobacteriaceae; Mycobacterium; Mycobacterium tuberculosis complex; Mycobacterium tuberculosis;	NCBI: 1423521	4404612	99.94	0.00	Yes
NCBI Assembly	GCA_000656275.1	NCBI: cellular organisms; Bacteria; Terrabacteria group; Actinobacteria; Actinomycetia; Corynebacteriales; Mycobacteriaceae; Mycobacterium; Mycobacterium tuberculosis complex; Mycobacterium tuberculosis;	NCBI: 1427322	4391648	99.94	0.00	Yes
NCBI Assembly	GCA_000659265.1	NCBI: cellular organisms; Bacteria; Terrabacteria group; Actinobacteria; Actinomycetia; Corynebacteriales; Mycobacteriaceae; Mycobacterium; Mycobacterium tuberculosis complex; Mycobacterium tuberculosis;	NCBI: 1295724	4410820	99.94	0.00	Yes
NCBI Assembly	GCA_000661775.1	NCBI: cellular organisms; Bacteria; Terrabacteria group; Actinobacteria; Actinomycetia; Corynebacteriales; Mycobacteriaceae; Mycobacterium; Mycobacterium tuberculosis complex; Mycobacterium tuberculosis;	NCBI: 1324297	4444756	99.94	0.00	Yes
NCBI Assembly	GCA_000663305.1	NCBI: cellular organisms; Bacteria; Terrabacteria group; Actinobacteria; Actinomycetia; Corynebacteriales; Mycobacteriaceae; Mycobacterium; Mycobacterium tuberculosis complex; Mycobacterium tuberculosis;	NCBI: 1448698	4420439	99.94	0.00	Yes
NCBI Assembly	GCA_000524015.1	NCBI: cellular organisms; Bacteria; Terrabacteria group; Actinobacteria; Actinomycetia; Corynebacteriales; Mycobacteriaceae; Mycobacterium; Mycobacterium avium complex (MAC); Mycobacterium intracellulare;	NCBI: 1335421	6331706	99.94	1.82	Yes
NCBI Assembly	GCA_000443365.1	NCBI: cellular organisms; Bacteria; Terrabacteria group; Actinobacteria; Actinomycetia; Corynebacteriales; Mycobacteriaceae; Mycobacterium; Mycobacterium tuberculosis complex; Mycobacterium tuberculosis;	NCBI: 1344047	4393994	99.94	0.00	Yes
NCBI Assembly	GCA_000658945.1	NCBI: cellular organisms; Bacteria; Terrabacteria group; Actinobacteria; Actinomycetia; Corynebacteriales; Mycobacteriaceae; Mycobacterium; Mycobacterium tuberculosis complex; Mycobacterium tuberculosis;	NCBI: 1423525	4418185	99.94	0.00	Yes
NCBI Assembly	GCA_000240425.2	NCBI: cellular organisms; Bacteria; Terrabacteria group; Actinobacteria; Actinomycetia; Corynebacteriales; Mycobacteriaceae; Mycobacterium; Mycobacterium avium complex (MAC); Mycobacterium avium; Mycobacterium avium subsp. paratuberculosis;	NCBI: 1078003	4738595	99.50	1.36	Yes
NCBI Assembly	GCA_000651595.1	NCBI: cellular organisms; Bacteria; Terrabacteria group; Actinobacteria; Actinomycetia; Corynebacteriales; Mycobacteriaceae; Mycobacterium; Mycobacterium tuberculosis complex; Mycobacterium tuberculosis;	NCBI: 1448404	4425068	99.94	0.00	Yes
NCBI Assembly	GCA_000661485.1	NCBI: cellular organisms; Bacteria; Terrabacteria group; Actinobacteria; Actinomycetia; Corynebacteriales; Mycobacteriaceae; Mycobacterium; Mycobacterium tuberculosis complex; Mycobacterium tuberculosis;	NCBI: 1324282	4403481	99.94	0.00	Yes
NCBI Assembly	GCA_000660545.1	NCBI: cellular organisms; Bacteria; Terrabacteria group; Actinobacteria; Actinomycetia; Corynebacteriales; Mycobacteriaceae; Mycobacterium; Mycobacterium tuberculosis complex; Mycobacterium tuberculosis;	NCBI: 1324233	4449227	99.94	0.00	Yes
NCBI Assembly	GCA_000612725.1	NCBI: cellular organisms; Bacteria; Terrabacteria group; Actinobacteria; Actinomycetia; Corynebacteriales; Mycobacteriaceae; Mycolicibacterium; Mycolicibacterium austroafricanum;	NCBI: 39687	6770972	99.62	0.12	Yes
NCBI Assembly	GCA_000656295.1	NCBI: cellular organisms; Bacteria; Terrabacteria group; Actinobacteria; Actinomycetia; Corynebacteriales; Mycobacteriaceae; Mycobacterium; Mycobacterium tuberculosis complex; Mycobacterium tuberculosis;	NCBI: 1427323	4422686	99.94	0.00	Yes
NCBI Assembly	GCA_000661795.1	NCBI: cellular organisms; Bacteria; Terrabacteria group; Actinobacteria; Actinomycetia; Corynebacteriales; Mycobacteriaceae; Mycobacterium; Mycobacterium tuberculosis complex; Mycobacterium tuberculosis;	NCBI: 1324298	4398833	99.94	0.00	Yes
NCBI Assembly	GCA_000659285.1	NCBI: cellular organisms; Bacteria; Terrabacteria group; Actinobacteria; Actinomycetia; Corynebacteriales; Mycobacteriaceae; Mycobacterium; Mycobacterium tuberculosis complex; Mycobacterium tuberculosis;	NCBI: 1295725	4399918	99.94	0.00	Yes
NCBI Assembly	GCA_000524035.1	NCBI: cellular organisms; Bacteria; Terrabacteria group; Actinobacteria; Actinomycetia; Corynebacteriales; Mycobacteriaceae; Mycobacterium; Mycobacterium ulcerans;	NCBI: 1299332	6247430	90.75	2.16	Yes

NCBI Assembly	GCA_000663325.1	NCBI: cellular organisms; Bacteria; Terrabacteria group; Actinobacteria; Actinomycetia; Corynebacteriales; Mycobacteriaceae; Mycobacterium; Mycobacterium tuberculosis complex; Mycobacterium tuberculosis;	NCBI: 1448699	4424029	99.94	0.00	Yes
NCBI Assembly	GCA_000443405.1	NCBI: cellular organisms; Bacteria; Terrabacteria group; Actinobacteria; Actinomycetia; Corynebacteriales; Mycobacteriaceae; Mycobacterium; Mycobacterium tuberculosis complex; Mycobacterium tuberculosis;	NCBI: 1344048	4408133	99.94	0.00	Yes
NCBI Assembly	GCA_000659005.1	NCBI: cellular organisms; Bacteria; Terrabacteria group; Actinobacteria; Actinomycetia; Corynebacteriales; Mycobacteriaceae; Mycobacterium; Mycobacterium tuberculosis complex; Mycobacterium tuberculosis;	NCBI: 1423515	4432884	99.94	0.00	Yes
NCBI Assembly	GCA_000651615.1	NCBI: cellular organisms; Bacteria; Terrabacteria group; Actinobacteria; Actinomycetia; Corynebacteriales; Mycobacteriaceae; Mycobacterium; Mycobacterium tuberculosis complex; Mycobacterium tuberculosis;	NCBI: 1448405	4424218	99.94	0.00	Yes
NCBI Assembly	GCA_000240445.2	NCBI: cellular organisms; Bacteria; Terrabacteria group; Actinobacteria; Actinomycetia; Corynebacteriales; Mycobacteriaceae; Mycobacterium; Mycobacterium avium complex (MAC); Mycobacterium avium; Mycobacterium avium subsp. paratuberculosis;	NCBI: 1078004	4725169	98.54	1.74	Yes
NCBI Assembly	GCA_000660565.1	NCBI: cellular organisms; Bacteria; Terrabacteria group; Actinobacteria; Actinomycetia; Corynebacteriales; Mycobacteriaceae; Mycobacterium; Mycobacterium tuberculosis complex; Mycobacterium tuberculosis;	NCBI: 1324234	4410931	99.94	0.00	Yes
NCBI Assembly	GCA_000661505.1	NCBI: cellular organisms; Bacteria; Terrabacteria group; Actinobacteria; Actinomycetia; Corynebacteriales; Mycobacteriaceae; Mycobacterium; Mycobacterium tuberculosis complex; Mycobacterium tuberculosis;	NCBI: 1324283	4402940	99.94	0.17	Yes
NCBI Assembly	GCA_000656315.1	NCBI: cellular organisms; Bacteria; Terrabacteria group; Actinobacteria; Actinomycetia; Corynebacteriales; Mycobacteriaceae; Mycobacterium; Mycobacterium tuberculosis complex; Mycobacterium tuberculosis;	NCBI: 1427324	4429027	99.94	0.00	Yes
NCBI Assembly	GCA_000524055.1	NCBI: cellular organisms; Bacteria; Terrabacteria group; Actinobacteria; Actinomycetia; Corynebacteriales; Mycobacteriaceae; Mycobacterium; Mycobacterium kansasii;	NCBI: 1299327	6456912	96.93	0.40	Yes
NCBI Assembly	GCA_000659305.1	NCBI: cellular organisms; Bacteria; Terrabacteria group; Actinobacteria; Actinomycetia; Corynebacteriales; Mycobacteriaceae; Mycobacterium; Mycobacterium tuberculosis complex; Mycobacterium tuberculosis;	NCBI: 1295726	4408638	99.94	0.00	Yes
NCBI Assembly	GCA_000661815.1	NCBI: cellular organisms; Bacteria; Terrabacteria group; Actinobacteria; Actinomycetia; Corynebacteriales; Mycobacteriaceae; Mycobacterium; Mycobacterium tuberculosis complex; Mycobacterium tuberculosis;	NCBI: 1324299	4414989	99.94	0.00	Yes
NCBI Assembly	GCA_000663345.1	NCBI: cellular organisms; Bacteria; Terrabacteria group; Actinobacteria; Actinomycetia; Corynebacteriales; Mycobacteriaceae; Mycobacterium; Mycobacterium tuberculosis complex; Mycobacterium tuberculosis;	NCBI: 1448700	4447115	99.94	0.00	Yes
NCBI Assembly	GCA_000443425.1	NCBI: cellular organisms; Bacteria; Terrabacteria group; Actinobacteria; Actinomycetia; Corynebacteriales; Mycobacteriaceae; Mycobacterium; Mycobacterium tuberculosis complex; Mycobacterium tuberculosis;	NCBI: 1344046	4398802	99.94	0.00	Yes
NCBI Assembly	GCA_000651635.1	NCBI: cellular organisms; Bacteria; Terrabacteria group; Actinobacteria; Actinomycetia; Corynebacteriales; Mycobacteriaceae; Mycobacterium; Mycobacterium tuberculosis complex; Mycobacterium tuberculosis;	NCBI: 1448406	4400581	99.94	0.00	Yes
NCBI Assembly	GCA_000659025.1	NCBI: cellular organisms; Bacteria; Terrabacteria group; Actinobacteria; Actinomycetia; Corynebacteriales; Mycobacteriaceae; Mycobacterium; Mycobacterium tuberculosis complex; Mycobacterium tuberculosis;	NCBI: 1267355	4419586	99.94	0.00	Yes
NCBI Assembly	GCA_000240465.2	NCBI: cellular organisms; Bacteria; Terrabacteria group; Actinobacteria; Actinomycetia; Corynebacteriales; Mycobacteriaceae; Mycobacterium; Mycobacterium avium complex (MAC); Mycobacterium avium; Mycobacterium avium subsp. paratuberculosis;	NCBI: 1078005	4736143	98.86	1.60	Yes
NCBI Assembly	GCA_000660585.1	NCBI: cellular organisms; Bacteria; Terrabacteria group; Actinobacteria; Actinomycetia; Corynebacteriales; Mycobacteriaceae; Mycobacterium; Mycobacterium tuberculosis complex; Mycobacterium tuberculosis;	NCBI: 1324235	4418438	99.94	0.17	Yes
NCBI Assembly	GCA_000661525.1	NCBI: cellular organisms; Bacteria; Terrabacteria group; Actinobacteria; Actinomycetia; Corynebacteriales; Mycobacteriaceae; Mycobacterium; Mycobacterium tuberculosis complex; Mycobacterium tuberculosis;	NCBI: 1324284	4401950	99.94	0.00	Yes

NCBI Assembly	GCA_000656335.1	NCBI: cellular organisms; Bacteria; Terrabacteria group; Actinobacteria; Actinomycetia; Corynebacteriales; Mycobacteriaceae; Mycobacterium; Mycobacterium tuberculosis complex; Mycobacterium tuberculosis;	NCBI: 1427325	4422504	99.94	0.00	Yes
NCBI Assembly	GCA_000659325.1	NCBI: cellular organisms; Bacteria; Terrabacteria group; Actinobacteria; Actinomycetia; Corynebacteriales; Mycobacteriaceae; Mycobacterium; Mycobacterium tuberculosis complex; Mycobacterium tuberculosis;	NCBI: 1295727	4407120	99.94	0.00	Yes
NCBI Assembly	GCA_000661835.1	NCBI: cellular organisms; Bacteria; Terrabacteria group; Actinobacteria; Actinomycetia; Corynebacteriales; Mycobacteriaceae; Mycobacterium; Mycobacterium tuberculosis complex; Mycobacterium tuberculosis;	NCBI: 1324300	4451331	99.94	0.00	Yes
NCBI Assembly	GCA_000524075.1	NCBI: cellular organisms; Bacteria; Terrabacteria group; Actinobacteria; Actinomycetia; Corynebacteriales; Mycobacteriaceae; Mycobacteroides; Mycobacteroides abscessus; Mycobacteroides abscessus subsp. bolletii;	NCBI: 1299325	5314248	100.00	0.00	Yes
NCBI Assembly	GCA_000663365.1	NCBI: cellular organisms; Bacteria; Terrabacteria group; Actinobacteria; Actinomycetia; Corynebacteriales; Mycobacteriaceae; Mycobacterium; Mycobacterium tuberculosis complex; Mycobacterium tuberculosis;	NCBI: 1448701	4418232	99.94	0.33	Yes
NCBI Assembly	GCA_000443445.1	NCBI: cellular organisms; Bacteria; Terrabacteria group; Actinobacteria; Actinomycetia; Corynebacteriales; Mycobacteriaceae; Mycobacterium; Mycobacterium tuberculosis complex; Mycobacterium tuberculosis;	NCBI: 1346765	4393218	99.94	0.00	Yes
NCBI Assembly	GCA_000240485.2	NCBI: cellular organisms; Bacteria; Terrabacteria group; Actinobacteria; Actinomycetia; Corynebacteriales; Mycobacteriaceae; Mycobacterium; Mycobacterium avium complex (MAC); Mycobacterium avium; Mycobacterium avium subsp. avium;	NCBI: 1078011	4959832	98.49	2.51	Yes
NCBI Assembly	GCA_000651655.1	NCBI: cellular organisms; Bacteria; Terrabacteria group; Actinobacteria; Actinomycetia; Corynebacteriales; Mycobacteriaceae; Mycobacterium; Mycobacterium tuberculosis complex; Mycobacterium tuberculosis;	NCBI: 1448407	4414779	99.94	0.00	Yes
NCBI Assembly	GCA_000659045.1	NCBI: cellular organisms; Bacteria; Terrabacteria group; Actinobacteria; Actinomycetia; Corynebacteriales; Mycobacteriaceae; Mycobacterium; Mycobacterium tuberculosis complex; Mycobacterium tuberculosis;	NCBI: 1267356	4397765	99.94	0.00	Yes
NCBI Assembly	GCA_000660605.1	NCBI: cellular organisms; Bacteria; Terrabacteria group; Actinobacteria; Actinomycetia; Corynebacteriales; Mycobacteriaceae; Mycobacterium; Mycobacterium tuberculosis complex; Mycobacterium tuberculosis;	NCBI: 1324236	4371519	99.28	0.00	Yes
NCBI Assembly	GCA_000612885.1	NCBI: cellular organisms; Bacteria; Terrabacteria group; Actinobacteria; Actinomycetia; Corynebacteriales; Mycobacteriaceae; Mycolicibacterium; Mycolicibacterium vulneris;	NCBI: 547163	6981439	99.81	1.32	Yes
NCBI Assembly	GCA_000661555.1	NCBI: cellular organisms; Bacteria; Terrabacteria group; Actinobacteria; Actinomycetia; Corynebacteriales; Mycobacteriaceae; Mycobacterium; Mycobacterium tuberculosis complex; Mycobacterium tuberculosis;	NCBI: 1324286	4412287	99.94	0.00	Yes
NCBI Assembly	GCA_000656355.1	NCBI: cellular organisms; Bacteria; Terrabacteria group; Actinobacteria; Actinomycetia; Corynebacteriales; Mycobacteriaceae; Mycobacterium; Mycobacterium tuberculosis complex; Mycobacterium tuberculosis;	NCBI: 1427327	4402016	99.94	0.00	Yes
NCBI Assembly	GCA_000661855.1	NCBI: cellular organisms; Bacteria; Terrabacteria group; Actinobacteria; Actinomycetia; Corynebacteriales; Mycobacteriaceae; Mycobacterium; Mycobacterium tuberculosis complex; Mycobacterium tuberculosis;	NCBI: 1324301	4415524	99.94	0.00	Yes
NCBI Assembly	GCA_000659345.1	NCBI: cellular organisms; Bacteria; Terrabacteria group; Actinobacteria; Actinomycetia; Corynebacteriales; Mycobacteriaceae; Mycobacterium; Mycobacterium tuberculosis complex; Mycobacterium tuberculosis;	NCBI: 1295739	4406107	99.94	0.00	Yes
NCBI Assembly	GCA_000443465.1	NCBI: cellular organisms; Bacteria; Terrabacteria group; Actinobacteria; Actinomycetia; Corynebacteriales; Mycobacteriaceae; Mycobacterium; Mycobacterium tuberculosis complex; Mycobacterium tuberculosis;	NCBI: 1346767	4404835	99.94	0.00	Yes
NCBI Assembly	GCA_000524095.1	NCBI: cellular organisms; Bacteria; Terrabacteria group; Actinobacteria; Actinomycetia; Corynebacteriales; Mycobacteriaceae; Mycobacterium; Mycobacterium kansasii;	NCBI: 1299328	6592549	95.43	0.76	Yes
NCBI Assembly	GCA_000663385.1	NCBI: cellular organisms; Bacteria; Terrabacteria group; Actinobacteria; Actinomycetia; Corynebacteriales; Mycobacteriaceae; Mycobacterium; Mycobacterium tuberculosis complex; Mycobacterium tuberculosis;	NCBI: 1448702	4431560	99.94	0.00	Yes

NCBI Assembly	GCA_000240505.2	NCBI: cellular organisms; Bacteria; Terrabacteria group; Actinobacteria; Actinomycetia; Corynebacteriales; Mycobacteriaceae; Mycobacterium; Mycobacterium avium complex (MAC); Mycobacterium avium; Mycobacterium avium subsp. avium;	NCBI: 1078013	4583671	97.16	2.29	Yes
NCBI Assembly	GCA_000651675.1	NCBI: cellular organisms; Bacteria; Terrabacteria group; Actinobacteria; Actinomycetia; Corynebacteriales; Mycobacteriaceae; Mycobacterium; Mycobacterium tuberculosis complex; Mycobacterium tuberculosis;	NCBI: 1448409	4427315	99.94	0.00	Yes
NCBI Assembly	GCA_000659065.1	NCBI: cellular organisms; Bacteria; Terrabacteria group; Actinobacteria; Actinomycetia; Corynebacteriales; Mycobacteriaceae; Mycobacterium; Mycobacterium tuberculosis complex; Mycobacterium tuberculosis;	NCBI: 1267358	4413173	99.94	0.00	Yes
NCBI Assembly	GCA_000660625.1	NCBI: cellular organisms; Bacteria; Terrabacteria group; Actinobacteria; Actinomycetia; Corynebacteriales; Mycobacteriaceae; Mycobacterium; Mycobacterium tuberculosis complex; Mycobacterium tuberculosis;	NCBI: 1324237	4409044	99.94	0.00	Yes
NCBI Assembly	GCA_000661575.1	NCBI: cellular organisms; Bacteria; Terrabacteria group; Actinobacteria; Actinomycetia; Corynebacteriales; Mycobacteriaceae; Mycobacterium; Mycobacterium tuberculosis complex; Mycobacterium tuberculosis;	NCBI: 1324287	4421863	99.94	0.00	Yes
NCBI Assembly	GCA_000656375.1	NCBI: cellular organisms; Bacteria; Terrabacteria group; Actinobacteria; Actinomycetia; Corynebacteriales; Mycobacteriaceae; Mycobacterium; Mycobacterium tuberculosis complex; Mycobacterium tuberculosis;	NCBI: 1427329	4423698	99.94	0.00	Yes
NCBI Assembly	GCA_000662355.1	NCBI: cellular organisms; Bacteria; Terrabacteria group; Actinobacteria; Actinomycetia; Corynebacteriales; Mycobacteriaceae; Mycobacterium; Mycobacterium tuberculosis complex; Mycobacterium tuberculosis;	NCBI: 1324285	4447995	99.94	0.00	Yes
NCBI Assembly	GCA_000443485.1	NCBI: cellular organisms; Bacteria; Terrabacteria group; Actinobacteria; Actinomycetia; Corynebacteriales; Mycobacteriaceae; Mycobacterium; Mycobacterium tuberculosis complex; Mycobacterium tuberculosis;	NCBI: 1346766	4404586	99.94	0.00	Yes
NCBI Assembly	GCA_000659365.1	NCBI: cellular organisms; Bacteria; Terrabacteria group; Actinobacteria; Actinomycetia; Corynebacteriales; Mycobacteriaceae; Mycobacterium; Mycobacterium tuberculosis complex; Mycobacterium tuberculosis;	NCBI: 1295740	4399238	99.94	0.00	Yes
NCBI Assembly	GCA_000524155.1	NCBI: cellular organisms; Bacteria; Terrabacteria group; Actinobacteria; Actinomycetia; Corynebacteriales; Mycobacteriaceae; Mycobacterium; Mycobacterium tuberculosis complex; Mycobacterium tuberculosis;	NCBI: 1441927	4391020	99.94	0.00	Yes
NCBI Assembly	GCA_000663405.1	NCBI: cellular organisms; Bacteria; Terrabacteria group; Actinobacteria; Actinomycetia; Corynebacteriales; Mycobacteriaceae; Mycobacterium; Mycobacterium tuberculosis complex; Mycobacterium tuberculosis;	NCBI: 1448703	4418739	99.94	0.00	Yes
NCBI Assembly	GCA_000651695.1	NCBI: cellular organisms; Bacteria; Terrabacteria group; Actinobacteria; Actinomycetia; Corynebacteriales; Mycobacteriaceae; Mycobacterium; Mycobacterium tuberculosis complex; Mycobacterium tuberculosis;	NCBI: 1448410	4416588	99.94	0.00	Yes
NCBI Assembly	GCA_000659085.1	NCBI: cellular organisms; Bacteria; Terrabacteria group; Actinobacteria; Actinomycetia; Corynebacteriales; Mycobacteriaceae; Mycobacterium; Mycobacterium tuberculosis complex; Mycobacterium tuberculosis;	NCBI: 1267359	4411683	99.28	0.00	Yes
NCBI Assembly	GCA_000240525.2	NCBI: cellular organisms; Bacteria; Terrabacteria group; Actinobacteria; Actinomycetia; Corynebacteriales; Mycobacteriaceae; Mycobacterium; Mycobacterium avium complex (MAC); Mycobacterium avium; Mycobacterium avium subsp. paratuberculosis;	NCBI: 1074454	4736327	98.44	4.15	Yes
NCBI Assembly	GCA_000660645.1	NCBI: cellular organisms; Bacteria; Terrabacteria group; Actinobacteria; Actinomycetia; Corynebacteriales; Mycobacteriaceae; Mycobacterium; Mycobacterium tuberculosis complex; Mycobacterium tuberculosis;	NCBI: 1324238	4423882	99.94	0.00	Yes
NCBI Assembly	GCA_000620625.1	NCBI: cellular organisms; Bacteria; Terrabacteria group; Actinobacteria; Actinomycetia; Corynebacteriales; Mycobacteriaceae; Mycobacterium; unclassified Mycobacterium;	NCBI: 1380386	7485871	100.00	0.81	Yes
NCBI Assembly	GCA_000656395.1	NCBI: cellular organisms; Bacteria; Terrabacteria group; Actinobacteria; Actinomycetia; Corynebacteriales; Mycobacteriaceae; Mycobacterium; Mycobacterium tuberculosis complex; Mycobacterium tuberculosis;	NCBI: 1427330	4426197	99.94	0.00	Yes
NCBI Assembly	GCA_000661595.1	NCBI: cellular organisms; Bacteria; Terrabacteria group; Actinobacteria; Actinomycetia; Corynebacteriales; Mycobacteriaceae; Mycobacterium; Mycobacterium tuberculosis complex; Mycobacterium tuberculosis;	NCBI: 1324288	4418437	99.94	0.00	Yes

NCBI Assembly	GCA_000662465.1	NCBI: cellular organisms; Bacteria; Terrabacteria group; Actinobacteria; Actinomycetia; Corynebacteriales; Mycobacteriaceae; Mycobacterium; Mycobacterium tuberculosis complex; Mycobacterium tuberculosis;	NCBI: 1448656	4420532	99.94	0.00	Yes
NCBI Assembly	GCA_000443505.1	NCBI: cellular organisms; Bacteria; Terrabacteria group; Actinobacteria; Actinomycetia; Corynebacteriales; Mycobacteriaceae; Mycobacterium; Mycobacterium tuberculosis complex; Mycobacterium tuberculosis;	NCBI: 1346768	4402591	99.94	0.00	Yes
NCBI Assembly	GCA_000659385.1	NCBI: cellular organisms; Bacteria; Terrabacteria group; Actinobacteria; Actinomycetia; Corynebacteriales; Mycobacteriaceae; Mycobacterium; Mycobacterium tuberculosis complex; Mycobacterium tuberculosis;	NCBI: 1295742	4400749	99.94	0.00	Yes
NCBI Assembly	GCA_000524175.1	NCBI: cellular organisms; Bacteria; Terrabacteria group; Actinobacteria; Actinomycetia; Corynebacteriales; Mycobacteriaceae; Mycobacterium; Mycobacterium tuberculosis complex; Mycobacterium tuberculosis;	NCBI: 1441925	4391145	99.94	0.00	Yes
NCBI Assembly	GCA_000663425.1	NCBI: cellular organisms; Bacteria; Terrabacteria group; Actinobacteria; Actinomycetia; Corynebacteriales; Mycobacteriaceae; Mycobacterium; Mycobacterium tuberculosis complex; Mycobacterium tuberculosis;	NCBI: 1448704	4430930	99.94	0.67	Yes
NCBI Assembly	GCA_000651715.1	NCBI: cellular organisms; Bacteria; Terrabacteria group; Actinobacteria; Actinomycetia; Corynebacteriales; Mycobacteriaceae; Mycobacterium; Mycobacterium tuberculosis complex; Mycobacterium tuberculosis;	NCBI: 1448411	4407994	99.94	0.00	Yes
NCBI Assembly	GCA_000659105.1	NCBI: cellular organisms; Bacteria; Terrabacteria group; Actinobacteria; Actinomycetia; Corynebacteriales; Mycobacteriaceae; Mycobacterium; Mycobacterium tuberculosis complex; Mycobacterium tuberculosis;	NCBI: 1267360	4408216	99.94	0.00	Yes
NCBI Assembly	GCA_000243415.3	NCBI: cellular organisms; Bacteria; Terrabacteria group; Actinobacteria; Actinomycetia; Corynebacteriales; Mycobacteriaceae; Mycolicibacterium; Mycolicibacterium tusciae;	NCBI: 710687	7306413	100.00	0.30	Yes
NCBI Assembly	GCA_000660665.1	NCBI: cellular organisms; Bacteria; Terrabacteria group; Actinobacteria; Actinomycetia; Corynebacteriales; Mycobacteriaceae; Mycobacterium; Mycobacterium tuberculosis complex; Mycobacterium tuberculosis;	NCBI: 1324239	4410609	99.94	0.00	Yes
NCBI Assembly	GCA_000656415.1	NCBI: cellular organisms; Bacteria; Terrabacteria group; Actinobacteria; Actinomycetia; Corynebacteriales; Mycobacteriaceae; Mycobacterium; Mycobacterium tuberculosis complex; Mycobacterium tuberculosis;	NCBI: 1438878	4398874	99.75	0.17	Yes
NCBI Assembly	GCA_000633085.1	NCBI: cellular organisms; Bacteria; Terrabacteria group; Actinobacteria; Actinomycetia; Corynebacteriales; Mycobacteriaceae; Mycobacterium; Mycobacterium tuberculosis complex; Mycobacterium tuberculosis;	NCBI: 1773	4230125	99.61	0.00	Yes
NCBI Assembly	GCA_000661615.1	NCBI: cellular organisms; Bacteria; Terrabacteria group; Actinobacteria; Actinomycetia; Corynebacteriales; Mycobacteriaceae; Mycobacterium; Mycobacterium tuberculosis complex; Mycobacterium tuberculosis;	NCBI: 1324289	4413098	99.94	0.00	Yes
NCBI Assembly	GCA_000443525.1	NCBI: cellular organisms; Bacteria; Terrabacteria group; Actinobacteria; Actinomycetia; Corynebacteriales; Mycobacteriaceae; Mycobacterium; Mycobacterium tuberculosis complex; Mycobacterium tuberculosis;	NCBI: 1346770	4405148	96.51	0.00	Yes
NCBI Assembly	GCA_000662485.1	NCBI: cellular organisms; Bacteria; Terrabacteria group; Actinobacteria; Actinomycetia; Corynebacteriales; Mycobacteriaceae; Mycobacterium; Mycobacterium tuberculosis complex; Mycobacterium tuberculosis;	NCBI: 1448657	4428970	99.94	0.00	Yes
NCBI Assembly	GCA_000526115.1	NCBI: cellular organisms; Bacteria; Terrabacteria group; Actinobacteria; Actinomycetia; Corynebacteriales; Mycobacteriaceae; Mycobacterium; Mycobacterium tuberculosis complex; Mycobacterium tuberculosis;	NCBI: 1441926	4379293	99.94	0.00	Yes
NCBI Assembly	GCA_000659405.1	NCBI: cellular organisms; Bacteria; Terrabacteria group; Actinobacteria; Actinomycetia; Corynebacteriales; Mycobacteriaceae; Mycobacterium; Mycobacterium tuberculosis complex; Mycobacterium tuberculosis;	NCBI: 1295744	4403314	99.94	0.00	Yes
NCBI Assembly	GCA_000663445.1	NCBI: cellular organisms; Bacteria; Terrabacteria group; Actinobacteria; Actinomycetia; Corynebacteriales; Mycobacteriaceae; Mycobacterium; Mycobacterium tuberculosis complex; Mycobacterium tuberculosis;	NCBI: 1448705	4438984	99.94	0.00	Yes
NCBI Assembly	GCA_000651735.1	NCBI: cellular organisms; Bacteria; Terrabacteria group; Actinobacteria; Actinomycetia; Corynebacteriales; Mycobacteriaceae; Mycobacterium; Mycobacterium tuberculosis complex; Mycobacterium tuberculosis;	NCBI: 1448412	4399338	99.94	0.00	Yes

NCBI Assembly	GCA_000256525.1	NCBI: cellular organisms; Bacteria; Terrabacteria group; Actinobacteria; Actinomycetia; Corynebacteriales; Mycobacteriaceae; Mycobacterium; Mycobacterium tuberculosis complex; Mycobacterium tuberculosis;	NCBI: 707237	4321569	99.44	0.00	Yes
NCBI Assembly	GCA_000659125.1	NCBI: cellular organisms; Bacteria; Terrabacteria group; Actinobacteria; Actinomycetia; Corynebacteriales; Mycobacteriaceae; Mycobacterium; Mycobacterium tuberculosis complex; Mycobacterium tuberculosis;	NCBI: 1267361	4412936	99.94	0.00	Yes
NCBI Assembly	GCA_000660685.1	NCBI: cellular organisms; Bacteria; Terrabacteria group; Actinobacteria; Actinomycetia; Corynebacteriales; Mycobacteriaceae; Mycobacterium; Mycobacterium tuberculosis complex; Mycobacterium tuberculosis;	NCBI: 1324240	4405292	99.94	0.00	Yes
NCBI Assembly	GCA_000656435.1	NCBI: cellular organisms; Bacteria; Terrabacteria group; Actinobacteria; Actinomycetia; Corynebacteriales; Mycobacteriaceae; Mycobacterium; Mycobacterium tuberculosis complex; Mycobacterium tuberculosis;	NCBI: 1438881	4400930	99.94	0.00	Yes
NCBI Assembly	GCA_000648395.1	NCBI: cellular organisms; Bacteria; Terrabacteria group; Actinobacteria; Actinomycetia; Corynebacteriales; Mycobacteriaceae; Mycobacterium; Mycobacterium tuberculosis complex; Mycobacterium tuberculosis;	NCBI: 1458472	4342105	99.47	1.21	Yes
NCBI Assembly	GCA_000661635.1	NCBI: cellular organisms; Bacteria; Terrabacteria group; Actinobacteria; Actinomycetia; Corynebacteriales; Mycobacteriaceae; Mycobacterium; Mycobacterium tuberculosis complex; Mycobacterium tuberculosis;	NCBI: 1324290	4410924	99.94	0.00	Yes
NCBI Assembly	GCA_000443545.1	NCBI: cellular organisms; Bacteria; Terrabacteria group; Actinobacteria; Actinomycetia; Corynebacteriales; Mycobacteriaceae; Mycobacterium; Mycobacterium tuberculosis complex; Mycobacterium tuberculosis;	NCBI: 1346771	4403568	99.94	0.00	Yes
NCBI Assembly	GCA_000662505.1	NCBI: cellular organisms; Bacteria; Terrabacteria group; Actinobacteria; Actinomycetia; Corynebacteriales; Mycobacteriaceae; Mycobacterium; Mycobacterium tuberculosis complex; Mycobacterium tuberculosis;	NCBI: 1448658	4437890	99.94	0.00	Yes
NCBI Assembly	GCA_000663465.1	NCBI: cellular organisms; Bacteria; Terrabacteria group; Actinobacteria; Actinomycetia; Corynebacteriales; Mycobacteriaceae; Mycobacterium; Mycobacterium tuberculosis complex; Mycobacterium tuberculosis;	NCBI: 1448706	4391247	99.94	0.08	Yes
NCBI Assembly	GCA_000659425.1	NCBI: cellular organisms; Bacteria; Terrabacteria group; Actinobacteria; Actinomycetia; Corynebacteriales; Mycobacteriaceae; Mycobacterium; Mycobacterium tuberculosis complex; Mycobacterium tuberculosis;	NCBI: 1295745	4401477	99.94	0.00	Yes
NCBI Assembly	GCA_000651755.1	NCBI: cellular organisms; Bacteria; Terrabacteria group; Actinobacteria; Actinomycetia; Corynebacteriales; Mycobacteriaceae; Mycobacterium; Mycobacterium tuberculosis complex; Mycobacterium tuberculosis;	NCBI: 1448414	4402452	99.94	0.00	Yes
NCBI Assembly	GCA_000257225.2	NCBI: cellular organisms; Bacteria; Terrabacteria group; Actinobacteria; Actinomycetia; Corynebacteriales; Mycobacteriaceae; Mycobacteroides; Mycobacteroides abscessus;	NCBI: 1149134	5073598	100.00	0.00	Yes
NCBI Assembly	GCA_000659145.1	NCBI: cellular organisms; Bacteria; Terrabacteria group; Actinobacteria; Actinomycetia; Corynebacteriales; Mycobacteriaceae; Mycobacterium; Mycobacterium tuberculosis complex; Mycobacterium tuberculosis;	NCBI: 1267362	4406038	99.86	0.00	Yes
NCBI Assembly	GCA_000660705.1	NCBI: cellular organisms; Bacteria; Terrabacteria group; Actinobacteria; Actinomycetia; Corynebacteriales; Mycobacteriaceae; Mycobacterium; Mycobacterium tuberculosis complex; Mycobacterium tuberculosis;	NCBI: 1324241	4409393	99.94	0.00	Yes
NCBI Assembly	GCA_000656455.1	NCBI: cellular organisms; Bacteria; Terrabacteria group; Actinobacteria; Actinomycetia; Corynebacteriales; Mycobacteriaceae; Mycobacterium; Mycobacterium tuberculosis complex; Mycobacterium tuberculosis;	NCBI: 1438839	4401206	99.94	0.00	Yes
NCBI Assembly	GCA_000661655.1	NCBI: cellular organisms; Bacteria; Terrabacteria group; Actinobacteria; Actinomycetia; Corynebacteriales; Mycobacteriaceae; Mycobacterium; Mycobacterium tuberculosis complex; Mycobacterium tuberculosis;	NCBI: 1324291	4424188	99.94	0.00	Yes
NCBI Assembly	GCA_000649215.1	NCBI: cellular organisms; Bacteria; Terrabacteria group; Actinobacteria; Actinomycetia; Corynebacteriales; Mycobacteriaceae; Mycobacterium; Mycobacterium tuberculosis complex; Mycobacterium tuberculosis;	NCBI: 1438865	4396752	99.94	0.00	Yes
NCBI Assembly	GCA_000443575.1	NCBI: cellular organisms; Bacteria; Terrabacteria group; Actinobacteria; Actinomycetia; Corynebacteriales; Mycobacteriaceae; Mycobacterium; Mycobacterium tuberculosis complex; Mycobacterium tuberculosis;	NCBI: 1346772	4404247	99.94	0.00	Yes

NCBI Assembly	GCA_000662525.1	NCBI: cellular organisms; Bacteria; Terrabacteria group; Actinobacteria; Actinomycetia; Corynebacteriales; Mycobacteriaceae; Mycobacterium; Mycobacterium tuberculosis complex; Mycobacterium tuberculosis;	NCBI: 1448659	4420575	99.61	0.00	Yes
NCBI Assembly	GCA_000559085.2	NCBI: cellular organisms; Bacteria; Terrabacteria group; Actinobacteria; Actinomycetia; Corynebacteriales; Mycobacteriaceae; Mycolicibacterium; Mycolicibacterium aromaticivorans;	NCBI: 1440774	6297623	100.00	0.06	Yes
NCBI Assembly	GCA_000663485.1	NCBI: cellular organisms; Bacteria; Terrabacteria group; Actinobacteria; Actinomycetia; Corynebacteriales; Mycobacteriaceae; Mycobacterium; Mycobacterium tuberculosis complex; Mycobacterium tuberculosis;	NCBI: 1448707	4414628	99.94	0.08	Yes
NCBI Assembly	GCA_000659445.1	NCBI: cellular organisms; Bacteria; Terrabacteria group; Actinobacteria; Actinomycetia; Corynebacteriales; Mycobacteriaceae; Mycobacterium; Mycobacterium tuberculosis complex; Mycobacterium tuberculosis;	NCBI: 1295746	4405831	99.94	0.00	Yes
NCBI Assembly	GCA_000651775.1	NCBI: cellular organisms; Bacteria; Terrabacteria group; Actinobacteria; Actinomycetia; Corynebacteriales; Mycobacteriaceae; Mycobacterium; Mycobacterium tuberculosis complex; Mycobacterium tuberculosis;	NCBI: 1448417	4424050	99.94	0.00	Yes
NCBI Assembly	GCA_000257245.2	NCBI: cellular organisms; Bacteria; Terrabacteria group; Actinobacteria; Actinomycetia; Corynebacteriales; Mycobacteriaceae; Mycobacteroides; Mycobacteroides abscessus;	NCBI: 1158135	5090110	99.61	0.00	Yes
NCBI Assembly	GCA_000659165.1	NCBI: cellular organisms; Bacteria; Terrabacteria group; Actinobacteria; Actinomycetia; Corynebacteriales; Mycobacteriaceae; Mycobacterium; Mycobacterium tuberculosis complex; Mycobacterium tuberculosis;	NCBI: 1267363	4427786	99.61	0.00	Yes
NCBI Assembly	GCA_000661675.1	NCBI: cellular organisms; Bacteria; Terrabacteria group; Actinobacteria; Actinomycetia; Corynebacteriales; Mycobacteriaceae; Mycobacterium; Mycobacterium tuberculosis complex; Mycobacterium tuberculosis;	NCBI: 1324292	4362546	99.94	0.00	Yes
NCBI Assembly	GCA_000660725.1	NCBI: cellular organisms; Bacteria; Terrabacteria group; Actinobacteria; Actinomycetia; Corynebacteriales; Mycobacteriaceae; Mycobacterium; Mycobacterium tuberculosis complex; Mycobacterium tuberculosis;	NCBI: 1324242	4416208	99.94	0.00	Yes
NCBI Assembly	GCA_000649235.1	NCBI: cellular organisms; Bacteria; Terrabacteria group; Actinobacteria; Actinomycetia; Corynebacteriales; Mycobacteriaceae; Mycobacterium; Mycobacterium tuberculosis complex; Mycobacterium tuberculosis;	NCBI: 1438843	4498376	99.94	2.83	Yes
NCBI Assembly	GCA_000332605.1	NCBI: cellular organisms; Bacteria; Terrabacteria group; Actinobacteria; Actinomycetia; Corynebacteriales; Mycobacteriaceae; Mycobacteroides; Mycobacteroides abscessus;	NCBI: 1246644	5218578	99.83	0.00	Yes
NCBI Assembly	GCA_000662545.1	NCBI: cellular organisms; Bacteria; Terrabacteria group; Actinobacteria; Actinomycetia; Corynebacteriales; Mycobacteriaceae; Mycobacterium; Mycobacterium tuberculosis complex; Mycobacterium tuberculosis;	NCBI: 1448660	4426785	99.94	0.00	Yes
NCBI Assembly	GCA_000656475.1	NCBI: cellular organisms; Bacteria; Terrabacteria group; Actinobacteria; Actinomycetia; Corynebacteriales; Mycobacteriaceae; Mycobacterium; Mycobacterium tuberculosis complex; Mycobacterium tuberculosis;	NCBI: 1438841	4422785	99.94	0.00	Yes
NCBI Assembly	GCA_000568275.1	NCBI: cellular organisms; Bacteria; Terrabacteria group; Actinobacteria; Actinomycetia; Corynebacteriales; Mycobacteriaceae; Mycobacterium; Mycobacterium tuberculosis complex; Mycobacterium tuberculosis;	NCBI: 1279039	4293767	99.94	0.06	Yes
NCBI Assembly	GCA_000663505.1	NCBI: cellular organisms; Bacteria; Terrabacteria group; Actinobacteria; Actinomycetia; Corynebacteriales; Mycobacteriaceae; Mycobacterium; Mycobacterium tuberculosis complex; Mycobacterium tuberculosis;	NCBI: 1448708	4411561	99.78	0.00	Yes
NCBI Assembly	GCA_000659465.1	NCBI: cellular organisms; Bacteria; Terrabacteria group; Actinobacteria; Actinomycetia; Corynebacteriales; Mycobacteriaceae; Mycobacterium; Mycobacterium tuberculosis complex; Mycobacterium tuberculosis;	NCBI: 1295747	4404630	99.94	0.00	Yes
NCBI Assembly	GCA_000652435.1	NCBI: cellular organisms; Bacteria; Terrabacteria group; Actinobacteria; Actinomycetia; Corynebacteriales; Mycobacteriaceae; Mycobacterium; Mycobacterium tuberculosis complex; Mycobacterium tuberculosis;	NCBI: 1448464	4401485	99.94	0.00	Yes
NCBI Assembly	GCA_000257725.1	NCBI: cellular organisms; Bacteria; Terrabacteria group; Actinobacteria; Actinomycetia; Corynebacteriales; Mycobacteriaceae; Mycolicibacterium; Mycolicibacterium phlei;	NCBI: 1150599	5681954	100.00	1.06	Yes

NCBI Assembly	GCA_000659185.1	NCBI: cellular organisms; Bacteria; Terrabacteria group; Actinobacteria; Actinomycetia; Corynebacteriales; Mycobacteriaceae; Mycobacterium; Mycobacterium tuberculosis complex; Mycobacterium tuberculosis;	NCBI: 1267364	4417406	99.94	0.00	Yes
NCBI Assembly	GCA_000649255.1	NCBI: cellular organisms; Bacteria; Terrabacteria group; Actinobacteria; Actinomycetia; Corynebacteriales; Mycobacteriaceae; Mycobacterium; Mycobacterium tuberculosis complex; Mycobacterium tuberculosis;	NCBI: 1438860	4481522	99.94	0.97	Yes
NCBI Assembly	GCA_000661695.1	NCBI: cellular organisms; Bacteria; Terrabacteria group; Actinobacteria; Actinomycetia; Corynebacteriales; Mycobacteriaceae; Mycobacterium; Mycobacterium tuberculosis complex; Mycobacterium tuberculosis;	NCBI: 1324293	4411818	99.94	0.00	Yes
NCBI Assembly	GCA_000660745.1	NCBI: cellular organisms; Bacteria; Terrabacteria group; Actinobacteria; Actinomycetia; Corynebacteriales; Mycobacteriaceae; Mycobacterium; Mycobacterium tuberculosis complex; Mycobacterium tuberculosis;	NCBI: 1324243	4407199	99.94	0.00	Yes
NCBI Assembly	GCA_000655555.1	NCBI: cellular organisms; Bacteria; Terrabacteria group; Actinobacteria; Actinomycetia; Corynebacteriales; Mycobacteriaceae; Mycobacterium; Mycobacterium tuberculosis complex; Mycobacterium tuberculosis;	NCBI: 1427286	4421841	99.94	0.00	Yes
NCBI Assembly	GCA_000662565.1	NCBI: cellular organisms; Bacteria; Terrabacteria group; Actinobacteria; Actinomycetia; Corynebacteriales; Mycobacteriaceae; Mycobacterium; Mycobacterium tuberculosis complex; Mycobacterium tuberculosis;	NCBI: 1448661	4452073	99.94	0.00	Yes
NCBI Assembly	GCA_000333695.1	NCBI: cellular organisms; Bacteria; Terrabacteria group; Actinobacteria; Actinomycetia; Corynebacteriales; Mycobacteriaceae; Mycobacteroides; Mycobacteroides abscessus;	NCBI: 1166017	4987320	100.00	0.00	Yes
NCBI Assembly	GCA_000663525.1	NCBI: cellular organisms; Bacteria; Terrabacteria group; Actinobacteria; Actinomycetia; Corynebacteriales; Mycobacteriaceae; Mycobacterium; Mycobacterium tuberculosis complex; Mycobacterium tuberculosis;	NCBI: 1448709	4435307	99.94	0.48	Yes
NCBI Assembly	GCA_000504945.1	NCBI: cellular organisms; Bacteria; Terrabacteria group; Actinobacteria; Actinomycetia; Corynebacteriales; Mycobacteriaceae; Mycobacterium; Mycobacterium avium complex (MAC); Mycobacterium avium; Mycobacterium avium subsp. avium;	NCBI: 1391991	4794583	95.22	1.63	Yes
NCBI Assembly	GCA_000659485.1	NCBI: cellular organisms; Bacteria; Terrabacteria group; Actinobacteria; Actinomycetia; Corynebacteriales; Mycobacteriaceae; Mycobacterium; Mycobacterium tuberculosis complex; Mycobacterium tuberculosis;	NCBI: 1295751	4402839	99.94	0.00	Yes
NCBI Assembly	GCA_000652455.1	NCBI: cellular organisms; Bacteria; Terrabacteria group; Actinobacteria; Actinomycetia; Corynebacteriales; Mycobacteriaceae; Mycobacterium; Mycobacterium tuberculosis complex; Mycobacterium tuberculosis;	NCBI: 1448465	4399087	99.94	0.00	Yes
NCBI Assembly	GCA_000271025.1	NCBI: cellular organisms; Bacteria; Terrabacteria group; Actinobacteria; Actinomycetia; Corynebacteriales; Mycobacteriaceae; Mycobacteroides; Mycobacteroides abscessus;	NCBI: 1001733	5239840	100.00	0.00	Yes
NCBI Assembly	GCA_000658195.1	NCBI: cellular organisms; Bacteria; Terrabacteria group; Actinobacteria; Actinomycetia; Corynebacteriales; Mycobacteriaceae; Mycobacterium; Mycobacterium tuberculosis complex; Mycobacterium tuberculosis;	NCBI: 1447436	4416563	99.61	0.36	Yes
NCBI Assembly	GCA_000649275.1	NCBI: cellular organisms; Bacteria; Terrabacteria group; Actinobacteria; Actinomycetia; Corynebacteriales; Mycobacteriaceae; Mycobacterium; Mycobacterium tuberculosis complex; Mycobacterium tuberculosis;	NCBI: 1438859	4435168	99.67	0.67	Yes
NCBI Assembly	GCA_000649935.1	NCBI: cellular organisms; Bacteria; Terrabacteria group; Actinobacteria; Actinomycetia; Corynebacteriales; Mycobacteriaceae; Mycobacterium; Mycobacterium tuberculosis complex; Mycobacterium tuberculosis;	NCBI: 1447443	4377753	99.94	0.00	Yes
NCBI Assembly	GCA_000649615.1	NCBI: cellular organisms; Bacteria; Terrabacteria group; Actinobacteria; Actinomycetia; Corynebacteriales; Mycobacteriaceae; Mycobacterium; Mycobacterium tuberculosis complex; Mycobacterium tuberculosis;	NCBI: 1438833	4406733	99.94	0.00	Yes
NCBI Assembly	GCA_000655575.1	NCBI: cellular organisms; Bacteria; Terrabacteria group; Actinobacteria; Actinomycetia; Corynebacteriales; Mycobacteriaceae; Mycobacterium; Mycobacterium tuberculosis complex; Mycobacterium tuberculosis;	NCBI: 1427287	4429878	99.94	0.00	Yes
NCBI Assembly	GCA_000650875.1	NCBI: cellular organisms; Bacteria; Terrabacteria group; Actinobacteria; Actinomycetia; Corynebacteriales; Mycobacteriaceae; Mycobacterium; Mycobacterium tuberculosis complex; Mycobacterium tuberculosis;	NCBI: 1447501	4384623	99.94	0.00	Yes

NCBI Assembly	GCA_000340435.3	NCBI: cellular organisms; Bacteria; Terrabacteria group; Actinobacteria; Actinomycetia; Corynebacteriales; Mycobacteriaceae; Mycobacterium; Mycobacterium haemophilum;	NCBI: 1202450	4235765	99.13	1.61	Yes
NCBI Assembly	GCA_000652115.1	NCBI: cellular organisms; Bacteria; Terrabacteria group; Actinobacteria; Actinomycetia; Corynebacteriales; Mycobacteriaceae; Mycobacterium; Mycobacterium tuberculosis complex; Mycobacterium tuberculosis;	NCBI: 1448446	4405925	99.94	0.00	Yes
NCBI Assembly	GCA_000660145.1	NCBI: cellular organisms; Bacteria; Terrabacteria group; Actinobacteria; Actinomycetia; Corynebacteriales; Mycobacteriaceae; Mycobacterium; Mycobacterium tuberculosis complex; Mycobacterium tuberculosis;	NCBI: 1295804	4393134	99.94	0.00	Yes
NCBI Assembly	GCA_000271045.1	NCBI: cellular organisms; Bacteria; Terrabacteria group; Actinobacteria; Actinomycetia; Corynebacteriales; Mycobacteriaceae; Mycobacteroides; Mycobacteroides abscessus;	NCBI: 1001734	5206300	98.67	0.00	Yes
NCBI Assembly	GCA_000652475.1	NCBI: cellular organisms; Bacteria; Terrabacteria group; Actinobacteria; Actinomycetia; Corynebacteriales; Mycobacteriaceae; Mycobacterium; Mycobacterium tuberculosis complex; Mycobacterium tuberculosis;	NCBI: 1448466	4421715	99.94	0.00	Yes
NCBI Assembly	GCA_000658215.1	NCBI: cellular organisms; Bacteria; Terrabacteria group; Actinobacteria; Actinomycetia; Corynebacteriales; Mycobacteriaceae; Mycobacterium; Mycobacterium tuberculosis complex; Mycobacterium tuberculosis;	NCBI: 1447448	4401782	99.94	0.00	Yes
NCBI Assembly	GCA_000504975.1	NCBI: cellular organisms; Bacteria; Terrabacteria group; Actinobacteria; Actinomycetia; Corynebacteriales; Mycobacteriaceae; Mycobacterium; Mycobacterium avium complex (MAC); Mycobacterium avium; Mycobacterium avium subsp. silvaticum;	NCBI: 1401690	4713173	96.53	1.44	Yes
NCBI Assembly	GCA_000499985.1	NCBI: cellular organisms; Bacteria; Terrabacteria group; Actinobacteria; Actinomycetia; Corynebacteriales; Mycobacteriaceae; Mycobacterium; Mycobacterium avium complex (MAC); Mycobacterium avium; Mycobacterium avium subsp. hominissuis;	NCBI: 1402214	5211349	85.86	4.62	No
NCBI Assembly	GCA_000649955.1	NCBI: cellular organisms; Bacteria; Terrabacteria group; Actinobacteria; Actinomycetia; Corynebacteriales; Mycobacteriaceae; Mycobacterium; Mycobacterium tuberculosis complex; Mycobacterium tuberculosis;	NCBI: 1447444	4430724	99.94	0.00	Yes
NCBI Assembly	GCA_000655595.1	NCBI: cellular organisms; Bacteria; Terrabacteria group; Actinobacteria; Actinomycetia; Corynebacteriales; Mycobacteriaceae; Mycobacterium; Mycobacterium tuberculosis complex; Mycobacterium tuberculosis;	NCBI: 1427288	4393762	99.94	0.00	Yes
NCBI Assembly	GCA_000649635.1	NCBI: cellular organisms; Bacteria; Terrabacteria group; Actinobacteria; Actinomycetia; Corynebacteriales; Mycobacteriaceae; Mycobacterium; Mycobacterium tuberculosis complex; Mycobacterium tuberculosis;	NCBI: 1438828	4390429	99.94	0.00	Yes
NCBI Assembly	GCA_000650895.1	NCBI: cellular organisms; Bacteria; Terrabacteria group; Actinobacteria; Actinomycetia; Corynebacteriales; Mycobacteriaceae; Mycobacterium; Mycobacterium tuberculosis complex; Mycobacterium tuberculosis;	NCBI: 1447502	4385693	99.94	0.00	Yes
NCBI Assembly	GCA_000652135.1	NCBI: cellular organisms; Bacteria; Terrabacteria group; Actinobacteria; Actinomycetia; Corynebacteriales; Mycobacteriaceae; Mycobacterium; Mycobacterium tuberculosis complex; Mycobacterium tuberculosis;	NCBI: 1448447	4421097	98.52	0.00	Yes
NCBI Assembly	GCA_000660165.1	NCBI: cellular organisms; Bacteria; Terrabacteria group; Actinobacteria; Actinomycetia; Corynebacteriales; Mycobacteriaceae; Mycobacterium; Mycobacterium tuberculosis complex; Mycobacterium tuberculosis;	NCBI: 1295805	4386306	99.78	0.00	Yes
NCBI Assembly	GCA_000271065.1	NCBI: cellular organisms; Bacteria; Terrabacteria group; Actinobacteria; Actinomycetia; Corynebacteriales; Mycobacteriaceae; Mycobacteroides; Mycobacteroides abscessus;	NCBI: 1001731	5323965	100.00	0.00	Yes
NCBI Assembly	GCA_000652495.1	NCBI: cellular organisms; Bacteria; Terrabacteria group; Actinobacteria; Actinomycetia; Corynebacteriales; Mycobacteriaceae; Mycobacterium; Mycobacterium tuberculosis complex; Mycobacterium tuberculosis;	NCBI: 1448468	4393621	99.94	0.00	Yes
NCBI Assembly	GCA_000504995.1	NCBI: cellular organisms; Bacteria; Terrabacteria group; Actinobacteria; Actinomycetia; Corynebacteriales; Mycobacteriaceae; Mycobacterium; Mycobacterium avium complex (MAC); Mycobacterium avium; Mycobacterium avium subsp. paratuberculosis;	NCBI: 1391993	4673684	93.28	2.54	Yes
NCBI Assembly	GCA_000658235.1	NCBI: cellular organisms; Bacteria; Terrabacteria group; Actinobacteria; Actinomycetia; Corynebacteriales; Mycobacteriaceae; Mycobacterium; Mycobacterium tuberculosis complex; Mycobacterium tuberculosis;	NCBI: 1447456	4385216	99.94	0.00	Yes

NCBI Assembly	GCA_000500005.1	NCBI: cellular organisms; Bacteria; Terrabacteria group; Actinobacteria; Actinomycetia; Corynebacteriales; Mycobacteriaceae; Mycobacterium; Mycobacterium avium complex (MAC); Mycobacterium avium; Mycobacterium avium subsp. hominissuis;	NCBI: 1402213	4793926	94.68	1.29	Yes
NCBI Assembly	GCA_000649975.1	NCBI: cellular organisms; Bacteria; Terrabacteria group; Actinobacteria; Actinomycetia; Corynebacteriales; Mycobacteriaceae; Mycobacterium; Mycobacterium tuberculosis complex; Mycobacterium tuberculosis;	NCBI: 1447446	4388605	99.94	0.00	Yes
NCBI Assembly	GCA_000655615.1	NCBI: cellular organisms; Bacteria; Terrabacteria group; Actinobacteria; Actinomycetia; Corynebacteriales; Mycobacteriaceae; Mycobacterium; Mycobacterium tuberculosis complex; Mycobacterium tuberculosis;	NCBI: 1427289	4400076	99.94	0.00	Yes
NCBI Assembly	GCA_000649655.1	NCBI: cellular organisms; Bacteria; Terrabacteria group; Actinobacteria; Actinomycetia; Corynebacteriales; Mycobacteriaceae; Mycobacterium; Mycobacterium tuberculosis complex; Mycobacterium tuberculosis; Mycobacterium tuberculosis variant bovis;	NCBI: 1438823	4272013	99.36	0.44	Yes
NCBI Assembly	GCA_000650915.1	NCBI: cellular organisms; Bacteria; Terrabacteria group; Actinobacteria; Actinomycetia; Corynebacteriales; Mycobacteriaceae; Mycobacterium; Mycobacterium tuberculosis complex; Mycobacterium tuberculosis;	NCBI: 1447504	4379435	99.94	0.00	Yes
NCBI Assembly	GCA_000652155.1	NCBI: cellular organisms; Bacteria; Terrabacteria group; Actinobacteria; Actinomycetia; Corynebacteriales; Mycobacteriaceae; Mycobacterium; Mycobacterium tuberculosis complex; Mycobacterium tuberculosis;	NCBI: 1448448	4413015	99.94	0.00	Yes
NCBI Assembly	GCA_000660185.1	NCBI: cellular organisms; Bacteria; Terrabacteria group; Actinobacteria; Actinomycetia; Corynebacteriales; Mycobacteriaceae; Mycobacterium; Mycobacterium tuberculosis complex; Mycobacterium tuberculosis;	NCBI: 1295806	4398280	99.61	0.00	Yes
NCBI Assembly	GCA_000271085.1	NCBI: cellular organisms; Bacteria; Terrabacteria group; Actinobacteria; Actinomycetia; Corynebacteriales; Mycobacteriaceae; Mycobacteroides; Mycobacteroides abscessus;	NCBI: 1001730	5253252	100.00	0.00	Yes
NCBI Assembly	GCA_000343915.1	NCBI: cellular organisms; Bacteria; Terrabacteria group; Actinobacteria; Actinomycetia; Corynebacteriales; Mycobacteriaceae; Mycobacterium; Mycobacterium tuberculosis complex; Mycobacterium canettii;	NCBI: 1246121	4341599	99.88	0.22	Yes
NCBI Assembly	GCA_000652515.1	NCBI: cellular organisms; Bacteria; Terrabacteria group; Actinobacteria; Actinomycetia; Corynebacteriales; Mycobacteriaceae; Mycobacterium; Mycobacterium tuberculosis complex; Mycobacterium tuberculosis;	NCBI: 1448469	4406669	99.94	0.00	Yes
NCBI Assembly	GCA_000500165.1	NCBI: cellular organisms; Bacteria; Terrabacteria group; Actinobacteria; Actinomycetia; Corynebacteriales; Mycobacteriaceae; Mycobacteroides; Mycobacteroides abscessus;	NCBI: 1335411	4798287	98.46	0.00	Yes
NCBI Assembly	GCA_000649995.1	NCBI: cellular organisms; Bacteria; Terrabacteria group; Actinobacteria; Actinomycetia; Corynebacteriales; Mycobacteriaceae; Mycobacterium; Mycobacterium tuberculosis complex; Mycobacterium tuberculosis;	NCBI: 1447447	4374662	99.94	0.00	Yes
NCBI Assembly	GCA_000505015.1	NCBI: cellular organisms; Bacteria; Terrabacteria group; Actinobacteria; Actinomycetia; Corynebacteriales; Mycobacteriaceae; Mycobacterium; Mycobacterium avium complex (MAC); Mycobacterium avium; Mycobacterium avium subsp. avium;	NCBI: 1391992	4835382	96.27	0.90	Yes
NCBI Assembly	GCA_000655635.1	NCBI: cellular organisms; Bacteria; Terrabacteria group; Actinobacteria; Actinomycetia; Corynebacteriales; Mycobacteriaceae; Mycobacterium; Mycobacterium tuberculosis complex; Mycobacterium tuberculosis;	NCBI: 1427290	4418259	99.94	0.00	Yes
NCBI Assembly	GCA_000658255.1	NCBI: cellular organisms; Bacteria; Terrabacteria group; Actinobacteria; Actinomycetia; Corynebacteriales; Mycobacteriaceae; Mycobacterium; Mycobacterium tuberculosis complex; Mycobacterium tuberculosis;	NCBI: 1447477	4385981	99.94	0.00	Yes
NCBI Assembly	GCA_000650935.1	NCBI: cellular organisms; Bacteria; Terrabacteria group; Actinobacteria; Actinomycetia; Corynebacteriales; Mycobacteriaceae; Mycobacterium; Mycobacterium tuberculosis complex; Mycobacterium tuberculosis;	NCBI: 1447507	4431458	99.94	0.40	Yes
NCBI Assembly	GCA_000649675.1	NCBI: cellular organisms; Bacteria; Terrabacteria group; Actinobacteria; Actinomycetia; Corynebacteriales; Mycobacteriaceae; Mycobacterium; Mycobacterium tuberculosis complex; Mycobacterium tuberculosis; Mycobacterium tuberculosis variant bovis;	NCBI: 1438822	4174847	96.99	0.00	Yes
NCBI Assembly	GCA_000652175.1	NCBI: cellular organisms; Bacteria; Terrabacteria group; Actinobacteria; Actinomycetia; Corynebacteriales; Mycobacteriaceae; Mycobacterium; Mycobacterium tuberculosis complex; Mycobacterium tuberculosis;	NCBI: 1448449	4416088	99.94	0.00	Yes

NCBI Assembly	GCA_000660205.1	NCBI: cellular organisms; Bacteria; Terrabacteria group; Actinobacteria; Actinomycetia; Corynebacteriales; Mycobacteriaceae; Mycobacterium; Mycobacterium tuberculosis complex; Mycobacterium tuberculosis;	NCBI: 1295807	4403424	99.94	0.00	Yes
NCBI Assembly	GCA_000271105.1	NCBI: cellular organisms; Bacteria; Terrabacteria group; Actinobacteria; Actinomycetia; Corynebacteriales; Mycobacteriaceae; Mycobacteroides; Mycobacteroides abscessus;	NCBI: 1001717	5287644	100.00	0.00	Yes
NCBI Assembly	GCA_000652535.1	NCBI: cellular organisms; Bacteria; Terrabacteria group; Actinobacteria; Actinomycetia; Corynebacteriales; Mycobacteriaceae; Mycobacterium; Mycobacterium tuberculosis complex; Mycobacterium tuberculosis;	NCBI: 1448470	4415573	99.94	0.00	Yes
NCBI Assembly	GCA_000509285.1	NCBI: cellular organisms; Bacteria; Terrabacteria group; Actinobacteria; Actinomycetia; Corynebacteriales; Mycobacteriaceae; Mycobacterium; Mycobacterium tuberculosis complex; Mycobacterium tuberculosis;	NCBI: 1338505	3914047	91.28	0.00	Yes
NCBI Assembly	GCA_000650015.1	NCBI: cellular organisms; Bacteria; Terrabacteria group; Actinobacteria; Actinomycetia; Corynebacteriales; Mycobacteriaceae; Mycobacterium; Mycobacterium tuberculosis complex; Mycobacterium tuberculosis;	NCBI: 1447449	4406592	99.94	0.00	Yes
NCBI Assembly	GCA_000500185.1	NCBI: cellular organisms; Bacteria; Terrabacteria group; Actinobacteria; Actinomycetia; Corynebacteriales; Mycobacteriaceae; Mycobacteroides; Mycobacteroides abscessus;	NCBI: 1335414	4832942	99.57	0.58	Yes
NCBI Assembly	GCA_000658275.1	NCBI: cellular organisms; Bacteria; Terrabacteria group; Actinobacteria; Actinomycetia; Corynebacteriales; Mycobacteriaceae; Mycobacterium; Mycobacterium tuberculosis complex; Mycobacterium tuberculosis;	NCBI: 1447478	4398786	99.94	0.54	Yes
NCBI Assembly	GCA_000655655.1	NCBI: cellular organisms; Bacteria; Terrabacteria group; Actinobacteria; Actinomycetia; Corynebacteriales; Mycobacteriaceae; Mycobacterium; Mycobacterium tuberculosis complex; Mycobacterium tuberculosis;	NCBI: 1427291	4400427	99.94	0.00	Yes
NCBI Assembly	GCA_000649695.1	NCBI: cellular organisms; Bacteria; Terrabacteria group; Actinobacteria; Actinomycetia; Corynebacteriales; Mycobacteriaceae; Mycobacterium; Mycobacterium tuberculosis complex; Mycobacterium tuberculosis;	NCBI: 1438829	4371859	99.94	0.00	Yes
NCBI Assembly	GCA_000650955.1	NCBI: cellular organisms; Bacteria; Terrabacteria group; Actinobacteria; Actinomycetia; Corynebacteriales; Mycobacteriaceae; Mycobacterium; Mycobacterium tuberculosis complex; Mycobacterium tuberculosis;	NCBI: 1447508	4417888	99.94	0.00	Yes
NCBI Assembly	GCA_000353205.1	NCBI: cellular organisms; Bacteria; Terrabacteria group; Actinobacteria; Actinomycetia; Corynebacteriales; Mycobacteriaceae; Mycobacterium; Mycobacterium tuberculosis complex; Mycobacterium orygis;	NCBI: 1305739	4282661	99.61	0.00	Yes
NCBI Assembly	GCA_000652195.1	NCBI: cellular organisms; Bacteria; Terrabacteria group; Actinobacteria; Actinomycetia; Corynebacteriales; Mycobacteriaceae; Mycobacterium; Mycobacterium tuberculosis complex; Mycobacterium tuberculosis;	NCBI: 1448450	4499402	99.61	0.00	Yes
NCBI Assembly	GCA_000660225.1	NCBI: cellular organisms; Bacteria; Terrabacteria group; Actinobacteria; Actinomycetia; Corynebacteriales; Mycobacteriaceae; Mycobacterium; Mycobacterium tuberculosis complex; Mycobacterium tuberculosis;	NCBI: 1295808	4415849	99.94	0.00	Yes
NCBI Assembly	GCA_000271125.1	NCBI: cellular organisms; Bacteria; Terrabacteria group; Actinobacteria; Actinomycetia; Corynebacteriales; Mycobacteriaceae; Mycobacteroides; Mycobacteroides abscessus;	NCBI: 1001722	4864284	100.00	0.00	Yes
NCBI Assembly	GCA_000652555.1	NCBI: cellular organisms; Bacteria; Terrabacteria group; Actinobacteria; Actinomycetia; Corynebacteriales; Mycobacteriaceae; Mycobacterium; Mycobacterium tuberculosis complex; Mycobacterium tuberculosis;	NCBI: 1448471	4415263	99.94	0.00	Yes
NCBI Assembly	GCA_000510935.1	NCBI: cellular organisms; Bacteria; Terrabacteria group; Actinobacteria; Actinomycetia; Corynebacteriales; Mycobacteriaceae; Mycobacterium; Mycobacterium tuberculosis complex; Mycobacterium tuberculosis;	NCBI: 1432058	4320561	99.17	1.00	Yes
NCBI Assembly	GCA_000650035.1	NCBI: cellular organisms; Bacteria; Terrabacteria group; Actinobacteria; Actinomycetia; Corynebacteriales; Mycobacteriaceae; Mycobacterium; Mycobacterium tuberculosis complex; Mycobacterium tuberculosis;	NCBI: 1447451	4394882	99.94	0.00	Yes
NCBI Assembly	GCA_000658315.1	NCBI: cellular organisms; Bacteria; Terrabacteria group; Actinobacteria; Actinomycetia; Corynebacteriales; Mycobacteriaceae; Mycobacterium; Mycobacterium tuberculosis complex; Mycobacterium tuberculosis;	NCBI: 1421970	4394779	99.94	0.00	Yes

NCBI Assembly	GCA_000504725.1	NCBI: cellular organisms; Bacteria; Terrabacteria group; Actinobacteria; Actinomycetia; Corynebacteriales; Mycobacteriaceae; Mycobacterium; Mycobacterium avium complex (MAC); Mycobacterium avium; Mycobacterium avium subsp. paratuberculosis;	NCBI: 1391998	4876082	93.10	1.00	Yes
NCBI Assembly	GCA_000655675.1	NCBI: cellular organisms; Bacteria; Terrabacteria group; Actinobacteria; Actinomycetia; Corynebacteriales; Mycobacteriaceae; Mycobacterium; Mycobacterium tuberculosis complex; Mycobacterium tuberculosis;	NCBI: 1427292	4428349	99.94	0.00	Yes
NCBI Assembly	GCA_000649715.1	NCBI: cellular organisms; Bacteria; Terrabacteria group; Actinobacteria; Actinomycetia; Corynebacteriales; Mycobacteriaceae; Mycobacterium; Mycobacterium tuberculosis complex; Mycobacterium tuberculosis;	NCBI: 1438827	4388747	99.94	0.00	Yes
NCBI Assembly	GCA_000650975.1	NCBI: cellular organisms; Bacteria; Terrabacteria group; Actinobacteria; Actinomycetia; Corynebacteriales; Mycobacteriaceae; Mycobacterium; Mycobacterium tuberculosis complex; Mycobacterium tuberculosis;	NCBI: 1447509	4380054	99.94	0.00	Yes
NCBI Assembly	GCA_000652215.1	NCBI: cellular organisms; Bacteria; Terrabacteria group; Actinobacteria; Actinomycetia; Corynebacteriales; Mycobacteriaceae; Mycobacterium; Mycobacterium tuberculosis complex; Mycobacterium tuberculosis;	NCBI: 1448452	4401319	99.94	0.00	Yes
NCBI Assembly	GCA_000660245.1	NCBI: cellular organisms; Bacteria; Terrabacteria group; Actinobacteria; Actinomycetia; Corynebacteriales; Mycobacteriaceae; Mycobacterium; Mycobacterium tuberculosis complex; Mycobacterium tuberculosis;	NCBI: 1324218	4412109	99.94	0.00	Yes
NCBI Assembly	GCA_000363915.2	NCBI: cellular organisms; Bacteria; Terrabacteria group; Actinobacteria; Actinomycetia; Corynebacteriales; Mycobacteriaceae; Mycobacteroides; Mycobacteroides abscessus; Mycobacteroides abscessus subsp. bolletii;	NCBI: 1087469	4941231	100.00	1.50	Yes
NCBI Assembly	GCA_000271145.1	NCBI: cellular organisms; Bacteria; Terrabacteria group; Actinobacteria; Actinomycetia; Corynebacteriales; Mycobacteriaceae; Mycobacteroides; Mycobacteroides abscessus;	NCBI: 1001738	5337035	100.00	0.00	Yes
NCBI Assembly	GCA_000652575.1	NCBI: cellular organisms; Bacteria; Terrabacteria group; Actinobacteria; Actinomycetia; Corynebacteriales; Mycobacteriaceae; Mycobacterium; Mycobacterium tuberculosis complex; Mycobacterium tuberculosis;	NCBI: 1448472	4391929	99.61	0.00	Yes
NCBI Assembly	GCA_000512045.1	NCBI: cellular organisms; Bacteria; Terrabacteria group; Actinobacteria; Actinomycetia; Corynebacteriales; Mycobacteriaceae; Mycobacterium; Mycobacterium tuberculosis complex; Mycobacterium tuberculosis;	NCBI: 1429887	4297193	99.78	0.41	Yes
NCBI Assembly	GCA_000504745.1	NCBI: cellular organisms; Bacteria; Terrabacteria group; Actinobacteria; Actinomycetia; Corynebacteriales; Mycobacteriaceae; Mycobacterium; Mycobacterium avium complex (MAC); Mycobacterium avium;	NCBI: 1392002	5156046	98.07	0.06	Yes
NCBI Assembly	GCA_000650055.1	NCBI: cellular organisms; Bacteria; Terrabacteria group; Actinobacteria; Actinomycetia; Corynebacteriales; Mycobacteriaceae; Mycobacterium; Mycobacterium tuberculosis complex; Mycobacterium tuberculosis;	NCBI: 1447452	4387164	99.94	0.43	Yes
NCBI Assembly	GCA_000658335.1	NCBI: cellular organisms; Bacteria; Terrabacteria group; Actinobacteria; Actinomycetia; Corynebacteriales; Mycobacteriaceae; Mycobacterium; Mycobacterium tuberculosis complex; Mycobacterium tuberculosis;	NCBI: 1448630	4414327	99.94	0.04	Yes
NCBI Assembly	GCA_000649735.1	NCBI: cellular organisms; Bacteria; Terrabacteria group; Actinobacteria; Actinomycetia; Corynebacteriales; Mycobacteriaceae; Mycobacterium; Mycobacterium tuberculosis complex; Mycobacterium tuberculosis;	NCBI: 1438826	4376709	99.94	0.00	Yes
NCBI Assembly	GCA_000655695.1	NCBI: cellular organisms; Bacteria; Terrabacteria group; Actinobacteria; Actinomycetia; Corynebacteriales; Mycobacteriaceae; Mycobacterium; Mycobacterium tuberculosis complex; Mycobacterium tuberculosis;	NCBI: 1427293	4437927	99.94	0.00	Yes
NCBI Assembly	GCA_000650995.1	NCBI: cellular organisms; Bacteria; Terrabacteria group; Actinobacteria; Actinomycetia; Corynebacteriales; Mycobacteriaceae; Mycobacterium; Mycobacterium tuberculosis complex; Mycobacterium tuberculosis;	NCBI: 1447510	4427499	99.61	0.00	Yes
NCBI Assembly	GCA_000364405.1	NCBI: cellular organisms; Bacteria; Terrabacteria group; Actinobacteria; Actinomycetia; Corynebacteriales; Mycobacteriaceae; Mycobacterium; unclassified Mycobacterium;	NCBI: 1168288	5443025	99.94	0.04	Yes
NCBI Assembly	GCA_000652235.1	NCBI: cellular organisms; Bacteria; Terrabacteria group; Actinobacteria; Actinomycetia; Corynebacteriales; Mycobacteriaceae; Mycobacterium; Mycobacterium tuberculosis complex; Mycobacterium tuberculosis;	NCBI: 1448453	4413434	99.94	0.00	Yes

NCBI Assembly	GCA_000660265.1	NCBI: cellular organisms; Bacteria; Terrabacteria group; Actinobacteria; Actinomycetia; Corynebacteriales; Mycobacteriaceae; Mycobacterium; Mycobacterium tuberculosis complex; Mycobacterium tuberculosis;	NCBI: 1324219	4406084	99.94	0.00	Yes
NCBI Assembly	GCA_000271165.1	NCBI: cellular organisms; Bacteria; Terrabacteria group; Actinobacteria; Actinomycetia; Corynebacteriales; Mycobacteriaceae; Mycobacteroides; Mycobacteroides abscessus; Mycobacteroides abscessus subsp. bolletii;	NCBI: 1001704	4895763	100.00	0.00	Yes
NCBI Assembly	GCA_000512065.1	NCBI: cellular organisms; Bacteria; Terrabacteria group; Actinobacteria; Actinomycetia; Corynebacteriales; Mycobacteriaceae; Mycobacterium; Mycobacterium tuberculosis complex; Mycobacterium tuberculosis;	NCBI: 1429886	4259567	99.58	0.42	Yes
NCBI Assembly	GCA_000652595.1	NCBI: cellular organisms; Bacteria; Terrabacteria group; Actinobacteria; Actinomycetia; Corynebacteriales; Mycobacteriaceae; Mycobacterium; Mycobacterium tuberculosis complex; Mycobacterium tuberculosis;	NCBI: 1448473	4415559	99.94	0.00	Yes
NCBI Assembly	GCA_000504765.1	NCBI: cellular organisms; Bacteria; Terrabacteria group; Actinobacteria; Actinomycetia; Corynebacteriales; Mycobacteriaceae; Mycobacterium; Mycobacterium avium complex (MAC); Mycobacterium avium; Mycobacterium avium subsp. hominissuis;	NCBI: 1391990	4786631	95.64	1.51	Yes
NCBI Assembly	GCA_000650075.1	NCBI: cellular organisms; Bacteria; Terrabacteria group; Actinobacteria; Actinomycetia; Corynebacteriales; Mycobacteriaceae; Mycobacterium; Mycobacterium tuberculosis complex; Mycobacterium tuberculosis;	NCBI: 1447453	4414695	99.94	0.00	Yes
NCBI Assembly	GCA_000649755.1	NCBI: cellular organisms; Bacteria; Terrabacteria group; Actinobacteria; Actinomycetia; Corynebacteriales; Mycobacteriaceae; Mycobacterium; Mycobacterium tuberculosis complex; Mycobacterium tuberculosis;	NCBI: 1447432	4385219	99.94	0.00	Yes
NCBI Assembly	GCA_000658355.1	NCBI: cellular organisms; Bacteria; Terrabacteria group; Actinobacteria; Actinomycetia; Corynebacteriales; Mycobacteriaceae; Mycobacterium; Mycobacterium tuberculosis complex; Mycobacterium tuberculosis;	NCBI: 1448631	4422795	99.94	0.00	Yes
NCBI Assembly	GCA_000655715.1	NCBI: cellular organisms; Bacteria; Terrabacteria group; Actinobacteria; Actinomycetia; Corynebacteriales; Mycobacteriaceae; Mycobacterium; Mycobacterium tuberculosis complex; Mycobacterium tuberculosis;	NCBI: 1427294	4424348	99.94	0.00	Yes
NCBI Assembly	GCA_000364945.1	NCBI: cellular organisms; Bacteria; Terrabacteria group; Actinobacteria; Actinomycetia; Corynebacteriales; Mycobacteriaceae; Mycobacterium; Mycobacterium tuberculosis complex; Mycobacterium tuberculosis;	NCBI: 1274431	4448432	93.00	0.00	Yes
NCBI Assembly	GCA_000651015.1	NCBI: cellular organisms; Bacteria; Terrabacteria group; Actinobacteria; Actinomycetia; Corynebacteriales; Mycobacteriaceae; Mycobacterium; Mycobacterium tuberculosis complex; Mycobacterium tuberculosis;	NCBI: 1447511	4413723	99.94	0.00	Yes
NCBI Assembly	GCA_000652255.1	NCBI: cellular organisms; Bacteria; Terrabacteria group; Actinobacteria; Actinomycetia; Corynebacteriales; Mycobacteriaceae; Mycobacterium; Mycobacterium tuberculosis complex; Mycobacterium tuberculosis;	NCBI: 1448454	4401677	99.94	0.00	Yes
NCBI Assembly	GCA_000660285.1	NCBI: cellular organisms; Bacteria; Terrabacteria group; Actinobacteria; Actinomycetia; Corynebacteriales; Mycobacteriaceae; Mycobacterium; Mycobacterium tuberculosis complex; Mycobacterium tuberculosis;	NCBI: 1324220	4405785	99.94	0.00	Yes
NCBI Assembly	GCA_000271185.1	NCBI: cellular organisms; Bacteria; Terrabacteria group; Actinobacteria; Actinomycetia; Corynebacteriales; Mycobacteriaceae; Mycobacteroides; Mycobacteroides abscessus;	NCBI: 1001736	5315024	100.00	0.00	Yes
NCBI Assembly	GCA_000512435.1	NCBI: cellular organisms; Bacteria; Terrabacteria group; Actinobacteria; Actinomycetia; Corynebacteriales; Mycobacteriaceae; Mycobacterium; Mycobacterium avium complex (MAC); Mycobacterium avium;	NCBI: 1391999	4843423	89.10	1.91	No
NCBI Assembly	GCA_000504785.1	NCBI: cellular organisms; Bacteria; Terrabacteria group; Actinobacteria; Actinomycetia; Corynebacteriales; Mycobacteriaceae; Mycobacterium; Mycobacterium avium complex (MAC); Mycobacterium avium; Mycobacterium avium subsp. paratuberculosis;	NCBI: 1391997	4814356	97.13	2.83	Yes
NCBI Assembly	GCA_000652615.1	NCBI: cellular organisms; Bacteria; Terrabacteria group; Actinobacteria; Actinomycetia; Corynebacteriales; Mycobacteriaceae; Mycobacterium; Mycobacterium tuberculosis complex; Mycobacterium tuberculosis;	NCBI: 1448474	4394694	99.61	0.00	Yes
NCBI Assembly	GCA_000650095.1	NCBI: cellular organisms; Bacteria; Terrabacteria group; Actinobacteria; Actinomycetia; Corynebacteriales; Mycobacteriaceae; Mycobacterium; Mycobacterium tuberculosis complex; Mycobacterium tuberculosis;	NCBI: 1447454	4381566	99.94	0.00	Yes

NCBI Assembly	GCA_000658375.1	NCBI: cellular organisms; Bacteria; Terrabacteria group; Actinobacteria; Actinomycetia; Corynebacteriales; Mycobacteriaceae; Mycobacterium; Mycobacterium tuberculosis complex; Mycobacterium tuberculosis;	NCBI: 1448727	4392995	99.94	0.17	Yes
NCBI Assembly	GCA_000649775.1	NCBI: cellular organisms; Bacteria; Terrabacteria group; Actinobacteria; Actinomycetia; Corynebacteriales; Mycobacteriaceae; Mycobacterium; Mycobacterium tuberculosis complex; Mycobacterium tuberculosis;	NCBI: 1447433	4362091	99.94	0.00	Yes
NCBI Assembly	GCA_000372545.3	NCBI: cellular organisms; Bacteria; Terrabacteria group; Actinobacteria; Actinomycetia; Corynebacteriales; Mycobacteriaceae; Mycobacterium; Mycobacterium tuberculosis complex; Mycobacterium tuberculosis;	NCBI: 1279011	4314365	99.61	0.07	Yes
NCBI Assembly	GCA_000655735.1	NCBI: cellular organisms; Bacteria; Terrabacteria group; Actinobacteria; Actinomycetia; Corynebacteriales; Mycobacteriaceae; Mycobacterium; Mycobacterium tuberculosis complex; Mycobacterium tuberculosis;	NCBI: 1427295	4422898	99.94	0.00	Yes
NCBI Assembly	GCA_000651035.1	NCBI: cellular organisms; Bacteria; Terrabacteria group; Actinobacteria; Actinomycetia; Corynebacteriales; Mycobacteriaceae; Mycobacterium; Mycobacterium tuberculosis complex; Mycobacterium tuberculosis;	NCBI: 1447512	4392365	99.94	0.00	Yes
NCBI Assembly	GCA_000652275.1	NCBI: cellular organisms; Bacteria; Terrabacteria group; Actinobacteria; Actinomycetia; Corynebacteriales; Mycobacteriaceae; Mycobacterium; Mycobacterium tuberculosis complex; Mycobacterium tuberculosis;	NCBI: 1448455	4403045	99.94	0.00	Yes
NCBI Assembly	GCA_000660305.1	NCBI: cellular organisms; Bacteria; Terrabacteria group; Actinobacteria; Actinomycetia; Corynebacteriales; Mycobacteriaceae; Mycobacterium; Mycobacterium tuberculosis complex; Mycobacterium tuberculosis;	NCBI: 1324221	4408210	99.94	0.00	Yes
NCBI Assembly	GCA_000271205.1	NCBI: cellular organisms; Bacteria; Terrabacteria group; Actinobacteria; Actinomycetia; Corynebacteriales; Mycobacteriaceae; Mycobacteroides; Mycobacteroides abscessus;	NCBI: 1001728	5253081	100.00	0.00	Yes
NCBI Assembly	GCA_000516395.1	NCBI: cellular organisms; Bacteria; Terrabacteria group; Actinobacteria; Actinomycetia; Corynebacteriales; Mycobacteriaceae; Mycobacterium; Mycobacterium tuberculosis complex; Mycobacterium tuberculosis;	NCBI: 1151113	4319955	99.94	0.11	Yes
NCBI Assembly	GCA_000504805.1	NCBI: cellular organisms; Bacteria; Terrabacteria group; Actinobacteria; Actinomycetia; Corynebacteriales; Mycobacteriaceae; Mycobacterium; Mycobacterium avium complex (MAC); Mycobacterium avium;	NCBI: 1392000	5330193	98.71	0.22	Yes
NCBI Assembly	GCA_000650115.1	NCBI: cellular organisms; Bacteria; Terrabacteria group; Actinobacteria; Actinomycetia; Corynebacteriales; Mycobacteriaceae; Mycobacterium; Mycobacterium tuberculosis complex; Mycobacterium tuberculosis;	NCBI: 1447455	4396396	99.94	0.33	Yes
NCBI Assembly	GCA_000652635.1	NCBI: cellular organisms; Bacteria; Terrabacteria group; Actinobacteria; Actinomycetia; Corynebacteriales; Mycobacteriaceae; Mycobacterium; Mycobacterium tuberculosis complex; Mycobacterium tuberculosis;	NCBI: 1448475	4400469	99.94	0.00	Yes
NCBI Assembly	GCA_000373905.1	NCBI: cellular organisms; Bacteria; Terrabacteria group; Actinobacteria; Actinomycetia; Corynebacteriales; Mycobacteriaceae; Mycobacterium; unclassified Mycobacterium;	NCBI: 1157943	4609894	100.00	0.00	Yes
NCBI Assembly	GCA_000649795.1	NCBI: cellular organisms; Bacteria; Terrabacteria group; Actinobacteria; Actinomycetia; Corynebacteriales; Mycobacteriaceae; Mycobacterium; Mycobacterium tuberculosis complex; Mycobacterium tuberculosis;	NCBI: 1447434	4428622	99.94	0.00	Yes
NCBI Assembly	GCA_000658395.1	NCBI: cellular organisms; Bacteria; Terrabacteria group; Actinobacteria; Actinomycetia; Corynebacteriales; Mycobacteriaceae; Mycobacterium; Mycobacterium tuberculosis complex; Mycobacterium tuberculosis;	NCBI: 1448777	4431977	99.94	0.00	Yes
NCBI Assembly	GCA_000655755.1	NCBI: cellular organisms; Bacteria; Terrabacteria group; Actinobacteria; Actinomycetia; Corynebacteriales; Mycobacteriaceae; Mycobacterium; Mycobacterium avium complex (MAC); Mycobacterium avium;	NCBI: 1427296	5130253	99.94	0.00	Yes
NCBI Assembly	GCA_000651055.1	NCBI: cellular organisms; Bacteria; Terrabacteria group; Actinobacteria; Actinomycetia; Corynebacteriales; Mycobacteriaceae; Mycobacterium; Mycobacterium tuberculosis complex; Mycobacterium tuberculosis;	NCBI: 1447513	4409145	99.86	0.33	Yes
NCBI Assembly	GCA_000652295.1	NCBI: cellular organisms; Bacteria; Terrabacteria group; Actinobacteria; Actinomycetia; Corynebacteriales; Mycobacteriaceae; Mycobacterium; Mycobacterium tuberculosis complex; Mycobacterium tuberculosis;	NCBI: 1448456	4413559	99.94	0.00	Yes

NCBI Assembly	GCA_000271225.1	NCBI: cellular organisms; Bacteria; Terrabacteria group; Actinobacteria; Actinomycetia; Corynebacteriales; Mycobacteriaceae; Mycobacteroides; Mycobacteroides abscessus;	NCBI: 1001729	5242408	100.00	0.00	Yes
NCBI Assembly	GCA_000660325.1	NCBI: cellular organisms; Bacteria; Terrabacteria group; Actinobacteria; Actinomycetia; Corynebacteriales; Mycobacteriaceae; Mycobacterium; Mycobacterium tuberculosis complex; Mycobacterium tuberculosis;	NCBI: 1324222	4397178	99.94	0.00	Yes
NCBI Assembly	GCA_000504825.1	NCBI: cellular organisms; Bacteria; Terrabacteria group; Actinobacteria; Actinomycetia; Corynebacteriales; Mycobacteriaceae; Mycobacterium; Mycobacterium avium complex (MAC); Mycobacterium avium; Mycobacterium avium subsp. hominissuis;	NCBI: 1381559	5529274	92.33	5.60	No
NCBI Assembly	GCA_000516415.1	NCBI: cellular organisms; Bacteria; Terrabacteria group; Actinobacteria; Actinomycetia; Corynebacteriales; Mycobacteriaceae; Mycobacterium; Mycobacterium tuberculosis complex; Mycobacterium tuberculosis;	NCBI: 1160716	4310512	99.94	0.00	Yes
NCBI Assembly	GCA_000652655.1	NCBI: cellular organisms; Bacteria; Terrabacteria group; Actinobacteria; Actinomycetia; Corynebacteriales; Mycobacteriaceae; Mycobacterium; Mycobacterium tuberculosis complex; Mycobacterium tuberculosis;	NCBI: 1448476	4407984	99.94	0.00	Yes
NCBI Assembly	GCA_000650135.1	NCBI: cellular organisms; Bacteria; Terrabacteria group; Actinobacteria; Actinomycetia; Corynebacteriales; Mycobacteriaceae; Mycobacterium; Mycobacterium tuberculosis complex; Mycobacterium tuberculosis;	NCBI: 1447457	4388768	99.94	0.00	Yes
NCBI Assembly	GCA_000649815.1	NCBI: cellular organisms; Bacteria; Terrabacteria group; Actinobacteria; Actinomycetia; Corynebacteriales; Mycobacteriaceae; Mycobacterium; Mycobacterium tuberculosis complex; Mycobacterium tuberculosis;	NCBI: 1447437	4385091	99.94	0.00	Yes
NCBI Assembly	GCA_000655775.1	NCBI: cellular organisms; Bacteria; Terrabacteria group; Actinobacteria; Actinomycetia; Corynebacteriales; Mycobacteriaceae; Mycobacterium; Mycobacterium tuberculosis complex; Mycobacterium tuberculosis;	NCBI: 1427297	4390354	99.94	0.00	Yes
NCBI Assembly	GCA_000658415.1	NCBI: cellular organisms; Bacteria; Terrabacteria group; Actinobacteria; Actinomycetia; Corynebacteriales; Mycobacteriaceae; Mycobacterium; Mycobacterium tuberculosis complex; Mycobacterium tuberculosis;	NCBI: 1423429	4395536	99.94	0.00	Yes
NCBI Assembly	GCA_000379865.1	NCBI: cellular organisms; Bacteria; Terrabacteria group; Actinobacteria; Actinomycetia; Corynebacteriales; Mycobacteriaceae; Mycolicibacterium; Mycolicibacterium hassiacum;	NCBI: 1122247	5082325	99.77	0.15	Yes
NCBI Assembly	GCA_000651075.1	NCBI: cellular organisms; Bacteria; Terrabacteria group; Actinobacteria; Actinomycetia; Corynebacteriales; Mycobacteriaceae; Mycobacterium; Mycobacterium tuberculosis complex; Mycobacterium tuberculosis;	NCBI: 1447514	4395159	99.94	0.00	Yes
NCBI Assembly	GCA_000652315.1	NCBI: cellular organisms; Bacteria; Terrabacteria group; Actinobacteria; Actinomycetia; Corynebacteriales; Mycobacteriaceae; Mycobacterium; Mycobacterium tuberculosis complex; Mycobacterium tuberculosis;	NCBI: 1448458	4408712	99.94	0.00	Yes
NCBI Assembly	GCA_000271245.1	NCBI: cellular organisms; Bacteria; Terrabacteria group; Actinobacteria; Actinomycetia; Corynebacteriales; Mycobacteriaceae; Mycobacteroides; Mycobacteroides abscessus;	NCBI: 1001726	4842208	100.00	0.00	Yes
NCBI Assembly	GCA_000660345.1	NCBI: cellular organisms; Bacteria; Terrabacteria group; Actinobacteria; Actinomycetia; Corynebacteriales; Mycobacteriaceae; Mycobacterium; Mycobacterium tuberculosis complex; Mycobacterium tuberculosis;	NCBI: 1324223	4404966	99.94	1.00	Yes
NCBI Assembly	GCA_000504845.1	NCBI: cellular organisms; Bacteria; Terrabacteria group; Actinobacteria; Actinomycetia; Corynebacteriales; Mycobacteriaceae; Mycobacterium; Mycobacterium avium complex (MAC); Mycobacterium avium; Mycobacterium avium subsp. paratuberculosis;	NCBI: 1391996	4814969	95.11	1.47	Yes
NCBI Assembly	GCA_000650155.1	NCBI: cellular organisms; Bacteria; Terrabacteria group; Actinobacteria; Actinomycetia; Corynebacteriales; Mycobacteriaceae; Mycobacterium; Mycobacterium tuberculosis complex; Mycobacterium tuberculosis;	NCBI: 1447458	4385827	99.94	0.00	Yes
NCBI Assembly	GCA_000652675.1	NCBI: cellular organisms; Bacteria; Terrabacteria group; Actinobacteria; Actinomycetia; Corynebacteriales; Mycobacteriaceae; Mycobacterium; Mycobacterium tuberculosis complex; Mycobacterium tuberculosis;	NCBI: 1448477	4433979	99.94	0.00	Yes
NCBI Assembly	GCA_000516435.1	NCBI: cellular organisms; Bacteria; Terrabacteria group; Actinobacteria; Actinomycetia; Corynebacteriales; Mycobacteriaceae; Mycobacterium; Mycobacterium tuberculosis complex; Mycobacterium tuberculosis;	NCBI: 1160714	4304203	99.61	0.11	Yes

NCBI Assembly	GCA_000649835.1	NCBI: cellular organisms; Bacteria; Terrabacteria group; Actinobacteria; Actinomycetia; Corynebacteriales; Mycobacteriaceae; Mycobacterium; Mycobacterium tuberculosis complex; Mycobacterium tuberculosis;	NCBI: 1447438	4421105	99.94	0.00	Yes
NCBI Assembly	GCA_000655795.1	NCBI: cellular organisms; Bacteria; Terrabacteria group; Actinobacteria; Actinomycetia; Corynebacteriales; Mycobacteriaceae; Mycobacterium; Mycobacterium tuberculosis complex; Mycobacterium tuberculosis;	NCBI: 1427298	4400480	99.94	0.00	Yes
NCBI Assembly	GCA_000658435.1	NCBI: cellular organisms; Bacteria; Terrabacteria group; Actinobacteria; Actinomycetia; Corynebacteriales; Mycobacteriaceae; Mycobacterium; Mycobacterium tuberculosis complex; Mycobacterium tuberculosis;	NCBI: 1438866	4462200	99.94	1.89	Yes
NCBI Assembly	GCA_000382405.1	NCBI: cellular organisms; Bacteria; Terrabacteria group; Actinobacteria; Actinomycetia; Corynebacteriales; Mycobacteriaceae; Mycobacterium; unclassified Mycobacterium;	NCBI: 1120797	4535740	100.00	0.09	Yes
NCBI Assembly	GCA_000651095.1	NCBI: cellular organisms; Bacteria; Terrabacteria group; Actinobacteria; Actinomycetia; Corynebacteriales; Mycobacteriaceae; Mycobacterium; Mycobacterium tuberculosis complex; Mycobacterium tuberculosis;	NCBI: 1447515	4425297	99.94	0.00	Yes
NCBI Assembly	GCA_000652335.1	NCBI: cellular organisms; Bacteria; Terrabacteria group; Actinobacteria; Actinomycetia; Corynebacteriales; Mycobacteriaceae; Mycobacterium; Mycobacterium tuberculosis complex; Mycobacterium tuberculosis;	NCBI: 1448459	4409576	99.94	0.00	Yes
NCBI Assembly	GCA_000660365.1	NCBI: cellular organisms; Bacteria; Terrabacteria group; Actinobacteria; Actinomycetia; Corynebacteriales; Mycobacteriaceae; Mycobacterium; Mycobacterium tuberculosis complex; Mycobacterium tuberculosis;	NCBI: 1324224	4427095	99.94	0.00	Yes
NCBI Assembly	GCA_000271265.1	NCBI: cellular organisms; Bacteria; Terrabacteria group; Actinobacteria; Actinomycetia; Corynebacteriales; Mycobacteriaceae; Mycobacteroides; Mycobacteroides abscessus;	NCBI: 1001727	4862293	100.00	0.00	Yes
NCBI Assembly	GCA_000504865.1	NCBI: cellular organisms; Bacteria; Terrabacteria group; Actinobacteria; Actinomycetia; Corynebacteriales; Mycobacteriaceae; Mycobacterium; Mycobacterium avium complex (MAC); Mycobacterium avium; Mycobacterium avium subsp. paratuberculosis;	NCBI: 1391995	4631752	93.87	2.20	Yes
NCBI Assembly	GCA_000650175.1	NCBI: cellular organisms; Bacteria; Terrabacteria group; Actinobacteria; Actinomycetia; Corynebacteriales; Mycobacteriaceae; Mycobacterium; Mycobacterium tuberculosis complex; Mycobacterium tuberculosis;	NCBI: 1447459	4410304	99.94	0.00	Yes
NCBI Assembly	GCA_000649855.1	NCBI: cellular organisms; Bacteria; Terrabacteria group; Actinobacteria; Actinomycetia; Corynebacteriales; Mycobacteriaceae; Mycobacterium; Mycobacterium tuberculosis complex; Mycobacterium tuberculosis;	NCBI: 1447439	4377772	99.94	0.00	Yes
NCBI Assembly	GCA_000652695.1	NCBI: cellular organisms; Bacteria; Terrabacteria group; Actinobacteria; Actinomycetia; Corynebacteriales; Mycobacteriaceae; Mycobacterium; Mycobacterium tuberculosis complex; Mycobacterium tuberculosis;	NCBI: 1448478	4403440	99.94	0.00	Yes
NCBI Assembly	GCA_000658455.1	NCBI: cellular organisms; Bacteria; Terrabacteria group; Actinobacteria; Actinomycetia; Corynebacteriales; Mycobacteriaceae; Mycobacterium; Mycobacterium tuberculosis complex; Mycobacterium tuberculosis;	NCBI: 1438864	4466138	99.94	2.11	Yes
NCBI Assembly	GCA_000655815.1	NCBI: cellular organisms; Bacteria; Terrabacteria group; Actinobacteria; Actinomycetia; Corynebacteriales; Mycobacteriaceae; Mycobacterium; Mycobacterium tuberculosis complex; Mycobacterium tuberculosis;	NCBI: 1427299	4432399	99.94	0.00	Yes
NCBI Assembly	GCA_000516455.1	NCBI: cellular organisms; Bacteria; Terrabacteria group; Actinobacteria; Actinomycetia; Corynebacteriales; Mycobacteriaceae; Mycobacterium; Mycobacterium tuberculosis complex; Mycobacterium tuberculosis;	NCBI: 1160715	4310154	99.88	0.50	Yes
NCBI Assembly	GCA_000383495.1	NCBI: cellular organisms; Bacteria; Terrabacteria group; Actinobacteria; Actinomycetia; Corynebacteriales; Mycobacteriaceae; Mycobacterium; unclassified Mycobacterium;	NCBI: 1172186	6057078	99.81	0.42	Yes
NCBI Assembly	GCA_000651115.1	NCBI: cellular organisms; Bacteria; Terrabacteria group; Actinobacteria; Actinomycetia; Corynebacteriales; Mycobacteriaceae; Mycobacterium; Mycobacterium tuberculosis complex; Mycobacterium tuberculosis;	NCBI: 1447516	4421396	99.94	0.00	Yes
NCBI Assembly	GCA_000652355.1	NCBI: cellular organisms; Bacteria; Terrabacteria group; Actinobacteria; Actinomycetia; Corynebacteriales; Mycobacteriaceae; Mycobacterium; Mycobacterium tuberculosis complex; Mycobacterium tuberculosis;	NCBI: 1448460	4405704	99.94	0.00	Yes

NCBI Assembly	GCA_000504885.1	NCBI: cellular organisms; Bacteria; Terrabacteria group; Actinobacteria; Actinomycetia; Corynebacteriales; Mycobacteriaceae; Mycobacterium; Mycobacterium avium complex (MAC); Mycobacterium avium; Mycobacterium avium subsp. paratuberculosis;	NCBI: 1391994	4516892	89.74	2.95	No
NCBI Assembly	GCA_000660385.1	NCBI: cellular organisms; Bacteria; Terrabacteria group; Actinobacteria; Actinomycetia; Corynebacteriales; Mycobacteriaceae; Mycobacterium; Mycobacterium tuberculosis complex; Mycobacterium tuberculosis;	NCBI: 1324225	4427858	99.94	0.00	Yes
NCBI Assembly	GCA_000271285.1	NCBI: cellular organisms; Bacteria; Terrabacteria group; Actinobacteria; Actinomycetia; Corynebacteriales; Mycobacteriaceae; Mycobacteroides; Mycobacteroides abscessus; Mycobacteroides abscessus subsp. bolletii;	NCBI: 1001705	4897666	100.00	0.00	Yes
NCBI Assembly	GCA_000649875.1	NCBI: cellular organisms; Bacteria; Terrabacteria group; Actinobacteria; Actinomycetia; Corynebacteriales; Mycobacteriaceae; Mycobacterium; Mycobacterium tuberculosis complex; Mycobacterium tuberculosis;	NCBI: 1447440	4374604	99.94	0.00	Yes
NCBI Assembly	GCA_000650195.1	NCBI: cellular organisms; Bacteria; Terrabacteria group; Actinobacteria; Actinomycetia; Corynebacteriales; Mycobacteriaceae; Mycobacterium; Mycobacterium tuberculosis complex; Mycobacterium tuberculosis;	NCBI: 1447460	4429077	99.94	0.58	Yes
NCBI Assembly	GCA_000658475.1	NCBI: cellular organisms; Bacteria; Terrabacteria group; Actinobacteria; Actinomycetia; Corynebacteriales; Mycobacteriaceae; Mycobacterium; Mycobacterium tuberculosis complex; Mycobacterium tuberculosis;	NCBI: 1438863	4439024	99.78	1.83	Yes
NCBI Assembly	GCA_000652715.1	NCBI: cellular organisms; Bacteria; Terrabacteria group; Actinobacteria; Actinomycetia; Corynebacteriales; Mycobacteriaceae; Mycobacterium; Mycobacterium tuberculosis complex; Mycobacterium tuberculosis;	NCBI: 1448479	4411291	99.94	0.00	Yes
NCBI Assembly	GCA_000655835.1	NCBI: cellular organisms; Bacteria; Terrabacteria group; Actinobacteria; Actinomycetia; Corynebacteriales; Mycobacteriaceae; Mycobacterium; Mycobacterium tuberculosis complex; Mycobacterium tuberculosis;	NCBI: 1427300	4424906	99.94	0.00	Yes
NCBI Assembly	GCA_000280595.1	NCBI: cellular organisms; Bacteria; Terrabacteria group; Actinobacteria; Actinomycetia; Corynebacteriales; Mycobacteriaceae; Mycobacteroides; Mycobacteroides abscessus;	NCBI: 1175632	5046600	99.67	0.00	Yes
NCBI Assembly	GCA_000651135.1	NCBI: cellular organisms; Bacteria; Terrabacteria group; Actinobacteria; Actinomycetia; Corynebacteriales; Mycobacteriaceae; Mycobacterium; Mycobacterium tuberculosis complex; Mycobacterium tuberculosis;	NCBI: 1447517	4382272	99.94	0.00	Yes
NCBI Assembly	GCA_000652375.1	NCBI: cellular organisms; Bacteria; Terrabacteria group; Actinobacteria; Actinomycetia; Corynebacteriales; Mycobacteriaceae; Mycobacterium; Mycobacterium tuberculosis complex; Mycobacterium tuberculosis;	NCBI: 1448461	4407860	99.94	0.00	Yes
NCBI Assembly	GCA_000504905.1	NCBI: cellular organisms; Bacteria; Terrabacteria group; Actinobacteria; Actinomycetia; Corynebacteriales; Mycobacteriaceae; Mycobacterium; Mycobacterium avium complex (MAC); Mycobacterium avium;	NCBI: 1392003	4680797	88.64	2.58	No
NCBI Assembly	GCA_000660405.1	NCBI: cellular organisms; Bacteria; Terrabacteria group; Actinobacteria; Actinomycetia; Corynebacteriales; Mycobacteriaceae; Mycobacterium; Mycobacterium tuberculosis complex; Mycobacterium tuberculosis;	NCBI: 1324226	4423782	99.94	0.00	Yes
NCBI Assembly	GCA_000277085.1	NCBI: cellular organisms; Bacteria; Terrabacteria group; Actinobacteria; Actinomycetia; Corynebacteriales; Mycobacteriaceae; Mycobacterium; Mycobacterium tuberculosis complex; Mycobacterium tuberculosis;	NCBI: 1091500	4380119	96.04	0.33	Yes
NCBI Assembly	GCA_000650215.1	NCBI: cellular organisms; Bacteria; Terrabacteria group; Actinobacteria; Actinomycetia; Corynebacteriales; Mycobacteriaceae; Mycobacterium; Mycobacterium tuberculosis complex; Mycobacterium tuberculosis;	NCBI: 1447461	4390434	99.94	0.00	Yes
NCBI Assembly	GCA_000658495.1	NCBI: cellular organisms; Bacteria; Terrabacteria group; Actinobacteria; Actinomycetia; Corynebacteriales; Mycobacteriaceae; Mycobacterium; Mycobacterium tuberculosis complex; Mycobacterium tuberculosis;	NCBI: 1438853	4210868	96.61	1.25	Yes
NCBI Assembly	GCA_000649895.1	NCBI: cellular organisms; Bacteria; Terrabacteria group; Actinobacteria; Actinomycetia; Corynebacteriales; Mycobacteriaceae; Mycobacterium; Mycobacterium tuberculosis complex; Mycobacterium tuberculosis;	NCBI: 1447441	4427007	99.94	0.00	Yes
NCBI Assembly	GCA_000652735.1	NCBI: cellular organisms; Bacteria; Terrabacteria group; Actinobacteria; Actinomycetia; Corynebacteriales; Mycobacteriaceae; Mycobacterium; Mycobacterium tuberculosis complex; Mycobacterium tuberculosis;	NCBI: 1448480	4409099	99.94	0.00	Yes

NCBI Assembly	GCA_000280615.1	NCBI: cellular organisms; Bacteria; Terrabacteria group; Actinobacteria; Actinomycetia; Corynebacteriales; Mycobacteriaceae; Mycobacteroides; Mycobacteroides abscessus;	NCBI: 1176125	4911666	100.00	0.00	Yes
NCBI Assembly	GCA_000655855.1	NCBI: cellular organisms; Bacteria; Terrabacteria group; Actinobacteria; Actinomycetia; Corynebacteriales; Mycobacteriaceae; Mycobacterium; Mycobacterium tuberculosis complex; Mycobacterium tuberculosis;	NCBI: 1427301	4391691	99.94	0.00	Yes
NCBI Assembly	GCA_000521845.1	NCBI: cellular organisms; Bacteria; Terrabacteria group; Actinobacteria; Actinomycetia; Corynebacteriales; Mycobacteriaceae; Mycolicibacterium; Mycolicibacterium smegmatis;	NCBI: 1445611	6823739	99.94	0.38	Yes
NCBI Assembly	GCA_000651155.1	NCBI: cellular organisms; Bacteria; Terrabacteria group; Actinobacteria; Actinomycetia; Corynebacteriales; Mycobacteriaceae; Mycobacterium; Mycobacterium tuberculosis complex; Mycobacterium tuberculosis;	NCBI: 1447519	4395036	99.94	0.11	Yes
NCBI Assembly	GCA_000652395.1	NCBI: cellular organisms; Bacteria; Terrabacteria group; Actinobacteria; Actinomycetia; Corynebacteriales; Mycobacteriaceae; Mycobacterium; Mycobacterium tuberculosis complex; Mycobacterium tuberculosis;	NCBI: 1448462	4408493	99.94	0.00	Yes
NCBI Assembly	GCA_000504925.1	NCBI: cellular organisms; Bacteria; Terrabacteria group; Actinobacteria; Actinomycetia; Corynebacteriales; Mycobacteriaceae; Mycobacterium; Mycobacterium avium complex (MAC); Mycobacterium avium;	NCBI: 1392001	5167261	91.93	1.90	Yes
NCBI Assembly	GCA_000277105.1	NCBI: cellular organisms; Bacteria; Terrabacteria group; Actinobacteria; Actinomycetia; Corynebacteriales; Mycobacteriaceae; Mycobacterium; Mycobacterium tuberculosis complex; Mycobacterium tuberculosis;	NCBI: 1091501	4406587	95.50	0.00	Yes
NCBI Assembly	GCA_000660425.1	NCBI: cellular organisms; Bacteria; Terrabacteria group; Actinobacteria; Actinomycetia; Corynebacteriales; Mycobacteriaceae; Mycobacterium; Mycobacterium tuberculosis complex; Mycobacterium tuberculosis;	NCBI: 1324227	4375389	99.94	0.00	Yes
NCBI Assembly	GCA_000650235.1	NCBI: cellular organisms; Bacteria; Terrabacteria group; Actinobacteria; Actinomycetia; Corynebacteriales; Mycobacteriaceae; Mycobacterium; Mycobacterium tuberculosis complex; Mycobacterium tuberculosis;	NCBI: 1447462	4423430	99.94	0.46	Yes
NCBI Assembly	GCA_000658515.1	NCBI: cellular organisms; Bacteria; Terrabacteria group; Actinobacteria; Actinomycetia; Corynebacteriales; Mycobacteriaceae; Mycobacterium; Mycobacterium tuberculosis complex; Mycobacterium tuberculosis;	NCBI: 1438856	4409324	99.94	0.08	Yes
NCBI Assembly	GCA_000649915.1	NCBI: cellular organisms; Bacteria; Terrabacteria group; Actinobacteria; Actinomycetia; Corynebacteriales; Mycobacteriaceae; Mycobacterium; Mycobacterium tuberculosis complex; Mycobacterium tuberculosis;	NCBI: 1447442	4391296	99.94	0.00	Yes
NCBI Assembly	GCA_000184005.1	NCBI: cellular organisms; Bacteria; Terrabacteria group; Actinobacteria; Actinomycetia; Corynebacteriales; Mycobacteriaceae; Mycobacterium; Mycobacterium tuberculosis complex; Mycobacterium tuberculosis;	NCBI: 747367	4404672	99.61	0.00	Yes
NCBI Assembly	GCA_000280635.2	NCBI: cellular organisms; Bacteria; Terrabacteria group; Actinobacteria; Actinomycetia; Corynebacteriales; Mycobacteriaceae; Mycobacteroides; Mycobacteroides abscessus;	NCBI: 1185414	5016396	99.85	0.00	Yes
NCBI Assembly	GCA_000655255.1	NCBI: cellular organisms; Bacteria; Terrabacteria group; Actinobacteria; Actinomycetia; Corynebacteriales; Mycobacteriaceae; Mycobacterium; Mycobacterium tuberculosis complex; Mycobacterium tuberculosis;	NCBI: 1427269	4418859	99.94	0.00	Yes
NCBI Assembly	GCA_000523575.1	NCBI: cellular organisms; Bacteria; Terrabacteria group; Actinobacteria; Actinomycetia; Corynebacteriales; Mycobacteriaceae; Mycobacteroides; Mycobacteroides abscessus;	NCBI: 1335410	4761644	97.36	0.33	Yes
NCBI Assembly	GCA_000651175.1	NCBI: cellular organisms; Bacteria; Terrabacteria group; Actinobacteria; Actinomycetia; Corynebacteriales; Mycobacteriaceae; Mycobacterium; Mycobacterium tuberculosis complex; Mycobacterium tuberculosis;	NCBI: 1447520	4430993	99.94	0.00	Yes
NCBI Assembly	GCA_000652415.1	NCBI: cellular organisms; Bacteria; Terrabacteria group; Actinobacteria; Actinomycetia; Corynebacteriales; Mycobacteriaceae; Mycobacterium; Mycobacterium tuberculosis complex; Mycobacterium tuberculosis;	NCBI: 1448463	4409805	99.94	0.00	Yes
NCBI Assembly	GCA_000162995.1	NCBI: cellular organisms; Bacteria; Terrabacteria group; Actinobacteria; Actinomycetia; Corynebacteriales; Mycobacteriaceae; Mycobacterium; Mycobacterium tuberculosis complex; Mycobacterium tuberculosis;	NCBI: 663887	4397242	99.94	0.00	Yes

NCBI Assembly	GCA_000270705.1	NCBI: cellular organisms; Bacteria; Terrabacteria group; Actinobacteria; Actinomycetia; Corynebacteriales; Mycobacteriaceae; Mycobacteroides; Mycobacteroides abscessus;	NCBI: 1001718	5386957	100.00	0.67	Yes
NCBI Assembly	GCA_000660445.1	NCBI: cellular organisms; Bacteria; Terrabacteria group; Actinobacteria; Actinomycetia; Corynebacteriales; Mycobacteriaceae; Mycobacterium; Mycobacterium tuberculosis complex; Mycobacterium tuberculosis;	NCBI: 1324228	4407538	99.94	0.00	Yes
NCBI Assembly	GCA_000152105.1	NCBI: cellular organisms; Bacteria; Terrabacteria group; Actinobacteria; Actinomycetia; Corynebacteriales; Mycobacteriaceae; Mycobacterium; Mycobacterium tuberculosis complex; Mycobacterium tuberculosis;	NCBI: 675512	4383267	99.53	0.00	Yes
NCBI Assembly	GCA_000184025.1	NCBI: cellular organisms; Bacteria; Terrabacteria group; Actinobacteria; Actinomycetia; Corynebacteriales; Mycobacteriaceae; Mycobacterium; Mycobacterium tuberculosis complex; Mycobacterium tuberculosis;	NCBI: 747368	4404714	99.94	0.00	Yes
NCBI Assembly	GCA_000218115.2	NCBI: cellular organisms; Bacteria; Terrabacteria group; Actinobacteria; Actinomycetia; Corynebacteriales; Mycobacteriaceae; Mycobacterium; Mycobacterium avium complex (MAC); Mycobacterium avium; Mycobacterium avium subsp. paratuberculosis;	NCBI: 1029819	4500072	92.05	2.22	Yes
NCBI Assembly	GCA_000280655.1	NCBI: cellular organisms; Bacteria; Terrabacteria group; Actinobacteria; Actinomycetia; Corynebacteriales; Mycobacteriaceae; Mycobacteroides; Mycobacteroides abscessus;	NCBI: 1175633	5138021	97.77	0.16	Yes
NCBI Assembly	GCA_000657895.1	NCBI: cellular organisms; Bacteria; Terrabacteria group; Actinobacteria; Actinomycetia; Corynebacteriales; Mycobacteriaceae; Mycobacterium; Mycobacterium tuberculosis complex; Mycobacterium tuberculosis;	NCBI: 1408927	4408967	99.94	0.00	Yes
NCBI Assembly	GCA_000655275.1	NCBI: cellular organisms; Bacteria; Terrabacteria group; Actinobacteria; Actinomycetia; Corynebacteriales; Mycobacteriaceae; Mycobacterium; Mycobacterium tuberculosis complex; Mycobacterium tuberculosis;	NCBI: 1427270	4399709	99.94	0.00	Yes
NCBI Assembly	GCA_000650555.1	NCBI: cellular organisms; Bacteria; Terrabacteria group; Actinobacteria; Actinomycetia; Corynebacteriales; Mycobacteriaceae; Mycobacterium; Mycobacterium tuberculosis complex; Mycobacterium tuberculosis;	NCBI: 1447483	4381823	99.19	0.25	Yes
NCBI Assembly	GCA_000651195.1	NCBI: cellular organisms; Bacteria; Terrabacteria group; Actinobacteria; Actinomycetia; Corynebacteriales; Mycobacteriaceae; Mycobacterium; Mycobacterium tuberculosis complex; Mycobacterium tuberculosis;	NCBI: 1447521	4396450	99.83	0.00	Yes
NCBI Assembly	GCA_000650255.1	NCBI: cellular organisms; Bacteria; Terrabacteria group; Actinobacteria; Actinomycetia; Corynebacteriales; Mycobacteriaceae; Mycobacterium; Mycobacterium tuberculosis complex; Mycobacterium tuberculosis;	NCBI: 1447463	4401830	99.94	0.00	Yes
NCBI Assembly	GCA_000163015.1	NCBI: cellular organisms; Bacteria; Terrabacteria group; Actinobacteria; Actinomycetia; Corynebacteriales; Mycobacteriaceae; Mycobacterium; Mycobacterium tuberculosis complex; Mycobacterium tuberculosis;	NCBI: 663886	4397243	99.94	0.00	Yes
NCBI Assembly	GCA_000270725.1	NCBI: cellular organisms; Bacteria; Terrabacteria group; Actinobacteria; Actinomycetia; Corynebacteriales; Mycobacteriaceae; Mycobacteroides; Mycobacteroides abscessus;	NCBI: 1001719	5249987	100.00	0.00	Yes
NCBI Assembly	GCA_000659825.1	NCBI: cellular organisms; Bacteria; Terrabacteria group; Actinobacteria; Actinomycetia; Corynebacteriales; Mycobacteriaceae; Mycobacterium; Mycobacterium tuberculosis complex; Mycobacterium tuberculosis;	NCBI: 1295775	4410103	99.94	0.00	Yes
NCBI Assembly	GCA_000152505.1	NCBI: cellular organisms; Bacteria; Terrabacteria group; Actinobacteria; Actinomycetia; Corynebacteriales; Mycobacteriaceae; Mycobacterium; Mycobacterium tuberculosis complex; Mycobacterium tuberculosis;	NCBI: 348776	4379118	95.88	0.08	Yes
NCBI Assembly	GCA_000184045.1	NCBI: cellular organisms; Bacteria; Terrabacteria group; Actinobacteria; Actinomycetia; Corynebacteriales; Mycobacteriaceae; Mycobacterium; Mycobacterium tuberculosis complex; Mycobacterium tuberculosis;	NCBI: 747369	4397271	99.94	0.00	Yes
NCBI Assembly	GCA_000218135.2	NCBI: cellular organisms; Bacteria; Terrabacteria group; Actinobacteria; Actinomycetia; Corynebacteriales; Mycobacteriaceae; Mycobacterium; Mycobacterium avium complex (MAC); Mycobacterium avium; Mycobacterium avium subsp. paratuberculosis;	NCBI: 1029820	4560778	94.48	1.95	Yes
NCBI Assembly	GCA_000657915.1	NCBI: cellular organisms; Bacteria; Terrabacteria group; Actinobacteria; Actinomycetia; Corynebacteriales; Mycobacteriaceae; Mycobacterium; Mycobacterium tuberculosis complex; Mycobacterium tuberculosis;	NCBI: 1408943	4410419	99.94	0.00	Yes

NCBI Assembly	GCA_000291765.1	NCBI: cellular organisms; Bacteria; Terrabacteria group; Actinobacteria; Actinomycetia; Corynebacteriales; Mycobacteriaceae; Mycobacterium; Mycobacterium tuberculosis complex; Mycobacterium tuberculosis;	NCBI: 1078763	4518959	98.97	0.00	Yes
NCBI Assembly	GCA_000655295.1	NCBI: cellular organisms; Bacteria; Terrabacteria group; Actinobacteria; Actinomycetia; Corynebacteriales; Mycobacteriaceae; Mycobacterium; Mycobacterium tuberculosis complex; Mycobacterium tuberculosis;	NCBI: 1427271	4445880	99.94	0.00	Yes
NCBI Assembly	GCA_000650575.1	NCBI: cellular organisms; Bacteria; Terrabacteria group; Actinobacteria; Actinomycetia; Corynebacteriales; Mycobacteriaceae; Mycobacterium; Mycobacterium tuberculosis complex; Mycobacterium tuberculosis;	NCBI: 1447484	4422030	99.94	0.00	Yes
NCBI Assembly	GCA_000651215.1	NCBI: cellular organisms; Bacteria; Terrabacteria group; Actinobacteria; Actinomycetia; Corynebacteriales; Mycobacteriaceae; Mycobacterium; Mycobacterium tuberculosis complex; Mycobacterium tuberculosis;	NCBI: 1447522	4407837	99.94	0.00	Yes
NCBI Assembly	GCA_000650275.1	NCBI: cellular organisms; Bacteria; Terrabacteria group; Actinobacteria; Actinomycetia; Corynebacteriales; Mycobacteriaceae; Mycobacterium; Mycobacterium tuberculosis complex; Mycobacterium tuberculosis;	NCBI: 1447464	4328633	99.94	0.00	Yes
NCBI Assembly	GCA_000164135.1	NCBI: cellular organisms; Bacteria; Terrabacteria group; Actinobacteria; Actinomycetia; Corynebacteriales; Mycobacteriaceae; Mycobacterium; Mycobacterium simiae complex; Mycobacterium parascrofulaceum;	NCBI: 525368	6564171	98.06	1.12	Yes
NCBI Assembly	GCA_000270745.2	NCBI: cellular organisms; Bacteria; Terrabacteria group; Actinobacteria; Actinomycetia; Corynebacteriales; Mycobacteriaceae; Mycobacteroides; Mycobacteroides abscessus;	NCBI: 1001723	4849390	100.00	0.00	Yes
NCBI Assembly	GCA_000190335.1	NCBI: cellular organisms; Bacteria; Terrabacteria group; Actinobacteria; Actinomycetia; Corynebacteriales; Mycobacteriaceae; Mycobacterium; Mycobacterium tuberculosis complex; Mycobacterium tuberculosis;	NCBI: 911237	4380214	99.78	0.00	Yes
NCBI Assembly	GCA_000659845.1	NCBI: cellular organisms; Bacteria; Terrabacteria group; Actinobacteria; Actinomycetia; Corynebacteriales; Mycobacteriaceae; Mycobacterium; Mycobacterium tuberculosis complex; Mycobacterium tuberculosis;	NCBI: 1295776	4405933	99.94	0.00	Yes
NCBI Assembly	GCA_000218155.2	NCBI: cellular organisms; Bacteria; Terrabacteria group; Actinobacteria; Actinomycetia; Corynebacteriales; Mycobacteriaceae; Mycobacterium; Mycobacterium avium complex (MAC); Mycobacterium avium; Mycobacterium avium subsp. paratuberculosis;	NCBI: 1029821	4669710	97.40	0.64	Yes
NCBI Assembly	GCA_000153685.2	NCBI: cellular organisms; Bacteria; Terrabacteria group; Actinobacteria; Actinomycetia; Corynebacteriales; Mycobacteriaceae; Mycobacterium; Mycobacterium tuberculosis complex; Mycobacterium tuberculosis;	NCBI: 395095	4408224	99.94	0.00	Yes
NCBI Assembly	GCA_000657935.1	NCBI: cellular organisms; Bacteria; Terrabacteria group; Actinobacteria; Actinomycetia; Corynebacteriales; Mycobacteriaceae; Mycobacterium; Mycobacterium tuberculosis complex; Mycobacterium tuberculosis;	NCBI: 1417011	4388827	99.94	0.00	Yes
NCBI Assembly	GCA_000655315.1	NCBI: cellular organisms; Bacteria; Terrabacteria group; Actinobacteria; Actinomycetia; Corynebacteriales; Mycobacteriaceae; Mycobacterium; Mycobacterium tuberculosis complex; Mycobacterium tuberculosis;	NCBI: 1427272	4420399	99.94	0.00	Yes
NCBI Assembly	GCA_000295825.1	NCBI: cellular organisms; Bacteria; Terrabacteria group; Actinobacteria; Actinomycetia; Corynebacteriales; Mycobacteriaceae; Mycolicibacterium; Mycolicibacterium vaccae;	NCBI: 1194972	6245372	99.31	0.00	Yes
NCBI Assembly	GCA_000650295.1	NCBI: cellular organisms; Bacteria; Terrabacteria group; Actinobacteria; Actinomycetia; Corynebacteriales; Mycobacteriaceae; Mycobacterium; Mycobacterium tuberculosis complex; Mycobacterium tuberculosis;	NCBI: 1447465	4422323	99.94	0.00	Yes
NCBI Assembly	GCA_000651235.1	NCBI: cellular organisms; Bacteria; Terrabacteria group; Actinobacteria; Actinomycetia; Corynebacteriales; Mycobacteriaceae; Mycobacterium; Mycobacterium tuberculosis complex; Mycobacterium tuberculosis;	NCBI: 1447523	4422489	99.94	0.00	Yes
NCBI Assembly	GCA_000650595.1	NCBI: cellular organisms; Bacteria; Terrabacteria group; Actinobacteria; Actinomycetia; Corynebacteriales; Mycobacteriaceae; Mycobacterium; Mycobacterium tuberculosis complex; Mycobacterium tuberculosis;	NCBI: 1447485	4413443	99.94	0.00	Yes
NCBI Assembly	GCA_000165695.1	NCBI: cellular organisms; Bacteria; Terrabacteria group; Actinobacteria; Actinomycetia; Corynebacteriales; Mycobacteriaceae; Mycobacterium; Mycobacterium tuberculosis complex; Mycobacterium tuberculosis;	NCBI: 675513	4401234	98.38	0.00	Yes

NCBI Assembly	GCA_000270765.1	NCBI: cellular organisms; Bacteria; Terrabacteria group; Actinobacteria; Actinomycetia; Corynebacteriales; Mycobacteriaceae; Mycobacteroides; Mycobacteroides abscessus; Mycobacteroides abscessus subsp. bolletii;	NCBI: 1001713	4809972	100.00	0.00	Yes
NCBI Assembly	GCA_000193185.2	NCBI: cellular organisms; Bacteria; Terrabacteria group; Actinobacteria; Actinomycetia; Corynebacteriales; Mycobacteriaceae; Mycobacterium; Mycobacterium tuberculosis complex; Mycobacterium tuberculosis;	NCBI: 659019	4418548	99.94	0.00	Yes
NCBI Assembly	GCA_000155145.1	NCBI: cellular organisms; Bacteria; Terrabacteria group; Actinobacteria; Actinomycetia; Corynebacteriales; Mycobacteriaceae; Mycobacterium; Mycobacterium tuberculosis complex; Mycobacterium tuberculosis;	NCBI: 515615	4493323	97.75	0.61	Yes
NCBI Assembly	GCA_000659865.1	NCBI: cellular organisms; Bacteria; Terrabacteria group; Actinobacteria; Actinomycetia; Corynebacteriales; Mycobacteriaceae; Mycobacterium; Mycobacterium tuberculosis complex; Mycobacterium tuberculosis;	NCBI: 1295781	4398895	99.94	0.00	Yes
NCBI Assembly	GCA_000657955.1	NCBI: cellular organisms; Bacteria; Terrabacteria group; Actinobacteria; Actinomycetia; Corynebacteriales; Mycobacteriaceae; Mycobacterium; Mycobacterium tuberculosis complex; Mycobacterium tuberculosis;	NCBI: 1417019	4368078	99.94	0.00	Yes
NCBI Assembly	GCA_000655335.1	NCBI: cellular organisms; Bacteria; Terrabacteria group; Actinobacteria; Actinomycetia; Corynebacteriales; Mycobacteriaceae; Mycobacterium; Mycobacterium tuberculosis complex; Mycobacterium tuberculosis;	NCBI: 1427273	4424891	99.94	0.00	Yes
NCBI Assembly	GCA_000295855.1	NCBI: cellular organisms; Bacteria; Terrabacteria group; Actinobacteria; Actinomycetia; Corynebacteriales; Mycobacteriaceae; Mycolicibacterium; Mycolicibacterium fortuitum; Mycolicibacterium fortuitum subsp. fortuitum;	NCBI: 1214102	6349738	97.61	0.50	Yes
NCBI Assembly	GCA_000650615.1	NCBI: cellular organisms; Bacteria; Terrabacteria group; Actinobacteria; Actinomycetia; Corynebacteriales; Mycobacteriaceae; Mycobacterium; Mycobacterium tuberculosis complex; Mycobacterium tuberculosis;	NCBI: 1447486	4426067	99.94	0.00	Yes
NCBI Assembly	GCA_000651255.1	NCBI: cellular organisms; Bacteria; Terrabacteria group; Actinobacteria; Actinomycetia; Corynebacteriales; Mycobacteriaceae; Mycobacterium; Mycobacterium tuberculosis complex; Mycobacterium tuberculosis;	NCBI: 1447524	4426270	99.94	0.00	Yes
NCBI Assembly	GCA_000650315.1	NCBI: cellular organisms; Bacteria; Terrabacteria group; Actinobacteria; Actinomycetia; Corynebacteriales; Mycobacteriaceae; Mycobacterium; Mycobacterium tuberculosis complex; Mycobacterium tuberculosis;	NCBI: 1447466	4391531	99.94	0.71	Yes
NCBI Assembly	GCA_000174035.1	NCBI: cellular organisms; Bacteria; Terrabacteria group; Actinobacteria; Actinomycetia; Corynebacteriales; Mycobacteriaceae; Mycobacterium; Mycobacterium avium complex (MAC); Mycobacterium avium; Mycobacterium avium subsp. avium;	NCBI: 553481	4857995	97.99	0.26	Yes
NCBI Assembly	GCA_000270785.1	NCBI: cellular organisms; Bacteria; Terrabacteria group; Actinobacteria; Actinomycetia; Corynebacteriales; Mycobacteriaceae; Mycobacteroides; Mycobacteroides abscessus; Mycobacteroides abscessus subsp. bolletii;	NCBI: 1001712	4809311	100.00	0.00	Yes
NCBI Assembly	GCA_000194035.2	NCBI: cellular organisms; Bacteria; Terrabacteria group; Actinobacteria; Actinomycetia; Corynebacteriales; Mycobacteriaceae; Mycobacterium; Mycobacterium tuberculosis complex; Mycobacterium tuberculosis; Mycobacterium tuberculosis variant bovis; Mycobacterium tuberculosis variant bovis BCG;	NCBI: 998090	4255552	98.97	0.00	Yes
NCBI Assembly	GCA_000155165.1	NCBI: cellular organisms; Bacteria; Terrabacteria group; Actinobacteria; Actinomycetia; Corynebacteriales; Mycobacteriaceae; Mycobacterium; Mycobacterium tuberculosis complex; Mycobacterium tuberculosis;	NCBI: 515616	4569070	97.80	0.60	Yes
NCBI Assembly	GCA_000657975.1	NCBI: cellular organisms; Bacteria; Terrabacteria group; Actinobacteria; Actinomycetia; Corynebacteriales; Mycobacteriaceae; Mycobacterium; Mycobacterium tuberculosis complex; Mycobacterium tuberculosis;	NCBI: 1417020	4384919	99.61	0.00	Yes
NCBI Assembly	GCA_000296155.1	NCBI: cellular organisms; Bacteria; Terrabacteria group; Actinobacteria; Actinomycetia; Corynebacteriales; Mycobacteriaceae; Mycobacterium; Mycobacterium tuberculosis complex; Mycobacterium tuberculosis;	NCBI: 1200348	4389939	98.24	1.72	Yes
NCBI Assembly	GCA_000659885.1	NCBI: cellular organisms; Bacteria; Terrabacteria group; Actinobacteria; Actinomycetia; Corynebacteriales; Mycobacteriaceae; Mycobacterium; Mycobacterium tuberculosis complex; Mycobacterium tuberculosis;	NCBI: 1295782	4420543	99.94	0.00	Yes
NCBI Assembly	GCA_000655355.1	NCBI: cellular organisms; Bacteria; Terrabacteria group; Actinobacteria; Actinomycetia; Corynebacteriales; Mycobacteriaceae; Mycobacterium; Mycobacterium tuberculosis complex; Mycobacterium tuberculosis;	NCBI: 1427274	4415372	99.94	0.00	Yes

NCBI Assembly	GCA_000651275.1	NCBI: cellular organisms; Bacteria; Terrabacteria group; Actinobacteria; Actinomycetia; Corynebacteriales; Mycobacteriaceae; Mycobacterium; Mycobacterium tuberculosis complex; Mycobacterium tuberculosis;	NCBI: 1447525	4390837	99.94	0.00	Yes
NCBI Assembly	GCA_000650635.1	NCBI: cellular organisms; Bacteria; Terrabacteria group; Actinobacteria; Actinomycetia; Corynebacteriales; Mycobacteriaceae; Mycobacterium; Mycobacterium tuberculosis complex; Mycobacterium tuberculosis;	NCBI: 1447487	4429510	99.94	0.80	Yes
NCBI Assembly	GCA_000650335.1	NCBI: cellular organisms; Bacteria; Terrabacteria group; Actinobacteria; Actinomycetia; Corynebacteriales; Mycobacteriaceae; Mycobacterium; Mycobacterium tuberculosis complex; Mycobacterium tuberculosis;	NCBI: 1447467	4399538	99.94	0.00	Yes
NCBI Assembly	GCA_000176315.1	NCBI: cellular organisms; Bacteria; Terrabacteria group; Actinobacteria; Actinomycetia; Corynebacteriales; Mycobacteriaceae; Mycobacterium; Mycobacterium tuberculosis complex; Mycobacterium tuberculosis;	NCBI: 164513	4395332	98.93	0.00	Yes
NCBI Assembly	GCA_000194055.2	NCBI: cellular organisms; Bacteria; Terrabacteria group; Actinobacteria; Actinomycetia; Corynebacteriales; Mycobacteriaceae; Mycobacterium; Mycobacterium tuberculosis complex; Mycobacterium tuberculosis; Mycobacterium tuberculosis variant bovis; [illegible]	NCBI: 998091	4271122	99.75	0.00	Yes
NCBI Assembly	GCA_000270805.1	NCBI: cellular organisms; Bacteria; Terrabacteria group; Actinobacteria; Actinomycetia; Corynebacteriales; Mycobacteriaceae; Mycobacteroides; Mycobacteroides abscessus; Mycobacteroides abscessus subsp. bolletii;	NCBI: 1001711	4809994	100.00	0.00	Yes
NCBI Assembly	GCA_000155185.1	NCBI: cellular organisms; Bacteria; Terrabacteria group; Actinobacteria; Actinomycetia; Corynebacteriales; Mycobacteriaceae; Mycobacterium; Mycobacterium tuberculosis complex; Mycobacterium tuberculosis;	NCBI: 515617	4377487	88.36	0.61	No
NCBI Assembly	GCA_000296175.1	NCBI: cellular organisms; Bacteria; Terrabacteria group; Actinobacteria; Actinomycetia; Corynebacteriales; Mycobacteriaceae; Mycobacterium; Mycobacterium tuberculosis complex; Mycobacterium tuberculosis;	NCBI: 1200347	4383364	98.54	0.73	Yes
NCBI Assembly	GCA_000657995.1	NCBI: cellular organisms; Bacteria; Terrabacteria group; Actinobacteria; Actinomycetia; Corynebacteriales; Mycobacteriaceae; Mycobacterium; Mycobacterium tuberculosis complex; Mycobacterium tuberculosis;	NCBI: 1417026	4384430	99.94	0.00	Yes
NCBI Assembly	GCA_000659905.1	NCBI: cellular organisms; Bacteria; Terrabacteria group; Actinobacteria; Actinomycetia; Corynebacteriales; Mycobacteriaceae; Mycobacterium; Mycobacterium tuberculosis complex; Mycobacterium tuberculosis;	NCBI: 1295790	4412057	99.94	0.00	Yes
NCBI Assembly	GCA_000655375.1	NCBI: cellular organisms; Bacteria; Terrabacteria group; Actinobacteria; Actinomycetia; Corynebacteriales; Mycobacteriaceae; Mycobacterium; Mycobacterium tuberculosis complex; Mycobacterium tuberculosis;	NCBI: 1427275	4426308	99.94	0.00	Yes
NCBI Assembly	GCA_000651295.1	NCBI: cellular organisms; Bacteria; Terrabacteria group; Actinobacteria; Actinomycetia; Corynebacteriales; Mycobacteriaceae; Mycobacterium; Mycobacterium tuberculosis complex; Mycobacterium tuberculosis;	NCBI: 1447526	4429922	99.94	0.00	Yes
NCBI Assembly	GCA_000650355.1	NCBI: cellular organisms; Bacteria; Terrabacteria group; Actinobacteria; Actinomycetia; Corynebacteriales; Mycobacteriaceae; Mycobacterium; Mycobacterium tuberculosis complex; Mycobacterium tuberculosis;	NCBI: 1447468	4391138	99.94	0.00	Yes
NCBI Assembly	GCA_000650655.1	NCBI: cellular organisms; Bacteria; Terrabacteria group; Actinobacteria; Actinomycetia; Corynebacteriales; Mycobacteriaceae; Mycobacterium; Mycobacterium tuberculosis complex; Mycobacterium tuberculosis;	NCBI: 1447489	4425170	99.94	0.00	Yes
NCBI Assembly	GCA_000177795.2	NCBI: cellular organisms; Bacteria; Terrabacteria group; Actinobacteria; Actinomycetia; Corynebacteriales; Mycobacteriaceae; Mycobacterium; Mycobacterium tuberculosis complex; Mycobacterium tuberculosis;	NCBI: 675514	4377068	99.60	0.00	Yes
NCBI Assembly	GCA_000270825.1	NCBI: cellular organisms; Bacteria; Terrabacteria group; Actinobacteria; Actinomycetia; Corynebacteriales; Mycobacteriaceae; Mycobacteroides; Mycobacteroides abscessus;	NCBI: 1001721	5281259	100.00	0.00	Yes
NCBI Assembly	GCA_000155225.1	NCBI: cellular organisms; Bacteria; Terrabacteria group; Actinobacteria; Actinomycetia; Corynebacteriales; Mycobacteriaceae; Mycobacterium; Mycobacterium tuberculosis complex; Mycobacterium tuberculosis;	NCBI: 520140	4434168	98.47	0.00	Yes
NCBI Assembly	GCA_000308015.1	NCBI: cellular organisms; Bacteria; Terrabacteria group; Actinobacteria; Actinomycetia; Corynebacteriales; Mycobacteriaceae; Mycobacteroides; Mycobacteroides abscessus; Mycobacteroides abscessus subsp. bolletii;	NCBI: 1001707	4895947	100.00	0.00	Yes

NCBI Assembly	GCA_000658015.1	NCBI: cellular organisms; Bacteria; Terrabacteria group; Actinobacteria; Actinomycetia; Corynebacteriales; Mycobacteriaceae; Mycobacterium; Mycobacterium tuberculosis complex; Mycobacterium tuberculosis;	NCBI: 1417028	4391674	99.94	0.00	Yes
NCBI Assembly	GCA_000659925.1	NCBI: cellular organisms; Bacteria; Terrabacteria group; Actinobacteria; Actinomycetia; Corynebacteriales; Mycobacteriaceae; Mycobacterium; Mycobacterium tuberculosis complex; Mycobacterium tuberculosis;	NCBI: 1295791	4405227	99.94	0.00	Yes
NCBI Assembly	GCA_000655395.1	NCBI: cellular organisms; Bacteria; Terrabacteria group; Actinobacteria; Actinomycetia; Corynebacteriales; Mycobacteriaceae; Mycobacterium; Mycobacterium tuberculosis complex; Mycobacterium tuberculosis;	NCBI: 1427276	4420942	99.94	0.00	Yes
NCBI Assembly	GCA_000651315.1	NCBI: cellular organisms; Bacteria; Terrabacteria group; Actinobacteria; Actinomycetia; Corynebacteriales; Mycobacteriaceae; Mycobacterium; Mycobacterium tuberculosis complex; Mycobacterium tuberculosis;	NCBI: 1447527	4425047	99.94	0.00	Yes
NCBI Assembly	GCA_000177815.2	NCBI: cellular organisms; Bacteria; Terrabacteria group; Actinobacteria; Actinomycetia; Corynebacteriales; Mycobacteriaceae; Mycobacterium; Mycobacterium tuberculosis complex; Mycobacterium tuberculosis;	NCBI: 675515	4371565	99.36	0.00	Yes
NCBI Assembly	GCA_000650675.1	NCBI: cellular organisms; Bacteria; Terrabacteria group; Actinobacteria; Actinomycetia; Corynebacteriales; Mycobacteriaceae; Mycobacterium; Mycobacterium tuberculosis complex; Mycobacterium tuberculosis;	NCBI: 1447490	4423548	99.94	0.00	Yes
NCBI Assembly	GCA_000650375.1	NCBI: cellular organisms; Bacteria; Terrabacteria group; Actinobacteria; Actinomycetia; Corynebacteriales; Mycobacteriaceae; Mycobacterium; Mycobacterium tuberculosis complex; Mycobacterium tuberculosis;	NCBI: 1447469	4376626	99.94	0.00	Yes
NCBI Assembly	GCA_000215815.2	NCBI: cellular organisms; Bacteria; Terrabacteria group; Actinobacteria; Actinomycetia; Corynebacteriales; Mycobacteriaceae; Mycobacterium; Mycobacterium avium complex (MAC); Mycobacterium avium; Mycobacterium avium subsp. paratuberculosis;	NCBI: 1029813	4707985	98.60	1.19	Yes
NCBI Assembly	GCA_000155245.1	NCBI: cellular organisms; Bacteria; Terrabacteria group; Actinobacteria; Actinomycetia; Corynebacteriales; Mycobacteriaceae; Mycobacterium; Mycobacterium tuberculosis complex; Mycobacterium tuberculosis;	NCBI: 520141	4424674	94.47	0.38	Yes
NCBI Assembly	GCA_000270845.1	NCBI: cellular organisms; Bacteria; Terrabacteria group; Actinobacteria; Actinomycetia; Corynebacteriales; Mycobacteriaceae; Mycobacteroides; Mycobacteroides abscessus;	NCBI: 1001715	5231383	100.00	0.00	Yes
NCBI Assembly	GCA_000658035.1	NCBI: cellular organisms; Bacteria; Terrabacteria group; Actinobacteria; Actinomycetia; Corynebacteriales; Mycobacteriaceae; Mycobacterium; Mycobacterium tuberculosis complex; Mycobacterium tuberculosis;	NCBI: 1438890	4395652	99.94	0.83	Yes
NCBI Assembly	GCA_000659945.1	NCBI: cellular organisms; Bacteria; Terrabacteria group; Actinobacteria; Actinomycetia; Corynebacteriales; Mycobacteriaceae; Mycobacterium; Mycobacterium tuberculosis complex; Mycobacterium tuberculosis;	NCBI: 1295792	4408664	99.94	0.00	Yes
NCBI Assembly	GCA_000177835.2	NCBI: cellular organisms; Bacteria; Terrabacteria group; Actinobacteria; Actinomycetia; Corynebacteriales; Mycobacteriaceae; Mycobacterium; Mycobacterium tuberculosis complex; Mycobacterium tuberculosis;	NCBI: 675516	4376086	99.88	0.00	Yes
NCBI Assembly	GCA_000651335.1	NCBI: cellular organisms; Bacteria; Terrabacteria group; Actinobacteria; Actinomycetia; Corynebacteriales; Mycobacteriaceae; Mycobacterium; Mycobacterium tuberculosis complex; Mycobacterium tuberculosis;	NCBI: 1448390	4416296	99.78	0.00	Yes
NCBI Assembly	GCA_000655415.1	NCBI: cellular organisms; Bacteria; Terrabacteria group; Actinobacteria; Actinomycetia; Corynebacteriales; Mycobacteriaceae; Mycobacterium; Mycobacterium tuberculosis complex; Mycobacterium tuberculosis;	NCBI: 1427277	4405292	99.94	0.00	Yes
NCBI Assembly	GCA_000650695.1	NCBI: cellular organisms; Bacteria; Terrabacteria group; Actinobacteria; Actinomycetia; Corynebacteriales; Mycobacteriaceae; Mycobacterium; Mycobacterium tuberculosis complex; Mycobacterium tuberculosis;	NCBI: 1447491	4361151	99.94	0.00	Yes
NCBI Assembly	GCA_000650395.1	NCBI: cellular organisms; Bacteria; Terrabacteria group; Actinobacteria; Actinomycetia; Corynebacteriales; Mycobacteriaceae; Mycobacterium; Mycobacterium tuberculosis complex; Mycobacterium tuberculosis;	NCBI: 1447470	4350484	99.94	0.00	Yes
NCBI Assembly	GCA_000155285.1	NCBI: cellular organisms; Bacteria; Terrabacteria group; Actinobacteria; Actinomycetia; Corynebacteriales; Mycobacteriaceae; Mycobacterium; Mycobacterium tuberculosis complex; Mycobacterium tuberculosis;	NCBI: 537209	4356653	94.06	0.50	Yes

NCBI Assembly	GCA_000215835.2	NCBI: cellular organisms; Bacteria; Terrabacteria group; Actinobacteria; Actinomycetia; Corynebacteriales; Mycobacteriaceae; Mycobacterium; Mycobacterium avium complex (MAC); Mycobacterium avium; Mycobacterium avium subsp. paratuberculosis;	NCBI: 1029812	4612386	94.65	2.39	Yes
NCBI Assembly	GCA_000270865.1	NCBI: cellular organisms; Bacteria; Terrabacteria group; Actinobacteria; Actinomycetia; Corynebacteriales; Mycobacteriaceae; Mycobacteroides; Mycobacteroides abscessus;	NCBI: 1001741	5328817	100.00	0.00	Yes
NCBI Assembly	GCA_000313215.2	NCBI: cellular organisms; Bacteria; Terrabacteria group; Actinobacteria; Actinomycetia; Corynebacteriales; Mycobacteriaceae; Mycobacterium; Mycobacterium tuberculosis complex; Mycobacterium tuberculosis;	NCBI: 1240677	4352382	99.94	0.00	Yes
NCBI Assembly	GCA_000658055.1	NCBI: cellular organisms; Bacteria; Terrabacteria group; Actinobacteria; Actinomycetia; Corynebacteriales; Mycobacteriaceae; Mycobacterium; Mycobacterium tuberculosis complex; Mycobacterium tuberculosis;	NCBI: 1438840	4398718	99.94	0.22	Yes
NCBI Assembly	GCA_000177855.2	NCBI: cellular organisms; Bacteria; Terrabacteria group; Actinobacteria; Actinomycetia; Corynebacteriales; Mycobacteriaceae; Mycobacterium; Mycobacterium tuberculosis complex; Mycobacterium tuberculosis;	NCBI: 675517	4365679	99.67	0.00	Yes
NCBI Assembly	GCA_000659965.1	NCBI: cellular organisms; Bacteria; Terrabacteria group; Actinobacteria; Actinomycetia; Corynebacteriales; Mycobacteriaceae; Mycobacterium; Mycobacterium tuberculosis complex; Mycobacterium tuberculosis;	NCBI: 1295795	4361251	99.94	0.00	Yes
NCBI Assembly	GCA_000651355.1	NCBI: cellular organisms; Bacteria; Terrabacteria group; Actinobacteria; Actinomycetia; Corynebacteriales; Mycobacteriaceae; Mycobacterium; Mycobacterium tuberculosis complex; Mycobacterium tuberculosis;	NCBI: 1448392	4416193	99.94	0.00	Yes
NCBI Assembly	GCA_000655435.1	NCBI: cellular organisms; Bacteria; Terrabacteria group; Actinobacteria; Actinomycetia; Corynebacteriales; Mycobacteriaceae; Mycobacterium; Mycobacterium tuberculosis complex; Mycobacterium tuberculosis;	NCBI: 1427278	4419213	99.94	0.00	Yes
NCBI Assembly	GCA_000650415.1	NCBI: cellular organisms; Bacteria; Terrabacteria group; Actinobacteria; Actinomycetia; Corynebacteriales; Mycobacteriaceae; Mycobacterium; Mycobacterium tuberculosis complex; Mycobacterium tuberculosis;	NCBI: 1447471	4418733	99.94	0.00	Yes
NCBI Assembly	GCA_000650715.1	NCBI: cellular organisms; Bacteria; Terrabacteria group; Actinobacteria; Actinomycetia; Corynebacteriales; Mycobacteriaceae; Mycobacterium; Mycobacterium tuberculosis complex; Mycobacterium tuberculosis;	NCBI: 1447492	4378494	99.94	0.00	Yes
NCBI Assembly	GCA_000155305.1	NCBI: cellular organisms; Bacteria; Terrabacteria group; Actinobacteria; Actinomycetia; Corynebacteriales; Mycobacteriaceae; Mycobacterium; Mycobacterium tuberculosis complex; Mycobacterium tuberculosis;	NCBI: 537210	4430528	91.87	0.42	Yes
NCBI Assembly	GCA_000216015.2	NCBI: cellular organisms; Bacteria; Terrabacteria group; Actinobacteria; Actinomycetia; Corynebacteriales; Mycobacteriaceae; Mycobacterium; Mycobacterium avium complex (MAC); Mycobacterium avium; Mycobacterium avium subsp. paratuberculosis;	NCBI: 1029814	4523318	93.38	1.54	Yes
NCBI Assembly	GCA_000270885.1	NCBI: cellular organisms; Bacteria; Terrabacteria group; Actinobacteria; Actinomycetia; Corynebacteriales; Mycobacteriaceae; Mycobacteroides; Mycobacteroides abscessus; Mycobacteroides abscessus subsp. bolletii;	NCBI: 1001706	4894488	100.00	0.00	Yes
NCBI Assembly	GCA_000177875.2	NCBI: cellular organisms; Bacteria; Terrabacteria group; Actinobacteria; Actinomycetia; Corynebacteriales; Mycobacteriaceae; Mycobacterium; Mycobacterium tuberculosis complex; Mycobacterium tuberculosis;	NCBI: 675518	4364362	99.88	0.00	Yes
NCBI Assembly	GCA_000658075.1	NCBI: cellular organisms; Bacteria; Terrabacteria group; Actinobacteria; Actinomycetia; Corynebacteriales; Mycobacteriaceae; Mycobacterium; Mycobacterium tuberculosis complex; Mycobacterium tuberculosis;	NCBI: 1438884	4409958	99.94	0.00	Yes
NCBI Assembly	GCA_000650735.1	NCBI: cellular organisms; Bacteria; Terrabacteria group; Actinobacteria; Actinomycetia; Corynebacteriales; Mycobacteriaceae; Mycobacterium; Mycobacterium tuberculosis complex; Mycobacterium tuberculosis;	NCBI: 1447493	4376308	99.94	0.00	Yes
NCBI Assembly	GCA_000659985.1	NCBI: cellular organisms; Bacteria; Terrabacteria group; Actinobacteria; Actinomycetia; Corynebacteriales; Mycobacteriaceae; Mycobacterium; Mycobacterium tuberculosis complex; Mycobacterium tuberculosis;	NCBI: 1295796	4409967	99.94	0.00	Yes
NCBI Assembly	GCA_000155795.1	NCBI: cellular organisms; Bacteria; Terrabacteria group; Actinobacteria; Actinomycetia; Corynebacteriales; Mycobacteriaceae; Mycobacterium; Mycobacterium tuberculosis complex; Mycobacterium tuberculosis;	NCBI: 555461	4335032	98.58	0.07	Yes

NCBI Assembly	GCA_000651375.1	NCBI: cellular organisms; Bacteria; Terrabacteria group; Actinobacteria; Actinomycetia; Corynebacteriales; Mycobacteriaceae; Mycobacterium; Mycobacterium tuberculosis complex; Mycobacterium tuberculosis;	NCBI: 1448393	4421552	99.94	0.00	Yes
NCBI Assembly	GCA_000650435.1	NCBI: cellular organisms; Bacteria; Terrabacteria group; Actinobacteria; Actinomycetia; Corynebacteriales; Mycobacteriaceae; Mycobacterium; Mycobacterium tuberculosis complex; Mycobacterium tuberculosis;	NCBI: 1447472	4386319	99.94	0.00	Yes
NCBI Assembly	GCA_000655455.1	NCBI: cellular organisms; Bacteria; Terrabacteria group; Actinobacteria; Actinomycetia; Corynebacteriales; Mycobacteriaceae; Mycobacterium; Mycobacterium tuberculosis complex; Mycobacterium tuberculosis;	NCBI: 1427280	4424205	99.61	0.00	Yes
NCBI Assembly	GCA_000270905.1	NCBI: cellular organisms; Bacteria; Terrabacteria group; Actinobacteria; Actinomycetia; Corynebacteriales; Mycobacteriaceae; Mycobacteroides; Mycobacteroides abscessus; Mycobacteroides abscessus subsp. bolletii;	NCBI: 1001708	4810868	100.00	0.00	Yes
NCBI Assembly	GCA_000218035.2	NCBI: cellular organisms; Bacteria; Terrabacteria group; Actinobacteria; Actinomycetia; Corynebacteriales; Mycobacteriaceae; Mycobacterium; Mycobacterium avium complex (MAC); Mycobacterium avium; Mycobacterium avium subsp. paratuberculosis;	NCBI: 1029815	4647327	97.50	0.89	Yes
NCBI Assembly	GCA_000177895.2	NCBI: cellular organisms; Bacteria; Terrabacteria group; Actinobacteria; Actinomycetia; Corynebacteriales; Mycobacteriaceae; Mycobacterium; Mycobacterium tuberculosis complex; Mycobacterium tuberculosis;	NCBI: 675519	4376842	99.85	0.00	Yes
NCBI Assembly	GCA_000330785.1	NCBI: cellular organisms; Bacteria; Terrabacteria group; Actinobacteria; Actinomycetia; Corynebacteriales; Mycobacteriaceae; Mycobacterium; Mycobacterium avium complex (MAC); Mycobacterium avium; Mycobacterium avium subsp. paratuberculosis;	NCBI: 1247747	4798157	97.74	0.33	Yes
NCBI Assembly	GCA_000658095.1	NCBI: cellular organisms; Bacteria; Terrabacteria group; Actinobacteria; Actinomycetia; Corynebacteriales; Mycobacteriaceae; Mycobacterium; Mycobacterium tuberculosis complex; Mycobacterium tuberculosis;	NCBI: 1427202	4421274	99.94	0.00	Yes
NCBI Assembly	GCA_000650755.1	NCBI: cellular organisms; Bacteria; Terrabacteria group; Actinobacteria; Actinomycetia; Corynebacteriales; Mycobacteriaceae; Mycobacterium; Mycobacterium tuberculosis complex; Mycobacterium tuberculosis;	NCBI: 1447494	4387129	99.94	0.00	Yes
NCBI Assembly	GCA_000660005.1	NCBI: cellular organisms; Bacteria; Terrabacteria group; Actinobacteria; Actinomycetia; Corynebacteriales; Mycobacteriaceae; Mycobacterium; Mycobacterium tuberculosis complex; Mycobacterium tuberculosis;	NCBI: 1295797	4400625	99.94	0.00	Yes
NCBI Assembly	GCA_000655475.1	NCBI: cellular organisms; Bacteria; Terrabacteria group; Actinobacteria; Actinomycetia; Corynebacteriales; Mycobacteriaceae; Mycobacterium; Mycobacterium tuberculosis complex; Mycobacterium tuberculosis;	NCBI: 1427281	4400420	99.94	0.00	Yes
NCBI Assembly	GCA_000651395.1	NCBI: cellular organisms; Bacteria; Terrabacteria group; Actinobacteria; Actinomycetia; Corynebacteriales; Mycobacteriaceae; Mycobacterium; Mycobacterium tuberculosis complex; Mycobacterium tuberculosis;	NCBI: 1448394	4418499	99.94	0.00	Yes
NCBI Assembly	GCA_000650455.1	NCBI: cellular organisms; Bacteria; Terrabacteria group; Actinobacteria; Actinomycetia; Corynebacteriales; Mycobacteriaceae; Mycobacterium; Mycobacterium tuberculosis complex; Mycobacterium tuberculosis;	NCBI: 1447474	4426729	99.94	0.00	Yes
NCBI Assembly	GCA_000177915.2	NCBI: cellular organisms; Bacteria; Terrabacteria group; Actinobacteria; Actinomycetia; Corynebacteriales; Mycobacteriaceae; Mycobacterium; Mycobacterium tuberculosis complex; Mycobacterium tuberculosis;	NCBI: 675520	4375659	99.46	0.00	Yes
NCBI Assembly	GCA_000218055.2	NCBI: cellular organisms; Bacteria; Terrabacteria group; Actinobacteria; Actinomycetia; Corynebacteriales; Mycobacteriaceae; Mycobacterium; Mycobacterium avium complex (MAC); Mycobacterium avium; Mycobacterium avium subsp. paratuberculosis;	NCBI: 1029816	4582918	94.95	0.78	Yes
NCBI Assembly	GCA_000270925.1	NCBI: cellular organisms; Bacteria; Terrabacteria group; Actinobacteria; Actinomycetia; Corynebacteriales; Mycobacteriaceae; Mycobacteroides; Mycobacteroides abscessus; Mycobacteroides abscessus subsp. bolletii;	NCBI: 1001709	4811264	100.00	0.00	Yes
NCBI Assembly	GCA_000658115.1	NCBI: cellular organisms; Bacteria; Terrabacteria group; Actinobacteria; Actinomycetia; Corynebacteriales; Mycobacteriaceae; Mycobacterium; Mycobacterium tuberculosis complex; Mycobacterium tuberculosis;	NCBI: 1427214	4418873	99.94	0.00	Yes
NCBI Assembly	GCA_000331165.2	NCBI: cellular organisms; Bacteria; Terrabacteria group; Actinobacteria; Actinomycetia; Corynebacteriales; Mycobacteriaceae; Mycolicibacterium; Mycolicibacterium smegmatis;	NCBI: 1214915	7119169	99.94	1.19	Yes

NCBI Assembly	GCA_000650775.1	NCBI: cellular organisms; Bacteria; Terrabacteria group; Actinobacteria; Actinomycetia; Corynebacteriales; Mycobacteriaceae; Mycobacterium; Mycobacterium tuberculosis complex; Mycobacterium tuberculosis;	NCBI: 1447495	4387848	99.94	0.00	Yes
NCBI Assembly	GCA_000660025.1	NCBI: cellular organisms; Bacteria; Terrabacteria group; Actinobacteria; Actinomycetia; Corynebacteriales; Mycobacteriaceae; Mycobacterium; Mycobacterium tuberculosis complex; Mycobacterium tuberculosis;	NCBI: 1295798	4424878	99.94	0.00	Yes
NCBI Assembly	GCA_000651415.1	NCBI: cellular organisms; Bacteria; Terrabacteria group; Actinobacteria; Actinomycetia; Corynebacteriales; Mycobacteriaceae; Mycobacterium; Mycobacterium tuberculosis complex; Mycobacterium tuberculosis;	NCBI: 1448395	4426629	99.94	0.00	Yes
NCBI Assembly	GCA_000177935.2	NCBI: cellular organisms; Bacteria; Terrabacteria group; Actinobacteria; Actinomycetia; Corynebacteriales; Mycobacteriaceae; Mycobacterium; Mycobacterium tuberculosis complex; Mycobacterium tuberculosis;	NCBI: 675521	4407182	99.52	0.00	Yes
NCBI Assembly	GCA_000650475.1	NCBI: cellular organisms; Bacteria; Terrabacteria group; Actinobacteria; Actinomycetia; Corynebacteriales; Mycobacteriaceae; Mycobacterium; Mycobacterium tuberculosis complex; Mycobacterium tuberculosis;	NCBI: 1447475	4429121	99.94	0.00	Yes
NCBI Assembly	GCA_000655495.1	NCBI: cellular organisms; Bacteria; Terrabacteria group; Actinobacteria; Actinomycetia; Corynebacteriales; Mycobacteriaceae; Mycobacterium; Mycobacterium tuberculosis complex; Mycobacterium tuberculosis;	NCBI: 1427282	4418937	99.94	0.00	Yes
NCBI Assembly	GCA_000218075.2	NCBI: cellular organisms; Bacteria; Terrabacteria group; Actinobacteria; Actinomycetia; Corynebacteriales; Mycobacteriaceae; Mycobacterium; Mycobacterium avium complex (MAC); Mycobacterium avium; Mycobacterium avium subsp. paratuberculosis;	NCBI: 1029817	4568858	95.09	2.06	Yes
NCBI Assembly	GCA_000270945.1	NCBI: cellular organisms; Bacteria; Terrabacteria group; Actinobacteria; Actinomycetia; Corynebacteriales; Mycobacteriaceae; Mycobacteroides; Mycobacteroides abscessus; Mycobacteroides abscessus subsp. bolletii;	NCBI: 1001710	4811369	100.00	0.00	Yes
NCBI Assembly	GCA_000159735.1	NCBI: cellular organisms; Bacteria; Terrabacteria group; Actinobacteria; Actinomycetia; Corynebacteriales; Mycobacteriaceae; Mycobacterium; Mycobacterium tuberculosis complex; Mycobacterium tuberculosis;	NCBI: 611302	4412529	97.32	0.50	Yes
NCBI Assembly	GCA_000445675.1	NCBI: cellular organisms; Bacteria; Terrabacteria group; Actinobacteria; Actinomycetia; Corynebacteriales; Mycobacteriaceae; Mycobacterium; Mycobacterium tuberculosis complex; Mycobacterium tuberculosis;	NCBI: 1352585	4405806	99.94	0.00	Yes
NCBI Assembly	GCA_000658135.1	NCBI: cellular organisms; Bacteria; Terrabacteria group; Actinobacteria; Actinomycetia; Corynebacteriales; Mycobacteriaceae; Mycobacterium; Mycobacterium tuberculosis complex; Mycobacterium tuberculosis;	NCBI: 1427231	4417623	99.94	0.00	Yes
NCBI Assembly	GCA_000650795.1	NCBI: cellular organisms; Bacteria; Terrabacteria group; Actinobacteria; Actinomycetia; Corynebacteriales; Mycobacteriaceae; Mycobacterium; Mycobacterium tuberculosis complex; Mycobacterium tuberculosis;	NCBI: 1447496	4415676	99.94	0.00	Yes
NCBI Assembly	GCA_000660045.1	NCBI: cellular organisms; Bacteria; Terrabacteria group; Actinobacteria; Actinomycetia; Corynebacteriales; Mycobacteriaceae; Mycobacterium; Mycobacterium tuberculosis complex; Mycobacterium tuberculosis;	NCBI: 1295799	4417427	99.94	0.00	Yes
NCBI Assembly	GCA_000177955.2	NCBI: cellular organisms; Bacteria; Terrabacteria group; Actinobacteria; Actinomycetia; Corynebacteriales; Mycobacteriaceae; Mycobacterium; Mycobacterium tuberculosis complex; Mycobacterium tuberculosis;	NCBI: 675522	4417971	98.74	0.00	Yes
NCBI Assembly	GCA_000650495.1	NCBI: cellular organisms; Bacteria; Terrabacteria group; Actinobacteria; Actinomycetia; Corynebacteriales; Mycobacteriaceae; Mycobacterium; Mycobacterium tuberculosis complex; Mycobacterium tuberculosis;	NCBI: 1447476	4393408	99.94	0.00	Yes
NCBI Assembly	GCA_000651435.1	NCBI: cellular organisms; Bacteria; Terrabacteria group; Actinobacteria; Actinomycetia; Corynebacteriales; Mycobacteriaceae; Mycobacterium; Mycobacterium tuberculosis complex; Mycobacterium tuberculosis;	NCBI: 1448396	4404560	99.94	0.00	Yes
NCBI Assembly	GCA_000655515.1	NCBI: cellular organisms; Bacteria; Terrabacteria group; Actinobacteria; Actinomycetia; Corynebacteriales; Mycobacteriaceae; Mycobacterium; Mycobacterium tuberculosis complex; Mycobacterium tuberculosis;	NCBI: 1427283	4429348	99.94	0.00	Yes
NCBI Assembly	GCA_000159755.1	NCBI: cellular organisms; Bacteria; Terrabacteria group; Actinobacteria; Actinomycetia; Corynebacteriales; Mycobacteriaceae; Mycobacterium; Mycobacterium tuberculosis complex; Mycobacterium tuberculosis;	NCBI: 611303	4420336	99.78	0.07	Yes

NCBI Assembly	GCA_000218095.2	NCBI: cellular organisms; Bacteria; Terrabacteria group; Actinobacteria; Actinomycetia; Corynebacteriales; Mycobacteriaceae; Mycobacterium; Mycobacterium avium complex (MAC); Mycobacterium avium; Mycobacterium avium subsp. paratuberculosis;	NCBI: 1029818	4582551	94.71	1.53	Yes
NCBI Assembly	GCA_000270965.1	NCBI: cellular organisms; Bacteria; Terrabacteria group; Actinobacteria; Actinomycetia; Corynebacteriales; Mycobacteriaceae; Mycobacteroides; Mycobacteroides abscessus;	NCBI: 1001732	5254648	100.00	0.00	Yes
NCBI Assembly	GCA_000445695.1	NCBI: cellular organisms; Bacteria; Terrabacteria group; Actinobacteria; Actinomycetia; Corynebacteriales; Mycobacteriaceae; Mycobacterium; Mycobacterium tuberculosis complex; Mycobacterium tuberculosis;	NCBI: 1352586	4399724	99.94	0.00	Yes
NCBI Assembly	GCA_000658155.1	NCBI: cellular organisms; Bacteria; Terrabacteria group; Actinobacteria; Actinomycetia; Corynebacteriales; Mycobacteriaceae; Mycobacterium; Mycobacterium tuberculosis complex; Mycobacterium tuberculosis;	NCBI: 1427253	4427587	99.94	0.00	Yes
NCBI Assembly	GCA_000177975.2	NCBI: cellular organisms; Bacteria; Terrabacteria group; Actinobacteria; Actinomycetia; Corynebacteriales; Mycobacteriaceae; Mycobacterium; Mycobacterium tuberculosis complex; Mycobacterium tuberculosis;	NCBI: 675523	4399576	97.68	0.33	Yes
NCBI Assembly	GCA_000660065.1	NCBI: cellular organisms; Bacteria; Terrabacteria group; Actinobacteria; Actinomycetia; Corynebacteriales; Mycobacteriaceae; Mycobacterium; Mycobacterium tuberculosis complex; Mycobacterium tuberculosis;	NCBI: 1295800	4415779	99.94	0.00	Yes
NCBI Assembly	GCA_000650815.1	NCBI: cellular organisms; Bacteria; Terrabacteria group; Actinobacteria; Actinomycetia; Corynebacteriales; Mycobacteriaceae; Mycobacterium; Mycobacterium tuberculosis complex; Mycobacterium tuberculosis;	NCBI: 1447497	4401242	99.94	0.44	Yes
NCBI Assembly	GCA_000651455.1	NCBI: cellular organisms; Bacteria; Terrabacteria group; Actinobacteria; Actinomycetia; Corynebacteriales; Mycobacteriaceae; Mycobacterium; Mycobacterium tuberculosis complex; Mycobacterium tuberculosis;	NCBI: 1448397	4421214	99.94	0.00	Yes
NCBI Assembly	GCA_000650515.1	NCBI: cellular organisms; Bacteria; Terrabacteria group; Actinobacteria; Actinomycetia; Corynebacteriales; Mycobacteriaceae; Mycobacterium; Mycobacterium tuberculosis complex; Mycobacterium tuberculosis;	NCBI: 1447479	4434992	99.94	0.08	Yes
NCBI Assembly	GCA_000159775.1	NCBI: cellular organisms; Bacteria; Terrabacteria group; Actinobacteria; Actinomycetia; Corynebacteriales; Mycobacteriaceae; Mycobacterium; Mycobacterium tuberculosis complex; Mycobacterium tuberculosis; Mycobacterium tuberculosis variant africanum;	NCBI: 611304	4432952	99.86	0.67	Yes
NCBI Assembly	GCA_000270985.1	NCBI: cellular organisms; Bacteria; Terrabacteria group; Actinobacteria; Actinomycetia; Corynebacteriales; Mycobacteriaceae; Mycobacteroides; Mycobacteroides abscessus;	NCBI: 1001740	5139423	100.00	0.00	Yes
NCBI Assembly	GCA_000655535.1	NCBI: cellular organisms; Bacteria; Terrabacteria group; Actinobacteria; Actinomycetia; Corynebacteriales; Mycobacteriaceae; Mycobacterium; Mycobacterium tuberculosis complex; Mycobacterium tuberculosis;	NCBI: 1427285	4430065	99.94	0.00	Yes
NCBI Assembly	GCA_000443935.1	NCBI: cellular organisms; Bacteria; Terrabacteria group; Actinobacteria; Actinomycetia; Corynebacteriales; Mycobacteriaceae; Mycobacterium; Mycobacterium tuberculosis complex; Mycobacterium tuberculosis;	NCBI: 1346781	4404380	99.55	0.00	Yes
NCBI Assembly	GCA_000445715.1	NCBI: cellular organisms; Bacteria; Terrabacteria group; Actinobacteria; Actinomycetia; Corynebacteriales; Mycobacteriaceae; Mycobacterium; Mycobacterium tuberculosis complex; Mycobacterium tuberculosis;	NCBI: 1352587	4400549	99.94	0.00	Yes
NCBI Assembly	GCA_000443595.1	NCBI: cellular organisms; Bacteria; Terrabacteria group; Actinobacteria; Actinomycetia; Corynebacteriales; Mycobacteriaceae; Mycobacterium; Mycobacterium tuberculosis complex; Mycobacterium tuberculosis;	NCBI: 1346773	4400856	99.94	0.00	Yes
NCBI Assembly	GCA_000658175.1	NCBI: cellular organisms; Bacteria; Terrabacteria group; Actinobacteria; Actinomycetia; Corynebacteriales; Mycobacteriaceae; Mycobacterium; Mycobacterium tuberculosis complex; Mycobacterium tuberculosis;	NCBI: 1447435	4416056	99.94	0.00	Yes
NCBI Assembly	GCA_000660085.1	NCBI: cellular organisms; Bacteria; Terrabacteria group; Actinobacteria; Actinomycetia; Corynebacteriales; Mycobacteriaceae; Mycobacterium; Mycobacterium tuberculosis complex; Mycobacterium tuberculosis;	NCBI: 1295801	4419334	99.94	0.00	Yes
NCBI Assembly	GCA_000650835.1	NCBI: cellular organisms; Bacteria; Terrabacteria group; Actinobacteria; Actinomycetia; Corynebacteriales; Mycobacteriaceae; Mycobacterium; Mycobacterium tuberculosis complex; Mycobacterium tuberculosis;	NCBI: 1447498	4360309	99.61	0.00	Yes

NCBI Assembly	GCA_000650535.1	NCBI: cellular organisms; Bacteria; Terrabacteria group; Actinobacteria; Actinomycetia; Corynebacteriales; Mycobacteriaceae; Mycobacterium; Mycobacterium tuberculosis complex; Mycobacterium tuberculosis;	NCBI: 1447480	4421449	99.94	0.00	Yes
NCBI Assembly	GCA_000651475.1	NCBI: cellular organisms; Bacteria; Terrabacteria group; Actinobacteria; Actinomycetia; Corynebacteriales; Mycobacteriaceae; Mycobacterium; Mycobacterium tuberculosis complex; Mycobacterium tuberculosis;	NCBI: 1448398	4420564	99.94	0.00	Yes
NCBI Assembly	GCA_000454325.1	NCBI: cellular organisms; Bacteria; Terrabacteria group; Actinobacteria; Actinomycetia; Corynebacteriales; Mycobacteriaceae; Mycobacterium; Mycobacterium tuberculosis complex; Mycobacterium tuberculosis;	NCBI: 1245275	4363305	99.94	0.00	Yes
NCBI Assembly	GCA_000271005.1	NCBI: cellular organisms; Bacteria; Terrabacteria group; Actinobacteria; Actinomycetia; Corynebacteriales; Mycobacteriaceae; Mycobacteroides; Mycobacteroides abscessus;	NCBI: 1001735	5248885	100.00	0.00	Yes
NCBI Assembly	GCA_000445245.1	NCBI: cellular organisms; Bacteria; Terrabacteria group; Actinobacteria; Actinomycetia; Corynebacteriales; Mycobacteriaceae; Mycobacterium; Mycobacterium tuberculosis complex; Mycobacterium tuberculosis;	NCBI: 1352570	4407393	99.94	0.00	Yes
NCBI Assembly	GCA_000657115.1	NCBI: cellular organisms; Bacteria; Terrabacteria group; Actinobacteria; Actinomycetia; Corynebacteriales; Mycobacteriaceae; Mycobacterium; Mycobacterium tuberculosis complex; Mycobacterium tuberculosis;	NCBI: 1455264	4429834	99.94	0.00	Yes
NCBI Assembly	GCA_000445735.1	NCBI: cellular organisms; Bacteria; Terrabacteria group; Actinobacteria; Actinomycetia; Corynebacteriales; Mycobacteriaceae; Mycobacterium; Mycobacterium tuberculosis complex; Mycobacterium tuberculosis;	NCBI: 1352588	4406371	99.94	0.00	Yes
NCBI Assembly	GCA_000443615.1	NCBI: cellular organisms; Bacteria; Terrabacteria group; Actinobacteria; Actinomycetia; Corynebacteriales; Mycobacteriaceae; Mycobacterium; Mycobacterium tuberculosis complex; Mycobacterium tuberculosis;	NCBI: 1346774	4412421	99.94	0.00	Yes
NCBI Assembly	GCA_000654295.1	NCBI: cellular organisms; Bacteria; Terrabacteria group; Actinobacteria; Actinomycetia; Corynebacteriales; Mycobacteriaceae; Mycobacterium; Mycobacterium tuberculosis complex; Mycobacterium tuberculosis;	NCBI: 1448613	4428150	99.94	0.00	Yes
NCBI Assembly	GCA_000660105.1	NCBI: cellular organisms; Bacteria; Terrabacteria group; Actinobacteria; Actinomycetia; Corynebacteriales; Mycobacteriaceae; Mycobacterium; Mycobacterium tuberculosis complex; Mycobacterium tuberculosis;	NCBI: 1295802	4410067	99.94	0.00	Yes
NCBI Assembly	GCA_000650855.1	NCBI: cellular organisms; Bacteria; Terrabacteria group; Actinobacteria; Actinomycetia; Corynebacteriales; Mycobacteriaceae; Mycobacterium; Mycobacterium tuberculosis complex; Mycobacterium tuberculosis;	NCBI: 1447500	4395068	99.94	0.00	Yes
NCBI Assembly	GCA_000454345.1	NCBI: cellular organisms; Bacteria; Terrabacteria group; Actinobacteria; Actinomycetia; Corynebacteriales; Mycobacteriaceae; Mycobacterium; Mycobacterium tuberculosis complex; Mycobacterium tuberculosis;	NCBI: 1245787	4373311	99.61	0.00	Yes
NCBI Assembly	GCA_000653375.1	NCBI: cellular organisms; Bacteria; Terrabacteria group; Actinobacteria; Actinomycetia; Corynebacteriales; Mycobacteriaceae; Mycobacterium; Mycobacterium tuberculosis complex; Mycobacterium tuberculosis;	NCBI: 1448526	4447652	99.94	1.29	Yes
NCBI Assembly	GCA_000652755.1	NCBI: cellular organisms; Bacteria; Terrabacteria group; Actinobacteria; Actinomycetia; Corynebacteriales; Mycobacteriaceae; Mycobacterium; Mycobacterium tuberculosis complex; Mycobacterium tuberculosis;	NCBI: 1448481	4396255	99.94	0.00	Yes
NCBI Assembly	GCA_000445265.1	NCBI: cellular organisms; Bacteria; Terrabacteria group; Actinobacteria; Actinomycetia; Corynebacteriales; Mycobacteriaceae; Mycobacterium; Mycobacterium tuberculosis complex; Mycobacterium tuberculosis;	NCBI: 1352571	4386949	99.94	0.00	Yes
NCBI Assembly	GCA_000653055.1	NCBI: cellular organisms; Bacteria; Terrabacteria group; Actinobacteria; Actinomycetia; Corynebacteriales; Mycobacteriaceae; Mycobacterium; Mycobacterium tuberculosis complex; Mycobacterium tuberculosis;	NCBI: 1448502	4402909	99.94	0.00	Yes
NCBI Assembly	GCA_000445755.1	NCBI: cellular organisms; Bacteria; Terrabacteria group; Actinobacteria; Actinomycetia; Corynebacteriales; Mycobacteriaceae; Mycobacterium; Mycobacterium tuberculosis complex; Mycobacterium tuberculosis;	NCBI: 1352589	4402266	99.94	0.00	Yes
NCBI Assembly	GCA_000657135.1	NCBI: cellular organisms; Bacteria; Terrabacteria group; Actinobacteria; Actinomycetia; Corynebacteriales; Mycobacteriaceae; Mycobacterium; Mycobacterium tuberculosis complex; Mycobacterium tuberculosis;	NCBI: 1455266	4400194	99.94	0.00	Yes

NCBI Assembly	GCA_000443635.1	NCBI: cellular organisms; Bacteria; Terrabacteria group; Actinobacteria; Actinomycetia; Corynebacteriales; Mycobacteriaceae; Mycobacterium; Mycobacterium tuberculosis complex; Mycobacterium tuberculosis;	NCBI: 1346775	4407862	99.86	0.00	Yes
NCBI Assembly	GCA_000654315.1	NCBI: cellular organisms; Bacteria; Terrabacteria group; Actinobacteria; Actinomycetia; Corynebacteriales; Mycobacteriaceae; Mycobacterium; Mycobacterium tuberculosis complex; Mycobacterium tuberculosis;	NCBI: 1448614	4429026	99.94	0.33	Yes
NCBI Assembly	GCA_000654935.1	NCBI: cellular organisms; Bacteria; Terrabacteria group; Actinobacteria; Actinomycetia; Corynebacteriales; Mycobacteriaceae; Mycobacterium; Mycobacterium tuberculosis complex; Mycobacterium tuberculosis;	NCBI: 1448647	4418603	99.94	0.00	Yes
NCBI Assembly	GCA_000660125.1	NCBI: cellular organisms; Bacteria; Terrabacteria group; Actinobacteria; Actinomycetia; Corynebacteriales; Mycobacteriaceae; Mycobacterium; Mycobacterium tuberculosis complex; Mycobacterium tuberculosis;	NCBI: 1295803	4402181	99.94	0.00	Yes
NCBI Assembly	GCA_000455125.1	NCBI: cellular organisms; Bacteria; Terrabacteria group; Actinobacteria; Actinomycetia; Corynebacteriales; Mycobacteriaceae; Mycobacterium; unclassified Mycobacterium;	NCBI: 1370120	4473234	100.00	0.61	Yes
NCBI Assembly	GCA_000445285.1	NCBI: cellular organisms; Bacteria; Terrabacteria group; Actinobacteria; Actinomycetia; Corynebacteriales; Mycobacteriaceae; Mycobacterium; Mycobacterium tuberculosis complex; Mycobacterium tuberculosis;	NCBI: 1352572	4407883	99.94	0.00	Yes
NCBI Assembly	GCA_000652775.1	NCBI: cellular organisms; Bacteria; Terrabacteria group; Actinobacteria; Actinomycetia; Corynebacteriales; Mycobacteriaceae; Mycobacterium; Mycobacterium tuberculosis complex; Mycobacterium tuberculosis;	NCBI: 1448482	4394079	99.94	0.00	Yes
NCBI Assembly	GCA_000653395.1	NCBI: cellular organisms; Bacteria; Terrabacteria group; Actinobacteria; Actinomycetia; Corynebacteriales; Mycobacteriaceae; Mycobacterium; Mycobacterium tuberculosis complex; Mycobacterium tuberculosis;	NCBI: 1448528	4417341	99.94	0.00	Yes
NCBI Assembly	GCA_000653075.1	NCBI: cellular organisms; Bacteria; Terrabacteria group; Actinobacteria; Actinomycetia; Corynebacteriales; Mycobacteriaceae; Mycobacterium; Mycobacterium tuberculosis complex; Mycobacterium tuberculosis;	NCBI: 1448504	4389363	99.94	0.00	Yes
NCBI Assembly	GCA_000445775.1	NCBI: cellular organisms; Bacteria; Terrabacteria group; Actinobacteria; Actinomycetia; Corynebacteriales; Mycobacteriaceae; Mycobacterium; Mycobacterium tuberculosis complex; Mycobacterium tuberculosis;	NCBI: 1352590	4394016	99.11	0.00	Yes
NCBI Assembly	GCA_000657155.1	NCBI: cellular organisms; Bacteria; Terrabacteria group; Actinobacteria; Actinomycetia; Corynebacteriales; Mycobacteriaceae; Mycobacterium; Mycobacterium tuberculosis complex; Mycobacterium tuberculosis;	NCBI: 1455268	4390857	99.94	0.00	Yes
NCBI Assembly	GCA_000443655.1	NCBI: cellular organisms; Bacteria; Terrabacteria group; Actinobacteria; Actinomycetia; Corynebacteriales; Mycobacteriaceae; Mycobacterium; Mycobacterium tuberculosis complex; Mycobacterium tuberculosis;	NCBI: 1346776	4392448	99.28	0.00	Yes
NCBI Assembly	GCA_000654335.1	NCBI: cellular organisms; Bacteria; Terrabacteria group; Actinobacteria; Actinomycetia; Corynebacteriales; Mycobacteriaceae; Mycobacterium; Mycobacterium tuberculosis complex; Mycobacterium tuberculosis;	NCBI: 1448615	4429501	99.61	0.25	Yes
NCBI Assembly	GCA_000654955.1	NCBI: cellular organisms; Bacteria; Terrabacteria group; Actinobacteria; Actinomycetia; Corynebacteriales; Mycobacteriaceae; Mycobacterium; Mycobacterium tuberculosis complex; Mycobacterium tuberculosis;	NCBI: 1448648	4419404	99.94	0.00	Yes
NCBI Assembly	GCA_000654615.1	NCBI: cellular organisms; Bacteria; Terrabacteria group; Actinobacteria; Actinomycetia; Corynebacteriales; Mycobacteriaceae; Mycobacterium; Mycobacterium tuberculosis complex; Mycobacterium tuberculosis;	NCBI: 1448629	4414190	99.94	0.00	Yes
NCBI Assembly	GCA_000455145.1	NCBI: cellular organisms; Bacteria; Terrabacteria group; Actinobacteria; Actinomycetia; Corynebacteriales; Mycobacteriaceae; Mycobacterium; unclassified Mycobacterium;	NCBI: 1370121	7690050	99.78	1.51	Yes
NCBI Assembly	GCA_000445305.1	NCBI: cellular organisms; Bacteria; Terrabacteria group; Actinobacteria; Actinomycetia; Corynebacteriales; Mycobacteriaceae; Mycobacterium; Mycobacterium tuberculosis complex; Mycobacterium tuberculosis;	NCBI: 1352573	4388875	95.70	0.00	Yes
NCBI Assembly	GCA_000653095.1	NCBI: cellular organisms; Bacteria; Terrabacteria group; Actinobacteria; Actinomycetia; Corynebacteriales; Mycobacteriaceae; Mycobacterium; Mycobacterium tuberculosis complex; Mycobacterium tuberculosis;	NCBI: 1448505	4403313	99.94	0.57	Yes

NCBI Assembly	GCA_000445795.1	NCBI: cellular organisms; Bacteria; Terrabacteria group; Actinobacteria; Actinomycetia; Corynebacteriales; Mycobacteriaceae; Mycobacterium; Mycobacterium tuberculosis complex; Mycobacterium tuberculosis;	NCBI: 1352591	4392209	99.36	0.00	Yes
NCBI Assembly	GCA_000652795.1	NCBI: cellular organisms; Bacteria; Terrabacteria group; Actinobacteria; Actinomycetia; Corynebacteriales; Mycobacteriaceae; Mycobacterium; Mycobacterium tuberculosis complex; Mycobacterium tuberculosis;	NCBI: 1448483	4401935	99.94	0.00	Yes
NCBI Assembly	GCA_000653415.1	NCBI: cellular organisms; Bacteria; Terrabacteria group; Actinobacteria; Actinomycetia; Corynebacteriales; Mycobacteriaceae; Mycobacterium; Mycobacterium tuberculosis complex; Mycobacterium tuberculosis;	NCBI: 1448529	4363650	99.94	0.04	Yes
NCBI Assembly	GCA_000657175.1	NCBI: cellular organisms; Bacteria; Terrabacteria group; Actinobacteria; Actinomycetia; Corynebacteriales; Mycobacteriaceae; Mycobacterium; Mycobacterium tuberculosis complex; Mycobacterium tuberculosis;	NCBI: 1455269	4389945	99.94	0.03	Yes
NCBI Assembly	GCA_000443675.1	NCBI: cellular organisms; Bacteria; Terrabacteria group; Actinobacteria; Actinomycetia; Corynebacteriales; Mycobacteriaceae; Mycobacterium; Mycobacterium tuberculosis complex; Mycobacterium tuberculosis;	NCBI: 1346777	4394000	99.94	0.00	Yes
NCBI Assembly	GCA_000654355.1	NCBI: cellular organisms; Bacteria; Terrabacteria group; Actinobacteria; Actinomycetia; Corynebacteriales; Mycobacteriaceae; Mycobacterium; Mycobacterium tuberculosis complex; Mycobacterium tuberculosis;	NCBI: 1448616	4412502	99.94	0.00	Yes
NCBI Assembly	GCA_000654635.1	NCBI: cellular organisms; Bacteria; Terrabacteria group; Actinobacteria; Actinomycetia; Corynebacteriales; Mycobacteriaceae; Mycobacterium; Mycobacterium tuberculosis complex; Mycobacterium tuberculosis;	NCBI: 1448632	4423746	99.78	0.00	Yes
NCBI Assembly	GCA_000654975.1	NCBI: cellular organisms; Bacteria; Terrabacteria group; Actinobacteria; Actinomycetia; Corynebacteriales; Mycobacteriaceae; Mycobacterium; Mycobacterium tuberculosis complex; Mycobacterium tuberculosis;	NCBI: 1448649	4438040	99.94	0.00	Yes
NCBI Assembly	GCA_000455165.1	NCBI: cellular organisms; Bacteria; Terrabacteria group; Actinobacteria; Actinomycetia; Corynebacteriales; Mycobacteriaceae; Mycolicibacterium; Mycolicibacterium iranicum;	NCBI: 1370125	6137283	100.00	0.12	Yes
NCBI Assembly	GCA_000445325.1	NCBI: cellular organisms; Bacteria; Terrabacteria group; Actinobacteria; Actinomycetia; Corynebacteriales; Mycobacteriaceae; Mycobacterium; Mycobacterium tuberculosis complex; Mycobacterium tuberculosis;	NCBI: 1352574	4406449	99.94	0.00	Yes
NCBI Assembly	GCA_000653115.1	NCBI: cellular organisms; Bacteria; Terrabacteria group; Actinobacteria; Actinomycetia; Corynebacteriales; Mycobacteriaceae; Mycobacterium; Mycobacterium tuberculosis complex; Mycobacterium tuberculosis;	NCBI: 1448506	4404044	99.94	0.00	Yes
NCBI Assembly	GCA_000445815.1	NCBI: cellular organisms; Bacteria; Terrabacteria group; Actinobacteria; Actinomycetia; Corynebacteriales; Mycobacteriaceae; Mycobacterium; Mycobacterium tuberculosis complex; Mycobacterium tuberculosis;	NCBI: 1352592	4402661	99.94	0.00	Yes
NCBI Assembly	GCA_000652815.1	NCBI: cellular organisms; Bacteria; Terrabacteria group; Actinobacteria; Actinomycetia; Corynebacteriales; Mycobacteriaceae; Mycobacterium; Mycobacterium tuberculosis complex; Mycobacterium tuberculosis;	NCBI: 1448484	4399204	99.94	0.00	Yes
NCBI Assembly	GCA_000653435.1	NCBI: cellular organisms; Bacteria; Terrabacteria group; Actinobacteria; Actinomycetia; Corynebacteriales; Mycobacteriaceae; Mycobacterium; Mycobacterium tuberculosis complex; Mycobacterium tuberculosis;	NCBI: 1448530	4408609	99.94	0.00	Yes
NCBI Assembly	GCA_000443695.1	NCBI: cellular organisms; Bacteria; Terrabacteria group; Actinobacteria; Actinomycetia; Corynebacteriales; Mycobacteriaceae; Mycobacterium; Mycobacterium tuberculosis complex; Mycobacterium tuberculosis;	NCBI: 1346778	4403042	99.50	0.00	Yes
NCBI Assembly	GCA_000657195.1	NCBI: cellular organisms; Bacteria; Terrabacteria group; Actinobacteria; Actinomycetia; Corynebacteriales; Mycobacteriaceae; Mycobacterium; Mycobacterium tuberculosis complex; Mycobacterium tuberculosis;	NCBI: 1455270	4383257	99.94	0.33	Yes
NCBI Assembly	GCA_000654375.1	NCBI: cellular organisms; Bacteria; Terrabacteria group; Actinobacteria; Actinomycetia; Corynebacteriales; Mycobacteriaceae; Mycobacterium; Mycobacterium tuberculosis complex; Mycobacterium tuberculosis;	NCBI: 1448617	4415235	99.94	0.00	Yes
NCBI Assembly	GCA_000654995.1	NCBI: cellular organisms; Bacteria; Terrabacteria group; Actinobacteria; Actinomycetia; Corynebacteriales; Mycobacteriaceae; Mycobacterium; Mycobacterium tuberculosis complex; Mycobacterium tuberculosis;	NCBI: 1448650	4419699	99.94	0.00	Yes

NCBI Assembly	GCA_000654655.1	NCBI: cellular organisms; Bacteria; Terrabacteria group; Actinobacteria; Actinomycetia; Corynebacteriales; Mycobacteriaceae; Mycobacterium; Mycobacterium tuberculosis complex; Mycobacterium tuberculosis;	NCBI: 1448633	4424808	99.94	0.00	Yes
NCBI Assembly	GCA_000455185.1	NCBI: cellular organisms; Bacteria; Terrabacteria group; Actinobacteria; Actinomycetia; Corynebacteriales; Mycobacteriaceae; Mycobacterium; unclassified Mycobacterium;	NCBI: 1370118	6775899	97.29	1.32	Yes
NCBI Assembly	GCA_000445345.1	NCBI: cellular organisms; Bacteria; Terrabacteria group; Actinobacteria; Actinomycetia; Corynebacteriales; Mycobacteriaceae; Mycobacterium; Mycobacterium tuberculosis complex; Mycobacterium tuberculosis;	NCBI: 1352575	4408055	99.94	0.00	Yes
NCBI Assembly	GCA_000653135.1	NCBI: cellular organisms; Bacteria; Terrabacteria group; Actinobacteria; Actinomycetia; Corynebacteriales; Mycobacteriaceae; Mycobacterium; Mycobacterium tuberculosis complex; Mycobacterium tuberculosis;	NCBI: 1448507	4404525	99.94	1.03	Yes
NCBI Assembly	GCA_000445835.1	NCBI: cellular organisms; Bacteria; Terrabacteria group; Actinobacteria; Actinomycetia; Corynebacteriales; Mycobacteriaceae; Mycobacterium; Mycobacterium tuberculosis complex; Mycobacterium tuberculosis;	NCBI: 1352593	4407151	99.94	0.00	Yes
NCBI Assembly	GCA_000652835.1	NCBI: cellular organisms; Bacteria; Terrabacteria group; Actinobacteria; Actinomycetia; Corynebacteriales; Mycobacteriaceae; Mycobacterium; Mycobacterium tuberculosis complex; Mycobacterium tuberculosis;	NCBI: 1448485	4406632	99.94	0.00	Yes
NCBI Assembly	GCA_000653455.1	NCBI: cellular organisms; Bacteria; Terrabacteria group; Actinobacteria; Actinomycetia; Corynebacteriales; Mycobacteriaceae; Mycobacterium; Mycobacterium tuberculosis complex; Mycobacterium tuberculosis;	NCBI: 1448531	4387444	99.94	0.00	Yes
NCBI Assembly	GCA_000443715.1	NCBI: cellular organisms; Bacteria; Terrabacteria group; Actinobacteria; Actinomycetia; Corynebacteriales; Mycobacteriaceae; Mycobacterium; Mycobacterium tuberculosis complex; Mycobacterium tuberculosis;	NCBI: 1346779	4392414	99.94	0.00	Yes
NCBI Assembly	GCA_000657215.1	NCBI: cellular organisms; Bacteria; Terrabacteria group; Actinobacteria; Actinomycetia; Corynebacteriales; Mycobacteriaceae; Mycobacterium; Mycobacterium tuberculosis complex; Mycobacterium tuberculosis;	NCBI: 1455272	4382110	99.94	0.04	Yes
NCBI Assembly	GCA_000655015.1	NCBI: cellular organisms; Bacteria; Terrabacteria group; Actinobacteria; Actinomycetia; Corynebacteriales; Mycobacteriaceae; Mycobacterium; Mycobacterium tuberculosis complex; Mycobacterium tuberculosis;	NCBI: 1448651	4414854	99.94	0.08	Yes
NCBI Assembly	GCA_000654395.1	NCBI: cellular organisms; Bacteria; Terrabacteria group; Actinobacteria; Actinomycetia; Corynebacteriales; Mycobacteriaceae; Mycobacterium; Mycobacterium tuberculosis complex; Mycobacterium tuberculosis;	NCBI: 1448618	4436258	99.94	0.00	Yes
NCBI Assembly	GCA_000654675.1	NCBI: cellular organisms; Bacteria; Terrabacteria group; Actinobacteria; Actinomycetia; Corynebacteriales; Mycobacteriaceae; Mycobacterium; Mycobacterium tuberculosis complex; Mycobacterium tuberculosis;	NCBI: 1448634	4432703	99.94	0.00	Yes
NCBI Assembly	GCA_000455205.1	NCBI: cellular organisms; Bacteria; Terrabacteria group; Actinobacteria; Actinomycetia; Corynebacteriales; Mycobacteriaceae; Mycobacterium; unclassified Mycobacterium;	NCBI: 1370119	6394793	98.64	2.99	Yes
NCBI Assembly	GCA_000445365.1	NCBI: cellular organisms; Bacteria; Terrabacteria group; Actinobacteria; Actinomycetia; Corynebacteriales; Mycobacteriaceae; Mycobacterium; Mycobacterium tuberculosis complex; Mycobacterium tuberculosis;	NCBI: 1352576	4388029	99.94	0.00	Yes
NCBI Assembly	GCA_000445855.1	NCBI: cellular organisms; Bacteria; Terrabacteria group; Actinobacteria; Actinomycetia; Corynebacteriales; Mycobacteriaceae; Mycobacterium; Mycobacterium tuberculosis complex; Mycobacterium tuberculosis;	NCBI: 1352594	4399991	99.94	0.00	Yes
NCBI Assembly	GCA_000653155.1	NCBI: cellular organisms; Bacteria; Terrabacteria group; Actinobacteria; Actinomycetia; Corynebacteriales; Mycobacteriaceae; Mycobacterium; Mycobacterium tuberculosis complex; Mycobacterium tuberculosis;	NCBI: 1448508	4356472	99.94	0.33	Yes
NCBI Assembly	GCA_000652855.1	NCBI: cellular organisms; Bacteria; Terrabacteria group; Actinobacteria; Actinomycetia; Corynebacteriales; Mycobacteriaceae; Mycobacterium; Mycobacterium tuberculosis complex; Mycobacterium tuberculosis;	NCBI: 1448486	4400522	99.94	0.00	Yes
NCBI Assembly	GCA_000443755.1	NCBI: cellular organisms; Bacteria; Terrabacteria group; Actinobacteria; Actinomycetia; Corynebacteriales; Mycobacteriaceae; Mycobacterium; Mycobacterium tuberculosis complex; Mycobacterium tuberculosis;	NCBI: 1346782	4390137	99.94	0.00	Yes

NCBI Assembly	GCA_000653475.1	NCBI: cellular organisms; Bacteria; Terrabacteria group; Actinobacteria; Actinomycetia; Corynebacteriales; Mycobacteriaceae; Mycobacterium; Mycobacterium tuberculosis complex; Mycobacterium tuberculosis;	NCBI: 1448532	4403599	99.94	0.76	Yes
NCBI Assembly	GCA_000657235.1	NCBI: cellular organisms; Bacteria; Terrabacteria group; Actinobacteria; Actinomycetia; Corynebacteriales; Mycobacteriaceae; Mycobacterium; Mycobacterium tuberculosis complex; Mycobacterium tuberculosis;	NCBI: 1455273	4335456	99.31	0.17	Yes
NCBI Assembly	GCA_000655035.1	NCBI: cellular organisms; Bacteria; Terrabacteria group; Actinobacteria; Actinomycetia; Corynebacteriales; Mycobacteriaceae; Mycobacterium; Mycobacterium tuberculosis complex; Mycobacterium tuberculosis;	NCBI: 1448652	4431009	99.94	0.00	Yes
NCBI Assembly	GCA_000654415.1	NCBI: cellular organisms; Bacteria; Terrabacteria group; Actinobacteria; Actinomycetia; Corynebacteriales; Mycobacteriaceae; Mycobacterium; Mycobacterium tuberculosis complex; Mycobacterium tuberculosis;	NCBI: 1448619	4417500	99.94	0.00	Yes
NCBI Assembly	GCA_000654695.1	NCBI: cellular organisms; Bacteria; Terrabacteria group; Actinobacteria; Actinomycetia; Corynebacteriales; Mycobacteriaceae; Mycobacterium; Mycobacterium tuberculosis complex; Mycobacterium tuberculosis;	NCBI: 1448635	4415371	99.94	0.00	Yes
NCBI Assembly	GCA_000445875.1	NCBI: cellular organisms; Bacteria; Terrabacteria group; Actinobacteria; Actinomycetia; Corynebacteriales; Mycobacteriaceae; Mycobacterium; Mycobacterium tuberculosis complex; Mycobacterium tuberculosis;	NCBI: 1352595	4400321	99.94	0.00	Yes
NCBI Assembly	GCA_000445515.1	NCBI: cellular organisms; Bacteria; Terrabacteria group; Actinobacteria; Actinomycetia; Corynebacteriales; Mycobacteriaceae; Mycobacterium; Mycobacterium tuberculosis complex; Mycobacterium tuberculosis;	NCBI: 1352577	4405197	99.94	0.00	Yes
NCBI Assembly	GCA_000652875.1	NCBI: cellular organisms; Bacteria; Terrabacteria group; Actinobacteria; Actinomycetia; Corynebacteriales; Mycobacteriaceae; Mycobacterium; Mycobacterium tuberculosis complex; Mycobacterium tuberculosis;	NCBI: 1448488	4409381	99.94	0.00	Yes
NCBI Assembly	GCA_000653175.1	NCBI: cellular organisms; Bacteria; Terrabacteria group; Actinobacteria; Actinomycetia; Corynebacteriales; Mycobacteriaceae; Mycobacterium; Mycobacterium tuberculosis complex; Mycobacterium tuberculosis;	NCBI: 1448509	4407699	99.94	0.28	Yes
NCBI Assembly	GCA_000653495.1	NCBI: cellular organisms; Bacteria; Terrabacteria group; Actinobacteria; Actinomycetia; Corynebacteriales; Mycobacteriaceae; Mycobacterium; Mycobacterium tuberculosis complex; Mycobacterium tuberculosis;	NCBI: 1448533	4391925	99.94	0.00	Yes
NCBI Assembly	GCA_000443795.1	NCBI: cellular organisms; Bacteria; Terrabacteria group; Actinobacteria; Actinomycetia; Corynebacteriales; Mycobacteriaceae; Mycobacterium; Mycobacterium tuberculosis complex; Mycobacterium tuberculosis;	NCBI: 1346780	4401613	99.94	0.00	Yes
NCBI Assembly	GCA_000657255.1	NCBI: cellular organisms; Bacteria; Terrabacteria group; Actinobacteria; Actinomycetia; Corynebacteriales; Mycobacteriaceae; Mycobacterium; Mycobacterium tuberculosis complex; Mycobacterium tuberculosis;	NCBI: 1455274	4367407	99.94	0.00	Yes
NCBI Assembly	GCA_000655055.1	NCBI: cellular organisms; Bacteria; Terrabacteria group; Actinobacteria; Actinomycetia; Corynebacteriales; Mycobacteriaceae; Mycobacterium; Mycobacterium tuberculosis complex; Mycobacterium tuberculosis;	NCBI: 1448653	4439094	99.94	0.00	Yes
NCBI Assembly	GCA_000654435.1	NCBI: cellular organisms; Bacteria; Terrabacteria group; Actinobacteria; Actinomycetia; Corynebacteriales; Mycobacteriaceae; Mycobacterium; Mycobacterium tuberculosis complex; Mycobacterium tuberculosis;	NCBI: 1448620	4436849	99.94	0.00	Yes
NCBI Assembly	GCA_000654715.1	NCBI: cellular organisms; Bacteria; Terrabacteria group; Actinobacteria; Actinomycetia; Corynebacteriales; Mycobacteriaceae; Mycobacterium; Mycobacterium tuberculosis complex; Mycobacterium tuberculosis;	NCBI: 1448636	4417789	99.94	0.00	Yes
NCBI Assembly	GCA_000445895.1	NCBI: cellular organisms; Bacteria; Terrabacteria group; Actinobacteria; Actinomycetia; Corynebacteriales; Mycobacteriaceae; Mycobacterium; Mycobacterium tuberculosis complex; Mycobacterium tuberculosis;	NCBI: 1352596	4400052	99.94	0.00	Yes
NCBI Assembly	GCA_000445535.1	NCBI: cellular organisms; Bacteria; Terrabacteria group; Actinobacteria; Actinomycetia; Corynebacteriales; Mycobacteriaceae; Mycobacterium; Mycobacterium tuberculosis complex; Mycobacterium tuberculosis;	NCBI: 1352578	4408738	99.94	0.00	Yes
NCBI Assembly	GCA_000455325.1	NCBI: cellular organisms; Bacteria; Terrabacteria group; Actinobacteria; Actinomycetia; Corynebacteriales; Mycobacteriaceae; Mycolicibacterium; Mycolicibacterium septicum;	NCBI: 1341646	6908835	98.58	0.55	Yes

NCBI Assembly	GCA_000652895.1	NCBI: cellular organisms; Bacteria; Terrabacteria group; Actinobacteria; Actinomycetia; Corynebacteriales; Mycobacteriaceae; Mycobacterium; Mycobacterium tuberculosis complex; Mycobacterium tuberculosis;	NCBI: 1448491	4448489	99.94	1.07	Yes
NCBI Assembly	GCA_000653195.1	NCBI: cellular organisms; Bacteria; Terrabacteria group; Actinobacteria; Actinomycetia; Corynebacteriales; Mycobacteriaceae; Mycobacterium; Mycobacterium tuberculosis complex; Mycobacterium tuberculosis;	NCBI: 1448512	4412106	99.94	0.00	Yes
NCBI Assembly	GCA_000653515.1	NCBI: cellular organisms; Bacteria; Terrabacteria group; Actinobacteria; Actinomycetia; Corynebacteriales; Mycobacteriaceae; Mycobacterium; Mycobacterium tuberculosis complex; Mycobacterium tuberculosis;	NCBI: 1448535	4408190	99.94	0.33	Yes
NCBI Assembly	GCA_000443815.1	NCBI: cellular organisms; Bacteria; Terrabacteria group; Actinobacteria; Actinomycetia; Corynebacteriales; Mycobacteriaceae; Mycobacterium; Mycobacterium tuberculosis complex; Mycobacterium tuberculosis;	NCBI: 1346783	4391527	99.94	0.00	Yes
NCBI Assembly	GCA_000657275.1	NCBI: cellular organisms; Bacteria; Terrabacteria group; Actinobacteria; Actinomycetia; Corynebacteriales; Mycobacteriaceae; Mycobacterium; Mycobacterium tuberculosis complex; Mycobacterium tuberculosis;	NCBI: 1455275	4351512	99.94	0.33	Yes
NCBI Assembly	GCA_000445915.1	NCBI: cellular organisms; Bacteria; Terrabacteria group; Actinobacteria; Actinomycetia; Corynebacteriales; Mycobacteriaceae; Mycobacterium; Mycobacterium tuberculosis complex; Mycobacterium tuberculosis;	NCBI: 1352597	4408002	99.94	0.00	Yes
NCBI Assembly	GCA_000654735.1	NCBI: cellular organisms; Bacteria; Terrabacteria group; Actinobacteria; Actinomycetia; Corynebacteriales; Mycobacteriaceae; Mycobacterium; Mycobacterium tuberculosis complex; Mycobacterium tuberculosis;	NCBI: 1448637	4424314	99.94	0.00	Yes
NCBI Assembly	GCA_000655075.1	NCBI: cellular organisms; Bacteria; Terrabacteria group; Actinobacteria; Actinomycetia; Corynebacteriales; Mycobacteriaceae; Mycobacterium; Mycobacterium tuberculosis complex; Mycobacterium tuberculosis;	NCBI: 1448654	4424924	99.94	0.00	Yes
NCBI Assembly	GCA_000445555.1	NCBI: cellular organisms; Bacteria; Terrabacteria group; Actinobacteria; Actinomycetia; Corynebacteriales; Mycobacteriaceae; Mycobacterium; Mycobacterium tuberculosis complex; Mycobacterium tuberculosis;	NCBI: 1352579	4405199	99.94	0.00	Yes
NCBI Assembly	GCA_000654455.1	NCBI: cellular organisms; Bacteria; Terrabacteria group; Actinobacteria; Actinomycetia; Corynebacteriales; Mycobacteriaceae; Mycobacterium; Mycobacterium tuberculosis complex; Mycobacterium tuberculosis;	NCBI: 1448621	4434189	99.94	0.00	Yes
NCBI Assembly	GCA_000465305.1	NCBI: cellular organisms; Bacteria; Terrabacteria group; Actinobacteria; Actinomycetia; Corynebacteriales; Mycobacteriaceae; Mycobacterium; Mycobacterium tuberculosis complex; Mycobacterium tuberculosis;	NCBI: 1348776	4381942	99.91	0.00	Yes
NCBI Assembly	GCA_000443835.1	NCBI: cellular organisms; Bacteria; Terrabacteria group; Actinobacteria; Actinomycetia; Corynebacteriales; Mycobacteriaceae; Mycobacterium; Mycobacterium tuberculosis complex; Mycobacterium tuberculosis;	NCBI: 1346784	4403994	99.94	0.00	Yes
NCBI Assembly	GCA_000652915.1	NCBI: cellular organisms; Bacteria; Terrabacteria group; Actinobacteria; Actinomycetia; Corynebacteriales; Mycobacteriaceae; Mycobacterium; Mycobacterium tuberculosis complex; Mycobacterium tuberculosis;	NCBI: 1448492	4438638	99.94	0.00	Yes
NCBI Assembly	GCA_000653215.1	NCBI: cellular organisms; Bacteria; Terrabacteria group; Actinobacteria; Actinomycetia; Corynebacteriales; Mycobacteriaceae; Mycobacterium; Mycobacterium tuberculosis complex; Mycobacterium tuberculosis;	NCBI: 1448513	4390505	99.89	0.00	Yes
NCBI Assembly	GCA_000653535.1	NCBI: cellular organisms; Bacteria; Terrabacteria group; Actinobacteria; Actinomycetia; Corynebacteriales; Mycobacteriaceae; Mycobacterium; Mycobacterium tuberculosis complex; Mycobacterium tuberculosis;	NCBI: 1448536	4387484	99.94	0.00	Yes
NCBI Assembly	GCA_000657295.1	NCBI: cellular organisms; Bacteria; Terrabacteria group; Actinobacteria; Actinomycetia; Corynebacteriales; Mycobacteriaceae; Mycobacterium; Mycobacterium tuberculosis complex; Mycobacterium tuberculosis;	NCBI: 1455276	4394025	99.94	0.29	Yes
NCBI Assembly	GCA_000445935.1	NCBI: cellular organisms; Bacteria; Terrabacteria group; Actinobacteria; Actinomycetia; Corynebacteriales; Mycobacteriaceae; Mycobacterium; Mycobacterium tuberculosis complex; Mycobacterium tuberculosis;	NCBI: 1352599	4398360	99.28	0.00	Yes
NCBI Assembly	GCA_000445575.1	NCBI: cellular organisms; Bacteria; Terrabacteria group; Actinobacteria; Actinomycetia; Corynebacteriales; Mycobacteriaceae; Mycobacterium; Mycobacterium tuberculosis complex; Mycobacterium tuberculosis;	NCBI: 1352580	4405944	99.94	0.00	Yes

NCBI Assembly	GCA_000654755.1	NCBI: cellular organisms; Bacteria; Terrabacteria group; Actinobacteria; Actinomycetia; Corynebacteriales; Mycobacteriaceae; Mycobacterium; Mycobacterium tuberculosis complex; Mycobacterium tuberculosis;	NCBI: 1448638	4422915	99.94	0.00	Yes
NCBI Assembly	GCA_000654475.1	NCBI: cellular organisms; Bacteria; Terrabacteria group; Actinobacteria; Actinomycetia; Corynebacteriales; Mycobacteriaceae; Mycobacterium; Mycobacterium tuberculosis complex; Mycobacterium tuberculosis;	NCBI: 1448622	4442128	99.94	0.90	Yes
NCBI Assembly	GCA_000655095.1	NCBI: cellular organisms; Bacteria; Terrabacteria group; Actinobacteria; Actinomycetia; Corynebacteriales; Mycobacteriaceae; Mycobacterium; Mycobacterium tuberculosis complex; Mycobacterium tuberculosis;	NCBI: 1427259	4422817	99.94	0.00	Yes
NCBI Assembly	GCA_000443855.1	NCBI: cellular organisms; Bacteria; Terrabacteria group; Actinobacteria; Actinomycetia; Corynebacteriales; Mycobacteriaceae; Mycobacterium; Mycobacterium tuberculosis complex; Mycobacterium tuberculosis;	NCBI: 1346785	4402981	99.94	0.00	Yes
NCBI Assembly	GCA_000466125.2	NCBI: cellular organisms; Bacteria; Terrabacteria group; Actinobacteria; Actinomycetia; Corynebacteriales; Mycobacteriaceae; Mycobacterium; Mycobacterium tuberculosis complex; Mycobacterium tuberculosis;	NCBI: 1370123	4267020	96.14	0.08	Yes
NCBI Assembly	GCA_000652935.1	NCBI: cellular organisms; Bacteria; Terrabacteria group; Actinobacteria; Actinomycetia; Corynebacteriales; Mycobacteriaceae; Mycobacterium; Mycobacterium tuberculosis complex; Mycobacterium tuberculosis;	NCBI: 1448493	4402819	99.94	0.33	Yes
NCBI Assembly	GCA_000653235.1	NCBI: cellular organisms; Bacteria; Terrabacteria group; Actinobacteria; Actinomycetia; Corynebacteriales; Mycobacteriaceae; Mycobacterium; Mycobacterium tuberculosis complex; Mycobacterium tuberculosis;	NCBI: 1448514	4426352	99.94	0.00	Yes
NCBI Assembly	GCA_000653555.1	NCBI: cellular organisms; Bacteria; Terrabacteria group; Actinobacteria; Actinomycetia; Corynebacteriales; Mycobacteriaceae; Mycobacterium; Mycobacterium tuberculosis complex; Mycobacterium tuberculosis;	NCBI: 1448538	4449752	99.94	0.50	Yes
NCBI Assembly	GCA_000445955.1	NCBI: cellular organisms; Bacteria; Terrabacteria group; Actinobacteria; Actinomycetia; Corynebacteriales; Mycobacteriaceae; Mycobacterium; Mycobacterium tuberculosis complex; Mycobacterium tuberculosis;	NCBI: 1352600	4369690	99.94	0.00	Yes
NCBI Assembly	GCA_000657315.1	NCBI: cellular organisms; Bacteria; Terrabacteria group; Actinobacteria; Actinomycetia; Corynebacteriales; Mycobacteriaceae; Mycobacterium; Mycobacterium tuberculosis complex; Mycobacterium tuberculosis;	NCBI: 1455278	4417270	99.94	0.33	Yes
NCBI Assembly	GCA_000445595.1	NCBI: cellular organisms; Bacteria; Terrabacteria group; Actinobacteria; Actinomycetia; Corynebacteriales; Mycobacteriaceae; Mycobacterium; Mycobacterium tuberculosis complex; Mycobacterium tuberculosis;	NCBI: 1352581	4402491	99.94	0.00	Yes
NCBI Assembly	GCA_000655115.1	NCBI: cellular organisms; Bacteria; Terrabacteria group; Actinobacteria; Actinomycetia; Corynebacteriales; Mycobacteriaceae; Mycobacterium; Mycobacterium tuberculosis complex; Mycobacterium tuberculosis;	NCBI: 1427260	4418361	99.94	0.00	Yes
NCBI Assembly	GCA_000654775.1	NCBI: cellular organisms; Bacteria; Terrabacteria group; Actinobacteria; Actinomycetia; Corynebacteriales; Mycobacteriaceae; Mycobacterium; Mycobacterium tuberculosis complex; Mycobacterium tuberculosis;	NCBI: 1448639	4419760	99.94	0.00	Yes
NCBI Assembly	GCA_000654495.1	NCBI: cellular organisms; Bacteria; Terrabacteria group; Actinobacteria; Actinomycetia; Corynebacteriales; Mycobacteriaceae; Mycobacterium; Mycobacterium tuberculosis complex; Mycobacterium tuberculosis;	NCBI: 1448623	4410894	99.94	0.00	Yes
NCBI Assembly	GCA_000443875.1	NCBI: cellular organisms; Bacteria; Terrabacteria group; Actinobacteria; Actinomycetia; Corynebacteriales; Mycobacteriaceae; Mycobacterium; Mycobacterium tuberculosis complex; Mycobacterium tuberculosis;	NCBI: 1346787	4397923	99.53	0.00	Yes
NCBI Assembly	GCA_000466145.1	NCBI: cellular organisms; Bacteria; Terrabacteria group; Actinobacteria; Actinomycetia; Corynebacteriales; Mycobacteriaceae; Mycobacterium; Mycobacterium tuberculosis complex; Mycobacterium tuberculosis;	NCBI: 1379257	4227747	96.02	0.00	Yes
NCBI Assembly	GCA_000653575.1	NCBI: cellular organisms; Bacteria; Terrabacteria group; Actinobacteria; Actinomycetia; Corynebacteriales; Mycobacteriaceae; Mycobacterium; Mycobacterium tuberculosis complex; Mycobacterium tuberculosis;	NCBI: 1448540	4388740	99.94	0.00	Yes
NCBI Assembly	GCA_000653255.1	NCBI: cellular organisms; Bacteria; Terrabacteria group; Actinobacteria; Actinomycetia; Corynebacteriales; Mycobacteriaceae; Mycobacterium; Mycobacterium tuberculosis complex; Mycobacterium tuberculosis;	NCBI: 1448517	4394361	99.94	0.00	Yes

NCBI Assembly	GCA_000652955.1	NCBI: cellular organisms; Bacteria; Terrabacteria group; Actinobacteria; Actinomycetia; Corynebacteriales; Mycobacteriaceae; Mycobacterium; Mycobacterium tuberculosis complex; Mycobacterium tuberculosis;	NCBI: 1448495	4409632	99.94	0.00	Yes
NCBI Assembly	GCA_000445975.1	NCBI: cellular organisms; Bacteria; Terrabacteria group; Actinobacteria; Actinomycetia; Corynebacteriales; Mycobacteriaceae; Mycobacterium; Mycobacterium tuberculosis complex; Mycobacterium tuberculosis;	NCBI: 1352598	4409116	99.94	0.00	Yes
NCBI Assembly	GCA_000445615.1	NCBI: cellular organisms; Bacteria; Terrabacteria group; Actinobacteria; Actinomycetia; Corynebacteriales; Mycobacteriaceae; Mycobacterium; Mycobacterium tuberculosis complex; Mycobacterium tuberculosis;	NCBI: 1352582	4398105	99.94	0.00	Yes
NCBI Assembly	GCA_000657335.1	NCBI: cellular organisms; Bacteria; Terrabacteria group; Actinobacteria; Actinomycetia; Corynebacteriales; Mycobacteriaceae; Mycobacterium; Mycobacterium tuberculosis complex; Mycobacterium tuberculosis;	NCBI: 1455279	4444114	99.94	0.00	Yes
NCBI Assembly	GCA_000654795.1	NCBI: cellular organisms; Bacteria; Terrabacteria group; Actinobacteria; Actinomycetia; Corynebacteriales; Mycobacteriaceae; Mycobacterium; Mycobacterium tuberculosis complex; Mycobacterium tuberculosis;	NCBI: 1448640	4417779	99.94	0.00	Yes
NCBI Assembly	GCA_000443895.1	NCBI: cellular organisms; Bacteria; Terrabacteria group; Actinobacteria; Actinomycetia; Corynebacteriales; Mycobacteriaceae; Mycobacterium; Mycobacterium tuberculosis complex; Mycobacterium tuberculosis;	NCBI: 1346788	4395537	99.94	0.00	Yes
NCBI Assembly	GCA_000655135.1	NCBI: cellular organisms; Bacteria; Terrabacteria group; Actinobacteria; Actinomycetia; Corynebacteriales; Mycobacteriaceae; Mycobacterium; Mycobacterium tuberculosis complex; Mycobacterium tuberculosis;	NCBI: 1427261	4389692	99.94	0.00	Yes
NCBI Assembly	GCA_000654515.1	NCBI: cellular organisms; Bacteria; Terrabacteria group; Actinobacteria; Actinomycetia; Corynebacteriales; Mycobacteriaceae; Mycobacterium; Mycobacterium tuberculosis complex; Mycobacterium tuberculosis;	NCBI: 1448624	4441365	99.94	0.00	Yes
NCBI Assembly	GCA_000474595.1	NCBI: cellular organisms; Bacteria; Terrabacteria group; Actinobacteria; Actinomycetia; Corynebacteriales; Mycobacteriaceae; Mycobacterium; Mycobacterium tuberculosis complex; Mycobacterium tuberculosis;	NCBI: 1370124	4206725	96.02	0.33	Yes
NCBI Assembly	GCA_000653275.1	NCBI: cellular organisms; Bacteria; Terrabacteria group; Actinobacteria; Actinomycetia; Corynebacteriales; Mycobacteriaceae; Mycobacterium; Mycobacterium tuberculosis complex; Mycobacterium tuberculosis;	NCBI: 1448520	4409336	99.94	0.00	Yes
NCBI Assembly	GCA_000652975.1	NCBI: cellular organisms; Bacteria; Terrabacteria group; Actinobacteria; Actinomycetia; Corynebacteriales; Mycobacteriaceae; Mycobacterium; Mycobacterium tuberculosis complex; Mycobacterium tuberculosis;	NCBI: 1448496	4390363	99.94	0.00	Yes
NCBI Assembly	GCA_000653595.1	NCBI: cellular organisms; Bacteria; Terrabacteria group; Actinobacteria; Actinomycetia; Corynebacteriales; Mycobacteriaceae; Mycobacterium; Mycobacterium tuberculosis complex; Mycobacterium tuberculosis;	NCBI: 1448542	4413087	99.94	0.48	Yes
NCBI Assembly	GCA_000653995.1	NCBI: cellular organisms; Bacteria; Terrabacteria group; Actinobacteria; Actinomycetia; Corynebacteriales; Mycobacteriaceae; Mycobacterium; Mycobacterium tuberculosis complex; Mycobacterium tuberculosis;	NCBI: 1448568	4405889	99.94	0.00	Yes
NCBI Assembly	GCA_000445635.1	NCBI: cellular organisms; Bacteria; Terrabacteria group; Actinobacteria; Actinomycetia; Corynebacteriales; Mycobacteriaceae; Mycobacterium; Mycobacterium tuberculosis complex; Mycobacterium tuberculosis;	NCBI: 1352583	4406983	99.94	0.00	Yes
NCBI Assembly	GCA_000443915.1	NCBI: cellular organisms; Bacteria; Terrabacteria group; Actinobacteria; Actinomycetia; Corynebacteriales; Mycobacteriaceae; Mycobacterium; Mycobacterium tuberculosis complex; Mycobacterium tuberculosis;	NCBI: 1346786	4386956	99.94	0.00	Yes
NCBI Assembly	GCA_000657355.1	NCBI: cellular organisms; Bacteria; Terrabacteria group; Actinobacteria; Actinomycetia; Corynebacteriales; Mycobacteriaceae; Mycobacterium; Mycobacterium tuberculosis complex; Mycobacterium tuberculosis;	NCBI: 1455283	4362135	99.90	0.48	Yes
NCBI Assembly	GCA_000654815.1	NCBI: cellular organisms; Bacteria; Terrabacteria group; Actinobacteria; Actinomycetia; Corynebacteriales; Mycobacteriaceae; Mycobacterium; Mycobacterium tuberculosis complex; Mycobacterium tuberculosis;	NCBI: 1448641	4440290	99.94	0.00	Yes
NCBI Assembly	GCA_000654535.1	NCBI: cellular organisms; Bacteria; Terrabacteria group; Actinobacteria; Actinomycetia; Corynebacteriales; Mycobacteriaceae; Mycobacterium; Mycobacterium tuberculosis complex; Mycobacterium tuberculosis;	NCBI: 1448625	4431000	99.94	0.00	Yes

NCBI Assembly	GCA_000655155.1	NCBI: cellular organisms; Bacteria; Terrabacteria group; Actinobacteria; Actinomycetia; Corynebacteriales; Mycobacteriaceae; Mycobacterium; Mycobacterium tuberculosis complex; Mycobacterium tuberculosis;	NCBI: 1427262	4390681	99.94	0.00	Yes
NCBI Assembly	GCA_000488915.1	NCBI: cellular organisms; Bacteria; Terrabacteria group; Actinobacteria; Actinomycetia; Corynebacteriales; Mycobacteriaceae; Mycobacterium; Mycobacterium tuberculosis complex; Mycobacterium tuberculosis; Mycobacterium tuberculosis variant bovis;	NCBI: 1384084	4313075	99.78	0.00	Yes
NCBI Assembly	GCA_000653295.1	NCBI: cellular organisms; Bacteria; Terrabacteria group; Actinobacteria; Actinomycetia; Corynebacteriales; Mycobacteriaceae; Mycobacterium; Mycobacterium tuberculosis complex; Mycobacterium tuberculosis;	NCBI: 1448521	4405213	99.94	0.21	Yes
NCBI Assembly	GCA_000652995.1	NCBI: cellular organisms; Bacteria; Terrabacteria group; Actinobacteria; Actinomycetia; Corynebacteriales; Mycobacteriaceae; Mycobacterium; Mycobacterium tuberculosis complex; Mycobacterium tuberculosis;	NCBI: 1448497	4404229	99.94	0.00	Yes
NCBI Assembly	GCA_000653615.1	NCBI: cellular organisms; Bacteria; Terrabacteria group; Actinobacteria; Actinomycetia; Corynebacteriales; Mycobacteriaceae; Mycobacterium; Mycobacterium tuberculosis complex; Mycobacterium tuberculosis;	NCBI: 1448544	4412199	99.94	0.33	Yes
NCBI Assembly	GCA_000654015.1	NCBI: cellular organisms; Bacteria; Terrabacteria group; Actinobacteria; Actinomycetia; Corynebacteriales; Mycobacteriaceae; Mycobacterium; Mycobacterium tuberculosis complex; Mycobacterium tuberculosis;	NCBI: 1448569	4403111	99.94	0.61	Yes
NCBI Assembly	GCA_000445655.1	NCBI: cellular organisms; Bacteria; Terrabacteria group; Actinobacteria; Actinomycetia; Corynebacteriales; Mycobacteriaceae; Mycobacterium; Mycobacterium tuberculosis complex; Mycobacterium tuberculosis;	NCBI: 1352584	4398277	99.94	0.00	Yes
NCBI Assembly	GCA_000654555.1	NCBI: cellular organisms; Bacteria; Terrabacteria group; Actinobacteria; Actinomycetia; Corynebacteriales; Mycobacteriaceae; Mycobacterium; Mycobacterium tuberculosis complex; Mycobacterium tuberculosis;	NCBI: 1448626	4425452	99.94	0.00	Yes
NCBI Assembly	GCA_000656795.1	NCBI: cellular organisms; Bacteria; Terrabacteria group; Actinobacteria; Actinomycetia; Corynebacteriales; Mycobacteriaceae; Mycobacterium; Mycobacterium tuberculosis complex; Mycobacterium tuberculosis;	NCBI: 1448592	4400961	99.94	0.00	Yes
NCBI Assembly	GCA_000654835.1	NCBI: cellular organisms; Bacteria; Terrabacteria group; Actinobacteria; Actinomycetia; Corynebacteriales; Mycobacteriaceae; Mycobacterium; Mycobacterium tuberculosis complex; Mycobacterium tuberculosis;	NCBI: 1448642	4430240	99.94	0.00	Yes
NCBI Assembly	GCA_000657375.1	NCBI: cellular organisms; Bacteria; Terrabacteria group; Actinobacteria; Actinomycetia; Corynebacteriales; Mycobacteriaceae; Mycobacterium; Mycobacterium tuberculosis complex; Mycobacterium tuberculosis;	NCBI: 1455284	4387534	99.94	0.00	Yes
NCBI Assembly	GCA_000655175.1	NCBI: cellular organisms; Bacteria; Terrabacteria group; Actinobacteria; Actinomycetia; Corynebacteriales; Mycobacteriaceae; Mycobacterium; Mycobacterium tuberculosis complex; Mycobacterium tuberculosis;	NCBI: 1427264	4431177	99.94	0.00	Yes
NCBI Assembly	GCA_000488935.1	NCBI: cellular organisms; Bacteria; Terrabacteria group; Actinobacteria; Actinomycetia; Corynebacteriales; Mycobacteriaceae; Mycobacterium; Mycobacterium tuberculosis complex; Mycobacterium tuberculosis; Mycobacterium tuberculosis variant bovis;	NCBI: 1389959	4325667	99.78	0.00	Yes
NCBI Assembly	GCA_000653315.1	NCBI: cellular organisms; Bacteria; Terrabacteria group; Actinobacteria; Actinomycetia; Corynebacteriales; Mycobacteriaceae; Mycobacterium; Mycobacterium tuberculosis complex; Mycobacterium tuberculosis;	NCBI: 1448522	4401230	99.94	0.00	Yes
NCBI Assembly	GCA_000653015.1	NCBI: cellular organisms; Bacteria; Terrabacteria group; Actinobacteria; Actinomycetia; Corynebacteriales; Mycobacteriaceae; Mycobacterium; Mycobacterium tuberculosis complex; Mycobacterium tuberculosis;	NCBI: 1448498	4410268	99.94	0.33	Yes
NCBI Assembly	GCA_000653635.1	NCBI: cellular organisms; Bacteria; Terrabacteria group; Actinobacteria; Actinomycetia; Corynebacteriales; Mycobacteriaceae; Mycobacterium; Mycobacterium tuberculosis complex; Mycobacterium tuberculosis;	NCBI: 1448545	4401281	99.94	0.33	Yes
NCBI Assembly	GCA_000654035.1	NCBI: cellular organisms; Bacteria; Terrabacteria group; Actinobacteria; Actinomycetia; Corynebacteriales; Mycobacteriaceae; Mycobacterium; Mycobacterium tuberculosis complex; Mycobacterium tuberculosis;	NCBI: 1448570	4402087	99.94	0.00	Yes
NCBI Assembly	GCA_000656495.1	NCBI: cellular organisms; Bacteria; Terrabacteria group; Actinobacteria; Actinomycetia; Corynebacteriales; Mycobacteriaceae; Mycobacterium; Mycobacterium tuberculosis complex; Mycobacterium tuberculosis;	NCBI: 1438887	4371397	99.94	0.19	Yes

NCBI Assembly	GCA_000656815.1	NCBI: cellular organisms; Bacteria; Terrabacteria group; Actinobacteria; Actinomycetia; Corynebacteriales; Mycobacteriaceae; Mycobacterium; Mycobacterium tuberculosis complex; Mycobacterium tuberculosis;	NCBI: 1448593	4390509	99.94	0.00	Yes
NCBI Assembly	GCA_000654855.1	NCBI: cellular organisms; Bacteria; Terrabacteria group; Actinobacteria; Actinomycetia; Corynebacteriales; Mycobacteriaceae; Mycobacterium; Mycobacterium tuberculosis complex; Mycobacterium tuberculosis;	NCBI: 1448643	4440819	99.94	0.00	Yes
NCBI Assembly	GCA_000654575.1	NCBI: cellular organisms; Bacteria; Terrabacteria group; Actinobacteria; Actinomycetia; Corynebacteriales; Mycobacteriaceae; Mycobacterium; Mycobacterium tuberculosis complex; Mycobacterium tuberculosis;	NCBI: 1448627	4434943	99.94	0.00	Yes
NCBI Assembly	GCA_000657395.1	NCBI: cellular organisms; Bacteria; Terrabacteria group; Actinobacteria; Actinomycetia; Corynebacteriales; Mycobacteriaceae; Mycobacterium; Mycobacterium tuberculosis complex; Mycobacterium tuberculosis;	NCBI: 1455285	4381692	99.94	0.00	Yes
NCBI Assembly	GCA_000655195.1	NCBI: cellular organisms; Bacteria; Terrabacteria group; Actinobacteria; Actinomycetia; Corynebacteriales; Mycobacteriaceae; Mycobacterium; Mycobacterium tuberculosis complex; Mycobacterium tuberculosis;	NCBI: 1427265	4441862	99.94	0.00	Yes
NCBI Assembly	GCA_000497265.2	NCBI: cellular organisms; Bacteria; Terrabacteria group; Actinobacteria; Actinomycetia; Corynebacteriales; Mycobacteriaceae; Mycobacteroides; Mycobacteroides abscessus; Mycobacteroides abscessus subsp. massiliense;	NCBI: 1001714	4978382	100.00	0.08	Yes
NCBI Assembly	GCA_000653335.1	NCBI: cellular organisms; Bacteria; Terrabacteria group; Actinobacteria; Actinomycetia; Corynebacteriales; Mycobacteriaceae; Mycobacterium; Mycobacterium tuberculosis complex; Mycobacterium tuberculosis;	NCBI: 1448523	4406513	99.94	0.00	Yes
NCBI Assembly	GCA_000653035.1	NCBI: cellular organisms; Bacteria; Terrabacteria group; Actinobacteria; Actinomycetia; Corynebacteriales; Mycobacteriaceae; Mycobacterium; Mycobacterium tuberculosis complex; Mycobacterium tuberculosis;	NCBI: 1448501	4414412	99.94	0.54	Yes
NCBI Assembly	GCA_000654055.1	NCBI: cellular organisms; Bacteria; Terrabacteria group; Actinobacteria; Actinomycetia; Corynebacteriales; Mycobacteriaceae; Mycobacterium; Mycobacterium tuberculosis complex; Mycobacterium tuberculosis;	NCBI: 1448571	4401459	99.94	0.00	Yes
NCBI Assembly	GCA_000653655.1	NCBI: cellular organisms; Bacteria; Terrabacteria group; Actinobacteria; Actinomycetia; Corynebacteriales; Mycobacteriaceae; Mycobacterium; Mycobacterium tuberculosis complex; Mycobacterium tuberculosis;	NCBI: 1448546	4399466	99.94	0.00	Yes
NCBI Assembly	GCA_000654875.1	NCBI: cellular organisms; Bacteria; Terrabacteria group; Actinobacteria; Actinomycetia; Corynebacteriales; Mycobacteriaceae; Mycobacterium; Mycobacterium tuberculosis complex; Mycobacterium tuberculosis;	NCBI: 1448644	4410968	99.94	0.00	Yes
NCBI Assembly	GCA_000656515.1	NCBI: cellular organisms; Bacteria; Terrabacteria group; Actinobacteria; Actinomycetia; Corynebacteriales; Mycobacteriaceae; Mycobacterium; Mycobacterium tuberculosis complex; Mycobacterium tuberculosis;	NCBI: 1438836	4422257	99.94	0.00	Yes
NCBI Assembly	GCA_000657415.1	NCBI: cellular organisms; Bacteria; Terrabacteria group; Actinobacteria; Actinomycetia; Corynebacteriales; Mycobacteriaceae; Mycobacterium; Mycobacterium tuberculosis complex; Mycobacterium tuberculosis;	NCBI: 1455286	4303445	98.71	0.69	Yes
NCBI Assembly	GCA_000656835.1	NCBI: cellular organisms; Bacteria; Terrabacteria group; Actinobacteria; Actinomycetia; Corynebacteriales; Mycobacteriaceae; Mycobacterium; Mycobacterium tuberculosis complex; Mycobacterium tuberculosis;	NCBI: 1448594	4392293	99.94	0.00	Yes
NCBI Assembly	GCA_000654595.1	NCBI: cellular organisms; Bacteria; Terrabacteria group; Actinobacteria; Actinomycetia; Corynebacteriales; Mycobacteriaceae; Mycobacterium; Mycobacterium tuberculosis complex; Mycobacterium tuberculosis;	NCBI: 1448628	4408183	99.94	0.00	Yes
NCBI Assembly	GCA_000655215.1	NCBI: cellular organisms; Bacteria; Terrabacteria group; Actinobacteria; Actinomycetia; Corynebacteriales; Mycobacteriaceae; Mycobacterium; Mycobacterium tuberculosis complex; Mycobacterium tuberculosis;	NCBI: 1427266	4390507	99.94	0.00	Yes
NCBI Assembly	GCA_000657435.1	NCBI: cellular organisms; Bacteria; Terrabacteria group; Actinobacteria; Actinomycetia; Corynebacteriales; Mycobacteriaceae; Mycobacterium; Mycobacterium tuberculosis complex; Mycobacterium tuberculosis;	NCBI: 1455287	4396620	99.94	0.00	Yes
NCBI Assembly	GCA_000653355.1	NCBI: cellular organisms; Bacteria; Terrabacteria group; Actinobacteria; Actinomycetia; Corynebacteriales; Mycobacteriaceae; Mycobacterium; Mycobacterium tuberculosis complex; Mycobacterium tuberculosis;	NCBI: 1448524	4394915	99.88	0.26	Yes

NCBI Assembly	GCA_000654075.1	NCBI: cellular organisms; Bacteria; Terrabacteria group; Actinobacteria; Actinomycetia; Corynebacteriales; Mycobacteriaceae; Mycobacterium; Mycobacterium tuberculosis complex; Mycobacterium tuberculosis;	NCBI: 1448572	4388355	99.94	0.00	Yes
NCBI Assembly	GCA_000653675.1	NCBI: cellular organisms; Bacteria; Terrabacteria group; Actinobacteria; Actinomycetia; Corynebacteriales; Mycobacteriaceae; Mycobacterium; Mycobacterium tuberculosis complex; Mycobacterium tuberculosis;	NCBI: 1448547	4422338	99.94	0.36	Yes
NCBI Assembly	GCA_000656535.1	NCBI: cellular organisms; Bacteria; Terrabacteria group; Actinobacteria; Actinomycetia; Corynebacteriales; Mycobacteriaceae; Mycobacterium; Mycobacterium tuberculosis complex; Mycobacterium tuberculosis;	NCBI: 1438832	4401387	99.94	0.50	Yes
NCBI Assembly	GCA_000656855.1	NCBI: cellular organisms; Bacteria; Terrabacteria group; Actinobacteria; Actinomycetia; Corynebacteriales; Mycobacteriaceae; Mycobacterium; Mycobacterium tuberculosis complex; Mycobacterium tuberculosis;	NCBI: 1448595	4402031	99.94	0.00	Yes
NCBI Assembly	GCA_000655235.1	NCBI: cellular organisms; Bacteria; Terrabacteria group; Actinobacteria; Actinomycetia; Corynebacteriales; Mycobacteriaceae; Mycobacterium; Mycobacterium tuberculosis complex; Mycobacterium tuberculosis;	NCBI: 1427268	4397663	99.94	0.00	Yes
NCBI Assembly	GCA_000654895.1	NCBI: cellular organisms; Bacteria; Terrabacteria group; Actinobacteria; Actinomycetia; Corynebacteriales; Mycobacteriaceae; Mycobacterium; Mycobacterium tuberculosis complex; Mycobacterium tuberculosis;	NCBI: 1448645	4417490	99.94	0.00	Yes
NCBI Assembly	GCA_000657455.1	NCBI: cellular organisms; Bacteria; Terrabacteria group; Actinobacteria; Actinomycetia; Corynebacteriales; Mycobacteriaceae; Mycobacterium; Mycobacterium tuberculosis complex; Mycobacterium tuberculosis;	NCBI: 1455288	4412366	99.94	0.00	Yes
NCBI Assembly	GCA_000220415.1	NCBI: cellular organisms; Bacteria; Terrabacteria group; Actinobacteria; Actinomycetia; Corynebacteriales; Mycobacteriaceae; Mycobacterium; Mycobacterium tuberculosis complex; Mycobacterium tuberculosis;	NCBI: 882098	4342996	97.44	0.00	Yes
NCBI Assembly	GCA_000654095.1	NCBI: cellular organisms; Bacteria; Terrabacteria group; Actinobacteria; Actinomycetia; Corynebacteriales; Mycobacteriaceae; Mycobacterium; Mycobacterium tuberculosis complex; Mycobacterium tuberculosis;	NCBI: 1448574	4400252	99.94	0.00	Yes
NCBI Assembly	GCA_000653695.1	NCBI: cellular organisms; Bacteria; Terrabacteria group; Actinobacteria; Actinomycetia; Corynebacteriales; Mycobacteriaceae; Mycobacterium; Mycobacterium tuberculosis complex; Mycobacterium tuberculosis;	NCBI: 1448548	4387886	99.94	0.00	Yes
NCBI Assembly	GCA_000656555.1	NCBI: cellular organisms; Bacteria; Terrabacteria group; Actinobacteria; Actinomycetia; Corynebacteriales; Mycobacteriaceae; Mycobacterium; Mycobacterium tuberculosis complex; Mycobacterium tuberculosis;	NCBI: 1438834	4419920	99.90	0.03	Yes
NCBI Assembly	GCA_000656875.1	NCBI: cellular organisms; Bacteria; Terrabacteria group; Actinobacteria; Actinomycetia; Corynebacteriales; Mycobacteriaceae; Mycobacterium; Mycobacterium tuberculosis complex; Mycobacterium tuberculosis;	NCBI: 1448596	4421313	99.94	0.00	Yes
NCBI Assembly	GCA_000654915.1	NCBI: cellular organisms; Bacteria; Terrabacteria group; Actinobacteria; Actinomycetia; Corynebacteriales; Mycobacteriaceae; Mycobacterium; Mycobacterium tuberculosis complex; Mycobacterium tuberculosis;	NCBI: 1448646	4430497	99.94	0.00	Yes
NCBI Assembly	GCA_000657475.1	NCBI: cellular organisms; Bacteria; Terrabacteria group; Actinobacteria; Actinomycetia; Corynebacteriales; Mycobacteriaceae; Mycobacterium; Mycobacterium tuberculosis complex; Mycobacterium tuberculosis;	NCBI: 1455289	4388363	99.94	0.00	Yes
NCBI Assembly	GCA_000220435.1	NCBI: cellular organisms; Bacteria; Terrabacteria group; Actinobacteria; Actinomycetia; Corynebacteriales; Mycobacteriaceae; Mycobacterium; Mycobacterium tuberculosis complex; Mycobacterium tuberculosis;	NCBI: 882099	4364777	98.30	0.00	Yes
NCBI Assembly	GCA_000654115.1	NCBI: cellular organisms; Bacteria; Terrabacteria group; Actinobacteria; Actinomycetia; Corynebacteriales; Mycobacteriaceae; Mycobacterium; Mycobacterium tuberculosis complex; Mycobacterium tuberculosis;	NCBI: 1448576	4401598	99.94	0.50	Yes
NCBI Assembly	GCA_000653715.1	NCBI: cellular organisms; Bacteria; Terrabacteria group; Actinobacteria; Actinomycetia; Corynebacteriales; Mycobacteriaceae; Mycobacterium; Mycobacterium tuberculosis complex; Mycobacterium tuberculosis;	NCBI: 1448549	4389875	99.94	0.00	Yes
NCBI Assembly	GCA_000656575.1	NCBI: cellular organisms; Bacteria; Terrabacteria group; Actinobacteria; Actinomycetia; Corynebacteriales; Mycobacteriaceae; Mycobacterium; Mycobacterium tuberculosis complex; Mycobacterium tuberculosis;	NCBI: 1438879	4397792	99.94	0.00	Yes

NCBI Assembly	GCA_000656895.1	NCBI: cellular organisms; Bacteria; Terrabacteria group; Actinobacteria; Actinomycetia; Corynebacteriales; Mycobacteriaceae; Mycobacterium; Mycobacterium tuberculosis complex; Mycobacterium tuberculosis;	NCBI: 1448597	4444011	99.61	0.61	Yes
NCBI Assembly	GCA_000657495.1	NCBI: cellular organisms; Bacteria; Terrabacteria group; Actinobacteria; Actinomycetia; Corynebacteriales; Mycobacteriaceae; Mycobacterium; Mycobacterium tuberculosis complex; Mycobacterium tuberculosis;	NCBI: 1455290	4389787	99.94	0.00	Yes
NCBI Assembly	GCA_000220455.1	NCBI: cellular organisms; Bacteria; Terrabacteria group; Actinobacteria; Actinomycetia; Corynebacteriales; Mycobacteriaceae; Mycobacterium; Mycobacterium tuberculosis complex; Mycobacterium tuberculosis;	NCBI: 882100	4342459	99.50	0.00	Yes
NCBI Assembly	GCA_000653735.1	NCBI: cellular organisms; Bacteria; Terrabacteria group; Actinobacteria; Actinomycetia; Corynebacteriales; Mycobacteriaceae; Mycobacterium; Mycobacterium tuberculosis complex; Mycobacterium tuberculosis;	NCBI: 1448550	4399849	99.94	0.67	Yes
NCBI Assembly	GCA_000654135.1	NCBI: cellular organisms; Bacteria; Terrabacteria group; Actinobacteria; Actinomycetia; Corynebacteriales; Mycobacteriaceae; Mycobacterium; Mycobacterium tuberculosis complex; Mycobacterium tuberculosis;	NCBI: 1448577	4384569	99.94	0.00	Yes
NCBI Assembly	GCA_000656915.1	NCBI: cellular organisms; Bacteria; Terrabacteria group; Actinobacteria; Actinomycetia; Corynebacteriales; Mycobacteriaceae; Mycobacterium; Mycobacterium tuberculosis complex; Mycobacterium tuberculosis;	NCBI: 1448599	4419089	99.94	0.41	Yes
NCBI Assembly	GCA_000656595.1	NCBI: cellular organisms; Bacteria; Terrabacteria group; Actinobacteria; Actinomycetia; Corynebacteriales; Mycobacteriaceae; Mycobacterium; Mycobacterium tuberculosis complex; Mycobacterium tuberculosis;	NCBI: 1438892	4403606	99.94	0.00	Yes
NCBI Assembly	GCA_000657515.1	NCBI: cellular organisms; Bacteria; Terrabacteria group; Actinobacteria; Actinomycetia; Corynebacteriales; Mycobacteriaceae; Mycobacterium; Mycobacterium tuberculosis complex; Mycobacterium tuberculosis;	NCBI: 1455291	4411038	99.94	1.00	Yes
NCBI Assembly	GCA_000230895.3	NCBI: cellular organisms; Bacteria; Terrabacteria group; Actinobacteria; Actinomycetia; Corynebacteriales; Mycobacteriaceae; Mycolicibacterium; Mycolicibacterium rhodesiae;	NCBI: 710685	6415739	99.09	0.38	Yes
NCBI Assembly	GCA_000654155.1	NCBI: cellular organisms; Bacteria; Terrabacteria group; Actinobacteria; Actinomycetia; Corynebacteriales; Mycobacteriaceae; Mycobacterium; Mycobacterium tuberculosis complex; Mycobacterium tuberculosis;	NCBI: 1448579	4399045	99.94	0.00	Yes
NCBI Assembly	GCA_000653755.1	NCBI: cellular organisms; Bacteria; Terrabacteria group; Actinobacteria; Actinomycetia; Corynebacteriales; Mycobacteriaceae; Mycobacterium; Mycobacterium tuberculosis complex; Mycobacterium tuberculosis;	NCBI: 1448551	4414242	99.94	0.00	Yes
NCBI Assembly	GCA_000656615.1	NCBI: cellular organisms; Bacteria; Terrabacteria group; Actinobacteria; Actinomycetia; Corynebacteriales; Mycobacteriaceae; Mycobacterium; Mycobacterium tuberculosis complex; Mycobacterium tuberculosis;	NCBI: 1438877	4404845	99.94	0.00	Yes
NCBI Assembly	GCA_000656935.1	NCBI: cellular organisms; Bacteria; Terrabacteria group; Actinobacteria; Actinomycetia; Corynebacteriales; Mycobacteriaceae; Mycobacterium; Mycobacterium tuberculosis complex; Mycobacterium tuberculosis;	NCBI: 1448600	4419812	99.94	1.33	Yes
NCBI Assembly	GCA_000657535.1	NCBI: cellular organisms; Bacteria; Terrabacteria group; Actinobacteria; Actinomycetia; Corynebacteriales; Mycobacteriaceae; Mycobacterium; Mycobacterium tuberculosis complex; Mycobacterium tuberculosis;	NCBI: 1455293	4390429	99.94	0.00	Yes
NCBI Assembly	GCA_000230935.2	NCBI: cellular organisms; Bacteria; Terrabacteria group; Actinobacteria; Actinomycetia; Corynebacteriales; Mycobacteriaceae; Mycolicibacterium; Mycolicibacterium rhodesiae;	NCBI: 931627	7281599	100.00	1.44	Yes
NCBI Assembly	GCA_000654175.1	NCBI: cellular organisms; Bacteria; Terrabacteria group; Actinobacteria; Actinomycetia; Corynebacteriales; Mycobacteriaceae; Mycobacterium; Mycobacterium tuberculosis complex; Mycobacterium tuberculosis;	NCBI: 1448582	4406027	99.94	0.00	Yes
NCBI Assembly	GCA_000653775.1	NCBI: cellular organisms; Bacteria; Terrabacteria group; Actinobacteria; Actinomycetia; Corynebacteriales; Mycobacteriaceae; Mycobacterium; Mycobacterium tuberculosis complex; Mycobacterium tuberculosis;	NCBI: 1448552	4390705	99.94	0.00	Yes
NCBI Assembly	GCA_000656955.1	NCBI: cellular organisms; Bacteria; Terrabacteria group; Actinobacteria; Actinomycetia; Corynebacteriales; Mycobacteriaceae; Mycobacterium; Mycobacterium tuberculosis complex; Mycobacterium tuberculosis;	NCBI: 1448601	4392737	99.61	0.00	Yes

NCBI Assembly	GCA_000656635.1	NCBI: cellular organisms; Bacteria; Terrabacteria group; Actinobacteria; Actinomycetia; Corynebacteriales; Mycobacteriaceae; Mycobacterium; Mycobacterium tuberculosis complex; Mycobacterium tuberculosis;	NCBI: 1438867	4363420	99.90	0.00	Yes
NCBI Assembly	GCA_000657555.1	NCBI: cellular organisms; Bacteria; Terrabacteria group; Actinobacteria; Actinomycetia; Corynebacteriales; Mycobacteriaceae; Mycobacterium; Mycobacterium tuberculosis complex; Mycobacterium tuberculosis;	NCBI: 1455294	4403690	99.94	0.17	Yes
NCBI Assembly	GCA_000231155.2	NCBI: cellular organisms; Bacteria; Terrabacteria group; Actinobacteria; Actinomycetia; Corynebacteriales; Mycobacteriaceae; Mycobacteroides; Mycobacteroides abscessus;	NCBI: 1087483	4874047	100.00	0.00	Yes
NCBI Assembly	GCA_000654195.1	NCBI: cellular organisms; Bacteria; Terrabacteria group; Actinobacteria; Actinomycetia; Corynebacteriales; Mycobacteriaceae; Mycobacterium; Mycobacterium tuberculosis complex; Mycobacterium tuberculosis;	NCBI: 1448584	4407355	99.94	0.00	Yes
NCBI Assembly	GCA_000653795.1	NCBI: cellular organisms; Bacteria; Terrabacteria group; Actinobacteria; Actinomycetia; Corynebacteriales; Mycobacteriaceae; Mycobacterium; Mycobacterium tuberculosis complex; Mycobacterium tuberculosis;	NCBI: 1448554	4411711	99.94	0.90	Yes
NCBI Assembly	GCA_000656975.1	NCBI: cellular organisms; Bacteria; Terrabacteria group; Actinobacteria; Actinomycetia; Corynebacteriales; Mycobacteriaceae; Mycobacterium; Mycobacterium tuberculosis complex; Mycobacterium tuberculosis;	NCBI: 1448602	4443619	99.94	0.00	Yes
NCBI Assembly	GCA_000656655.1	NCBI: cellular organisms; Bacteria; Terrabacteria group; Actinobacteria; Actinomycetia; Corynebacteriales; Mycobacteriaceae; Mycobacterium; Mycobacterium tuberculosis complex; Mycobacterium tuberculosis;	NCBI: 1438862	4416049	99.94	0.89	Yes
NCBI Assembly	GCA_000657575.1	NCBI: cellular organisms; Bacteria; Terrabacteria group; Actinobacteria; Actinomycetia; Corynebacteriales; Mycobacteriaceae; Mycobacterium; Mycobacterium tuberculosis complex; Mycobacterium tuberculosis;	NCBI: 1455295	4375702	99.94	0.00	Yes
NCBI Assembly	GCA_000233935.2	NCBI: cellular organisms; Bacteria; Terrabacteria group; Actinobacteria; Actinomycetia; Corynebacteriales; Mycobacteriaceae; Mycolicibacterium; Mycolicibacterium conceptionense;	NCBI: 451644	6499679	100.00	1.45	Yes
NCBI Assembly	GCA_000656995.1	NCBI: cellular organisms; Bacteria; Terrabacteria group; Actinobacteria; Actinomycetia; Corynebacteriales; Mycobacteriaceae; Mycobacterium; Mycobacterium tuberculosis complex; Mycobacterium tuberculosis;	NCBI: 1448604	4363053	99.94	0.00	Yes
NCBI Assembly	GCA_000653815.1	NCBI: cellular organisms; Bacteria; Terrabacteria group; Actinobacteria; Actinomycetia; Corynebacteriales; Mycobacteriaceae; Mycobacterium; Mycobacterium tuberculosis complex; Mycobacterium tuberculosis;	NCBI: 1448555	4431625	99.94	0.26	Yes
NCBI Assembly	GCA_000654215.1	NCBI: cellular organisms; Bacteria; Terrabacteria group; Actinobacteria; Actinomycetia; Corynebacteriales; Mycobacteriaceae; Mycobacterium; Mycobacterium tuberculosis complex; Mycobacterium tuberculosis;	NCBI: 1448585	4399711	99.94	0.00	Yes
NCBI Assembly	GCA_000656675.1	NCBI: cellular organisms; Bacteria; Terrabacteria group; Actinobacteria; Actinomycetia; Corynebacteriales; Mycobacteriaceae; Mycobacterium; Mycobacterium tuberculosis complex; Mycobacterium tuberculosis;	NCBI: 1438873	4487379	99.94	4.18	Yes
NCBI Assembly	GCA_000657595.1	NCBI: cellular organisms; Bacteria; Terrabacteria group; Actinobacteria; Actinomycetia; Corynebacteriales; Mycobacteriaceae; Mycobacterium; Mycobacterium tuberculosis complex; Mycobacterium tuberculosis;	NCBI: 1455305	4391825	99.61	0.11	Yes
NCBI Assembly	GCA_000654235.1	NCBI: cellular organisms; Bacteria; Terrabacteria group; Actinobacteria; Actinomycetia; Corynebacteriales; Mycobacteriaceae; Mycobacterium; Mycobacterium tuberculosis complex; Mycobacterium tuberculosis;	NCBI: 1448610	4431876	99.94	0.00	Yes
NCBI Assembly	GCA_000657015.1	NCBI: cellular organisms; Bacteria; Terrabacteria group; Actinobacteria; Actinomycetia; Corynebacteriales; Mycobacteriaceae; Mycobacterium; Mycobacterium tuberculosis complex; Mycobacterium tuberculosis;	NCBI: 1448605	4418494	99.94	0.00	Yes
NCBI Assembly	GCA_000234585.2	NCBI: cellular organisms; Bacteria; Terrabacteria group; Actinobacteria; Actinomycetia; Corynebacteriales; Mycobacteriaceae; Mycolicibacterium; Mycolicibacterium thermoresistibile;	NCBI: 1078020	4870742	99.32	0.00	Yes
NCBI Assembly	GCA_000653835.1	NCBI: cellular organisms; Bacteria; Terrabacteria group; Actinobacteria; Actinomycetia; Corynebacteriales; Mycobacteriaceae; Mycobacterium; Mycobacterium tuberculosis complex; Mycobacterium tuberculosis;	NCBI: 1448556	4404262	99.94	0.00	Yes

NCBI Assembly	GCA_000656695.1	NCBI: cellular organisms; Bacteria; Terrabacteria group; Actinobacteria; Actinomycetia; Corynebacteriales; Mycobacteriaceae; Mycobacterium; Mycobacterium tuberculosis complex; Mycobacterium tuberculosis;	NCBI: 1448587	4393679	99.94	0.00	Yes
NCBI Assembly	GCA_000657615.1	NCBI: cellular organisms; Bacteria; Terrabacteria group; Actinobacteria; Actinomycetia; Corynebacteriales; Mycobacteriaceae; Mycobacterium; Mycobacterium tuberculosis complex; Mycobacterium tuberculosis;	NCBI: 1455307	4432326	99.94	0.00	Yes
NCBI Assembly	GCA_000239055.2	NCBI: cellular organisms; Bacteria; Terrabacteria group; Actinobacteria; Actinomycetia; Corynebacteriales; Mycobacteriaceae; Mycobacteroides; Mycobacteroides abscessus; Mycobacteroides abscessus subsp. massiliense;	NCBI: 1001714	4969787	100.00	0.11	Yes
NCBI Assembly	GCA_000654255.1	NCBI: cellular organisms; Bacteria; Terrabacteria group; Actinobacteria; Actinomycetia; Corynebacteriales; Mycobacteriaceae; Mycobacterium; Mycobacterium tuberculosis complex; Mycobacterium tuberculosis;	NCBI: 1448611	4438481	99.94	0.00	Yes
NCBI Assembly	GCA_000657035.1	NCBI: cellular organisms; Bacteria; Terrabacteria group; Actinobacteria; Actinomycetia; Corynebacteriales; Mycobacteriaceae; Mycobacterium; Mycobacterium tuberculosis complex; Mycobacterium tuberculosis;	NCBI: 1448607	4425690	99.94	0.00	Yes
NCBI Assembly	GCA_000653855.1	NCBI: cellular organisms; Bacteria; Terrabacteria group; Actinobacteria; Actinomycetia; Corynebacteriales; Mycobacteriaceae; Mycobacterium; Mycobacterium tuberculosis complex; Mycobacterium tuberculosis;	NCBI: 1448559	4409745	99.94	0.00	Yes
NCBI Assembly	GCA_000656715.1	NCBI: cellular organisms; Bacteria; Terrabacteria group; Actinobacteria; Actinomycetia; Corynebacteriales; Mycobacteriaceae; Mycobacterium; Mycobacterium tuberculosis complex; Mycobacterium tuberculosis;	NCBI: 1448588	4399787	99.94	0.00	Yes
NCBI Assembly	GCA_000657635.1	NCBI: cellular organisms; Bacteria; Terrabacteria group; Actinobacteria; Actinomycetia; Corynebacteriales; Mycobacteriaceae; Mycobacterium; Mycobacterium tuberculosis complex; Mycobacterium tuberculosis;	NCBI: 1455308	4416108	99.94	0.15	Yes
NCBI Assembly	GCA_000240245.2	NCBI: cellular organisms; Bacteria; Terrabacteria group; Actinobacteria; Actinomycetia; Corynebacteriales; Mycobacteriaceae; Mycobacterium; Mycobacterium tuberculosis complex; Mycobacterium tuberculosis;	NCBI: 1126682	4285124	99.19	0.33	Yes
NCBI Assembly	GCA_000654275.1	NCBI: cellular organisms; Bacteria; Terrabacteria group; Actinobacteria; Actinomycetia; Corynebacteriales; Mycobacteriaceae; Mycobacterium; Mycobacterium tuberculosis complex; Mycobacterium tuberculosis;	NCBI: 1448612	4433132	99.78	0.00	Yes
NCBI Assembly	GCA_000657055.1	NCBI: cellular organisms; Bacteria; Terrabacteria group; Actinobacteria; Actinomycetia; Corynebacteriales; Mycobacteriaceae; Mycobacterium; Mycobacterium tuberculosis complex; Mycobacterium tuberculosis;	NCBI: 1448608	4406047	99.94	0.00	Yes
NCBI Assembly	GCA_000653875.1	NCBI: cellular organisms; Bacteria; Terrabacteria group; Actinobacteria; Actinomycetia; Corynebacteriales; Mycobacteriaceae; Mycobacterium; Mycobacterium tuberculosis complex; Mycobacterium tuberculosis;	NCBI: 1448560	4413740	99.94	0.00	Yes
NCBI Assembly	GCA_000656735.1	NCBI: cellular organisms; Bacteria; Terrabacteria group; Actinobacteria; Actinomycetia; Corynebacteriales; Mycobacteriaceae; Mycobacterium; Mycobacterium tuberculosis complex; Mycobacterium tuberculosis;	NCBI: 1448589	4411312	99.94	0.00	Yes
NCBI Assembly	GCA_000657655.1	NCBI: cellular organisms; Bacteria; Terrabacteria group; Actinobacteria; Actinomycetia; Corynebacteriales; Mycobacteriaceae; Mycobacterium; Mycobacterium tuberculosis complex; Mycobacterium tuberculosis;	NCBI: 1455311	4432292	99.61	0.00	Yes
NCBI Assembly	GCA_000240265.2	NCBI: cellular organisms; Bacteria; Terrabacteria group; Actinobacteria; Actinomycetia; Corynebacteriales; Mycobacteriaceae; Mycobacterium; Mycobacterium tuberculosis complex; Mycobacterium tuberculosis;	NCBI: 1126683	4226640	98.31	1.08	Yes
NCBI Assembly	GCA_000653895.1	NCBI: cellular organisms; Bacteria; Terrabacteria group; Actinobacteria; Actinomycetia; Corynebacteriales; Mycobacteriaceae; Mycobacterium; Mycobacterium tuberculosis complex; Mycobacterium tuberculosis;	NCBI: 1448561	4388663	99.94	0.00	Yes
NCBI Assembly	GCA_000657075.1	NCBI: cellular organisms; Bacteria; Terrabacteria group; Actinobacteria; Actinomycetia; Corynebacteriales; Mycobacteriaceae; Mycobacterium; Mycobacterium tuberculosis complex; Mycobacterium tuberculosis;	NCBI: 1448609	4400696	99.94	0.00	Yes
NCBI Assembly	GCA_000656755.1	NCBI: cellular organisms; Bacteria; Terrabacteria group; Actinobacteria; Actinomycetia; Corynebacteriales; Mycobacteriaceae; Mycobacterium; Mycobacterium tuberculosis complex; Mycobacterium tuberculosis;	NCBI: 1448590	4401797	99.94	0.00	Yes

NCBI Assembly	GCA_000657675.1	NCBI: cellular organisms; Bacteria; Terrabacteria group; Actinobacteria; Actinomycetia; Corynebacteriales; Mycobacteriaceae; Mycobacterium; Mycobacterium tuberculosis complex; Mycobacterium tuberculosis;	NCBI: 1455312	4416351	99.94	0.00	Yes
NCBI Assembly	GCA_000240285.2	NCBI: cellular organisms; Bacteria; Terrabacteria group; Actinobacteria; Actinomycetia; Corynebacteriales; Mycobacteriaceae; Mycobacterium; Mycobacterium tuberculosis complex; Mycobacterium tuberculosis;	NCBI: 1126684	4266804	99.28	1.92	Yes
NCBI Assembly	GCA_000653915.1	NCBI: cellular organisms; Bacteria; Terrabacteria group; Actinobacteria; Actinomycetia; Corynebacteriales; Mycobacteriaceae; Mycobacterium; Mycobacterium tuberculosis complex; Mycobacterium tuberculosis;	NCBI: 1448562	4423456	99.94	0.00	Yes
NCBI Assembly	GCA_000657095.1	NCBI: cellular organisms; Bacteria; Terrabacteria group; Actinobacteria; Actinomycetia; Corynebacteriales; Mycobacteriaceae; Mycobacterium; Mycobacterium tuberculosis complex; Mycobacterium tuberculosis;	NCBI: 1455263	4326776	99.81	0.13	Yes
NCBI Assembly	GCA_000656775.1	NCBI: cellular organisms; Bacteria; Terrabacteria group; Actinobacteria; Actinomycetia; Corynebacteriales; Mycobacteriaceae; Mycobacterium; Mycobacterium tuberculosis complex; Mycobacterium tuberculosis;	NCBI: 1448591	4388949	99.94	0.00	Yes
NCBI Assembly	GCA_000657835.1	NCBI: cellular organisms; Bacteria; Terrabacteria group; Actinobacteria; Actinomycetia; Corynebacteriales; Mycobacteriaceae; Mycobacterium; Mycobacterium tuberculosis complex; Mycobacterium tuberculosis;	NCBI: 1324246	4398586	99.94	0.00	Yes
NCBI Assembly	GCA_000653935.1	NCBI: cellular organisms; Bacteria; Terrabacteria group; Actinobacteria; Actinomycetia; Corynebacteriales; Mycobacteriaceae; Mycobacterium; Mycobacterium tuberculosis complex; Mycobacterium tuberculosis;	NCBI: 1448563	4450392	99.83	1.14	Yes
NCBI Assembly	GCA_000657855.1	NCBI: cellular organisms; Bacteria; Terrabacteria group; Actinobacteria; Actinomycetia; Corynebacteriales; Mycobacteriaceae; Mycobacterium; Mycobacterium tuberculosis complex; Mycobacterium tuberculosis;	NCBI: 1402478	4364678	99.61	0.00	Yes
NCBI Assembly	GCA_000653955.1	NCBI: cellular organisms; Bacteria; Terrabacteria group; Actinobacteria; Actinomycetia; Corynebacteriales; Mycobacteriaceae; Mycobacterium; Mycobacterium tuberculosis complex; Mycobacterium tuberculosis;	NCBI: 1448565	4419714	99.94	0.00	Yes
NCBI Assembly	GCA_000657875.1	NCBI: cellular organisms; Bacteria; Terrabacteria group; Actinobacteria; Actinomycetia; Corynebacteriales; Mycobacteriaceae; Mycobacterium; Mycobacterium tuberculosis complex; Mycobacterium tuberculosis;	NCBI: 1408916	4425951	99.94	0.00	Yes
NCBI Assembly	GCA_000653975.1	NCBI: cellular organisms; Bacteria; Terrabacteria group; Actinobacteria; Actinomycetia; Corynebacteriales; Mycobacteriaceae; Mycobacterium; Mycobacterium tuberculosis complex; Mycobacterium tuberculosis;	NCBI: 1448567	4392147	99.94	0.00	Yes
NCBI Assembly	GCA_000679975.1	NCBI: cellular organisms; Bacteria; Terrabacteria group; Actinobacteria; Actinomycetia; Corynebacteriales; Mycobacteriaceae; Mycobacterium; Mycobacterium tuberculosis complex; Mycobacterium tuberculosis;	NCBI: 1427182	4420228	99.94	0.00	Yes
NCBI Assembly	GCA_000678715.1	NCBI: cellular organisms; Bacteria; Terrabacteria group; Actinobacteria; Actinomycetia; Corynebacteriales; Mycobacteriaceae; Mycobacterium; Mycobacterium tuberculosis complex; Mycobacterium tuberculosis;	NCBI: 1423514	4409910	99.94	0.00	Yes
NCBI Assembly	GCA_000677475.1	NCBI: cellular organisms; Bacteria; Terrabacteria group; Actinobacteria; Actinomycetia; Corynebacteriales; Mycobacteriaceae; Mycobacterium; Mycobacterium tuberculosis complex; Mycobacterium tuberculosis;	NCBI: 1423445	4413476	99.94	0.00	Yes
NCBI Assembly	GCA_000678395.1	NCBI: cellular organisms; Bacteria; Terrabacteria group; Actinobacteria; Actinomycetia; Corynebacteriales; Mycobacteriaceae; Mycobacterium; Mycobacterium tuberculosis complex; Mycobacterium tuberculosis;	NCBI: 1423496	4418651	99.94	0.00	Yes
NCBI Assembly	GCA_000677155.1	NCBI: cellular organisms; Bacteria; Terrabacteria group; Actinobacteria; Actinomycetia; Corynebacteriales; Mycobacteriaceae; Mycobacterium; Mycobacterium tuberculosis complex; Mycobacterium tuberculosis;	NCBI: 1423425	4421157	99.94	0.00	Yes
NCBI Assembly	GCA_000679655.1	NCBI: cellular organisms; Bacteria; Terrabacteria group; Actinobacteria; Actinomycetia; Corynebacteriales; Mycobacteriaceae; Mycobacterium; Mycobacterium tuberculosis complex; Mycobacterium tuberculosis;	NCBI: 1423566	4405400	99.94	0.00	Yes
NCBI Assembly	GCA_000681215.1	NCBI: cellular organisms; Bacteria; Terrabacteria group; Actinobacteria; Actinomycetia; Corynebacteriales; Mycobacteriaceae; Mycobacterium; Mycobacterium tuberculosis complex; Mycobacterium tuberculosis;	NCBI: 1427252	4426560	99.94	0.00	Yes

NCBI Assembly	GCA_000670415.1	NCBI: cellular organisms; Bacteria; Terrabacteria group; Actinobacteria; Actinomycetia; Corynebacteriales; Mycobacteriaceae; Mycobacterium; Mycobacterium tuberculosis complex; Mycobacterium tuberculosis;	NCBI: 1421945	4396227	99.94	0.00	Yes
NCBI Assembly	GCA_000706785.1	NCBI: cellular organisms; Bacteria; Terrabacteria group; Actinobacteria; Actinomycetia; Corynebacteriales; Mycobacteriaceae; Mycobacterium; Mycobacterium tuberculosis complex; Mycobacterium tuberculosis;	NCBI: 1400897	4422415	99.94	0.00	Yes
NCBI Assembly	GCA_000675895.1	NCBI: cellular organisms; Bacteria; Terrabacteria group; Actinobacteria; Actinomycetia; Corynebacteriales; Mycobacteriaceae; Mycobacterium; Mycobacterium tuberculosis complex; Mycobacterium tuberculosis;	NCBI: 1422048	4416829	99.94	0.00	Yes
NCBI Assembly	GCA_000680895.1	NCBI: cellular organisms; Bacteria; Terrabacteria group; Actinobacteria; Actinomycetia; Corynebacteriales; Mycobacteriaceae; Mycobacterium; Mycobacterium tuberculosis complex; Mycobacterium tuberculosis;	NCBI: 1427235	4422579	99.94	0.00	Yes
NCBI Assembly	GCA_000698475.1	NCBI: cellular organisms; Bacteria; Terrabacteria group; Actinobacteria; Actinomycetia; Corynebacteriales; Mycobacteriaceae; Mycobacterium; Mycobacterium tuberculosis complex; Mycobacterium tuberculosis;	NCBI: 1249615	4385518	99.94	0.00	Yes
NCBI Assembly	GCA_000677495.1	NCBI: cellular organisms; Bacteria; Terrabacteria group; Actinobacteria; Actinomycetia; Corynebacteriales; Mycobacteriaceae; Mycobacterium; Mycobacterium tuberculosis complex; Mycobacterium tuberculosis;	NCBI: 1423446	4416177	99.94	0.00	Yes
NCBI Assembly	GCA_000678735.1	NCBI: cellular organisms; Bacteria; Terrabacteria group; Actinobacteria; Actinomycetia; Corynebacteriales; Mycobacteriaceae; Mycobacterium; Mycobacterium tuberculosis complex; Mycobacterium tuberculosis;	NCBI: 1423516	4416909	99.94	0.00	Yes
NCBI Assembly	GCA_000677175.1	NCBI: cellular organisms; Bacteria; Terrabacteria group; Actinobacteria; Actinomycetia; Corynebacteriales; Mycobacteriaceae; Mycobacterium; Mycobacterium tuberculosis complex; Mycobacterium tuberculosis;	NCBI: 1423426	4416133	99.94	0.00	Yes
NCBI Assembly	GCA_000678415.1	NCBI: cellular organisms; Bacteria; Terrabacteria group; Actinobacteria; Actinomycetia; Corynebacteriales; Mycobacteriaceae; Mycobacterium; Mycobacterium tuberculosis complex; Mycobacterium tuberculosis;	NCBI: 1423497	4430970	99.94	0.00	Yes
NCBI Assembly	GCA_000670435.1	NCBI: cellular organisms; Bacteria; Terrabacteria group; Actinobacteria; Actinomycetia; Corynebacteriales; Mycobacteriaceae; Mycobacterium; Mycobacterium tuberculosis complex; Mycobacterium tuberculosis;	NCBI: 1421946	4416135	99.94	0.00	Yes
NCBI Assembly	GCA_000679995.1	NCBI: cellular organisms; Bacteria; Terrabacteria group; Actinobacteria; Actinomycetia; Corynebacteriales; Mycobacteriaceae; Mycobacterium; Mycobacterium tuberculosis complex; Mycobacterium tuberculosis;	NCBI: 1427183	4399769	99.94	0.00	Yes
NCBI Assembly	GCA_000680915.1	NCBI: cellular organisms; Bacteria; Terrabacteria group; Actinobacteria; Actinomycetia; Corynebacteriales; Mycobacteriaceae; Mycobacterium; Mycobacterium tuberculosis complex; Mycobacterium tuberculosis;	NCBI: 1427236	4427542	99.94	0.00	Yes
NCBI Assembly	GCA_000675915.1	NCBI: cellular organisms; Bacteria; Terrabacteria group; Actinobacteria; Actinomycetia; Corynebacteriales; Mycobacteriaceae; Mycobacterium; Mycobacterium tuberculosis complex; Mycobacterium tuberculosis;	NCBI: 1422049	4400141	99.94	0.00	Yes
NCBI Assembly	GCA_000681235.1	NCBI: cellular organisms; Bacteria; Terrabacteria group; Actinobacteria; Actinomycetia; Corynebacteriales; Mycobacteriaceae; Mycobacterium; Mycobacterium tuberculosis complex; Mycobacterium tuberculosis;	NCBI: 1427254	4418543	99.94	0.00	Yes
NCBI Assembly	GCA_000709305.1	NCBI: cellular organisms; Bacteria; Terrabacteria group; Actinobacteria; Actinomycetia; Corynebacteriales; Mycobacteriaceae; Mycobacterium; Mycobacterium tuberculosis complex; unclassified Mycobacterium tuberculosis complex;	NCBI: 1324269	5682965	99.94	0.32	Yes
NCBI Assembly	GCA_000679675.1	NCBI: cellular organisms; Bacteria; Terrabacteria group; Actinobacteria; Actinomycetia; Corynebacteriales; Mycobacteriaceae; Mycobacterium; Mycobacterium tuberculosis complex; Mycobacterium tuberculosis;	NCBI: 1423567	4409565	99.94	0.00	Yes
NCBI Assembly	GCA_000705655.1	NCBI: cellular organisms; Bacteria; Terrabacteria group; Actinobacteria; Actinomycetia; Corynebacteriales; Mycobacteriaceae; Mycobacterium; Mycobacterium tuberculosis complex; Mycobacterium tuberculosis; Mycobacterium tuberculosis variant bovis;	NCBI: 33892	4333130	99.78	0.60	Yes
NCBI Assembly	GCA_000678755.1	NCBI: cellular organisms; Bacteria; Terrabacteria group; Actinobacteria; Actinomycetia; Corynebacteriales; Mycobacteriaceae; Mycobacterium; Mycobacterium tuberculosis complex; Mycobacterium tuberculosis;	NCBI: 1423517	4428142	99.94	0.00	Yes

NCBI Assembly	GCA_000677515.1	NCBI: cellular organisms; Bacteria; Terrabacteria group; Actinobacteria; Actinomycetia; Corynebacteriales; Mycobacteriaceae; Mycobacterium; Mycobacterium tuberculosis complex; Mycobacterium tuberculosis;	NCBI: 1423447	4411372	99.94	0.00	Yes
NCBI Assembly	GCA_000670455.1	NCBI: cellular organisms; Bacteria; Terrabacteria group; Actinobacteria; Actinomycetia; Corynebacteriales; Mycobacteriaceae; Mycobacterium; Mycobacterium tuberculosis complex; Mycobacterium tuberculosis;	NCBI: 1421947	4404293	99.94	0.00	Yes
NCBI Assembly	GCA_000678435.1	NCBI: cellular organisms; Bacteria; Terrabacteria group; Actinobacteria; Actinomycetia; Corynebacteriales; Mycobacteriaceae; Mycobacterium; Mycobacterium tuberculosis complex; Mycobacterium tuberculosis;	NCBI: 1423498	4402728	99.94	0.00	Yes
NCBI Assembly	GCA_000680935.1	NCBI: cellular organisms; Bacteria; Terrabacteria group; Actinobacteria; Actinomycetia; Corynebacteriales; Mycobacteriaceae; Mycobacterium; Mycobacterium tuberculosis complex; Mycobacterium tuberculosis;	NCBI: 1427237	4432054	99.78	0.00	Yes
NCBI Assembly	GCA_000677195.1	NCBI: cellular organisms; Bacteria; Terrabacteria group; Actinobacteria; Actinomycetia; Corynebacteriales; Mycobacteriaceae; Mycobacterium; Mycobacterium tuberculosis complex; Mycobacterium tuberculosis;	NCBI: 1423428	4424962	99.94	0.00	Yes
NCBI Assembly	GCA_000680015.1	NCBI: cellular organisms; Bacteria; Terrabacteria group; Actinobacteria; Actinomycetia; Corynebacteriales; Mycobacteriaceae; Mycobacterium; Mycobacterium tuberculosis complex; Mycobacterium tuberculosis;	NCBI: 1427185	4437452	99.94	0.33	Yes
NCBI Assembly	GCA_000675935.1	NCBI: cellular organisms; Bacteria; Terrabacteria group; Actinobacteria; Actinomycetia; Corynebacteriales; Mycobacteriaceae; Mycobacterium; Mycobacterium tuberculosis complex; Mycobacterium tuberculosis;	NCBI: 1422050	4434225	99.94	0.00	Yes
NCBI Assembly	GCA_000681255.1	NCBI: cellular organisms; Bacteria; Terrabacteria group; Actinobacteria; Actinomycetia; Corynebacteriales; Mycobacteriaceae; Mycobacterium; Mycobacterium tuberculosis complex; Mycobacterium tuberculosis;	NCBI: 1427255	4423612	99.94	0.00	Yes
NCBI Assembly	GCA_000709575.1	NCBI: cellular organisms; Bacteria; Terrabacteria group; Actinobacteria; Actinomycetia; Corynebacteriales; Mycobacteriaceae; Mycobacterium; Mycobacterium tuberculosis complex; Mycobacterium tuberculosis;	NCBI: 1354276	4202119	97.19	1.13	Yes
NCBI Assembly	GCA_000679695.1	NCBI: cellular organisms; Bacteria; Terrabacteria group; Actinobacteria; Actinomycetia; Corynebacteriales; Mycobacteriaceae; Mycobacterium; Mycobacterium tuberculosis complex; Mycobacterium tuberculosis;	NCBI: 1423568	4407802	99.94	0.00	Yes
NCBI Assembly	GCA_000705705.1	NCBI: cellular organisms; Bacteria; Terrabacteria group; Actinobacteria; Actinomycetia; Corynebacteriales; Mycobacteriaceae; Mycobacterium; Mycobacterium tuberculosis complex; Mycobacterium tuberculosis;	NCBI: 1455302	4417440	99.94	0.00	Yes
NCBI Assembly	GCA_000670475.1	NCBI: cellular organisms; Bacteria; Terrabacteria group; Actinobacteria; Actinomycetia; Corynebacteriales; Mycobacteriaceae; Mycobacterium; Mycobacterium tuberculosis complex; Mycobacterium tuberculosis;	NCBI: 1421948	4405655	99.94	0.00	Yes
NCBI Assembly	GCA_000678775.1	NCBI: cellular organisms; Bacteria; Terrabacteria group; Actinobacteria; Actinomycetia; Corynebacteriales; Mycobacteriaceae; Mycobacterium; Mycobacterium tuberculosis complex; Mycobacterium tuberculosis;	NCBI: 1423518	4438418	99.94	0.00	Yes
NCBI Assembly	GCA_000677535.1	NCBI: cellular organisms; Bacteria; Terrabacteria group; Actinobacteria; Actinomycetia; Corynebacteriales; Mycobacteriaceae; Mycobacterium; Mycobacterium tuberculosis complex; Mycobacterium tuberculosis;	NCBI: 1423448	4411753	99.94	0.00	Yes
NCBI Assembly	GCA_000680955.1	NCBI: cellular organisms; Bacteria; Terrabacteria group; Actinobacteria; Actinomycetia; Corynebacteriales; Mycobacteriaceae; Mycobacterium; Mycobacterium tuberculosis complex; Mycobacterium tuberculosis;	NCBI: 1427238	4422792	99.94	0.00	Yes
NCBI Assembly	GCA_000678455.1	NCBI: cellular organisms; Bacteria; Terrabacteria group; Actinobacteria; Actinomycetia; Corynebacteriales; Mycobacteriaceae; Mycobacterium; Mycobacterium tuberculosis complex; Mycobacterium tuberculosis;	NCBI: 1423499	4415005	99.94	0.00	Yes
NCBI Assembly	GCA_000675955.1	NCBI: cellular organisms; Bacteria; Terrabacteria group; Actinobacteria; Actinomycetia; Corynebacteriales; Mycobacteriaceae; Mycobacterium; Mycobacterium tuberculosis complex; Mycobacterium tuberculosis;	NCBI: 1422051	4424602	99.94	0.00	Yes
NCBI Assembly	GCA_000680035.1	NCBI: cellular organisms; Bacteria; Terrabacteria group; Actinobacteria; Actinomycetia; Corynebacteriales; Mycobacteriaceae; Mycobacterium; Mycobacterium tuberculosis complex; Mycobacterium tuberculosis;	NCBI: 1427186	4409933	99.94	0.00	Yes

NCBI Assembly	GCA_000677215.1	NCBI: cellular organisms; Bacteria; Terrabacteria group; Actinobacteria; Actinomycetia; Corynebacteriales; Mycobacteriaceae; Mycobacterium; Mycobacterium tuberculosis complex; Mycobacterium tuberculosis;	NCBI: 1423430	4409847	99.94	0.00	Yes
NCBI Assembly	GCA_000709595.1	NCBI: cellular organisms; Bacteria; Terrabacteria group; Actinobacteria; Actinomycetia; Corynebacteriales; Mycobacteriaceae; Mycobacterium; Mycobacterium tuberculosis complex; Mycobacterium tuberculosis;	NCBI: 1353527	4421067	99.05	1.11	Yes
NCBI Assembly	GCA_000681275.1	NCBI: cellular organisms; Bacteria; Terrabacteria group; Actinobacteria; Actinomycetia; Corynebacteriales; Mycobacteriaceae; Mycobacterium; Mycobacterium tuberculosis complex; Mycobacterium tuberculosis;	NCBI: 1427256	4429981	99.94	0.00	Yes
NCBI Assembly	GCA_000679715.1	NCBI: cellular organisms; Bacteria; Terrabacteria group; Actinobacteria; Actinomycetia; Corynebacteriales; Mycobacteriaceae; Mycobacterium; Mycobacterium tuberculosis complex; Mycobacterium tuberculosis;	NCBI: 1423570	4413538	99.94	0.00	Yes
NCBI Assembly	GCA_000705725.1	NCBI: cellular organisms; Bacteria; Terrabacteria group; Actinobacteria; Actinomycetia; Corynebacteriales; Mycobacteriaceae; Mycobacterium; Mycobacterium tuberculosis complex; Mycobacterium tuberculosis;	NCBI: 1448537	4423262	99.94	0.00	Yes
NCBI Assembly	GCA_000677555.1	NCBI: cellular organisms; Bacteria; Terrabacteria group; Actinobacteria; Actinomycetia; Corynebacteriales; Mycobacteriaceae; Mycobacterium; Mycobacterium tuberculosis complex; Mycobacterium tuberculosis;	NCBI: 1423449	4425282	99.94	0.00	Yes
NCBI Assembly	GCA_000670495.1	NCBI: cellular organisms; Bacteria; Terrabacteria group; Actinobacteria; Actinomycetia; Corynebacteriales; Mycobacteriaceae; Mycobacterium; Mycobacterium tuberculosis complex; Mycobacterium tuberculosis;	NCBI: 1421949	4410849	99.94	0.00	Yes
NCBI Assembly	GCA_000675975.1	NCBI: cellular organisms; Bacteria; Terrabacteria group; Actinobacteria; Actinomycetia; Corynebacteriales; Mycobacteriaceae; Mycobacterium; Mycobacterium tuberculosis complex; Mycobacterium tuberculosis;	NCBI: 1422052	4426295	99.94	0.00	Yes
NCBI Assembly	GCA_000680975.1	NCBI: cellular organisms; Bacteria; Terrabacteria group; Actinobacteria; Actinomycetia; Corynebacteriales; Mycobacteriaceae; Mycobacterium; Mycobacterium tuberculosis complex; Mycobacterium tuberculosis;	NCBI: 1427239	4420032	99.94	0.00	Yes
NCBI Assembly	GCA_000680055.1	NCBI: cellular organisms; Bacteria; Terrabacteria group; Actinobacteria; Actinomycetia; Corynebacteriales; Mycobacteriaceae; Mycobacterium; Mycobacterium tuberculosis complex; Mycobacterium tuberculosis;	NCBI: 1427187	4432981	99.94	0.00	Yes
NCBI Assembly	GCA_000678795.1	NCBI: cellular organisms; Bacteria; Terrabacteria group; Actinobacteria; Actinomycetia; Corynebacteriales; Mycobacteriaceae; Mycobacterium; Mycobacterium tuberculosis complex; Mycobacterium tuberculosis;	NCBI: 1423520	4395474	99.94	0.00	Yes
NCBI Assembly	GCA_000677235.1	NCBI: cellular organisms; Bacteria; Terrabacteria group; Actinobacteria; Actinomycetia; Corynebacteriales; Mycobacteriaceae; Mycobacterium; Mycobacterium tuberculosis complex; Mycobacterium tuberculosis;	NCBI: 1423431	4467415	99.61	0.00	Yes
NCBI Assembly	GCA_000678475.1	NCBI: cellular organisms; Bacteria; Terrabacteria group; Actinobacteria; Actinomycetia; Corynebacteriales; Mycobacteriaceae; Mycobacterium; Mycobacterium tuberculosis complex; Mycobacterium tuberculosis;	NCBI: 1423500	4400681	99.94	0.00	Yes
NCBI Assembly	GCA_000709615.1	NCBI: cellular organisms; Bacteria; Terrabacteria group; Actinobacteria; Actinomycetia; Corynebacteriales; Mycobacteriaceae; Mycobacterium; Mycobacterium tuberculosis complex; Mycobacterium tuberculosis;	NCBI: 1355018	4200876	97.82	1.07	Yes
NCBI Assembly	GCA_000681295.1	NCBI: cellular organisms; Bacteria; Terrabacteria group; Actinobacteria; Actinomycetia; Corynebacteriales; Mycobacteriaceae; Mycobacterium; Mycobacterium tuberculosis complex; Mycobacterium tuberculosis;	NCBI: 1427257	4447920	99.94	0.33	Yes
NCBI Assembly	GCA_000679735.1	NCBI: cellular organisms; Bacteria; Terrabacteria group; Actinobacteria; Actinomycetia; Corynebacteriales; Mycobacteriaceae; Mycobacterium; Mycobacterium tuberculosis complex; Mycobacterium tuberculosis;	NCBI: 1423571	4398858	99.94	0.00	Yes
NCBI Assembly	GCA_000670515.1	NCBI: cellular organisms; Bacteria; Terrabacteria group; Actinobacteria; Actinomycetia; Corynebacteriales; Mycobacteriaceae; Mycobacterium; Mycobacterium tuberculosis complex; Mycobacterium tuberculosis;	NCBI: 1421950	4404127	99.94	0.00	Yes
NCBI Assembly	GCA_000677575.1	NCBI: cellular organisms; Bacteria; Terrabacteria group; Actinobacteria; Actinomycetia; Corynebacteriales; Mycobacteriaceae; Mycobacterium; Mycobacterium tuberculosis complex; Mycobacterium tuberculosis;	NCBI: 1423450	4377831	99.94	0.00	Yes

NCBI Assembly	GCA_000705745.1	NCBI: cellular organisms; Bacteria; Terrabacteria group; Actinobacteria; Actinomycetia; Corynebacteriales; Mycobacteriaceae; Mycobacterium; Mycobacterium tuberculosis complex; Mycobacterium tuberculosis;	NCBI: 1448519	4424415	99.94	0.00	Yes
NCBI Assembly	GCA_000678495.1	NCBI: cellular organisms; Bacteria; Terrabacteria group; Actinobacteria; Actinomycetia; Corynebacteriales; Mycobacteriaceae; Mycobacterium; Mycobacterium tuberculosis complex; Mycobacterium tuberculosis;	NCBI: 1423501	4416839	99.94	0.00	Yes
NCBI Assembly	GCA_000675995.1	NCBI: cellular organisms; Bacteria; Terrabacteria group; Actinobacteria; Actinomycetia; Corynebacteriales; Mycobacteriaceae; Mycobacterium; Mycobacterium tuberculosis complex; Mycobacterium tuberculosis;	NCBI: 1422053	4409762	99.94	0.00	Yes
NCBI Assembly	GCA_000677255.1	NCBI: cellular organisms; Bacteria; Terrabacteria group; Actinobacteria; Actinomycetia; Corynebacteriales; Mycobacteriaceae; Mycobacterium; Mycobacterium tuberculosis complex; Mycobacterium tuberculosis;	NCBI: 1423432	4401970	99.78	2.42	Yes
NCBI Assembly	GCA_000680995.1	NCBI: cellular organisms; Bacteria; Terrabacteria group; Actinobacteria; Actinomycetia; Corynebacteriales; Mycobacteriaceae; Mycobacterium; Mycobacterium tuberculosis complex; Mycobacterium tuberculosis;	NCBI: 1427240	4429370	99.94	0.00	Yes
NCBI Assembly	GCA_000680075.1	NCBI: cellular organisms; Bacteria; Terrabacteria group; Actinobacteria; Actinomycetia; Corynebacteriales; Mycobacteriaceae; Mycobacterium; Mycobacterium tuberculosis complex; Mycobacterium tuberculosis;	NCBI: 1427188	4421021	99.94	0.00	Yes
NCBI Assembly	GCA_000678815.1	NCBI: cellular organisms; Bacteria; Terrabacteria group; Actinobacteria; Actinomycetia; Corynebacteriales; Mycobacteriaceae; Mycobacterium; Mycobacterium tuberculosis complex; Mycobacterium tuberculosis;	NCBI: 1423522	4446048	99.94	0.00	Yes
NCBI Assembly	GCA_000709635.1	NCBI: cellular organisms; Bacteria; Terrabacteria group; Actinobacteria; Actinomycetia; Corynebacteriales; Mycobacteriaceae; Mycobacterium; Mycobacterium tuberculosis complex; Mycobacterium tuberculosis;	NCBI: 1355019	4204858	98.09	1.07	Yes
NCBI Assembly	GCA_000681315.1	NCBI: cellular organisms; Bacteria; Terrabacteria group; Actinobacteria; Actinomycetia; Corynebacteriales; Mycobacteriaceae; Mycobacterium; Mycobacterium tuberculosis complex; Mycobacterium tuberculosis;	NCBI: 1427258	4433008	99.78	0.00	Yes
NCBI Assembly	GCA_000679755.1	NCBI: cellular organisms; Bacteria; Terrabacteria group; Actinobacteria; Actinomycetia; Corynebacteriales; Mycobacteriaceae; Mycobacterium; Mycobacterium tuberculosis complex; Mycobacterium tuberculosis;	NCBI: 1423572	4379951	99.94	0.00	Yes
NCBI Assembly	GCA_000670535.1	NCBI: cellular organisms; Bacteria; Terrabacteria group; Actinobacteria; Actinomycetia; Corynebacteriales; Mycobacteriaceae; Mycobacterium; Mycobacterium tuberculosis complex; Mycobacterium tuberculosis;	NCBI: 1421951	4435469	99.94	0.00	Yes
NCBI Assembly	GCA_000677595.1	NCBI: cellular organisms; Bacteria; Terrabacteria group; Actinobacteria; Actinomycetia; Corynebacteriales; Mycobacteriaceae; Mycobacterium; Mycobacterium tuberculosis complex; Mycobacterium tuberculosis;	NCBI: 1423451	4422263	99.94	0.00	Yes
NCBI Assembly	GCA_000705765.1	NCBI: cellular organisms; Bacteria; Terrabacteria group; Actinobacteria; Actinomycetia; Corynebacteriales; Mycobacteriaceae; Mycobacterium; Mycobacterium tuberculosis complex; Mycobacterium tuberculosis;	NCBI: 1448436	4414031	99.94	0.00	Yes
NCBI Assembly	GCA_000678515.1	NCBI: cellular organisms; Bacteria; Terrabacteria group; Actinobacteria; Actinomycetia; Corynebacteriales; Mycobacteriaceae; Mycobacterium; Mycobacterium tuberculosis complex; Mycobacterium tuberculosis;	NCBI: 1423502	4407746	99.94	0.00	Yes
NCBI Assembly	GCA_000676015.1	NCBI: cellular organisms; Bacteria; Terrabacteria group; Actinobacteria; Actinomycetia; Corynebacteriales; Mycobacteriaceae; Mycobacterium; Mycobacterium tuberculosis complex; Mycobacterium tuberculosis;	NCBI: 1422054	4410727	99.94	0.00	Yes
NCBI Assembly	GCA_000681015.1	NCBI: cellular organisms; Bacteria; Terrabacteria group; Actinobacteria; Actinomycetia; Corynebacteriales; Mycobacteriaceae; Mycobacterium; Mycobacterium tuberculosis complex; Mycobacterium tuberculosis;	NCBI: 1427241	4423030	99.94	0.00	Yes
NCBI Assembly	GCA_000677275.1	NCBI: cellular organisms; Bacteria; Terrabacteria group; Actinobacteria; Actinomycetia; Corynebacteriales; Mycobacteriaceae; Mycobacterium; Mycobacterium tuberculosis complex; Mycobacterium tuberculosis;	NCBI: 1423435	4420187	99.94	0.00	Yes
NCBI Assembly	GCA_000678835.1	NCBI: cellular organisms; Bacteria; Terrabacteria group; Actinobacteria; Actinomycetia; Corynebacteriales; Mycobacteriaceae; Mycobacterium; Mycobacterium tuberculosis complex; Mycobacterium tuberculosis;	NCBI: 1423523	4418470	99.94	0.00	Yes

NCBI Assembly	GCA_000709655.1	NCBI: cellular organisms; Bacteria; Terrabacteria group; Actinobacteria; Actinomycetia; Corynebacteriales; Mycobacteriaceae; Mycobacterium; Mycobacterium tuberculosis complex; Mycobacterium tuberculosis;	NCBI: 1355020	4188099	97.15	1.30	Yes
NCBI Assembly	GCA_000680095.1	NCBI: cellular organisms; Bacteria; Terrabacteria group; Actinobacteria; Actinomycetia; Corynebacteriales; Mycobacteriaceae; Mycobacterium; Mycobacterium tuberculosis complex; Mycobacterium tuberculosis;	NCBI: 1427189	4411286	99.94	0.00	Yes
NCBI Assembly	GCA_000681335.1	NCBI: cellular organisms; Bacteria; Terrabacteria group; Actinobacteria; Actinomycetia; Corynebacteriales; Mycobacteriaceae; Mycobacterium; Mycobacterium tuberculosis complex; Mycobacterium tuberculosis;	NCBI: 1402586	4406375	99.94	0.00	Yes
NCBI Assembly	GCA_000679775.1	NCBI: cellular organisms; Bacteria; Terrabacteria group; Actinobacteria; Actinomycetia; Corynebacteriales; Mycobacteriaceae; Mycobacterium; Mycobacterium tuberculosis complex; Mycobacterium tuberculosis;	NCBI: 1423573	4381330	99.94	0.00	Yes
NCBI Assembly	GCA_000670555.1	NCBI: cellular organisms; Bacteria; Terrabacteria group; Actinobacteria; Actinomycetia; Corynebacteriales; Mycobacteriaceae; Mycobacterium; Mycobacterium tuberculosis complex; Mycobacterium tuberculosis;	NCBI: 1421952	4402094	99.94	0.00	Yes
NCBI Assembly	GCA_000677615.1	NCBI: cellular organisms; Bacteria; Terrabacteria group; Actinobacteria; Actinomycetia; Corynebacteriales; Mycobacteriaceae; Mycobacterium; Mycobacterium tuberculosis complex; Mycobacterium tuberculosis;	NCBI: 1423452	4405545	99.94	0.00	Yes
NCBI Assembly	GCA_000705785.1	NCBI: cellular organisms; Bacteria; Terrabacteria group; Actinobacteria; Actinomycetia; Corynebacteriales; Mycobacteriaceae; Mycobacterium; Mycobacterium tuberculosis complex; Mycobacterium tuberculosis;	NCBI: 1438869	4408739	99.94	0.00	Yes
NCBI Assembly	GCA_000676035.1	NCBI: cellular organisms; Bacteria; Terrabacteria group; Actinobacteria; Actinomycetia; Corynebacteriales; Mycobacteriaceae; Mycobacterium; Mycobacterium tuberculosis complex; Mycobacterium tuberculosis;	NCBI: 1422055	4400191	99.94	0.17	Yes
NCBI Assembly	GCA_000678535.1	NCBI: cellular organisms; Bacteria; Terrabacteria group; Actinobacteria; Actinomycetia; Corynebacteriales; Mycobacteriaceae; Mycobacterium; Mycobacterium tuberculosis complex; Mycobacterium tuberculosis;	NCBI: 1423503	4406204	99.94	0.00	Yes
NCBI Assembly	GCA_000681035.1	NCBI: cellular organisms; Bacteria; Terrabacteria group; Actinobacteria; Actinomycetia; Corynebacteriales; Mycobacteriaceae; Mycobacterium; Mycobacterium tuberculosis complex; Mycobacterium tuberculosis;	NCBI: 1427242	4418956	99.94	0.00	Yes
NCBI Assembly	GCA_000677295.1	NCBI: cellular organisms; Bacteria; Terrabacteria group; Actinobacteria; Actinomycetia; Corynebacteriales; Mycobacteriaceae; Mycobacterium; Mycobacterium tuberculosis complex; Mycobacterium tuberculosis;	NCBI: 1423436	4413615	99.94	0.00	Yes
NCBI Assembly	GCA_000678855.1	NCBI: cellular organisms; Bacteria; Terrabacteria group; Actinobacteria; Actinomycetia; Corynebacteriales; Mycobacteriaceae; Mycobacterium; Mycobacterium tuberculosis complex; Mycobacterium tuberculosis;	NCBI: 1423526	4403121	99.94	0.00	Yes
NCBI Assembly	GCA_000709675.1	NCBI: cellular organisms; Bacteria; Terrabacteria group; Actinobacteria; Actinomycetia; Corynebacteriales; Mycobacteriaceae; Mycobacterium; Mycobacterium tuberculosis complex; Mycobacterium tuberculosis;	NCBI: 1365190	4192557	97.57	1.49	Yes
NCBI Assembly	GCA_000680115.1	NCBI: cellular organisms; Bacteria; Terrabacteria group; Actinobacteria; Actinomycetia; Corynebacteriales; Mycobacteriaceae; Mycobacterium; Mycobacterium tuberculosis complex; Mycobacterium tuberculosis;	NCBI: 1427190	4409533	99.94	0.00	Yes
NCBI Assembly	GCA_000685425.1	NCBI: cellular organisms; Bacteria; Terrabacteria group; Actinobacteria; Actinomycetia; Corynebacteriales; Mycobacteriaceae; Mycobacterium; Mycobacterium tuberculosis complex; Mycobacterium tuberculosis;	NCBI: 1773	4453307	99.94	1.00	Yes
NCBI Assembly	GCA_000679795.1	NCBI: cellular organisms; Bacteria; Terrabacteria group; Actinobacteria; Actinomycetia; Corynebacteriales; Mycobacteriaceae; Mycobacterium; Mycobacterium tuberculosis complex; Mycobacterium tuberculosis;	NCBI: 1423574	4439854	99.94	0.00	Yes
NCBI Assembly	GCA_000670575.1	NCBI: cellular organisms; Bacteria; Terrabacteria group; Actinobacteria; Actinomycetia; Corynebacteriales; Mycobacteriaceae; Mycobacterium; Mycobacterium tuberculosis complex; Mycobacterium tuberculosis;	NCBI: 1421953	4434936	99.78	0.67	Yes
NCBI Assembly	GCA_000677635.1	NCBI: cellular organisms; Bacteria; Terrabacteria group; Actinobacteria; Actinomycetia; Corynebacteriales; Mycobacteriaceae; Mycobacterium; Mycobacterium tuberculosis complex; Mycobacterium tuberculosis;	NCBI: 1423453	4412434	99.94	0.00	Yes

NCBI Assembly	GCA_000705805.1	NCBI: cellular organisms; Bacteria; Terrabacteria group; Actinobacteria; Actinomycetia; Corynebacteriales; Mycobacteriaceae; Mycobacterium; Mycobacterium tuberculosis complex; Mycobacterium tuberculosis;	NCBI: 1438847	4406218	99.94	0.00	Yes
NCBI Assembly	GCA_000681055.1	NCBI: cellular organisms; Bacteria; Terrabacteria group; Actinobacteria; Actinomycetia; Corynebacteriales; Mycobacteriaceae; Mycobacterium; Mycobacterium tuberculosis complex; Mycobacterium tuberculosis;	NCBI: 1427243	4429763	99.94	0.00	Yes
NCBI Assembly	GCA_000676055.1	NCBI: cellular organisms; Bacteria; Terrabacteria group; Actinobacteria; Actinomycetia; Corynebacteriales; Mycobacteriaceae; Mycobacterium; Mycobacterium tuberculosis complex; Mycobacterium tuberculosis;	NCBI: 1422056	4404995	99.94	0.00	Yes
NCBI Assembly	GCA_000678555.1	NCBI: cellular organisms; Bacteria; Terrabacteria group; Actinobacteria; Actinomycetia; Corynebacteriales; Mycobacteriaceae; Mycobacterium; Mycobacterium tuberculosis complex; Mycobacterium tuberculosis;	NCBI: 1423504	4418663	99.94	0.00	Yes
NCBI Assembly	GCA_000677315.1	NCBI: cellular organisms; Bacteria; Terrabacteria group; Actinobacteria; Actinomycetia; Corynebacteriales; Mycobacteriaceae; Mycobacterium; Mycobacterium tuberculosis complex; Mycobacterium tuberculosis;	NCBI: 1423437	4401177	99.94	0.00	Yes
NCBI Assembly	GCA_000678875.1	NCBI: cellular organisms; Bacteria; Terrabacteria group; Actinobacteria; Actinomycetia; Corynebacteriales; Mycobacteriaceae; Mycobacterium; Mycobacterium tuberculosis complex; Mycobacterium tuberculosis;	NCBI: 1423527	4412632	99.94	0.00	Yes
NCBI Assembly	GCA_000709695.1	NCBI: cellular organisms; Bacteria; Terrabacteria group; Actinobacteria; Actinomycetia; Corynebacteriales; Mycobacteriaceae; Mycobacterium; Mycobacterium tuberculosis complex; Mycobacterium tuberculosis;	NCBI: 1365185	4169481	97.15	1.23	Yes
NCBI Assembly	GCA_000680135.1	NCBI: cellular organisms; Bacteria; Terrabacteria group; Actinobacteria; Actinomycetia; Corynebacteriales; Mycobacteriaceae; Mycobacterium; Mycobacterium tuberculosis complex; Mycobacterium tuberculosis;	NCBI: 1427191	4419694	99.94	0.00	Yes
NCBI Assembly	GCA_000686745.1	NCBI: cellular organisms; Bacteria; Terrabacteria group; Actinobacteria; Actinomycetia; Corynebacteriales; Mycobacteriaceae; Mycobacterium; unclassified Mycobacterium;	NCBI: 1449059	6052684	99.81	0.80	Yes
NCBI Assembly	GCA_000679815.1	NCBI: cellular organisms; Bacteria; Terrabacteria group; Actinobacteria; Actinomycetia; Corynebacteriales; Mycobacteriaceae; Mycobacterium; Mycobacterium tuberculosis complex; Mycobacterium tuberculosis;	NCBI: 1423575	4401345	99.94	0.00	Yes
NCBI Assembly	GCA_000677655.1	NCBI: cellular organisms; Bacteria; Terrabacteria group; Actinobacteria; Actinomycetia; Corynebacteriales; Mycobacteriaceae; Mycobacterium; Mycobacterium tuberculosis complex; Mycobacterium tuberculosis;	NCBI: 1423454	4449152	99.94	0.00	Yes
NCBI Assembly	GCA_000670595.1	NCBI: cellular organisms; Bacteria; Terrabacteria group; Actinobacteria; Actinomycetia; Corynebacteriales; Mycobacteriaceae; Mycobacterium; Mycobacterium tuberculosis complex; Mycobacterium tuberculosis;	NCBI: 1421954	4401176	99.94	0.00	Yes
NCBI Assembly	GCA_000681075.1	NCBI: cellular organisms; Bacteria; Terrabacteria group; Actinobacteria; Actinomycetia; Corynebacteriales; Mycobacteriaceae; Mycobacterium; Mycobacterium tuberculosis complex; Mycobacterium tuberculosis;	NCBI: 1427244	4393769	99.94	0.00	Yes
NCBI Assembly	GCA_000705825.1	NCBI: cellular organisms; Bacteria; Terrabacteria group; Actinobacteria; Actinomycetia; Corynebacteriales; Mycobacteriaceae; Mycobacterium; Mycobacterium tuberculosis complex; Mycobacterium tuberculosis;	NCBI: 1438871	4391390	99.94	0.00	Yes
NCBI Assembly	GCA_000676075.1	NCBI: cellular organisms; Bacteria; Terrabacteria group; Actinobacteria; Actinomycetia; Corynebacteriales; Mycobacteriaceae; Mycobacterium; Mycobacterium tuberculosis complex; Mycobacterium tuberculosis;	NCBI: 1422057	4415066	99.94	0.00	Yes
NCBI Assembly	GCA_000678575.1	NCBI: cellular organisms; Bacteria; Terrabacteria group; Actinobacteria; Actinomycetia; Corynebacteriales; Mycobacteriaceae; Mycobacterium; Mycobacterium tuberculosis complex; Mycobacterium tuberculosis;	NCBI: 1423505	4397388	99.94	0.00	Yes
NCBI Assembly	GCA_000678895.1	NCBI: cellular organisms; Bacteria; Terrabacteria group; Actinobacteria; Actinomycetia; Corynebacteriales; Mycobacteriaceae; Mycobacterium; Mycobacterium tuberculosis complex; Mycobacterium tuberculosis;	NCBI: 1423528	4411096	99.94	0.00	Yes
NCBI Assembly	GCA_000677335.1	NCBI: cellular organisms; Bacteria; Terrabacteria group; Actinobacteria; Actinomycetia; Corynebacteriales; Mycobacteriaceae; Mycobacterium; Mycobacterium tuberculosis complex; Mycobacterium tuberculosis;	NCBI: 1423438	4420731	99.94	0.00	Yes

NCBI Assembly	GCA_000680155.1	NCBI: cellular organisms; Bacteria; Terrabacteria group; Actinobacteria; Actinomycetia; Corynebacteriales; Mycobacteriaceae; Mycobacterium; Mycobacterium tuberculosis complex; Mycobacterium tuberculosis;	NCBI: 1427192	4421038	99.94	0.00	Yes
NCBI Assembly	GCA_000709975.1	NCBI: cellular organisms; Bacteria; Terrabacteria group; Actinobacteria; Actinomycetia; Corynebacteriales; Mycobacteriaceae; Mycobacterium; Mycobacterium tuberculosis complex; Mycobacterium tuberculosis;	NCBI: 1773	4332044	99.01	0.07	Yes
NCBI Assembly	GCA_000689255.1	NCBI: cellular organisms; Bacteria; Terrabacteria group; Actinobacteria; Actinomycetia; Corynebacteriales; Mycobacteriaceae; Mycobacterium; Mycobacterium simiae complex; Mycobacterium triplex;	NCBI: 47839	6382840	99.53	0.56	Yes
NCBI Assembly	GCA_000679835.1	NCBI: cellular organisms; Bacteria; Terrabacteria group; Actinobacteria; Actinomycetia; Corynebacteriales; Mycobacteriaceae; Mycobacterium; Mycobacterium tuberculosis complex; Mycobacterium tuberculosis;	NCBI: 1423576	4405656	99.94	0.00	Yes
NCBI Assembly	GCA_000670615.1	NCBI: cellular organisms; Bacteria; Terrabacteria group; Actinobacteria; Actinomycetia; Corynebacteriales; Mycobacteriaceae; Mycobacterium; Mycobacterium tuberculosis complex; Mycobacterium tuberculosis;	NCBI: 1421955	4427088	99.94	0.00	Yes
NCBI Assembly	GCA_000677675.1	NCBI: cellular organisms; Bacteria; Terrabacteria group; Actinobacteria; Actinomycetia; Corynebacteriales; Mycobacteriaceae; Mycobacterium; Mycobacterium tuberculosis complex; Mycobacterium tuberculosis;	NCBI: 1423455	4400093	99.94	0.17	Yes
NCBI Assembly	GCA_000681095.1	NCBI: cellular organisms; Bacteria; Terrabacteria group; Actinobacteria; Actinomycetia; Corynebacteriales; Mycobacteriaceae; Mycobacterium; Mycobacterium tuberculosis complex; Mycobacterium tuberculosis;	NCBI: 1427245	4441744	99.94	0.00	Yes
NCBI Assembly	GCA_000676095.1	NCBI: cellular organisms; Bacteria; Terrabacteria group; Actinobacteria; Actinomycetia; Corynebacteriales; Mycobacteriaceae; Mycobacterium; Mycobacterium tuberculosis complex; Mycobacterium tuberculosis;	NCBI: 1422058	4425347	99.94	0.00	Yes
NCBI Assembly	GCA_000705845.1	NCBI: cellular organisms; Bacteria; Terrabacteria group; Actinobacteria; Actinomycetia; Corynebacteriales; Mycobacteriaceae; Mycobacterium; Mycobacterium tuberculosis complex; Mycobacterium tuberculosis;	NCBI: 1438850	4456345	99.94	2.54	Yes
NCBI Assembly	GCA_000678595.1	NCBI: cellular organisms; Bacteria; Terrabacteria group; Actinobacteria; Actinomycetia; Corynebacteriales; Mycobacteriaceae; Mycobacterium; Mycobacterium tuberculosis complex; Mycobacterium tuberculosis;	NCBI: 1423507	4401441	99.94	0.00	Yes
NCBI Assembly	GCA_000678915.1	NCBI: cellular organisms; Bacteria; Terrabacteria group; Actinobacteria; Actinomycetia; Corynebacteriales; Mycobacteriaceae; Mycobacterium; Mycobacterium tuberculosis complex; Mycobacterium tuberculosis;	NCBI: 1423529	4444271	99.94	0.00	Yes
NCBI Assembly	GCA_000680175.1	NCBI: cellular organisms; Bacteria; Terrabacteria group; Actinobacteria; Actinomycetia; Corynebacteriales; Mycobacteriaceae; Mycobacterium; Mycobacterium tuberculosis complex; Mycobacterium tuberculosis;	NCBI: 1427193	4382625	99.94	0.44	Yes
NCBI Assembly	GCA_000677355.1	NCBI: cellular organisms; Bacteria; Terrabacteria group; Actinobacteria; Actinomycetia; Corynebacteriales; Mycobacteriaceae; Mycobacterium; Mycobacterium tuberculosis complex; Mycobacterium tuberculosis;	NCBI: 1423439	4401520	99.94	0.00	Yes
NCBI Assembly	GCA_000679855.1	NCBI: cellular organisms; Bacteria; Terrabacteria group; Actinobacteria; Actinomycetia; Corynebacteriales; Mycobacteriaceae; Mycobacterium; Mycobacterium tuberculosis complex; Mycobacterium tuberculosis;	NCBI: 1423577	4414713	99.94	0.00	Yes
NCBI Assembly	GCA_000689535.1	NCBI: cellular organisms; Bacteria; Terrabacteria group; Actinobacteria; Actinomycetia; Corynebacteriales; Mycobacteriaceae; Mycobacterium; Mycobacterium tuberculosis complex; Mycobacterium tuberculosis;	NCBI: 1773	4224610	99.28	0.00	Yes
NCBI Assembly	GCA_000710055.1	NCBI: cellular organisms; Bacteria; Terrabacteria group; Actinobacteria; Actinomycetia; Corynebacteriales; Mycobacteriaceae; Mycobacterium; Mycobacterium tuberculosis complex; Mycobacterium tuberculosis;	NCBI: 1773	4327456	99.36	0.00	Yes
NCBI Assembly	GCA_000677695.1	NCBI: cellular organisms; Bacteria; Terrabacteria group; Actinobacteria; Actinomycetia; Corynebacteriales; Mycobacteriaceae; Mycobacterium; Mycobacterium tuberculosis complex; Mycobacterium tuberculosis;	NCBI: 1423456	4422353	99.94	0.00	Yes
NCBI Assembly	GCA_000670635.1	NCBI: cellular organisms; Bacteria; Terrabacteria group; Actinobacteria; Actinomycetia; Corynebacteriales; Mycobacteriaceae; Mycobacterium; Mycobacterium tuberculosis complex; Mycobacterium tuberculosis;	NCBI: 1421956	4417816	99.94	0.00	Yes

NCBI Assembly	GCA_000681115.1	NCBI: cellular organisms; Bacteria; Terrabacteria group; Actinobacteria; Actinomycetia; Corynebacteriales; Mycobacteriaceae; Mycobacterium; Mycobacterium tuberculosis complex; Mycobacterium tuberculosis;	NCBI: 1427246	4424708	99.94	0.00	Yes
NCBI Assembly	GCA_000676115.1	NCBI: cellular organisms; Bacteria; Terrabacteria group; Actinobacteria; Actinomycetia; Corynebacteriales; Mycobacteriaceae; Mycobacterium; Mycobacterium tuberculosis complex; Mycobacterium tuberculosis;	NCBI: 1422059	4388384	99.94	0.00	Yes
NCBI Assembly	GCA_000705865.1	NCBI: cellular organisms; Bacteria; Terrabacteria group; Actinobacteria; Actinomycetia; Corynebacteriales; Mycobacteriaceae; Mycobacterium; Mycobacterium tuberculosis complex; Mycobacterium tuberculosis;	NCBI: 1438861	4453263	99.94	1.89	Yes
NCBI Assembly	GCA_000678615.1	NCBI: cellular organisms; Bacteria; Terrabacteria group; Actinobacteria; Actinomycetia; Corynebacteriales; Mycobacteriaceae; Mycobacterium; Mycobacterium tuberculosis complex; Mycobacterium tuberculosis;	NCBI: 1423508	4410404	99.94	0.00	Yes
NCBI Assembly	GCA_000680195.1	NCBI: cellular organisms; Bacteria; Terrabacteria group; Actinobacteria; Actinomycetia; Corynebacteriales; Mycobacteriaceae; Mycobacterium; Mycobacterium tuberculosis complex; Mycobacterium tuberculosis;	NCBI: 1427194	4416977	99.94	0.00	Yes
NCBI Assembly	GCA_000677375.1	NCBI: cellular organisms; Bacteria; Terrabacteria group; Actinobacteria; Actinomycetia; Corynebacteriales; Mycobacteriaceae; Mycobacterium; Mycobacterium tuberculosis complex; Mycobacterium tuberculosis;	NCBI: 1423440	4359980	99.94	0.00	Yes
NCBI Assembly	GCA_000678935.1	NCBI: cellular organisms; Bacteria; Terrabacteria group; Actinobacteria; Actinomycetia; Corynebacteriales; Mycobacteriaceae; Mycobacterium; Mycobacterium tuberculosis complex; Mycobacterium tuberculosis;	NCBI: 1423530	4397625	99.94	0.00	Yes
NCBI Assembly	GCA_000679875.1	NCBI: cellular organisms; Bacteria; Terrabacteria group; Actinobacteria; Actinomycetia; Corynebacteriales; Mycobacteriaceae; Mycobacterium; Mycobacterium tuberculosis complex; Mycobacterium tuberculosis;	NCBI: 1427177	4426457	99.94	0.00	Yes
NCBI Assembly	GCA_000710415.1	NCBI: cellular organisms; Bacteria; Terrabacteria group; Actinobacteria; Actinomycetia; Corynebacteriales; Mycobacteriaceae; Mycobacterium; Mycobacterium tuberculosis complex; Mycobacterium tuberculosis;	NCBI: 1773	4323870	99.22	0.00	Yes
NCBI Assembly	GCA_000670655.1	NCBI: cellular organisms; Bacteria; Terrabacteria group; Actinobacteria; Actinomycetia; Corynebacteriales; Mycobacteriaceae; Mycobacterium; Mycobacterium tuberculosis complex; Mycobacterium tuberculosis;	NCBI: 1421957	4410734	99.94	0.00	Yes
NCBI Assembly	GCA_000691525.1	NCBI: cellular organisms; Bacteria; Terrabacteria group; Actinobacteria; Actinomycetia; Corynebacteriales; Mycobacteriaceae; Mycolicibacterium; Mycolicibacterium neoaurum;	NCBI: 1265312	5468381	99.62	1.25	Yes
NCBI Assembly	GCA_000677715.1	NCBI: cellular organisms; Bacteria; Terrabacteria group; Actinobacteria; Actinomycetia; Corynebacteriales; Mycobacteriaceae; Mycobacterium; Mycobacterium tuberculosis complex; Mycobacterium tuberculosis;	NCBI: 1423457	4404484	99.94	0.00	Yes
NCBI Assembly	GCA_000681135.1	NCBI: cellular organisms; Bacteria; Terrabacteria group; Actinobacteria; Actinomycetia; Corynebacteriales; Mycobacteriaceae; Mycobacterium; Mycobacterium tuberculosis complex; Mycobacterium tuberculosis;	NCBI: 1427248	4418594	99.94	0.00	Yes
NCBI Assembly	GCA_000676135.1	NCBI: cellular organisms; Bacteria; Terrabacteria group; Actinobacteria; Actinomycetia; Corynebacteriales; Mycobacteriaceae; Mycobacterium; Mycobacterium tuberculosis complex; Mycobacterium tuberculosis;	NCBI: 1422060	4428948	99.35	0.97	Yes
NCBI Assembly	GCA_000705885.1	NCBI: cellular organisms; Bacteria; Terrabacteria group; Actinobacteria; Actinomycetia; Corynebacteriales; Mycobacteriaceae; Mycobacterium; Mycobacterium tuberculosis complex; Mycobacterium tuberculosis;	NCBI: 1423569	4432543	99.94	0.00	Yes
NCBI Assembly	GCA_000678635.1	NCBI: cellular organisms; Bacteria; Terrabacteria group; Actinobacteria; Actinomycetia; Corynebacteriales; Mycobacteriaceae; Mycobacterium; Mycobacterium tuberculosis complex; Mycobacterium tuberculosis;	NCBI: 1423509	4385904	99.78	0.00	Yes
NCBI Assembly	GCA_000680215.1	NCBI: cellular organisms; Bacteria; Terrabacteria group; Actinobacteria; Actinomycetia; Corynebacteriales; Mycobacteriaceae; Mycobacterium; Mycobacterium tuberculosis complex; Mycobacterium tuberculosis;	NCBI: 1427195	4388513	99.94	0.00	Yes
NCBI Assembly	GCA_000677395.1	NCBI: cellular organisms; Bacteria; Terrabacteria group; Actinobacteria; Actinomycetia; Corynebacteriales; Mycobacteriaceae; Mycobacterium; Mycobacterium tuberculosis complex; Mycobacterium tuberculosis;	NCBI: 1423441	4424854	99.94	0.00	Yes

NCBI Assembly	GCA_000678955.1	NCBI: cellular organisms; Bacteria; Terrabacteria group; Actinobacteria; Actinomycetia; Corynebacteriales; Mycobacteriaceae; Mycobacterium; Mycobacterium tuberculosis complex; Mycobacterium tuberculosis;	NCBI: 1423531	4394170	99.94	0.00	Yes
NCBI Assembly	GCA_000679895.1	NCBI: cellular organisms; Bacteria; Terrabacteria group; Actinobacteria; Actinomycetia; Corynebacteriales; Mycobacteriaceae; Mycobacterium; Mycobacterium tuberculosis complex; Mycobacterium tuberculosis;	NCBI: 1427178	4418894	99.94	0.00	Yes
NCBI Assembly	GCA_000711635.1	NCBI: cellular organisms; Bacteria; Terrabacteria group; Actinobacteria; Actinomycetia; Corynebacteriales; Mycobacteriaceae; Mycobacterium; Mycobacterium tuberculosis complex; Mycobacterium tuberculosis;	NCBI: 1438854	4412659	99.94	0.00	Yes
NCBI Assembly	GCA_000677735.1	NCBI: cellular organisms; Bacteria; Terrabacteria group; Actinobacteria; Actinomycetia; Corynebacteriales; Mycobacteriaceae; Mycobacterium; Mycobacterium tuberculosis complex; Mycobacterium tuberculosis;	NCBI: 1423458	4412915	99.94	0.00	Yes
NCBI Assembly	GCA_000670675.1	NCBI: cellular organisms; Bacteria; Terrabacteria group; Actinobacteria; Actinomycetia; Corynebacteriales; Mycobacteriaceae; Mycobacterium; Mycobacterium tuberculosis complex; Mycobacterium tuberculosis;	NCBI: 1421958	4403073	99.94	0.00	Yes
NCBI Assembly	GCA_000681155.1	NCBI: cellular organisms; Bacteria; Terrabacteria group; Actinobacteria; Actinomycetia; Corynebacteriales; Mycobacteriaceae; Mycobacterium; Mycobacterium tuberculosis complex; Mycobacterium tuberculosis;	NCBI: 1427249	4423975	99.94	0.00	Yes
NCBI Assembly	GCA_000676155.1	NCBI: cellular organisms; Bacteria; Terrabacteria group; Actinobacteria; Actinomycetia; Corynebacteriales; Mycobacteriaceae; Mycobacterium; Mycobacterium tuberculosis complex; Mycobacterium tuberculosis;	NCBI: 1422061	4419385	99.94	0.00	Yes
NCBI Assembly	GCA_000696715.1	NCBI: cellular organisms; Bacteria; Terrabacteria group; Actinobacteria; Actinomycetia; Corynebacteriales; Mycobacteriaceae; Mycobacterium; Mycobacterium avium complex (MAC); Mycobacterium avium; Mycobacterium avium subsp. hominissuis;	NCBI: 1160711	4870506	99.94	0.00	Yes
NCBI Assembly	GCA_000705905.1	NCBI: cellular organisms; Bacteria; Terrabacteria group; Actinobacteria; Actinomycetia; Corynebacteriales; Mycobacteriaceae; Mycobacterium; Mycobacterium tuberculosis complex; Mycobacterium tuberculosis;	NCBI: 1423524	4405947	99.94	0.00	Yes
NCBI Assembly	GCA_000678655.1	NCBI: cellular organisms; Bacteria; Terrabacteria group; Actinobacteria; Actinomycetia; Corynebacteriales; Mycobacteriaceae; Mycobacterium; Mycobacterium tuberculosis complex; Mycobacterium tuberculosis;	NCBI: 1423510	4406983	99.94	0.00	Yes
NCBI Assembly	GCA_000680235.1	NCBI: cellular organisms; Bacteria; Terrabacteria group; Actinobacteria; Actinomycetia; Corynebacteriales; Mycobacteriaceae; Mycobacterium; Mycobacterium tuberculosis complex; Mycobacterium tuberculosis;	NCBI: 1427196	4415073	99.94	0.00	Yes
NCBI Assembly	GCA_000678975.1	NCBI: cellular organisms; Bacteria; Terrabacteria group; Actinobacteria; Actinomycetia; Corynebacteriales; Mycobacteriaceae; Mycobacterium; Mycobacterium tuberculosis complex; Mycobacterium tuberculosis;	NCBI: 1423532	4408538	99.94	0.00	Yes
NCBI Assembly	GCA_000679915.1	NCBI: cellular organisms; Bacteria; Terrabacteria group; Actinobacteria; Actinomycetia; Corynebacteriales; Mycobacteriaceae; Mycobacterium; Mycobacterium tuberculosis complex; Mycobacterium tuberculosis;	NCBI: 1427179	4442739	99.94	0.00	Yes
NCBI Assembly	GCA_000677415.1	NCBI: cellular organisms; Bacteria; Terrabacteria group; Actinobacteria; Actinomycetia; Corynebacteriales; Mycobacteriaceae; Mycobacterium; Mycobacterium tuberculosis complex; Mycobacterium tuberculosis;	NCBI: 1423442	4407622	99.94	0.00	Yes
NCBI Assembly	GCA_000711645.1	NCBI: cellular organisms; Bacteria; Terrabacteria group; Actinobacteria; Actinomycetia; Corynebacteriales; Mycobacteriaceae; Mycobacterium; Mycobacterium tuberculosis complex; Mycobacterium tuberculosis;	NCBI: 1455292	4400599	99.61	0.00	Yes
NCBI Assembly	GCA_000677755.1	NCBI: cellular organisms; Bacteria; Terrabacteria group; Actinobacteria; Actinomycetia; Corynebacteriales; Mycobacteriaceae; Mycobacterium; Mycobacterium tuberculosis complex; Mycobacterium tuberculosis;	NCBI: 1423459	4373386	99.94	0.00	Yes
NCBI Assembly	GCA_000670695.1	NCBI: cellular organisms; Bacteria; Terrabacteria group; Actinobacteria; Actinomycetia; Corynebacteriales; Mycobacteriaceae; Mycobacterium; Mycobacterium tuberculosis complex; Mycobacterium tuberculosis;	NCBI: 1421959	4410431	99.94	0.00	Yes
NCBI Assembly	GCA_000696735.1	NCBI: cellular organisms; Bacteria; Terrabacteria group; Actinobacteria; Actinomycetia; Corynebacteriales; Mycobacteriaceae; Mycobacterium; Mycobacterium avium complex (MAC); Mycobacterium avium; Mycobacterium avium subsp. hominissuis;	NCBI: 1402966	5498713	99.94	0.78	Yes

NCBI Assembly	GCA_000676175.1	NCBI: cellular organisms; Bacteria; Terrabacteria group; Actinobacteria; Actinomycetia; Corynebacteriales; Mycobacteriaceae; Mycobacterium; Mycobacterium tuberculosis complex; Mycobacterium tuberculosis;	NCBI: 1422062	4415908	99.94	0.00	Yes
NCBI Assembly	GCA_000681175.1	NCBI: cellular organisms; Bacteria; Terrabacteria group; Actinobacteria; Actinomycetia; Corynebacteriales; Mycobacteriaceae; Mycobacterium; Mycobacterium tuberculosis complex; Mycobacterium tuberculosis;	NCBI: 1427250	4429425	99.94	0.00	Yes
NCBI Assembly	GCA_000705925.1	NCBI: cellular organisms; Bacteria; Terrabacteria group; Actinobacteria; Actinomycetia; Corynebacteriales; Mycobacteriaceae; Mycobacterium; Mycobacterium tuberculosis complex; Mycobacterium tuberculosis;	NCBI: 1423495	4411636	99.94	0.00	Yes
NCBI Assembly	GCA_000678675.1	NCBI: cellular organisms; Bacteria; Terrabacteria group; Actinobacteria; Actinomycetia; Corynebacteriales; Mycobacteriaceae; Mycobacterium; Mycobacterium tuberculosis complex; Mycobacterium tuberculosis;	NCBI: 1423511	4417130	99.94	0.00	Yes
NCBI Assembly	GCA_000680255.1	NCBI: cellular organisms; Bacteria; Terrabacteria group; Actinobacteria; Actinomycetia; Corynebacteriales; Mycobacteriaceae; Mycobacterium; Mycobacterium tuberculosis complex; Mycobacterium tuberculosis;	NCBI: 1427197	4421939	99.94	0.00	Yes
NCBI Assembly	GCA_000678995.1	NCBI: cellular organisms; Bacteria; Terrabacteria group; Actinobacteria; Actinomycetia; Corynebacteriales; Mycobacteriaceae; Mycobacterium; Mycobacterium tuberculosis complex; Mycobacterium tuberculosis;	NCBI: 1423533	4428924	99.61	0.03	Yes
NCBI Assembly	GCA_000679935.1	NCBI: cellular organisms; Bacteria; Terrabacteria group; Actinobacteria; Actinomycetia; Corynebacteriales; Mycobacteriaceae; Mycobacterium; Mycobacterium tuberculosis complex; Mycobacterium tuberculosis;	NCBI: 1427180	4410878	99.94	0.00	Yes
NCBI Assembly	GCA_000677435.1	NCBI: cellular organisms; Bacteria; Terrabacteria group; Actinobacteria; Actinomycetia; Corynebacteriales; Mycobacteriaceae; Mycobacterium; Mycobacterium tuberculosis complex; Mycobacterium tuberculosis;	NCBI: 1423443	4408859	99.83	0.00	Yes
NCBI Assembly	GCA_000677775.1	NCBI: cellular organisms; Bacteria; Terrabacteria group; Actinobacteria; Actinomycetia; Corynebacteriales; Mycobacteriaceae; Mycobacterium; Mycobacterium tuberculosis complex; Mycobacterium tuberculosis;	NCBI: 1423460	4409101	99.94	0.00	Yes
NCBI Assembly	GCA_000670715.1	NCBI: cellular organisms; Bacteria; Terrabacteria group; Actinobacteria; Actinomycetia; Corynebacteriales; Mycobacteriaceae; Mycobacterium; Mycobacterium tuberculosis complex; Mycobacterium tuberculosis;	NCBI: 1421960	4398418	99.94	0.00	Yes
NCBI Assembly	GCA_000711655.1	NCBI: cellular organisms; Bacteria; Terrabacteria group; Actinobacteria; Actinomycetia; Corynebacteriales; Mycobacteriaceae; Mycobacterium; Mycobacterium tuberculosis complex; Mycobacterium tuberculosis;	NCBI: 1447445	4382711	99.94	0.00	Yes
NCBI Assembly	GCA_000696755.1	NCBI: cellular organisms; Bacteria; Terrabacteria group; Actinobacteria; Actinomycetia; Corynebacteriales; Mycobacteriaceae; Mycobacterium; Mycobacterium avium complex (MAC); Mycobacterium avium; Mycobacterium avium subsp. hominissuis;	NCBI: 1402967	5465242	99.94	0.17	Yes
NCBI Assembly	GCA_000676195.1	NCBI: cellular organisms; Bacteria; Terrabacteria group; Actinobacteria; Actinomycetia; Corynebacteriales; Mycobacteriaceae; Mycobacterium; Mycobacterium tuberculosis complex; Mycobacterium tuberculosis;	NCBI: 1422063	4420250	99.94	0.00	Yes
NCBI Assembly	GCA_000681195.1	NCBI: cellular organisms; Bacteria; Terrabacteria group; Actinobacteria; Actinomycetia; Corynebacteriales; Mycobacteriaceae; Mycobacterium; Mycobacterium tuberculosis complex; Mycobacterium tuberculosis;	NCBI: 1427251	4406771	99.94	0.00	Yes
NCBI Assembly	GCA_000705945.1	NCBI: cellular organisms; Bacteria; Terrabacteria group; Actinobacteria; Actinomycetia; Corynebacteriales; Mycobacteriaceae; Mycobacterium; Mycobacterium tuberculosis complex; Mycobacterium tuberculosis;	NCBI: 1423477	4430843	99.94	0.00	Yes
NCBI Assembly	GCA_000678695.1	NCBI: cellular organisms; Bacteria; Terrabacteria group; Actinobacteria; Actinomycetia; Corynebacteriales; Mycobacteriaceae; Mycobacterium; Mycobacterium tuberculosis complex; Mycobacterium tuberculosis;	NCBI: 1423513	4432137	99.94	0.00	Yes
NCBI Assembly	GCA_000680275.1	NCBI: cellular organisms; Bacteria; Terrabacteria group; Actinobacteria; Actinomycetia; Corynebacteriales; Mycobacteriaceae; Mycobacterium; Mycobacterium tuberculosis complex; Mycobacterium tuberculosis;	NCBI: 1427199	4392588	99.94	0.00	Yes
NCBI Assembly	GCA_000679015.1	NCBI: cellular organisms; Bacteria; Terrabacteria group; Actinobacteria; Actinomycetia; Corynebacteriales; Mycobacteriaceae; Mycobacterium; Mycobacterium tuberculosis complex; Mycobacterium tuberculosis;	NCBI: 1423534	4402506	99.94	0.00	Yes

NCBI Assembly	GCA_000679955.1	NCBI: cellular organisms; Bacteria; Terrabacteria group; Actinobacteria; Actinomycetia; Corynebacteriales; Mycobacteriaceae; Mycobacterium; Mycobacterium tuberculosis complex; Mycobacterium tuberculosis;	NCBI: 1427181	4382734	99.94	0.00	Yes
NCBI Assembly	GCA_000677795.1	NCBI: cellular organisms; Bacteria; Terrabacteria group; Actinobacteria; Actinomycetia; Corynebacteriales; Mycobacteriaceae; Mycobacterium; Mycobacterium tuberculosis complex; Mycobacterium tuberculosis;	NCBI: 1423461	4416913	99.94	0.00	Yes
NCBI Assembly	GCA_000677455.1	NCBI: cellular organisms; Bacteria; Terrabacteria group; Actinobacteria; Actinomycetia; Corynebacteriales; Mycobacteriaceae; Mycobacterium; Mycobacterium tuberculosis complex; Mycobacterium tuberculosis;	NCBI: 1423444	4428656	99.94	0.00	Yes
NCBI Assembly	GCA_000670115.1	NCBI: cellular organisms; Bacteria; Terrabacteria group; Actinobacteria; Actinomycetia; Corynebacteriales; Mycobacteriaceae; Mycobacterium; Mycobacterium tuberculosis complex; Mycobacterium tuberculosis;	NCBI: 1421930	4416851	99.94	0.00	Yes
NCBI Assembly	GCA_000715195.2	NCBI: cellular organisms; Bacteria; Terrabacteria group; Actinobacteria; Actinomycetia; Corynebacteriales; Mycobacteriaceae; Mycobacterium; Mycobacterium tuberculosis complex; Mycobacterium tuberculosis;	NCBI: 1773	4369955	99.94	0.00	Yes
NCBI Assembly	GCA_000675595.1	NCBI: cellular organisms; Bacteria; Terrabacteria group; Actinobacteria; Actinomycetia; Corynebacteriales; Mycobacteriaceae; Mycobacterium; Mycobacterium tuberculosis complex; Mycobacterium tuberculosis;	NCBI: 1422033	4410673	99.94	0.00	Yes
NCBI Assembly	GCA_000696775.1	NCBI: cellular organisms; Bacteria; Terrabacteria group; Actinobacteria; Actinomycetia; Corynebacteriales; Mycobacteriaceae; Mycobacterium; Mycobacterium avium complex (MAC); Mycobacterium avium; Mycobacterium avium subsp. hominissuis;	NCBI: 1160712	5051896	99.56	0.19	Yes
NCBI Assembly	GCA_000680595.1	NCBI: cellular organisms; Bacteria; Terrabacteria group; Actinobacteria; Actinomycetia; Corynebacteriales; Mycobacteriaceae; Mycobacterium; Mycobacterium tuberculosis complex; Mycobacterium tuberculosis;	NCBI: 1427218	4410010	99.94	0.00	Yes
NCBI Assembly	GCA_000705965.1	NCBI: cellular organisms; Bacteria; Terrabacteria group; Actinobacteria; Actinomycetia; Corynebacteriales; Mycobacteriaceae; Mycobacterium; Mycobacterium tuberculosis complex; Mycobacterium tuberculosis;	NCBI: 1423473	4381763	99.94	0.00	Yes
NCBI Assembly	GCA_000678095.1	NCBI: cellular organisms; Bacteria; Terrabacteria group; Actinobacteria; Actinomycetia; Corynebacteriales; Mycobacteriaceae; Mycobacterium; Mycobacterium tuberculosis complex; Mycobacterium tuberculosis;	NCBI: 1423479	4415988	99.94	0.00	Yes
NCBI Assembly	GCA_000680295.1	NCBI: cellular organisms; Bacteria; Terrabacteria group; Actinobacteria; Actinomycetia; Corynebacteriales; Mycobacteriaceae; Mycobacterium; Mycobacterium tuberculosis complex; Mycobacterium tuberculosis;	NCBI: 1427200	4433964	99.94	0.00	Yes
NCBI Assembly	GCA_000676855.1	NCBI: cellular organisms; Bacteria; Terrabacteria group; Actinobacteria; Actinomycetia; Corynebacteriales; Mycobacteriaceae; Mycobacterium; Mycobacterium tuberculosis complex; Mycobacterium tuberculosis;	NCBI: 1422096	4417223	99.94	0.00	Yes
NCBI Assembly	GCA_000679355.1	NCBI: cellular organisms; Bacteria; Terrabacteria group; Actinobacteria; Actinomycetia; Corynebacteriales; Mycobacteriaceae; Mycobacterium; Mycobacterium tuberculosis complex; Mycobacterium tuberculosis;	NCBI: 1423551	4465621	99.94	0.00	Yes
NCBI Assembly	GCA_000679035.1	NCBI: cellular organisms; Bacteria; Terrabacteria group; Actinobacteria; Actinomycetia; Corynebacteriales; Mycobacteriaceae; Mycobacterium; Mycobacterium tuberculosis complex; Mycobacterium tuberculosis;	NCBI: 1423535	4350230	99.94	0.00	Yes
NCBI Assembly	GCA_000677815.1	NCBI: cellular organisms; Bacteria; Terrabacteria group; Actinobacteria; Actinomycetia; Corynebacteriales; Mycobacteriaceae; Mycobacterium; Mycobacterium tuberculosis complex; Mycobacterium tuberculosis;	NCBI: 1423462	4423841	99.94	0.00	Yes
NCBI Assembly	GCA_000670135.1	NCBI: cellular organisms; Bacteria; Terrabacteria group; Actinobacteria; Actinomycetia; Corynebacteriales; Mycobacteriaceae; Mycobacterium; Mycobacterium tuberculosis complex; Mycobacterium tuberculosis;	NCBI: 1421931	4408825	99.94	0.00	Yes
NCBI Assembly	GCA_000675615.1	NCBI: cellular organisms; Bacteria; Terrabacteria group; Actinobacteria; Actinomycetia; Corynebacteriales; Mycobacteriaceae; Mycobacterium; Mycobacterium tuberculosis complex; Mycobacterium tuberculosis;	NCBI: 1422034	4403603	99.94	0.00	Yes
NCBI Assembly	GCA_000715255.1	NCBI: cellular organisms; Bacteria; Terrabacteria group; Actinobacteria; Actinomycetia; Corynebacteriales; Mycobacteriaceae; Mycobacterium; Mycobacterium tuberculosis complex; Mycobacterium tuberculosis;	NCBI: 1773	4293333	98.90	0.00	Yes

NCBI Assembly	GCA_000674975.1	NCBI: cellular organisms; Bacteria; Terrabacteria group; Actinobacteria; Actinomycetia; Corynebacteriales; Mycobacteriaceae; Mycobacterium; Mycobacterium tuberculosis complex; Mycobacterium tuberculosis;	NCBI: 1421999	4421977	99.94	0.00	Yes
NCBI Assembly	GCA_000680615.1	NCBI: cellular organisms; Bacteria; Terrabacteria group; Actinobacteria; Actinomycetia; Corynebacteriales; Mycobacteriaceae; Mycobacterium; Mycobacterium tuberculosis complex; Mycobacterium tuberculosis;	NCBI: 1427219	4408488	99.94	0.00	Yes
NCBI Assembly	GCA_000678115.1	NCBI: cellular organisms; Bacteria; Terrabacteria group; Actinobacteria; Actinomycetia; Corynebacteriales; Mycobacteriaceae; Mycobacterium; Mycobacterium tuberculosis complex; Mycobacterium tuberculosis;	NCBI: 1423480	4407659	99.94	0.00	Yes
NCBI Assembly	GCA_000749665.1	NCBI: cellular organisms; Bacteria; Terrabacteria group; Actinobacteria; Actinomycetia; Corynebacteriales; Mycobacteriaceae; Mycobacterium; Mycobacterium tuberculosis complex; Mycobacterium tuberculosis; Mycobacterium tuberculosis variant bovis;	NCBI: 1765	4275484	99.78	0.00	Yes
NCBI Assembly	GCA_000680315.1	NCBI: cellular organisms; Bacteria; Terrabacteria group; Actinobacteria; Actinomycetia; Corynebacteriales; Mycobacteriaceae; Mycobacterium; Mycobacterium tuberculosis complex; Mycobacterium tuberculosis;	NCBI: 1427203	4427665	99.94	0.00	Yes
NCBI Assembly	GCA_000679055.1	NCBI: cellular organisms; Bacteria; Terrabacteria group; Actinobacteria; Actinomycetia; Corynebacteriales; Mycobacteriaceae; Mycobacterium; Mycobacterium tuberculosis complex; Mycobacterium tuberculosis;	NCBI: 1423536	4400556	99.94	0.00	Yes
NCBI Assembly	GCA_000676875.1	NCBI: cellular organisms; Bacteria; Terrabacteria group; Actinobacteria; Actinomycetia; Corynebacteriales; Mycobacteriaceae; Mycobacterium; Mycobacterium tuberculosis complex; Mycobacterium tuberculosis;	NCBI: 1422097	4415485	99.94	0.00	Yes
NCBI Assembly	GCA_000679375.1	NCBI: cellular organisms; Bacteria; Terrabacteria group; Actinobacteria; Actinomycetia; Corynebacteriales; Mycobacteriaceae; Mycobacterium; Mycobacterium tuberculosis complex; Mycobacterium tuberculosis;	NCBI: 1423552	4408139	99.94	0.00	Yes
NCBI Assembly	GCA_000677835.1	NCBI: cellular organisms; Bacteria; Terrabacteria group; Actinobacteria; Actinomycetia; Corynebacteriales; Mycobacteriaceae; Mycobacterium; Mycobacterium tuberculosis complex; Mycobacterium tuberculosis;	NCBI: 1423463	4402212	99.94	0.00	Yes
NCBI Assembly	GCA_000675635.1	NCBI: cellular organisms; Bacteria; Terrabacteria group; Actinobacteria; Actinomycetia; Corynebacteriales; Mycobacteriaceae; Mycobacterium; Mycobacterium tuberculosis complex; Mycobacterium tuberculosis;	NCBI: 1422035	4409034	99.94	0.00	Yes
NCBI Assembly	GCA_000670155.1	NCBI: cellular organisms; Bacteria; Terrabacteria group; Actinobacteria; Actinomycetia; Corynebacteriales; Mycobacteriaceae; Mycobacterium; Mycobacterium tuberculosis complex; Mycobacterium tuberculosis;	NCBI: 1421932	4407119	99.94	0.00	Yes
NCBI Assembly	GCA_000715335.1	NCBI: cellular organisms; Bacteria; Terrabacteria group; Actinobacteria; Actinomycetia; Corynebacteriales; Mycobacteriaceae; Mycobacteroides; Mycobacteroides abscessus;	NCBI: 1335413	5424471	99.83	4.92	Yes
NCBI Assembly	GCA_000680635.1	NCBI: cellular organisms; Bacteria; Terrabacteria group; Actinobacteria; Actinomycetia; Corynebacteriales; Mycobacteriaceae; Mycobacterium; Mycobacterium tuberculosis complex; Mycobacterium tuberculosis;	NCBI: 1427221	4419836	99.94	0.00	Yes
NCBI Assembly	GCA_000678135.1	NCBI: cellular organisms; Bacteria; Terrabacteria group; Actinobacteria; Actinomycetia; Corynebacteriales; Mycobacteriaceae; Mycobacterium; Mycobacterium tuberculosis complex; Mycobacterium tuberculosis;	NCBI: 1423481	4384176	99.94	0.08	Yes
NCBI Assembly	GCA_000674995.1	NCBI: cellular organisms; Bacteria; Terrabacteria group; Actinobacteria; Actinomycetia; Corynebacteriales; Mycobacteriaceae; Mycobacterium; Mycobacterium tuberculosis complex; Mycobacterium tuberculosis;	NCBI: 1422000	4402578	99.94	0.00	Yes
NCBI Assembly	GCA_000749675.1	NCBI: cellular organisms; Bacteria; Terrabacteria group; Actinobacteria; Actinomycetia; Corynebacteriales; Mycobacteriaceae; Mycobacterium; Mycobacterium tuberculosis complex; Mycobacterium tuberculosis; Mycobacterium tuberculosis variant bovis;	NCBI: 1765	4311106	99.78	0.00	Yes
NCBI Assembly	GCA_000680335.1	NCBI: cellular organisms; Bacteria; Terrabacteria group; Actinobacteria; Actinomycetia; Corynebacteriales; Mycobacteriaceae; Mycobacterium; Mycobacterium tuberculosis complex; Mycobacterium tuberculosis;	NCBI: 1427204	4436007	99.94	0.00	Yes
NCBI Assembly	GCA_000679075.1	NCBI: cellular organisms; Bacteria; Terrabacteria group; Actinobacteria; Actinomycetia; Corynebacteriales; Mycobacteriaceae; Mycobacterium; Mycobacterium tuberculosis complex; Mycobacterium tuberculosis;	NCBI: 1423537	4386466	99.72	0.00	Yes

NCBI Assembly	GCA_000676895.1	NCBI: cellular organisms; Bacteria; Terrabacteria group; Actinobacteria; Actinomycetia; Corynebacteriales; Mycobacteriaceae; Mycobacterium; Mycobacterium tuberculosis complex; Mycobacterium tuberculosis;	NCBI: 1422098	4396882	99.61	0.00	Yes
NCBI Assembly	GCA_000679395.1	NCBI: cellular organisms; Bacteria; Terrabacteria group; Actinobacteria; Actinomycetia; Corynebacteriales; Mycobacteriaceae; Mycobacterium; Mycobacterium tuberculosis complex; Mycobacterium tuberculosis;	NCBI: 1423553	4414701	99.61	0.00	Yes
NCBI Assembly	GCA_000675655.1	NCBI: cellular organisms; Bacteria; Terrabacteria group; Actinobacteria; Actinomycetia; Corynebacteriales; Mycobacteriaceae; Mycobacterium; Mycobacterium tuberculosis complex; Mycobacterium tuberculosis;	NCBI: 1422036	4423543	99.94	0.00	Yes
NCBI Assembly	GCA_000677855.1	NCBI: cellular organisms; Bacteria; Terrabacteria group; Actinobacteria; Actinomycetia; Corynebacteriales; Mycobacteriaceae; Mycobacterium; Mycobacterium tuberculosis complex; Mycobacterium tuberculosis;	NCBI: 1423465	4402512	99.94	0.00	Yes
NCBI Assembly	GCA_000670175.1	NCBI: cellular organisms; Bacteria; Terrabacteria group; Actinobacteria; Actinomycetia; Corynebacteriales; Mycobacteriaceae; Mycobacterium; Mycobacterium tuberculosis complex; Mycobacterium tuberculosis;	NCBI: 1421933	4414305	99.94	0.00	Yes
NCBI Assembly	GCA_000715375.1	NCBI: cellular organisms; Bacteria; Terrabacteria group; Actinobacteria; Actinomycetia; Corynebacteriales; Mycobacteriaceae; Mycobacterium; Mycobacterium kansasii;	NCBI: 1768	6572554	99.94	0.38	Yes
NCBI Assembly	GCA_000680655.1	NCBI: cellular organisms; Bacteria; Terrabacteria group; Actinobacteria; Actinomycetia; Corynebacteriales; Mycobacteriaceae; Mycobacterium; Mycobacterium tuberculosis complex; Mycobacterium tuberculosis;	NCBI: 1427222	4400214	99.94	0.00	Yes
NCBI Assembly	GCA_000675015.1	NCBI: cellular organisms; Bacteria; Terrabacteria group; Actinobacteria; Actinomycetia; Corynebacteriales; Mycobacteriaceae; Mycobacterium; Mycobacterium tuberculosis complex; Mycobacterium tuberculosis;	NCBI: 1422001	4412976	99.94	0.00	Yes
NCBI Assembly	GCA_000678155.1	NCBI: cellular organisms; Bacteria; Terrabacteria group; Actinobacteria; Actinomycetia; Corynebacteriales; Mycobacteriaceae; Mycobacterium; Mycobacterium tuberculosis complex; Mycobacterium tuberculosis;	NCBI: 1423482	4420783	99.94	0.00	Yes
NCBI Assembly	GCA_000680355.1	NCBI: cellular organisms; Bacteria; Terrabacteria group; Actinobacteria; Actinomycetia; Corynebacteriales; Mycobacteriaceae; Mycobacterium; Mycobacterium tuberculosis complex; Mycobacterium tuberculosis;	NCBI: 1427205	4425552	99.78	0.00	Yes
NCBI Assembly	GCA_000676915.1	NCBI: cellular organisms; Bacteria; Terrabacteria group; Actinobacteria; Actinomycetia; Corynebacteriales; Mycobacteriaceae; Mycobacterium; Mycobacterium tuberculosis complex; Mycobacterium tuberculosis;	NCBI: 1422100	4389272	99.94	0.40	Yes
NCBI Assembly	GCA_000749685.1	NCBI: cellular organisms; Bacteria; Terrabacteria group; Actinobacteria; Actinomycetia; Corynebacteriales; Mycobacteriaceae; Mycobacterium; Mycobacterium tuberculosis complex; Mycobacterium tuberculosis; Mycobacterium tuberculosis variant bovis;	NCBI: 1765	4308937	99.78	0.00	Yes
NCBI Assembly	GCA_000679095.1	NCBI: cellular organisms; Bacteria; Terrabacteria group; Actinobacteria; Actinomycetia; Corynebacteriales; Mycobacteriaceae; Mycobacterium; Mycobacterium tuberculosis complex; Mycobacterium tuberculosis;	NCBI: 1423538	4405085	99.94	0.00	Yes
NCBI Assembly	GCA_000679415.1	NCBI: cellular organisms; Bacteria; Terrabacteria group; Actinobacteria; Actinomycetia; Corynebacteriales; Mycobacteriaceae; Mycobacterium; Mycobacterium tuberculosis complex; Mycobacterium tuberculosis;	NCBI: 1423554	4420332	99.94	0.00	Yes
NCBI Assembly	GCA_000675675.1	NCBI: cellular organisms; Bacteria; Terrabacteria group; Actinobacteria; Actinomycetia; Corynebacteriales; Mycobacteriaceae; Mycobacterium; Mycobacterium tuberculosis complex; Mycobacterium tuberculosis;	NCBI: 1422037	4405516	99.94	0.00	Yes
NCBI Assembly	GCA_000677875.1	NCBI: cellular organisms; Bacteria; Terrabacteria group; Actinobacteria; Actinomycetia; Corynebacteriales; Mycobacteriaceae; Mycobacterium; Mycobacterium tuberculosis complex; Mycobacterium tuberculosis;	NCBI: 1423466	4414366	99.94	0.00	Yes
NCBI Assembly	GCA_000670195.1	NCBI: cellular organisms; Bacteria; Terrabacteria group; Actinobacteria; Actinomycetia; Corynebacteriales; Mycobacteriaceae; Mycobacterium; Mycobacterium tuberculosis complex; Mycobacterium tuberculosis;	NCBI: 1421934	4400675	99.94	0.00	Yes
NCBI Assembly	GCA_000675035.1	NCBI: cellular organisms; Bacteria; Terrabacteria group; Actinobacteria; Actinomycetia; Corynebacteriales; Mycobacteriaceae; Mycobacterium; Mycobacterium tuberculosis complex; Mycobacterium tuberculosis;	NCBI: 1422002	4432314	99.94	0.00	Yes

NCBI Assembly	GCA_000715415.1	NCBI: cellular organisms; Bacteria; Terrabacteria group; Actinobacteria; Actinomycetia; Corynebacteriales; Mycobacteriaceae; Mycobacterium; Mycobacterium tuberculosis complex; Mycobacterium tuberculosis;	NCBI: 1516074	4342964	99.94	0.00	Yes
NCBI Assembly	GCA_000678175.1	NCBI: cellular organisms; Bacteria; Terrabacteria group; Actinobacteria; Actinomycetia; Corynebacteriales; Mycobacteriaceae; Mycobacterium; Mycobacterium tuberculosis complex; Mycobacterium tuberculosis;	NCBI: 1423483	4409874	99.94	0.00	Yes
NCBI Assembly	GCA_000680675.1	NCBI: cellular organisms; Bacteria; Terrabacteria group; Actinobacteria; Actinomycetia; Corynebacteriales; Mycobacteriaceae; Mycobacterium; Mycobacterium tuberculosis complex; Mycobacterium tuberculosis;	NCBI: 1427223	4424641	99.94	0.00	Yes
NCBI Assembly	GCA_000680375.1	NCBI: cellular organisms; Bacteria; Terrabacteria group; Actinobacteria; Actinomycetia; Corynebacteriales; Mycobacteriaceae; Mycobacterium; Mycobacterium tuberculosis complex; Mycobacterium tuberculosis;	NCBI: 1427206	4426352	99.94	0.00	Yes
NCBI Assembly	GCA_000676935.1	NCBI: cellular organisms; Bacteria; Terrabacteria group; Actinobacteria; Actinomycetia; Corynebacteriales; Mycobacteriaceae; Mycobacterium; Mycobacterium tuberculosis complex; Mycobacterium tuberculosis;	NCBI: 1422103	4438227	99.94	0.00	Yes
NCBI Assembly	GCA_000679115.1	NCBI: cellular organisms; Bacteria; Terrabacteria group; Actinobacteria; Actinomycetia; Corynebacteriales; Mycobacteriaceae; Mycobacterium; Mycobacterium tuberculosis complex; Mycobacterium tuberculosis;	NCBI: 1423539	4410442	99.94	0.00	Yes
NCBI Assembly	GCA_000749725.1	NCBI: cellular organisms; Bacteria; Terrabacteria group; Actinobacteria; Actinomycetia; Corynebacteriales; Mycobacteriaceae; Mycobacterium; Mycobacterium tuberculosis complex; Mycobacterium tuberculosis; Mycobacterium tuberculosis variant bovis;	NCBI: 1765	4325986	99.78	0.00	Yes
NCBI Assembly	GCA_000675695.1	NCBI: cellular organisms; Bacteria; Terrabacteria group; Actinobacteria; Actinomycetia; Corynebacteriales; Mycobacteriaceae; Mycobacterium; Mycobacterium tuberculosis complex; Mycobacterium tuberculosis;	NCBI: 1422038	4419864	99.94	0.00	Yes
NCBI Assembly	GCA_000679435.1	NCBI: cellular organisms; Bacteria; Terrabacteria group; Actinobacteria; Actinomycetia; Corynebacteriales; Mycobacteriaceae; Mycobacterium; Mycobacterium tuberculosis complex; Mycobacterium tuberculosis;	NCBI: 1423555	4406243	99.94	0.17	Yes
NCBI Assembly	GCA_000677895.1	NCBI: cellular organisms; Bacteria; Terrabacteria group; Actinobacteria; Actinomycetia; Corynebacteriales; Mycobacteriaceae; Mycobacterium; Mycobacterium tuberculosis complex; Mycobacterium tuberculosis;	NCBI: 1423467	4408087	99.94	0.00	Yes
NCBI Assembly	GCA_000670215.1	NCBI: cellular organisms; Bacteria; Terrabacteria group; Actinobacteria; Actinomycetia; Corynebacteriales; Mycobacteriaceae; Mycobacterium; Mycobacterium tuberculosis complex; Mycobacterium tuberculosis;	NCBI: 1421935	4416002	99.94	0.00	Yes
NCBI Assembly	GCA_000723385.1	NCBI: cellular organisms; Bacteria; Terrabacteria group; Actinobacteria; Actinomycetia; Corynebacteriales; Mycobacteriaceae; Mycolicibacterium; Mycolicibacterium farcinogenes;	NCBI: 1802	6139893	98.95	0.94	Yes
NCBI Assembly	GCA_000675055.1	NCBI: cellular organisms; Bacteria; Terrabacteria group; Actinobacteria; Actinomycetia; Corynebacteriales; Mycobacteriaceae; Mycobacterium; Mycobacterium tuberculosis complex; Mycobacterium tuberculosis;	NCBI: 1422003	4405220	99.94	0.00	Yes
NCBI Assembly	GCA_000678195.1	NCBI: cellular organisms; Bacteria; Terrabacteria group; Actinobacteria; Actinomycetia; Corynebacteriales; Mycobacteriaceae; Mycobacterium; Mycobacterium tuberculosis complex; Mycobacterium tuberculosis;	NCBI: 1423484	4412732	99.94	0.00	Yes
NCBI Assembly	GCA_000680695.1	NCBI: cellular organisms; Bacteria; Terrabacteria group; Actinobacteria; Actinomycetia; Corynebacteriales; Mycobacteriaceae; Mycobacterium; Mycobacterium tuberculosis complex; Mycobacterium tuberculosis;	NCBI: 1427224	4420308	99.94	0.00	Yes
NCBI Assembly	GCA_000680395.1	NCBI: cellular organisms; Bacteria; Terrabacteria group; Actinobacteria; Actinomycetia; Corynebacteriales; Mycobacteriaceae; Mycobacterium; Mycobacterium tuberculosis complex; Mycobacterium tuberculosis;	NCBI: 1427207	4385118	99.94	0.00	Yes
NCBI Assembly	GCA_000676955.1	NCBI: cellular organisms; Bacteria; Terrabacteria group; Actinobacteria; Actinomycetia; Corynebacteriales; Mycobacteriaceae; Mycobacterium; Mycobacterium tuberculosis complex; Mycobacterium tuberculosis;	NCBI: 1422104	4416715	99.94	0.00	Yes
NCBI Assembly	GCA_000679135.1	NCBI: cellular organisms; Bacteria; Terrabacteria group; Actinobacteria; Actinomycetia; Corynebacteriales; Mycobacteriaceae; Mycobacterium; Mycobacterium tuberculosis complex; Mycobacterium tuberculosis;	NCBI: 1423540	4416866	99.94	0.00	Yes

NCBI Assembly	GCA_000749745.1	NCBI: cellular organisms; Bacteria; Terrabacteria group; Actinobacteria; Actinomycetia; Corynebacteriales; Mycobacteriaceae; Mycobacterium; Mycobacterium tuberculosis complex; Mycobacterium tuberculosis; Mycobacterium tuberculosis variant bovis;	NCBI: 1765	4301774	99.78	0.00	Yes
NCBI Assembly	GCA_000679455.1	NCBI: cellular organisms; Bacteria; Terrabacteria group; Actinobacteria; Actinomycetia; Corynebacteriales; Mycobacteriaceae; Mycobacterium; Mycobacterium tuberculosis complex; Mycobacterium tuberculosis;	NCBI: 1423556	4419319	99.94	0.00	Yes
NCBI Assembly	GCA_000677915.1	NCBI: cellular organisms; Bacteria; Terrabacteria group; Actinobacteria; Actinomycetia; Corynebacteriales; Mycobacteriaceae; Mycobacterium; Mycobacterium tuberculosis complex; Mycobacterium tuberculosis;	NCBI: 1423468	4407216	99.94	0.00	Yes
NCBI Assembly	GCA_000675715.1	NCBI: cellular organisms; Bacteria; Terrabacteria group; Actinobacteria; Actinomycetia; Corynebacteriales; Mycobacteriaceae; Mycobacterium; Mycobacterium tuberculosis complex; Mycobacterium tuberculosis;	NCBI: 1422039	4435448	99.94	0.00	Yes
NCBI Assembly	GCA_000670235.1	NCBI: cellular organisms; Bacteria; Terrabacteria group; Actinobacteria; Actinomycetia; Corynebacteriales; Mycobacteriaceae; Mycobacterium; Mycobacterium tuberculosis complex; Mycobacterium tuberculosis;	NCBI: 1421936	4413168	99.94	0.00	Yes
NCBI Assembly	GCA_000678215.1	NCBI: cellular organisms; Bacteria; Terrabacteria group; Actinobacteria; Actinomycetia; Corynebacteriales; Mycobacteriaceae; Mycobacterium; Mycobacterium tuberculosis complex; Mycobacterium tuberculosis;	NCBI: 1423485	4413555	99.94	0.00	Yes
NCBI Assembly	GCA_000680715.1	NCBI: cellular organisms; Bacteria; Terrabacteria group; Actinobacteria; Actinomycetia; Corynebacteriales; Mycobacteriaceae; Mycobacterium; Mycobacterium tuberculosis complex; Mycobacterium tuberculosis;	NCBI: 1427225	4389183	99.94	0.00	Yes
NCBI Assembly	GCA_000675075.1	NCBI: cellular organisms; Bacteria; Terrabacteria group; Actinobacteria; Actinomycetia; Corynebacteriales; Mycobacteriaceae; Mycobacterium; Mycobacterium tuberculosis complex; Mycobacterium tuberculosis;	NCBI: 1422004	4403102	99.94	0.00	Yes
NCBI Assembly	GCA_000680415.1	NCBI: cellular organisms; Bacteria; Terrabacteria group; Actinobacteria; Actinomycetia; Corynebacteriales; Mycobacteriaceae; Mycobacterium; Mycobacterium tuberculosis complex; Mycobacterium tuberculosis;	NCBI: 1427208	4428140	99.94	0.00	Yes
NCBI Assembly	GCA_000723425.2	NCBI: cellular organisms; Bacteria; Terrabacteria group; Actinobacteria; Actinomycetia; Corynebacteriales; Mycobacteriaceae; Mycobacterium; Mycobacterium marinum;	NCBI: 1131442	6450522	99.88	0.23	Yes
NCBI Assembly	GCA_000676975.1	NCBI: cellular organisms; Bacteria; Terrabacteria group; Actinobacteria; Actinomycetia; Corynebacteriales; Mycobacteriaceae; Mycobacterium; Mycobacterium tuberculosis complex; Mycobacterium tuberculosis;	NCBI: 1422105	4412944	99.94	0.00	Yes
NCBI Assembly	GCA_000679155.1	NCBI: cellular organisms; Bacteria; Terrabacteria group; Actinobacteria; Actinomycetia; Corynebacteriales; Mycobacteriaceae; Mycobacterium; Mycobacterium tuberculosis complex; Mycobacterium tuberculosis;	NCBI: 1423541	4402190	99.94	0.00	Yes
NCBI Assembly	GCA_000679475.1	NCBI: cellular organisms; Bacteria; Terrabacteria group; Actinobacteria; Actinomycetia; Corynebacteriales; Mycobacteriaceae; Mycobacterium; Mycobacterium tuberculosis complex; Mycobacterium tuberculosis;	NCBI: 1423557	4423466	99.94	0.67	Yes
NCBI Assembly	GCA_000749765.1	NCBI: cellular organisms; Bacteria; Terrabacteria group; Actinobacteria; Actinomycetia; Corynebacteriales; Mycobacteriaceae; Mycobacterium; Mycobacterium tuberculosis complex; Mycobacterium tuberculosis; Mycobacterium tuberculosis variant bovis;	NCBI: 1765	4332493	99.78	0.00	Yes
NCBI Assembly	GCA_000677935.1	NCBI: cellular organisms; Bacteria; Terrabacteria group; Actinobacteria; Actinomycetia; Corynebacteriales; Mycobacteriaceae; Mycobacterium; Mycobacterium tuberculosis complex; Mycobacterium tuberculosis;	NCBI: 1423469	4399575	99.94	0.00	Yes
NCBI Assembly	GCA_000675735.1	NCBI: cellular organisms; Bacteria; Terrabacteria group; Actinobacteria; Actinomycetia; Corynebacteriales; Mycobacteriaceae; Mycobacterium; Mycobacterium tuberculosis complex; Mycobacterium tuberculosis;	NCBI: 1422040	4426864	99.94	0.00	Yes
NCBI Assembly	GCA_000670255.1	NCBI: cellular organisms; Bacteria; Terrabacteria group; Actinobacteria; Actinomycetia; Corynebacteriales; Mycobacteriaceae; Mycobacterium; Mycobacterium tuberculosis complex; Mycobacterium tuberculosis;	NCBI: 1421937	4404990	99.94	0.00	Yes
NCBI Assembly	GCA_000678235.1	NCBI: cellular organisms; Bacteria; Terrabacteria group; Actinobacteria; Actinomycetia; Corynebacteriales; Mycobacteriaceae; Mycobacterium; Mycobacterium tuberculosis complex; Mycobacterium tuberculosis;	NCBI: 1423486	4421300	99.94	0.00	Yes

NCBI Assembly	GCA_000675095.1	NCBI: cellular organisms; Bacteria; Terrabacteria group; Actinobacteria; Actinomycetia; Corynebacteriales; Mycobacteriaceae; Mycobacterium; Mycobacterium tuberculosis complex; Mycobacterium tuberculosis;	NCBI: 1422005	4438834	99.94	0.00	Yes
NCBI Assembly	GCA_000680435.1	NCBI: cellular organisms; Bacteria; Terrabacteria group; Actinobacteria; Actinomycetia; Corynebacteriales; Mycobacteriaceae; Mycobacterium; Mycobacterium tuberculosis complex; Mycobacterium tuberculosis;	NCBI: 1427209	4420330	99.94	0.00	Yes
NCBI Assembly	GCA_000680735.1	NCBI: cellular organisms; Bacteria; Terrabacteria group; Actinobacteria; Actinomycetia; Corynebacteriales; Mycobacteriaceae; Mycobacterium; Mycobacterium tuberculosis complex; Mycobacterium tuberculosis;	NCBI: 1427226	4417398	99.94	0.00	Yes
NCBI Assembly	GCA_000676995.1	NCBI: cellular organisms; Bacteria; Terrabacteria group; Actinobacteria; Actinomycetia; Corynebacteriales; Mycobacteriaceae; Mycobacterium; Mycobacterium tuberculosis complex; Mycobacterium tuberculosis;	NCBI: 1422106	4410891	99.94	0.00	Yes
NCBI Assembly	GCA_000724065.1	NCBI: cellular organisms; Bacteria; Terrabacteria group; Actinobacteria; Actinomycetia; Corynebacteriales; Mycobacteriaceae; Mycolicibacterium; Mycolicibacterium neoaurum;	NCBI: 1795	5536033	98.96	1.56	Yes
NCBI Assembly	GCA_000679175.1	NCBI: cellular organisms; Bacteria; Terrabacteria group; Actinobacteria; Actinomycetia; Corynebacteriales; Mycobacteriaceae; Mycobacterium; Mycobacterium tuberculosis complex; Mycobacterium tuberculosis;	NCBI: 1423542	4448021	99.94	0.00	Yes
NCBI Assembly	GCA_000675755.1	NCBI: cellular organisms; Bacteria; Terrabacteria group; Actinobacteria; Actinomycetia; Corynebacteriales; Mycobacteriaceae; Mycobacterium; Mycobacterium tuberculosis complex; Mycobacterium tuberculosis;	NCBI: 1422041	4358350	99.94	0.00	Yes
NCBI Assembly	GCA_000679495.1	NCBI: cellular organisms; Bacteria; Terrabacteria group; Actinobacteria; Actinomycetia; Corynebacteriales; Mycobacteriaceae; Mycobacterium; Mycobacterium tuberculosis complex; Mycobacterium tuberculosis;	NCBI: 1423558	4403342	99.94	0.00	Yes
NCBI Assembly	GCA_000670275.1	NCBI: cellular organisms; Bacteria; Terrabacteria group; Actinobacteria; Actinomycetia; Corynebacteriales; Mycobacteriaceae; Mycobacterium; Mycobacterium tuberculosis complex; Mycobacterium tuberculosis;	NCBI: 1421938	4404320	99.94	0.00	Yes
NCBI Assembly	GCA_000677955.1	NCBI: cellular organisms; Bacteria; Terrabacteria group; Actinobacteria; Actinomycetia; Corynebacteriales; Mycobacteriaceae; Mycobacterium; Mycobacterium tuberculosis complex; Mycobacterium tuberculosis;	NCBI: 1423470	4399548	99.94	0.00	Yes
NCBI Assembly	GCA_000749785.1	NCBI: cellular organisms; Bacteria; Terrabacteria group; Actinobacteria; Actinomycetia; Corynebacteriales; Mycobacteriaceae; Mycobacterium; Mycobacterium tuberculosis complex; Mycobacterium tuberculosis; Mycobacterium tuberculosis variant bovis;	NCBI: 1765	4320055	99.78	0.00	Yes
NCBI Assembly	GCA_000678255.1	NCBI: cellular organisms; Bacteria; Terrabacteria group; Actinobacteria; Actinomycetia; Corynebacteriales; Mycobacteriaceae; Mycobacterium; Mycobacterium tuberculosis complex; Mycobacterium tuberculosis;	NCBI: 1423487	4478284	99.94	0.00	Yes
NCBI Assembly	GCA_000675115.1	NCBI: cellular organisms; Bacteria; Terrabacteria group; Actinobacteria; Actinomycetia; Corynebacteriales; Mycobacteriaceae; Mycobacterium; Mycobacterium tuberculosis complex; Mycobacterium tuberculosis;	NCBI: 1422006	4438880	99.94	0.00	Yes
NCBI Assembly	GCA_000680455.1	NCBI: cellular organisms; Bacteria; Terrabacteria group; Actinobacteria; Actinomycetia; Corynebacteriales; Mycobacteriaceae; Mycobacterium; Mycobacterium tuberculosis complex; Mycobacterium tuberculosis;	NCBI: 1427210	4402297	99.94	0.00	Yes
NCBI Assembly	GCA_000680755.1	NCBI: cellular organisms; Bacteria; Terrabacteria group; Actinobacteria; Actinomycetia; Corynebacteriales; Mycobacteriaceae; Mycobacterium; Mycobacterium tuberculosis complex; Mycobacterium tuberculosis;	NCBI: 1427227	4431230	99.94	0.00	Yes
NCBI Assembly	GCA_000677015.1	NCBI: cellular organisms; Bacteria; Terrabacteria group; Actinobacteria; Actinomycetia; Corynebacteriales; Mycobacteriaceae; Mycobacterium; Mycobacterium tuberculosis complex; Mycobacterium tuberculosis;	NCBI: 1422107	4417422	99.94	0.00	Yes
NCBI Assembly	GCA_000679195.1	NCBI: cellular organisms; Bacteria; Terrabacteria group; Actinobacteria; Actinomycetia; Corynebacteriales; Mycobacteriaceae; Mycobacterium; Mycobacterium tuberculosis complex; Mycobacterium tuberculosis;	NCBI: 1423543	4447044	99.94	0.00	Yes
NCBI Assembly	GCA_000729745.1	NCBI: cellular organisms; Bacteria; Terrabacteria group; Actinobacteria; Actinomycetia; Corynebacteriales; Mycobacteriaceae; Mycobacterium; Mycobacterium tuberculosis complex; Mycobacterium tuberculosis; Mycobacterium tuberculosis variant bovis;	NCBI: 1765	4327459	99.78	0.00	Yes

NCBI Assembly	GCA_000675775.1	NCBI: cellular organisms; Bacteria; Terrabacteria group; Actinobacteria; Actinomycetia; Corynebacteriales; Mycobacteriaceae; Mycobacterium; Mycobacterium tuberculosis complex; Mycobacterium tuberculosis;	NCBI: 1422042	4413694	99.94	0.00	Yes
NCBI Assembly	GCA_000670295.1	NCBI: cellular organisms; Bacteria; Terrabacteria group; Actinobacteria; Actinomycetia; Corynebacteriales; Mycobacteriaceae; Mycobacterium; Mycobacterium tuberculosis complex; Mycobacterium tuberculosis;	NCBI: 1421939	4403129	99.94	0.00	Yes
NCBI Assembly	GCA_000677975.1	NCBI: cellular organisms; Bacteria; Terrabacteria group; Actinobacteria; Actinomycetia; Corynebacteriales; Mycobacteriaceae; Mycobacterium; Mycobacterium tuberculosis complex; Mycobacterium tuberculosis;	NCBI: 1423471	4411239	99.94	0.00	Yes
NCBI Assembly	GCA_000679515.1	NCBI: cellular organisms; Bacteria; Terrabacteria group; Actinobacteria; Actinomycetia; Corynebacteriales; Mycobacteriaceae; Mycobacterium; Mycobacterium tuberculosis complex; Mycobacterium tuberculosis;	NCBI: 1423559	4422976	99.94	0.00	Yes
NCBI Assembly	GCA_000749805.1	NCBI: cellular organisms; Bacteria; Terrabacteria group; Actinobacteria; Actinomycetia; Corynebacteriales; Mycobacteriaceae; Mycobacterium; Mycobacterium tuberculosis complex; Mycobacterium tuberculosis; Mycobacterium tuberculosis variant bovis;	NCBI: 1765	4334537	99.78	0.00	Yes
NCBI Assembly	GCA_000678275.1	NCBI: cellular organisms; Bacteria; Terrabacteria group; Actinobacteria; Actinomycetia; Corynebacteriales; Mycobacteriaceae; Mycobacterium; Mycobacterium tuberculosis complex; Mycobacterium tuberculosis;	NCBI: 1423488	4398741	99.94	0.00	Yes
NCBI Assembly	GCA_000675135.1	NCBI: cellular organisms; Bacteria; Terrabacteria group; Actinobacteria; Actinomycetia; Corynebacteriales; Mycobacteriaceae; Mycobacterium; Mycobacterium tuberculosis complex; Mycobacterium tuberculosis;	NCBI: 1422007	4410023	99.94	0.00	Yes
NCBI Assembly	GCA_000677035.1	NCBI: cellular organisms; Bacteria; Terrabacteria group; Actinobacteria; Actinomycetia; Corynebacteriales; Mycobacteriaceae; Mycobacterium; Mycobacterium tuberculosis complex; Mycobacterium tuberculosis;	NCBI: 1422108	4398645	99.94	0.00	Yes
NCBI Assembly	GCA_000680475.1	NCBI: cellular organisms; Bacteria; Terrabacteria group; Actinobacteria; Actinomycetia; Corynebacteriales; Mycobacteriaceae; Mycobacterium; Mycobacterium tuberculosis complex; Mycobacterium tuberculosis;	NCBI: 1427211	4427275	99.78	0.00	Yes
NCBI Assembly	GCA_000680775.1	NCBI: cellular organisms; Bacteria; Terrabacteria group; Actinobacteria; Actinomycetia; Corynebacteriales; Mycobacteriaceae; Mycobacterium; Mycobacterium tuberculosis complex; Mycobacterium tuberculosis;	NCBI: 1427228	4429847	99.94	0.00	Yes
NCBI Assembly	GCA_000679215.1	NCBI: cellular organisms; Bacteria; Terrabacteria group; Actinobacteria; Actinomycetia; Corynebacteriales; Mycobacteriaceae; Mycobacterium; Mycobacterium tuberculosis complex; Mycobacterium tuberculosis;	NCBI: 1423544	4355387	99.94	0.00	Yes
NCBI Assembly	GCA_000675795.1	NCBI: cellular organisms; Bacteria; Terrabacteria group; Actinobacteria; Actinomycetia; Corynebacteriales; Mycobacteriaceae; Mycobacterium; Mycobacterium tuberculosis complex; Mycobacterium tuberculosis;	NCBI: 1422043	4412783	99.94	0.00	Yes
NCBI Assembly	GCA_000670315.1	NCBI: cellular organisms; Bacteria; Terrabacteria group; Actinobacteria; Actinomycetia; Corynebacteriales; Mycobacteriaceae; Mycobacterium; Mycobacterium tuberculosis complex; Mycobacterium tuberculosis;	NCBI: 1421940	4416710	99.94	0.00	Yes
NCBI Assembly	GCA_000729755.1	NCBI: cellular organisms; Bacteria; Terrabacteria group; Actinobacteria; Actinomycetia; Corynebacteriales; Mycobacteriaceae; Mycobacterium; Mycobacterium tuberculosis complex; Mycobacterium tuberculosis; Mycobacterium tuberculosis variant bovis;	NCBI: 1765	4328889	99.78	0.00	Yes
NCBI Assembly	GCA_000677995.1	NCBI: cellular organisms; Bacteria; Terrabacteria group; Actinobacteria; Actinomycetia; Corynebacteriales; Mycobacteriaceae; Mycobacterium; Mycobacterium tuberculosis complex; Mycobacterium tuberculosis;	NCBI: 1423472	4404572	99.94	0.00	Yes
NCBI Assembly	GCA_000679535.1	NCBI: cellular organisms; Bacteria; Terrabacteria group; Actinobacteria; Actinomycetia; Corynebacteriales; Mycobacteriaceae; Mycobacterium; Mycobacterium tuberculosis complex; Mycobacterium tuberculosis;	NCBI: 1423560	4429449	99.94	0.00	Yes
NCBI Assembly	GCA_000675155.1	NCBI: cellular organisms; Bacteria; Terrabacteria group; Actinobacteria; Actinomycetia; Corynebacteriales; Mycobacteriaceae; Mycobacterium; Mycobacterium tuberculosis complex; Mycobacterium tuberculosis;	NCBI: 1422008	4406239	99.94	0.00	Yes
NCBI Assembly	GCA_000678295.1	NCBI: cellular organisms; Bacteria; Terrabacteria group; Actinobacteria; Actinomycetia; Corynebacteriales; Mycobacteriaceae; Mycobacterium; Mycobacterium tuberculosis complex; Mycobacterium tuberculosis;	NCBI: 1423489	4404298	99.94	0.00	Yes

NCBI Assembly	GCA_000677055.1	NCBI: cellular organisms; Bacteria; Terrabacteria group; Actinobacteria; Actinomycetia; Corynebacteriales; Mycobacteriaceae; Mycobacterium; Mycobacterium tuberculosis complex; Mycobacterium tuberculosis;	NCBI: 1422109	4448714	99.78	0.00	Yes
NCBI Assembly	GCA_000680795.1	NCBI: cellular organisms; Bacteria; Terrabacteria group; Actinobacteria; Actinomycetia; Corynebacteriales; Mycobacteriaceae; Mycobacterium; Mycobacterium tuberculosis complex; Mycobacterium tuberculosis;	NCBI: 1427229	4430432	99.78	0.00	Yes
NCBI Assembly	GCA_000680495.1	NCBI: cellular organisms; Bacteria; Terrabacteria group; Actinobacteria; Actinomycetia; Corynebacteriales; Mycobacteriaceae; Mycobacterium; Mycobacterium tuberculosis complex; Mycobacterium tuberculosis;	NCBI: 1427212	4400062	99.94	0.00	Yes
NCBI Assembly	GCA_000679235.1	NCBI: cellular organisms; Bacteria; Terrabacteria group; Actinobacteria; Actinomycetia; Corynebacteriales; Mycobacteriaceae; Mycobacterium; Mycobacterium tuberculosis complex; Mycobacterium tuberculosis;	NCBI: 1423545	4411323	99.94	0.00	Yes
NCBI Assembly	GCA_000670335.1	NCBI: cellular organisms; Bacteria; Terrabacteria group; Actinobacteria; Actinomycetia; Corynebacteriales; Mycobacteriaceae; Mycobacterium; Mycobacterium tuberculosis complex; Mycobacterium tuberculosis;	NCBI: 1421941	4413446	99.94	0.00	Yes
NCBI Assembly	GCA_000675815.1	NCBI: cellular organisms; Bacteria; Terrabacteria group; Actinobacteria; Actinomycetia; Corynebacteriales; Mycobacteriaceae; Mycobacterium; Mycobacterium tuberculosis complex; Mycobacterium tuberculosis;	NCBI: 1422044	4405284	99.94	0.00	Yes
NCBI Assembly	GCA_000679555.1	NCBI: cellular organisms; Bacteria; Terrabacteria group; Actinobacteria; Actinomycetia; Corynebacteriales; Mycobacteriaceae; Mycobacterium; Mycobacterium tuberculosis complex; Mycobacterium tuberculosis;	NCBI: 1423561	4408650	99.94	0.00	Yes
NCBI Assembly	GCA_000678015.1	NCBI: cellular organisms; Bacteria; Terrabacteria group; Actinobacteria; Actinomycetia; Corynebacteriales; Mycobacteriaceae; Mycobacterium; Mycobacterium tuberculosis complex; Mycobacterium tuberculosis;	NCBI: 1423474	4404234	99.94	0.00	Yes
NCBI Assembly	GCA_000729765.1	NCBI: cellular organisms; Bacteria; Terrabacteria group; Actinobacteria; Actinomycetia; Corynebacteriales; Mycobacteriaceae; Mycobacterium; Mycobacterium tuberculosis complex; Mycobacterium tuberculosis; Mycobacterium tuberculosis variant bovis;	NCBI: 1765	4308143	99.78	0.00	Yes
NCBI Assembly	GCA_000678315.1	NCBI: cellular organisms; Bacteria; Terrabacteria group; Actinobacteria; Actinomycetia; Corynebacteriales; Mycobacteriaceae; Mycobacterium; Mycobacterium tuberculosis complex; Mycobacterium tuberculosis;	NCBI: 1423490	4371551	99.94	0.00	Yes
NCBI Assembly	GCA_000751475.1	NCBI: cellular organisms; Bacteria; Terrabacteria group; Actinobacteria; Actinomycetia; Corynebacteriales; Mycobacteriaceae; Mycobacterium; Mycobacterium tuberculosis complex; Mycobacterium tuberculosis;	NCBI: 757421	4410169	99.94	0.00	Yes
NCBI Assembly	GCA_000675175.1	NCBI: cellular organisms; Bacteria; Terrabacteria group; Actinobacteria; Actinomycetia; Corynebacteriales; Mycobacteriaceae; Mycobacterium; Mycobacterium tuberculosis complex; Mycobacterium tuberculosis;	NCBI: 1422009	4399808	99.94	0.00	Yes
NCBI Assembly	GCA_000680815.1	NCBI: cellular organisms; Bacteria; Terrabacteria group; Actinobacteria; Actinomycetia; Corynebacteriales; Mycobacteriaceae; Mycobacterium; Mycobacterium tuberculosis complex; Mycobacterium tuberculosis;	NCBI: 1427230	4413037	99.94	0.00	Yes
NCBI Assembly	GCA_000680515.1	NCBI: cellular organisms; Bacteria; Terrabacteria group; Actinobacteria; Actinomycetia; Corynebacteriales; Mycobacteriaceae; Mycobacterium; Mycobacterium tuberculosis complex; Mycobacterium tuberculosis;	NCBI: 1427213	4423980	99.94	0.00	Yes
NCBI Assembly	GCA_000677075.1	NCBI: cellular organisms; Bacteria; Terrabacteria group; Actinobacteria; Actinomycetia; Corynebacteriales; Mycobacteriaceae; Mycobacterium; Mycobacterium tuberculosis complex; Mycobacterium tuberculosis;	NCBI: 1422110	4388211	99.94	0.00	Yes
NCBI Assembly	GCA_000679255.1	NCBI: cellular organisms; Bacteria; Terrabacteria group; Actinobacteria; Actinomycetia; Corynebacteriales; Mycobacteriaceae; Mycobacterium; Mycobacterium tuberculosis complex; Mycobacterium tuberculosis;	NCBI: 1423546	4406335	99.94	0.00	Yes
NCBI Assembly	GCA_000670355.1	NCBI: cellular organisms; Bacteria; Terrabacteria group; Actinobacteria; Actinomycetia; Corynebacteriales; Mycobacteriaceae; Mycobacterium; Mycobacterium tuberculosis complex; Mycobacterium tuberculosis;	NCBI: 1421942	4409346	99.94	0.00	Yes
NCBI Assembly	GCA_000675835.1	NCBI: cellular organisms; Bacteria; Terrabacteria group; Actinobacteria; Actinomycetia; Corynebacteriales; Mycobacteriaceae; Mycobacterium; Mycobacterium tuberculosis complex; Mycobacterium tuberculosis;	NCBI: 1422045	4423119	99.94	0.00	Yes

NCBI Assembly	GCA_000679575.1	NCBI: cellular organisms; Bacteria; Terrabacteria group; Actinobacteria; Actinomycetia; Corynebacteriales; Mycobacteriaceae; Mycobacterium; Mycobacterium tuberculosis complex; Mycobacterium tuberculosis;	NCBI: 1423562	4411362	99.94	0.00	Yes
NCBI Assembly	GCA_000678035.1	NCBI: cellular organisms; Bacteria; Terrabacteria group; Actinobacteria; Actinomycetia; Corynebacteriales; Mycobacteriaceae; Mycobacterium; Mycobacterium tuberculosis complex; Mycobacterium tuberculosis;	NCBI: 1423475	4410927	99.94	0.00	Yes
NCBI Assembly	GCA_000675195.1	NCBI: cellular organisms; Bacteria; Terrabacteria group; Actinobacteria; Actinomycetia; Corynebacteriales; Mycobacteriaceae; Mycobacterium; Mycobacterium tuberculosis complex; Mycobacterium tuberculosis;	NCBI: 1422010	4412179	99.94	0.00	Yes
NCBI Assembly	GCA_000678335.1	NCBI: cellular organisms; Bacteria; Terrabacteria group; Actinobacteria; Actinomycetia; Corynebacteriales; Mycobacteriaceae; Mycobacterium; Mycobacterium tuberculosis complex; Mycobacterium tuberculosis;	NCBI: 1423492	4431459	99.94	0.00	Yes
NCBI Assembly	GCA_000751615.1	NCBI: cellular organisms; Bacteria; Terrabacteria group; Actinobacteria; Actinomycetia; Corynebacteriales; Mycobacteriaceae; Mycobacterium; Mycobacterium tuberculosis complex; Mycobacterium tuberculosis;	NCBI: 757418	4410911	99.94	0.00	Yes
NCBI Assembly	GCA_000677095.1	NCBI: cellular organisms; Bacteria; Terrabacteria group; Actinobacteria; Actinomycetia; Corynebacteriales; Mycobacteriaceae; Mycobacterium; Mycobacterium tuberculosis complex; Mycobacterium tuberculosis;	NCBI: 1422111	4415156	99.94	0.00	Yes
NCBI Assembly	GCA_000680535.1	NCBI: cellular organisms; Bacteria; Terrabacteria group; Actinobacteria; Actinomycetia; Corynebacteriales; Mycobacteriaceae; Mycobacterium; Mycobacterium tuberculosis complex; Mycobacterium tuberculosis;	NCBI: 1427215	4415899	99.94	0.00	Yes
NCBI Assembly	GCA_000680835.1	NCBI: cellular organisms; Bacteria; Terrabacteria group; Actinobacteria; Actinomycetia; Corynebacteriales; Mycobacteriaceae; Mycobacterium; Mycobacterium tuberculosis complex; Mycobacterium tuberculosis;	NCBI: 1427232	4422165	99.94	0.00	Yes
NCBI Assembly	GCA_000679275.1	NCBI: cellular organisms; Bacteria; Terrabacteria group; Actinobacteria; Actinomycetia; Corynebacteriales; Mycobacteriaceae; Mycobacterium; Mycobacterium tuberculosis complex; Mycobacterium tuberculosis;	NCBI: 1423547	4387014	99.94	0.00	Yes
NCBI Assembly	GCA_000670375.1	NCBI: cellular organisms; Bacteria; Terrabacteria group; Actinobacteria; Actinomycetia; Corynebacteriales; Mycobacteriaceae; Mycobacterium; Mycobacterium tuberculosis complex; Mycobacterium tuberculosis;	NCBI: 1421943	4409995	99.94	0.00	Yes
NCBI Assembly	GCA_000675855.1	NCBI: cellular organisms; Bacteria; Terrabacteria group; Actinobacteria; Actinomycetia; Corynebacteriales; Mycobacteriaceae; Mycobacterium; Mycobacterium tuberculosis complex; Mycobacterium tuberculosis;	NCBI: 1422046	4390308	99.94	0.00	Yes
NCBI Assembly	GCA_000679595.1	NCBI: cellular organisms; Bacteria; Terrabacteria group; Actinobacteria; Actinomycetia; Corynebacteriales; Mycobacteriaceae; Mycobacterium; Mycobacterium tuberculosis complex; Mycobacterium tuberculosis;	NCBI: 1423563	4439090	99.94	0.00	Yes
NCBI Assembly	GCA_000678355.1	NCBI: cellular organisms; Bacteria; Terrabacteria group; Actinobacteria; Actinomycetia; Corynebacteriales; Mycobacteriaceae; Mycobacterium; Mycobacterium tuberculosis complex; Mycobacterium tuberculosis;	NCBI: 1423493	4391784	99.94	0.00	Yes
NCBI Assembly	GCA_000678055.1	NCBI: cellular organisms; Bacteria; Terrabacteria group; Actinobacteria; Actinomycetia; Corynebacteriales; Mycobacteriaceae; Mycobacterium; Mycobacterium tuberculosis complex; Mycobacterium tuberculosis;	NCBI: 1423476	4372884	99.94	0.00	Yes
NCBI Assembly	GCA_000675215.1	NCBI: cellular organisms; Bacteria; Terrabacteria group; Actinobacteria; Actinomycetia; Corynebacteriales; Mycobacteriaceae; Mycobacterium; Mycobacterium tuberculosis complex; Mycobacterium tuberculosis;	NCBI: 1422011	4532485	98.74	0.33	Yes
NCBI Assembly	GCA_000735795.1	NCBI: cellular organisms; Bacteria; Terrabacteria group; Actinobacteria; Actinomycetia; Corynebacteriales; Mycobacteriaceae; Mycobacterium; Mycobacterium tuberculosis complex; Mycobacterium tuberculosis;	NCBI: 1773	4539435	98.08	0.57	Yes
NCBI Assembly	GCA_000756525.1	NCBI: cellular organisms; Bacteria; Terrabacteria group; Actinobacteria; Actinomycetia; Corynebacteriales; Mycobacteriaceae; Mycobacterium; Mycobacterium tuberculosis complex; Mycobacterium tuberculosis;	NCBI: 1773	4379376	99.61	0.00	Yes
NCBI Assembly	GCA_000677115.1	NCBI: cellular organisms; Bacteria; Terrabacteria group; Actinobacteria; Actinomycetia; Corynebacteriales; Mycobacteriaceae; Mycobacterium; Mycobacterium tuberculosis complex; Mycobacterium tuberculosis;	NCBI: 1422112	4420115	99.94	0.00	Yes

NCBI Assembly	GCA_000680855.1	NCBI: cellular organisms; Bacteria; Terrabacteria group; Actinobacteria; Actinomycetia; Corynebacteriales; Mycobacteriaceae; Mycobacterium; Mycobacterium tuberculosis complex; Mycobacterium tuberculosis;	NCBI: 1427233	4396351	99.94	0.00	Yes
NCBI Assembly	GCA_000680555.1	NCBI: cellular organisms; Bacteria; Terrabacteria group; Actinobacteria; Actinomycetia; Corynebacteriales; Mycobacteriaceae; Mycobacterium; Mycobacterium tuberculosis complex; Mycobacterium tuberculosis;	NCBI: 1427216	4393259	99.94	0.00	Yes
NCBI Assembly	GCA_000679295.1	NCBI: cellular organisms; Bacteria; Terrabacteria group; Actinobacteria; Actinomycetia; Corynebacteriales; Mycobacteriaceae; Mycobacterium; Mycobacterium tuberculosis complex; Mycobacterium tuberculosis;	NCBI: 1423548	4407242	99.94	0.00	Yes
NCBI Assembly	GCA_000670395.1	NCBI: cellular organisms; Bacteria; Terrabacteria group; Actinobacteria; Actinomycetia; Corynebacteriales; Mycobacteriaceae; Mycobacterium; Mycobacterium tuberculosis complex; Mycobacterium tuberculosis;	NCBI: 1421944	4370696	99.94	0.07	Yes
NCBI Assembly	GCA_000675875.1	NCBI: cellular organisms; Bacteria; Terrabacteria group; Actinobacteria; Actinomycetia; Corynebacteriales; Mycobacteriaceae; Mycobacterium; Mycobacterium tuberculosis complex; Mycobacterium tuberculosis;	NCBI: 1422047	4442148	99.61	0.07	Yes
NCBI Assembly	GCA_000679615.1	NCBI: cellular organisms; Bacteria; Terrabacteria group; Actinobacteria; Actinomycetia; Corynebacteriales; Mycobacteriaceae; Mycobacterium; Mycobacterium tuberculosis complex; Mycobacterium tuberculosis;	NCBI: 1423564	4427956	99.94	0.00	Yes
NCBI Assembly	GCA_000678075.1	NCBI: cellular organisms; Bacteria; Terrabacteria group; Actinobacteria; Actinomycetia; Corynebacteriales; Mycobacteriaceae; Mycobacterium; Mycobacterium tuberculosis complex; Mycobacterium tuberculosis;	NCBI: 1423478	4441965	99.94	0.33	Yes
NCBI Assembly	GCA_000678375.1	NCBI: cellular organisms; Bacteria; Terrabacteria group; Actinobacteria; Actinomycetia; Corynebacteriales; Mycobacteriaceae; Mycobacterium; Mycobacterium tuberculosis complex; Mycobacterium tuberculosis;	NCBI: 1423494	4397706	99.94	0.00	Yes
NCBI Assembly	GCA_000675235.1	NCBI: cellular organisms; Bacteria; Terrabacteria group; Actinobacteria; Actinomycetia; Corynebacteriales; Mycobacteriaceae; Mycobacterium; Mycobacterium tuberculosis complex; Mycobacterium tuberculosis;	NCBI: 1422012	4368150	99.94	0.00	Yes
NCBI Assembly	GCA_000735815.1	NCBI: cellular organisms; Bacteria; Terrabacteria group; Actinobacteria; Actinomycetia; Corynebacteriales; Mycobacteriaceae; Mycobacterium; Mycobacterium tuberculosis complex; Mycobacterium tuberculosis;	NCBI: 1773	4539435	98.19	0.50	Yes
NCBI Assembly	GCA_000680575.1	NCBI: cellular organisms; Bacteria; Terrabacteria group; Actinobacteria; Actinomycetia; Corynebacteriales; Mycobacteriaceae; Mycobacterium; Mycobacterium tuberculosis complex; Mycobacterium tuberculosis;	NCBI: 1427217	4406314	99.94	0.00	Yes
NCBI Assembly	GCA_000680875.1	NCBI: cellular organisms; Bacteria; Terrabacteria group; Actinobacteria; Actinomycetia; Corynebacteriales; Mycobacteriaceae; Mycobacterium; Mycobacterium tuberculosis complex; Mycobacterium tuberculosis;	NCBI: 1427234	4421176	99.94	0.00	Yes
NCBI Assembly	GCA_000756545.1	NCBI: cellular organisms; Bacteria; Terrabacteria group; Actinobacteria; Actinomycetia; Corynebacteriales; Mycobacteriaceae; Mycobacterium; Mycobacterium tuberculosis complex; Mycobacterium tuberculosis;	NCBI: 1773	4410945	99.94	0.00	Yes
NCBI Assembly	GCA_000677135.1	NCBI: cellular organisms; Bacteria; Terrabacteria group; Actinobacteria; Actinomycetia; Corynebacteriales; Mycobacteriaceae; Mycobacterium; Mycobacterium tuberculosis complex; Mycobacterium tuberculosis;	NCBI: 1422113	4407935	99.94	0.22	Yes
NCBI Assembly	GCA_000679315.1	NCBI: cellular organisms; Bacteria; Terrabacteria group; Actinobacteria; Actinomycetia; Corynebacteriales; Mycobacteriaceae; Mycobacterium; Mycobacterium tuberculosis complex; Mycobacterium tuberculosis;	NCBI: 1423549	4419441	99.94	0.00	Yes
NCBI Assembly	GCA_000676535.1	NCBI: cellular organisms; Bacteria; Terrabacteria group; Actinobacteria; Actinomycetia; Corynebacteriales; Mycobacteriaceae; Mycobacterium; Mycobacterium tuberculosis complex; Mycobacterium tuberculosis;	NCBI: 1422080	4417823	99.94	0.00	Yes
NCBI Assembly	GCA_000671055.1	NCBI: cellular organisms; Bacteria; Terrabacteria group; Actinobacteria; Actinomycetia; Corynebacteriales; Mycobacteriaceae; Mycobacterium; Mycobacterium tuberculosis complex; Mycobacterium tuberculosis;	NCBI: 1421980	4398560	99.94	0.00	Yes
NCBI Assembly	GCA_000674655.1	NCBI: cellular organisms; Bacteria; Terrabacteria group; Actinobacteria; Actinomycetia; Corynebacteriales; Mycobacteriaceae; Mycobacterium; Mycobacterium tuberculosis complex; Mycobacterium tuberculosis;	NCBI: 1402483	4436225	99.94	0.00	Yes

NCBI Assembly	GCA_000679635.1	NCBI: cellular organisms; Bacteria; Terrabacteria group; Actinobacteria; Actinomycetia; Corynebacteriales; Mycobacteriaceae; Mycobacterium; Mycobacterium tuberculosis complex; Mycobacterium tuberculosis;	NCBI: 1423565	4406004	99.94	0.00	Yes
NCBI Assembly	GCA_000675255.1	NCBI: cellular organisms; Bacteria; Terrabacteria group; Actinobacteria; Actinomycetia; Corynebacteriales; Mycobacteriaceae; Mycobacterium; Mycobacterium tuberculosis complex; Mycobacterium tuberculosis;	NCBI: 1422013	4411074	99.94	0.00	Yes
NCBI Assembly	GCA_000675275.1	NCBI: cellular organisms; Bacteria; Terrabacteria group; Actinobacteria; Actinomycetia; Corynebacteriales; Mycobacteriaceae; Mycobacterium; Mycobacterium tuberculosis complex; Mycobacterium tuberculosis;	NCBI: 1422014	4456267	99.94	0.00	Yes
NCBI Assembly	GCA_000676215.1	NCBI: cellular organisms; Bacteria; Terrabacteria group; Actinobacteria; Actinomycetia; Corynebacteriales; Mycobacteriaceae; Mycobacterium; Mycobacterium tuberculosis complex; Mycobacterium tuberculosis;	NCBI: 1422064	4349998	99.94	0.00	Yes
NCBI Assembly	GCA_000674355.1	NCBI: cellular organisms; Bacteria; Terrabacteria group; Actinobacteria; Actinomycetia; Corynebacteriales; Mycobacteriaceae; Mycobacterium; Mycobacterium tuberculosis complex; Mycobacterium tuberculosis;	NCBI: 1400929	4388101	99.94	0.00	Yes
NCBI Assembly	GCA_000735835.1	NCBI: cellular organisms; Bacteria; Terrabacteria group; Actinobacteria; Actinomycetia; Corynebacteriales; Mycobacteriaceae; Mycobacterium; Mycobacterium tuberculosis complex; Mycobacterium tuberculosis;	NCBI: 1773	4539435	97.89	0.57	Yes
NCBI Assembly	GCA_000706285.1	NCBI: cellular organisms; Bacteria; Terrabacteria group; Actinobacteria; Actinomycetia; Corynebacteriales; Mycobacteriaceae; Mycobacterium; Mycobacterium tuberculosis complex; Mycobacterium tuberculosis;	NCBI: 1447506	4424169	99.94	0.00	Yes
NCBI Assembly	GCA_000679335.1	NCBI: cellular organisms; Bacteria; Terrabacteria group; Actinobacteria; Actinomycetia; Corynebacteriales; Mycobacteriaceae; Mycobacterium; Mycobacterium tuberculosis complex; Mycobacterium tuberculosis;	NCBI: 1423550	4379143	99.83	0.00	Yes
NCBI Assembly	GCA_000759695.1	NCBI: cellular organisms; Bacteria; Terrabacteria group; Actinobacteria; Actinomycetia; Corynebacteriales; Mycobacteriaceae; Mycobacterium; Mycobacterium kyorinense;	NCBI: 487514	5302980	98.18	0.74	Yes
NCBI Assembly	GCA_000676555.1	NCBI: cellular organisms; Bacteria; Terrabacteria group; Actinobacteria; Actinomycetia; Corynebacteriales; Mycobacteriaceae; Mycobacterium; Mycobacterium tuberculosis complex; Mycobacterium tuberculosis;	NCBI: 1422081	4421248	99.83	0.00	Yes
NCBI Assembly	GCA_000671075.1	NCBI: cellular organisms; Bacteria; Terrabacteria group; Actinobacteria; Actinomycetia; Corynebacteriales; Mycobacteriaceae; Mycobacterium; Mycobacterium tuberculosis complex; Mycobacterium tuberculosis;	NCBI: 1421982	4424181	99.94	0.00	Yes
NCBI Assembly	GCA_000673395.1	NCBI: cellular organisms; Bacteria; Terrabacteria group; Actinobacteria; Actinomycetia; Corynebacteriales; Mycobacteriaceae; Mycobacterium; Mycobacterium tuberculosis complex; Mycobacterium tuberculosis;	NCBI: 1397936	4410913	99.94	0.00	Yes
NCBI Assembly	GCA_000674675.1	NCBI: cellular organisms; Bacteria; Terrabacteria group; Actinobacteria; Actinomycetia; Corynebacteriales; Mycobacteriaceae; Mycobacterium; Mycobacterium tuberculosis complex; Mycobacterium tuberculosis;	NCBI: 1408904	4404207	99.94	0.00	Yes
NCBI Assembly	GCA_000675295.1	NCBI: cellular organisms; Bacteria; Terrabacteria group; Actinobacteria; Actinomycetia; Corynebacteriales; Mycobacteriaceae; Mycobacterium; Mycobacterium tuberculosis complex; Mycobacterium tuberculosis;	NCBI: 1422015	4472160	99.94	0.00	Yes
NCBI Assembly	GCA_000666845.1	NCBI: cellular organisms; Bacteria; Terrabacteria group; Actinobacteria; Actinomycetia; Corynebacteriales; Mycobacteriaceae; Mycobacterium; Mycobacterium tuberculosis complex; Mycobacterium tuberculosis; Mycobacterium tuberculosis variant africanum;	NCBI: 1354183	4390721	99.94	0.00	Yes
NCBI Assembly	GCA_000676235.1	NCBI: cellular organisms; Bacteria; Terrabacteria group; Actinobacteria; Actinomycetia; Corynebacteriales; Mycobacteriaceae; Mycobacterium; Mycobacterium tuberculosis complex; Mycobacterium tuberculosis;	NCBI: 1422065	4455712	99.94	0.00	Yes
NCBI Assembly	GCA_000674375.1	NCBI: cellular organisms; Bacteria; Terrabacteria group; Actinobacteria; Actinomycetia; Corynebacteriales; Mycobacteriaceae; Mycobacterium; Mycobacterium tuberculosis complex; Mycobacterium tuberculosis;	NCBI: 1400930	4470110	99.94	0.17	Yes
NCBI Assembly	GCA_000706305.1	NCBI: cellular organisms; Bacteria; Terrabacteria group; Actinobacteria; Actinomycetia; Corynebacteriales; Mycobacteriaceae; Mycobacterium; Mycobacterium tuberculosis complex; Mycobacterium tuberculosis;	NCBI: 1447505	4427313	99.94	0.44	Yes

NCBI Assembly	GCA_000735855.1	NCBI: cellular organisms; Bacteria; Terrabacteria group; Actinobacteria; Actinomycetia; Corynebacteriales; Mycobacteriaceae; Mycobacterium; Mycobacterium tuberculosis complex; Mycobacterium tuberculosis;	NCBI: 1773	4539435	95.77	0.50	Yes
NCBI Assembly	GCA_000705985.1	NCBI: cellular organisms; Bacteria; Terrabacteria group; Actinobacteria; Actinomycetia; Corynebacteriales; Mycobacteriaceae; Mycobacterium; Mycobacterium tuberculosis complex; Mycobacterium tuberculosis;	NCBI: 1423464	4401073	99.94	0.00	Yes
NCBI Assembly	GCA_000671095.1	NCBI: cellular organisms; Bacteria; Terrabacteria group; Actinobacteria; Actinomycetia; Corynebacteriales; Mycobacteriaceae; Mycobacterium; Mycobacterium tuberculosis complex; Mycobacterium tuberculosis;	NCBI: 1421983	4399205	99.94	0.00	Yes
NCBI Assembly	GCA_000676575.1	NCBI: cellular organisms; Bacteria; Terrabacteria group; Actinobacteria; Actinomycetia; Corynebacteriales; Mycobacteriaceae; Mycobacterium; Mycobacterium tuberculosis complex; Mycobacterium tuberculosis;	NCBI: 1422082	4413699	99.94	0.00	Yes
NCBI Assembly	GCA_000762985.1	NCBI: cellular organisms; Bacteria; Terrabacteria group; Actinobacteria; Actinomycetia; Corynebacteriales; Mycobacteriaceae; Mycolicibacterium; Mycolicibacterium rufum;	NCBI: 318424	6176413	100.00	0.60	Yes
NCBI Assembly	GCA_000673415.1	NCBI: cellular organisms; Bacteria; Terrabacteria group; Actinobacteria; Actinomycetia; Corynebacteriales; Mycobacteriaceae; Mycobacterium; Mycobacterium tuberculosis complex; Mycobacterium tuberculosis;	NCBI: 1397937	4400397	99.94	0.00	Yes
NCBI Assembly	GCA_000674695.1	NCBI: cellular organisms; Bacteria; Terrabacteria group; Actinobacteria; Actinomycetia; Corynebacteriales; Mycobacteriaceae; Mycobacterium; Mycobacterium tuberculosis complex; Mycobacterium tuberculosis;	NCBI: 1408905	4510026	99.94	0.00	Yes
NCBI Assembly	GCA_000675315.1	NCBI: cellular organisms; Bacteria; Terrabacteria group; Actinobacteria; Actinomycetia; Corynebacteriales; Mycobacteriaceae; Mycobacterium; Mycobacterium tuberculosis complex; Mycobacterium tuberculosis;	NCBI: 1422016	4401746	99.94	0.00	Yes
NCBI Assembly	GCA_000666865.1	NCBI: cellular organisms; Bacteria; Terrabacteria group; Actinobacteria; Actinomycetia; Corynebacteriales; Mycobacteriaceae; Mycobacterium; Mycobacterium tuberculosis complex; Mycobacterium tuberculosis; Mycobacterium tuberculosis variant africanum;	NCBI: 1354186	4381902	99.94	0.00	Yes
NCBI Assembly	GCA_000676255.1	NCBI: cellular organisms; Bacteria; Terrabacteria group; Actinobacteria; Actinomycetia; Corynebacteriales; Mycobacteriaceae; Mycobacterium; Mycobacterium tuberculosis complex; Mycobacterium tuberculosis;	NCBI: 1422066	4416654	99.94	0.00	Yes
NCBI Assembly	GCA_000674395.1	NCBI: cellular organisms; Bacteria; Terrabacteria group; Actinobacteria; Actinomycetia; Corynebacteriales; Mycobacteriaceae; Mycobacterium; Mycobacterium tuberculosis complex; Mycobacterium tuberculosis;	NCBI: 1400931	4396968	99.94	0.00	Yes
NCBI Assembly	GCA_000706325.1	NCBI: cellular organisms; Bacteria; Terrabacteria group; Actinobacteria; Actinomycetia; Corynebacteriales; Mycobacteriaceae; Mycobacterium; Mycobacterium tuberculosis complex; Mycobacterium tuberculosis;	NCBI: 1447503	4424810	99.94	0.00	Yes
NCBI Assembly	GCA_000672475.1	NCBI: cellular organisms; Bacteria; Terrabacteria group; Actinobacteria; Actinomycetia; Corynebacteriales; Mycobacteriaceae; Mycobacterium; Mycobacterium tuberculosis complex; Mycobacterium tuberculosis;	NCBI: 1397889	4450112	99.86	0.00	Yes
NCBI Assembly	GCA_000706005.1	NCBI: cellular organisms; Bacteria; Terrabacteria group; Actinobacteria; Actinomycetia; Corynebacteriales; Mycobacteriaceae; Mycobacterium; Mycobacterium tuberculosis complex; Mycobacterium tuberculosis;	NCBI: 1423427	4474038	99.88	0.36	Yes
NCBI Assembly	GCA_000671115.1	NCBI: cellular organisms; Bacteria; Terrabacteria group; Actinobacteria; Actinomycetia; Corynebacteriales; Mycobacteriaceae; Mycobacterium; Mycobacterium tuberculosis complex; Mycobacterium tuberculosis;	NCBI: 1421984	4427463	99.94	0.59	Yes
NCBI Assembly	GCA_000676595.1	NCBI: cellular organisms; Bacteria; Terrabacteria group; Actinobacteria; Actinomycetia; Corynebacteriales; Mycobacteriaceae; Mycobacterium; Mycobacterium tuberculosis complex; Mycobacterium tuberculosis;	NCBI: 1422083	4425048	99.94	0.00	Yes
NCBI Assembly	GCA_000673435.1	NCBI: cellular organisms; Bacteria; Terrabacteria group; Actinobacteria; Actinomycetia; Corynebacteriales; Mycobacteriaceae; Mycobacterium; Mycobacterium tuberculosis complex; Mycobacterium tuberculosis;	NCBI: 1397938	4445450	99.94	0.00	Yes
NCBI Assembly	GCA_000767665.1	NCBI: cellular organisms; Bacteria; Terrabacteria group; Actinobacteria; Actinomycetia; Corynebacteriales; Mycobacteriaceae; Mycolicibacterium; Mycolicibacterium smegmatis;	NCBI: 1772	6988337	99.94	1.13	Yes

NCBI Assembly	GCA_000674715.1	NCBI: cellular organisms; Bacteria; Terrabacteria group; Actinobacteria; Actinomycetia; Corynebacteriales; Mycobacteriaceae; Mycobacterium; Mycobacterium tuberculosis complex; Mycobacterium tuberculosis;	NCBI: 1408906	4403244	99.94	0.00	Yes
NCBI Assembly	GCA_000666885.1	NCBI: cellular organisms; Bacteria; Terrabacteria group; Actinobacteria; Actinomycetia; Corynebacteriales; Mycobacteriaceae; Mycobacterium; Mycobacterium tuberculosis complex; Mycobacterium tuberculosis;	NCBI: 1354137	4407681	99.94	0.00	Yes
NCBI Assembly	GCA_000676275.1	NCBI: cellular organisms; Bacteria; Terrabacteria group; Actinobacteria; Actinomycetia; Corynebacteriales; Mycobacteriaceae; Mycobacterium; Mycobacterium tuberculosis complex; Mycobacterium tuberculosis;	NCBI: 1422067	4393390	99.94	0.00	Yes
NCBI Assembly	GCA_000675335.1	NCBI: cellular organisms; Bacteria; Terrabacteria group; Actinobacteria; Actinomycetia; Corynebacteriales; Mycobacteriaceae; Mycobacterium; Mycobacterium tuberculosis complex; Mycobacterium tuberculosis;	NCBI: 1422017	4433821	99.94	0.00	Yes
NCBI Assembly	GCA_000706345.1	NCBI: cellular organisms; Bacteria; Terrabacteria group; Actinobacteria; Actinomycetia; Corynebacteriales; Mycobacteriaceae; Mycobacterium; Mycobacterium tuberculosis complex; Mycobacterium tuberculosis;	NCBI: 1447499	4427147	99.61	0.00	Yes
NCBI Assembly	GCA_000674415.1	NCBI: cellular organisms; Bacteria; Terrabacteria group; Actinobacteria; Actinomycetia; Corynebacteriales; Mycobacteriaceae; Mycobacterium; Mycobacterium tuberculosis complex; Mycobacterium tuberculosis;	NCBI: 1400932	4399561	99.94	0.00	Yes
NCBI Assembly	GCA_000672495.1	NCBI: cellular organisms; Bacteria; Terrabacteria group; Actinobacteria; Actinomycetia; Corynebacteriales; Mycobacteriaceae; Mycobacterium; Mycobacterium tuberculosis complex; Mycobacterium tuberculosis;	NCBI: 1397890	4417206	99.94	0.00	Yes
NCBI Assembly	GCA_000706025.1	NCBI: cellular organisms; Bacteria; Terrabacteria group; Actinobacteria; Actinomycetia; Corynebacteriales; Mycobacteriaceae; Mycobacterium; Mycobacterium tuberculosis complex; Mycobacterium tuberculosis;	NCBI: 1448835	4406462	99.94	0.00	Yes
NCBI Assembly	GCA_000671135.1	NCBI: cellular organisms; Bacteria; Terrabacteria group; Actinobacteria; Actinomycetia; Corynebacteriales; Mycobacteriaceae; Mycobacterium; Mycobacterium tuberculosis complex; Mycobacterium tuberculosis;	NCBI: 1421985	4392784	99.94	0.00	Yes
NCBI Assembly	GCA_000676615.1	NCBI: cellular organisms; Bacteria; Terrabacteria group; Actinobacteria; Actinomycetia; Corynebacteriales; Mycobacteriaceae; Mycobacterium; Mycobacterium tuberculosis complex; Mycobacterium tuberculosis;	NCBI: 1422084	4438563	99.94	0.00	Yes
NCBI Assembly	GCA_000673455.1	NCBI: cellular organisms; Bacteria; Terrabacteria group; Actinobacteria; Actinomycetia; Corynebacteriales; Mycobacteriaceae; Mycobacterium; Mycobacterium tuberculosis complex; Mycobacterium tuberculosis;	NCBI: 1397939	4407290	99.94	0.00	Yes
NCBI Assembly	GCA_000674735.1	NCBI: cellular organisms; Bacteria; Terrabacteria group; Actinobacteria; Actinomycetia; Corynebacteriales; Mycobacteriaceae; Mycobacterium; Mycobacterium tuberculosis complex; Mycobacterium tuberculosis;	NCBI: 1408907	4415785	99.94	0.00	Yes
NCBI Assembly	GCA_000767705.1	NCBI: cellular organisms; Bacteria; Terrabacteria group; Actinobacteria; Actinomycetia; Corynebacteriales; Mycobacteriaceae; Mycolicibacterium; Mycolicibacterium smegmatis;	NCBI: 1772	6988302	99.94	1.13	Yes
NCBI Assembly	GCA_000666905.1	NCBI: cellular organisms; Bacteria; Terrabacteria group; Actinobacteria; Actinomycetia; Corynebacteriales; Mycobacteriaceae; Mycobacterium; Mycobacterium tuberculosis complex; Mycobacterium tuberculosis;	NCBI: 1354138	4433072	99.94	0.00	Yes
NCBI Assembly	GCA_000676295.1	NCBI: cellular organisms; Bacteria; Terrabacteria group; Actinobacteria; Actinomycetia; Corynebacteriales; Mycobacteriaceae; Mycobacterium; Mycobacterium tuberculosis complex; Mycobacterium tuberculosis;	NCBI: 1422068	4408773	99.94	0.33	Yes
NCBI Assembly	GCA_000675355.1	NCBI: cellular organisms; Bacteria; Terrabacteria group; Actinobacteria; Actinomycetia; Corynebacteriales; Mycobacteriaceae; Mycobacterium; Mycobacterium tuberculosis complex; Mycobacterium tuberculosis;	NCBI: 1422018	4419758	99.94	0.00	Yes
NCBI Assembly	GCA_000706385.1	NCBI: cellular organisms; Bacteria; Terrabacteria group; Actinobacteria; Actinomycetia; Corynebacteriales; Mycobacteriaceae; Mycobacterium; Mycobacterium tuberculosis complex; Mycobacterium tuberculosis;	NCBI: 1447482	4383689	99.94	0.00	Yes
NCBI Assembly	GCA_000674435.1	NCBI: cellular organisms; Bacteria; Terrabacteria group; Actinobacteria; Actinomycetia; Corynebacteriales; Mycobacteriaceae; Mycobacterium; Mycobacterium tuberculosis complex; Mycobacterium tuberculosis;	NCBI: 1400933	4407949	99.94	0.00	Yes

NCBI Assembly	GCA_000672515.1	NCBI: cellular organisms; Bacteria; Terrabacteria group; Actinobacteria; Actinomycetia; Corynebacteriales; Mycobacteriaceae; Mycobacterium; Mycobacterium tuberculosis complex; Mycobacterium tuberculosis;	NCBI: 1397891	4416584	98.92	0.00	Yes
NCBI Assembly	GCA_000706045.1	NCBI: cellular organisms; Bacteria; Terrabacteria group; Actinobacteria; Actinomycetia; Corynebacteriales; Mycobacteriaceae; Mycobacterium; Mycobacterium tuberculosis complex; Mycobacterium tuberculosis;	NCBI: 1448831	4421159	99.94	0.00	Yes
NCBI Assembly	GCA_000671155.1	NCBI: cellular organisms; Bacteria; Terrabacteria group; Actinobacteria; Actinomycetia; Corynebacteriales; Mycobacteriaceae; Mycobacterium; Mycobacterium tuberculosis complex; Mycobacterium tuberculosis;	NCBI: 1421986	4413890	99.94	0.00	Yes
NCBI Assembly	GCA_000676635.1	NCBI: cellular organisms; Bacteria; Terrabacteria group; Actinobacteria; Actinomycetia; Corynebacteriales; Mycobacteriaceae; Mycobacterium; Mycobacterium tuberculosis complex; Mycobacterium tuberculosis;	NCBI: 1422085	4490445	96.12	0.56	Yes
NCBI Assembly	GCA_000673475.1	NCBI: cellular organisms; Bacteria; Terrabacteria group; Actinobacteria; Actinomycetia; Corynebacteriales; Mycobacteriaceae; Mycobacterium; Mycobacterium tuberculosis complex; Mycobacterium tuberculosis;	NCBI: 1397940	4437328	99.94	0.00	Yes
NCBI Assembly	GCA_000674755.1	NCBI: cellular organisms; Bacteria; Terrabacteria group; Actinobacteria; Actinomycetia; Corynebacteriales; Mycobacteriaceae; Mycobacterium; Mycobacterium tuberculosis complex; Mycobacterium tuberculosis;	NCBI: 1408909	4431444	99.94	0.33	Yes
NCBI Assembly	GCA_000786505.1	NCBI: cellular organisms; Bacteria; Terrabacteria group; Actinobacteria; Actinomycetia; Corynebacteriales; Mycobacteriaceae; Mycobacterium; Mycobacterium tuberculosis complex; Mycobacterium tuberculosis;	NCBI: 1427516	4412379	99.94	0.00	Yes
NCBI Assembly	GCA_000674455.1	NCBI: cellular organisms; Bacteria; Terrabacteria group; Actinobacteria; Actinomycetia; Corynebacteriales; Mycobacteriaceae; Mycobacterium; Mycobacterium tuberculosis complex; Mycobacterium tuberculosis;	NCBI: 1400934	4434949	99.94	0.33	Yes
NCBI Assembly	GCA_000676315.1	NCBI: cellular organisms; Bacteria; Terrabacteria group; Actinobacteria; Actinomycetia; Corynebacteriales; Mycobacteriaceae; Mycobacterium; Mycobacterium tuberculosis complex; Mycobacterium tuberculosis;	NCBI: 1422069	4392524	99.94	0.00	Yes
NCBI Assembly	GCA_000675375.1	NCBI: cellular organisms; Bacteria; Terrabacteria group; Actinobacteria; Actinomycetia; Corynebacteriales; Mycobacteriaceae; Mycobacterium; Mycobacterium tuberculosis complex; Mycobacterium tuberculosis;	NCBI: 1422019	4439267	99.94	0.00	Yes
NCBI Assembly	GCA_000666925.1	NCBI: cellular organisms; Bacteria; Terrabacteria group; Actinobacteria; Actinomycetia; Corynebacteriales; Mycobacteriaceae; Mycobacterium; Mycobacterium tuberculosis complex; Mycobacterium tuberculosis;	NCBI: 1354192	4420842	99.94	0.00	Yes
NCBI Assembly	GCA_000706365.1	NCBI: cellular organisms; Bacteria; Terrabacteria group; Actinobacteria; Actinomycetia; Corynebacteriales; Mycobacteriaceae; Mycobacterium; Mycobacterium tuberculosis complex; Mycobacterium tuberculosis;	NCBI: 1447488	4417181	99.94	0.00	Yes
NCBI Assembly	GCA_000672535.1	NCBI: cellular organisms; Bacteria; Terrabacteria group; Actinobacteria; Actinomycetia; Corynebacteriales; Mycobacteriaceae; Mycobacterium; Mycobacterium tuberculosis complex; Mycobacterium tuberculosis;	NCBI: 1397892	4453656	99.94	0.00	Yes
NCBI Assembly	GCA_000706065.1	NCBI: cellular organisms; Bacteria; Terrabacteria group; Actinobacteria; Actinomycetia; Corynebacteriales; Mycobacteriaceae; Mycobacterium; Mycobacterium tuberculosis complex; Mycobacterium tuberculosis;	NCBI: 1448827	4423530	99.94	0.11	Yes
NCBI Assembly	GCA_000671175.1	NCBI: cellular organisms; Bacteria; Terrabacteria group; Actinobacteria; Actinomycetia; Corynebacteriales; Mycobacteriaceae; Mycobacterium; Mycobacterium tuberculosis complex; Mycobacterium tuberculosis;	NCBI: 1421987	4399949	99.94	0.00	Yes
NCBI Assembly	GCA_000673495.1	NCBI: cellular organisms; Bacteria; Terrabacteria group; Actinobacteria; Actinomycetia; Corynebacteriales; Mycobacteriaceae; Mycobacterium; Mycobacterium tuberculosis complex; Mycobacterium tuberculosis;	NCBI: 1397941	4406354	99.94	0.00	Yes
NCBI Assembly	GCA_000676655.1	NCBI: cellular organisms; Bacteria; Terrabacteria group; Actinobacteria; Actinomycetia; Corynebacteriales; Mycobacteriaceae; Mycobacterium; Mycobacterium tuberculosis complex; Mycobacterium tuberculosis;	NCBI: 1422086	4407677	99.94	0.00	Yes
NCBI Assembly	GCA_000674775.1	NCBI: cellular organisms; Bacteria; Terrabacteria group; Actinobacteria; Actinomycetia; Corynebacteriales; Mycobacteriaceae; Mycobacterium; Mycobacterium tuberculosis complex; Mycobacterium tuberculosis;	NCBI: 1408911	4392377	99.94	0.00	Yes

NCBI Assembly	GCA_000674475.1	NCBI: cellular organisms; Bacteria; Terrabacteria group; Actinobacteria; Actinomycetia; Corynebacteriales; Mycobacteriaceae; Mycobacterium; Mycobacterium tuberculosis complex; Mycobacterium tuberculosis;	NCBI: 1400935	4394252	99.94	0.00	Yes
NCBI Assembly	GCA_000666945.1	NCBI: cellular organisms; Bacteria; Terrabacteria group; Actinobacteria; Actinomycetia; Corynebacteriales; Mycobacteriaceae; Mycobacterium; Mycobacterium tuberculosis complex; Mycobacterium tuberculosis;	NCBI: 1354139	4416047	99.94	0.00	Yes
NCBI Assembly	GCA_000676335.1	NCBI: cellular organisms; Bacteria; Terrabacteria group; Actinobacteria; Actinomycetia; Corynebacteriales; Mycobacteriaceae; Mycobacterium; Mycobacterium tuberculosis complex; Mycobacterium tuberculosis;	NCBI: 1422070	4393136	99.94	0.00	Yes
NCBI Assembly	GCA_000675395.1	NCBI: cellular organisms; Bacteria; Terrabacteria group; Actinobacteria; Actinomycetia; Corynebacteriales; Mycobacteriaceae; Mycobacterium; Mycobacterium tuberculosis complex; Mycobacterium tuberculosis;	NCBI: 1422020	4401627	99.94	0.00	Yes
NCBI Assembly	GCA_000706405.1	NCBI: cellular organisms; Bacteria; Terrabacteria group; Actinobacteria; Actinomycetia; Corynebacteriales; Mycobacteriaceae; Mycobacterium; Mycobacterium tuberculosis complex; Mycobacterium tuberculosis;	NCBI: 1447473	4423621	99.94	0.00	Yes
NCBI Assembly	GCA_000672555.1	NCBI: cellular organisms; Bacteria; Terrabacteria group; Actinobacteria; Actinomycetia; Corynebacteriales; Mycobacteriaceae; Mycobacterium; Mycobacterium tuberculosis complex; Mycobacterium tuberculosis;	NCBI: 1397893	4421984	99.94	0.00	Yes
NCBI Assembly	GCA_000797435.1	NCBI: cellular organisms; Bacteria; Terrabacteria group; Actinobacteria; Actinomycetia; Corynebacteriales; Mycobacteriaceae; Mycobacterium; Mycobacterium tuberculosis complex; Mycobacterium tuberculosis;	NCBI: 1773	4313329	96.06	0.83	Yes
NCBI Assembly	GCA_000671195.1	NCBI: cellular organisms; Bacteria; Terrabacteria group; Actinobacteria; Actinomycetia; Corynebacteriales; Mycobacteriaceae; Mycobacterium; Mycobacterium tuberculosis complex; Mycobacterium tuberculosis;	NCBI: 1421988	4411220	99.94	0.00	Yes
NCBI Assembly	GCA_000706085.1	NCBI: cellular organisms; Bacteria; Terrabacteria group; Actinobacteria; Actinomycetia; Corynebacteriales; Mycobacteriaceae; Mycobacterium; Mycobacterium tuberculosis complex; Mycobacterium tuberculosis;	NCBI: 1448773	4419021	99.94	0.00	Yes
NCBI Assembly	GCA_000673515.1	NCBI: cellular organisms; Bacteria; Terrabacteria group; Actinobacteria; Actinomycetia; Corynebacteriales; Mycobacteriaceae; Mycobacterium; Mycobacterium tuberculosis complex; Mycobacterium tuberculosis;	NCBI: 1400870	4401693	99.94	0.00	Yes
NCBI Assembly	GCA_000676675.1	NCBI: cellular organisms; Bacteria; Terrabacteria group; Actinobacteria; Actinomycetia; Corynebacteriales; Mycobacteriaceae; Mycobacterium; Mycobacterium tuberculosis complex; Mycobacterium tuberculosis;	NCBI: 1422087	4412266	99.94	0.00	Yes
NCBI Assembly	GCA_000674795.1	NCBI: cellular organisms; Bacteria; Terrabacteria group; Actinobacteria; Actinomycetia; Corynebacteriales; Mycobacteriaceae; Mycobacterium; Mycobacterium tuberculosis complex; Mycobacterium tuberculosis;	NCBI: 1408912	4410975	99.94	0.00	Yes
NCBI Assembly	GCA_000666965.1	NCBI: cellular organisms; Bacteria; Terrabacteria group; Actinobacteria; Actinomycetia; Corynebacteriales; Mycobacteriaceae; Mycobacterium; Mycobacterium tuberculosis complex; Mycobacterium tuberculosis;	NCBI: 1354140	4402186	99.94	0.00	Yes
NCBI Assembly	GCA_000706425.1	NCBI: cellular organisms; Bacteria; Terrabacteria group; Actinobacteria; Actinomycetia; Corynebacteriales; Mycobacteriaceae; Mycobacterium; Mycobacterium tuberculosis complex; Mycobacterium tuberculosis;	NCBI: 1447450	4372861	99.94	0.00	Yes
NCBI Assembly	GCA_000674495.1	NCBI: cellular organisms; Bacteria; Terrabacteria group; Actinobacteria; Actinomycetia; Corynebacteriales; Mycobacteriaceae; Mycobacterium; Mycobacterium tuberculosis complex; Mycobacterium tuberculosis;	NCBI: 1400936	4435118	99.94	0.00	Yes
NCBI Assembly	GCA_000676355.1	NCBI: cellular organisms; Bacteria; Terrabacteria group; Actinobacteria; Actinomycetia; Corynebacteriales; Mycobacteriaceae; Mycobacterium; Mycobacterium tuberculosis complex; Mycobacterium tuberculosis;	NCBI: 1422071	4390526	99.69	0.33	Yes
NCBI Assembly	GCA_000675415.1	NCBI: cellular organisms; Bacteria; Terrabacteria group; Actinobacteria; Actinomycetia; Corynebacteriales; Mycobacteriaceae; Mycobacterium; Mycobacterium tuberculosis complex; Mycobacterium tuberculosis;	NCBI: 1422021	4405280	99.94	0.00	Yes
NCBI Assembly	GCA_000672575.1	NCBI: cellular organisms; Bacteria; Terrabacteria group; Actinobacteria; Actinomycetia; Corynebacteriales; Mycobacteriaceae; Mycobacterium; Mycobacterium tuberculosis complex; Mycobacterium tuberculosis;	NCBI: 1397894	4399964	99.94	0.00	Yes

NCBI Assembly	GCA_000803725.1	NCBI: cellular organisms; Bacteria; Terrabacteria group; Actinobacteria; Actinomycetia; Corynebacteriales; Mycobacteriaceae; Mycobacterium; Mycobacterium tuberculosis complex; Mycobacterium tuberculosis;	NCBI: 1773	4365663	99.94	0.00	Yes
NCBI Assembly	GCA_000671215.1	NCBI: cellular organisms; Bacteria; Terrabacteria group; Actinobacteria; Actinomycetia; Corynebacteriales; Mycobacteriaceae; Mycobacterium; Mycobacterium tuberculosis complex; Mycobacterium tuberculosis;	NCBI: 1421989	4411151	99.94	0.00	Yes
NCBI Assembly	GCA_000706105.1	NCBI: cellular organisms; Bacteria; Terrabacteria group; Actinobacteria; Actinomycetia; Corynebacteriales; Mycobacteriaceae; Mycobacterium; Mycobacterium tuberculosis complex; Mycobacterium tuberculosis;	NCBI: 1448749	4396032	99.94	0.28	Yes
NCBI Assembly	GCA_000676695.1	NCBI: cellular organisms; Bacteria; Terrabacteria group; Actinobacteria; Actinomycetia; Corynebacteriales; Mycobacteriaceae; Mycobacterium; Mycobacterium tuberculosis complex; Mycobacterium tuberculosis;	NCBI: 1422088	4407855	99.94	0.00	Yes
NCBI Assembly	GCA_000673535.1	NCBI: cellular organisms; Bacteria; Terrabacteria group; Actinobacteria; Actinomycetia; Corynebacteriales; Mycobacteriaceae; Mycobacterium; Mycobacterium tuberculosis complex; Mycobacterium tuberculosis;	NCBI: 1400871	4427245	99.94	0.00	Yes
NCBI Assembly	GCA_000674815.1	NCBI: cellular organisms; Bacteria; Terrabacteria group; Actinobacteria; Actinomycetia; Corynebacteriales; Mycobacteriaceae; Mycobacterium; Mycobacterium tuberculosis complex; Mycobacterium tuberculosis;	NCBI: 1408913	4411278	99.94	0.33	Yes
NCBI Assembly	GCA_000674515.1	NCBI: cellular organisms; Bacteria; Terrabacteria group; Actinobacteria; Actinomycetia; Corynebacteriales; Mycobacteriaceae; Mycobacterium; Mycobacterium tuberculosis complex; Mycobacterium tuberculosis;	NCBI: 1400937	4405909	99.94	0.00	Yes
NCBI Assembly	GCA_000666985.1	NCBI: cellular organisms; Bacteria; Terrabacteria group; Actinobacteria; Actinomycetia; Corynebacteriales; Mycobacteriaceae; Mycobacterium; Mycobacterium tuberculosis complex; Mycobacterium tuberculosis; Mycobacterium tuberculosis variant africanum;	NCBI: 1354187	4388747	99.94	0.00	Yes
NCBI Assembly	GCA_000676375.1	NCBI: cellular organisms; Bacteria; Terrabacteria group; Actinobacteria; Actinomycetia; Corynebacteriales; Mycobacteriaceae; Mycobacterium; Mycobacterium tuberculosis complex; Mycobacterium tuberculosis;	NCBI: 1422072	4407630	99.88	1.08	Yes
NCBI Assembly	GCA_000675435.1	NCBI: cellular organisms; Bacteria; Terrabacteria group; Actinobacteria; Actinomycetia; Corynebacteriales; Mycobacteriaceae; Mycobacterium; Mycobacterium tuberculosis complex; Mycobacterium tuberculosis;	NCBI: 1422022	4420026	99.94	0.00	Yes
NCBI Assembly	GCA_000672595.1	NCBI: cellular organisms; Bacteria; Terrabacteria group; Actinobacteria; Actinomycetia; Corynebacteriales; Mycobacteriaceae; Mycobacterium; Mycobacterium tuberculosis complex; Mycobacterium tuberculosis;	NCBI: 1397895	4428739	99.94	0.00	Yes
NCBI Assembly	GCA_000706445.1	NCBI: cellular organisms; Bacteria; Terrabacteria group; Actinobacteria; Actinomycetia; Corynebacteriales; Mycobacteriaceae; Mycobacterium; Mycobacterium tuberculosis complex; Mycobacterium tuberculosis;	NCBI: 1427279	4392053	99.94	0.00	Yes
NCBI Assembly	GCA_000671235.1	NCBI: cellular organisms; Bacteria; Terrabacteria group; Actinobacteria; Actinomycetia; Corynebacteriales; Mycobacteriaceae; Mycobacterium; Mycobacterium tuberculosis complex; Mycobacterium tuberculosis;	NCBI: 1421990	4412616	99.94	0.00	Yes
NCBI Assembly	GCA_000803735.1	NCBI: cellular organisms; Bacteria; Terrabacteria group; Actinobacteria; Actinomycetia; Corynebacteriales; Mycobacteriaceae; Mycobacterium; Mycobacterium tuberculosis complex; Mycobacterium tuberculosis;	NCBI: 1773	4359702	99.94	0.00	Yes
NCBI Assembly	GCA_000673555.1	NCBI: cellular organisms; Bacteria; Terrabacteria group; Actinobacteria; Actinomycetia; Corynebacteriales; Mycobacteriaceae; Mycobacterium; Mycobacterium tuberculosis complex; Mycobacterium tuberculosis;	NCBI: 1400872	4423697	99.61	0.00	Yes
NCBI Assembly	GCA_000706125.1	NCBI: cellular organisms; Bacteria; Terrabacteria group; Actinobacteria; Actinomycetia; Corynebacteriales; Mycobacteriaceae; Mycobacterium; Mycobacterium tuberculosis complex; Mycobacterium tuberculosis;	NCBI: 1448714	4430742	99.94	0.19	Yes
NCBI Assembly	GCA_000676715.1	NCBI: cellular organisms; Bacteria; Terrabacteria group; Actinobacteria; Actinomycetia; Corynebacteriales; Mycobacteriaceae; Mycobacterium; Mycobacterium tuberculosis complex; Mycobacterium tuberculosis;	NCBI: 1422089	4413988	99.94	0.33	Yes
NCBI Assembly	GCA_000674835.1	NCBI: cellular organisms; Bacteria; Terrabacteria group; Actinobacteria; Actinomycetia; Corynebacteriales; Mycobacteriaceae; Mycobacterium; Mycobacterium tuberculosis complex; Mycobacterium tuberculosis;	NCBI: 1408914	4430951	99.94	0.00	Yes

NCBI Assembly	GCA_000674535.1	NCBI: cellular organisms; Bacteria; Terrabacteria group; Actinobacteria; Actinomycetia; Corynebacteriales; Mycobacteriaceae; Mycobacterium; Mycobacterium tuberculosis complex; Mycobacterium tuberculosis;	NCBI: 1400939	4402534	99.90	0.00	Yes
NCBI Assembly	GCA_000672615.1	NCBI: cellular organisms; Bacteria; Terrabacteria group; Actinobacteria; Actinomycetia; Corynebacteriales; Mycobacteriaceae; Mycobacterium; Mycobacterium tuberculosis complex; Mycobacterium tuberculosis;	NCBI: 1397896	4430365	99.94	0.00	Yes
NCBI Assembly	GCA_000676395.1	NCBI: cellular organisms; Bacteria; Terrabacteria group; Actinobacteria; Actinomycetia; Corynebacteriales; Mycobacteriaceae; Mycobacterium; Mycobacterium tuberculosis complex; Mycobacterium tuberculosis;	NCBI: 1422073	4413470	99.94	0.00	Yes
NCBI Assembly	GCA_000675455.1	NCBI: cellular organisms; Bacteria; Terrabacteria group; Actinobacteria; Actinomycetia; Corynebacteriales; Mycobacteriaceae; Mycobacterium; Mycobacterium tuberculosis complex; Mycobacterium tuberculosis;	NCBI: 1422023	4456891	99.81	0.50	Yes
NCBI Assembly	GCA_000667005.1	NCBI: cellular organisms; Bacteria; Terrabacteria group; Actinobacteria; Actinomycetia; Corynebacteriales; Mycobacteriaceae; Mycobacterium; Mycobacterium tuberculosis complex; Mycobacterium tuberculosis; Mycobacterium tuberculosis variant africanum;	NCBI: 1354141	4397782	99.94	0.00	Yes
NCBI Assembly	GCA_000706465.1	NCBI: cellular organisms; Bacteria; Terrabacteria group; Actinobacteria; Actinomycetia; Corynebacteriales; Mycobacteriaceae; Mycobacterium; Mycobacterium tuberculosis complex; Mycobacterium tuberculosis;	NCBI: 1408972	4405838	99.94	0.00	Yes
NCBI Assembly	GCA_000671255.1	NCBI: cellular organisms; Bacteria; Terrabacteria group; Actinobacteria; Actinomycetia; Corynebacteriales; Mycobacteriaceae; Mycobacterium; Mycobacterium tuberculosis complex; Mycobacterium tuberculosis;	NCBI: 1421991	4439011	99.94	0.00	Yes
NCBI Assembly	GCA_000706145.1	NCBI: cellular organisms; Bacteria; Terrabacteria group; Actinobacteria; Actinomycetia; Corynebacteriales; Mycobacteriaceae; Mycobacterium; Mycobacterium tuberculosis complex; Mycobacterium tuberculosis;	NCBI: 1448655	4421955	99.94	0.00	Yes
NCBI Assembly	GCA_000676735.1	NCBI: cellular organisms; Bacteria; Terrabacteria group; Actinobacteria; Actinomycetia; Corynebacteriales; Mycobacteriaceae; Mycobacterium; Mycobacterium tuberculosis complex; Mycobacterium tuberculosis;	NCBI: 1422090	4411062	99.94	0.00	Yes
NCBI Assembly	GCA_000803745.1	NCBI: cellular organisms; Bacteria; Terrabacteria group; Actinobacteria; Actinomycetia; Corynebacteriales; Mycobacteriaceae; Mycobacterium; Mycobacterium tuberculosis complex; Mycobacterium tuberculosis;	NCBI: 1773	4375585	99.94	0.00	Yes
NCBI Assembly	GCA_000673575.1	NCBI: cellular organisms; Bacteria; Terrabacteria group; Actinobacteria; Actinomycetia; Corynebacteriales; Mycobacteriaceae; Mycobacterium; Mycobacterium tuberculosis complex; Mycobacterium tuberculosis;	NCBI: 1400873	4400684	99.94	0.00	Yes
NCBI Assembly	GCA_000674555.1	NCBI: cellular organisms; Bacteria; Terrabacteria group; Actinobacteria; Actinomycetia; Corynebacteriales; Mycobacteriaceae; Mycobacterium; Mycobacterium tuberculosis complex; Mycobacterium tuberculosis;	NCBI: 1400940	4393333	99.94	0.00	Yes
NCBI Assembly	GCA_000674855.1	NCBI: cellular organisms; Bacteria; Terrabacteria group; Actinobacteria; Actinomycetia; Corynebacteriales; Mycobacteriaceae; Mycobacterium; Mycobacterium tuberculosis complex; Mycobacterium tuberculosis;	NCBI: 1408915	4357533	99.94	0.00	Yes
NCBI Assembly	GCA_000675475.1	NCBI: cellular organisms; Bacteria; Terrabacteria group; Actinobacteria; Actinomycetia; Corynebacteriales; Mycobacteriaceae; Mycobacterium; Mycobacterium tuberculosis complex; Mycobacterium tuberculosis;	NCBI: 1422024	4408545	99.94	0.00	Yes
NCBI Assembly	GCA_000667025.1	NCBI: cellular organisms; Bacteria; Terrabacteria group; Actinobacteria; Actinomycetia; Corynebacteriales; Mycobacteriaceae; Mycobacterium; Mycobacterium tuberculosis complex; Mycobacterium tuberculosis;	NCBI: 1354122	4422781	99.94	0.00	Yes
NCBI Assembly	GCA_000676415.1	NCBI: cellular organisms; Bacteria; Terrabacteria group; Actinobacteria; Actinomycetia; Corynebacteriales; Mycobacteriaceae; Mycobacterium; Mycobacterium tuberculosis complex; Mycobacterium tuberculosis;	NCBI: 1422074	4433615	99.94	0.00	Yes
NCBI Assembly	GCA_000672635.1	NCBI: cellular organisms; Bacteria; Terrabacteria group; Actinobacteria; Actinomycetia; Corynebacteriales; Mycobacteriaceae; Mycobacterium; Mycobacterium tuberculosis complex; Mycobacterium tuberculosis;	NCBI: 1397897	4420289	99.94	0.00	Yes
NCBI Assembly	GCA_000706485.1	NCBI: cellular organisms; Bacteria; Terrabacteria group; Actinobacteria; Actinomycetia; Corynebacteriales; Mycobacteriaceae; Mycobacterium; Mycobacterium tuberculosis complex; Mycobacterium tuberculosis;	NCBI: 1400938	4396548	99.94	0.00	Yes

NCBI Assembly	GCA_000671275.1	NCBI: cellular organisms; Bacteria; Terrabacteria group; Actinobacteria; Actinomycetia; Corynebacteriales; Mycobacteriaceae; Mycobacterium; Mycobacterium tuberculosis complex; Mycobacterium tuberculosis;	NCBI: 1421992	4451857	99.94	0.00	Yes
NCBI Assembly	GCA_000673595.1	NCBI: cellular organisms; Bacteria; Terrabacteria group; Actinobacteria; Actinomycetia; Corynebacteriales; Mycobacteriaceae; Mycobacterium; Mycobacterium tuberculosis complex; Mycobacterium tuberculosis;	NCBI: 1400874	4401020	99.94	0.00	Yes
NCBI Assembly	GCA_000706165.1	NCBI: cellular organisms; Bacteria; Terrabacteria group; Actinobacteria; Actinomycetia; Corynebacteriales; Mycobacteriaceae; Mycobacterium; Mycobacterium tuberculosis complex; Mycobacterium tuberculosis;	NCBI: 1421968	4404078	99.94	0.00	Yes
NCBI Assembly	GCA_000676755.1	NCBI: cellular organisms; Bacteria; Terrabacteria group; Actinobacteria; Actinomycetia; Corynebacteriales; Mycobacteriaceae; Mycobacterium; Mycobacterium tuberculosis complex; Mycobacterium tuberculosis;	NCBI: 1422091	4415268	99.94	0.00	Yes
NCBI Assembly	GCA_000674575.1	NCBI: cellular organisms; Bacteria; Terrabacteria group; Actinobacteria; Actinomycetia; Corynebacteriales; Mycobacteriaceae; Mycobacterium; Mycobacterium tuberculosis complex; Mycobacterium tuberculosis;	NCBI: 1400941	4420833	99.94	0.00	Yes
NCBI Assembly	GCA_000676435.1	NCBI: cellular organisms; Bacteria; Terrabacteria group; Actinobacteria; Actinomycetia; Corynebacteriales; Mycobacteriaceae; Mycobacterium; Mycobacterium tuberculosis complex; Mycobacterium tuberculosis;	NCBI: 1422075	4392618	99.94	0.00	Yes
NCBI Assembly	GCA_000667045.1	NCBI: cellular organisms; Bacteria; Terrabacteria group; Actinobacteria; Actinomycetia; Corynebacteriales; Mycobacteriaceae; Mycobacterium; Mycobacterium tuberculosis complex; Mycobacterium tuberculosis;	NCBI: 1354123	4431914	99.94	0.00	Yes
NCBI Assembly	GCA_000803755.1	NCBI: cellular organisms; Bacteria; Terrabacteria group; Actinobacteria; Actinomycetia; Corynebacteriales; Mycobacteriaceae; Mycobacterium; Mycobacterium tuberculosis complex; Mycobacterium tuberculosis;	NCBI: 1773	4367796	99.94	0.03	Yes
NCBI Assembly	GCA_000675495.1	NCBI: cellular organisms; Bacteria; Terrabacteria group; Actinobacteria; Actinomycetia; Corynebacteriales; Mycobacteriaceae; Mycobacterium; Mycobacterium tuberculosis complex; Mycobacterium tuberculosis;	NCBI: 1422025	4404894	99.94	0.00	Yes
NCBI Assembly	GCA_000672655.1	NCBI: cellular organisms; Bacteria; Terrabacteria group; Actinobacteria; Actinomycetia; Corynebacteriales; Mycobacteriaceae; Mycobacterium; Mycobacterium tuberculosis complex; Mycobacterium tuberculosis;	NCBI: 1397899	4434355	99.94	0.00	Yes
NCBI Assembly	GCA_000674875.1	NCBI: cellular organisms; Bacteria; Terrabacteria group; Actinobacteria; Actinomycetia; Corynebacteriales; Mycobacteriaceae; Mycobacterium; Mycobacterium tuberculosis complex; Mycobacterium tuberculosis;	NCBI: 1421994	4407937	99.94	0.00	Yes
NCBI Assembly	GCA_000706505.1	NCBI: cellular organisms; Bacteria; Terrabacteria group; Actinobacteria; Actinomycetia; Corynebacteriales; Mycobacteriaceae; Mycobacterium; Mycobacterium tuberculosis complex; Mycobacterium tuberculosis;	NCBI: 1400922	4424057	99.94	0.00	Yes
NCBI Assembly	GCA_000673615.1	NCBI: cellular organisms; Bacteria; Terrabacteria group; Actinobacteria; Actinomycetia; Corynebacteriales; Mycobacteriaceae; Mycobacterium; Mycobacterium tuberculosis complex; Mycobacterium tuberculosis;	NCBI: 1400875	4411411	99.94	0.00	Yes
NCBI Assembly	GCA_000671775.1	NCBI: cellular organisms; Bacteria; Terrabacteria group; Actinobacteria; Actinomycetia; Corynebacteriales; Mycobacteriaceae; Mycobacterium; Mycobacterium tuberculosis complex; Mycobacterium tuberculosis;	NCBI: 1397853	4418681	99.94	0.00	Yes
NCBI Assembly	GCA_000706185.1	NCBI: cellular organisms; Bacteria; Terrabacteria group; Actinobacteria; Actinomycetia; Corynebacteriales; Mycobacteriaceae; Mycobacterium; Mycobacterium tuberculosis complex; Mycobacterium tuberculosis;	NCBI: 1354113	4435668	99.94	0.00	Yes
NCBI Assembly	GCA_000676775.1	NCBI: cellular organisms; Bacteria; Terrabacteria group; Actinobacteria; Actinomycetia; Corynebacteriales; Mycobacteriaceae; Mycobacterium; Mycobacterium tuberculosis complex; Mycobacterium tuberculosis;	NCBI: 1422092	4397362	99.94	0.69	Yes
NCBI Assembly	GCA_000667065.1	NCBI: cellular organisms; Bacteria; Terrabacteria group; Actinobacteria; Actinomycetia; Corynebacteriales; Mycobacteriaceae; Mycobacterium; Mycobacterium tuberculosis complex; Mycobacterium tuberculosis;	NCBI: 1354124	4414338	99.94	0.00	Yes
NCBI Assembly	GCA_000676455.1	NCBI: cellular organisms; Bacteria; Terrabacteria group; Actinobacteria; Actinomycetia; Corynebacteriales; Mycobacteriaceae; Mycobacterium; Mycobacterium tuberculosis complex; Mycobacterium tuberculosis;	NCBI: 1422076	4376032	99.94	0.22	Yes

NCBI Assembly	GCA_000674595.1	NCBI: cellular organisms; Bacteria; Terrabacteria group; Actinobacteria; Actinomycetia; Corynebacteriales; Mycobacteriaceae; Mycobacterium; Mycobacterium tuberculosis complex; Mycobacterium tuberculosis;	NCBI: 1402473	4367991	99.94	0.00	Yes
NCBI Assembly	GCA_000675515.1	NCBI: cellular organisms; Bacteria; Terrabacteria group; Actinobacteria; Actinomycetia; Corynebacteriales; Mycobacteriaceae; Mycobacterium; Mycobacterium tuberculosis complex; Mycobacterium tuberculosis;	NCBI: 1422026	4412677	99.94	0.00	Yes
NCBI Assembly	GCA_000674895.1	NCBI: cellular organisms; Bacteria; Terrabacteria group; Actinobacteria; Actinomycetia; Corynebacteriales; Mycobacteriaceae; Mycobacterium; Mycobacterium tuberculosis complex; Mycobacterium tuberculosis;	NCBI: 1421995	4422717	99.94	0.00	Yes
NCBI Assembly	GCA_000672675.1	NCBI: cellular organisms; Bacteria; Terrabacteria group; Actinobacteria; Actinomycetia; Corynebacteriales; Mycobacteriaceae; Mycobacterium; Mycobacterium tuberculosis complex; Mycobacterium tuberculosis;	NCBI: 1397900	4413455	99.94	0.00	Yes
NCBI Assembly	GCA_000803805.1	NCBI: cellular organisms; Bacteria; Terrabacteria group; Actinobacteria; Actinomycetia; Corynebacteriales; Mycobacteriaceae; Mycobacterium; Mycobacterium tuberculosis complex; Mycobacterium tuberculosis;	NCBI: 1773	4357814	99.94	0.00	Yes
NCBI Assembly	GCA_000706525.1	NCBI: cellular organisms; Bacteria; Terrabacteria group; Actinobacteria; Actinomycetia; Corynebacteriales; Mycobacteriaceae; Mycobacterium; Mycobacterium tuberculosis complex; Mycobacterium tuberculosis;	NCBI: 1400885	4414653	99.83	0.00	Yes
NCBI Assembly	GCA_000676795.1	NCBI: cellular organisms; Bacteria; Terrabacteria group; Actinobacteria; Actinomycetia; Corynebacteriales; Mycobacteriaceae; Mycobacterium; Mycobacterium tuberculosis complex; Mycobacterium tuberculosis;	NCBI: 1422093	4417610	99.94	0.06	Yes
NCBI Assembly	GCA_000673635.1	NCBI: cellular organisms; Bacteria; Terrabacteria group; Actinobacteria; Actinomycetia; Corynebacteriales; Mycobacteriaceae; Mycobacterium; Mycobacterium tuberculosis complex; Mycobacterium tuberculosis;	NCBI: 1400877	4394989	99.94	0.00	Yes
NCBI Assembly	GCA_000706205.1	NCBI: cellular organisms; Bacteria; Terrabacteria group; Actinobacteria; Actinomycetia; Corynebacteriales; Mycobacteriaceae; Mycobacterium; Mycobacterium tuberculosis complex; Mycobacterium tuberculosis;	NCBI: 1354156	4410222	99.94	0.00	Yes
NCBI Assembly	GCA_000671795.1	NCBI: cellular organisms; Bacteria; Terrabacteria group; Actinobacteria; Actinomycetia; Corynebacteriales; Mycobacteriaceae; Mycobacterium; Mycobacterium tuberculosis complex; Mycobacterium tuberculosis;	NCBI: 1397854	4416426	99.94	0.00	Yes
NCBI Assembly	GCA_000676475.1	NCBI: cellular organisms; Bacteria; Terrabacteria group; Actinobacteria; Actinomycetia; Corynebacteriales; Mycobacteriaceae; Mycobacterium; Mycobacterium tuberculosis complex; Mycobacterium tuberculosis;	NCBI: 1422077	4393864	99.94	0.00	Yes
NCBI Assembly	GCA_000667085.1	NCBI: cellular organisms; Bacteria; Terrabacteria group; Actinobacteria; Actinomycetia; Corynebacteriales; Mycobacteriaceae; Mycobacterium; Mycobacterium tuberculosis complex; Mycobacterium tuberculosis;	NCBI: 1354125	4416118	99.94	0.00	Yes
NCBI Assembly	GCA_000674615.1	NCBI: cellular organisms; Bacteria; Terrabacteria group; Actinobacteria; Actinomycetia; Corynebacteriales; Mycobacteriaceae; Mycobacterium; Mycobacterium tuberculosis complex; Mycobacterium tuberculosis;	NCBI: 1402475	4386485	99.94	0.00	Yes
NCBI Assembly	GCA_000674915.1	NCBI: cellular organisms; Bacteria; Terrabacteria group; Actinobacteria; Actinomycetia; Corynebacteriales; Mycobacteriaceae; Mycobacterium; Mycobacterium tuberculosis complex; Mycobacterium tuberculosis;	NCBI: 1421996	4407189	99.94	0.00	Yes
NCBI Assembly	GCA_000672695.1	NCBI: cellular organisms; Bacteria; Terrabacteria group; Actinobacteria; Actinomycetia; Corynebacteriales; Mycobacteriaceae; Mycobacterium; Mycobacterium tuberculosis complex; Mycobacterium tuberculosis;	NCBI: 1397901	4442504	99.94	0.00	Yes
NCBI Assembly	GCA_000675535.1	NCBI: cellular organisms; Bacteria; Terrabacteria group; Actinobacteria; Actinomycetia; Corynebacteriales; Mycobacteriaceae; Mycobacterium; Mycobacterium tuberculosis complex; Mycobacterium tuberculosis;	NCBI: 1422027	4424182	99.94	0.00	Yes
NCBI Assembly	GCA_000803825.1	NCBI: cellular organisms; Bacteria; Terrabacteria group; Actinobacteria; Actinomycetia; Corynebacteriales; Mycobacteriaceae; Mycobacterium; Mycobacterium tuberculosis complex; Mycobacterium tuberculosis;	NCBI: 1773	4357371	99.94	0.11	Yes
NCBI Assembly	GCA_000706545.1	NCBI: cellular organisms; Bacteria; Terrabacteria group; Actinobacteria; Actinomycetia; Corynebacteriales; Mycobacteriaceae; Mycobacterium; Mycobacterium tuberculosis complex; Mycobacterium tuberculosis;	NCBI: 1267357	4411623	99.94	0.00	Yes

NCBI Assembly	GCA_000676815.1	NCBI: cellular organisms; Bacteria; Terrabacteria group; Actinobacteria; Actinomycetia; Corynebacteriales; Mycobacteriaceae; Mycobacterium; Mycobacterium tuberculosis complex; Mycobacterium tuberculosis;	NCBI: 1422094	4411486	99.94	0.00	Yes
NCBI Assembly	GCA_000671815.1	NCBI: cellular organisms; Bacteria; Terrabacteria group; Actinobacteria; Actinomycetia; Corynebacteriales; Mycobacteriaceae; Mycobacterium; Mycobacterium tuberculosis complex; Mycobacterium tuberculosis;	NCBI: 1397855	4416961	99.94	0.00	Yes
NCBI Assembly	GCA_000673655.1	NCBI: cellular organisms; Bacteria; Terrabacteria group; Actinobacteria; Actinomycetia; Corynebacteriales; Mycobacteriaceae; Mycobacterium; Mycobacterium tuberculosis complex; Mycobacterium tuberculosis;	NCBI: 1400879	4403786	99.94	0.17	Yes
NCBI Assembly	GCA_000667105.1	NCBI: cellular organisms; Bacteria; Terrabacteria group; Actinobacteria; Actinomycetia; Corynebacteriales; Mycobacteriaceae; Mycobacterium; Mycobacterium tuberculosis complex; Mycobacterium tuberculosis;	NCBI: 1354126	4428177	99.94	0.00	Yes
NCBI Assembly	GCA_000676495.1	NCBI: cellular organisms; Bacteria; Terrabacteria group; Actinobacteria; Actinomycetia; Corynebacteriales; Mycobacteriaceae; Mycobacterium; Mycobacterium tuberculosis complex; Mycobacterium tuberculosis;	NCBI: 1422078	4398918	99.94	0.00	Yes
NCBI Assembly	GCA_000674635.1	NCBI: cellular organisms; Bacteria; Terrabacteria group; Actinobacteria; Actinomycetia; Corynebacteriales; Mycobacteriaceae; Mycobacterium; Mycobacterium tuberculosis complex; Mycobacterium tuberculosis;	NCBI: 1402480	4392095	99.94	0.00	Yes
NCBI Assembly	GCA_000706225.1	NCBI: cellular organisms; Bacteria; Terrabacteria group; Actinobacteria; Actinomycetia; Corynebacteriales; Mycobacteriaceae; Mycobacterium; Mycobacterium tuberculosis complex; Mycobacterium tuberculosis;	NCBI: 1354159	4412063	99.94	0.00	Yes
NCBI Assembly	GCA_000674935.1	NCBI: cellular organisms; Bacteria; Terrabacteria group; Actinobacteria; Actinomycetia; Corynebacteriales; Mycobacteriaceae; Mycobacterium; Mycobacterium tuberculosis complex; Mycobacterium tuberculosis;	NCBI: 1421997	4397284	99.94	0.00	Yes
NCBI Assembly	GCA_000675555.1	NCBI: cellular organisms; Bacteria; Terrabacteria group; Actinobacteria; Actinomycetia; Corynebacteriales; Mycobacteriaceae; Mycobacterium; Mycobacterium tuberculosis complex; Mycobacterium tuberculosis;	NCBI: 1422029	4462579	99.83	0.15	Yes
NCBI Assembly	GCA_000672715.1	NCBI: cellular organisms; Bacteria; Terrabacteria group; Actinobacteria; Actinomycetia; Corynebacteriales; Mycobacteriaceae; Mycobacterium; Mycobacterium tuberculosis complex; Mycobacterium tuberculosis;	NCBI: 1397902	4404562	99.94	0.00	Yes
NCBI Assembly	GCA_000706565.1	NCBI: cellular organisms; Bacteria; Terrabacteria group; Actinobacteria; Actinomycetia; Corynebacteriales; Mycobacteriaceae; Mycobacterium; Mycobacterium tuberculosis complex; Mycobacterium tuberculosis;	NCBI: 1448586	4409529	99.94	0.00	Yes
NCBI Assembly	GCA_000803845.1	NCBI: cellular organisms; Bacteria; Terrabacteria group; Actinobacteria; Actinomycetia; Corynebacteriales; Mycobacteriaceae; Mycobacterium; Mycobacterium tuberculosis complex; Mycobacterium tuberculosis;	NCBI: 1773	4371933	99.86	0.00	Yes
NCBI Assembly	GCA_000673675.1	NCBI: cellular organisms; Bacteria; Terrabacteria group; Actinobacteria; Actinomycetia; Corynebacteriales; Mycobacteriaceae; Mycobacterium; Mycobacterium tuberculosis complex; Mycobacterium tuberculosis;	NCBI: 1400880	4415064	99.94	0.00	Yes
NCBI Assembly	GCA_000667125.1	NCBI: cellular organisms; Bacteria; Terrabacteria group; Actinobacteria; Actinomycetia; Corynebacteriales; Mycobacteriaceae; Mycobacterium; Mycobacterium tuberculosis complex; Mycobacterium tuberculosis;	NCBI: 1354127	4417108	99.94	0.00	Yes
NCBI Assembly	GCA_000676835.1	NCBI: cellular organisms; Bacteria; Terrabacteria group; Actinobacteria; Actinomycetia; Corynebacteriales; Mycobacteriaceae; Mycobacterium; Mycobacterium tuberculosis complex; Mycobacterium tuberculosis;	NCBI: 1422095	4404017	99.94	0.00	Yes
NCBI Assembly	GCA_000674955.1	NCBI: cellular organisms; Bacteria; Terrabacteria group; Actinobacteria; Actinomycetia; Corynebacteriales; Mycobacteriaceae; Mycobacterium; Mycobacterium tuberculosis complex; Mycobacterium tuberculosis;	NCBI: 1421998	4395768	99.94	0.00	Yes
NCBI Assembly	GCA_000671835.1	NCBI: cellular organisms; Bacteria; Terrabacteria group; Actinobacteria; Actinomycetia; Corynebacteriales; Mycobacteriaceae; Mycobacterium; Mycobacterium tuberculosis complex; Mycobacterium tuberculosis;	NCBI: 1397856	4407969	99.94	0.00	Yes
NCBI Assembly	GCA_000706245.1	NCBI: cellular organisms; Bacteria; Terrabacteria group; Actinobacteria; Actinomycetia; Corynebacteriales; Mycobacteriaceae; Mycobacterium; Mycobacterium tuberculosis complex; Mycobacterium tuberculosis;	NCBI: 1354157	4419023	99.94	0.00	Yes

NCBI Assembly	GCA_000676515.1	NCBI: cellular organisms; Bacteria; Terrabacteria group; Actinobacteria; Actinomycetia; Corynebacteriales; Mycobacteriaceae; Mycobacterium; Mycobacterium tuberculosis complex; Mycobacterium tuberculosis;	NCBI: 1422079	4410144	99.94	0.00	Yes
NCBI Assembly	GCA_000675575.1	NCBI: cellular organisms; Bacteria; Terrabacteria group; Actinobacteria; Actinomycetia; Corynebacteriales; Mycobacteriaceae; Mycobacterium; Mycobacterium tuberculosis complex; Mycobacterium tuberculosis;	NCBI: 1422030	4416700	99.94	0.17	Yes
NCBI Assembly	GCA_000672735.1	NCBI: cellular organisms; Bacteria; Terrabacteria group; Actinobacteria; Actinomycetia; Corynebacteriales; Mycobacteriaceae; Mycobacterium; Mycobacterium tuberculosis complex; Mycobacterium tuberculosis;	NCBI: 1397903	4409378	99.94	0.00	Yes
NCBI Assembly	GCA_000736175.1	NCBI: cellular organisms; Bacteria; Terrabacteria group; Actinobacteria; Actinomycetia; Corynebacteriales; Mycobacteriaceae; Mycobacterium; Mycobacterium tuberculosis complex; Mycobacterium tuberculosis;	NCBI: 1773	4539435	97.18	0.57	Yes
NCBI Assembly	GCA_000803865.1	NCBI: cellular organisms; Bacteria; Terrabacteria group; Actinobacteria; Actinomycetia; Corynebacteriales; Mycobacteriaceae; Mycobacterium; Mycobacterium tuberculosis complex; Mycobacterium tuberculosis;	NCBI: 1773	4359670	99.94	0.00	Yes
NCBI Assembly	GCA_000706665.1	NCBI: cellular organisms; Bacteria; Terrabacteria group; Actinobacteria; Actinomycetia; Corynebacteriales; Mycobacteriaceae; Mycobacterium; Mycobacterium tuberculosis complex; Mycobacterium tuberculosis;	NCBI: 1773	4410788	99.94	0.33	Yes
NCBI Assembly	GCA_000667145.1	NCBI: cellular organisms; Bacteria; Terrabacteria group; Actinobacteria; Actinomycetia; Corynebacteriales; Mycobacteriaceae; Mycobacterium; Mycobacterium tuberculosis complex; Mycobacterium tuberculosis;	NCBI: 1354128	4412007	99.94	0.00	Yes
NCBI Assembly	GCA_000673695.1	NCBI: cellular organisms; Bacteria; Terrabacteria group; Actinobacteria; Actinomycetia; Corynebacteriales; Mycobacteriaceae; Mycobacterium; Mycobacterium tuberculosis complex; Mycobacterium tuberculosis;	NCBI: 1400881	4390654	99.94	0.00	Yes
NCBI Assembly	GCA_000674035.1	NCBI: cellular organisms; Bacteria; Terrabacteria group; Actinobacteria; Actinomycetia; Corynebacteriales; Mycobacteriaceae; Mycobacterium; Mycobacterium tuberculosis complex; Mycobacterium tuberculosis;	NCBI: 1400908	4572617	99.94	0.17	Yes
NCBI Assembly	GCA_000670735.1	NCBI: cellular organisms; Bacteria; Terrabacteria group; Actinobacteria; Actinomycetia; Corynebacteriales; Mycobacteriaceae; Mycobacterium; Mycobacterium tuberculosis complex; Mycobacterium tuberculosis;	NCBI: 1421961	4399247	99.94	0.00	Yes
NCBI Assembly	GCA_000673095.1	NCBI: cellular organisms; Bacteria; Terrabacteria group; Actinobacteria; Actinomycetia; Corynebacteriales; Mycobacteriaceae; Mycobacterium; Mycobacterium tuberculosis complex; Mycobacterium tuberculosis;	NCBI: 1397921	4424291	99.94	0.00	Yes
NCBI Assembly	GCA_000735875.1	NCBI: cellular organisms; Bacteria; Terrabacteria group; Actinobacteria; Actinomycetia; Corynebacteriales; Mycobacteriaceae; Mycobacterium; Mycobacterium tuberculosis complex; Mycobacterium tuberculosis;	NCBI: 1773	4539435	94.99	0.57	Yes
NCBI Assembly	GCA_000706265.1	NCBI: cellular organisms; Bacteria; Terrabacteria group; Actinobacteria; Actinomycetia; Corynebacteriales; Mycobacteriaceae; Mycobacterium; Mycobacterium tuberculosis complex; Mycobacterium tuberculosis;	NCBI: 1447518	4438207	99.94	0.09	Yes
NCBI Assembly	GCA_000672155.1	NCBI: cellular organisms; Bacteria; Terrabacteria group; Actinobacteria; Actinomycetia; Corynebacteriales; Mycobacteriaceae; Mycobacterium; Mycobacterium tuberculosis complex; Mycobacterium tuberculosis;	NCBI: 1397873	4398759	99.94	0.00	Yes
NCBI Assembly	GCA_000672755.1	NCBI: cellular organisms; Bacteria; Terrabacteria group; Actinobacteria; Actinomycetia; Corynebacteriales; Mycobacteriaceae; Mycobacterium; Mycobacterium tuberculosis complex; Mycobacterium tuberculosis;	NCBI: 1397904	4409364	99.94	0.00	Yes
NCBI Assembly	GCA_000736195.1	NCBI: cellular organisms; Bacteria; Terrabacteria group; Actinobacteria; Actinomycetia; Corynebacteriales; Mycobacteriaceae; Mycobacterium; Mycobacterium tuberculosis complex; Mycobacterium tuberculosis;	NCBI: 1773	4539435	84.31	0.50	No
NCBI Assembly	GCA_000665085.1	NCBI: cellular organisms; Bacteria; Terrabacteria group; Actinobacteria; Actinomycetia; Corynebacteriales; Mycobacteriaceae; Mycobacterium; Mycobacterium tuberculosis complex; Mycobacterium tuberculosis;	NCBI: 1448795	4423458	99.83	0.00	Yes
NCBI Assembly	GCA_000803885.1	NCBI: cellular organisms; Bacteria; Terrabacteria group; Actinobacteria; Actinomycetia; Corynebacteriales; Mycobacteriaceae; Mycobacterium; Mycobacterium tuberculosis complex; Mycobacterium tuberculosis;	NCBI: 1773	4364458	99.94	0.00	Yes

NCBI Assembly	GCA_000667165.1	NCBI: cellular organisms; Bacteria; Terrabacteria group; Actinobacteria; Actinomycetia; Corynebacteriales; Mycobacteriaceae; Mycobacterium; Mycobacterium tuberculosis complex; Mycobacterium tuberculosis;	NCBI: 1354129	4416076	99.94	0.00	Yes
NCBI Assembly	GCA_000668155.1	NCBI: cellular organisms; Bacteria; Terrabacteria group; Actinobacteria; Actinomycetia; Corynebacteriales; Mycobacteriaceae; Mycobacterium; Mycobacterium tuberculosis complex; Mycobacterium tuberculosis;	NCBI: 1402599	4407019	99.94	0.00	Yes
NCBI Assembly	GCA_000669435.1	NCBI: cellular organisms; Bacteria; Terrabacteria group; Actinobacteria; Actinomycetia; Corynebacteriales; Mycobacteriaceae; Mycobacterium; Mycobacterium tuberculosis complex; Mycobacterium tuberculosis;	NCBI: 1417015	4377728	99.94	0.00	Yes
NCBI Assembly	GCA_000670755.1	NCBI: cellular organisms; Bacteria; Terrabacteria group; Actinobacteria; Actinomycetia; Corynebacteriales; Mycobacteriaceae; Mycobacterium; Mycobacterium tuberculosis complex; Mycobacterium tuberculosis;	NCBI: 1421962	4410698	99.94	0.00	Yes
NCBI Assembly	GCA_000672175.1	NCBI: cellular organisms; Bacteria; Terrabacteria group; Actinobacteria; Actinomycetia; Corynebacteriales; Mycobacteriaceae; Mycobacterium; Mycobacterium tuberculosis complex; Mycobacterium tuberculosis;	NCBI: 1397874	4408950	99.94	0.00	Yes
NCBI Assembly	GCA_000674055.1	NCBI: cellular organisms; Bacteria; Terrabacteria group; Actinobacteria; Actinomycetia; Corynebacteriales; Mycobacteriaceae; Mycobacterium; Mycobacterium tuberculosis complex; Mycobacterium tuberculosis;	NCBI: 1400909	4399549	99.94	0.00	Yes
NCBI Assembly	GCA_000673115.1	NCBI: cellular organisms; Bacteria; Terrabacteria group; Actinobacteria; Actinomycetia; Corynebacteriales; Mycobacteriaceae; Mycobacterium; Mycobacterium tuberculosis complex; Mycobacterium tuberculosis;	NCBI: 1397922	4398215	99.94	0.00	Yes
NCBI Assembly	GCA_000735895.1	NCBI: cellular organisms; Bacteria; Terrabacteria group; Actinobacteria; Actinomycetia; Corynebacteriales; Mycobacteriaceae; Mycobacterium; Mycobacterium tuberculosis complex; Mycobacterium tuberculosis;	NCBI: 1773	4539435	95.67	0.57	Yes
NCBI Assembly	GCA_000672775.1	NCBI: cellular organisms; Bacteria; Terrabacteria group; Actinobacteria; Actinomycetia; Corynebacteriales; Mycobacteriaceae; Mycobacterium; Mycobacterium tuberculosis complex; Mycobacterium tuberculosis;	NCBI: 1397905	4402709	99.94	0.00	Yes
NCBI Assembly	GCA_000736215.1	NCBI: cellular organisms; Bacteria; Terrabacteria group; Actinobacteria; Actinomycetia; Corynebacteriales; Mycobacteriaceae; Mycobacterium; Mycobacterium tuberculosis complex; Mycobacterium tuberculosis;	NCBI: 1773	4539435	96.74	0.50	Yes
NCBI Assembly	GCA_000667185.1	NCBI: cellular organisms; Bacteria; Terrabacteria group; Actinobacteria; Actinomycetia; Corynebacteriales; Mycobacteriaceae; Mycobacterium; Mycobacterium tuberculosis complex; Mycobacterium tuberculosis;	NCBI: 1354130	4412861	99.94	0.00	Yes
NCBI Assembly	GCA_000665105.1	NCBI: cellular organisms; Bacteria; Terrabacteria group; Actinobacteria; Actinomycetia; Corynebacteriales; Mycobacteriaceae; Mycobacterium; Mycobacterium tuberculosis complex; Mycobacterium tuberculosis;	NCBI: 1448796	4415210	99.94	0.00	Yes
NCBI Assembly	GCA_000803905.1	NCBI: cellular organisms; Bacteria; Terrabacteria group; Actinobacteria; Actinomycetia; Corynebacteriales; Mycobacteriaceae; Mycobacterium; Mycobacterium tuberculosis complex; Mycobacterium tuberculosis;	NCBI: 1773	4358972	99.94	0.00	Yes
NCBI Assembly	GCA_000670775.1	NCBI: cellular organisms; Bacteria; Terrabacteria group; Actinobacteria; Actinomycetia; Corynebacteriales; Mycobacteriaceae; Mycobacterium; Mycobacterium tuberculosis complex; Mycobacterium tuberculosis;	NCBI: 1421964	4409653	99.94	0.00	Yes
NCBI Assembly	GCA_000668175.1	NCBI: cellular organisms; Bacteria; Terrabacteria group; Actinobacteria; Actinomycetia; Corynebacteriales; Mycobacteriaceae; Mycobacterium; Mycobacterium tuberculosis complex; Mycobacterium tuberculosis;	NCBI: 1402600	4418851	99.94	0.00	Yes
NCBI Assembly	GCA_000669455.1	NCBI: cellular organisms; Bacteria; Terrabacteria group; Actinobacteria; Actinomycetia; Corynebacteriales; Mycobacteriaceae; Mycobacterium; Mycobacterium tuberculosis complex; Mycobacterium tuberculosis;	NCBI: 1417016	4393923	99.94	0.00	Yes
NCBI Assembly	GCA_000672195.1	NCBI: cellular organisms; Bacteria; Terrabacteria group; Actinobacteria; Actinomycetia; Corynebacteriales; Mycobacteriaceae; Mycobacterium; Mycobacterium tuberculosis complex; Mycobacterium tuberculosis;	NCBI: 1397875	4404262	99.94	0.00	Yes
NCBI Assembly	GCA_000674075.1	NCBI: cellular organisms; Bacteria; Terrabacteria group; Actinobacteria; Actinomycetia; Corynebacteriales; Mycobacteriaceae; Mycobacterium; Mycobacterium tuberculosis complex; Mycobacterium tuberculosis;	NCBI: 1400911	4396837	99.61	0.00	Yes

NCBI Assembly	GCA_000672795.1	NCBI: cellular organisms; Bacteria; Terrabacteria group; Actinobacteria; Actinomycetia; Corynebacteriales; Mycobacteriaceae; Mycobacterium; Mycobacterium tuberculosis complex; Mycobacterium tuberculosis;	NCBI: 1397906	4409634	99.94	0.00	Yes
NCBI Assembly	GCA_000673135.1	NCBI: cellular organisms; Bacteria; Terrabacteria group; Actinobacteria; Actinomycetia; Corynebacteriales; Mycobacteriaceae; Mycobacterium; Mycobacterium tuberculosis complex; Mycobacterium tuberculosis;	NCBI: 1397923	4445334	99.94	0.00	Yes
NCBI Assembly	GCA_000735915.1	NCBI: cellular organisms; Bacteria; Terrabacteria group; Actinobacteria; Actinomycetia; Corynebacteriales; Mycobacteriaceae; Mycobacterium; Mycobacterium tuberculosis complex; Mycobacterium tuberculosis;	NCBI: 1773	4539435	91.56	0.57	Yes
NCBI Assembly	GCA_000667205.1	NCBI: cellular organisms; Bacteria; Terrabacteria group; Actinobacteria; Actinomycetia; Corynebacteriales; Mycobacteriaceae; Mycobacterium; Mycobacterium tuberculosis complex; Mycobacterium tuberculosis;	NCBI: 1354131	4425930	99.94	0.00	Yes
NCBI Assembly	GCA_000736235.1	NCBI: cellular organisms; Bacteria; Terrabacteria group; Actinobacteria; Actinomycetia; Corynebacteriales; Mycobacteriaceae; Mycobacterium; Mycobacterium tuberculosis complex; Mycobacterium tuberculosis;	NCBI: 1773	4539435	97.33	0.57	Yes
NCBI Assembly	GCA_000665125.1	NCBI: cellular organisms; Bacteria; Terrabacteria group; Actinobacteria; Actinomycetia; Corynebacteriales; Mycobacteriaceae; Mycobacterium; Mycobacterium tuberculosis complex; Mycobacterium tuberculosis;	NCBI: 1448797	4393717	99.94	0.00	Yes
NCBI Assembly	GCA_000668195.1	NCBI: cellular organisms; Bacteria; Terrabacteria group; Actinobacteria; Actinomycetia; Corynebacteriales; Mycobacteriaceae; Mycobacterium; Mycobacterium tuberculosis complex; Mycobacterium tuberculosis;	NCBI: 1402601	4426235	99.94	0.00	Yes
NCBI Assembly	GCA_000669475.1	NCBI: cellular organisms; Bacteria; Terrabacteria group; Actinobacteria; Actinomycetia; Corynebacteriales; Mycobacteriaceae; Mycobacterium; Mycobacterium tuberculosis complex; Mycobacterium tuberculosis;	NCBI: 1417017	4425749	99.94	1.00	Yes
NCBI Assembly	GCA_000670795.1	NCBI: cellular organisms; Bacteria; Terrabacteria group; Actinobacteria; Actinomycetia; Corynebacteriales; Mycobacteriaceae; Mycobacterium; Mycobacterium tuberculosis complex; Mycobacterium tuberculosis;	NCBI: 1421965	4430324	99.94	0.00	Yes
NCBI Assembly	GCA_000672215.1	NCBI: cellular organisms; Bacteria; Terrabacteria group; Actinobacteria; Actinomycetia; Corynebacteriales; Mycobacteriaceae; Mycobacterium; Mycobacterium tuberculosis complex; Mycobacterium tuberculosis;	NCBI: 1397876	4402453	99.94	0.00	Yes
NCBI Assembly	GCA_000803925.1	NCBI: cellular organisms; Bacteria; Terrabacteria group; Actinobacteria; Actinomycetia; Corynebacteriales; Mycobacteriaceae; Mycobacterium; Mycobacterium tuberculosis complex; Mycobacterium tuberculosis;	NCBI: 1773	4350857	99.94	0.00	Yes
NCBI Assembly	GCA_000674095.1	NCBI: cellular organisms; Bacteria; Terrabacteria group; Actinobacteria; Actinomycetia; Corynebacteriales; Mycobacteriaceae; Mycobacterium; Mycobacterium tuberculosis complex; Mycobacterium tuberculosis;	NCBI: 1400912	4481811	99.94	0.00	Yes
NCBI Assembly	GCA_000672815.1	NCBI: cellular organisms; Bacteria; Terrabacteria group; Actinobacteria; Actinomycetia; Corynebacteriales; Mycobacteriaceae; Mycobacterium; Mycobacterium tuberculosis complex; Mycobacterium tuberculosis;	NCBI: 1397907	4407543	99.94	0.00	Yes
NCBI Assembly	GCA_000673155.1	NCBI: cellular organisms; Bacteria; Terrabacteria group; Actinobacteria; Actinomycetia; Corynebacteriales; Mycobacteriaceae; Mycobacterium; Mycobacterium tuberculosis complex; Mycobacterium tuberculosis;	NCBI: 1397924	4408292	99.94	0.00	Yes
NCBI Assembly	GCA_000735935.1	NCBI: cellular organisms; Bacteria; Terrabacteria group; Actinobacteria; Actinomycetia; Corynebacteriales; Mycobacteriaceae; Mycobacterium; Mycobacterium tuberculosis complex; Mycobacterium tuberculosis;	NCBI: 1773	4539435	94.81	0.50	Yes
NCBI Assembly	GCA_000667225.1	NCBI: cellular organisms; Bacteria; Terrabacteria group; Actinobacteria; Actinomycetia; Corynebacteriales; Mycobacteriaceae; Mycobacterium; Mycobacterium tuberculosis complex; Mycobacterium tuberculosis;	NCBI: 1354132	4410076	99.94	0.00	Yes
NCBI Assembly	GCA_000736255.1	NCBI: cellular organisms; Bacteria; Terrabacteria group; Actinobacteria; Actinomycetia; Corynebacteriales; Mycobacteriaceae; Mycobacterium; Mycobacterium tuberculosis complex; Mycobacterium tuberculosis;	NCBI: 1773	4539435	97.33	0.57	Yes
NCBI Assembly	GCA_000665145.1	NCBI: cellular organisms; Bacteria; Terrabacteria group; Actinobacteria; Actinomycetia; Corynebacteriales; Mycobacteriaceae; Mycobacterium; Mycobacterium tuberculosis complex; Mycobacterium tuberculosis;	NCBI: 1448798	4393057	99.94	0.00	Yes

NCBI Assembly	GCA_000668215.1	NCBI: cellular organisms; Bacteria; Terrabacteria group; Actinobacteria; Actinomycetia; Corynebacteriales; Mycobacteriaceae; Mycobacterium; Mycobacterium tuberculosis complex; Mycobacterium tuberculosis;	NCBI: 1402602	4440769	99.94	0.00	Yes
NCBI Assembly	GCA_000669495.1	NCBI: cellular organisms; Bacteria; Terrabacteria group; Actinobacteria; Actinomycetia; Corynebacteriales; Mycobacteriaceae; Mycobacterium; Mycobacterium tuberculosis complex; Mycobacterium tuberculosis;	NCBI: 1417018	4375779	99.94	0.00	Yes
NCBI Assembly	GCA_000670815.1	NCBI: cellular organisms; Bacteria; Terrabacteria group; Actinobacteria; Actinomycetia; Corynebacteriales; Mycobacteriaceae; Mycobacterium; Mycobacterium tuberculosis complex; Mycobacterium tuberculosis;	NCBI: 1421966	4403018	99.94	0.00	Yes
NCBI Assembly	GCA_000672235.1	NCBI: cellular organisms; Bacteria; Terrabacteria group; Actinobacteria; Actinomycetia; Corynebacteriales; Mycobacteriaceae; Mycobacterium; Mycobacterium tuberculosis complex; Mycobacterium tuberculosis;	NCBI: 1397877	4415582	99.94	0.00	Yes
NCBI Assembly	GCA_000674115.1	NCBI: cellular organisms; Bacteria; Terrabacteria group; Actinobacteria; Actinomycetia; Corynebacteriales; Mycobacteriaceae; Mycobacterium; Mycobacterium tuberculosis complex; Mycobacterium tuberculosis;	NCBI: 1400914	4390593	99.94	0.00	Yes
NCBI Assembly	GCA_000672835.1	NCBI: cellular organisms; Bacteria; Terrabacteria group; Actinobacteria; Actinomycetia; Corynebacteriales; Mycobacteriaceae; Mycobacterium; Mycobacterium tuberculosis complex; Mycobacterium tuberculosis;	NCBI: 1397908	4405483	99.94	0.00	Yes
NCBI Assembly	GCA_000673175.1	NCBI: cellular organisms; Bacteria; Terrabacteria group; Actinobacteria; Actinomycetia; Corynebacteriales; Mycobacteriaceae; Mycobacterium; Mycobacterium tuberculosis complex; Mycobacterium tuberculosis;	NCBI: 1397925	4412946	99.94	0.00	Yes
NCBI Assembly	GCA_000803945.1	NCBI: cellular organisms; Bacteria; Terrabacteria group; Actinobacteria; Actinomycetia; Corynebacteriales; Mycobacteriaceae; Mycobacterium; Mycobacterium tuberculosis complex; Mycobacterium tuberculosis;	NCBI: 1773	4390884	99.94	0.00	Yes
NCBI Assembly	GCA_000667245.1	NCBI: cellular organisms; Bacteria; Terrabacteria group; Actinobacteria; Actinomycetia; Corynebacteriales; Mycobacteriaceae; Mycobacterium; Mycobacterium tuberculosis complex; Mycobacterium tuberculosis;	NCBI: 1354133	4429596	99.94	0.00	Yes
NCBI Assembly	GCA_000735955.1	NCBI: cellular organisms; Bacteria; Terrabacteria group; Actinobacteria; Actinomycetia; Corynebacteriales; Mycobacteriaceae; Mycobacterium; Mycobacterium tuberculosis complex; Mycobacterium tuberculosis;	NCBI: 1773	4539435	93.63	0.50	Yes
NCBI Assembly	GCA_000668255.1	NCBI: cellular organisms; Bacteria; Terrabacteria group; Actinobacteria; Actinomycetia; Corynebacteriales; Mycobacteriaceae; Mycobacterium; Mycobacterium tuberculosis complex; Mycobacterium tuberculosis;	NCBI: 1408918	4412615	99.94	0.56	Yes
NCBI Assembly	GCA_000665165.1	NCBI: cellular organisms; Bacteria; Terrabacteria group; Actinobacteria; Actinomycetia; Corynebacteriales; Mycobacteriaceae; Mycobacterium; Mycobacterium tuberculosis complex; Mycobacterium tuberculosis;	NCBI: 1448799	4427854	99.94	0.00	Yes
NCBI Assembly	GCA_000670835.1	NCBI: cellular organisms; Bacteria; Terrabacteria group; Actinobacteria; Actinomycetia; Corynebacteriales; Mycobacteriaceae; Mycobacterium; Mycobacterium tuberculosis complex; Mycobacterium tuberculosis;	NCBI: 1421967	4396347	99.94	0.00	Yes
NCBI Assembly	GCA_000669515.1	NCBI: cellular organisms; Bacteria; Terrabacteria group; Actinobacteria; Actinomycetia; Corynebacteriales; Mycobacteriaceae; Mycobacterium; Mycobacterium tuberculosis complex; Mycobacterium tuberculosis;	NCBI: 1417021	4368236	99.94	0.00	Yes
NCBI Assembly	GCA_000736275.1	NCBI: cellular organisms; Bacteria; Terrabacteria group; Actinobacteria; Actinomycetia; Corynebacteriales; Mycobacteriaceae; Mycobacterium; Mycobacterium tuberculosis complex; Mycobacterium tuberculosis;	NCBI: 1773	4539433	82.82	0.67	No
NCBI Assembly	GCA_000674135.1	NCBI: cellular organisms; Bacteria; Terrabacteria group; Actinobacteria; Actinomycetia; Corynebacteriales; Mycobacteriaceae; Mycobacterium; Mycobacterium tuberculosis complex; Mycobacterium tuberculosis;	NCBI: 1400915	4425094	99.94	0.33	Yes
NCBI Assembly	GCA_000672855.1	NCBI: cellular organisms; Bacteria; Terrabacteria group; Actinobacteria; Actinomycetia; Corynebacteriales; Mycobacteriaceae; Mycobacterium; Mycobacterium tuberculosis complex; Mycobacterium tuberculosis;	NCBI: 1397909	4433379	99.94	0.00	Yes
NCBI Assembly	GCA_000672255.1	NCBI: cellular organisms; Bacteria; Terrabacteria group; Actinobacteria; Actinomycetia; Corynebacteriales; Mycobacteriaceae; Mycobacterium; Mycobacterium tuberculosis complex; Mycobacterium tuberculosis;	NCBI: 1397878	4414437	99.94	0.00	Yes

NCBI Assembly	GCA_000673195.1	NCBI: cellular organisms; Bacteria; Terrabacteria group; Actinobacteria; Actinomycetia; Corynebacteriales; Mycobacteriaceae; Mycobacterium; Mycobacterium tuberculosis complex; Mycobacterium tuberculosis;	NCBI: 1397926	4406721	99.94	0.00	Yes
NCBI Assembly	GCA_000803965.1	NCBI: cellular organisms; Bacteria; Terrabacteria group; Actinobacteria; Actinomycetia; Corynebacteriales; Mycobacteriaceae; Mycobacterium; Mycobacterium tuberculosis complex; Mycobacterium tuberculosis;	NCBI: 1773	4364860	99.94	0.00	Yes
NCBI Assembly	GCA_000667265.1	NCBI: cellular organisms; Bacteria; Terrabacteria group; Actinobacteria; Actinomycetia; Corynebacteriales; Mycobacteriaceae; Mycobacterium; Mycobacterium tuberculosis complex; Mycobacterium tuberculosis;	NCBI: 1354134	4419940	99.94	0.00	Yes
NCBI Assembly	GCA_000665185.1	NCBI: cellular organisms; Bacteria; Terrabacteria group; Actinobacteria; Actinomycetia; Corynebacteriales; Mycobacteriaceae; Mycobacterium; Mycobacterium tuberculosis complex; Mycobacterium tuberculosis;	NCBI: 1448800	4420488	99.94	0.00	Yes
NCBI Assembly	GCA_000668275.1	NCBI: cellular organisms; Bacteria; Terrabacteria group; Actinobacteria; Actinomycetia; Corynebacteriales; Mycobacteriaceae; Mycobacterium; Mycobacterium tuberculosis complex; Mycobacterium tuberculosis;	NCBI: 1408919	4397587	99.94	0.00	Yes
NCBI Assembly	GCA_000669535.1	NCBI: cellular organisms; Bacteria; Terrabacteria group; Actinobacteria; Actinomycetia; Corynebacteriales; Mycobacteriaceae; Mycobacterium; Mycobacterium tuberculosis complex; Mycobacterium tuberculosis;	NCBI: 1417023	4386858	99.61	0.00	Yes
NCBI Assembly	GCA_000735975.1	NCBI: cellular organisms; Bacteria; Terrabacteria group; Actinobacteria; Actinomycetia; Corynebacteriales; Mycobacteriaceae; Mycobacterium; Mycobacterium tuberculosis complex; Mycobacterium tuberculosis;	NCBI: 1773	4539435	98.24	0.57	Yes
NCBI Assembly	GCA_000670855.1	NCBI: cellular organisms; Bacteria; Terrabacteria group; Actinobacteria; Actinomycetia; Corynebacteriales; Mycobacteriaceae; Mycobacterium; Mycobacterium tuberculosis complex; Mycobacterium tuberculosis;	NCBI: 1421969	4401917	99.94	0.00	Yes
NCBI Assembly	GCA_000672875.1	NCBI: cellular organisms; Bacteria; Terrabacteria group; Actinobacteria; Actinomycetia; Corynebacteriales; Mycobacteriaceae; Mycobacterium; Mycobacterium tuberculosis complex; Mycobacterium tuberculosis;	NCBI: 1397910	4411408	99.94	0.00	Yes
NCBI Assembly	GCA_000674155.1	NCBI: cellular organisms; Bacteria; Terrabacteria group; Actinobacteria; Actinomycetia; Corynebacteriales; Mycobacteriaceae; Mycobacterium; Mycobacterium tuberculosis complex; Mycobacterium tuberculosis;	NCBI: 1400916	4388859	99.94	0.00	Yes
NCBI Assembly	GCA_000737385.1	NCBI: cellular organisms; Bacteria; Terrabacteria group; Actinobacteria; Actinomycetia; Corynebacteriales; Mycobacteriaceae; Mycobacterium; Mycobacterium tuberculosis complex; Mycobacterium tuberculosis;	NCBI: 1773	4487096	99.94	0.08	Yes
NCBI Assembly	GCA_000672275.1	NCBI: cellular organisms; Bacteria; Terrabacteria group; Actinobacteria; Actinomycetia; Corynebacteriales; Mycobacteriaceae; Mycobacterium; Mycobacterium tuberculosis complex; Mycobacterium tuberculosis;	NCBI: 1397879	4412857	99.94	0.33	Yes
NCBI Assembly	GCA_000673215.1	NCBI: cellular organisms; Bacteria; Terrabacteria group; Actinobacteria; Actinomycetia; Corynebacteriales; Mycobacteriaceae; Mycobacterium; Mycobacterium tuberculosis complex; Mycobacterium tuberculosis;	NCBI: 1397927	4431491	99.94	0.00	Yes
NCBI Assembly	GCA_000669115.1	NCBI: cellular organisms; Bacteria; Terrabacteria group; Actinobacteria; Actinomycetia; Corynebacteriales; Mycobacteriaceae; Mycobacterium; Mycobacterium tuberculosis complex; Mycobacterium tuberculosis;	NCBI: 1408964	4398695	99.94	0.00	Yes
NCBI Assembly	GCA_000667285.1	NCBI: cellular organisms; Bacteria; Terrabacteria group; Actinobacteria; Actinomycetia; Corynebacteriales; Mycobacteriaceae; Mycobacterium; Mycobacterium tuberculosis complex; Mycobacterium tuberculosis;	NCBI: 1354135	4371542	99.94	0.00	Yes
NCBI Assembly	GCA_000665205.1	NCBI: cellular organisms; Bacteria; Terrabacteria group; Actinobacteria; Actinomycetia; Corynebacteriales; Mycobacteriaceae; Mycobacterium; Mycobacterium tuberculosis complex; Mycobacterium tuberculosis;	NCBI: 1448801	4419471	99.94	0.00	Yes
NCBI Assembly	GCA_000669555.1	NCBI: cellular organisms; Bacteria; Terrabacteria group; Actinobacteria; Actinomycetia; Corynebacteriales; Mycobacteriaceae; Mycobacterium; Mycobacterium tuberculosis complex; Mycobacterium tuberculosis;	NCBI: 1417027	4378398	99.94	0.00	Yes
NCBI Assembly	GCA_000668295.1	NCBI: cellular organisms; Bacteria; Terrabacteria group; Actinobacteria; Actinomycetia; Corynebacteriales; Mycobacteriaceae; Mycobacterium; Mycobacterium tuberculosis complex; Mycobacterium tuberculosis;	NCBI: 1408920	4408534	99.94	0.06	Yes

NCBI Assembly	GCA_000670875.1	NCBI: cellular organisms; Bacteria; Terrabacteria group; Actinobacteria; Actinomycetia; Corynebacteriales; Mycobacteriaceae; Mycobacterium; Mycobacterium tuberculosis complex; Mycobacterium tuberculosis;	NCBI: 1421971	4400159	99.94	0.00	Yes
NCBI Assembly	GCA_000672895.1	NCBI: cellular organisms; Bacteria; Terrabacteria group; Actinobacteria; Actinomycetia; Corynebacteriales; Mycobacteriaceae; Mycobacterium; Mycobacterium tuberculosis complex; Mycobacterium tuberculosis;	NCBI: 1397911	4417913	99.94	0.00	Yes
NCBI Assembly	GCA_000674175.1	NCBI: cellular organisms; Bacteria; Terrabacteria group; Actinobacteria; Actinomycetia; Corynebacteriales; Mycobacteriaceae; Mycobacterium; Mycobacterium tuberculosis complex; Mycobacterium tuberculosis;	NCBI: 1400918	4462619	99.94	0.67	Yes
NCBI Assembly	GCA_000735995.1	NCBI: cellular organisms; Bacteria; Terrabacteria group; Actinobacteria; Actinomycetia; Corynebacteriales; Mycobacteriaceae; Mycobacterium; Mycobacterium tuberculosis complex; Mycobacterium tuberculosis;	NCBI: 1773	4539435	79.00	0.50	No
NCBI Assembly	GCA_000672295.1	NCBI: cellular organisms; Bacteria; Terrabacteria group; Actinobacteria; Actinomycetia; Corynebacteriales; Mycobacteriaceae; Mycobacterium; Mycobacterium tuberculosis complex; Mycobacterium tuberculosis;	NCBI: 1397880	4429281	99.94	0.00	Yes
NCBI Assembly	GCA_000673235.1	NCBI: cellular organisms; Bacteria; Terrabacteria group; Actinobacteria; Actinomycetia; Corynebacteriales; Mycobacteriaceae; Mycobacterium; Mycobacterium tuberculosis complex; Mycobacterium tuberculosis;	NCBI: 1397928	4398038	99.94	0.00	Yes
NCBI Assembly	GCA_000738445.1	NCBI: cellular organisms; Bacteria; Terrabacteria group; Actinobacteria; Actinomycetia; Corynebacteriales; Mycobacteriaceae; Mycobacterium; Mycobacterium tuberculosis complex; Mycobacterium tuberculosis;	NCBI: 1773	4411507	99.94	0.00	Yes
NCBI Assembly	GCA_000669135.1	NCBI: cellular organisms; Bacteria; Terrabacteria group; Actinobacteria; Actinomycetia; Corynebacteriales; Mycobacteriaceae; Mycobacterium; Mycobacterium tuberculosis complex; Mycobacterium tuberculosis;	NCBI: 1408965	4439606	99.94	0.00	Yes
NCBI Assembly	GCA_000667305.1	NCBI: cellular organisms; Bacteria; Terrabacteria group; Actinobacteria; Actinomycetia; Corynebacteriales; Mycobacteriaceae; Mycobacterium; Mycobacterium tuberculosis complex; Mycobacterium tuberculosis;	NCBI: 1354136	4411964	99.94	0.00	Yes
NCBI Assembly	GCA_000665225.1	NCBI: cellular organisms; Bacteria; Terrabacteria group; Actinobacteria; Actinomycetia; Corynebacteriales; Mycobacteriaceae; Mycobacterium; Mycobacterium tuberculosis complex; Mycobacterium tuberculosis;	NCBI: 1448802	4418862	99.94	0.22	Yes
NCBI Assembly	GCA_000669575.1	NCBI: cellular organisms; Bacteria; Terrabacteria group; Actinobacteria; Actinomycetia; Corynebacteriales; Mycobacteriaceae; Mycobacterium; Mycobacterium tuberculosis complex; Mycobacterium tuberculosis;	NCBI: 1417029	4380149	99.94	0.00	Yes
NCBI Assembly	GCA_000668315.1	NCBI: cellular organisms; Bacteria; Terrabacteria group; Actinobacteria; Actinomycetia; Corynebacteriales; Mycobacteriaceae; Mycobacterium; Mycobacterium tuberculosis complex; Mycobacterium tuberculosis;	NCBI: 1408921	4434698	99.94	0.00	Yes
NCBI Assembly	GCA_000670895.1	NCBI: cellular organisms; Bacteria; Terrabacteria group; Actinobacteria; Actinomycetia; Corynebacteriales; Mycobacteriaceae; Mycobacterium; Mycobacterium tuberculosis complex; Mycobacterium tuberculosis;	NCBI: 1421972	4402532	99.94	0.00	Yes
NCBI Assembly	GCA_000672915.1	NCBI: cellular organisms; Bacteria; Terrabacteria group; Actinobacteria; Actinomycetia; Corynebacteriales; Mycobacteriaceae; Mycobacterium; Mycobacterium tuberculosis complex; Mycobacterium tuberculosis;	NCBI: 1397912	4413211	99.94	0.00	Yes
NCBI Assembly	GCA_000674195.1	NCBI: cellular organisms; Bacteria; Terrabacteria group; Actinobacteria; Actinomycetia; Corynebacteriales; Mycobacteriaceae; Mycobacterium; Mycobacterium tuberculosis complex; Mycobacterium tuberculosis;	NCBI: 1400920	4407766	99.61	0.00	Yes
NCBI Assembly	GCA_000673255.1	NCBI: cellular organisms; Bacteria; Terrabacteria group; Actinobacteria; Actinomycetia; Corynebacteriales; Mycobacteriaceae; Mycobacterium; Mycobacterium tuberculosis complex; Mycobacterium tuberculosis;	NCBI: 1397929	4420966	99.94	0.00	Yes
NCBI Assembly	GCA_000736015.1	NCBI: cellular organisms; Bacteria; Terrabacteria group; Actinobacteria; Actinomycetia; Corynebacteriales; Mycobacteriaceae; Mycobacterium; Mycobacterium tuberculosis complex; Mycobacterium tuberculosis;	NCBI: 1773	4539435	98.14	0.50	Yes
NCBI Assembly	GCA_000672315.1	NCBI: cellular organisms; Bacteria; Terrabacteria group; Actinobacteria; Actinomycetia; Corynebacteriales; Mycobacteriaceae; Mycobacterium; Mycobacterium tuberculosis complex; Mycobacterium tuberculosis;	NCBI: 1397881	4422192	99.94	0.33	Yes

NCBI Assembly	GCA_000738475.1	NCBI: cellular organisms; Bacteria; Terrabacteria group; Actinobacteria; Actinomycetia; Corynebacteriales; Mycobacteriaceae; Mycobacterium; Mycobacterium tuberculosis complex; Mycobacterium tuberculosis;	NCBI: 1773	4411515	99.94	0.00	Yes
NCBI Assembly	GCA_000669155.1	NCBI: cellular organisms; Bacteria; Terrabacteria group; Actinobacteria; Actinomycetia; Corynebacteriales; Mycobacteriaceae; Mycobacterium; Mycobacterium tuberculosis complex; Mycobacterium tuberculosis;	NCBI: 1408967	4422693	99.94	0.00	Yes
NCBI Assembly	GCA_000667325.1	NCBI: cellular organisms; Bacteria; Terrabacteria group; Actinobacteria; Actinomycetia; Corynebacteriales; Mycobacteriaceae; Mycobacterium; Mycobacterium tuberculosis complex; Mycobacterium tuberculosis; Mycobacterium tuberculosis variant africanum;	NCBI: 1354191	4383982	99.94	0.00	Yes
NCBI Assembly	GCA_000665245.1	NCBI: cellular organisms; Bacteria; Terrabacteria group; Actinobacteria; Actinomycetia; Corynebacteriales; Mycobacteriaceae; Mycobacterium; Mycobacterium tuberculosis complex; Mycobacterium tuberculosis;	NCBI: 1448803	4402729	99.94	0.44	Yes
NCBI Assembly	GCA_000669595.1	NCBI: cellular organisms; Bacteria; Terrabacteria group; Actinobacteria; Actinomycetia; Corynebacteriales; Mycobacteriaceae; Mycobacterium; Mycobacterium tuberculosis complex; Mycobacterium tuberculosis;	NCBI: 1417030	4380469	99.94	0.00	Yes
NCBI Assembly	GCA_000670915.1	NCBI: cellular organisms; Bacteria; Terrabacteria group; Actinobacteria; Actinomycetia; Corynebacteriales; Mycobacteriaceae; Mycobacterium; Mycobacterium tuberculosis complex; Mycobacterium tuberculosis;	NCBI: 1421973	4514078	99.94	2.89	Yes
NCBI Assembly	GCA_000668335.1	NCBI: cellular organisms; Bacteria; Terrabacteria group; Actinobacteria; Actinomycetia; Corynebacteriales; Mycobacteriaceae; Mycobacterium; Mycobacterium tuberculosis complex; Mycobacterium tuberculosis;	NCBI: 1408922	4409765	99.94	0.00	Yes
NCBI Assembly	GCA_000672935.1	NCBI: cellular organisms; Bacteria; Terrabacteria group; Actinobacteria; Actinomycetia; Corynebacteriales; Mycobacteriaceae; Mycobacterium; Mycobacterium tuberculosis complex; Mycobacterium tuberculosis;	NCBI: 1397913	4405382	99.94	0.00	Yes
NCBI Assembly	GCA_000674215.1	NCBI: cellular organisms; Bacteria; Terrabacteria group; Actinobacteria; Actinomycetia; Corynebacteriales; Mycobacteriaceae; Mycobacterium; Mycobacterium tuberculosis complex; Mycobacterium tuberculosis;	NCBI: 1400921	4398157	99.94	0.00	Yes
NCBI Assembly	GCA_000673275.1	NCBI: cellular organisms; Bacteria; Terrabacteria group; Actinobacteria; Actinomycetia; Corynebacteriales; Mycobacteriaceae; Mycobacterium; Mycobacterium tuberculosis complex; Mycobacterium tuberculosis;	NCBI: 1397930	4401715	99.94	0.00	Yes
NCBI Assembly	GCA_000672335.1	NCBI: cellular organisms; Bacteria; Terrabacteria group; Actinobacteria; Actinomycetia; Corynebacteriales; Mycobacteriaceae; Mycobacterium; Mycobacterium tuberculosis complex; Mycobacterium tuberculosis;	NCBI: 1397882	4411923	99.94	0.00	Yes
NCBI Assembly	GCA_000736035.1	NCBI: cellular organisms; Bacteria; Terrabacteria group; Actinobacteria; Actinomycetia; Corynebacteriales; Mycobacteriaceae; Mycobacterium; Mycobacterium tuberculosis complex; Mycobacterium tuberculosis;	NCBI: 1773	4539433	75.42	0.57	No
NCBI Assembly	GCA_000743595.1	NCBI: cellular organisms; Bacteria; Terrabacteria group; Actinobacteria; Actinomycetia; Corynebacteriales; Mycobacteriaceae; Mycobacterium; Mycobacterium tuberculosis complex; Mycobacterium tuberculosis;	NCBI: 1773	4329965	99.94	0.00	Yes
NCBI Assembly	GCA_000669175.1	NCBI: cellular organisms; Bacteria; Terrabacteria group; Actinobacteria; Actinomycetia; Corynebacteriales; Mycobacteriaceae; Mycobacterium; Mycobacterium tuberculosis complex; Mycobacterium tuberculosis;	NCBI: 1408968	4417436	99.94	0.00	Yes
NCBI Assembly	GCA_000667345.1	NCBI: cellular organisms; Bacteria; Terrabacteria group; Actinobacteria; Actinomycetia; Corynebacteriales; Mycobacteriaceae; Mycobacterium; Mycobacterium tuberculosis complex; Mycobacterium tuberculosis;	NCBI: 1354142	4426237	99.94	0.00	Yes
NCBI Assembly	GCA_000665265.1	NCBI: cellular organisms; Bacteria; Terrabacteria group; Actinobacteria; Actinomycetia; Corynebacteriales; Mycobacteriaceae; Mycobacterium; Mycobacterium tuberculosis complex; Mycobacterium tuberculosis;	NCBI: 1448804	4377197	99.94	0.00	Yes
NCBI Assembly	GCA_000670935.1	NCBI: cellular organisms; Bacteria; Terrabacteria group; Actinobacteria; Actinomycetia; Corynebacteriales; Mycobacteriaceae; Mycobacterium; Mycobacterium tuberculosis complex; Mycobacterium tuberculosis;	NCBI: 1421974	4478118	99.94	2.22	Yes
NCBI Assembly	GCA_000669615.1	NCBI: cellular organisms; Bacteria; Terrabacteria group; Actinobacteria; Actinomycetia; Corynebacteriales; Mycobacteriaceae; Mycobacterium; Mycobacterium tuberculosis complex; Mycobacterium tuberculosis;	NCBI: 1417031	4391143	99.94	0.00	Yes

NCBI Assembly	GCA_000668355.1	NCBI: cellular organisms; Bacteria; Terrabacteria group; Actinobacteria; Actinomycetia; Corynebacteriales; Mycobacteriaceae; Mycobacterium; Mycobacterium tuberculosis complex; Mycobacterium tuberculosis;	NCBI: 1408923	4437509	99.61	0.00	Yes
NCBI Assembly	GCA_000672955.1	NCBI: cellular organisms; Bacteria; Terrabacteria group; Actinobacteria; Actinomycetia; Corynebacteriales; Mycobacteriaceae; Mycobacterium; Mycobacterium tuberculosis complex; Mycobacterium tuberculosis;	NCBI: 1397914	4403216	99.94	0.00	Yes
NCBI Assembly	GCA_000672355.1	NCBI: cellular organisms; Bacteria; Terrabacteria group; Actinobacteria; Actinomycetia; Corynebacteriales; Mycobacteriaceae; Mycobacterium; Mycobacterium tuberculosis complex; Mycobacterium tuberculosis;	NCBI: 1397883	4425160	99.94	0.45	Yes
NCBI Assembly	GCA_000673295.1	NCBI: cellular organisms; Bacteria; Terrabacteria group; Actinobacteria; Actinomycetia; Corynebacteriales; Mycobacteriaceae; Mycobacterium; Mycobacterium tuberculosis complex; Mycobacterium tuberculosis;	NCBI: 1397931	4398866	99.94	0.00	Yes
NCBI Assembly	GCA_000674235.1	NCBI: cellular organisms; Bacteria; Terrabacteria group; Actinobacteria; Actinomycetia; Corynebacteriales; Mycobacteriaceae; Mycobacterium; Mycobacterium tuberculosis complex; Mycobacterium tuberculosis;	NCBI: 1400923	4392600	99.94	0.00	Yes
NCBI Assembly	GCA_000736055.1	NCBI: cellular organisms; Bacteria; Terrabacteria group; Actinobacteria; Actinomycetia; Corynebacteriales; Mycobacteriaceae; Mycobacterium; Mycobacterium tuberculosis complex; Mycobacterium tuberculosis;	NCBI: 1773	4539433	94.39	0.50	Yes
NCBI Assembly	GCA_000744355.1	NCBI: cellular organisms; Bacteria; Terrabacteria group; Actinobacteria; Actinomycetia; Corynebacteriales; Mycobacteriaceae; Mycobacterium; unclassified Mycobacterium;	NCBI: 1449060	5561884	100.00	1.29	Yes
NCBI Assembly	GCA_000669195.1	NCBI: cellular organisms; Bacteria; Terrabacteria group; Actinobacteria; Actinomycetia; Corynebacteriales; Mycobacteriaceae; Mycobacterium; Mycobacterium tuberculosis complex; Mycobacterium tuberculosis;	NCBI: 1408969	4407773	99.94	0.00	Yes
NCBI Assembly	GCA_000665285.1	NCBI: cellular organisms; Bacteria; Terrabacteria group; Actinobacteria; Actinomycetia; Corynebacteriales; Mycobacteriaceae; Mycobacterium; Mycobacterium tuberculosis complex; Mycobacterium tuberculosis;	NCBI: 1448805	4422382	99.94	0.00	Yes
NCBI Assembly	GCA_000667365.1	NCBI: cellular organisms; Bacteria; Terrabacteria group; Actinobacteria; Actinomycetia; Corynebacteriales; Mycobacteriaceae; Mycobacterium; Mycobacterium tuberculosis complex; Mycobacterium tuberculosis; Mycobacterium tuberculosis variant africanum;	NCBI: 1354188	4385261	99.94	0.00	Yes
NCBI Assembly	GCA_000670955.1	NCBI: cellular organisms; Bacteria; Terrabacteria group; Actinobacteria; Actinomycetia; Corynebacteriales; Mycobacteriaceae; Mycobacterium; Mycobacterium tuberculosis complex; Mycobacterium tuberculosis;	NCBI: 1421975	4422732	99.94	0.00	Yes
NCBI Assembly	GCA_000669635.1	NCBI: cellular organisms; Bacteria; Terrabacteria group; Actinobacteria; Actinomycetia; Corynebacteriales; Mycobacteriaceae; Mycobacterium; Mycobacterium tuberculosis complex; Mycobacterium tuberculosis;	NCBI: 1417005	4403368	99.94	0.00	Yes
NCBI Assembly	GCA_000672375.1	NCBI: cellular organisms; Bacteria; Terrabacteria group; Actinobacteria; Actinomycetia; Corynebacteriales; Mycobacteriaceae; Mycobacterium; Mycobacterium tuberculosis complex; Mycobacterium tuberculosis;	NCBI: 1397884	4403362	99.94	0.00	Yes
NCBI Assembly	GCA_000668375.1	NCBI: cellular organisms; Bacteria; Terrabacteria group; Actinobacteria; Actinomycetia; Corynebacteriales; Mycobacteriaceae; Mycobacterium; Mycobacterium tuberculosis complex; Mycobacterium tuberculosis;	NCBI: 1408924	4405406	99.94	0.00	Yes
NCBI Assembly	GCA_000674255.1	NCBI: cellular organisms; Bacteria; Terrabacteria group; Actinobacteria; Actinomycetia; Corynebacteriales; Mycobacteriaceae; Mycobacterium; Mycobacterium tuberculosis complex; Mycobacterium tuberculosis;	NCBI: 1400924	4431153	99.61	0.00	Yes
NCBI Assembly	GCA_000672975.1	NCBI: cellular organisms; Bacteria; Terrabacteria group; Actinobacteria; Actinomycetia; Corynebacteriales; Mycobacteriaceae; Mycobacterium; Mycobacterium tuberculosis complex; Mycobacterium tuberculosis;	NCBI: 1397915	4392740	99.94	0.00	Yes
NCBI Assembly	GCA_000673315.1	NCBI: cellular organisms; Bacteria; Terrabacteria group; Actinobacteria; Actinomycetia; Corynebacteriales; Mycobacteriaceae; Mycobacterium; Mycobacterium tuberculosis complex; Mycobacterium tuberculosis;	NCBI: 1397932	4415985	99.94	0.00	Yes
NCBI Assembly	GCA_000669215.1	NCBI: cellular organisms; Bacteria; Terrabacteria group; Actinobacteria; Actinomycetia; Corynebacteriales; Mycobacteriaceae; Mycobacterium; Mycobacterium tuberculosis complex; Mycobacterium tuberculosis;	NCBI: 1408970	4517933	99.94	0.00	Yes

NCBI Assembly	GCA_000746215.1	NCBI: cellular organisms; Bacteria; Terrabacteria group; Actinobacteria; Actinomycetia; Corynebacteriales; Mycobacteriaceae; Mycobacterium; unclassified Mycobacterium;	NCBI: 1449048	5696868	99.62	0.49	Yes
NCBI Assembly	GCA_000667385.1	NCBI: cellular organisms; Bacteria; Terrabacteria group; Actinobacteria; Actinomycetia; Corynebacteriales; Mycobacteriaceae; Mycobacterium; Mycobacterium tuberculosis complex; Mycobacterium tuberculosis;	NCBI: 1354119	4423419	99.83	0.00	Yes
NCBI Assembly	GCA_000736075.1	NCBI: cellular organisms; Bacteria; Terrabacteria group; Actinobacteria; Actinomycetia; Corynebacteriales; Mycobacteriaceae; Mycobacterium; Mycobacterium tuberculosis complex; Mycobacterium tuberculosis;	NCBI: 1773	4539435	93.26	0.57	Yes
NCBI Assembly	GCA_000665305.1	NCBI: cellular organisms; Bacteria; Terrabacteria group; Actinobacteria; Actinomycetia; Corynebacteriales; Mycobacteriaceae; Mycobacterium; Mycobacterium tuberculosis complex; Mycobacterium tuberculosis;	NCBI: 1448806	4422221	99.94	0.00	Yes
NCBI Assembly	GCA_000669695.1	NCBI: cellular organisms; Bacteria; Terrabacteria group; Actinobacteria; Actinomycetia; Corynebacteriales; Mycobacteriaceae; Mycobacterium; Mycobacterium tuberculosis complex; Mycobacterium tuberculosis;	NCBI: 1418250	4413939	99.94	0.00	Yes
NCBI Assembly	GCA_000670975.1	NCBI: cellular organisms; Bacteria; Terrabacteria group; Actinobacteria; Actinomycetia; Corynebacteriales; Mycobacteriaceae; Mycobacterium; Mycobacterium tuberculosis complex; Mycobacterium tuberculosis;	NCBI: 1421976	4416915	99.94	0.00	Yes
NCBI Assembly	GCA_000672995.1	NCBI: cellular organisms; Bacteria; Terrabacteria group; Actinobacteria; Actinomycetia; Corynebacteriales; Mycobacteriaceae; Mycobacterium; Mycobacterium tuberculosis complex; Mycobacterium tuberculosis;	NCBI: 1397916	4416959	99.94	0.00	Yes
NCBI Assembly	GCA_000672395.1	NCBI: cellular organisms; Bacteria; Terrabacteria group; Actinobacteria; Actinomycetia; Corynebacteriales; Mycobacteriaceae; Mycobacterium; Mycobacterium tuberculosis complex; Mycobacterium tuberculosis;	NCBI: 1397885	4397993	99.94	0.00	Yes
NCBI Assembly	GCA_000668395.1	NCBI: cellular organisms; Bacteria; Terrabacteria group; Actinobacteria; Actinomycetia; Corynebacteriales; Mycobacteriaceae; Mycobacterium; Mycobacterium tuberculosis complex; Mycobacterium tuberculosis;	NCBI: 1408925	4472660	99.94	0.00	Yes
NCBI Assembly	GCA_000674275.1	NCBI: cellular organisms; Bacteria; Terrabacteria group; Actinobacteria; Actinomycetia; Corynebacteriales; Mycobacteriaceae; Mycobacterium; Mycobacterium tuberculosis complex; Mycobacterium tuberculosis;	NCBI: 1400925	4416636	99.94	0.00	Yes
NCBI Assembly	GCA_000673335.1	NCBI: cellular organisms; Bacteria; Terrabacteria group; Actinobacteria; Actinomycetia; Corynebacteriales; Mycobacteriaceae; Mycobacterium; Mycobacterium tuberculosis complex; Mycobacterium tuberculosis;	NCBI: 1397933	4414539	99.94	0.00	Yes
NCBI Assembly	GCA_000746805.1	NCBI: cellular organisms; Bacteria; Terrabacteria group; Actinobacteria; Actinomycetia; Corynebacteriales; Mycobacteriaceae; Mycobacterium; Mycobacterium tuberculosis complex; Mycobacterium tuberculosis; Mycobacterium tuberculosis H37Rv;	NCBI: 757417	4410946	99.94	0.00	Yes
NCBI Assembly	GCA_000669235.1	NCBI: cellular organisms; Bacteria; Terrabacteria group; Actinobacteria; Actinomycetia; Corynebacteriales; Mycobacteriaceae; Mycobacterium; Mycobacterium tuberculosis complex; Mycobacterium tuberculosis;	NCBI: 1408971	4417057	99.94	0.00	Yes
NCBI Assembly	GCA_000665325.1	NCBI: cellular organisms; Bacteria; Terrabacteria group; Actinobacteria; Actinomycetia; Corynebacteriales; Mycobacteriaceae; Mycobacterium; Mycobacterium tuberculosis complex; Mycobacterium tuberculosis;	NCBI: 1448807	4424023	99.94	0.00	Yes
NCBI Assembly	GCA_000667405.1	NCBI: cellular organisms; Bacteria; Terrabacteria group; Actinobacteria; Actinomycetia; Corynebacteriales; Mycobacteriaceae; Mycobacterium; Mycobacterium tuberculosis complex; Mycobacterium tuberculosis;	NCBI: 1354120	4429148	99.94	0.00	Yes
NCBI Assembly	GCA_000669735.1	NCBI: cellular organisms; Bacteria; Terrabacteria group; Actinobacteria; Actinomycetia; Corynebacteriales; Mycobacteriaceae; Mycobacterium; Mycobacterium tuberculosis complex; Mycobacterium tuberculosis;	NCBI: 1418251	4399510	99.94	0.50	Yes
NCBI Assembly	GCA_000670995.1	NCBI: cellular organisms; Bacteria; Terrabacteria group; Actinobacteria; Actinomycetia; Corynebacteriales; Mycobacteriaceae; Mycobacterium; Mycobacterium tuberculosis complex; Mycobacterium tuberculosis;	NCBI: 1421977	4427938	99.94	0.00	Yes
NCBI Assembly	GCA_000736095.1	NCBI: cellular organisms; Bacteria; Terrabacteria group; Actinobacteria; Actinomycetia; Corynebacteriales; Mycobacteriaceae; Mycobacterium; Mycobacterium tuberculosis complex; Mycobacterium tuberculosis;	NCBI: 1773	4539435	93.34	0.50	Yes

NCBI Assembly	GCA_000672415.1	NCBI: cellular organisms; Bacteria; Terrabacteria group; Actinobacteria; Actinomycetia; Corynebacteriales; Mycobacteriaceae; Mycobacterium; Mycobacterium tuberculosis complex; Mycobacterium tuberculosis;	NCBI: 1397886	4429784	99.94	0.00	Yes
NCBI Assembly	GCA_000668415.1	NCBI: cellular organisms; Bacteria; Terrabacteria group; Actinobacteria; Actinomycetia; Corynebacteriales; Mycobacteriaceae; Mycobacterium; Mycobacterium tuberculosis complex; Mycobacterium tuberculosis;	NCBI: 1408926	4373294	99.94	0.00	Yes
NCBI Assembly	GCA_000674295.1	NCBI: cellular organisms; Bacteria; Terrabacteria group; Actinobacteria; Actinomycetia; Corynebacteriales; Mycobacteriaceae; Mycobacterium; Mycobacterium tuberculosis complex; Mycobacterium tuberculosis;	NCBI: 1400926	4409569	99.94	0.00	Yes
NCBI Assembly	GCA_000673015.1	NCBI: cellular organisms; Bacteria; Terrabacteria group; Actinobacteria; Actinomycetia; Corynebacteriales; Mycobacteriaceae; Mycobacterium; Mycobacterium tuberculosis complex; Mycobacterium tuberculosis;	NCBI: 1397917	4399600	99.94	0.00	Yes
NCBI Assembly	GCA_000673355.1	NCBI: cellular organisms; Bacteria; Terrabacteria group; Actinobacteria; Actinomycetia; Corynebacteriales; Mycobacteriaceae; Mycobacterium; Mycobacterium tuberculosis complex; Mycobacterium tuberculosis;	NCBI: 1397934	4413552	99.94	0.00	Yes
NCBI Assembly	GCA_000669255.1	NCBI: cellular organisms; Bacteria; Terrabacteria group; Actinobacteria; Actinomycetia; Corynebacteriales; Mycobacteriaceae; Mycobacterium; Mycobacterium tuberculosis complex; Mycobacterium tuberculosis;	NCBI: 1408973	4358547	99.94	0.00	Yes
NCBI Assembly	GCA_000667425.1	NCBI: cellular organisms; Bacteria; Terrabacteria group; Actinobacteria; Actinomycetia; Corynebacteriales; Mycobacteriaceae; Mycobacterium; Mycobacterium tuberculosis complex; Mycobacterium tuberculosis;	NCBI: 1354121	4412513	99.94	0.00	Yes
NCBI Assembly	GCA_000665345.1	NCBI: cellular organisms; Bacteria; Terrabacteria group; Actinobacteria; Actinomycetia; Corynebacteriales; Mycobacteriaceae; Mycobacterium; Mycobacterium tuberculosis complex; Mycobacterium tuberculosis;	NCBI: 1448808	4388381	99.94	0.00	Yes
NCBI Assembly	GCA_000749605.1	NCBI: cellular organisms; Bacteria; Terrabacteria group; Actinobacteria; Actinomycetia; Corynebacteriales; Mycobacteriaceae; Mycobacterium; Mycobacterium tuberculosis complex; Mycobacterium tuberculosis; Mycobacterium tuberculosis variant bovis;	NCBI: 1765	4333756	99.78	0.00	Yes
NCBI Assembly	GCA_000669755.1	NCBI: cellular organisms; Bacteria; Terrabacteria group; Actinobacteria; Actinomycetia; Corynebacteriales; Mycobacteriaceae; Mycobacterium; Mycobacterium tuberculosis complex; Mycobacterium tuberculosis;	NCBI: 1418252	4389482	99.94	0.00	Yes
NCBI Assembly	GCA_000671015.1	NCBI: cellular organisms; Bacteria; Terrabacteria group; Actinobacteria; Actinomycetia; Corynebacteriales; Mycobacteriaceae; Mycobacterium; Mycobacterium tuberculosis complex; Mycobacterium tuberculosis;	NCBI: 1421978	4420105	99.94	0.00	Yes
NCBI Assembly	GCA_000672435.1	NCBI: cellular organisms; Bacteria; Terrabacteria group; Actinobacteria; Actinomycetia; Corynebacteriales; Mycobacteriaceae; Mycobacterium; Mycobacterium tuberculosis complex; Mycobacterium tuberculosis;	NCBI: 1397887	4411620	99.94	0.00	Yes
NCBI Assembly	GCA_000668435.1	NCBI: cellular organisms; Bacteria; Terrabacteria group; Actinobacteria; Actinomycetia; Corynebacteriales; Mycobacteriaceae; Mycobacterium; Mycobacterium tuberculosis complex; Mycobacterium tuberculosis;	NCBI: 1408928	4343421	98.93	0.07	Yes
NCBI Assembly	GCA_000736115.1	NCBI: cellular organisms; Bacteria; Terrabacteria group; Actinobacteria; Actinomycetia; Corynebacteriales; Mycobacteriaceae; Mycobacterium; Mycobacterium tuberculosis complex; Mycobacterium tuberculosis;	NCBI: 1773	4539435	96.86	0.57	Yes
NCBI Assembly	GCA_000673035.1	NCBI: cellular organisms; Bacteria; Terrabacteria group; Actinobacteria; Actinomycetia; Corynebacteriales; Mycobacteriaceae; Mycobacterium; Mycobacterium tuberculosis complex; Mycobacterium tuberculosis;	NCBI: 1397918	4398852	99.94	0.00	Yes
NCBI Assembly	GCA_000674315.1	NCBI: cellular organisms; Bacteria; Terrabacteria group; Actinobacteria; Actinomycetia; Corynebacteriales; Mycobacteriaceae; Mycobacterium; Mycobacterium tuberculosis complex; Mycobacterium tuberculosis;	NCBI: 1400927	4419358	99.94	0.00	Yes
NCBI Assembly	GCA_000673375.1	NCBI: cellular organisms; Bacteria; Terrabacteria group; Actinobacteria; Actinomycetia; Corynebacteriales; Mycobacteriaceae; Mycobacterium; Mycobacterium tuberculosis complex; Mycobacterium tuberculosis;	NCBI: 1397935	4399861	99.94	0.00	Yes
NCBI Assembly	GCA_000669275.1	NCBI: cellular organisms; Bacteria; Terrabacteria group; Actinobacteria; Actinomycetia; Corynebacteriales; Mycobacteriaceae; Mycobacterium; Mycobacterium tuberculosis complex; Mycobacterium tuberculosis;	NCBI: 1408974	4436430	98.94	0.00	Yes

NCBI Assembly	GCA_000665365.1	NCBI: cellular organisms; Bacteria; Terrabacteria group; Actinobacteria; Actinomycetia; Corynebacteriales; Mycobacteriaceae; Mycobacterium; Mycobacterium tuberculosis complex; Mycobacterium tuberculosis;	NCBI: 1448810	4406894	99.94	0.03	Yes
NCBI Assembly	GCA_000667445.1	NCBI: cellular organisms; Bacteria; Terrabacteria group; Actinobacteria; Actinomycetia; Corynebacteriales; Mycobacteriaceae; Mycobacterium; Mycobacterium tuberculosis complex; Mycobacterium tuberculosis; Mycobacterium tuberculosis variant africanum;	NCBI: 1354189	4395062	99.94	0.00	Yes
NCBI Assembly	GCA_000669775.1	NCBI: cellular organisms; Bacteria; Terrabacteria group; Actinobacteria; Actinomycetia; Corynebacteriales; Mycobacteriaceae; Mycobacterium; Mycobacterium tuberculosis complex; Mycobacterium tuberculosis;	NCBI: 1418253	4392604	99.94	0.05	Yes
NCBI Assembly	GCA_000672455.1	NCBI: cellular organisms; Bacteria; Terrabacteria group; Actinobacteria; Actinomycetia; Corynebacteriales; Mycobacteriaceae; Mycobacterium; Mycobacterium tuberculosis complex; Mycobacterium tuberculosis;	NCBI: 1397888	4422857	99.94	0.00	Yes
NCBI Assembly	GCA_000671035.1	NCBI: cellular organisms; Bacteria; Terrabacteria group; Actinobacteria; Actinomycetia; Corynebacteriales; Mycobacteriaceae; Mycobacterium; Mycobacterium tuberculosis complex; Mycobacterium tuberculosis;	NCBI: 1421979	4405601	99.94	0.00	Yes
NCBI Assembly	GCA_000668455.1	NCBI: cellular organisms; Bacteria; Terrabacteria group; Actinobacteria; Actinomycetia; Corynebacteriales; Mycobacteriaceae; Mycobacterium; Mycobacterium tuberculosis complex; Mycobacterium tuberculosis;	NCBI: 1408929	4431287	99.94	0.00	Yes
NCBI Assembly	GCA_000749615.1	NCBI: cellular organisms; Bacteria; Terrabacteria group; Actinobacteria; Actinomycetia; Corynebacteriales; Mycobacteriaceae; Mycobacterium; Mycobacterium tuberculosis complex; Mycobacterium tuberculosis; Mycobacterium tuberculosis variant bovis;	NCBI: 1765	4314192	99.78	0.00	Yes
NCBI Assembly	GCA_000673055.1	NCBI: cellular organisms; Bacteria; Terrabacteria group; Actinobacteria; Actinomycetia; Corynebacteriales; Mycobacteriaceae; Mycobacterium; Mycobacterium tuberculosis complex; Mycobacterium tuberculosis;	NCBI: 1397919	4396596	99.94	0.00	Yes
NCBI Assembly	GCA_000674335.1	NCBI: cellular organisms; Bacteria; Terrabacteria group; Actinobacteria; Actinomycetia; Corynebacteriales; Mycobacteriaceae; Mycobacterium; Mycobacterium tuberculosis complex; Mycobacterium tuberculosis;	NCBI: 1400928	4406912	99.94	0.00	Yes
NCBI Assembly	GCA_000669795.1	NCBI: cellular organisms; Bacteria; Terrabacteria group; Actinobacteria; Actinomycetia; Corynebacteriales; Mycobacteriaceae; Mycobacterium; Mycobacterium tuberculosis complex; Mycobacterium tuberculosis;	NCBI: 1418254	4395099	99.94	0.46	Yes
NCBI Assembly	GCA_000736135.1	NCBI: cellular organisms; Bacteria; Terrabacteria group; Actinobacteria; Actinomycetia; Corynebacteriales; Mycobacteriaceae; Mycobacterium; Mycobacterium tuberculosis complex; Mycobacterium tuberculosis;	NCBI: 1773	4539435	97.47	0.57	Yes
NCBI Assembly	GCA_000669295.1	NCBI: cellular organisms; Bacteria; Terrabacteria group; Actinobacteria; Actinomycetia; Corynebacteriales; Mycobacteriaceae; Mycobacterium; Mycobacterium tuberculosis complex; Mycobacterium tuberculosis;	NCBI: 1408975	4381449	99.94	0.33	Yes
NCBI Assembly	GCA_000665385.1	NCBI: cellular organisms; Bacteria; Terrabacteria group; Actinobacteria; Actinomycetia; Corynebacteriales; Mycobacteriaceae; Mycobacterium; Mycobacterium tuberculosis complex; Mycobacterium tuberculosis;	NCBI: 1448811	4406689	99.94	0.00	Yes
NCBI Assembly	GCA_000666525.1	NCBI: cellular organisms; Bacteria; Terrabacteria group; Actinobacteria; Actinomycetia; Corynebacteriales; Mycobacteriaceae; Mycobacterium; Mycobacterium tuberculosis complex; Mycobacterium tuberculosis;	NCBI: 1354151	4419361	99.94	0.00	Yes
NCBI Assembly	GCA_000671855.1	NCBI: cellular organisms; Bacteria; Terrabacteria group; Actinobacteria; Actinomycetia; Corynebacteriales; Mycobacteriaceae; Mycobacterium; Mycobacterium tuberculosis complex; Mycobacterium tuberculosis;	NCBI: 1397857	4406712	99.94	0.00	Yes
NCBI Assembly	GCA_000667765.1	NCBI: cellular organisms; Bacteria; Terrabacteria group; Actinobacteria; Actinomycetia; Corynebacteriales; Mycobacteriaceae; Mycobacterium; Mycobacterium tuberculosis complex; Mycobacterium tuberculosis;	NCBI: 1354117	4412810	99.94	0.00	Yes
NCBI Assembly	GCA_000664165.1	NCBI: cellular organisms; Bacteria; Terrabacteria group; Actinobacteria; Actinomycetia; Corynebacteriales; Mycobacteriaceae; Mycobacterium; Mycobacterium tuberculosis complex; Mycobacterium tuberculosis;	NCBI: 1448744	4409927	99.94	0.00	Yes
NCBI Assembly	GCA_000673715.1	NCBI: cellular organisms; Bacteria; Terrabacteria group; Actinobacteria; Actinomycetia; Corynebacteriales; Mycobacteriaceae; Mycobacterium; Mycobacterium tuberculosis complex; Mycobacterium tuberculosis;	NCBI: 1400882	4371151	99.61	0.00	Yes

NCBI Assembly	GCA_000673075.1	NCBI: cellular organisms; Bacteria; Terrabacteria group; Actinobacteria; Actinomycetia; Corynebacteriales; Mycobacteriaceae; Mycobacterium; Mycobacterium tuberculosis complex; Mycobacterium tuberculosis;	NCBI: 1397920	4405127	99.94	0.00	Yes
NCBI Assembly	GCA_000668475.1	NCBI: cellular organisms; Bacteria; Terrabacteria group; Actinobacteria; Actinomycetia; Corynebacteriales; Mycobacteriaceae; Mycobacterium; Mycobacterium tuberculosis complex; Mycobacterium tuberculosis;	NCBI: 1408930	4436765	99.94	0.00	Yes
NCBI Assembly	GCA_000669815.1	NCBI: cellular organisms; Bacteria; Terrabacteria group; Actinobacteria; Actinomycetia; Corynebacteriales; Mycobacteriaceae; Mycobacterium; Mycobacterium tuberculosis complex; Mycobacterium tuberculosis;	NCBI: 1418255	4388631	99.94	0.00	Yes
NCBI Assembly	GCA_000749625.1	NCBI: cellular organisms; Bacteria; Terrabacteria group; Actinobacteria; Actinomycetia; Corynebacteriales; Mycobacteriaceae; Mycobacterium; Mycobacterium tuberculosis complex; Mycobacterium tuberculosis; Mycobacterium tuberculosis variant bovis;	NCBI: 1765	4329318	99.78	0.00	Yes
NCBI Assembly	GCA_000736155.1	NCBI: cellular organisms; Bacteria; Terrabacteria group; Actinobacteria; Actinomycetia; Corynebacteriales; Mycobacteriaceae; Mycobacterium; Mycobacterium tuberculosis complex; Mycobacterium tuberculosis;	NCBI: 1773	4539435	97.59	0.57	Yes
NCBI Assembly	GCA_000669315.1	NCBI: cellular organisms; Bacteria; Terrabacteria group; Actinobacteria; Actinomycetia; Corynebacteriales; Mycobacteriaceae; Mycobacterium; Mycobacterium tuberculosis complex; Mycobacterium tuberculosis;	NCBI: 1417008	4382556	99.94	0.00	Yes
NCBI Assembly	GCA_000666545.1	NCBI: cellular organisms; Bacteria; Terrabacteria group; Actinobacteria; Actinomycetia; Corynebacteriales; Mycobacteriaceae; Mycobacterium; Mycobacterium tuberculosis complex; Mycobacterium tuberculosis;	NCBI: 1354152	4393967	99.94	0.00	Yes
NCBI Assembly	GCA_000664785.1	NCBI: cellular organisms; Bacteria; Terrabacteria group; Actinobacteria; Actinomycetia; Corynebacteriales; Mycobacteriaceae; Mycobacterium; Mycobacterium tuberculosis complex; Mycobacterium tuberculosis;	NCBI: 1448778	4423989	99.94	0.00	Yes
NCBI Assembly	GCA_000671875.1	NCBI: cellular organisms; Bacteria; Terrabacteria group; Actinobacteria; Actinomycetia; Corynebacteriales; Mycobacteriaceae; Mycobacterium; Mycobacterium tuberculosis complex; Mycobacterium tuberculosis;	NCBI: 1397858	4401897	99.94	0.00	Yes
NCBI Assembly	GCA_000667845.1	NCBI: cellular organisms; Bacteria; Terrabacteria group; Actinobacteria; Actinomycetia; Corynebacteriales; Mycobacteriaceae; Mycobacterium; Mycobacterium tuberculosis complex; Mycobacterium tuberculosis;	NCBI: 668349	4403876	99.94	0.00	Yes
NCBI Assembly	GCA_000664185.1	NCBI: cellular organisms; Bacteria; Terrabacteria group; Actinobacteria; Actinomycetia; Corynebacteriales; Mycobacteriaceae; Mycobacterium; Mycobacterium tuberculosis complex; Mycobacterium tuberculosis;	NCBI: 1448745	4430943	99.94	0.03	Yes
NCBI Assembly	GCA_000666225.1	NCBI: cellular organisms; Bacteria; Terrabacteria group; Actinobacteria; Actinomycetia; Corynebacteriales; Mycobacteriaceae; Mycobacterium; Mycobacterium tuberculosis complex; Mycobacterium tuberculosis; Mycobacterium tuberculosis variant africanum;	NCBI: 1354164	4400807	99.94	0.00	Yes
NCBI Assembly	GCA_000673735.1	NCBI: cellular organisms; Bacteria; Terrabacteria group; Actinobacteria; Actinomycetia; Corynebacteriales; Mycobacteriaceae; Mycobacterium; Mycobacterium tuberculosis complex; Mycobacterium tuberculosis;	NCBI: 1400883	4400957	99.94	0.00	Yes
NCBI Assembly	GCA_000668495.1	NCBI: cellular organisms; Bacteria; Terrabacteria group; Actinobacteria; Actinomycetia; Corynebacteriales; Mycobacteriaceae; Mycobacterium; Mycobacterium tuberculosis complex; Mycobacterium tuberculosis;	NCBI: 1408931	4408068	99.61	0.00	Yes
NCBI Assembly	GCA_000669835.1	NCBI: cellular organisms; Bacteria; Terrabacteria group; Actinobacteria; Actinomycetia; Corynebacteriales; Mycobacteriaceae; Mycobacterium; Mycobacterium tuberculosis complex; Mycobacterium tuberculosis;	NCBI: 1418256	4407169	99.94	0.11	Yes
NCBI Assembly	GCA_000663845.1	NCBI: cellular organisms; Bacteria; Terrabacteria group; Actinobacteria; Actinomycetia; Corynebacteriales; Mycobacteriaceae; Mycobacterium; Mycobacterium tuberculosis complex; Mycobacterium tuberculosis;	NCBI: 1448726	4392679	99.94	0.00	Yes
NCBI Assembly	GCA_000664465.1	NCBI: cellular organisms; Bacteria; Terrabacteria group; Actinobacteria; Actinomycetia; Corynebacteriales; Mycobacteriaceae; Mycobacterium; Mycobacterium tuberculosis complex; Mycobacterium tuberculosis;	NCBI: 1448760	4424742	99.94	0.00	Yes
NCBI Assembly	GCA_000669335.1	NCBI: cellular organisms; Bacteria; Terrabacteria group; Actinobacteria; Actinomycetia; Corynebacteriales; Mycobacteriaceae; Mycobacterium; Mycobacterium tuberculosis complex; Mycobacterium tuberculosis;	NCBI: 1417009	4399185	99.94	0.33	Yes

NCBI Assembly	GCA_000666565.1	NCBI: cellular organisms; Bacteria; Terrabacteria group; Actinobacteria; Actinomycetia; Corynebacteriales; Mycobacteriaceae; Mycobacterium; Mycobacterium tuberculosis complex; Mycobacterium tuberculosis;	NCBI: 1354153	4410333	99.94	0.00	Yes
NCBI Assembly	GCA_000664805.1	NCBI: cellular organisms; Bacteria; Terrabacteria group; Actinobacteria; Actinomycetia; Corynebacteriales; Mycobacteriaceae; Mycobacterium; Mycobacterium tuberculosis complex; Mycobacterium tuberculosis;	NCBI: 1448779	4424314	99.94	0.06	Yes
NCBI Assembly	GCA_000671895.1	NCBI: cellular organisms; Bacteria; Terrabacteria group; Actinobacteria; Actinomycetia; Corynebacteriales; Mycobacteriaceae; Mycobacterium; Mycobacterium tuberculosis complex; Mycobacterium tuberculosis;	NCBI: 1397859	4405034	99.94	0.00	Yes
NCBI Assembly	GCA_000664205.1	NCBI: cellular organisms; Bacteria; Terrabacteria group; Actinobacteria; Actinomycetia; Corynebacteriales; Mycobacteriaceae; Mycobacterium; Mycobacterium tuberculosis complex; Mycobacterium tuberculosis;	NCBI: 1448746	4420338	99.94	0.38	Yes
NCBI Assembly	GCA_000667865.1	NCBI: cellular organisms; Bacteria; Terrabacteria group; Actinobacteria; Actinomycetia; Corynebacteriales; Mycobacteriaceae; Mycobacterium; Mycobacterium tuberculosis complex; Mycobacterium tuberculosis;	NCBI: 1402583	4409890	99.94	0.00	Yes
NCBI Assembly	GCA_000666245.1	NCBI: cellular organisms; Bacteria; Terrabacteria group; Actinobacteria; Actinomycetia; Corynebacteriales; Mycobacteriaceae; Mycobacterium; Mycobacterium tuberculosis complex; Mycobacterium tuberculosis;	NCBI: 1354143	4423376	99.94	0.00	Yes
NCBI Assembly	GCA_000668515.1	NCBI: cellular organisms; Bacteria; Terrabacteria group; Actinobacteria; Actinomycetia; Corynebacteriales; Mycobacteriaceae; Mycobacterium; Mycobacterium tuberculosis complex; Mycobacterium tuberculosis;	NCBI: 1408932	4461646	99.94	1.67	Yes
NCBI Assembly	GCA_000673755.1	NCBI: cellular organisms; Bacteria; Terrabacteria group; Actinobacteria; Actinomycetia; Corynebacteriales; Mycobacteriaceae; Mycobacterium; Mycobacterium tuberculosis complex; Mycobacterium tuberculosis;	NCBI: 1400884	4419080	99.94	0.00	Yes
NCBI Assembly	GCA_000669855.1	NCBI: cellular organisms; Bacteria; Terrabacteria group; Actinobacteria; Actinomycetia; Corynebacteriales; Mycobacteriaceae; Mycobacterium; Mycobacterium tuberculosis complex; Mycobacterium tuberculosis;	NCBI: 1421917	4396239	99.94	0.00	Yes
NCBI Assembly	GCA_000663865.1	NCBI: cellular organisms; Bacteria; Terrabacteria group; Actinobacteria; Actinomycetia; Corynebacteriales; Mycobacteriaceae; Mycobacterium; Mycobacterium tuberculosis complex; Mycobacterium tuberculosis;	NCBI: 1448728	4426154	99.94	0.00	Yes
NCBI Assembly	GCA_000664485.1	NCBI: cellular organisms; Bacteria; Terrabacteria group; Actinobacteria; Actinomycetia; Corynebacteriales; Mycobacteriaceae; Mycobacterium; Mycobacterium tuberculosis complex; Mycobacterium tuberculosis;	NCBI: 1448761	4419861	99.94	0.00	Yes
NCBI Assembly	GCA_000669355.1	NCBI: cellular organisms; Bacteria; Terrabacteria group; Actinobacteria; Actinomycetia; Corynebacteriales; Mycobacteriaceae; Mycobacterium; Mycobacterium tuberculosis complex; Mycobacterium tuberculosis;	NCBI: 1417010	4394026	99.94	0.00	Yes
NCBI Assembly	GCA_000664825.1	NCBI: cellular organisms; Bacteria; Terrabacteria group; Actinobacteria; Actinomycetia; Corynebacteriales; Mycobacteriaceae; Mycobacterium; Mycobacterium tuberculosis complex; Mycobacterium tuberculosis;	NCBI: 1448780	4417676	99.94	0.08	Yes
NCBI Assembly	GCA_000666585.1	NCBI: cellular organisms; Bacteria; Terrabacteria group; Actinobacteria; Actinomycetia; Corynebacteriales; Mycobacteriaceae; Mycobacterium; Mycobacterium tuberculosis complex; Mycobacterium tuberculosis; Mycobacterium tuberculosis variant africanum;	NCBI: 1354174	4387410	99.94	0.00	Yes
NCBI Assembly	GCA_000664225.1	NCBI: cellular organisms; Bacteria; Terrabacteria group; Actinobacteria; Actinomycetia; Corynebacteriales; Mycobacteriaceae; Mycobacterium; Mycobacterium tuberculosis complex; Mycobacterium tuberculosis;	NCBI: 1448747	4422195	99.94	0.00	Yes
NCBI Assembly	GCA_000667885.1	NCBI: cellular organisms; Bacteria; Terrabacteria group; Actinobacteria; Actinomycetia; Corynebacteriales; Mycobacteriaceae; Mycobacterium; Mycobacterium tuberculosis complex; Mycobacterium tuberculosis;	NCBI: 1402584	4438776	99.94	0.00	Yes
NCBI Assembly	GCA_000671915.1	NCBI: cellular organisms; Bacteria; Terrabacteria group; Actinobacteria; Actinomycetia; Corynebacteriales; Mycobacteriaceae; Mycobacterium; Mycobacterium tuberculosis complex; Mycobacterium tuberculosis;	NCBI: 1397860	4404880	99.94	0.00	Yes
NCBI Assembly	GCA_000668535.1	NCBI: cellular organisms; Bacteria; Terrabacteria group; Actinobacteria; Actinomycetia; Corynebacteriales; Mycobacteriaceae; Mycobacterium; Mycobacterium tuberculosis complex; Mycobacterium tuberculosis;	NCBI: 1408933	4370930	99.94	0.00	Yes

NCBI Assembly	GCA_000666265.1	NCBI: cellular organisms; Bacteria; Terrabacteria group; Actinobacteria; Actinomycetia; Corynebacteriales; Mycobacteriaceae; Mycobacterium; Mycobacterium tuberculosis complex; Mycobacterium tuberculosis;	NCBI: 1354165	4419918	99.94	0.00	Yes
NCBI Assembly	GCA_000669875.1	NCBI: cellular organisms; Bacteria; Terrabacteria group; Actinobacteria; Actinomycetia; Corynebacteriales; Mycobacteriaceae; Mycobacterium; Mycobacterium tuberculosis complex; Mycobacterium tuberculosis;	NCBI: 1421918	4410914	99.94	0.00	Yes
NCBI Assembly	GCA_000663885.1	NCBI: cellular organisms; Bacteria; Terrabacteria group; Actinobacteria; Actinomycetia; Corynebacteriales; Mycobacteriaceae; Mycobacterium; Mycobacterium tuberculosis complex; Mycobacterium tuberculosis;	NCBI: 1448729	4419058	99.94	0.00	Yes
NCBI Assembly	GCA_000673775.1	NCBI: cellular organisms; Bacteria; Terrabacteria group; Actinobacteria; Actinomycetia; Corynebacteriales; Mycobacteriaceae; Mycobacterium; Mycobacterium tuberculosis complex; Mycobacterium tuberculosis;	NCBI: 1400886	4523586	99.11	0.00	Yes
NCBI Assembly	GCA_000664505.1	NCBI: cellular organisms; Bacteria; Terrabacteria group; Actinobacteria; Actinomycetia; Corynebacteriales; Mycobacteriaceae; Mycobacterium; Mycobacterium tuberculosis complex; Mycobacterium tuberculosis;	NCBI: 1448762	4412892	99.94	0.00	Yes
NCBI Assembly	GCA_000669375.1	NCBI: cellular organisms; Bacteria; Terrabacteria group; Actinobacteria; Actinomycetia; Corynebacteriales; Mycobacteriaceae; Mycobacterium; Mycobacterium tuberculosis complex; Mycobacterium tuberculosis;	NCBI: 1417012	4396993	99.94	0.00	Yes
NCBI Assembly	GCA_000664845.1	NCBI: cellular organisms; Bacteria; Terrabacteria group; Actinobacteria; Actinomycetia; Corynebacteriales; Mycobacteriaceae; Mycobacterium; Mycobacterium tuberculosis complex; Mycobacterium tuberculosis;	NCBI: 1448781	4413325	99.94	0.00	Yes
NCBI Assembly	GCA_000664245.1	NCBI: cellular organisms; Bacteria; Terrabacteria group; Actinobacteria; Actinomycetia; Corynebacteriales; Mycobacteriaceae; Mycobacterium; Mycobacterium tuberculosis complex; Mycobacterium tuberculosis;	NCBI: 1448748	4426330	99.94	0.00	Yes
NCBI Assembly	GCA_000667905.1	NCBI: cellular organisms; Bacteria; Terrabacteria group; Actinobacteria; Actinomycetia; Corynebacteriales; Mycobacteriaceae; Mycobacterium; Mycobacterium tuberculosis complex; Mycobacterium tuberculosis;	NCBI: 1402585	4403777	99.94	0.00	Yes
NCBI Assembly	GCA_000666605.1	NCBI: cellular organisms; Bacteria; Terrabacteria group; Actinobacteria; Actinomycetia; Corynebacteriales; Mycobacteriaceae; Mycobacterium; Mycobacterium tuberculosis complex; Mycobacterium tuberculosis; Mycobacterium tuberculosis variant africanum;	NCBI: 1354175	4396426	99.94	0.00	Yes
NCBI Assembly	GCA_000671935.1	NCBI: cellular organisms; Bacteria; Terrabacteria group; Actinobacteria; Actinomycetia; Corynebacteriales; Mycobacteriaceae; Mycobacterium; Mycobacterium tuberculosis complex; Mycobacterium tuberculosis;	NCBI: 1397861	4403191	99.94	0.00	Yes
NCBI Assembly	GCA_000668555.1	NCBI: cellular organisms; Bacteria; Terrabacteria group; Actinobacteria; Actinomycetia; Corynebacteriales; Mycobacteriaceae; Mycobacterium; Mycobacterium tuberculosis complex; Mycobacterium tuberculosis;	NCBI: 1408934	4414160	99.94	0.00	Yes
NCBI Assembly	GCA_000666285.1	NCBI: cellular organisms; Bacteria; Terrabacteria group; Actinobacteria; Actinomycetia; Corynebacteriales; Mycobacteriaceae; Mycobacterium; Mycobacterium tuberculosis complex; Mycobacterium tuberculosis;	NCBI: 1354144	4415881	99.94	0.00	Yes
NCBI Assembly	GCA_000663905.1	NCBI: cellular organisms; Bacteria; Terrabacteria group; Actinobacteria; Actinomycetia; Corynebacteriales; Mycobacteriaceae; Mycobacterium; Mycobacterium tuberculosis complex; Mycobacterium tuberculosis;	NCBI: 1448730	4397133	99.94	0.07	Yes
NCBI Assembly	GCA_000669895.1	NCBI: cellular organisms; Bacteria; Terrabacteria group; Actinobacteria; Actinomycetia; Corynebacteriales; Mycobacteriaceae; Mycobacterium; Mycobacterium tuberculosis complex; Mycobacterium tuberculosis;	NCBI: 1421919	4403402	99.94	0.00	Yes
NCBI Assembly	GCA_000664525.1	NCBI: cellular organisms; Bacteria; Terrabacteria group; Actinobacteria; Actinomycetia; Corynebacteriales; Mycobacteriaceae; Mycobacterium; Mycobacterium tuberculosis complex; Mycobacterium tuberculosis;	NCBI: 1448763	4407820	99.94	0.00	Yes
NCBI Assembly	GCA_000673795.1	NCBI: cellular organisms; Bacteria; Terrabacteria group; Actinobacteria; Actinomycetia; Corynebacteriales; Mycobacteriaceae; Mycobacterium; Mycobacterium tuberculosis complex; Mycobacterium tuberculosis;	NCBI: 1400887	4390328	99.94	0.10	Yes
NCBI Assembly	GCA_000669395.1	NCBI: cellular organisms; Bacteria; Terrabacteria group; Actinobacteria; Actinomycetia; Corynebacteriales; Mycobacteriaceae; Mycobacterium; Mycobacterium tuberculosis complex; Mycobacterium tuberculosis;	NCBI: 1417013	4407066	99.94	0.00	Yes

NCBI Assembly	GCA_000664865.1	NCBI: cellular organisms; Bacteria; Terrabacteria group; Actinobacteria; Actinomycetia; Corynebacteriales; Mycobacteriaceae; Mycobacterium; Mycobacterium tuberculosis complex; Mycobacterium tuberculosis;	NCBI: 1448783	4413793	99.94	0.00	Yes
NCBI Assembly	GCA_000664265.1	NCBI: cellular organisms; Bacteria; Terrabacteria group; Actinobacteria; Actinomycetia; Corynebacteriales; Mycobacteriaceae; Mycobacterium; Mycobacterium tuberculosis complex; Mycobacterium tuberculosis;	NCBI: 1448750	4395796	99.94	0.00	Yes
NCBI Assembly	GCA_000667935.1	NCBI: cellular organisms; Bacteria; Terrabacteria group; Actinobacteria; Actinomycetia; Corynebacteriales; Mycobacteriaceae; Mycobacterium; Mycobacterium tuberculosis complex; Mycobacterium tuberculosis;	NCBI: 1402587	4408928	99.94	0.00	Yes
NCBI Assembly	GCA_000666625.1	NCBI: cellular organisms; Bacteria; Terrabacteria group; Actinobacteria; Actinomycetia; Corynebacteriales; Mycobacteriaceae; Mycobacterium; Mycobacterium tuberculosis complex; Mycobacterium tuberculosis; Mycobacterium tuberculosis variant africanum;	NCBI: 1354176	4416566	99.94	0.00	Yes
NCBI Assembly	GCA_000671955.1	NCBI: cellular organisms; Bacteria; Terrabacteria group; Actinobacteria; Actinomycetia; Corynebacteriales; Mycobacteriaceae; Mycobacterium; Mycobacterium tuberculosis complex; Mycobacterium tuberculosis;	NCBI: 1397862	4405094	99.94	0.00	Yes
NCBI Assembly	GCA_000668575.1	NCBI: cellular organisms; Bacteria; Terrabacteria group; Actinobacteria; Actinomycetia; Corynebacteriales; Mycobacteriaceae; Mycobacterium; Mycobacterium tuberculosis complex; Mycobacterium tuberculosis;	NCBI: 1408935	4418887	99.94	1.00	Yes
NCBI Assembly	GCA_000673815.1	NCBI: cellular organisms; Bacteria; Terrabacteria group; Actinobacteria; Actinomycetia; Corynebacteriales; Mycobacteriaceae; Mycobacterium; Mycobacterium tuberculosis complex; Mycobacterium tuberculosis;	NCBI: 1400888	4416019	99.94	0.00	Yes
NCBI Assembly	GCA_000663925.1	NCBI: cellular organisms; Bacteria; Terrabacteria group; Actinobacteria; Actinomycetia; Corynebacteriales; Mycobacteriaceae; Mycobacterium; Mycobacterium tuberculosis complex; Mycobacterium tuberculosis;	NCBI: 1448731	4430602	99.94	0.00	Yes
NCBI Assembly	GCA_000664545.1	NCBI: cellular organisms; Bacteria; Terrabacteria group; Actinobacteria; Actinomycetia; Corynebacteriales; Mycobacteriaceae; Mycobacterium; Mycobacterium tuberculosis complex; Mycobacterium tuberculosis;	NCBI: 1448764	4418293	99.94	0.00	Yes
NCBI Assembly	GCA_000669915.1	NCBI: cellular organisms; Bacteria; Terrabacteria group; Actinobacteria; Actinomycetia; Corynebacteriales; Mycobacteriaceae; Mycobacterium; Mycobacterium tuberculosis complex; Mycobacterium tuberculosis;	NCBI: 1421920	4405475	99.94	0.00	Yes
NCBI Assembly	GCA_000666305.1	NCBI: cellular organisms; Bacteria; Terrabacteria group; Actinobacteria; Actinomycetia; Corynebacteriales; Mycobacteriaceae; Mycobacterium; Mycobacterium tuberculosis complex; Mycobacterium tuberculosis; Mycobacterium tuberculosis variant africanum;	NCBI: 1354166	4389797	99.94	0.00	Yes
NCBI Assembly	GCA_000669415.1	NCBI: cellular organisms; Bacteria; Terrabacteria group; Actinobacteria; Actinomycetia; Corynebacteriales; Mycobacteriaceae; Mycobacterium; Mycobacterium tuberculosis complex; Mycobacterium tuberculosis;	NCBI: 1417014	4375025	99.94	0.00	Yes
NCBI Assembly	GCA_000664885.1	NCBI: cellular organisms; Bacteria; Terrabacteria group; Actinobacteria; Actinomycetia; Corynebacteriales; Mycobacteriaceae; Mycobacterium; Mycobacterium tuberculosis complex; Mycobacterium tuberculosis;	NCBI: 1448784	4407982	99.94	0.00	Yes
NCBI Assembly	GCA_000664285.1	NCBI: cellular organisms; Bacteria; Terrabacteria group; Actinobacteria; Actinomycetia; Corynebacteriales; Mycobacteriaceae; Mycobacterium; Mycobacterium tuberculosis complex; Mycobacterium tuberculosis;	NCBI: 1448751	4399380	99.94	0.00	Yes
NCBI Assembly	GCA_000667955.1	NCBI: cellular organisms; Bacteria; Terrabacteria group; Actinobacteria; Actinomycetia; Corynebacteriales; Mycobacteriaceae; Mycobacterium; Mycobacterium tuberculosis complex; Mycobacterium tuberculosis;	NCBI: 1402588	4403327	99.94	0.00	Yes
NCBI Assembly	GCA_000668595.1	NCBI: cellular organisms; Bacteria; Terrabacteria group; Actinobacteria; Actinomycetia; Corynebacteriales; Mycobacteriaceae; Mycobacterium; Mycobacterium tuberculosis complex; Mycobacterium tuberculosis;	NCBI: 1408936	4429827	99.94	0.00	Yes
NCBI Assembly	GCA_000671975.1	NCBI: cellular organisms; Bacteria; Terrabacteria group; Actinobacteria; Actinomycetia; Corynebacteriales; Mycobacteriaceae; Mycobacterium; Mycobacterium tuberculosis complex; Mycobacterium tuberculosis;	NCBI: 1397863	4416592	99.94	0.00	Yes
NCBI Assembly	GCA_000666645.1	NCBI: cellular organisms; Bacteria; Terrabacteria group; Actinobacteria; Actinomycetia; Corynebacteriales; Mycobacteriaceae; Mycobacterium; Mycobacterium tuberculosis complex; Mycobacterium tuberculosis; Mycobacterium tuberculosis variant africanum;	NCBI: 1354177	4380004	99.94	0.00	Yes

NCBI Assembly	GCA_000663945.1	NCBI: cellular organisms; Bacteria; Terrabacteria group; Actinobacteria; Actinomycetia; Corynebacteriales; Mycobacteriaceae; Mycobacterium; Mycobacterium tuberculosis complex; Mycobacterium tuberculosis;	NCBI: 1448732	4410616	99.94	0.00	Yes
NCBI Assembly	GCA_000664565.1	NCBI: cellular organisms; Bacteria; Terrabacteria group; Actinobacteria; Actinomycetia; Corynebacteriales; Mycobacteriaceae; Mycobacterium; Mycobacterium tuberculosis complex; Mycobacterium tuberculosis;	NCBI: 1448765	4423474	99.83	0.00	Yes
NCBI Assembly	GCA_000669935.1	NCBI: cellular organisms; Bacteria; Terrabacteria group; Actinobacteria; Actinomycetia; Corynebacteriales; Mycobacteriaceae; Mycobacterium; Mycobacterium tuberculosis complex; Mycobacterium tuberculosis;	NCBI: 1421921	4413599	99.94	0.00	Yes
NCBI Assembly	GCA_000673835.1	NCBI: cellular organisms; Bacteria; Terrabacteria group; Actinobacteria; Actinomycetia; Corynebacteriales; Mycobacteriaceae; Mycobacterium; Mycobacterium tuberculosis complex; Mycobacterium tuberculosis;	NCBI: 1400889	4406354	99.94	0.00	Yes
NCBI Assembly	GCA_000666325.1	NCBI: cellular organisms; Bacteria; Terrabacteria group; Actinobacteria; Actinomycetia; Corynebacteriales; Mycobacteriaceae; Mycobacterium; Mycobacterium tuberculosis complex; Mycobacterium tuberculosis;	NCBI: 1354145	4422200	99.94	0.00	Yes
NCBI Assembly	GCA_000664905.1	NCBI: cellular organisms; Bacteria; Terrabacteria group; Actinobacteria; Actinomycetia; Corynebacteriales; Mycobacteriaceae; Mycobacterium; Mycobacterium tuberculosis complex; Mycobacterium tuberculosis;	NCBI: 1448785	4411801	99.94	0.00	Yes
NCBI Assembly	GCA_000668815.1	NCBI: cellular organisms; Bacteria; Terrabacteria group; Actinobacteria; Actinomycetia; Corynebacteriales; Mycobacteriaceae; Mycobacterium; Mycobacterium tuberculosis complex; Mycobacterium tuberculosis;	NCBI: 1408948	4403825	99.94	0.00	Yes
NCBI Assembly	GCA_000667975.1	NCBI: cellular organisms; Bacteria; Terrabacteria group; Actinobacteria; Actinomycetia; Corynebacteriales; Mycobacteriaceae; Mycobacterium; Mycobacterium tuberculosis complex; Mycobacterium tuberculosis;	NCBI: 1402589	4401679	99.94	0.00	Yes
NCBI Assembly	GCA_000671995.1	NCBI: cellular organisms; Bacteria; Terrabacteria group; Actinobacteria; Actinomycetia; Corynebacteriales; Mycobacteriaceae; Mycobacterium; Mycobacterium tuberculosis complex; Mycobacterium tuberculosis;	NCBI: 1397864	4407861	99.94	0.00	Yes
NCBI Assembly	GCA_000668615.1	NCBI: cellular organisms; Bacteria; Terrabacteria group; Actinobacteria; Actinomycetia; Corynebacteriales; Mycobacteriaceae; Mycobacterium; Mycobacterium tuberculosis complex; Mycobacterium tuberculosis;	NCBI: 1408937	4401378	99.94	0.00	Yes
NCBI Assembly	GCA_000664305.1	NCBI: cellular organisms; Bacteria; Terrabacteria group; Actinobacteria; Actinomycetia; Corynebacteriales; Mycobacteriaceae; Mycobacterium; Mycobacterium tuberculosis complex; Mycobacterium tuberculosis;	NCBI: 1448752	4415032	99.94	0.25	Yes
NCBI Assembly	GCA_000666665.1	NCBI: cellular organisms; Bacteria; Terrabacteria group; Actinobacteria; Actinomycetia; Corynebacteriales; Mycobacteriaceae; Mycobacterium; Mycobacterium tuberculosis complex; Mycobacterium tuberculosis;	NCBI: 1354158	4409692	99.94	0.00	Yes
NCBI Assembly	GCA_000663965.1	NCBI: cellular organisms; Bacteria; Terrabacteria group; Actinobacteria; Actinomycetia; Corynebacteriales; Mycobacteriaceae; Mycobacterium; Mycobacterium tuberculosis complex; Mycobacterium tuberculosis;	NCBI: 1448733	4386357	99.94	0.00	Yes
NCBI Assembly	GCA_000669955.1	NCBI: cellular organisms; Bacteria; Terrabacteria group; Actinobacteria; Actinomycetia; Corynebacteriales; Mycobacteriaceae; Mycobacterium; Mycobacterium tuberculosis complex; Mycobacterium tuberculosis;	NCBI: 1421922	4396146	99.94	0.00	Yes
NCBI Assembly	GCA_000664585.1	NCBI: cellular organisms; Bacteria; Terrabacteria group; Actinobacteria; Actinomycetia; Corynebacteriales; Mycobacteriaceae; Mycobacterium; Mycobacterium tuberculosis complex; Mycobacterium tuberculosis;	NCBI: 1448766	4427505	99.94	0.00	Yes
NCBI Assembly	GCA_000666345.1	NCBI: cellular organisms; Bacteria; Terrabacteria group; Actinobacteria; Actinomycetia; Corynebacteriales; Mycobacteriaceae; Mycobacterium; Mycobacterium tuberculosis complex; Mycobacterium tuberculosis;	NCBI: 1354146	4415937	99.94	0.00	Yes
NCBI Assembly	GCA_000673855.1	NCBI: cellular organisms; Bacteria; Terrabacteria group; Actinobacteria; Actinomycetia; Corynebacteriales; Mycobacteriaceae; Mycobacterium; Mycobacterium tuberculosis complex; Mycobacterium tuberculosis;	NCBI: 1400891	4414368	99.94	0.00	Yes
NCBI Assembly	GCA_000664925.1	NCBI: cellular organisms; Bacteria; Terrabacteria group; Actinobacteria; Actinomycetia; Corynebacteriales; Mycobacteriaceae; Mycobacterium; Mycobacterium tuberculosis complex; Mycobacterium tuberculosis;	NCBI: 1448786	4432879	99.94	0.00	Yes

NCBI Assembly	GCA_000668835.1	NCBI: cellular organisms; Bacteria; Terrabacteria group; Actinobacteria; Actinomycetia; Corynebacteriales; Mycobacteriaceae; Mycobacterium; Mycobacterium tuberculosis complex; Mycobacterium tuberculosis;	NCBI: 1408949	4397623	99.94	0.00	Yes
NCBI Assembly	GCA_000664325.1	NCBI: cellular organisms; Bacteria; Terrabacteria group; Actinobacteria; Actinomycetia; Corynebacteriales; Mycobacteriaceae; Mycobacterium; Mycobacterium tuberculosis complex; Mycobacterium tuberculosis;	NCBI: 1448753	4423081	99.94	0.00	Yes
NCBI Assembly	GCA_000668635.1	NCBI: cellular organisms; Bacteria; Terrabacteria group; Actinobacteria; Actinomycetia; Corynebacteriales; Mycobacteriaceae; Mycobacterium; Mycobacterium tuberculosis complex; Mycobacterium tuberculosis;	NCBI: 1408938	4390027	99.94	0.00	Yes
NCBI Assembly	GCA_000663985.1	NCBI: cellular organisms; Bacteria; Terrabacteria group; Actinobacteria; Actinomycetia; Corynebacteriales; Mycobacteriaceae; Mycobacterium; Mycobacterium tuberculosis complex; Mycobacterium tuberculosis;	NCBI: 1448734	4423441	99.94	0.00	Yes
NCBI Assembly	GCA_000667995.1	NCBI: cellular organisms; Bacteria; Terrabacteria group; Actinobacteria; Actinomycetia; Corynebacteriales; Mycobacteriaceae; Mycobacterium; Mycobacterium tuberculosis complex; Mycobacterium tuberculosis;	NCBI: 1402590	4423003	99.94	0.00	Yes
NCBI Assembly	GCA_000672015.1	NCBI: cellular organisms; Bacteria; Terrabacteria group; Actinobacteria; Actinomycetia; Corynebacteriales; Mycobacteriaceae; Mycobacterium; Mycobacterium tuberculosis complex; Mycobacterium tuberculosis;	NCBI: 1397865	4403298	99.94	0.00	Yes
NCBI Assembly	GCA_000666685.1	NCBI: cellular organisms; Bacteria; Terrabacteria group; Actinobacteria; Actinomycetia; Corynebacteriales; Mycobacteriaceae; Mycobacterium; Mycobacterium tuberculosis complex; Mycobacterium tuberculosis; Mycobacterium tuberculosis variant africanum;	NCBI: 1354178	4385125	99.94	0.00	Yes
NCBI Assembly	GCA_000666365.1	NCBI: cellular organisms; Bacteria; Terrabacteria group; Actinobacteria; Actinomycetia; Corynebacteriales; Mycobacteriaceae; Mycobacterium; Mycobacterium tuberculosis complex; Mycobacterium tuberculosis;	NCBI: 1354147	4411490	99.94	0.00	Yes
NCBI Assembly	GCA_000669975.1	NCBI: cellular organisms; Bacteria; Terrabacteria group; Actinobacteria; Actinomycetia; Corynebacteriales; Mycobacteriaceae; Mycobacterium; Mycobacterium tuberculosis complex; Mycobacterium tuberculosis;	NCBI: 1421923	4425593	99.94	0.00	Yes
NCBI Assembly	GCA_000664605.1	NCBI: cellular organisms; Bacteria; Terrabacteria group; Actinobacteria; Actinomycetia; Corynebacteriales; Mycobacteriaceae; Mycobacterium; Mycobacterium tuberculosis complex; Mycobacterium tuberculosis;	NCBI: 1448767	4432342	99.94	0.11	Yes
NCBI Assembly	GCA_000673875.1	NCBI: cellular organisms; Bacteria; Terrabacteria group; Actinobacteria; Actinomycetia; Corynebacteriales; Mycobacteriaceae; Mycobacterium; Mycobacterium tuberculosis complex; Mycobacterium tuberculosis;	NCBI: 1400893	4459218	99.94	0.33	Yes
NCBI Assembly	GCA_000664945.1	NCBI: cellular organisms; Bacteria; Terrabacteria group; Actinobacteria; Actinomycetia; Corynebacteriales; Mycobacteriaceae; Mycobacterium; Mycobacterium tuberculosis complex; Mycobacterium tuberculosis;	NCBI: 1448787	4410820	99.94	0.00	Yes
NCBI Assembly	GCA_000668855.1	NCBI: cellular organisms; Bacteria; Terrabacteria group; Actinobacteria; Actinomycetia; Corynebacteriales; Mycobacteriaceae; Mycobacterium; Mycobacterium tuberculosis complex; Mycobacterium tuberculosis;	NCBI: 1408950	4393797	99.94	0.00	Yes
NCBI Assembly	GCA_000664345.1	NCBI: cellular organisms; Bacteria; Terrabacteria group; Actinobacteria; Actinomycetia; Corynebacteriales; Mycobacteriaceae; Mycobacterium; Mycobacterium tuberculosis complex; Mycobacterium tuberculosis;	NCBI: 1448754	4424725	99.94	0.00	Yes
NCBI Assembly	GCA_000664005.1	NCBI: cellular organisms; Bacteria; Terrabacteria group; Actinobacteria; Actinomycetia; Corynebacteriales; Mycobacteriaceae; Mycobacterium; Mycobacterium tuberculosis complex; Mycobacterium tuberculosis;	NCBI: 1448735	4444926	99.94	0.33	Yes
NCBI Assembly	GCA_000668655.1	NCBI: cellular organisms; Bacteria; Terrabacteria group; Actinobacteria; Actinomycetia; Corynebacteriales; Mycobacteriaceae; Mycobacterium; Mycobacterium tuberculosis complex; Mycobacterium tuberculosis;	NCBI: 1408939	4414791	99.94	0.00	Yes
NCBI Assembly	GCA_000668015.1	NCBI: cellular organisms; Bacteria; Terrabacteria group; Actinobacteria; Actinomycetia; Corynebacteriales; Mycobacteriaceae; Mycobacterium; Mycobacterium tuberculosis complex; Mycobacterium tuberculosis;	NCBI: 1402591	4408819	99.94	0.00	Yes
NCBI Assembly	GCA_000672035.1	NCBI: cellular organisms; Bacteria; Terrabacteria group; Actinobacteria; Actinomycetia; Corynebacteriales; Mycobacteriaceae; Mycobacterium; Mycobacterium tuberculosis complex; Mycobacterium tuberculosis;	NCBI: 1397866	4396498	99.94	0.00	Yes

NCBI Assembly	GCA_000666705.1	NCBI: cellular organisms; Bacteria; Terrabacteria group; Actinobacteria; Actinomycetia; Corynebacteriales; Mycobacteriaceae; Mycobacterium; Mycobacterium tuberculosis complex; Mycobacterium tuberculosis; Mycobacterium tuberculosis variant africanum;	NCBI: 1354179	4384811	99.94	0.00	Yes
NCBI Assembly	GCA_000666385.1	NCBI: cellular organisms; Bacteria; Terrabacteria group; Actinobacteria; Actinomycetia; Corynebacteriales; Mycobacteriaceae; Mycobacterium; Mycobacterium tuberculosis complex; Mycobacterium tuberculosis; Mycobacterium tuberculosis variant bovis;	NCBI: 1354193	4391304	99.94	0.00	Yes
NCBI Assembly	GCA_000664625.1	NCBI: cellular organisms; Bacteria; Terrabacteria group; Actinobacteria; Actinomycetia; Corynebacteriales; Mycobacteriaceae; Mycobacterium; Mycobacterium tuberculosis complex; Mycobacterium tuberculosis;	NCBI: 1448768	4420081	99.94	0.00	Yes
NCBI Assembly	GCA_000669995.1	NCBI: cellular organisms; Bacteria; Terrabacteria group; Actinobacteria; Actinomycetia; Corynebacteriales; Mycobacteriaceae; Mycobacterium; Mycobacterium tuberculosis complex; Mycobacterium tuberculosis;	NCBI: 1421924	4406341	99.94	0.00	Yes
NCBI Assembly	GCA_000673895.1	NCBI: cellular organisms; Bacteria; Terrabacteria group; Actinobacteria; Actinomycetia; Corynebacteriales; Mycobacteriaceae; Mycobacterium; Mycobacterium tuberculosis complex; Mycobacterium tuberculosis;	NCBI: 1400894	4396062	99.94	0.00	Yes
NCBI Assembly	GCA_000664965.1	NCBI: cellular organisms; Bacteria; Terrabacteria group; Actinobacteria; Actinomycetia; Corynebacteriales; Mycobacteriaceae; Mycobacterium; Mycobacterium tuberculosis complex; Mycobacterium tuberculosis;	NCBI: 1448788	4424869	99.94	0.00	Yes
NCBI Assembly	GCA_000668875.1	NCBI: cellular organisms; Bacteria; Terrabacteria group; Actinobacteria; Actinomycetia; Corynebacteriales; Mycobacteriaceae; Mycobacterium; Mycobacterium tuberculosis complex; Mycobacterium tuberculosis;	NCBI: 1408951	4419488	99.94	0.00	Yes
NCBI Assembly	GCA_000664365.1	NCBI: cellular organisms; Bacteria; Terrabacteria group; Actinobacteria; Actinomycetia; Corynebacteriales; Mycobacteriaceae; Mycobacterium; Mycobacterium tuberculosis complex; Mycobacterium tuberculosis;	NCBI: 1448755	4425666	99.94	0.00	Yes
NCBI Assembly	GCA_000668675.1	NCBI: cellular organisms; Bacteria; Terrabacteria group; Actinobacteria; Actinomycetia; Corynebacteriales; Mycobacteriaceae; Mycobacterium; Mycobacterium tuberculosis complex; Mycobacterium tuberculosis;	NCBI: 1408940	4431719	99.94	0.00	Yes
NCBI Assembly	GCA_000664025.1	NCBI: cellular organisms; Bacteria; Terrabacteria group; Actinobacteria; Actinomycetia; Corynebacteriales; Mycobacteriaceae; Mycobacterium; Mycobacterium tuberculosis complex; Mycobacterium tuberculosis;	NCBI: 1448736	4421400	99.94	0.00	Yes
NCBI Assembly	GCA_000668035.1	NCBI: cellular organisms; Bacteria; Terrabacteria group; Actinobacteria; Actinomycetia; Corynebacteriales; Mycobacteriaceae; Mycobacterium; Mycobacterium tuberculosis complex; Mycobacterium tuberculosis;	NCBI: 1402592	4447185	99.94	0.00	Yes
NCBI Assembly	GCA_000672055.1	NCBI: cellular organisms; Bacteria; Terrabacteria group; Actinobacteria; Actinomycetia; Corynebacteriales; Mycobacteriaceae; Mycobacterium; Mycobacterium tuberculosis complex; Mycobacterium tuberculosis;	NCBI: 1397867	4401880	99.94	0.00	Yes
NCBI Assembly	GCA_000666725.1	NCBI: cellular organisms; Bacteria; Terrabacteria group; Actinobacteria; Actinomycetia; Corynebacteriales; Mycobacteriaceae; Mycobacterium; Mycobacterium tuberculosis complex; Mycobacterium tuberculosis; Mycobacterium tuberculosis variant africanum;	NCBI: 1354180	4403369	99.91	0.00	Yes
NCBI Assembly	GCA_000664645.1	NCBI: cellular organisms; Bacteria; Terrabacteria group; Actinobacteria; Actinomycetia; Corynebacteriales; Mycobacteriaceae; Mycobacterium; Mycobacterium tuberculosis complex; Mycobacterium tuberculosis;	NCBI: 1448769	4393077	99.83	0.00	Yes
NCBI Assembly	GCA_000666405.1	NCBI: cellular organisms; Bacteria; Terrabacteria group; Actinobacteria; Actinomycetia; Corynebacteriales; Mycobacteriaceae; Mycobacterium; Mycobacterium tuberculosis complex; Mycobacterium tuberculosis; Mycobacterium tuberculosis variant africanum;	NCBI: 1354171	4388539	99.94	0.00	Yes
NCBI Assembly	GCA_000673915.1	NCBI: cellular organisms; Bacteria; Terrabacteria group; Actinobacteria; Actinomycetia; Corynebacteriales; Mycobacteriaceae; Mycobacterium; Mycobacterium tuberculosis complex; Mycobacterium tuberculosis;	NCBI: 1400895	4421220	99.94	0.00	Yes
NCBI Assembly	GCA_000670015.1	NCBI: cellular organisms; Bacteria; Terrabacteria group; Actinobacteria; Actinomycetia; Corynebacteriales; Mycobacteriaceae; Mycobacterium; Mycobacterium tuberculosis complex; Mycobacterium tuberculosis;	NCBI: 1421925	4403008	99.94	0.00	Yes
NCBI Assembly	GCA_000664985.1	NCBI: cellular organisms; Bacteria; Terrabacteria group; Actinobacteria; Actinomycetia; Corynebacteriales; Mycobacteriaceae; Mycobacterium; Mycobacterium tuberculosis complex; Mycobacterium tuberculosis;	NCBI: 1448789	4390910	99.94	0.00	Yes

NCBI Assembly	GCA_000668895.1	NCBI: cellular organisms; Bacteria; Terrabacteria group; Actinobacteria; Actinomycetia; Corynebacteriales; Mycobacteriaceae; Mycobacterium; Mycobacterium tuberculosis complex; Mycobacterium tuberculosis;	NCBI: 1408952	4392496	99.78	0.00	Yes
NCBI Assembly	GCA_000664385.1	NCBI: cellular organisms; Bacteria; Terrabacteria group; Actinobacteria; Actinomycetia; Corynebacteriales; Mycobacteriaceae; Mycobacterium; Mycobacterium tuberculosis complex; Mycobacterium tuberculosis;	NCBI: 1448756	4389875	99.94	0.00	Yes
NCBI Assembly	GCA_000668695.1	NCBI: cellular organisms; Bacteria; Terrabacteria group; Actinobacteria; Actinomycetia; Corynebacteriales; Mycobacteriaceae; Mycobacterium; Mycobacterium tuberculosis complex; Mycobacterium tuberculosis;	NCBI: 1408941	4394605	99.94	0.33	Yes
NCBI Assembly	GCA_000664045.1	NCBI: cellular organisms; Bacteria; Terrabacteria group; Actinobacteria; Actinomycetia; Corynebacteriales; Mycobacteriaceae; Mycobacterium; Mycobacterium tuberculosis complex; Mycobacterium tuberculosis;	NCBI: 1448737	4388482	99.94	0.00	Yes
NCBI Assembly	GCA_000668055.1	NCBI: cellular organisms; Bacteria; Terrabacteria group; Actinobacteria; Actinomycetia; Corynebacteriales; Mycobacteriaceae; Mycobacterium; Mycobacterium tuberculosis complex; Mycobacterium tuberculosis;	NCBI: 1402593	4399336	99.94	0.00	Yes
NCBI Assembly	GCA_000672075.1	NCBI: cellular organisms; Bacteria; Terrabacteria group; Actinobacteria; Actinomycetia; Corynebacteriales; Mycobacteriaceae; Mycobacterium; Mycobacterium tuberculosis complex; Mycobacterium tuberculosis;	NCBI: 1397868	4430328	99.94	0.00	Yes
NCBI Assembly	GCA_000666745.1	NCBI: cellular organisms; Bacteria; Terrabacteria group; Actinobacteria; Actinomycetia; Corynebacteriales; Mycobacteriaceae; Mycobacterium; Mycobacterium tuberculosis complex; Mycobacterium tuberculosis;	NCBI: 1354160	4442972	99.86	0.00	Yes
NCBI Assembly	GCA_000664665.1	NCBI: cellular organisms; Bacteria; Terrabacteria group; Actinobacteria; Actinomycetia; Corynebacteriales; Mycobacteriaceae; Mycobacterium; Mycobacterium tuberculosis complex; Mycobacterium tuberculosis;	NCBI: 1448770	4436657	99.94	0.00	Yes
NCBI Assembly	GCA_000666425.1	NCBI: cellular organisms; Bacteria; Terrabacteria group; Actinobacteria; Actinomycetia; Corynebacteriales; Mycobacteriaceae; Mycobacterium; Mycobacterium tuberculosis complex; Mycobacterium tuberculosis; Mycobacterium tuberculosis variant africanum;	NCBI: 1354172	4427405	99.94	0.00	Yes
NCBI Assembly	GCA_000670035.1	NCBI: cellular organisms; Bacteria; Terrabacteria group; Actinobacteria; Actinomycetia; Corynebacteriales; Mycobacteriaceae; Mycobacterium; Mycobacterium tuberculosis complex; Mycobacterium tuberculosis;	NCBI: 1421926	4389427	99.94	0.00	Yes
NCBI Assembly	GCA_000673935.1	NCBI: cellular organisms; Bacteria; Terrabacteria group; Actinobacteria; Actinomycetia; Corynebacteriales; Mycobacteriaceae; Mycobacterium; Mycobacterium tuberculosis complex; Mycobacterium tuberculosis;	NCBI: 1400898	4401152	99.94	0.00	Yes
NCBI Assembly	GCA_000665005.1	NCBI: cellular organisms; Bacteria; Terrabacteria group; Actinobacteria; Actinomycetia; Corynebacteriales; Mycobacteriaceae; Mycobacterium; Mycobacterium tuberculosis complex; Mycobacterium tuberculosis;	NCBI: 1448790	4442130	99.61	0.00	Yes
NCBI Assembly	GCA_000668915.1	NCBI: cellular organisms; Bacteria; Terrabacteria group; Actinobacteria; Actinomycetia; Corynebacteriales; Mycobacteriaceae; Mycobacterium; Mycobacterium tuberculosis complex; Mycobacterium tuberculosis;	NCBI: 1408953	4402015	99.94	0.00	Yes
NCBI Assembly	GCA_000668075.1	NCBI: cellular organisms; Bacteria; Terrabacteria group; Actinobacteria; Actinomycetia; Corynebacteriales; Mycobacteriaceae; Mycobacterium; Mycobacterium tuberculosis complex; Mycobacterium tuberculosis;	NCBI: 1402595	4401455	99.94	0.00	Yes
NCBI Assembly	GCA_000664405.1	NCBI: cellular organisms; Bacteria; Terrabacteria group; Actinobacteria; Actinomycetia; Corynebacteriales; Mycobacteriaceae; Mycobacterium; Mycobacterium tuberculosis complex; Mycobacterium tuberculosis;	NCBI: 1448757	4394022	99.94	0.00	Yes
NCBI Assembly	GCA_000668715.1	NCBI: cellular organisms; Bacteria; Terrabacteria group; Actinobacteria; Actinomycetia; Corynebacteriales; Mycobacteriaceae; Mycobacterium; Mycobacterium tuberculosis complex; Mycobacterium tuberculosis;	NCBI: 1408942	4460289	99.78	0.00	Yes
NCBI Assembly	GCA_000664065.1	NCBI: cellular organisms; Bacteria; Terrabacteria group; Actinobacteria; Actinomycetia; Corynebacteriales; Mycobacteriaceae; Mycobacterium; Mycobacterium tuberculosis complex; Mycobacterium tuberculosis;	NCBI: 1448738	4438914	99.94	0.36	Yes
NCBI Assembly	GCA_000672095.1	NCBI: cellular organisms; Bacteria; Terrabacteria group; Actinobacteria; Actinomycetia; Corynebacteriales; Mycobacteriaceae; Mycobacterium; Mycobacterium tuberculosis complex; Mycobacterium tuberculosis;	NCBI: 1397869	4416566	99.94	0.00	Yes

NCBI Assembly	GCA_000664685.1	NCBI: cellular organisms; Bacteria; Terrabacteria group; Actinobacteria; Actinomycetia; Corynebacteriales; Mycobacteriaceae; Mycobacterium; Mycobacterium tuberculosis complex; Mycobacterium tuberculosis;	NCBI: 1448771	4411860	99.78	0.00	Yes
NCBI Assembly	GCA_000666765.1	NCBI: cellular organisms; Bacteria; Terrabacteria group; Actinobacteria; Actinomycetia; Corynebacteriales; Mycobacteriaceae; Mycobacterium; Mycobacterium tuberculosis complex; Mycobacterium tuberculosis; Mycobacterium tuberculosis variant africanum;	NCBI: 1354181	4410558	99.94	0.33	Yes
NCBI Assembly	GCA_000673955.1	NCBI: cellular organisms; Bacteria; Terrabacteria group; Actinobacteria; Actinomycetia; Corynebacteriales; Mycobacteriaceae; Mycobacterium; Mycobacterium tuberculosis complex; Mycobacterium tuberculosis;	NCBI: 1400899	4469091	99.94	0.00	Yes
NCBI Assembly	GCA_000670055.1	NCBI: cellular organisms; Bacteria; Terrabacteria group; Actinobacteria; Actinomycetia; Corynebacteriales; Mycobacteriaceae; Mycobacterium; Mycobacterium tuberculosis complex; Mycobacterium tuberculosis;	NCBI: 1421927	4396669	99.94	0.00	Yes
NCBI Assembly	GCA_000665025.1	NCBI: cellular organisms; Bacteria; Terrabacteria group; Actinobacteria; Actinomycetia; Corynebacteriales; Mycobacteriaceae; Mycobacterium; Mycobacterium tuberculosis complex; Mycobacterium tuberculosis;	NCBI: 1448791	4416762	99.78	0.00	Yes
NCBI Assembly	GCA_000666445.1	NCBI: cellular organisms; Bacteria; Terrabacteria group; Actinobacteria; Actinomycetia; Corynebacteriales; Mycobacteriaceae; Mycobacterium; Mycobacterium tuberculosis complex; Mycobacterium tuberculosis; Mycobacterium tuberculosis variant africanum;	NCBI: 1354173	4392898	99.94	0.00	Yes
NCBI Assembly	GCA_000668935.1	NCBI: cellular organisms; Bacteria; Terrabacteria group; Actinobacteria; Actinomycetia; Corynebacteriales; Mycobacteriaceae; Mycobacterium; Mycobacterium tuberculosis complex; Mycobacterium tuberculosis;	NCBI: 1408954	4408414	99.94	0.00	Yes
NCBI Assembly	GCA_000668095.1	NCBI: cellular organisms; Bacteria; Terrabacteria group; Actinobacteria; Actinomycetia; Corynebacteriales; Mycobacteriaceae; Mycobacterium; Mycobacterium tuberculosis complex; Mycobacterium tuberculosis;	NCBI: 1402596	4408567	99.94	0.00	Yes
NCBI Assembly	GCA_000664425.1	NCBI: cellular organisms; Bacteria; Terrabacteria group; Actinobacteria; Actinomycetia; Corynebacteriales; Mycobacteriaceae; Mycobacterium; Mycobacterium tuberculosis complex; Mycobacterium tuberculosis;	NCBI: 1448758	4398911	99.94	1.06	Yes
NCBI Assembly	GCA_000668735.1	NCBI: cellular organisms; Bacteria; Terrabacteria group; Actinobacteria; Actinomycetia; Corynebacteriales; Mycobacteriaceae; Mycobacterium; Mycobacterium tuberculosis complex; Mycobacterium tuberculosis;	NCBI: 1408944	4422013	99.94	0.00	Yes
NCBI Assembly	GCA_000664085.1	NCBI: cellular organisms; Bacteria; Terrabacteria group; Actinobacteria; Actinomycetia; Corynebacteriales; Mycobacteriaceae; Mycobacterium; Mycobacterium tuberculosis complex; Mycobacterium tuberculosis;	NCBI: 1448739	4419212	99.94	0.00	Yes
NCBI Assembly	GCA_000672115.1	NCBI: cellular organisms; Bacteria; Terrabacteria group; Actinobacteria; Actinomycetia; Corynebacteriales; Mycobacteriaceae; Mycobacterium; Mycobacterium tuberculosis complex; Mycobacterium tuberculosis;	NCBI: 1397871	4406067	99.94	0.33	Yes
NCBI Assembly	GCA_000664705.1	NCBI: cellular organisms; Bacteria; Terrabacteria group; Actinobacteria; Actinomycetia; Corynebacteriales; Mycobacteriaceae; Mycobacterium; Mycobacterium tuberculosis complex; Mycobacterium tuberculosis;	NCBI: 1448772	4419491	99.94	0.00	Yes
NCBI Assembly	GCA_000666785.1	NCBI: cellular organisms; Bacteria; Terrabacteria group; Actinobacteria; Actinomycetia; Corynebacteriales; Mycobacteriaceae; Mycobacterium; Mycobacterium tuberculosis complex; Mycobacterium tuberculosis;	NCBI: 1354154	4393063	99.94	0.00	Yes
NCBI Assembly	GCA_000670075.1	NCBI: cellular organisms; Bacteria; Terrabacteria group; Actinobacteria; Actinomycetia; Corynebacteriales; Mycobacteriaceae; Mycobacterium; Mycobacterium tuberculosis complex; Mycobacterium tuberculosis;	NCBI: 1421928	4394125	99.94	0.17	Yes
NCBI Assembly	GCA_000673975.1	NCBI: cellular organisms; Bacteria; Terrabacteria group; Actinobacteria; Actinomycetia; Corynebacteriales; Mycobacteriaceae; Mycobacterium; Mycobacterium tuberculosis complex; Mycobacterium tuberculosis;	NCBI: 1400902	4448947	99.94	0.00	Yes
NCBI Assembly	GCA_000665045.1	NCBI: cellular organisms; Bacteria; Terrabacteria group; Actinobacteria; Actinomycetia; Corynebacteriales; Mycobacteriaceae; Mycobacterium; Mycobacterium tuberculosis complex; Mycobacterium tuberculosis;	NCBI: 1448792	4425314	99.94	0.00	Yes
NCBI Assembly	GCA_000666465.1	NCBI: cellular organisms; Bacteria; Terrabacteria group; Actinobacteria; Actinomycetia; Corynebacteriales; Mycobacteriaceae; Mycobacterium; Mycobacterium tuberculosis complex; Mycobacterium tuberculosis;	NCBI: 1354148	4416859	99.94	0.00	Yes

NCBI Assembly	GCA_000668955.1	NCBI: cellular organisms; Bacteria; Terrabacteria group; Actinobacteria; Actinomycetia; Corynebacteriales; Mycobacteriaceae; Mycobacterium; Mycobacterium tuberculosis complex; Mycobacterium tuberculosis;	NCBI: 1408955	4423090	99.94	0.00	Yes
NCBI Assembly	GCA_000668115.1	NCBI: cellular organisms; Bacteria; Terrabacteria group; Actinobacteria; Actinomycetia; Corynebacteriales; Mycobacteriaceae; Mycobacterium; Mycobacterium tuberculosis complex; Mycobacterium tuberculosis;	NCBI: 1402597	4436970	99.94	0.00	Yes
NCBI Assembly	GCA_000664445.1	NCBI: cellular organisms; Bacteria; Terrabacteria group; Actinobacteria; Actinomycetia; Corynebacteriales; Mycobacteriaceae; Mycobacterium; Mycobacterium tuberculosis complex; Mycobacterium tuberculosis;	NCBI: 1448759	4432715	99.94	0.00	Yes
NCBI Assembly	GCA_000668755.1	NCBI: cellular organisms; Bacteria; Terrabacteria group; Actinobacteria; Actinomycetia; Corynebacteriales; Mycobacteriaceae; Mycobacterium; Mycobacterium tuberculosis complex; Mycobacterium tuberculosis;	NCBI: 1408945	4400923	99.94	0.00	Yes
NCBI Assembly	GCA_000664105.1	NCBI: cellular organisms; Bacteria; Terrabacteria group; Actinobacteria; Actinomycetia; Corynebacteriales; Mycobacteriaceae; Mycobacterium; Mycobacterium tuberculosis complex; Mycobacterium tuberculosis;	NCBI: 1448740	4423677	99.94	0.00	Yes
NCBI Assembly	GCA_000672135.1	NCBI: cellular organisms; Bacteria; Terrabacteria group; Actinobacteria; Actinomycetia; Corynebacteriales; Mycobacteriaceae; Mycobacterium; Mycobacterium tuberculosis complex; Mycobacterium tuberculosis;	NCBI: 1397872	4428032	99.94	0.00	Yes
NCBI Assembly	GCA_000664725.1	NCBI: cellular organisms; Bacteria; Terrabacteria group; Actinobacteria; Actinomycetia; Corynebacteriales; Mycobacteriaceae; Mycobacterium; Mycobacterium tuberculosis complex; Mycobacterium tuberculosis;	NCBI: 1448774	4427569	99.94	0.00	Yes
NCBI Assembly	GCA_000666805.1	NCBI: cellular organisms; Bacteria; Terrabacteria group; Actinobacteria; Actinomycetia; Corynebacteriales; Mycobacteriaceae; Mycobacterium; Mycobacterium tuberculosis complex; Mycobacterium tuberculosis;	NCBI: 1354155	4422487	99.67	0.00	Yes
NCBI Assembly	GCA_000670095.1	NCBI: cellular organisms; Bacteria; Terrabacteria group; Actinobacteria; Actinomycetia; Corynebacteriales; Mycobacteriaceae; Mycobacterium; Mycobacterium tuberculosis complex; Mycobacterium tuberculosis;	NCBI: 1421929	4408945	99.94	0.00	Yes
NCBI Assembly	GCA_000673995.1	NCBI: cellular organisms; Bacteria; Terrabacteria group; Actinobacteria; Actinomycetia; Corynebacteriales; Mycobacteriaceae; Mycobacterium; Mycobacterium tuberculosis complex; Mycobacterium tuberculosis;	NCBI: 1400904	4400926	99.94	0.00	Yes
NCBI Assembly	GCA_000665065.1	NCBI: cellular organisms; Bacteria; Terrabacteria group; Actinobacteria; Actinomycetia; Corynebacteriales; Mycobacteriaceae; Mycobacterium; Mycobacterium tuberculosis complex; Mycobacterium tuberculosis;	NCBI: 1448794	4423786	99.94	0.00	Yes
NCBI Assembly	GCA_000666485.1	NCBI: cellular organisms; Bacteria; Terrabacteria group; Actinobacteria; Actinomycetia; Corynebacteriales; Mycobacteriaceae; Mycobacterium; Mycobacterium tuberculosis complex; Mycobacterium tuberculosis;	NCBI: 1354149	4416656	99.94	0.00	Yes
NCBI Assembly	GCA_000668135.1	NCBI: cellular organisms; Bacteria; Terrabacteria group; Actinobacteria; Actinomycetia; Corynebacteriales; Mycobacteriaceae; Mycobacterium; Mycobacterium tuberculosis complex; Mycobacterium tuberculosis;	NCBI: 1402598	4412579	99.94	0.00	Yes
NCBI Assembly	GCA_000668975.1	NCBI: cellular organisms; Bacteria; Terrabacteria group; Actinobacteria; Actinomycetia; Corynebacteriales; Mycobacteriaceae; Mycobacterium; Mycobacterium tuberculosis complex; Mycobacterium tuberculosis;	NCBI: 1408956	4421742	99.94	0.00	Yes
NCBI Assembly	GCA_000668775.1	NCBI: cellular organisms; Bacteria; Terrabacteria group; Actinobacteria; Actinomycetia; Corynebacteriales; Mycobacteriaceae; Mycobacterium; Mycobacterium tuberculosis complex; Mycobacterium tuberculosis;	NCBI: 1408946	4401392	99.94	0.00	Yes
NCBI Assembly	GCA_000667465.1	NCBI: cellular organisms; Bacteria; Terrabacteria group; Actinobacteria; Actinomycetia; Corynebacteriales; Mycobacteriaceae; Mycobacterium; Mycobacterium tuberculosis complex; Mycobacterium tuberculosis;	NCBI: 1354101	4428938	99.94	0.00	Yes
NCBI Assembly	GCA_000665725.1	NCBI: cellular organisms; Bacteria; Terrabacteria group; Actinobacteria; Actinomycetia; Corynebacteriales; Mycobacteriaceae; Mycobacterium; Mycobacterium tuberculosis complex; Mycobacterium tuberculosis;	NCBI: 1448830	4428648	99.94	0.52	Yes
NCBI Assembly	GCA_000664125.1	NCBI: cellular organisms; Bacteria; Terrabacteria group; Actinobacteria; Actinomycetia; Corynebacteriales; Mycobacteriaceae; Mycobacterium; Mycobacterium tuberculosis complex; Mycobacterium tuberculosis;	NCBI: 1448742	4427961	99.94	0.00	Yes

NCBI Assembly	GCA_000664745.1	NCBI: cellular organisms; Bacteria; Terrabacteria group; Actinobacteria; Actinomycetia; Corynebacteriales; Mycobacteriaceae; Mycobacterium; Mycobacterium tuberculosis complex; Mycobacterium tuberculosis;	NCBI: 1448775	4407229	99.94	0.00	Yes
NCBI Assembly	GCA_000666825.1	NCBI: cellular organisms; Bacteria; Terrabacteria group; Actinobacteria; Actinomycetia; Corynebacteriales; Mycobacteriaceae; Mycobacterium; Mycobacterium tuberculosis complex; Mycobacterium tuberculosis;	NCBI: 1354182	4421203	99.94	0.00	Yes
NCBI Assembly	GCA_000674015.1	NCBI: cellular organisms; Bacteria; Terrabacteria group; Actinobacteria; Actinomycetia; Corynebacteriales; Mycobacteriaceae; Mycobacterium; Mycobacterium tuberculosis complex; Mycobacterium tuberculosis;	NCBI: 1400907	4546534	99.94	0.07	Yes
NCBI Assembly	GCA_000663545.1	NCBI: cellular organisms; Bacteria; Terrabacteria group; Actinobacteria; Actinomycetia; Corynebacteriales; Mycobacteriaceae; Mycobacterium; Mycobacterium tuberculosis complex; Mycobacterium tuberculosis;	NCBI: 1448710	4403217	99.94	0.00	Yes
NCBI Assembly	GCA_000666505.1	NCBI: cellular organisms; Bacteria; Terrabacteria group; Actinobacteria; Actinomycetia; Corynebacteriales; Mycobacteriaceae; Mycobacterium; Mycobacterium tuberculosis complex; Mycobacterium tuberculosis;	NCBI: 1354150	4430712	99.94	0.00	Yes
NCBI Assembly	GCA_000665405.1	NCBI: cellular organisms; Bacteria; Terrabacteria group; Actinobacteria; Actinomycetia; Corynebacteriales; Mycobacteriaceae; Mycobacterium; Mycobacterium tuberculosis complex; Mycobacterium tuberculosis;	NCBI: 1448812	4383351	99.94	0.00	Yes
NCBI Assembly	GCA_000668995.1	NCBI: cellular organisms; Bacteria; Terrabacteria group; Actinobacteria; Actinomycetia; Corynebacteriales; Mycobacteriaceae; Mycobacterium; Mycobacterium tuberculosis complex; Mycobacterium tuberculosis;	NCBI: 1408957	4466031	99.94	0.05	Yes
NCBI Assembly	GCA_000665745.1	NCBI: cellular organisms; Bacteria; Terrabacteria group; Actinobacteria; Actinomycetia; Corynebacteriales; Mycobacteriaceae; Mycobacterium; Mycobacterium tuberculosis complex; Mycobacterium tuberculosis;	NCBI: 1448833	4416540	99.94	0.00	Yes
NCBI Assembly	GCA_000667485.1	NCBI: cellular organisms; Bacteria; Terrabacteria group; Actinobacteria; Actinomycetia; Corynebacteriales; Mycobacteriaceae; Mycobacterium; Mycobacterium tuberculosis complex; Mycobacterium tuberculosis;	NCBI: 1354102	4422672	99.94	0.00	Yes
NCBI Assembly	GCA_000668795.1	NCBI: cellular organisms; Bacteria; Terrabacteria group; Actinobacteria; Actinomycetia; Corynebacteriales; Mycobacteriaceae; Mycobacterium; Mycobacterium tuberculosis complex; Mycobacterium tuberculosis;	NCBI: 1408947	4407186	99.94	0.00	Yes
NCBI Assembly	GCA_000664145.1	NCBI: cellular organisms; Bacteria; Terrabacteria group; Actinobacteria; Actinomycetia; Corynebacteriales; Mycobacteriaceae; Mycobacterium; Mycobacterium tuberculosis complex; Mycobacterium tuberculosis;	NCBI: 1448743	4402015	99.94	0.04	Yes
NCBI Assembly	GCA_000664765.1	NCBI: cellular organisms; Bacteria; Terrabacteria group; Actinobacteria; Actinomycetia; Corynebacteriales; Mycobacteriaceae; Mycobacterium; Mycobacterium tuberculosis complex; Mycobacterium tuberculosis;	NCBI: 1448776	4426311	99.94	0.00	Yes
NCBI Assembly	GCA_000663565.1	NCBI: cellular organisms; Bacteria; Terrabacteria group; Actinobacteria; Actinomycetia; Corynebacteriales; Mycobacteriaceae; Mycobacterium; Mycobacterium tuberculosis complex; Mycobacterium tuberculosis;	NCBI: 1448711	4427283	99.94	0.00	Yes
NCBI Assembly	GCA_000665425.1	NCBI: cellular organisms; Bacteria; Terrabacteria group; Actinobacteria; Actinomycetia; Corynebacteriales; Mycobacteriaceae; Mycobacterium; Mycobacterium tuberculosis complex; Mycobacterium tuberculosis;	NCBI: 1448813	4412432	99.94	0.00	Yes
NCBI Assembly	GCA_000669015.1	NCBI: cellular organisms; Bacteria; Terrabacteria group; Actinobacteria; Actinomycetia; Corynebacteriales; Mycobacteriaceae; Mycobacterium; Mycobacterium tuberculosis complex; Mycobacterium tuberculosis;	NCBI: 1408958	4398772	99.94	0.00	Yes
NCBI Assembly	GCA_000667505.1	NCBI: cellular organisms; Bacteria; Terrabacteria group; Actinobacteria; Actinomycetia; Corynebacteriales; Mycobacteriaceae; Mycobacterium; Mycobacterium tuberculosis complex; Mycobacterium tuberculosis;	NCBI: 1354103	4384818	99.90	0.00	Yes
NCBI Assembly	GCA_000665765.1	NCBI: cellular organisms; Bacteria; Terrabacteria group; Actinobacteria; Actinomycetia; Corynebacteriales; Mycobacteriaceae; Mycobacterium; Mycobacterium tuberculosis complex; Mycobacterium tuberculosis;	NCBI: 1448834	4408222	99.94	0.17	Yes
NCBI Assembly	GCA_000663585.1	NCBI: cellular organisms; Bacteria; Terrabacteria group; Actinobacteria; Actinomycetia; Corynebacteriales; Mycobacteriaceae; Mycobacterium; Mycobacterium tuberculosis complex; Mycobacterium tuberculosis;	NCBI: 1448712	4382240	99.94	0.00	Yes

NCBI Assembly	GCA_000665445.1	NCBI: cellular organisms; Bacteria; Terrabacteria group; Actinobacteria; Actinomycetia; Corynebacteriales; Mycobacteriaceae; Mycobacterium; Mycobacterium tuberculosis complex; Mycobacterium tuberculosis;	NCBI: 1448814	4435413	99.94	0.00	Yes
NCBI Assembly	GCA_000669035.1	NCBI: cellular organisms; Bacteria; Terrabacteria group; Actinobacteria; Actinomycetia; Corynebacteriales; Mycobacteriaceae; Mycobacterium; Mycobacterium tuberculosis complex; Mycobacterium tuberculosis;	NCBI: 1408959	4382894	99.94	0.00	Yes
NCBI Assembly	GCA_000665785.1	NCBI: cellular organisms; Bacteria; Terrabacteria group; Actinobacteria; Actinomycetia; Corynebacteriales; Mycobacteriaceae; Mycobacterium; Mycobacterium tuberculosis complex; Mycobacterium tuberculosis;	NCBI: 1448836	4427817	99.94	0.22	Yes
NCBI Assembly	GCA_000667525.1	NCBI: cellular organisms; Bacteria; Terrabacteria group; Actinobacteria; Actinomycetia; Corynebacteriales; Mycobacteriaceae; Mycobacterium; Mycobacterium tuberculosis complex; Mycobacterium tuberculosis; Mycobacterium tuberculosis variant africanum;	NCBI: 1354104	4387100	99.83	0.00	Yes
NCBI Assembly	GCA_000663605.1	NCBI: cellular organisms; Bacteria; Terrabacteria group; Actinobacteria; Actinomycetia; Corynebacteriales; Mycobacteriaceae; Mycobacterium; Mycobacterium tuberculosis complex; Mycobacterium tuberculosis;	NCBI: 1448713	4424164	99.94	0.00	Yes
NCBI Assembly	GCA_000665465.1	NCBI: cellular organisms; Bacteria; Terrabacteria group; Actinobacteria; Actinomycetia; Corynebacteriales; Mycobacteriaceae; Mycobacterium; Mycobacterium tuberculosis complex; Mycobacterium tuberculosis;	NCBI: 1448815	4405304	99.94	0.31	Yes
NCBI Assembly	GCA_000669055.1	NCBI: cellular organisms; Bacteria; Terrabacteria group; Actinobacteria; Actinomycetia; Corynebacteriales; Mycobacteriaceae; Mycobacterium; Mycobacterium tuberculosis complex; Mycobacterium tuberculosis;	NCBI: 1408960	4443983	99.94	0.00	Yes
NCBI Assembly	GCA_000665805.1	NCBI: cellular organisms; Bacteria; Terrabacteria group; Actinobacteria; Actinomycetia; Corynebacteriales; Mycobacteriaceae; Mycobacterium; Mycobacterium tuberculosis complex; Mycobacterium tuberculosis;	NCBI: 1448837	4410612	99.94	0.71	Yes
NCBI Assembly	GCA_000667545.1	NCBI: cellular organisms; Bacteria; Terrabacteria group; Actinobacteria; Actinomycetia; Corynebacteriales; Mycobacteriaceae; Mycobacterium; Mycobacterium tuberculosis complex; Mycobacterium tuberculosis;	NCBI: 1354105	4426285	99.94	0.00	Yes
NCBI Assembly	GCA_000663625.1	NCBI: cellular organisms; Bacteria; Terrabacteria group; Actinobacteria; Actinomycetia; Corynebacteriales; Mycobacteriaceae; Mycobacterium; Mycobacterium tuberculosis complex; Mycobacterium tuberculosis;	NCBI: 1448715	4384985	99.91	0.84	Yes
NCBI Assembly	GCA_000665485.1	NCBI: cellular organisms; Bacteria; Terrabacteria group; Actinobacteria; Actinomycetia; Corynebacteriales; Mycobacteriaceae; Mycobacterium; Mycobacterium tuberculosis complex; Mycobacterium tuberculosis;	NCBI: 1448816	4425402	99.94	0.14	Yes
NCBI Assembly	GCA_000669075.1	NCBI: cellular organisms; Bacteria; Terrabacteria group; Actinobacteria; Actinomycetia; Corynebacteriales; Mycobacteriaceae; Mycobacterium; Mycobacterium tuberculosis complex; Mycobacterium tuberculosis;	NCBI: 1408962	4383260	99.94	0.00	Yes
NCBI Assembly	GCA_000667565.1	NCBI: cellular organisms; Bacteria; Terrabacteria group; Actinobacteria; Actinomycetia; Corynebacteriales; Mycobacteriaceae; Mycobacterium; Mycobacterium tuberculosis complex; Mycobacterium tuberculosis;	NCBI: 1354106	4409196	99.94	0.00	Yes
NCBI Assembly	GCA_000665825.1	NCBI: cellular organisms; Bacteria; Terrabacteria group; Actinobacteria; Actinomycetia; Corynebacteriales; Mycobacteriaceae; Mycobacterium; Mycobacterium tuberculosis complex; Mycobacterium tuberculosis;	NCBI: 1448838	4433135	99.94	1.03	Yes
NCBI Assembly	GCA_000663645.1	NCBI: cellular organisms; Bacteria; Terrabacteria group; Actinobacteria; Actinomycetia; Corynebacteriales; Mycobacteriaceae; Mycobacterium; Mycobacterium tuberculosis complex; Mycobacterium tuberculosis;	NCBI: 1448716	4416839	99.94	0.00	Yes
NCBI Assembly	GCA_000665505.1	NCBI: cellular organisms; Bacteria; Terrabacteria group; Actinobacteria; Actinomycetia; Corynebacteriales; Mycobacteriaceae; Mycobacterium; Mycobacterium tuberculosis complex; Mycobacterium tuberculosis;	NCBI: 1448817	4426112	99.94	0.00	Yes
NCBI Assembly	GCA_000669095.1	NCBI: cellular organisms; Bacteria; Terrabacteria group; Actinobacteria; Actinomycetia; Corynebacteriales; Mycobacteriaceae; Mycobacterium; Mycobacterium tuberculosis complex; Mycobacterium tuberculosis;	NCBI: 1408963	4414868	99.94	0.00	Yes
NCBI Assembly	GCA_000667585.1	NCBI: cellular organisms; Bacteria; Terrabacteria group; Actinobacteria; Actinomycetia; Corynebacteriales; Mycobacteriaceae; Mycobacterium; Mycobacterium tuberculosis complex; Mycobacterium tuberculosis;	NCBI: 1354107	4447860	99.94	0.00	Yes

NCBI Assembly	GCA_000663665.1	NCBI: cellular organisms; Bacteria; Terrabacteria group; Actinobacteria; Actinomycetia; Corynebacteriales; Mycobacteriaceae; Mycobacterium; Mycobacterium tuberculosis complex; Mycobacterium tuberculosis;	NCBI: 1448717	4410848	99.94	0.00	Yes
NCBI Assembly	GCA_000666025.1	NCBI: cellular organisms; Bacteria; Terrabacteria group; Actinobacteria; Actinomycetia; Corynebacteriales; Mycobacteriaceae; Mycobacterium; Mycobacterium tuberculosis complex; Mycobacterium tuberculosis;	NCBI: 1773	4425502	99.94	0.00	Yes
NCBI Assembly	GCA_000665525.1	NCBI: cellular organisms; Bacteria; Terrabacteria group; Actinobacteria; Actinomycetia; Corynebacteriales; Mycobacteriaceae; Mycobacterium; Mycobacterium tuberculosis complex; Mycobacterium tuberculosis;	NCBI: 1448818	4390152	99.94	0.00	Yes
NCBI Assembly	GCA_000667605.1	NCBI: cellular organisms; Bacteria; Terrabacteria group; Actinobacteria; Actinomycetia; Corynebacteriales; Mycobacteriaceae; Mycobacterium; Mycobacterium tuberculosis complex; Mycobacterium tuberculosis;	NCBI: 1354108	4405752	99.94	0.00	Yes
NCBI Assembly	GCA_000663685.1	NCBI: cellular organisms; Bacteria; Terrabacteria group; Actinobacteria; Actinomycetia; Corynebacteriales; Mycobacteriaceae; Mycobacterium; Mycobacterium tuberculosis complex; Mycobacterium tuberculosis;	NCBI: 1448718	4387384	99.94	0.00	Yes
NCBI Assembly	GCA_000666045.1	NCBI: cellular organisms; Bacteria; Terrabacteria group; Actinobacteria; Actinomycetia; Corynebacteriales; Mycobacteriaceae; Mycobacterium; Mycobacterium tuberculosis complex; Mycobacterium tuberculosis;	NCBI: 1773	4427362	99.94	0.07	Yes
NCBI Assembly	GCA_000665545.1	NCBI: cellular organisms; Bacteria; Terrabacteria group; Actinobacteria; Actinomycetia; Corynebacteriales; Mycobacteriaceae; Mycobacterium; Mycobacterium tuberculosis complex; Mycobacterium tuberculosis;	NCBI: 1448819	4440873	99.94	1.17	Yes
NCBI Assembly	GCA_000667625.1	NCBI: cellular organisms; Bacteria; Terrabacteria group; Actinobacteria; Actinomycetia; Corynebacteriales; Mycobacteriaceae; Mycobacterium; Mycobacterium tuberculosis complex; Mycobacterium tuberculosis;	NCBI: 1354109	4413692	99.94	0.00	Yes
NCBI Assembly	GCA_000663705.1	NCBI: cellular organisms; Bacteria; Terrabacteria group; Actinobacteria; Actinomycetia; Corynebacteriales; Mycobacteriaceae; Mycobacterium; Mycobacterium tuberculosis complex; Mycobacterium tuberculosis;	NCBI: 1448719	4419282	99.94	0.19	Yes
NCBI Assembly	GCA_000666065.1	NCBI: cellular organisms; Bacteria; Terrabacteria group; Actinobacteria; Actinomycetia; Corynebacteriales; Mycobacteriaceae; Mycobacterium; Mycobacterium tuberculosis complex; Mycobacterium tuberculosis; Mycobacterium tuberculosis variant africanum;	NCBI: 33894	4394304	99.94	0.00	Yes
NCBI Assembly	GCA_000665565.1	NCBI: cellular organisms; Bacteria; Terrabacteria group; Actinobacteria; Actinomycetia; Corynebacteriales; Mycobacteriaceae; Mycobacterium; Mycobacterium tuberculosis complex; Mycobacterium tuberculosis;	NCBI: 1448821	4409096	99.94	0.78	Yes
NCBI Assembly	GCA_000667645.1	NCBI: cellular organisms; Bacteria; Terrabacteria group; Actinobacteria; Actinomycetia; Corynebacteriales; Mycobacteriaceae; Mycobacterium; Mycobacterium tuberculosis complex; Mycobacterium tuberculosis;	NCBI: 1354110	4390455	99.94	0.00	Yes
NCBI Assembly	GCA_000663725.1	NCBI: cellular organisms; Bacteria; Terrabacteria group; Actinobacteria; Actinomycetia; Corynebacteriales; Mycobacteriaceae; Mycobacterium; Mycobacterium tuberculosis complex; Mycobacterium tuberculosis;	NCBI: 1448720	4386546	99.94	0.00	Yes
NCBI Assembly	GCA_000666085.1	NCBI: cellular organisms; Bacteria; Terrabacteria group; Actinobacteria; Actinomycetia; Corynebacteriales; Mycobacteriaceae; Mycobacterium; Mycobacterium tuberculosis complex; Mycobacterium tuberculosis;	NCBI: 1773	4409573	99.94	0.00	Yes
NCBI Assembly	GCA_000665585.1	NCBI: cellular organisms; Bacteria; Terrabacteria group; Actinobacteria; Actinomycetia; Corynebacteriales; Mycobacteriaceae; Mycobacterium; Mycobacterium tuberculosis complex; Mycobacterium tuberculosis;	NCBI: 1448822	4417639	99.78	0.00	Yes
NCBI Assembly	GCA_000667665.1	NCBI: cellular organisms; Bacteria; Terrabacteria group; Actinobacteria; Actinomycetia; Corynebacteriales; Mycobacteriaceae; Mycobacterium; Mycobacterium tuberculosis complex; Mycobacterium tuberculosis;	NCBI: 1354111	4465239	99.94	0.00	Yes
NCBI Assembly	GCA_000663745.1	NCBI: cellular organisms; Bacteria; Terrabacteria group; Actinobacteria; Actinomycetia; Corynebacteriales; Mycobacteriaceae; Mycobacterium; Mycobacterium tuberculosis complex; Mycobacterium tuberculosis;	NCBI: 1448721	4395239	99.94	0.00	Yes
NCBI Assembly	GCA_000666105.1	NCBI: cellular organisms; Bacteria; Terrabacteria group; Actinobacteria; Actinomycetia; Corynebacteriales; Mycobacteriaceae; Mycobacterium; Mycobacterium tuberculosis complex; Mycobacterium tuberculosis;	NCBI: 1773	4419286	99.94	0.00	Yes

NCBI Assembly	GCA_000665605.1	NCBI: cellular organisms; Bacteria; Terrabacteria group; Actinobacteria; Actinomycetia; Corynebacteriales; Mycobacteriaceae; Mycobacterium; Mycobacterium tuberculosis complex; Mycobacterium tuberculosis;	NCBI: 1448823	4428590	99.94	0.33	Yes
NCBI Assembly	GCA_000667685.1	NCBI: cellular organisms; Bacteria; Terrabacteria group; Actinobacteria; Actinomycetia; Corynebacteriales; Mycobacteriaceae; Mycobacterium; Mycobacterium tuberculosis complex; Mycobacterium tuberculosis;	NCBI: 1354112	4410063	99.94	0.00	Yes
NCBI Assembly	GCA_000663765.1	NCBI: cellular organisms; Bacteria; Terrabacteria group; Actinobacteria; Actinomycetia; Corynebacteriales; Mycobacteriaceae; Mycobacterium; Mycobacterium tuberculosis complex; Mycobacterium tuberculosis;	NCBI: 1448722	4416857	99.94	0.17	Yes
NCBI Assembly	GCA_000665625.1	NCBI: cellular organisms; Bacteria; Terrabacteria group; Actinobacteria; Actinomycetia; Corynebacteriales; Mycobacteriaceae; Mycobacterium; Mycobacterium tuberculosis complex; Mycobacterium tuberculosis;	NCBI: 1448824	4428849	99.94	0.00	Yes
NCBI Assembly	GCA_000666125.1	NCBI: cellular organisms; Bacteria; Terrabacteria group; Actinobacteria; Actinomycetia; Corynebacteriales; Mycobacteriaceae; Mycobacterium; Mycobacterium tuberculosis complex; Mycobacterium tuberculosis; Mycobacterium tuberculosis variant africanum;	NCBI: 33894	4389704	99.94	0.00	Yes
NCBI Assembly	GCA_000667705.1	NCBI: cellular organisms; Bacteria; Terrabacteria group; Actinobacteria; Actinomycetia; Corynebacteriales; Mycobacteriaceae; Mycobacterium; Mycobacterium tuberculosis complex; Mycobacterium tuberculosis;	NCBI: 1354114	4415933	99.94	0.00	Yes
NCBI Assembly	GCA_000663785.1	NCBI: cellular organisms; Bacteria; Terrabacteria group; Actinobacteria; Actinomycetia; Corynebacteriales; Mycobacteriaceae; Mycobacterium; Mycobacterium tuberculosis complex; Mycobacterium tuberculosis;	NCBI: 1448723	4389755	99.94	0.00	Yes
NCBI Assembly	GCA_000665645.1	NCBI: cellular organisms; Bacteria; Terrabacteria group; Actinobacteria; Actinomycetia; Corynebacteriales; Mycobacteriaceae; Mycobacterium; Mycobacterium tuberculosis complex; Mycobacterium tuberculosis;	NCBI: 1448825	4429587	99.94	1.26	Yes
NCBI Assembly	GCA_000666145.1	NCBI: cellular organisms; Bacteria; Terrabacteria group; Actinobacteria; Actinomycetia; Corynebacteriales; Mycobacteriaceae; Mycobacterium; Mycobacterium tuberculosis complex; Mycobacterium tuberculosis;	NCBI: 1773	4413752	99.94	0.00	Yes
NCBI Assembly	GCA_000667725.1	NCBI: cellular organisms; Bacteria; Terrabacteria group; Actinobacteria; Actinomycetia; Corynebacteriales; Mycobacteriaceae; Mycobacterium; Mycobacterium tuberculosis complex; Mycobacterium tuberculosis;	NCBI: 1354115	4423130	99.94	0.17	Yes
NCBI Assembly	GCA_000663805.1	NCBI: cellular organisms; Bacteria; Terrabacteria group; Actinobacteria; Actinomycetia; Corynebacteriales; Mycobacteriaceae; Mycobacterium; Mycobacterium tuberculosis complex; Mycobacterium tuberculosis;	NCBI: 1448724	4415703	99.94	0.00	Yes
NCBI Assembly	GCA_000665665.1	NCBI: cellular organisms; Bacteria; Terrabacteria group; Actinobacteria; Actinomycetia; Corynebacteriales; Mycobacteriaceae; Mycobacterium; Mycobacterium tuberculosis complex; Mycobacterium tuberculosis;	NCBI: 1448826	4421153	99.94	0.42	Yes
NCBI Assembly	GCA_000666165.1	NCBI: cellular organisms; Bacteria; Terrabacteria group; Actinobacteria; Actinomycetia; Corynebacteriales; Mycobacteriaceae; Mycobacterium; Mycobacterium tuberculosis complex; Mycobacterium tuberculosis;	NCBI: 1773	4411899	99.94	0.00	Yes
NCBI Assembly	GCA_000667745.1	NCBI: cellular organisms; Bacteria; Terrabacteria group; Actinobacteria; Actinomycetia; Corynebacteriales; Mycobacteriaceae; Mycobacterium; Mycobacterium tuberculosis complex; Mycobacterium tuberculosis;	NCBI: 1354116	4423729	99.94	0.00	Yes
NCBI Assembly	GCA_000663825.1	NCBI: cellular organisms; Bacteria; Terrabacteria group; Actinobacteria; Actinomycetia; Corynebacteriales; Mycobacteriaceae; Mycobacterium; Mycobacterium tuberculosis complex; Mycobacterium tuberculosis;	NCBI: 1448725	4409840	99.94	0.00	Yes
NCBI Assembly	GCA_000665685.1	NCBI: cellular organisms; Bacteria; Terrabacteria group; Actinobacteria; Actinomycetia; Corynebacteriales; Mycobacteriaceae; Mycobacterium; Mycobacterium tuberculosis complex; Mycobacterium tuberculosis;	NCBI: 1448828	4392060	99.94	0.00	Yes
NCBI Assembly	GCA_000666185.1	NCBI: cellular organisms; Bacteria; Terrabacteria group; Actinobacteria; Actinomycetia; Corynebacteriales; Mycobacteriaceae; Mycobacterium; Mycobacterium tuberculosis complex; Mycobacterium tuberculosis; Mycobacterium tuberculosis variant africanum;	NCBI: 1354161	4384783	99.94	0.00	Yes
NCBI Assembly	GCA_000665705.1	NCBI: cellular organisms; Bacteria; Terrabacteria group; Actinobacteria; Actinomycetia; Corynebacteriales; Mycobacteriaceae; Mycobacterium; Mycobacterium tuberculosis complex; Mycobacterium tuberculosis;	NCBI: 1448829	4418862	99.94	0.00	Yes

NCBI Assembly	GCA_000666205.1	NCBI: cellular organisms; Bacteria; Terrabacteria group; Actinobacteria; Actinomycetia; Corynebacteriales; Mycobacteriaceae; Mycobacterium; Mycobacterium tuberculosis complex; Mycobacterium tuberculosis; Mycobacterium tuberculosis variant africanum;	NCBI: 1354162	4385838	99.94	0.00	Yes
NCBI Assembly	GCA_001051255.1	NCBI: cellular organisms; Bacteria; Terrabacteria group; Actinobacteria; Actinomycetia; Corynebacteriales; Mycobacteriaceae; Mycobacterium; Mycobacterium tuberculosis complex; Mycobacterium tuberculosis;	NCBI: 1773	4362451	99.94	0.00	Yes
NCBI Assembly	GCA_001325665.1	NCBI: cellular organisms; Bacteria; Terrabacteria group; Actinobacteria; Actinomycetia; Corynebacteriales; Mycobacteriaceae; Mycobacterium; Mycobacterium tuberculosis complex; Mycobacterium tuberculosis;	NCBI: 1773	4364261	99.94	0.00	Yes
NCBI Assembly	GCA_001132305.1	NCBI: cellular organisms; Bacteria; Terrabacteria group; Actinobacteria; Actinomycetia; Corynebacteriales; Mycobacteriaceae; Mycobacterium; Mycobacterium tuberculosis complex; Mycobacterium tuberculosis;	NCBI: 1773	4209314	89.60	1.03	No
NCBI Assembly	GCA_001245615.1	NCBI: cellular organisms; Bacteria; Terrabacteria group; Actinobacteria; Actinomycetia; Corynebacteriales; Mycobacteriaceae; Mycolicibacterium; Mycolicibacterium neworleansense;	NCBI: 146018	6287317	98.99	0.25	Yes
NCBI Assembly	GCA_001209825.1	NCBI: cellular organisms; Bacteria; Terrabacteria group; Actinobacteria; Actinomycetia; Corynebacteriales; Mycobacteriaceae; Mycobacterium; Mycobacterium tuberculosis complex; Mycobacterium tuberculosis;	NCBI: 1773	4221127	99.28	2.53	Yes
NCBI Assembly	GCA_001201935.1	NCBI: cellular organisms; Bacteria; Terrabacteria group; Actinobacteria; Actinomycetia; Corynebacteriales; Mycobacteriaceae; Mycobacterium; Mycobacterium tuberculosis complex; Mycobacterium tuberculosis;	NCBI: 1773	4306970	99.61	0.00	Yes
NCBI Assembly	GCA_001376855.1	NCBI: cellular organisms; Bacteria; Terrabacteria group; Actinobacteria; Actinomycetia; Corynebacteriales; Mycobacteriaceae; Mycobacterium; Mycobacterium tuberculosis complex; Mycobacterium tuberculosis;	NCBI: 1773	4357274	99.94	0.00	Yes
NCBI Assembly	GCA_001378115.1	NCBI: cellular organisms; Bacteria; Terrabacteria group; Actinobacteria; Actinomycetia; Corynebacteriales; Mycobacteriaceae; Mycobacterium; Mycobacterium tuberculosis complex; Mycobacterium tuberculosis;	NCBI: 1773	4362305	99.94	0.00	Yes
NCBI Assembly	GCA_001378435.1	NCBI: cellular organisms; Bacteria; Terrabacteria group; Actinobacteria; Actinomycetia; Corynebacteriales; Mycobacteriaceae; Mycobacterium; Mycobacterium tuberculosis complex; Mycobacterium tuberculosis;	NCBI: 1773	4360749	99.94	0.00	Yes
NCBI Assembly	GCA_001174865.1	NCBI: cellular organisms; Bacteria; Terrabacteria group; Actinobacteria; Actinomycetia; Corynebacteriales; Mycobacteriaceae; Mycobacterium; Mycobacterium tuberculosis complex; Mycobacterium tuberculosis;	NCBI: 1773	4244205	97.57	2.22	Yes
NCBI Assembly	GCA_001379675.1	NCBI: cellular organisms; Bacteria; Terrabacteria group; Actinobacteria; Actinomycetia; Corynebacteriales; Mycobacteriaceae; Mycobacterium; Mycobacterium tuberculosis complex; Mycobacterium tuberculosis;	NCBI: 1773	4374453	99.94	0.00	Yes
NCBI Assembly	GCA_001318905.1	NCBI: cellular organisms; Bacteria; Terrabacteria group; Actinobacteria; Actinomycetia; Corynebacteriales; Mycobacteriaceae; Mycobacterium; Mycobacterium tuberculosis complex; Mycobacterium tuberculosis;	NCBI: 1773	3999240	84.99	3.60	No
NCBI Assembly	GCA_001052995.1	NCBI: cellular organisms; Bacteria; Terrabacteria group; Actinobacteria; Actinomycetia; Corynebacteriales; Mycobacteriaceae; Mycolicibacterium; Mycolicibacterium conceptionense;	NCBI: 451644	6388267	93.53	0.47	Yes
NCBI Assembly	GCA_001133005.1	NCBI: cellular organisms; Bacteria; Terrabacteria group; Actinobacteria; Actinomycetia; Corynebacteriales; Mycobacteriaceae; Mycobacterium; Mycobacterium tuberculosis complex; Mycobacterium tuberculosis;	NCBI: 1773	4174730	97.61	1.93	Yes
NCBI Assembly	GCA_001325685.1	NCBI: cellular organisms; Bacteria; Terrabacteria group; Actinobacteria; Actinomycetia; Corynebacteriales; Mycobacteriaceae; Mycobacterium; Mycobacterium tuberculosis complex; Mycobacterium tuberculosis;	NCBI: 1773	4384928	99.94	0.00	Yes
NCBI Assembly	GCA_001379695.1	NCBI: cellular organisms; Bacteria; Terrabacteria group; Actinobacteria; Actinomycetia; Corynebacteriales; Mycobacteriaceae; Mycobacterium; Mycobacterium tuberculosis complex; Mycobacterium tuberculosis;	NCBI: 1773	4369439	99.94	0.00	Yes
NCBI Assembly	GCA_001183005.1	NCBI: cellular organisms; Bacteria; Terrabacteria group; Actinobacteria; Actinomycetia; Corynebacteriales; Mycobacteriaceae; Mycobacterium; Mycobacterium tuberculosis complex; Mycobacterium tuberculosis;	NCBI: 1773	4361922	99.94	0.00	Yes

NCBI Assembly	GCA_001376875.1	NCBI: cellular organisms; Bacteria; Terrabacteria group; Actinobacteria; Actinomycetia; Corynebacteriales; Mycobacteriaceae; Mycobacterium; Mycobacterium tuberculosis complex; Mycobacterium tuberculosis;	NCBI: 1773	4396748	99.94	0.00	Yes
NCBI Assembly	GCA_001202555.1	NCBI: cellular organisms; Bacteria; Terrabacteria group; Actinobacteria; Actinomycetia; Corynebacteriales; Mycobacteriaceae; Mycobacterium; Mycobacterium tuberculosis complex; Mycobacterium tuberculosis;	NCBI: 1773	4321118	99.61	0.00	Yes
NCBI Assembly	GCA_001270365.1	NCBI: cellular organisms; Bacteria; Terrabacteria group; Actinobacteria; Actinomycetia; Corynebacteriales; Mycobacteriaceae; Mycobacterium; Mycobacterium tuberculosis complex; Mycobacterium tuberculosis;	NCBI: 1773	4346549	99.61	0.00	Yes
NCBI Assembly	GCA_001378455.1	NCBI: cellular organisms; Bacteria; Terrabacteria group; Actinobacteria; Actinomycetia; Corynebacteriales; Mycobacteriaceae; Mycobacterium; Mycobacterium tuberculosis complex; Mycobacterium tuberculosis;	NCBI: 1773	4369030	99.94	0.17	Yes
NCBI Assembly	GCA_001209945.1	NCBI: cellular organisms; Bacteria; Terrabacteria group; Actinobacteria; Actinomycetia; Corynebacteriales; Mycobacteriaceae; Mycobacterium; Mycobacterium tuberculosis complex; Mycobacterium tuberculosis;	NCBI: 1773	4320240	99.94	0.00	Yes
NCBI Assembly	GCA_001378135.1	NCBI: cellular organisms; Bacteria; Terrabacteria group; Actinobacteria; Actinomycetia; Corynebacteriales; Mycobacteriaceae; Mycobacterium; Mycobacterium tuberculosis complex; Mycobacterium tuberculosis;	NCBI: 1773	4365873	99.94	0.00	Yes
NCBI Assembly	GCA_001318925.1	NCBI: cellular organisms; Bacteria; Terrabacteria group; Actinobacteria; Actinomycetia; Corynebacteriales; Mycobacteriaceae; Mycobacterium; Mycobacterium tuberculosis complex; Mycobacterium tuberculosis;	NCBI: 1773	4064094	84.33	2.47	No
NCBI Assembly	GCA_001133325.1	NCBI: cellular organisms; Bacteria; Terrabacteria group; Actinobacteria; Actinomycetia; Corynebacteriales; Mycobacteriaceae; Mycobacterium; Mycobacterium tuberculosis complex; Mycobacterium tuberculosis;	NCBI: 1773	4237965	98.84	1.88	Yes
NCBI Assembly	GCA_001183025.1	NCBI: cellular organisms; Bacteria; Terrabacteria group; Actinobacteria; Actinomycetia; Corynebacteriales; Mycobacteriaceae; Mycobacterium; Mycobacterium tuberculosis complex; Mycobacterium tuberculosis;	NCBI: 1773	4368987	99.94	0.00	Yes
NCBI Assembly	GCA_001202635.1	NCBI: cellular organisms; Bacteria; Terrabacteria group; Actinobacteria; Actinomycetia; Corynebacteriales; Mycobacteriaceae; Mycobacterium; Mycobacterium tuberculosis complex; Mycobacterium tuberculosis;	NCBI: 1773	4154177	94.49	0.99	Yes
NCBI Assembly	GCA_001325705.1	NCBI: cellular organisms; Bacteria; Terrabacteria group; Actinobacteria; Actinomycetia; Corynebacteriales; Mycobacteriaceae; Mycobacterium; Mycobacterium tuberculosis complex; Mycobacterium tuberculosis;	NCBI: 1773	4351109	99.94	0.00	Yes
NCBI Assembly	GCA_001209965.1	NCBI: cellular organisms; Bacteria; Terrabacteria group; Actinobacteria; Actinomycetia; Corynebacteriales; Mycobacteriaceae; Mycobacterium; Mycobacterium tuberculosis complex; Mycobacterium tuberculosis;	NCBI: 1773	4283318	98.80	1.88	Yes
NCBI Assembly	GCA_001379715.1	NCBI: cellular organisms; Bacteria; Terrabacteria group; Actinobacteria; Actinomycetia; Corynebacteriales; Mycobacteriaceae; Mycobacterium; Mycobacterium tuberculosis complex; Mycobacterium tuberculosis;	NCBI: 1773	4386477	99.94	0.00	Yes
NCBI Assembly	GCA_001376895.1	NCBI: cellular organisms; Bacteria; Terrabacteria group; Actinobacteria; Actinomycetia; Corynebacteriales; Mycobacteriaceae; Mycobacterium; Mycobacterium tuberculosis complex; Mycobacterium tuberculosis;	NCBI: 1773	4362866	99.94	0.00	Yes
NCBI Assembly	GCA_001378155.1	NCBI: cellular organisms; Bacteria; Terrabacteria group; Actinobacteria; Actinomycetia; Corynebacteriales; Mycobacteriaceae; Mycobacterium; Mycobacterium tuberculosis complex; Mycobacterium tuberculosis;	NCBI: 1773	4362993	99.94	0.00	Yes
NCBI Assembly	GCA_001318945.1	NCBI: cellular organisms; Bacteria; Terrabacteria group; Actinobacteria; Actinomycetia; Corynebacteriales; Mycobacteriaceae; Mycobacterium; Mycobacterium tuberculosis complex; Mycobacterium tuberculosis;	NCBI: 1773	4342013	99.72	0.33	Yes
NCBI Assembly	GCA_001378475.1	NCBI: cellular organisms; Bacteria; Terrabacteria group; Actinobacteria; Actinomycetia; Corynebacteriales; Mycobacteriaceae; Mycobacterium; Mycobacterium tuberculosis complex; Mycobacterium tuberculosis;	NCBI: 1773	4365940	99.94	0.00	Yes
NCBI Assembly	GCA_001077745.1	NCBI: cellular organisms; Bacteria; Terrabacteria group; Actinobacteria; Actinomycetia; Corynebacteriales; Mycobacteriaceae; Mycolicibacterium; Mycolicibacterium conceptionense;	NCBI: 451644	7098887	99.87	0.94	Yes

NCBI Assembly	GCA_001133345.1	NCBI: cellular organisms; Bacteria; Terrabacteria group; Actinobacteria; Actinomycetia; Corynebacteriales; Mycobacteriaceae; Mycobacterium; Mycobacterium tuberculosis complex; Mycobacterium tuberculosis;	NCBI: 1773	4361334	99.94	0.00	Yes
NCBI Assembly	GCA_001325725.1	NCBI: cellular organisms; Bacteria; Terrabacteria group; Actinobacteria; Actinomycetia; Corynebacteriales; Mycobacteriaceae; Mycobacterium; Mycobacterium tuberculosis complex; Mycobacterium tuberculosis;	NCBI: 1773	4355860	99.94	0.00	Yes
NCBI Assembly	GCA_001202675.1	NCBI: cellular organisms; Bacteria; Terrabacteria group; Actinobacteria; Actinomycetia; Corynebacteriales; Mycobacteriaceae; Mycobacterium; Mycobacterium tuberculosis complex; Mycobacterium tuberculosis;	NCBI: 1773	4223827	99.04	1.71	Yes
NCBI Assembly	GCA_001210105.1	NCBI: cellular organisms; Bacteria; Terrabacteria group; Actinobacteria; Actinomycetia; Corynebacteriales; Mycobacteriaceae; Mycobacterium; Mycobacterium tuberculosis complex; Mycobacterium tuberculosis;	NCBI: 1773	4285787	99.61	0.73	Yes
NCBI Assembly	GCA_001183035.1	NCBI: cellular organisms; Bacteria; Terrabacteria group; Actinobacteria; Actinomycetia; Corynebacteriales; Mycobacteriaceae; Mycobacterium; Mycobacterium tuberculosis complex; Mycobacterium tuberculosis;	NCBI: 1773	4362476	99.94	0.00	Yes
NCBI Assembly	GCA_001275565.2	NCBI: cellular organisms; Bacteria; Terrabacteria group; Actinobacteria; Actinomycetia; Corynebacteriales; Mycobacteriaceae; Mycobacterium; Mycobacterium tuberculosis complex; Mycobacterium tuberculosis;	NCBI: 1773	4411829	99.94	0.00	Yes
NCBI Assembly	GCA_001379735.1	NCBI: cellular organisms; Bacteria; Terrabacteria group; Actinobacteria; Actinomycetia; Corynebacteriales; Mycobacteriaceae; Mycobacterium; Mycobacterium tuberculosis complex; Mycobacterium tuberculosis;	NCBI: 1773	4367191	99.61	0.00	Yes
NCBI Assembly	GCA_001318965.1	NCBI: cellular organisms; Bacteria; Terrabacteria group; Actinobacteria; Actinomycetia; Corynebacteriales; Mycobacteriaceae; Mycobacterium; Mycobacterium tuberculosis complex; Mycobacterium tuberculosis;	NCBI: 1773	4323827	99.28	0.00	Yes
NCBI Assembly	GCA_001378175.1	NCBI: cellular organisms; Bacteria; Terrabacteria group; Actinobacteria; Actinomycetia; Corynebacteriales; Mycobacteriaceae; Mycobacterium; Mycobacterium tuberculosis complex; Mycobacterium tuberculosis;	NCBI: 1773	4382038	99.94	0.00	Yes
NCBI Assembly	GCA_001378495.1	NCBI: cellular organisms; Bacteria; Terrabacteria group; Actinobacteria; Actinomycetia; Corynebacteriales; Mycobacteriaceae; Mycobacterium; Mycobacterium tuberculosis complex; Mycobacterium tuberculosis;	NCBI: 1773	4362843	99.94	0.00	Yes
NCBI Assembly	GCA_001376915.1	NCBI: cellular organisms; Bacteria; Terrabacteria group; Actinobacteria; Actinomycetia; Corynebacteriales; Mycobacteriaceae; Mycobacterium; Mycobacterium tuberculosis complex; Mycobacterium tuberculosis;	NCBI: 1773	4362401	99.94	0.00	Yes
NCBI Assembly	GCA_001133985.1	NCBI: cellular organisms; Bacteria; Terrabacteria group; Actinobacteria; Actinomycetia; Corynebacteriales; Mycobacteriaceae; Mycobacterium; Mycobacterium tuberculosis complex; Mycobacterium tuberculosis;	NCBI: 1773	4372399	99.94	0.00	Yes
NCBI Assembly	GCA_001202715.1	NCBI: cellular organisms; Bacteria; Terrabacteria group; Actinobacteria; Actinomycetia; Corynebacteriales; Mycobacteriaceae; Mycobacterium; Mycobacterium tuberculosis complex; Mycobacterium tuberculosis;	NCBI: 1773	4293791	99.61	0.33	Yes
NCBI Assembly	GCA_001210125.1	NCBI: cellular organisms; Bacteria; Terrabacteria group; Actinobacteria; Actinomycetia; Corynebacteriales; Mycobacteriaceae; Mycobacterium; Mycobacterium tuberculosis complex; Mycobacterium tuberculosis;	NCBI: 1773	4259443	99.30	1.72	Yes
NCBI Assembly	GCA_001184735.1	NCBI: cellular organisms; Bacteria; Terrabacteria group; Actinobacteria; Actinomycetia; Corynebacteriales; Mycobacteriaceae; Mycobacterium; Mycobacterium tuberculosis complex; Mycobacterium tuberculosis; Mycobacterium tuberculosis variant bovis;	NCBI: 1765	4295606	99.78	0.00	Yes
NCBI Assembly	GCA_001325745.1	NCBI: cellular organisms; Bacteria; Terrabacteria group; Actinobacteria; Actinomycetia; Corynebacteriales; Mycobacteriaceae; Mycobacterium; Mycobacterium tuberculosis complex; Mycobacterium tuberculosis;	NCBI: 1773	4356457	99.89	0.00	Yes
NCBI Assembly	GCA_001318985.1	NCBI: cellular organisms; Bacteria; Terrabacteria group; Actinobacteria; Actinomycetia; Corynebacteriales; Mycobacteriaceae; Mycobacterium; Mycobacterium tuberculosis complex; Mycobacterium tuberculosis;	NCBI: 1773	4335622	98.88	0.33	Yes
NCBI Assembly	GCA_001282445.1	NCBI: cellular organisms; Bacteria; Terrabacteria group; Actinobacteria; Actinomycetia; Corynebacteriales; Mycobacteriaceae; Mycobacterium; Mycobacterium tuberculosis complex; Mycobacterium tuberculosis;	NCBI: 1773	4354228	99.94	0.67	Yes

NCBI Assembly	GCA_001378515.1	NCBI: cellular organisms; Bacteria; Terrabacteria group; Actinobacteria; Actinomycetia; Corynebacteriales; Mycobacteriaceae; Mycobacterium; Mycobacterium tuberculosis complex; Mycobacterium tuberculosis;	NCBI: 1773	4367193	99.94	0.00	Yes
NCBI Assembly	GCA_001379755.1	NCBI: cellular organisms; Bacteria; Terrabacteria group; Actinobacteria; Actinomycetia; Corynebacteriales; Mycobacteriaceae; Mycobacterium; Mycobacterium tuberculosis complex; Mycobacterium tuberculosis;	NCBI: 1773	4369509	99.94	0.00	Yes
NCBI Assembly	GCA_001376935.1	NCBI: cellular organisms; Bacteria; Terrabacteria group; Actinobacteria; Actinomycetia; Corynebacteriales; Mycobacteriaceae; Mycobacterium; Mycobacterium tuberculosis complex; Mycobacterium tuberculosis;	NCBI: 1773	4363706	99.94	0.00	Yes
NCBI Assembly	GCA_001378195.1	NCBI: cellular organisms; Bacteria; Terrabacteria group; Actinobacteria; Actinomycetia; Corynebacteriales; Mycobacteriaceae; Mycobacterium; Mycobacterium tuberculosis complex; Mycobacterium tuberculosis;	NCBI: 1773	4358352	99.94	0.00	Yes
NCBI Assembly	GCA_001134265.1	NCBI: cellular organisms; Bacteria; Terrabacteria group; Actinobacteria; Actinomycetia; Corynebacteriales; Mycobacteriaceae; Mycobacterium; Mycobacterium tuberculosis complex; Mycobacterium tuberculosis;	NCBI: 1773	4360819	99.94	0.00	Yes
NCBI Assembly	GCA_001202835.1	NCBI: cellular organisms; Bacteria; Terrabacteria group; Actinobacteria; Actinomycetia; Corynebacteriales; Mycobacteriaceae; Mycobacterium; Mycobacterium tuberculosis complex; Mycobacterium tuberculosis;	NCBI: 1773	4264643	99.22	1.73	Yes
NCBI Assembly	GCA_001187505.1	NCBI: cellular organisms; Bacteria; Terrabacteria group; Actinobacteria; Actinomycetia; Corynebacteriales; Mycobacteriaceae; Mycolicibacterium; Mycolicibacterium goodii;	NCBI: 134601	7105933	99.78	3.98	Yes
NCBI Assembly	GCA_001325765.1	NCBI: cellular organisms; Bacteria; Terrabacteria group; Actinobacteria; Actinomycetia; Corynebacteriales; Mycobacteriaceae; Mycobacterium; Mycobacterium tuberculosis complex; Mycobacterium tuberculosis;	NCBI: 1773	4355651	99.94	0.00	Yes
NCBI Assembly	GCA_001210305.1	NCBI: cellular organisms; Bacteria; Terrabacteria group; Actinobacteria; Actinomycetia; Corynebacteriales; Mycobacteriaceae; Mycobacterium; Mycobacterium tuberculosis complex; Mycobacterium tuberculosis;	NCBI: 1773	4296417	99.61	0.00	Yes
NCBI Assembly	GCA_001376955.1	NCBI: cellular organisms; Bacteria; Terrabacteria group; Actinobacteria; Actinomycetia; Corynebacteriales; Mycobacteriaceae; Mycobacterium; Mycobacterium tuberculosis complex; Mycobacterium tuberculosis;	NCBI: 1773	5063968	99.94	5.75	No
NCBI Assembly	GCA_001282455.1	NCBI: cellular organisms; Bacteria; Terrabacteria group; Actinobacteria; Actinomycetia; Corynebacteriales; Mycobacteriaceae; Mycobacterium; Mycobacterium tuberculosis complex; Mycobacterium tuberculosis;	NCBI: 1773	4476887	99.91	3.67	Yes
NCBI Assembly	GCA_001378535.1	NCBI: cellular organisms; Bacteria; Terrabacteria group; Actinobacteria; Actinomycetia; Corynebacteriales; Mycobacteriaceae; Mycobacterium; Mycobacterium tuberculosis complex; Mycobacterium tuberculosis;	NCBI: 1773	4370459	99.94	0.17	Yes
NCBI Assembly	GCA_001319005.1	NCBI: cellular organisms; Bacteria; Terrabacteria group; Actinobacteria; Actinomycetia; Corynebacteriales; Mycobacteriaceae; Mycobacterium; Mycobacterium tuberculosis complex; Mycobacterium tuberculosis;	NCBI: 1773	4291774	99.58	0.00	Yes
NCBI Assembly	GCA_001379775.1	NCBI: cellular organisms; Bacteria; Terrabacteria group; Actinobacteria; Actinomycetia; Corynebacteriales; Mycobacteriaceae; Mycobacterium; Mycobacterium tuberculosis complex; Mycobacterium tuberculosis;	NCBI: 1773	4373383	99.94	0.00	Yes
NCBI Assembly	GCA_001378215.1	NCBI: cellular organisms; Bacteria; Terrabacteria group; Actinobacteria; Actinomycetia; Corynebacteriales; Mycobacteriaceae; Mycobacterium; Mycobacterium tuberculosis complex; Mycobacterium tuberculosis;	NCBI: 1773	4382652	99.94	0.00	Yes
NCBI Assembly	GCA_001083085.1	NCBI: cellular organisms; Bacteria; Terrabacteria group; Actinobacteria; Actinomycetia; Corynebacteriales; Mycobacteriaceae; Mycobacterium; Mycobacterium tuberculosis complex; Mycobacterium tuberculosis;	NCBI: 1773	4362266	99.94	0.00	Yes
NCBI Assembly	GCA_001134885.1	NCBI: cellular organisms; Bacteria; Terrabacteria group; Actinobacteria; Actinomycetia; Corynebacteriales; Mycobacteriaceae; Mycobacterium; Mycobacterium tuberculosis complex; Mycobacterium tuberculosis;	NCBI: 1773	4239654	98.43	3.08	Yes
NCBI Assembly	GCA_001203055.1	NCBI: cellular organisms; Bacteria; Terrabacteria group; Actinobacteria; Actinomycetia; Corynebacteriales; Mycobacteriaceae; Mycobacterium; Mycobacterium tuberculosis complex; Mycobacterium tuberculosis;	NCBI: 1773	4296347	99.12	2.31	Yes

NCBI Assembly	GCA_001191715.1	NCBI: cellular organisms; Bacteria; Terrabacteria group; Actinobacteria; Actinomycetia; Corynebacteriales; Mycobacteriaceae; Mycobacterium; Mycobacterium tuberculosis complex; Mycobacterium tuberculosis;	NCBI: 1773	4293985	99.28	0.00	Yes
NCBI Assembly	GCA_001376975.1	NCBI: cellular organisms; Bacteria; Terrabacteria group; Actinobacteria; Actinomycetia; Corynebacteriales; Mycobacteriaceae; Mycobacterium; Mycobacterium tuberculosis complex; Mycobacterium tuberculosis;	NCBI: 1773	4355282	99.94	0.00	Yes
NCBI Assembly	GCA_001210385.1	NCBI: cellular organisms; Bacteria; Terrabacteria group; Actinobacteria; Actinomycetia; Corynebacteriales; Mycobacteriaceae; Mycobacterium; Mycobacterium tuberculosis complex; Mycobacterium tuberculosis;	NCBI: 1773	4299026	99.61	0.00	Yes
NCBI Assembly	GCA_001319025.1	NCBI: cellular organisms; Bacteria; Terrabacteria group; Actinobacteria; Actinomycetia; Corynebacteriales; Mycobacteriaceae; Mycobacterium; Mycobacterium tuberculosis complex; Mycobacterium tuberculosis;	NCBI: 1773	4318056	99.07	0.33	Yes
NCBI Assembly	GCA_001378555.1	NCBI: cellular organisms; Bacteria; Terrabacteria group; Actinobacteria; Actinomycetia; Corynebacteriales; Mycobacteriaceae; Mycobacterium; Mycobacterium tuberculosis complex; Mycobacterium tuberculosis;	NCBI: 1773	4367521	99.94	0.00	Yes
NCBI Assembly	GCA_001325785.1	NCBI: cellular organisms; Bacteria; Terrabacteria group; Actinobacteria; Actinomycetia; Corynebacteriales; Mycobacteriaceae; Mycobacterium; Mycobacterium tuberculosis complex; Mycobacterium tuberculosis;	NCBI: 1773	4350291	99.94	0.00	Yes
NCBI Assembly	GCA_001282465.1	NCBI: cellular organisms; Bacteria; Terrabacteria group; Actinobacteria; Actinomycetia; Corynebacteriales; Mycobacteriaceae; Mycobacterium; Mycobacterium tuberculosis complex; Mycobacterium tuberculosis;	NCBI: 1773	4456070	99.94	4.33	Yes
NCBI Assembly	GCA_001379795.1	NCBI: cellular organisms; Bacteria; Terrabacteria group; Actinobacteria; Actinomycetia; Corynebacteriales; Mycobacteriaceae; Mycobacterium; Mycobacterium tuberculosis complex; Mycobacterium tuberculosis;	NCBI: 1773	4398242	99.94	0.00	Yes
NCBI Assembly	GCA_001378235.1	NCBI: cellular organisms; Bacteria; Terrabacteria group; Actinobacteria; Actinomycetia; Corynebacteriales; Mycobacteriaceae; Mycobacterium; Mycobacterium tuberculosis complex; Mycobacterium tuberculosis;	NCBI: 1773	4361053	99.94	0.00	Yes
NCBI Assembly	GCA_001083565.1	NCBI: cellular organisms; Bacteria; Terrabacteria group; Actinobacteria; Actinomycetia; Corynebacteriales; Mycobacteriaceae; Mycobacterium; Mycobacterium tuberculosis complex; Mycobacterium tuberculosis;	NCBI: 1773	4365343	99.94	0.00	Yes
NCBI Assembly	GCA_001136885.1	NCBI: cellular organisms; Bacteria; Terrabacteria group; Actinobacteria; Actinomycetia; Corynebacteriales; Mycobacteriaceae; Mycobacterium; Mycobacterium tuberculosis complex; Mycobacterium tuberculosis;	NCBI: 1773	4392329	99.94	0.00	Yes
NCBI Assembly	GCA_001203635.1	NCBI: cellular organisms; Bacteria; Terrabacteria group; Actinobacteria; Actinomycetia; Corynebacteriales; Mycobacteriaceae; Mycobacterium; Mycobacterium tuberculosis complex; Mycobacterium tuberculosis;	NCBI: 1773	4316122	99.94	0.33	Yes
NCBI Assembly	GCA_001191955.1	NCBI: cellular organisms; Bacteria; Terrabacteria group; Actinobacteria; Actinomycetia; Corynebacteriales; Mycobacteriaceae; Mycobacterium; Mycobacterium tuberculosis complex; Mycobacterium tuberculosis;	NCBI: 1773	4285605	99.86	0.73	Yes
NCBI Assembly	GCA_001210505.1	NCBI: cellular organisms; Bacteria; Terrabacteria group; Actinobacteria; Actinomycetia; Corynebacteriales; Mycobacteriaceae; Mycobacterium; Mycobacterium tuberculosis complex; Mycobacterium tuberculosis;	NCBI: 1773	4312908	99.16	0.00	Yes
NCBI Assembly	GCA_001282475.1	NCBI: cellular organisms; Bacteria; Terrabacteria group; Actinobacteria; Actinomycetia; Corynebacteriales; Mycobacteriaceae; Mycobacterium; Mycobacterium tuberculosis complex; Mycobacterium tuberculosis;	NCBI: 1773	4298815	99.14	1.67	Yes
NCBI Assembly	GCA_001376995.1	NCBI: cellular organisms; Bacteria; Terrabacteria group; Actinobacteria; Actinomycetia; Corynebacteriales; Mycobacteriaceae; Mycobacterium; Mycobacterium tuberculosis complex; Mycobacterium tuberculosis;	NCBI: 1773	4395515	99.94	0.00	Yes
NCBI Assembly	GCA_001319045.1	NCBI: cellular organisms; Bacteria; Terrabacteria group; Actinobacteria; Actinomycetia; Corynebacteriales; Mycobacteriaceae; Mycobacterium; Mycobacterium tuberculosis complex; Mycobacterium tuberculosis;	NCBI: 1773	4263692	99.09	2.26	Yes
NCBI Assembly	GCA_001325805.1	NCBI: cellular organisms; Bacteria; Terrabacteria group; Actinobacteria; Actinomycetia; Corynebacteriales; Mycobacteriaceae; Mycobacterium; Mycobacterium tuberculosis complex; Mycobacterium tuberculosis;	NCBI: 1773	4352385	99.94	0.00	Yes

NCBI Assembly	GCA_001378575.1	NCBI: cellular organisms; Bacteria; Terrabacteria group; Actinobacteria; Actinomycetia; Corynebacteriales; Mycobacteriaceae; Mycobacterium; Mycobacterium tuberculosis complex; Mycobacterium tuberculosis;	NCBI: 1773	4367775	99.94	0.00	Yes
NCBI Assembly	GCA_001379815.1	NCBI: cellular organisms; Bacteria; Terrabacteria group; Actinobacteria; Actinomycetia; Corynebacteriales; Mycobacteriaceae; Mycobacterium; Mycobacterium tuberculosis complex; Mycobacterium tuberculosis;	NCBI: 1773	4353304	99.94	0.00	Yes
NCBI Assembly	GCA_001378255.1	NCBI: cellular organisms; Bacteria; Terrabacteria group; Actinobacteria; Actinomycetia; Corynebacteriales; Mycobacteriaceae; Mycobacterium; Mycobacterium tuberculosis complex; Mycobacterium tuberculosis;	NCBI: 1773	4361532	99.94	0.00	Yes
NCBI Assembly	GCA_001084845.1	NCBI: cellular organisms; Bacteria; Terrabacteria group; Actinobacteria; Actinomycetia; Corynebacteriales; Mycobacteriaceae; Mycobacterium; Mycobacterium tuberculosis complex; Mycobacterium tuberculosis;	NCBI: 1773	4292191	99.85	0.00	Yes
NCBI Assembly	GCA_001138485.1	NCBI: cellular organisms; Bacteria; Terrabacteria group; Actinobacteria; Actinomycetia; Corynebacteriales; Mycobacteriaceae; Mycobacterium; Mycobacterium tuberculosis complex; Mycobacterium tuberculosis;	NCBI: 1773	4361056	99.94	0.00	Yes
NCBI Assembly	GCA_001203995.1	NCBI: cellular organisms; Bacteria; Terrabacteria group; Actinobacteria; Actinomycetia; Corynebacteriales; Mycobacteriaceae; Mycobacterium; Mycobacterium tuberculosis complex; Mycobacterium tuberculosis;	NCBI: 1773	4264848	95.69	1.07	Yes
NCBI Assembly	GCA_001192435.1	NCBI: cellular organisms; Bacteria; Terrabacteria group; Actinobacteria; Actinomycetia; Corynebacteriales; Mycobacteriaceae; Mycobacterium; Mycobacterium tuberculosis complex; Mycobacterium tuberculosis;	NCBI: 1773	4308500	99.94	1.51	Yes
NCBI Assembly	GCA_001282525.1	NCBI: cellular organisms; Bacteria; Terrabacteria group; Actinobacteria; Actinomycetia; Corynebacteriales; Mycobacteriaceae; Mycobacterium; Mycobacterium tuberculosis complex; Mycobacterium tuberculosis;	NCBI: 1773	4354187	99.94	0.00	Yes
NCBI Assembly	GCA_001210525.1	NCBI: cellular organisms; Bacteria; Terrabacteria group; Actinobacteria; Actinomycetia; Corynebacteriales; Mycobacteriaceae; Mycobacterium; Mycobacterium tuberculosis complex; Mycobacterium tuberculosis;	NCBI: 1773	4281036	99.86	0.33	Yes
NCBI Assembly	GCA_001377015.1	NCBI: cellular organisms; Bacteria; Terrabacteria group; Actinobacteria; Actinomycetia; Corynebacteriales; Mycobacteriaceae; Mycobacterium; Mycobacterium tuberculosis complex; Mycobacterium tuberculosis;	NCBI: 1773	4389043	99.94	0.00	Yes
NCBI Assembly	GCA_001321665.1	NCBI: cellular organisms; Bacteria; Terrabacteria group; Actinobacteria; Actinomycetia; Corynebacteriales; Mycobacteriaceae; Mycobacterium; Mycobacterium tuberculosis complex; Mycobacterium tuberculosis;	NCBI: 1773	4366208	99.94	0.00	Yes
NCBI Assembly	GCA_001325825.1	NCBI: cellular organisms; Bacteria; Terrabacteria group; Actinobacteria; Actinomycetia; Corynebacteriales; Mycobacteriaceae; Mycobacterium; Mycobacterium tuberculosis complex; Mycobacterium tuberculosis;	NCBI: 1773	4354548	99.94	0.00	Yes
NCBI Assembly	GCA_001378595.1	NCBI: cellular organisms; Bacteria; Terrabacteria group; Actinobacteria; Actinomycetia; Corynebacteriales; Mycobacteriaceae; Mycobacterium; Mycobacterium tuberculosis complex; Mycobacterium tuberculosis;	NCBI: 1773	4362133	99.94	0.00	Yes
NCBI Assembly	GCA_001379835.1	NCBI: cellular organisms; Bacteria; Terrabacteria group; Actinobacteria; Actinomycetia; Corynebacteriales; Mycobacteriaceae; Mycobacterium; Mycobacterium tuberculosis complex; Mycobacterium tuberculosis;	NCBI: 1773	4377531	99.94	0.00	Yes
NCBI Assembly	GCA_001378275.1	NCBI: cellular organisms; Bacteria; Terrabacteria group; Actinobacteria; Actinomycetia; Corynebacteriales; Mycobacteriaceae; Mycobacterium; Mycobacterium tuberculosis complex; Mycobacterium tuberculosis;	NCBI: 1773	4373807	99.94	0.00	Yes
NCBI Assembly	GCA_001140405.1	NCBI: cellular organisms; Bacteria; Terrabacteria group; Actinobacteria; Actinomycetia; Corynebacteriales; Mycobacteriaceae; Mycobacterium; Mycobacterium tuberculosis complex; Mycobacterium tuberculosis;	NCBI: 1773	4305972	99.61	0.00	Yes
NCBI Assembly	GCA_001085125.1	NCBI: cellular organisms; Bacteria; Terrabacteria group; Actinobacteria; Actinomycetia; Corynebacteriales; Mycobacteriaceae; Mycobacterium; Mycobacterium tuberculosis complex; Mycobacterium tuberculosis;	NCBI: 1773	4361156	99.94	0.00	Yes
NCBI Assembly	GCA_001204175.1	NCBI: cellular organisms; Bacteria; Terrabacteria group; Actinobacteria; Actinomycetia; Corynebacteriales; Mycobacteriaceae; Mycobacterium; Mycobacterium tuberculosis complex; Mycobacterium tuberculosis;	NCBI: 1773	4288636	99.94	2.04	Yes

NCBI Assembly	GCA_001282535.1	NCBI: cellular organisms; Bacteria; Terrabacteria group; Actinobacteria; Actinomycetia; Corynebacteriales; Mycobacteriaceae; Mycobacterium; Mycobacterium tuberculosis complex; Mycobacterium tuberculosis;	NCBI: 1773	4351834	99.94	0.67	Yes
NCBI Assembly	GCA_001192515.1	NCBI: cellular organisms; Bacteria; Terrabacteria group; Actinobacteria; Actinomycetia; Corynebacteriales; Mycobacteriaceae; Mycobacterium; Mycobacterium tuberculosis complex; Mycobacterium tuberculosis;	NCBI: 1773	4311365	99.94	0.40	Yes
NCBI Assembly	GCA_001210545.1	NCBI: cellular organisms; Bacteria; Terrabacteria group; Actinobacteria; Actinomycetia; Corynebacteriales; Mycobacteriaceae; Mycobacterium; Mycobacterium tuberculosis complex; Mycobacterium tuberculosis;	NCBI: 1773	4297834	99.66	2.18	Yes
NCBI Assembly	GCA_001321685.1	NCBI: cellular organisms; Bacteria; Terrabacteria group; Actinobacteria; Actinomycetia; Corynebacteriales; Mycobacteriaceae; Mycobacterium; Mycobacterium tuberculosis complex; Mycobacterium tuberculosis;	NCBI: 1773	4354848	99.94	0.00	Yes
NCBI Assembly	GCA_001325845.1	NCBI: cellular organisms; Bacteria; Terrabacteria group; Actinobacteria; Actinomycetia; Corynebacteriales; Mycobacteriaceae; Mycobacterium; Mycobacterium tuberculosis complex; Mycobacterium tuberculosis;	NCBI: 1773	4370294	99.94	0.00	Yes
NCBI Assembly	GCA_001377035.1	NCBI: cellular organisms; Bacteria; Terrabacteria group; Actinobacteria; Actinomycetia; Corynebacteriales; Mycobacteriaceae; Mycobacterium; Mycobacterium tuberculosis complex; Mycobacterium tuberculosis;	NCBI: 1773	4354469	99.94	0.00	Yes
NCBI Assembly	GCA_001378615.1	NCBI: cellular organisms; Bacteria; Terrabacteria group; Actinobacteria; Actinomycetia; Corynebacteriales; Mycobacteriaceae; Mycobacterium; Mycobacterium tuberculosis complex; Mycobacterium tuberculosis;	NCBI: 1773	4365312	99.94	0.00	Yes
NCBI Assembly	GCA_001379855.1	NCBI: cellular organisms; Bacteria; Terrabacteria group; Actinobacteria; Actinomycetia; Corynebacteriales; Mycobacteriaceae; Mycobacterium; Mycobacterium tuberculosis complex; Mycobacterium tuberculosis;	NCBI: 1773	4390223	99.94	0.33	Yes
NCBI Assembly	GCA_001378295.1	NCBI: cellular organisms; Bacteria; Terrabacteria group; Actinobacteria; Actinomycetia; Corynebacteriales; Mycobacteriaceae; Mycobacterium; Mycobacterium tuberculosis complex; Mycobacterium tuberculosis;	NCBI: 1773	4362010	99.94	0.00	Yes
NCBI Assembly	GCA_001086825.1	NCBI: cellular organisms; Bacteria; Terrabacteria group; Actinobacteria; Actinomycetia; Corynebacteriales; Mycobacteriaceae; Mycobacterium; Mycobacterium tuberculosis complex; Mycobacterium tuberculosis;	NCBI: 1773	4365757	99.94	0.13	Yes
NCBI Assembly	GCA_001141505.1	NCBI: cellular organisms; Bacteria; Terrabacteria group; Actinobacteria; Actinomycetia; Corynebacteriales; Mycobacteriaceae; Mycobacterium; Mycobacterium tuberculosis complex; Mycobacterium tuberculosis;	NCBI: 1773	4304123	99.94	0.00	Yes
NCBI Assembly	GCA_001204235.1	NCBI: cellular organisms; Bacteria; Terrabacteria group; Actinobacteria; Actinomycetia; Corynebacteriales; Mycobacteriaceae; Mycobacterium; Mycobacterium tuberculosis complex; Mycobacterium tuberculosis;	NCBI: 1773	4309716	99.94	0.00	Yes
NCBI Assembly	GCA_001192575.1	NCBI: cellular organisms; Bacteria; Terrabacteria group; Actinobacteria; Actinomycetia; Corynebacteriales; Mycobacteriaceae; Mycobacterium; Mycobacterium tuberculosis complex; Mycobacterium tuberculosis;	NCBI: 1773	4200236	97.31	2.61	Yes
NCBI Assembly	GCA_001210665.1	NCBI: cellular organisms; Bacteria; Terrabacteria group; Actinobacteria; Actinomycetia; Corynebacteriales; Mycobacteriaceae; Mycobacterium; Mycobacterium tuberculosis complex; Mycobacterium tuberculosis;	NCBI: 1773	4197101	96.91	3.83	Yes
NCBI Assembly	GCA_001282555.1	NCBI: cellular organisms; Bacteria; Terrabacteria group; Actinobacteria; Actinomycetia; Corynebacteriales; Mycobacteriaceae; Mycobacterium; Mycobacterium tuberculosis complex; Mycobacterium tuberculosis;	NCBI: 1773	4432938	99.94	3.33	Yes
NCBI Assembly	GCA_001321705.1	NCBI: cellular organisms; Bacteria; Terrabacteria group; Actinobacteria; Actinomycetia; Corynebacteriales; Mycobacteriaceae; Mycobacterium; Mycobacterium tuberculosis complex; Mycobacterium tuberculosis;	NCBI: 1773	4362894	99.94	0.00	Yes
NCBI Assembly	GCA_001325865.1	NCBI: cellular organisms; Bacteria; Terrabacteria group; Actinobacteria; Actinomycetia; Corynebacteriales; Mycobacteriaceae; Mycobacterium; Mycobacterium tuberculosis complex; Mycobacterium tuberculosis;	NCBI: 1773	4357796	99.94	0.00	Yes
NCBI Assembly	GCA_001377055.1	NCBI: cellular organisms; Bacteria; Terrabacteria group; Actinobacteria; Actinomycetia; Corynebacteriales; Mycobacteriaceae; Mycobacterium; Mycobacterium tuberculosis complex; Mycobacterium tuberculosis;	NCBI: 1773	4386893	99.94	0.00	Yes

NCBI Assembly	GCA_001378635.1	NCBI: cellular organisms; Bacteria; Terrabacteria group; Actinobacteria; Actinomycetia; Corynebacteriales; Mycobacteriaceae; Mycobacterium; Mycobacterium tuberculosis complex; Mycobacterium tuberculosis;	NCBI: 1773	4362535	99.94	0.00	Yes
NCBI Assembly	GCA_001378315.1	NCBI: cellular organisms; Bacteria; Terrabacteria group; Actinobacteria; Actinomycetia; Corynebacteriales; Mycobacteriaceae; Mycobacterium; Mycobacterium tuberculosis complex; Mycobacterium tuberculosis;	NCBI: 1773	4363771	99.94	0.00	Yes
NCBI Assembly	GCA_001379875.1	NCBI: cellular organisms; Bacteria; Terrabacteria group; Actinobacteria; Actinomycetia; Corynebacteriales; Mycobacteriaceae; Mycobacterium; Mycobacterium tuberculosis complex; Mycobacterium tuberculosis;	NCBI: 1773	4367995	99.94	0.00	Yes
NCBI Assembly	GCA_001086845.1	NCBI: cellular organisms; Bacteria; Terrabacteria group; Actinobacteria; Actinomycetia; Corynebacteriales; Mycobacteriaceae; Mycobacterium; Mycobacterium tuberculosis complex; Mycobacterium tuberculosis;	NCBI: 1773	4095943	92.33	4.80	Yes
NCBI Assembly	GCA_001141625.1	NCBI: cellular organisms; Bacteria; Terrabacteria group; Actinobacteria; Actinomycetia; Corynebacteriales; Mycobacteriaceae; Mycobacterium; Mycobacterium tuberculosis complex; Mycobacterium tuberculosis;	NCBI: 1773	4360813	99.94	0.00	Yes
NCBI Assembly	GCA_001204535.1	NCBI: cellular organisms; Bacteria; Terrabacteria group; Actinobacteria; Actinomycetia; Corynebacteriales; Mycobacteriaceae; Mycobacterium; Mycobacterium tuberculosis complex; Mycobacterium tuberculosis;	NCBI: 1773	4251181	98.93	3.53	Yes
NCBI Assembly	GCA_001193015.1	NCBI: cellular organisms; Bacteria; Terrabacteria group; Actinobacteria; Actinomycetia; Corynebacteriales; Mycobacteriaceae; Mycobacterium; Mycobacterium tuberculosis complex; Mycobacterium tuberculosis;	NCBI: 1773	4292113	99.61	1.00	Yes
NCBI Assembly	GCA_001282575.1	NCBI: cellular organisms; Bacteria; Terrabacteria group; Actinobacteria; Actinomycetia; Corynebacteriales; Mycobacteriaceae; Mycobacterium; Mycobacterium tuberculosis complex; Mycobacterium tuberculosis;	NCBI: 1773	4456700	99.94	3.67	Yes
NCBI Assembly	GCA_001210825.1	NCBI: cellular organisms; Bacteria; Terrabacteria group; Actinobacteria; Actinomycetia; Corynebacteriales; Mycobacteriaceae; Mycobacterium; Mycobacterium tuberculosis complex; Mycobacterium tuberculosis;	NCBI: 1773	4250233	98.91	1.73	Yes
NCBI Assembly	GCA_001321725.1	NCBI: cellular organisms; Bacteria; Terrabacteria group; Actinobacteria; Actinomycetia; Corynebacteriales; Mycobacteriaceae; Mycobacterium; Mycobacterium tuberculosis complex; Mycobacterium tuberculosis;	NCBI: 1773	4362474	99.94	0.00	Yes
NCBI Assembly	GCA_001325885.1	NCBI: cellular organisms; Bacteria; Terrabacteria group; Actinobacteria; Actinomycetia; Corynebacteriales; Mycobacteriaceae; Mycobacterium; Mycobacterium tuberculosis complex; Mycobacterium tuberculosis;	NCBI: 1773	4349205	99.94	0.00	Yes
NCBI Assembly	GCA_001377075.1	NCBI: cellular organisms; Bacteria; Terrabacteria group; Actinobacteria; Actinomycetia; Corynebacteriales; Mycobacteriaceae; Mycobacterium; Mycobacterium tuberculosis complex; Mycobacterium tuberculosis;	NCBI: 1773	4355002	99.94	0.00	Yes
NCBI Assembly	GCA_001378655.1	NCBI: cellular organisms; Bacteria; Terrabacteria group; Actinobacteria; Actinomycetia; Corynebacteriales; Mycobacteriaceae; Mycobacterium; Mycobacterium tuberculosis complex; Mycobacterium tuberculosis;	NCBI: 1773	4369026	99.94	0.00	Yes
NCBI Assembly	GCA_001379895.1	NCBI: cellular organisms; Bacteria; Terrabacteria group; Actinobacteria; Actinomycetia; Corynebacteriales; Mycobacteriaceae; Mycobacterium; Mycobacterium tuberculosis complex; Mycobacterium tuberculosis;	NCBI: 1773	4365333	99.94	0.00	Yes
NCBI Assembly	GCA_001378335.1	NCBI: cellular organisms; Bacteria; Terrabacteria group; Actinobacteria; Actinomycetia; Corynebacteriales; Mycobacteriaceae; Mycobacterium; Mycobacterium tuberculosis complex; Mycobacterium tuberculosis;	NCBI: 1773	4361519	99.94	0.00	Yes
NCBI Assembly	GCA_001086985.1	NCBI: cellular organisms; Bacteria; Terrabacteria group; Actinobacteria; Actinomycetia; Corynebacteriales; Mycobacteriaceae; Mycobacterium; Mycobacterium tuberculosis complex; Mycobacterium tuberculosis;	NCBI: 1773	4372294	99.94	0.00	Yes
NCBI Assembly	GCA_001141705.1	NCBI: cellular organisms; Bacteria; Terrabacteria group; Actinobacteria; Actinomycetia; Corynebacteriales; Mycobacteriaceae; Mycobacterium; Mycobacterium tuberculosis complex; Mycobacterium tuberculosis;	NCBI: 1773	4405066	99.94	0.00	Yes
NCBI Assembly	GCA_001204575.1	NCBI: cellular organisms; Bacteria; Terrabacteria group; Actinobacteria; Actinomycetia; Corynebacteriales; Mycobacteriaceae; Mycobacterium; Mycobacterium tuberculosis complex; Mycobacterium tuberculosis;	NCBI: 1773	4262314	99.86	0.00	Yes

NCBI Assembly	GCA_001193075.1	NCBI: cellular organisms; Bacteria; Terrabacteria group; Actinobacteria; Actinomycetia; Corynebacteriales; Mycobacteriaceae; Mycobacterium; Mycobacterium tuberculosis complex; Mycobacterium tuberculosis;	NCBI: 1773	4322988	98.94	0.33	Yes
NCBI Assembly	GCA_001287005.1	NCBI: cellular organisms; Bacteria; Terrabacteria group; Actinobacteria; Actinomycetia; Corynebacteriales; Mycobacteriaceae; Mycobacterium; Mycobacterium tuberculosis complex; Mycobacterium tuberculosis; Mycobacterium tuberculosis variant bovis; [illegible]	NCBI: 33892	4311781	99.78	0.11	Yes
NCBI Assembly	GCA_001211105.1	NCBI: cellular organisms; Bacteria; Terrabacteria group; Actinobacteria; Actinomycetia; Corynebacteriales; Mycobacteriaceae; Mycobacterium; Mycobacterium tuberculosis complex; Mycobacterium tuberculosis;	NCBI: 1773	4308582	99.28	1.07	Yes
NCBI Assembly	GCA_001321745.1	NCBI: cellular organisms; Bacteria; Terrabacteria group; Actinobacteria; Actinomycetia; Corynebacteriales; Mycobacteriaceae; Mycobacterium; Mycobacterium tuberculosis complex; Mycobacterium tuberculosis;	NCBI: 1773	4368929	99.94	0.00	Yes
NCBI Assembly	GCA_001325905.1	NCBI: cellular organisms; Bacteria; Terrabacteria group; Actinobacteria; Actinomycetia; Corynebacteriales; Mycobacteriaceae; Mycobacterium; Mycobacterium tuberculosis complex; Mycobacterium tuberculosis;	NCBI: 1773	4356409	99.94	0.11	Yes
NCBI Assembly	GCA_001377095.1	NCBI: cellular organisms; Bacteria; Terrabacteria group; Actinobacteria; Actinomycetia; Corynebacteriales; Mycobacteriaceae; Mycobacterium; Mycobacterium tuberculosis complex; Mycobacterium tuberculosis;	NCBI: 1773	4349310	99.94	0.00	Yes
NCBI Assembly	GCA_001378675.1	NCBI: cellular organisms; Bacteria; Terrabacteria group; Actinobacteria; Actinomycetia; Corynebacteriales; Mycobacteriaceae; Mycobacterium; Mycobacterium tuberculosis complex; Mycobacterium tuberculosis;	NCBI: 1773	4370369	99.94	0.17	Yes
NCBI Assembly	GCA_001379915.1	NCBI: cellular organisms; Bacteria; Terrabacteria group; Actinobacteria; Actinomycetia; Corynebacteriales; Mycobacteriaceae; Mycobacterium; Mycobacterium tuberculosis complex; Mycobacterium tuberculosis;	NCBI: 1773	4368761	99.94	0.00	Yes
NCBI Assembly	GCA_001088145.1	NCBI: cellular organisms; Bacteria; Terrabacteria group; Actinobacteria; Actinomycetia; Corynebacteriales; Mycobacteriaceae; Mycobacterium; Mycobacterium tuberculosis complex; Mycobacterium tuberculosis;	NCBI: 1773	4017482	89.94	5.89	No
NCBI Assembly	GCA_001378355.1	NCBI: cellular organisms; Bacteria; Terrabacteria group; Actinobacteria; Actinomycetia; Corynebacteriales; Mycobacteriaceae; Mycobacterium; Mycobacterium tuberculosis complex; Mycobacterium tuberculosis;	NCBI: 1773	4365222	99.94	0.00	Yes
NCBI Assembly	GCA_001142645.1	NCBI: cellular organisms; Bacteria; Terrabacteria group; Actinobacteria; Actinomycetia; Corynebacteriales; Mycobacteriaceae; Mycobacterium; Mycobacterium tuberculosis complex; Mycobacterium tuberculosis;	NCBI: 1773	4374685	99.94	0.00	Yes
NCBI Assembly	GCA_001204615.1	NCBI: cellular organisms; Bacteria; Terrabacteria group; Actinobacteria; Actinomycetia; Corynebacteriales; Mycobacteriaceae; Mycobacterium; Mycobacterium tuberculosis complex; Mycobacterium tuberculosis;	NCBI: 1773	3590067	65.76	6.51	No
NCBI Assembly	GCA_001193195.1	NCBI: cellular organisms; Bacteria; Terrabacteria group; Actinobacteria; Actinomycetia; Corynebacteriales; Mycobacteriaceae; Mycobacterium; Mycobacterium tuberculosis complex; Mycobacterium tuberculosis;	NCBI: 1773	4252453	98.70	2.33	Yes
NCBI Assembly	GCA_001287045.1	NCBI: cellular organisms; Bacteria; Terrabacteria group; Actinobacteria; Actinomycetia; Corynebacteriales; Mycobacteriaceae; Mycobacterium; Mycobacterium tuberculosis complex; Mycobacterium tuberculosis; Mycobacterium tuberculosis variant bovis; [illegible]	NCBI: 33892	4284346	99.78	0.33	Yes
NCBI Assembly	GCA_001211145.1	NCBI: cellular organisms; Bacteria; Terrabacteria group; Actinobacteria; Actinomycetia; Corynebacteriales; Mycobacteriaceae; Mycobacterium; Mycobacterium tuberculosis complex; Mycobacterium tuberculosis;	NCBI: 1773	3992249	73.34	2.62	No
NCBI Assembly	GCA_001321765.1	NCBI: cellular organisms; Bacteria; Terrabacteria group; Actinobacteria; Actinomycetia; Corynebacteriales; Mycobacteriaceae; Mycobacterium; Mycobacterium tuberculosis complex; Mycobacterium tuberculosis;	NCBI: 1773	4357967	99.94	0.00	Yes
NCBI Assembly	GCA_001325925.1	NCBI: cellular organisms; Bacteria; Terrabacteria group; Actinobacteria; Actinomycetia; Corynebacteriales; Mycobacteriaceae; Mycobacterium; Mycobacterium tuberculosis complex; Mycobacterium tuberculosis;	NCBI: 1773	4357797	99.94	0.00	Yes
NCBI Assembly	GCA_001377115.1	NCBI: cellular organisms; Bacteria; Terrabacteria group; Actinobacteria; Actinomycetia; Corynebacteriales; Mycobacteriaceae; Mycobacterium; Mycobacterium tuberculosis complex; Mycobacterium tuberculosis;	NCBI: 1773	4363503	99.94	0.00	Yes

NCBI Assembly	GCA_001379935.1	NCBI: cellular organisms; Bacteria; Terrabacteria group; Actinobacteria; Actinomycetia; Corynebacteriales; Mycobacteriaceae; Mycobacterium; Mycobacterium tuberculosis complex; Mycobacterium tuberculosis;	NCBI: 1773	4365163	99.94	0.00	Yes
NCBI Assembly	GCA_001378695.1	NCBI: cellular organisms; Bacteria; Terrabacteria group; Actinobacteria; Actinomycetia; Corynebacteriales; Mycobacteriaceae; Mycobacterium; Mycobacterium tuberculosis complex; Mycobacterium tuberculosis;	NCBI: 1773	4366148	99.94	0.00	Yes
NCBI Assembly	GCA_001088225.1	NCBI: cellular organisms; Bacteria; Terrabacteria group; Actinobacteria; Actinomycetia; Corynebacteriales; Mycobacteriaceae; Mycobacterium; Mycobacterium tuberculosis complex; Mycobacterium tuberculosis;	NCBI: 1773	4354480	99.94	0.00	Yes
NCBI Assembly	GCA_001143165.1	NCBI: cellular organisms; Bacteria; Terrabacteria group; Actinobacteria; Actinomycetia; Corynebacteriales; Mycobacteriaceae; Mycobacterium; Mycobacterium tuberculosis complex; Mycobacterium tuberculosis;	NCBI: 1773	4364350	99.94	0.00	Yes
NCBI Assembly	GCA_001378375.1	NCBI: cellular organisms; Bacteria; Terrabacteria group; Actinobacteria; Actinomycetia; Corynebacteriales; Mycobacteriaceae; Mycobacterium; Mycobacterium tuberculosis complex; Mycobacterium tuberculosis;	NCBI: 1773	4363119	99.94	0.00	Yes
NCBI Assembly	GCA_001204695.1	NCBI: cellular organisms; Bacteria; Terrabacteria group; Actinobacteria; Actinomycetia; Corynebacteriales; Mycobacteriaceae; Mycobacterium; Mycobacterium tuberculosis complex; Mycobacterium tuberculosis;	NCBI: 1773	4282421	99.91	1.00	Yes
NCBI Assembly	GCA_001193775.1	NCBI: cellular organisms; Bacteria; Terrabacteria group; Actinobacteria; Actinomycetia; Corynebacteriales; Mycobacteriaceae; Mycobacterium; Mycobacterium tuberculosis complex; Mycobacterium tuberculosis;	NCBI: 1773	4302668	99.61	0.33	Yes
NCBI Assembly	GCA_001287065.1	NCBI: cellular organisms; Bacteria; Terrabacteria group; Actinobacteria; Actinomycetia; Corynebacteriales; Mycobacteriaceae; Mycobacterium; Mycobacterium tuberculosis complex; Mycobacterium tuberculosis; Mycobacterium tuberculosis variant bovis; [illegible]	NCBI: 33892	4306135	99.78	0.00	Yes
NCBI Assembly	GCA_001211205.1	NCBI: cellular organisms; Bacteria; Terrabacteria group; Actinobacteria; Actinomycetia; Corynebacteriales; Mycobacteriaceae; Mycobacterium; Mycobacterium tuberculosis complex; Mycobacterium tuberculosis;	NCBI: 1773	4317014	98.94	0.33	Yes
NCBI Assembly	GCA_001321785.1	NCBI: cellular organisms; Bacteria; Terrabacteria group; Actinobacteria; Actinomycetia; Corynebacteriales; Mycobacteriaceae; Mycobacterium; Mycobacterium tuberculosis complex; Mycobacterium tuberculosis;	NCBI: 1773	4363145	99.94	0.00	Yes
NCBI Assembly	GCA_001325945.1	NCBI: cellular organisms; Bacteria; Terrabacteria group; Actinobacteria; Actinomycetia; Corynebacteriales; Mycobacteriaceae; Mycobacterium; Mycobacterium tuberculosis complex; Mycobacterium tuberculosis;	NCBI: 1773	4352808	99.94	0.00	Yes
NCBI Assembly	GCA_001377135.1	NCBI: cellular organisms; Bacteria; Terrabacteria group; Actinobacteria; Actinomycetia; Corynebacteriales; Mycobacteriaceae; Mycobacterium; Mycobacterium tuberculosis complex; Mycobacterium tuberculosis;	NCBI: 1773	4355755	99.94	0.00	Yes
NCBI Assembly	GCA_001088285.1	NCBI: cellular organisms; Bacteria; Terrabacteria group; Actinobacteria; Actinomycetia; Corynebacteriales; Mycobacteriaceae; Mycobacterium; Mycobacterium tuberculosis complex; Mycobacterium tuberculosis;	NCBI: 1773	4272343	99.28	0.36	Yes
NCBI Assembly	GCA_001379955.1	NCBI: cellular organisms; Bacteria; Terrabacteria group; Actinobacteria; Actinomycetia; Corynebacteriales; Mycobacteriaceae; Mycobacterium; Mycobacterium tuberculosis complex; Mycobacterium tuberculosis;	NCBI: 1773	4364647	99.94	0.00	Yes
NCBI Assembly	GCA_001378715.1	NCBI: cellular organisms; Bacteria; Terrabacteria group; Actinobacteria; Actinomycetia; Corynebacteriales; Mycobacteriaceae; Mycobacterium; Mycobacterium tuberculosis complex; Mycobacterium tuberculosis;	NCBI: 1773	4384266	99.94	0.00	Yes
NCBI Assembly	GCA_001143545.1	NCBI: cellular organisms; Bacteria; Terrabacteria group; Actinobacteria; Actinomycetia; Corynebacteriales; Mycobacteriaceae; Mycobacterium; Mycobacterium tuberculosis complex; Mycobacterium tuberculosis;	NCBI: 1773	4361494	99.94	0.17	Yes
NCBI Assembly	GCA_001378395.1	NCBI: cellular organisms; Bacteria; Terrabacteria group; Actinobacteria; Actinomycetia; Corynebacteriales; Mycobacteriaceae; Mycobacterium; Mycobacterium tuberculosis complex; Mycobacterium tuberculosis;	NCBI: 1773	4373682	99.94	0.00	Yes
NCBI Assembly	GCA_001204715.1	NCBI: cellular organisms; Bacteria; Terrabacteria group; Actinobacteria; Actinomycetia; Corynebacteriales; Mycobacteriaceae; Mycobacterium; Mycobacterium tuberculosis complex; Mycobacterium tuberculosis;	NCBI: 1773	4273379	99.66	1.61	Yes

NCBI Assembly	GCA_001193955.1	NCBI: cellular organisms; Bacteria; Terrabacteria group; Actinobacteria; Actinomycetia; Corynebacteriales; Mycobacteriaceae; Mycobacterium; Mycobacterium tuberculosis complex; Mycobacterium tuberculosis;	NCBI: 1773	4247577	99.61	0.00	Yes
NCBI Assembly	GCA_001287105.1	NCBI: cellular organisms; Bacteria; Terrabacteria group; Actinobacteria; Actinomycetia; Corynebacteriales; Mycobacteriaceae; Mycobacterium; Mycobacterium tuberculosis complex; Mycobacterium tuberculosis; Mycobacterium tuberculosis variant bovis; Mycobacterium tuberculosis variant bovis BCG;	NCBI: 33892	4307175	99.78	0.00	Yes
NCBI Assembly	GCA_001211425.1	NCBI: cellular organisms; Bacteria; Terrabacteria group; Actinobacteria; Actinomycetia; Corynebacteriales; Mycobacteriaceae; Mycobacterium; Mycobacterium tuberculosis complex; Mycobacterium tuberculosis;	NCBI: 1773	4301248	99.28	1.04	Yes
NCBI Assembly	GCA_001321805.1	NCBI: cellular organisms; Bacteria; Terrabacteria group; Actinobacteria; Actinomycetia; Corynebacteriales; Mycobacteriaceae; Mycobacterium; Mycobacterium tuberculosis complex; Mycobacterium tuberculosis;	NCBI: 1773	4350255	99.94	0.00	Yes
NCBI Assembly	GCA_001325965.1	NCBI: cellular organisms; Bacteria; Terrabacteria group; Actinobacteria; Actinomycetia; Corynebacteriales; Mycobacteriaceae; Mycobacterium; Mycobacterium tuberculosis complex; Mycobacterium tuberculosis;	NCBI: 1773	4356714	99.94	0.00	Yes
NCBI Assembly	GCA_001021505.1	NCBI: cellular organisms; Bacteria; Terrabacteria group; Actinobacteria; Actinomycetia; Corynebacteriales; Mycobacteriaceae; Mycolicibacter; Mycolicibacter heraklionensis;	NCBI: 512402	5109749	99.55	1.06	Yes
NCBI Assembly	GCA_001377155.1	NCBI: cellular organisms; Bacteria; Terrabacteria group; Actinobacteria; Actinomycetia; Corynebacteriales; Mycobacteriaceae; Mycobacterium; Mycobacterium tuberculosis complex; Mycobacterium tuberculosis;	NCBI: 1773	4364341	99.94	0.00	Yes
NCBI Assembly	GCA_001379975.1	NCBI: cellular organisms; Bacteria; Terrabacteria group; Actinobacteria; Actinomycetia; Corynebacteriales; Mycobacteriaceae; Mycobacterium; Mycobacterium tuberculosis complex; Mycobacterium tuberculosis;	NCBI: 1773	4368219	99.94	0.17	Yes
NCBI Assembly	GCA_001378735.1	NCBI: cellular organisms; Bacteria; Terrabacteria group; Actinobacteria; Actinomycetia; Corynebacteriales; Mycobacteriaceae; Mycobacterium; Mycobacterium tuberculosis complex; Mycobacterium tuberculosis;	NCBI: 1773	4360945	99.94	0.33	Yes
NCBI Assembly	GCA_001378415.1	NCBI: cellular organisms; Bacteria; Terrabacteria group; Actinobacteria; Actinomycetia; Corynebacteriales; Mycobacteriaceae; Mycobacterium; Mycobacterium tuberculosis complex; Mycobacterium tuberculosis;	NCBI: 1773	4351663	99.94	0.00	Yes
NCBI Assembly	GCA_001144065.1	NCBI: cellular organisms; Bacteria; Terrabacteria group; Actinobacteria; Actinomycetia; Corynebacteriales; Mycobacteriaceae; Mycobacterium; Mycobacterium tuberculosis complex; Mycobacterium tuberculosis;	NCBI: 1773	4095150	93.14	4.70	Yes
NCBI Assembly	GCA_001204895.1	NCBI: cellular organisms; Bacteria; Terrabacteria group; Actinobacteria; Actinomycetia; Corynebacteriales; Mycobacteriaceae; Mycobacterium; Mycobacterium tuberculosis complex; Mycobacterium tuberculosis;	NCBI: 1773	4320135	99.61	0.33	Yes
NCBI Assembly	GCA_001194055.1	NCBI: cellular organisms; Bacteria; Terrabacteria group; Actinobacteria; Actinomycetia; Corynebacteriales; Mycobacteriaceae; Mycobacterium; Mycobacterium tuberculosis complex; Mycobacterium tuberculosis;	NCBI: 1773	4313915	99.44	0.33	Yes
NCBI Assembly	GCA_001211465.1	NCBI: cellular organisms; Bacteria; Terrabacteria group; Actinobacteria; Actinomycetia; Corynebacteriales; Mycobacteriaceae; Mycobacterium; Mycobacterium tuberculosis complex; Mycobacterium tuberculosis;	NCBI: 1773	4327355	99.94	0.00	Yes
NCBI Assembly	GCA_001207985.1	NCBI: cellular organisms; Bacteria; Terrabacteria group; Actinobacteria; Actinomycetia; Corynebacteriales; Mycobacteriaceae; Mycobacterium; Mycobacterium tuberculosis complex; Mycobacterium tuberculosis;	NCBI: 1773	4262908	98.99	2.32	Yes
NCBI Assembly	GCA_001318585.1	NCBI: cellular organisms; Bacteria; Terrabacteria group; Actinobacteria; Actinomycetia; Corynebacteriales; Mycobacteriaceae; Mycobacterium; Mycobacterium tuberculosis complex; Mycobacterium tuberculosis;	NCBI: 1773	4293901	99.50	0.40	Yes
NCBI Assembly	GCA_001325985.1	NCBI: cellular organisms; Bacteria; Terrabacteria group; Actinobacteria; Actinomycetia; Corynebacteriales; Mycobacteriaceae; Mycobacterium; Mycobacterium tuberculosis complex; Mycobacterium tuberculosis;	NCBI: 1773	4375673	99.61	0.00	Yes
NCBI Assembly	GCA_001378755.1	NCBI: cellular organisms; Bacteria; Terrabacteria group; Actinobacteria; Actinomycetia; Corynebacteriales; Mycobacteriaceae; Mycobacterium; Mycobacterium tuberculosis complex; Mycobacterium tuberculosis;	NCBI: 1773	4365132	99.94	0.00	Yes

NCBI Assembly	GCA_001376555.1	NCBI: cellular organisms; Bacteria; Terrabacteria group; Actinobacteria; Actinomycetia; Corynebacteriales; Mycobacteriaceae; Mycobacterium; Mycobacterium tuberculosis complex; Mycobacterium tuberculosis;	NCBI: 1773	4355042	99.94	0.00	Yes
NCBI Assembly	GCA_001379995.1	NCBI: cellular organisms; Bacteria; Terrabacteria group; Actinobacteria; Actinomycetia; Corynebacteriales; Mycobacteriaceae; Mycobacterium; Mycobacterium tuberculosis complex; Mycobacterium tuberculosis;	NCBI: 1773	4367354	99.94	0.17	Yes
NCBI Assembly	GCA_001144145.1	NCBI: cellular organisms; Bacteria; Terrabacteria group; Actinobacteria; Actinomycetia; Corynebacteriales; Mycobacteriaceae; Mycobacterium; Mycobacterium tuberculosis complex; Mycobacterium tuberculosis;	NCBI: 1773	4359262	99.94	0.00	Yes
NCBI Assembly	GCA_001205115.1	NCBI: cellular organisms; Bacteria; Terrabacteria group; Actinobacteria; Actinomycetia; Corynebacteriales; Mycobacteriaceae; Mycobacterium; Mycobacterium tuberculosis complex; Mycobacterium tuberculosis;	NCBI: 1773	4960060	99.94	7.94	No
NCBI Assembly	GCA_001377815.1	NCBI: cellular organisms; Bacteria; Terrabacteria group; Actinobacteria; Actinomycetia; Corynebacteriales; Mycobacteriaceae; Mycobacterium; Mycobacterium tuberculosis complex; Mycobacterium tuberculosis;	NCBI: 1773	4380819	99.94	0.00	Yes
NCBI Assembly	GCA_001194365.1	NCBI: cellular organisms; Bacteria; Terrabacteria group; Actinobacteria; Actinomycetia; Corynebacteriales; Mycobacteriaceae; Mycobacterium; Mycobacterium tuberculosis complex; Mycobacterium tuberculosis;	NCBI: 1773	4171409	89.24	1.52	No
NCBI Assembly	GCA_001211585.1	NCBI: cellular organisms; Bacteria; Terrabacteria group; Actinobacteria; Actinomycetia; Corynebacteriales; Mycobacteriaceae; Mycobacterium; Mycobacterium tuberculosis complex; Mycobacterium tuberculosis;	NCBI: 1773	4240870	96.50	1.84	Yes
NCBI Assembly	GCA_001208125.1	NCBI: cellular organisms; Bacteria; Terrabacteria group; Actinobacteria; Actinomycetia; Corynebacteriales; Mycobacteriaceae; Mycobacterium; Mycobacterium tuberculosis complex; Mycobacterium tuberculosis;	NCBI: 1773	4136837	91.19	1.93	Yes
NCBI Assembly	GCA_001318605.1	NCBI: cellular organisms; Bacteria; Terrabacteria group; Actinobacteria; Actinomycetia; Corynebacteriales; Mycobacteriaceae; Mycobacterium; Mycobacterium tuberculosis complex; Mycobacterium tuberculosis;	NCBI: 1773	4310350	99.94	0.00	Yes
NCBI Assembly	GCA_001326005.1	NCBI: cellular organisms; Bacteria; Terrabacteria group; Actinobacteria; Actinomycetia; Corynebacteriales; Mycobacteriaceae; Mycobacterium; Mycobacterium tuberculosis complex; Mycobacterium tuberculosis;	NCBI: 1773	4351378	99.91	0.00	Yes
NCBI Assembly	GCA_001376575.1	NCBI: cellular organisms; Bacteria; Terrabacteria group; Actinobacteria; Actinomycetia; Corynebacteriales; Mycobacteriaceae; Mycobacterium; Mycobacterium tuberculosis complex; Mycobacterium tuberculosis;	NCBI: 1773	4360482	99.94	0.00	Yes
NCBI Assembly	GCA_001378775.1	NCBI: cellular organisms; Bacteria; Terrabacteria group; Actinobacteria; Actinomycetia; Corynebacteriales; Mycobacteriaceae; Mycobacterium; Mycobacterium tuberculosis complex; Mycobacterium tuberculosis;	NCBI: 1773	4366460	99.94	0.00	Yes
NCBI Assembly	GCA_001044235.1	NCBI: cellular organisms; Bacteria; Terrabacteria group; Actinobacteria; Actinomycetia; Corynebacteriales; Mycobacteriaceae; Mycolicibacterium; Mycolicibacterium chlorophenolicum;	NCBI: 37916	7379520	100.00	1.12	Yes
NCBI Assembly	GCA_001144605.1	NCBI: cellular organisms; Bacteria; Terrabacteria group; Actinobacteria; Actinomycetia; Corynebacteriales; Mycobacteriaceae; Mycobacterium; Mycobacterium tuberculosis complex; Mycobacterium tuberculosis;	NCBI: 1773	4358353	99.94	0.17	Yes
NCBI Assembly	GCA_001380015.1	NCBI: cellular organisms; Bacteria; Terrabacteria group; Actinobacteria; Actinomycetia; Corynebacteriales; Mycobacteriaceae; Mycobacterium; Mycobacterium tuberculosis complex; Mycobacterium tuberculosis;	NCBI: 1773	4366780	99.94	0.00	Yes
NCBI Assembly	GCA_001205215.1	NCBI: cellular organisms; Bacteria; Terrabacteria group; Actinobacteria; Actinomycetia; Corynebacteriales; Mycobacteriaceae; Mycobacterium; Mycobacterium tuberculosis complex; Mycobacterium tuberculosis;	NCBI: 1773	4324083	99.11	1.11	Yes
NCBI Assembly	GCA_001194425.1	NCBI: cellular organisms; Bacteria; Terrabacteria group; Actinobacteria; Actinomycetia; Corynebacteriales; Mycobacteriaceae; Mycobacterium; Mycobacterium tuberculosis complex; Mycobacterium tuberculosis;	NCBI: 1773	4322729	99.61	0.40	Yes
NCBI Assembly	GCA_001377835.1	NCBI: cellular organisms; Bacteria; Terrabacteria group; Actinobacteria; Actinomycetia; Corynebacteriales; Mycobacteriaceae; Mycobacterium; Mycobacterium tuberculosis complex; Mycobacterium tuberculosis;	NCBI: 1773	4358532	99.94	0.00	Yes

NCBI Assembly	GCA_001211605.1	NCBI: cellular organisms; Bacteria; Terrabacteria group; Actinobacteria; Actinomycetia; Corynebacteriales; Mycobacteriaceae; Mycobacterium; Mycobacterium tuberculosis complex; Mycobacterium tuberculosis;	NCBI: 1773	4287927	99.12	1.41	Yes
NCBI Assembly	GCA_001318625.1	NCBI: cellular organisms; Bacteria; Terrabacteria group; Actinobacteria; Actinomycetia; Corynebacteriales; Mycobacteriaceae; Mycobacterium; Mycobacterium tuberculosis complex; Mycobacterium tuberculosis;	NCBI: 1773	4278049	99.61	0.00	Yes
NCBI Assembly	GCA_001044245.1	NCBI: cellular organisms; Bacteria; Terrabacteria group; Actinobacteria; Actinomycetia; Corynebacteriales; Mycobacteriaceae; Mycolicibacterium; Mycolicibacterium obuense;	NCBI: 1807	5710643	99.44	0.24	Yes
NCBI Assembly	GCA_001208165.1	NCBI: cellular organisms; Bacteria; Terrabacteria group; Actinobacteria; Actinomycetia; Corynebacteriales; Mycobacteriaceae; Mycobacterium; Mycobacterium tuberculosis complex; Mycobacterium tuberculosis;	NCBI: 1773	4250236	95.73	1.23	Yes
NCBI Assembly	GCA_001326025.1	NCBI: cellular organisms; Bacteria; Terrabacteria group; Actinobacteria; Actinomycetia; Corynebacteriales; Mycobacteriaceae; Mycobacterium; Mycobacterium tuberculosis complex; Mycobacterium tuberculosis;	NCBI: 1773	4353145	99.94	0.00	Yes
NCBI Assembly	GCA_001378795.1	NCBI: cellular organisms; Bacteria; Terrabacteria group; Actinobacteria; Actinomycetia; Corynebacteriales; Mycobacteriaceae; Mycobacterium; Mycobacterium tuberculosis complex; Mycobacterium tuberculosis;	NCBI: 1773	4373613	99.94	0.00	Yes
NCBI Assembly	GCA_001145765.1	NCBI: cellular organisms; Bacteria; Terrabacteria group; Actinobacteria; Actinomycetia; Corynebacteriales; Mycobacteriaceae; Mycobacterium; Mycobacterium tuberculosis complex; Mycobacterium tuberculosis;	NCBI: 1773	4396169	99.94	0.00	Yes
NCBI Assembly	GCA_001376595.1	NCBI: cellular organisms; Bacteria; Terrabacteria group; Actinobacteria; Actinomycetia; Corynebacteriales; Mycobacteriaceae; Mycobacterium; Mycobacterium tuberculosis complex; Mycobacterium tuberculosis;	NCBI: 1773	4379896	99.94	0.00	Yes
NCBI Assembly	GCA_001380035.1	NCBI: cellular organisms; Bacteria; Terrabacteria group; Actinobacteria; Actinomycetia; Corynebacteriales; Mycobacteriaceae; Mycobacterium; Mycobacterium tuberculosis complex; Mycobacterium tuberculosis;	NCBI: 1773	4364895	99.94	0.33	Yes
NCBI Assembly	GCA_001194925.1	NCBI: cellular organisms; Bacteria; Terrabacteria group; Actinobacteria; Actinomycetia; Corynebacteriales; Mycobacteriaceae; Mycobacterium; Mycobacterium tuberculosis complex; Mycobacterium tuberculosis;	NCBI: 1773	4310631	99.28	0.33	Yes
NCBI Assembly	GCA_001205295.1	NCBI: cellular organisms; Bacteria; Terrabacteria group; Actinobacteria; Actinomycetia; Corynebacteriales; Mycobacteriaceae; Mycobacterium; Mycobacterium tuberculosis complex; Mycobacterium tuberculosis;	NCBI: 1773	4304496	99.41	0.33	Yes
NCBI Assembly	GCA_001377855.1	NCBI: cellular organisms; Bacteria; Terrabacteria group; Actinobacteria; Actinomycetia; Corynebacteriales; Mycobacteriaceae; Mycobacterium; Mycobacterium tuberculosis complex; Mycobacterium tuberculosis;	NCBI: 1773	4355346	99.94	0.00	Yes
NCBI Assembly	GCA_001211765.1	NCBI: cellular organisms; Bacteria; Terrabacteria group; Actinobacteria; Actinomycetia; Corynebacteriales; Mycobacteriaceae; Mycobacterium; Mycobacterium tuberculosis complex; Mycobacterium tuberculosis;	NCBI: 1773	4317228	99.94	0.00	Yes
NCBI Assembly	GCA_001044255.1	NCBI: cellular organisms; Bacteria; Terrabacteria group; Actinobacteria; Actinomycetia; Corynebacteriales; Mycobacteriaceae; Mycolicibacterium; Mycolicibacterium chubuense;	NCBI: 1800	5945009	99.34	0.18	Yes
NCBI Assembly	GCA_001318665.1	NCBI: cellular organisms; Bacteria; Terrabacteria group; Actinobacteria; Actinomycetia; Corynebacteriales; Mycobacteriaceae; Mycobacterium; Mycobacterium tuberculosis complex; Mycobacterium tuberculosis;	NCBI: 1773	4298416	99.94	1.07	Yes
NCBI Assembly	GCA_001208465.1	NCBI: cellular organisms; Bacteria; Terrabacteria group; Actinobacteria; Actinomycetia; Corynebacteriales; Mycobacteriaceae; Mycobacterium; Mycobacterium tuberculosis complex; Mycobacterium tuberculosis;	NCBI: 1773	4290020	99.94	0.67	Yes
NCBI Assembly	GCA_001326045.1	NCBI: cellular organisms; Bacteria; Terrabacteria group; Actinobacteria; Actinomycetia; Corynebacteriales; Mycobacteriaceae; Mycobacterium; Mycobacterium tuberculosis complex; Mycobacterium tuberculosis;	NCBI: 1773	4353378	99.94	0.00	Yes
NCBI Assembly	GCA_001145965.1	NCBI: cellular organisms; Bacteria; Terrabacteria group; Actinobacteria; Actinomycetia; Corynebacteriales; Mycobacteriaceae; Mycobacterium; Mycobacterium tuberculosis complex; Mycobacterium tuberculosis;	NCBI: 1773	3938799	88.82	4.54	No

NCBI Assembly	GCA_001378815.1	NCBI: cellular organisms; Bacteria; Terrabacteria group; Actinobacteria; Actinomycetia; Corynebacteriales; Mycobacteriaceae; Mycobacterium; Mycobacterium tuberculosis complex; Mycobacterium tuberculosis;	NCBI: 1773	4361665	99.94	0.00	Yes
NCBI Assembly	GCA_001376615.1	NCBI: cellular organisms; Bacteria; Terrabacteria group; Actinobacteria; Actinomycetia; Corynebacteriales; Mycobacteriaceae; Mycobacterium; Mycobacterium tuberculosis complex; Mycobacterium tuberculosis;	NCBI: 1773	4356693	99.94	0.00	Yes
NCBI Assembly	GCA_001380055.1	NCBI: cellular organisms; Bacteria; Terrabacteria group; Actinobacteria; Actinomycetia; Corynebacteriales; Mycobacteriaceae; Mycobacterium; Mycobacterium tuberculosis complex; Mycobacterium tuberculosis;	NCBI: 1773	4369116	99.94	0.00	Yes
NCBI Assembly	GCA_001205775.1	NCBI: cellular organisms; Bacteria; Terrabacteria group; Actinobacteria; Actinomycetia; Corynebacteriales; Mycobacteriaceae; Mycobacterium; Mycobacterium tuberculosis complex; Mycobacterium tuberculosis;	NCBI: 1773	4307140	99.28	0.33	Yes
NCBI Assembly	GCA_001195185.1	NCBI: cellular organisms; Bacteria; Terrabacteria group; Actinobacteria; Actinomycetia; Corynebacteriales; Mycobacteriaceae; Mycobacterium; Mycobacterium tuberculosis complex; Mycobacterium tuberculosis;	NCBI: 1773	4302287	99.94	0.00	Yes
NCBI Assembly	GCA_001377875.1	NCBI: cellular organisms; Bacteria; Terrabacteria group; Actinobacteria; Actinomycetia; Corynebacteriales; Mycobacteriaceae; Mycobacterium; Mycobacterium tuberculosis complex; Mycobacterium tuberculosis;	NCBI: 1773	4362514	99.94	0.00	Yes
NCBI Assembly	GCA_001212345.1	NCBI: cellular organisms; Bacteria; Terrabacteria group; Actinobacteria; Actinomycetia; Corynebacteriales; Mycobacteriaceae; Mycobacterium; Mycobacterium tuberculosis complex; Mycobacterium tuberculosis;	NCBI: 1773	4296476	99.94	0.00	Yes
NCBI Assembly	GCA_001049355.1	NCBI: cellular organisms; Bacteria; Terrabacteria group; Actinobacteria; Actinomycetia; Corynebacteriales; Mycobacteriaceae; Mycolicibacterium; Mycolicibacterium aurum;	NCBI: 1791	6019325	100.00	0.05	Yes
NCBI Assembly	GCA_001208505.1	NCBI: cellular organisms; Bacteria; Terrabacteria group; Actinobacteria; Actinomycetia; Corynebacteriales; Mycobacteriaceae; Mycobacterium; Mycobacterium tuberculosis complex; Mycobacterium tuberculosis;	NCBI: 1773	4005821	77.64	3.31	No
NCBI Assembly	GCA_001318685.1	NCBI: cellular organisms; Bacteria; Terrabacteria group; Actinobacteria; Actinomycetia; Corynebacteriales; Mycobacteriaceae; Mycobacterium; Mycobacterium tuberculosis complex; Mycobacterium tuberculosis;	NCBI: 1773	4296862	99.36	2.14	Yes
NCBI Assembly	GCA_001147025.1	NCBI: cellular organisms; Bacteria; Terrabacteria group; Actinobacteria; Actinomycetia; Corynebacteriales; Mycobacteriaceae; Mycobacterium; Mycobacterium tuberculosis complex; Mycobacterium tuberculosis;	NCBI: 1773	4358731	99.94	0.00	Yes
NCBI Assembly	GCA_001326065.1	NCBI: cellular organisms; Bacteria; Terrabacteria group; Actinobacteria; Actinomycetia; Corynebacteriales; Mycobacteriaceae; Mycobacterium; Mycobacterium tuberculosis complex; Mycobacterium tuberculosis;	NCBI: 1773	4371519	99.94	0.00	Yes
NCBI Assembly	GCA_001378835.1	NCBI: cellular organisms; Bacteria; Terrabacteria group; Actinobacteria; Actinomycetia; Corynebacteriales; Mycobacteriaceae; Mycobacterium; Mycobacterium tuberculosis complex; Mycobacterium tuberculosis;	NCBI: 1773	4401935	99.94	0.00	Yes
NCBI Assembly	GCA_001376635.1	NCBI: cellular organisms; Bacteria; Terrabacteria group; Actinobacteria; Actinomycetia; Corynebacteriales; Mycobacteriaceae; Mycobacterium; Mycobacterium tuberculosis complex; Mycobacterium tuberculosis;	NCBI: 1773	4362625	99.94	0.00	Yes
NCBI Assembly	GCA_001206155.1	NCBI: cellular organisms; Bacteria; Terrabacteria group; Actinobacteria; Actinomycetia; Corynebacteriales; Mycobacteriaceae; Mycobacterium; Mycobacterium tuberculosis complex; Mycobacterium tuberculosis;	NCBI: 1773	4293509	99.28	1.36	Yes
NCBI Assembly	GCA_001380075.1	NCBI: cellular organisms; Bacteria; Terrabacteria group; Actinobacteria; Actinomycetia; Corynebacteriales; Mycobacteriaceae; Mycobacterium; Mycobacterium tuberculosis complex; Mycobacterium tuberculosis;	NCBI: 1773	4400336	99.94	0.00	Yes
NCBI Assembly	GCA_001195325.1	NCBI: cellular organisms; Bacteria; Terrabacteria group; Actinobacteria; Actinomycetia; Corynebacteriales; Mycobacteriaceae; Mycobacterium; Mycobacterium tuberculosis complex; Mycobacterium tuberculosis;	NCBI: 1773	4312907	99.28	0.67	Yes
NCBI Assembly	GCA_001377895.1	NCBI: cellular organisms; Bacteria; Terrabacteria group; Actinobacteria; Actinomycetia; Corynebacteriales; Mycobacteriaceae; Mycobacterium; Mycobacterium tuberculosis complex; Mycobacterium tuberculosis;	NCBI: 1773	4364173	99.94	0.00	Yes

NCBI Assembly	GCA_001213085.1	NCBI: cellular organisms; Bacteria; Terrabacteria group; Actinobacteria; Actinomycetia; Corynebacteriales; Mycobacteriaceae; Mycobacteroides; Mycobacteroides abscessus;	NCBI: 36809	8596915	100.00	53.64	No
NCBI Assembly	GCA_001049395.1	NCBI: cellular organisms; Bacteria; Terrabacteria group; Actinobacteria; Actinomycetia; Corynebacteriales; Mycobacteriaceae; Mycobacterium; Mycobacterium tuberculosis complex; Mycobacterium tuberculosis;	NCBI: 1773	4497391	99.94	2.06	Yes
NCBI Assembly	GCA_001208525.1	NCBI: cellular organisms; Bacteria; Terrabacteria group; Actinobacteria; Actinomycetia; Corynebacteriales; Mycobacteriaceae; Mycobacterium; Mycobacterium tuberculosis complex; Mycobacterium tuberculosis;	NCBI: 1773	4229924	98.66	1.67	Yes
NCBI Assembly	GCA_001318705.1	NCBI: cellular organisms; Bacteria; Terrabacteria group; Actinobacteria; Actinomycetia; Corynebacteriales; Mycobacteriaceae; Mycobacterium; Mycobacterium tuberculosis complex; Mycobacterium tuberculosis;	NCBI: 1773	4291567	99.61	0.71	Yes
NCBI Assembly	GCA_001147145.1	NCBI: cellular organisms; Bacteria; Terrabacteria group; Actinobacteria; Actinomycetia; Corynebacteriales; Mycobacteriaceae; Mycobacterium; Mycobacterium tuberculosis complex; Mycobacterium tuberculosis;	NCBI: 1773	4315242	99.94	0.00	Yes
NCBI Assembly	GCA_001326095.1	NCBI: cellular organisms; Bacteria; Terrabacteria group; Actinobacteria; Actinomycetia; Corynebacteriales; Mycobacteriaceae; Mycobacterium; Mycobacterium tuberculosis complex; Mycobacterium tuberculosis;	NCBI: 1773	4394542	99.94	0.00	Yes
NCBI Assembly	GCA_001378855.1	NCBI: cellular organisms; Bacteria; Terrabacteria group; Actinobacteria; Actinomycetia; Corynebacteriales; Mycobacteriaceae; Mycobacterium; Mycobacterium tuberculosis complex; Mycobacterium tuberculosis;	NCBI: 1773	4368292	99.94	0.17	Yes
NCBI Assembly	GCA_001376655.1	NCBI: cellular organisms; Bacteria; Terrabacteria group; Actinobacteria; Actinomycetia; Corynebacteriales; Mycobacteriaceae; Mycobacterium; Mycobacterium tuberculosis complex; Mycobacterium tuberculosis;	NCBI: 1773	4388134	99.94	0.00	Yes
NCBI Assembly	GCA_001206715.1	NCBI: cellular organisms; Bacteria; Terrabacteria group; Actinobacteria; Actinomycetia; Corynebacteriales; Mycobacteriaceae; Mycobacterium; Mycobacterium tuberculosis complex; Mycobacterium tuberculosis;	NCBI: 1773	4275150	98.35	0.73	Yes
NCBI Assembly	GCA_001380095.1	NCBI: cellular organisms; Bacteria; Terrabacteria group; Actinobacteria; Actinomycetia; Corynebacteriales; Mycobacteriaceae; Mycobacterium; Mycobacterium tuberculosis complex; Mycobacterium tuberculosis;	NCBI: 1773	4363522	99.94	0.17	Yes
NCBI Assembly	GCA_001195645.1	NCBI: cellular organisms; Bacteria; Terrabacteria group; Actinobacteria; Actinomycetia; Corynebacteriales; Mycobacteriaceae; Mycobacterium; Mycobacterium tuberculosis complex; Mycobacterium tuberculosis;	NCBI: 1773	4258084	98.75	1.70	Yes
NCBI Assembly	GCA_001377915.1	NCBI: cellular organisms; Bacteria; Terrabacteria group; Actinobacteria; Actinomycetia; Corynebacteriales; Mycobacteriaceae; Mycobacterium; Mycobacterium tuberculosis complex; Mycobacterium tuberculosis;	NCBI: 1773	4374826	99.94	0.00	Yes
NCBI Assembly	GCA_001213925.1	NCBI: cellular organisms; Bacteria; Terrabacteria group; Actinobacteria; Actinomycetia; Corynebacteriales; Mycobacteriaceae; Mycobacteroides; Mycobacteroides abscessus;	NCBI: 36809	6259237	98.92	8.46	No
NCBI Assembly	GCA_001049415.1	NCBI: cellular organisms; Bacteria; Terrabacteria group; Actinobacteria; Actinomycetia; Corynebacteriales; Mycobacteriaceae; Mycobacterium; Mycobacterium tuberculosis complex; Mycobacterium tuberculosis;	NCBI: 1773	4450560	99.61	1.67	Yes
NCBI Assembly	GCA_001147745.1	NCBI: cellular organisms; Bacteria; Terrabacteria group; Actinobacteria; Actinomycetia; Corynebacteriales; Mycobacteriaceae; Mycobacterium; Mycobacterium tuberculosis complex; Mycobacterium tuberculosis;	NCBI: 1773	4381379	99.94	0.33	Yes
NCBI Assembly	GCA_001208625.1	NCBI: cellular organisms; Bacteria; Terrabacteria group; Actinobacteria; Actinomycetia; Corynebacteriales; Mycobacteriaceae; Mycobacterium; Mycobacterium tuberculosis complex; Mycobacterium tuberculosis;	NCBI: 1773	4288347	99.61	2.15	Yes
NCBI Assembly	GCA_001318725.1	NCBI: cellular organisms; Bacteria; Terrabacteria group; Actinobacteria; Actinomycetia; Corynebacteriales; Mycobacteriaceae; Mycobacterium; Mycobacterium tuberculosis complex; Mycobacterium tuberculosis;	NCBI: 1773	4333275	99.49	0.03	Yes
NCBI Assembly	GCA_001378875.1	NCBI: cellular organisms; Bacteria; Terrabacteria group; Actinobacteria; Actinomycetia; Corynebacteriales; Mycobacteriaceae; Mycobacterium; Mycobacterium tuberculosis complex; Mycobacterium tuberculosis;	NCBI: 1773	4359426	99.94	0.00	Yes

NCBI Assembly	GCA_001326115.1	NCBI: cellular organisms; Bacteria; Terrabacteria group; Actinobacteria; Actinomycetia; Corynebacteriales; Mycobacteriaceae; Mycobacterium; Mycobacterium tuberculosis complex; Mycobacterium tuberculosis;	NCBI: 1773	4386892	99.94	0.00	Yes
NCBI Assembly	GCA_001376675.1	NCBI: cellular organisms; Bacteria; Terrabacteria group; Actinobacteria; Actinomycetia; Corynebacteriales; Mycobacteriaceae; Mycobacterium; Mycobacterium tuberculosis complex; Mycobacterium tuberculosis;	NCBI: 1773	4364965	99.94	0.00	Yes
NCBI Assembly	GCA_001380115.1	NCBI: cellular organisms; Bacteria; Terrabacteria group; Actinobacteria; Actinomycetia; Corynebacteriales; Mycobacteriaceae; Mycobacterium; Mycobacterium tuberculosis complex; Mycobacterium tuberculosis;	NCBI: 1773	4401109	99.94	0.00	Yes
NCBI Assembly	GCA_001206955.1	NCBI: cellular organisms; Bacteria; Terrabacteria group; Actinobacteria; Actinomycetia; Corynebacteriales; Mycobacteriaceae; Mycobacterium; Mycobacterium tuberculosis complex; Mycobacterium tuberculosis;	NCBI: 1773	4265922	99.64	0.11	Yes
NCBI Assembly	GCA_001195665.1	NCBI: cellular organisms; Bacteria; Terrabacteria group; Actinobacteria; Actinomycetia; Corynebacteriales; Mycobacteriaceae; Mycobacterium; Mycobacterium tuberculosis complex; Mycobacterium tuberculosis;	NCBI: 1773	4299785	99.94	0.00	Yes
NCBI Assembly	GCA_001214385.1	NCBI: cellular organisms; Bacteria; Terrabacteria group; Actinobacteria; Actinomycetia; Corynebacteriales; Mycobacteriaceae; Mycobacteroides; Mycobacteroides abscessus;	NCBI: 36809	5146820	99.08	0.08	Yes
NCBI Assembly	GCA_001377935.1	NCBI: cellular organisms; Bacteria; Terrabacteria group; Actinobacteria; Actinomycetia; Corynebacteriales; Mycobacteriaceae; Mycobacterium; Mycobacterium tuberculosis complex; Mycobacterium tuberculosis;	NCBI: 1773	4356687	99.94	0.00	Yes
NCBI Assembly	GCA_001049435.1	NCBI: cellular organisms; Bacteria; Terrabacteria group; Actinobacteria; Actinomycetia; Corynebacteriales; Mycobacteriaceae; Mycobacterium; Mycobacterium tuberculosis complex; Mycobacterium tuberculosis;	NCBI: 1773	4350572	99.90	0.00	Yes
NCBI Assembly	GCA_001148065.1	NCBI: cellular organisms; Bacteria; Terrabacteria group; Actinobacteria; Actinomycetia; Corynebacteriales; Mycobacteriaceae; Mycobacterium; Mycobacterium tuberculosis complex; Mycobacterium tuberculosis;	NCBI: 1773	4238542	90.15	0.33	Yes
NCBI Assembly	GCA_001318745.1	NCBI: cellular organisms; Bacteria; Terrabacteria group; Actinobacteria; Actinomycetia; Corynebacteriales; Mycobacteriaceae; Mycobacterium; Mycobacterium tuberculosis complex; Mycobacterium tuberculosis;	NCBI: 1773	4284104	99.94	0.00	Yes
NCBI Assembly	GCA_001208685.1	NCBI: cellular organisms; Bacteria; Terrabacteria group; Actinobacteria; Actinomycetia; Corynebacteriales; Mycobacteriaceae; Mycobacterium; Mycobacterium tuberculosis complex; Mycobacterium tuberculosis;	NCBI: 1773	4220486	94.47	0.86	Yes
NCBI Assembly	GCA_001378895.1	NCBI: cellular organisms; Bacteria; Terrabacteria group; Actinobacteria; Actinomycetia; Corynebacteriales; Mycobacteriaceae; Mycobacterium; Mycobacterium tuberculosis complex; Mycobacterium tuberculosis;	NCBI: 1773	4363700	99.94	0.00	Yes
NCBI Assembly	GCA_001327595.1	NCBI: cellular organisms; Bacteria; Terrabacteria group; Actinobacteria; Actinomycetia; Corynebacteriales; Mycobacteriaceae; Mycobacterium; Mycobacterium tuberculosis complex; Mycobacterium tuberculosis;	NCBI: 1773	4302810	99.72	0.00	Yes
NCBI Assembly	GCA_001376695.1	NCBI: cellular organisms; Bacteria; Terrabacteria group; Actinobacteria; Actinomycetia; Corynebacteriales; Mycobacteriaceae; Mycobacterium; Mycobacterium tuberculosis complex; Mycobacterium tuberculosis;	NCBI: 1773	4387678	99.94	0.00	Yes
NCBI Assembly	GCA_001380135.1	NCBI: cellular organisms; Bacteria; Terrabacteria group; Actinobacteria; Actinomycetia; Corynebacteriales; Mycobacteriaceae; Mycobacterium; Mycobacterium tuberculosis complex; Mycobacterium tuberculosis;	NCBI: 1773	4368847	99.94	0.00	Yes
NCBI Assembly	GCA_001195685.1	NCBI: cellular organisms; Bacteria; Terrabacteria group; Actinobacteria; Actinomycetia; Corynebacteriales; Mycobacteriaceae; Mycobacterium; Mycobacterium tuberculosis complex; Mycobacterium tuberculosis;	NCBI: 1773	4302074	99.61	0.00	Yes
NCBI Assembly	GCA_001206975.1	NCBI: cellular organisms; Bacteria; Terrabacteria group; Actinobacteria; Actinomycetia; Corynebacteriales; Mycobacteriaceae; Mycobacterium; Mycobacterium tuberculosis complex; Mycobacterium tuberculosis;	NCBI: 1773	4285045	99.28	0.03	Yes
NCBI Assembly	GCA_001214905.1	NCBI: cellular organisms; Bacteria; Terrabacteria group; Actinobacteria; Actinomycetia; Corynebacteriales; Mycobacteriaceae; Mycobacteroides; Mycobacteroides abscessus;	NCBI: 36809	5157927	99.38	0.00	Yes

NCBI Assembly	GCA_001377955.1	NCBI: cellular organisms; Bacteria; Terrabacteria group; Actinobacteria; Actinomycetia; Corynebacteriales; Mycobacteriaceae; Mycobacterium; Mycobacterium tuberculosis complex; Mycobacterium tuberculosis;	NCBI: 1773	4356704	99.94	0.00	Yes
NCBI Assembly	GCA_001049455.1	NCBI: cellular organisms; Bacteria; Terrabacteria group; Actinobacteria; Actinomycetia; Corynebacteriales; Mycobacteriaceae; Mycobacterium; Mycobacterium tuberculosis complex; Mycobacterium tuberculosis;	NCBI: 1773	4489350	99.94	1.89	Yes
NCBI Assembly	GCA_001149285.1	NCBI: cellular organisms; Bacteria; Terrabacteria group; Actinobacteria; Actinomycetia; Corynebacteriales; Mycobacteriaceae; Mycobacterium; Mycobacterium tuberculosis complex; Mycobacterium tuberculosis;	NCBI: 1773	4297988	91.10	0.33	Yes
NCBI Assembly	GCA_001318765.1	NCBI: cellular organisms; Bacteria; Terrabacteria group; Actinobacteria; Actinomycetia; Corynebacteriales; Mycobacteriaceae; Mycobacterium; Mycobacterium tuberculosis complex; Mycobacterium tuberculosis;	NCBI: 1773	4154084	96.68	2.99	Yes
NCBI Assembly	GCA_001208765.1	NCBI: cellular organisms; Bacteria; Terrabacteria group; Actinobacteria; Actinomycetia; Corynebacteriales; Mycobacteriaceae; Mycobacterium; Mycobacterium tuberculosis complex; Mycobacterium tuberculosis;	NCBI: 1773	4323503	99.53	0.00	Yes
NCBI Assembly	GCA_001378915.1	NCBI: cellular organisms; Bacteria; Terrabacteria group; Actinobacteria; Actinomycetia; Corynebacteriales; Mycobacteriaceae; Mycobacterium; Mycobacterium tuberculosis complex; Mycobacterium tuberculosis;	NCBI: 1773	4367703	99.94	0.00	Yes
NCBI Assembly	GCA_001327615.1	NCBI: cellular organisms; Bacteria; Terrabacteria group; Actinobacteria; Actinomycetia; Corynebacteriales; Mycobacteriaceae; Mycobacterium; Mycobacterium tuberculosis complex; Mycobacterium tuberculosis;	NCBI: 1773	4310594	99.94	0.00	Yes
NCBI Assembly	GCA_001376715.1	NCBI: cellular organisms; Bacteria; Terrabacteria group; Actinobacteria; Actinomycetia; Corynebacteriales; Mycobacteriaceae; Mycobacterium; Mycobacterium tuberculosis complex; Mycobacterium tuberculosis;	NCBI: 1773	4355766	99.94	0.00	Yes
NCBI Assembly	GCA_001380155.1	NCBI: cellular organisms; Bacteria; Terrabacteria group; Actinobacteria; Actinomycetia; Corynebacteriales; Mycobacteriaceae; Mycobacterium; Mycobacterium tuberculosis complex; Mycobacterium tuberculosis;	NCBI: 1773	4352604	99.94	0.17	Yes
NCBI Assembly	GCA_001195725.1	NCBI: cellular organisms; Bacteria; Terrabacteria group; Actinobacteria; Actinomycetia; Corynebacteriales; Mycobacteriaceae; Mycobacterium; Mycobacterium tuberculosis complex; Mycobacterium tuberculosis;	NCBI: 1773	4297859	99.94	0.00	Yes
NCBI Assembly	GCA_001207015.1	NCBI: cellular organisms; Bacteria; Terrabacteria group; Actinobacteria; Actinomycetia; Corynebacteriales; Mycobacteriaceae; Mycobacterium; Mycobacterium tuberculosis complex; Mycobacterium tuberculosis;	NCBI: 1773	1943762	29.62	2.20	No
NCBI Assembly	GCA_001215125.1	NCBI: cellular organisms; Bacteria; Terrabacteria group; Actinobacteria; Actinomycetia; Corynebacteriales; Mycobacteriaceae; Mycobacteroides; Mycobacteroides abscessus;	NCBI: 36809	4908841	99.33	0.14	Yes
NCBI Assembly	GCA_001377975.1	NCBI: cellular organisms; Bacteria; Terrabacteria group; Actinobacteria; Actinomycetia; Corynebacteriales; Mycobacteriaceae; Mycobacterium; Mycobacterium tuberculosis complex; Mycobacterium tuberculosis;	NCBI: 1773	4379357	99.94	0.00	Yes
NCBI Assembly	GCA_001049475.1	NCBI: cellular organisms; Bacteria; Terrabacteria group; Actinobacteria; Actinomycetia; Corynebacteriales; Mycobacteriaceae; Mycobacterium; Mycobacterium tuberculosis complex; Mycobacterium tuberculosis;	NCBI: 1773	4308235	97.61	0.00	Yes
NCBI Assembly	GCA_001151565.1	NCBI: cellular organisms; Bacteria; Terrabacteria group; Actinobacteria; Actinomycetia; Corynebacteriales; Mycobacteriaceae; Mycobacterium; Mycobacterium tuberculosis complex; Mycobacterium tuberculosis;	NCBI: 1773	4360408	99.94	0.00	Yes
NCBI Assembly	GCA_001318785.1	NCBI: cellular organisms; Bacteria; Terrabacteria group; Actinobacteria; Actinomycetia; Corynebacteriales; Mycobacteriaceae; Mycobacterium; Mycobacterium tuberculosis complex; Mycobacterium tuberculosis;	NCBI: 1773	4085103	95.32	2.56	Yes
NCBI Assembly	GCA_001209145.1	NCBI: cellular organisms; Bacteria; Terrabacteria group; Actinobacteria; Actinomycetia; Corynebacteriales; Mycobacteriaceae; Mycobacterium; Mycobacterium tuberculosis complex; Mycobacterium tuberculosis;	NCBI: 1773	4294002	99.61	1.81	Yes
NCBI Assembly	GCA_001327635.1	NCBI: cellular organisms; Bacteria; Terrabacteria group; Actinobacteria; Actinomycetia; Corynebacteriales; Mycobacteriaceae; Mycobacterium; Mycobacterium tuberculosis complex; Mycobacterium tuberculosis;	NCBI: 1773	4304119	99.78	0.00	Yes

NCBI Assembly	GCA_001380175.1	NCBI: cellular organisms; Bacteria; Terrabacteria group; Actinobacteria; Actinomycetia; Corynebacteriales; Mycobacteriaceae; Mycobacterium; Mycobacterium tuberculosis complex; Mycobacterium tuberculosis;	NCBI: 1773	4362751	99.94	0.00	Yes
NCBI Assembly	GCA_001378935.1	NCBI: cellular organisms; Bacteria; Terrabacteria group; Actinobacteria; Actinomycetia; Corynebacteriales; Mycobacteriaceae; Mycobacterium; Mycobacterium tuberculosis complex; Mycobacterium tuberculosis;	NCBI: 1773	4401963	99.94	0.00	Yes
NCBI Assembly	GCA_001376735.1	NCBI: cellular organisms; Bacteria; Terrabacteria group; Actinobacteria; Actinomycetia; Corynebacteriales; Mycobacteriaceae; Mycobacterium; Mycobacterium tuberculosis complex; Mycobacterium tuberculosis;	NCBI: 1773	4362914	99.94	0.00	Yes
NCBI Assembly	GCA_001195765.1	NCBI: cellular organisms; Bacteria; Terrabacteria group; Actinobacteria; Actinomycetia; Corynebacteriales; Mycobacteriaceae; Mycobacterium; Mycobacterium tuberculosis complex; Mycobacterium tuberculosis;	NCBI: 1773	4256760	98.37	2.26	Yes
NCBI Assembly	GCA_001215425.1	NCBI: cellular organisms; Bacteria; Terrabacteria group; Actinobacteria; Actinomycetia; Corynebacteriales; Mycobacteriaceae; Mycobacteroides; Mycobacteroides abscessus;	NCBI: 36809	6168506	99.12	30.88	No
NCBI Assembly	GCA_001377995.1	NCBI: cellular organisms; Bacteria; Terrabacteria group; Actinobacteria; Actinomycetia; Corynebacteriales; Mycobacteriaceae; Mycobacterium; Mycobacterium tuberculosis complex; Mycobacterium tuberculosis;	NCBI: 1773	4362155	99.94	0.00	Yes
NCBI Assembly	GCA_001207075.1	NCBI: cellular organisms; Bacteria; Terrabacteria group; Actinobacteria; Actinomycetia; Corynebacteriales; Mycobacteriaceae; Mycobacterium; Mycobacterium tuberculosis complex; Mycobacterium tuberculosis;	NCBI: 1773	4273178	99.03	1.96	Yes
NCBI Assembly	GCA_001050015.1	NCBI: cellular organisms; Bacteria; Terrabacteria group; Actinobacteria; Actinomycetia; Corynebacteriales; Mycobacteriaceae; Mycolicibacterium; Mycolicibacterium malmesburyense;	NCBI: 1566886	5470965	99.77	1.81	Yes
NCBI Assembly	GCA_001209385.1	NCBI: cellular organisms; Bacteria; Terrabacteria group; Actinobacteria; Actinomycetia; Corynebacteriales; Mycobacteriaceae; Mycobacterium; Mycobacterium tuberculosis complex; Mycobacterium tuberculosis;	NCBI: 1773	4232938	99.25	1.78	Yes
NCBI Assembly	GCA_001151685.1	NCBI: cellular organisms; Bacteria; Terrabacteria group; Actinobacteria; Actinomycetia; Corynebacteriales; Mycobacteriaceae; Mycobacterium; Mycobacterium tuberculosis complex; Mycobacterium tuberculosis;	NCBI: 1773	4387362	99.94	0.00	Yes
NCBI Assembly	GCA_001318805.1	NCBI: cellular organisms; Bacteria; Terrabacteria group; Actinobacteria; Actinomycetia; Corynebacteriales; Mycobacteriaceae; Mycobacterium; Mycobacterium tuberculosis complex; Mycobacterium tuberculosis;	NCBI: 1773	3416310	58.84	5.35	No
NCBI Assembly	GCA_001327655.1	NCBI: cellular organisms; Bacteria; Terrabacteria group; Actinobacteria; Actinomycetia; Corynebacteriales; Mycobacteriaceae; Mycobacterium; Mycobacterium tuberculosis complex; Mycobacterium tuberculosis;	NCBI: 1773	4348955	99.94	0.00	Yes
NCBI Assembly	GCA_001380195.1	NCBI: cellular organisms; Bacteria; Terrabacteria group; Actinobacteria; Actinomycetia; Corynebacteriales; Mycobacteriaceae; Mycobacterium; Mycobacterium tuberculosis complex; Mycobacterium tuberculosis;	NCBI: 1773	4398878	99.94	0.00	Yes
NCBI Assembly	GCA_001378955.1	NCBI: cellular organisms; Bacteria; Terrabacteria group; Actinobacteria; Actinomycetia; Corynebacteriales; Mycobacteriaceae; Mycobacterium; Mycobacterium tuberculosis complex; Mycobacterium tuberculosis;	NCBI: 1773	4380877	99.94	0.00	Yes
NCBI Assembly	GCA_001376755.1	NCBI: cellular organisms; Bacteria; Terrabacteria group; Actinobacteria; Actinomycetia; Corynebacteriales; Mycobacteriaceae; Mycobacterium; Mycobacterium tuberculosis complex; Mycobacterium tuberculosis;	NCBI: 1773	4362838	99.94	0.00	Yes
NCBI Assembly	GCA_001195845.1	NCBI: cellular organisms; Bacteria; Terrabacteria group; Actinobacteria; Actinomycetia; Corynebacteriales; Mycobacteriaceae; Mycobacterium; Mycobacterium tuberculosis complex; Mycobacterium tuberculosis;	NCBI: 1773	4289988	99.61	0.00	Yes
NCBI Assembly	GCA_001207215.1	NCBI: cellular organisms; Bacteria; Terrabacteria group; Actinobacteria; Actinomycetia; Corynebacteriales; Mycobacteriaceae; Mycobacterium; Mycobacterium tuberculosis complex; Mycobacterium tuberculosis;	NCBI: 1773	4288829	99.53	1.00	Yes
NCBI Assembly	GCA_001215805.1	NCBI: cellular organisms; Bacteria; Terrabacteria group; Actinobacteria; Actinomycetia; Corynebacteriales; Mycobacteriaceae; Mycobacteroides; Mycobacteroides abscessus;	NCBI: 36809	6565703	100.00	34.20	No

NCBI Assembly	GCA_001378015.1	NCBI: cellular organisms; Bacteria; Terrabacteria group; Actinobacteria; Actinomycetia; Corynebacteriales; Mycobacteriaceae; Mycobacterium; Mycobacterium tuberculosis complex; Mycobacterium tuberculosis;	NCBI: 1773	4363035	99.94	0.00	Yes
NCBI Assembly	GCA_001050035.1	NCBI: cellular organisms; Bacteria; Terrabacteria group; Actinobacteria; Actinomycetia; Corynebacteriales; Mycobacteriaceae; Mycolicibacterium; Mycolicibacterium komanii;	NCBI: 1566885	5378970	100.00	1.36	Yes
NCBI Assembly	GCA_001209445.1	NCBI: cellular organisms; Bacteria; Terrabacteria group; Actinobacteria; Actinomycetia; Corynebacteriales; Mycobacteriaceae; Mycobacterium; Mycobacterium tuberculosis complex; Mycobacterium tuberculosis;	NCBI: 1773	4300053	99.28	0.00	Yes
NCBI Assembly	GCA_001152945.1	NCBI: cellular organisms; Bacteria; Terrabacteria group; Actinobacteria; Actinomycetia; Corynebacteriales; Mycobacteriaceae; Mycobacterium; Mycobacterium tuberculosis complex; Mycobacterium tuberculosis;	NCBI: 1773	4363513	99.94	0.00	Yes
NCBI Assembly	GCA_001318825.1	NCBI: cellular organisms; Bacteria; Terrabacteria group; Actinobacteria; Actinomycetia; Corynebacteriales; Mycobacteriaceae; Mycobacterium; Mycobacterium tuberculosis complex; Mycobacterium tuberculosis;	NCBI: 1773	4228453	99.09	0.94	Yes
NCBI Assembly	GCA_001378975.1	NCBI: cellular organisms; Bacteria; Terrabacteria group; Actinobacteria; Actinomycetia; Corynebacteriales; Mycobacteriaceae; Mycobacterium; Mycobacterium tuberculosis complex; Mycobacterium tuberculosis;	NCBI: 1773	4364973	99.94	0.17	Yes
NCBI Assembly	GCA_001327675.1	NCBI: cellular organisms; Bacteria; Terrabacteria group; Actinobacteria; Actinomycetia; Corynebacteriales; Mycobacteriaceae; Mycobacterium; Mycobacterium tuberculosis complex; Mycobacterium tuberculosis;	NCBI: 1773	4317924	99.72	0.00	Yes
NCBI Assembly	GCA_001195905.1	NCBI: cellular organisms; Bacteria; Terrabacteria group; Actinobacteria; Actinomycetia; Corynebacteriales; Mycobacteriaceae; Mycobacterium; Mycobacterium tuberculosis complex; Mycobacterium tuberculosis;	NCBI: 1773	4249059	99.28	0.33	Yes
NCBI Assembly	GCA_001380215.1	NCBI: cellular organisms; Bacteria; Terrabacteria group; Actinobacteria; Actinomycetia; Corynebacteriales; Mycobacteriaceae; Mycobacterium; Mycobacterium tuberculosis complex; Mycobacterium tuberculosis;	NCBI: 1773	4369377	99.94	0.00	Yes
NCBI Assembly	GCA_001376775.1	NCBI: cellular organisms; Bacteria; Terrabacteria group; Actinobacteria; Actinomycetia; Corynebacteriales; Mycobacteriaceae; Mycobacterium; Mycobacterium tuberculosis complex; Mycobacterium tuberculosis;	NCBI: 1773	4363081	99.94	0.00	Yes
NCBI Assembly	GCA_001207555.1	NCBI: cellular organisms; Bacteria; Terrabacteria group; Actinobacteria; Actinomycetia; Corynebacteriales; Mycobacteriaceae; Mycobacterium; Mycobacterium tuberculosis complex; Mycobacterium tuberculosis;	NCBI: 1773	4319615	99.88	0.00	Yes
NCBI Assembly	GCA_001378035.1	NCBI: cellular organisms; Bacteria; Terrabacteria group; Actinobacteria; Actinomycetia; Corynebacteriales; Mycobacteriaceae; Mycobacterium; Mycobacterium tuberculosis complex; Mycobacterium tuberculosis;	NCBI: 1773	4361359	99.94	0.00	Yes
NCBI Assembly	GCA_001215825.1	NCBI: cellular organisms; Bacteria; Terrabacteria group; Actinobacteria; Actinomycetia; Corynebacteriales; Mycobacteriaceae; Mycobacteroides; Mycobacteroides abscessus;	NCBI: 36809	5160135	98.21	0.08	Yes
NCBI Assembly	GCA_001050055.1	NCBI: cellular organisms; Bacteria; Terrabacteria group; Actinobacteria; Actinomycetia; Corynebacteriales; Mycobacteriaceae; Mycolicibacter; Mycolicibacter nonchromogenicus;	NCBI: 1782	6353214	88.91	18.80	No
NCBI Assembly	GCA_001155105.1	NCBI: cellular organisms; Bacteria; Terrabacteria group; Actinobacteria; Actinomycetia; Corynebacteriales; Mycobacteriaceae; Mycobacterium; Mycobacterium tuberculosis complex; Mycobacterium tuberculosis;	NCBI: 1773	4703020	99.94	5.96	No
NCBI Assembly	GCA_001209585.1	NCBI: cellular organisms; Bacteria; Terrabacteria group; Actinobacteria; Actinomycetia; Corynebacteriales; Mycobacteriaceae; Mycobacterium; Mycobacterium tuberculosis complex; Mycobacterium tuberculosis;	NCBI: 1773	4303360	99.50	2.64	Yes
NCBI Assembly	GCA_001378995.1	NCBI: cellular organisms; Bacteria; Terrabacteria group; Actinobacteria; Actinomycetia; Corynebacteriales; Mycobacteriaceae; Mycobacterium; Mycobacterium tuberculosis complex; Mycobacterium tuberculosis;	NCBI: 1773	4366271	99.94	0.00	Yes
NCBI Assembly	GCA_001327695.1	NCBI: cellular organisms; Bacteria; Terrabacteria group; Actinobacteria; Actinomycetia; Corynebacteriales; Mycobacteriaceae; Mycobacterium; Mycobacterium tuberculosis complex; Mycobacterium tuberculosis;	NCBI: 1773	4321043	99.61	0.00	Yes

NCBI Assembly	GCA_001318845.1	NCBI: cellular organisms; Bacteria; Terrabacteria group; Actinobacteria; Actinomycetia; Corynebacteriales; Mycobacteriaceae; Mycobacterium; Mycobacterium tuberculosis complex; Mycobacterium tuberculosis;	NCBI: 1773	4250367	97.56	1.28	Yes
NCBI Assembly	GCA_001195965.1	NCBI: cellular organisms; Bacteria; Terrabacteria group; Actinobacteria; Actinomycetia; Corynebacteriales; Mycobacteriaceae; Mycobacterium; Mycobacterium tuberculosis complex; Mycobacterium tuberculosis;	NCBI: 1773	4284678	99.89	1.33	Yes
NCBI Assembly	GCA_001380235.1	NCBI: cellular organisms; Bacteria; Terrabacteria group; Actinobacteria; Actinomycetia; Corynebacteriales; Mycobacteriaceae; Mycobacterium; Mycobacterium tuberculosis complex; Mycobacterium tuberculosis;	NCBI: 1773	4364736	99.94	0.00	Yes
NCBI Assembly	GCA_001376795.1	NCBI: cellular organisms; Bacteria; Terrabacteria group; Actinobacteria; Actinomycetia; Corynebacteriales; Mycobacteriaceae; Mycobacterium; Mycobacterium tuberculosis complex; Mycobacterium tuberculosis;	NCBI: 1773	4385452	99.94	0.00	Yes
NCBI Assembly	GCA_001207715.1	NCBI: cellular organisms; Bacteria; Terrabacteria group; Actinobacteria; Actinomycetia; Corynebacteriales; Mycobacteriaceae; Mycobacterium; Mycobacterium tuberculosis complex; Mycobacterium tuberculosis;	NCBI: 1773	4235853	93.24	0.56	Yes
NCBI Assembly	GCA_001378055.1	NCBI: cellular organisms; Bacteria; Terrabacteria group; Actinobacteria; Actinomycetia; Corynebacteriales; Mycobacteriaceae; Mycobacterium; Mycobacterium tuberculosis complex; Mycobacterium tuberculosis;	NCBI: 1773	4357534	99.94	0.00	Yes
NCBI Assembly	GCA_001216105.1	NCBI: cellular organisms; Bacteria; Terrabacteria group; Actinobacteria; Actinomycetia; Corynebacteriales; Mycobacteriaceae; Mycobacteroides; Mycobacteroides abscessus;	NCBI: 36809	5159122	99.14	0.08	Yes
NCBI Assembly	GCA_001050395.1	NCBI: cellular organisms; Bacteria; Terrabacteria group; Actinobacteria; Actinomycetia; Corynebacteriales; Mycobacteriaceae; Mycobacteroides; Mycobacteroides abscessus;	NCBI: 1132508	5257136	99.90	0.08	Yes
NCBI Assembly	GCA_001155525.1	NCBI: cellular organisms; Bacteria; Terrabacteria group; Actinobacteria; Actinomycetia; Corynebacteriales; Mycobacteriaceae; Mycobacterium; Mycobacterium tuberculosis complex; Mycobacterium tuberculosis;	NCBI: 1773	4335473	99.94	0.00	Yes
NCBI Assembly	GCA_001209665.1	NCBI: cellular organisms; Bacteria; Terrabacteria group; Actinobacteria; Actinomycetia; Corynebacteriales; Mycobacteriaceae; Mycobacterium; Mycobacterium tuberculosis complex; Mycobacterium tuberculosis;	NCBI: 1773	4281624	98.86	1.36	Yes
NCBI Assembly	GCA_001327715.1	NCBI: cellular organisms; Bacteria; Terrabacteria group; Actinobacteria; Actinomycetia; Corynebacteriales; Mycobacteriaceae; Mycobacterium; Mycobacterium tuberculosis complex; Mycobacterium tuberculosis;	NCBI: 1773	4306514	99.94	0.00	Yes
NCBI Assembly	GCA_001196185.1	NCBI: cellular organisms; Bacteria; Terrabacteria group; Actinobacteria; Actinomycetia; Corynebacteriales; Mycobacteriaceae; Mycobacterium; Mycobacterium tuberculosis complex; Mycobacterium tuberculosis;	NCBI: 1773	4321187	99.28	0.33	Yes
NCBI Assembly	GCA_001318865.1	NCBI: cellular organisms; Bacteria; Terrabacteria group; Actinobacteria; Actinomycetia; Corynebacteriales; Mycobacteriaceae; Mycobacterium; Mycobacterium tuberculosis complex; Mycobacterium tuberculosis;	NCBI: 1773	4252702	99.18	1.16	Yes
NCBI Assembly	GCA_001379015.1	NCBI: cellular organisms; Bacteria; Terrabacteria group; Actinobacteria; Actinomycetia; Corynebacteriales; Mycobacteriaceae; Mycobacterium; Mycobacterium tuberculosis complex; Mycobacterium tuberculosis;	NCBI: 1773	4359312	99.94	0.00	Yes
NCBI Assembly	GCA_001380255.1	NCBI: cellular organisms; Bacteria; Terrabacteria group; Actinobacteria; Actinomycetia; Corynebacteriales; Mycobacteriaceae; Mycobacterium; Mycobacterium tuberculosis complex; Mycobacterium tuberculosis;	NCBI: 1773	4364868	99.94	0.00	Yes
NCBI Assembly	GCA_001376815.1	NCBI: cellular organisms; Bacteria; Terrabacteria group; Actinobacteria; Actinomycetia; Corynebacteriales; Mycobacteriaceae; Mycobacterium; Mycobacterium tuberculosis complex; Mycobacterium tuberculosis;	NCBI: 1773	4371212	99.94	0.00	Yes
NCBI Assembly	GCA_001207945.1	NCBI: cellular organisms; Bacteria; Terrabacteria group; Actinobacteria; Actinomycetia; Corynebacteriales; Mycobacteriaceae; Mycobacterium; Mycobacterium tuberculosis complex; Mycobacterium tuberculosis;	NCBI: 1773	4292032	99.94	0.11	Yes
NCBI Assembly	GCA_001378075.1	NCBI: cellular organisms; Bacteria; Terrabacteria group; Actinobacteria; Actinomycetia; Corynebacteriales; Mycobacteriaceae; Mycobacterium; Mycobacterium tuberculosis complex; Mycobacterium tuberculosis;	NCBI: 1773	4361858	99.94	0.00	Yes

NCBI Assembly	GCA_001216285.1	NCBI: cellular organisms; Bacteria; Terrabacteria group; Actinobacteria; Actinomycetia; Corynebacteriales; Mycobacteriaceae; Mycobacteroides; Mycobacteroides abscessus;	NCBI: 36809	7975112	100.00	100.00	No
NCBI Assembly	GCA_001099065.1	NCBI: cellular organisms; Bacteria; Terrabacteria group; Actinobacteria; Actinomycetia; Corynebacteriales; Mycobacteriaceae; Mycobacterium; Mycobacterium tuberculosis complex; Mycobacterium tuberculosis;	NCBI: 1773	4365776	99.94	0.00	Yes
NCBI Assembly	GCA_001121325.1	NCBI: cellular organisms; Bacteria; Terrabacteria group; Actinobacteria; Actinomycetia; Corynebacteriales; Mycobacteriaceae; Mycobacterium; Mycobacterium tuberculosis complex; Mycobacterium tuberculosis;	NCBI: 1773	4296648	89.46	0.00	No
NCBI Assembly	GCA_001196225.1	NCBI: cellular organisms; Bacteria; Terrabacteria group; Actinobacteria; Actinomycetia; Corynebacteriales; Mycobacteriaceae; Mycobacterium; Mycobacterium tuberculosis complex; Mycobacterium tuberculosis;	NCBI: 1773	4289688	99.61	0.67	Yes
NCBI Assembly	GCA_001209805.1	NCBI: cellular organisms; Bacteria; Terrabacteria group; Actinobacteria; Actinomycetia; Corynebacteriales; Mycobacteriaceae; Mycobacterium; Mycobacterium tuberculosis complex; Mycobacterium tuberculosis;	NCBI: 1773	4260152	93.99	0.00	Yes
NCBI Assembly	GCA_001379035.1	NCBI: cellular organisms; Bacteria; Terrabacteria group; Actinobacteria; Actinomycetia; Corynebacteriales; Mycobacteriaceae; Mycobacterium; Mycobacterium tuberculosis complex; Mycobacterium tuberculosis;	NCBI: 1773	4372453	99.94	0.00	Yes
NCBI Assembly	GCA_001318885.1	NCBI: cellular organisms; Bacteria; Terrabacteria group; Actinobacteria; Actinomycetia; Corynebacteriales; Mycobacteriaceae; Mycobacterium; Mycobacterium tuberculosis complex; Mycobacterium tuberculosis;	NCBI: 1773	4308126	99.53	1.81	Yes
NCBI Assembly	GCA_001327735.1	NCBI: cellular organisms; Bacteria; Terrabacteria group; Actinobacteria; Actinomycetia; Corynebacteriales; Mycobacteriaceae; Mycobacterium; Mycobacterium tuberculosis complex; Mycobacterium tuberculosis;	NCBI: 1773	4290555	99.94	0.00	Yes
NCBI Assembly	GCA_001376835.1	NCBI: cellular organisms; Bacteria; Terrabacteria group; Actinobacteria; Actinomycetia; Corynebacteriales; Mycobacteriaceae; Mycobacterium; Mycobacterium tuberculosis complex; Mycobacterium tuberculosis;	NCBI: 1773	4357876	99.94	0.00	Yes
NCBI Assembly	GCA_001380275.1	NCBI: cellular organisms; Bacteria; Terrabacteria group; Actinobacteria; Actinomycetia; Corynebacteriales; Mycobacteriaceae; Mycobacterium; Mycobacterium tuberculosis complex; Mycobacterium tuberculosis;	NCBI: 1773	4370034	99.94	0.00	Yes
NCBI Assembly	GCA_001198575.1	NCBI: cellular organisms; Bacteria; Terrabacteria group; Actinobacteria; Actinomycetia; Corynebacteriales; Mycobacteriaceae; Mycobacterium; Mycobacterium tuberculosis complex; Mycobacterium tuberculosis;	NCBI: 1773	4268105	96.93	0.83	Yes
NCBI Assembly	GCA_001378095.1	NCBI: cellular organisms; Bacteria; Terrabacteria group; Actinobacteria; Actinomycetia; Corynebacteriales; Mycobacteriaceae; Mycobacterium; Mycobacterium tuberculosis complex; Mycobacterium tuberculosis;	NCBI: 1773	4358039	99.94	0.00	Yes
NCBI Assembly	GCA_001216945.1	NCBI: cellular organisms; Bacteria; Terrabacteria group; Actinobacteria; Actinomycetia; Corynebacteriales; Mycobacteriaceae; Mycobacteroides; Mycobacteroides abscessus;	NCBI: 36809	9772395	99.31	45.50	No
NCBI Assembly	GCA_001099745.1	NCBI: cellular organisms; Bacteria; Terrabacteria group; Actinobacteria; Actinomycetia; Corynebacteriales; Mycobacteriaceae; Mycobacterium; Mycobacterium tuberculosis complex; Mycobacterium tuberculosis;	NCBI: 1773	4379165	99.94	0.00	Yes
NCBI Assembly	GCA_001121545.1	NCBI: cellular organisms; Bacteria; Terrabacteria group; Actinobacteria; Actinomycetia; Corynebacteriales; Mycobacteriaceae; Mycobacterium; Mycobacterium tuberculosis complex; Mycobacterium tuberculosis;	NCBI: 1773	4403033	99.94	0.00	Yes
NCBI Assembly	GCA_000954155.1	NCBI: cellular organisms; Bacteria; Terrabacteria group; Actinobacteria; Actinomycetia; Corynebacteriales; Mycobacteriaceae; Mycobacterium; Mycobacterium tuberculosis complex; Mycobacterium tuberculosis;	NCBI: 1773	4411216	98.96	0.00	Yes
NCBI Assembly	GCA_001166845.1	NCBI: cellular organisms; Bacteria; Terrabacteria group; Actinobacteria; Actinomycetia; Corynebacteriales; Mycobacteriaceae; Mycobacterium; Mycobacterium tuberculosis complex; Mycobacterium tuberculosis;	NCBI: 1773	4246095	99.28	0.33	Yes
NCBI Assembly	GCA_001314105.1	NCBI: cellular organisms; Bacteria; Terrabacteria group; Actinobacteria; Actinomycetia; Corynebacteriales; Mycobacteriaceae; Mycolicibacterium; Mycolicibacterium pyrenivorans;	NCBI: 1305860	5132161	47.74	1.86	No

NCBI Assembly	GCA_001325345.1	NCBI: cellular organisms; Bacteria; Terrabacteria group; Actinobacteria; Actinomycetia; Corynebacteriales; Mycobacteriaceae; Mycobacterium; Mycobacterium tuberculosis complex; Mycobacterium tuberculosis;	NCBI: 1773	4354123	99.94	0.00	Yes
NCBI Assembly	GCA_001377495.1	NCBI: cellular organisms; Bacteria; Terrabacteria group; Actinobacteria; Actinomycetia; Corynebacteriales; Mycobacteriaceae; Mycobacterium; Mycobacterium tuberculosis complex; Mycobacterium tuberculosis;	NCBI: 1773	4355397	99.94	0.00	Yes
NCBI Assembly	GCA_001322225.1	NCBI: cellular organisms; Bacteria; Terrabacteria group; Actinobacteria; Actinomycetia; Corynebacteriales; Mycobacteriaceae; Mycobacterium; Mycobacterium tuberculosis complex; Mycobacterium tuberculosis;	NCBI: 1773	4355441	99.94	0.00	Yes
NCBI Assembly	GCA_001088465.1	NCBI: cellular organisms; Bacteria; Terrabacteria group; Actinobacteria; Actinomycetia; Corynebacteriales; Mycobacteriaceae; Mycobacterium; Mycobacterium tuberculosis complex; Mycobacterium tuberculosis;	NCBI: 1773	4358066	99.94	0.00	Yes
NCBI Assembly	GCA_001380295.1	NCBI: cellular organisms; Bacteria; Terrabacteria group; Actinobacteria; Actinomycetia; Corynebacteriales; Mycobacteriaceae; Mycobacterium; Mycobacterium tuberculosis complex; Mycobacterium tuberculosis;	NCBI: 1773	4369688	99.94	0.00	Yes
NCBI Assembly	GCA_001198855.1	NCBI: cellular organisms; Bacteria; Terrabacteria group; Actinobacteria; Actinomycetia; Corynebacteriales; Mycobacteriaceae; Mycobacterium; Mycobacterium tuberculosis complex; Mycobacterium tuberculosis;	NCBI: 1773	4283163	98.98	2.41	Yes
NCBI Assembly	GCA_001296275.1	NCBI: cellular organisms; Bacteria; Terrabacteria group; Actinobacteria; Actinomycetia; Corynebacteriales; Mycobacteriaceae; Mycobacteroides; Mycobacteroides immunogenum;	NCBI: 83262	5719131	99.61	2.15	Yes
NCBI Assembly	GCA_001099945.1	NCBI: cellular organisms; Bacteria; Terrabacteria group; Actinobacteria; Actinomycetia; Corynebacteriales; Mycobacteriaceae; Mycobacterium; Mycobacterium tuberculosis complex; Mycobacterium tuberculosis;	NCBI: 1773	4262191	99.18	1.04	Yes
NCBI Assembly	GCA_001122345.1	NCBI: cellular organisms; Bacteria; Terrabacteria group; Actinobacteria; Actinomycetia; Corynebacteriales; Mycobacteriaceae; Mycobacterium; Mycobacterium tuberculosis complex; Mycobacterium tuberculosis;	NCBI: 1773	4360200	99.94	0.00	Yes
NCBI Assembly	GCA_000959045.1	NCBI: cellular organisms; Bacteria; Terrabacteria group; Actinobacteria; Actinomycetia; Corynebacteriales; Mycobacteriaceae; Mycolicibacterium; Mycolicibacterium neoaurum;	NCBI: 1795	5390529	99.62	1.44	Yes
NCBI Assembly	GCA_001166985.1	NCBI: cellular organisms; Bacteria; Terrabacteria group; Actinobacteria; Actinomycetia; Corynebacteriales; Mycobacteriaceae; Mycobacterium; Mycobacterium tuberculosis complex; Mycobacterium tuberculosis;	NCBI: 1773	4359919	99.94	0.33	Yes
NCBI Assembly	GCA_001314125.1	NCBI: cellular organisms; Bacteria; Terrabacteria group; Actinobacteria; Actinomycetia; Corynebacteriales; Mycobacteriaceae; Mycolicibacterium; Mycolicibacterium aromaticivorans;	NCBI: 1440774	5199648	55.84	1.29	No
NCBI Assembly	GCA_001322245.1	NCBI: cellular organisms; Bacteria; Terrabacteria group; Actinobacteria; Actinomycetia; Corynebacteriales; Mycobacteriaceae; Mycobacterium; Mycobacterium tuberculosis complex; Mycobacterium tuberculosis;	NCBI: 1773	4364619	99.94	0.00	Yes
NCBI Assembly	GCA_001088665.1	NCBI: cellular organisms; Bacteria; Terrabacteria group; Actinobacteria; Actinomycetia; Corynebacteriales; Mycobacteriaceae; Mycobacterium; Mycobacterium tuberculosis complex; Mycobacterium tuberculosis;	NCBI: 1773	4373026	99.94	0.00	Yes
NCBI Assembly	GCA_001325365.1	NCBI: cellular organisms; Bacteria; Terrabacteria group; Actinobacteria; Actinomycetia; Corynebacteriales; Mycobacteriaceae; Mycobacterium; Mycobacterium tuberculosis complex; Mycobacterium tuberculosis;	NCBI: 1773	4355767	99.90	0.00	Yes
NCBI Assembly	GCA_001199055.1	NCBI: cellular organisms; Bacteria; Terrabacteria group; Actinobacteria; Actinomycetia; Corynebacteriales; Mycobacteriaceae; Mycobacterium; Mycobacterium tuberculosis complex; Mycobacterium tuberculosis;	NCBI: 1773	1948694	34.99	5.63	No
NCBI Assembly	GCA_001296325.1	NCBI: cellular organisms; Bacteria; Terrabacteria group; Actinobacteria; Actinomycetia; Corynebacteriales; Mycobacteriaceae; Mycobacteroides; Mycobacteroides immunogenum;	NCBI: 83262	6063718	99.61	2.24	Yes
NCBI Assembly	GCA_001377515.1	NCBI: cellular organisms; Bacteria; Terrabacteria group; Actinobacteria; Actinomycetia; Corynebacteriales; Mycobacteriaceae; Mycobacterium; Mycobacterium tuberculosis complex; Mycobacterium tuberculosis;	NCBI: 1773	4387910	99.94	0.00	Yes

NCBI Assembly	GCA_001379355.1	NCBI: cellular organisms; Bacteria; Terrabacteria group; Actinobacteria; Actinomycetia; Corynebacteriales; Mycobacteriaceae; Mycobacterium; Mycobacterium tuberculosis complex; Mycobacterium tuberculosis;	NCBI: 1773	4357737	99.94	0.00	Yes
NCBI Assembly	GCA_001102605.1	NCBI: cellular organisms; Bacteria; Terrabacteria group; Actinobacteria; Actinomycetia; Corynebacteriales; Mycobacteriaceae; Mycobacterium; Mycobacterium tuberculosis complex; Mycobacterium tuberculosis;	NCBI: 1773	4360108	99.94	0.00	Yes
NCBI Assembly	GCA_001123765.1	NCBI: cellular organisms; Bacteria; Terrabacteria group; Actinobacteria; Actinomycetia; Corynebacteriales; Mycobacteriaceae; Mycobacterium; Mycobacterium tuberculosis complex; Mycobacterium tuberculosis;	NCBI: 1773	4383144	99.94	0.00	Yes
NCBI Assembly	GCA_000966355.1	NCBI: cellular organisms; Bacteria; Terrabacteria group; Actinobacteria; Actinomycetia; Corynebacteriales; Mycobacteriaceae; Mycobacterium; Mycobacterium lepromatosis;	NCBI: 480418	3206741	81.06	0.00	No
NCBI Assembly	GCA_001167185.1	NCBI: cellular organisms; Bacteria; Terrabacteria group; Actinobacteria; Actinomycetia; Corynebacteriales; Mycobacteriaceae; Mycobacterium; Mycobacterium tuberculosis complex; Mycobacterium tuberculosis;	NCBI: 1773	4210890	97.28	1.74	Yes
NCBI Assembly	GCA_001314145.1	NCBI: cellular organisms; Bacteria; Terrabacteria group; Actinobacteria; Actinomycetia; Corynebacteriales; Mycobacteriaceae; Mycolicibacterium; Mycolicibacterium crocinum;	NCBI: 1305862	5190569	57.40	1.96	No
NCBI Assembly	GCA_001090285.1	NCBI: cellular organisms; Bacteria; Terrabacteria group; Actinobacteria; Actinomycetia; Corynebacteriales; Mycobacteriaceae; Mycobacterium; Mycobacterium tuberculosis complex; Mycobacterium tuberculosis;	NCBI: 1773	4297587	98.94	0.00	Yes
NCBI Assembly	GCA_001322265.1	NCBI: cellular organisms; Bacteria; Terrabacteria group; Actinobacteria; Actinomycetia; Corynebacteriales; Mycobacteriaceae; Mycobacterium; Mycobacterium tuberculosis complex; Mycobacterium tuberculosis;	NCBI: 1773	4359900	99.94	0.00	Yes
NCBI Assembly	GCA_001296335.1	NCBI: cellular organisms; Bacteria; Terrabacteria group; Actinobacteria; Actinomycetia; Corynebacteriales; Mycobacteriaceae; Mycobacteroides; Mycobacteroides immunogenum;	NCBI: 83262	5682787	99.61	2.82	Yes
NCBI Assembly	GCA_001199275.1	NCBI: cellular organisms; Bacteria; Terrabacteria group; Actinobacteria; Actinomycetia; Corynebacteriales; Mycobacteriaceae; Mycobacterium; Mycobacterium tuberculosis complex; Mycobacterium tuberculosis;	NCBI: 1773	4305207	99.94	0.41	Yes
NCBI Assembly	GCA_001325385.1	NCBI: cellular organisms; Bacteria; Terrabacteria group; Actinobacteria; Actinomycetia; Corynebacteriales; Mycobacteriaceae; Mycobacterium; Mycobacterium tuberculosis complex; Mycobacterium tuberculosis;	NCBI: 1773	4352188	99.88	0.00	Yes
NCBI Assembly	GCA_001377535.1	NCBI: cellular organisms; Bacteria; Terrabacteria group; Actinobacteria; Actinomycetia; Corynebacteriales; Mycobacteriaceae; Mycobacterium; Mycobacterium tuberculosis complex; Mycobacterium tuberculosis;	NCBI: 1773	4362679	99.94	0.00	Yes
NCBI Assembly	GCA_001379375.1	NCBI: cellular organisms; Bacteria; Terrabacteria group; Actinobacteria; Actinomycetia; Corynebacteriales; Mycobacteriaceae; Mycobacterium; Mycobacterium tuberculosis complex; Mycobacterium tuberculosis;	NCBI: 1773	4365784	99.94	0.00	Yes
NCBI Assembly	GCA_001102685.1	NCBI: cellular organisms; Bacteria; Terrabacteria group; Actinobacteria; Actinomycetia; Corynebacteriales; Mycobacteriaceae; Mycobacterium; Mycobacterium tuberculosis complex; Mycobacterium tuberculosis;	NCBI: 1773	4366866	99.94	0.00	Yes
NCBI Assembly	GCA_001123785.1	NCBI: cellular organisms; Bacteria; Terrabacteria group; Actinobacteria; Actinomycetia; Corynebacteriales; Mycobacteriaceae; Mycobacterium; Mycobacterium tuberculosis complex; Mycobacterium tuberculosis;	NCBI: 1773	4337104	99.94	0.33	Yes
NCBI Assembly	GCA_001168405.1	NCBI: cellular organisms; Bacteria; Terrabacteria group; Actinobacteria; Actinomycetia; Corynebacteriales; Mycobacteriaceae; Mycobacterium; Mycobacterium tuberculosis complex; Mycobacterium tuberculosis;	NCBI: 1773	3836020	81.01	7.03	No
NCBI Assembly	GCA_001090305.1	NCBI: cellular organisms; Bacteria; Terrabacteria group; Actinobacteria; Actinomycetia; Corynebacteriales; Mycobacteriaceae; Mycobacterium; Mycobacterium tuberculosis complex; Mycobacterium tuberculosis;	NCBI: 1773	4378820	99.94	0.00	Yes
NCBI Assembly	GCA_001322285.1	NCBI: cellular organisms; Bacteria; Terrabacteria group; Actinobacteria; Actinomycetia; Corynebacteriales; Mycobacteriaceae; Mycobacterium; Mycobacterium tuberculosis complex; Mycobacterium tuberculosis;	NCBI: 1773	4364607	99.94	0.00	Yes

NCBI Assembly	GCA_001296345.1	NCBI: cellular organisms; Bacteria; Terrabacteria group; Actinobacteria; Actinomycetia; Corynebacteriales; Mycobacteriaceae; Mycobacteroides; Mycobacteroides immunogenum;	NCBI: 83262	5988570	99.61	2.24	Yes
NCBI Assembly	GCA_001325405.1	NCBI: cellular organisms; Bacteria; Terrabacteria group; Actinobacteria; Actinomycetia; Corynebacteriales; Mycobacteriaceae; Mycobacterium; Mycobacterium tuberculosis complex; Mycobacterium tuberculosis;	NCBI: 1773	4354519	99.94	0.00	Yes
NCBI Assembly	GCA_001377555.1	NCBI: cellular organisms; Bacteria; Terrabacteria group; Actinobacteria; Actinomycetia; Corynebacteriales; Mycobacteriaceae; Mycobacterium; Mycobacterium tuberculosis complex; Mycobacterium tuberculosis;	NCBI: 1773	4348236	99.61	0.00	Yes
NCBI Assembly	GCA_001200015.1	NCBI: cellular organisms; Bacteria; Terrabacteria group; Actinobacteria; Actinomycetia; Corynebacteriales; Mycobacteriaceae; Mycobacterium; Mycobacterium tuberculosis complex; Mycobacterium tuberculosis;	NCBI: 1773	4270750	99.08	1.45	Yes
NCBI Assembly	GCA_001379395.1	NCBI: cellular organisms; Bacteria; Terrabacteria group; Actinobacteria; Actinomycetia; Corynebacteriales; Mycobacteriaceae; Mycobacterium; Mycobacterium tuberculosis complex; Mycobacterium tuberculosis;	NCBI: 1773	4370419	99.94	0.00	Yes
NCBI Assembly	GCA_001102705.1	NCBI: cellular organisms; Bacteria; Terrabacteria group; Actinobacteria; Actinomycetia; Corynebacteriales; Mycobacteriaceae; Mycobacterium; Mycobacterium tuberculosis complex; Mycobacterium tuberculosis;	NCBI: 1773	4360120	99.94	0.00	Yes
NCBI Assembly	GCA_001125745.1	NCBI: cellular organisms; Bacteria; Terrabacteria group; Actinobacteria; Actinomycetia; Corynebacteriales; Mycobacteriaceae; Mycobacterium; Mycobacterium tuberculosis complex; Mycobacterium tuberculosis;	NCBI: 1773	4376731	99.94	0.00	Yes
NCBI Assembly	GCA_001168805.1	NCBI: cellular organisms; Bacteria; Terrabacteria group; Actinobacteria; Actinomycetia; Corynebacteriales; Mycobacteriaceae; Mycobacterium; Mycobacterium tuberculosis complex; Mycobacterium tuberculosis;	NCBI: 1773	4363442	99.94	0.00	Yes
NCBI Assembly	GCA_000970875.1	NCBI: cellular organisms; Bacteria; Terrabacteria group; Actinobacteria; Actinomycetia; Corynebacteriales; Mycobacteriaceae; Mycobacterium; Mycobacterium nebraskense;	NCBI: 244292	6460620	98.77	4.45	Yes
NCBI Assembly	GCA_001314185.1	NCBI: cellular organisms; Bacteria; Terrabacteria group; Actinobacteria; Actinomycetia; Corynebacteriales; Mycobacteriaceae; Mycolicibacterium; Mycolicibacterium rutilum;	NCBI: 1305864	3563602	37.49	1.44	No
NCBI Assembly	GCA_001090405.1	NCBI: cellular organisms; Bacteria; Terrabacteria group; Actinobacteria; Actinomycetia; Corynebacteriales; Mycobacteriaceae; Mycobacterium; Mycobacterium tuberculosis complex; Mycobacterium tuberculosis;	NCBI: 1773	4389635	99.94	0.00	Yes
NCBI Assembly	GCA_001200255.1	NCBI: cellular organisms; Bacteria; Terrabacteria group; Actinobacteria; Actinomycetia; Corynebacteriales; Mycobacteriaceae; Mycobacterium; Mycobacterium tuberculosis complex; Mycobacterium tuberculosis;	NCBI: 1773	4303264	98.94	0.50	Yes
NCBI Assembly	GCA_001322305.1	NCBI: cellular organisms; Bacteria; Terrabacteria group; Actinobacteria; Actinomycetia; Corynebacteriales; Mycobacteriaceae; Mycobacterium; Mycobacterium tuberculosis complex; Mycobacterium tuberculosis;	NCBI: 1773	4364191	99.94	0.00	Yes
NCBI Assembly	GCA_001296385.1	NCBI: cellular organisms; Bacteria; Terrabacteria group; Actinobacteria; Actinomycetia; Corynebacteriales; Mycobacteriaceae; Mycobacteroides; Mycobacteroides immunogenum;	NCBI: 83262	5860610	99.61	3.49	Yes
NCBI Assembly	GCA_001325425.1	NCBI: cellular organisms; Bacteria; Terrabacteria group; Actinobacteria; Actinomycetia; Corynebacteriales; Mycobacteriaceae; Mycobacterium; Mycobacterium tuberculosis complex; Mycobacterium tuberculosis;	NCBI: 1773	4356363	99.94	0.00	Yes
NCBI Assembly	GCA_001377575.1	NCBI: cellular organisms; Bacteria; Terrabacteria group; Actinobacteria; Actinomycetia; Corynebacteriales; Mycobacteriaceae; Mycobacterium; Mycobacterium tuberculosis complex; Mycobacterium tuberculosis;	NCBI: 1773	4348033	99.61	0.00	Yes
NCBI Assembly	GCA_001379415.1	NCBI: cellular organisms; Bacteria; Terrabacteria group; Actinobacteria; Actinomycetia; Corynebacteriales; Mycobacteriaceae; Mycobacterium; Mycobacterium tuberculosis complex; Mycobacterium tuberculosis;	NCBI: 1773	4363743	99.94	0.00	Yes
NCBI Assembly	GCA_001103285.1	NCBI: cellular organisms; Bacteria; Terrabacteria group; Actinobacteria; Actinomycetia; Corynebacteriales; Mycobacteriaceae; Mycobacterium; Mycobacterium tuberculosis complex; Mycobacterium tuberculosis;	NCBI: 1773	4303463	99.61	0.00	Yes

NCBI Assembly	GCA_001125785.1	NCBI: cellular organisms; Bacteria; Terrabacteria group; Actinobacteria; Actinomycetia; Corynebacteriales; Mycobacteriaceae; Mycobacterium; Mycobacterium tuberculosis complex; Mycobacterium tuberculosis;	NCBI: 1773	4364878	99.94	0.00	Yes
NCBI Assembly	GCA_000970885.2	NCBI: cellular organisms; Bacteria; Terrabacteria group; Actinobacteria; Actinomycetia; Corynebacteriales; Mycobacteriaceae; Mycolicibacter; Mycolicibacter arupensis;	NCBI: 342002	4441410	99.55	0.61	Yes
NCBI Assembly	GCA_001169425.1	NCBI: cellular organisms; Bacteria; Terrabacteria group; Actinobacteria; Actinomycetia; Corynebacteriales; Mycobacteriaceae; Mycobacterium; Mycobacterium tuberculosis complex; Mycobacterium tuberculosis;	NCBI: 1773	4389283	99.94	0.00	Yes
NCBI Assembly	GCA_001091605.1	NCBI: cellular organisms; Bacteria; Terrabacteria group; Actinobacteria; Actinomycetia; Corynebacteriales; Mycobacteriaceae; Mycobacterium; Mycobacterium tuberculosis complex; Mycobacterium tuberculosis;	NCBI: 1773	4266363	90.36	0.00	Yes
NCBI Assembly	GCA_001317365.1	NCBI: cellular organisms; Bacteria; Terrabacteria group; Actinobacteria; Actinomycetia; Corynebacteriales; Mycobacteriaceae; Mycobacterium; Mycobacterium tuberculosis complex; Mycobacterium tuberculosis;	NCBI: 1773	4505084	99.94	0.67	Yes
NCBI Assembly	GCA_001200395.1	NCBI: cellular organisms; Bacteria; Terrabacteria group; Actinobacteria; Actinomycetia; Corynebacteriales; Mycobacteriaceae; Mycobacterium; Mycobacterium tuberculosis complex; Mycobacterium tuberculosis;	NCBI: 1773	3209257	54.27	4.30	No
NCBI Assembly	GCA_001296405.1	NCBI: cellular organisms; Bacteria; Terrabacteria group; Actinobacteria; Actinomycetia; Corynebacteriales; Mycobacteriaceae; Mycobacteroides; Mycobacteroides immunogenum;	NCBI: 83262	5759306	99.61	2.15	Yes
NCBI Assembly	GCA_001322325.1	NCBI: cellular organisms; Bacteria; Terrabacteria group; Actinobacteria; Actinomycetia; Corynebacteriales; Mycobacteriaceae; Mycobacterium; Mycobacterium tuberculosis complex; Mycobacterium tuberculosis;	NCBI: 1773	4352338	99.94	0.00	Yes
NCBI Assembly	GCA_001325445.1	NCBI: cellular organisms; Bacteria; Terrabacteria group; Actinobacteria; Actinomycetia; Corynebacteriales; Mycobacteriaceae; Mycobacterium; Mycobacterium tuberculosis complex; Mycobacterium tuberculosis;	NCBI: 1773	4352865	99.94	0.00	Yes
NCBI Assembly	GCA_001377595.1	NCBI: cellular organisms; Bacteria; Terrabacteria group; Actinobacteria; Actinomycetia; Corynebacteriales; Mycobacteriaceae; Mycobacterium; Mycobacterium tuberculosis complex; Mycobacterium tuberculosis;	NCBI: 1773	4356291	99.94	0.00	Yes
NCBI Assembly	GCA_001103985.1	NCBI: cellular organisms; Bacteria; Terrabacteria group; Actinobacteria; Actinomycetia; Corynebacteriales; Mycobacteriaceae; Mycobacterium; Mycobacterium tuberculosis complex; Mycobacterium tuberculosis;	NCBI: 1773	4366567	99.94	0.00	Yes
NCBI Assembly	GCA_001379435.1	NCBI: cellular organisms; Bacteria; Terrabacteria group; Actinobacteria; Actinomycetia; Corynebacteriales; Mycobacteriaceae; Mycobacterium; Mycobacterium tuberculosis complex; Mycobacterium tuberculosis;	NCBI: 1773	4379372	99.94	0.00	Yes
NCBI Assembly	GCA_001126925.1	NCBI: cellular organisms; Bacteria; Terrabacteria group; Actinobacteria; Actinomycetia; Corynebacteriales; Mycobacteriaceae; Mycobacterium; Mycobacterium tuberculosis complex; Mycobacterium tuberculosis;	NCBI: 1773	4325492	99.61	0.00	Yes
NCBI Assembly	GCA_000971805.1	NCBI: cellular organisms; Bacteria; Terrabacteria group; Actinobacteria; Actinomycetia; Corynebacteriales; Mycobacteriaceae; Mycobacteroides; Mycobacteroides chelonae;	NCBI: 1774	4898027	98.83	0.03	Yes
NCBI Assembly	GCA_001169565.1	NCBI: cellular organisms; Bacteria; Terrabacteria group; Actinobacteria; Actinomycetia; Corynebacteriales; Mycobacteriaceae; Mycobacterium; Mycobacterium tuberculosis complex; Mycobacterium tuberculosis;	NCBI: 1773	4364550	99.94	0.33	Yes
NCBI Assembly	GCA_001091965.1	NCBI: cellular organisms; Bacteria; Terrabacteria group; Actinobacteria; Actinomycetia; Corynebacteriales; Mycobacteriaceae; Mycobacterium; Mycobacterium tuberculosis complex; Mycobacterium tuberculosis;	NCBI: 1773	4357899	99.94	0.00	Yes
NCBI Assembly	GCA_001200495.1	NCBI: cellular organisms; Bacteria; Terrabacteria group; Actinobacteria; Actinomycetia; Corynebacteriales; Mycobacteriaceae; Mycobacterium; Mycobacterium tuberculosis complex; Mycobacterium tuberculosis;	NCBI: 1773	4351336	99.90	0.00	Yes
NCBI Assembly	GCA_001317865.1	NCBI: cellular organisms; Bacteria; Terrabacteria group; Actinobacteria; Actinomycetia; Corynebacteriales; Mycobacteriaceae; Mycobacterium; Mycobacterium tuberculosis complex; Mycobacterium tuberculosis;	NCBI: 1773	4292115	99.94	2.04	Yes

NCBI Assembly	GCA_001296415.1	NCBI: cellular organisms; Bacteria; Terrabacteria group; Actinobacteria; Actinomycetia; Corynebacteriales; Mycobacteriaceae; Mycobacteroides; Mycobacteroides immunogenum;	NCBI: 83262	5810596	99.61	2.24	Yes
NCBI Assembly	GCA_001322345.1	NCBI: cellular organisms; Bacteria; Terrabacteria group; Actinobacteria; Actinomycetia; Corynebacteriales; Mycobacteriaceae; Mycobacterium; Mycobacterium tuberculosis complex; Mycobacterium tuberculosis;	NCBI: 1773	4360992	99.94	0.00	Yes
NCBI Assembly	GCA_001325465.1	NCBI: cellular organisms; Bacteria; Terrabacteria group; Actinobacteria; Actinomycetia; Corynebacteriales; Mycobacteriaceae; Mycobacterium; Mycobacterium tuberculosis complex; Mycobacterium tuberculosis;	NCBI: 1773	4350510	99.94	0.00	Yes
NCBI Assembly	GCA_001105365.1	NCBI: cellular organisms; Bacteria; Terrabacteria group; Actinobacteria; Actinomycetia; Corynebacteriales; Mycobacteriaceae; Mycobacterium; Mycobacterium tuberculosis complex; Mycobacterium tuberculosis;	NCBI: 1773	4113087	93.52	3.02	Yes
NCBI Assembly	GCA_001377615.1	NCBI: cellular organisms; Bacteria; Terrabacteria group; Actinobacteria; Actinomycetia; Corynebacteriales; Mycobacteriaceae; Mycobacterium; Mycobacterium tuberculosis complex; Mycobacterium tuberculosis;	NCBI: 1773	4355021	99.94	0.00	Yes
NCBI Assembly	GCA_001379455.1	NCBI: cellular organisms; Bacteria; Terrabacteria group; Actinobacteria; Actinomycetia; Corynebacteriales; Mycobacteriaceae; Mycobacterium; Mycobacterium tuberculosis complex; Mycobacterium tuberculosis;	NCBI: 1773	4390821	99.94	0.00	Yes
NCBI Assembly	GCA_001127945.1	NCBI: cellular organisms; Bacteria; Terrabacteria group; Actinobacteria; Actinomycetia; Corynebacteriales; Mycobacteriaceae; Mycobacterium; Mycobacterium tuberculosis complex; Mycobacterium tuberculosis;	NCBI: 1773	4284647	99.94	0.00	Yes
NCBI Assembly	GCA_000972185.1	NCBI: cellular organisms; Bacteria; Terrabacteria group; Actinobacteria; Actinomycetia; Corynebacteriales; Mycobacteriaceae; Mycobacterium; Mycobacterium tuberculosis complex; Mycobacterium tuberculosis;	NCBI: 1773	4294796	99.94	0.11	Yes
NCBI Assembly	GCA_001169865.1	NCBI: cellular organisms; Bacteria; Terrabacteria group; Actinobacteria; Actinomycetia; Corynebacteriales; Mycobacteriaceae; Mycobacterium; Mycobacterium tuberculosis complex; Mycobacterium tuberculosis;	NCBI: 1773	4237826	97.90	1.63	Yes
NCBI Assembly	GCA_001092505.1	NCBI: cellular organisms; Bacteria; Terrabacteria group; Actinobacteria; Actinomycetia; Corynebacteriales; Mycobacteriaceae; Mycobacterium; Mycobacterium tuberculosis complex; Mycobacterium tuberculosis;	NCBI: 1773	4382119	99.94	0.00	Yes
NCBI Assembly	GCA_001200555.1	NCBI: cellular organisms; Bacteria; Terrabacteria group; Actinobacteria; Actinomycetia; Corynebacteriales; Mycobacteriaceae; Mycobacterium; Mycobacterium tuberculosis complex; Mycobacterium tuberculosis;	NCBI: 1773	4272635	99.41	2.31	Yes
NCBI Assembly	GCA_001296425.1	NCBI: cellular organisms; Bacteria; Terrabacteria group; Actinobacteria; Actinomycetia; Corynebacteriales; Mycobacteriaceae; Mycobacteroides; Mycobacteroides immunogenum;	NCBI: 83262	5975867	99.61	2.24	Yes
NCBI Assembly	GCA_001317885.1	NCBI: cellular organisms; Bacteria; Terrabacteria group; Actinobacteria; Actinomycetia; Corynebacteriales; Mycobacteriaceae; Mycobacterium; Mycobacterium tuberculosis complex; Mycobacterium tuberculosis;	NCBI: 1773	4284736	99.57	1.40	Yes
NCBI Assembly	GCA_001325485.1	NCBI: cellular organisms; Bacteria; Terrabacteria group; Actinobacteria; Actinomycetia; Corynebacteriales; Mycobacteriaceae; Mycobacterium; Mycobacterium tuberculosis complex; Mycobacterium tuberculosis;	NCBI: 1773	4354012	99.94	0.00	Yes
NCBI Assembly	GCA_001322365.1	NCBI: cellular organisms; Bacteria; Terrabacteria group; Actinobacteria; Actinomycetia; Corynebacteriales; Mycobacteriaceae; Mycobacterium; Mycobacterium tuberculosis complex; Mycobacterium tuberculosis;	NCBI: 1773	4364114	99.94	0.00	Yes
NCBI Assembly	GCA_001379475.1	NCBI: cellular organisms; Bacteria; Terrabacteria group; Actinobacteria; Actinomycetia; Corynebacteriales; Mycobacteriaceae; Mycobacterium; Mycobacterium tuberculosis complex; Mycobacterium tuberculosis;	NCBI: 1773	4366561	99.94	0.00	Yes
NCBI Assembly	GCA_001105885.1	NCBI: cellular organisms; Bacteria; Terrabacteria group; Actinobacteria; Actinomycetia; Corynebacteriales; Mycobacteriaceae; Mycobacterium; Mycobacterium tuberculosis complex; Mycobacterium tuberculosis;	NCBI: 1773	4286996	87.18	0.33	No
NCBI Assembly	GCA_001377635.1	NCBI: cellular organisms; Bacteria; Terrabacteria group; Actinobacteria; Actinomycetia; Corynebacteriales; Mycobacteriaceae; Mycobacterium; Mycobacterium tuberculosis complex; Mycobacterium tuberculosis;	NCBI: 1773	4355263	99.94	0.00	Yes

NCBI Assembly	GCA_001128125.1	NCBI: cellular organisms; Bacteria; Terrabacteria group; Actinobacteria; Actinomycetia; Corynebacteriales; Mycobacteriaceae; Mycobacterium; Mycobacterium tuberculosis complex; Mycobacterium tuberculosis;	NCBI: 1773	4254175	89.41	0.50	No
NCBI Assembly	GCA_000972425.1	NCBI: cellular organisms; Bacteria; Terrabacteria group; Actinobacteria; Actinomycetia; Corynebacteriales; Mycobacteriaceae; Mycobacterium; Mycobacterium tuberculosis complex; Mycobacterium tuberculosis;	NCBI: 1773	4338492	99.94	0.00	Yes
NCBI Assembly	GCA_001170265.1	NCBI: cellular organisms; Bacteria; Terrabacteria group; Actinobacteria; Actinomycetia; Corynebacteriales; Mycobacteriaceae; Mycobacterium; Mycobacterium tuberculosis complex; Mycobacterium tuberculosis;	NCBI: 1773	4257423	98.29	1.91	Yes
NCBI Assembly	GCA_001093225.1	NCBI: cellular organisms; Bacteria; Terrabacteria group; Actinobacteria; Actinomycetia; Corynebacteriales; Mycobacteriaceae; Mycobacterium; Mycobacterium tuberculosis complex; Mycobacterium tuberculosis;	NCBI: 1773	4389051	99.94	0.33	Yes
NCBI Assembly	GCA_001201055.1	NCBI: cellular organisms; Bacteria; Terrabacteria group; Actinobacteria; Actinomycetia; Corynebacteriales; Mycobacteriaceae; Mycobacterium; Mycobacterium tuberculosis complex; Mycobacterium tuberculosis;	NCBI: 1773	4296972	97.85	0.00	Yes
NCBI Assembly	GCA_001317905.1	NCBI: cellular organisms; Bacteria; Terrabacteria group; Actinobacteria; Actinomycetia; Corynebacteriales; Mycobacteriaceae; Mycobacterium; Mycobacterium tuberculosis complex; Mycobacterium tuberculosis;	NCBI: 1773	4247239	98.86	1.72	Yes
NCBI Assembly	GCA_001307335.1	NCBI: cellular organisms; Bacteria; Terrabacteria group; Actinobacteria; Actinomycetia; Corynebacteriales; Mycobacteriaceae; Mycobacterium; Mycobacterium avium complex (MAC); Mycobacterium intracellulare; Mycobacterium intracellulare subsp. chimaera;	NCBI: 222805	6087047	99.94	1.39	Yes
NCBI Assembly	GCA_001105945.1	NCBI: cellular organisms; Bacteria; Terrabacteria group; Actinobacteria; Actinomycetia; Corynebacteriales; Mycobacteriaceae; Mycobacterium; Mycobacterium tuberculosis complex; Mycobacterium tuberculosis;	NCBI: 1773	4375414	99.94	0.00	Yes
NCBI Assembly	GCA_001325505.1	NCBI: cellular organisms; Bacteria; Terrabacteria group; Actinobacteria; Actinomycetia; Corynebacteriales; Mycobacteriaceae; Mycobacterium; Mycobacterium tuberculosis complex; Mycobacterium tuberculosis;	NCBI: 1773	4352236	99.94	0.00	Yes
NCBI Assembly	GCA_001322385.1	NCBI: cellular organisms; Bacteria; Terrabacteria group; Actinobacteria; Actinomycetia; Corynebacteriales; Mycobacteriaceae; Mycobacterium; Mycobacterium tuberculosis complex; Mycobacterium tuberculosis;	NCBI: 1773	4367274	99.94	0.00	Yes
NCBI Assembly	GCA_001377655.1	NCBI: cellular organisms; Bacteria; Terrabacteria group; Actinobacteria; Actinomycetia; Corynebacteriales; Mycobacteriaceae; Mycobacterium; Mycobacterium tuberculosis complex; Mycobacterium tuberculosis;	NCBI: 1773	4385520	99.94	0.00	Yes
NCBI Assembly	GCA_001379495.1	NCBI: cellular organisms; Bacteria; Terrabacteria group; Actinobacteria; Actinomycetia; Corynebacteriales; Mycobacteriaceae; Mycobacterium; Mycobacterium tuberculosis complex; Mycobacterium tuberculosis;	NCBI: 1773	4384240	99.94	0.00	Yes
NCBI Assembly	GCA_000972445.1	NCBI: cellular organisms; Bacteria; Terrabacteria group; Actinobacteria; Actinomycetia; Corynebacteriales; Mycobacteriaceae; Mycobacterium; Mycobacterium tuberculosis complex; Mycobacterium tuberculosis;	NCBI: 1773	4349175	99.94	0.07	Yes
NCBI Assembly	GCA_001128725.1	NCBI: cellular organisms; Bacteria; Terrabacteria group; Actinobacteria; Actinomycetia; Corynebacteriales; Mycobacteriaceae; Mycobacterium; Mycobacterium tuberculosis complex; Mycobacterium tuberculosis;	NCBI: 1773	4292881	99.94	0.00	Yes
NCBI Assembly	GCA_001201115.1	NCBI: cellular organisms; Bacteria; Terrabacteria group; Actinobacteria; Actinomycetia; Corynebacteriales; Mycobacteriaceae; Mycobacterium; Mycobacterium tuberculosis complex; Mycobacterium tuberculosis;	NCBI: 1773	4293318	99.94	1.11	Yes
NCBI Assembly	GCA_001170685.1	NCBI: cellular organisms; Bacteria; Terrabacteria group; Actinobacteria; Actinomycetia; Corynebacteriales; Mycobacteriaceae; Mycobacterium; Mycobacterium tuberculosis complex; Mycobacterium tuberculosis;	NCBI: 1773	4243296	99.91	1.00	Yes
NCBI Assembly	GCA_001093985.1	NCBI: cellular organisms; Bacteria; Terrabacteria group; Actinobacteria; Actinomycetia; Corynebacteriales; Mycobacteriaceae; Mycobacterium; Mycobacterium tuberculosis complex; Mycobacterium tuberculosis;	NCBI: 1773	3879620	85.46	3.01	No
NCBI Assembly	GCA_001307395.1	NCBI: cellular organisms; Bacteria; Terrabacteria group; Actinobacteria; Actinomycetia; Corynebacteriales; Mycobacteriaceae; Mycobacterium; Mycobacterium avium complex (MAC); Mycobacterium intracellulare; Mycobacterium intracellulare subsp. chimaera;	NCBI: 222805	6451412	99.94	1.15	Yes

NCBI Assembly	GCA_001317925.1	NCBI: cellular organisms; Bacteria; Terrabacteria group; Actinobacteria; Actinomycetia; Corynebacteriales; Mycobacteriaceae; Mycobacterium; Mycobacterium tuberculosis complex; Mycobacterium tuberculosis;	NCBI: 1773	4278482	98.91	1.00	Yes
NCBI Assembly	GCA_001106545.1	NCBI: cellular organisms; Bacteria; Terrabacteria group; Actinobacteria; Actinomycetia; Corynebacteriales; Mycobacteriaceae; Mycobacterium; Mycobacterium tuberculosis complex; Mycobacterium tuberculosis;	NCBI: 1773	4253981	98.44	1.41	Yes
NCBI Assembly	GCA_001325525.1	NCBI: cellular organisms; Bacteria; Terrabacteria group; Actinobacteria; Actinomycetia; Corynebacteriales; Mycobacteriaceae; Mycobacterium; Mycobacterium tuberculosis complex; Mycobacterium tuberculosis;	NCBI: 1773	4343460	99.94	0.00	Yes
NCBI Assembly	GCA_001379515.1	NCBI: cellular organisms; Bacteria; Terrabacteria group; Actinobacteria; Actinomycetia; Corynebacteriales; Mycobacteriaceae; Mycobacterium; Mycobacterium tuberculosis complex; Mycobacterium tuberculosis;	NCBI: 1773	4375754	99.94	0.00	Yes
NCBI Assembly	GCA_001377675.1	NCBI: cellular organisms; Bacteria; Terrabacteria group; Actinobacteria; Actinomycetia; Corynebacteriales; Mycobacteriaceae; Mycobacterium; Mycobacterium tuberculosis complex; Mycobacterium tuberculosis;	NCBI: 1773	4362303	99.94	0.00	Yes
NCBI Assembly	GCA_001322405.1	NCBI: cellular organisms; Bacteria; Terrabacteria group; Actinobacteria; Actinomycetia; Corynebacteriales; Mycobacteriaceae; Mycobacterium; Mycobacterium tuberculosis complex; Mycobacterium tuberculosis;	NCBI: 1773	4367353	99.94	0.00	Yes
NCBI Assembly	GCA_000972465.1	NCBI: cellular organisms; Bacteria; Terrabacteria group; Actinobacteria; Actinomycetia; Corynebacteriales; Mycobacteriaceae; Mycobacterium; Mycobacterium tuberculosis complex; Mycobacterium tuberculosis;	NCBI: 1773	4358280	99.94	0.00	Yes
NCBI Assembly	GCA_001128805.1	NCBI: cellular organisms; Bacteria; Terrabacteria group; Actinobacteria; Actinomycetia; Corynebacteriales; Mycobacteriaceae; Mycobacterium; Mycobacterium tuberculosis complex; Mycobacterium tuberculosis;	NCBI: 1773	4426726	99.94	0.33	Yes
NCBI Assembly	GCA_001172005.1	NCBI: cellular organisms; Bacteria; Terrabacteria group; Actinobacteria; Actinomycetia; Corynebacteriales; Mycobacteriaceae; Mycobacterium; Mycobacterium tuberculosis complex; Mycobacterium tuberculosis;	NCBI: 1773	4363137	99.94	0.00	Yes
NCBI Assembly	GCA_001094405.1	NCBI: cellular organisms; Bacteria; Terrabacteria group; Actinobacteria; Actinomycetia; Corynebacteriales; Mycobacteriaceae; Mycobacterium; Mycobacterium tuberculosis complex; Mycobacterium tuberculosis;	NCBI: 1773	4356592	99.94	0.00	Yes
NCBI Assembly	GCA_001201335.1	NCBI: cellular organisms; Bacteria; Terrabacteria group; Actinobacteria; Actinomycetia; Corynebacteriales; Mycobacteriaceae; Mycobacterium; Mycobacterium tuberculosis complex; Mycobacterium tuberculosis;	NCBI: 1773	4267055	99.41	1.87	Yes
NCBI Assembly	GCA_001307415.1	NCBI: cellular organisms; Bacteria; Terrabacteria group; Actinobacteria; Actinomycetia; Corynebacteriales; Mycobacteriaceae; Mycobacterium; Mycobacterium avium complex (MAC); Mycobacterium intracellulare; Mycobacterium intracellulare subsp. chimaera;	NCBI: 222805	6020776	99.94	1.04	Yes
NCBI Assembly	GCA_001317945.1	NCBI: cellular organisms; Bacteria; Terrabacteria group; Actinobacteria; Actinomycetia; Corynebacteriales; Mycobacteriaceae; Mycobacterium; Mycobacterium tuberculosis complex; Mycobacterium tuberculosis;	NCBI: 1773	4241855	99.56	0.93	Yes
NCBI Assembly	GCA_001107145.1	NCBI: cellular organisms; Bacteria; Terrabacteria group; Actinobacteria; Actinomycetia; Corynebacteriales; Mycobacteriaceae; Mycobacterium; Mycobacterium tuberculosis complex; Mycobacterium tuberculosis;	NCBI: 1773	4366105	99.94	0.00	Yes
NCBI Assembly	GCA_001325545.1	NCBI: cellular organisms; Bacteria; Terrabacteria group; Actinobacteria; Actinomycetia; Corynebacteriales; Mycobacteriaceae; Mycobacterium; Mycobacterium tuberculosis complex; Mycobacterium tuberculosis;	NCBI: 1773	4389948	99.94	0.00	Yes
NCBI Assembly	GCA_000972905.1	NCBI: cellular organisms; Bacteria; Terrabacteria group; Actinobacteria; Actinomycetia; Corynebacteriales; Mycobacteriaceae; Mycobacterium; unclassified Mycobacterium;	NCBI: 1545693	4505725	99.09	1.15	Yes
NCBI Assembly	GCA_001322425.1	NCBI: cellular organisms; Bacteria; Terrabacteria group; Actinobacteria; Actinomycetia; Corynebacteriales; Mycobacteriaceae; Mycobacterium; Mycobacterium tuberculosis complex; Mycobacterium tuberculosis;	NCBI: 1773	4361211	99.94	0.00	Yes
NCBI Assembly	GCA_001377695.1	NCBI: cellular organisms; Bacteria; Terrabacteria group; Actinobacteria; Actinomycetia; Corynebacteriales; Mycobacteriaceae; Mycobacterium; Mycobacterium tuberculosis complex; Mycobacterium tuberculosis;	NCBI: 1773	4393671	99.94	0.00	Yes

NCBI Assembly	GCA_001379535.1	NCBI: cellular organisms; Bacteria; Terrabacteria group; Actinobacteria; Actinomycetia; Corynebacteriales; Mycobacteriaceae; Mycobacterium; Mycobacterium tuberculosis complex; Mycobacterium tuberculosis;	NCBI: 1773	4368254	99.94	0.00	Yes
NCBI Assembly	GCA_001128865.1	NCBI: cellular organisms; Bacteria; Terrabacteria group; Actinobacteria; Actinomycetia; Corynebacteriales; Mycobacteriaceae; Mycobacterium; Mycobacterium tuberculosis complex; Mycobacterium tuberculosis;	NCBI: 1773	4356910	99.94	0.00	Yes
NCBI Assembly	GCA_001096245.1	NCBI: cellular organisms; Bacteria; Terrabacteria group; Actinobacteria; Actinomycetia; Corynebacteriales; Mycobacteriaceae; Mycobacterium; Mycobacterium tuberculosis complex; Mycobacterium tuberculosis;	NCBI: 1773	4269577	98.99	2.36	Yes
NCBI Assembly	GCA_001172585.1	NCBI: cellular organisms; Bacteria; Terrabacteria group; Actinobacteria; Actinomycetia; Corynebacteriales; Mycobacteriaceae; Mycobacterium; Mycobacterium tuberculosis complex; Mycobacterium tuberculosis;	NCBI: 1773	4327860	99.94	0.00	Yes
NCBI Assembly	GCA_001201495.1	NCBI: cellular organisms; Bacteria; Terrabacteria group; Actinobacteria; Actinomycetia; Corynebacteriales; Mycobacteriaceae; Mycobacterium; Mycobacterium tuberculosis complex; Mycobacterium tuberculosis;	NCBI: 1773	4267410	96.58	0.83	Yes
NCBI Assembly	GCA_001108845.1	NCBI: cellular organisms; Bacteria; Terrabacteria group; Actinobacteria; Actinomycetia; Corynebacteriales; Mycobacteriaceae; Mycobacterium; Mycobacterium tuberculosis complex; Mycobacterium tuberculosis;	NCBI: 1773	3935636	88.26	5.44	No
NCBI Assembly	GCA_001317965.1	NCBI: cellular organisms; Bacteria; Terrabacteria group; Actinobacteria; Actinomycetia; Corynebacteriales; Mycobacteriaceae; Mycobacterium; Mycobacterium tuberculosis complex; Mycobacterium tuberculosis;	NCBI: 1773	4272097	99.23	0.83	Yes
NCBI Assembly	GCA_000972915.1	NCBI: cellular organisms; Bacteria; Terrabacteria group; Actinobacteria; Actinomycetia; Corynebacteriales; Mycobacteriaceae; Mycobacterium; unclassified Mycobacterium;	NCBI: 1545692	4237839	99.09	0.61	Yes
NCBI Assembly	GCA_001325565.1	NCBI: cellular organisms; Bacteria; Terrabacteria group; Actinobacteria; Actinomycetia; Corynebacteriales; Mycobacteriaceae; Mycobacterium; Mycobacterium tuberculosis complex; Mycobacterium tuberculosis;	NCBI: 1773	4351633	99.90	0.00	Yes
NCBI Assembly	GCA_001322445.1	NCBI: cellular organisms; Bacteria; Terrabacteria group; Actinobacteria; Actinomycetia; Corynebacteriales; Mycobacteriaceae; Mycobacterium; Mycobacterium tuberculosis complex; Mycobacterium tuberculosis;	NCBI: 1773	4355876	99.94	0.00	Yes
NCBI Assembly	GCA_001377715.1	NCBI: cellular organisms; Bacteria; Terrabacteria group; Actinobacteria; Actinomycetia; Corynebacteriales; Mycobacteriaceae; Mycobacterium; Mycobacterium tuberculosis complex; Mycobacterium tuberculosis;	NCBI: 1773	4358764	99.94	0.00	Yes
NCBI Assembly	GCA_001379555.1	NCBI: cellular organisms; Bacteria; Terrabacteria group; Actinobacteria; Actinomycetia; Corynebacteriales; Mycobacteriaceae; Mycobacterium; Mycobacterium tuberculosis complex; Mycobacterium tuberculosis;	NCBI: 1773	4368803	99.94	0.00	Yes
NCBI Assembly	GCA_001130685.1	NCBI: cellular organisms; Bacteria; Terrabacteria group; Actinobacteria; Actinomycetia; Corynebacteriales; Mycobacteriaceae; Mycobacterium; Mycobacterium tuberculosis complex; Mycobacterium tuberculosis;	NCBI: 1773	4363754	99.94	0.00	Yes
NCBI Assembly	GCA_001096785.1	NCBI: cellular organisms; Bacteria; Terrabacteria group; Actinobacteria; Actinomycetia; Corynebacteriales; Mycobacteriaceae; Mycobacterium; Mycobacterium tuberculosis complex; Mycobacterium tuberculosis;	NCBI: 1773	4251107	99.94	0.00	Yes
NCBI Assembly	GCA_001201655.1	NCBI: cellular organisms; Bacteria; Terrabacteria group; Actinobacteria; Actinomycetia; Corynebacteriales; Mycobacteriaceae; Mycobacterium; Mycobacterium tuberculosis complex; Mycobacterium tuberculosis;	NCBI: 1773	4307734	99.78	0.33	Yes
NCBI Assembly	GCA_001172865.1	NCBI: cellular organisms; Bacteria; Terrabacteria group; Actinobacteria; Actinomycetia; Corynebacteriales; Mycobacteriaceae; Mycobacterium; Mycobacterium tuberculosis complex; Mycobacterium tuberculosis;	NCBI: 1773	4361439	99.94	0.00	Yes
NCBI Assembly	GCA_001109345.1	NCBI: cellular organisms; Bacteria; Terrabacteria group; Actinobacteria; Actinomycetia; Corynebacteriales; Mycobacteriaceae; Mycobacterium; Mycobacterium tuberculosis complex; Mycobacterium tuberculosis;	NCBI: 1773	4361571	99.94	0.00	Yes
NCBI Assembly	GCA_000972925.1	NCBI: cellular organisms; Bacteria; Terrabacteria group; Actinobacteria; Actinomycetia; Corynebacteriales; Mycobacteriaceae; Mycobacterium; unclassified Mycobacterium;	NCBI: 1545691	4517831	98.64	1.33	Yes

NCBI Assembly	GCA_001317985.1	NCBI: cellular organisms; Bacteria; Terrabacteria group; Actinobacteria; Actinomycetia; Corynebacteriales; Mycobacteriaceae; Mycobacterium; Mycobacterium tuberculosis complex; Mycobacterium tuberculosis;	NCBI: 1773	4279454	98.83	1.86	Yes
NCBI Assembly	GCA_001313385.1	NCBI: cellular organisms; Bacteria; Terrabacteria group; Actinobacteria; Actinomycetia; Corynebacteriales; Mycobacteriaceae; Mycolicibacterium; Mycolicibacterium flavescens;	NCBI: 1303678	5507867	40.78	0.75	No
NCBI Assembly	GCA_001325585.1	NCBI: cellular organisms; Bacteria; Terrabacteria group; Actinobacteria; Actinomycetia; Corynebacteriales; Mycobacteriaceae; Mycobacterium; Mycobacterium tuberculosis complex; Mycobacterium tuberculosis;	NCBI: 1773	4314447	99.94	0.00	Yes
NCBI Assembly	GCA_001322465.1	NCBI: cellular organisms; Bacteria; Terrabacteria group; Actinobacteria; Actinomycetia; Corynebacteriales; Mycobacteriaceae; Mycobacterium; Mycobacterium tuberculosis complex; Mycobacterium tuberculosis;	NCBI: 1773	4366633	99.94	0.00	Yes
NCBI Assembly	GCA_001377735.1	NCBI: cellular organisms; Bacteria; Terrabacteria group; Actinobacteria; Actinomycetia; Corynebacteriales; Mycobacteriaceae; Mycobacterium; Mycobacterium tuberculosis complex; Mycobacterium tuberculosis;	NCBI: 1773	4385243	99.94	0.00	Yes
NCBI Assembly	GCA_001379575.1	NCBI: cellular organisms; Bacteria; Terrabacteria group; Actinobacteria; Actinomycetia; Corynebacteriales; Mycobacteriaceae; Mycobacterium; Mycobacterium tuberculosis complex; Mycobacterium tuberculosis;	NCBI: 1773	4355555	99.94	0.00	Yes
NCBI Assembly	GCA_001131705.1	NCBI: cellular organisms; Bacteria; Terrabacteria group; Actinobacteria; Actinomycetia; Corynebacteriales; Mycobacteriaceae; Mycobacterium; Mycobacterium tuberculosis complex; Mycobacterium tuberculosis;	NCBI: 1773	4362075	99.94	0.00	Yes
NCBI Assembly	GCA_001096945.1	NCBI: cellular organisms; Bacteria; Terrabacteria group; Actinobacteria; Actinomycetia; Corynebacteriales; Mycobacteriaceae; Mycobacterium; Mycobacterium tuberculosis complex; Mycobacterium tuberculosis;	NCBI: 1773	4383737	99.94	0.00	Yes
NCBI Assembly	GCA_001173105.1	NCBI: cellular organisms; Bacteria; Terrabacteria group; Actinobacteria; Actinomycetia; Corynebacteriales; Mycobacteriaceae; Mycobacterium; Mycobacterium tuberculosis complex; Mycobacterium tuberculosis;	NCBI: 1773	4359367	99.94	0.00	Yes
NCBI Assembly	GCA_001201895.1	NCBI: cellular organisms; Bacteria; Terrabacteria group; Actinobacteria; Actinomycetia; Corynebacteriales; Mycobacteriaceae; Mycobacterium; Mycobacterium tuberculosis complex; Mycobacterium tuberculosis;	NCBI: 1773	4288192	99.94	1.00	Yes
NCBI Assembly	GCA_000972935.1	NCBI: cellular organisms; Bacteria; Terrabacteria group; Actinobacteria; Actinomycetia; Corynebacteriales; Mycobacteriaceae; Mycobacterium; unclassified Mycobacterium;	NCBI: 1545694	5067383	100.00	2.58	Yes
NCBI Assembly	GCA_001110005.1	NCBI: cellular organisms; Bacteria; Terrabacteria group; Actinobacteria; Actinomycetia; Corynebacteriales; Mycobacteriaceae; Mycobacterium; Mycobacterium tuberculosis complex; Mycobacterium tuberculosis;	NCBI: 1773	4302996	99.25	0.00	Yes
NCBI Assembly	GCA_001313545.1	NCBI: cellular organisms; Bacteria; Terrabacteria group; Actinobacteria; Actinomycetia; Corynebacteriales; Mycobacteriaceae; Mycolicibacterium; Mycolicibacterium chlorophenolicum;	NCBI: 1303685	4296785	40.99	1.31	No
NCBI Assembly	GCA_001318005.1	NCBI: cellular organisms; Bacteria; Terrabacteria group; Actinobacteria; Actinomycetia; Corynebacteriales; Mycobacteriaceae; Mycobacterium; Mycobacterium tuberculosis complex; Mycobacterium tuberculosis;	NCBI: 1773	4329482	99.67	0.00	Yes
NCBI Assembly	GCA_001325605.1	NCBI: cellular organisms; Bacteria; Terrabacteria group; Actinobacteria; Actinomycetia; Corynebacteriales; Mycobacteriaceae; Mycobacterium; Mycobacterium tuberculosis complex; Mycobacterium tuberculosis;	NCBI: 1773	4376995	99.94	0.15	Yes
NCBI Assembly	GCA_001322485.1	NCBI: cellular organisms; Bacteria; Terrabacteria group; Actinobacteria; Actinomycetia; Corynebacteriales; Mycobacteriaceae; Mycobacterium; Mycobacterium tuberculosis complex; Mycobacterium tuberculosis;	NCBI: 1773	4397462	99.94	0.00	Yes
NCBI Assembly	GCA_001377755.1	NCBI: cellular organisms; Bacteria; Terrabacteria group; Actinobacteria; Actinomycetia; Corynebacteriales; Mycobacteriaceae; Mycobacterium; Mycobacterium tuberculosis complex; Mycobacterium tuberculosis;	NCBI: 1773	4394407	99.94	0.00	Yes
NCBI Assembly	GCA_001131785.1	NCBI: cellular organisms; Bacteria; Terrabacteria group; Actinobacteria; Actinomycetia; Corynebacteriales; Mycobacteriaceae; Mycobacterium; Mycobacterium tuberculosis complex; Mycobacterium tuberculosis;	NCBI: 1773	4366692	99.94	0.00	Yes

NCBI Assembly	GCA_001379595.1	NCBI: cellular organisms; Bacteria; Terrabacteria group; Actinobacteria; Actinomycetia; Corynebacteriales; Mycobacteriaceae; Mycobacterium; Mycobacterium tuberculosis complex; Mycobacterium tuberculosis;	NCBI: 1773	4371645	99.94	0.00	Yes
NCBI Assembly	GCA_001097325.1	NCBI: cellular organisms; Bacteria; Terrabacteria group; Actinobacteria; Actinomycetia; Corynebacteriales; Mycobacteriaceae; Mycobacterium; Mycobacterium tuberculosis complex; Mycobacterium tuberculosis;	NCBI: 1773	4366948	99.94	0.00	Yes
NCBI Assembly	GCA_001201915.1	NCBI: cellular organisms; Bacteria; Terrabacteria group; Actinobacteria; Actinomycetia; Corynebacteriales; Mycobacteriaceae; Mycobacterium; Mycobacterium tuberculosis complex; Mycobacterium tuberculosis;	NCBI: 1773	4283118	99.28	2.07	Yes
NCBI Assembly	GCA_001173265.1	NCBI: cellular organisms; Bacteria; Terrabacteria group; Actinobacteria; Actinomycetia; Corynebacteriales; Mycobacteriaceae; Mycobacterium; Mycobacterium tuberculosis complex; Mycobacterium tuberculosis;	NCBI: 1773	4326573	99.05	0.00	Yes
NCBI Assembly	GCA_001111525.1	NCBI: cellular organisms; Bacteria; Terrabacteria group; Actinobacteria; Actinomycetia; Corynebacteriales; Mycobacteriaceae; Mycobacterium; Mycobacterium tuberculosis complex; Mycobacterium tuberculosis;	NCBI: 1773	4359290	99.94	0.00	Yes
NCBI Assembly	GCA_000974705.1	NCBI: cellular organisms; Bacteria; Terrabacteria group; Actinobacteria; Actinomycetia; Corynebacteriales; Mycobacteriaceae; Mycobacterium; Mycobacterium celatum;	NCBI: 28045	4662053	95.09	3.25	Yes
NCBI Assembly	GCA_001313805.1	NCBI: cellular organisms; Bacteria; Terrabacteria group; Actinobacteria; Actinomycetia; Corynebacteriales; Mycobacteriaceae; Mycolicibacterium; Mycolicibacterium hodleri;	NCBI: 1305625	3874367	37.86	0.66	No
NCBI Assembly	GCA_001318025.1	NCBI: cellular organisms; Bacteria; Terrabacteria group; Actinobacteria; Actinomycetia; Corynebacteriales; Mycobacteriaceae; Mycobacterium; Mycobacterium tuberculosis complex; Mycobacterium tuberculosis;	NCBI: 1773	4271699	98.97	0.33	Yes
NCBI Assembly	GCA_001325625.1	NCBI: cellular organisms; Bacteria; Terrabacteria group; Actinobacteria; Actinomycetia; Corynebacteriales; Mycobacteriaceae; Mycobacterium; Mycobacterium tuberculosis complex; Mycobacterium tuberculosis;	NCBI: 1773	4354956	99.94	0.00	Yes
NCBI Assembly	GCA_001322505.1	NCBI: cellular organisms; Bacteria; Terrabacteria group; Actinobacteria; Actinomycetia; Corynebacteriales; Mycobacteriaceae; Mycobacterium; Mycobacterium tuberculosis complex; Mycobacterium tuberculosis;	NCBI: 1773	4364336	99.94	0.00	Yes
NCBI Assembly	GCA_001377775.1	NCBI: cellular organisms; Bacteria; Terrabacteria group; Actinobacteria; Actinomycetia; Corynebacteriales; Mycobacteriaceae; Mycobacterium; Mycobacterium tuberculosis complex; Mycobacterium tuberculosis;	NCBI: 1773	4354714	99.94	0.00	Yes
NCBI Assembly	GCA_001318045.1	NCBI: cellular organisms; Bacteria; Terrabacteria group; Actinobacteria; Actinomycetia; Corynebacteriales; Mycobacteriaceae; Mycobacterium; Mycobacterium tuberculosis complex; Mycobacterium tuberculosis;	NCBI: 1773	4305928	99.28	0.33	Yes
NCBI Assembly	GCA_001097745.1	NCBI: cellular organisms; Bacteria; Terrabacteria group; Actinobacteria; Actinomycetia; Corynebacteriales; Mycobacteriaceae; Mycobacterium; Mycobacterium tuberculosis complex; Mycobacterium tuberculosis;	NCBI: 1773	4252214	99.91	0.00	Yes
NCBI Assembly	GCA_001379615.1	NCBI: cellular organisms; Bacteria; Terrabacteria group; Actinobacteria; Actinomycetia; Corynebacteriales; Mycobacteriaceae; Mycobacterium; Mycobacterium tuberculosis complex; Mycobacterium tuberculosis;	NCBI: 1773	4364904	99.94	0.17	Yes
NCBI Assembly	GCA_001196295.1	NCBI: cellular organisms; Bacteria; Terrabacteria group; Actinobacteria; Actinomycetia; Corynebacteriales; Mycobacteriaceae; Mycobacterium; Mycobacterium tuberculosis complex; Mycobacterium tuberculosis;	NCBI: 1773	4155644	85.22	0.53	No
NCBI Assembly	GCA_001174265.1	NCBI: cellular organisms; Bacteria; Terrabacteria group; Actinobacteria; Actinomycetia; Corynebacteriales; Mycobacteriaceae; Mycobacterium; Mycobacterium tuberculosis complex; Mycobacterium tuberculosis;	NCBI: 1773	4362169	99.94	0.00	Yes
NCBI Assembly	GCA_001111885.1	NCBI: cellular organisms; Bacteria; Terrabacteria group; Actinobacteria; Actinomycetia; Corynebacteriales; Mycobacteriaceae; Mycobacterium; Mycobacterium tuberculosis complex; Mycobacterium tuberculosis;	NCBI: 1773	4362537	99.94	0.00	Yes
NCBI Assembly	GCA_000974925.2	NCBI: cellular organisms; Bacteria; Terrabacteria group; Actinobacteria; Actinomycetia; Corynebacteriales; Mycobacteriaceae; Mycolicibacterium; Mycolicibacterium obuense;	NCBI: 1807	6381766	99.19	1.68	Yes

NCBI Assembly	GCA_001287125.1	NCBI: cellular organisms; Bacteria; Terrabacteria group; Actinobacteria; Actinomycetia; Corynebacteriales; Mycobacteriaceae; Mycobacterium; Mycobacterium tuberculosis complex; Mycobacterium tuberculosis; Mycobacterium tuberculosis variant bovis; [illegible]	NCBI: 33892	4294196	99.78	0.00	Yes
NCBI Assembly	GCA_001313925.1	NCBI: cellular organisms; Bacteria; Terrabacteria group; Actinobacteria; Actinomycetia; Corynebacteriales; Mycobacteriaceae; Mycolicibacterium; Mycolicibacterium rufum;	NCBI: 1305629	3814631	52.54	0.62	No
NCBI Assembly	GCA_001322525.1	NCBI: cellular organisms; Bacteria; Terrabacteria group; Actinobacteria; Actinomycetia; Corynebacteriales; Mycobacteriaceae; Mycobacterium; Mycobacterium tuberculosis complex; Mycobacterium tuberculosis;	NCBI: 1773	4328921	99.94	1.04	Yes
NCBI Assembly	GCA_001325645.1	NCBI: cellular organisms; Bacteria; Terrabacteria group; Actinobacteria; Actinomycetia; Corynebacteriales; Mycobacteriaceae; Mycobacterium; Mycobacterium tuberculosis complex; Mycobacterium tuberculosis;	NCBI: 1773	4365850	99.94	0.00	Yes
NCBI Assembly	GCA_001377795.1	NCBI: cellular organisms; Bacteria; Terrabacteria group; Actinobacteria; Actinomycetia; Corynebacteriales; Mycobacteriaceae; Mycobacterium; Mycobacterium tuberculosis complex; Mycobacterium tuberculosis;	NCBI: 1773	4366345	99.94	0.00	Yes
NCBI Assembly	GCA_001318065.1	NCBI: cellular organisms; Bacteria; Terrabacteria group; Actinobacteria; Actinomycetia; Corynebacteriales; Mycobacteriaceae; Mycobacterium; Mycobacterium tuberculosis complex; Mycobacterium tuberculosis;	NCBI: 1773	3740402	79.25	8.31	No
NCBI Assembly	GCA_001196315.1	NCBI: cellular organisms; Bacteria; Terrabacteria group; Actinobacteria; Actinomycetia; Corynebacteriales; Mycobacteriaceae; Mycobacterium; Mycobacterium tuberculosis complex; Mycobacterium tuberculosis;	NCBI: 1773	4294725	98.74	0.00	Yes
NCBI Assembly	GCA_001379055.1	NCBI: cellular organisms; Bacteria; Terrabacteria group; Actinobacteria; Actinomycetia; Corynebacteriales; Mycobacteriaceae; Mycobacterium; Mycobacterium tuberculosis complex; Mycobacterium tuberculosis;	NCBI: 1773	4363576	99.94	0.00	Yes
NCBI Assembly	GCA_001379635.1	NCBI: cellular organisms; Bacteria; Terrabacteria group; Actinobacteria; Actinomycetia; Corynebacteriales; Mycobacteriaceae; Mycobacterium; Mycobacterium tuberculosis complex; Mycobacterium tuberculosis;	NCBI: 1773	4375565	99.94	0.00	Yes
NCBI Assembly	GCA_001155865.1	NCBI: cellular organisms; Bacteria; Terrabacteria group; Actinobacteria; Actinomycetia; Corynebacteriales; Mycobacteriaceae; Mycobacterium; Mycobacterium tuberculosis complex; Mycobacterium tuberculosis;	NCBI: 1773	4371875	99.94	0.00	Yes
NCBI Assembly	GCA_001112325.1	NCBI: cellular organisms; Bacteria; Terrabacteria group; Actinobacteria; Actinomycetia; Corynebacteriales; Mycobacteriaceae; Mycobacterium; Mycobacterium tuberculosis complex; Mycobacterium tuberculosis;	NCBI: 1773	4413310	99.94	0.00	Yes
NCBI Assembly	GCA_000975265.1	NCBI: cellular organisms; Bacteria; Terrabacteria group; Actinobacteria; Actinomycetia; Corynebacteriales; Mycobacteriaceae; Mycobacterium; Mycobacterium lepromatosis;	NCBI: 480418	3215823	77.34	0.33	No
NCBI Assembly	GCA_001324085.1	NCBI: cellular organisms; Bacteria; Terrabacteria group; Actinobacteria; Actinomycetia; Corynebacteriales; Mycobacteriaceae; Mycobacterium; Mycobacterium tuberculosis complex; Mycobacterium tuberculosis;	NCBI: 1773	4369745	99.94	0.00	Yes
NCBI Assembly	GCA_001287165.1	NCBI: cellular organisms; Bacteria; Terrabacteria group; Actinobacteria; Actinomycetia; Corynebacteriales; Mycobacteriaceae; Mycobacterium; Mycobacterium tuberculosis complex; Mycobacterium tuberculosis; Mycobacterium tuberculosis variant bovis; [illegible]	NCBI: 33892	4279194	99.78	0.00	Yes
NCBI Assembly	GCA_001321905.1	NCBI: cellular organisms; Bacteria; Terrabacteria group; Actinobacteria; Actinomycetia; Corynebacteriales; Mycobacteriaceae; Mycobacterium; Mycobacterium tuberculosis complex; Mycobacterium tuberculosis;	NCBI: 1773	4357003	99.94	0.00	Yes
NCBI Assembly	GCA_001325045.1	NCBI: cellular organisms; Bacteria; Terrabacteria group; Actinobacteria; Actinomycetia; Corynebacteriales; Mycobacteriaceae; Mycobacterium; Mycobacterium tuberculosis complex; Mycobacterium tuberculosis;	NCBI: 1773	4353048	99.94	0.00	Yes
NCBI Assembly	GCA_001377175.1	NCBI: cellular organisms; Bacteria; Terrabacteria group; Actinobacteria; Actinomycetia; Corynebacteriales; Mycobacteriaceae; Mycobacterium; Mycobacterium tuberculosis complex; Mycobacterium tuberculosis;	NCBI: 1773	4361008	99.94	0.00	Yes
NCBI Assembly	GCA_001196335.1	NCBI: cellular organisms; Bacteria; Terrabacteria group; Actinobacteria; Actinomycetia; Corynebacteriales; Mycobacteriaceae; Mycobacterium; Mycobacterium tuberculosis complex; Mycobacterium tuberculosis;	NCBI: 1773	4252556	99.94	0.33	Yes

NCBI Assembly	GCA_001318085.1	NCBI: cellular organisms; Bacteria; Terrabacteria group; Actinobacteria; Actinomycetia; Corynebacteriales; Mycobacteriaceae; Mycobacterium; Mycobacterium tuberculosis complex; Mycobacterium tuberculosis;	NCBI: 1773	4291158	99.88	0.07	Yes
NCBI Assembly	GCA_001379075.1	NCBI: cellular organisms; Bacteria; Terrabacteria group; Actinobacteria; Actinomycetia; Corynebacteriales; Mycobacteriaceae; Mycobacterium; Mycobacterium tuberculosis complex; Mycobacterium tuberculosis;	NCBI: 1773	4365256	99.94	0.00	Yes
NCBI Assembly	GCA_001156445.1	NCBI: cellular organisms; Bacteria; Terrabacteria group; Actinobacteria; Actinomycetia; Corynebacteriales; Mycobacteriaceae; Mycobacterium; Mycobacterium tuberculosis complex; Mycobacterium tuberculosis;	NCBI: 1773	4362966	99.94	0.00	Yes
NCBI Assembly	GCA_001112865.1	NCBI: cellular organisms; Bacteria; Terrabacteria group; Actinobacteria; Actinomycetia; Corynebacteriales; Mycobacteriaceae; Mycobacterium; Mycobacterium tuberculosis complex; Mycobacterium tuberculosis;	NCBI: 1773	4226798	97.12	2.24	Yes
NCBI Assembly	GCA_001379655.1	NCBI: cellular organisms; Bacteria; Terrabacteria group; Actinobacteria; Actinomycetia; Corynebacteriales; Mycobacteriaceae; Mycobacterium; Mycobacterium tuberculosis complex; Mycobacterium tuberculosis;	NCBI: 1773	4362733	99.94	0.00	Yes
NCBI Assembly	GCA_000981265.1	NCBI: cellular organisms; Bacteria; Terrabacteria group; Actinobacteria; Actinomycetia; Corynebacteriales; Mycobacteriaceae; Mycobacterium; Mycobacterium tuberculosis complex; Mycobacterium tuberculosis;	NCBI: 1773	4329824	99.94	0.00	Yes
NCBI Assembly	GCA_001287185.1	NCBI: cellular organisms; Bacteria; Terrabacteria group; Actinobacteria; Actinomycetia; Corynebacteriales; Mycobacteriaceae; Mycobacterium; Mycobacterium tuberculosis complex; Mycobacterium tuberculosis; Mycobacterium tuberculosis variant bovis; Mycobacterium tuberculosis variant bovis BCG;	NCBI: 33892	4303180	99.78	0.00	Yes
NCBI Assembly	GCA_001324105.1	NCBI: cellular organisms; Bacteria; Terrabacteria group; Actinobacteria; Actinomycetia; Corynebacteriales; Mycobacteriaceae; Mycobacterium; Mycobacterium tuberculosis complex; Mycobacterium tuberculosis;	NCBI: 1773	4364692	99.94	0.00	Yes
NCBI Assembly	GCA_001321925.1	NCBI: cellular organisms; Bacteria; Terrabacteria group; Actinobacteria; Actinomycetia; Corynebacteriales; Mycobacteriaceae; Mycobacterium; Mycobacterium tuberculosis complex; Mycobacterium tuberculosis;	NCBI: 1773	4365357	99.94	0.00	Yes
NCBI Assembly	GCA_001325065.1	NCBI: cellular organisms; Bacteria; Terrabacteria group; Actinobacteria; Actinomycetia; Corynebacteriales; Mycobacteriaceae; Mycobacterium; Mycobacterium tuberculosis complex; Mycobacterium tuberculosis;	NCBI: 1773	4380595	99.94	0.00	Yes
NCBI Assembly	GCA_001377195.1	NCBI: cellular organisms; Bacteria; Terrabacteria group; Actinobacteria; Actinomycetia; Corynebacteriales; Mycobacteriaceae; Mycobacterium; Mycobacterium tuberculosis complex; Mycobacterium tuberculosis;	NCBI: 1773	4385168	99.94	0.00	Yes
NCBI Assembly	GCA_001318105.1	NCBI: cellular organisms; Bacteria; Terrabacteria group; Actinobacteria; Actinomycetia; Corynebacteriales; Mycobacteriaceae; Mycobacterium; Mycobacterium tuberculosis complex; Mycobacterium tuberculosis;	NCBI: 1773	4293748	99.24	1.33	Yes
NCBI Assembly	GCA_001196635.1	NCBI: cellular organisms; Bacteria; Terrabacteria group; Actinobacteria; Actinomycetia; Corynebacteriales; Mycobacteriaceae; Mycobacterium; Mycobacterium tuberculosis complex; Mycobacterium tuberculosis;	NCBI: 1773	4285099	99.94	0.00	Yes
NCBI Assembly	GCA_001157085.1	NCBI: cellular organisms; Bacteria; Terrabacteria group; Actinobacteria; Actinomycetia; Corynebacteriales; Mycobacteriaceae; Mycobacterium; Mycobacterium tuberculosis complex; Mycobacterium tuberculosis;	NCBI: 1773	4378993	99.94	0.00	Yes
NCBI Assembly	GCA_001379095.1	NCBI: cellular organisms; Bacteria; Terrabacteria group; Actinobacteria; Actinomycetia; Corynebacteriales; Mycobacteriaceae; Mycobacterium; Mycobacterium tuberculosis complex; Mycobacterium tuberculosis;	NCBI: 1773	4368706	99.94	0.00	Yes
NCBI Assembly	GCA_001112945.1	NCBI: cellular organisms; Bacteria; Terrabacteria group; Actinobacteria; Actinomycetia; Corynebacteriales; Mycobacteriaceae; Mycobacterium; Mycobacterium tuberculosis complex; Mycobacterium tuberculosis;	NCBI: 1773	4032560	89.48	2.90	No
NCBI Assembly	GCA_001328375.1	NCBI: cellular organisms; Bacteria; Terrabacteria group; Actinobacteria; Actinomycetia; Corynebacteriales; Mycobacteriaceae; Mycobacterium; Mycobacterium tuberculosis complex; Mycobacterium tuberculosis;	NCBI: 1773	4304443	99.58	0.00	Yes
NCBI Assembly	GCA_000982795.1	NCBI: cellular organisms; Bacteria; Terrabacteria group; Actinobacteria; Actinomycetia; Corynebacteriales; Mycobacteriaceae; Mycobacterium; Mycobacterium tuberculosis complex; Mycobacterium tuberculosis;	NCBI: 1773	4372159	99.94	0.00	Yes

NCBI Assembly	GCA_001287205.1	NCBI: cellular organisms; Bacteria; Terrabacteria group; Actinobacteria; Actinomycetia; Corynebacteriales; Mycobacteriaceae; Mycobacterium; Mycobacterium tuberculosis complex; Mycobacterium tuberculosis; Mycobacterium tuberculosis variant bovis; Mycobacterium tuberculosis variant bovis BCG;	NCBI: 33892	4337122	99.78	0.22	Yes
NCBI Assembly	GCA_001321945.1	NCBI: cellular organisms; Bacteria; Terrabacteria group; Actinobacteria; Actinomycetia; Corynebacteriales; Mycobacteriaceae; Mycobacterium; Mycobacterium tuberculosis complex; Mycobacterium tuberculosis;	NCBI: 1773	4362862	99.94	0.00	Yes
NCBI Assembly	GCA_001324125.1	NCBI: cellular organisms; Bacteria; Terrabacteria group; Actinobacteria; Actinomycetia; Corynebacteriales; Mycobacteriaceae; Mycobacterium; Mycobacterium tuberculosis complex; Mycobacterium tuberculosis;	NCBI: 1773	4360945	99.94	0.00	Yes
NCBI Assembly	GCA_001325085.1	NCBI: cellular organisms; Bacteria; Terrabacteria group; Actinobacteria; Actinomycetia; Corynebacteriales; Mycobacteriaceae; Mycobacterium; Mycobacterium tuberculosis complex; Mycobacterium tuberculosis;	NCBI: 1773	4401671	99.94	0.33	Yes
NCBI Assembly	GCA_001196715.1	NCBI: cellular organisms; Bacteria; Terrabacteria group; Actinobacteria; Actinomycetia; Corynebacteriales; Mycobacteriaceae; Mycobacterium; Mycobacterium tuberculosis complex; Mycobacterium tuberculosis;	NCBI: 1773	4217249	93.63	1.07	Yes
NCBI Assembly	GCA_001318125.1	NCBI: cellular organisms; Bacteria; Terrabacteria group; Actinobacteria; Actinomycetia; Corynebacteriales; Mycobacteriaceae; Mycobacterium; Mycobacterium tuberculosis complex; Mycobacterium tuberculosis;	NCBI: 1773	4094176	94.30	2.49	Yes
NCBI Assembly	GCA_001377215.1	NCBI: cellular organisms; Bacteria; Terrabacteria group; Actinobacteria; Actinomycetia; Corynebacteriales; Mycobacteriaceae; Mycobacterium; Mycobacterium tuberculosis complex; Mycobacterium tuberculosis;	NCBI: 1773	4364204	99.94	0.00	Yes
NCBI Assembly	GCA_001158445.1	NCBI: cellular organisms; Bacteria; Terrabacteria group; Actinobacteria; Actinomycetia; Corynebacteriales; Mycobacteriaceae; Mycobacterium; Mycobacterium tuberculosis complex; Mycobacterium tuberculosis;	NCBI: 1773	4296804	87.87	0.33	No
NCBI Assembly	GCA_000987455.1	NCBI: cellular organisms; Bacteria; Terrabacteria group; Actinobacteria; Actinomycetia; Corynebacteriales; Mycobacteriaceae; Mycobacterium; unclassified Mycobacterium;	NCBI: 1385419	6809170	99.14	1.69	Yes
NCBI Assembly	GCA_001114085.1	NCBI: cellular organisms; Bacteria; Terrabacteria group; Actinobacteria; Actinomycetia; Corynebacteriales; Mycobacteriaceae; Mycobacterium; Mycobacterium tuberculosis complex; Mycobacterium tuberculosis;	NCBI: 1773	4355979	99.94	0.00	Yes
NCBI Assembly	GCA_001328395.1	NCBI: cellular organisms; Bacteria; Terrabacteria group; Actinobacteria; Actinomycetia; Corynebacteriales; Mycobacteriaceae; Mycobacterium; Mycobacterium tuberculosis complex; Mycobacterium tuberculosis;	NCBI: 1773	4297336	99.61	0.00	Yes
NCBI Assembly	GCA_001379115.1	NCBI: cellular organisms; Bacteria; Terrabacteria group; Actinobacteria; Actinomycetia; Corynebacteriales; Mycobacteriaceae; Mycobacterium; Mycobacterium tuberculosis complex; Mycobacterium tuberculosis;	NCBI: 1773	4367497	99.94	0.00	Yes
NCBI Assembly	GCA_001287225.1	NCBI: cellular organisms; Bacteria; Terrabacteria group; Actinobacteria; Actinomycetia; Corynebacteriales; Mycobacteriaceae; Mycobacterium; Mycobacterium tuberculosis complex; Mycobacterium tuberculosis; Mycobacterium tuberculosis variant bovis; Mycobacterium tuberculosis variant bovis BCG;	NCBI: 33892	4297448	99.78	0.00	Yes
NCBI Assembly	GCA_001321965.1	NCBI: cellular organisms; Bacteria; Terrabacteria group; Actinobacteria; Actinomycetia; Corynebacteriales; Mycobacteriaceae; Mycobacterium; Mycobacterium tuberculosis complex; Mycobacterium tuberculosis;	NCBI: 1773	4338322	99.94	0.33	Yes
NCBI Assembly	GCA_001324145.1	NCBI: cellular organisms; Bacteria; Terrabacteria group; Actinobacteria; Actinomycetia; Corynebacteriales; Mycobacteriaceae; Mycobacterium; Mycobacterium tuberculosis complex; Mycobacterium tuberculosis;	NCBI: 1773	4357686	99.94	0.00	Yes
NCBI Assembly	GCA_001318145.1	NCBI: cellular organisms; Bacteria; Terrabacteria group; Actinobacteria; Actinomycetia; Corynebacteriales; Mycobacteriaceae; Mycobacterium; Mycobacterium tuberculosis complex; Mycobacterium tuberculosis;	NCBI: 1773	4281216	99.54	0.00	Yes
NCBI Assembly	GCA_001325105.1	NCBI: cellular organisms; Bacteria; Terrabacteria group; Actinobacteria; Actinomycetia; Corynebacteriales; Mycobacteriaceae; Mycobacterium; Mycobacterium tuberculosis complex; Mycobacterium tuberculosis;	NCBI: 1773	4354958	99.94	0.00	Yes
NCBI Assembly	GCA_001377235.1	NCBI: cellular organisms; Bacteria; Terrabacteria group; Actinobacteria; Actinomycetia; Corynebacteriales; Mycobacteriaceae; Mycobacterium; Mycobacterium tuberculosis complex; Mycobacterium tuberculosis;	NCBI: 1773	4355463	99.94	0.00	Yes

NCBI Assembly	GCA_001196835.1	NCBI: cellular organisms; Bacteria; Terrabacteria group; Actinobacteria; Actinomycetia; Corynebacteriales; Mycobacteriaceae; Mycobacterium; Mycobacterium tuberculosis complex; Mycobacterium tuberculosis;	NCBI: 1773	4317096	99.94	0.00	Yes
NCBI Assembly	GCA_001005175.1	NCBI: cellular organisms; Bacteria; Terrabacteria group; Actinobacteria; Actinomycetia; Corynebacteriales; Mycobacteriaceae; Mycolicibacterium; Mycolicibacterium elephantis;	NCBI: 81858	5187616	100.00	1.36	Yes
NCBI Assembly	GCA_001159945.1	NCBI: cellular organisms; Bacteria; Terrabacteria group; Actinobacteria; Actinomycetia; Corynebacteriales; Mycobacteriaceae; Mycobacterium; Mycobacterium tuberculosis complex; Mycobacterium tuberculosis;	NCBI: 1773	4392936	99.94	0.00	Yes
NCBI Assembly	GCA_001115905.1	NCBI: cellular organisms; Bacteria; Terrabacteria group; Actinobacteria; Actinomycetia; Corynebacteriales; Mycobacteriaceae; Mycobacterium; Mycobacterium tuberculosis complex; Mycobacterium tuberculosis;	NCBI: 1773	4327834	99.94	0.00	Yes
NCBI Assembly	GCA_001328415.1	NCBI: cellular organisms; Bacteria; Terrabacteria group; Actinobacteria; Actinomycetia; Corynebacteriales; Mycobacteriaceae; Mycobacterium; Mycobacterium tuberculosis complex; Mycobacterium tuberculosis;	NCBI: 1773	4291110	99.83	0.00	Yes
NCBI Assembly	GCA_001379135.1	NCBI: cellular organisms; Bacteria; Terrabacteria group; Actinobacteria; Actinomycetia; Corynebacteriales; Mycobacteriaceae; Mycobacterium; Mycobacterium tuberculosis complex; Mycobacterium tuberculosis;	NCBI: 1773	4373809	99.94	0.00	Yes
NCBI Assembly	GCA_001287245.1	NCBI: cellular organisms; Bacteria; Terrabacteria group; Actinobacteria; Actinomycetia; Corynebacteriales; Mycobacteriaceae; Mycobacterium; Mycobacterium tuberculosis complex; Mycobacterium tuberculosis; Mycobacterium tuberculosis variant bovis; Mycobacterium tuberculosis variant bovis BCG;	NCBI: 33892	4318337	99.78	0.11	Yes
NCBI Assembly	GCA_001321985.1	NCBI: cellular organisms; Bacteria; Terrabacteria group; Actinobacteria; Actinomycetia; Corynebacteriales; Mycobacteriaceae; Mycobacterium; Mycobacterium tuberculosis complex; Mycobacterium tuberculosis;	NCBI: 1773	4368843	99.94	0.00	Yes
NCBI Assembly	GCA_001324165.1	NCBI: cellular organisms; Bacteria; Terrabacteria group; Actinobacteria; Actinomycetia; Corynebacteriales; Mycobacteriaceae; Mycobacterium; Mycobacterium tuberculosis complex; Mycobacterium tuberculosis;	NCBI: 1773	4366962	99.94	0.00	Yes
NCBI Assembly	GCA_001325125.1	NCBI: cellular organisms; Bacteria; Terrabacteria group; Actinobacteria; Actinomycetia; Corynebacteriales; Mycobacteriaceae; Mycobacterium; Mycobacterium tuberculosis complex; Mycobacterium tuberculosis;	NCBI: 1773	4352562	99.94	0.11	Yes
NCBI Assembly	GCA_001377255.1	NCBI: cellular organisms; Bacteria; Terrabacteria group; Actinobacteria; Actinomycetia; Corynebacteriales; Mycobacteriaceae; Mycobacterium; Mycobacterium tuberculosis complex; Mycobacterium tuberculosis;	NCBI: 1773	4367200	99.94	0.00	Yes
NCBI Assembly	GCA_001160225.1	NCBI: cellular organisms; Bacteria; Terrabacteria group; Actinobacteria; Actinomycetia; Corynebacteriales; Mycobacteriaceae; Mycobacterium; Mycobacterium tuberculosis complex; Mycobacterium tuberculosis;	NCBI: 1773	12705387	100.00	70.02	No
NCBI Assembly	GCA_001012795.1	NCBI: cellular organisms; Bacteria; Terrabacteria group; Actinobacteria; Actinomycetia; Corynebacteriales; Mycobacteriaceae; Mycolicibacterium; Mycolicibacterium senegalense;	NCBI: 1796	6738555	99.87	0.13	Yes
NCBI Assembly	GCA_001196935.1	NCBI: cellular organisms; Bacteria; Terrabacteria group; Actinobacteria; Actinomycetia; Corynebacteriales; Mycobacteriaceae; Mycobacterium; Mycobacterium tuberculosis complex; Mycobacterium tuberculosis;	NCBI: 1773	4066414	84.87	2.42	No
NCBI Assembly	GCA_001318165.1	NCBI: cellular organisms; Bacteria; Terrabacteria group; Actinobacteria; Actinomycetia; Corynebacteriales; Mycobacteriaceae; Mycobacterium; Mycobacterium tuberculosis complex; Mycobacterium tuberculosis;	NCBI: 1773	1922119	35.94	3.73	No
NCBI Assembly	GCA_001116025.1	NCBI: cellular organisms; Bacteria; Terrabacteria group; Actinobacteria; Actinomycetia; Corynebacteriales; Mycobacteriaceae; Mycobacterium; Mycobacterium tuberculosis complex; Mycobacterium tuberculosis;	NCBI: 1773	4380508	99.94	0.00	Yes
NCBI Assembly	GCA_001328435.1	NCBI: cellular organisms; Bacteria; Terrabacteria group; Actinobacteria; Actinomycetia; Corynebacteriales; Mycobacteriaceae; Mycobacterium; Mycobacterium tuberculosis complex; Mycobacterium tuberculosis;	NCBI: 1773	4313370	99.86	0.00	Yes
NCBI Assembly	GCA_001379155.1	NCBI: cellular organisms; Bacteria; Terrabacteria group; Actinobacteria; Actinomycetia; Corynebacteriales; Mycobacteriaceae; Mycobacterium; Mycobacterium tuberculosis complex; Mycobacterium tuberculosis;	NCBI: 1773	4395117	99.94	0.00	Yes

NCBI Assembly	GCA_001287325.1	NCBI: cellular organisms; Bacteria; Terrabacteria group; Actinobacteria; Actinomycetia; Corynebacteriales; Mycobacteriaceae; Mycobacterium; Mycobacterium tuberculosis complex; Mycobacterium tuberculosis; Mycobacterium tuberculosis variant bovis; Mycobacterium tuberculosis variant bovis BCG;	NCBI: 33892	4293535	99.78	0.15	Yes
NCBI Assembly	GCA_001322005.1	NCBI: cellular organisms; Bacteria; Terrabacteria group; Actinobacteria; Actinomycetia; Corynebacteriales; Mycobacteriaceae; Mycobacterium; Mycobacterium tuberculosis complex; Mycobacterium tuberculosis;	NCBI: 1773	4362920	99.94	0.00	Yes
NCBI Assembly	GCA_001324185.1	NCBI: cellular organisms; Bacteria; Terrabacteria group; Actinobacteria; Actinomycetia; Corynebacteriales; Mycobacteriaceae; Mycobacterium; Mycobacterium tuberculosis complex; Mycobacterium tuberculosis;	NCBI: 1773	4957617	99.94	16.51	No
NCBI Assembly	GCA_001325145.1	NCBI: cellular organisms; Bacteria; Terrabacteria group; Actinobacteria; Actinomycetia; Corynebacteriales; Mycobacteriaceae; Mycobacterium; Mycobacterium tuberculosis complex; Mycobacterium tuberculosis;	NCBI: 1773	4386383	99.94	0.33	Yes
NCBI Assembly	GCA_001116745.1	NCBI: cellular organisms; Bacteria; Terrabacteria group; Actinobacteria; Actinomycetia; Corynebacteriales; Mycobacteriaceae; Mycobacterium; Mycobacterium tuberculosis complex; Mycobacterium tuberculosis;	NCBI: 1773	4267151	99.58	0.90	Yes
NCBI Assembly	GCA_001017695.1	NCBI: cellular organisms; Bacteria; Terrabacteria group; Actinobacteria; Actinomycetia; Corynebacteriales; Mycobacteriaceae; Mycobacterium; Mycobacterium tuberculosis complex; Mycobacterium tuberculosis;	NCBI: 1773	4327685	99.90	0.03	Yes
NCBI Assembly	GCA_001377275.1	NCBI: cellular organisms; Bacteria; Terrabacteria group; Actinobacteria; Actinomycetia; Corynebacteriales; Mycobacteriaceae; Mycobacterium; Mycobacterium tuberculosis complex; Mycobacterium tuberculosis;	NCBI: 1773	4362916	99.94	0.00	Yes
NCBI Assembly	GCA_001160825.1	NCBI: cellular organisms; Bacteria; Terrabacteria group; Actinobacteria; Actinomycetia; Corynebacteriales; Mycobacteriaceae; Mycobacterium; Mycobacterium tuberculosis complex; Mycobacterium tuberculosis;	NCBI: 1773	4311913	99.94	0.33	Yes
NCBI Assembly	GCA_001197035.1	NCBI: cellular organisms; Bacteria; Terrabacteria group; Actinobacteria; Actinomycetia; Corynebacteriales; Mycobacteriaceae; Mycobacterium; Mycobacterium tuberculosis complex; Mycobacterium tuberculosis;	NCBI: 1773	4290639	99.28	1.67	Yes
NCBI Assembly	GCA_001318285.1	NCBI: cellular organisms; Bacteria; Terrabacteria group; Actinobacteria; Actinomycetia; Corynebacteriales; Mycobacteriaceae; Mycobacterium; Mycobacterium tuberculosis complex; Mycobacterium tuberculosis;	NCBI: 1773	4211392	95.02	0.55	Yes
NCBI Assembly	GCA_001328455.1	NCBI: cellular organisms; Bacteria; Terrabacteria group; Actinobacteria; Actinomycetia; Corynebacteriales; Mycobacteriaceae; Mycobacterium; Mycobacterium tuberculosis complex; Mycobacterium tuberculosis;	NCBI: 1773	4324169	99.53	0.67	Yes
NCBI Assembly	GCA_001379175.1	NCBI: cellular organisms; Bacteria; Terrabacteria group; Actinobacteria; Actinomycetia; Corynebacteriales; Mycobacteriaceae; Mycobacterium; Mycobacterium tuberculosis complex; Mycobacterium tuberculosis;	NCBI: 1773	4366133	99.94	0.00	Yes
NCBI Assembly	GCA_001287365.1	NCBI: cellular organisms; Bacteria; Terrabacteria group; Actinobacteria; Actinomycetia; Corynebacteriales; Mycobacteriaceae; Mycobacterium; Mycobacterium tuberculosis complex; Mycobacterium tuberculosis; Mycobacterium tuberculosis variant bovis; Mycobacterium tuberculosis variant bovis BCG;	NCBI: 33892	4317624	99.78	0.11	Yes
NCBI Assembly	GCA_001324205.1	NCBI: cellular organisms; Bacteria; Terrabacteria group; Actinobacteria; Actinomycetia; Corynebacteriales; Mycobacteriaceae; Mycobacterium; Mycobacterium tuberculosis complex; Mycobacterium tuberculosis;	NCBI: 1773	4354919	99.94	0.00	Yes
NCBI Assembly	GCA_001322025.1	NCBI: cellular organisms; Bacteria; Terrabacteria group; Actinobacteria; Actinomycetia; Corynebacteriales; Mycobacteriaceae; Mycobacterium; Mycobacterium tuberculosis complex; Mycobacterium tuberculosis;	NCBI: 1773	4365949	99.94	0.00	Yes
NCBI Assembly	GCA_001021385.1	NCBI: cellular organisms; Bacteria; Terrabacteria group; Actinobacteria; Actinomycetia; Corynebacteriales; Mycobacteriaceae; Mycobacterium; unclassified Mycobacterium;	NCBI: 1545728	6177406	99.81	0.06	Yes
NCBI Assembly	GCA_001117725.1	NCBI: cellular organisms; Bacteria; Terrabacteria group; Actinobacteria; Actinomycetia; Corynebacteriales; Mycobacteriaceae; Mycobacterium; Mycobacterium tuberculosis complex; Mycobacterium tuberculosis;	NCBI: 1773	4362672	99.94	0.00	Yes
NCBI Assembly	GCA_001325165.1	NCBI: cellular organisms; Bacteria; Terrabacteria group; Actinobacteria; Actinomycetia; Corynebacteriales; Mycobacteriaceae; Mycobacterium; Mycobacterium tuberculosis complex; Mycobacterium tuberculosis;	NCBI: 1773	4350894	99.91	0.00	Yes

NCBI Assembly	GCA_001162825.1	NCBI: cellular organisms; Bacteria; Terrabacteria group; Actinobacteria; Actinomycetia; Corynebacteriales; Mycobacteriaceae; Mycobacterium; Mycobacterium tuberculosis complex; Mycobacterium tuberculosis;	NCBI: 1773	4254409	99.61	0.00	Yes
NCBI Assembly	GCA_001197095.1	NCBI: cellular organisms; Bacteria; Terrabacteria group; Actinobacteria; Actinomycetia; Corynebacteriales; Mycobacteriaceae; Mycobacterium; Mycobacterium tuberculosis complex; Mycobacterium tuberculosis;	NCBI: 1773	4272712	99.82	1.00	Yes
NCBI Assembly	GCA_001328475.1	NCBI: cellular organisms; Bacteria; Terrabacteria group; Actinobacteria; Actinomycetia; Corynebacteriales; Mycobacteriaceae; Mycobacterium; Mycobacterium tuberculosis complex; Mycobacterium tuberculosis;	NCBI: 1773	4322024	99.94	0.00	Yes
NCBI Assembly	GCA_001377295.1	NCBI: cellular organisms; Bacteria; Terrabacteria group; Actinobacteria; Actinomycetia; Corynebacteriales; Mycobacteriaceae; Mycobacterium; Mycobacterium tuberculosis complex; Mycobacterium tuberculosis;	NCBI: 1773	4352597	99.94	0.00	Yes
NCBI Assembly	GCA_001379195.1	NCBI: cellular organisms; Bacteria; Terrabacteria group; Actinobacteria; Actinomycetia; Corynebacteriales; Mycobacteriaceae; Mycobacterium; Mycobacterium tuberculosis complex; Mycobacterium tuberculosis;	NCBI: 1773	4381786	99.94	0.17	Yes
NCBI Assembly	GCA_001318425.1	NCBI: cellular organisms; Bacteria; Terrabacteria group; Actinobacteria; Actinomycetia; Corynebacteriales; Mycobacteriaceae; Mycobacterium; Mycobacterium tuberculosis complex; Mycobacterium tuberculosis;	NCBI: 1773	6194963	93.81	36.20	No
NCBI Assembly	GCA_001287425.1	NCBI: cellular organisms; Bacteria; Terrabacteria group; Actinobacteria; Actinomycetia; Corynebacteriales; Mycobacteriaceae; Mycobacterium; Mycobacterium tuberculosis complex; Mycobacterium tuberculosis; Mycobacterium tuberculosis variant bovis; Mycobacterium tuberculosis variant bovis BCG;	NCBI: 33892	4322672	99.78	0.00	Yes
NCBI Assembly	GCA_001324225.1	NCBI: cellular organisms; Bacteria; Terrabacteria group; Actinobacteria; Actinomycetia; Corynebacteriales; Mycobacteriaceae; Mycobacterium; Mycobacterium tuberculosis complex; Mycobacterium tuberculosis;	NCBI: 1773	4367230	99.94	0.17	Yes
NCBI Assembly	GCA_001021405.1	NCBI: cellular organisms; Bacteria; Terrabacteria group; Actinobacteria; Actinomycetia; Corynebacteriales; Mycobacteriaceae; Mycobacterium; Mycobacterium haemophilum;	NCBI: 29311	4363991	98.88	1.12	Yes
NCBI Assembly	GCA_001322045.1	NCBI: cellular organisms; Bacteria; Terrabacteria group; Actinobacteria; Actinomycetia; Corynebacteriales; Mycobacteriaceae; Mycobacterium; Mycobacterium tuberculosis complex; Mycobacterium tuberculosis;	NCBI: 1773	4348945	99.94	0.00	Yes
NCBI Assembly	GCA_001118545.1	NCBI: cellular organisms; Bacteria; Terrabacteria group; Actinobacteria; Actinomycetia; Corynebacteriales; Mycobacteriaceae; Mycobacterium; Mycobacterium tuberculosis complex; Mycobacterium tuberculosis;	NCBI: 1773	4304978	99.29	1.61	Yes
NCBI Assembly	GCA_001325185.1	NCBI: cellular organisms; Bacteria; Terrabacteria group; Actinobacteria; Actinomycetia; Corynebacteriales; Mycobacteriaceae; Mycobacterium; Mycobacterium tuberculosis complex; Mycobacterium tuberculosis;	NCBI: 1773	4350862	99.61	0.00	Yes
NCBI Assembly	GCA_001197435.1	NCBI: cellular organisms; Bacteria; Terrabacteria group; Actinobacteria; Actinomycetia; Corynebacteriales; Mycobacteriaceae; Mycobacterium; Mycobacterium tuberculosis complex; Mycobacterium tuberculosis;	NCBI: 1773	4282205	99.91	0.33	Yes
NCBI Assembly	GCA_001163125.1	NCBI: cellular organisms; Bacteria; Terrabacteria group; Actinobacteria; Actinomycetia; Corynebacteriales; Mycobacteriaceae; Mycobacterium; Mycobacterium tuberculosis complex; Mycobacterium tuberculosis;	NCBI: 1773	4293563	99.36	0.73	Yes
NCBI Assembly	GCA_001377315.1	NCBI: cellular organisms; Bacteria; Terrabacteria group; Actinobacteria; Actinomycetia; Corynebacteriales; Mycobacteriaceae; Mycobacterium; Mycobacterium tuberculosis complex; Mycobacterium tuberculosis;	NCBI: 1773	4368418	99.94	0.00	Yes
NCBI Assembly	GCA_001328495.1	NCBI: cellular organisms; Bacteria; Terrabacteria group; Actinobacteria; Actinomycetia; Corynebacteriales; Mycobacteriaceae; Mycobacterium; Mycobacterium tuberculosis complex; Mycobacterium tuberculosis;	NCBI: 1773	4290934	99.61	0.00	Yes
NCBI Assembly	GCA_001379215.1	NCBI: cellular organisms; Bacteria; Terrabacteria group; Actinobacteria; Actinomycetia; Corynebacteriales; Mycobacteriaceae; Mycobacterium; Mycobacterium tuberculosis complex; Mycobacterium tuberculosis;	NCBI: 1773	4392348	99.94	0.00	Yes
NCBI Assembly	GCA_001318445.1	NCBI: cellular organisms; Bacteria; Terrabacteria group; Actinobacteria; Actinomycetia; Corynebacteriales; Mycobacteriaceae; Mycobacterium; Mycobacterium tuberculosis complex; Mycobacterium tuberculosis;	NCBI: 1773	4199395	86.54	2.06	No

NCBI Assembly	GCA_001324245.1	NCBI: cellular organisms; Bacteria; Terrabacteria group; Actinobacteria; Actinomycetia; Corynebacteriales; Mycobacteriaceae; Mycobacterium; Mycobacterium tuberculosis complex; Mycobacterium tuberculosis;	NCBI: 1773	4369783	99.94	0.00	Yes
NCBI Assembly	GCA_001287485.1	NCBI: cellular organisms; Bacteria; Terrabacteria group; Actinobacteria; Actinomycetia; Corynebacteriales; Mycobacteriaceae; Mycobacterium; Mycobacterium tuberculosis complex; Mycobacterium tuberculosis; Mycobacterium tuberculosis variant bovis; Mycobacterium tuberculosis variant bovis BCG;	NCBI: 33892	4285339	99.78	0.00	Yes
NCBI Assembly	GCA_001021415.1	NCBI: cellular organisms; Bacteria; Terrabacteria group; Actinobacteria; Actinomycetia; Corynebacteriales; Mycobacteriaceae; Mycobacterium; Mycobacterium haemophilum;	NCBI: 29311	4369428	98.88	1.12	Yes
NCBI Assembly	GCA_001119505.1	NCBI: cellular organisms; Bacteria; Terrabacteria group; Actinobacteria; Actinomycetia; Corynebacteriales; Mycobacteriaceae; Mycobacterium; Mycobacterium tuberculosis complex; Mycobacterium tuberculosis;	NCBI: 1773	4364347	99.94	0.00	Yes
NCBI Assembly	GCA_001197475.1	NCBI: cellular organisms; Bacteria; Terrabacteria group; Actinobacteria; Actinomycetia; Corynebacteriales; Mycobacteriaceae; Mycobacterium; Mycobacterium tuberculosis complex; Mycobacterium tuberculosis;	NCBI: 1773	1358013	21.39	2.01	No
NCBI Assembly	GCA_001325205.1	NCBI: cellular organisms; Bacteria; Terrabacteria group; Actinobacteria; Actinomycetia; Corynebacteriales; Mycobacteriaceae; Mycobacterium; Mycobacterium tuberculosis complex; Mycobacterium tuberculosis;	NCBI: 1773	4357908	99.86	0.00	Yes
NCBI Assembly	GCA_001322065.1	NCBI: cellular organisms; Bacteria; Terrabacteria group; Actinobacteria; Actinomycetia; Corynebacteriales; Mycobacteriaceae; Mycobacterium; Mycobacterium tuberculosis complex; Mycobacterium tuberculosis;	NCBI: 1773	4291918	99.10	2.34	Yes
NCBI Assembly	GCA_001163485.1	NCBI: cellular organisms; Bacteria; Terrabacteria group; Actinobacteria; Actinomycetia; Corynebacteriales; Mycobacteriaceae; Mycobacterium; Mycobacterium tuberculosis complex; Mycobacterium tuberculosis;	NCBI: 1773	4359110	99.94	0.00	Yes
NCBI Assembly	GCA_001328515.1	NCBI: cellular organisms; Bacteria; Terrabacteria group; Actinobacteria; Actinomycetia; Corynebacteriales; Mycobacteriaceae; Mycobacterium; Mycobacterium tuberculosis complex; Mycobacterium tuberculosis;	NCBI: 1773	4314752	99.75	0.00	Yes
NCBI Assembly	GCA_001377335.1	NCBI: cellular organisms; Bacteria; Terrabacteria group; Actinobacteria; Actinomycetia; Corynebacteriales; Mycobacteriaceae; Mycobacterium; Mycobacterium tuberculosis complex; Mycobacterium tuberculosis;	NCBI: 1773	4364064	99.94	0.00	Yes
NCBI Assembly	GCA_001379235.1	NCBI: cellular organisms; Bacteria; Terrabacteria group; Actinobacteria; Actinomycetia; Corynebacteriales; Mycobacteriaceae; Mycobacterium; Mycobacterium tuberculosis complex; Mycobacterium tuberculosis;	NCBI: 1773	4366532	99.94	0.17	Yes
NCBI Assembly	GCA_001318465.1	NCBI: cellular organisms; Bacteria; Terrabacteria group; Actinobacteria; Actinomycetia; Corynebacteriales; Mycobacteriaceae; Mycobacterium; Mycobacterium tuberculosis complex; Mycobacterium tuberculosis;	NCBI: 1773	4279715	98.99	1.91	Yes
NCBI Assembly	GCA_001324265.1	NCBI: cellular organisms; Bacteria; Terrabacteria group; Actinobacteria; Actinomycetia; Corynebacteriales; Mycobacteriaceae; Mycobacterium; Mycobacterium tuberculosis complex; Mycobacterium tuberculosis;	NCBI: 1773	4376654	99.94	0.00	Yes
NCBI Assembly	GCA_001119665.1	NCBI: cellular organisms; Bacteria; Terrabacteria group; Actinobacteria; Actinomycetia; Corynebacteriales; Mycobacteriaceae; Mycobacterium; Mycobacterium tuberculosis complex; Mycobacterium tuberculosis;	NCBI: 1773	4286000	99.53	1.03	Yes
NCBI Assembly	GCA_001291445.1	NCBI: cellular organisms; Bacteria; Terrabacteria group; Actinobacteria; Actinomycetia; Corynebacteriales; Mycobacteriaceae; Mycolicibacterium; Mycolicibacterium mucogenicum;	NCBI: 56689	6210127	99.81	0.55	Yes
NCBI Assembly	GCA_001197555.1	NCBI: cellular organisms; Bacteria; Terrabacteria group; Actinobacteria; Actinomycetia; Corynebacteriales; Mycobacteriaceae; Mycobacterium; Mycobacterium tuberculosis complex; Mycobacterium tuberculosis;	NCBI: 1773	4324584	99.94	0.00	Yes
NCBI Assembly	GCA_001325225.1	NCBI: cellular organisms; Bacteria; Terrabacteria group; Actinobacteria; Actinomycetia; Corynebacteriales; Mycobacteriaceae; Mycobacterium; Mycobacterium tuberculosis complex; Mycobacterium tuberculosis;	NCBI: 1773	4348146	99.94	0.00	Yes
NCBI Assembly	GCA_001322085.1	NCBI: cellular organisms; Bacteria; Terrabacteria group; Actinobacteria; Actinomycetia; Corynebacteriales; Mycobacteriaceae; Mycobacterium; Mycobacterium tuberculosis complex; Mycobacterium tuberculosis;	NCBI: 1773	4380564	99.94	0.00	Yes

NCBI Assembly	GCA_001164205.1	NCBI: cellular organisms; Bacteria; Terrabacteria group; Actinobacteria; Actinomycetia; Corynebacteriales; Mycobacteriaceae; Mycobacterium; Mycobacterium tuberculosis complex; Mycobacterium tuberculosis;	NCBI: 1773	4299969	99.61	0.33	Yes
NCBI Assembly	GCA_001328535.1	NCBI: cellular organisms; Bacteria; Terrabacteria group; Actinobacteria; Actinomycetia; Corynebacteriales; Mycobacteriaceae; Mycobacterium; Mycobacterium tuberculosis complex; Mycobacterium tuberculosis;	NCBI: 1773	4283976	99.61	0.07	Yes
NCBI Assembly	GCA_001377355.1	NCBI: cellular organisms; Bacteria; Terrabacteria group; Actinobacteria; Actinomycetia; Corynebacteriales; Mycobacteriaceae; Mycobacterium; Mycobacterium tuberculosis complex; Mycobacterium tuberculosis;	NCBI: 1773	4357141	99.94	0.00	Yes
NCBI Assembly	GCA_001379255.1	NCBI: cellular organisms; Bacteria; Terrabacteria group; Actinobacteria; Actinomycetia; Corynebacteriales; Mycobacteriaceae; Mycobacterium; Mycobacterium tuberculosis complex; Mycobacterium tuberculosis;	NCBI: 1773	4374100	99.94	0.00	Yes
NCBI Assembly	GCA_001318485.1	NCBI: cellular organisms; Bacteria; Terrabacteria group; Actinobacteria; Actinomycetia; Corynebacteriales; Mycobacteriaceae; Mycobacterium; Mycobacterium tuberculosis complex; Mycobacterium tuberculosis;	NCBI: 1773	4271351	99.04	2.04	Yes
NCBI Assembly	GCA_001021435.1	NCBI: cellular organisms; Bacteria; Terrabacteria group; Actinobacteria; Actinomycetia; Corynebacteriales; Mycobacteriaceae; Mycobacterium; Mycobacterium haemophilum;	NCBI: 29311	4369071	98.88	1.12	Yes
NCBI Assembly	GCA_001324285.1	NCBI: cellular organisms; Bacteria; Terrabacteria group; Actinobacteria; Actinomycetia; Corynebacteriales; Mycobacteriaceae; Mycobacterium; Mycobacterium tuberculosis complex; Mycobacterium tuberculosis;	NCBI: 1773	4368241	99.94	0.00	Yes
NCBI Assembly	GCA_001119945.1	NCBI: cellular organisms; Bacteria; Terrabacteria group; Actinobacteria; Actinomycetia; Corynebacteriales; Mycobacteriaceae; Mycobacterium; Mycobacterium tuberculosis complex; Mycobacterium tuberculosis;	NCBI: 1773	4286293	99.88	0.00	Yes
NCBI Assembly	GCA_001197655.1	NCBI: cellular organisms; Bacteria; Terrabacteria group; Actinobacteria; Actinomycetia; Corynebacteriales; Mycobacteriaceae; Mycobacterium; Mycobacterium tuberculosis complex; Mycobacterium tuberculosis;	NCBI: 1773	4300178	99.28	0.00	Yes
NCBI Assembly	GCA_001325245.1	NCBI: cellular organisms; Bacteria; Terrabacteria group; Actinobacteria; Actinomycetia; Corynebacteriales; Mycobacteriaceae; Mycobacterium; Mycobacterium tuberculosis complex; Mycobacterium tuberculosis;	NCBI: 1773	4359782	99.61	0.00	Yes
NCBI Assembly	GCA_001293105.1	NCBI: cellular organisms; Bacteria; Terrabacteria group; Actinobacteria; Actinomycetia; Corynebacteriales; Mycobacteriaceae; Mycobacterium; Mycobacterium tuberculosis complex; Mycobacterium tuberculosis; Mycobacterium tuberculosis variant bovis;	NCBI: 1765	4363945	99.61	1.20	Yes
NCBI Assembly	GCA_001164225.1	NCBI: cellular organisms; Bacteria; Terrabacteria group; Actinobacteria; Actinomycetia; Corynebacteriales; Mycobacteriaceae; Mycobacterium; Mycobacterium tuberculosis complex; Mycobacterium tuberculosis;	NCBI: 1773	4394899	99.94	0.00	Yes
NCBI Assembly	GCA_001322105.1	NCBI: cellular organisms; Bacteria; Terrabacteria group; Actinobacteria; Actinomycetia; Corynebacteriales; Mycobacteriaceae; Mycobacterium; Mycobacterium tuberculosis complex; Mycobacterium tuberculosis;	NCBI: 1773	4362325	99.94	0.00	Yes
NCBI Assembly	GCA_001328555.1	NCBI: cellular organisms; Bacteria; Terrabacteria group; Actinobacteria; Actinomycetia; Corynebacteriales; Mycobacteriaceae; Mycobacterium; Mycobacterium tuberculosis complex; Mycobacterium tuberculosis;	NCBI: 1773	4272006	99.73	0.33	Yes
NCBI Assembly	GCA_001377375.1	NCBI: cellular organisms; Bacteria; Terrabacteria group; Actinobacteria; Actinomycetia; Corynebacteriales; Mycobacteriaceae; Mycobacterium; Mycobacterium tuberculosis complex; Mycobacterium tuberculosis;	NCBI: 1773	4426339	99.94	0.56	Yes
NCBI Assembly	GCA_001379275.1	NCBI: cellular organisms; Bacteria; Terrabacteria group; Actinobacteria; Actinomycetia; Corynebacteriales; Mycobacteriaceae; Mycobacterium; Mycobacterium tuberculosis complex; Mycobacterium tuberculosis;	NCBI: 1773	4389601	99.94	0.00	Yes
NCBI Assembly	GCA_001318505.1	NCBI: cellular organisms; Bacteria; Terrabacteria group; Actinobacteria; Actinomycetia; Corynebacteriales; Mycobacteriaceae; Mycobacterium; Mycobacterium tuberculosis complex; Mycobacterium tuberculosis;	NCBI: 1773	4272122	98.83	1.41	Yes
NCBI Assembly	GCA_001324305.1	NCBI: cellular organisms; Bacteria; Terrabacteria group; Actinobacteria; Actinomycetia; Corynebacteriales; Mycobacteriaceae; Mycobacterium; Mycobacterium tuberculosis complex; Mycobacterium tuberculosis;	NCBI: 1773	4391669	99.94	0.00	Yes

NCBI Assembly	GCA_001021485.1	NCBI: cellular organisms; Bacteria; Terrabacteria group; Actinobacteria; Actinomycetia; Corynebacteriales; Mycobacteriaceae; Mycobacterium; Mycobacterium haemophilum;	NCBI: 29311	4366495	98.88	1.12	Yes
NCBI Assembly	GCA_001120965.1	NCBI: cellular organisms; Bacteria; Terrabacteria group; Actinobacteria; Actinomycetia; Corynebacteriales; Mycobacteriaceae; Mycobacterium; Mycobacterium tuberculosis complex; Mycobacterium tuberculosis;	NCBI: 1773	4324599	99.94	0.00	Yes
NCBI Assembly	GCA_001197895.1	NCBI: cellular organisms; Bacteria; Terrabacteria group; Actinobacteria; Actinomycetia; Corynebacteriales; Mycobacteriaceae; Mycobacterium; Mycobacterium tuberculosis complex; Mycobacterium tuberculosis;	NCBI: 1773	4288029	99.25	0.07	Yes
NCBI Assembly	GCA_001165005.1	NCBI: cellular organisms; Bacteria; Terrabacteria group; Actinobacteria; Actinomycetia; Corynebacteriales; Mycobacteriaceae; Mycobacterium; Mycobacterium tuberculosis complex; Mycobacterium tuberculosis;	NCBI: 1773	4254079	90.56	0.33	Yes
NCBI Assembly	GCA_001296245.1	NCBI: cellular organisms; Bacteria; Terrabacteria group; Actinobacteria; Actinomycetia; Corynebacteriales; Mycobacteriaceae; Mycobacteroides; Mycobacteroides immunogenum;	NCBI: 83262	5649917	99.61	2.15	Yes
NCBI Assembly	GCA_001325265.1	NCBI: cellular organisms; Bacteria; Terrabacteria group; Actinobacteria; Actinomycetia; Corynebacteriales; Mycobacteriaceae; Mycobacterium; Mycobacterium tuberculosis complex; Mycobacterium tuberculosis;	NCBI: 1773	4346713	99.94	0.00	Yes
NCBI Assembly	GCA_001322125.1	NCBI: cellular organisms; Bacteria; Terrabacteria group; Actinobacteria; Actinomycetia; Corynebacteriales; Mycobacteriaceae; Mycobacterium; Mycobacterium tuberculosis complex; Mycobacterium tuberculosis;	NCBI: 1773	4363127	99.94	0.00	Yes
NCBI Assembly	GCA_001345075.1	NCBI: cellular organisms; Bacteria; Terrabacteria group; Actinobacteria; Actinomycetia; Corynebacteriales; Mycobacteriaceae; Mycobacterium; Mycobacterium tuberculosis complex; Mycobacterium tuberculosis;	NCBI: 1773	4332140	99.61	0.00	Yes
NCBI Assembly	GCA_001377395.1	NCBI: cellular organisms; Bacteria; Terrabacteria group; Actinobacteria; Actinomycetia; Corynebacteriales; Mycobacteriaceae; Mycobacterium; Mycobacterium tuberculosis complex; Mycobacterium tuberculosis;	NCBI: 1773	4365065	99.94	0.00	Yes
NCBI Assembly	GCA_001379295.1	NCBI: cellular organisms; Bacteria; Terrabacteria group; Actinobacteria; Actinomycetia; Corynebacteriales; Mycobacteriaceae; Mycobacterium; Mycobacterium tuberculosis complex; Mycobacterium tuberculosis;	NCBI: 1773	4370578	99.94	0.00	Yes
NCBI Assembly	GCA_001318525.1	NCBI: cellular organisms; Bacteria; Terrabacteria group; Actinobacteria; Actinomycetia; Corynebacteriales; Mycobacteriaceae; Mycobacterium; Mycobacterium tuberculosis complex; Mycobacterium tuberculosis;	NCBI: 1773	4298093	99.61	0.00	Yes
NCBI Assembly	GCA_001021495.1	NCBI: cellular organisms; Bacteria; Terrabacteria group; Actinobacteria; Actinomycetia; Corynebacteriales; Mycobacteriaceae; Mycobacterium; Mycobacterium nebraskense;	NCBI: 244292	6539988	99.94	1.16	Yes
NCBI Assembly	GCA_000805385.1	NCBI: cellular organisms; Bacteria; Terrabacteria group; Actinobacteria; Actinomycetia; Corynebacteriales; Mycobacteriaceae; Mycolicibacterium; Mycolicibacterium setense;	NCBI: 431269	6265049	99.81	1.32	Yes
NCBI Assembly	GCA_001324325.1	NCBI: cellular organisms; Bacteria; Terrabacteria group; Actinobacteria; Actinomycetia; Corynebacteriales; Mycobacteriaceae; Mycobacterium; Mycobacterium tuberculosis complex; Mycobacterium tuberculosis;	NCBI: 1773	4368843	99.94	0.00	Yes
NCBI Assembly	GCA_001198275.1	NCBI: cellular organisms; Bacteria; Terrabacteria group; Actinobacteria; Actinomycetia; Corynebacteriales; Mycobacteriaceae; Mycobacterium; Mycobacterium tuberculosis complex; Mycobacterium tuberculosis;	NCBI: 1773	4316335	99.61	0.00	Yes
NCBI Assembly	GCA_001165085.1	NCBI: cellular organisms; Bacteria; Terrabacteria group; Actinobacteria; Actinomycetia; Corynebacteriales; Mycobacteriaceae; Mycobacterium; Mycobacterium tuberculosis complex; Mycobacterium tuberculosis;	NCBI: 1773	4236876	99.94	0.73	Yes
NCBI Assembly	GCA_001296255.1	NCBI: cellular organisms; Bacteria; Terrabacteria group; Actinobacteria; Actinomycetia; Corynebacteriales; Mycobacteriaceae; Mycobacteroides; Mycobacteroides immunogenum;	NCBI: 83262	5723552	99.61	2.15	Yes
NCBI Assembly	GCA_001325285.1	NCBI: cellular organisms; Bacteria; Terrabacteria group; Actinobacteria; Actinomycetia; Corynebacteriales; Mycobacteriaceae; Mycobacterium; Mycobacterium tuberculosis complex; Mycobacterium tuberculosis;	NCBI: 1773	4385048	99.94	0.00	Yes

NCBI Assembly	GCA_001322145.1	NCBI: cellular organisms; Bacteria; Terrabacteria group; Actinobacteria; Actinomycetia; Corynebacteriales; Mycobacteriaceae; Mycobacterium; Mycobacterium tuberculosis complex; Mycobacterium tuberculosis;	NCBI: 1773	4366262	99.94	0.00	Yes
NCBI Assembly	GCA_001345095.1	NCBI: cellular organisms; Bacteria; Terrabacteria group; Actinobacteria; Actinomycetia; Corynebacteriales; Mycobacteriaceae; Mycobacterium; Mycobacterium tuberculosis complex; Mycobacterium tuberculosis;	NCBI: 1773	4313331	99.61	0.00	Yes
NCBI Assembly	GCA_001377415.1	NCBI: cellular organisms; Bacteria; Terrabacteria group; Actinobacteria; Actinomycetia; Corynebacteriales; Mycobacteriaceae; Mycobacterium; Mycobacterium tuberculosis complex; Mycobacterium tuberculosis;	NCBI: 1773	4350651	99.94	0.00	Yes
NCBI Assembly	GCA_001379315.1	NCBI: cellular organisms; Bacteria; Terrabacteria group; Actinobacteria; Actinomycetia; Corynebacteriales; Mycobacteriaceae; Mycobacterium; Mycobacterium tuberculosis complex; Mycobacterium tuberculosis;	NCBI: 1773	4365095	99.94	0.00	Yes
NCBI Assembly	GCA_001318545.1	NCBI: cellular organisms; Bacteria; Terrabacteria group; Actinobacteria; Actinomycetia; Corynebacteriales; Mycobacteriaceae; Mycobacterium; Mycobacterium tuberculosis complex; Mycobacterium tuberculosis;	NCBI: 1773	4278775	99.58	1.38	Yes
NCBI Assembly	GCA_000805885.1	NCBI: cellular organisms; Bacteria; Terrabacteria group; Actinobacteria; Actinomycetia; Corynebacteriales; Mycobacteriaceae; Mycobacterium; Mycobacterium tuberculosis complex; Mycobacterium tuberculosis;	NCBI: 1773	4240703	99.11	0.00	Yes
NCBI Assembly	GCA_001375935.1	NCBI: cellular organisms; Bacteria; Terrabacteria group; Actinobacteria; Actinomycetia; Corynebacteriales; Mycobacteriaceae; Mycobacterium; Mycobacterium tuberculosis complex; Mycobacterium tuberculosis;	NCBI: 1773	4356942	99.94	0.00	Yes
NCBI Assembly	GCA_001324345.1	NCBI: cellular organisms; Bacteria; Terrabacteria group; Actinobacteria; Actinomycetia; Corynebacteriales; Mycobacteriaceae; Mycobacterium; Mycobacterium tuberculosis complex; Mycobacterium tuberculosis;	NCBI: 1773	4368930	99.94	0.00	Yes
NCBI Assembly	GCA_001166245.1	NCBI: cellular organisms; Bacteria; Terrabacteria group; Actinobacteria; Actinomycetia; Corynebacteriales; Mycobacteriaceae; Mycobacterium; Mycobacterium tuberculosis complex; Mycobacterium tuberculosis;	NCBI: 1773	4376163	99.94	0.00	Yes
NCBI Assembly	GCA_001328055.1	NCBI: cellular organisms; Bacteria; Terrabacteria group; Actinobacteria; Actinomycetia; Corynebacteriales; Mycobacteriaceae; Mycobacterium; Mycobacterium tuberculosis complex; Mycobacterium tuberculosis;	NCBI: 1773	4299509	99.50	0.33	Yes
NCBI Assembly	GCA_001325305.1	NCBI: cellular organisms; Bacteria; Terrabacteria group; Actinobacteria; Actinomycetia; Corynebacteriales; Mycobacteriaceae; Mycobacterium; Mycobacterium tuberculosis complex; Mycobacterium tuberculosis;	NCBI: 1773	4344429	99.94	0.00	Yes
NCBI Assembly	GCA_001296265.1	NCBI: cellular organisms; Bacteria; Terrabacteria group; Actinobacteria; Actinomycetia; Corynebacteriales; Mycobacteriaceae; Mycobacteroides; Mycobacteroides immunogenum;	NCBI: 83262	5644131	99.61	2.15	Yes
NCBI Assembly	GCA_001322165.1	NCBI: cellular organisms; Bacteria; Terrabacteria group; Actinobacteria; Actinomycetia; Corynebacteriales; Mycobacteriaceae; Mycobacterium; Mycobacterium tuberculosis complex; Mycobacterium tuberculosis;	NCBI: 1773	4354780	99.94	0.00	Yes
NCBI Assembly	GCA_001345115.1	NCBI: cellular organisms; Bacteria; Terrabacteria group; Actinobacteria; Actinomycetia; Corynebacteriales; Mycobacteriaceae; Mycobacterium; Mycobacterium tuberculosis complex; Mycobacterium tuberculosis;	NCBI: 1773	4355881	99.94	0.00	Yes
NCBI Assembly	GCA_001377435.1	NCBI: cellular organisms; Bacteria; Terrabacteria group; Actinobacteria; Actinomycetia; Corynebacteriales; Mycobacteriaceae; Mycobacterium; Mycobacterium tuberculosis complex; Mycobacterium tuberculosis;	NCBI: 1773	4361414	99.94	0.00	Yes
NCBI Assembly	GCA_001379335.1	NCBI: cellular organisms; Bacteria; Terrabacteria group; Actinobacteria; Actinomycetia; Corynebacteriales; Mycobacteriaceae; Mycobacterium; Mycobacterium tuberculosis complex; Mycobacterium tuberculosis;	NCBI: 1773	4365232	99.94	0.00	Yes
NCBI Assembly	GCA_001318565.1	NCBI: cellular organisms; Bacteria; Terrabacteria group; Actinobacteria; Actinomycetia; Corynebacteriales; Mycobacteriaceae; Mycobacterium; Mycobacterium tuberculosis complex; Mycobacterium tuberculosis;	NCBI: 1773	4297174	98.33	1.00	Yes
NCBI Assembly	GCA_000827085.1	NCBI: cellular organisms; Bacteria; Terrabacteria group; Actinobacteria; Actinomycetia; Corynebacteriales; Mycobacteriaceae; Mycobacterium; Mycobacterium tuberculosis complex; Mycobacterium tuberculosis;	NCBI: 1437856	4410911	99.94	0.00	Yes

NCBI Assembly	GCA_001375955.1	NCBI: cellular organisms; Bacteria; Terrabacteria group; Actinobacteria; Actinomycetia; Corynebacteriales; Mycobacteriaceae; Mycobacterium; Mycobacterium tuberculosis complex; Mycobacterium tuberculosis;	NCBI: 1773	4369616	99.94	0.00	Yes
NCBI Assembly	GCA_001324365.1	NCBI: cellular organisms; Bacteria; Terrabacteria group; Actinobacteria; Actinomycetia; Corynebacteriales; Mycobacteriaceae; Mycobacterium; Mycobacterium tuberculosis complex; Mycobacterium tuberculosis;	NCBI: 1773	4366577	99.94	0.00	Yes
NCBI Assembly	GCA_001328075.1	NCBI: cellular organisms; Bacteria; Terrabacteria group; Actinobacteria; Actinomycetia; Corynebacteriales; Mycobacteriaceae; Mycobacterium; Mycobacterium tuberculosis complex; Mycobacterium tuberculosis;	NCBI: 1773	4314369	99.94	0.00	Yes
NCBI Assembly	GCA_001327755.1	NCBI: cellular organisms; Bacteria; Terrabacteria group; Actinobacteria; Actinomycetia; Corynebacteriales; Mycobacteriaceae; Mycobacterium; Mycobacterium tuberculosis complex; Mycobacterium tuberculosis;	NCBI: 1773	4331328	99.94	0.00	Yes
NCBI Assembly	GCA_001345195.1	NCBI: cellular organisms; Bacteria; Terrabacteria group; Actinobacteria; Actinomycetia; Corynebacteriales; Mycobacteriaceae; Mycobacterium; Mycobacterium tuberculosis complex; Mycobacterium tuberculosis;	NCBI: 1773	4193070	98.30	1.94	Yes
NCBI Assembly	GCA_001325325.1	NCBI: cellular organisms; Bacteria; Terrabacteria group; Actinobacteria; Actinomycetia; Corynebacteriales; Mycobacteriaceae; Mycobacterium; Mycobacterium tuberculosis complex; Mycobacterium tuberculosis;	NCBI: 1773	4364525	99.94	0.33	Yes
NCBI Assembly	GCA_001322185.1	NCBI: cellular organisms; Bacteria; Terrabacteria group; Actinobacteria; Actinomycetia; Corynebacteriales; Mycobacteriaceae; Mycobacterium; Mycobacterium tuberculosis complex; Mycobacterium tuberculosis;	NCBI: 1773	4363551	99.94	0.00	Yes
NCBI Assembly	GCA_001345135.1	NCBI: cellular organisms; Bacteria; Terrabacteria group; Actinobacteria; Actinomycetia; Corynebacteriales; Mycobacteriaceae; Mycobacterium; Mycobacterium tuberculosis complex; Mycobacterium tuberculosis;	NCBI: 1773	4269321	99.04	0.76	Yes
NCBI Assembly	GCA_001377455.1	NCBI: cellular organisms; Bacteria; Terrabacteria group; Actinobacteria; Actinomycetia; Corynebacteriales; Mycobacteriaceae; Mycobacterium; Mycobacterium tuberculosis complex; Mycobacterium tuberculosis;	NCBI: 1773	4357015	99.94	0.00	Yes
NCBI Assembly	GCA_000828995.1	NCBI: cellular organisms; Bacteria; Terrabacteria group; Actinobacteria; Actinomycetia; Corynebacteriales; Mycobacteriaceae; Mycobacterium; Mycobacterium tuberculosis complex; Mycobacterium tuberculosis;	NCBI: 1445606	4415078	99.94	0.00	Yes
NCBI Assembly	GCA_000803985.1	NCBI: cellular organisms; Bacteria; Terrabacteria group; Actinobacteria; Actinomycetia; Corynebacteriales; Mycobacteriaceae; Mycobacterium; Mycobacterium tuberculosis complex; Mycobacterium tuberculosis;	NCBI: 1773	4374716	99.94	0.00	Yes
NCBI Assembly	GCA_001323165.1	NCBI: cellular organisms; Bacteria; Terrabacteria group; Actinobacteria; Actinomycetia; Corynebacteriales; Mycobacteriaceae; Mycobacterium; Mycobacterium tuberculosis complex; Mycobacterium tuberculosis;	NCBI: 1773	4369168	99.94	0.33	Yes
NCBI Assembly	GCA_001375975.1	NCBI: cellular organisms; Bacteria; Terrabacteria group; Actinobacteria; Actinomycetia; Corynebacteriales; Mycobacteriaceae; Mycobacterium; Mycobacterium tuberculosis complex; Mycobacterium tuberculosis;	NCBI: 1773	4362752	99.94	0.00	Yes
NCBI Assembly	GCA_001324385.1	NCBI: cellular organisms; Bacteria; Terrabacteria group; Actinobacteria; Actinomycetia; Corynebacteriales; Mycobacteriaceae; Mycobacterium; Mycobacterium tuberculosis complex; Mycobacterium tuberculosis;	NCBI: 1773	4364626	99.94	0.00	Yes
NCBI Assembly	GCA_001327775.1	NCBI: cellular organisms; Bacteria; Terrabacteria group; Actinobacteria; Actinomycetia; Corynebacteriales; Mycobacteriaceae; Mycobacterium; Mycobacterium tuberculosis complex; Mycobacterium tuberculosis;	NCBI: 1773	4328302	99.94	0.00	Yes
NCBI Assembly	GCA_001328095.1	NCBI: cellular organisms; Bacteria; Terrabacteria group; Actinobacteria; Actinomycetia; Corynebacteriales; Mycobacteriaceae; Mycobacterium; Mycobacterium tuberculosis complex; Mycobacterium tuberculosis;	NCBI: 1773	4321711	99.24	0.00	Yes
NCBI Assembly	GCA_001322845.1	NCBI: cellular organisms; Bacteria; Terrabacteria group; Actinobacteria; Actinomycetia; Corynebacteriales; Mycobacteriaceae; Mycobacterium; Mycobacterium tuberculosis complex; Mycobacterium tuberculosis;	NCBI: 1773	4391082	99.94	0.00	Yes
NCBI Assembly	GCA_001345215.1	NCBI: cellular organisms; Bacteria; Terrabacteria group; Actinobacteria; Actinomycetia; Corynebacteriales; Mycobacteriaceae; Mycobacterium; Mycobacterium tuberculosis complex; Mycobacterium tuberculosis;	NCBI: 1773	4311174	99.61	0.00	Yes

NCBI Assembly	GCA_001345155.1	NCBI: cellular organisms; Bacteria; Terrabacteria group; Actinobacteria; Actinomycetia; Corynebacteriales; Mycobacteriaceae; Mycobacterium; Mycobacterium tuberculosis complex; Mycobacterium tuberculosis;	NCBI: 1773	4248504	98.48	1.00	Yes
NCBI Assembly	GCA_001322205.1	NCBI: cellular organisms; Bacteria; Terrabacteria group; Actinobacteria; Actinomycetia; Corynebacteriales; Mycobacteriaceae; Mycobacterium; Mycobacterium tuberculosis complex; Mycobacterium tuberculosis;	NCBI: 1773	4362128	99.94	0.00	Yes
NCBI Assembly	GCA_000804005.1	NCBI: cellular organisms; Bacteria; Terrabacteria group; Actinobacteria; Actinomycetia; Corynebacteriales; Mycobacteriaceae; Mycobacterium; Mycobacterium tuberculosis complex; Mycobacterium tuberculosis;	NCBI: 1773	4376843	99.00	0.00	Yes
NCBI Assembly	GCA_000829075.1	NCBI: cellular organisms; Bacteria; Terrabacteria group; Actinobacteria; Actinomycetia; Corynebacteriales; Mycobacteriaceae; Mycobacterium; Mycobacterium avium complex (MAC); Mycobacterium avium; Mycobacterium avium subsp. hominissuis;	NCBI: 1229671	5145928	99.94	0.17	Yes
NCBI Assembly	GCA_001377475.1	NCBI: cellular organisms; Bacteria; Terrabacteria group; Actinobacteria; Actinomycetia; Corynebacteriales; Mycobacteriaceae; Mycobacterium; Mycobacterium tuberculosis complex; Mycobacterium tuberculosis;	NCBI: 1773	4356043	99.94	0.00	Yes
NCBI Assembly	GCA_001323185.1	NCBI: cellular organisms; Bacteria; Terrabacteria group; Actinobacteria; Actinomycetia; Corynebacteriales; Mycobacteriaceae; Mycobacterium; Mycobacterium tuberculosis complex; Mycobacterium tuberculosis;	NCBI: 1773	4398728	99.94	0.00	Yes
NCBI Assembly	GCA_001375995.1	NCBI: cellular organisms; Bacteria; Terrabacteria group; Actinobacteria; Actinomycetia; Corynebacteriales; Mycobacteriaceae; Mycobacterium; Mycobacterium tuberculosis complex; Mycobacterium tuberculosis;	NCBI: 1773	4376071	99.94	0.00	Yes
NCBI Assembly	GCA_001323785.1	NCBI: cellular organisms; Bacteria; Terrabacteria group; Actinobacteria; Actinomycetia; Corynebacteriales; Mycobacteriaceae; Mycobacterium; Mycobacterium tuberculosis complex; Mycobacterium tuberculosis;	NCBI: 1773	4369497	99.94	0.17	Yes
NCBI Assembly	GCA_001327795.1	NCBI: cellular organisms; Bacteria; Terrabacteria group; Actinobacteria; Actinomycetia; Corynebacteriales; Mycobacteriaceae; Mycobacterium; Mycobacterium tuberculosis complex; Mycobacterium tuberculosis;	NCBI: 1773	4303610	99.94	0.00	Yes
NCBI Assembly	GCA_001322865.1	NCBI: cellular organisms; Bacteria; Terrabacteria group; Actinobacteria; Actinomycetia; Corynebacteriales; Mycobacteriaceae; Mycobacterium; Mycobacterium tuberculosis complex; Mycobacterium tuberculosis;	NCBI: 1773	4404440	99.94	0.00	Yes
NCBI Assembly	GCA_001345235.1	NCBI: cellular organisms; Bacteria; Terrabacteria group; Actinobacteria; Actinomycetia; Corynebacteriales; Mycobacteriaceae; Mycobacterium; Mycobacterium tuberculosis complex; Mycobacterium tuberculosis;	NCBI: 1773	4346837	99.94	0.00	Yes
NCBI Assembly	GCA_001328115.1	NCBI: cellular organisms; Bacteria; Terrabacteria group; Actinobacteria; Actinomycetia; Corynebacteriales; Mycobacteriaceae; Mycobacterium; Mycobacterium tuberculosis complex; Mycobacterium tuberculosis;	NCBI: 1773	4315006	99.61	0.07	Yes
NCBI Assembly	GCA_000804025.1	NCBI: cellular organisms; Bacteria; Terrabacteria group; Actinobacteria; Actinomycetia; Corynebacteriales; Mycobacteriaceae; Mycobacterium; Mycobacterium tuberculosis complex; Mycobacterium tuberculosis;	NCBI: 1773	4352793	99.94	0.00	Yes
NCBI Assembly	GCA_001323465.1	NCBI: cellular organisms; Bacteria; Terrabacteria group; Actinobacteria; Actinomycetia; Corynebacteriales; Mycobacteriaceae; Mycobacterium; Mycobacterium tuberculosis complex; Mycobacterium tuberculosis;	NCBI: 1773	4364164	99.61	0.00	Yes
NCBI Assembly	GCA_001345175.1	NCBI: cellular organisms; Bacteria; Terrabacteria group; Actinobacteria; Actinomycetia; Corynebacteriales; Mycobacteriaceae; Mycobacterium; Mycobacterium tuberculosis complex; Mycobacterium tuberculosis;	NCBI: 1773	4281527	98.97	0.33	Yes
NCBI Assembly	GCA_000830545.2	NCBI: cellular organisms; Bacteria; Terrabacteria group; Actinobacteria; Actinomycetia; Corynebacteriales; Mycobacteriaceae; Mycobacterium; Mycobacterium tuberculosis complex; Mycobacterium tuberculosis;	NCBI: 1773	4334870	99.94	0.00	Yes
NCBI Assembly	GCA_001369335.1	NCBI: cellular organisms; Bacteria; Terrabacteria group; Actinobacteria; Actinomycetia; Corynebacteriales; Mycobacteriaceae; Mycobacterium; Mycobacterium tuberculosis complex; Mycobacterium tuberculosis; Mycobacterium tuberculosis variant bovis;	NCBI: 1765	4255612	99.78	0.00	Yes
NCBI Assembly	GCA_001323205.1	NCBI: cellular organisms; Bacteria; Terrabacteria group; Actinobacteria; Actinomycetia; Corynebacteriales; Mycobacteriaceae; Mycobacterium; Mycobacterium tuberculosis complex; Mycobacterium tuberculosis;	NCBI: 1773	4370695	99.94	0.00	Yes

NCBI Assembly	GCA_001323805.1	NCBI: cellular organisms; Bacteria; Terrabacteria group; Actinobacteria; Actinomycetia; Corynebacteriales; Mycobacteriaceae; Mycobacterium; Mycobacterium tuberculosis complex; Mycobacterium tuberculosis;	NCBI: 1773	4391247	99.94	0.00	Yes
NCBI Assembly	GCA_001376015.1	NCBI: cellular organisms; Bacteria; Terrabacteria group; Actinobacteria; Actinomycetia; Corynebacteriales; Mycobacteriaceae; Mycobacterium; Mycobacterium tuberculosis complex; Mycobacterium tuberculosis;	NCBI: 1773	4366598	99.94	0.00	Yes
NCBI Assembly	GCA_001322885.1	NCBI: cellular organisms; Bacteria; Terrabacteria group; Actinobacteria; Actinomycetia; Corynebacteriales; Mycobacteriaceae; Mycobacterium; Mycobacterium tuberculosis complex; Mycobacterium tuberculosis;	NCBI: 1773	4366838	99.94	0.00	Yes
NCBI Assembly	GCA_001345255.1	NCBI: cellular organisms; Bacteria; Terrabacteria group; Actinobacteria; Actinomycetia; Corynebacteriales; Mycobacteriaceae; Mycobacterium; Mycobacterium tuberculosis complex; Mycobacterium tuberculosis;	NCBI: 1773	4285454	99.41	0.36	Yes
NCBI Assembly	GCA_001327815.1	NCBI: cellular organisms; Bacteria; Terrabacteria group; Actinobacteria; Actinomycetia; Corynebacteriales; Mycobacteriaceae; Mycobacterium; Mycobacterium tuberculosis complex; Mycobacterium tuberculosis;	NCBI: 1773	4336613	99.94	0.00	Yes
NCBI Assembly	GCA_001328135.1	NCBI: cellular organisms; Bacteria; Terrabacteria group; Actinobacteria; Actinomycetia; Corynebacteriales; Mycobacteriaceae; Mycobacterium; Mycobacterium tuberculosis complex; Mycobacterium tuberculosis;	NCBI: 1773	4342316	99.94	0.00	Yes
NCBI Assembly	GCA_000835125.1	NCBI: cellular organisms; Bacteria; Terrabacteria group; Actinobacteria; Actinomycetia; Corynebacteriales; Mycobacteriaceae; Mycobacterium; Mycobacterium tuberculosis complex; Mycobacterium tuberculosis;	NCBI: 1452723	4411953	99.94	0.00	Yes
NCBI Assembly	GCA_000804045.1	NCBI: cellular organisms; Bacteria; Terrabacteria group; Actinobacteria; Actinomycetia; Corynebacteriales; Mycobacteriaceae; Mycobacterium; Mycobacterium tuberculosis complex; Mycobacterium tuberculosis;	NCBI: 1773	4352308	99.94	0.00	Yes
NCBI Assembly	GCA_001323485.1	NCBI: cellular organisms; Bacteria; Terrabacteria group; Actinobacteria; Actinomycetia; Corynebacteriales; Mycobacteriaceae; Mycobacterium; Mycobacterium tuberculosis complex; Mycobacterium tuberculosis;	NCBI: 1773	4367527	99.94	0.00	Yes
NCBI Assembly	GCA_001322545.1	NCBI: cellular organisms; Bacteria; Terrabacteria group; Actinobacteria; Actinomycetia; Corynebacteriales; Mycobacteriaceae; Mycobacterium; Mycobacterium tuberculosis complex; Mycobacterium tuberculosis;	NCBI: 1773	4362874	99.94	0.00	Yes
NCBI Assembly	GCA_001372235.1	NCBI: cellular organisms; Bacteria; Terrabacteria group; Actinobacteria; Actinomycetia; Corynebacteriales; Mycobacteriaceae; Mycobacterium; Mycobacterium tuberculosis complex; Mycobacterium tuberculosis;	NCBI: 1773	4145007	96.09	0.33	Yes
NCBI Assembly	GCA_001323225.1	NCBI: cellular organisms; Bacteria; Terrabacteria group; Actinobacteria; Actinomycetia; Corynebacteriales; Mycobacteriaceae; Mycobacterium; Mycobacterium tuberculosis complex; Mycobacterium tuberculosis;	NCBI: 1773	4380756	99.94	0.00	Yes
NCBI Assembly	GCA_001323825.1	NCBI: cellular organisms; Bacteria; Terrabacteria group; Actinobacteria; Actinomycetia; Corynebacteriales; Mycobacteriaceae; Mycobacterium; Mycobacterium tuberculosis complex; Mycobacterium tuberculosis;	NCBI: 1773	4366994	99.94	0.00	Yes
NCBI Assembly	GCA_001376035.1	NCBI: cellular organisms; Bacteria; Terrabacteria group; Actinobacteria; Actinomycetia; Corynebacteriales; Mycobacteriaceae; Mycobacterium; Mycobacterium tuberculosis complex; Mycobacterium tuberculosis;	NCBI: 1773	4365841	99.94	0.00	Yes
NCBI Assembly	GCA_001322905.1	NCBI: cellular organisms; Bacteria; Terrabacteria group; Actinobacteria; Actinomycetia; Corynebacteriales; Mycobacteriaceae; Mycobacterium; Mycobacterium tuberculosis complex; Mycobacterium tuberculosis;	NCBI: 1773	4470862	99.94	2.73	Yes
NCBI Assembly	GCA_001327835.1	NCBI: cellular organisms; Bacteria; Terrabacteria group; Actinobacteria; Actinomycetia; Corynebacteriales; Mycobacteriaceae; Mycobacterium; Mycobacterium tuberculosis complex; Mycobacterium tuberculosis;	NCBI: 1773	4309244	99.61	0.00	Yes
NCBI Assembly	GCA_001328155.1	NCBI: cellular organisms; Bacteria; Terrabacteria group; Actinobacteria; Actinomycetia; Corynebacteriales; Mycobacteriaceae; Mycobacterium; Mycobacterium tuberculosis complex; Mycobacterium tuberculosis;	NCBI: 1773	4271532	99.94	0.00	Yes
NCBI Assembly	GCA_000804055.1	NCBI: cellular organisms; Bacteria; Terrabacteria group; Actinobacteria; Actinomycetia; Corynebacteriales; Mycobacteriaceae; Mycobacterium; Mycobacterium tuberculosis complex; Mycobacterium tuberculosis;	NCBI: 1773	4366392	99.94	0.00	Yes

NCBI Assembly	GCA_001345275.1	NCBI: cellular organisms; Bacteria; Terrabacteria group; Actinobacteria; Actinomycetia; Corynebacteriales; Mycobacteriaceae; Mycobacterium; Mycobacterium tuberculosis complex; Mycobacterium tuberculosis;	NCBI: 1773	4299419	99.28	0.00	Yes
NCBI Assembly	GCA_000835225.1	NCBI: cellular organisms; Bacteria; Terrabacteria group; Actinobacteria; Actinomycetia; Corynebacteriales; Mycobacteriaceae; Mycobacterium; Mycobacterium avium complex (MAC); Mycobacterium avium; Mycobacterium avium subsp. paratuberculosis;	NCBI: 1770	4781002	97.89	0.33	Yes
NCBI Assembly	GCA_001323505.1	NCBI: cellular organisms; Bacteria; Terrabacteria group; Actinobacteria; Actinomycetia; Corynebacteriales; Mycobacteriaceae; Mycobacterium; Mycobacterium tuberculosis complex; Mycobacterium tuberculosis;	NCBI: 1773	4366924	99.94	0.00	Yes
NCBI Assembly	GCA_001322565.1	NCBI: cellular organisms; Bacteria; Terrabacteria group; Actinobacteria; Actinomycetia; Corynebacteriales; Mycobacteriaceae; Mycobacterium; Mycobacterium tuberculosis complex; Mycobacterium tuberculosis;	NCBI: 1773	4317535	99.94	0.33	Yes
NCBI Assembly	GCA_001323245.1	NCBI: cellular organisms; Bacteria; Terrabacteria group; Actinobacteria; Actinomycetia; Corynebacteriales; Mycobacteriaceae; Mycobacterium; Mycobacterium tuberculosis complex; Mycobacterium tuberculosis;	NCBI: 1773	4372054	99.94	0.00	Yes
NCBI Assembly	GCA_000804065.1	NCBI: cellular organisms; Bacteria; Terrabacteria group; Actinobacteria; Actinomycetia; Corynebacteriales; Mycobacteriaceae; Mycobacterium; Mycobacterium tuberculosis complex; Mycobacterium tuberculosis;	NCBI: 1773	4363699	99.94	0.00	Yes
NCBI Assembly	GCA_001323845.1	NCBI: cellular organisms; Bacteria; Terrabacteria group; Actinobacteria; Actinomycetia; Corynebacteriales; Mycobacteriaceae; Mycobacterium; Mycobacterium tuberculosis complex; Mycobacterium tuberculosis;	NCBI: 1773	4366934	99.94	0.00	Yes
NCBI Assembly	GCA_001322925.1	NCBI: cellular organisms; Bacteria; Terrabacteria group; Actinobacteria; Actinomycetia; Corynebacteriales; Mycobacteriaceae; Mycobacterium; Mycobacterium tuberculosis complex; Mycobacterium tuberculosis;	NCBI: 1773	4390972	99.94	0.00	Yes
NCBI Assembly	GCA_001328175.1	NCBI: cellular organisms; Bacteria; Terrabacteria group; Actinobacteria; Actinomycetia; Corynebacteriales; Mycobacteriaceae; Mycobacterium; Mycobacterium tuberculosis complex; Mycobacterium tuberculosis;	NCBI: 1773	4332037	99.61	0.00	Yes
NCBI Assembly	GCA_001327855.1	NCBI: cellular organisms; Bacteria; Terrabacteria group; Actinobacteria; Actinomycetia; Corynebacteriales; Mycobacteriaceae; Mycobacterium; Mycobacterium tuberculosis complex; Mycobacterium tuberculosis;	NCBI: 1773	4289378	99.94	0.00	Yes
NCBI Assembly	GCA_000835265.1	NCBI: cellular organisms; Bacteria; Terrabacteria group; Actinobacteria; Actinomycetia; Corynebacteriales; Mycobacteriaceae; Mycobacterium; Mycobacterium avium complex (MAC); Mycobacterium avium; Mycobacterium avium subsp. paratuberculosis;	NCBI: 1770	4786065	99.21	0.33	Yes
NCBI Assembly	GCA_001376055.1	NCBI: cellular organisms; Bacteria; Terrabacteria group; Actinobacteria; Actinomycetia; Corynebacteriales; Mycobacteriaceae; Mycobacterium; Mycobacterium tuberculosis complex; Mycobacterium tuberculosis;	NCBI: 1773	4350362	99.94	0.00	Yes
NCBI Assembly	GCA_001345295.1	NCBI: cellular organisms; Bacteria; Terrabacteria group; Actinobacteria; Actinomycetia; Corynebacteriales; Mycobacteriaceae; Mycobacterium; Mycobacterium tuberculosis complex; Mycobacterium tuberculosis;	NCBI: 1773	4247649	98.96	1.36	Yes
NCBI Assembly	GCA_001323525.1	NCBI: cellular organisms; Bacteria; Terrabacteria group; Actinobacteria; Actinomycetia; Corynebacteriales; Mycobacteriaceae; Mycobacterium; Mycobacterium tuberculosis complex; Mycobacterium tuberculosis;	NCBI: 1773	4368874	99.94	0.00	Yes
NCBI Assembly	GCA_001322585.1	NCBI: cellular organisms; Bacteria; Terrabacteria group; Actinobacteria; Actinomycetia; Corynebacteriales; Mycobacteriaceae; Mycobacterium; Mycobacterium tuberculosis complex; Mycobacterium tuberculosis;	NCBI: 1773	4388990	99.94	0.00	Yes
NCBI Assembly	GCA_001323265.1	NCBI: cellular organisms; Bacteria; Terrabacteria group; Actinobacteria; Actinomycetia; Corynebacteriales; Mycobacteriaceae; Mycobacterium; Mycobacterium tuberculosis complex; Mycobacterium tuberculosis;	NCBI: 1773	4373767	99.94	0.00	Yes
NCBI Assembly	GCA_001373515.1	NCBI: cellular organisms; Bacteria; Terrabacteria group; Actinobacteria; Actinomycetia; Corynebacteriales; Mycobacteriaceae; Mycobacterium; Mycobacterium simiae complex; Mycobacterium europaeum;	NCBI: 761804	6152523	99.94	0.88	Yes
NCBI Assembly	GCA_000804105.1	NCBI: cellular organisms; Bacteria; Terrabacteria group; Actinobacteria; Actinomycetia; Corynebacteriales; Mycobacteriaceae; Mycobacterium; Mycobacterium tuberculosis complex; Mycobacterium tuberculosis;	NCBI: 1773	4356542	99.61	0.00	Yes

NCBI Assembly	GCA_001322945.1	NCBI: cellular organisms; Bacteria; Terrabacteria group; Actinobacteria; Actinomycetia; Corynebacteriales; Mycobacteriaceae; Mycobacterium; Mycobacterium tuberculosis complex; Mycobacterium tuberculosis;	NCBI: 1773	4365142	99.94	0.00	Yes
NCBI Assembly	GCA_001327875.1	NCBI: cellular organisms; Bacteria; Terrabacteria group; Actinobacteria; Actinomycetia; Corynebacteriales; Mycobacteriaceae; Mycobacterium; Mycobacterium tuberculosis complex; Mycobacterium tuberculosis;	NCBI: 1773	4293545	99.94	0.00	Yes
NCBI Assembly	GCA_001323865.1	NCBI: cellular organisms; Bacteria; Terrabacteria group; Actinobacteria; Actinomycetia; Corynebacteriales; Mycobacteriaceae; Mycobacterium; Mycobacterium tuberculosis complex; Mycobacterium tuberculosis;	NCBI: 1773	4368641	99.94	0.00	Yes
NCBI Assembly	GCA_000878195.1	NCBI: cellular organisms; Bacteria; Terrabacteria group; Actinobacteria; Actinomycetia; Corynebacteriales; Mycobacteriaceae; Mycolicibacterium; Mycolicibacterium llatzerense;	NCBI: 280871	6091591	99.81	1.38	Yes
NCBI Assembly	GCA_001328195.1	NCBI: cellular organisms; Bacteria; Terrabacteria group; Actinobacteria; Actinomycetia; Corynebacteriales; Mycobacteriaceae; Mycobacterium; Mycobacterium tuberculosis complex; Mycobacterium tuberculosis;	NCBI: 1773	4294803	99.89	0.00	Yes
NCBI Assembly	GCA_001376075.1	NCBI: cellular organisms; Bacteria; Terrabacteria group; Actinobacteria; Actinomycetia; Corynebacteriales; Mycobacteriaceae; Mycobacterium; Mycobacterium tuberculosis complex; Mycobacterium tuberculosis;	NCBI: 1773	4361650	99.94	0.00	Yes
NCBI Assembly	GCA_001345315.1	NCBI: cellular organisms; Bacteria; Terrabacteria group; Actinobacteria; Actinomycetia; Corynebacteriales; Mycobacteriaceae; Mycobacterium; Mycobacterium tuberculosis complex; Mycobacterium tuberculosis;	NCBI: 1773	4297256	99.86	1.43	Yes
NCBI Assembly	GCA_001322605.1	NCBI: cellular organisms; Bacteria; Terrabacteria group; Actinobacteria; Actinomycetia; Corynebacteriales; Mycobacteriaceae; Mycobacterium; Mycobacterium tuberculosis complex; Mycobacterium tuberculosis;	NCBI: 1773	4361007	99.94	0.00	Yes
NCBI Assembly	GCA_001323545.1	NCBI: cellular organisms; Bacteria; Terrabacteria group; Actinobacteria; Actinomycetia; Corynebacteriales; Mycobacteriaceae; Mycobacterium; Mycobacterium tuberculosis complex; Mycobacterium tuberculosis;	NCBI: 1773	4363549	99.94	0.00	Yes
NCBI Assembly	GCA_001375575.1	NCBI: cellular organisms; Bacteria; Terrabacteria group; Actinobacteria; Actinomycetia; Corynebacteriales; Mycobacteriaceae; Mycobacterium; Mycobacterium tuberculosis complex; Mycobacterium tuberculosis;	NCBI: 1773	4110293	95.34	0.17	Yes
NCBI Assembly	GCA_001323285.1	NCBI: cellular organisms; Bacteria; Terrabacteria group; Actinobacteria; Actinomycetia; Corynebacteriales; Mycobacteriaceae; Mycobacterium; Mycobacterium tuberculosis complex; Mycobacterium tuberculosis;	NCBI: 1773	4379271	99.94	0.00	Yes
NCBI Assembly	GCA_000804145.1	NCBI: cellular organisms; Bacteria; Terrabacteria group; Actinobacteria; Actinomycetia; Corynebacteriales; Mycobacteriaceae; Mycobacterium; Mycobacterium tuberculosis complex; Mycobacterium tuberculosis;	NCBI: 1773	4377731	99.94	0.00	Yes
NCBI Assembly	GCA_001322965.1	NCBI: cellular organisms; Bacteria; Terrabacteria group; Actinobacteria; Actinomycetia; Corynebacteriales; Mycobacteriaceae; Mycobacterium; Mycobacterium tuberculosis complex; Mycobacterium tuberculosis;	NCBI: 1773	4362866	99.94	0.00	Yes
NCBI Assembly	GCA_001323885.1	NCBI: cellular organisms; Bacteria; Terrabacteria group; Actinobacteria; Actinomycetia; Corynebacteriales; Mycobacteriaceae; Mycobacterium; Mycobacterium tuberculosis complex; Mycobacterium tuberculosis;	NCBI: 1773	4368677	99.94	0.00	Yes
NCBI Assembly	GCA_000878425.1	NCBI: cellular organisms; Bacteria; Terrabacteria group; Actinobacteria; Actinomycetia; Corynebacteriales; Mycobacteriaceae; Mycobacteroides; Mycobacteroides immunogenum;	NCBI: 83262	5566719	99.61	2.15	Yes
NCBI Assembly	GCA_001327895.1	NCBI: cellular organisms; Bacteria; Terrabacteria group; Actinobacteria; Actinomycetia; Corynebacteriales; Mycobacteriaceae; Mycobacterium; Mycobacterium tuberculosis complex; Mycobacterium tuberculosis;	NCBI: 1773	4297702	99.94	0.33	Yes
NCBI Assembly	GCA_001376095.1	NCBI: cellular organisms; Bacteria; Terrabacteria group; Actinobacteria; Actinomycetia; Corynebacteriales; Mycobacteriaceae; Mycobacterium; Mycobacterium tuberculosis complex; Mycobacterium tuberculosis;	NCBI: 1773	4394019	99.94	0.00	Yes
NCBI Assembly	GCA_001345335.1	NCBI: cellular organisms; Bacteria; Terrabacteria group; Actinobacteria; Actinomycetia; Corynebacteriales; Mycobacteriaceae; Mycobacterium; Mycobacterium tuberculosis complex; Mycobacterium tuberculosis;	NCBI: 1773	4216457	99.33	1.36	Yes

NCBI Assembly	GCA_001328215.1	NCBI: cellular organisms; Bacteria; Terrabacteria group; Actinobacteria; Actinomycetia; Corynebacteriales; Mycobacteriaceae; Mycobacterium; Mycobacterium tuberculosis complex; Mycobacterium tuberculosis;	NCBI: 1773	4309617	99.94	0.33	Yes
NCBI Assembly	GCA_001322625.1	NCBI: cellular organisms; Bacteria; Terrabacteria group; Actinobacteria; Actinomycetia; Corynebacteriales; Mycobacteriaceae; Mycobacterium; Mycobacterium tuberculosis complex; Mycobacterium tuberculosis;	NCBI: 1773	4394152	99.94	0.00	Yes
NCBI Assembly	GCA_001323565.1	NCBI: cellular organisms; Bacteria; Terrabacteria group; Actinobacteria; Actinomycetia; Corynebacteriales; Mycobacteriaceae; Mycobacterium; Mycobacterium tuberculosis complex; Mycobacterium tuberculosis;	NCBI: 1773	4366228	99.94	0.00	Yes
NCBI Assembly	GCA_001323305.1	NCBI: cellular organisms; Bacteria; Terrabacteria group; Actinobacteria; Actinomycetia; Corynebacteriales; Mycobacteriaceae; Mycobacterium; Mycobacterium tuberculosis complex; Mycobacterium tuberculosis;	NCBI: 1773	4376453	99.94	0.00	Yes
NCBI Assembly	GCA_001375715.1	NCBI: cellular organisms; Bacteria; Terrabacteria group; Actinobacteria; Actinomycetia; Corynebacteriales; Mycobacteriaceae; Mycobacterium; Mycobacterium tuberculosis complex; Mycobacterium tuberculosis;	NCBI: 1773	4362591	99.94	0.00	Yes
NCBI Assembly	GCA_000804165.1	NCBI: cellular organisms; Bacteria; Terrabacteria group; Actinobacteria; Actinomycetia; Corynebacteriales; Mycobacteriaceae; Mycobacterium; Mycobacterium tuberculosis complex; Mycobacterium tuberculosis;	NCBI: 1773	4361326	99.94	0.00	Yes
NCBI Assembly	GCA_000878485.1	NCBI: cellular organisms; Bacteria; Terrabacteria group; Actinobacteria; Actinomycetia; Corynebacteriales; Mycobacteriaceae; Mycobacterium; Mycobacterium tuberculosis complex; Mycobacterium tuberculosis; Mycobacterium tuberculosis variant bovis;	NCBI: 1765	4304865	99.44	0.00	Yes
NCBI Assembly	GCA_001322985.1	NCBI: cellular organisms; Bacteria; Terrabacteria group; Actinobacteria; Actinomycetia; Corynebacteriales; Mycobacteriaceae; Mycobacterium; Mycobacterium tuberculosis complex; Mycobacterium tuberculosis;	NCBI: 1773	5129750	99.83	13.30	No
NCBI Assembly	GCA_001323905.1	NCBI: cellular organisms; Bacteria; Terrabacteria group; Actinobacteria; Actinomycetia; Corynebacteriales; Mycobacteriaceae; Mycobacterium; Mycobacterium tuberculosis complex; Mycobacterium tuberculosis;	NCBI: 1773	4366238	99.94	0.00	Yes
NCBI Assembly	GCA_001345355.1	NCBI: cellular organisms; Bacteria; Terrabacteria group; Actinobacteria; Actinomycetia; Corynebacteriales; Mycobacteriaceae; Mycobacterium; Mycobacterium tuberculosis complex; Mycobacterium tuberculosis;	NCBI: 1773	4276268	99.94	0.67	Yes
NCBI Assembly	GCA_001328235.1	NCBI: cellular organisms; Bacteria; Terrabacteria group; Actinobacteria; Actinomycetia; Corynebacteriales; Mycobacteriaceae; Mycobacterium; Mycobacterium tuberculosis complex; Mycobacterium tuberculosis;	NCBI: 1773	4292149	99.83	0.00	Yes
NCBI Assembly	GCA_001376115.1	NCBI: cellular organisms; Bacteria; Terrabacteria group; Actinobacteria; Actinomycetia; Corynebacteriales; Mycobacteriaceae; Mycobacterium; Mycobacterium tuberculosis complex; Mycobacterium tuberculosis;	NCBI: 1773	4363261	99.94	0.00	Yes
NCBI Assembly	GCA_001327915.1	NCBI: cellular organisms; Bacteria; Terrabacteria group; Actinobacteria; Actinomycetia; Corynebacteriales; Mycobacteriaceae; Mycobacterium; Mycobacterium tuberculosis complex; Mycobacterium tuberculosis;	NCBI: 1773	4313345	99.94	0.00	Yes
NCBI Assembly	GCA_001322645.1	NCBI: cellular organisms; Bacteria; Terrabacteria group; Actinobacteria; Actinomycetia; Corynebacteriales; Mycobacteriaceae; Mycobacterium; Mycobacterium tuberculosis complex; Mycobacterium tuberculosis;	NCBI: 1773	4364676	99.94	0.00	Yes
NCBI Assembly	GCA_001323585.1	NCBI: cellular organisms; Bacteria; Terrabacteria group; Actinobacteria; Actinomycetia; Corynebacteriales; Mycobacteriaceae; Mycobacterium; Mycobacterium tuberculosis complex; Mycobacterium tuberculosis;	NCBI: 1773	4367343	99.94	0.00	Yes
NCBI Assembly	GCA_001375735.1	NCBI: cellular organisms; Bacteria; Terrabacteria group; Actinobacteria; Actinomycetia; Corynebacteriales; Mycobacteriaceae; Mycobacterium; Mycobacterium tuberculosis complex; Mycobacterium tuberculosis;	NCBI: 1773	4366232	99.94	0.00	Yes
NCBI Assembly	GCA_001323325.1	NCBI: cellular organisms; Bacteria; Terrabacteria group; Actinobacteria; Actinomycetia; Corynebacteriales; Mycobacteriaceae; Mycobacterium; Mycobacterium tuberculosis complex; Mycobacterium tuberculosis;	NCBI: 1773	4377369	99.94	0.00	Yes
NCBI Assembly	GCA_000804185.1	NCBI: cellular organisms; Bacteria; Terrabacteria group; Actinobacteria; Actinomycetia; Corynebacteriales; Mycobacteriaceae; Mycobacterium; Mycobacterium tuberculosis complex; Mycobacterium tuberculosis;	NCBI: 1773	4360528	99.94	0.00	Yes

NCBI Assembly	GCA_001323005.1	NCBI: cellular organisms; Bacteria; Terrabacteria group; Actinobacteria; Actinomycetia; Corynebacteriales; Mycobacteriaceae; Mycobacterium; Mycobacterium tuberculosis complex; Mycobacterium tuberculosis;	NCBI: 1773	4369067	99.94	0.17	Yes
NCBI Assembly	GCA_001350475.1	NCBI: cellular organisms; Bacteria; Terrabacteria group; Actinobacteria; Actinomycetia; Corynebacteriales; Mycobacteriaceae; Mycobacterium; Mycobacterium tuberculosis complex; Mycobacterium tuberculosis;	NCBI: 1773	4360936	99.94	0.00	Yes
NCBI Assembly	GCA_001323925.1	NCBI: cellular organisms; Bacteria; Terrabacteria group; Actinobacteria; Actinomycetia; Corynebacteriales; Mycobacteriaceae; Mycobacterium; Mycobacterium tuberculosis complex; Mycobacterium tuberculosis;	NCBI: 1773	4375482	99.94	0.00	Yes
NCBI Assembly	GCA_001376135.1	NCBI: cellular organisms; Bacteria; Terrabacteria group; Actinobacteria; Actinomycetia; Corynebacteriales; Mycobacteriaceae; Mycobacterium; Mycobacterium tuberculosis complex; Mycobacterium tuberculosis;	NCBI: 1773	4364616	99.94	0.00	Yes
NCBI Assembly	GCA_001328255.1	NCBI: cellular organisms; Bacteria; Terrabacteria group; Actinobacteria; Actinomycetia; Corynebacteriales; Mycobacteriaceae; Mycobacterium; Mycobacterium tuberculosis complex; Mycobacterium tuberculosis;	NCBI: 1773	4348854	99.94	0.33	Yes
NCBI Assembly	GCA_001322665.1	NCBI: cellular organisms; Bacteria; Terrabacteria group; Actinobacteria; Actinomycetia; Corynebacteriales; Mycobacteriaceae; Mycobacterium; Mycobacterium tuberculosis complex; Mycobacterium tuberculosis;	NCBI: 1773	4341705	99.94	0.00	Yes
NCBI Assembly	GCA_001323605.1	NCBI: cellular organisms; Bacteria; Terrabacteria group; Actinobacteria; Actinomycetia; Corynebacteriales; Mycobacteriaceae; Mycobacterium; Mycobacterium tuberculosis complex; Mycobacterium tuberculosis;	NCBI: 1773	4364074	99.94	0.00	Yes
NCBI Assembly	GCA_001327935.1	NCBI: cellular organisms; Bacteria; Terrabacteria group; Actinobacteria; Actinomycetia; Corynebacteriales; Mycobacteriaceae; Mycobacterium; Mycobacterium tuberculosis complex; Mycobacterium tuberculosis;	NCBI: 1773	4326759	99.94	0.04	Yes
NCBI Assembly	GCA_000804205.1	NCBI: cellular organisms; Bacteria; Terrabacteria group; Actinobacteria; Actinomycetia; Corynebacteriales; Mycobacteriaceae; Mycobacterium; Mycobacterium tuberculosis complex; Mycobacterium tuberculosis;	NCBI: 1773	4366625	99.94	0.00	Yes
NCBI Assembly	GCA_001375755.1	NCBI: cellular organisms; Bacteria; Terrabacteria group; Actinobacteria; Actinomycetia; Corynebacteriales; Mycobacteriaceae; Mycobacterium; Mycobacterium tuberculosis complex; Mycobacterium tuberculosis;	NCBI: 1773	4394794	99.94	0.00	Yes
NCBI Assembly	GCA_001323345.1	NCBI: cellular organisms; Bacteria; Terrabacteria group; Actinobacteria; Actinomycetia; Corynebacteriales; Mycobacteriaceae; Mycobacterium; Mycobacterium tuberculosis complex; Mycobacterium tuberculosis;	NCBI: 1773	4357791	99.61	0.00	Yes
NCBI Assembly	GCA_000934585.1	NCBI: cellular organisms; Bacteria; Terrabacteria group; Actinobacteria; Actinomycetia; Corynebacteriales; Mycobacteriaceae; Mycobacterium; Mycobacterium tuberculosis complex; Mycobacterium tuberculosis;	NCBI: 1773	4392860	98.94	0.00	Yes
NCBI Assembly	GCA_001350495.1	NCBI: cellular organisms; Bacteria; Terrabacteria group; Actinobacteria; Actinomycetia; Corynebacteriales; Mycobacteriaceae; Mycobacterium; Mycobacterium tuberculosis complex; Mycobacterium tuberculosis;	NCBI: 1773	4380725	99.94	0.00	Yes
NCBI Assembly	GCA_001323025.1	NCBI: cellular organisms; Bacteria; Terrabacteria group; Actinobacteria; Actinomycetia; Corynebacteriales; Mycobacteriaceae; Mycobacterium; Mycobacterium tuberculosis complex; Mycobacterium tuberculosis;	NCBI: 1773	4365349	99.94	0.00	Yes
NCBI Assembly	GCA_001323945.1	NCBI: cellular organisms; Bacteria; Terrabacteria group; Actinobacteria; Actinomycetia; Corynebacteriales; Mycobacteriaceae; Mycobacterium; Mycobacterium tuberculosis complex; Mycobacterium tuberculosis;	NCBI: 1773	4370268	99.94	0.17	Yes
NCBI Assembly	GCA_001376155.1	NCBI: cellular organisms; Bacteria; Terrabacteria group; Actinobacteria; Actinomycetia; Corynebacteriales; Mycobacteriaceae; Mycobacterium; Mycobacterium tuberculosis complex; Mycobacterium tuberculosis;	NCBI: 1773	4377459	99.94	0.00	Yes
NCBI Assembly	GCA_001328275.1	NCBI: cellular organisms; Bacteria; Terrabacteria group; Actinobacteria; Actinomycetia; Corynebacteriales; Mycobacteriaceae; Mycobacterium; Mycobacterium tuberculosis complex; Mycobacterium tuberculosis;	NCBI: 1773	4323359	99.90	0.00	Yes
NCBI Assembly	GCA_001323625.1	NCBI: cellular organisms; Bacteria; Terrabacteria group; Actinobacteria; Actinomycetia; Corynebacteriales; Mycobacteriaceae; Mycobacterium; Mycobacterium tuberculosis complex; Mycobacterium tuberculosis;	NCBI: 1773	4367146	99.94	0.00	Yes

NCBI Assembly	GCA_001327955.1	NCBI: cellular organisms; Bacteria; Terrabacteria group; Actinobacteria; Actinomycetia; Corynebacteriales; Mycobacteriaceae; Mycobacterium; Mycobacterium tuberculosis complex; Mycobacterium tuberculosis;	NCBI: 1773	4301538	99.72	0.00	Yes
NCBI Assembly	GCA_001322685.1	NCBI: cellular organisms; Bacteria; Terrabacteria group; Actinobacteria; Actinomycetia; Corynebacteriales; Mycobacteriaceae; Mycobacterium; Mycobacterium tuberculosis complex; Mycobacterium tuberculosis;	NCBI: 1773	4357077	99.94	0.00	Yes
NCBI Assembly	GCA_000804225.1	NCBI: cellular organisms; Bacteria; Terrabacteria group; Actinobacteria; Actinomycetia; Corynebacteriales; Mycobacteriaceae; Mycobacterium; Mycobacterium tuberculosis complex; Mycobacterium tuberculosis;	NCBI: 1773	4362883	99.94	0.00	Yes
NCBI Assembly	GCA_000947875.1	NCBI: cellular organisms; Bacteria; Terrabacteria group; Actinobacteria; Actinomycetia; Corynebacteriales; Mycobacteriaceae; Mycobacterium; Mycobacterium tuberculosis complex; Mycobacterium tuberculosis; Mycobacterium tuberculosis variant bovis;	NCBI: 1765	4275214	99.56	0.00	Yes
NCBI Assembly	GCA_001375775.1	NCBI: cellular organisms; Bacteria; Terrabacteria group; Actinobacteria; Actinomycetia; Corynebacteriales; Mycobacteriaceae; Mycobacterium; Mycobacterium tuberculosis complex; Mycobacterium tuberculosis;	NCBI: 1773	4365600	99.94	0.00	Yes
NCBI Assembly	GCA_001323365.1	NCBI: cellular organisms; Bacteria; Terrabacteria group; Actinobacteria; Actinomycetia; Corynebacteriales; Mycobacteriaceae; Mycobacterium; Mycobacterium tuberculosis complex; Mycobacterium tuberculosis;	NCBI: 1773	4437892	99.94	0.28	Yes
NCBI Assembly	GCA_001350515.1	NCBI: cellular organisms; Bacteria; Terrabacteria group; Actinobacteria; Actinomycetia; Corynebacteriales; Mycobacteriaceae; Mycobacterium; Mycobacterium tuberculosis complex; Mycobacterium tuberculosis;	NCBI: 1773	4371126	99.94	0.00	Yes
NCBI Assembly	GCA_001323045.1	NCBI: cellular organisms; Bacteria; Terrabacteria group; Actinobacteria; Actinomycetia; Corynebacteriales; Mycobacteriaceae; Mycobacterium; Mycobacterium tuberculosis complex; Mycobacterium tuberculosis;	NCBI: 1773	4366282	99.94	0.00	Yes
NCBI Assembly	GCA_001323965.1	NCBI: cellular organisms; Bacteria; Terrabacteria group; Actinobacteria; Actinomycetia; Corynebacteriales; Mycobacteriaceae; Mycobacterium; Mycobacterium tuberculosis complex; Mycobacterium tuberculosis;	NCBI: 1773	4327835	99.94	0.00	Yes
NCBI Assembly	GCA_001376175.1	NCBI: cellular organisms; Bacteria; Terrabacteria group; Actinobacteria; Actinomycetia; Corynebacteriales; Mycobacteriaceae; Mycobacterium; Mycobacterium tuberculosis complex; Mycobacterium tuberculosis;	NCBI: 1773	4361963	99.94	0.00	Yes
NCBI Assembly	GCA_001323645.1	NCBI: cellular organisms; Bacteria; Terrabacteria group; Actinobacteria; Actinomycetia; Corynebacteriales; Mycobacteriaceae; Mycobacterium; Mycobacterium tuberculosis complex; Mycobacterium tuberculosis;	NCBI: 1773	4362460	99.94	0.00	Yes
NCBI Assembly	GCA_001327975.1	NCBI: cellular organisms; Bacteria; Terrabacteria group; Actinobacteria; Actinomycetia; Corynebacteriales; Mycobacteriaceae; Mycobacterium; Mycobacterium tuberculosis complex; Mycobacterium tuberculosis;	NCBI: 1773	4345420	99.61	0.00	Yes
NCBI Assembly	GCA_001328295.1	NCBI: cellular organisms; Bacteria; Terrabacteria group; Actinobacteria; Actinomycetia; Corynebacteriales; Mycobacteriaceae; Mycobacterium; Mycobacterium tuberculosis complex; Mycobacterium tuberculosis;	NCBI: 1773	4312521	99.61	0.00	Yes
NCBI Assembly	GCA_001322705.1	NCBI: cellular organisms; Bacteria; Terrabacteria group; Actinobacteria; Actinomycetia; Corynebacteriales; Mycobacteriaceae; Mycobacterium; Mycobacterium tuberculosis complex; Mycobacterium tuberculosis;	NCBI: 1773	4366696	99.94	0.00	Yes
NCBI Assembly	GCA_000804265.1	NCBI: cellular organisms; Bacteria; Terrabacteria group; Actinobacteria; Actinomycetia; Corynebacteriales; Mycobacteriaceae; Mycobacterium; Mycobacterium tuberculosis complex; Mycobacterium tuberculosis;	NCBI: 1773	4342679	99.94	0.00	Yes
NCBI Assembly	GCA_000947895.1	NCBI: cellular organisms; Bacteria; Terrabacteria group; Actinobacteria; Actinomycetia; Corynebacteriales; Mycobacteriaceae; Mycobacterium; Mycobacterium tuberculosis complex; Mycobacterium tuberculosis; Mycobacterium tuberculosis variant bovis;	NCBI: 1765	4259788	98.87	0.00	Yes
NCBI Assembly	GCA_001323385.1	NCBI: cellular organisms; Bacteria; Terrabacteria group; Actinobacteria; Actinomycetia; Corynebacteriales; Mycobacteriaceae; Mycobacterium; Mycobacterium tuberculosis complex; Mycobacterium tuberculosis;	NCBI: 1773	4369721	99.94	0.00	Yes
NCBI Assembly	GCA_001375795.1	NCBI: cellular organisms; Bacteria; Terrabacteria group; Actinobacteria; Actinomycetia; Corynebacteriales; Mycobacteriaceae; Mycobacterium; Mycobacterium tuberculosis complex; Mycobacterium tuberculosis;	NCBI: 1773	4368611	99.94	0.00	Yes

NCBI Assembly	GCA_001350535.1	NCBI: cellular organisms; Bacteria; Terrabacteria group; Actinobacteria; Actinomycetia; Corynebacteriales; Mycobacteriaceae; Mycobacterium; Mycobacterium tuberculosis complex; Mycobacterium tuberculosis;	NCBI: 1773	4362270	99.94	0.00	Yes
NCBI Assembly	GCA_001323065.1	NCBI: cellular organisms; Bacteria; Terrabacteria group; Actinobacteria; Actinomycetia; Corynebacteriales; Mycobacteriaceae; Mycobacterium; Mycobacterium tuberculosis complex; Mycobacterium tuberculosis;	NCBI: 1773	4390606	99.94	0.00	Yes
NCBI Assembly	GCA_001323985.1	NCBI: cellular organisms; Bacteria; Terrabacteria group; Actinobacteria; Actinomycetia; Corynebacteriales; Mycobacteriaceae; Mycobacterium; Mycobacterium tuberculosis complex; Mycobacterium tuberculosis;	NCBI: 1773	4357769	99.94	0.00	Yes
NCBI Assembly	GCA_001376195.1	NCBI: cellular organisms; Bacteria; Terrabacteria group; Actinobacteria; Actinomycetia; Corynebacteriales; Mycobacteriaceae; Mycobacterium; Mycobacterium tuberculosis complex; Mycobacterium tuberculosis;	NCBI: 1773	4365577	99.94	0.17	Yes
NCBI Assembly	GCA_001328315.1	NCBI: cellular organisms; Bacteria; Terrabacteria group; Actinobacteria; Actinomycetia; Corynebacteriales; Mycobacteriaceae; Mycobacterium; Mycobacterium tuberculosis complex; Mycobacterium tuberculosis;	NCBI: 1773	4283461	99.57	0.00	Yes
NCBI Assembly	GCA_001323665.1	NCBI: cellular organisms; Bacteria; Terrabacteria group; Actinobacteria; Actinomycetia; Corynebacteriales; Mycobacteriaceae; Mycobacterium; Mycobacterium tuberculosis complex; Mycobacterium tuberculosis;	NCBI: 1773	4368155	99.94	0.00	Yes
NCBI Assembly	GCA_001327995.1	NCBI: cellular organisms; Bacteria; Terrabacteria group; Actinobacteria; Actinomycetia; Corynebacteriales; Mycobacteriaceae; Mycobacterium; Mycobacterium tuberculosis complex; Mycobacterium tuberculosis;	NCBI: 1773	4314501	99.94	0.00	Yes
NCBI Assembly	GCA_000804285.1	NCBI: cellular organisms; Bacteria; Terrabacteria group; Actinobacteria; Actinomycetia; Corynebacteriales; Mycobacteriaceae; Mycobacterium; Mycobacterium tuberculosis complex; Mycobacterium tuberculosis;	NCBI: 1773	4377030	99.94	0.00	Yes
NCBI Assembly	GCA_001322725.1	NCBI: cellular organisms; Bacteria; Terrabacteria group; Actinobacteria; Actinomycetia; Corynebacteriales; Mycobacteriaceae; Mycobacterium; Mycobacterium tuberculosis complex; Mycobacterium tuberculosis;	NCBI: 1773	4367437	99.94	0.00	Yes
NCBI Assembly	GCA_001350615.1	NCBI: cellular organisms; Bacteria; Terrabacteria group; Actinobacteria; Actinomycetia; Corynebacteriales; Mycobacteriaceae; Mycobacterium; Mycobacterium tuberculosis complex; Mycobacterium tuberculosis;	NCBI: 1773	4380220	99.94	0.00	Yes
NCBI Assembly	GCA_001323405.1	NCBI: cellular organisms; Bacteria; Terrabacteria group; Actinobacteria; Actinomycetia; Corynebacteriales; Mycobacteriaceae; Mycobacterium; Mycobacterium tuberculosis complex; Mycobacterium tuberculosis;	NCBI: 1773	4361759	99.94	0.00	Yes
NCBI Assembly	GCA_001375815.1	NCBI: cellular organisms; Bacteria; Terrabacteria group; Actinobacteria; Actinomycetia; Corynebacteriales; Mycobacteriaceae; Mycobacterium; Mycobacterium tuberculosis complex; Mycobacterium tuberculosis;	NCBI: 1773	4355617	99.90	0.00	Yes
NCBI Assembly	GCA_001350555.1	NCBI: cellular organisms; Bacteria; Terrabacteria group; Actinobacteria; Actinomycetia; Corynebacteriales; Mycobacteriaceae; Mycobacterium; Mycobacterium tuberculosis complex; Mycobacterium tuberculosis;	NCBI: 1773	4375330	99.94	0.00	Yes
NCBI Assembly	GCA_001323085.1	NCBI: cellular organisms; Bacteria; Terrabacteria group; Actinobacteria; Actinomycetia; Corynebacteriales; Mycobacteriaceae; Mycobacterium; Mycobacterium tuberculosis complex; Mycobacterium tuberculosis;	NCBI: 1773	4362127	99.94	0.00	Yes
NCBI Assembly	GCA_001324005.1	NCBI: cellular organisms; Bacteria; Terrabacteria group; Actinobacteria; Actinomycetia; Corynebacteriales; Mycobacteriaceae; Mycobacterium; Mycobacterium tuberculosis complex; Mycobacterium tuberculosis;	NCBI: 1773	4407589	99.94	0.33	Yes
NCBI Assembly	GCA_000805375.1	NCBI: cellular organisms; Bacteria; Terrabacteria group; Actinobacteria; Actinomycetia; Corynebacteriales; Mycobacteriaceae; Mycolicibacterium; Mycolicibacterium setense;	NCBI: 431269	6065527	99.81	0.57	Yes
NCBI Assembly	GCA_001376215.1	NCBI: cellular organisms; Bacteria; Terrabacteria group; Actinobacteria; Actinomycetia; Corynebacteriales; Mycobacteriaceae; Mycobacterium; Mycobacterium tuberculosis complex; Mycobacterium tuberculosis;	NCBI: 1773	4362825	99.94	0.00	Yes
NCBI Assembly	GCA_001328015.1	NCBI: cellular organisms; Bacteria; Terrabacteria group; Actinobacteria; Actinomycetia; Corynebacteriales; Mycobacteriaceae; Mycobacterium; Mycobacterium tuberculosis complex; Mycobacterium tuberculosis;	NCBI: 1773	4308846	99.83	0.00	Yes

NCBI Assembly	GCA_001323685.1	NCBI: cellular organisms; Bacteria; Terrabacteria group; Actinobacteria; Actinomycetia; Corynebacteriales; Mycobacteriaceae; Mycobacterium; Mycobacterium tuberculosis complex; Mycobacterium tuberculosis;	NCBI: 1773	4360971	99.94	0.00	Yes
NCBI Assembly	GCA_001328335.1	NCBI: cellular organisms; Bacteria; Terrabacteria group; Actinobacteria; Actinomycetia; Corynebacteriales; Mycobacteriaceae; Mycobacterium; Mycobacterium tuberculosis complex; Mycobacterium tuberculosis;	NCBI: 1773	4293894	99.78	0.00	Yes
NCBI Assembly	GCA_001322745.1	NCBI: cellular organisms; Bacteria; Terrabacteria group; Actinobacteria; Actinomycetia; Corynebacteriales; Mycobacteriaceae; Mycobacterium; Mycobacterium tuberculosis complex; Mycobacterium tuberculosis;	NCBI: 1773	4367952	99.94	0.00	Yes
NCBI Assembly	GCA_001350635.1	NCBI: cellular organisms; Bacteria; Terrabacteria group; Actinobacteria; Actinomycetia; Corynebacteriales; Mycobacteriaceae; Mycobacterium; Mycobacterium tuberculosis complex; Mycobacterium tuberculosis;	NCBI: 1773	4360891	99.94	0.17	Yes
NCBI Assembly	GCA_001375835.1	NCBI: cellular organisms; Bacteria; Terrabacteria group; Actinobacteria; Actinomycetia; Corynebacteriales; Mycobacteriaceae; Mycobacterium; Mycobacterium tuberculosis complex; Mycobacterium tuberculosis;	NCBI: 1773	4394931	99.94	0.00	Yes
NCBI Assembly	GCA_001323425.1	NCBI: cellular organisms; Bacteria; Terrabacteria group; Actinobacteria; Actinomycetia; Corynebacteriales; Mycobacteriaceae; Mycobacterium; Mycobacterium tuberculosis complex; Mycobacterium tuberculosis;	NCBI: 1773	4365343	99.94	0.00	Yes
NCBI Assembly	GCA_001324725.1	NCBI: cellular organisms; Bacteria; Terrabacteria group; Actinobacteria; Actinomycetia; Corynebacteriales; Mycobacteriaceae; Mycobacterium; Mycobacterium tuberculosis complex; Mycobacterium tuberculosis;	NCBI: 1773	4358634	99.94	0.00	Yes
NCBI Assembly	GCA_001350575.1	NCBI: cellular organisms; Bacteria; Terrabacteria group; Actinobacteria; Actinomycetia; Corynebacteriales; Mycobacteriaceae; Mycobacterium; Mycobacterium tuberculosis complex; Mycobacterium tuberculosis;	NCBI: 1773	4357008	99.94	0.00	Yes
NCBI Assembly	GCA_001323105.1	NCBI: cellular organisms; Bacteria; Terrabacteria group; Actinobacteria; Actinomycetia; Corynebacteriales; Mycobacteriaceae; Mycobacterium; Mycobacterium tuberculosis complex; Mycobacterium tuberculosis;	NCBI: 1773	4367250	99.94	0.00	Yes
NCBI Assembly	GCA_001324025.1	NCBI: cellular organisms; Bacteria; Terrabacteria group; Actinobacteria; Actinomycetia; Corynebacteriales; Mycobacteriaceae; Mycobacterium; Mycobacterium tuberculosis complex; Mycobacterium tuberculosis;	NCBI: 1773	4369943	99.94	0.17	Yes
NCBI Assembly	GCA_001328035.1	NCBI: cellular organisms; Bacteria; Terrabacteria group; Actinobacteria; Actinomycetia; Corynebacteriales; Mycobacteriaceae; Mycobacterium; Mycobacterium tuberculosis complex; Mycobacterium tuberculosis;	NCBI: 1773	4331152	99.94	0.38	Yes
NCBI Assembly	GCA_001323705.1	NCBI: cellular organisms; Bacteria; Terrabacteria group; Actinobacteria; Actinomycetia; Corynebacteriales; Mycobacteriaceae; Mycobacterium; Mycobacterium tuberculosis complex; Mycobacterium tuberculosis;	NCBI: 1773	4367007	99.94	0.00	Yes
NCBI Assembly	GCA_001376235.1	NCBI: cellular organisms; Bacteria; Terrabacteria group; Actinobacteria; Actinomycetia; Corynebacteriales; Mycobacteriaceae; Mycobacterium; Mycobacterium tuberculosis complex; Mycobacterium tuberculosis;	NCBI: 1773	4351582	99.94	0.00	Yes
NCBI Assembly	GCA_001328355.1	NCBI: cellular organisms; Bacteria; Terrabacteria group; Actinobacteria; Actinomycetia; Corynebacteriales; Mycobacteriaceae; Mycobacterium; Mycobacterium tuberculosis complex; Mycobacterium tuberculosis;	NCBI: 1773	4312947	99.71	0.17	Yes
NCBI Assembly	GCA_001322765.1	NCBI: cellular organisms; Bacteria; Terrabacteria group; Actinobacteria; Actinomycetia; Corynebacteriales; Mycobacteriaceae; Mycobacterium; Mycobacterium tuberculosis complex; Mycobacterium tuberculosis;	NCBI: 1773	4366745	99.94	0.17	Yes
NCBI Assembly	GCA_001350655.1	NCBI: cellular organisms; Bacteria; Terrabacteria group; Actinobacteria; Actinomycetia; Corynebacteriales; Mycobacteriaceae; Mycobacterium; Mycobacterium tuberculosis complex; Mycobacterium tuberculosis;	NCBI: 1773	4359086	99.94	0.00	Yes
NCBI Assembly	GCA_001375855.1	NCBI: cellular organisms; Bacteria; Terrabacteria group; Actinobacteria; Actinomycetia; Corynebacteriales; Mycobacteriaceae; Mycobacterium; Mycobacterium tuberculosis complex; Mycobacterium tuberculosis;	NCBI: 1773	4362670	99.94	0.00	Yes
NCBI Assembly	GCA_001323445.1	NCBI: cellular organisms; Bacteria; Terrabacteria group; Actinobacteria; Actinomycetia; Corynebacteriales; Mycobacteriaceae; Mycobacterium; Mycobacterium tuberculosis complex; Mycobacterium tuberculosis;	NCBI: 1773	4365307	99.94	0.17	Yes

NCBI Assembly	GCA_001324045.1	NCBI: cellular organisms; Bacteria; Terrabacteria group; Actinobacteria; Actinomycetia; Corynebacteriales; Mycobacteriaceae; Mycobacterium; Mycobacterium tuberculosis complex; Mycobacterium tuberculosis;	NCBI: 1773	4382804	99.94	0.00	Yes
NCBI Assembly	GCA_001350595.1	NCBI: cellular organisms; Bacteria; Terrabacteria group; Actinobacteria; Actinomycetia; Corynebacteriales; Mycobacteriaceae; Mycobacterium; Mycobacterium tuberculosis complex; Mycobacterium tuberculosis;	NCBI: 1773	4362366	99.94	0.00	Yes
NCBI Assembly	GCA_001324745.1	NCBI: cellular organisms; Bacteria; Terrabacteria group; Actinobacteria; Actinomycetia; Corynebacteriales; Mycobacteriaceae; Mycobacterium; Mycobacterium tuberculosis complex; Mycobacterium tuberculosis;	NCBI: 1773	4357175	99.94	0.00	Yes
NCBI Assembly	GCA_001323125.1	NCBI: cellular organisms; Bacteria; Terrabacteria group; Actinobacteria; Actinomycetia; Corynebacteriales; Mycobacteriaceae; Mycobacterium; Mycobacterium tuberculosis complex; Mycobacterium tuberculosis;	NCBI: 1773	4363648	99.94	0.00	Yes
NCBI Assembly	GCA_001376255.1	NCBI: cellular organisms; Bacteria; Terrabacteria group; Actinobacteria; Actinomycetia; Corynebacteriales; Mycobacteriaceae; Mycobacterium; Mycobacterium tuberculosis complex; Mycobacterium tuberculosis;	NCBI: 1773	4369535	99.94	0.00	Yes
NCBI Assembly	GCA_001323725.1	NCBI: cellular organisms; Bacteria; Terrabacteria group; Actinobacteria; Actinomycetia; Corynebacteriales; Mycobacteriaceae; Mycobacterium; Mycobacterium tuberculosis complex; Mycobacterium tuberculosis;	NCBI: 1773	4366357	99.94	0.00	Yes
NCBI Assembly	GCA_001324405.1	NCBI: cellular organisms; Bacteria; Terrabacteria group; Actinobacteria; Actinomycetia; Corynebacteriales; Mycobacteriaceae; Mycobacterium; Mycobacterium tuberculosis complex; Mycobacterium tuberculosis;	NCBI: 1773	4364581	99.94	0.00	Yes
NCBI Assembly	GCA_001322785.1	NCBI: cellular organisms; Bacteria; Terrabacteria group; Actinobacteria; Actinomycetia; Corynebacteriales; Mycobacteriaceae; Mycobacterium; Mycobacterium tuberculosis complex; Mycobacterium tuberculosis;	NCBI: 1773	4369842	99.94	0.00	Yes
NCBI Assembly	GCA_001350675.1	NCBI: cellular organisms; Bacteria; Terrabacteria group; Actinobacteria; Actinomycetia; Corynebacteriales; Mycobacteriaceae; Mycobacterium; Mycobacterium tuberculosis complex; Mycobacterium tuberculosis;	NCBI: 1773	4359226	99.94	0.00	Yes
NCBI Assembly	GCA_001375875.1	NCBI: cellular organisms; Bacteria; Terrabacteria group; Actinobacteria; Actinomycetia; Corynebacteriales; Mycobacteriaceae; Mycobacterium; Mycobacterium tuberculosis complex; Mycobacterium tuberculosis;	NCBI: 1773	4356042	99.94	0.00	Yes
NCBI Assembly	GCA_001324065.1	NCBI: cellular organisms; Bacteria; Terrabacteria group; Actinobacteria; Actinomycetia; Corynebacteriales; Mycobacteriaceae; Mycobacterium; Mycobacterium tuberculosis complex; Mycobacterium tuberculosis;	NCBI: 1773	4362900	99.94	0.00	Yes
NCBI Assembly	GCA_001323145.1	NCBI: cellular organisms; Bacteria; Terrabacteria group; Actinobacteria; Actinomycetia; Corynebacteriales; Mycobacteriaceae; Mycobacterium; Mycobacterium tuberculosis complex; Mycobacterium tuberculosis;	NCBI: 1773	4364689	99.94	0.00	Yes
NCBI Assembly	GCA_001324765.1	NCBI: cellular organisms; Bacteria; Terrabacteria group; Actinobacteria; Actinomycetia; Corynebacteriales; Mycobacteriaceae; Mycobacterium; Mycobacterium tuberculosis complex; Mycobacterium tuberculosis;	NCBI: 1773	4353689	99.94	0.00	Yes
NCBI Assembly	GCA_001324425.1	NCBI: cellular organisms; Bacteria; Terrabacteria group; Actinobacteria; Actinomycetia; Corynebacteriales; Mycobacteriaceae; Mycobacterium; Mycobacterium tuberculosis complex; Mycobacterium tuberculosis;	NCBI: 1773	4356315	99.94	0.00	Yes
NCBI Assembly	GCA_001376275.1	NCBI: cellular organisms; Bacteria; Terrabacteria group; Actinobacteria; Actinomycetia; Corynebacteriales; Mycobacteriaceae; Mycobacterium; Mycobacterium tuberculosis complex; Mycobacterium tuberculosis;	NCBI: 1773	4362863	99.94	0.00	Yes
NCBI Assembly	GCA_001323745.1	NCBI: cellular organisms; Bacteria; Terrabacteria group; Actinobacteria; Actinomycetia; Corynebacteriales; Mycobacteriaceae; Mycobacterium; Mycobacterium tuberculosis complex; Mycobacterium tuberculosis;	NCBI: 1773	4369215	99.94	0.00	Yes
NCBI Assembly	GCA_001322805.1	NCBI: cellular organisms; Bacteria; Terrabacteria group; Actinobacteria; Actinomycetia; Corynebacteriales; Mycobacteriaceae; Mycobacterium; Mycobacterium tuberculosis complex; Mycobacterium tuberculosis;	NCBI: 1773	4367786	99.94	0.00	Yes
NCBI Assembly	GCA_001350695.1	NCBI: cellular organisms; Bacteria; Terrabacteria group; Actinobacteria; Actinomycetia; Corynebacteriales; Mycobacteriaceae; Mycobacterium; Mycobacterium tuberculosis complex; Mycobacterium tuberculosis;	NCBI: 1773	4386925	99.94	0.00	Yes

NCBI Assembly	GCA_001375895.1	NCBI: cellular organisms; Bacteria; Terrabacteria group; Actinobacteria; Actinomycetia; Corynebacteriales; Mycobacteriaceae; Mycobacterium; Mycobacterium tuberculosis complex; Mycobacterium tuberculosis;	NCBI: 1773	4370919	99.94	0.00	Yes
NCBI Assembly	GCA_001324785.1	NCBI: cellular organisms; Bacteria; Terrabacteria group; Actinobacteria; Actinomycetia; Corynebacteriales; Mycobacteriaceae; Mycobacterium; Mycobacterium tuberculosis complex; Mycobacterium tuberculosis;	NCBI: 1773	4359554	99.94	0.00	Yes
NCBI Assembly	GCA_001323765.1	NCBI: cellular organisms; Bacteria; Terrabacteria group; Actinobacteria; Actinomycetia; Corynebacteriales; Mycobacteriaceae; Mycobacterium; Mycobacterium tuberculosis complex; Mycobacterium tuberculosis;	NCBI: 1773	4368182	99.94	0.17	Yes
NCBI Assembly	GCA_001324445.1	NCBI: cellular organisms; Bacteria; Terrabacteria group; Actinobacteria; Actinomycetia; Corynebacteriales; Mycobacteriaceae; Mycobacterium; Mycobacterium tuberculosis complex; Mycobacterium tuberculosis;	NCBI: 1773	4376906	99.94	0.00	Yes
NCBI Assembly	GCA_001376295.1	NCBI: cellular organisms; Bacteria; Terrabacteria group; Actinobacteria; Actinomycetia; Corynebacteriales; Mycobacteriaceae; Mycobacterium; Mycobacterium tuberculosis complex; Mycobacterium tuberculosis;	NCBI: 1773	4364146	99.94	0.00	Yes
NCBI Assembly	GCA_001322825.1	NCBI: cellular organisms; Bacteria; Terrabacteria group; Actinobacteria; Actinomycetia; Corynebacteriales; Mycobacteriaceae; Mycobacterium; Mycobacterium tuberculosis complex; Mycobacterium tuberculosis;	NCBI: 1773	4361575	99.94	0.03	Yes
NCBI Assembly	GCA_001350715.1	NCBI: cellular organisms; Bacteria; Terrabacteria group; Actinobacteria; Actinomycetia; Corynebacteriales; Mycobacteriaceae; Mycobacterium; Mycobacterium tuberculosis complex; Mycobacterium tuberculosis;	NCBI: 1773	4363545	99.94	0.00	Yes
NCBI Assembly	GCA_001375915.1	NCBI: cellular organisms; Bacteria; Terrabacteria group; Actinobacteria; Actinomycetia; Corynebacteriales; Mycobacteriaceae; Mycobacterium; Mycobacterium tuberculosis complex; Mycobacterium tuberculosis;	NCBI: 1773	4367595	99.94	0.00	Yes
NCBI Assembly	GCA_001324805.1	NCBI: cellular organisms; Bacteria; Terrabacteria group; Actinobacteria; Actinomycetia; Corynebacteriales; Mycobacteriaceae; Mycobacterium; Mycobacterium tuberculosis complex; Mycobacterium tuberculosis;	NCBI: 1773	4356683	99.94	0.00	Yes
NCBI Assembly	GCA_001324465.1	NCBI: cellular organisms; Bacteria; Terrabacteria group; Actinobacteria; Actinomycetia; Corynebacteriales; Mycobacteriaceae; Mycobacterium; Mycobacterium tuberculosis complex; Mycobacterium tuberculosis;	NCBI: 1773	4365869	99.94	0.00	Yes
NCBI Assembly	GCA_001376315.1	NCBI: cellular organisms; Bacteria; Terrabacteria group; Actinobacteria; Actinomycetia; Corynebacteriales; Mycobacteriaceae; Mycobacterium; Mycobacterium tuberculosis complex; Mycobacterium tuberculosis;	NCBI: 1773	4365554	99.94	0.00	Yes
NCBI Assembly	GCA_001350735.1	NCBI: cellular organisms; Bacteria; Terrabacteria group; Actinobacteria; Actinomycetia; Corynebacteriales; Mycobacteriaceae; Mycobacterium; Mycobacterium tuberculosis complex; Mycobacterium tuberculosis;	NCBI: 1773	4365720	99.94	0.00	Yes
NCBI Assembly	GCA_001324825.1	NCBI: cellular organisms; Bacteria; Terrabacteria group; Actinobacteria; Actinomycetia; Corynebacteriales; Mycobacteriaceae; Mycobacterium; Mycobacterium tuberculosis complex; Mycobacterium tuberculosis;	NCBI: 1773	4354546	99.94	0.00	Yes
NCBI Assembly	GCA_001324485.1	NCBI: cellular organisms; Bacteria; Terrabacteria group; Actinobacteria; Actinomycetia; Corynebacteriales; Mycobacteriaceae; Mycobacterium; Mycobacterium tuberculosis complex; Mycobacterium tuberculosis;	NCBI: 1773	4364688	99.94	0.00	Yes
NCBI Assembly	GCA_001376335.1	NCBI: cellular organisms; Bacteria; Terrabacteria group; Actinobacteria; Actinomycetia; Corynebacteriales; Mycobacteriaceae; Mycobacterium; Mycobacterium tuberculosis complex; Mycobacterium tuberculosis;	NCBI: 1773	4358239	99.94	0.00	Yes
NCBI Assembly	GCA_001350755.1	NCBI: cellular organisms; Bacteria; Terrabacteria group; Actinobacteria; Actinomycetia; Corynebacteriales; Mycobacteriaceae; Mycobacterium; Mycobacterium tuberculosis complex; Mycobacterium tuberculosis;	NCBI: 1773	4354751	99.94	0.00	Yes
NCBI Assembly	GCA_001324845.1	NCBI: cellular organisms; Bacteria; Terrabacteria group; Actinobacteria; Actinomycetia; Corynebacteriales; Mycobacteriaceae; Mycobacterium; Mycobacterium tuberculosis complex; Mycobacterium tuberculosis;	NCBI: 1773	4358133	99.94	0.00	Yes
NCBI Assembly	GCA_001324505.1	NCBI: cellular organisms; Bacteria; Terrabacteria group; Actinobacteria; Actinomycetia; Corynebacteriales; Mycobacteriaceae; Mycobacterium; Mycobacterium tuberculosis complex; Mycobacterium tuberculosis;	NCBI: 1773	4364686	99.94	0.00	Yes

NCBI Assembly	GCA_001376355.1	NCBI: cellular organisms; Bacteria; Terrabacteria group; Actinobacteria; Actinomycetia; Corynebacteriales; Mycobacteriaceae; Mycobacterium; Mycobacterium tuberculosis complex; Mycobacterium tuberculosis;	NCBI: 1773	4363802	99.94	0.00	Yes
NCBI Assembly	GCA_001350775.1	NCBI: cellular organisms; Bacteria; Terrabacteria group; Actinobacteria; Actinomycetia; Corynebacteriales; Mycobacteriaceae; Mycobacterium; Mycobacterium tuberculosis complex; Mycobacterium tuberculosis;	NCBI: 1773	4385193	99.94	0.00	Yes
NCBI Assembly	GCA_001324865.1	NCBI: cellular organisms; Bacteria; Terrabacteria group; Actinobacteria; Actinomycetia; Corynebacteriales; Mycobacteriaceae; Mycobacterium; Mycobacterium tuberculosis complex; Mycobacterium tuberculosis;	NCBI: 1773	4362281	99.94	0.00	Yes
NCBI Assembly	GCA_001324525.1	NCBI: cellular organisms; Bacteria; Terrabacteria group; Actinobacteria; Actinomycetia; Corynebacteriales; Mycobacteriaceae; Mycobacterium; Mycobacterium tuberculosis complex; Mycobacterium tuberculosis;	NCBI: 1773	4360319	99.94	0.00	Yes
NCBI Assembly	GCA_001376375.1	NCBI: cellular organisms; Bacteria; Terrabacteria group; Actinobacteria; Actinomycetia; Corynebacteriales; Mycobacteriaceae; Mycobacterium; Mycobacterium tuberculosis complex; Mycobacterium tuberculosis;	NCBI: 1773	4397080	99.94	0.00	Yes
NCBI Assembly	GCA_001350795.1	NCBI: cellular organisms; Bacteria; Terrabacteria group; Actinobacteria; Actinomycetia; Corynebacteriales; Mycobacteriaceae; Mycobacterium; Mycobacterium tuberculosis complex; Mycobacterium tuberculosis;	NCBI: 1773	9043378	100.00	150.99	No
NCBI Assembly	GCA_001324885.1	NCBI: cellular organisms; Bacteria; Terrabacteria group; Actinobacteria; Actinomycetia; Corynebacteriales; Mycobacteriaceae; Mycobacterium; Mycobacterium tuberculosis complex; Mycobacterium tuberculosis;	NCBI: 1773	4416826	99.94	0.33	Yes
NCBI Assembly	GCA_001324545.1	NCBI: cellular organisms; Bacteria; Terrabacteria group; Actinobacteria; Actinomycetia; Corynebacteriales; Mycobacteriaceae; Mycobacterium; Mycobacterium tuberculosis complex; Mycobacterium tuberculosis;	NCBI: 1773	4386788	99.94	0.00	Yes
NCBI Assembly	GCA_001376395.1	NCBI: cellular organisms; Bacteria; Terrabacteria group; Actinobacteria; Actinomycetia; Corynebacteriales; Mycobacteriaceae; Mycobacterium; Mycobacterium tuberculosis complex; Mycobacterium tuberculosis;	NCBI: 1773	4351182	99.94	0.00	Yes
NCBI Assembly	GCA_001350815.1	NCBI: cellular organisms; Bacteria; Terrabacteria group; Actinobacteria; Actinomycetia; Corynebacteriales; Mycobacteriaceae; Mycobacterium; Mycobacterium tuberculosis complex; Mycobacterium tuberculosis;	NCBI: 1773	4376094	99.94	0.00	Yes
NCBI Assembly	GCA_001324905.1	NCBI: cellular organisms; Bacteria; Terrabacteria group; Actinobacteria; Actinomycetia; Corynebacteriales; Mycobacteriaceae; Mycobacterium; Mycobacterium tuberculosis complex; Mycobacterium tuberculosis;	NCBI: 1773	4357477	99.94	0.00	Yes
NCBI Assembly	GCA_001324565.1	NCBI: cellular organisms; Bacteria; Terrabacteria group; Actinobacteria; Actinomycetia; Corynebacteriales; Mycobacteriaceae; Mycobacterium; Mycobacterium tuberculosis complex; Mycobacterium tuberculosis;	NCBI: 1773	4372264	99.94	0.00	Yes
NCBI Assembly	GCA_001376415.1	NCBI: cellular organisms; Bacteria; Terrabacteria group; Actinobacteria; Actinomycetia; Corynebacteriales; Mycobacteriaceae; Mycobacterium; Mycobacterium tuberculosis complex; Mycobacterium tuberculosis;	NCBI: 1773	4349815	99.94	0.00	Yes
NCBI Assembly	GCA_001350835.1	NCBI: cellular organisms; Bacteria; Terrabacteria group; Actinobacteria; Actinomycetia; Corynebacteriales; Mycobacteriaceae; Mycobacterium; Mycobacterium tuberculosis complex; Mycobacterium tuberculosis;	NCBI: 1773	4372079	99.94	0.00	Yes
NCBI Assembly	GCA_001324925.1	NCBI: cellular organisms; Bacteria; Terrabacteria group; Actinobacteria; Actinomycetia; Corynebacteriales; Mycobacteriaceae; Mycobacterium; Mycobacterium tuberculosis complex; Mycobacterium tuberculosis;	NCBI: 1773	4358339	99.94	0.00	Yes
NCBI Assembly	GCA_001324585.1	NCBI: cellular organisms; Bacteria; Terrabacteria group; Actinobacteria; Actinomycetia; Corynebacteriales; Mycobacteriaceae; Mycobacterium; Mycobacterium tuberculosis complex; Mycobacterium tuberculosis;	NCBI: 1773	4362098	99.94	0.00	Yes
NCBI Assembly	GCA_001376435.1	NCBI: cellular organisms; Bacteria; Terrabacteria group; Actinobacteria; Actinomycetia; Corynebacteriales; Mycobacteriaceae; Mycobacterium; Mycobacterium tuberculosis complex; Mycobacterium tuberculosis;	NCBI: 1773	4365140	99.94	0.00	Yes
NCBI Assembly	GCA_001350855.1	NCBI: cellular organisms; Bacteria; Terrabacteria group; Actinobacteria; Actinomycetia; Corynebacteriales; Mycobacteriaceae; Mycobacterium; Mycobacterium tuberculosis complex; Mycobacterium tuberculosis;	NCBI: 1773	4364890	99.94	0.00	Yes

NCBI Assembly	GCA_001324945.1	NCBI: cellular organisms; Bacteria; Terrabacteria group; Actinobacteria; Actinomycetia; Corynebacteriales; Mycobacteriaceae; Mycobacterium; Mycobacterium tuberculosis complex; Mycobacterium tuberculosis;	NCBI: 1773	4360093	99.57	0.00	Yes
NCBI Assembly	GCA_001324605.1	NCBI: cellular organisms; Bacteria; Terrabacteria group; Actinobacteria; Actinomycetia; Corynebacteriales; Mycobacteriaceae; Mycobacterium; Mycobacterium tuberculosis complex; Mycobacterium tuberculosis;	NCBI: 1773	4369396	99.94	0.00	Yes
NCBI Assembly	GCA_001376455.1	NCBI: cellular organisms; Bacteria; Terrabacteria group; Actinobacteria; Actinomycetia; Corynebacteriales; Mycobacteriaceae; Mycobacterium; Mycobacterium tuberculosis complex; Mycobacterium tuberculosis;	NCBI: 1773	4363936	99.94	0.00	Yes
NCBI Assembly	GCA_001350875.1	NCBI: cellular organisms; Bacteria; Terrabacteria group; Actinobacteria; Actinomycetia; Corynebacteriales; Mycobacteriaceae; Mycobacterium; Mycobacterium tuberculosis complex; Mycobacterium tuberculosis;	NCBI: 1773	4365449	99.94	0.00	Yes
NCBI Assembly	GCA_001324965.1	NCBI: cellular organisms; Bacteria; Terrabacteria group; Actinobacteria; Actinomycetia; Corynebacteriales; Mycobacteriaceae; Mycobacterium; Mycobacterium tuberculosis complex; Mycobacterium tuberculosis;	NCBI: 1773	4377586	99.94	0.00	Yes
NCBI Assembly	GCA_001324625.1	NCBI: cellular organisms; Bacteria; Terrabacteria group; Actinobacteria; Actinomycetia; Corynebacteriales; Mycobacteriaceae; Mycobacterium; Mycobacterium tuberculosis complex; Mycobacterium tuberculosis;	NCBI: 1773	4366329	99.94	0.00	Yes
NCBI Assembly	GCA_001376475.1	NCBI: cellular organisms; Bacteria; Terrabacteria group; Actinobacteria; Actinomycetia; Corynebacteriales; Mycobacteriaceae; Mycobacterium; Mycobacterium tuberculosis complex; Mycobacterium tuberculosis;	NCBI: 1773	4366614	99.94	0.17	Yes
NCBI Assembly	GCA_001369315.1	NCBI: cellular organisms; Bacteria; Terrabacteria group; Actinobacteria; Actinomycetia; Corynebacteriales; Mycobacteriaceae; Mycobacterium; Mycobacterium tuberculosis complex; Mycobacterium tuberculosis; Mycobacterium tuberculosis variant caprae;	NCBI: 115862	4288871	99.78	0.00	Yes
NCBI Assembly	GCA_001324985.1	NCBI: cellular organisms; Bacteria; Terrabacteria group; Actinobacteria; Actinomycetia; Corynebacteriales; Mycobacteriaceae; Mycobacterium; Mycobacterium tuberculosis complex; Mycobacterium tuberculosis;	NCBI: 1773	4358670	99.94	0.00	Yes
NCBI Assembly	GCA_001324645.1	NCBI: cellular organisms; Bacteria; Terrabacteria group; Actinobacteria; Actinomycetia; Corynebacteriales; Mycobacteriaceae; Mycobacterium; Mycobacterium tuberculosis complex; Mycobacterium tuberculosis;	NCBI: 1773	4394655	99.94	0.00	Yes
NCBI Assembly	GCA_001376495.1	NCBI: cellular organisms; Bacteria; Terrabacteria group; Actinobacteria; Actinomycetia; Corynebacteriales; Mycobacteriaceae; Mycobacterium; Mycobacterium tuberculosis complex; Mycobacterium tuberculosis;	NCBI: 1773	4353417	99.94	0.00	Yes
NCBI Assembly	GCA_001324665.1	NCBI: cellular organisms; Bacteria; Terrabacteria group; Actinobacteria; Actinomycetia; Corynebacteriales; Mycobacteriaceae; Mycobacterium; Mycobacterium tuberculosis complex; Mycobacterium tuberculosis;	NCBI: 1773	4386106	99.94	0.00	Yes
NCBI Assembly	GCA_001325005.1	NCBI: cellular organisms; Bacteria; Terrabacteria group; Actinobacteria; Actinomycetia; Corynebacteriales; Mycobacteriaceae; Mycobacterium; Mycobacterium tuberculosis complex; Mycobacterium tuberculosis;	NCBI: 1773	4358604	99.78	0.00	Yes
NCBI Assembly	GCA_001376515.1	NCBI: cellular organisms; Bacteria; Terrabacteria group; Actinobacteria; Actinomycetia; Corynebacteriales; Mycobacteriaceae; Mycobacterium; Mycobacterium tuberculosis complex; Mycobacterium tuberculosis;	NCBI: 1773	4357165	99.94	0.00	Yes
NCBI Assembly	GCA_001324685.1	NCBI: cellular organisms; Bacteria; Terrabacteria group; Actinobacteria; Actinomycetia; Corynebacteriales; Mycobacteriaceae; Mycobacterium; Mycobacterium tuberculosis complex; Mycobacterium tuberculosis;	NCBI: 1773	4365181	99.94	0.00	Yes
NCBI Assembly	GCA_001376535.1	NCBI: cellular organisms; Bacteria; Terrabacteria group; Actinobacteria; Actinomycetia; Corynebacteriales; Mycobacteriaceae; Mycobacterium; Mycobacterium tuberculosis complex; Mycobacterium tuberculosis;	NCBI: 1773	4365778	99.94	0.00	Yes
NCBI Assembly	GCA_001325025.1	NCBI: cellular organisms; Bacteria; Terrabacteria group; Actinobacteria; Actinomycetia; Corynebacteriales; Mycobacteriaceae; Mycobacterium; Mycobacterium tuberculosis complex; Mycobacterium tuberculosis;	NCBI: 1773	4353704	99.94	0.00	Yes
NCBI Assembly	GCA_001324705.1	NCBI: cellular organisms; Bacteria; Terrabacteria group; Actinobacteria; Actinomycetia; Corynebacteriales; Mycobacteriaceae; Mycobacterium; Mycobacterium tuberculosis complex; Mycobacterium tuberculosis;	NCBI: 1773	4355220	99.94	0.00	Yes

NCBI Assembly	GCA_001386855.1	NCBI: cellular organisms; Bacteria; Terrabacteria group; Actinobacteria; Actinomycetia; Corynebacteriales; Mycobacteriaceae; Mycobacterium; Mycobacterium tuberculosis complex; Mycobacterium tuberculosis;	NCBI: 1773	4355509	99.78	0.00	Yes
NCBI Assembly	GCA_001393115.1	NCBI: cellular organisms; Bacteria; Terrabacteria group; Actinobacteria; Actinomycetia; Corynebacteriales; Mycobacteriaceae; Mycobacterium; Mycobacterium tuberculosis complex; Mycobacterium tuberculosis;	NCBI: 1773	4341842	99.94	0.00	Yes
NCBI Assembly	GCA_001391855.1	NCBI: cellular organisms; Bacteria; Terrabacteria group; Actinobacteria; Actinomycetia; Corynebacteriales; Mycobacteriaceae; Mycobacterium; Mycobacterium tuberculosis complex; Mycobacterium tuberculosis;	NCBI: 1773	4327372	99.94	0.00	Yes
NCBI Assembly	GCA_001393435.1	NCBI: cellular organisms; Bacteria; Terrabacteria group; Actinobacteria; Actinomycetia; Corynebacteriales; Mycobacteriaceae; Mycobacterium; Mycobacterium tuberculosis complex; Mycobacterium tuberculosis;	NCBI: 1773	4306174	99.94	0.00	Yes
NCBI Assembly	GCA_001394675.1	NCBI: cellular organisms; Bacteria; Terrabacteria group; Actinobacteria; Actinomycetia; Corynebacteriales; Mycobacteriaceae; Mycobacterium; Mycobacterium tuberculosis complex; Mycobacterium tuberculosis;	NCBI: 1773	4293198	99.54	0.00	Yes
NCBI Assembly	GCA_001394055.1	NCBI: cellular organisms; Bacteria; Terrabacteria group; Actinobacteria; Actinomycetia; Corynebacteriales; Mycobacteriaceae; Mycobacterium; Mycobacterium tuberculosis complex; Mycobacterium tuberculosis;	NCBI: 1773	4303022	99.94	0.00	Yes
NCBI Assembly	GCA_001396855.1	NCBI: cellular organisms; Bacteria; Terrabacteria group; Actinobacteria; Actinomycetia; Corynebacteriales; Mycobacteriaceae; Mycobacterium; Mycobacterium tuberculosis complex; Mycobacterium tuberculosis;	NCBI: 1773	4290555	99.39	0.04	Yes
NCBI Assembly	GCA_001389355.1	NCBI: cellular organisms; Bacteria; Terrabacteria group; Actinobacteria; Actinomycetia; Corynebacteriales; Mycobacteriaceae; Mycobacterium; Mycobacterium tuberculosis complex; Mycobacterium tuberculosis;	NCBI: 1773	4355182	99.61	0.00	Yes
NCBI Assembly	GCA_001544735.1	NCBI: cellular organisms; Bacteria; Terrabacteria group; Actinobacteria; Actinomycetia; Corynebacteriales; Mycobacteriaceae; Mycobacterium; Mycobacterium tuberculosis complex; Mycobacterium tuberculosis; Mycobacterium tuberculosis variant bovis; Mycobacterium tuberculosis variant bovis BCG;	NCBI: 33892	4351313	95.37	1.00	Yes
NCBI Assembly	GCA_001394355.1	NCBI: cellular organisms; Bacteria; Terrabacteria group; Actinobacteria; Actinomycetia; Corynebacteriales; Mycobacteriaceae; Mycobacterium; Mycobacterium tuberculosis complex; Mycobacterium tuberculosis;	NCBI: 1773	5107721	97.59	15.27	No
NCBI Assembly	GCA_001383435.1	NCBI: cellular organisms; Bacteria; Terrabacteria group; Actinobacteria; Actinomycetia; Corynebacteriales; Mycobacteriaceae; Mycobacterium; Mycobacterium tuberculosis complex; Mycobacterium tuberculosis;	NCBI: 1773	4359611	99.94	0.00	Yes
NCBI Assembly	GCA_001440205.1	NCBI: cellular organisms; Bacteria; Terrabacteria group; Actinobacteria; Actinomycetia; Corynebacteriales; Mycobacteriaceae; Mycobacteroides; unclassified Mycobacteroides;	NCBI: 1720572	5726565	99.44	0.39	Yes
NCBI Assembly	GCA_001386875.1	NCBI: cellular organisms; Bacteria; Terrabacteria group; Actinobacteria; Actinomycetia; Corynebacteriales; Mycobacteriaceae; Mycobacterium; Mycobacterium tuberculosis complex; Mycobacterium tuberculosis;	NCBI: 1773	4375431	99.11	0.00	Yes
NCBI Assembly	GCA_001393135.1	NCBI: cellular organisms; Bacteria; Terrabacteria group; Actinobacteria; Actinomycetia; Corynebacteriales; Mycobacteriaceae; Mycobacterium; Mycobacterium tuberculosis complex; Mycobacterium tuberculosis;	NCBI: 1773	4311274	99.44	0.15	Yes
NCBI Assembly	GCA_001393455.1	NCBI: cellular organisms; Bacteria; Terrabacteria group; Actinobacteria; Actinomycetia; Corynebacteriales; Mycobacteriaceae; Mycobacterium; Mycobacterium tuberculosis complex; Mycobacterium tuberculosis;	NCBI: 1773	4303530	99.89	0.00	Yes
NCBI Assembly	GCA_001391875.1	NCBI: cellular organisms; Bacteria; Terrabacteria group; Actinobacteria; Actinomycetia; Corynebacteriales; Mycobacteriaceae; Mycobacterium; Mycobacterium tuberculosis complex; Mycobacterium tuberculosis;	NCBI: 1773	4286794	99.94	0.00	Yes
NCBI Assembly	GCA_001394375.1	NCBI: cellular organisms; Bacteria; Terrabacteria group; Actinobacteria; Actinomycetia; Corynebacteriales; Mycobacteriaceae; Mycobacterium; Mycobacterium tuberculosis complex; Mycobacterium tuberculosis;	NCBI: 1773	4336152	99.77	0.00	Yes
NCBI Assembly	GCA_001544775.1	NCBI: cellular organisms; Bacteria; Terrabacteria group; Actinobacteria; Actinomycetia; Corynebacteriales; Mycobacteriaceae; Mycobacterium; Mycobacterium tuberculosis complex; Mycobacterium tuberculosis; Mycobacterium tuberculosis variant bovis;	NCBI: 1765	4336227	99.56	0.00	Yes

NCBI Assembly	GCA_001394695.1	NCBI: cellular organisms; Bacteria; Terrabacteria group; Actinobacteria; Actinomycetia; Corynebacteriales; Mycobacteriaceae; Mycobacterium; Mycobacterium tuberculosis complex; Mycobacterium tuberculosis;	NCBI: 1773	4305557	99.83	0.00	Yes
NCBI Assembly	GCA_001396875.1	NCBI: cellular organisms; Bacteria; Terrabacteria group; Actinobacteria; Actinomycetia; Corynebacteriales; Mycobacteriaceae; Mycobacterium; Mycobacterium tuberculosis complex; Mycobacterium tuberculosis;	NCBI: 1773	4275565	99.55	1.03	Yes
NCBI Assembly	GCA_001383455.1	NCBI: cellular organisms; Bacteria; Terrabacteria group; Actinobacteria; Actinomycetia; Corynebacteriales; Mycobacteriaceae; Mycobacterium; Mycobacterium tuberculosis complex; Mycobacterium tuberculosis;	NCBI: 1773	4371707	99.94	0.00	Yes
NCBI Assembly	GCA_001389375.1	NCBI: cellular organisms; Bacteria; Terrabacteria group; Actinobacteria; Actinomycetia; Corynebacteriales; Mycobacteriaceae; Mycobacterium; Mycobacterium tuberculosis complex; Mycobacterium tuberculosis;	NCBI: 1773	4369654	99.94	0.00	Yes
NCBI Assembly	GCA_001394075.1	NCBI: cellular organisms; Bacteria; Terrabacteria group; Actinobacteria; Actinomycetia; Corynebacteriales; Mycobacteriaceae; Mycobacterium; Mycobacterium tuberculosis complex; Mycobacterium tuberculosis;	NCBI: 1773	4314526	99.94	0.00	Yes
NCBI Assembly	GCA_001440225.1	NCBI: cellular organisms; Bacteria; Terrabacteria group; Actinobacteria; Actinomycetia; Corynebacteriales; Mycobacteriaceae; Mycobacteroides; unclassified Mycobacteroides;	NCBI: 1720573	5715094	99.28	0.39	Yes
NCBI Assembly	GCA_001386895.1	NCBI: cellular organisms; Bacteria; Terrabacteria group; Actinobacteria; Actinomycetia; Corynebacteriales; Mycobacteriaceae; Mycobacterium; Mycobacterium tuberculosis complex; Mycobacterium tuberculosis;	NCBI: 1773	4359293	99.88	0.00	Yes
NCBI Assembly	GCA_001393475.1	NCBI: cellular organisms; Bacteria; Terrabacteria group; Actinobacteria; Actinomycetia; Corynebacteriales; Mycobacteriaceae; Mycobacterium; Mycobacterium tuberculosis complex; Mycobacterium tuberculosis;	NCBI: 1773	4296044	99.83	0.00	Yes
NCBI Assembly	GCA_001391895.1	NCBI: cellular organisms; Bacteria; Terrabacteria group; Actinobacteria; Actinomycetia; Corynebacteriales; Mycobacteriaceae; Mycobacterium; Mycobacterium tuberculosis complex; Mycobacterium tuberculosis;	NCBI: 1773	4355366	99.94	0.33	Yes
NCBI Assembly	GCA_001393155.1	NCBI: cellular organisms; Bacteria; Terrabacteria group; Actinobacteria; Actinomycetia; Corynebacteriales; Mycobacteriaceae; Mycobacterium; Mycobacterium tuberculosis complex; Mycobacterium tuberculosis;	NCBI: 1773	4331635	99.94	0.33	Yes
NCBI Assembly	GCA_001383475.1	NCBI: cellular organisms; Bacteria; Terrabacteria group; Actinobacteria; Actinomycetia; Corynebacteriales; Mycobacteriaceae; Mycobacterium; Mycobacterium tuberculosis complex; Mycobacterium tuberculosis;	NCBI: 1773	4361435	99.94	0.00	Yes
NCBI Assembly	GCA_001394395.1	NCBI: cellular organisms; Bacteria; Terrabacteria group; Actinobacteria; Actinomycetia; Corynebacteriales; Mycobacteriaceae; Mycobacterium; Mycobacterium tuberculosis complex; Mycobacterium tuberculosis;	NCBI: 1773	4255438	99.78	0.00	Yes
NCBI Assembly	GCA_001394715.1	NCBI: cellular organisms; Bacteria; Terrabacteria group; Actinobacteria; Actinomycetia; Corynebacteriales; Mycobacteriaceae; Mycobacterium; Mycobacterium tuberculosis complex; Mycobacterium tuberculosis;	NCBI: 1773	4291785	99.78	0.00	Yes
NCBI Assembly	GCA_001396895.1	NCBI: cellular organisms; Bacteria; Terrabacteria group; Actinobacteria; Actinomycetia; Corynebacteriales; Mycobacteriaceae; Mycobacterium; Mycobacterium tuberculosis complex; Mycobacterium tuberculosis;	NCBI: 1773	4291020	99.56	0.00	Yes
NCBI Assembly	GCA_001544815.1	NCBI: cellular organisms; Bacteria; Terrabacteria group; Actinobacteria; Actinomycetia; Corynebacteriales; Mycobacteriaceae; Mycobacterium; Mycobacterium tuberculosis complex; Mycobacterium tuberculosis; Mycobacterium tuberculosis variant microti;	NCBI: 1806	4370115	99.27	0.00	Yes
NCBI Assembly	GCA_001440245.1	NCBI: cellular organisms; Bacteria; Terrabacteria group; Actinobacteria; Actinomycetia; Corynebacteriales; Mycobacteriaceae; Mycobacteroides; unclassified Mycobacteroides;	NCBI: 1720574	5723221	99.44	0.39	Yes
NCBI Assembly	GCA_001394115.1	NCBI: cellular organisms; Bacteria; Terrabacteria group; Actinobacteria; Actinomycetia; Corynebacteriales; Mycobacteriaceae; Mycobacterium; Mycobacterium tuberculosis complex; Mycobacterium tuberculosis;	NCBI: 1773	5060746	96.07	16.68	No
NCBI Assembly	GCA_001389395.1	NCBI: cellular organisms; Bacteria; Terrabacteria group; Actinobacteria; Actinomycetia; Corynebacteriales; Mycobacteriaceae; Mycobacterium; Mycobacterium tuberculosis complex; Mycobacterium tuberculosis;	NCBI: 1773	4351256	99.94	0.00	Yes

NCBI Assembly	GCA_001391915.1	NCBI: cellular organisms; Bacteria; Terrabacteria group; Actinobacteria; Actinomycetia; Corynebacteriales; Mycobacteriaceae; Mycobacterium; Mycobacterium tuberculosis complex; Mycobacterium tuberculosis;	NCBI: 1773	4288341	99.61	0.07	Yes
NCBI Assembly	GCA_001394415.1	NCBI: cellular organisms; Bacteria; Terrabacteria group; Actinobacteria; Actinomycetia; Corynebacteriales; Mycobacteriaceae; Mycobacterium; Mycobacterium tuberculosis complex; Mycobacterium tuberculosis;	NCBI: 1773	4279175	99.90	0.38	Yes
NCBI Assembly	GCA_001386915.1	NCBI: cellular organisms; Bacteria; Terrabacteria group; Actinobacteria; Actinomycetia; Corynebacteriales; Mycobacteriaceae; Mycobacterium; Mycobacterium tuberculosis complex; Mycobacterium tuberculosis;	NCBI: 1773	4362719	99.91	0.00	Yes
NCBI Assembly	GCA_001383495.1	NCBI: cellular organisms; Bacteria; Terrabacteria group; Actinobacteria; Actinomycetia; Corynebacteriales; Mycobacteriaceae; Mycobacterium; Mycobacterium tuberculosis complex; Mycobacterium tuberculosis;	NCBI: 1773	4360799	99.94	0.00	Yes
NCBI Assembly	GCA_001393175.1	NCBI: cellular organisms; Bacteria; Terrabacteria group; Actinobacteria; Actinomycetia; Corynebacteriales; Mycobacteriaceae; Mycobacterium; Mycobacterium tuberculosis complex; Mycobacterium tuberculosis;	NCBI: 1773	4331458	99.86	0.00	Yes
NCBI Assembly	GCA_001440265.1	NCBI: cellular organisms; Bacteria; Terrabacteria group; Actinobacteria; Actinomycetia; Corynebacteriales; Mycobacteriaceae; Mycobacteroides; unclassified Mycobacteroides;	NCBI: 1720575	5707994	99.44	0.39	Yes
NCBI Assembly	GCA_001389415.1	NCBI: cellular organisms; Bacteria; Terrabacteria group; Actinobacteria; Actinomycetia; Corynebacteriales; Mycobacteriaceae; Mycobacterium; Mycobacterium tuberculosis complex; Mycobacterium tuberculosis;	NCBI: 1773	4357011	99.94	0.00	Yes
NCBI Assembly	GCA_001394735.1	NCBI: cellular organisms; Bacteria; Terrabacteria group; Actinobacteria; Actinomycetia; Corynebacteriales; Mycobacteriaceae; Mycobacterium; Mycobacterium tuberculosis complex; Mycobacterium tuberculosis;	NCBI: 1773	4286933	99.94	0.00	Yes
NCBI Assembly	GCA_001393495.1	NCBI: cellular organisms; Bacteria; Terrabacteria group; Actinobacteria; Actinomycetia; Corynebacteriales; Mycobacteriaceae; Mycobacterium; Mycobacterium tuberculosis complex; Mycobacterium tuberculosis;	NCBI: 1773	4318562	99.61	0.00	Yes
NCBI Assembly	GCA_001396915.1	NCBI: cellular organisms; Bacteria; Terrabacteria group; Actinobacteria; Actinomycetia; Corynebacteriales; Mycobacteriaceae; Mycobacterium; Mycobacterium tuberculosis complex; Mycobacterium tuberculosis;	NCBI: 1773	3704217	87.66	3.92	No
NCBI Assembly	GCA_001544855.1	NCBI: cellular organisms; Bacteria; Terrabacteria group; Actinobacteria; Actinomycetia; Corynebacteriales; Mycobacteriaceae; Mycobacterium; Mycobacterium tuberculosis complex; Mycobacterium tuberculosis; Mycobacterium tuberculosis variant africanum;	NCBI: 33894	4386422	95.92	0.00	Yes
NCBI Assembly	GCA_001394095.1	NCBI: cellular organisms; Bacteria; Terrabacteria group; Actinobacteria; Actinomycetia; Corynebacteriales; Mycobacteriaceae; Mycobacterium; Mycobacterium tuberculosis complex; Mycobacterium tuberculosis;	NCBI: 1773	4302410	99.94	0.07	Yes
NCBI Assembly	GCA_001391935.1	NCBI: cellular organisms; Bacteria; Terrabacteria group; Actinobacteria; Actinomycetia; Corynebacteriales; Mycobacteriaceae; Mycobacterium; Mycobacterium tuberculosis complex; Mycobacterium tuberculosis;	NCBI: 1773	4325755	99.94	0.04	Yes
NCBI Assembly	GCA_001386935.1	NCBI: cellular organisms; Bacteria; Terrabacteria group; Actinobacteria; Actinomycetia; Corynebacteriales; Mycobacteriaceae; Mycobacterium; Mycobacterium tuberculosis complex; Mycobacterium tuberculosis;	NCBI: 1773	4359087	99.94	0.00	Yes
NCBI Assembly	GCA_001389435.1	NCBI: cellular organisms; Bacteria; Terrabacteria group; Actinobacteria; Actinomycetia; Corynebacteriales; Mycobacteriaceae; Mycobacterium; Mycobacterium tuberculosis complex; Mycobacterium tuberculosis;	NCBI: 1773	4352280	99.94	0.00	Yes
NCBI Assembly	GCA_001394435.1	NCBI: cellular organisms; Bacteria; Terrabacteria group; Actinobacteria; Actinomycetia; Corynebacteriales; Mycobacteriaceae; Mycobacterium; Mycobacterium tuberculosis complex; Mycobacterium tuberculosis;	NCBI: 1773	4310028	99.94	0.00	Yes
NCBI Assembly	GCA_001394755.1	NCBI: cellular organisms; Bacteria; Terrabacteria group; Actinobacteria; Actinomycetia; Corynebacteriales; Mycobacteriaceae; Mycobacterium; Mycobacterium tuberculosis complex; Mycobacterium tuberculosis;	NCBI: 1773	4321886	99.94	0.00	Yes
NCBI Assembly	GCA_001383515.1	NCBI: cellular organisms; Bacteria; Terrabacteria group; Actinobacteria; Actinomycetia; Corynebacteriales; Mycobacteriaceae; Mycobacterium; Mycobacterium tuberculosis complex; Mycobacterium tuberculosis;	NCBI: 1773	4371504	99.94	0.00	Yes

NCBI Assembly	GCA_001393195.1	NCBI: cellular organisms; Bacteria; Terrabacteria group; Actinobacteria; Actinomycetia; Corynebacteriales; Mycobacteriaceae; Mycobacterium; Mycobacterium tuberculosis complex; Mycobacterium tuberculosis;	NCBI: 1773	4318106	99.94	0.00	Yes
NCBI Assembly	GCA_001396935.1	NCBI: cellular organisms; Bacteria; Terrabacteria group; Actinobacteria; Actinomycetia; Corynebacteriales; Mycobacteriaceae; Mycobacterium; Mycobacterium tuberculosis complex; Mycobacterium tuberculosis;	NCBI: 1773	4280631	99.78	0.00	Yes
NCBI Assembly	GCA_001440275.1	NCBI: cellular organisms; Bacteria; Terrabacteria group; Actinobacteria; Actinomycetia; Corynebacteriales; Mycobacteriaceae; Mycobacteroides; unclassified Mycobacteroides;	NCBI: 1720576	5620117	99.44	0.39	Yes
NCBI Assembly	GCA_001544895.1	NCBI: cellular organisms; Bacteria; Terrabacteria group; Actinobacteria; Actinomycetia; Corynebacteriales; Mycobacteriaceae; Mycobacterium; Mycobacterium tuberculosis complex; Mycobacterium tuberculosis;	NCBI: 1773	4419839	97.70	0.00	Yes
NCBI Assembly	GCA_001394135.1	NCBI: cellular organisms; Bacteria; Terrabacteria group; Actinobacteria; Actinomycetia; Corynebacteriales; Mycobacteriaceae; Mycobacterium; Mycobacterium tuberculosis complex; Mycobacterium tuberculosis;	NCBI: 1773	4322361	99.94	0.00	Yes
NCBI Assembly	GCA_001393515.1	NCBI: cellular organisms; Bacteria; Terrabacteria group; Actinobacteria; Actinomycetia; Corynebacteriales; Mycobacteriaceae; Mycobacterium; Mycobacterium tuberculosis complex; Mycobacterium tuberculosis;	NCBI: 1773	4296945	99.90	0.00	Yes
NCBI Assembly	GCA_001383535.1	NCBI: cellular organisms; Bacteria; Terrabacteria group; Actinobacteria; Actinomycetia; Corynebacteriales; Mycobacteriaceae; Mycobacterium; Mycobacterium tuberculosis complex; Mycobacterium tuberculosis;	NCBI: 1773	4391170	99.94	0.00	Yes
NCBI Assembly	GCA_001386955.1	NCBI: cellular organisms; Bacteria; Terrabacteria group; Actinobacteria; Actinomycetia; Corynebacteriales; Mycobacteriaceae; Mycobacterium; Mycobacterium tuberculosis complex; Mycobacterium tuberculosis;	NCBI: 1773	4353475	99.94	0.00	Yes
NCBI Assembly	GCA_001391955.1	NCBI: cellular organisms; Bacteria; Terrabacteria group; Actinobacteria; Actinomycetia; Corynebacteriales; Mycobacteriaceae; Mycobacterium; Mycobacterium tuberculosis complex; Mycobacterium tuberculosis;	NCBI: 1773	4322514	99.94	0.00	Yes
NCBI Assembly	GCA_001389455.1	NCBI: cellular organisms; Bacteria; Terrabacteria group; Actinobacteria; Actinomycetia; Corynebacteriales; Mycobacteriaceae; Mycobacterium; Mycobacterium tuberculosis complex; Mycobacterium tuberculosis;	NCBI: 1773	4350256	99.94	0.00	Yes
NCBI Assembly	GCA_001394775.1	NCBI: cellular organisms; Bacteria; Terrabacteria group; Actinobacteria; Actinomycetia; Corynebacteriales; Mycobacteriaceae; Mycobacterium; Mycobacterium tuberculosis complex; Mycobacterium tuberculosis;	NCBI: 1773	4319284	99.61	0.04	Yes
NCBI Assembly	GCA_001393215.1	NCBI: cellular organisms; Bacteria; Terrabacteria group; Actinobacteria; Actinomycetia; Corynebacteriales; Mycobacteriaceae; Mycobacterium; Mycobacterium tuberculosis complex; Mycobacterium tuberculosis;	NCBI: 1773	4287823	99.94	0.00	Yes
NCBI Assembly	GCA_001394455.1	NCBI: cellular organisms; Bacteria; Terrabacteria group; Actinobacteria; Actinomycetia; Corynebacteriales; Mycobacteriaceae; Mycobacterium; Mycobacterium tuberculosis complex; Mycobacterium tuberculosis;	NCBI: 1773	4317094	99.50	0.00	Yes
NCBI Assembly	GCA_001440305.1	NCBI: cellular organisms; Bacteria; Terrabacteria group; Actinobacteria; Actinomycetia; Corynebacteriales; Mycobacteriaceae; Mycobacteroides; unclassified Mycobacteroides;	NCBI: 1720577	5724611	99.44	0.39	Yes
NCBI Assembly	GCA_001396955.1	NCBI: cellular organisms; Bacteria; Terrabacteria group; Actinobacteria; Actinomycetia; Corynebacteriales; Mycobacteriaceae; Mycobacterium; Mycobacterium tuberculosis complex; Mycobacterium tuberculosis;	NCBI: 1773	4302828	99.44	0.00	Yes
NCBI Assembly	GCA_001394155.1	NCBI: cellular organisms; Bacteria; Terrabacteria group; Actinobacteria; Actinomycetia; Corynebacteriales; Mycobacteriaceae; Mycobacterium; Mycobacterium tuberculosis complex; Mycobacterium tuberculosis;	NCBI: 1773	4308517	99.83	0.00	Yes
NCBI Assembly	GCA_001393535.1	NCBI: cellular organisms; Bacteria; Terrabacteria group; Actinobacteria; Actinomycetia; Corynebacteriales; Mycobacteriaceae; Mycobacterium; Mycobacterium tuberculosis complex; Mycobacterium tuberculosis;	NCBI: 1773	4286676	99.94	0.00	Yes
NCBI Assembly	GCA_001544935.1	NCBI: cellular organisms; Bacteria; Terrabacteria group; Actinobacteria; Actinomycetia; Corynebacteriales; Mycobacteriaceae; Mycobacterium; Mycobacterium tuberculosis complex; Mycobacterium tuberculosis;	NCBI: 1773	4405033	96.75	0.00	Yes

NCBI Assembly	GCA_001383555.1	NCBI: cellular organisms; Bacteria; Terrabacteria group; Actinobacteria; Actinomycetia; Corynebacteriales; Mycobacteriaceae; Mycobacterium; Mycobacterium tuberculosis complex; Mycobacterium tuberculosis;	NCBI: 1773	4363107	99.94	0.00	Yes
NCBI Assembly	GCA_001394795.1	NCBI: cellular organisms; Bacteria; Terrabacteria group; Actinobacteria; Actinomycetia; Corynebacteriales; Mycobacteriaceae; Mycobacterium; Mycobacterium tuberculosis complex; Mycobacterium tuberculosis;	NCBI: 1773	4284425	99.63	0.00	Yes
NCBI Assembly	GCA_001386975.1	NCBI: cellular organisms; Bacteria; Terrabacteria group; Actinobacteria; Actinomycetia; Corynebacteriales; Mycobacteriaceae; Mycobacterium; Mycobacterium tuberculosis complex; Mycobacterium tuberculosis;	NCBI: 1773	4344933	99.28	0.00	Yes
NCBI Assembly	GCA_001391975.1	NCBI: cellular organisms; Bacteria; Terrabacteria group; Actinobacteria; Actinomycetia; Corynebacteriales; Mycobacteriaceae; Mycobacterium; Mycobacterium tuberculosis complex; Mycobacterium tuberculosis;	NCBI: 1773	4327315	99.94	0.00	Yes
NCBI Assembly	GCA_001389475.1	NCBI: cellular organisms; Bacteria; Terrabacteria group; Actinobacteria; Actinomycetia; Corynebacteriales; Mycobacteriaceae; Mycobacterium; Mycobacterium tuberculosis complex; Mycobacterium tuberculosis;	NCBI: 1773	4351224	99.94	0.00	Yes
NCBI Assembly	GCA_001396975.1	NCBI: cellular organisms; Bacteria; Terrabacteria group; Actinobacteria; Actinomycetia; Corynebacteriales; Mycobacteriaceae; Mycobacterium; Mycobacterium tuberculosis complex; Mycobacterium tuberculosis;	NCBI: 1773	4307586	99.94	0.00	Yes
NCBI Assembly	GCA_001394475.1	NCBI: cellular organisms; Bacteria; Terrabacteria group; Actinobacteria; Actinomycetia; Corynebacteriales; Mycobacteriaceae; Mycobacterium; Mycobacterium tuberculosis complex; Mycobacterium tuberculosis;	NCBI: 1773	4299988	99.43	0.00	Yes
NCBI Assembly	GCA_001393235.1	NCBI: cellular organisms; Bacteria; Terrabacteria group; Actinobacteria; Actinomycetia; Corynebacteriales; Mycobacteriaceae; Mycobacterium; Mycobacterium tuberculosis complex; Mycobacterium tuberculosis;	NCBI: 1773	4270387	99.94	0.00	Yes
NCBI Assembly	GCA_001443085.1	NCBI: cellular organisms; Bacteria; Terrabacteria group; Actinobacteria; Actinomycetia; Corynebacteriales; Mycobacteriaceae; Mycobacterium; Mycobacterium tuberculosis complex; Mycobacterium tuberculosis;	NCBI: 1773	4330115	99.94	0.33	Yes
NCBI Assembly	GCA_001394175.1	NCBI: cellular organisms; Bacteria; Terrabacteria group; Actinobacteria; Actinomycetia; Corynebacteriales; Mycobacteriaceae; Mycobacterium; Mycobacterium tuberculosis complex; Mycobacterium tuberculosis;	NCBI: 1773	4249444	99.78	0.00	Yes
NCBI Assembly	GCA_001393555.1	NCBI: cellular organisms; Bacteria; Terrabacteria group; Actinobacteria; Actinomycetia; Corynebacteriales; Mycobacteriaceae; Mycobacterium; Mycobacterium tuberculosis complex; Mycobacterium tuberculosis;	NCBI: 1773	4308896	99.94	0.00	Yes
NCBI Assembly	GCA_001544955.1	NCBI: cellular organisms; Bacteria; Terrabacteria group; Actinobacteria; Actinomycetia; Corynebacteriales; Mycobacteriaceae; Mycobacterium; Mycobacterium tuberculosis complex; Mycobacterium tuberculosis;	NCBI: 1773	4401829	99.58	0.00	Yes
NCBI Assembly	GCA_001383575.1	NCBI: cellular organisms; Bacteria; Terrabacteria group; Actinobacteria; Actinomycetia; Corynebacteriales; Mycobacteriaceae; Mycobacterium; Mycobacterium tuberculosis complex; Mycobacterium tuberculosis;	NCBI: 1773	4382630	99.94	0.00	Yes
NCBI Assembly	GCA_001386995.1	NCBI: cellular organisms; Bacteria; Terrabacteria group; Actinobacteria; Actinomycetia; Corynebacteriales; Mycobacteriaceae; Mycobacterium; Mycobacterium tuberculosis complex; Mycobacterium tuberculosis;	NCBI: 1773	4349439	99.94	0.00	Yes
NCBI Assembly	GCA_001391995.1	NCBI: cellular organisms; Bacteria; Terrabacteria group; Actinobacteria; Actinomycetia; Corynebacteriales; Mycobacteriaceae; Mycobacterium; Mycobacterium tuberculosis complex; Mycobacterium tuberculosis;	NCBI: 1773	4303925	99.94	0.00	Yes
NCBI Assembly	GCA_001394815.1	NCBI: cellular organisms; Bacteria; Terrabacteria group; Actinobacteria; Actinomycetia; Corynebacteriales; Mycobacteriaceae; Mycobacterium; Mycobacterium tuberculosis complex; Mycobacterium tuberculosis;	NCBI: 1773	4345995	99.94	0.00	Yes
NCBI Assembly	GCA_001389495.1	NCBI: cellular organisms; Bacteria; Terrabacteria group; Actinobacteria; Actinomycetia; Corynebacteriales; Mycobacteriaceae; Mycobacterium; Mycobacterium tuberculosis complex; Mycobacterium tuberculosis;	NCBI: 1773	4359912	99.94	0.00	Yes
NCBI Assembly	GCA_001393255.1	NCBI: cellular organisms; Bacteria; Terrabacteria group; Actinobacteria; Actinomycetia; Corynebacteriales; Mycobacteriaceae; Mycobacterium; Mycobacterium tuberculosis complex; Mycobacterium tuberculosis;	NCBI: 1773	4321398	99.94	0.00	Yes

NCBI Assembly	GCA_001394495.1	NCBI: cellular organisms; Bacteria; Terrabacteria group; Actinobacteria; Actinomycetia; Corynebacteriales; Mycobacteriaceae; Mycobacterium; Mycobacterium tuberculosis complex; Mycobacterium tuberculosis;	NCBI: 1773	4340846	99.94	0.00	Yes
NCBI Assembly	GCA_001396995.1	NCBI: cellular organisms; Bacteria; Terrabacteria group; Actinobacteria; Actinomycetia; Corynebacteriales; Mycobacteriaceae; Mycobacterium; Mycobacterium tuberculosis complex; Mycobacterium tuberculosis;	NCBI: 1773	4368389	99.94	0.00	Yes
NCBI Assembly	GCA_001394195.1	NCBI: cellular organisms; Bacteria; Terrabacteria group; Actinobacteria; Actinomycetia; Corynebacteriales; Mycobacteriaceae; Mycobacterium; Mycobacterium tuberculosis complex; Mycobacterium tuberculosis;	NCBI: 1773	5156263	96.02	21.20	No
NCBI Assembly	GCA_001443105.1	NCBI: cellular organisms; Bacteria; Terrabacteria group; Actinobacteria; Actinomycetia; Corynebacteriales; Mycobacteriaceae; Mycobacterium; Mycobacterium tuberculosis complex; Mycobacterium tuberculosis;	NCBI: 1773	4303775	99.94	0.00	Yes
NCBI Assembly	GCA_001393575.1	NCBI: cellular organisms; Bacteria; Terrabacteria group; Actinobacteria; Actinomycetia; Corynebacteriales; Mycobacteriaceae; Mycobacterium; Mycobacterium tuberculosis complex; Mycobacterium tuberculosis;	NCBI: 1773	4318534	99.94	0.00	Yes
NCBI Assembly	GCA_001544985.1	NCBI: cellular organisms; Bacteria; Terrabacteria group; Actinobacteria; Actinomycetia; Corynebacteriales; Mycobacteriaceae; Mycobacterium; Mycobacterium tuberculosis complex; Mycobacterium tuberculosis;	NCBI: 1773	4417090	98.89	0.00	Yes
NCBI Assembly	GCA_001383595.1	NCBI: cellular organisms; Bacteria; Terrabacteria group; Actinobacteria; Actinomycetia; Corynebacteriales; Mycobacteriaceae; Mycobacterium; Mycobacterium tuberculosis complex; Mycobacterium tuberculosis;	NCBI: 1773	4364509	99.94	0.00	Yes
NCBI Assembly	GCA_001389515.1	NCBI: cellular organisms; Bacteria; Terrabacteria group; Actinobacteria; Actinomycetia; Corynebacteriales; Mycobacteriaceae; Mycobacterium; Mycobacterium tuberculosis complex; Mycobacterium tuberculosis;	NCBI: 1773	4365776	99.88	0.00	Yes
NCBI Assembly	GCA_001394835.1	NCBI: cellular organisms; Bacteria; Terrabacteria group; Actinobacteria; Actinomycetia; Corynebacteriales; Mycobacteriaceae; Mycobacterium; Mycobacterium tuberculosis complex; Mycobacterium tuberculosis;	NCBI: 1773	4344006	99.57	0.00	Yes
NCBI Assembly	GCA_001393275.1	NCBI: cellular organisms; Bacteria; Terrabacteria group; Actinobacteria; Actinomycetia; Corynebacteriales; Mycobacteriaceae; Mycobacterium; Mycobacterium tuberculosis complex; Mycobacterium tuberculosis;	NCBI: 1773	4301682	99.94	0.00	Yes
NCBI Assembly	GCA_001387015.1	NCBI: cellular organisms; Bacteria; Terrabacteria group; Actinobacteria; Actinomycetia; Corynebacteriales; Mycobacteriaceae; Mycobacterium; Mycobacterium tuberculosis complex; Mycobacterium tuberculosis;	NCBI: 1773	4341602	99.94	0.00	Yes
NCBI Assembly	GCA_001392015.1	NCBI: cellular organisms; Bacteria; Terrabacteria group; Actinobacteria; Actinomycetia; Corynebacteriales; Mycobacteriaceae; Mycobacterium; Mycobacterium tuberculosis complex; Mycobacterium tuberculosis;	NCBI: 1773	4332893	99.86	0.00	Yes
NCBI Assembly	GCA_001394515.1	NCBI: cellular organisms; Bacteria; Terrabacteria group; Actinobacteria; Actinomycetia; Corynebacteriales; Mycobacteriaceae; Mycobacterium; Mycobacterium tuberculosis complex; Mycobacterium tuberculosis;	NCBI: 1773	4340832	99.94	0.00	Yes
NCBI Assembly	GCA_001397015.1	NCBI: cellular organisms; Bacteria; Terrabacteria group; Actinobacteria; Actinomycetia; Corynebacteriales; Mycobacteriaceae; Mycobacterium; Mycobacterium tuberculosis complex; Mycobacterium tuberculosis;	NCBI: 1773	4286156	99.58	0.00	Yes
NCBI Assembly	GCA_001393595.1	NCBI: cellular organisms; Bacteria; Terrabacteria group; Actinobacteria; Actinomycetia; Corynebacteriales; Mycobacteriaceae; Mycobacterium; Mycobacterium tuberculosis complex; Mycobacterium tuberculosis;	NCBI: 1773	4296258	99.94	0.00	Yes
NCBI Assembly	GCA_001394215.1	NCBI: cellular organisms; Bacteria; Terrabacteria group; Actinobacteria; Actinomycetia; Corynebacteriales; Mycobacteriaceae; Mycobacterium; Mycobacterium tuberculosis complex; Mycobacterium tuberculosis;	NCBI: 1773	4325275	99.94	0.00	Yes
NCBI Assembly	GCA_001445055.1	NCBI: cellular organisms; Bacteria; Terrabacteria group; Actinobacteria; Actinomycetia; Corynebacteriales; Mycobacteriaceae; Mycobacterium; Mycobacterium tuberculosis complex; Mycobacterium tuberculosis;	NCBI: 1773	4351613	99.94	0.33	Yes
NCBI Assembly	GCA_001545015.1	NCBI: cellular organisms; Bacteria; Terrabacteria group; Actinobacteria; Actinomycetia; Corynebacteriales; Mycobacteriaceae; Mycobacterium; Mycobacterium tuberculosis complex; Mycobacterium tuberculosis;	NCBI: 1773	4399422	99.94	0.00	Yes

NCBI Assembly	GCA_001383615.1	NCBI: cellular organisms; Bacteria; Terrabacteria group; Actinobacteria; Actinomycetia; Corynebacteriales; Mycobacteriaceae; Mycobacterium; Mycobacterium tuberculosis complex; Mycobacterium tuberculosis;	NCBI: 1773	4362224	99.94	0.00	Yes
NCBI Assembly	GCA_001389535.1	NCBI: cellular organisms; Bacteria; Terrabacteria group; Actinobacteria; Actinomycetia; Corynebacteriales; Mycobacteriaceae; Mycobacterium; Mycobacterium tuberculosis complex; Mycobacterium tuberculosis;	NCBI: 1773	4343680	99.94	0.00	Yes
NCBI Assembly	GCA_001394855.1	NCBI: cellular organisms; Bacteria; Terrabacteria group; Actinobacteria; Actinomycetia; Corynebacteriales; Mycobacteriaceae; Mycobacterium; Mycobacterium tuberculosis complex; Mycobacterium tuberculosis;	NCBI: 1773	5425737	96.49	23.12	No
NCBI Assembly	GCA_001394535.1	NCBI: cellular organisms; Bacteria; Terrabacteria group; Actinobacteria; Actinomycetia; Corynebacteriales; Mycobacteriaceae; Mycobacterium; Mycobacterium tuberculosis complex; Mycobacterium tuberculosis;	NCBI: 1773	4316439	99.94	0.00	Yes
NCBI Assembly	GCA_001397035.1	NCBI: cellular organisms; Bacteria; Terrabacteria group; Actinobacteria; Actinomycetia; Corynebacteriales; Mycobacteriaceae; Mycobacterium; Mycobacterium tuberculosis complex; Mycobacterium tuberculosis;	NCBI: 1773	4296927	99.94	0.00	Yes
NCBI Assembly	GCA_001392035.1	NCBI: cellular organisms; Bacteria; Terrabacteria group; Actinobacteria; Actinomycetia; Corynebacteriales; Mycobacteriaceae; Mycobacterium; Mycobacterium tuberculosis complex; Mycobacterium tuberculosis;	NCBI: 1773	4297641	99.94	0.00	Yes
NCBI Assembly	GCA_001387035.1	NCBI: cellular organisms; Bacteria; Terrabacteria group; Actinobacteria; Actinomycetia; Corynebacteriales; Mycobacteriaceae; Mycobacterium; Mycobacterium tuberculosis complex; Mycobacterium tuberculosis;	NCBI: 1773	4384520	99.94	0.00	Yes
NCBI Assembly	GCA_001393295.1	NCBI: cellular organisms; Bacteria; Terrabacteria group; Actinobacteria; Actinomycetia; Corynebacteriales; Mycobacteriaceae; Mycobacterium; Mycobacterium tuberculosis complex; Mycobacterium tuberculosis;	NCBI: 1773	4316813	99.94	0.00	Yes
NCBI Assembly	GCA_001394235.1	NCBI: cellular organisms; Bacteria; Terrabacteria group; Actinobacteria; Actinomycetia; Corynebacteriales; Mycobacteriaceae; Mycobacterium; Mycobacterium tuberculosis complex; Mycobacterium tuberculosis;	NCBI: 1773	4311874	99.94	0.00	Yes
NCBI Assembly	GCA_001393615.1	NCBI: cellular organisms; Bacteria; Terrabacteria group; Actinobacteria; Actinomycetia; Corynebacteriales; Mycobacteriaceae; Mycobacterium; Mycobacterium tuberculosis complex; Mycobacterium tuberculosis;	NCBI: 1773	4323514	99.94	0.00	Yes
NCBI Assembly	GCA_001445075.1	NCBI: cellular organisms; Bacteria; Terrabacteria group; Actinobacteria; Actinomycetia; Corynebacteriales; Mycobacteriaceae; Mycobacterium; Mycobacterium tuberculosis complex; Mycobacterium tuberculosis;	NCBI: 1773	4336327	99.94	0.00	Yes
NCBI Assembly	GCA_001545055.1	NCBI: cellular organisms; Bacteria; Terrabacteria group; Actinobacteria; Actinomycetia; Corynebacteriales; Mycobacteriaceae; Mycobacterium; Mycobacterium tuberculosis complex; Mycobacterium tuberculosis;	NCBI: 1773	4426489	99.67	0.00	Yes
NCBI Assembly	GCA_001389555.1	NCBI: cellular organisms; Bacteria; Terrabacteria group; Actinobacteria; Actinomycetia; Corynebacteriales; Mycobacteriaceae; Mycobacterium; Mycobacterium tuberculosis complex; Mycobacterium tuberculosis;	NCBI: 1773	4346732	99.94	0.00	Yes
NCBI Assembly	GCA_001383635.1	NCBI: cellular organisms; Bacteria; Terrabacteria group; Actinobacteria; Actinomycetia; Corynebacteriales; Mycobacteriaceae; Mycobacterium; Mycobacterium tuberculosis complex; Mycobacterium tuberculosis;	NCBI: 1773	4367852	99.94	0.00	Yes
NCBI Assembly	GCA_001392055.1	NCBI: cellular organisms; Bacteria; Terrabacteria group; Actinobacteria; Actinomycetia; Corynebacteriales; Mycobacteriaceae; Mycobacterium; Mycobacterium tuberculosis complex; Mycobacterium tuberculosis;	NCBI: 1773	4269197	99.78	0.00	Yes
NCBI Assembly	GCA_001394875.1	NCBI: cellular organisms; Bacteria; Terrabacteria group; Actinobacteria; Actinomycetia; Corynebacteriales; Mycobacteriaceae; Mycobacterium; Mycobacterium tuberculosis complex; Mycobacterium tuberculosis;	NCBI: 1773	4283182	99.57	0.38	Yes
NCBI Assembly	GCA_001394555.1	NCBI: cellular organisms; Bacteria; Terrabacteria group; Actinobacteria; Actinomycetia; Corynebacteriales; Mycobacteriaceae; Mycobacterium; Mycobacterium tuberculosis complex; Mycobacterium tuberculosis;	NCBI: 1773	4580354	99.91	2.71	Yes
NCBI Assembly	GCA_001387055.1	NCBI: cellular organisms; Bacteria; Terrabacteria group; Actinobacteria; Actinomycetia; Corynebacteriales; Mycobacteriaceae; Mycobacterium; Mycobacterium tuberculosis complex; Mycobacterium tuberculosis;	NCBI: 1773	4347659	99.94	0.00	Yes

NCBI Assembly	GCA_001393635.1	NCBI: cellular organisms; Bacteria; Terrabacteria group; Actinobacteria; Actinomycetia; Corynebacteriales; Mycobacteriaceae; Mycobacterium; Mycobacterium tuberculosis complex; Mycobacterium tuberculosis;	NCBI: 1773	4244267	99.50	0.00	Yes
NCBI Assembly	GCA_001393315.1	NCBI: cellular organisms; Bacteria; Terrabacteria group; Actinobacteria; Actinomycetia; Corynebacteriales; Mycobacteriaceae; Mycobacterium; Mycobacterium tuberculosis complex; Mycobacterium tuberculosis;	NCBI: 1773	4291320	99.89	0.00	Yes
NCBI Assembly	GCA_001397055.1	NCBI: cellular organisms; Bacteria; Terrabacteria group; Actinobacteria; Actinomycetia; Corynebacteriales; Mycobacteriaceae; Mycobacterium; Mycobacterium tuberculosis complex; Mycobacterium tuberculosis;	NCBI: 1773	4306396	99.88	0.00	Yes
NCBI Assembly	GCA_001394255.1	NCBI: cellular organisms; Bacteria; Terrabacteria group; Actinobacteria; Actinomycetia; Corynebacteriales; Mycobacteriaceae; Mycobacterium; Mycobacterium tuberculosis complex; Mycobacterium tuberculosis;	NCBI: 1773	4335857	99.94	0.07	Yes
NCBI Assembly	GCA_001445095.1	NCBI: cellular organisms; Bacteria; Terrabacteria group; Actinobacteria; Actinomycetia; Corynebacteriales; Mycobacteriaceae; Mycobacterium; Mycobacterium tuberculosis complex; Mycobacterium tuberculosis;	NCBI: 1773	4393041	99.94	0.00	Yes
NCBI Assembly	GCA_001545135.1	NCBI: cellular organisms; Bacteria; Terrabacteria group; Actinobacteria; Actinomycetia; Corynebacteriales; Mycobacteriaceae; Mycolicibacterium; Mycolicibacterium wolinskyi;	NCBI: 59750	7449739	99.94	1.55	Yes
NCBI Assembly	GCA_001383655.1	NCBI: cellular organisms; Bacteria; Terrabacteria group; Actinobacteria; Actinomycetia; Corynebacteriales; Mycobacteriaceae; Mycobacterium; Mycobacterium tuberculosis complex; Mycobacterium tuberculosis;	NCBI: 1773	4374233	99.94	0.50	Yes
NCBI Assembly	GCA_001389575.1	NCBI: cellular organisms; Bacteria; Terrabacteria group; Actinobacteria; Actinomycetia; Corynebacteriales; Mycobacteriaceae; Mycobacterium; Mycobacterium tuberculosis complex; Mycobacterium tuberculosis;	NCBI: 1773	4346378	99.88	0.00	Yes
NCBI Assembly	GCA_001392075.1	NCBI: cellular organisms; Bacteria; Terrabacteria group; Actinobacteria; Actinomycetia; Corynebacteriales; Mycobacteriaceae; Mycobacterium; Mycobacterium tuberculosis complex; Mycobacterium tuberculosis;	NCBI: 1773	4355302	99.94	0.00	Yes
NCBI Assembly	GCA_001387075.1	NCBI: cellular organisms; Bacteria; Terrabacteria group; Actinobacteria; Actinomycetia; Corynebacteriales; Mycobacteriaceae; Mycobacterium; Mycobacterium tuberculosis complex; Mycobacterium tuberculosis;	NCBI: 1773	4352967	99.88	0.00	Yes
NCBI Assembly	GCA_001393655.1	NCBI: cellular organisms; Bacteria; Terrabacteria group; Actinobacteria; Actinomycetia; Corynebacteriales; Mycobacteriaceae; Mycobacterium; Mycobacterium tuberculosis complex; Mycobacterium tuberculosis;	NCBI: 1773	4307751	99.81	0.00	Yes
NCBI Assembly	GCA_001393335.1	NCBI: cellular organisms; Bacteria; Terrabacteria group; Actinobacteria; Actinomycetia; Corynebacteriales; Mycobacteriaceae; Mycobacterium; Mycobacterium tuberculosis complex; Mycobacterium tuberculosis;	NCBI: 1773	4283607	99.78	0.00	Yes
NCBI Assembly	GCA_001394895.1	NCBI: cellular organisms; Bacteria; Terrabacteria group; Actinobacteria; Actinomycetia; Corynebacteriales; Mycobacteriaceae; Mycobacterium; Mycobacterium tuberculosis complex; Mycobacterium tuberculosis;	NCBI: 1773	4339982	99.94	0.00	Yes
NCBI Assembly	GCA_001397075.1	NCBI: cellular organisms; Bacteria; Terrabacteria group; Actinobacteria; Actinomycetia; Corynebacteriales; Mycobacteriaceae; Mycobacterium; Mycobacterium tuberculosis complex; Mycobacterium tuberculosis;	NCBI: 1773	4305582	99.94	0.00	Yes
NCBI Assembly	GCA_001394275.1	NCBI: cellular organisms; Bacteria; Terrabacteria group; Actinobacteria; Actinomycetia; Corynebacteriales; Mycobacteriaceae; Mycobacterium; Mycobacterium tuberculosis complex; Mycobacterium tuberculosis;	NCBI: 1773	4299348	99.94	0.00	Yes
NCBI Assembly	GCA_001394575.1	NCBI: cellular organisms; Bacteria; Terrabacteria group; Actinobacteria; Actinomycetia; Corynebacteriales; Mycobacteriaceae; Mycobacterium; Mycobacterium tuberculosis complex; Mycobacterium tuberculosis;	NCBI: 1773	4252548	99.78	0.00	Yes
NCBI Assembly	GCA_001445115.1	NCBI: cellular organisms; Bacteria; Terrabacteria group; Actinobacteria; Actinomycetia; Corynebacteriales; Mycobacteriaceae; Mycobacterium; Mycobacterium tuberculosis complex; Mycobacterium tuberculosis;	NCBI: 1773	4435898	99.94	3.67	Yes
NCBI Assembly	GCA_001545925.1	NCBI: cellular organisms; Bacteria; Terrabacteria group; Actinobacteria; Actinomycetia; Corynebacteriales; Mycobacteriaceae; Mycobacterium; unclassified Mycobacterium;	NCBI: 1747766	6094859	98.39	4.04	Yes

NCBI Assembly	GCA_001383675.1	NCBI: cellular organisms; Bacteria; Terrabacteria group; Actinobacteria; Actinomycetia; Corynebacteriales; Mycobacteriaceae; Mycobacterium; Mycobacterium tuberculosis complex; Mycobacterium tuberculosis;	NCBI: 1773	4364792	99.94	0.17	Yes
NCBI Assembly	GCA_001389595.1	NCBI: cellular organisms; Bacteria; Terrabacteria group; Actinobacteria; Actinomycetia; Corynebacteriales; Mycobacteriaceae; Mycobacterium; Mycobacterium tuberculosis complex; Mycobacterium tuberculosis;	NCBI: 1773	4350195	99.94	0.07	Yes
NCBI Assembly	GCA_001393675.1	NCBI: cellular organisms; Bacteria; Terrabacteria group; Actinobacteria; Actinomycetia; Corynebacteriales; Mycobacteriaceae; Mycobacterium; Mycobacterium tuberculosis complex; Mycobacterium tuberculosis;	NCBI: 1773	4303943	99.88	0.00	Yes
NCBI Assembly	GCA_001392095.1	NCBI: cellular organisms; Bacteria; Terrabacteria group; Actinobacteria; Actinomycetia; Corynebacteriales; Mycobacteriaceae; Mycobacterium; Mycobacterium tuberculosis complex; Mycobacterium tuberculosis;	NCBI: 1773	4328083	99.94	0.00	Yes
NCBI Assembly	GCA_001394915.1	NCBI: cellular organisms; Bacteria; Terrabacteria group; Actinobacteria; Actinomycetia; Corynebacteriales; Mycobacteriaceae; Mycobacterium; Mycobacterium tuberculosis complex; Mycobacterium tuberculosis;	NCBI: 1773	4309129	99.94	0.33	Yes
NCBI Assembly	GCA_001394595.1	NCBI: cellular organisms; Bacteria; Terrabacteria group; Actinobacteria; Actinomycetia; Corynebacteriales; Mycobacteriaceae; Mycobacterium; Mycobacterium tuberculosis complex; Mycobacterium tuberculosis;	NCBI: 1773	4337832	99.94	0.00	Yes
NCBI Assembly	GCA_001393355.1	NCBI: cellular organisms; Bacteria; Terrabacteria group; Actinobacteria; Actinomycetia; Corynebacteriales; Mycobacteriaceae; Mycobacterium; Mycobacterium tuberculosis complex; Mycobacterium tuberculosis;	NCBI: 1773	4335296	99.94	0.33	Yes
NCBI Assembly	GCA_001387095.1	NCBI: cellular organisms; Bacteria; Terrabacteria group; Actinobacteria; Actinomycetia; Corynebacteriales; Mycobacteriaceae; Mycobacterium; Mycobacterium tuberculosis complex; Mycobacterium tuberculosis;	NCBI: 1773	4339729	99.94	0.00	Yes
NCBI Assembly	GCA_001394295.1	NCBI: cellular organisms; Bacteria; Terrabacteria group; Actinobacteria; Actinomycetia; Corynebacteriales; Mycobacteriaceae; Mycobacterium; Mycobacterium tuberculosis complex; Mycobacterium tuberculosis;	NCBI: 1773	4351089	99.94	0.00	Yes
NCBI Assembly	GCA_001397095.1	NCBI: cellular organisms; Bacteria; Terrabacteria group; Actinobacteria; Actinomycetia; Corynebacteriales; Mycobacteriaceae; Mycobacterium; Mycobacterium tuberculosis complex; Mycobacterium tuberculosis;	NCBI: 1773	4317877	99.94	0.04	Yes
NCBI Assembly	GCA_001445135.1	NCBI: cellular organisms; Bacteria; Terrabacteria group; Actinobacteria; Actinomycetia; Corynebacteriales; Mycobacteriaceae; Mycobacterium; Mycobacterium tuberculosis complex; Mycobacterium tuberculosis;	NCBI: 1773	4414111	99.94	1.33	Yes
NCBI Assembly	GCA_001552315.1	NCBI: cellular organisms; Bacteria; Terrabacteria group; Actinobacteria; Actinomycetia; Corynebacteriales; Mycobacteriaceae; Mycolicibacterium; Mycolicibacterium chlorophenolicum;	NCBI: 1303685	7330216	100.00	1.12	Yes
NCBI Assembly	GCA_001389615.1	NCBI: cellular organisms; Bacteria; Terrabacteria group; Actinobacteria; Actinomycetia; Corynebacteriales; Mycobacteriaceae; Mycobacterium; Mycobacterium tuberculosis complex; Mycobacterium tuberculosis;	NCBI: 1773	4387860	99.94	0.00	Yes
NCBI Assembly	GCA_001383695.1	NCBI: cellular organisms; Bacteria; Terrabacteria group; Actinobacteria; Actinomycetia; Corynebacteriales; Mycobacteriaceae; Mycobacterium; Mycobacterium tuberculosis complex; Mycobacterium tuberculosis;	NCBI: 1773	4382599	99.94	0.00	Yes
NCBI Assembly	GCA_001392115.1	NCBI: cellular organisms; Bacteria; Terrabacteria group; Actinobacteria; Actinomycetia; Corynebacteriales; Mycobacteriaceae; Mycobacterium; Mycobacterium tuberculosis complex; Mycobacterium tuberculosis;	NCBI: 1773	4327139	99.61	0.00	Yes
NCBI Assembly	GCA_001387115.1	NCBI: cellular organisms; Bacteria; Terrabacteria group; Actinobacteria; Actinomycetia; Corynebacteriales; Mycobacteriaceae; Mycobacterium; Mycobacterium tuberculosis complex; Mycobacterium tuberculosis;	NCBI: 1773	4351332	99.94	0.00	Yes
NCBI Assembly	GCA_001393695.1	NCBI: cellular organisms; Bacteria; Terrabacteria group; Actinobacteria; Actinomycetia; Corynebacteriales; Mycobacteriaceae; Mycobacterium; Mycobacterium tuberculosis complex; Mycobacterium tuberculosis;	NCBI: 1773	4307924	99.94	0.00	Yes
NCBI Assembly	GCA_001393375.1	NCBI: cellular organisms; Bacteria; Terrabacteria group; Actinobacteria; Actinomycetia; Corynebacteriales; Mycobacteriaceae; Mycobacterium; Mycobacterium tuberculosis complex; Mycobacterium tuberculosis;	NCBI: 1773	4313311	99.72	0.00	Yes

NCBI Assembly	GCA_001394935.1	NCBI: cellular organisms; Bacteria; Terrabacteria group; Actinobacteria; Actinomycetia; Corynebacteriales; Mycobacteriaceae; Mycobacterium; Mycobacterium tuberculosis complex; Mycobacterium tuberculosis;	NCBI: 1773	4303388	99.94	0.04	Yes
NCBI Assembly	GCA_001394615.1	NCBI: cellular organisms; Bacteria; Terrabacteria group; Actinobacteria; Actinomycetia; Corynebacteriales; Mycobacteriaceae; Mycobacterium; Mycobacterium tuberculosis complex; Mycobacterium tuberculosis;	NCBI: 1773	4297260	99.83	0.00	Yes
NCBI Assembly	GCA_001394315.1	NCBI: cellular organisms; Bacteria; Terrabacteria group; Actinobacteria; Actinomycetia; Corynebacteriales; Mycobacteriaceae; Mycobacterium; Mycobacterium tuberculosis complex; Mycobacterium tuberculosis;	NCBI: 1773	4856293	95.58	14.27	No
NCBI Assembly	GCA_001397115.1	NCBI: cellular organisms; Bacteria; Terrabacteria group; Actinobacteria; Actinomycetia; Corynebacteriales; Mycobacteriaceae; Mycobacterium; Mycobacterium tuberculosis complex; Mycobacterium tuberculosis;	NCBI: 1773	4370351	99.94	0.33	Yes
NCBI Assembly	GCA_001445155.1	NCBI: cellular organisms; Bacteria; Terrabacteria group; Actinobacteria; Actinomycetia; Corynebacteriales; Mycobacteriaceae; Mycobacterium; Mycobacterium tuberculosis complex; Mycobacterium tuberculosis;	NCBI: 1773	4383883	99.94	0.00	Yes
NCBI Assembly	GCA_001552715.1	NCBI: cellular organisms; Bacteria; Terrabacteria group; Actinobacteria; Actinomycetia; Corynebacteriales; Mycobacteriaceae; Mycolicibacterium; Mycolicibacterium vaccae;	NCBI: 1223499	6168821	99.50	0.21	Yes
NCBI Assembly	GCA_001383715.1	NCBI: cellular organisms; Bacteria; Terrabacteria group; Actinobacteria; Actinomycetia; Corynebacteriales; Mycobacteriaceae; Mycobacterium; Mycobacterium tuberculosis complex; Mycobacterium tuberculosis;	NCBI: 1773	4369940	99.94	0.17	Yes
NCBI Assembly	GCA_001387135.1	NCBI: cellular organisms; Bacteria; Terrabacteria group; Actinobacteria; Actinomycetia; Corynebacteriales; Mycobacteriaceae; Mycobacterium; Mycobacterium tuberculosis complex; Mycobacterium tuberculosis;	NCBI: 1773	4380961	99.94	0.00	Yes
NCBI Assembly	GCA_001392135.1	NCBI: cellular organisms; Bacteria; Terrabacteria group; Actinobacteria; Actinomycetia; Corynebacteriales; Mycobacteriaceae; Mycobacterium; Mycobacterium tuberculosis complex; Mycobacterium tuberculosis;	NCBI: 1773	4327879	99.94	0.07	Yes
NCBI Assembly	GCA_001389635.1	NCBI: cellular organisms; Bacteria; Terrabacteria group; Actinobacteria; Actinomycetia; Corynebacteriales; Mycobacteriaceae; Mycobacterium; Mycobacterium tuberculosis complex; Mycobacterium tuberculosis;	NCBI: 1773	4358099	99.94	0.00	Yes
NCBI Assembly	GCA_001393395.1	NCBI: cellular organisms; Bacteria; Terrabacteria group; Actinobacteria; Actinomycetia; Corynebacteriales; Mycobacteriaceae; Mycobacterium; Mycobacterium tuberculosis complex; Mycobacterium tuberculosis;	NCBI: 1773	4298135	99.61	0.00	Yes
NCBI Assembly	GCA_001393715.1	NCBI: cellular organisms; Bacteria; Terrabacteria group; Actinobacteria; Actinomycetia; Corynebacteriales; Mycobacteriaceae; Mycobacterium; Mycobacterium tuberculosis complex; Mycobacterium tuberculosis;	NCBI: 1773	4298715	99.94	0.11	Yes
NCBI Assembly	GCA_001394955.1	NCBI: cellular organisms; Bacteria; Terrabacteria group; Actinobacteria; Actinomycetia; Corynebacteriales; Mycobacteriaceae; Mycobacterium; Mycobacterium tuberculosis complex; Mycobacterium tuberculosis;	NCBI: 1773	4318365	99.61	0.00	Yes
NCBI Assembly	GCA_001394335.1	NCBI: cellular organisms; Bacteria; Terrabacteria group; Actinobacteria; Actinomycetia; Corynebacteriales; Mycobacteriaceae; Mycobacterium; Mycobacterium tuberculosis complex; Mycobacterium tuberculosis;	NCBI: 1773	4333737	99.94	0.00	Yes
NCBI Assembly	GCA_001394635.1	NCBI: cellular organisms; Bacteria; Terrabacteria group; Actinobacteria; Actinomycetia; Corynebacteriales; Mycobacteriaceae; Mycobacterium; Mycobacterium tuberculosis complex; Mycobacterium tuberculosis;	NCBI: 1773	4296808	99.94	0.00	Yes
NCBI Assembly	GCA_001397135.1	NCBI: cellular organisms; Bacteria; Terrabacteria group; Actinobacteria; Actinomycetia; Corynebacteriales; Mycobacteriaceae; Mycobacterium; Mycobacterium tuberculosis complex; Mycobacterium tuberculosis;	NCBI: 1773	4310373	99.92	0.00	Yes
NCBI Assembly	GCA_001445175.1	NCBI: cellular organisms; Bacteria; Terrabacteria group; Actinobacteria; Actinomycetia; Corynebacteriales; Mycobacteriaceae; Mycobacterium; Mycobacterium tuberculosis complex; Mycobacterium tuberculosis;	NCBI: 1773	4405690	99.94	1.00	Yes
NCBI Assembly	GCA_001556905.1	NCBI: cellular organisms; Bacteria; Terrabacteria group; Actinobacteria; Actinomycetia; Corynebacteriales; Mycobacteriaceae; Mycolicibacterium; Mycolicibacterium mucogenicum;	NCBI: 56689	6361488	97.44	1.51	Yes

NCBI Assembly	GCA_001387155.1	NCBI: cellular organisms; Bacteria; Terrabacteria group; Actinobacteria; Actinomycetia; Corynebacteriales; Mycobacteriaceae; Mycobacterium; Mycobacterium tuberculosis complex; Mycobacterium tuberculosis;	NCBI: 1773	4363055	99.94	0.00	Yes
NCBI Assembly	GCA_001389655.1	NCBI: cellular organisms; Bacteria; Terrabacteria group; Actinobacteria; Actinomycetia; Corynebacteriales; Mycobacteriaceae; Mycobacterium; Mycobacterium tuberculosis complex; Mycobacterium tuberculosis;	NCBI: 1773	4361961	99.94	0.00	Yes
NCBI Assembly	GCA_001383735.1	NCBI: cellular organisms; Bacteria; Terrabacteria group; Actinobacteria; Actinomycetia; Corynebacteriales; Mycobacteriaceae; Mycobacterium; Mycobacterium tuberculosis complex; Mycobacterium tuberculosis;	NCBI: 1773	4386036	99.94	0.00	Yes
NCBI Assembly	GCA_001393735.1	NCBI: cellular organisms; Bacteria; Terrabacteria group; Actinobacteria; Actinomycetia; Corynebacteriales; Mycobacteriaceae; Mycobacterium; Mycobacterium tuberculosis complex; Mycobacterium tuberculosis;	NCBI: 1773	4320020	99.94	0.00	Yes
NCBI Assembly	GCA_001394655.1	NCBI: cellular organisms; Bacteria; Terrabacteria group; Actinobacteria; Actinomycetia; Corynebacteriales; Mycobacteriaceae; Mycobacterium; Mycobacterium tuberculosis complex; Mycobacterium tuberculosis;	NCBI: 1773	4327781	99.94	0.00	Yes
NCBI Assembly	GCA_001392155.1	NCBI: cellular organisms; Bacteria; Terrabacteria group; Actinobacteria; Actinomycetia; Corynebacteriales; Mycobacteriaceae; Mycobacterium; Mycobacterium tuberculosis complex; Mycobacterium tuberculosis;	NCBI: 1773	4282729	99.94	0.07	Yes
NCBI Assembly	GCA_001392815.1	NCBI: cellular organisms; Bacteria; Terrabacteria group; Actinobacteria; Actinomycetia; Corynebacteriales; Mycobacteriaceae; Mycobacterium; Mycobacterium tuberculosis complex; Mycobacterium tuberculosis;	NCBI: 1773	4335884	99.94	0.00	Yes
NCBI Assembly	GCA_001397155.1	NCBI: cellular organisms; Bacteria; Terrabacteria group; Actinobacteria; Actinomycetia; Corynebacteriales; Mycobacteriaceae; Mycobacterium; Mycobacterium tuberculosis complex; Mycobacterium tuberculosis;	NCBI: 1773	4293269	99.90	0.00	Yes
NCBI Assembly	GCA_001394975.1	NCBI: cellular organisms; Bacteria; Terrabacteria group; Actinobacteria; Actinomycetia; Corynebacteriales; Mycobacteriaceae; Mycobacterium; Mycobacterium tuberculosis complex; Mycobacterium tuberculosis;	NCBI: 1773	4327807	99.90	0.00	Yes
NCBI Assembly	GCA_001393415.1	NCBI: cellular organisms; Bacteria; Terrabacteria group; Actinobacteria; Actinomycetia; Corynebacteriales; Mycobacteriaceae; Mycobacterium; Mycobacterium tuberculosis complex; Mycobacterium tuberculosis;	NCBI: 1773	4313463	99.83	0.00	Yes
NCBI Assembly	GCA_001445185.1	NCBI: cellular organisms; Bacteria; Terrabacteria group; Actinobacteria; Actinomycetia; Corynebacteriales; Mycobacteriaceae; Mycobacterium; Mycobacterium tuberculosis complex; Mycobacterium tuberculosis;	NCBI: 1773	4400334	99.94	2.00	Yes
NCBI Assembly	GCA_001557045.1	NCBI: cellular organisms; Bacteria; Terrabacteria group; Actinobacteria; Actinomycetia; Corynebacteriales; Mycobacteriaceae; Mycolicibacterium; Mycolicibacterium mucogenicum;	NCBI: 56689	6470571	96.92	0.63	Yes
NCBI Assembly	GCA_001386555.1	NCBI: cellular organisms; Bacteria; Terrabacteria group; Actinobacteria; Actinomycetia; Corynebacteriales; Mycobacteriaceae; Mycobacterium; Mycobacterium tuberculosis complex; Mycobacterium tuberculosis;	NCBI: 1773	4363446	99.94	0.00	Yes
NCBI Assembly	GCA_001383755.1	NCBI: cellular organisms; Bacteria; Terrabacteria group; Actinobacteria; Actinomycetia; Corynebacteriales; Mycobacteriaceae; Mycobacterium; Mycobacterium tuberculosis complex; Mycobacterium tuberculosis;	NCBI: 1773	4366320	99.94	0.00	Yes
NCBI Assembly	GCA_001391555.1	NCBI: cellular organisms; Bacteria; Terrabacteria group; Actinobacteria; Actinomycetia; Corynebacteriales; Mycobacteriaceae; Mycobacterium; Mycobacterium tuberculosis complex; Mycobacterium tuberculosis;	NCBI: 1773	4332083	99.94	0.00	Yes
NCBI Assembly	GCA_001389055.1	NCBI: cellular organisms; Bacteria; Terrabacteria group; Actinobacteria; Actinomycetia; Corynebacteriales; Mycobacteriaceae; Mycobacterium; Mycobacterium tuberculosis complex; Mycobacterium tuberculosis;	NCBI: 1773	4340696	99.94	0.00	Yes
NCBI Assembly	GCA_001393755.1	NCBI: cellular organisms; Bacteria; Terrabacteria group; Actinobacteria; Actinomycetia; Corynebacteriales; Mycobacteriaceae; Mycobacterium; Mycobacterium tuberculosis complex; Mycobacterium tuberculosis;	NCBI: 1773	4300362	99.94	0.00	Yes
NCBI Assembly	GCA_001388435.1	NCBI: cellular organisms; Bacteria; Terrabacteria group; Actinobacteria; Actinomycetia; Corynebacteriales; Mycobacteriaceae; Mycobacterium; Mycobacterium tuberculosis complex; Mycobacterium tuberculosis;	NCBI: 1773	4350057	99.94	0.00	Yes

NCBI Assembly	GCA_001396555.1	NCBI: cellular organisms; Bacteria; Terrabacteria group; Actinobacteria; Actinomycetia; Corynebacteriales; Mycobacteriaceae; Mycobacterium; Mycobacterium tuberculosis complex; Mycobacterium tuberculosis;	NCBI: 1773	4292386	99.61	0.00	Yes
NCBI Assembly	GCA_001414075.1	NCBI: cellular organisms; Bacteria; Terrabacteria group; Actinobacteria; Actinomycetia; Corynebacteriales; Mycobacteriaceae; Mycobacterium; unclassified Mycobacterium;	NCBI: 1638773	5219141	99.56	0.67	Yes
NCBI Assembly	GCA_001392835.1	NCBI: cellular organisms; Bacteria; Terrabacteria group; Actinobacteria; Actinomycetia; Corynebacteriales; Mycobacteriaceae; Mycobacterium; Mycobacterium tuberculosis complex; Mycobacterium tuberculosis;	NCBI: 1773	4250714	99.61	0.00	Yes
NCBI Assembly	GCA_001394995.1	NCBI: cellular organisms; Bacteria; Terrabacteria group; Actinobacteria; Actinomycetia; Corynebacteriales; Mycobacteriaceae; Mycobacterium; Mycobacterium tuberculosis complex; Mycobacterium tuberculosis;	NCBI: 1773	4298654	99.94	0.00	Yes
NCBI Assembly	GCA_001445215.1	NCBI: cellular organisms; Bacteria; Terrabacteria group; Actinobacteria; Actinomycetia; Corynebacteriales; Mycobacteriaceae; Mycobacterium; Mycobacterium tuberculosis complex; Mycobacterium tuberculosis;	NCBI: 1773	4346054	99.94	0.00	Yes
NCBI Assembly	GCA_001566015.1	NCBI: cellular organisms; Bacteria; Terrabacteria group; Actinobacteria; Actinomycetia; Corynebacteriales; Mycobacteriaceae; Mycobacterium; Mycobacterium tuberculosis complex; Mycobacterium tuberculosis;	NCBI: 1773	4310030	99.94	0.00	Yes
NCBI Assembly	GCA_001386575.1	NCBI: cellular organisms; Bacteria; Terrabacteria group; Actinobacteria; Actinomycetia; Corynebacteriales; Mycobacteriaceae; Mycobacterium; Mycobacterium tuberculosis complex; Mycobacterium tuberculosis;	NCBI: 1773	4362514	99.94	0.00	Yes
NCBI Assembly	GCA_001383775.1	NCBI: cellular organisms; Bacteria; Terrabacteria group; Actinobacteria; Actinomycetia; Corynebacteriales; Mycobacteriaceae; Mycobacterium; Mycobacterium tuberculosis complex; Mycobacterium tuberculosis;	NCBI: 1773	4365771	99.94	0.00	Yes
NCBI Assembly	GCA_001393775.1	NCBI: cellular organisms; Bacteria; Terrabacteria group; Actinobacteria; Actinomycetia; Corynebacteriales; Mycobacteriaceae; Mycobacterium; Mycobacterium tuberculosis complex; Mycobacterium tuberculosis;	NCBI: 1773	4268339	99.78	0.00	Yes
NCBI Assembly	GCA_001389075.1	NCBI: cellular organisms; Bacteria; Terrabacteria group; Actinobacteria; Actinomycetia; Corynebacteriales; Mycobacteriaceae; Mycobacterium; Mycobacterium tuberculosis complex; Mycobacterium tuberculosis;	NCBI: 1773	4348697	99.94	0.00	Yes
NCBI Assembly	GCA_001414095.1	NCBI: cellular organisms; Bacteria; Terrabacteria group; Actinobacteria; Actinomycetia; Corynebacteriales; Mycobacteriaceae; Mycobacterium; unclassified Mycobacterium;	NCBI: 1638774	5226159	99.58	0.67	Yes
NCBI Assembly	GCA_001388455.1	NCBI: cellular organisms; Bacteria; Terrabacteria group; Actinobacteria; Actinomycetia; Corynebacteriales; Mycobacteriaceae; Mycobacterium; Mycobacterium tuberculosis complex; Mycobacterium tuberculosis;	NCBI: 1773	4355651	99.94	0.00	Yes
NCBI Assembly	GCA_001395015.1	NCBI: cellular organisms; Bacteria; Terrabacteria group; Actinobacteria; Actinomycetia; Corynebacteriales; Mycobacteriaceae; Mycobacterium; Mycobacterium tuberculosis complex; Mycobacterium tuberculosis;	NCBI: 1773	4327185	99.94	0.00	Yes
NCBI Assembly	GCA_001391575.1	NCBI: cellular organisms; Bacteria; Terrabacteria group; Actinobacteria; Actinomycetia; Corynebacteriales; Mycobacteriaceae; Mycobacterium; Mycobacterium tuberculosis complex; Mycobacterium tuberculosis;	NCBI: 1773	4310741	99.94	0.04	Yes
NCBI Assembly	GCA_001396575.1	NCBI: cellular organisms; Bacteria; Terrabacteria group; Actinobacteria; Actinomycetia; Corynebacteriales; Mycobacteriaceae; Mycobacterium; Mycobacterium tuberculosis complex; Mycobacterium tuberculosis;	NCBI: 1773	4288288	99.21	0.43	Yes
NCBI Assembly	GCA_001392855.1	NCBI: cellular organisms; Bacteria; Terrabacteria group; Actinobacteria; Actinomycetia; Corynebacteriales; Mycobacteriaceae; Mycobacterium; Mycobacterium tuberculosis complex; Mycobacterium tuberculosis;	NCBI: 1773	4336938	99.94	0.00	Yes
NCBI Assembly	GCA_001445235.1	NCBI: cellular organisms; Bacteria; Terrabacteria group; Actinobacteria; Actinomycetia; Corynebacteriales; Mycobacteriaceae; Mycobacterium; Mycobacterium tuberculosis complex; Mycobacterium tuberculosis;	NCBI: 1773	4440254	99.94	2.00	Yes
NCBI Assembly	GCA_001570425.1	NCBI: cellular organisms; Bacteria; Terrabacteria group; Actinobacteria; Actinomycetia; Corynebacteriales; Mycobacteriaceae; Mycolicibacterium; Mycolicibacterium brisbanense;	NCBI: 146020	7387494	100.00	2.77	Yes

NCBI Assembly	GCA_001386595.1	NCBI: cellular organisms; Bacteria; Terrabacteria group; Actinobacteria; Actinomycetia; Corynebacteriales; Mycobacteriaceae; Mycobacterium; Mycobacterium tuberculosis complex; Mycobacterium tuberculosis;	NCBI: 1773	4362123	99.94	0.00	Yes
NCBI Assembly	GCA_001383795.1	NCBI: cellular organisms; Bacteria; Terrabacteria group; Actinobacteria; Actinomycetia; Corynebacteriales; Mycobacteriaceae; Mycobacterium; Mycobacterium tuberculosis complex; Mycobacterium tuberculosis;	NCBI: 1773	4367089	99.94	0.00	Yes
NCBI Assembly	GCA_001388475.1	NCBI: cellular organisms; Bacteria; Terrabacteria group; Actinobacteria; Actinomycetia; Corynebacteriales; Mycobacteriaceae; Mycobacterium; Mycobacterium tuberculosis complex; Mycobacterium tuberculosis;	NCBI: 1773	4353032	99.54	0.00	Yes
NCBI Assembly	GCA_001389095.1	NCBI: cellular organisms; Bacteria; Terrabacteria group; Actinobacteria; Actinomycetia; Corynebacteriales; Mycobacteriaceae; Mycobacterium; Mycobacterium tuberculosis complex; Mycobacterium tuberculosis;	NCBI: 1773	4372678	99.94	0.50	Yes
NCBI Assembly	GCA_001391595.1	NCBI: cellular organisms; Bacteria; Terrabacteria group; Actinobacteria; Actinomycetia; Corynebacteriales; Mycobacteriaceae; Mycobacterium; Mycobacterium tuberculosis complex; Mycobacterium tuberculosis;	NCBI: 1773	4305630	99.94	0.00	Yes
NCBI Assembly	GCA_001393795.1	NCBI: cellular organisms; Bacteria; Terrabacteria group; Actinobacteria; Actinomycetia; Corynebacteriales; Mycobacteriaceae; Mycobacterium; Mycobacterium tuberculosis complex; Mycobacterium tuberculosis;	NCBI: 1773	4325389	99.78	0.00	Yes
NCBI Assembly	GCA_001417955.2	NCBI: cellular organisms; Bacteria; Terrabacteria group; Actinobacteria; Actinomycetia; Corynebacteriales; Mycobacteriaceae; Mycobacterium; Mycobacterium gordonae;	NCBI: 1778	7552315	100.00	2.34	Yes
NCBI Assembly	GCA_001396595.1	NCBI: cellular organisms; Bacteria; Terrabacteria group; Actinobacteria; Actinomycetia; Corynebacteriales; Mycobacteriaceae; Mycobacterium; Mycobacterium tuberculosis complex; Mycobacterium tuberculosis;	NCBI: 1773	4295600	99.28	0.00	Yes
NCBI Assembly	GCA_001395035.1	NCBI: cellular organisms; Bacteria; Terrabacteria group; Actinobacteria; Actinomycetia; Corynebacteriales; Mycobacteriaceae; Mycobacterium; Mycobacterium tuberculosis complex; Mycobacterium tuberculosis;	NCBI: 1773	4285274	99.83	0.00	Yes
NCBI Assembly	GCA_001392875.1	NCBI: cellular organisms; Bacteria; Terrabacteria group; Actinobacteria; Actinomycetia; Corynebacteriales; Mycobacteriaceae; Mycobacterium; Mycobacterium tuberculosis complex; Mycobacterium tuberculosis;	NCBI: 1773	4323571	99.89	0.00	Yes
NCBI Assembly	GCA_001445255.1	NCBI: cellular organisms; Bacteria; Terrabacteria group; Actinobacteria; Actinomycetia; Corynebacteriales; Mycobacteriaceae; Mycobacterium; Mycobacterium tuberculosis complex; Mycobacterium tuberculosis;	NCBI: 1773	4378675	99.94	1.00	Yes
NCBI Assembly	GCA_001570445.1	NCBI: cellular organisms; Bacteria; Terrabacteria group; Actinobacteria; Actinomycetia; Corynebacteriales; Mycobacteriaceae; Mycolicibacterium; Mycolicibacterium canariasense;	NCBI: 228230	6734610	99.89	2.14	Yes
NCBI Assembly	GCA_001388495.1	NCBI: cellular organisms; Bacteria; Terrabacteria group; Actinobacteria; Actinomycetia; Corynebacteriales; Mycobacteriaceae; Mycobacterium; Mycobacterium tuberculosis complex; Mycobacterium tuberculosis;	NCBI: 1773	4348091	99.94	0.00	Yes
NCBI Assembly	GCA_001383815.1	NCBI: cellular organisms; Bacteria; Terrabacteria group; Actinobacteria; Actinomycetia; Corynebacteriales; Mycobacteriaceae; Mycobacterium; Mycobacterium tuberculosis complex; Mycobacterium tuberculosis;	NCBI: 1773	4362799	99.94	0.00	Yes
NCBI Assembly	GCA_001386615.1	NCBI: cellular organisms; Bacteria; Terrabacteria group; Actinobacteria; Actinomycetia; Corynebacteriales; Mycobacteriaceae; Mycobacterium; Mycobacterium tuberculosis complex; Mycobacterium tuberculosis;	NCBI: 1773	4354763	99.94	0.00	Yes
NCBI Assembly	GCA_001389115.1	NCBI: cellular organisms; Bacteria; Terrabacteria group; Actinobacteria; Actinomycetia; Corynebacteriales; Mycobacteriaceae; Mycobacterium; Mycobacterium tuberculosis complex; Mycobacterium tuberculosis;	NCBI: 1773	4349361	99.94	0.00	Yes
NCBI Assembly	GCA_001396615.1	NCBI: cellular organisms; Bacteria; Terrabacteria group; Actinobacteria; Actinomycetia; Corynebacteriales; Mycobacteriaceae; Mycobacterium; Mycobacterium tuberculosis complex; Mycobacterium tuberculosis;	NCBI: 1773	4290430	99.84	0.00	Yes
NCBI Assembly	GCA_001395055.1	NCBI: cellular organisms; Bacteria; Terrabacteria group; Actinobacteria; Actinomycetia; Corynebacteriales; Mycobacteriaceae; Mycobacterium; Mycobacterium tuberculosis complex; Mycobacterium tuberculosis;	NCBI: 1773	4328015	99.61	0.00	Yes

NCBI Assembly	GCA_001392895.1	NCBI: cellular organisms; Bacteria; Terrabacteria group; Actinobacteria; Actinomycetia; Corynebacteriales; Mycobacteriaceae; Mycobacterium; Mycobacterium tuberculosis complex; Mycobacterium tuberculosis;	NCBI: 1773	4314014	99.94	0.07	Yes
NCBI Assembly	GCA_001393815.1	NCBI: cellular organisms; Bacteria; Terrabacteria group; Actinobacteria; Actinomycetia; Corynebacteriales; Mycobacteriaceae; Mycobacterium; Mycobacterium tuberculosis complex; Mycobacterium tuberculosis;	NCBI: 1773	4315196	99.94	0.04	Yes
NCBI Assembly	GCA_001391615.1	NCBI: cellular organisms; Bacteria; Terrabacteria group; Actinobacteria; Actinomycetia; Corynebacteriales; Mycobacteriaceae; Mycobacterium; Mycobacterium tuberculosis complex; Mycobacterium tuberculosis;	NCBI: 1773	4252512	99.06	0.00	Yes
NCBI Assembly	GCA_001421035.1	NCBI: cellular organisms; Bacteria; Terrabacteria group; Actinobacteria; Actinomycetia; Corynebacteriales; Mycobacteriaceae; Mycobacterium; Mycobacterium tuberculosis complex; Mycobacterium tuberculosis;	NCBI: 1773	4298162	99.94	0.00	Yes
NCBI Assembly	GCA_001445275.1	NCBI: cellular organisms; Bacteria; Terrabacteria group; Actinobacteria; Actinomycetia; Corynebacteriales; Mycobacteriaceae; Mycobacterium; Mycobacterium tuberculosis complex; Mycobacterium tuberculosis;	NCBI: 1773	4426810	99.91	1.97	Yes
NCBI Assembly	GCA_001570465.1	NCBI: cellular organisms; Bacteria; Terrabacteria group; Actinobacteria; Actinomycetia; Corynebacteriales; Mycobacteriaceae; Mycolicibacterium; Mycolicibacterium fortuitum; Mycolicibacterium fortuitum subsp. acetamidolyticum;	NCBI: 144550	7101918	99.43	0.69	Yes
NCBI Assembly	GCA_001383835.1	NCBI: cellular organisms; Bacteria; Terrabacteria group; Actinobacteria; Actinomycetia; Corynebacteriales; Mycobacteriaceae; Mycobacterium; Mycobacterium tuberculosis complex; Mycobacterium tuberculosis;	NCBI: 1773	4366658	99.94	0.00	Yes
NCBI Assembly	GCA_001388515.1	NCBI: cellular organisms; Bacteria; Terrabacteria group; Actinobacteria; Actinomycetia; Corynebacteriales; Mycobacteriaceae; Mycobacterium; Mycobacterium tuberculosis complex; Mycobacterium tuberculosis;	NCBI: 1773	4352909	99.54	0.00	Yes
NCBI Assembly	GCA_001392915.1	NCBI: cellular organisms; Bacteria; Terrabacteria group; Actinobacteria; Actinomycetia; Corynebacteriales; Mycobacteriaceae; Mycobacterium; Mycobacterium tuberculosis complex; Mycobacterium tuberculosis;	NCBI: 1773	4285402	99.94	0.00	Yes
NCBI Assembly	GCA_001389135.1	NCBI: cellular organisms; Bacteria; Terrabacteria group; Actinobacteria; Actinomycetia; Corynebacteriales; Mycobacteriaceae; Mycobacterium; Mycobacterium tuberculosis complex; Mycobacterium tuberculosis;	NCBI: 1773	4382926	99.91	0.00	Yes
NCBI Assembly	GCA_001386635.1	NCBI: cellular organisms; Bacteria; Terrabacteria group; Actinobacteria; Actinomycetia; Corynebacteriales; Mycobacteriaceae; Mycobacterium; Mycobacterium tuberculosis complex; Mycobacterium tuberculosis;	NCBI: 1773	4360853	99.94	0.00	Yes
NCBI Assembly	GCA_001395075.1	NCBI: cellular organisms; Bacteria; Terrabacteria group; Actinobacteria; Actinomycetia; Corynebacteriales; Mycobacteriaceae; Mycobacterium; Mycobacterium tuberculosis complex; Mycobacterium tuberculosis;	NCBI: 1773	4325592	99.94	0.00	Yes
NCBI Assembly	GCA_001396635.1	NCBI: cellular organisms; Bacteria; Terrabacteria group; Actinobacteria; Actinomycetia; Corynebacteriales; Mycobacteriaceae; Mycobacterium; Mycobacterium tuberculosis complex; Mycobacterium tuberculosis;	NCBI: 1773	4343174	99.78	0.00	Yes
NCBI Assembly	GCA_001426545.1	NCBI: cellular organisms; Bacteria; Terrabacteria group; Actinobacteria; Actinomycetia; Corynebacteriales; Mycobacteriaceae; Mycobacterium; unclassified Mycobacterium;	NCBI: 1736457	6501425	100.00	1.51	Yes
NCBI Assembly	GCA_001391635.1	NCBI: cellular organisms; Bacteria; Terrabacteria group; Actinobacteria; Actinomycetia; Corynebacteriales; Mycobacteriaceae; Mycobacterium; Mycobacterium tuberculosis complex; Mycobacterium tuberculosis;	NCBI: 1773	4303267	99.84	0.00	Yes
NCBI Assembly	GCA_001393835.1	NCBI: cellular organisms; Bacteria; Terrabacteria group; Actinobacteria; Actinomycetia; Corynebacteriales; Mycobacteriaceae; Mycobacterium; Mycobacterium tuberculosis complex; Mycobacterium tuberculosis;	NCBI: 1773	4747807	97.27	15.46	No
NCBI Assembly	GCA_001445295.1	NCBI: cellular organisms; Bacteria; Terrabacteria group; Actinobacteria; Actinomycetia; Corynebacteriales; Mycobacteriaceae; Mycobacterium; Mycobacterium tuberculosis complex; Mycobacterium tuberculosis;	NCBI: 1773	4361384	99.94	0.00	Yes
NCBI Assembly	GCA_001570485.1	NCBI: cellular organisms; Bacteria; Terrabacteria group; Actinobacteria; Actinomycetia; Corynebacteriales; Mycobacteriaceae; Mycolicibacterium; Mycolicibacterium novocastrense;	NCBI: 59813	6228220	100.00	0.61	Yes

NCBI Assembly	GCA_001383855.1	NCBI: cellular organisms; Bacteria; Terrabacteria group; Actinobacteria; Actinomycetia; Corynebacteriales; Mycobacteriaceae; Mycobacterium; Mycobacterium tuberculosis complex; Mycobacterium tuberculosis;	NCBI: 1773	4391526	99.94	0.00	Yes
NCBI Assembly	GCA_001388535.1	NCBI: cellular organisms; Bacteria; Terrabacteria group; Actinobacteria; Actinomycetia; Corynebacteriales; Mycobacteriaceae; Mycobacterium; Mycobacterium tuberculosis complex; Mycobacterium tuberculosis;	NCBI: 1773	4352061	99.94	0.00	Yes
NCBI Assembly	GCA_001395095.1	NCBI: cellular organisms; Bacteria; Terrabacteria group; Actinobacteria; Actinomycetia; Corynebacteriales; Mycobacteriaceae; Mycobacterium; Mycobacterium tuberculosis complex; Mycobacterium tuberculosis;	NCBI: 1773	4318421	99.94	0.00	Yes
NCBI Assembly	GCA_001392935.1	NCBI: cellular organisms; Bacteria; Terrabacteria group; Actinobacteria; Actinomycetia; Corynebacteriales; Mycobacteriaceae; Mycobacterium; Mycobacterium tuberculosis complex; Mycobacterium tuberculosis;	NCBI: 1773	4287866	99.94	0.00	Yes
NCBI Assembly	GCA_001386655.1	NCBI: cellular organisms; Bacteria; Terrabacteria group; Actinobacteria; Actinomycetia; Corynebacteriales; Mycobacteriaceae; Mycobacterium; Mycobacterium tuberculosis complex; Mycobacterium tuberculosis;	NCBI: 1773	4368361	99.94	0.00	Yes
NCBI Assembly	GCA_001428285.1	NCBI: cellular organisms; Bacteria; Terrabacteria group; Actinobacteria; Actinomycetia; Corynebacteriales; Mycobacteriaceae; Mycobacterium; unclassified Mycobacterium;	NCBI: 1736391	5747657	100.00	0.18	Yes
NCBI Assembly	GCA_001396655.1	NCBI: cellular organisms; Bacteria; Terrabacteria group; Actinobacteria; Actinomycetia; Corynebacteriales; Mycobacteriaceae; Mycobacterium; Mycobacterium tuberculosis complex; Mycobacterium tuberculosis;	NCBI: 1773	4348085	99.67	0.33	Yes
NCBI Assembly	GCA_001389155.1	NCBI: cellular organisms; Bacteria; Terrabacteria group; Actinobacteria; Actinomycetia; Corynebacteriales; Mycobacteriaceae; Mycobacterium; Mycobacterium tuberculosis complex; Mycobacterium tuberculosis;	NCBI: 1773	4358506	99.94	0.00	Yes
NCBI Assembly	GCA_001393855.1	NCBI: cellular organisms; Bacteria; Terrabacteria group; Actinobacteria; Actinomycetia; Corynebacteriales; Mycobacteriaceae; Mycobacterium; Mycobacterium tuberculosis complex; Mycobacterium tuberculosis;	NCBI: 1773	4305963	99.94	0.00	Yes
NCBI Assembly	GCA_001391655.1	NCBI: cellular organisms; Bacteria; Terrabacteria group; Actinobacteria; Actinomycetia; Corynebacteriales; Mycobacteriaceae; Mycobacterium; Mycobacterium tuberculosis complex; Mycobacterium tuberculosis;	NCBI: 1773	4338068	99.74	0.00	Yes
NCBI Assembly	GCA_001445315.1	NCBI: cellular organisms; Bacteria; Terrabacteria group; Actinobacteria; Actinomycetia; Corynebacteriales; Mycobacteriaceae; Mycobacterium; Mycobacterium tuberculosis complex; Mycobacterium tuberculosis;	NCBI: 1773	4414304	99.94	1.33	Yes
NCBI Assembly	GCA_001570505.1	NCBI: cellular organisms; Bacteria; Terrabacteria group; Actinobacteria; Actinomycetia; Corynebacteriales; Mycobacteriaceae; Mycolicibacterium; Mycolicibacterium thermoresistibile;	NCBI: 1797	4893136	99.32	0.00	Yes
NCBI Assembly	GCA_001383875.1	NCBI: cellular organisms; Bacteria; Terrabacteria group; Actinobacteria; Actinomycetia; Corynebacteriales; Mycobacteriaceae; Mycobacterium; Mycobacterium tuberculosis complex; Mycobacterium tuberculosis;	NCBI: 1773	4367243	99.94	0.00	Yes
NCBI Assembly	GCA_001388555.1	NCBI: cellular organisms; Bacteria; Terrabacteria group; Actinobacteria; Actinomycetia; Corynebacteriales; Mycobacteriaceae; Mycobacterium; Mycobacterium tuberculosis complex; Mycobacterium tuberculosis;	NCBI: 1773	4352490	99.94	0.00	Yes
NCBI Assembly	GCA_001395115.1	NCBI: cellular organisms; Bacteria; Terrabacteria group; Actinobacteria; Actinomycetia; Corynebacteriales; Mycobacteriaceae; Mycobacterium; Mycobacterium tuberculosis complex; Mycobacterium tuberculosis;	NCBI: 1773	4323748	99.94	0.00	Yes
NCBI Assembly	GCA_001392955.1	NCBI: cellular organisms; Bacteria; Terrabacteria group; Actinobacteria; Actinomycetia; Corynebacteriales; Mycobacteriaceae; Mycobacterium; Mycobacterium tuberculosis complex; Mycobacterium tuberculosis;	NCBI: 1773	4315272	99.94	0.00	Yes
NCBI Assembly	GCA_001428895.1	NCBI: cellular organisms; Bacteria; Terrabacteria group; Actinobacteria; Actinomycetia; Corynebacteriales; Mycobacteriaceae; Mycobacterium; unclassified Mycobacterium;	NCBI: 1736504	6672097	99.67	1.06	Yes
NCBI Assembly	GCA_001386675.1	NCBI: cellular organisms; Bacteria; Terrabacteria group; Actinobacteria; Actinomycetia; Corynebacteriales; Mycobacteriaceae; Mycobacterium; Mycobacterium tuberculosis complex; Mycobacterium tuberculosis;	NCBI: 1773	4362418	99.94	0.00	Yes

NCBI Assembly	GCA_001396675.1	NCBI: cellular organisms; Bacteria; Terrabacteria group; Actinobacteria; Actinomycetia; Corynebacteriales; Mycobacteriaceae; Mycobacterium; Mycobacterium tuberculosis complex; Mycobacterium tuberculosis;	NCBI: 1773	4315102	99.11	0.07	Yes
NCBI Assembly	GCA_001389175.1	NCBI: cellular organisms; Bacteria; Terrabacteria group; Actinobacteria; Actinomycetia; Corynebacteriales; Mycobacteriaceae; Mycobacterium; Mycobacterium tuberculosis complex; Mycobacterium tuberculosis;	NCBI: 1773	4358394	99.94	0.00	Yes
NCBI Assembly	GCA_001391675.1	NCBI: cellular organisms; Bacteria; Terrabacteria group; Actinobacteria; Actinomycetia; Corynebacteriales; Mycobacteriaceae; Mycobacterium; Mycobacterium tuberculosis complex; Mycobacterium tuberculosis;	NCBI: 1773	4323140	99.94	0.00	Yes
NCBI Assembly	GCA_001393875.1	NCBI: cellular organisms; Bacteria; Terrabacteria group; Actinobacteria; Actinomycetia; Corynebacteriales; Mycobacteriaceae; Mycobacterium; Mycobacterium tuberculosis complex; Mycobacterium tuberculosis;	NCBI: 1773	4298489	99.94	0.15	Yes
NCBI Assembly	GCA_001445335.1	NCBI: cellular organisms; Bacteria; Terrabacteria group; Actinobacteria; Actinomycetia; Corynebacteriales; Mycobacteriaceae; Mycobacterium; Mycobacterium tuberculosis complex; Mycobacterium tuberculosis;	NCBI: 1773	4355477	99.94	0.00	Yes
NCBI Assembly	GCA_001573045.1	NCBI: cellular organisms; Bacteria; Terrabacteria group; Actinobacteria; Actinomycetia; Corynebacteriales; Mycobacteriaceae; Mycobacterium; Mycobacterium tuberculosis complex; Mycobacterium tuberculosis;	NCBI: 1773	4329127	99.94	0.33	Yes
NCBI Assembly	GCA_001383895.1	NCBI: cellular organisms; Bacteria; Terrabacteria group; Actinobacteria; Actinomycetia; Corynebacteriales; Mycobacteriaceae; Mycobacterium; Mycobacterium tuberculosis complex; Mycobacterium tuberculosis;	NCBI: 1773	4368370	99.94	0.00	Yes
NCBI Assembly	GCA_001439455.1	NCBI: cellular organisms; Bacteria; Terrabacteria group; Actinobacteria; Actinomycetia; Corynebacteriales; Mycobacteriaceae; Mycobacterium; Mycobacterium tuberculosis complex; Mycobacterium tuberculosis;	NCBI: 1350393	4313998	98.83	0.00	Yes
NCBI Assembly	GCA_001386695.1	NCBI: cellular organisms; Bacteria; Terrabacteria group; Actinobacteria; Actinomycetia; Corynebacteriales; Mycobacteriaceae; Mycobacterium; Mycobacterium tuberculosis complex; Mycobacterium tuberculosis;	NCBI: 1773	4354642	99.61	0.00	Yes
NCBI Assembly	GCA_001388575.1	NCBI: cellular organisms; Bacteria; Terrabacteria group; Actinobacteria; Actinomycetia; Corynebacteriales; Mycobacteriaceae; Mycobacterium; Mycobacterium tuberculosis complex; Mycobacterium tuberculosis;	NCBI: 1773	4352946	99.94	0.00	Yes
NCBI Assembly	GCA_001392975.1	NCBI: cellular organisms; Bacteria; Terrabacteria group; Actinobacteria; Actinomycetia; Corynebacteriales; Mycobacteriaceae; Mycobacterium; Mycobacterium tuberculosis complex; Mycobacterium tuberculosis;	NCBI: 1773	4306906	99.94	0.00	Yes
NCBI Assembly	GCA_001396695.1	NCBI: cellular organisms; Bacteria; Terrabacteria group; Actinobacteria; Actinomycetia; Corynebacteriales; Mycobacteriaceae; Mycobacterium; Mycobacterium tuberculosis complex; Mycobacterium tuberculosis;	NCBI: 1773	4321382	99.77	0.00	Yes
NCBI Assembly	GCA_001389195.1	NCBI: cellular organisms; Bacteria; Terrabacteria group; Actinobacteria; Actinomycetia; Corynebacteriales; Mycobacteriaceae; Mycobacterium; Mycobacterium tuberculosis complex; Mycobacterium tuberculosis;	NCBI: 1773	4358081	99.94	0.00	Yes
NCBI Assembly	GCA_001395135.1	NCBI: cellular organisms; Bacteria; Terrabacteria group; Actinobacteria; Actinomycetia; Corynebacteriales; Mycobacteriaceae; Mycobacterium; Mycobacterium tuberculosis complex; Mycobacterium tuberculosis;	NCBI: 1773	4318897	99.94	0.00	Yes
NCBI Assembly	GCA_001391695.1	NCBI: cellular organisms; Bacteria; Terrabacteria group; Actinobacteria; Actinomycetia; Corynebacteriales; Mycobacteriaceae; Mycobacterium; Mycobacterium tuberculosis complex; Mycobacterium tuberculosis;	NCBI: 1773	4259083	99.78	0.00	Yes
NCBI Assembly	GCA_001393895.1	NCBI: cellular organisms; Bacteria; Terrabacteria group; Actinobacteria; Actinomycetia; Corynebacteriales; Mycobacteriaceae; Mycobacterium; Mycobacterium tuberculosis complex; Mycobacterium tuberculosis;	NCBI: 1773	4294003	99.94	0.00	Yes
NCBI Assembly	GCA_001445345.1	NCBI: cellular organisms; Bacteria; Terrabacteria group; Actinobacteria; Actinomycetia; Corynebacteriales; Mycobacteriaceae; Mycobacterium; Mycobacterium tuberculosis complex; Mycobacterium tuberculosis;	NCBI: 1773	4358565	99.94	0.00	Yes
NCBI Assembly	GCA_001581995.1	NCBI: cellular organisms; Bacteria; Terrabacteria group; Actinobacteria; Actinomycetia; Corynebacteriales; Mycobacteriaceae; Mycolicibacterium; Mycolicibacterium phlei;	NCBI: 1335621	5311375	100.00	0.15	Yes

NCBI Assembly	GCA_001439515.1	NCBI: cellular organisms; Bacteria; Terrabacteria group; Actinobacteria; Actinomycetia; Corynebacteriales; Mycobacteriaceae; Mycobacterium; Mycobacterium kyorinense;	NCBI: 487514	5355645	100.00	0.24	Yes
NCBI Assembly	GCA_001383915.1	NCBI: cellular organisms; Bacteria; Terrabacteria group; Actinobacteria; Actinomycetia; Corynebacteriales; Mycobacteriaceae; Mycobacterium; Mycobacterium tuberculosis complex; Mycobacterium tuberculosis;	NCBI: 1773	4367232	99.94	0.00	Yes
NCBI Assembly	GCA_001386715.1	NCBI: cellular organisms; Bacteria; Terrabacteria group; Actinobacteria; Actinomycetia; Corynebacteriales; Mycobacteriaceae; Mycobacterium; Mycobacterium tuberculosis complex; Mycobacterium tuberculosis;	NCBI: 1773	4354176	99.94	0.00	Yes
NCBI Assembly	GCA_001388595.1	NCBI: cellular organisms; Bacteria; Terrabacteria group; Actinobacteria; Actinomycetia; Corynebacteriales; Mycobacteriaceae; Mycobacterium; Mycobacterium tuberculosis complex; Mycobacterium tuberculosis;	NCBI: 1773	4366808	99.94	0.00	Yes
NCBI Assembly	GCA_001396715.1	NCBI: cellular organisms; Bacteria; Terrabacteria group; Actinobacteria; Actinomycetia; Corynebacteriales; Mycobacteriaceae; Mycobacterium; Mycobacterium tuberculosis complex; Mycobacterium tuberculosis;	NCBI: 1773	4330905	99.78	0.00	Yes
NCBI Assembly	GCA_001395155.1	NCBI: cellular organisms; Bacteria; Terrabacteria group; Actinobacteria; Actinomycetia; Corynebacteriales; Mycobacteriaceae; Mycobacterium; Mycobacterium tuberculosis complex; Mycobacterium tuberculosis;	NCBI: 1773	4332814	99.86	0.00	Yes
NCBI Assembly	GCA_001392995.1	NCBI: cellular organisms; Bacteria; Terrabacteria group; Actinobacteria; Actinomycetia; Corynebacteriales; Mycobacteriaceae; Mycobacterium; Mycobacterium tuberculosis complex; Mycobacterium tuberculosis;	NCBI: 1773	5044771	98.28	28.13	No
NCBI Assembly	GCA_001389215.1	NCBI: cellular organisms; Bacteria; Terrabacteria group; Actinobacteria; Actinomycetia; Corynebacteriales; Mycobacteriaceae; Mycobacterium; Mycobacterium tuberculosis complex; Mycobacterium tuberculosis;	NCBI: 1773	4387146	99.94	0.00	Yes
NCBI Assembly	GCA_001391715.1	NCBI: cellular organisms; Bacteria; Terrabacteria group; Actinobacteria; Actinomycetia; Corynebacteriales; Mycobacteriaceae; Mycobacterium; Mycobacterium tuberculosis complex; Mycobacterium tuberculosis;	NCBI: 1773	4317504	99.94	0.15	Yes
NCBI Assembly	GCA_001393915.1	NCBI: cellular organisms; Bacteria; Terrabacteria group; Actinobacteria; Actinomycetia; Corynebacteriales; Mycobacteriaceae; Mycobacterium; Mycobacterium tuberculosis complex; Mycobacterium tuberculosis;	NCBI: 1773	4601240	96.38	12.32	No
NCBI Assembly	GCA_001445375.1	NCBI: cellular organisms; Bacteria; Terrabacteria group; Actinobacteria; Actinomycetia; Corynebacteriales; Mycobacteriaceae; Mycobacterium; Mycobacterium tuberculosis complex; Mycobacterium tuberculosis;	NCBI: 1773	4403816	99.94	2.03	Yes
NCBI Assembly	GCA_001582005.1	NCBI: cellular organisms; Bacteria; Terrabacteria group; Actinobacteria; Actinomycetia; Corynebacteriales; Mycobacteriaceae; Mycolicibacterium; Mycolicibacterium phlei;	NCBI: 1335620	5304064	100.00	0.15	Yes
NCBI Assembly	GCA_001383935.1	NCBI: cellular organisms; Bacteria; Terrabacteria group; Actinobacteria; Actinomycetia; Corynebacteriales; Mycobacteriaceae; Mycobacterium; Mycobacterium tuberculosis complex; Mycobacterium tuberculosis;	NCBI: 1773	4369475	99.94	0.17	Yes
NCBI Assembly	GCA_001388615.1	NCBI: cellular organisms; Bacteria; Terrabacteria group; Actinobacteria; Actinomycetia; Corynebacteriales; Mycobacteriaceae; Mycobacterium; Mycobacterium tuberculosis complex; Mycobacterium tuberculosis;	NCBI: 1773	4361109	99.94	0.00	Yes
NCBI Assembly	GCA_001386735.1	NCBI: cellular organisms; Bacteria; Terrabacteria group; Actinobacteria; Actinomycetia; Corynebacteriales; Mycobacteriaceae; Mycobacterium; Mycobacterium tuberculosis complex; Mycobacterium tuberculosis;	NCBI: 1773	4357234	99.94	0.00	Yes
NCBI Assembly	GCA_001440005.1	NCBI: cellular organisms; Bacteria; Terrabacteria group; Actinobacteria; Actinomycetia; Corynebacteriales; Mycobacteriaceae; Mycobacteroides; unclassified Mycobacteroides;	NCBI: 1720565	5450353	99.83	0.90	Yes
NCBI Assembly	GCA_001393015.1	NCBI: cellular organisms; Bacteria; Terrabacteria group; Actinobacteria; Actinomycetia; Corynebacteriales; Mycobacteriaceae; Mycobacterium; Mycobacterium tuberculosis complex; Mycobacterium tuberculosis;	NCBI: 1773	4300182	99.94	0.00	Yes
NCBI Assembly	GCA_001389235.1	NCBI: cellular organisms; Bacteria; Terrabacteria group; Actinobacteria; Actinomycetia; Corynebacteriales; Mycobacteriaceae; Mycobacterium; Mycobacterium tuberculosis complex; Mycobacterium tuberculosis;	NCBI: 1773	4351615	99.61	0.00	Yes

NCBI Assembly	GCA_001396735.1	NCBI: cellular organisms; Bacteria; Terrabacteria group; Actinobacteria; Actinomycetia; Corynebacteriales; Mycobacteriaceae; Mycobacterium; Mycobacterium tuberculosis complex; Mycobacterium tuberculosis;	NCBI: 1773	4303188	99.94	0.00	Yes
NCBI Assembly	GCA_001395175.1	NCBI: cellular organisms; Bacteria; Terrabacteria group; Actinobacteria; Actinomycetia; Corynebacteriales; Mycobacteriaceae; Mycobacterium; Mycobacterium tuberculosis complex; Mycobacterium tuberculosis;	NCBI: 1773	4340197	99.94	0.00	Yes
NCBI Assembly	GCA_001391735.1	NCBI: cellular organisms; Bacteria; Terrabacteria group; Actinobacteria; Actinomycetia; Corynebacteriales; Mycobacteriaceae; Mycobacterium; Mycobacterium tuberculosis complex; Mycobacterium tuberculosis;	NCBI: 1773	4298888	99.50	0.00	Yes
NCBI Assembly	GCA_001393935.1	NCBI: cellular organisms; Bacteria; Terrabacteria group; Actinobacteria; Actinomycetia; Corynebacteriales; Mycobacteriaceae; Mycobacterium; Mycobacterium tuberculosis complex; Mycobacterium tuberculosis;	NCBI: 1773	4276700	99.94	0.00	Yes
NCBI Assembly	GCA_001445395.1	NCBI: cellular organisms; Bacteria; Terrabacteria group; Actinobacteria; Actinomycetia; Corynebacteriales; Mycobacteriaceae; Mycobacterium; Mycobacterium tuberculosis complex; Mycobacterium tuberculosis;	NCBI: 1773	4510327	99.94	1.00	Yes
NCBI Assembly	GCA_001582015.1	NCBI: cellular organisms; Bacteria; Terrabacteria group; Actinobacteria; Actinomycetia; Corynebacteriales; Mycobacteriaceae; Mycolicibacterium; Mycolicibacterium phlei;	NCBI: 1226750	5322335	100.00	0.15	Yes
NCBI Assembly	GCA_001386755.1	NCBI: cellular organisms; Bacteria; Terrabacteria group; Actinobacteria; Actinomycetia; Corynebacteriales; Mycobacteriaceae; Mycobacterium; Mycobacterium tuberculosis complex; Mycobacterium tuberculosis;	NCBI: 1773	4358133	99.94	0.00	Yes
NCBI Assembly	GCA_001440085.1	NCBI: cellular organisms; Bacteria; Terrabacteria group; Actinobacteria; Actinomycetia; Corynebacteriales; Mycobacteriaceae; Mycobacteroides; unclassified Mycobacteroides;	NCBI: 1720566	5421590	99.83	0.85	Yes
NCBI Assembly	GCA_001383955.1	NCBI: cellular organisms; Bacteria; Terrabacteria group; Actinobacteria; Actinomycetia; Corynebacteriales; Mycobacteriaceae; Mycobacterium; Mycobacterium tuberculosis complex; Mycobacterium tuberculosis;	NCBI: 1773	4366993	99.94	0.00	Yes
NCBI Assembly	GCA_001388635.1	NCBI: cellular organisms; Bacteria; Terrabacteria group; Actinobacteria; Actinomycetia; Corynebacteriales; Mycobacteriaceae; Mycobacterium; Mycobacterium tuberculosis complex; Mycobacterium tuberculosis;	NCBI: 1773	4376462	99.94	0.00	Yes
NCBI Assembly	GCA_001389255.1	NCBI: cellular organisms; Bacteria; Terrabacteria group; Actinobacteria; Actinomycetia; Corynebacteriales; Mycobacteriaceae; Mycobacterium; Mycobacterium tuberculosis complex; Mycobacterium tuberculosis;	NCBI: 1773	4356702	99.94	0.07	Yes
NCBI Assembly	GCA_001393035.1	NCBI: cellular organisms; Bacteria; Terrabacteria group; Actinobacteria; Actinomycetia; Corynebacteriales; Mycobacteriaceae; Mycobacterium; Mycobacterium tuberculosis complex; Mycobacterium tuberculosis;	NCBI: 1773	4320792	99.94	0.00	Yes
NCBI Assembly	GCA_001396755.1	NCBI: cellular organisms; Bacteria; Terrabacteria group; Actinobacteria; Actinomycetia; Corynebacteriales; Mycobacteriaceae; Mycobacterium; Mycobacterium tuberculosis complex; Mycobacterium tuberculosis;	NCBI: 1773	4294543	99.84	0.00	Yes
NCBI Assembly	GCA_001395195.1	NCBI: cellular organisms; Bacteria; Terrabacteria group; Actinobacteria; Actinomycetia; Corynebacteriales; Mycobacteriaceae; Mycobacterium; Mycobacterium tuberculosis complex; Mycobacterium tuberculosis;	NCBI: 1773	4317431	99.94	0.00	Yes
NCBI Assembly	GCA_001393955.1	NCBI: cellular organisms; Bacteria; Terrabacteria group; Actinobacteria; Actinomycetia; Corynebacteriales; Mycobacteriaceae; Mycobacterium; Mycobacterium tuberculosis complex; Mycobacterium tuberculosis;	NCBI: 1773	4322704	99.94	0.00	Yes
NCBI Assembly	GCA_001391755.1	NCBI: cellular organisms; Bacteria; Terrabacteria group; Actinobacteria; Actinomycetia; Corynebacteriales; Mycobacteriaceae; Mycobacterium; Mycobacterium tuberculosis complex; Mycobacterium tuberculosis;	NCBI: 1773	4344576	99.94	0.00	Yes
NCBI Assembly	GCA_001445415.1	NCBI: cellular organisms; Bacteria; Terrabacteria group; Actinobacteria; Actinomycetia; Corynebacteriales; Mycobacteriaceae; Mycobacterium; Mycobacterium tuberculosis complex; Mycobacterium tuberculosis;	NCBI: 1773	4392778	99.45	1.50	Yes
NCBI Assembly	GCA_001582025.1	NCBI: cellular organisms; Bacteria; Terrabacteria group; Actinobacteria; Actinomycetia; Corynebacteriales; Mycobacteriaceae; Mycolicibacterium; Mycolicibacterium phlei;	NCBI: 1384515	5306822	100.00	0.15	Yes

NCBI Assembly	GCA_001386775.1	NCBI: cellular organisms; Bacteria; Terrabacteria group; Actinobacteria; Actinomycetia; Corynebacteriales; Mycobacteriaceae; Mycobacterium; Mycobacterium tuberculosis complex; Mycobacterium tuberculosis;	NCBI: 1773	4339676	99.94	0.00	Yes
NCBI Assembly	GCA_001383975.1	NCBI: cellular organisms; Bacteria; Terrabacteria group; Actinobacteria; Actinomycetia; Corynebacteriales; Mycobacteriaceae; Mycobacterium; Mycobacterium tuberculosis complex; Mycobacterium tuberculosis;	NCBI: 1773	4367287	99.94	0.00	Yes
NCBI Assembly	GCA_001393055.1	NCBI: cellular organisms; Bacteria; Terrabacteria group; Actinobacteria; Actinomycetia; Corynebacteriales; Mycobacteriaceae; Mycobacterium; Mycobacterium tuberculosis complex; Mycobacterium tuberculosis;	NCBI: 1773	4333618	99.94	0.07	Yes
NCBI Assembly	GCA_001389275.1	NCBI: cellular organisms; Bacteria; Terrabacteria group; Actinobacteria; Actinomycetia; Corynebacteriales; Mycobacteriaceae; Mycobacterium; Mycobacterium tuberculosis complex; Mycobacterium tuberculosis;	NCBI: 1773	4355895	99.94	0.00	Yes
NCBI Assembly	GCA_001440105.1	NCBI: cellular organisms; Bacteria; Terrabacteria group; Actinobacteria; Actinomycetia; Corynebacteriales; Mycobacteriaceae; Mycobacteroides; unclassified Mycobacteroides;	NCBI: 1720568	5449380	99.83	0.90	Yes
NCBI Assembly	GCA_001388655.1	NCBI: cellular organisms; Bacteria; Terrabacteria group; Actinobacteria; Actinomycetia; Corynebacteriales; Mycobacteriaceae; Mycobacterium; Mycobacterium tuberculosis complex; Mycobacterium tuberculosis;	NCBI: 1773	4360347	99.94	0.00	Yes
NCBI Assembly	GCA_001396775.1	NCBI: cellular organisms; Bacteria; Terrabacteria group; Actinobacteria; Actinomycetia; Corynebacteriales; Mycobacteriaceae; Mycobacterium; Mycobacterium tuberculosis complex; Mycobacterium tuberculosis;	NCBI: 1773	4303564	99.28	0.00	Yes
NCBI Assembly	GCA_001395215.1	NCBI: cellular organisms; Bacteria; Terrabacteria group; Actinobacteria; Actinomycetia; Corynebacteriales; Mycobacteriaceae; Mycobacterium; Mycobacterium tuberculosis complex; Mycobacterium tuberculosis;	NCBI: 1773	4307785	99.86	0.00	Yes
NCBI Assembly	GCA_001393975.1	NCBI: cellular organisms; Bacteria; Terrabacteria group; Actinobacteria; Actinomycetia; Corynebacteriales; Mycobacteriaceae; Mycobacterium; Mycobacterium tuberculosis complex; Mycobacterium tuberculosis;	NCBI: 1773	4299138	99.81	0.00	Yes
NCBI Assembly	GCA_001391775.1	NCBI: cellular organisms; Bacteria; Terrabacteria group; Actinobacteria; Actinomycetia; Corynebacteriales; Mycobacteriaceae; Mycobacterium; Mycobacterium tuberculosis complex; Mycobacterium tuberculosis;	NCBI: 1773	4331877	99.75	0.00	Yes
NCBI Assembly	GCA_001445435.1	NCBI: cellular organisms; Bacteria; Terrabacteria group; Actinobacteria; Actinomycetia; Corynebacteriales; Mycobacteriaceae; Mycobacterium; Mycobacterium tuberculosis complex; Mycobacterium tuberculosis;	NCBI: 1773	4362225	99.94	0.67	Yes
NCBI Assembly	GCA_001583415.1	NCBI: cellular organisms; Bacteria; Terrabacteria group; Actinobacteria; Actinomycetia; Corynebacteriales; Mycobacteriaceae; Mycolicibacterium; Mycolicibacterium phlei;	NCBI: 1771	5349645	100.00	0.15	Yes
NCBI Assembly	GCA_001386795.1	NCBI: cellular organisms; Bacteria; Terrabacteria group; Actinobacteria; Actinomycetia; Corynebacteriales; Mycobacteriaceae; Mycobacterium; Mycobacterium tuberculosis complex; Mycobacterium tuberculosis;	NCBI: 1773	4362563	99.88	0.00	Yes
NCBI Assembly	GCA_001383995.1	NCBI: cellular organisms; Bacteria; Terrabacteria group; Actinobacteria; Actinomycetia; Corynebacteriales; Mycobacteriaceae; Mycobacterium; Mycobacterium tuberculosis complex; Mycobacterium tuberculosis;	NCBI: 1773	4368741	99.94	0.17	Yes
NCBI Assembly	GCA_001389295.1	NCBI: cellular organisms; Bacteria; Terrabacteria group; Actinobacteria; Actinomycetia; Corynebacteriales; Mycobacteriaceae; Mycobacterium; Mycobacterium tuberculosis complex; Mycobacterium tuberculosis;	NCBI: 1773	4361740	99.69	0.04	Yes
NCBI Assembly	GCA_001440125.1	NCBI: cellular organisms; Bacteria; Terrabacteria group; Actinobacteria; Actinomycetia; Corynebacteriales; Mycobacteriaceae; Mycobacteroides; unclassified Mycobacteroides;	NCBI: 1720567	5423024	99.83	0.85	Yes
NCBI Assembly	GCA_001393075.1	NCBI: cellular organisms; Bacteria; Terrabacteria group; Actinobacteria; Actinomycetia; Corynebacteriales; Mycobacteriaceae; Mycobacterium; Mycobacterium tuberculosis complex; Mycobacterium tuberculosis;	NCBI: 1773	4321052	99.94	0.00	Yes
NCBI Assembly	GCA_001396795.1	NCBI: cellular organisms; Bacteria; Terrabacteria group; Actinobacteria; Actinomycetia; Corynebacteriales; Mycobacteriaceae; Mycobacterium; Mycobacterium tuberculosis complex; Mycobacterium tuberculosis;	NCBI: 1773	4331493	99.50	0.00	Yes

NCBI Assembly	GCA_001395235.1	NCBI: cellular organisms; Bacteria; Terrabacteria group; Actinobacteria; Actinomycetia; Corynebacteriales; Mycobacteriaceae; Mycobacterium; Mycobacterium tuberculosis complex; Mycobacterium tuberculosis;	NCBI: 1773	4332043	99.88	0.15	Yes
NCBI Assembly	GCA_001388675.1	NCBI: cellular organisms; Bacteria; Terrabacteria group; Actinobacteria; Actinomycetia; Corynebacteriales; Mycobacteriaceae; Mycobacterium; Mycobacterium tuberculosis complex; Mycobacterium tuberculosis;	NCBI: 1773	4365957	99.94	0.00	Yes
NCBI Assembly	GCA_001393995.1	NCBI: cellular organisms; Bacteria; Terrabacteria group; Actinobacteria; Actinomycetia; Corynebacteriales; Mycobacteriaceae; Mycobacterium; Mycobacterium tuberculosis complex; Mycobacterium tuberculosis;	NCBI: 1773	4290365	99.94	0.00	Yes
NCBI Assembly	GCA_001391795.1	NCBI: cellular organisms; Bacteria; Terrabacteria group; Actinobacteria; Actinomycetia; Corynebacteriales; Mycobacteriaceae; Mycobacterium; Mycobacterium tuberculosis complex; Mycobacterium tuberculosis;	NCBI: 1773	4239158	99.92	0.00	Yes
NCBI Assembly	GCA_001445445.1	NCBI: cellular organisms; Bacteria; Terrabacteria group; Actinobacteria; Actinomycetia; Corynebacteriales; Mycobacteriaceae; Mycobacterium; Mycobacterium tuberculosis complex; Mycobacterium tuberculosis;	NCBI: 1773	4391872	99.94	0.00	Yes
NCBI Assembly	GCA_001583545.1	NCBI: cellular organisms; Bacteria; Terrabacteria group; Actinobacteria; Actinomycetia; Corynebacteriales; Mycobacteriaceae; Mycobacterium; Mycobacterium avium complex (MAC); Mycobacterium avium; Mycobacterium avium subsp. hominissuis;	NCBI: 439334	5158387	99.94	0.04	Yes
NCBI Assembly	GCA_001384015.1	NCBI: cellular organisms; Bacteria; Terrabacteria group; Actinobacteria; Actinomycetia; Corynebacteriales; Mycobacteriaceae; Mycobacterium; Mycobacterium tuberculosis complex; Mycobacterium tuberculosis;	NCBI: 1773	4365824	99.94	0.17	Yes
NCBI Assembly	GCA_001393095.1	NCBI: cellular organisms; Bacteria; Terrabacteria group; Actinobacteria; Actinomycetia; Corynebacteriales; Mycobacteriaceae; Mycobacterium; Mycobacterium tuberculosis complex; Mycobacterium tuberculosis;	NCBI: 1773	4290529	99.94	0.00	Yes
NCBI Assembly	GCA_001386815.1	NCBI: cellular organisms; Bacteria; Terrabacteria group; Actinobacteria; Actinomycetia; Corynebacteriales; Mycobacteriaceae; Mycobacterium; Mycobacterium tuberculosis complex; Mycobacterium tuberculosis;	NCBI: 1773	4348944	99.61	0.00	Yes
NCBI Assembly	GCA_001389315.1	NCBI: cellular organisms; Bacteria; Terrabacteria group; Actinobacteria; Actinomycetia; Corynebacteriales; Mycobacteriaceae; Mycobacterium; Mycobacterium tuberculosis complex; Mycobacterium tuberculosis;	NCBI: 1773	4386561	99.78	0.00	Yes
NCBI Assembly	GCA_001440135.1	NCBI: cellular organisms; Bacteria; Terrabacteria group; Actinobacteria; Actinomycetia; Corynebacteriales; Mycobacteriaceae; Mycobacteroides; unclassified Mycobacteroides;	NCBI: 1720569	5588459	99.44	0.39	Yes
NCBI Assembly	GCA_001388695.1	NCBI: cellular organisms; Bacteria; Terrabacteria group; Actinobacteria; Actinomycetia; Corynebacteriales; Mycobacteriaceae; Mycobacterium; Mycobacterium tuberculosis complex; Mycobacterium tuberculosis;	NCBI: 1773	4349016	99.94	0.00	Yes
NCBI Assembly	GCA_001396815.1	NCBI: cellular organisms; Bacteria; Terrabacteria group; Actinobacteria; Actinomycetia; Corynebacteriales; Mycobacteriaceae; Mycobacterium; Mycobacterium tuberculosis complex; Mycobacterium tuberculosis;	NCBI: 1773	4330251	99.69	0.00	Yes
NCBI Assembly	GCA_001395255.1	NCBI: cellular organisms; Bacteria; Terrabacteria group; Actinobacteria; Actinomycetia; Corynebacteriales; Mycobacteriaceae; Mycobacterium; Mycobacterium tuberculosis complex; Mycobacterium tuberculosis;	NCBI: 1773	4317618	99.94	0.00	Yes
NCBI Assembly	GCA_001394015.1	NCBI: cellular organisms; Bacteria; Terrabacteria group; Actinobacteria; Actinomycetia; Corynebacteriales; Mycobacteriaceae; Mycobacterium; Mycobacterium tuberculosis complex; Mycobacterium tuberculosis;	NCBI: 1773	4279197	99.61	0.00	Yes
NCBI Assembly	GCA_001391815.1	NCBI: cellular organisms; Bacteria; Terrabacteria group; Actinobacteria; Actinomycetia; Corynebacteriales; Mycobacteriaceae; Mycobacterium; Mycobacterium tuberculosis complex; Mycobacterium tuberculosis;	NCBI: 1773	4319222	99.94	0.00	Yes
NCBI Assembly	GCA_001445455.1	NCBI: cellular organisms; Bacteria; Terrabacteria group; Actinobacteria; Actinomycetia; Corynebacteriales; Mycobacteriaceae; Mycobacterium; Mycobacterium tuberculosis complex; Mycobacterium tuberculosis;	NCBI: 1773	4375657	99.94	0.00	Yes
NCBI Assembly	GCA_001584745.1	NCBI: cellular organisms; Bacteria; Terrabacteria group; Actinobacteria; Actinomycetia; Corynebacteriales; Mycobacteriaceae; Mycobacterium; Mycobacterium tuberculosis complex; Mycobacterium tuberculosis;	NCBI: 1773	4433130	99.44	0.00	Yes

NCBI Assembly	GCA_001384035.1	NCBI: cellular organisms; Bacteria; Terrabacteria group; Actinobacteria; Actinomycetia; Corynebacteriales; Mycobacteriaceae; Mycobacterium; Mycobacterium tuberculosis complex; Mycobacterium tuberculosis;	NCBI: 1773	4368080	99.94	0.00	Yes
NCBI Assembly	GCA_001388115.1	NCBI: cellular organisms; Bacteria; Terrabacteria group; Actinobacteria; Actinomycetia; Corynebacteriales; Mycobacteriaceae; Mycobacterium; Mycobacterium tuberculosis complex; Mycobacterium tuberculosis;	NCBI: 1773	4345560	99.94	0.00	Yes
NCBI Assembly	GCA_001388715.1	NCBI: cellular organisms; Bacteria; Terrabacteria group; Actinobacteria; Actinomycetia; Corynebacteriales; Mycobacteriaceae; Mycobacterium; Mycobacterium tuberculosis complex; Mycobacterium tuberculosis;	NCBI: 1773	4351885	99.61	0.00	Yes
NCBI Assembly	GCA_001386835.1	NCBI: cellular organisms; Bacteria; Terrabacteria group; Actinobacteria; Actinomycetia; Corynebacteriales; Mycobacteriaceae; Mycobacterium; Mycobacterium tuberculosis complex; Mycobacterium tuberculosis;	NCBI: 1773	4346557	99.94	0.00	Yes
NCBI Assembly	GCA_001440155.1	NCBI: cellular organisms; Bacteria; Terrabacteria group; Actinobacteria; Actinomycetia; Corynebacteriales; Mycobacteriaceae; Mycobacteroides; unclassified Mycobacteroides;	NCBI: 1720570	5739247	99.44	0.39	Yes
NCBI Assembly	GCA_001389335.1	NCBI: cellular organisms; Bacteria; Terrabacteria group; Actinobacteria; Actinomycetia; Corynebacteriales; Mycobacteriaceae; Mycobacterium; Mycobacterium tuberculosis complex; Mycobacterium tuberculosis;	NCBI: 1773	4363737	99.94	0.00	Yes
NCBI Assembly	GCA_001396835.1	NCBI: cellular organisms; Bacteria; Terrabacteria group; Actinobacteria; Actinomycetia; Corynebacteriales; Mycobacteriaceae; Mycobacterium; Mycobacterium tuberculosis complex; Mycobacterium tuberculosis;	NCBI: 1773	4337021	99.83	0.00	Yes
NCBI Assembly	GCA_001395275.1	NCBI: cellular organisms; Bacteria; Terrabacteria group; Actinobacteria; Actinomycetia; Corynebacteriales; Mycobacteriaceae; Mycobacterium; Mycobacterium tuberculosis complex; Mycobacterium tuberculosis;	NCBI: 1773	4278569	99.94	0.00	Yes
NCBI Assembly	GCA_001394035.1	NCBI: cellular organisms; Bacteria; Terrabacteria group; Actinobacteria; Actinomycetia; Corynebacteriales; Mycobacteriaceae; Mycobacterium; Mycobacterium tuberculosis complex; Mycobacterium tuberculosis;	NCBI: 1773	5614959	99.14	19.68	No
NCBI Assembly	GCA_001445495.1	NCBI: cellular organisms; Bacteria; Terrabacteria group; Actinobacteria; Actinomycetia; Corynebacteriales; Mycobacteriaceae; Mycobacterium; Mycobacterium tuberculosis complex; Mycobacterium tuberculosis;	NCBI: 1773	4317741	99.50	0.34	Yes
NCBI Assembly	GCA_001391835.1	NCBI: cellular organisms; Bacteria; Terrabacteria group; Actinobacteria; Actinomycetia; Corynebacteriales; Mycobacteriaceae; Mycobacterium; Mycobacterium tuberculosis complex; Mycobacterium tuberculosis;	NCBI: 1773	4339240	99.94	0.00	Yes
NCBI Assembly	GCA_001593225.1	NCBI: cellular organisms; Bacteria; Terrabacteria group; Actinobacteria; Actinomycetia; Corynebacteriales; Mycobacteriaceae; Mycobacterium; Mycobacterium tuberculosis complex; Mycobacterium tuberculosis; Mycobacterium tuberculosis variant africanum;	NCBI: 33894	4493502	99.57	0.00	Yes
NCBI Assembly	GCA_001383115.1	NCBI: cellular organisms; Bacteria; Terrabacteria group; Actinobacteria; Actinomycetia; Corynebacteriales; Mycobacteriaceae; Mycobacterium; Mycobacterium tuberculosis complex; Mycobacterium tuberculosis;	NCBI: 1773	4367101	99.94	0.00	Yes
NCBI Assembly	GCA_001388135.1	NCBI: cellular organisms; Bacteria; Terrabacteria group; Actinobacteria; Actinomycetia; Corynebacteriales; Mycobacteriaceae; Mycobacterium; Mycobacterium tuberculosis complex; Mycobacterium tuberculosis;	NCBI: 1773	4355903	99.94	0.00	Yes
NCBI Assembly	GCA_001387495.1	NCBI: cellular organisms; Bacteria; Terrabacteria group; Actinobacteria; Actinomycetia; Corynebacteriales; Mycobacteriaceae; Mycobacterium; Mycobacterium tuberculosis complex; Mycobacterium tuberculosis;	NCBI: 1773	4368352	99.94	0.00	Yes
NCBI Assembly	GCA_001388735.1	NCBI: cellular organisms; Bacteria; Terrabacteria group; Actinobacteria; Actinomycetia; Corynebacteriales; Mycobacteriaceae; Mycobacterium; Mycobacterium tuberculosis complex; Mycobacterium tuberculosis;	NCBI: 1773	4360976	99.61	0.00	Yes
NCBI Assembly	GCA_001440185.1	NCBI: cellular organisms; Bacteria; Terrabacteria group; Actinobacteria; Actinomycetia; Corynebacteriales; Mycobacteriaceae; Mycobacteroides; unclassified Mycobacteroides;	NCBI: 1720571	4562420	99.50	0.03	Yes
NCBI Assembly	GCA_001389995.1	NCBI: cellular organisms; Bacteria; Terrabacteria group; Actinobacteria; Actinomycetia; Corynebacteriales; Mycobacteriaceae; Mycobacterium; Mycobacterium tuberculosis complex; Mycobacterium tuberculosis;	NCBI: 1773	4352258	99.61	0.00	Yes

NCBI Assembly	GCA_001397495.1	NCBI: cellular organisms; Bacteria; Terrabacteria group; Actinobacteria; Actinomycetia; Corynebacteriales; Mycobacteriaceae; Mycobacterium; Mycobacterium tuberculosis complex; Mycobacterium tuberculosis;	NCBI: 1773	4271174	98.94	0.36	Yes
NCBI Assembly	GCA_001395295.1	NCBI: cellular organisms; Bacteria; Terrabacteria group; Actinobacteria; Actinomycetia; Corynebacteriales; Mycobacteriaceae; Mycobacterium; Mycobacterium tuberculosis complex; Mycobacterium tuberculosis;	NCBI: 1773	4333201	99.86	0.00	Yes
NCBI Assembly	GCA_001397815.1	NCBI: cellular organisms; Bacteria; Terrabacteria group; Actinobacteria; Actinomycetia; Corynebacteriales; Mycobacteriaceae; Mycobacterium; Mycobacterium tuberculosis complex; Mycobacterium tuberculosis;	NCBI: 1773	4342848	99.94	0.00	Yes
NCBI Assembly	GCA_001385935.1	NCBI: cellular organisms; Bacteria; Terrabacteria group; Actinobacteria; Actinomycetia; Corynebacteriales; Mycobacteriaceae; Mycobacterium; Mycobacterium tuberculosis complex; Mycobacterium tuberculosis;	NCBI: 1773	4355541	99.94	0.17	Yes
NCBI Assembly	GCA_001392495.1	NCBI: cellular organisms; Bacteria; Terrabacteria group; Actinobacteria; Actinomycetia; Corynebacteriales; Mycobacteriaceae; Mycobacterium; Mycobacterium tuberculosis complex; Mycobacterium tuberculosis;	NCBI: 1773	4240564	99.90	0.00	Yes
NCBI Assembly	GCA_001388155.1	NCBI: cellular organisms; Bacteria; Terrabacteria group; Actinobacteria; Actinomycetia; Corynebacteriales; Mycobacteriaceae; Mycobacterium; Mycobacterium tuberculosis complex; Mycobacterium tuberculosis;	NCBI: 1773	4340175	99.94	0.00	Yes
NCBI Assembly	GCA_001383135.1	NCBI: cellular organisms; Bacteria; Terrabacteria group; Actinobacteria; Actinomycetia; Corynebacteriales; Mycobacteriaceae; Mycobacterium; Mycobacterium tuberculosis complex; Mycobacterium tuberculosis;	NCBI: 1773	4379454	99.94	0.00	Yes
NCBI Assembly	GCA_001499905.1	NCBI: cellular organisms; Bacteria; Terrabacteria group; Actinobacteria; Actinomycetia; Corynebacteriales; Mycobacteriaceae; Mycobacterium; unclassified Mycobacterium;	NCBI: 1772282	4811582	100.00	0.00	Yes
NCBI Assembly	GCA_001388755.1	NCBI: cellular organisms; Bacteria; Terrabacteria group; Actinobacteria; Actinomycetia; Corynebacteriales; Mycobacteriaceae; Mycobacterium; Mycobacterium tuberculosis complex; Mycobacterium tuberculosis;	NCBI: 1773	4366387	99.78	0.00	Yes
NCBI Assembly	GCA_001387515.1	NCBI: cellular organisms; Bacteria; Terrabacteria group; Actinobacteria; Actinomycetia; Corynebacteriales; Mycobacteriaceae; Mycobacterium; Mycobacterium tuberculosis complex; Mycobacterium tuberculosis;	NCBI: 1773	4358988	99.48	0.00	Yes
NCBI Assembly	GCA_001390015.1	NCBI: cellular organisms; Bacteria; Terrabacteria group; Actinobacteria; Actinomycetia; Corynebacteriales; Mycobacteriaceae; Mycobacterium; Mycobacterium tuberculosis complex; Mycobacterium tuberculosis;	NCBI: 1773	4356627	99.94	0.00	Yes
NCBI Assembly	GCA_001397515.1	NCBI: cellular organisms; Bacteria; Terrabacteria group; Actinobacteria; Actinomycetia; Corynebacteriales; Mycobacteriaceae; Mycobacterium; Mycobacterium tuberculosis complex; Mycobacterium tuberculosis;	NCBI: 1773	4296307	99.83	0.00	Yes
NCBI Assembly	GCA_001395935.1	NCBI: cellular organisms; Bacteria; Terrabacteria group; Actinobacteria; Actinomycetia; Corynebacteriales; Mycobacteriaceae; Mycobacterium; Mycobacterium tuberculosis complex; Mycobacterium tuberculosis;	NCBI: 1773	4322224	99.94	0.00	Yes
NCBI Assembly	GCA_001385955.1	NCBI: cellular organisms; Bacteria; Terrabacteria group; Actinobacteria; Actinomycetia; Corynebacteriales; Mycobacteriaceae; Mycobacterium; Mycobacterium tuberculosis complex; Mycobacterium tuberculosis;	NCBI: 1773	4365312	99.94	0.00	Yes
NCBI Assembly	GCA_001397835.1	NCBI: cellular organisms; Bacteria; Terrabacteria group; Actinobacteria; Actinomycetia; Corynebacteriales; Mycobacteriaceae; Mycobacterium; Mycobacterium tuberculosis complex; Mycobacterium tuberculosis;	NCBI: 1773	4285868	99.57	0.33	Yes
NCBI Assembly	GCA_001392515.1	NCBI: cellular organisms; Bacteria; Terrabacteria group; Actinobacteria; Actinomycetia; Corynebacteriales; Mycobacteriaceae; Mycobacterium; Mycobacterium tuberculosis complex; Mycobacterium tuberculosis;	NCBI: 1773	4290684	99.94	0.33	Yes
NCBI Assembly	GCA_001388175.1	NCBI: cellular organisms; Bacteria; Terrabacteria group; Actinobacteria; Actinomycetia; Corynebacteriales; Mycobacteriaceae; Mycobacterium; Mycobacterium tuberculosis complex; Mycobacterium tuberculosis;	NCBI: 1773	4354051	99.88	0.00	Yes
NCBI Assembly	GCA_001383155.1	NCBI: cellular organisms; Bacteria; Terrabacteria group; Actinobacteria; Actinomycetia; Corynebacteriales; Mycobacteriaceae; Mycobacterium; Mycobacterium tuberculosis complex; Mycobacterium tuberculosis;	NCBI: 1773	4366166	99.94	0.00	Yes

NCBI Assembly	GCA_001387535.1	NCBI: cellular organisms; Bacteria; Terrabacteria group; Actinobacteria; Actinomycetia; Corynebacteriales; Mycobacteriaceae; Mycobacterium; Mycobacterium tuberculosis complex; Mycobacterium tuberculosis;	NCBI: 1773	4355687	99.78	0.00	Yes
NCBI Assembly	GCA_001388775.1	NCBI: cellular organisms; Bacteria; Terrabacteria group; Actinobacteria; Actinomycetia; Corynebacteriales; Mycobacteriaceae; Mycobacterium; Mycobacterium tuberculosis complex; Mycobacterium tuberculosis;	NCBI: 1773	4349166	99.94	0.00	Yes
NCBI Assembly	GCA_001499915.1	NCBI: cellular organisms; Bacteria; Terrabacteria group; Actinobacteria; Actinomycetia; Corynebacteriales; Mycobacteriaceae; Mycolicibacterium; Mycolicibacterium acapulense;	NCBI: 114977	5203567	100.00	0.45	Yes
NCBI Assembly	GCA_001390035.1	NCBI: cellular organisms; Bacteria; Terrabacteria group; Actinobacteria; Actinomycetia; Corynebacteriales; Mycobacteriaceae; Mycobacterium; Mycobacterium tuberculosis complex; Mycobacterium tuberculosis;	NCBI: 1773	4354221	99.88	0.00	Yes
NCBI Assembly	GCA_001397535.1	NCBI: cellular organisms; Bacteria; Terrabacteria group; Actinobacteria; Actinomycetia; Corynebacteriales; Mycobacteriaceae; Mycobacterium; Mycobacterium tuberculosis complex; Mycobacterium tuberculosis;	NCBI: 1773	4284680	99.94	0.08	Yes
NCBI Assembly	GCA_001395955.1	NCBI: cellular organisms; Bacteria; Terrabacteria group; Actinobacteria; Actinomycetia; Corynebacteriales; Mycobacteriaceae; Mycobacterium; Mycobacterium tuberculosis complex; Mycobacterium tuberculosis;	NCBI: 1773	4336612	99.94	0.00	Yes
NCBI Assembly	GCA_001385975.1	NCBI: cellular organisms; Bacteria; Terrabacteria group; Actinobacteria; Actinomycetia; Corynebacteriales; Mycobacteriaceae; Mycobacterium; Mycobacterium tuberculosis complex; Mycobacterium tuberculosis;	NCBI: 1773	4366706	99.94	0.00	Yes
NCBI Assembly	GCA_001392535.1	NCBI: cellular organisms; Bacteria; Terrabacteria group; Actinobacteria; Actinomycetia; Corynebacteriales; Mycobacteriaceae; Mycobacterium; Mycobacterium tuberculosis complex; Mycobacterium tuberculosis;	NCBI: 1773	4341859	99.94	0.00	Yes
NCBI Assembly	GCA_001397855.1	NCBI: cellular organisms; Bacteria; Terrabacteria group; Actinobacteria; Actinomycetia; Corynebacteriales; Mycobacteriaceae; Mycobacterium; Mycobacterium tuberculosis complex; Mycobacterium tuberculosis;	NCBI: 1773	4277079	99.05	1.04	Yes
NCBI Assembly	GCA_001388195.1	NCBI: cellular organisms; Bacteria; Terrabacteria group; Actinobacteria; Actinomycetia; Corynebacteriales; Mycobacteriaceae; Mycobacterium; Mycobacterium tuberculosis complex; Mycobacterium tuberculosis;	NCBI: 1773	4350901	99.94	0.00	Yes
NCBI Assembly	GCA_001383175.1	NCBI: cellular organisms; Bacteria; Terrabacteria group; Actinobacteria; Actinomycetia; Corynebacteriales; Mycobacteriaceae; Mycobacterium; Mycobacterium tuberculosis complex; Mycobacterium tuberculosis;	NCBI: 1773	4367811	99.94	0.17	Yes
NCBI Assembly	GCA_001388795.1	NCBI: cellular organisms; Bacteria; Terrabacteria group; Actinobacteria; Actinomycetia; Corynebacteriales; Mycobacteriaceae; Mycobacterium; Mycobacterium tuberculosis complex; Mycobacterium tuberculosis;	NCBI: 1773	4352618	99.94	0.00	Yes
NCBI Assembly	GCA_001387555.1	NCBI: cellular organisms; Bacteria; Terrabacteria group; Actinobacteria; Actinomycetia; Corynebacteriales; Mycobacteriaceae; Mycobacterium; Mycobacterium tuberculosis complex; Mycobacterium tuberculosis;	NCBI: 1773	4358373	99.51	0.11	Yes
NCBI Assembly	GCA_001397555.1	NCBI: cellular organisms; Bacteria; Terrabacteria group; Actinobacteria; Actinomycetia; Corynebacteriales; Mycobacteriaceae; Mycobacterium; Mycobacterium tuberculosis complex; Mycobacterium tuberculosis;	NCBI: 1773	4274933	99.90	0.00	Yes
NCBI Assembly	GCA_001390055.1	NCBI: cellular organisms; Bacteria; Terrabacteria group; Actinobacteria; Actinomycetia; Corynebacteriales; Mycobacteriaceae; Mycobacterium; Mycobacterium tuberculosis complex; Mycobacterium tuberculosis;	NCBI: 1773	4357435	99.94	0.00	Yes
NCBI Assembly	GCA_001395975.1	NCBI: cellular organisms; Bacteria; Terrabacteria group; Actinobacteria; Actinomycetia; Corynebacteriales; Mycobacteriaceae; Mycobacterium; Mycobacterium tuberculosis complex; Mycobacterium tuberculosis;	NCBI: 1773	4295542	99.86	0.00	Yes
NCBI Assembly	GCA_001385995.1	NCBI: cellular organisms; Bacteria; Terrabacteria group; Actinobacteria; Actinomycetia; Corynebacteriales; Mycobacteriaceae; Mycobacterium; Mycobacterium tuberculosis complex; Mycobacterium tuberculosis;	NCBI: 1773	4371486	99.94	0.00	Yes
NCBI Assembly	GCA_001392555.1	NCBI: cellular organisms; Bacteria; Terrabacteria group; Actinobacteria; Actinomycetia; Corynebacteriales; Mycobacteriaceae; Mycobacterium; Mycobacterium tuberculosis complex; Mycobacterium tuberculosis;	NCBI: 1773	4305058	99.61	0.00	Yes

NCBI Assembly	GCA_001499925.1	NCBI: cellular organisms; Bacteria; Terrabacteria group; Actinobacteria; Actinomycetia; Corynebacteriales; Mycobacteriaceae; Mycobacterium; Mycobacterium lehmannii;	NCBI: 2048550	5130549	100.00	1.14	Yes
NCBI Assembly	GCA_001397875.1	NCBI: cellular organisms; Bacteria; Terrabacteria group; Actinobacteria; Actinomycetia; Corynebacteriales; Mycobacteriaceae; Mycobacterium; Mycobacterium tuberculosis complex; Mycobacterium tuberculosis;	NCBI: 1773	4282848	99.94	0.36	Yes
NCBI Assembly	GCA_001383195.1	NCBI: cellular organisms; Bacteria; Terrabacteria group; Actinobacteria; Actinomycetia; Corynebacteriales; Mycobacteriaceae; Mycobacterium; Mycobacterium tuberculosis complex; Mycobacterium tuberculosis;	NCBI: 1773	4364961	99.94	0.00	Yes
NCBI Assembly	GCA_001388815.1	NCBI: cellular organisms; Bacteria; Terrabacteria group; Actinobacteria; Actinomycetia; Corynebacteriales; Mycobacteriaceae; Mycobacterium; Mycobacterium tuberculosis complex; Mycobacterium tuberculosis;	NCBI: 1773	4352811	99.61	0.00	Yes
NCBI Assembly	GCA_001388215.1	NCBI: cellular organisms; Bacteria; Terrabacteria group; Actinobacteria; Actinomycetia; Corynebacteriales; Mycobacteriaceae; Mycobacterium; Mycobacterium tuberculosis complex; Mycobacterium tuberculosis;	NCBI: 1773	4365983	99.94	0.00	Yes
NCBI Assembly	GCA_001387575.1	NCBI: cellular organisms; Bacteria; Terrabacteria group; Actinobacteria; Actinomycetia; Corynebacteriales; Mycobacteriaceae; Mycobacterium; Mycobacterium tuberculosis complex; Mycobacterium tuberculosis;	NCBI: 1773	4348346	99.94	0.00	Yes
NCBI Assembly	GCA_001397575.1	NCBI: cellular organisms; Bacteria; Terrabacteria group; Actinobacteria; Actinomycetia; Corynebacteriales; Mycobacteriaceae; Mycobacterium; Mycobacterium tuberculosis complex; Mycobacterium tuberculosis;	NCBI: 1773	4248567	99.09	0.70	Yes
NCBI Assembly	GCA_001390075.1	NCBI: cellular organisms; Bacteria; Terrabacteria group; Actinobacteria; Actinomycetia; Corynebacteriales; Mycobacteriaceae; Mycobacterium; Mycobacterium tuberculosis complex; Mycobacterium tuberculosis;	NCBI: 1773	4359869	99.61	0.00	Yes
NCBI Assembly	GCA_001386015.1	NCBI: cellular organisms; Bacteria; Terrabacteria group; Actinobacteria; Actinomycetia; Corynebacteriales; Mycobacteriaceae; Mycobacterium; Mycobacterium tuberculosis complex; Mycobacterium tuberculosis;	NCBI: 1773	4365858	99.94	0.00	Yes
NCBI Assembly	GCA_001499965.1	NCBI: cellular organisms; Bacteria; Terrabacteria group; Actinobacteria; Actinomycetia; Corynebacteriales; Mycobacteriaceae; Mycobacterium; unclassified Mycobacterium;	NCBI: 1772283	6503379	100.00	0.28	Yes
NCBI Assembly	GCA_001392575.1	NCBI: cellular organisms; Bacteria; Terrabacteria group; Actinobacteria; Actinomycetia; Corynebacteriales; Mycobacteriaceae; Mycobacterium; Mycobacterium tuberculosis complex; Mycobacterium tuberculosis;	NCBI: 1773	4274010	99.67	0.00	Yes
NCBI Assembly	GCA_001395995.1	NCBI: cellular organisms; Bacteria; Terrabacteria group; Actinobacteria; Actinomycetia; Corynebacteriales; Mycobacteriaceae; Mycobacterium; Mycobacterium tuberculosis complex; Mycobacterium tuberculosis;	NCBI: 1773	4291852	99.83	0.00	Yes
NCBI Assembly	GCA_001397895.1	NCBI: cellular organisms; Bacteria; Terrabacteria group; Actinobacteria; Actinomycetia; Corynebacteriales; Mycobacteriaceae; Mycobacterium; Mycobacterium tuberculosis complex; Mycobacterium tuberculosis;	NCBI: 1773	4318701	99.91	0.00	Yes
NCBI Assembly	GCA_001383215.1	NCBI: cellular organisms; Bacteria; Terrabacteria group; Actinobacteria; Actinomycetia; Corynebacteriales; Mycobacteriaceae; Mycobacterium; Mycobacterium tuberculosis complex; Mycobacterium tuberculosis;	NCBI: 1773	4366506	99.94	0.17	Yes
NCBI Assembly	GCA_001388235.1	NCBI: cellular organisms; Bacteria; Terrabacteria group; Actinobacteria; Actinomycetia; Corynebacteriales; Mycobacteriaceae; Mycobacterium; Mycobacterium tuberculosis complex; Mycobacterium tuberculosis;	NCBI: 1773	4368903	99.61	0.00	Yes
NCBI Assembly	GCA_001388835.1	NCBI: cellular organisms; Bacteria; Terrabacteria group; Actinobacteria; Actinomycetia; Corynebacteriales; Mycobacteriaceae; Mycobacterium; Mycobacterium tuberculosis complex; Mycobacterium tuberculosis;	NCBI: 1773	4357433	99.94	0.00	Yes
NCBI Assembly	GCA_001387595.1	NCBI: cellular organisms; Bacteria; Terrabacteria group; Actinobacteria; Actinomycetia; Corynebacteriales; Mycobacteriaceae; Mycobacterium; Mycobacterium tuberculosis complex; Mycobacterium tuberculosis;	NCBI: 1773	4351113	99.94	0.00	Yes
NCBI Assembly	GCA_001397595.1	NCBI: cellular organisms; Bacteria; Terrabacteria group; Actinobacteria; Actinomycetia; Corynebacteriales; Mycobacteriaceae; Mycobacterium; Mycobacterium tuberculosis complex; Mycobacterium tuberculosis;	NCBI: 1773	4381838	99.56	0.54	Yes

NCBI Assembly	GCA_001499985.1	NCBI: cellular organisms; Bacteria; Terrabacteria group; Actinobacteria; Actinomycetia; Corynebacteriales; Mycobacteriaceae; Mycobacterium; unclassified Mycobacterium;	NCBI: 1772276	5214044	100.00	0.45	Yes
NCBI Assembly	GCA_001390095.1	NCBI: cellular organisms; Bacteria; Terrabacteria group; Actinobacteria; Actinomycetia; Corynebacteriales; Mycobacteriaceae; Mycobacterium; Mycobacterium tuberculosis complex; Mycobacterium tuberculosis;	NCBI: 1773	4355833	99.94	0.00	Yes
NCBI Assembly	GCA_001386035.1	NCBI: cellular organisms; Bacteria; Terrabacteria group; Actinobacteria; Actinomycetia; Corynebacteriales; Mycobacteriaceae; Mycobacterium; Mycobacterium tuberculosis complex; Mycobacterium tuberculosis;	NCBI: 1773	4369189	99.94	0.00	Yes
NCBI Assembly	GCA_001392595.1	NCBI: cellular organisms; Bacteria; Terrabacteria group; Actinobacteria; Actinomycetia; Corynebacteriales; Mycobacteriaceae; Mycobacterium; Mycobacterium tuberculosis complex; Mycobacterium tuberculosis;	NCBI: 1773	4309915	99.94	0.00	Yes
NCBI Assembly	GCA_001396015.1	NCBI: cellular organisms; Bacteria; Terrabacteria group; Actinobacteria; Actinomycetia; Corynebacteriales; Mycobacteriaceae; Mycobacterium; Mycobacterium tuberculosis complex; Mycobacterium tuberculosis;	NCBI: 1773	4309997	99.94	0.00	Yes
NCBI Assembly	GCA_001397915.1	NCBI: cellular organisms; Bacteria; Terrabacteria group; Actinobacteria; Actinomycetia; Corynebacteriales; Mycobacteriaceae; Mycobacterium; Mycobacterium tuberculosis complex; Mycobacterium tuberculosis;	NCBI: 1773	4304432	99.61	0.00	Yes
NCBI Assembly	GCA_001388255.1	NCBI: cellular organisms; Bacteria; Terrabacteria group; Actinobacteria; Actinomycetia; Corynebacteriales; Mycobacteriaceae; Mycobacterium; Mycobacterium tuberculosis complex; Mycobacterium tuberculosis;	NCBI: 1773	4363423	99.90	0.00	Yes
NCBI Assembly	GCA_001383235.1	NCBI: cellular organisms; Bacteria; Terrabacteria group; Actinobacteria; Actinomycetia; Corynebacteriales; Mycobacteriaceae; Mycobacterium; Mycobacterium tuberculosis complex; Mycobacterium tuberculosis;	NCBI: 1773	4365378	99.94	0.00	Yes
NCBI Assembly	GCA_001388855.1	NCBI: cellular organisms; Bacteria; Terrabacteria group; Actinobacteria; Actinomycetia; Corynebacteriales; Mycobacteriaceae; Mycobacterium; Mycobacterium tuberculosis complex; Mycobacterium tuberculosis;	NCBI: 1773	4354891	99.94	0.00	Yes
NCBI Assembly	GCA_001387615.1	NCBI: cellular organisms; Bacteria; Terrabacteria group; Actinobacteria; Actinomycetia; Corynebacteriales; Mycobacteriaceae; Mycobacterium; Mycobacterium tuberculosis complex; Mycobacterium tuberculosis;	NCBI: 1773	4382427	99.89	0.00	Yes
NCBI Assembly	GCA_001499995.1	NCBI: cellular organisms; Bacteria; Terrabacteria group; Actinobacteria; Actinomycetia; Corynebacteriales; Mycobacteriaceae; Mycobacterium; unclassified Mycobacterium;	NCBI: 1772284	5286172	100.00	0.00	Yes
NCBI Assembly	GCA_001386055.1	NCBI: cellular organisms; Bacteria; Terrabacteria group; Actinobacteria; Actinomycetia; Corynebacteriales; Mycobacteriaceae; Mycobacterium; Mycobacterium tuberculosis complex; Mycobacterium tuberculosis;	NCBI: 1773	4366148	99.94	0.00	Yes
NCBI Assembly	GCA_001397615.1	NCBI: cellular organisms; Bacteria; Terrabacteria group; Actinobacteria; Actinomycetia; Corynebacteriales; Mycobacteriaceae; Mycobacterium; Mycobacterium tuberculosis complex; Mycobacterium tuberculosis;	NCBI: 1773	4331770	99.91	0.50	Yes
NCBI Assembly	GCA_001392615.1	NCBI: cellular organisms; Bacteria; Terrabacteria group; Actinobacteria; Actinomycetia; Corynebacteriales; Mycobacteriaceae; Mycobacterium; Mycobacterium tuberculosis complex; Mycobacterium tuberculosis;	NCBI: 1773	4298167	99.94	0.00	Yes
NCBI Assembly	GCA_001390115.1	NCBI: cellular organisms; Bacteria; Terrabacteria group; Actinobacteria; Actinomycetia; Corynebacteriales; Mycobacteriaceae; Mycobacterium; Mycobacterium tuberculosis complex; Mycobacterium tuberculosis;	NCBI: 1773	4349317	99.94	0.00	Yes
NCBI Assembly	GCA_001396035.1	NCBI: cellular organisms; Bacteria; Terrabacteria group; Actinobacteria; Actinomycetia; Corynebacteriales; Mycobacteriaceae; Mycobacterium; Mycobacterium tuberculosis complex; Mycobacterium tuberculosis;	NCBI: 1773	4303202	99.94	0.00	Yes
NCBI Assembly	GCA_001397935.1	NCBI: cellular organisms; Bacteria; Terrabacteria group; Actinobacteria; Actinomycetia; Corynebacteriales; Mycobacteriaceae; Mycobacterium; Mycobacterium tuberculosis complex; Mycobacterium tuberculosis;	NCBI: 1773	4289884	99.11	0.40	Yes
NCBI Assembly	GCA_001383255.1	NCBI: cellular organisms; Bacteria; Terrabacteria group; Actinobacteria; Actinomycetia; Corynebacteriales; Mycobacteriaceae; Mycobacterium; Mycobacterium tuberculosis complex; Mycobacterium tuberculosis;	NCBI: 1773	4377903	99.94	0.67	Yes

NCBI Assembly	GCA_001388275.1	NCBI: cellular organisms; Bacteria; Terrabacteria group; Actinobacteria; Actinomycetia; Corynebacteriales; Mycobacteriaceae; Mycobacterium; Mycobacterium tuberculosis complex; Mycobacterium tuberculosis;	NCBI: 1773	4356485	99.94	0.00	Yes
NCBI Assembly	GCA_001387635.1	NCBI: cellular organisms; Bacteria; Terrabacteria group; Actinobacteria; Actinomycetia; Corynebacteriales; Mycobacteriaceae; Mycobacterium; Mycobacterium tuberculosis complex; Mycobacterium tuberculosis;	NCBI: 1773	4374489	99.94	0.33	Yes
NCBI Assembly	GCA_001388875.1	NCBI: cellular organisms; Bacteria; Terrabacteria group; Actinobacteria; Actinomycetia; Corynebacteriales; Mycobacteriaceae; Mycobacterium; Mycobacterium tuberculosis complex; Mycobacterium tuberculosis;	NCBI: 1773	4347330	99.94	0.00	Yes
NCBI Assembly	GCA_001500025.1	NCBI: cellular organisms; Bacteria; Terrabacteria group; Actinobacteria; Actinomycetia; Corynebacteriales; Mycobacteriaceae; Mycobacterium; unclassified Mycobacterium;	NCBI: 1772286	5382377	100.00	0.61	Yes
NCBI Assembly	GCA_001390135.1	NCBI: cellular organisms; Bacteria; Terrabacteria group; Actinobacteria; Actinomycetia; Corynebacteriales; Mycobacteriaceae; Mycobacterium; Mycobacterium tuberculosis complex; Mycobacterium tuberculosis;	NCBI: 1773	4383736	99.94	0.00	Yes
NCBI Assembly	GCA_001392635.1	NCBI: cellular organisms; Bacteria; Terrabacteria group; Actinobacteria; Actinomycetia; Corynebacteriales; Mycobacteriaceae; Mycobacterium; Mycobacterium tuberculosis complex; Mycobacterium tuberculosis;	NCBI: 1773	4344734	99.94	0.00	Yes
NCBI Assembly	GCA_001386075.1	NCBI: cellular organisms; Bacteria; Terrabacteria group; Actinobacteria; Actinomycetia; Corynebacteriales; Mycobacteriaceae; Mycobacterium; Mycobacterium tuberculosis complex; Mycobacterium tuberculosis;	NCBI: 1773	4360599	99.94	0.00	Yes
NCBI Assembly	GCA_001397635.1	NCBI: cellular organisms; Bacteria; Terrabacteria group; Actinobacteria; Actinomycetia; Corynebacteriales; Mycobacteriaceae; Mycobacterium; Mycobacterium tuberculosis complex; Mycobacterium tuberculosis;	NCBI: 1773	4315166	99.71	0.00	Yes
NCBI Assembly	GCA_001396055.1	NCBI: cellular organisms; Bacteria; Terrabacteria group; Actinobacteria; Actinomycetia; Corynebacteriales; Mycobacteriaceae; Mycobacterium; Mycobacterium tuberculosis complex; Mycobacterium tuberculosis;	NCBI: 1773	4279726	99.90	0.00	Yes
NCBI Assembly	GCA_001397955.1	NCBI: cellular organisms; Bacteria; Terrabacteria group; Actinobacteria; Actinomycetia; Corynebacteriales; Mycobacteriaceae; Mycobacterium; Mycobacterium tuberculosis complex; Mycobacterium tuberculosis;	NCBI: 1773	4288390	98.94	0.36	Yes
NCBI Assembly	GCA_001383275.1	NCBI: cellular organisms; Bacteria; Terrabacteria group; Actinobacteria; Actinomycetia; Corynebacteriales; Mycobacteriaceae; Mycobacterium; Mycobacterium tuberculosis complex; Mycobacterium tuberculosis;	NCBI: 1773	4363029	99.94	0.00	Yes
NCBI Assembly	GCA_001445515.1	NCBI: cellular organisms; Bacteria; Terrabacteria group; Actinobacteria; Actinomycetia; Corynebacteriales; Mycobacteriaceae; Mycobacterium; Mycobacterium tuberculosis complex; Mycobacterium tuberculosis;	NCBI: 1773	4326119	99.28	0.64	Yes
NCBI Assembly	GCA_001388295.1	NCBI: cellular organisms; Bacteria; Terrabacteria group; Actinobacteria; Actinomycetia; Corynebacteriales; Mycobacteriaceae; Mycobacterium; Mycobacterium tuberculosis complex; Mycobacterium tuberculosis;	NCBI: 1773	4353181	99.94	0.00	Yes
NCBI Assembly	GCA_001387655.1	NCBI: cellular organisms; Bacteria; Terrabacteria group; Actinobacteria; Actinomycetia; Corynebacteriales; Mycobacteriaceae; Mycobacterium; Mycobacterium tuberculosis complex; Mycobacterium tuberculosis;	NCBI: 1773	4363807	99.28	0.00	Yes
NCBI Assembly	GCA_001500045.1	NCBI: cellular organisms; Bacteria; Terrabacteria group; Actinobacteria; Actinomycetia; Corynebacteriales; Mycobacteriaceae; Mycobacterium; unclassified Mycobacterium;	NCBI: 1772287	5438806	100.00	0.70	Yes
NCBI Assembly	GCA_001388895.1	NCBI: cellular organisms; Bacteria; Terrabacteria group; Actinobacteria; Actinomycetia; Corynebacteriales; Mycobacteriaceae; Mycobacterium; Mycobacterium tuberculosis complex; Mycobacterium tuberculosis;	NCBI: 1773	4358887	99.61	0.00	Yes
NCBI Assembly	GCA_001386095.1	NCBI: cellular organisms; Bacteria; Terrabacteria group; Actinobacteria; Actinomycetia; Corynebacteriales; Mycobacteriaceae; Mycobacterium; Mycobacterium tuberculosis complex; Mycobacterium tuberculosis;	NCBI: 1773	4366774	99.94	0.00	Yes
NCBI Assembly	GCA_001392655.1	NCBI: cellular organisms; Bacteria; Terrabacteria group; Actinobacteria; Actinomycetia; Corynebacteriales; Mycobacteriaceae; Mycobacterium; Mycobacterium tuberculosis complex; Mycobacterium tuberculosis;	NCBI: 1773	4308649	99.94	0.00	Yes

NCBI Assembly	GCA_001397655.1	NCBI: cellular organisms; Bacteria; Terrabacteria group; Actinobacteria; Actinomycetia; Corynebacteriales; Mycobacteriaceae; Mycobacterium; Mycobacterium tuberculosis complex; Mycobacterium tuberculosis;	NCBI: 1773	4341237	99.90	0.04	Yes
NCBI Assembly	GCA_001390155.1	NCBI: cellular organisms; Bacteria; Terrabacteria group; Actinobacteria; Actinomycetia; Corynebacteriales; Mycobacteriaceae; Mycobacterium; Mycobacterium tuberculosis complex; Mycobacterium tuberculosis;	NCBI: 1773	4350119	99.78	0.00	Yes
NCBI Assembly	GCA_001396075.1	NCBI: cellular organisms; Bacteria; Terrabacteria group; Actinobacteria; Actinomycetia; Corynebacteriales; Mycobacteriaceae; Mycobacterium; Mycobacterium tuberculosis complex; Mycobacterium tuberculosis;	NCBI: 1773	4308282	99.61	0.00	Yes
NCBI Assembly	GCA_001397975.1	NCBI: cellular organisms; Bacteria; Terrabacteria group; Actinobacteria; Actinomycetia; Corynebacteriales; Mycobacteriaceae; Mycobacterium; Mycobacterium tuberculosis complex; Mycobacterium tuberculosis;	NCBI: 1773	4293205	99.94	0.00	Yes
NCBI Assembly	GCA_001383295.1	NCBI: cellular organisms; Bacteria; Terrabacteria group; Actinobacteria; Actinomycetia; Corynebacteriales; Mycobacteriaceae; Mycobacterium; Mycobacterium tuberculosis complex; Mycobacterium tuberculosis;	NCBI: 1773	4368621	99.94	0.00	Yes
NCBI Assembly	GCA_001388315.1	NCBI: cellular organisms; Bacteria; Terrabacteria group; Actinobacteria; Actinomycetia; Corynebacteriales; Mycobacteriaceae; Mycobacterium; Mycobacterium tuberculosis complex; Mycobacterium tuberculosis;	NCBI: 1773	4357950	99.94	0.00	Yes
NCBI Assembly	GCA_001445525.1	NCBI: cellular organisms; Bacteria; Terrabacteria group; Actinobacteria; Actinomycetia; Corynebacteriales; Mycobacteriaceae; Mycobacterium; Mycobacterium tuberculosis complex; Mycobacterium tuberculosis;	NCBI: 1773	4369343	99.94	0.00	Yes
NCBI Assembly	GCA_001500065.1	NCBI: cellular organisms; Bacteria; Terrabacteria group; Actinobacteria; Actinomycetia; Corynebacteriales; Mycobacteriaceae; Mycobacterium; unclassified Mycobacterium;	NCBI: 1772277	5168733	100.00	3.64	Yes
NCBI Assembly	GCA_001387675.1	NCBI: cellular organisms; Bacteria; Terrabacteria group; Actinobacteria; Actinomycetia; Corynebacteriales; Mycobacteriaceae; Mycobacterium; Mycobacterium tuberculosis complex; Mycobacterium tuberculosis;	NCBI: 1773	4356112	99.94	0.00	Yes
NCBI Assembly	GCA_001388915.1	NCBI: cellular organisms; Bacteria; Terrabacteria group; Actinobacteria; Actinomycetia; Corynebacteriales; Mycobacteriaceae; Mycobacterium; Mycobacterium tuberculosis complex; Mycobacterium tuberculosis;	NCBI: 1773	4361533	99.94	0.00	Yes
NCBI Assembly	GCA_001397675.1	NCBI: cellular organisms; Bacteria; Terrabacteria group; Actinobacteria; Actinomycetia; Corynebacteriales; Mycobacteriaceae; Mycobacterium; Mycobacterium tuberculosis complex; Mycobacterium tuberculosis;	NCBI: 1773	4256390	99.94	0.00	Yes
NCBI Assembly	GCA_001392675.1	NCBI: cellular organisms; Bacteria; Terrabacteria group; Actinobacteria; Actinomycetia; Corynebacteriales; Mycobacteriaceae; Mycobacterium; Mycobacterium tuberculosis complex; Mycobacterium tuberculosis;	NCBI: 1773	4267805	99.50	0.00	Yes
NCBI Assembly	GCA_001386115.1	NCBI: cellular organisms; Bacteria; Terrabacteria group; Actinobacteria; Actinomycetia; Corynebacteriales; Mycobacteriaceae; Mycobacterium; Mycobacterium tuberculosis complex; Mycobacterium tuberculosis;	NCBI: 1773	4367558	99.94	0.17	Yes
NCBI Assembly	GCA_001390175.1	NCBI: cellular organisms; Bacteria; Terrabacteria group; Actinobacteria; Actinomycetia; Corynebacteriales; Mycobacteriaceae; Mycobacterium; Mycobacterium tuberculosis complex; Mycobacterium tuberculosis;	NCBI: 1773	4354470	99.94	0.00	Yes
NCBI Assembly	GCA_001396095.1	NCBI: cellular organisms; Bacteria; Terrabacteria group; Actinobacteria; Actinomycetia; Corynebacteriales; Mycobacteriaceae; Mycobacterium; Mycobacterium tuberculosis complex; Mycobacterium tuberculosis;	NCBI: 1773	4321824	99.61	0.00	Yes
NCBI Assembly	GCA_001397995.1	NCBI: cellular organisms; Bacteria; Terrabacteria group; Actinobacteria; Actinomycetia; Corynebacteriales; Mycobacteriaceae; Mycobacterium; Mycobacterium tuberculosis complex; Mycobacterium tuberculosis;	NCBI: 1773	4327398	99.74	0.00	Yes
NCBI Assembly	GCA_001388335.1	NCBI: cellular organisms; Bacteria; Terrabacteria group; Actinobacteria; Actinomycetia; Corynebacteriales; Mycobacteriaceae; Mycobacterium; Mycobacterium tuberculosis complex; Mycobacterium tuberculosis;	NCBI: 1773	4366503	99.61	0.00	Yes
NCBI Assembly	GCA_001383315.1	NCBI: cellular organisms; Bacteria; Terrabacteria group; Actinobacteria; Actinomycetia; Corynebacteriales; Mycobacteriaceae; Mycobacterium; Mycobacterium tuberculosis complex; Mycobacterium tuberculosis;	NCBI: 1773	4359039	99.94	0.00	Yes

NCBI Assembly	GCA_001387695.1	NCBI: cellular organisms; Bacteria; Terrabacteria group; Actinobacteria; Actinomycetia; Corynebacteriales; Mycobacteriaceae; Mycobacterium; Mycobacterium tuberculosis complex; Mycobacterium tuberculosis;	NCBI: 1773	4356555	99.94	0.00	Yes
NCBI Assembly	GCA_001388935.1	NCBI: cellular organisms; Bacteria; Terrabacteria group; Actinobacteria; Actinomycetia; Corynebacteriales; Mycobacteriaceae; Mycobacterium; Mycobacterium tuberculosis complex; Mycobacterium tuberculosis;	NCBI: 1773	4350746	99.94	0.00	Yes
NCBI Assembly	GCA_001500085.1	NCBI: cellular organisms; Bacteria; Terrabacteria group; Actinobacteria; Actinomycetia; Corynebacteriales; Mycobacteriaceae; Mycolicibacterium; Mycolicibacterium acapulense;	NCBI: 114977	5239336	100.00	0.45	Yes
NCBI Assembly	GCA_001445535.1	NCBI: cellular organisms; Bacteria; Terrabacteria group; Actinobacteria; Actinomycetia; Corynebacteriales; Mycobacteriaceae; Mycobacterium; Mycobacterium tuberculosis complex; Mycobacterium tuberculosis;	NCBI: 1773	4303839	99.22	1.71	Yes
NCBI Assembly	GCA_001397695.1	NCBI: cellular organisms; Bacteria; Terrabacteria group; Actinobacteria; Actinomycetia; Corynebacteriales; Mycobacteriaceae; Mycobacterium; Mycobacterium tuberculosis complex; Mycobacterium tuberculosis;	NCBI: 1773	4332800	99.94	0.00	Yes
NCBI Assembly	GCA_001386135.1	NCBI: cellular organisms; Bacteria; Terrabacteria group; Actinobacteria; Actinomycetia; Corynebacteriales; Mycobacteriaceae; Mycobacterium; Mycobacterium tuberculosis complex; Mycobacterium tuberculosis;	NCBI: 1773	4363415	99.94	0.00	Yes
NCBI Assembly	GCA_001392695.1	NCBI: cellular organisms; Bacteria; Terrabacteria group; Actinobacteria; Actinomycetia; Corynebacteriales; Mycobacteriaceae; Mycobacterium; Mycobacterium tuberculosis complex; Mycobacterium tuberculosis;	NCBI: 1773	4320871	99.94	0.00	Yes
NCBI Assembly	GCA_001396115.1	NCBI: cellular organisms; Bacteria; Terrabacteria group; Actinobacteria; Actinomycetia; Corynebacteriales; Mycobacteriaceae; Mycobacterium; Mycobacterium tuberculosis complex; Mycobacterium tuberculosis;	NCBI: 1773	4304025	99.94	0.00	Yes
NCBI Assembly	GCA_001390195.1	NCBI: cellular organisms; Bacteria; Terrabacteria group; Actinobacteria; Actinomycetia; Corynebacteriales; Mycobacteriaceae; Mycobacterium; Mycobacterium tuberculosis complex; Mycobacterium tuberculosis;	NCBI: 1773	4353701	99.61	0.00	Yes
NCBI Assembly	GCA_001398015.1	NCBI: cellular organisms; Bacteria; Terrabacteria group; Actinobacteria; Actinomycetia; Corynebacteriales; Mycobacteriaceae; Mycobacterium; Mycobacterium tuberculosis complex; Mycobacterium tuberculosis;	NCBI: 1773	4348120	99.94	0.00	Yes
NCBI Assembly	GCA_001383335.1	NCBI: cellular organisms; Bacteria; Terrabacteria group; Actinobacteria; Actinomycetia; Corynebacteriales; Mycobacteriaceae; Mycobacterium; Mycobacterium tuberculosis complex; Mycobacterium tuberculosis;	NCBI: 1773	4370521	99.94	0.00	Yes
NCBI Assembly	GCA_001387715.1	NCBI: cellular organisms; Bacteria; Terrabacteria group; Actinobacteria; Actinomycetia; Corynebacteriales; Mycobacteriaceae; Mycobacterium; Mycobacterium tuberculosis complex; Mycobacterium tuberculosis;	NCBI: 1773	4349998	99.78	0.00	Yes
NCBI Assembly	GCA_001388355.1	NCBI: cellular organisms; Bacteria; Terrabacteria group; Actinobacteria; Actinomycetia; Corynebacteriales; Mycobacteriaceae; Mycobacterium; Mycobacterium tuberculosis complex; Mycobacterium tuberculosis;	NCBI: 1773	4359325	99.83	0.00	Yes
NCBI Assembly	GCA_001388955.1	NCBI: cellular organisms; Bacteria; Terrabacteria group; Actinobacteria; Actinomycetia; Corynebacteriales; Mycobacteriaceae; Mycobacterium; Mycobacterium tuberculosis complex; Mycobacterium tuberculosis;	NCBI: 1773	4371795	99.94	0.00	Yes
NCBI Assembly	GCA_001500105.1	NCBI: cellular organisms; Bacteria; Terrabacteria group; Actinobacteria; Actinomycetia; Corynebacteriales; Mycobacteriaceae; Mycolicibacterium; Mycolicibacterium acapulense;	NCBI: 114977	5190594	100.00	0.45	Yes
NCBI Assembly	GCA_001455165.1	NCBI: cellular organisms; Bacteria; Terrabacteria group; Actinobacteria; Actinomycetia; Corynebacteriales; Mycobacteriaceae; Mycobacterium; Mycobacterium tuberculosis complex; Mycobacterium tuberculosis; Mycobacterium tuberculosis variant bovis;	NCBI: 1765	4299850	99.78	0.00	Yes
NCBI Assembly	GCA_001386155.1	NCBI: cellular organisms; Bacteria; Terrabacteria group; Actinobacteria; Actinomycetia; Corynebacteriales; Mycobacteriaceae; Mycobacterium; Mycobacterium tuberculosis complex; Mycobacterium tuberculosis;	NCBI: 1773	4376871	99.94	0.00	Yes
NCBI Assembly	GCA_001397715.1	NCBI: cellular organisms; Bacteria; Terrabacteria group; Actinobacteria; Actinomycetia; Corynebacteriales; Mycobacteriaceae; Mycobacterium; Mycobacterium tuberculosis complex; Mycobacterium tuberculosis;	NCBI: 1773	4316167	99.88	0.00	Yes

NCBI Assembly	GCA_001392715.1	NCBI: cellular organisms; Bacteria; Terrabacteria group; Actinobacteria; Actinomycetia; Corynebacteriales; Mycobacteriaceae; Mycobacterium; Mycobacterium tuberculosis complex; Mycobacterium tuberculosis;	NCBI: 1773	4269612	99.83	0.33	Yes
NCBI Assembly	GCA_001390215.1	NCBI: cellular organisms; Bacteria; Terrabacteria group; Actinobacteria; Actinomycetia; Corynebacteriales; Mycobacteriaceae; Mycobacterium; Mycobacterium tuberculosis complex; Mycobacterium tuberculosis;	NCBI: 1773	4361333	99.94	0.00	Yes
NCBI Assembly	GCA_001396135.1	NCBI: cellular organisms; Bacteria; Terrabacteria group; Actinobacteria; Actinomycetia; Corynebacteriales; Mycobacteriaceae; Mycobacterium; Mycobacterium tuberculosis complex; Mycobacterium tuberculosis;	NCBI: 1773	4288136	99.83	0.00	Yes
NCBI Assembly	GCA_001398035.1	NCBI: cellular organisms; Bacteria; Terrabacteria group; Actinobacteria; Actinomycetia; Corynebacteriales; Mycobacteriaceae; Mycobacterium; Mycobacterium tuberculosis complex; Mycobacterium tuberculosis;	NCBI: 1773	4353888	99.79	0.00	Yes
NCBI Assembly	GCA_001383355.1	NCBI: cellular organisms; Bacteria; Terrabacteria group; Actinobacteria; Actinomycetia; Corynebacteriales; Mycobacteriaceae; Mycobacterium; Mycobacterium tuberculosis complex; Mycobacterium tuberculosis;	NCBI: 1773	4359218	99.94	0.00	Yes
NCBI Assembly	GCA_001387735.1	NCBI: cellular organisms; Bacteria; Terrabacteria group; Actinobacteria; Actinomycetia; Corynebacteriales; Mycobacteriaceae; Mycobacterium; Mycobacterium tuberculosis complex; Mycobacterium tuberculosis;	NCBI: 1773	4350949	99.94	0.00	Yes
NCBI Assembly	GCA_001388375.1	NCBI: cellular organisms; Bacteria; Terrabacteria group; Actinobacteria; Actinomycetia; Corynebacteriales; Mycobacteriaceae; Mycobacterium; Mycobacterium tuberculosis complex; Mycobacterium tuberculosis;	NCBI: 1773	4354613	99.94	0.00	Yes
NCBI Assembly	GCA_001388975.1	NCBI: cellular organisms; Bacteria; Terrabacteria group; Actinobacteria; Actinomycetia; Corynebacteriales; Mycobacteriaceae; Mycobacterium; Mycobacterium tuberculosis complex; Mycobacterium tuberculosis;	NCBI: 1773	4357393	99.94	0.00	Yes
NCBI Assembly	GCA_001500125.1	NCBI: cellular organisms; Bacteria; Terrabacteria group; Actinobacteria; Actinomycetia; Corynebacteriales; Mycobacteriaceae; Mycobacterium; unclassified Mycobacterium;	NCBI: 1772285	5239195	99.81	0.00	Yes
NCBI Assembly	GCA_001456355.1	NCBI: cellular organisms; Bacteria; Terrabacteria group; Actinobacteria; Actinomycetia; Corynebacteriales; Mycobacteriaceae; Mycobacteroides; Mycobacteroides saopaulense;	NCBI: 1578165	4649175	99.50	0.00	Yes
NCBI Assembly	GCA_001386175.1	NCBI: cellular organisms; Bacteria; Terrabacteria group; Actinobacteria; Actinomycetia; Corynebacteriales; Mycobacteriaceae; Mycobacterium; Mycobacterium tuberculosis complex; Mycobacterium tuberculosis;	NCBI: 1773	4366998	99.94	0.00	Yes
NCBI Assembly	GCA_001397735.1	NCBI: cellular organisms; Bacteria; Terrabacteria group; Actinobacteria; Actinomycetia; Corynebacteriales; Mycobacteriaceae; Mycobacterium; Mycobacterium tuberculosis complex; Mycobacterium tuberculosis;	NCBI: 1773	4371642	99.61	0.47	Yes
NCBI Assembly	GCA_001392735.1	NCBI: cellular organisms; Bacteria; Terrabacteria group; Actinobacteria; Actinomycetia; Corynebacteriales; Mycobacteriaceae; Mycobacterium; Mycobacterium tuberculosis complex; Mycobacterium tuberculosis;	NCBI: 1773	4305355	99.94	0.00	Yes
NCBI Assembly	GCA_001390235.1	NCBI: cellular organisms; Bacteria; Terrabacteria group; Actinobacteria; Actinomycetia; Corynebacteriales; Mycobacteriaceae; Mycobacterium; Mycobacterium tuberculosis complex; Mycobacterium tuberculosis;	NCBI: 1773	4350395	99.88	0.00	Yes
NCBI Assembly	GCA_001396155.1	NCBI: cellular organisms; Bacteria; Terrabacteria group; Actinobacteria; Actinomycetia; Corynebacteriales; Mycobacteriaceae; Mycobacterium; Mycobacterium tuberculosis complex; Mycobacterium tuberculosis;	NCBI: 1773	4287597	99.94	0.00	Yes
NCBI Assembly	GCA_001398055.1	NCBI: cellular organisms; Bacteria; Terrabacteria group; Actinobacteria; Actinomycetia; Corynebacteriales; Mycobacteriaceae; Mycobacterium; Mycobacterium tuberculosis complex; Mycobacterium tuberculosis;	NCBI: 1773	4274184	99.28	1.03	Yes
NCBI Assembly	GCA_001383375.1	NCBI: cellular organisms; Bacteria; Terrabacteria group; Actinobacteria; Actinomycetia; Corynebacteriales; Mycobacteriaceae; Mycobacterium; Mycobacterium tuberculosis complex; Mycobacterium tuberculosis;	NCBI: 1773	4363515	99.94	0.00	Yes
NCBI Assembly	GCA_001387755.1	NCBI: cellular organisms; Bacteria; Terrabacteria group; Actinobacteria; Actinomycetia; Corynebacteriales; Mycobacteriaceae; Mycobacterium; Mycobacterium tuberculosis complex; Mycobacterium tuberculosis;	NCBI: 1773	4360092	99.94	0.07	Yes

NCBI Assembly	GCA_001388395.1	NCBI: cellular organisms; Bacteria; Terrabacteria group; Actinobacteria; Actinomycetia; Corynebacteriales; Mycobacteriaceae; Mycobacterium; Mycobacterium tuberculosis complex; Mycobacterium tuberculosis;	NCBI: 1773	4357974	99.61	0.00	Yes
NCBI Assembly	GCA_001388995.1	NCBI: cellular organisms; Bacteria; Terrabacteria group; Actinobacteria; Actinomycetia; Corynebacteriales; Mycobacteriaceae; Mycobacterium; Mycobacterium tuberculosis complex; Mycobacterium tuberculosis;	NCBI: 1773	4390105	99.78	0.33	Yes
NCBI Assembly	GCA_001500145.1	NCBI: cellular organisms; Bacteria; Terrabacteria group; Actinobacteria; Actinomycetia; Corynebacteriales; Mycobacteriaceae; Mycobacterium; unclassified Mycobacterium;	NCBI: 1772275	5153052	100.00	0.45	Yes
NCBI Assembly	GCA_001457595.1	NCBI: cellular organisms; Bacteria; Terrabacteria group; Actinobacteria; Actinomycetia; Corynebacteriales; Mycobacteriaceae; Mycolicibacterium; Mycolicibacterium smegmatis;	NCBI: 1772	6983267	99.94	0.50	Yes
NCBI Assembly	GCA_001386195.1	NCBI: cellular organisms; Bacteria; Terrabacteria group; Actinobacteria; Actinomycetia; Corynebacteriales; Mycobacteriaceae; Mycobacterium; Mycobacterium tuberculosis complex; Mycobacterium tuberculosis;	NCBI: 1773	4367710	99.94	0.17	Yes
NCBI Assembly	GCA_001397755.1	NCBI: cellular organisms; Bacteria; Terrabacteria group; Actinobacteria; Actinomycetia; Corynebacteriales; Mycobacteriaceae; Mycobacterium; Mycobacterium tuberculosis complex; Mycobacterium tuberculosis;	NCBI: 1773	4332215	99.56	0.11	Yes
NCBI Assembly	GCA_001392755.1	NCBI: cellular organisms; Bacteria; Terrabacteria group; Actinobacteria; Actinomycetia; Corynebacteriales; Mycobacteriaceae; Mycobacterium; Mycobacterium tuberculosis complex; Mycobacterium tuberculosis;	NCBI: 1773	4305520	99.94	0.00	Yes
NCBI Assembly	GCA_001390255.1	NCBI: cellular organisms; Bacteria; Terrabacteria group; Actinobacteria; Actinomycetia; Corynebacteriales; Mycobacteriaceae; Mycobacterium; Mycobacterium tuberculosis complex; Mycobacterium tuberculosis;	NCBI: 1773	4361370	99.88	0.42	Yes
NCBI Assembly	GCA_001396175.1	NCBI: cellular organisms; Bacteria; Terrabacteria group; Actinobacteria; Actinomycetia; Corynebacteriales; Mycobacteriaceae; Mycobacterium; Mycobacterium tuberculosis complex; Mycobacterium tuberculosis;	NCBI: 1773	4355679	99.94	0.33	Yes
NCBI Assembly	GCA_001398075.1	NCBI: cellular organisms; Bacteria; Terrabacteria group; Actinobacteria; Actinomycetia; Corynebacteriales; Mycobacteriaceae; Mycobacterium; Mycobacterium tuberculosis complex; Mycobacterium tuberculosis;	NCBI: 1773	4347230	99.83	1.33	Yes
NCBI Assembly	GCA_001383395.1	NCBI: cellular organisms; Bacteria; Terrabacteria group; Actinobacteria; Actinomycetia; Corynebacteriales; Mycobacteriaceae; Mycobacterium; Mycobacterium tuberculosis complex; Mycobacterium tuberculosis;	NCBI: 1773	4365529	99.94	0.17	Yes
NCBI Assembly	GCA_001387775.1	NCBI: cellular organisms; Bacteria; Terrabacteria group; Actinobacteria; Actinomycetia; Corynebacteriales; Mycobacteriaceae; Mycobacterium; Mycobacterium tuberculosis complex; Mycobacterium tuberculosis;	NCBI: 1773	4361056	99.94	0.00	Yes
NCBI Assembly	GCA_001389015.1	NCBI: cellular organisms; Bacteria; Terrabacteria group; Actinobacteria; Actinomycetia; Corynebacteriales; Mycobacteriaceae; Mycobacterium; Mycobacterium tuberculosis complex; Mycobacterium tuberculosis;	NCBI: 1773	4369492	99.94	0.00	Yes
NCBI Assembly	GCA_001388415.1	NCBI: cellular organisms; Bacteria; Terrabacteria group; Actinobacteria; Actinomycetia; Corynebacteriales; Mycobacteriaceae; Mycobacterium; Mycobacterium tuberculosis complex; Mycobacterium tuberculosis;	NCBI: 1773	4363237	99.61	0.00	Yes
NCBI Assembly	GCA_001386215.1	NCBI: cellular organisms; Bacteria; Terrabacteria group; Actinobacteria; Actinomycetia; Corynebacteriales; Mycobacteriaceae; Mycobacterium; Mycobacterium tuberculosis complex; Mycobacterium tuberculosis;	NCBI: 1773	4364522	99.94	0.00	Yes
NCBI Assembly	GCA_001392775.1	NCBI: cellular organisms; Bacteria; Terrabacteria group; Actinobacteria; Actinomycetia; Corynebacteriales; Mycobacteriaceae; Mycobacterium; Mycobacterium tuberculosis complex; Mycobacterium tuberculosis;	NCBI: 1773	4324293	99.94	0.00	Yes
NCBI Assembly	GCA_001468445.1	NCBI: cellular organisms; Bacteria; Terrabacteria group; Actinobacteria; Actinomycetia; Corynebacteriales; Mycobacteriaceae; Mycobacterium; Mycobacterium tuberculosis complex; Mycobacterium tuberculosis;	NCBI: 1773	4282569	99.94	0.00	Yes
NCBI Assembly	GCA_001510785.1	NCBI: cellular organisms; Bacteria; Terrabacteria group; Actinobacteria; Actinomycetia; Corynebacteriales; Mycobacteriaceae; Mycolicibacterium; Mycolicibacterium neoaurum;	NCBI: 1795	5383007	99.62	1.44	Yes

NCBI Assembly	GCA_001397775.1	NCBI: cellular organisms; Bacteria; Terrabacteria group; Actinobacteria; Actinomycetia; Corynebacteriales; Mycobacteriaceae; Mycobacterium; Mycobacterium tuberculosis complex; Mycobacterium tuberculosis;	NCBI: 1773	4275454	99.74	0.73	Yes
NCBI Assembly	GCA_001390275.1	NCBI: cellular organisms; Bacteria; Terrabacteria group; Actinobacteria; Actinomycetia; Corynebacteriales; Mycobacteriaceae; Mycobacterium; Mycobacterium tuberculosis complex; Mycobacterium tuberculosis;	NCBI: 1773	4347348	99.61	0.00	Yes
NCBI Assembly	GCA_001396195.1	NCBI: cellular organisms; Bacteria; Terrabacteria group; Actinobacteria; Actinomycetia; Corynebacteriales; Mycobacteriaceae; Mycobacterium; Mycobacterium tuberculosis complex; Mycobacterium tuberculosis;	NCBI: 1773	4277722	99.94	0.00	Yes
NCBI Assembly	GCA_001403655.1	NCBI: cellular organisms; Bacteria; Terrabacteria group; Actinobacteria; Actinomycetia; Corynebacteriales; Mycobacteriaceae; Mycolicibacterium; Mycolicibacterium peregrinum;	NCBI: 43304	7109837	99.86	0.50	Yes
NCBI Assembly	GCA_001383415.1	NCBI: cellular organisms; Bacteria; Terrabacteria group; Actinobacteria; Actinomycetia; Corynebacteriales; Mycobacteriaceae; Mycobacterium; Mycobacterium tuberculosis complex; Mycobacterium tuberculosis;	NCBI: 1773	4365056	99.94	0.00	Yes
NCBI Assembly	GCA_001387795.1	NCBI: cellular organisms; Bacteria; Terrabacteria group; Actinobacteria; Actinomycetia; Corynebacteriales; Mycobacteriaceae; Mycobacterium; Mycobacterium tuberculosis complex; Mycobacterium tuberculosis;	NCBI: 1773	4355166	99.94	0.00	Yes
NCBI Assembly	GCA_001387815.1	NCBI: cellular organisms; Bacteria; Terrabacteria group; Actinobacteria; Actinomycetia; Corynebacteriales; Mycobacteriaceae; Mycobacterium; Mycobacterium tuberculosis complex; Mycobacterium tuberculosis;	NCBI: 1773	4349903	99.94	0.00	Yes
NCBI Assembly	GCA_001389035.1	NCBI: cellular organisms; Bacteria; Terrabacteria group; Actinobacteria; Actinomycetia; Corynebacteriales; Mycobacteriaceae; Mycobacterium; Mycobacterium tuberculosis complex; Mycobacterium tuberculosis;	NCBI: 1773	4383452	99.94	0.00	Yes
NCBI Assembly	GCA_001386235.1	NCBI: cellular organisms; Bacteria; Terrabacteria group; Actinobacteria; Actinomycetia; Corynebacteriales; Mycobacteriaceae; Mycobacterium; Mycobacterium tuberculosis complex; Mycobacterium tuberculosis;	NCBI: 1773	4389735	99.94	0.00	Yes
NCBI Assembly	GCA_001390295.1	NCBI: cellular organisms; Bacteria; Terrabacteria group; Actinobacteria; Actinomycetia; Corynebacteriales; Mycobacteriaceae; Mycobacterium; Mycobacterium tuberculosis complex; Mycobacterium tuberculosis;	NCBI: 1773	4388866	99.94	0.22	Yes
NCBI Assembly	GCA_001392795.1	NCBI: cellular organisms; Bacteria; Terrabacteria group; Actinobacteria; Actinomycetia; Corynebacteriales; Mycobacteriaceae; Mycobacterium; Mycobacterium tuberculosis complex; Mycobacterium tuberculosis;	NCBI: 1773	4318806	99.61	0.00	Yes
NCBI Assembly	GCA_001397795.1	NCBI: cellular organisms; Bacteria; Terrabacteria group; Actinobacteria; Actinomycetia; Corynebacteriales; Mycobacteriaceae; Mycobacterium; Mycobacterium tuberculosis complex; Mycobacterium tuberculosis;	NCBI: 1773	4292214	99.50	1.04	Yes
NCBI Assembly	GCA_001468545.1	NCBI: cellular organisms; Bacteria; Terrabacteria group; Actinobacteria; Actinomycetia; Corynebacteriales; Mycobacteriaceae; Mycobacterium; Mycobacterium tuberculosis complex; Mycobacterium tuberculosis;	NCBI: 1773	4338921	99.94	0.00	Yes
NCBI Assembly	GCA_001544675.1	NCBI: cellular organisms; Bacteria; Terrabacteria group; Actinobacteria; Actinomycetia; Corynebacteriales; Mycobacteriaceae; Mycobacterium; Mycobacterium tuberculosis complex; Mycobacterium tuberculosis;	NCBI: 1773	4428621	98.86	0.67	Yes
NCBI Assembly	GCA_001396215.1	NCBI: cellular organisms; Bacteria; Terrabacteria group; Actinobacteria; Actinomycetia; Corynebacteriales; Mycobacteriaceae; Mycobacterium; Mycobacterium tuberculosis complex; Mycobacterium tuberculosis;	NCBI: 1773	4287279	99.94	0.00	Yes
NCBI Assembly	GCA_001381855.1	NCBI: cellular organisms; Bacteria; Terrabacteria group; Actinobacteria; Actinomycetia; Corynebacteriales; Mycobacteriaceae; Mycobacterium; Mycobacterium tuberculosis complex; Mycobacterium tuberculosis;	NCBI: 1773	4366276	99.94	0.00	Yes
NCBI Assembly	GCA_001387835.1	NCBI: cellular organisms; Bacteria; Terrabacteria group; Actinobacteria; Actinomycetia; Corynebacteriales; Mycobacteriaceae; Mycobacterium; Mycobacterium tuberculosis complex; Mycobacterium tuberculosis;	NCBI: 1773	4348188	99.94	0.00	Yes
NCBI Assembly	GCA_001382815.1	NCBI: cellular organisms; Bacteria; Terrabacteria group; Actinobacteria; Actinomycetia; Corynebacteriales; Mycobacteriaceae; Mycobacterium; Mycobacterium tuberculosis complex; Mycobacterium tuberculosis;	NCBI: 1773	4361653	99.94	0.00	Yes

NCBI Assembly	GCA_001387175.1	NCBI: cellular organisms; Bacteria; Terrabacteria group; Actinobacteria; Actinomycetia; Corynebacteriales; Mycobacteriaceae; Mycobacterium; Mycobacterium tuberculosis complex; Mycobacterium tuberculosis;	NCBI: 1773	4370266	99.94	0.00	Yes
NCBI Assembly	GCA_001395615.1	NCBI: cellular organisms; Bacteria; Terrabacteria group; Actinobacteria; Actinomycetia; Corynebacteriales; Mycobacteriaceae; Mycobacterium; Mycobacterium tuberculosis complex; Mycobacterium tuberculosis;	NCBI: 1773	4308555	99.83	0.00	Yes
NCBI Assembly	GCA_001475495.2	NCBI: cellular organisms; Bacteria; Terrabacteria group; Actinobacteria; Actinomycetia; Corynebacteriales; Mycobacteriaceae; Mycolicibacterium; Mycolicibacterium fortuitum; Mycolicibacterium fortuitum subsp. fortuitum;	NCBI: 1214102	6257075	89.07	0.13	No
NCBI Assembly	GCA_001386255.1	NCBI: cellular organisms; Bacteria; Terrabacteria group; Actinobacteria; Actinomycetia; Corynebacteriales; Mycobacteriaceae; Mycobacterium; Mycobacterium tuberculosis complex; Mycobacterium tuberculosis;	NCBI: 1773	4365040	99.94	0.00	Yes
NCBI Assembly	GCA_001389675.1	NCBI: cellular organisms; Bacteria; Terrabacteria group; Actinobacteria; Actinomycetia; Corynebacteriales; Mycobacteriaceae; Mycobacterium; Mycobacterium tuberculosis complex; Mycobacterium tuberculosis;	NCBI: 1773	4355024	99.81	0.00	Yes
NCBI Assembly	GCA_001392175.1	NCBI: cellular organisms; Bacteria; Terrabacteria group; Actinobacteria; Actinomycetia; Corynebacteriales; Mycobacteriaceae; Mycobacterium; Mycobacterium tuberculosis complex; Mycobacterium tuberculosis;	NCBI: 1773	4305938	99.61	0.33	Yes
NCBI Assembly	GCA_001396235.1	NCBI: cellular organisms; Bacteria; Terrabacteria group; Actinobacteria; Actinomycetia; Corynebacteriales; Mycobacteriaceae; Mycobacterium; Mycobacterium tuberculosis complex; Mycobacterium tuberculosis;	NCBI: 1773	4296447	99.78	0.00	Yes
NCBI Assembly	GCA_001397175.1	NCBI: cellular organisms; Bacteria; Terrabacteria group; Actinobacteria; Actinomycetia; Corynebacteriales; Mycobacteriaceae; Mycobacterium; Mycobacterium tuberculosis complex; Mycobacterium tuberculosis;	NCBI: 1773	4321749	99.53	0.00	Yes
NCBI Assembly	GCA_001544705.1	NCBI: cellular organisms; Bacteria; Terrabacteria group; Actinobacteria; Actinomycetia; Corynebacteriales; Mycobacteriaceae; Mycobacterium; Mycobacterium tuberculosis complex; Mycobacterium tuberculosis;	NCBI: 1773	4421903	99.61	0.00	Yes
NCBI Assembly	GCA_001381875.1	NCBI: cellular organisms; Bacteria; Terrabacteria group; Actinobacteria; Actinomycetia; Corynebacteriales; Mycobacteriaceae; Mycobacterium; Mycobacterium tuberculosis complex; Mycobacterium tuberculosis;	NCBI: 1773	4369741	99.94	0.00	Yes
NCBI Assembly	GCA_001387855.1	NCBI: cellular organisms; Bacteria; Terrabacteria group; Actinobacteria; Actinomycetia; Corynebacteriales; Mycobacteriaceae; Mycobacterium; Mycobacterium tuberculosis complex; Mycobacterium tuberculosis;	NCBI: 1773	4365459	99.94	0.00	Yes
NCBI Assembly	GCA_001387195.1	NCBI: cellular organisms; Bacteria; Terrabacteria group; Actinobacteria; Actinomycetia; Corynebacteriales; Mycobacteriaceae; Mycobacterium; Mycobacterium tuberculosis complex; Mycobacterium tuberculosis;	NCBI: 1773	4362088	99.94	0.00	Yes
NCBI Assembly	GCA_001382835.1	NCBI: cellular organisms; Bacteria; Terrabacteria group; Actinobacteria; Actinomycetia; Corynebacteriales; Mycobacteriaceae; Mycobacterium; Mycobacterium tuberculosis complex; Mycobacterium tuberculosis;	NCBI: 1773	4369762	99.94	0.00	Yes
NCBI Assembly	GCA_001386275.1	NCBI: cellular organisms; Bacteria; Terrabacteria group; Actinobacteria; Actinomycetia; Corynebacteriales; Mycobacteriaceae; Mycobacterium; Mycobacterium tuberculosis complex; Mycobacterium tuberculosis;	NCBI: 1773	4357300	99.94	0.00	Yes
NCBI Assembly	GCA_001395635.1	NCBI: cellular organisms; Bacteria; Terrabacteria group; Actinobacteria; Actinomycetia; Corynebacteriales; Mycobacteriaceae; Mycobacterium; Mycobacterium tuberculosis complex; Mycobacterium tuberculosis;	NCBI: 1773	4313116	99.94	0.00	Yes
NCBI Assembly	GCA_001397195.1	NCBI: cellular organisms; Bacteria; Terrabacteria group; Actinobacteria; Actinomycetia; Corynebacteriales; Mycobacteriaceae; Mycobacterium; Mycobacterium tuberculosis complex; Mycobacterium tuberculosis;	NCBI: 1773	4303642	99.69	0.00	Yes
NCBI Assembly	GCA_001392195.1	NCBI: cellular organisms; Bacteria; Terrabacteria group; Actinobacteria; Actinomycetia; Corynebacteriales; Mycobacteriaceae; Mycobacterium; Mycobacterium tuberculosis complex; Mycobacterium tuberculosis;	NCBI: 1773	4313693	99.61	0.00	Yes
NCBI Assembly	GCA_001389695.1	NCBI: cellular organisms; Bacteria; Terrabacteria group; Actinobacteria; Actinomycetia; Corynebacteriales; Mycobacteriaceae; Mycobacterium; Mycobacterium tuberculosis complex; Mycobacterium tuberculosis;	NCBI: 1773	4377509	99.88	0.00	Yes

NCBI Assembly	GCA_001396255.1	NCBI: cellular organisms; Bacteria; Terrabacteria group; Actinobacteria; Actinomycetia; Corynebacteriales; Mycobacteriaceae; Mycobacterium; Mycobacterium tuberculosis complex; Mycobacterium tuberculosis;	NCBI: 1773	4322009	99.14	0.00	Yes
NCBI Assembly	GCA_001395315.1	NCBI: cellular organisms; Bacteria; Terrabacteria group; Actinobacteria; Actinomycetia; Corynebacteriales; Mycobacteriaceae; Mycobacterium; Mycobacterium tuberculosis complex; Mycobacterium tuberculosis;	NCBI: 1773	4333999	99.94	0.00	Yes
NCBI Assembly	GCA_001381895.1	NCBI: cellular organisms; Bacteria; Terrabacteria group; Actinobacteria; Actinomycetia; Corynebacteriales; Mycobacteriaceae; Mycobacterium; Mycobacterium tuberculosis complex; Mycobacterium tuberculosis;	NCBI: 1773	4368529	99.94	0.00	Yes
NCBI Assembly	GCA_001382855.1	NCBI: cellular organisms; Bacteria; Terrabacteria group; Actinobacteria; Actinomycetia; Corynebacteriales; Mycobacteriaceae; Mycobacterium; Mycobacterium tuberculosis complex; Mycobacterium tuberculosis;	NCBI: 1773	4369417	99.94	0.33	Yes
NCBI Assembly	GCA_001387215.1	NCBI: cellular organisms; Bacteria; Terrabacteria group; Actinobacteria; Actinomycetia; Corynebacteriales; Mycobacteriaceae; Mycobacterium; Mycobacterium tuberculosis complex; Mycobacterium tuberculosis;	NCBI: 1773	4350659	99.94	0.00	Yes
NCBI Assembly	GCA_001387875.1	NCBI: cellular organisms; Bacteria; Terrabacteria group; Actinobacteria; Actinomycetia; Corynebacteriales; Mycobacteriaceae; Mycobacterium; Mycobacterium tuberculosis complex; Mycobacterium tuberculosis;	NCBI: 1773	4365122	99.94	0.00	Yes
NCBI Assembly	GCA_001386295.1	NCBI: cellular organisms; Bacteria; Terrabacteria group; Actinobacteria; Actinomycetia; Corynebacteriales; Mycobacteriaceae; Mycobacterium; Mycobacterium tuberculosis complex; Mycobacterium tuberculosis;	NCBI: 1773	4364852	99.94	0.00	Yes
NCBI Assembly	GCA_001395655.1	NCBI: cellular organisms; Bacteria; Terrabacteria group; Actinobacteria; Actinomycetia; Corynebacteriales; Mycobacteriaceae; Mycobacterium; Mycobacterium tuberculosis complex; Mycobacterium tuberculosis;	NCBI: 1773	4309573	99.94	0.00	Yes
NCBI Assembly	GCA_001494595.1	NCBI: cellular organisms; Bacteria; Terrabacteria group; Actinobacteria; Actinomycetia; Corynebacteriales; Mycobacteriaceae; Mycobacterium; unclassified Mycobacterium;	NCBI: 1762962	5732010	99.58	0.81	Yes
NCBI Assembly	GCA_001397215.1	NCBI: cellular organisms; Bacteria; Terrabacteria group; Actinobacteria; Actinomycetia; Corynebacteriales; Mycobacteriaceae; Mycobacterium; Mycobacterium tuberculosis complex; Mycobacterium tuberculosis;	NCBI: 1773	4306485	99.78	0.00	Yes
NCBI Assembly	GCA_001389715.1	NCBI: cellular organisms; Bacteria; Terrabacteria group; Actinobacteria; Actinomycetia; Corynebacteriales; Mycobacteriaceae; Mycobacterium; Mycobacterium tuberculosis complex; Mycobacterium tuberculosis;	NCBI: 1773	4382613	99.94	0.00	Yes
NCBI Assembly	GCA_001396275.1	NCBI: cellular organisms; Bacteria; Terrabacteria group; Actinobacteria; Actinomycetia; Corynebacteriales; Mycobacteriaceae; Mycobacterium; Mycobacterium tuberculosis complex; Mycobacterium tuberculosis;	NCBI: 1773	4296753	99.94	0.33	Yes
NCBI Assembly	GCA_001392215.1	NCBI: cellular organisms; Bacteria; Terrabacteria group; Actinobacteria; Actinomycetia; Corynebacteriales; Mycobacteriaceae; Mycobacterium; Mycobacterium tuberculosis complex; Mycobacterium tuberculosis;	NCBI: 1773	4275514	99.83	0.11	Yes
NCBI Assembly	GCA_001395335.1	NCBI: cellular organisms; Bacteria; Terrabacteria group; Actinobacteria; Actinomycetia; Corynebacteriales; Mycobacteriaceae; Mycobacterium; Mycobacterium tuberculosis complex; Mycobacterium tuberculosis;	NCBI: 1773	4302238	99.90	0.33	Yes
NCBI Assembly	GCA_001381915.1	NCBI: cellular organisms; Bacteria; Terrabacteria group; Actinobacteria; Actinomycetia; Corynebacteriales; Mycobacteriaceae; Mycobacterium; Mycobacterium tuberculosis complex; Mycobacterium tuberculosis;	NCBI: 1773	4367588	99.78	0.17	Yes
NCBI Assembly	GCA_001387235.1	NCBI: cellular organisms; Bacteria; Terrabacteria group; Actinobacteria; Actinomycetia; Corynebacteriales; Mycobacteriaceae; Mycobacterium; Mycobacterium tuberculosis complex; Mycobacterium tuberculosis;	NCBI: 1773	4403098	99.88	0.00	Yes
NCBI Assembly	GCA_001382875.1	NCBI: cellular organisms; Bacteria; Terrabacteria group; Actinobacteria; Actinomycetia; Corynebacteriales; Mycobacteriaceae; Mycobacterium; Mycobacterium tuberculosis complex; Mycobacterium tuberculosis;	NCBI: 1773	4365626	99.94	0.00	Yes
NCBI Assembly	GCA_001395675.1	NCBI: cellular organisms; Bacteria; Terrabacteria group; Actinobacteria; Actinomycetia; Corynebacteriales; Mycobacteriaceae; Mycobacterium; Mycobacterium tuberculosis complex; Mycobacterium tuberculosis;	NCBI: 1773	4327991	99.71	0.00	Yes

NCBI Assembly	GCA_001387895.1	NCBI: cellular organisms; Bacteria; Terrabacteria group; Actinobacteria; Actinomycetia; Corynebacteriales; Mycobacteriaceae; Mycobacterium; Mycobacterium tuberculosis complex; Mycobacterium tuberculosis;	NCBI: 1773	4359340	99.94	0.00	Yes
NCBI Assembly	GCA_001389735.1	NCBI: cellular organisms; Bacteria; Terrabacteria group; Actinobacteria; Actinomycetia; Corynebacteriales; Mycobacteriaceae; Mycobacterium; Mycobacterium tuberculosis complex; Mycobacterium tuberculosis;	NCBI: 1773	4355154	99.81	0.00	Yes
NCBI Assembly	GCA_001386315.1	NCBI: cellular organisms; Bacteria; Terrabacteria group; Actinobacteria; Actinomycetia; Corynebacteriales; Mycobacteriaceae; Mycobacterium; Mycobacterium tuberculosis complex; Mycobacterium tuberculosis;	NCBI: 1773	4355830	96.43	0.00	Yes
NCBI Assembly	GCA_001499825.1	NCBI: cellular organisms; Bacteria; Terrabacteria group; Actinobacteria; Actinomycetia; Corynebacteriales; Mycobacteriaceae; Mycolicibacterium; Mycolicibacterium novocastrense;	NCBI: 59813	5639806	100.00	0.61	Yes
NCBI Assembly	GCA_001397235.1	NCBI: cellular organisms; Bacteria; Terrabacteria group; Actinobacteria; Actinomycetia; Corynebacteriales; Mycobacteriaceae; Mycobacterium; Mycobacterium tuberculosis complex; Mycobacterium tuberculosis;	NCBI: 1773	4310144	99.61	0.00	Yes
NCBI Assembly	GCA_001396295.1	NCBI: cellular organisms; Bacteria; Terrabacteria group; Actinobacteria; Actinomycetia; Corynebacteriales; Mycobacteriaceae; Mycobacterium; Mycobacterium tuberculosis complex; Mycobacterium tuberculosis;	NCBI: 1773	4300982	99.92	0.00	Yes
NCBI Assembly	GCA_001395355.1	NCBI: cellular organisms; Bacteria; Terrabacteria group; Actinobacteria; Actinomycetia; Corynebacteriales; Mycobacteriaceae; Mycobacterium; Mycobacterium tuberculosis complex; Mycobacterium tuberculosis;	NCBI: 1773	4289100	99.94	0.00	Yes
NCBI Assembly	GCA_001392235.1	NCBI: cellular organisms; Bacteria; Terrabacteria group; Actinobacteria; Actinomycetia; Corynebacteriales; Mycobacteriaceae; Mycobacterium; Mycobacterium tuberculosis complex; Mycobacterium tuberculosis;	NCBI: 1773	4272429	99.94	0.00	Yes
NCBI Assembly	GCA_001381935.1	NCBI: cellular organisms; Bacteria; Terrabacteria group; Actinobacteria; Actinomycetia; Corynebacteriales; Mycobacteriaceae; Mycobacterium; Mycobacterium tuberculosis complex; Mycobacterium tuberculosis;	NCBI: 1773	4361801	99.94	0.00	Yes
NCBI Assembly	GCA_001382895.1	NCBI: cellular organisms; Bacteria; Terrabacteria group; Actinobacteria; Actinomycetia; Corynebacteriales; Mycobacteriaceae; Mycobacterium; Mycobacterium tuberculosis complex; Mycobacterium tuberculosis;	NCBI: 1773	4365509	99.94	0.00	Yes
NCBI Assembly	GCA_001387255.1	NCBI: cellular organisms; Bacteria; Terrabacteria group; Actinobacteria; Actinomycetia; Corynebacteriales; Mycobacteriaceae; Mycobacterium; Mycobacterium tuberculosis complex; Mycobacterium tuberculosis;	NCBI: 1773	4356483	99.94	0.00	Yes
NCBI Assembly	GCA_001387915.1	NCBI: cellular organisms; Bacteria; Terrabacteria group; Actinobacteria; Actinomycetia; Corynebacteriales; Mycobacteriaceae; Mycobacterium; Mycobacterium tuberculosis complex; Mycobacterium tuberculosis;	NCBI: 1773	4351653	99.94	0.00	Yes
NCBI Assembly	GCA_001395695.1	NCBI: cellular organisms; Bacteria; Terrabacteria group; Actinobacteria; Actinomycetia; Corynebacteriales; Mycobacteriaceae; Mycobacterium; Mycobacterium tuberculosis complex; Mycobacterium tuberculosis;	NCBI: 1773	4274859	99.44	0.00	Yes
NCBI Assembly	GCA_001499835.1	NCBI: cellular organisms; Bacteria; Terrabacteria group; Actinobacteria; Actinomycetia; Corynebacteriales; Mycobacteriaceae; Mycolicibacterium; Mycolicibacterium novocastrense;	NCBI: 59813	5702270	100.00	1.15	Yes
NCBI Assembly	GCA_001386335.1	NCBI: cellular organisms; Bacteria; Terrabacteria group; Actinobacteria; Actinomycetia; Corynebacteriales; Mycobacteriaceae; Mycobacterium; Mycobacterium tuberculosis complex; Mycobacterium tuberculosis;	NCBI: 1773	4354868	99.94	0.00	Yes
NCBI Assembly	GCA_001389755.1	NCBI: cellular organisms; Bacteria; Terrabacteria group; Actinobacteria; Actinomycetia; Corynebacteriales; Mycobacteriaceae; Mycobacterium; Mycobacterium tuberculosis complex; Mycobacterium tuberculosis;	NCBI: 1773	4349744	99.57	0.00	Yes
NCBI Assembly	GCA_001396315.1	NCBI: cellular organisms; Bacteria; Terrabacteria group; Actinobacteria; Actinomycetia; Corynebacteriales; Mycobacteriaceae; Mycobacterium; Mycobacterium tuberculosis complex; Mycobacterium tuberculosis;	NCBI: 1773	4251670	99.09	0.71	Yes
NCBI Assembly	GCA_001397255.1	NCBI: cellular organisms; Bacteria; Terrabacteria group; Actinobacteria; Actinomycetia; Corynebacteriales; Mycobacteriaceae; Mycobacterium; Mycobacterium tuberculosis complex; Mycobacterium tuberculosis;	NCBI: 1773	4357813	99.54	0.00	Yes

NCBI Assembly	GCA_001392255.1	NCBI: cellular organisms; Bacteria; Terrabacteria group; Actinobacteria; Actinomycetia; Corynebacteriales; Mycobacteriaceae; Mycobacterium; Mycobacterium tuberculosis complex; Mycobacterium tuberculosis;	NCBI: 1773	4307272	99.86	0.00	Yes
NCBI Assembly	GCA_001395375.1	NCBI: cellular organisms; Bacteria; Terrabacteria group; Actinobacteria; Actinomycetia; Corynebacteriales; Mycobacteriaceae; Mycobacterium; Mycobacterium tuberculosis complex; Mycobacterium tuberculosis;	NCBI: 1773	4281386	99.61	0.33	Yes
NCBI Assembly	GCA_001381955.1	NCBI: cellular organisms; Bacteria; Terrabacteria group; Actinobacteria; Actinomycetia; Corynebacteriales; Mycobacteriaceae; Mycobacterium; Mycobacterium tuberculosis complex; Mycobacterium tuberculosis;	NCBI: 1773	4369367	99.94	0.17	Yes
NCBI Assembly	GCA_001382915.1	NCBI: cellular organisms; Bacteria; Terrabacteria group; Actinobacteria; Actinomycetia; Corynebacteriales; Mycobacteriaceae; Mycobacterium; Mycobacterium tuberculosis complex; Mycobacterium tuberculosis;	NCBI: 1773	4362964	99.94	0.00	Yes
NCBI Assembly	GCA_001387275.1	NCBI: cellular organisms; Bacteria; Terrabacteria group; Actinobacteria; Actinomycetia; Corynebacteriales; Mycobacteriaceae; Mycobacterium; Mycobacterium tuberculosis complex; Mycobacterium tuberculosis;	NCBI: 1773	4350673	99.94	0.00	Yes
NCBI Assembly	GCA_001387935.1	NCBI: cellular organisms; Bacteria; Terrabacteria group; Actinobacteria; Actinomycetia; Corynebacteriales; Mycobacteriaceae; Mycobacterium; Mycobacterium tuberculosis complex; Mycobacterium tuberculosis;	NCBI: 1773	4351729	99.61	0.00	Yes
NCBI Assembly	GCA_001389775.1	NCBI: cellular organisms; Bacteria; Terrabacteria group; Actinobacteria; Actinomycetia; Corynebacteriales; Mycobacteriaceae; Mycobacterium; Mycobacterium tuberculosis complex; Mycobacterium tuberculosis;	NCBI: 1773	4352074	99.94	0.00	Yes
NCBI Assembly	GCA_001386355.1	NCBI: cellular organisms; Bacteria; Terrabacteria group; Actinobacteria; Actinomycetia; Corynebacteriales; Mycobacteriaceae; Mycobacterium; Mycobacterium tuberculosis complex; Mycobacterium tuberculosis;	NCBI: 1773	4362975	99.94	0.00	Yes
NCBI Assembly	GCA_001397275.1	NCBI: cellular organisms; Bacteria; Terrabacteria group; Actinobacteria; Actinomycetia; Corynebacteriales; Mycobacteriaceae; Mycobacterium; Mycobacterium tuberculosis complex; Mycobacterium tuberculosis;	NCBI: 1773	4336227	99.57	0.00	Yes
NCBI Assembly	GCA_001395715.1	NCBI: cellular organisms; Bacteria; Terrabacteria group; Actinobacteria; Actinomycetia; Corynebacteriales; Mycobacteriaceae; Mycobacterium; Mycobacterium tuberculosis complex; Mycobacterium tuberculosis;	NCBI: 1773	4310579	99.94	0.00	Yes
NCBI Assembly	GCA_001499845.1	NCBI: cellular organisms; Bacteria; Terrabacteria group; Actinobacteria; Actinomycetia; Corynebacteriales; Mycobacteriaceae; Mycolicibacterium; Mycolicibacterium novocastrense;	NCBI: 59813	5644919	100.00	0.61	Yes
NCBI Assembly	GCA_001395395.1	NCBI: cellular organisms; Bacteria; Terrabacteria group; Actinobacteria; Actinomycetia; Corynebacteriales; Mycobacteriaceae; Mycobacterium; Mycobacterium tuberculosis complex; Mycobacterium tuberculosis;	NCBI: 1773	4249142	99.28	0.00	Yes
NCBI Assembly	GCA_001396335.1	NCBI: cellular organisms; Bacteria; Terrabacteria group; Actinobacteria; Actinomycetia; Corynebacteriales; Mycobacteriaceae; Mycobacterium; Mycobacterium tuberculosis complex; Mycobacterium tuberculosis;	NCBI: 1773	4289848	99.81	0.67	Yes
NCBI Assembly	GCA_001392275.1	NCBI: cellular organisms; Bacteria; Terrabacteria group; Actinobacteria; Actinomycetia; Corynebacteriales; Mycobacteriaceae; Mycobacterium; Mycobacterium tuberculosis complex; Mycobacterium tuberculosis;	NCBI: 1773	4324596	99.83	0.00	Yes
NCBI Assembly	GCA_001381975.1	NCBI: cellular organisms; Bacteria; Terrabacteria group; Actinobacteria; Actinomycetia; Corynebacteriales; Mycobacteriaceae; Mycobacterium; Mycobacterium tuberculosis complex; Mycobacterium tuberculosis;	NCBI: 1773	4353124	99.94	0.00	Yes
NCBI Assembly	GCA_001382935.1	NCBI: cellular organisms; Bacteria; Terrabacteria group; Actinobacteria; Actinomycetia; Corynebacteriales; Mycobacteriaceae; Mycobacterium; Mycobacterium tuberculosis complex; Mycobacterium tuberculosis;	NCBI: 1773	4372071	99.94	0.00	Yes
NCBI Assembly	GCA_001387955.1	NCBI: cellular organisms; Bacteria; Terrabacteria group; Actinobacteria; Actinomycetia; Corynebacteriales; Mycobacteriaceae; Mycobacterium; Mycobacterium tuberculosis complex; Mycobacterium tuberculosis;	NCBI: 1773	4370930	99.78	0.00	Yes
NCBI Assembly	GCA_001386375.1	NCBI: cellular organisms; Bacteria; Terrabacteria group; Actinobacteria; Actinomycetia; Corynebacteriales; Mycobacteriaceae; Mycobacterium; Mycobacterium tuberculosis complex; Mycobacterium tuberculosis;	NCBI: 1773	4361725	99.94	0.00	Yes

NCBI Assembly	GCA_001389795.1	NCBI: cellular organisms; Bacteria; Terrabacteria group; Actinobacteria; Actinomycetia; Corynebacteriales; Mycobacteriaceae; Mycobacterium; Mycobacterium tuberculosis complex; Mycobacterium tuberculosis;	NCBI: 1773	4359813	99.94	0.00	Yes
NCBI Assembly	GCA_001387295.1	NCBI: cellular organisms; Bacteria; Terrabacteria group; Actinobacteria; Actinomycetia; Corynebacteriales; Mycobacteriaceae; Mycobacterium; Mycobacterium tuberculosis complex; Mycobacterium tuberculosis;	NCBI: 1773	4347334	99.94	0.00	Yes
NCBI Assembly	GCA_001499855.1	NCBI: cellular organisms; Bacteria; Terrabacteria group; Actinobacteria; Actinomycetia; Corynebacteriales; Mycobacteriaceae; Mycobacterium; unclassified Mycobacterium;	NCBI: 1772274	5148113	100.00	0.45	Yes
NCBI Assembly	GCA_001395735.1	NCBI: cellular organisms; Bacteria; Terrabacteria group; Actinobacteria; Actinomycetia; Corynebacteriales; Mycobacteriaceae; Mycobacterium; Mycobacterium tuberculosis complex; Mycobacterium tuberculosis;	NCBI: 1773	4308448	99.89	0.00	Yes
NCBI Assembly	GCA_001396355.1	NCBI: cellular organisms; Bacteria; Terrabacteria group; Actinobacteria; Actinomycetia; Corynebacteriales; Mycobacteriaceae; Mycobacterium; Mycobacterium tuberculosis complex; Mycobacterium tuberculosis;	NCBI: 1773	4352474	99.53	0.20	Yes
NCBI Assembly	GCA_001395415.1	NCBI: cellular organisms; Bacteria; Terrabacteria group; Actinobacteria; Actinomycetia; Corynebacteriales; Mycobacteriaceae; Mycobacterium; Mycobacterium tuberculosis complex; Mycobacterium tuberculosis;	NCBI: 1773	4319066	99.94	0.07	Yes
NCBI Assembly	GCA_001392295.1	NCBI: cellular organisms; Bacteria; Terrabacteria group; Actinobacteria; Actinomycetia; Corynebacteriales; Mycobacteriaceae; Mycobacterium; Mycobacterium tuberculosis complex; Mycobacterium tuberculosis;	NCBI: 1773	4323045	99.94	0.00	Yes
NCBI Assembly	GCA_001397295.1	NCBI: cellular organisms; Bacteria; Terrabacteria group; Actinobacteria; Actinomycetia; Corynebacteriales; Mycobacteriaceae; Mycobacterium; Mycobacterium tuberculosis complex; Mycobacterium tuberculosis;	NCBI: 1773	4280797	99.61	0.00	Yes
NCBI Assembly	GCA_001381995.1	NCBI: cellular organisms; Bacteria; Terrabacteria group; Actinobacteria; Actinomycetia; Corynebacteriales; Mycobacteriaceae; Mycobacterium; Mycobacterium tuberculosis complex; Mycobacterium tuberculosis;	NCBI: 1773	4381292	99.94	0.00	Yes
NCBI Assembly	GCA_001387975.1	NCBI: cellular organisms; Bacteria; Terrabacteria group; Actinobacteria; Actinomycetia; Corynebacteriales; Mycobacteriaceae; Mycobacterium; Mycobacterium tuberculosis complex; Mycobacterium tuberculosis;	NCBI: 1773	4321178	99.61	0.00	Yes
NCBI Assembly	GCA_001382955.1	NCBI: cellular organisms; Bacteria; Terrabacteria group; Actinobacteria; Actinomycetia; Corynebacteriales; Mycobacteriaceae; Mycobacterium; Mycobacterium tuberculosis complex; Mycobacterium tuberculosis;	NCBI: 1773	4367700	99.94	0.00	Yes
NCBI Assembly	GCA_001387315.1	NCBI: cellular organisms; Bacteria; Terrabacteria group; Actinobacteria; Actinomycetia; Corynebacteriales; Mycobacteriaceae; Mycobacterium; Mycobacterium tuberculosis complex; Mycobacterium tuberculosis;	NCBI: 1773	4361429	99.94	0.00	Yes
NCBI Assembly	GCA_001386395.1	NCBI: cellular organisms; Bacteria; Terrabacteria group; Actinobacteria; Actinomycetia; Corynebacteriales; Mycobacteriaceae; Mycobacterium; Mycobacterium tuberculosis complex; Mycobacterium tuberculosis;	NCBI: 1773	4356484	99.94	0.00	Yes
NCBI Assembly	GCA_001385615.1	NCBI: cellular organisms; Bacteria; Terrabacteria group; Actinobacteria; Actinomycetia; Corynebacteriales; Mycobacteriaceae; Mycobacterium; Mycobacterium tuberculosis complex; Mycobacterium tuberculosis;	NCBI: 1773	4368520	99.94	0.00	Yes
NCBI Assembly	GCA_001389815.1	NCBI: cellular organisms; Bacteria; Terrabacteria group; Actinobacteria; Actinomycetia; Corynebacteriales; Mycobacteriaceae; Mycobacterium; Mycobacterium tuberculosis complex; Mycobacterium tuberculosis;	NCBI: 1773	4358102	99.94	0.00	Yes
NCBI Assembly	GCA_001396375.1	NCBI: cellular organisms; Bacteria; Terrabacteria group; Actinobacteria; Actinomycetia; Corynebacteriales; Mycobacteriaceae; Mycobacterium; Mycobacterium tuberculosis complex; Mycobacterium tuberculosis;	NCBI: 1773	4306100	99.94	0.00	Yes
NCBI Assembly	GCA_001392315.1	NCBI: cellular organisms; Bacteria; Terrabacteria group; Actinobacteria; Actinomycetia; Corynebacteriales; Mycobacteriaceae; Mycobacterium; Mycobacterium tuberculosis complex; Mycobacterium tuberculosis;	NCBI: 1773	4327101	99.94	0.00	Yes
NCBI Assembly	GCA_001395755.1	NCBI: cellular organisms; Bacteria; Terrabacteria group; Actinobacteria; Actinomycetia; Corynebacteriales; Mycobacteriaceae; Mycobacterium; Mycobacterium tuberculosis complex; Mycobacterium tuberculosis;	NCBI: 1773	4322092	99.94	0.33	Yes

NCBI Assembly	GCA_001395435.1	NCBI: cellular organisms; Bacteria; Terrabacteria group; Actinobacteria; Actinomycetia; Corynebacteriales; Mycobacteriaceae; Mycobacterium; Mycobacterium tuberculosis complex; Mycobacterium tuberculosis;	NCBI: 1773	4645497	96.15	12.38	No
NCBI Assembly	GCA_001397315.1	NCBI: cellular organisms; Bacteria; Terrabacteria group; Actinobacteria; Actinomycetia; Corynebacteriales; Mycobacteriaceae; Mycobacterium; Mycobacterium tuberculosis complex; Mycobacterium tuberculosis;	NCBI: 1773	4300130	99.61	0.00	Yes
NCBI Assembly	GCA_001382015.1	NCBI: cellular organisms; Bacteria; Terrabacteria group; Actinobacteria; Actinomycetia; Corynebacteriales; Mycobacteriaceae; Mycobacterium; Mycobacterium tuberculosis complex; Mycobacterium tuberculosis;	NCBI: 1773	4367040	99.94	0.00	Yes
NCBI Assembly	GCA_001387995.1	NCBI: cellular organisms; Bacteria; Terrabacteria group; Actinobacteria; Actinomycetia; Corynebacteriales; Mycobacteriaceae; Mycobacterium; Mycobacterium tuberculosis complex; Mycobacterium tuberculosis;	NCBI: 1773	4367750	99.94	0.00	Yes
NCBI Assembly	GCA_001382975.1	NCBI: cellular organisms; Bacteria; Terrabacteria group; Actinobacteria; Actinomycetia; Corynebacteriales; Mycobacteriaceae; Mycobacterium; Mycobacterium tuberculosis complex; Mycobacterium tuberculosis;	NCBI: 1773	4370655	99.94	0.00	Yes
NCBI Assembly	GCA_001386415.1	NCBI: cellular organisms; Bacteria; Terrabacteria group; Actinobacteria; Actinomycetia; Corynebacteriales; Mycobacteriaceae; Mycobacterium; Mycobacterium tuberculosis complex; Mycobacterium tuberculosis;	NCBI: 1773	4341509	99.94	0.00	Yes
NCBI Assembly	GCA_001389835.1	NCBI: cellular organisms; Bacteria; Terrabacteria group; Actinobacteria; Actinomycetia; Corynebacteriales; Mycobacteriaceae; Mycobacterium; Mycobacterium tuberculosis complex; Mycobacterium tuberculosis;	NCBI: 1773	4319433	99.57	0.00	Yes
NCBI Assembly	GCA_001387335.1	NCBI: cellular organisms; Bacteria; Terrabacteria group; Actinobacteria; Actinomycetia; Corynebacteriales; Mycobacteriaceae; Mycobacterium; Mycobacterium tuberculosis complex; Mycobacterium tuberculosis;	NCBI: 1773	4356622	99.94	0.00	Yes
NCBI Assembly	GCA_001385635.1	NCBI: cellular organisms; Bacteria; Terrabacteria group; Actinobacteria; Actinomycetia; Corynebacteriales; Mycobacteriaceae; Mycobacterium; Mycobacterium tuberculosis complex; Mycobacterium tuberculosis;	NCBI: 1773	4362369	99.94	0.17	Yes
NCBI Assembly	GCA_001392335.1	NCBI: cellular organisms; Bacteria; Terrabacteria group; Actinobacteria; Actinomycetia; Corynebacteriales; Mycobacteriaceae; Mycobacterium; Mycobacterium tuberculosis complex; Mycobacterium tuberculosis;	NCBI: 1773	4293797	99.94	0.00	Yes
NCBI Assembly	GCA_001396395.1	NCBI: cellular organisms; Bacteria; Terrabacteria group; Actinobacteria; Actinomycetia; Corynebacteriales; Mycobacteriaceae; Mycobacterium; Mycobacterium tuberculosis complex; Mycobacterium tuberculosis;	NCBI: 1773	4343790	99.94	0.00	Yes
NCBI Assembly	GCA_001395775.1	NCBI: cellular organisms; Bacteria; Terrabacteria group; Actinobacteria; Actinomycetia; Corynebacteriales; Mycobacteriaceae; Mycobacterium; Mycobacterium tuberculosis complex; Mycobacterium tuberculosis;	NCBI: 1773	4345874	99.94	0.33	Yes
NCBI Assembly	GCA_001397335.1	NCBI: cellular organisms; Bacteria; Terrabacteria group; Actinobacteria; Actinomycetia; Corynebacteriales; Mycobacteriaceae; Mycobacterium; Mycobacterium tuberculosis complex; Mycobacterium tuberculosis;	NCBI: 1773	4295628	99.94	0.00	Yes
NCBI Assembly	GCA_001395455.1	NCBI: cellular organisms; Bacteria; Terrabacteria group; Actinobacteria; Actinomycetia; Corynebacteriales; Mycobacteriaceae; Mycobacterium; Mycobacterium tuberculosis complex; Mycobacterium tuberculosis;	NCBI: 1773	4341865	99.94	0.00	Yes
NCBI Assembly	GCA_001382035.1	NCBI: cellular organisms; Bacteria; Terrabacteria group; Actinobacteria; Actinomycetia; Corynebacteriales; Mycobacteriaceae; Mycobacterium; Mycobacterium tuberculosis complex; Mycobacterium tuberculosis;	NCBI: 1773	4365896	99.94	0.00	Yes
NCBI Assembly	GCA_001388015.1	NCBI: cellular organisms; Bacteria; Terrabacteria group; Actinobacteria; Actinomycetia; Corynebacteriales; Mycobacteriaceae; Mycobacterium; Mycobacterium tuberculosis complex; Mycobacterium tuberculosis;	NCBI: 1773	4362660	99.88	0.00	Yes
NCBI Assembly	GCA_001382995.1	NCBI: cellular organisms; Bacteria; Terrabacteria group; Actinobacteria; Actinomycetia; Corynebacteriales; Mycobacteriaceae; Mycobacterium; Mycobacterium tuberculosis complex; Mycobacterium tuberculosis;	NCBI: 1773	4367663	99.94	0.17	Yes
NCBI Assembly	GCA_001386435.1	NCBI: cellular organisms; Bacteria; Terrabacteria group; Actinobacteria; Actinomycetia; Corynebacteriales; Mycobacteriaceae; Mycobacterium; Mycobacterium tuberculosis complex; Mycobacterium tuberculosis;	NCBI: 1773	4365004	99.94	0.00	Yes

NCBI Assembly	GCA_001385655.1	NCBI: cellular organisms; Bacteria; Terrabacteria group; Actinobacteria; Actinomycetia; Corynebacteriales; Mycobacteriaceae; Mycobacterium; Mycobacterium tuberculosis complex; Mycobacterium tuberculosis;	NCBI: 1773	4368204	99.94	0.00	Yes
NCBI Assembly	GCA_001389855.1	NCBI: cellular organisms; Bacteria; Terrabacteria group; Actinobacteria; Actinomycetia; Corynebacteriales; Mycobacteriaceae; Mycobacterium; Mycobacterium tuberculosis complex; Mycobacterium tuberculosis;	NCBI: 1773	4373403	99.94	0.00	Yes
NCBI Assembly	GCA_001387355.1	NCBI: cellular organisms; Bacteria; Terrabacteria group; Actinobacteria; Actinomycetia; Corynebacteriales; Mycobacteriaceae; Mycobacterium; Mycobacterium tuberculosis complex; Mycobacterium tuberculosis;	NCBI: 1773	4358026	99.90	0.00	Yes
NCBI Assembly	GCA_001392355.1	NCBI: cellular organisms; Bacteria; Terrabacteria group; Actinobacteria; Actinomycetia; Corynebacteriales; Mycobacteriaceae; Mycobacterium; Mycobacterium tuberculosis complex; Mycobacterium tuberculosis;	NCBI: 1773	4323671	99.83	0.00	Yes
NCBI Assembly	GCA_001395795.1	NCBI: cellular organisms; Bacteria; Terrabacteria group; Actinobacteria; Actinomycetia; Corynebacteriales; Mycobacteriaceae; Mycobacterium; Mycobacterium tuberculosis complex; Mycobacterium tuberculosis;	NCBI: 1773	4288307	99.61	0.00	Yes
NCBI Assembly	GCA_001396415.1	NCBI: cellular organisms; Bacteria; Terrabacteria group; Actinobacteria; Actinomycetia; Corynebacteriales; Mycobacteriaceae; Mycobacterium; Mycobacterium tuberculosis complex; Mycobacterium tuberculosis;	NCBI: 1773	4349644	99.90	0.33	Yes
NCBI Assembly	GCA_001397355.1	NCBI: cellular organisms; Bacteria; Terrabacteria group; Actinobacteria; Actinomycetia; Corynebacteriales; Mycobacteriaceae; Mycobacterium; Mycobacterium tuberculosis complex; Mycobacterium tuberculosis;	NCBI: 1773	4337973	99.77	0.38	Yes
NCBI Assembly	GCA_001395475.1	NCBI: cellular organisms; Bacteria; Terrabacteria group; Actinobacteria; Actinomycetia; Corynebacteriales; Mycobacteriaceae; Mycobacterium; Mycobacterium tuberculosis complex; Mycobacterium tuberculosis;	NCBI: 1773	4320603	99.88	0.00	Yes
NCBI Assembly	GCA_001382055.1	NCBI: cellular organisms; Bacteria; Terrabacteria group; Actinobacteria; Actinomycetia; Corynebacteriales; Mycobacteriaceae; Mycobacterium; Mycobacterium tuberculosis complex; Mycobacterium tuberculosis;	NCBI: 1773	4365932	99.44	0.33	Yes
NCBI Assembly	GCA_001388035.1	NCBI: cellular organisms; Bacteria; Terrabacteria group; Actinobacteria; Actinomycetia; Corynebacteriales; Mycobacteriaceae; Mycobacterium; Mycobacterium tuberculosis complex; Mycobacterium tuberculosis;	NCBI: 1773	4350878	99.94	0.00	Yes
NCBI Assembly	GCA_001385675.1	NCBI: cellular organisms; Bacteria; Terrabacteria group; Actinobacteria; Actinomycetia; Corynebacteriales; Mycobacteriaceae; Mycobacterium; Mycobacterium tuberculosis complex; Mycobacterium tuberculosis;	NCBI: 1773	4493866	99.94	1.67	Yes
NCBI Assembly	GCA_001386455.1	NCBI: cellular organisms; Bacteria; Terrabacteria group; Actinobacteria; Actinomycetia; Corynebacteriales; Mycobacteriaceae; Mycobacterium; Mycobacterium tuberculosis complex; Mycobacterium tuberculosis;	NCBI: 1773	4364489	99.94	0.00	Yes
NCBI Assembly	GCA_001383015.1	NCBI: cellular organisms; Bacteria; Terrabacteria group; Actinobacteria; Actinomycetia; Corynebacteriales; Mycobacteriaceae; Mycobacterium; Mycobacterium tuberculosis complex; Mycobacterium tuberculosis;	NCBI: 1773	4390752	99.94	0.00	Yes
NCBI Assembly	GCA_001389875.1	NCBI: cellular organisms; Bacteria; Terrabacteria group; Actinobacteria; Actinomycetia; Corynebacteriales; Mycobacteriaceae; Mycobacterium; Mycobacterium tuberculosis complex; Mycobacterium tuberculosis;	NCBI: 1773	4362766	99.91	0.00	Yes
NCBI Assembly	GCA_001387375.1	NCBI: cellular organisms; Bacteria; Terrabacteria group; Actinobacteria; Actinomycetia; Corynebacteriales; Mycobacteriaceae; Mycobacterium; Mycobacterium tuberculosis complex; Mycobacterium tuberculosis;	NCBI: 1773	4344189	99.94	0.00	Yes
NCBI Assembly	GCA_001397375.1	NCBI: cellular organisms; Bacteria; Terrabacteria group; Actinobacteria; Actinomycetia; Corynebacteriales; Mycobacteriaceae; Mycobacterium; Mycobacterium tuberculosis complex; Mycobacterium tuberculosis;	NCBI: 1773	4318459	99.91	0.00	Yes
NCBI Assembly	GCA_001396435.1	NCBI: cellular organisms; Bacteria; Terrabacteria group; Actinobacteria; Actinomycetia; Corynebacteriales; Mycobacteriaceae; Mycobacterium; Mycobacterium tuberculosis complex; Mycobacterium tuberculosis;	NCBI: 1773	4326215	99.81	0.00	Yes
NCBI Assembly	GCA_001392375.1	NCBI: cellular organisms; Bacteria; Terrabacteria group; Actinobacteria; Actinomycetia; Corynebacteriales; Mycobacteriaceae; Mycobacterium; Mycobacterium tuberculosis complex; Mycobacterium tuberculosis;	NCBI: 1773	4339113	99.61	0.00	Yes

NCBI Assembly	GCA_001395815.1	NCBI: cellular organisms; Bacteria; Terrabacteria group; Actinobacteria; Actinomycetia; Corynebacteriales; Mycobacteriaceae; Mycobacterium; Mycobacterium tuberculosis complex; Mycobacterium tuberculosis;	NCBI: 1773	4316319	99.94	0.00	Yes
NCBI Assembly	GCA_001395495.1	NCBI: cellular organisms; Bacteria; Terrabacteria group; Actinobacteria; Actinomycetia; Corynebacteriales; Mycobacteriaceae; Mycobacterium; Mycobacterium tuberculosis complex; Mycobacterium tuberculosis;	NCBI: 1773	4298284	99.94	0.00	Yes
NCBI Assembly	GCA_001382075.1	NCBI: cellular organisms; Bacteria; Terrabacteria group; Actinobacteria; Actinomycetia; Corynebacteriales; Mycobacteriaceae; Mycobacterium; Mycobacterium tuberculosis complex; Mycobacterium tuberculosis;	NCBI: 1773	4364842	99.78	0.00	Yes
NCBI Assembly	GCA_001386475.1	NCBI: cellular organisms; Bacteria; Terrabacteria group; Actinobacteria; Actinomycetia; Corynebacteriales; Mycobacteriaceae; Mycobacterium; Mycobacterium tuberculosis complex; Mycobacterium tuberculosis;	NCBI: 1773	4398626	99.94	0.17	Yes
NCBI Assembly	GCA_001388055.1	NCBI: cellular organisms; Bacteria; Terrabacteria group; Actinobacteria; Actinomycetia; Corynebacteriales; Mycobacteriaceae; Mycobacterium; Mycobacterium tuberculosis complex; Mycobacterium tuberculosis;	NCBI: 1773	4354199	99.94	0.00	Yes
NCBI Assembly	GCA_001387395.1	NCBI: cellular organisms; Bacteria; Terrabacteria group; Actinobacteria; Actinomycetia; Corynebacteriales; Mycobacteriaceae; Mycobacterium; Mycobacterium tuberculosis complex; Mycobacterium tuberculosis;	NCBI: 1773	4352585	99.57	0.00	Yes
NCBI Assembly	GCA_001383035.1	NCBI: cellular organisms; Bacteria; Terrabacteria group; Actinobacteria; Actinomycetia; Corynebacteriales; Mycobacteriaceae; Mycobacterium; Mycobacterium tuberculosis complex; Mycobacterium tuberculosis;	NCBI: 1773	4367087	99.94	0.00	Yes
NCBI Assembly	GCA_001385695.1	NCBI: cellular organisms; Bacteria; Terrabacteria group; Actinobacteria; Actinomycetia; Corynebacteriales; Mycobacteriaceae; Mycobacterium; Mycobacterium tuberculosis complex; Mycobacterium tuberculosis;	NCBI: 1773	4365015	99.94	0.00	Yes
NCBI Assembly	GCA_001389895.1	NCBI: cellular organisms; Bacteria; Terrabacteria group; Actinobacteria; Actinomycetia; Corynebacteriales; Mycobacteriaceae; Mycobacterium; Mycobacterium tuberculosis complex; Mycobacterium tuberculosis;	NCBI: 1773	4356311	99.94	0.00	Yes
NCBI Assembly	GCA_001395835.1	NCBI: cellular organisms; Bacteria; Terrabacteria group; Actinobacteria; Actinomycetia; Corynebacteriales; Mycobacteriaceae; Mycobacterium; Mycobacterium tuberculosis complex; Mycobacterium tuberculosis;	NCBI: 1773	4347840	99.88	0.00	Yes
NCBI Assembly	GCA_001395515.1	NCBI: cellular organisms; Bacteria; Terrabacteria group; Actinobacteria; Actinomycetia; Corynebacteriales; Mycobacteriaceae; Mycobacterium; Mycobacterium tuberculosis complex; Mycobacterium tuberculosis;	NCBI: 1773	4286397	99.94	0.33	Yes
NCBI Assembly	GCA_001397395.1	NCBI: cellular organisms; Bacteria; Terrabacteria group; Actinobacteria; Actinomycetia; Corynebacteriales; Mycobacteriaceae; Mycobacterium; Mycobacterium tuberculosis complex; Mycobacterium tuberculosis;	NCBI: 1773	4305456	99.47	0.33	Yes
NCBI Assembly	GCA_001396455.1	NCBI: cellular organisms; Bacteria; Terrabacteria group; Actinobacteria; Actinomycetia; Corynebacteriales; Mycobacteriaceae; Mycobacterium; Mycobacterium tuberculosis complex; Mycobacterium tuberculosis;	NCBI: 1773	4309335	99.47	0.40	Yes
NCBI Assembly	GCA_001392395.1	NCBI: cellular organisms; Bacteria; Terrabacteria group; Actinobacteria; Actinomycetia; Corynebacteriales; Mycobacteriaceae; Mycobacterium; Mycobacterium tuberculosis complex; Mycobacterium tuberculosis;	NCBI: 1773	4303739	99.94	0.00	Yes
NCBI Assembly	GCA_001382095.1	NCBI: cellular organisms; Bacteria; Terrabacteria group; Actinobacteria; Actinomycetia; Corynebacteriales; Mycobacteriaceae; Mycobacterium; Mycobacterium tuberculosis complex; Mycobacterium tuberculosis;	NCBI: 1773	4358208	99.94	0.00	Yes
NCBI Assembly	GCA_001386495.1	NCBI: cellular organisms; Bacteria; Terrabacteria group; Actinobacteria; Actinomycetia; Corynebacteriales; Mycobacteriaceae; Mycobacterium; Mycobacterium tuberculosis complex; Mycobacterium tuberculosis;	NCBI: 1773	73427	0.86	0.00	No
NCBI Assembly	GCA_001388075.1	NCBI: cellular organisms; Bacteria; Terrabacteria group; Actinobacteria; Actinomycetia; Corynebacteriales; Mycobacteriaceae; Mycobacterium; Mycobacterium tuberculosis complex; Mycobacterium tuberculosis;	NCBI: 1773	4374480	99.94	0.00	Yes
NCBI Assembly	GCA_001385715.1	NCBI: cellular organisms; Bacteria; Terrabacteria group; Actinobacteria; Actinomycetia; Corynebacteriales; Mycobacteriaceae; Mycobacterium; Mycobacterium tuberculosis complex; Mycobacterium tuberculosis;	NCBI: 1773	4367391	99.94	0.00	Yes

NCBI Assembly	GCA_001383055.1	NCBI: cellular organisms; Bacteria; Terrabacteria group; Actinobacteria; Actinomycetia; Corynebacteriales; Mycobacteriaceae; Mycobacterium; Mycobacterium tuberculosis complex; Mycobacterium tuberculosis;	NCBI: 1773	4389336	99.94	0.00	Yes
NCBI Assembly	GCA_001387415.1	NCBI: cellular organisms; Bacteria; Terrabacteria group; Actinobacteria; Actinomycetia; Corynebacteriales; Mycobacteriaceae; Mycobacterium; Mycobacterium tuberculosis complex; Mycobacterium tuberculosis;	NCBI: 1773	4358505	99.92	0.00	Yes
NCBI Assembly	GCA_001395535.1	NCBI: cellular organisms; Bacteria; Terrabacteria group; Actinobacteria; Actinomycetia; Corynebacteriales; Mycobacteriaceae; Mycobacterium; Mycobacterium tuberculosis complex; Mycobacterium tuberculosis;	NCBI: 1773	4299841	99.94	0.00	Yes
NCBI Assembly	GCA_001389915.1	NCBI: cellular organisms; Bacteria; Terrabacteria group; Actinobacteria; Actinomycetia; Corynebacteriales; Mycobacteriaceae; Mycobacterium; Mycobacterium tuberculosis complex; Mycobacterium tuberculosis;	NCBI: 1773	4392461	99.94	0.00	Yes
NCBI Assembly	GCA_001397415.1	NCBI: cellular organisms; Bacteria; Terrabacteria group; Actinobacteria; Actinomycetia; Corynebacteriales; Mycobacteriaceae; Mycobacterium; Mycobacterium tuberculosis complex; Mycobacterium tuberculosis;	NCBI: 1773	6036092	97.41	46.12	No
NCBI Assembly	GCA_001395855.1	NCBI: cellular organisms; Bacteria; Terrabacteria group; Actinobacteria; Actinomycetia; Corynebacteriales; Mycobacteriaceae; Mycobacterium; Mycobacterium tuberculosis complex; Mycobacterium tuberculosis;	NCBI: 1773	4355120	99.94	0.00	Yes
NCBI Assembly	GCA_001392415.1	NCBI: cellular organisms; Bacteria; Terrabacteria group; Actinobacteria; Actinomycetia; Corynebacteriales; Mycobacteriaceae; Mycobacterium; Mycobacterium tuberculosis complex; Mycobacterium tuberculosis;	NCBI: 1773	4344573	99.94	0.00	Yes
NCBI Assembly	GCA_001396475.1	NCBI: cellular organisms; Bacteria; Terrabacteria group; Actinobacteria; Actinomycetia; Corynebacteriales; Mycobacteriaceae; Mycobacterium; Mycobacterium tuberculosis complex; Mycobacterium tuberculosis;	NCBI: 1773	4299147	99.54	0.03	Yes
NCBI Assembly	GCA_001382115.1	NCBI: cellular organisms; Bacteria; Terrabacteria group; Actinobacteria; Actinomycetia; Corynebacteriales; Mycobacteriaceae; Mycobacterium; Mycobacterium tuberculosis complex; Mycobacterium tuberculosis;	NCBI: 1773	4402561	99.94	0.33	Yes
NCBI Assembly	GCA_001385735.1	NCBI: cellular organisms; Bacteria; Terrabacteria group; Actinobacteria; Actinomycetia; Corynebacteriales; Mycobacteriaceae; Mycobacterium; Mycobacterium tuberculosis complex; Mycobacterium tuberculosis;	NCBI: 1773	4367235	99.94	0.00	Yes
NCBI Assembly	GCA_001383075.1	NCBI: cellular organisms; Bacteria; Terrabacteria group; Actinobacteria; Actinomycetia; Corynebacteriales; Mycobacteriaceae; Mycobacterium; Mycobacterium tuberculosis complex; Mycobacterium tuberculosis;	NCBI: 1773	4369172	99.94	0.00	Yes
NCBI Assembly	GCA_001386515.1	NCBI: cellular organisms; Bacteria; Terrabacteria group; Actinobacteria; Actinomycetia; Corynebacteriales; Mycobacteriaceae; Mycobacterium; Mycobacterium tuberculosis complex; Mycobacterium tuberculosis;	NCBI: 1773	4356396	99.94	0.00	Yes
NCBI Assembly	GCA_001387435.1	NCBI: cellular organisms; Bacteria; Terrabacteria group; Actinobacteria; Actinomycetia; Corynebacteriales; Mycobacteriaceae; Mycobacterium; Mycobacterium tuberculosis complex; Mycobacterium tuberculosis;	NCBI: 1773	4361097	99.94	0.00	Yes
NCBI Assembly	GCA_001388095.1	NCBI: cellular organisms; Bacteria; Terrabacteria group; Actinobacteria; Actinomycetia; Corynebacteriales; Mycobacteriaceae; Mycobacterium; Mycobacterium tuberculosis complex; Mycobacterium tuberculosis;	NCBI: 1773	4367460	99.94	0.00	Yes
NCBI Assembly	GCA_001389935.1	NCBI: cellular organisms; Bacteria; Terrabacteria group; Actinobacteria; Actinomycetia; Corynebacteriales; Mycobacteriaceae; Mycobacterium; Mycobacterium tuberculosis complex; Mycobacterium tuberculosis;	NCBI: 1773	4361556	99.94	0.67	Yes
NCBI Assembly	GCA_001395555.1	NCBI: cellular organisms; Bacteria; Terrabacteria group; Actinobacteria; Actinomycetia; Corynebacteriales; Mycobacteriaceae; Mycobacterium; Mycobacterium tuberculosis complex; Mycobacterium tuberculosis;	NCBI: 1773	4278595	99.17	0.00	Yes
NCBI Assembly	GCA_001395875.1	NCBI: cellular organisms; Bacteria; Terrabacteria group; Actinobacteria; Actinomycetia; Corynebacteriales; Mycobacteriaceae; Mycobacterium; Mycobacterium tuberculosis complex; Mycobacterium tuberculosis;	NCBI: 1773	4302547	99.78	0.00	Yes
NCBI Assembly	GCA_001396495.1	NCBI: cellular organisms; Bacteria; Terrabacteria group; Actinobacteria; Actinomycetia; Corynebacteriales; Mycobacteriaceae; Mycobacterium; Mycobacterium tuberculosis complex; Mycobacterium tuberculosis;	NCBI: 1773	4299904	99.78	0.07	Yes

NCBI Assembly	GCA_001397435.1	NCBI: cellular organisms; Bacteria; Terrabacteria group; Actinobacteria; Actinomycetia; Corynebacteriales; Mycobacteriaceae; Mycobacterium; Mycobacterium tuberculosis complex; Mycobacterium tuberculosis;	NCBI: 1773	4305327	99.83	0.00	Yes
NCBI Assembly	GCA_001392435.1	NCBI: cellular organisms; Bacteria; Terrabacteria group; Actinobacteria; Actinomycetia; Corynebacteriales; Mycobacteriaceae; Mycobacterium; Mycobacterium tuberculosis complex; Mycobacterium tuberculosis;	NCBI: 1773	4318569	99.94	0.00	Yes
NCBI Assembly	GCA_001382135.1	NCBI: cellular organisms; Bacteria; Terrabacteria group; Actinobacteria; Actinomycetia; Corynebacteriales; Mycobacteriaceae; Mycobacterium; Mycobacterium tuberculosis complex; Mycobacterium tuberculosis;	NCBI: 1773	4359223	99.94	0.00	Yes
NCBI Assembly	GCA_001385755.1	NCBI: cellular organisms; Bacteria; Terrabacteria group; Actinobacteria; Actinomycetia; Corynebacteriales; Mycobacteriaceae; Mycobacterium; Mycobacterium tuberculosis complex; Mycobacterium tuberculosis;	NCBI: 1773	4367971	99.94	0.00	Yes
NCBI Assembly	GCA_001383095.1	NCBI: cellular organisms; Bacteria; Terrabacteria group; Actinobacteria; Actinomycetia; Corynebacteriales; Mycobacteriaceae; Mycobacterium; Mycobacterium tuberculosis complex; Mycobacterium tuberculosis;	NCBI: 1773	4367923	99.94	0.17	Yes
NCBI Assembly	GCA_001386535.1	NCBI: cellular organisms; Bacteria; Terrabacteria group; Actinobacteria; Actinomycetia; Corynebacteriales; Mycobacteriaceae; Mycobacterium; Mycobacterium tuberculosis complex; Mycobacterium tuberculosis;	NCBI: 1773	4363886	99.94	0.00	Yes
NCBI Assembly	GCA_001389955.1	NCBI: cellular organisms; Bacteria; Terrabacteria group; Actinobacteria; Actinomycetia; Corynebacteriales; Mycobacteriaceae; Mycobacterium; Mycobacterium tuberculosis complex; Mycobacterium tuberculosis;	NCBI: 1773	4352936	99.94	0.00	Yes
NCBI Assembly	GCA_001390935.1	NCBI: cellular organisms; Bacteria; Terrabacteria group; Actinobacteria; Actinomycetia; Corynebacteriales; Mycobacteriaceae; Mycobacterium; Mycobacterium tuberculosis complex; Mycobacterium tuberculosis;	NCBI: 1773	4348795	99.94	0.00	Yes
NCBI Assembly	GCA_001387455.1	NCBI: cellular organisms; Bacteria; Terrabacteria group; Actinobacteria; Actinomycetia; Corynebacteriales; Mycobacteriaceae; Mycobacterium; Mycobacterium tuberculosis complex; Mycobacterium tuberculosis;	NCBI: 1773	4375757	99.88	0.00	Yes
NCBI Assembly	GCA_001395575.1	NCBI: cellular organisms; Bacteria; Terrabacteria group; Actinobacteria; Actinomycetia; Corynebacteriales; Mycobacteriaceae; Mycobacterium; Mycobacterium tuberculosis complex; Mycobacterium tuberculosis;	NCBI: 1773	4317664	99.78	0.00	Yes
NCBI Assembly	GCA_001392455.1	NCBI: cellular organisms; Bacteria; Terrabacteria group; Actinobacteria; Actinomycetia; Corynebacteriales; Mycobacteriaceae; Mycobacterium; Mycobacterium tuberculosis complex; Mycobacterium tuberculosis;	NCBI: 1773	4333276	99.94	0.00	Yes
NCBI Assembly	GCA_001395895.1	NCBI: cellular organisms; Bacteria; Terrabacteria group; Actinobacteria; Actinomycetia; Corynebacteriales; Mycobacteriaceae; Mycobacterium; Mycobacterium tuberculosis complex; Mycobacterium tuberculosis;	NCBI: 1773	4326310	99.94	0.00	Yes
NCBI Assembly	GCA_001396515.1	NCBI: cellular organisms; Bacteria; Terrabacteria group; Actinobacteria; Actinomycetia; Corynebacteriales; Mycobacteriaceae; Mycobacterium; Mycobacterium tuberculosis complex; Mycobacterium tuberculosis;	NCBI: 1773	4302585	99.94	0.00	Yes
NCBI Assembly	GCA_001397455.1	NCBI: cellular organisms; Bacteria; Terrabacteria group; Actinobacteria; Actinomycetia; Corynebacteriales; Mycobacteriaceae; Mycobacterium; Mycobacterium tuberculosis complex; Mycobacterium tuberculosis;	NCBI: 1773	4304726	99.63	1.03	Yes
NCBI Assembly	GCA_001382155.1	NCBI: cellular organisms; Bacteria; Terrabacteria group; Actinobacteria; Actinomycetia; Corynebacteriales; Mycobacteriaceae; Mycobacterium; Mycobacterium tuberculosis complex; Mycobacterium tuberculosis;	NCBI: 1773	4368298	99.94	0.00	Yes
NCBI Assembly	GCA_001384675.1	NCBI: cellular organisms; Bacteria; Terrabacteria group; Actinobacteria; Actinomycetia; Corynebacteriales; Mycobacteriaceae; Mycobacterium; Mycobacterium tuberculosis complex; Mycobacterium tuberculosis;	NCBI: 1773	4371403	99.94	0.17	Yes
NCBI Assembly	GCA_001385775.1	NCBI: cellular organisms; Bacteria; Terrabacteria group; Actinobacteria; Actinomycetia; Corynebacteriales; Mycobacteriaceae; Mycobacterium; Mycobacterium tuberculosis complex; Mycobacterium tuberculosis;	NCBI: 1773	4367651	99.94	0.00	Yes
NCBI Assembly	GCA_001384355.1	NCBI: cellular organisms; Bacteria; Terrabacteria group; Actinobacteria; Actinomycetia; Corynebacteriales; Mycobacteriaceae; Mycobacterium; Mycobacterium tuberculosis complex; Mycobacterium tuberculosis;	NCBI: 1773	4361102	99.94	0.00	Yes

NCBI Assembly	GCA_001390955.1	NCBI: cellular organisms; Bacteria; Terrabacteria group; Actinobacteria; Actinomycetia; Corynebacteriales; Mycobacteriaceae; Mycobacterium; Mycobacterium tuberculosis complex; Mycobacterium tuberculosis;	NCBI: 1773	4379898	99.94	0.00	Yes
NCBI Assembly	GCA_001387475.1	NCBI: cellular organisms; Bacteria; Terrabacteria group; Actinobacteria; Actinomycetia; Corynebacteriales; Mycobacteriaceae; Mycobacterium; Mycobacterium tuberculosis complex; Mycobacterium tuberculosis;	NCBI: 1773	4358520	99.94	0.00	Yes
NCBI Assembly	GCA_001392475.1	NCBI: cellular organisms; Bacteria; Terrabacteria group; Actinobacteria; Actinomycetia; Corynebacteriales; Mycobacteriaceae; Mycobacterium; Mycobacterium tuberculosis complex; Mycobacterium tuberculosis;	NCBI: 1773	4339369	99.86	0.00	Yes
NCBI Assembly	GCA_001389975.1	NCBI: cellular organisms; Bacteria; Terrabacteria group; Actinobacteria; Actinomycetia; Corynebacteriales; Mycobacteriaceae; Mycobacterium; Mycobacterium tuberculosis complex; Mycobacterium tuberculosis;	NCBI: 1773	4353679	99.86	0.00	Yes
NCBI Assembly	GCA_001395595.1	NCBI: cellular organisms; Bacteria; Terrabacteria group; Actinobacteria; Actinomycetia; Corynebacteriales; Mycobacteriaceae; Mycobacterium; Mycobacterium tuberculosis complex; Mycobacterium tuberculosis;	NCBI: 1773	4291410	99.83	0.00	Yes
NCBI Assembly	GCA_001395915.1	NCBI: cellular organisms; Bacteria; Terrabacteria group; Actinobacteria; Actinomycetia; Corynebacteriales; Mycobacteriaceae; Mycobacterium; Mycobacterium tuberculosis complex; Mycobacterium tuberculosis;	NCBI: 1773	4341794	99.94	0.00	Yes
NCBI Assembly	GCA_001396535.1	NCBI: cellular organisms; Bacteria; Terrabacteria group; Actinobacteria; Actinomycetia; Corynebacteriales; Mycobacteriaceae; Mycobacterium; Mycobacterium tuberculosis complex; Mycobacterium tuberculosis;	NCBI: 1773	4321409	99.66	0.00	Yes
NCBI Assembly	GCA_001397475.1	NCBI: cellular organisms; Bacteria; Terrabacteria group; Actinobacteria; Actinomycetia; Corynebacteriales; Mycobacteriaceae; Mycobacterium; Mycobacterium tuberculosis complex; Mycobacterium tuberculosis;	NCBI: 1773	4276595	99.42	0.00	Yes
NCBI Assembly	GCA_001381555.1	NCBI: cellular organisms; Bacteria; Terrabacteria group; Actinobacteria; Actinomycetia; Corynebacteriales; Mycobacteriaceae; Mycobacterium; Mycobacterium tuberculosis complex; Mycobacterium tuberculosis;	NCBI: 1773	4365809	99.94	0.17	Yes
NCBI Assembly	GCA_001384695.1	NCBI: cellular organisms; Bacteria; Terrabacteria group; Actinobacteria; Actinomycetia; Corynebacteriales; Mycobacteriaceae; Mycobacterium; Mycobacterium tuberculosis complex; Mycobacterium tuberculosis;	NCBI: 1773	4364510	99.94	0.00	Yes
NCBI Assembly	GCA_001384375.1	NCBI: cellular organisms; Bacteria; Terrabacteria group; Actinobacteria; Actinomycetia; Corynebacteriales; Mycobacteriaceae; Mycobacterium; Mycobacterium tuberculosis complex; Mycobacterium tuberculosis;	NCBI: 1773	4365734	99.94	0.00	Yes
NCBI Assembly	GCA_001390975.1	NCBI: cellular organisms; Bacteria; Terrabacteria group; Actinobacteria; Actinomycetia; Corynebacteriales; Mycobacteriaceae; Mycobacterium; Mycobacterium tuberculosis complex; Mycobacterium tuberculosis;	NCBI: 1773	4357009	99.94	0.67	Yes
NCBI Assembly	GCA_001385795.1	NCBI: cellular organisms; Bacteria; Terrabacteria group; Actinobacteria; Actinomycetia; Corynebacteriales; Mycobacteriaceae; Mycobacterium; Mycobacterium tuberculosis complex; Mycobacterium tuberculosis;	NCBI: 1773	4403535	99.94	0.33	Yes
NCBI Assembly	GCA_001385315.1	NCBI: cellular organisms; Bacteria; Terrabacteria group; Actinobacteria; Actinomycetia; Corynebacteriales; Mycobacteriaceae; Mycobacterium; Mycobacterium tuberculosis complex; Mycobacterium tuberculosis;	NCBI: 1773	4367206	99.94	0.00	Yes
NCBI Assembly	GCA_001384995.1	NCBI: cellular organisms; Bacteria; Terrabacteria group; Actinobacteria; Actinomycetia; Corynebacteriales; Mycobacteriaceae; Mycobacterium; Mycobacterium tuberculosis complex; Mycobacterium tuberculosis;	NCBI: 1773	4374755	99.94	0.00	Yes
NCBI Assembly	GCA_001384055.1	NCBI: cellular organisms; Bacteria; Terrabacteria group; Actinobacteria; Actinomycetia; Corynebacteriales; Mycobacteriaceae; Mycobacterium; Mycobacterium tuberculosis complex; Mycobacterium tuberculosis;	NCBI: 1773	4356215	99.78	0.00	Yes
NCBI Assembly	GCA_001380935.1	NCBI: cellular organisms; Bacteria; Terrabacteria group; Actinobacteria; Actinomycetia; Corynebacteriales; Mycobacteriaceae; Mycobacterium; Mycobacterium tuberculosis complex; Mycobacterium tuberculosis;	NCBI: 1773	4368135	99.94	0.00	Yes
NCBI Assembly	GCA_001380615.1	NCBI: cellular organisms; Bacteria; Terrabacteria group; Actinobacteria; Actinomycetia; Corynebacteriales; Mycobacteriaceae; Mycobacterium; Mycobacterium tuberculosis complex; Mycobacterium tuberculosis;	NCBI: 1773	4367228	99.94	0.17	Yes

NCBI Assembly	GCA_001381235.1	NCBI: cellular organisms; Bacteria; Terrabacteria group; Actinobacteria; Actinomycetia; Corynebacteriales; Mycobacteriaceae; Mycobacterium; Mycobacterium tuberculosis complex; Mycobacterium tuberculosis;	NCBI: 1773	4365322	99.94	0.00	Yes
NCBI Assembly	GCA_001380315.1	NCBI: cellular organisms; Bacteria; Terrabacteria group; Actinobacteria; Actinomycetia; Corynebacteriales; Mycobacteriaceae; Mycobacterium; Mycobacterium tuberculosis complex; Mycobacterium tuberculosis;	NCBI: 1773	4366607	99.94	0.33	Yes
NCBI Assembly	GCA_001381575.1	NCBI: cellular organisms; Bacteria; Terrabacteria group; Actinobacteria; Actinomycetia; Corynebacteriales; Mycobacteriaceae; Mycobacterium; Mycobacterium tuberculosis complex; Mycobacterium tuberculosis;	NCBI: 1773	4381109	99.94	0.00	Yes
NCBI Assembly	GCA_001385015.1	NCBI: cellular organisms; Bacteria; Terrabacteria group; Actinobacteria; Actinomycetia; Corynebacteriales; Mycobacteriaceae; Mycobacterium; Mycobacterium tuberculosis complex; Mycobacterium tuberculosis;	NCBI: 1773	4369951	99.94	0.00	Yes
NCBI Assembly	GCA_001384395.1	NCBI: cellular organisms; Bacteria; Terrabacteria group; Actinobacteria; Actinomycetia; Corynebacteriales; Mycobacteriaceae; Mycobacterium; Mycobacterium tuberculosis complex; Mycobacterium tuberculosis;	NCBI: 1773	4367999	99.94	0.00	Yes
NCBI Assembly	GCA_001384075.1	NCBI: cellular organisms; Bacteria; Terrabacteria group; Actinobacteria; Actinomycetia; Corynebacteriales; Mycobacteriaceae; Mycobacterium; Mycobacterium tuberculosis complex; Mycobacterium tuberculosis;	NCBI: 1773	4367276	99.94	0.00	Yes
NCBI Assembly	GCA_001385335.1	NCBI: cellular organisms; Bacteria; Terrabacteria group; Actinobacteria; Actinomycetia; Corynebacteriales; Mycobacteriaceae; Mycobacterium; Mycobacterium tuberculosis complex; Mycobacterium tuberculosis;	NCBI: 1773	4366010	99.94	0.00	Yes
NCBI Assembly	GCA_001384715.1	NCBI: cellular organisms; Bacteria; Terrabacteria group; Actinobacteria; Actinomycetia; Corynebacteriales; Mycobacteriaceae; Mycobacterium; Mycobacterium tuberculosis complex; Mycobacterium tuberculosis;	NCBI: 1773	4360672	99.94	0.17	Yes
NCBI Assembly	GCA_001385815.1	NCBI: cellular organisms; Bacteria; Terrabacteria group; Actinobacteria; Actinomycetia; Corynebacteriales; Mycobacteriaceae; Mycobacterium; Mycobacterium tuberculosis complex; Mycobacterium tuberculosis;	NCBI: 1773	4366937	99.94	0.17	Yes
NCBI Assembly	GCA_001390995.1	NCBI: cellular organisms; Bacteria; Terrabacteria group; Actinobacteria; Actinomycetia; Corynebacteriales; Mycobacteriaceae; Mycobacterium; Mycobacterium tuberculosis complex; Mycobacterium tuberculosis;	NCBI: 1773	4344334	99.94	0.00	Yes
NCBI Assembly	GCA_001380955.1	NCBI: cellular organisms; Bacteria; Terrabacteria group; Actinobacteria; Actinomycetia; Corynebacteriales; Mycobacteriaceae; Mycobacterium; Mycobacterium tuberculosis complex; Mycobacterium tuberculosis;	NCBI: 1773	4363952	99.94	0.00	Yes
NCBI Assembly	GCA_001380635.1	NCBI: cellular organisms; Bacteria; Terrabacteria group; Actinobacteria; Actinomycetia; Corynebacteriales; Mycobacteriaceae; Mycobacterium; Mycobacterium tuberculosis complex; Mycobacterium tuberculosis;	NCBI: 1773	4366719	99.94	0.00	Yes
NCBI Assembly	GCA_001381255.1	NCBI: cellular organisms; Bacteria; Terrabacteria group; Actinobacteria; Actinomycetia; Corynebacteriales; Mycobacteriaceae; Mycobacterium; Mycobacterium tuberculosis complex; Mycobacterium tuberculosis;	NCBI: 1773	4355232	99.94	0.00	Yes
NCBI Assembly	GCA_001380335.1	NCBI: cellular organisms; Bacteria; Terrabacteria group; Actinobacteria; Actinomycetia; Corynebacteriales; Mycobacteriaceae; Mycobacterium; Mycobacterium tuberculosis complex; Mycobacterium tuberculosis;	NCBI: 1773	4366967	99.94	0.17	Yes
NCBI Assembly	GCA_001381595.1	NCBI: cellular organisms; Bacteria; Terrabacteria group; Actinobacteria; Actinomycetia; Corynebacteriales; Mycobacteriaceae; Mycobacterium; Mycobacterium tuberculosis complex; Mycobacterium tuberculosis;	NCBI: 1773	4366604	99.94	0.00	Yes
NCBI Assembly	GCA_001384095.1	NCBI: cellular organisms; Bacteria; Terrabacteria group; Actinobacteria; Actinomycetia; Corynebacteriales; Mycobacteriaceae; Mycobacterium; Mycobacterium tuberculosis complex; Mycobacterium tuberculosis;	NCBI: 1773	4366841	99.94	0.00	Yes
NCBI Assembly	GCA_001384735.1	NCBI: cellular organisms; Bacteria; Terrabacteria group; Actinobacteria; Actinomycetia; Corynebacteriales; Mycobacteriaceae; Mycobacterium; Mycobacterium tuberculosis complex; Mycobacterium tuberculosis;	NCBI: 1773	4400002	99.94	0.00	Yes
NCBI Assembly	GCA_001385035.1	NCBI: cellular organisms; Bacteria; Terrabacteria group; Actinobacteria; Actinomycetia; Corynebacteriales; Mycobacteriaceae; Mycobacterium; Mycobacterium tuberculosis complex; Mycobacterium tuberculosis;	NCBI: 1773	4365191	99.94	0.00	Yes

NCBI Assembly	GCA_001385355.1	NCBI: cellular organisms; Bacteria; Terrabacteria group; Actinobacteria; Actinomycetia; Corynebacteriales; Mycobacteriaceae; Mycobacterium; Mycobacterium tuberculosis complex; Mycobacterium tuberculosis;	NCBI: 1773	4388070	99.94	0.00	Yes
NCBI Assembly	GCA_001384415.1	NCBI: cellular organisms; Bacteria; Terrabacteria group; Actinobacteria; Actinomycetia; Corynebacteriales; Mycobacteriaceae; Mycobacterium; Mycobacterium tuberculosis complex; Mycobacterium tuberculosis;	NCBI: 1773	4385014	99.94	0.00	Yes
NCBI Assembly	GCA_001385835.1	NCBI: cellular organisms; Bacteria; Terrabacteria group; Actinobacteria; Actinomycetia; Corynebacteriales; Mycobacteriaceae; Mycobacterium; Mycobacterium tuberculosis complex; Mycobacterium tuberculosis;	NCBI: 1773	4413305	99.94	0.00	Yes
NCBI Assembly	GCA_001380975.1	NCBI: cellular organisms; Bacteria; Terrabacteria group; Actinobacteria; Actinomycetia; Corynebacteriales; Mycobacteriaceae; Mycobacterium; Mycobacterium tuberculosis complex; Mycobacterium tuberculosis;	NCBI: 1773	4365401	99.94	0.00	Yes
NCBI Assembly	GCA_001391015.1	NCBI: cellular organisms; Bacteria; Terrabacteria group; Actinobacteria; Actinomycetia; Corynebacteriales; Mycobacteriaceae; Mycobacterium; Mycobacterium tuberculosis complex; Mycobacterium tuberculosis;	NCBI: 1773	4352934	99.94	0.00	Yes
NCBI Assembly	GCA_001381275.1	NCBI: cellular organisms; Bacteria; Terrabacteria group; Actinobacteria; Actinomycetia; Corynebacteriales; Mycobacteriaceae; Mycobacterium; Mycobacterium tuberculosis complex; Mycobacterium tuberculosis;	NCBI: 1773	4371032	99.94	0.00	Yes
NCBI Assembly	GCA_001380655.1	NCBI: cellular organisms; Bacteria; Terrabacteria group; Actinobacteria; Actinomycetia; Corynebacteriales; Mycobacteriaceae; Mycobacterium; Mycobacterium tuberculosis complex; Mycobacterium tuberculosis;	NCBI: 1773	4369708	99.94	0.00	Yes
NCBI Assembly	GCA_001380355.1	NCBI: cellular organisms; Bacteria; Terrabacteria group; Actinobacteria; Actinomycetia; Corynebacteriales; Mycobacteriaceae; Mycobacterium; Mycobacterium tuberculosis complex; Mycobacterium tuberculosis;	NCBI: 1773	4366648	99.94	0.17	Yes
NCBI Assembly	GCA_001381615.1	NCBI: cellular organisms; Bacteria; Terrabacteria group; Actinobacteria; Actinomycetia; Corynebacteriales; Mycobacteriaceae; Mycobacterium; Mycobacterium tuberculosis complex; Mycobacterium tuberculosis;	NCBI: 1773	4364765	99.94	0.17	Yes
NCBI Assembly	GCA_001385055.1	NCBI: cellular organisms; Bacteria; Terrabacteria group; Actinobacteria; Actinomycetia; Corynebacteriales; Mycobacteriaceae; Mycobacterium; Mycobacterium tuberculosis complex; Mycobacterium tuberculosis;	NCBI: 1773	4461626	99.94	0.11	Yes
NCBI Assembly	GCA_001384115.1	NCBI: cellular organisms; Bacteria; Terrabacteria group; Actinobacteria; Actinomycetia; Corynebacteriales; Mycobacteriaceae; Mycobacterium; Mycobacterium tuberculosis complex; Mycobacterium tuberculosis;	NCBI: 1773	4366417	99.94	0.00	Yes
NCBI Assembly	GCA_001385375.1	NCBI: cellular organisms; Bacteria; Terrabacteria group; Actinobacteria; Actinomycetia; Corynebacteriales; Mycobacteriaceae; Mycobacterium; Mycobacterium tuberculosis complex; Mycobacterium tuberculosis;	NCBI: 1773	4400337	99.94	0.00	Yes
NCBI Assembly	GCA_001384755.1	NCBI: cellular organisms; Bacteria; Terrabacteria group; Actinobacteria; Actinomycetia; Corynebacteriales; Mycobacteriaceae; Mycobacterium; Mycobacterium tuberculosis complex; Mycobacterium tuberculosis;	NCBI: 1773	4367685	99.94	0.17	Yes
NCBI Assembly	GCA_001384435.1	NCBI: cellular organisms; Bacteria; Terrabacteria group; Actinobacteria; Actinomycetia; Corynebacteriales; Mycobacteriaceae; Mycobacterium; Mycobacterium tuberculosis complex; Mycobacterium tuberculosis;	NCBI: 1773	4369796	99.94	0.00	Yes
NCBI Assembly	GCA_001385855.1	NCBI: cellular organisms; Bacteria; Terrabacteria group; Actinobacteria; Actinomycetia; Corynebacteriales; Mycobacteriaceae; Mycobacterium; Mycobacterium tuberculosis complex; Mycobacterium tuberculosis;	NCBI: 1773	4365310	99.94	0.17	Yes
NCBI Assembly	GCA_001380995.1	NCBI: cellular organisms; Bacteria; Terrabacteria group; Actinobacteria; Actinomycetia; Corynebacteriales; Mycobacteriaceae; Mycobacterium; Mycobacterium tuberculosis complex; Mycobacterium tuberculosis;	NCBI: 1773	4369505	99.94	0.00	Yes
NCBI Assembly	GCA_001381295.1	NCBI: cellular organisms; Bacteria; Terrabacteria group; Actinobacteria; Actinomycetia; Corynebacteriales; Mycobacteriaceae; Mycobacterium; Mycobacterium tuberculosis complex; Mycobacterium tuberculosis;	NCBI: 1773	4364308	99.94	0.00	Yes
NCBI Assembly	GCA_001391035.1	NCBI: cellular organisms; Bacteria; Terrabacteria group; Actinobacteria; Actinomycetia; Corynebacteriales; Mycobacteriaceae; Mycobacterium; Mycobacterium tuberculosis complex; Mycobacterium tuberculosis;	NCBI: 1773	4382892	99.89	0.00	Yes

NCBI Assembly	GCA_001380675.1	NCBI: cellular organisms; Bacteria; Terrabacteria group; Actinobacteria; Actinomycetia; Corynebacteriales; Mycobacteriaceae; Mycobacterium; Mycobacterium tuberculosis complex; Mycobacterium tuberculosis;	NCBI: 1773	4376141	99.94	0.17	Yes
NCBI Assembly	GCA_001380375.1	NCBI: cellular organisms; Bacteria; Terrabacteria group; Actinobacteria; Actinomycetia; Corynebacteriales; Mycobacteriaceae; Mycobacterium; Mycobacterium tuberculosis complex; Mycobacterium tuberculosis;	NCBI: 1773	4383185	99.94	0.33	Yes
NCBI Assembly	GCA_001381635.1	NCBI: cellular organisms; Bacteria; Terrabacteria group; Actinobacteria; Actinomycetia; Corynebacteriales; Mycobacteriaceae; Mycobacterium; Mycobacterium tuberculosis complex; Mycobacterium tuberculosis;	NCBI: 1773	4366708	99.94	0.00	Yes
NCBI Assembly	GCA_001384135.1	NCBI: cellular organisms; Bacteria; Terrabacteria group; Actinobacteria; Actinomycetia; Corynebacteriales; Mycobacteriaceae; Mycobacterium; Mycobacterium tuberculosis complex; Mycobacterium tuberculosis;	NCBI: 1773	4402748	99.94	0.00	Yes
NCBI Assembly	GCA_001385075.1	NCBI: cellular organisms; Bacteria; Terrabacteria group; Actinobacteria; Actinomycetia; Corynebacteriales; Mycobacteriaceae; Mycobacterium; Mycobacterium tuberculosis complex; Mycobacterium tuberculosis;	NCBI: 1773	4364413	99.94	0.00	Yes
NCBI Assembly	GCA_001384775.1	NCBI: cellular organisms; Bacteria; Terrabacteria group; Actinobacteria; Actinomycetia; Corynebacteriales; Mycobacteriaceae; Mycobacterium; Mycobacterium tuberculosis complex; Mycobacterium tuberculosis;	NCBI: 1773	4368860	99.94	0.00	Yes
NCBI Assembly	GCA_001385395.1	NCBI: cellular organisms; Bacteria; Terrabacteria group; Actinobacteria; Actinomycetia; Corynebacteriales; Mycobacteriaceae; Mycobacterium; Mycobacterium tuberculosis complex; Mycobacterium tuberculosis;	NCBI: 1773	4368199	99.94	0.17	Yes
NCBI Assembly	GCA_001385875.1	NCBI: cellular organisms; Bacteria; Terrabacteria group; Actinobacteria; Actinomycetia; Corynebacteriales; Mycobacteriaceae; Mycobacterium; Mycobacterium tuberculosis complex; Mycobacterium tuberculosis;	NCBI: 1773	4403394	99.94	0.33	Yes
NCBI Assembly	GCA_001384455.1	NCBI: cellular organisms; Bacteria; Terrabacteria group; Actinobacteria; Actinomycetia; Corynebacteriales; Mycobacteriaceae; Mycobacterium; Mycobacterium tuberculosis complex; Mycobacterium tuberculosis;	NCBI: 1773	4365200	99.94	0.00	Yes
NCBI Assembly	GCA_001381015.1	NCBI: cellular organisms; Bacteria; Terrabacteria group; Actinobacteria; Actinomycetia; Corynebacteriales; Mycobacteriaceae; Mycobacterium; Mycobacterium tuberculosis complex; Mycobacterium tuberculosis;	NCBI: 1773	4364499	99.94	0.00	Yes
NCBI Assembly	GCA_001381315.1	NCBI: cellular organisms; Bacteria; Terrabacteria group; Actinobacteria; Actinomycetia; Corynebacteriales; Mycobacteriaceae; Mycobacterium; Mycobacterium tuberculosis complex; Mycobacterium tuberculosis;	NCBI: 1773	4366903	99.94	0.00	Yes
NCBI Assembly	GCA_001391055.1	NCBI: cellular organisms; Bacteria; Terrabacteria group; Actinobacteria; Actinomycetia; Corynebacteriales; Mycobacteriaceae; Mycobacterium; Mycobacterium tuberculosis complex; Mycobacterium tuberculosis;	NCBI: 1773	4346192	99.94	0.00	Yes
NCBI Assembly	GCA_001380695.1	NCBI: cellular organisms; Bacteria; Terrabacteria group; Actinobacteria; Actinomycetia; Corynebacteriales; Mycobacteriaceae; Mycobacterium; Mycobacterium tuberculosis complex; Mycobacterium tuberculosis;	NCBI: 1773	4367571	99.94	0.17	Yes
NCBI Assembly	GCA_001380395.1	NCBI: cellular organisms; Bacteria; Terrabacteria group; Actinobacteria; Actinomycetia; Corynebacteriales; Mycobacteriaceae; Mycobacterium; Mycobacterium tuberculosis complex; Mycobacterium tuberculosis;	NCBI: 1773	4381668	99.94	0.33	Yes
NCBI Assembly	GCA_001381655.1	NCBI: cellular organisms; Bacteria; Terrabacteria group; Actinobacteria; Actinomycetia; Corynebacteriales; Mycobacteriaceae; Mycobacterium; Mycobacterium tuberculosis complex; Mycobacterium tuberculosis;	NCBI: 1773	4364649	99.94	0.17	Yes
NCBI Assembly	GCA_001384155.1	NCBI: cellular organisms; Bacteria; Terrabacteria group; Actinobacteria; Actinomycetia; Corynebacteriales; Mycobacteriaceae; Mycobacterium; Mycobacterium tuberculosis complex; Mycobacterium tuberculosis;	NCBI: 1773	4368138	99.94	0.17	Yes
NCBI Assembly	GCA_001384795.1	NCBI: cellular organisms; Bacteria; Terrabacteria group; Actinobacteria; Actinomycetia; Corynebacteriales; Mycobacteriaceae; Mycobacterium; Mycobacterium tuberculosis complex; Mycobacterium tuberculosis;	NCBI: 1773	4362636	99.94	0.00	Yes
NCBI Assembly	GCA_001385095.1	NCBI: cellular organisms; Bacteria; Terrabacteria group; Actinobacteria; Actinomycetia; Corynebacteriales; Mycobacteriaceae; Mycobacterium; Mycobacterium tuberculosis complex; Mycobacterium tuberculosis;	NCBI: 1773	4347462	99.94	0.00	Yes

NCBI Assembly	GCA_001385415.1	NCBI: cellular organisms; Bacteria; Terrabacteria group; Actinobacteria; Actinomycetia; Corynebacteriales; Mycobacteriaceae; Mycobacterium; Mycobacterium tuberculosis complex; Mycobacterium tuberculosis;	NCBI: 1773	4369592	99.94	0.00	Yes
NCBI Assembly	GCA_001385895.1	NCBI: cellular organisms; Bacteria; Terrabacteria group; Actinobacteria; Actinomycetia; Corynebacteriales; Mycobacteriaceae; Mycobacterium; Mycobacterium tuberculosis complex; Mycobacterium tuberculosis;	NCBI: 1773	4366333	99.94	0.17	Yes
NCBI Assembly	GCA_001384475.1	NCBI: cellular organisms; Bacteria; Terrabacteria group; Actinobacteria; Actinomycetia; Corynebacteriales; Mycobacteriaceae; Mycobacterium; Mycobacterium tuberculosis complex; Mycobacterium tuberculosis;	NCBI: 1773	4393223	99.94	0.33	Yes
NCBI Assembly	GCA_001381035.1	NCBI: cellular organisms; Bacteria; Terrabacteria group; Actinobacteria; Actinomycetia; Corynebacteriales; Mycobacteriaceae; Mycobacterium; Mycobacterium tuberculosis complex; Mycobacterium tuberculosis;	NCBI: 1773	4362833	99.94	0.00	Yes
NCBI Assembly	GCA_001381335.1	NCBI: cellular organisms; Bacteria; Terrabacteria group; Actinobacteria; Actinomycetia; Corynebacteriales; Mycobacteriaceae; Mycobacterium; Mycobacterium tuberculosis complex; Mycobacterium tuberculosis;	NCBI: 1773	4389181	99.94	0.00	Yes
NCBI Assembly	GCA_001391075.1	NCBI: cellular organisms; Bacteria; Terrabacteria group; Actinobacteria; Actinomycetia; Corynebacteriales; Mycobacteriaceae; Mycobacterium; Mycobacterium tuberculosis complex; Mycobacterium tuberculosis;	NCBI: 1773	4362380	99.94	0.00	Yes
NCBI Assembly	GCA_001380715.1	NCBI: cellular organisms; Bacteria; Terrabacteria group; Actinobacteria; Actinomycetia; Corynebacteriales; Mycobacteriaceae; Mycobacterium; Mycobacterium tuberculosis complex; Mycobacterium tuberculosis;	NCBI: 1773	4375523	99.94	0.04	Yes
NCBI Assembly	GCA_001380415.1	NCBI: cellular organisms; Bacteria; Terrabacteria group; Actinobacteria; Actinomycetia; Corynebacteriales; Mycobacteriaceae; Mycobacterium; Mycobacterium tuberculosis complex; Mycobacterium tuberculosis;	NCBI: 1773	4380962	99.94	0.00	Yes
NCBI Assembly	GCA_001384175.1	NCBI: cellular organisms; Bacteria; Terrabacteria group; Actinobacteria; Actinomycetia; Corynebacteriales; Mycobacteriaceae; Mycobacterium; Mycobacterium tuberculosis complex; Mycobacterium tuberculosis;	NCBI: 1773	4382645	99.94	0.00	Yes
NCBI Assembly	GCA_001381675.1	NCBI: cellular organisms; Bacteria; Terrabacteria group; Actinobacteria; Actinomycetia; Corynebacteriales; Mycobacteriaceae; Mycobacterium; Mycobacterium tuberculosis complex; Mycobacterium tuberculosis;	NCBI: 1773	4368419	99.94	0.00	Yes
NCBI Assembly	GCA_001384815.1	NCBI: cellular organisms; Bacteria; Terrabacteria group; Actinobacteria; Actinomycetia; Corynebacteriales; Mycobacteriaceae; Mycobacterium; Mycobacterium tuberculosis complex; Mycobacterium tuberculosis;	NCBI: 1773	4363014	99.94	0.17	Yes
NCBI Assembly	GCA_001385115.1	NCBI: cellular organisms; Bacteria; Terrabacteria group; Actinobacteria; Actinomycetia; Corynebacteriales; Mycobacteriaceae; Mycobacterium; Mycobacterium tuberculosis complex; Mycobacterium tuberculosis;	NCBI: 1773	4359756	99.94	0.17	Yes
NCBI Assembly	GCA_001385435.1	NCBI: cellular organisms; Bacteria; Terrabacteria group; Actinobacteria; Actinomycetia; Corynebacteriales; Mycobacteriaceae; Mycobacterium; Mycobacterium tuberculosis complex; Mycobacterium tuberculosis;	NCBI: 1773	4370759	99.94	0.17	Yes
NCBI Assembly	GCA_001381055.1	NCBI: cellular organisms; Bacteria; Terrabacteria group; Actinobacteria; Actinomycetia; Corynebacteriales; Mycobacteriaceae; Mycobacterium; Mycobacterium tuberculosis complex; Mycobacterium tuberculosis;	NCBI: 1773	4359618	99.94	0.17	Yes
NCBI Assembly	GCA_001384495.1	NCBI: cellular organisms; Bacteria; Terrabacteria group; Actinobacteria; Actinomycetia; Corynebacteriales; Mycobacteriaceae; Mycobacterium; Mycobacterium tuberculosis complex; Mycobacterium tuberculosis;	NCBI: 1773	4359423	99.94	0.00	Yes
NCBI Assembly	GCA_001385915.1	NCBI: cellular organisms; Bacteria; Terrabacteria group; Actinobacteria; Actinomycetia; Corynebacteriales; Mycobacteriaceae; Mycobacterium; Mycobacterium tuberculosis complex; Mycobacterium tuberculosis;	NCBI: 1773	4367081	99.94	0.00	Yes
NCBI Assembly	GCA_001381355.1	NCBI: cellular organisms; Bacteria; Terrabacteria group; Actinobacteria; Actinomycetia; Corynebacteriales; Mycobacteriaceae; Mycobacterium; Mycobacterium tuberculosis complex; Mycobacterium tuberculosis;	NCBI: 1773	4367838	99.94	0.00	Yes
NCBI Assembly	GCA_001391095.1	NCBI: cellular organisms; Bacteria; Terrabacteria group; Actinobacteria; Actinomycetia; Corynebacteriales; Mycobacteriaceae; Mycobacterium; Mycobacterium tuberculosis complex; Mycobacterium tuberculosis;	NCBI: 1773	4357662	99.94	0.00	Yes

NCBI Assembly	GCA_001380735.1	NCBI: cellular organisms; Bacteria; Terrabacteria group; Actinobacteria; Actinomycetia; Corynebacteriales; Mycobacteriaceae; Mycobacterium; Mycobacterium tuberculosis complex; Mycobacterium tuberculosis;	NCBI: 1773	4368566	99.94	0.00	Yes
NCBI Assembly	GCA_001380435.1	NCBI: cellular organisms; Bacteria; Terrabacteria group; Actinobacteria; Actinomycetia; Corynebacteriales; Mycobacteriaceae; Mycobacterium; Mycobacterium tuberculosis complex; Mycobacterium tuberculosis;	NCBI: 1773	4368019	99.94	0.00	Yes
NCBI Assembly	GCA_001384195.1	NCBI: cellular organisms; Bacteria; Terrabacteria group; Actinobacteria; Actinomycetia; Corynebacteriales; Mycobacteriaceae; Mycobacterium; Mycobacterium tuberculosis complex; Mycobacterium tuberculosis;	NCBI: 1773	4368197	99.94	0.00	Yes
NCBI Assembly	GCA_001381695.1	NCBI: cellular organisms; Bacteria; Terrabacteria group; Actinobacteria; Actinomycetia; Corynebacteriales; Mycobacteriaceae; Mycobacterium; Mycobacterium tuberculosis complex; Mycobacterium tuberculosis;	NCBI: 1773	4366856	99.94	0.00	Yes
NCBI Assembly	GCA_001384515.1	NCBI: cellular organisms; Bacteria; Terrabacteria group; Actinobacteria; Actinomycetia; Corynebacteriales; Mycobacteriaceae; Mycobacterium; Mycobacterium tuberculosis complex; Mycobacterium tuberculosis;	NCBI: 1773	4368492	99.94	0.00	Yes
NCBI Assembly	GCA_001385135.1	NCBI: cellular organisms; Bacteria; Terrabacteria group; Actinobacteria; Actinomycetia; Corynebacteriales; Mycobacteriaceae; Mycobacterium; Mycobacterium tuberculosis complex; Mycobacterium tuberculosis;	NCBI: 1773	4362971	99.94	0.00	Yes
NCBI Assembly	GCA_001385455.1	NCBI: cellular organisms; Bacteria; Terrabacteria group; Actinobacteria; Actinomycetia; Corynebacteriales; Mycobacteriaceae; Mycobacterium; Mycobacterium tuberculosis complex; Mycobacterium tuberculosis;	NCBI: 1773	4359937	99.94	0.00	Yes
NCBI Assembly	GCA_001384835.1	NCBI: cellular organisms; Bacteria; Terrabacteria group; Actinobacteria; Actinomycetia; Corynebacteriales; Mycobacteriaceae; Mycobacterium; Mycobacterium tuberculosis complex; Mycobacterium tuberculosis;	NCBI: 1773	4359789	99.94	0.00	Yes
NCBI Assembly	GCA_001381075.1	NCBI: cellular organisms; Bacteria; Terrabacteria group; Actinobacteria; Actinomycetia; Corynebacteriales; Mycobacteriaceae; Mycobacterium; Mycobacterium tuberculosis complex; Mycobacterium tuberculosis;	NCBI: 1773	4375263	99.94	0.00	Yes
NCBI Assembly	GCA_001381375.1	NCBI: cellular organisms; Bacteria; Terrabacteria group; Actinobacteria; Actinomycetia; Corynebacteriales; Mycobacteriaceae; Mycobacterium; Mycobacterium tuberculosis complex; Mycobacterium tuberculosis;	NCBI: 1773	4455802	99.94	1.22	Yes
NCBI Assembly	GCA_001382495.1	NCBI: cellular organisms; Bacteria; Terrabacteria group; Actinobacteria; Actinomycetia; Corynebacteriales; Mycobacteriaceae; Mycobacterium; Mycobacterium tuberculosis complex; Mycobacterium tuberculosis;	NCBI: 1773	4367731	99.94	0.00	Yes
NCBI Assembly	GCA_001391115.1	NCBI: cellular organisms; Bacteria; Terrabacteria group; Actinobacteria; Actinomycetia; Corynebacteriales; Mycobacteriaceae; Mycobacterium; Mycobacterium tuberculosis complex; Mycobacterium tuberculosis;	NCBI: 1773	4355240	99.94	0.00	Yes
NCBI Assembly	GCA_001380755.1	NCBI: cellular organisms; Bacteria; Terrabacteria group; Actinobacteria; Actinomycetia; Corynebacteriales; Mycobacteriaceae; Mycobacterium; Mycobacterium tuberculosis complex; Mycobacterium tuberculosis;	NCBI: 1773	4363301	99.94	0.00	Yes
NCBI Assembly	GCA_001380455.1	NCBI: cellular organisms; Bacteria; Terrabacteria group; Actinobacteria; Actinomycetia; Corynebacteriales; Mycobacteriaceae; Mycobacterium; Mycobacterium tuberculosis complex; Mycobacterium tuberculosis;	NCBI: 1773	4369806	99.94	0.00	Yes
NCBI Assembly	GCA_001384215.1	NCBI: cellular organisms; Bacteria; Terrabacteria group; Actinobacteria; Actinomycetia; Corynebacteriales; Mycobacteriaceae; Mycobacterium; Mycobacterium tuberculosis complex; Mycobacterium tuberculosis;	NCBI: 1773	4380888	99.94	0.00	Yes
NCBI Assembly	GCA_001381715.1	NCBI: cellular organisms; Bacteria; Terrabacteria group; Actinobacteria; Actinomycetia; Corynebacteriales; Mycobacteriaceae; Mycobacterium; Mycobacterium tuberculosis complex; Mycobacterium tuberculosis;	NCBI: 1773	4365145	99.94	0.00	Yes
NCBI Assembly	GCA_001381095.1	NCBI: cellular organisms; Bacteria; Terrabacteria group; Actinobacteria; Actinomycetia; Corynebacteriales; Mycobacteriaceae; Mycobacterium; Mycobacterium tuberculosis complex; Mycobacterium tuberculosis;	NCBI: 1773	4366862	99.94	0.33	Yes
NCBI Assembly	GCA_001385475.1	NCBI: cellular organisms; Bacteria; Terrabacteria group; Actinobacteria; Actinomycetia; Corynebacteriales; Mycobacteriaceae; Mycobacterium; Mycobacterium tuberculosis complex; Mycobacterium tuberculosis;	NCBI: 1773	4364503	99.94	0.00	Yes

NCBI Assembly	GCA_001385155.1	NCBI: cellular organisms; Bacteria; Terrabacteria group; Actinobacteria; Actinomycetia; Corynebacteriales; Mycobacteriaceae; Mycobacterium; Mycobacterium tuberculosis complex; Mycobacterium tuberculosis;	NCBI: 1773	4362788	99.94	0.17	Yes
NCBI Assembly	GCA_001384535.1	NCBI: cellular organisms; Bacteria; Terrabacteria group; Actinobacteria; Actinomycetia; Corynebacteriales; Mycobacteriaceae; Mycobacterium; Mycobacterium tuberculosis complex; Mycobacterium tuberculosis;	NCBI: 1773	4365941	99.94	0.00	Yes
NCBI Assembly	GCA_001384855.1	NCBI: cellular organisms; Bacteria; Terrabacteria group; Actinobacteria; Actinomycetia; Corynebacteriales; Mycobacteriaceae; Mycobacterium; Mycobacterium tuberculosis complex; Mycobacterium tuberculosis;	NCBI: 1773	4358075	99.94	0.00	Yes
NCBI Assembly	GCA_001381395.1	NCBI: cellular organisms; Bacteria; Terrabacteria group; Actinobacteria; Actinomycetia; Corynebacteriales; Mycobacteriaceae; Mycobacterium; Mycobacterium tuberculosis complex; Mycobacterium tuberculosis;	NCBI: 1773	4378409	99.94	0.00	Yes
NCBI Assembly	GCA_001382515.1	NCBI: cellular organisms; Bacteria; Terrabacteria group; Actinobacteria; Actinomycetia; Corynebacteriales; Mycobacteriaceae; Mycobacterium; Mycobacterium tuberculosis complex; Mycobacterium tuberculosis;	NCBI: 1773	4366167	99.94	0.17	Yes
NCBI Assembly	GCA_001391135.1	NCBI: cellular organisms; Bacteria; Terrabacteria group; Actinobacteria; Actinomycetia; Corynebacteriales; Mycobacteriaceae; Mycobacterium; Mycobacterium tuberculosis complex; Mycobacterium tuberculosis;	NCBI: 1773	4372554	99.94	0.00	Yes
NCBI Assembly	GCA_001380775.1	NCBI: cellular organisms; Bacteria; Terrabacteria group; Actinobacteria; Actinomycetia; Corynebacteriales; Mycobacteriaceae; Mycobacterium; Mycobacterium tuberculosis complex; Mycobacterium tuberculosis;	NCBI: 1773	4363637	99.94	0.17	Yes
NCBI Assembly	GCA_001380475.1	NCBI: cellular organisms; Bacteria; Terrabacteria group; Actinobacteria; Actinomycetia; Corynebacteriales; Mycobacteriaceae; Mycobacterium; Mycobacterium tuberculosis complex; Mycobacterium tuberculosis;	NCBI: 1773	4364007	99.94	0.00	Yes
NCBI Assembly	GCA_001381735.1	NCBI: cellular organisms; Bacteria; Terrabacteria group; Actinobacteria; Actinomycetia; Corynebacteriales; Mycobacteriaceae; Mycobacterium; Mycobacterium tuberculosis complex; Mycobacterium tuberculosis;	NCBI: 1773	4363680	99.94	0.00	Yes
NCBI Assembly	GCA_001384235.1	NCBI: cellular organisms; Bacteria; Terrabacteria group; Actinobacteria; Actinomycetia; Corynebacteriales; Mycobacteriaceae; Mycobacterium; Mycobacterium tuberculosis complex; Mycobacterium tuberculosis;	NCBI: 1773	4364198	99.94	0.00	Yes
NCBI Assembly	GCA_001385495.1	NCBI: cellular organisms; Bacteria; Terrabacteria group; Actinobacteria; Actinomycetia; Corynebacteriales; Mycobacteriaceae; Mycobacterium; Mycobacterium tuberculosis complex; Mycobacterium tuberculosis;	NCBI: 1773	4373754	99.94	0.00	Yes
NCBI Assembly	GCA_001381115.1	NCBI: cellular organisms; Bacteria; Terrabacteria group; Actinobacteria; Actinomycetia; Corynebacteriales; Mycobacteriaceae; Mycobacterium; Mycobacterium tuberculosis complex; Mycobacterium tuberculosis;	NCBI: 1773	4364999	99.94	0.00	Yes
NCBI Assembly	GCA_001384555.1	NCBI: cellular organisms; Bacteria; Terrabacteria group; Actinobacteria; Actinomycetia; Corynebacteriales; Mycobacteriaceae; Mycobacterium; Mycobacterium tuberculosis complex; Mycobacterium tuberculosis;	NCBI: 1773	4379279	99.94	0.00	Yes
NCBI Assembly	GCA_001385175.1	NCBI: cellular organisms; Bacteria; Terrabacteria group; Actinobacteria; Actinomycetia; Corynebacteriales; Mycobacteriaceae; Mycobacterium; Mycobacterium tuberculosis complex; Mycobacterium tuberculosis;	NCBI: 1773	4375157	99.61	0.00	Yes
NCBI Assembly	GCA_001384875.1	NCBI: cellular organisms; Bacteria; Terrabacteria group; Actinobacteria; Actinomycetia; Corynebacteriales; Mycobacteriaceae; Mycobacterium; Mycobacterium tuberculosis complex; Mycobacterium tuberculosis;	NCBI: 1773	4366706	99.94	0.00	Yes
NCBI Assembly	GCA_001381415.1	NCBI: cellular organisms; Bacteria; Terrabacteria group; Actinobacteria; Actinomycetia; Corynebacteriales; Mycobacteriaceae; Mycobacterium; Mycobacterium tuberculosis complex; Mycobacterium tuberculosis;	NCBI: 1773	4368325	99.94	0.00	Yes
NCBI Assembly	GCA_001382535.1	NCBI: cellular organisms; Bacteria; Terrabacteria group; Actinobacteria; Actinomycetia; Corynebacteriales; Mycobacteriaceae; Mycobacterium; Mycobacterium tuberculosis complex; Mycobacterium tuberculosis;	NCBI: 1773	4365892	99.94	0.00	Yes
NCBI Assembly	GCA_001391155.1	NCBI: cellular organisms; Bacteria; Terrabacteria group; Actinobacteria; Actinomycetia; Corynebacteriales; Mycobacteriaceae; Mycobacterium; Mycobacterium tuberculosis complex; Mycobacterium tuberculosis;	NCBI: 1773	4352422	99.94	0.00	Yes

NCBI Assembly	GCA_001380795.1	NCBI: cellular organisms; Bacteria; Terrabacteria group; Actinobacteria; Actinomycetia; Corynebacteriales; Mycobacteriaceae; Mycobacterium; Mycobacterium tuberculosis complex; Mycobacterium tuberculosis;	NCBI: 1773	4368027	99.94	0.00	Yes
NCBI Assembly	GCA_001380495.1	NCBI: cellular organisms; Bacteria; Terrabacteria group; Actinobacteria; Actinomycetia; Corynebacteriales; Mycobacteriaceae; Mycobacterium; Mycobacterium tuberculosis complex; Mycobacterium tuberculosis;	NCBI: 1773	4360729	99.94	0.00	Yes
NCBI Assembly	GCA_001381755.1	NCBI: cellular organisms; Bacteria; Terrabacteria group; Actinobacteria; Actinomycetia; Corynebacteriales; Mycobacteriaceae; Mycobacterium; Mycobacterium tuberculosis complex; Mycobacterium tuberculosis;	NCBI: 1773	4364408	99.94	0.00	Yes
NCBI Assembly	GCA_001381135.1	NCBI: cellular organisms; Bacteria; Terrabacteria group; Actinobacteria; Actinomycetia; Corynebacteriales; Mycobacteriaceae; Mycobacterium; Mycobacterium tuberculosis complex; Mycobacterium tuberculosis;	NCBI: 1773	4367336	99.94	0.00	Yes
NCBI Assembly	GCA_001384255.1	NCBI: cellular organisms; Bacteria; Terrabacteria group; Actinobacteria; Actinomycetia; Corynebacteriales; Mycobacteriaceae; Mycobacterium; Mycobacterium tuberculosis complex; Mycobacterium tuberculosis;	NCBI: 1773	4386916	99.94	0.00	Yes
NCBI Assembly	GCA_001385195.1	NCBI: cellular organisms; Bacteria; Terrabacteria group; Actinobacteria; Actinomycetia; Corynebacteriales; Mycobacteriaceae; Mycobacterium; Mycobacterium tuberculosis complex; Mycobacterium tuberculosis;	NCBI: 1773	4376598	99.61	0.33	Yes
NCBI Assembly	GCA_001381435.1	NCBI: cellular organisms; Bacteria; Terrabacteria group; Actinobacteria; Actinomycetia; Corynebacteriales; Mycobacteriaceae; Mycobacterium; Mycobacterium tuberculosis complex; Mycobacterium tuberculosis;	NCBI: 1773	4353831	99.94	0.00	Yes
NCBI Assembly	GCA_001384895.1	NCBI: cellular organisms; Bacteria; Terrabacteria group; Actinobacteria; Actinomycetia; Corynebacteriales; Mycobacteriaceae; Mycobacterium; Mycobacterium tuberculosis complex; Mycobacterium tuberculosis;	NCBI: 1773	4486174	99.94	1.39	Yes
NCBI Assembly	GCA_001385515.1	NCBI: cellular organisms; Bacteria; Terrabacteria group; Actinobacteria; Actinomycetia; Corynebacteriales; Mycobacteriaceae; Mycobacterium; Mycobacterium tuberculosis complex; Mycobacterium tuberculosis;	NCBI: 1773	4372991	99.94	0.33	Yes
NCBI Assembly	GCA_001384575.1	NCBI: cellular organisms; Bacteria; Terrabacteria group; Actinobacteria; Actinomycetia; Corynebacteriales; Mycobacteriaceae; Mycobacterium; Mycobacterium tuberculosis complex; Mycobacterium tuberculosis;	NCBI: 1773	4365095	99.94	0.00	Yes
NCBI Assembly	GCA_001382555.1	NCBI: cellular organisms; Bacteria; Terrabacteria group; Actinobacteria; Actinomycetia; Corynebacteriales; Mycobacteriaceae; Mycobacterium; Mycobacterium tuberculosis complex; Mycobacterium tuberculosis;	NCBI: 1773	4368557	99.94	0.00	Yes
NCBI Assembly	GCA_001391175.1	NCBI: cellular organisms; Bacteria; Terrabacteria group; Actinobacteria; Actinomycetia; Corynebacteriales; Mycobacteriaceae; Mycobacterium; Mycobacterium tuberculosis complex; Mycobacterium tuberculosis;	NCBI: 1773	4343045	99.94	0.00	Yes
NCBI Assembly	GCA_001380815.1	NCBI: cellular organisms; Bacteria; Terrabacteria group; Actinobacteria; Actinomycetia; Corynebacteriales; Mycobacteriaceae; Mycobacterium; Mycobacterium tuberculosis complex; Mycobacterium tuberculosis;	NCBI: 1773	4364995	99.94	0.00	Yes
NCBI Assembly	GCA_001380515.1	NCBI: cellular organisms; Bacteria; Terrabacteria group; Actinobacteria; Actinomycetia; Corynebacteriales; Mycobacteriaceae; Mycobacterium; Mycobacterium tuberculosis complex; Mycobacterium tuberculosis;	NCBI: 1773	4368063	99.94	0.00	Yes
NCBI Assembly	GCA_001385215.1	NCBI: cellular organisms; Bacteria; Terrabacteria group; Actinobacteria; Actinomycetia; Corynebacteriales; Mycobacteriaceae; Mycobacterium; Mycobacterium tuberculosis complex; Mycobacterium tuberculosis;	NCBI: 1773	4368220	99.94	0.00	Yes
NCBI Assembly	GCA_001381775.1	NCBI: cellular organisms; Bacteria; Terrabacteria group; Actinobacteria; Actinomycetia; Corynebacteriales; Mycobacteriaceae; Mycobacterium; Mycobacterium tuberculosis complex; Mycobacterium tuberculosis;	NCBI: 1773	4371893	99.94	0.33	Yes
NCBI Assembly	GCA_001381155.1	NCBI: cellular organisms; Bacteria; Terrabacteria group; Actinobacteria; Actinomycetia; Corynebacteriales; Mycobacteriaceae; Mycobacterium; Mycobacterium tuberculosis complex; Mycobacterium tuberculosis;	NCBI: 1773	4402434	99.94	0.00	Yes
NCBI Assembly	GCA_001384275.1	NCBI: cellular organisms; Bacteria; Terrabacteria group; Actinobacteria; Actinomycetia; Corynebacteriales; Mycobacteriaceae; Mycobacterium; Mycobacterium tuberculosis complex; Mycobacterium tuberculosis;	NCBI: 1773	4365139	99.94	0.00	Yes

NCBI Assembly	GCA_001381455.1	NCBI: cellular organisms; Bacteria; Terrabacteria group; Actinobacteria; Actinomycetia; Corynebacteriales; Mycobacteriaceae; Mycobacterium; Mycobacterium tuberculosis complex; Mycobacterium tuberculosis;	NCBI: 1773	4372027	99.94	0.00	Yes
NCBI Assembly	GCA_001385535.1	NCBI: cellular organisms; Bacteria; Terrabacteria group; Actinobacteria; Actinomycetia; Corynebacteriales; Mycobacteriaceae; Mycobacterium; Mycobacterium tuberculosis complex; Mycobacterium tuberculosis;	NCBI: 1773	4365020	99.78	0.00	Yes
NCBI Assembly	GCA_001384595.1	NCBI: cellular organisms; Bacteria; Terrabacteria group; Actinobacteria; Actinomycetia; Corynebacteriales; Mycobacteriaceae; Mycobacterium; Mycobacterium tuberculosis complex; Mycobacterium tuberculosis;	NCBI: 1773	4364623	99.94	0.00	Yes
NCBI Assembly	GCA_001384915.1	NCBI: cellular organisms; Bacteria; Terrabacteria group; Actinobacteria; Actinomycetia; Corynebacteriales; Mycobacteriaceae; Mycobacterium; Mycobacterium tuberculosis complex; Mycobacterium tuberculosis;	NCBI: 1773	4365079	99.94	0.00	Yes
NCBI Assembly	GCA_001382575.1	NCBI: cellular organisms; Bacteria; Terrabacteria group; Actinobacteria; Actinomycetia; Corynebacteriales; Mycobacteriaceae; Mycobacterium; Mycobacterium tuberculosis complex; Mycobacterium tuberculosis;	NCBI: 1773	4368066	99.94	0.17	Yes
NCBI Assembly	GCA_001391195.1	NCBI: cellular organisms; Bacteria; Terrabacteria group; Actinobacteria; Actinomycetia; Corynebacteriales; Mycobacteriaceae; Mycobacterium; Mycobacterium tuberculosis complex; Mycobacterium tuberculosis;	NCBI: 1773	4356254	99.94	0.00	Yes
NCBI Assembly	GCA_001380835.1	NCBI: cellular organisms; Bacteria; Terrabacteria group; Actinobacteria; Actinomycetia; Corynebacteriales; Mycobacteriaceae; Mycobacterium; Mycobacterium tuberculosis complex; Mycobacterium tuberculosis;	NCBI: 1773	4387210	99.94	0.00	Yes
NCBI Assembly	GCA_001380535.1	NCBI: cellular organisms; Bacteria; Terrabacteria group; Actinobacteria; Actinomycetia; Corynebacteriales; Mycobacteriaceae; Mycobacterium; Mycobacterium tuberculosis complex; Mycobacterium tuberculosis;	NCBI: 1773	4358791	99.94	0.00	Yes
NCBI Assembly	GCA_001385235.1	NCBI: cellular organisms; Bacteria; Terrabacteria group; Actinobacteria; Actinomycetia; Corynebacteriales; Mycobacteriaceae; Mycobacterium; Mycobacterium tuberculosis complex; Mycobacterium tuberculosis;	NCBI: 1773	4365226	99.94	0.17	Yes
NCBI Assembly	GCA_001381175.1	NCBI: cellular organisms; Bacteria; Terrabacteria group; Actinobacteria; Actinomycetia; Corynebacteriales; Mycobacteriaceae; Mycobacterium; Mycobacterium tuberculosis complex; Mycobacterium tuberculosis;	NCBI: 1773	4367285	99.94	0.00	Yes
NCBI Assembly	GCA_001381795.1	NCBI: cellular organisms; Bacteria; Terrabacteria group; Actinobacteria; Actinomycetia; Corynebacteriales; Mycobacteriaceae; Mycobacterium; Mycobacterium tuberculosis complex; Mycobacterium tuberculosis;	NCBI: 1773	4365929	99.94	0.00	Yes
NCBI Assembly	GCA_001384295.1	NCBI: cellular organisms; Bacteria; Terrabacteria group; Actinobacteria; Actinomycetia; Corynebacteriales; Mycobacteriaceae; Mycobacterium; Mycobacterium tuberculosis complex; Mycobacterium tuberculosis;	NCBI: 1773	4363906	99.94	0.00	Yes
NCBI Assembly	GCA_001381475.1	NCBI: cellular organisms; Bacteria; Terrabacteria group; Actinobacteria; Actinomycetia; Corynebacteriales; Mycobacteriaceae; Mycobacterium; Mycobacterium tuberculosis complex; Mycobacterium tuberculosis;	NCBI: 1773	4383350	99.94	0.00	Yes
NCBI Assembly	GCA_001385555.1	NCBI: cellular organisms; Bacteria; Terrabacteria group; Actinobacteria; Actinomycetia; Corynebacteriales; Mycobacteriaceae; Mycobacterium; Mycobacterium tuberculosis complex; Mycobacterium tuberculosis;	NCBI: 1773	4364517	99.94	0.00	Yes
NCBI Assembly	GCA_001384935.1	NCBI: cellular organisms; Bacteria; Terrabacteria group; Actinobacteria; Actinomycetia; Corynebacteriales; Mycobacteriaceae; Mycobacterium; Mycobacterium tuberculosis complex; Mycobacterium tuberculosis;	NCBI: 1773	4365823	99.94	0.00	Yes
NCBI Assembly	GCA_001382595.1	NCBI: cellular organisms; Bacteria; Terrabacteria group; Actinobacteria; Actinomycetia; Corynebacteriales; Mycobacteriaceae; Mycobacterium; Mycobacterium tuberculosis complex; Mycobacterium tuberculosis;	NCBI: 1773	4360713	99.94	0.00	Yes
NCBI Assembly	GCA_001384615.1	NCBI: cellular organisms; Bacteria; Terrabacteria group; Actinobacteria; Actinomycetia; Corynebacteriales; Mycobacteriaceae; Mycobacterium; Mycobacterium tuberculosis complex; Mycobacterium tuberculosis;	NCBI: 1773	4360513	99.94	0.00	Yes
NCBI Assembly	GCA_001380855.1	NCBI: cellular organisms; Bacteria; Terrabacteria group; Actinobacteria; Actinomycetia; Corynebacteriales; Mycobacteriaceae; Mycobacterium; Mycobacterium tuberculosis complex; Mycobacterium tuberculosis;	NCBI: 1773	4368588	99.94	0.00	Yes

NCBI Assembly	GCA_001391215.1	NCBI: cellular organisms; Bacteria; Terrabacteria group; Actinobacteria; Actinomycetia; Corynebacteriales; Mycobacteriaceae; Mycobacterium; Mycobacterium tuberculosis complex; Mycobacterium tuberculosis;	NCBI: 1773	4350790	99.94	0.00	Yes
NCBI Assembly	GCA_001380555.1	NCBI: cellular organisms; Bacteria; Terrabacteria group; Actinobacteria; Actinomycetia; Corynebacteriales; Mycobacteriaceae; Mycobacterium; Mycobacterium tuberculosis complex; Mycobacterium tuberculosis;	NCBI: 1773	4368075	99.94	0.00	Yes
NCBI Assembly	GCA_001385255.1	NCBI: cellular organisms; Bacteria; Terrabacteria group; Actinobacteria; Actinomycetia; Corynebacteriales; Mycobacteriaceae; Mycobacterium; Mycobacterium tuberculosis complex; Mycobacterium tuberculosis;	NCBI: 1773	4362468	99.94	0.00	Yes
NCBI Assembly	GCA_001381195.1	NCBI: cellular organisms; Bacteria; Terrabacteria group; Actinobacteria; Actinomycetia; Corynebacteriales; Mycobacteriaceae; Mycobacterium; Mycobacterium tuberculosis complex; Mycobacterium tuberculosis;	NCBI: 1773	4382551	99.94	0.00	Yes
NCBI Assembly	GCA_001381815.1	NCBI: cellular organisms; Bacteria; Terrabacteria group; Actinobacteria; Actinomycetia; Corynebacteriales; Mycobacteriaceae; Mycobacterium; Mycobacterium tuberculosis complex; Mycobacterium tuberculosis;	NCBI: 1773	4371958	99.94	0.17	Yes
NCBI Assembly	GCA_001384315.1	NCBI: cellular organisms; Bacteria; Terrabacteria group; Actinobacteria; Actinomycetia; Corynebacteriales; Mycobacteriaceae; Mycobacterium; Mycobacterium tuberculosis complex; Mycobacterium tuberculosis;	NCBI: 1773	4402675	99.94	0.00	Yes
NCBI Assembly	GCA_001381495.1	NCBI: cellular organisms; Bacteria; Terrabacteria group; Actinobacteria; Actinomycetia; Corynebacteriales; Mycobacteriaceae; Mycobacterium; Mycobacterium tuberculosis complex; Mycobacterium tuberculosis;	NCBI: 1773	4367573	99.94	0.00	Yes
NCBI Assembly	GCA_001384955.1	NCBI: cellular organisms; Bacteria; Terrabacteria group; Actinobacteria; Actinomycetia; Corynebacteriales; Mycobacteriaceae; Mycobacterium; Mycobacterium tuberculosis complex; Mycobacterium tuberculosis;	NCBI: 1773	4395558	99.94	0.00	Yes
NCBI Assembly	GCA_001385575.1	NCBI: cellular organisms; Bacteria; Terrabacteria group; Actinobacteria; Actinomycetia; Corynebacteriales; Mycobacteriaceae; Mycobacterium; Mycobacterium tuberculosis complex; Mycobacterium tuberculosis;	NCBI: 1773	4365967	99.94	0.00	Yes
NCBI Assembly	GCA_001384635.1	NCBI: cellular organisms; Bacteria; Terrabacteria group; Actinobacteria; Actinomycetia; Corynebacteriales; Mycobacteriaceae; Mycobacterium; Mycobacterium tuberculosis complex; Mycobacterium tuberculosis;	NCBI: 1773	4366236	99.94	0.00	Yes
NCBI Assembly	GCA_001382615.1	NCBI: cellular organisms; Bacteria; Terrabacteria group; Actinobacteria; Actinomycetia; Corynebacteriales; Mycobacteriaceae; Mycobacterium; Mycobacterium tuberculosis complex; Mycobacterium tuberculosis;	NCBI: 1773	4367654	99.94	0.00	Yes
NCBI Assembly	GCA_001380875.1	NCBI: cellular organisms; Bacteria; Terrabacteria group; Actinobacteria; Actinomycetia; Corynebacteriales; Mycobacteriaceae; Mycobacterium; Mycobacterium tuberculosis complex; Mycobacterium tuberculosis;	NCBI: 1773	4368344	99.94	0.00	Yes
NCBI Assembly	GCA_001391235.1	NCBI: cellular organisms; Bacteria; Terrabacteria group; Actinobacteria; Actinomycetia; Corynebacteriales; Mycobacteriaceae; Mycobacterium; Mycobacterium tuberculosis complex; Mycobacterium tuberculosis;	NCBI: 1773	4353703	99.94	0.00	Yes
NCBI Assembly	GCA_001380575.1	NCBI: cellular organisms; Bacteria; Terrabacteria group; Actinobacteria; Actinomycetia; Corynebacteriales; Mycobacteriaceae; Mycobacterium; Mycobacterium tuberculosis complex; Mycobacterium tuberculosis;	NCBI: 1773	4368091	99.94	0.17	Yes
NCBI Assembly	GCA_001381215.1	NCBI: cellular organisms; Bacteria; Terrabacteria group; Actinobacteria; Actinomycetia; Corynebacteriales; Mycobacteriaceae; Mycobacterium; Mycobacterium tuberculosis complex; Mycobacterium tuberculosis;	NCBI: 1773	4384023	99.94	0.00	Yes
NCBI Assembly	GCA_001385275.1	NCBI: cellular organisms; Bacteria; Terrabacteria group; Actinobacteria; Actinomycetia; Corynebacteriales; Mycobacteriaceae; Mycobacterium; Mycobacterium tuberculosis complex; Mycobacterium tuberculosis;	NCBI: 1773	4364733	99.94	0.17	Yes
NCBI Assembly	GCA_001381835.1	NCBI: cellular organisms; Bacteria; Terrabacteria group; Actinobacteria; Actinomycetia; Corynebacteriales; Mycobacteriaceae; Mycobacterium; Mycobacterium tuberculosis complex; Mycobacterium tuberculosis;	NCBI: 1773	4368299	99.94	0.00	Yes
NCBI Assembly	GCA_001381515.1	NCBI: cellular organisms; Bacteria; Terrabacteria group; Actinobacteria; Actinomycetia; Corynebacteriales; Mycobacteriaceae; Mycobacterium; Mycobacterium tuberculosis complex; Mycobacterium tuberculosis;	NCBI: 1773	4399441	99.94	0.00	Yes

NCBI Assembly	GCA_001384335.1	NCBI: cellular organisms; Bacteria; Terrabacteria group; Actinobacteria; Actinomycetia; Corynebacteriales; Mycobacteriaceae; Mycobacterium; Mycobacterium tuberculosis complex; Mycobacterium tuberculosis;	NCBI: 1773	4369280	99.94	0.00	Yes
NCBI Assembly	GCA_001384975.1	NCBI: cellular organisms; Bacteria; Terrabacteria group; Actinobacteria; Actinomycetia; Corynebacteriales; Mycobacteriaceae; Mycobacterium; Mycobacterium tuberculosis complex; Mycobacterium tuberculosis;	NCBI: 1773	4364151	99.94	0.00	Yes
NCBI Assembly	GCA_001385595.1	NCBI: cellular organisms; Bacteria; Terrabacteria group; Actinobacteria; Actinomycetia; Corynebacteriales; Mycobacteriaceae; Mycobacterium; Mycobacterium tuberculosis complex; Mycobacterium tuberculosis;	NCBI: 1773	4367581	99.94	0.00	Yes
NCBI Assembly	GCA_001384655.1	NCBI: cellular organisms; Bacteria; Terrabacteria group; Actinobacteria; Actinomycetia; Corynebacteriales; Mycobacteriaceae; Mycobacterium; Mycobacterium tuberculosis complex; Mycobacterium tuberculosis;	NCBI: 1773	4402712	99.94	0.17	Yes
NCBI Assembly	GCA_001382635.1	NCBI: cellular organisms; Bacteria; Terrabacteria group; Actinobacteria; Actinomycetia; Corynebacteriales; Mycobacteriaceae; Mycobacterium; Mycobacterium tuberculosis complex; Mycobacterium tuberculosis;	NCBI: 1773	4368338	99.94	0.00	Yes
NCBI Assembly	GCA_001380895.1	NCBI: cellular organisms; Bacteria; Terrabacteria group; Actinobacteria; Actinomycetia; Corynebacteriales; Mycobacteriaceae; Mycobacterium; Mycobacterium tuberculosis complex; Mycobacterium tuberculosis;	NCBI: 1773	4364590	99.94	0.00	Yes
NCBI Assembly	GCA_001391255.1	NCBI: cellular organisms; Bacteria; Terrabacteria group; Actinobacteria; Actinomycetia; Corynebacteriales; Mycobacteriaceae; Mycobacterium; Mycobacterium tuberculosis complex; Mycobacterium tuberculosis;	NCBI: 1773	4348831	99.94	0.00	Yes
NCBI Assembly	GCA_001380595.1	NCBI: cellular organisms; Bacteria; Terrabacteria group; Actinobacteria; Actinomycetia; Corynebacteriales; Mycobacteriaceae; Mycobacterium; Mycobacterium tuberculosis complex; Mycobacterium tuberculosis;	NCBI: 1773	4364234	99.94	0.00	Yes
NCBI Assembly	GCA_001390615.1	NCBI: cellular organisms; Bacteria; Terrabacteria group; Actinobacteria; Actinomycetia; Corynebacteriales; Mycobacteriaceae; Mycobacterium; Mycobacterium tuberculosis complex; Mycobacterium tuberculosis;	NCBI: 1773	4352207	99.94	0.00	Yes
NCBI Assembly	GCA_001385295.1	NCBI: cellular organisms; Bacteria; Terrabacteria group; Actinobacteria; Actinomycetia; Corynebacteriales; Mycobacteriaceae; Mycobacterium; Mycobacterium tuberculosis complex; Mycobacterium tuberculosis;	NCBI: 1773	4362798	99.94	0.00	Yes
NCBI Assembly	GCA_001390315.1	NCBI: cellular organisms; Bacteria; Terrabacteria group; Actinobacteria; Actinomycetia; Corynebacteriales; Mycobacteriaceae; Mycobacterium; Mycobacterium tuberculosis complex; Mycobacterium tuberculosis;	NCBI: 1773	4350911	99.94	0.00	Yes
NCBI Assembly	GCA_001381535.1	NCBI: cellular organisms; Bacteria; Terrabacteria group; Actinobacteria; Actinomycetia; Corynebacteriales; Mycobacteriaceae; Mycobacterium; Mycobacterium tuberculosis complex; Mycobacterium tuberculosis;	NCBI: 1773	4358538	99.94	0.00	Yes
NCBI Assembly	GCA_001382175.1	NCBI: cellular organisms; Bacteria; Terrabacteria group; Actinobacteria; Actinomycetia; Corynebacteriales; Mycobacteriaceae; Mycobacterium; Mycobacterium tuberculosis complex; Mycobacterium tuberculosis;	NCBI: 1773	4367360	99.94	0.17	Yes
NCBI Assembly	GCA_001380915.1	NCBI: cellular organisms; Bacteria; Terrabacteria group; Actinobacteria; Actinomycetia; Corynebacteriales; Mycobacteriaceae; Mycobacterium; Mycobacterium tuberculosis complex; Mycobacterium tuberculosis;	NCBI: 1773	4365006	99.94	0.00	Yes
NCBI Assembly	GCA_001382655.1	NCBI: cellular organisms; Bacteria; Terrabacteria group; Actinobacteria; Actinomycetia; Corynebacteriales; Mycobacteriaceae; Mycobacterium; Mycobacterium tuberculosis complex; Mycobacterium tuberculosis;	NCBI: 1773	4365932	99.94	0.00	Yes
NCBI Assembly	GCA_001391275.1	NCBI: cellular organisms; Bacteria; Terrabacteria group; Actinobacteria; Actinomycetia; Corynebacteriales; Mycobacteriaceae; Mycobacterium; Mycobacterium tuberculosis complex; Mycobacterium tuberculosis;	NCBI: 1773	4353519	99.94	0.00	Yes
NCBI Assembly	GCA_001390635.1	NCBI: cellular organisms; Bacteria; Terrabacteria group; Actinobacteria; Actinomycetia; Corynebacteriales; Mycobacteriaceae; Mycobacterium; Mycobacterium tuberculosis complex; Mycobacterium tuberculosis;	NCBI: 1773	4397573	99.88	0.00	Yes
NCBI Assembly	GCA_001382195.1	NCBI: cellular organisms; Bacteria; Terrabacteria group; Actinobacteria; Actinomycetia; Corynebacteriales; Mycobacteriaceae; Mycobacterium; Mycobacterium tuberculosis complex; Mycobacterium tuberculosis;	NCBI: 1773	4369144	99.94	0.00	Yes

NCBI Assembly	GCA_001390335.1	NCBI: cellular organisms; Bacteria; Terrabacteria group; Actinobacteria; Actinomycetia; Corynebacteriales; Mycobacteriaceae; Mycobacterium; Mycobacterium tuberculosis complex; Mycobacterium tuberculosis;	NCBI: 1773	4373472	99.94	0.00	Yes
NCBI Assembly	GCA_001382675.1	NCBI: cellular organisms; Bacteria; Terrabacteria group; Actinobacteria; Actinomycetia; Corynebacteriales; Mycobacteriaceae; Mycobacterium; Mycobacterium tuberculosis complex; Mycobacterium tuberculosis;	NCBI: 1773	4365892	99.94	0.00	Yes
NCBI Assembly	GCA_001391295.1	NCBI: cellular organisms; Bacteria; Terrabacteria group; Actinobacteria; Actinomycetia; Corynebacteriales; Mycobacteriaceae; Mycobacterium; Mycobacterium tuberculosis complex; Mycobacterium tuberculosis;	NCBI: 1773	4355210	99.94	0.00	Yes
NCBI Assembly	GCA_001390655.1	NCBI: cellular organisms; Bacteria; Terrabacteria group; Actinobacteria; Actinomycetia; Corynebacteriales; Mycobacteriaceae; Mycobacterium; Mycobacterium tuberculosis complex; Mycobacterium tuberculosis;	NCBI: 1773	4408802	99.94	0.00	Yes
NCBI Assembly	GCA_001390355.1	NCBI: cellular organisms; Bacteria; Terrabacteria group; Actinobacteria; Actinomycetia; Corynebacteriales; Mycobacteriaceae; Mycobacterium; Mycobacterium tuberculosis complex; Mycobacterium tuberculosis;	NCBI: 1773	4357634	99.86	0.00	Yes
NCBI Assembly	GCA_001382215.1	NCBI: cellular organisms; Bacteria; Terrabacteria group; Actinobacteria; Actinomycetia; Corynebacteriales; Mycobacteriaceae; Mycobacterium; Mycobacterium tuberculosis complex; Mycobacterium tuberculosis;	NCBI: 1773	4397477	99.94	0.00	Yes
NCBI Assembly	GCA_001382695.1	NCBI: cellular organisms; Bacteria; Terrabacteria group; Actinobacteria; Actinomycetia; Corynebacteriales; Mycobacteriaceae; Mycobacterium; Mycobacterium tuberculosis complex; Mycobacterium tuberculosis;	NCBI: 1773	4367028	99.94	0.00	Yes
NCBI Assembly	GCA_001391315.1	NCBI: cellular organisms; Bacteria; Terrabacteria group; Actinobacteria; Actinomycetia; Corynebacteriales; Mycobacteriaceae; Mycobacterium; Mycobacterium tuberculosis complex; Mycobacterium tuberculosis;	NCBI: 1773	4354695	99.94	0.00	Yes
NCBI Assembly	GCA_001390675.1	NCBI: cellular organisms; Bacteria; Terrabacteria group; Actinobacteria; Actinomycetia; Corynebacteriales; Mycobacteriaceae; Mycobacterium; Mycobacterium tuberculosis complex; Mycobacterium tuberculosis;	NCBI: 1773	4364435	99.94	0.00	Yes
NCBI Assembly	GCA_001390375.1	NCBI: cellular organisms; Bacteria; Terrabacteria group; Actinobacteria; Actinomycetia; Corynebacteriales; Mycobacteriaceae; Mycobacterium; Mycobacterium tuberculosis complex; Mycobacterium tuberculosis;	NCBI: 1773	4351665	99.88	0.00	Yes
NCBI Assembly	GCA_001382235.1	NCBI: cellular organisms; Bacteria; Terrabacteria group; Actinobacteria; Actinomycetia; Corynebacteriales; Mycobacteriaceae; Mycobacterium; Mycobacterium tuberculosis complex; Mycobacterium tuberculosis;	NCBI: 1773	4364759	99.94	0.00	Yes
NCBI Assembly	GCA_001382715.1	NCBI: cellular organisms; Bacteria; Terrabacteria group; Actinobacteria; Actinomycetia; Corynebacteriales; Mycobacteriaceae; Mycobacterium; Mycobacterium tuberculosis complex; Mycobacterium tuberculosis;	NCBI: 1773	4390557	99.94	0.00	Yes
NCBI Assembly	GCA_001391335.1	NCBI: cellular organisms; Bacteria; Terrabacteria group; Actinobacteria; Actinomycetia; Corynebacteriales; Mycobacteriaceae; Mycobacterium; Mycobacterium tuberculosis complex; Mycobacterium tuberculosis;	NCBI: 1773	4360331	99.94	0.00	Yes
NCBI Assembly	GCA_001390695.1	NCBI: cellular organisms; Bacteria; Terrabacteria group; Actinobacteria; Actinomycetia; Corynebacteriales; Mycobacteriaceae; Mycobacterium; Mycobacterium tuberculosis complex; Mycobacterium tuberculosis;	NCBI: 1773	4354239	99.83	0.00	Yes
NCBI Assembly	GCA_001390395.1	NCBI: cellular organisms; Bacteria; Terrabacteria group; Actinobacteria; Actinomycetia; Corynebacteriales; Mycobacteriaceae; Mycobacterium; Mycobacterium tuberculosis complex; Mycobacterium tuberculosis;	NCBI: 1773	4353719	99.94	0.00	Yes
NCBI Assembly	GCA_001382255.1	NCBI: cellular organisms; Bacteria; Terrabacteria group; Actinobacteria; Actinomycetia; Corynebacteriales; Mycobacteriaceae; Mycobacterium; Mycobacterium tuberculosis complex; Mycobacterium tuberculosis;	NCBI: 1773	4367055	99.94	0.17	Yes
NCBI Assembly	GCA_001382735.1	NCBI: cellular organisms; Bacteria; Terrabacteria group; Actinobacteria; Actinomycetia; Corynebacteriales; Mycobacteriaceae; Mycobacterium; Mycobacterium tuberculosis complex; Mycobacterium tuberculosis;	NCBI: 1773	4379862	99.94	0.00	Yes
NCBI Assembly	GCA_001391355.1	NCBI: cellular organisms; Bacteria; Terrabacteria group; Actinobacteria; Actinomycetia; Corynebacteriales; Mycobacteriaceae; Mycobacterium; Mycobacterium tuberculosis complex; Mycobacterium tuberculosis;	NCBI: 1773	4261748	99.94	0.00	Yes

NCBI Assembly	GCA_001390715.1	NCBI: cellular organisms; Bacteria; Terrabacteria group; Actinobacteria; Actinomycetia; Corynebacteriales; Mycobacteriaceae; Mycobacterium; Mycobacterium tuberculosis complex; Mycobacterium tuberculosis;	NCBI: 1773	4375029	99.94	0.11	Yes
NCBI Assembly	GCA_001390415.1	NCBI: cellular organisms; Bacteria; Terrabacteria group; Actinobacteria; Actinomycetia; Corynebacteriales; Mycobacteriaceae; Mycobacterium; Mycobacterium tuberculosis complex; Mycobacterium tuberculosis;	NCBI: 1773	4375923	99.94	0.11	Yes
NCBI Assembly	GCA_001382275.1	NCBI: cellular organisms; Bacteria; Terrabacteria group; Actinobacteria; Actinomycetia; Corynebacteriales; Mycobacteriaceae; Mycobacterium; Mycobacterium tuberculosis complex; Mycobacterium tuberculosis;	NCBI: 1773	4363391	99.94	0.00	Yes
NCBI Assembly	GCA_001382755.1	NCBI: cellular organisms; Bacteria; Terrabacteria group; Actinobacteria; Actinomycetia; Corynebacteriales; Mycobacteriaceae; Mycobacterium; Mycobacterium tuberculosis complex; Mycobacterium tuberculosis;	NCBI: 1773	4374403	99.94	0.17	Yes
NCBI Assembly	GCA_001391375.1	NCBI: cellular organisms; Bacteria; Terrabacteria group; Actinobacteria; Actinomycetia; Corynebacteriales; Mycobacteriaceae; Mycobacterium; Mycobacterium tuberculosis complex; Mycobacterium tuberculosis;	NCBI: 1773	4286093	99.79	0.00	Yes
NCBI Assembly	GCA_001390735.1	NCBI: cellular organisms; Bacteria; Terrabacteria group; Actinobacteria; Actinomycetia; Corynebacteriales; Mycobacteriaceae; Mycobacterium; Mycobacterium tuberculosis complex; Mycobacterium tuberculosis;	NCBI: 1773	4361921	99.94	0.00	Yes
NCBI Assembly	GCA_001390435.1	NCBI: cellular organisms; Bacteria; Terrabacteria group; Actinobacteria; Actinomycetia; Corynebacteriales; Mycobacteriaceae; Mycobacterium; Mycobacterium tuberculosis complex; Mycobacterium tuberculosis;	NCBI: 1773	4364017	99.61	0.00	Yes
NCBI Assembly	GCA_001382295.1	NCBI: cellular organisms; Bacteria; Terrabacteria group; Actinobacteria; Actinomycetia; Corynebacteriales; Mycobacteriaceae; Mycobacterium; Mycobacterium tuberculosis complex; Mycobacterium tuberculosis;	NCBI: 1773	4362807	99.94	0.00	Yes
NCBI Assembly	GCA_001382775.1	NCBI: cellular organisms; Bacteria; Terrabacteria group; Actinobacteria; Actinomycetia; Corynebacteriales; Mycobacteriaceae; Mycobacterium; Mycobacterium tuberculosis complex; Mycobacterium tuberculosis;	NCBI: 1773	4365714	99.94	0.00	Yes
NCBI Assembly	GCA_001391395.1	NCBI: cellular organisms; Bacteria; Terrabacteria group; Actinobacteria; Actinomycetia; Corynebacteriales; Mycobacteriaceae; Mycobacterium; Mycobacterium tuberculosis complex; Mycobacterium tuberculosis;	NCBI: 1773	4328877	99.94	0.00	Yes
NCBI Assembly	GCA_001390755.1	NCBI: cellular organisms; Bacteria; Terrabacteria group; Actinobacteria; Actinomycetia; Corynebacteriales; Mycobacteriaceae; Mycobacterium; Mycobacterium tuberculosis complex; Mycobacterium tuberculosis;	NCBI: 1773	4360194	99.94	0.00	Yes
NCBI Assembly	GCA_001390455.1	NCBI: cellular organisms; Bacteria; Terrabacteria group; Actinobacteria; Actinomycetia; Corynebacteriales; Mycobacteriaceae; Mycobacterium; Mycobacterium tuberculosis complex; Mycobacterium tuberculosis;	NCBI: 1773	4421609	99.86	0.67	Yes
NCBI Assembly	GCA_001382315.1	NCBI: cellular organisms; Bacteria; Terrabacteria group; Actinobacteria; Actinomycetia; Corynebacteriales; Mycobacteriaceae; Mycobacterium; Mycobacterium tuberculosis complex; Mycobacterium tuberculosis;	NCBI: 1773	4379984	99.94	0.00	Yes
NCBI Assembly	GCA_001382795.1	NCBI: cellular organisms; Bacteria; Terrabacteria group; Actinobacteria; Actinomycetia; Corynebacteriales; Mycobacteriaceae; Mycobacterium; Mycobacterium tuberculosis complex; Mycobacterium tuberculosis;	NCBI: 1773	4367593	99.94	0.00	Yes
NCBI Assembly	GCA_001391415.1	NCBI: cellular organisms; Bacteria; Terrabacteria group; Actinobacteria; Actinomycetia; Corynebacteriales; Mycobacteriaceae; Mycobacterium; Mycobacterium tuberculosis complex; Mycobacterium tuberculosis;	NCBI: 1773	4283668	99.78	0.00	Yes
NCBI Assembly	GCA_001390775.1	NCBI: cellular organisms; Bacteria; Terrabacteria group; Actinobacteria; Actinomycetia; Corynebacteriales; Mycobacteriaceae; Mycobacterium; Mycobacterium tuberculosis complex; Mycobacterium tuberculosis;	NCBI: 1773	4356523	99.94	0.00	Yes
NCBI Assembly	GCA_001382335.1	NCBI: cellular organisms; Bacteria; Terrabacteria group; Actinobacteria; Actinomycetia; Corynebacteriales; Mycobacteriaceae; Mycobacterium; Mycobacterium tuberculosis complex; Mycobacterium tuberculosis;	NCBI: 1773	4397137	99.94	0.00	Yes
NCBI Assembly	GCA_001390475.1	NCBI: cellular organisms; Bacteria; Terrabacteria group; Actinobacteria; Actinomycetia; Corynebacteriales; Mycobacteriaceae; Mycobacterium; Mycobacterium tuberculosis complex; Mycobacterium tuberculosis;	NCBI: 1773	4355348	99.94	0.00	Yes

NCBI Assembly	GCA_001391435.1	NCBI: cellular organisms; Bacteria; Terrabacteria group; Actinobacteria; Actinomycetia; Corynebacteriales; Mycobacteriaceae; Mycobacterium; Mycobacterium tuberculosis complex; Mycobacterium tuberculosis;	NCBI: 1773	4286241	99.94	0.00	Yes
NCBI Assembly	GCA_001390795.1	NCBI: cellular organisms; Bacteria; Terrabacteria group; Actinobacteria; Actinomycetia; Corynebacteriales; Mycobacteriaceae; Mycobacterium; Mycobacterium tuberculosis complex; Mycobacterium tuberculosis;	NCBI: 1773	4365040	99.94	0.00	Yes
NCBI Assembly	GCA_001382355.1	NCBI: cellular organisms; Bacteria; Terrabacteria group; Actinobacteria; Actinomycetia; Corynebacteriales; Mycobacteriaceae; Mycobacterium; Mycobacterium tuberculosis complex; Mycobacterium tuberculosis;	NCBI: 1773	4365492	99.94	0.00	Yes
NCBI Assembly	GCA_001390495.1	NCBI: cellular organisms; Bacteria; Terrabacteria group; Actinobacteria; Actinomycetia; Corynebacteriales; Mycobacteriaceae; Mycobacterium; Mycobacterium tuberculosis complex; Mycobacterium tuberculosis;	NCBI: 1773	4352260	99.88	0.00	Yes
NCBI Assembly	GCA_001391455.1	NCBI: cellular organisms; Bacteria; Terrabacteria group; Actinobacteria; Actinomycetia; Corynebacteriales; Mycobacteriaceae; Mycobacterium; Mycobacterium tuberculosis complex; Mycobacterium tuberculosis;	NCBI: 1773	4329095	99.90	0.28	Yes
NCBI Assembly	GCA_001390815.1	NCBI: cellular organisms; Bacteria; Terrabacteria group; Actinobacteria; Actinomycetia; Corynebacteriales; Mycobacteriaceae; Mycobacterium; Mycobacterium tuberculosis complex; Mycobacterium tuberculosis;	NCBI: 1773	4354448	99.94	0.00	Yes
NCBI Assembly	GCA_001382375.1	NCBI: cellular organisms; Bacteria; Terrabacteria group; Actinobacteria; Actinomycetia; Corynebacteriales; Mycobacteriaceae; Mycobacterium; Mycobacterium tuberculosis complex; Mycobacterium tuberculosis;	NCBI: 1773	4354888	99.94	0.00	Yes
NCBI Assembly	GCA_001390515.1	NCBI: cellular organisms; Bacteria; Terrabacteria group; Actinobacteria; Actinomycetia; Corynebacteriales; Mycobacteriaceae; Mycobacterium; Mycobacterium tuberculosis complex; Mycobacterium tuberculosis;	NCBI: 1773	4356624	99.94	0.00	Yes
NCBI Assembly	GCA_001391475.1	NCBI: cellular organisms; Bacteria; Terrabacteria group; Actinobacteria; Actinomycetia; Corynebacteriales; Mycobacteriaceae; Mycobacterium; Mycobacterium tuberculosis complex; Mycobacterium tuberculosis;	NCBI: 1773	4317573	99.94	0.00	Yes
NCBI Assembly	GCA_001390835.1	NCBI: cellular organisms; Bacteria; Terrabacteria group; Actinobacteria; Actinomycetia; Corynebacteriales; Mycobacteriaceae; Mycobacterium; Mycobacterium tuberculosis complex; Mycobacterium tuberculosis;	NCBI: 1773	4377923	99.57	0.00	Yes
NCBI Assembly	GCA_001382395.1	NCBI: cellular organisms; Bacteria; Terrabacteria group; Actinobacteria; Actinomycetia; Corynebacteriales; Mycobacteriaceae; Mycobacterium; Mycobacterium tuberculosis complex; Mycobacterium tuberculosis;	NCBI: 1773	4365733	99.94	0.00	Yes
NCBI Assembly	GCA_001390535.1	NCBI: cellular organisms; Bacteria; Terrabacteria group; Actinobacteria; Actinomycetia; Corynebacteriales; Mycobacteriaceae; Mycobacterium; Mycobacterium tuberculosis complex; Mycobacterium tuberculosis;	NCBI: 1773	4382289	99.94	0.00	Yes
NCBI Assembly	GCA_001391495.1	NCBI: cellular organisms; Bacteria; Terrabacteria group; Actinobacteria; Actinomycetia; Corynebacteriales; Mycobacteriaceae; Mycobacterium; Mycobacterium tuberculosis complex; Mycobacterium tuberculosis;	NCBI: 1773	4308866	99.77	0.00	Yes
NCBI Assembly	GCA_001390855.1	NCBI: cellular organisms; Bacteria; Terrabacteria group; Actinobacteria; Actinomycetia; Corynebacteriales; Mycobacteriaceae; Mycobacterium; Mycobacterium tuberculosis complex; Mycobacterium tuberculosis;	NCBI: 1773	4357742	99.94	0.00	Yes
NCBI Assembly	GCA_001382415.1	NCBI: cellular organisms; Bacteria; Terrabacteria group; Actinobacteria; Actinomycetia; Corynebacteriales; Mycobacteriaceae; Mycobacterium; Mycobacterium tuberculosis complex; Mycobacterium tuberculosis;	NCBI: 1773	4367257	99.94	0.00	Yes
NCBI Assembly	GCA_001390555.1	NCBI: cellular organisms; Bacteria; Terrabacteria group; Actinobacteria; Actinomycetia; Corynebacteriales; Mycobacteriaceae; Mycobacterium; Mycobacterium tuberculosis complex; Mycobacterium tuberculosis;	NCBI: 1773	4372370	99.94	0.11	Yes
NCBI Assembly	GCA_001391515.1	NCBI: cellular organisms; Bacteria; Terrabacteria group; Actinobacteria; Actinomycetia; Corynebacteriales; Mycobacteriaceae; Mycobacterium; Mycobacterium tuberculosis complex; Mycobacterium tuberculosis;	NCBI: 1773	4321172	99.94	0.00	Yes
NCBI Assembly	GCA_001390875.1	NCBI: cellular organisms; Bacteria; Terrabacteria group; Actinobacteria; Actinomycetia; Corynebacteriales; Mycobacteriaceae; Mycobacterium; Mycobacterium tuberculosis complex; Mycobacterium tuberculosis;	NCBI: 1773	4365009	99.88	0.00	Yes

NCBI Assembly	GCA_001382435.1	NCBI: cellular organisms; Bacteria; Terrabacteria group; Actinobacteria; Actinomycetia; Corynebacteriales; Mycobacteriaceae; Mycobacterium; Mycobacterium tuberculosis complex; Mycobacterium tuberculosis;	NCBI: 1773	4381269	99.94	0.00	Yes
NCBI Assembly	GCA_001390575.1	NCBI: cellular organisms; Bacteria; Terrabacteria group; Actinobacteria; Actinomycetia; Corynebacteriales; Mycobacteriaceae; Mycobacterium; Mycobacterium tuberculosis complex; Mycobacterium tuberculosis;	NCBI: 1773	4349866	99.94	0.00	Yes
NCBI Assembly	GCA_001391535.1	NCBI: cellular organisms; Bacteria; Terrabacteria group; Actinobacteria; Actinomycetia; Corynebacteriales; Mycobacteriaceae; Mycobacterium; Mycobacterium tuberculosis complex; Mycobacterium tuberculosis;	NCBI: 1773	4287876	99.94	0.00	Yes
NCBI Assembly	GCA_001390895.1	NCBI: cellular organisms; Bacteria; Terrabacteria group; Actinobacteria; Actinomycetia; Corynebacteriales; Mycobacteriaceae; Mycobacterium; Mycobacterium tuberculosis complex; Mycobacterium tuberculosis;	NCBI: 1773	4350972	99.94	0.00	Yes
NCBI Assembly	GCA_001382455.1	NCBI: cellular organisms; Bacteria; Terrabacteria group; Actinobacteria; Actinomycetia; Corynebacteriales; Mycobacteriaceae; Mycobacterium; Mycobacterium tuberculosis complex; Mycobacterium tuberculosis;	NCBI: 1773	4372883	99.94	0.00	Yes
NCBI Assembly	GCA_001390595.1	NCBI: cellular organisms; Bacteria; Terrabacteria group; Actinobacteria; Actinomycetia; Corynebacteriales; Mycobacteriaceae; Mycobacterium; Mycobacterium tuberculosis complex; Mycobacterium tuberculosis;	NCBI: 1773	4355665	99.94	0.33	Yes
NCBI Assembly	GCA_001390915.1	NCBI: cellular organisms; Bacteria; Terrabacteria group; Actinobacteria; Actinomycetia; Corynebacteriales; Mycobacteriaceae; Mycobacterium; Mycobacterium tuberculosis complex; Mycobacterium tuberculosis;	NCBI: 1773	4365825	99.69	0.00	Yes
NCBI Assembly	GCA_001382475.1	NCBI: cellular organisms; Bacteria; Terrabacteria group; Actinobacteria; Actinomycetia; Corynebacteriales; Mycobacteriaceae; Mycobacterium; Mycobacterium tuberculosis complex; Mycobacterium tuberculosis;	NCBI: 1773	4366641	99.94	0.17	Yes
NCBI Assembly	GCA_001665625.1	NCBI: cellular organisms; Bacteria; Terrabacteria group; Actinobacteria; Actinomycetia; Corynebacteriales; Mycobacteriaceae; Mycolicibacterium; Mycolicibacterium peregrinum;	NCBI: 43304	6751207	99.62	1.00	Yes
NCBI Assembly	GCA_001851425.1	NCBI: cellular organisms; Bacteria; Terrabacteria group; Actinobacteria; Actinomycetia; Corynebacteriales; Mycobacteriaceae; Mycobacterium; Mycobacterium tuberculosis complex; Mycobacterium tuberculosis;	NCBI: 1773	4398307	99.61	0.00	Yes
NCBI Assembly	GCA_001842615.1	NCBI: cellular organisms; Bacteria; Terrabacteria group; Actinobacteria; Actinomycetia; Corynebacteriales; Mycobacteriaceae; Mycobacterium; Mycobacterium tuberculosis complex; Mycobacterium tuberculosis;	NCBI: 1773	4383018	99.94	0.00	Yes
NCBI Assembly	GCA_001840435.1	NCBI: cellular organisms; Bacteria; Terrabacteria group; Actinobacteria; Actinomycetia; Corynebacteriales; Mycobacteriaceae; Mycobacterium; Mycobacterium tuberculosis complex; Mycobacterium tuberculosis;	NCBI: 1773	4388656	99.94	0.00	Yes
NCBI Assembly	GCA_001853005.1	NCBI: cellular organisms; Bacteria; Terrabacteria group; Actinobacteria; Actinomycetia; Corynebacteriales; Mycobacteriaceae; Mycobacterium; Mycobacterium tuberculosis complex; Mycobacterium tuberculosis;	NCBI: 1773	4401477	99.94	0.00	Yes
NCBI Assembly	GCA_001840125.1	NCBI: cellular organisms; Bacteria; Terrabacteria group; Actinobacteria; Actinomycetia; Corynebacteriales; Mycobacteriaceae; Mycobacterium; Mycobacterium tuberculosis complex; Mycobacterium tuberculosis;	NCBI: 1773	4384965	99.94	0.00	Yes
NCBI Assembly	GCA_001843005.1	NCBI: cellular organisms; Bacteria; Terrabacteria group; Actinobacteria; Actinomycetia; Corynebacteriales; Mycobacteriaceae; Mycobacterium; Mycobacterium tuberculosis complex; Mycobacterium tuberculosis;	NCBI: 1773	4405358	99.94	0.00	Yes
NCBI Assembly	GCA_001843925.1	NCBI: cellular organisms; Bacteria; Terrabacteria group; Actinobacteria; Actinomycetia; Corynebacteriales; Mycobacteriaceae; Mycobacterium; Mycobacterium tuberculosis complex; Mycobacterium tuberculosis;	NCBI: 1773	4399853	99.94	0.00	Yes
NCBI Assembly	GCA_001848005.1	NCBI: cellular organisms; Bacteria; Terrabacteria group; Actinobacteria; Actinomycetia; Corynebacteriales; Mycobacteriaceae; Mycobacterium; Mycobacterium tuberculosis complex; Mycobacterium tuberculosis;	NCBI: 1773	4324965	99.94	0.00	Yes
NCBI Assembly	GCA_001842015.1	NCBI: cellular organisms; Bacteria; Terrabacteria group; Actinobacteria; Actinomycetia; Corynebacteriales; Mycobacteriaceae; Mycobacterium; Mycobacterium tuberculosis complex; Mycobacterium tuberculosis;	NCBI: 1773	4382613	99.94	0.00	Yes

NCBI Assembly	GCA_001841375.1	NCBI: cellular organisms; Bacteria; Terrabacteria group; Actinobacteria; Actinomycetia; Corynebacteriales; Mycobacteriaceae; Mycobacterium; Mycobacterium tuberculosis complex; Mycobacterium tuberculosis;	NCBI: 1773	4390944	99.94	0.00	Yes
NCBI Assembly	GCA_001672685.1	NCBI: cellular organisms; Bacteria; Terrabacteria group; Actinobacteria; Actinomycetia; Corynebacteriales; Mycobacteriaceae; Mycobacterium; unclassified Mycobacterium;	NCBI: 1834133	5120731	98.77	3.51	Yes
NCBI Assembly	GCA_001665645.1	NCBI: cellular organisms; Bacteria; Terrabacteria group; Actinobacteria; Actinomycetia; Corynebacteriales; Mycobacteriaceae; Mycobacterium; unclassified Mycobacterium;	NCBI: 1834088	5273695	99.94	0.87	Yes
NCBI Assembly	GCA_001853025.1	NCBI: cellular organisms; Bacteria; Terrabacteria group; Actinobacteria; Actinomycetia; Corynebacteriales; Mycobacteriaceae; Mycobacterium; Mycobacterium tuberculosis complex; Mycobacterium tuberculosis;	NCBI: 1773	4357457	99.94	0.00	Yes
NCBI Assembly	GCA_001842665.1	NCBI: cellular organisms; Bacteria; Terrabacteria group; Actinobacteria; Actinomycetia; Corynebacteriales; Mycobacteriaceae; Mycobacterium; Mycobacterium tuberculosis complex; Mycobacterium tuberculosis;	NCBI: 1773	4396593	99.94	0.00	Yes
NCBI Assembly	GCA_001851435.1	NCBI: cellular organisms; Bacteria; Terrabacteria group; Actinobacteria; Actinomycetia; Corynebacteriales; Mycobacteriaceae; Mycobacterium; Mycobacterium tuberculosis complex; Mycobacterium tuberculosis;	NCBI: 1773	4398597	99.94	0.00	Yes
NCBI Assembly	GCA_001840465.1	NCBI: cellular organisms; Bacteria; Terrabacteria group; Actinobacteria; Actinomycetia; Corynebacteriales; Mycobacteriaceae; Mycobacterium; Mycobacterium tuberculosis complex; Mycobacterium tuberculosis;	NCBI: 1773	4391931	99.94	0.00	Yes
NCBI Assembly	GCA_001842045.1	NCBI: cellular organisms; Bacteria; Terrabacteria group; Actinobacteria; Actinomycetia; Corynebacteriales; Mycobacteriaceae; Mycobacterium; Mycobacterium tuberculosis complex; Mycobacterium tuberculosis;	NCBI: 1773	4395860	99.94	0.00	Yes
NCBI Assembly	GCA_001843025.1	NCBI: cellular organisms; Bacteria; Terrabacteria group; Actinobacteria; Actinomycetia; Corynebacteriales; Mycobacteriaceae; Mycobacterium; Mycobacterium tuberculosis complex; Mycobacterium tuberculosis;	NCBI: 1773	4393480	99.78	0.00	Yes
NCBI Assembly	GCA_001840145.1	NCBI: cellular organisms; Bacteria; Terrabacteria group; Actinobacteria; Actinomycetia; Corynebacteriales; Mycobacteriaceae; Mycobacterium; Mycobacterium tuberculosis complex; Mycobacterium tuberculosis;	NCBI: 1773	4392463	99.94	0.00	Yes
NCBI Assembly	GCA_001843935.1	NCBI: cellular organisms; Bacteria; Terrabacteria group; Actinobacteria; Actinomycetia; Corynebacteriales; Mycobacteriaceae; Mycobacterium; Mycobacterium tuberculosis complex; Mycobacterium tuberculosis;	NCBI: 1773	4378132	99.94	0.00	Yes
NCBI Assembly	GCA_001672745.1	NCBI: cellular organisms; Bacteria; Terrabacteria group; Actinobacteria; Actinomycetia; Corynebacteriales; Mycobacteriaceae; Mycobacterium; unclassified Mycobacterium;	NCBI: 1834126	5647745	99.22	1.88	Yes
NCBI Assembly	GCA_001848015.1	NCBI: cellular organisms; Bacteria; Terrabacteria group; Actinobacteria; Actinomycetia; Corynebacteriales; Mycobacteriaceae; Mycobacterium; Mycobacterium tuberculosis complex; Mycobacterium tuberculosis;	NCBI: 1773	4392616	99.94	0.00	Yes
NCBI Assembly	GCA_001841395.1	NCBI: cellular organisms; Bacteria; Terrabacteria group; Actinobacteria; Actinomycetia; Corynebacteriales; Mycobacteriaceae; Mycobacterium; Mycobacterium tuberculosis complex; Mycobacterium tuberculosis;	NCBI: 1773	4392037	99.61	0.00	Yes
NCBI Assembly	GCA_001665685.1	NCBI: cellular organisms; Bacteria; Terrabacteria group; Actinobacteria; Actinomycetia; Corynebacteriales; Mycobacteriaceae; Mycobacterium; unclassified Mycobacterium;	NCBI: 1834109	7847729	99.87	1.57	Yes
NCBI Assembly	GCA_001842675.1	NCBI: cellular organisms; Bacteria; Terrabacteria group; Actinobacteria; Actinomycetia; Corynebacteriales; Mycobacteriaceae; Mycobacterium; Mycobacterium tuberculosis complex; Mycobacterium tuberculosis;	NCBI: 1773	4385098	99.94	0.00	Yes
NCBI Assembly	GCA_001853035.1	NCBI: cellular organisms; Bacteria; Terrabacteria group; Actinobacteria; Actinomycetia; Corynebacteriales; Mycobacteriaceae; Mycobacterium; Mycobacterium tuberculosis complex; Mycobacterium tuberculosis;	NCBI: 1773	4352604	99.94	0.00	Yes
NCBI Assembly	GCA_001840475.1	NCBI: cellular organisms; Bacteria; Terrabacteria group; Actinobacteria; Actinomycetia; Corynebacteriales; Mycobacteriaceae; Mycobacterium; Mycobacterium tuberculosis complex; Mycobacterium tuberculosis;	NCBI: 1773	4410865	99.94	0.00	Yes

NCBI Assembly	GCA_001842055.1	NCBI: cellular organisms; Bacteria; Terrabacteria group; Actinobacteria; Actinomycetia; Corynebacteriales; Mycobacteriaceae; Mycobacterium; Mycobacterium tuberculosis complex; Mycobacterium tuberculosis;	NCBI: 1773	4383653	99.94	0.00	Yes
NCBI Assembly	GCA_001843965.1	NCBI: cellular organisms; Bacteria; Terrabacteria group; Actinobacteria; Actinomycetia; Corynebacteriales; Mycobacteriaceae; Mycobacterium; Mycobacterium tuberculosis complex; Mycobacterium tuberculosis;	NCBI: 1773	4415519	99.94	0.00	Yes
NCBI Assembly	GCA_001843045.1	NCBI: cellular organisms; Bacteria; Terrabacteria group; Actinobacteria; Actinomycetia; Corynebacteriales; Mycobacteriaceae; Mycobacterium; Mycobacterium tuberculosis complex; Mycobacterium tuberculosis;	NCBI: 1773	4398214	99.94	0.00	Yes
NCBI Assembly	GCA_001851465.1	NCBI: cellular organisms; Bacteria; Terrabacteria group; Actinobacteria; Actinomycetia; Corynebacteriales; Mycobacteriaceae; Mycobacterium; Mycobacterium tuberculosis complex; Mycobacterium tuberculosis;	NCBI: 1773	4375826	99.61	0.00	Yes
NCBI Assembly	GCA_001840155.1	NCBI: cellular organisms; Bacteria; Terrabacteria group; Actinobacteria; Actinomycetia; Corynebacteriales; Mycobacteriaceae; Mycobacterium; Mycobacterium tuberculosis complex; Mycobacterium tuberculosis;	NCBI: 1773	4383893	99.94	0.00	Yes
NCBI Assembly	GCA_001672755.1	NCBI: cellular organisms; Bacteria; Terrabacteria group; Actinobacteria; Actinomycetia; Corynebacteriales; Mycobacteriaceae; Mycobacterium; Mycobacterium avium complex (MAC); Mycobacterium colombiense;	NCBI: 339268	5545780	99.94	1.37	Yes
NCBI Assembly	GCA_001841425.1	NCBI: cellular organisms; Bacteria; Terrabacteria group; Actinobacteria; Actinomycetia; Corynebacteriales; Mycobacteriaceae; Mycobacterium; Mycobacterium tuberculosis complex; Mycobacterium tuberculosis;	NCBI: 1773	4377815	99.61	0.00	Yes
NCBI Assembly	GCA_001848045.1	NCBI: cellular organisms; Bacteria; Terrabacteria group; Actinobacteria; Actinomycetia; Corynebacteriales; Mycobacteriaceae; Mycobacterium; Mycobacterium tuberculosis complex; Mycobacterium tuberculosis;	NCBI: 1773	4392442	99.94	0.00	Yes
NCBI Assembly	GCA_001665755.1	NCBI: cellular organisms; Bacteria; Terrabacteria group; Actinobacteria; Actinomycetia; Corynebacteriales; Mycobacteriaceae; Mycolicibacterium; Mycolicibacterium elephantis;	NCBI: 81858	5297365	100.00	1.14	Yes
NCBI Assembly	GCA_001840505.1	NCBI: cellular organisms; Bacteria; Terrabacteria group; Actinobacteria; Actinomycetia; Corynebacteriales; Mycobacteriaceae; Mycobacterium; Mycobacterium tuberculosis complex; Mycobacterium tuberculosis;	NCBI: 1773	4398097	99.94	0.00	Yes
NCBI Assembly	GCA_001842085.1	NCBI: cellular organisms; Bacteria; Terrabacteria group; Actinobacteria; Actinomycetia; Corynebacteriales; Mycobacteriaceae; Mycobacterium; Mycobacterium tuberculosis complex; Mycobacterium tuberculosis;	NCBI: 1773	4404693	99.94	0.00	Yes
NCBI Assembly	GCA_001842745.1	NCBI: cellular organisms; Bacteria; Terrabacteria group; Actinobacteria; Actinomycetia; Corynebacteriales; Mycobacteriaceae; Mycobacterium; Mycobacterium tuberculosis complex; Mycobacterium tuberculosis;	NCBI: 1773	4399520	99.94	0.00	Yes
NCBI Assembly	GCA_001851475.1	NCBI: cellular organisms; Bacteria; Terrabacteria group; Actinobacteria; Actinomycetia; Corynebacteriales; Mycobacteriaceae; Mycobacterium; Mycobacterium tuberculosis complex; Mycobacterium tuberculosis;	NCBI: 1773	4390468	99.94	0.00	Yes
NCBI Assembly	GCA_001853045.1	NCBI: cellular organisms; Bacteria; Terrabacteria group; Actinobacteria; Actinomycetia; Corynebacteriales; Mycobacteriaceae; Mycobacterium; Mycobacterium tuberculosis complex; Mycobacterium tuberculosis;	NCBI: 1773	4399079	99.94	0.00	Yes
NCBI Assembly	GCA_001840165.1	NCBI: cellular organisms; Bacteria; Terrabacteria group; Actinobacteria; Actinomycetia; Corynebacteriales; Mycobacteriaceae; Mycobacterium; Mycobacterium tuberculosis complex; Mycobacterium tuberculosis;	NCBI: 1773	4408139	99.94	0.00	Yes
NCBI Assembly	GCA_001843985.1	NCBI: cellular organisms; Bacteria; Terrabacteria group; Actinobacteria; Actinomycetia; Corynebacteriales; Mycobacteriaceae; Mycobacterium; Mycobacterium tuberculosis complex; Mycobacterium tuberculosis;	NCBI: 1773	4383837	99.94	0.00	Yes
NCBI Assembly	GCA_001843055.1	NCBI: cellular organisms; Bacteria; Terrabacteria group; Actinobacteria; Actinomycetia; Corynebacteriales; Mycobacteriaceae; Mycobacterium; Mycobacterium tuberculosis complex; Mycobacterium tuberculosis;	NCBI: 1773	4398120	99.94	0.00	Yes
NCBI Assembly	GCA_001672775.1	NCBI: cellular organisms; Bacteria; Terrabacteria group; Actinobacteria; Actinomycetia; Corynebacteriales; Mycobacteriaceae; Mycobacterium; Mycobacterium kyorinense;	NCBI: 487514	4830972	99.77	2.14	Yes

NCBI Assembly	GCA_001848065.1	NCBI: cellular organisms; Bacteria; Terrabacteria group; Actinobacteria; Actinomycetia; Corynebacteriales; Mycobacteriaceae; Mycobacterium; Mycobacterium tuberculosis complex; Mycobacterium tuberculosis;	NCBI: 1773	4386609	99.94	0.00	Yes
NCBI Assembly	GCA_001841445.1	NCBI: cellular organisms; Bacteria; Terrabacteria group; Actinobacteria; Actinomycetia; Corynebacteriales; Mycobacteriaceae; Mycobacterium; Mycobacterium tuberculosis complex; Mycobacterium tuberculosis;	NCBI: 1773	4378007	99.94	0.00	Yes
NCBI Assembly	GCA_001665785.1	NCBI: cellular organisms; Bacteria; Terrabacteria group; Actinobacteria; Actinomycetia; Corynebacteriales; Mycobacteriaceae; Mycolicibacterium; Mycolicibacterium peregrinum;	NCBI: 43304	6795181	100.00	0.88	Yes
NCBI Assembly	GCA_001840515.1	NCBI: cellular organisms; Bacteria; Terrabacteria group; Actinobacteria; Actinomycetia; Corynebacteriales; Mycobacteriaceae; Mycobacterium; Mycobacterium tuberculosis complex; Mycobacterium tuberculosis;	NCBI: 1773	4395493	99.94	0.00	Yes
NCBI Assembly	GCA_001842765.1	NCBI: cellular organisms; Bacteria; Terrabacteria group; Actinobacteria; Actinomycetia; Corynebacteriales; Mycobacteriaceae; Mycobacterium; Mycobacterium tuberculosis complex; Mycobacterium tuberculosis;	NCBI: 1773	4411836	99.94	0.00	Yes
NCBI Assembly	GCA_001842095.1	NCBI: cellular organisms; Bacteria; Terrabacteria group; Actinobacteria; Actinomycetia; Corynebacteriales; Mycobacteriaceae; Mycobacterium; Mycobacterium tuberculosis complex; Mycobacterium tuberculosis;	NCBI: 1773	4411162	99.94	0.00	Yes
NCBI Assembly	GCA_001851505.1	NCBI: cellular organisms; Bacteria; Terrabacteria group; Actinobacteria; Actinomycetia; Corynebacteriales; Mycobacteriaceae; Mycobacterium; Mycobacterium tuberculosis complex; Mycobacterium tuberculosis;	NCBI: 1773	4400526	99.61	0.00	Yes
NCBI Assembly	GCA_001853085.1	NCBI: cellular organisms; Bacteria; Terrabacteria group; Actinobacteria; Actinomycetia; Corynebacteriales; Mycobacteriaceae; Mycobacterium; Mycobacterium tuberculosis complex; Mycobacterium tuberculosis;	NCBI: 1773	4400540	99.94	0.00	Yes
NCBI Assembly	GCA_001843085.1	NCBI: cellular organisms; Bacteria; Terrabacteria group; Actinobacteria; Actinomycetia; Corynebacteriales; Mycobacteriaceae; Mycobacterium; Mycobacterium tuberculosis complex; Mycobacterium tuberculosis;	NCBI: 1773	4414147	99.94	0.00	Yes
NCBI Assembly	GCA_001840205.1	NCBI: cellular organisms; Bacteria; Terrabacteria group; Actinobacteria; Actinomycetia; Corynebacteriales; Mycobacteriaceae; Mycobacterium; Mycobacterium tuberculosis complex; Mycobacterium tuberculosis;	NCBI: 1773	4408415	99.94	0.00	Yes
NCBI Assembly	GCA_001672815.1	NCBI: cellular organisms; Bacteria; Terrabacteria group; Actinobacteria; Actinomycetia; Corynebacteriales; Mycobacteriaceae; Mycobacterium; unclassified Mycobacterium;	NCBI: 1834151	6531408	99.44	2.59	Yes
NCBI Assembly	GCA_001844005.1	NCBI: cellular organisms; Bacteria; Terrabacteria group; Actinobacteria; Actinomycetia; Corynebacteriales; Mycobacteriaceae; Mycobacterium; Mycobacterium tuberculosis complex; Mycobacterium tuberculosis;	NCBI: 1773	4403939	99.94	0.00	Yes
NCBI Assembly	GCA_001841455.1	NCBI: cellular organisms; Bacteria; Terrabacteria group; Actinobacteria; Actinomycetia; Corynebacteriales; Mycobacteriaceae; Mycobacterium; Mycobacterium tuberculosis complex; Mycobacterium tuberculosis;	NCBI: 1773	4400047	99.61	0.00	Yes
NCBI Assembly	GCA_001848085.1	NCBI: cellular organisms; Bacteria; Terrabacteria group; Actinobacteria; Actinomycetia; Corynebacteriales; Mycobacteriaceae; Mycobacterium; Mycobacterium tuberculosis complex; Mycobacterium tuberculosis;	NCBI: 1773	4395963	99.94	0.00	Yes
NCBI Assembly	GCA_001665795.1	NCBI: cellular organisms; Bacteria; Terrabacteria group; Actinobacteria; Actinomycetia; Corynebacteriales; Mycobacteriaceae; Mycolicibacterium; Mycolicibacterium fortuitum;	NCBI: 1766	6542049	99.81	0.13	Yes
NCBI Assembly	GCA_001842775.1	NCBI: cellular organisms; Bacteria; Terrabacteria group; Actinobacteria; Actinomycetia; Corynebacteriales; Mycobacteriaceae; Mycobacterium; Mycobacterium tuberculosis complex; Mycobacterium tuberculosis;	NCBI: 1773	4393954	99.94	0.00	Yes
NCBI Assembly	GCA_001840545.1	NCBI: cellular organisms; Bacteria; Terrabacteria group; Actinobacteria; Actinomycetia; Corynebacteriales; Mycobacteriaceae; Mycobacterium; Mycobacterium tuberculosis complex; Mycobacterium tuberculosis;	NCBI: 1773	4372256	99.94	0.00	Yes
NCBI Assembly	GCA_001842105.1	NCBI: cellular organisms; Bacteria; Terrabacteria group; Actinobacteria; Actinomycetia; Corynebacteriales; Mycobacteriaceae; Mycobacterium; Mycobacterium tuberculosis complex; Mycobacterium tuberculosis;	NCBI: 1773	4394953	99.94	0.00	Yes

NCBI Assembly	GCA_001851515.1	NCBI: cellular organisms; Bacteria; Terrabacteria group; Actinobacteria; Actinomycetia; Corynebacteriales; Mycobacteriaceae; Mycobacterium; Mycobacterium tuberculosis complex; Mycobacterium tuberculosis;	NCBI: 1773	4398467	99.61	0.00	Yes
NCBI Assembly	GCA_001853105.1	NCBI: cellular organisms; Bacteria; Terrabacteria group; Actinobacteria; Actinomycetia; Corynebacteriales; Mycobacteriaceae; Mycobacterium; Mycobacterium tuberculosis complex; Mycobacterium tuberculosis;	NCBI: 1773	4391112	99.72	0.00	Yes
NCBI Assembly	GCA_001844015.1	NCBI: cellular organisms; Bacteria; Terrabacteria group; Actinobacteria; Actinomycetia; Corynebacteriales; Mycobacteriaceae; Mycobacterium; Mycobacterium tuberculosis complex; Mycobacterium tuberculosis;	NCBI: 1773	4402799	99.94	0.00	Yes
NCBI Assembly	GCA_001840225.1	NCBI: cellular organisms; Bacteria; Terrabacteria group; Actinobacteria; Actinomycetia; Corynebacteriales; Mycobacteriaceae; Mycobacterium; Mycobacterium tuberculosis complex; Mycobacterium tuberculosis;	NCBI: 1773	4402161	99.94	0.00	Yes
NCBI Assembly	GCA_001843095.1	NCBI: cellular organisms; Bacteria; Terrabacteria group; Actinobacteria; Actinomycetia; Corynebacteriales; Mycobacteriaceae; Mycobacterium; Mycobacterium tuberculosis complex; Mycobacterium tuberculosis;	NCBI: 1773	4398695	99.94	0.00	Yes
NCBI Assembly	GCA_001672835.1	NCBI: cellular organisms; Bacteria; Terrabacteria group; Actinobacteria; Actinomycetia; Corynebacteriales; Mycobacteriaceae; Mycolicibacterium; Mycolicibacterium fortuitum;	NCBI: 1766	6126360	99.43	1.48	Yes
NCBI Assembly	GCA_001848095.1	NCBI: cellular organisms; Bacteria; Terrabacteria group; Actinobacteria; Actinomycetia; Corynebacteriales; Mycobacteriaceae; Mycobacterium; Mycobacterium tuberculosis complex; Mycobacterium tuberculosis;	NCBI: 1773	4419950	99.94	0.00	Yes
NCBI Assembly	GCA_001841475.1	NCBI: cellular organisms; Bacteria; Terrabacteria group; Actinobacteria; Actinomycetia; Corynebacteriales; Mycobacteriaceae; Mycobacterium; Mycobacterium tuberculosis complex; Mycobacterium tuberculosis;	NCBI: 1773	4387777	99.94	0.00	Yes
NCBI Assembly	GCA_001665825.1	NCBI: cellular organisms; Bacteria; Terrabacteria group; Actinobacteria; Actinomycetia; Corynebacteriales; Mycobacteriaceae; Mycolicibacterium; Mycolicibacterium monacense;	NCBI: 85693	5627631	99.81	0.31	Yes
NCBI Assembly	GCA_001842785.1	NCBI: cellular organisms; Bacteria; Terrabacteria group; Actinobacteria; Actinomycetia; Corynebacteriales; Mycobacteriaceae; Mycobacterium; Mycobacterium tuberculosis complex; Mycobacterium tuberculosis;	NCBI: 1773	4398443	99.94	0.00	Yes
NCBI Assembly	GCA_001840555.1	NCBI: cellular organisms; Bacteria; Terrabacteria group; Actinobacteria; Actinomycetia; Corynebacteriales; Mycobacteriaceae; Mycobacterium; Mycobacterium tuberculosis complex; Mycobacterium tuberculosis;	NCBI: 1773	4398352	99.94	0.00	Yes
NCBI Assembly	GCA_001844045.1	NCBI: cellular organisms; Bacteria; Terrabacteria group; Actinobacteria; Actinomycetia; Corynebacteriales; Mycobacteriaceae; Mycobacterium; Mycobacterium tuberculosis complex; Mycobacterium tuberculosis;	NCBI: 1773	4395720	99.94	0.00	Yes
NCBI Assembly	GCA_001851545.1	NCBI: cellular organisms; Bacteria; Terrabacteria group; Actinobacteria; Actinomycetia; Corynebacteriales; Mycobacteriaceae; Mycobacterium; Mycobacterium tuberculosis complex; Mycobacterium tuberculosis;	NCBI: 1773	4342917	99.94	0.00	Yes
NCBI Assembly	GCA_001840235.1	NCBI: cellular organisms; Bacteria; Terrabacteria group; Actinobacteria; Actinomycetia; Corynebacteriales; Mycobacteriaceae; Mycobacterium; Mycobacterium tuberculosis complex; Mycobacterium tuberculosis;	NCBI: 1773	4405523	99.94	0.00	Yes
NCBI Assembly	GCA_001843105.1	NCBI: cellular organisms; Bacteria; Terrabacteria group; Actinobacteria; Actinomycetia; Corynebacteriales; Mycobacteriaceae; Mycobacterium; Mycobacterium tuberculosis complex; Mycobacterium tuberculosis;	NCBI: 1773	4353197	99.94	0.00	Yes
NCBI Assembly	GCA_001853125.1	NCBI: cellular organisms; Bacteria; Terrabacteria group; Actinobacteria; Actinomycetia; Corynebacteriales; Mycobacteriaceae; Mycobacterium; Mycobacterium tuberculosis complex; Mycobacterium tuberculosis;	NCBI: 1773	4401231	99.94	0.00	Yes
NCBI Assembly	GCA_001842135.1	NCBI: cellular organisms; Bacteria; Terrabacteria group; Actinobacteria; Actinomycetia; Corynebacteriales; Mycobacteriaceae; Mycobacterium; Mycobacterium tuberculosis complex; Mycobacterium tuberculosis;	NCBI: 1773	4397743	99.94	0.00	Yes
NCBI Assembly	GCA_001672845.1	NCBI: cellular organisms; Bacteria; Terrabacteria group; Actinobacteria; Actinomycetia; Corynebacteriales; Mycobacteriaceae; Mycolicibacterium; Mycolicibacterium fortuitum;	NCBI: 1766	6352455	99.81	0.88	Yes

NCBI Assembly	GCA_001848115.1	NCBI: cellular organisms; Bacteria; Terrabacteria group; Actinobacteria; Actinomycetia; Corynebacteriales; Mycobacteriaceae; Mycobacterium; Mycobacterium tuberculosis complex; Mycobacterium tuberculosis;	NCBI: 1773	4393786	99.94	0.00	Yes
NCBI Assembly	GCA_001841505.1	NCBI: cellular organisms; Bacteria; Terrabacteria group; Actinobacteria; Actinomycetia; Corynebacteriales; Mycobacteriaceae; Mycobacterium; Mycobacterium tuberculosis complex; Mycobacterium tuberculosis;	NCBI: 1773	4387145	99.94	0.00	Yes
NCBI Assembly	GCA_001665835.1	NCBI: cellular organisms; Bacteria; Terrabacteria group; Actinobacteria; Actinomycetia; Corynebacteriales; Mycobacteriaceae; Mycobacterium; Mycobacterium avium complex (MAC); Mycobacterium colombiense;	NCBI: 339268	5547515	99.94	0.47	Yes
NCBI Assembly	GCA_001840575.1	NCBI: cellular organisms; Bacteria; Terrabacteria group; Actinobacteria; Actinomycetia; Corynebacteriales; Mycobacteriaceae; Mycobacterium; Mycobacterium tuberculosis complex; Mycobacterium tuberculosis;	NCBI: 1773	4399319	99.61	0.00	Yes
NCBI Assembly	GCA_001842825.1	NCBI: cellular organisms; Bacteria; Terrabacteria group; Actinobacteria; Actinomycetia; Corynebacteriales; Mycobacteriaceae; Mycobacterium; Mycobacterium tuberculosis complex; Mycobacterium tuberculosis;	NCBI: 1773	4401083	99.94	0.00	Yes
NCBI Assembly	GCA_001842165.1	NCBI: cellular organisms; Bacteria; Terrabacteria group; Actinobacteria; Actinomycetia; Corynebacteriales; Mycobacteriaceae; Mycobacterium; Mycobacterium tuberculosis complex; Mycobacterium tuberculosis;	NCBI: 1773	4413057	99.94	0.00	Yes
NCBI Assembly	GCA_001843145.1	NCBI: cellular organisms; Bacteria; Terrabacteria group; Actinobacteria; Actinomycetia; Corynebacteriales; Mycobacteriaceae; Mycobacterium; Mycobacterium tuberculosis complex; Mycobacterium tuberculosis;	NCBI: 1773	4406908	99.94	0.00	Yes
NCBI Assembly	GCA_001840245.1	NCBI: cellular organisms; Bacteria; Terrabacteria group; Actinobacteria; Actinomycetia; Corynebacteriales; Mycobacteriaceae; Mycobacterium; Mycobacterium tuberculosis complex; Mycobacterium tuberculosis;	NCBI: 1773	4378020	99.88	0.00	Yes
NCBI Assembly	GCA_001844065.1	NCBI: cellular organisms; Bacteria; Terrabacteria group; Actinobacteria; Actinomycetia; Corynebacteriales; Mycobacteriaceae; Mycobacterium; Mycobacterium tuberculosis complex; Mycobacterium tuberculosis;	NCBI: 1773	4378050	99.94	0.00	Yes
NCBI Assembly	GCA_001672855.1	NCBI: cellular organisms; Bacteria; Terrabacteria group; Actinobacteria; Actinomycetia; Corynebacteriales; Mycobacteriaceae; Mycolicibacterium; Mycolicibacterium fortuitum;	NCBI: 1766	6228643	99.81	1.26	Yes
NCBI Assembly	GCA_001848145.1	NCBI: cellular organisms; Bacteria; Terrabacteria group; Actinobacteria; Actinomycetia; Corynebacteriales; Mycobacteriaceae; Mycobacterium; Mycobacterium tuberculosis complex; Mycobacterium tuberculosis;	NCBI: 1773	4384915	99.94	0.00	Yes
NCBI Assembly	GCA_001851555.1	NCBI: cellular organisms; Bacteria; Terrabacteria group; Actinobacteria; Actinomycetia; Corynebacteriales; Mycobacteriaceae; Mycobacterium; Mycobacterium tuberculosis complex; Mycobacterium tuberculosis;	NCBI: 1773	4387223	99.94	0.00	Yes
NCBI Assembly	GCA_001853135.1	NCBI: cellular organisms; Bacteria; Terrabacteria group; Actinobacteria; Actinomycetia; Corynebacteriales; Mycobacteriaceae; Mycobacterium; Mycobacterium tuberculosis complex; Mycobacterium tuberculosis;	NCBI: 1773	4403023	99.94	0.00	Yes
NCBI Assembly	GCA_001841525.1	NCBI: cellular organisms; Bacteria; Terrabacteria group; Actinobacteria; Actinomycetia; Corynebacteriales; Mycobacteriaceae; Mycobacterium; Mycobacterium tuberculosis complex; Mycobacterium tuberculosis;	NCBI: 1773	4401711	99.94	0.00	Yes
NCBI Assembly	GCA_001665875.1	NCBI: cellular organisms; Bacteria; Terrabacteria group; Actinobacteria; Actinomycetia; Corynebacteriales; Mycobacteriaceae; Mycobacterium; unclassified Mycobacterium;	NCBI: 1834089	5383904	99.94	0.73	Yes
NCBI Assembly	GCA_001840595.1	NCBI: cellular organisms; Bacteria; Terrabacteria group; Actinobacteria; Actinomycetia; Corynebacteriales; Mycobacteriaceae; Mycobacterium; Mycobacterium tuberculosis complex; Mycobacterium tuberculosis;	NCBI: 1773	4395299	99.61	0.00	Yes
NCBI Assembly	GCA_001842175.1	NCBI: cellular organisms; Bacteria; Terrabacteria group; Actinobacteria; Actinomycetia; Corynebacteriales; Mycobacteriaceae; Mycobacterium; Mycobacterium tuberculosis complex; Mycobacterium tuberculosis;	NCBI: 1773	4394173	99.94	0.00	Yes
NCBI Assembly	GCA_001843165.1	NCBI: cellular organisms; Bacteria; Terrabacteria group; Actinobacteria; Actinomycetia; Corynebacteriales; Mycobacteriaceae; Mycobacterium; Mycobacterium tuberculosis complex; Mycobacterium tuberculosis;	NCBI: 1773	4378130	99.94	0.00	Yes

NCBI Assembly	GCA_001840285.1	NCBI: cellular organisms; Bacteria; Terrabacteria group; Actinobacteria; Actinomycetia; Corynebacteriales; Mycobacteriaceae; Mycobacterium; Mycobacterium tuberculosis complex; Mycobacterium tuberculosis;	NCBI: 1773	4399787	99.94	0.00	Yes
NCBI Assembly	GCA_001851575.1	NCBI: cellular organisms; Bacteria; Terrabacteria group; Actinobacteria; Actinomycetia; Corynebacteriales; Mycobacteriaceae; Mycobacterium; Mycobacterium tuberculosis complex; Mycobacterium tuberculosis;	NCBI: 1773	4376669	99.94	0.00	Yes
NCBI Assembly	GCA_001672895.1	NCBI: cellular organisms; Bacteria; Terrabacteria group; Actinobacteria; Actinomycetia; Corynebacteriales; Mycobacteriaceae; Mycobacterium; unclassified Mycobacterium;	NCBI: 1834149	4619780	99.77	0.68	Yes
NCBI Assembly	GCA_001842845.1	NCBI: cellular organisms; Bacteria; Terrabacteria group; Actinobacteria; Actinomycetia; Corynebacteriales; Mycobacteriaceae; Mycobacterium; Mycobacterium tuberculosis complex; Mycobacterium tuberculosis;	NCBI: 1773	4382675	99.94	0.00	Yes
NCBI Assembly	GCA_001848165.1	NCBI: cellular organisms; Bacteria; Terrabacteria group; Actinobacteria; Actinomycetia; Corynebacteriales; Mycobacteriaceae; Mycobacterium; Mycobacterium tuberculosis complex; Mycobacterium tuberculosis;	NCBI: 1773	4404418	99.94	0.00	Yes
NCBI Assembly	GCA_001853165.1	NCBI: cellular organisms; Bacteria; Terrabacteria group; Actinobacteria; Actinomycetia; Corynebacteriales; Mycobacteriaceae; Mycobacterium; Mycobacterium tuberculosis complex; Mycobacterium tuberculosis;	NCBI: 1773	4377711	99.94	0.00	Yes
NCBI Assembly	GCA_001844085.1	NCBI: cellular organisms; Bacteria; Terrabacteria group; Actinobacteria; Actinomycetia; Corynebacteriales; Mycobacteriaceae; Mycobacterium; Mycobacterium tuberculosis complex; Mycobacterium tuberculosis;	NCBI: 1773	4388386	99.61	0.00	Yes
NCBI Assembly	GCA_001841545.1	NCBI: cellular organisms; Bacteria; Terrabacteria group; Actinobacteria; Actinomycetia; Corynebacteriales; Mycobacteriaceae; Mycobacterium; Mycobacterium tuberculosis complex; Mycobacterium tuberculosis;	NCBI: 1773	4367967	99.94	0.00	Yes
NCBI Assembly	GCA_001666745.1	NCBI: cellular organisms; Bacteria; Terrabacteria group; Actinobacteria; Actinomycetia; Corynebacteriales; Mycobacteriaceae; Mycobacterium; unclassified Mycobacterium;	NCBI: 1834137	5733507	99.94	2.59	Yes
NCBI Assembly	GCA_001840625.1	NCBI: cellular organisms; Bacteria; Terrabacteria group; Actinobacteria; Actinomycetia; Corynebacteriales; Mycobacteriaceae; Mycobacterium; Mycobacterium tuberculosis complex; Mycobacterium tuberculosis;	NCBI: 1773	4389758	99.61	0.00	Yes
NCBI Assembly	GCA_001840305.1	NCBI: cellular organisms; Bacteria; Terrabacteria group; Actinobacteria; Actinomycetia; Corynebacteriales; Mycobacteriaceae; Mycobacterium; Mycobacterium tuberculosis complex; Mycobacterium tuberculosis;	NCBI: 1773	4392381	99.94	0.00	Yes
NCBI Assembly	GCA_001843175.1	NCBI: cellular organisms; Bacteria; Terrabacteria group; Actinobacteria; Actinomycetia; Corynebacteriales; Mycobacteriaceae; Mycobacterium; Mycobacterium tuberculosis complex; Mycobacterium tuberculosis;	NCBI: 1773	4396691	99.94	0.00	Yes
NCBI Assembly	GCA_001672915.1	NCBI: cellular organisms; Bacteria; Terrabacteria group; Actinobacteria; Actinomycetia; Corynebacteriales; Mycobacteriaceae; Mycobacterium; unclassified Mycobacterium;	NCBI: 1856862	6098823	99.61	1.30	Yes
NCBI Assembly	GCA_001842195.1	NCBI: cellular organisms; Bacteria; Terrabacteria group; Actinobacteria; Actinomycetia; Corynebacteriales; Mycobacteriaceae; Mycobacterium; Mycobacterium tuberculosis complex; Mycobacterium tuberculosis;	NCBI: 1773	4404310	99.94	0.00	Yes
NCBI Assembly	GCA_001851595.1	NCBI: cellular organisms; Bacteria; Terrabacteria group; Actinobacteria; Actinomycetia; Corynebacteriales; Mycobacteriaceae; Mycobacterium; Mycobacterium tuberculosis complex; Mycobacterium tuberculosis;	NCBI: 1773	4395592	99.94	0.00	Yes
NCBI Assembly	GCA_001853185.1	NCBI: cellular organisms; Bacteria; Terrabacteria group; Actinobacteria; Actinomycetia; Corynebacteriales; Mycobacteriaceae; Mycobacterium; Mycobacterium tuberculosis complex; Mycobacterium tuberculosis;	NCBI: 1773	4392824	99.94	0.00	Yes
NCBI Assembly	GCA_001842865.1	NCBI: cellular organisms; Bacteria; Terrabacteria group; Actinobacteria; Actinomycetia; Corynebacteriales; Mycobacteriaceae; Mycobacterium; Mycobacterium tuberculosis complex; Mycobacterium tuberculosis;	NCBI: 1773	4383671	99.94	0.00	Yes
NCBI Assembly	GCA_001844095.1	NCBI: cellular organisms; Bacteria; Terrabacteria group; Actinobacteria; Actinomycetia; Corynebacteriales; Mycobacteriaceae; Mycobacterium; Mycobacterium tuberculosis complex; Mycobacterium tuberculosis;	NCBI: 1773	4400249	99.94	0.00	Yes

NCBI Assembly	GCA_001848175.1	NCBI: cellular organisms; Bacteria; Terrabacteria group; Actinobacteria; Actinomycetia; Corynebacteriales; Mycobacteriaceae; Mycobacterium; Mycobacterium tuberculosis complex; Mycobacterium tuberculosis;	NCBI: 1773	4388624	99.94	0.00	Yes
NCBI Assembly	GCA_001841565.1	NCBI: cellular organisms; Bacteria; Terrabacteria group; Actinobacteria; Actinomycetia; Corynebacteriales; Mycobacteriaceae; Mycobacterium; Mycobacterium tuberculosis complex; Mycobacterium tuberculosis;	NCBI: 1773	4399003	99.94	0.00	Yes
NCBI Assembly	GCA_001666755.1	NCBI: cellular organisms; Bacteria; Terrabacteria group; Actinobacteria; Actinomycetia; Corynebacteriales; Mycobacteriaceae; Mycobacterium; unclassified Mycobacterium;	NCBI: 1834123	5355781	99.91	2.50	Yes
NCBI Assembly	GCA_001840645.1	NCBI: cellular organisms; Bacteria; Terrabacteria group; Actinobacteria; Actinomycetia; Corynebacteriales; Mycobacteriaceae; Mycobacterium; Mycobacterium tuberculosis complex; Mycobacterium tuberculosis;	NCBI: 1773	4349337	99.44	0.00	Yes
NCBI Assembly	GCA_001840315.1	NCBI: cellular organisms; Bacteria; Terrabacteria group; Actinobacteria; Actinomycetia; Corynebacteriales; Mycobacteriaceae; Mycobacterium; Mycobacterium tuberculosis complex; Mycobacterium tuberculosis;	NCBI: 1773	4387194	99.94	0.00	Yes
NCBI Assembly	GCA_001672925.1	NCBI: cellular organisms; Bacteria; Terrabacteria group; Actinobacteria; Actinomycetia; Corynebacteriales; Mycobacteriaceae; Mycobacterium; Mycobacterium asiaticum;	NCBI: 1790	6116072	99.61	0.67	Yes
NCBI Assembly	GCA_001842225.1	NCBI: cellular organisms; Bacteria; Terrabacteria group; Actinobacteria; Actinomycetia; Corynebacteriales; Mycobacteriaceae; Mycobacterium; Mycobacterium tuberculosis complex; Mycobacterium tuberculosis;	NCBI: 1773	4400568	99.94	0.00	Yes
NCBI Assembly	GCA_001843185.1	NCBI: cellular organisms; Bacteria; Terrabacteria group; Actinobacteria; Actinomycetia; Corynebacteriales; Mycobacteriaceae; Mycobacterium; Mycobacterium tuberculosis complex; Mycobacterium tuberculosis;	NCBI: 1773	4411143	99.94	0.00	Yes
NCBI Assembly	GCA_001853195.1	NCBI: cellular organisms; Bacteria; Terrabacteria group; Actinobacteria; Actinomycetia; Corynebacteriales; Mycobacteriaceae; Mycobacterium; Mycobacterium tuberculosis complex; Mycobacterium tuberculosis;	NCBI: 1773	4381336	99.94	0.00	Yes
NCBI Assembly	GCA_001844105.1	NCBI: cellular organisms; Bacteria; Terrabacteria group; Actinobacteria; Actinomycetia; Corynebacteriales; Mycobacteriaceae; Mycobacterium; Mycobacterium tuberculosis complex; Mycobacterium tuberculosis;	NCBI: 1773	4404355	99.94	0.00	Yes
NCBI Assembly	GCA_001842875.1	NCBI: cellular organisms; Bacteria; Terrabacteria group; Actinobacteria; Actinomycetia; Corynebacteriales; Mycobacteriaceae; Mycobacterium; Mycobacterium tuberculosis complex; Mycobacterium tuberculosis;	NCBI: 1773	4411969	99.94	0.00	Yes
NCBI Assembly	GCA_001851625.1	NCBI: cellular organisms; Bacteria; Terrabacteria group; Actinobacteria; Actinomycetia; Corynebacteriales; Mycobacteriaceae; Mycobacterium; Mycobacterium tuberculosis complex; Mycobacterium tuberculosis;	NCBI: 1773	4392760	99.61	0.00	Yes
NCBI Assembly	GCA_001848185.1	NCBI: cellular organisms; Bacteria; Terrabacteria group; Actinobacteria; Actinomycetia; Corynebacteriales; Mycobacteriaceae; Mycobacterium; Mycobacterium tuberculosis complex; Mycobacterium tuberculosis;	NCBI: 1773	4355060	99.94	0.00	Yes
NCBI Assembly	GCA_001841575.1	NCBI: cellular organisms; Bacteria; Terrabacteria group; Actinobacteria; Actinomycetia; Corynebacteriales; Mycobacteriaceae; Mycobacterium; Mycobacterium tuberculosis complex; Mycobacterium tuberculosis;	NCBI: 1773	4399046	99.94	0.00	Yes
NCBI Assembly	GCA_001666785.1	NCBI: cellular organisms; Bacteria; Terrabacteria group; Actinobacteria; Actinomycetia; Corynebacteriales; Mycobacteriaceae; Mycobacterium; unclassified Mycobacterium;	NCBI: 1834145	5652161	99.78	1.61	Yes
NCBI Assembly	GCA_001840665.1	NCBI: cellular organisms; Bacteria; Terrabacteria group; Actinobacteria; Actinomycetia; Corynebacteriales; Mycobacteriaceae; Mycobacterium; Mycobacterium tuberculosis complex; Mycobacterium tuberculosis;	NCBI: 1773	4401430	99.61	0.00	Yes
NCBI Assembly	GCA_001840325.1	NCBI: cellular organisms; Bacteria; Terrabacteria group; Actinobacteria; Actinomycetia; Corynebacteriales; Mycobacteriaceae; Mycobacterium; Mycobacterium tuberculosis complex; Mycobacterium tuberculosis;	NCBI: 1773	4403733	99.94	0.00	Yes
NCBI Assembly	GCA_001853205.1	NCBI: cellular organisms; Bacteria; Terrabacteria group; Actinobacteria; Actinomycetia; Corynebacteriales; Mycobacteriaceae; Mycobacterium; Mycobacterium tuberculosis complex; Mycobacterium tuberculosis;	NCBI: 1773	4381855	99.94	0.00	Yes

NCBI Assembly	GCA_001844145.1	NCBI: cellular organisms; Bacteria; Terrabacteria group; Actinobacteria; Actinomycetia; Corynebacteriales; Mycobacteriaceae; Mycobacterium; Mycobacterium tuberculosis complex; Mycobacterium tuberculosis;	NCBI: 1773	4381648	99.94	0.00	Yes
NCBI Assembly	GCA_001843215.1	NCBI: cellular organisms; Bacteria; Terrabacteria group; Actinobacteria; Actinomycetia; Corynebacteriales; Mycobacteriaceae; Mycobacterium; Mycobacterium tuberculosis complex; Mycobacterium tuberculosis;	NCBI: 1773	4345836	99.94	0.00	Yes
NCBI Assembly	GCA_001672935.1	NCBI: cellular organisms; Bacteria; Terrabacteria group; Actinobacteria; Actinomycetia; Corynebacteriales; Mycobacteriaceae; Mycobacterium; Mycobacterium alsense;	NCBI: 324058	5473558	99.83	0.93	Yes
NCBI Assembly	GCA_001842245.1	NCBI: cellular organisms; Bacteria; Terrabacteria group; Actinobacteria; Actinomycetia; Corynebacteriales; Mycobacteriaceae; Mycobacterium; Mycobacterium tuberculosis complex; Mycobacterium tuberculosis;	NCBI: 1773	4393416	99.94	0.00	Yes
NCBI Assembly	GCA_001851635.1	NCBI: cellular organisms; Bacteria; Terrabacteria group; Actinobacteria; Actinomycetia; Corynebacteriales; Mycobacteriaceae; Mycobacterium; Mycobacterium tuberculosis complex; Mycobacterium tuberculosis;	NCBI: 1773	4404752	99.61	0.00	Yes
NCBI Assembly	GCA_001842895.1	NCBI: cellular organisms; Bacteria; Terrabacteria group; Actinobacteria; Actinomycetia; Corynebacteriales; Mycobacteriaceae; Mycobacterium; Mycobacterium tuberculosis complex; Mycobacterium tuberculosis;	NCBI: 1773	4392250	99.94	0.00	Yes
NCBI Assembly	GCA_001848215.1	NCBI: cellular organisms; Bacteria; Terrabacteria group; Actinobacteria; Actinomycetia; Corynebacteriales; Mycobacteriaceae; Mycobacterium; Mycobacterium tuberculosis complex; Mycobacterium tuberculosis;	NCBI: 1773	4384422	99.94	0.00	Yes
NCBI Assembly	GCA_001841605.1	NCBI: cellular organisms; Bacteria; Terrabacteria group; Actinobacteria; Actinomycetia; Corynebacteriales; Mycobacteriaceae; Mycobacterium; Mycobacterium tuberculosis complex; Mycobacterium tuberculosis;	NCBI: 1773	4399128	99.94	0.00	Yes
NCBI Assembly	GCA_001666795.1	NCBI: cellular organisms; Bacteria; Terrabacteria group; Actinobacteria; Actinomycetia; Corynebacteriales; Mycobacteriaceae; Mycolicibacterium; Mycolicibacterium fortuitum;	NCBI: 1766	6332502	99.81	0.87	Yes
NCBI Assembly	GCA_001840675.1	NCBI: cellular organisms; Bacteria; Terrabacteria group; Actinobacteria; Actinomycetia; Corynebacteriales; Mycobacteriaceae; Mycobacterium; Mycobacterium tuberculosis complex; Mycobacterium tuberculosis;	NCBI: 1773	4400632	99.94	0.00	Yes
NCBI Assembly	GCA_001672975.1	NCBI: cellular organisms; Bacteria; Terrabacteria group; Actinobacteria; Actinomycetia; Corynebacteriales; Mycobacteriaceae; Mycobacterium; unclassified Mycobacterium;	NCBI: 1834078	5922017	99.78	3.49	Yes
NCBI Assembly	GCA_001840365.1	NCBI: cellular organisms; Bacteria; Terrabacteria group; Actinobacteria; Actinomycetia; Corynebacteriales; Mycobacteriaceae; Mycobacterium; Mycobacterium tuberculosis complex; Mycobacterium tuberculosis;	NCBI: 1773	4387046	99.94	0.00	Yes
NCBI Assembly	GCA_001844165.1	NCBI: cellular organisms; Bacteria; Terrabacteria group; Actinobacteria; Actinomycetia; Corynebacteriales; Mycobacteriaceae; Mycobacterium; Mycobacterium tuberculosis complex; Mycobacterium tuberculosis;	NCBI: 1773	4376824	99.61	0.00	Yes
NCBI Assembly	GCA_001853245.1	NCBI: cellular organisms; Bacteria; Terrabacteria group; Actinobacteria; Actinomycetia; Corynebacteriales; Mycobacteriaceae; Mycobacterium; Mycobacterium tuberculosis complex; Mycobacterium tuberculosis;	NCBI: 1773	4385765	99.94	0.00	Yes
NCBI Assembly	GCA_001843245.1	NCBI: cellular organisms; Bacteria; Terrabacteria group; Actinobacteria; Actinomycetia; Corynebacteriales; Mycobacteriaceae; Mycobacterium; Mycobacterium tuberculosis complex; Mycobacterium tuberculosis;	NCBI: 1773	4389971	99.94	0.00	Yes
NCBI Assembly	GCA_001842915.1	NCBI: cellular organisms; Bacteria; Terrabacteria group; Actinobacteria; Actinomycetia; Corynebacteriales; Mycobacteriaceae; Mycobacterium; Mycobacterium tuberculosis complex; Mycobacterium tuberculosis;	NCBI: 1773	4418848	99.94	0.00	Yes
NCBI Assembly	GCA_001851655.1	NCBI: cellular organisms; Bacteria; Terrabacteria group; Actinobacteria; Actinomycetia; Corynebacteriales; Mycobacteriaceae; Mycobacterium; Mycobacterium tuberculosis complex; Mycobacterium tuberculosis;	NCBI: 1773	4381330	99.61	0.00	Yes
NCBI Assembly	GCA_001848255.1	NCBI: cellular organisms; Bacteria; Terrabacteria group; Actinobacteria; Actinomycetia; Corynebacteriales; Mycobacteriaceae; Mycobacterium; Mycobacterium tuberculosis complex; Mycobacterium tuberculosis;	NCBI: 1773	4396708	99.94	0.00	Yes

NCBI Assembly	GCA_001842255.1	NCBI: cellular organisms; Bacteria; Terrabacteria group; Actinobacteria; Actinomycetia; Corynebacteriales; Mycobacteriaceae; Mycobacterium; Mycobacterium tuberculosis complex; Mycobacterium tuberculosis;	NCBI: 1773	4403353	99.94	0.00	Yes
NCBI Assembly	GCA_001841615.1	NCBI: cellular organisms; Bacteria; Terrabacteria group; Actinobacteria; Actinomycetia; Corynebacteriales; Mycobacteriaceae; Mycobacterium; Mycobacterium tuberculosis complex; Mycobacterium tuberculosis;	NCBI: 1773	4383588	99.94	0.00	Yes
NCBI Assembly	GCA_001666815.1	NCBI: cellular organisms; Bacteria; Terrabacteria group; Actinobacteria; Actinomycetia; Corynebacteriales; Mycobacteriaceae; Mycobacterium; Mycobacterium alsense;	NCBI: 324058	5656398	99.50	1.05	Yes
NCBI Assembly	GCA_001672995.1	NCBI: cellular organisms; Bacteria; Terrabacteria group; Actinobacteria; Actinomycetia; Corynebacteriales; Mycobacteriaceae; Mycobacterium; unclassified Mycobacterium;	NCBI: 1834081	5714246	99.94	0.97	Yes
NCBI Assembly	GCA_001840705.1	NCBI: cellular organisms; Bacteria; Terrabacteria group; Actinobacteria; Actinomycetia; Corynebacteriales; Mycobacteriaceae; Mycobacterium; Mycobacterium tuberculosis complex; Mycobacterium tuberculosis;	NCBI: 1773	4433721	99.94	1.33	Yes
NCBI Assembly	GCA_001844175.1	NCBI: cellular organisms; Bacteria; Terrabacteria group; Actinobacteria; Actinomycetia; Corynebacteriales; Mycobacteriaceae; Mycobacterium; Mycobacterium tuberculosis complex; Mycobacterium tuberculosis;	NCBI: 1773	4421972	99.94	0.00	Yes
NCBI Assembly	GCA_001840385.1	NCBI: cellular organisms; Bacteria; Terrabacteria group; Actinobacteria; Actinomycetia; Corynebacteriales; Mycobacteriaceae; Mycobacterium; Mycobacterium tuberculosis complex; Mycobacterium tuberculosis;	NCBI: 1773	4411863	99.94	0.00	Yes
NCBI Assembly	GCA_001853265.1	NCBI: cellular organisms; Bacteria; Terrabacteria group; Actinobacteria; Actinomycetia; Corynebacteriales; Mycobacteriaceae; Mycobacterium; Mycobacterium tuberculosis complex; Mycobacterium tuberculosis;	NCBI: 1773	4391956	99.94	0.00	Yes
NCBI Assembly	GCA_001842265.1	NCBI: cellular organisms; Bacteria; Terrabacteria group; Actinobacteria; Actinomycetia; Corynebacteriales; Mycobacteriaceae; Mycobacterium; Mycobacterium tuberculosis complex; Mycobacterium tuberculosis;	NCBI: 1773	4393209	99.94	0.00	Yes
NCBI Assembly	GCA_001843255.1	NCBI: cellular organisms; Bacteria; Terrabacteria group; Actinobacteria; Actinomycetia; Corynebacteriales; Mycobacteriaceae; Mycobacterium; Mycobacterium tuberculosis complex; Mycobacterium tuberculosis;	NCBI: 1773	4353364	99.94	0.00	Yes
NCBI Assembly	GCA_001842945.1	NCBI: cellular organisms; Bacteria; Terrabacteria group; Actinobacteria; Actinomycetia; Corynebacteriales; Mycobacteriaceae; Mycobacterium; Mycobacterium tuberculosis complex; Mycobacterium tuberculosis;	NCBI: 1773	4393281	99.94	0.00	Yes
NCBI Assembly	GCA_001848245.1	NCBI: cellular organisms; Bacteria; Terrabacteria group; Actinobacteria; Actinomycetia; Corynebacteriales; Mycobacteriaceae; Mycobacterium; Mycobacterium tuberculosis complex; Mycobacterium tuberculosis;	NCBI: 1773	4382327	99.94	0.00	Yes
NCBI Assembly	GCA_001851665.1	NCBI: cellular organisms; Bacteria; Terrabacteria group; Actinobacteria; Actinomycetia; Corynebacteriales; Mycobacteriaceae; Mycobacterium; Mycobacterium tuberculosis complex; Mycobacterium tuberculosis;	NCBI: 1773	4381845	99.61	0.00	Yes
NCBI Assembly	GCA_001666835.1	NCBI: cellular organisms; Bacteria; Terrabacteria group; Actinobacteria; Actinomycetia; Corynebacteriales; Mycobacteriaceae; Mycobacterium; unclassified Mycobacterium;	NCBI: 1834144	5619324	99.61	1.23	Yes
NCBI Assembly	GCA_001841645.1	NCBI: cellular organisms; Bacteria; Terrabacteria group; Actinobacteria; Actinomycetia; Corynebacteriales; Mycobacteriaceae; Mycobacterium; Mycobacterium tuberculosis complex; Mycobacterium tuberculosis;	NCBI: 1773	4392188	99.94	0.00	Yes
NCBI Assembly	GCA_001673005.1	NCBI: cellular organisms; Bacteria; Terrabacteria group; Actinobacteria; Actinomycetia; Corynebacteriales; Mycobacteriaceae; Mycobacterium; Mycobacterium avium complex (MAC); Mycobacterium colombiense;	NCBI: 339268	5807855	99.28	1.09	Yes
NCBI Assembly	GCA_001840725.1	NCBI: cellular organisms; Bacteria; Terrabacteria group; Actinobacteria; Actinomycetia; Corynebacteriales; Mycobacteriaceae; Mycobacterium; Mycobacterium tuberculosis complex; Mycobacterium tuberculosis;	NCBI: 1773	4377523	99.61	0.00	Yes
NCBI Assembly	GCA_001844195.1	NCBI: cellular organisms; Bacteria; Terrabacteria group; Actinobacteria; Actinomycetia; Corynebacteriales; Mycobacteriaceae; Mycobacterium; Mycobacterium tuberculosis complex; Mycobacterium tuberculosis;	NCBI: 1773	4405809	99.94	0.00	Yes

NCBI Assembly	GCA_001840395.1	NCBI: cellular organisms; Bacteria; Terrabacteria group; Actinobacteria; Actinomycetia; Corynebacteriales; Mycobacteriaceae; Mycobacterium; Mycobacterium tuberculosis complex; Mycobacterium tuberculosis;	NCBI: 1773	4411615	99.94	0.00	Yes
NCBI Assembly	GCA_001843275.1	NCBI: cellular organisms; Bacteria; Terrabacteria group; Actinobacteria; Actinomycetia; Corynebacteriales; Mycobacteriaceae; Mycobacterium; Mycobacterium tuberculosis complex; Mycobacterium tuberculosis;	NCBI: 1773	4407415	99.94	0.00	Yes
NCBI Assembly	GCA_001848265.1	NCBI: cellular organisms; Bacteria; Terrabacteria group; Actinobacteria; Actinomycetia; Corynebacteriales; Mycobacteriaceae; Mycobacterium; Mycobacterium tuberculosis complex; Mycobacterium tuberculosis;	NCBI: 1773	4390312	99.94	0.00	Yes
NCBI Assembly	GCA_001853285.1	NCBI: cellular organisms; Bacteria; Terrabacteria group; Actinobacteria; Actinomycetia; Corynebacteriales; Mycobacteriaceae; Mycobacterium; Mycobacterium tuberculosis complex; Mycobacterium tuberculosis;	NCBI: 1773	4378957	99.61	0.00	Yes
NCBI Assembly	GCA_001842295.1	NCBI: cellular organisms; Bacteria; Terrabacteria group; Actinobacteria; Actinomycetia; Corynebacteriales; Mycobacteriaceae; Mycobacterium; Mycobacterium tuberculosis complex; Mycobacterium tuberculosis;	NCBI: 1773	4391183	99.94	0.00	Yes
NCBI Assembly	GCA_001666865.1	NCBI: cellular organisms; Bacteria; Terrabacteria group; Actinobacteria; Actinomycetia; Corynebacteriales; Mycobacteriaceae; Mycobacterium; unclassified Mycobacterium;	NCBI: 1834125	5523633	99.09	2.12	Yes
NCBI Assembly	GCA_001842955.1	NCBI: cellular organisms; Bacteria; Terrabacteria group; Actinobacteria; Actinomycetia; Corynebacteriales; Mycobacteriaceae; Mycobacterium; Mycobacterium tuberculosis complex; Mycobacterium tuberculosis;	NCBI: 1773	4401879	99.94	0.00	Yes
NCBI Assembly	GCA_001851705.1	NCBI: cellular organisms; Bacteria; Terrabacteria group; Actinobacteria; Actinomycetia; Corynebacteriales; Mycobacteriaceae; Mycobacterium; Mycobacterium tuberculosis complex; Mycobacterium tuberculosis;	NCBI: 1773	4407759	99.61	0.00	Yes
NCBI Assembly	GCA_001841665.1	NCBI: cellular organisms; Bacteria; Terrabacteria group; Actinobacteria; Actinomycetia; Corynebacteriales; Mycobacteriaceae; Mycobacterium; Mycobacterium tuberculosis complex; Mycobacterium tuberculosis;	NCBI: 1773	4421830	99.94	0.00	Yes
NCBI Assembly	GCA_001673015.1	NCBI: cellular organisms; Bacteria; Terrabacteria group; Actinobacteria; Actinomycetia; Corynebacteriales; Mycobacteriaceae; Mycobacterium; Mycobacterium avium complex (MAC); Mycobacterium colombiense;	NCBI: 339268	5635613	99.28	1.61	Yes
NCBI Assembly	GCA_001840745.1	NCBI: cellular organisms; Bacteria; Terrabacteria group; Actinobacteria; Actinomycetia; Corynebacteriales; Mycobacteriaceae; Mycobacterium; Mycobacterium tuberculosis complex; Mycobacterium tuberculosis;	NCBI: 1773	4390856	99.94	0.33	Yes
NCBI Assembly	GCA_001665235.1	NCBI: cellular organisms; Bacteria; Terrabacteria group; Actinobacteria; Actinomycetia; Corynebacteriales; Mycobacteriaceae; Mycobacterium; unclassified Mycobacterium;	NCBI: 1834067	5670445	99.61	2.33	Yes
NCBI Assembly	GCA_001843295.1	NCBI: cellular organisms; Bacteria; Terrabacteria group; Actinobacteria; Actinomycetia; Corynebacteriales; Mycobacteriaceae; Mycobacterium; Mycobacterium tuberculosis complex; Mycobacterium tuberculosis;	NCBI: 1773	4389740	99.94	0.00	Yes
NCBI Assembly	GCA_001844215.1	NCBI: cellular organisms; Bacteria; Terrabacteria group; Actinobacteria; Actinomycetia; Corynebacteriales; Mycobacteriaceae; Mycobacterium; Mycobacterium tuberculosis complex; Mycobacterium tuberculosis;	NCBI: 1773	4396398	99.94	0.00	Yes
NCBI Assembly	GCA_001853295.1	NCBI: cellular organisms; Bacteria; Terrabacteria group; Actinobacteria; Actinomycetia; Corynebacteriales; Mycobacteriaceae; Mycobacterium; Mycobacterium tuberculosis complex; Mycobacterium tuberculosis;	NCBI: 1773	4396999	99.94	0.00	Yes
NCBI Assembly	GCA_001840405.1	NCBI: cellular organisms; Bacteria; Terrabacteria group; Actinobacteria; Actinomycetia; Corynebacteriales; Mycobacteriaceae; Mycobacterium; Mycobacterium tuberculosis complex; Mycobacterium tuberculosis;	NCBI: 1773	4393112	99.94	0.00	Yes
NCBI Assembly	GCA_001842985.1	NCBI: cellular organisms; Bacteria; Terrabacteria group; Actinobacteria; Actinomycetia; Corynebacteriales; Mycobacteriaceae; Mycobacterium; Mycobacterium tuberculosis complex; Mycobacterium tuberculosis;	NCBI: 1773	4343692	99.94	0.00	Yes
NCBI Assembly	GCA_001848285.1	NCBI: cellular organisms; Bacteria; Terrabacteria group; Actinobacteria; Actinomycetia; Corynebacteriales; Mycobacteriaceae; Mycobacterium; Mycobacterium tuberculosis complex; Mycobacterium tuberculosis;	NCBI: 1773	4354451	99.94	0.00	Yes

NCBI Assembly	GCA_001842325.1	NCBI: cellular organisms; Bacteria; Terrabacteria group; Actinobacteria; Actinomycetia; Corynebacteriales; Mycobacteriaceae; Mycobacterium; Mycobacterium tuberculosis complex; Mycobacterium tuberculosis;	NCBI: 1773	4386208	99.94	0.00	Yes
NCBI Assembly	GCA_001851715.1	NCBI: cellular organisms; Bacteria; Terrabacteria group; Actinobacteria; Actinomycetia; Corynebacteriales; Mycobacteriaceae; Mycobacterium; Mycobacterium tuberculosis complex; Mycobacterium tuberculosis;	NCBI: 1773	4393797	99.61	0.00	Yes
NCBI Assembly	GCA_001841685.1	NCBI: cellular organisms; Bacteria; Terrabacteria group; Actinobacteria; Actinomycetia; Corynebacteriales; Mycobacteriaceae; Mycobacterium; Mycobacterium tuberculosis complex; Mycobacterium tuberculosis;	NCBI: 1773	4351413	99.94	0.00	Yes
NCBI Assembly	GCA_001673055.1	NCBI: cellular organisms; Bacteria; Terrabacteria group; Actinobacteria; Actinomycetia; Corynebacteriales; Mycobacteriaceae; Mycobacterium; unclassified Mycobacterium;	NCBI: 1834075	5769712	99.28	1.59	Yes
NCBI Assembly	GCA_001665295.1	NCBI: cellular organisms; Bacteria; Terrabacteria group; Actinobacteria; Actinomycetia; Corynebacteriales; Mycobacteriaceae; Mycobacterium; unclassified Mycobacterium;	NCBI: 1834082	6144564	99.83	2.08	Yes
NCBI Assembly	GCA_001840755.1	NCBI: cellular organisms; Bacteria; Terrabacteria group; Actinobacteria; Actinomycetia; Corynebacteriales; Mycobacteriaceae; Mycobacterium; Mycobacterium tuberculosis complex; Mycobacterium tuberculosis;	NCBI: 1773	4398334	99.94	0.00	Yes
NCBI Assembly	GCA_001842345.1	NCBI: cellular organisms; Bacteria; Terrabacteria group; Actinobacteria; Actinomycetia; Corynebacteriales; Mycobacteriaceae; Mycobacterium; Mycobacterium tuberculosis complex; Mycobacterium tuberculosis;	NCBI: 1773	4339984	99.94	0.00	Yes
NCBI Assembly	GCA_001853525.1	NCBI: cellular organisms; Bacteria; Terrabacteria group; Actinobacteria; Actinomycetia; Corynebacteriales; Mycobacteriaceae; Mycobacterium; Mycobacterium syngnathidarum;	NCBI: 1908205	6434367	99.81	0.68	Yes
NCBI Assembly	GCA_001843615.1	NCBI: cellular organisms; Bacteria; Terrabacteria group; Actinobacteria; Actinomycetia; Corynebacteriales; Mycobacteriaceae; Mycobacterium; Mycobacterium tuberculosis complex; Mycobacterium tuberculosis;	NCBI: 1773	4406546	99.61	0.00	Yes
NCBI Assembly	GCA_001839815.1	NCBI: cellular organisms; Bacteria; Terrabacteria group; Actinobacteria; Actinomycetia; Corynebacteriales; Mycobacteriaceae; Mycobacterium; Mycobacterium tuberculosis complex; Mycobacterium tuberculosis;	NCBI: 1773	4394679	99.94	0.00	Yes
NCBI Assembly	GCA_001848325.1	NCBI: cellular organisms; Bacteria; Terrabacteria group; Actinobacteria; Actinomycetia; Corynebacteriales; Mycobacteriaceae; Mycobacterium; Mycobacterium tuberculosis complex; Mycobacterium tuberculosis;	NCBI: 1773	4366968	99.94	0.00	Yes
NCBI Assembly	GCA_001843325.1	NCBI: cellular organisms; Bacteria; Terrabacteria group; Actinobacteria; Actinomycetia; Corynebacteriales; Mycobacteriaceae; Mycobacterium; Mycobacterium tuberculosis complex; Mycobacterium tuberculosis;	NCBI: 1773	4354236	99.94	0.00	Yes
NCBI Assembly	GCA_001841705.1	NCBI: cellular organisms; Bacteria; Terrabacteria group; Actinobacteria; Actinomycetia; Corynebacteriales; Mycobacteriaceae; Mycobacterium; Mycobacterium tuberculosis complex; Mycobacterium tuberculosis;	NCBI: 1773	4363994	99.94	0.00	Yes
NCBI Assembly	GCA_001851115.1	NCBI: cellular organisms; Bacteria; Terrabacteria group; Actinobacteria; Actinomycetia; Corynebacteriales; Mycobacteriaceae; Mycobacterium; Mycobacterium tuberculosis complex; Mycobacterium tuberculosis;	NCBI: 1773	4392240	99.61	0.00	Yes
NCBI Assembly	GCA_001841065.1	NCBI: cellular organisms; Bacteria; Terrabacteria group; Actinobacteria; Actinomycetia; Corynebacteriales; Mycobacteriaceae; Mycobacterium; Mycobacterium tuberculosis complex; Mycobacterium tuberculosis;	NCBI: 1773	4392978	99.61	0.00	Yes
NCBI Assembly	GCA_001673075.1	NCBI: cellular organisms; Bacteria; Terrabacteria group; Actinobacteria; Actinomycetia; Corynebacteriales; Mycobacteriaceae; Mycobacterium; Mycobacterium avium complex (MAC); Mycobacterium colombiense;	NCBI: 339268	5722443	99.78	2.13	Yes
NCBI Assembly	GCA_001665315.1	NCBI: cellular organisms; Bacteria; Terrabacteria group; Actinobacteria; Actinomycetia; Corynebacteriales; Mycobacteriaceae; Mycolicibacterium; Mycolicibacterium fortuitum;	NCBI: 1766	6134932	99.43	2.23	Yes
NCBI Assembly	GCA_001840775.1	NCBI: cellular organisms; Bacteria; Terrabacteria group; Actinobacteria; Actinomycetia; Corynebacteriales; Mycobacteriaceae; Mycobacterium; Mycobacterium tuberculosis complex; Mycobacterium tuberculosis;	NCBI: 1773	4348559	99.61	0.00	Yes

NCBI Assembly	GCA_001853535.1	NCBI: cellular organisms; Bacteria; Terrabacteria group; Actinobacteria; Actinomycetia; Corynebacteriales; Mycobacteriaceae; Mycobacteroides; Mycobacteroides chelonae;	NCBI: 1774	5092024	99.50	0.03	Yes
NCBI Assembly	GCA_001839845.1	NCBI: cellular organisms; Bacteria; Terrabacteria group; Actinobacteria; Actinomycetia; Corynebacteriales; Mycobacteriaceae; Mycobacterium; Mycobacterium tuberculosis complex; Mycobacterium tuberculosis;	NCBI: 1773	4384737	99.94	0.00	Yes
NCBI Assembly	GCA_001842355.1	NCBI: cellular organisms; Bacteria; Terrabacteria group; Actinobacteria; Actinomycetia; Corynebacteriales; Mycobacteriaceae; Mycobacterium; Mycobacterium tuberculosis complex; Mycobacterium tuberculosis;	NCBI: 1773	4399494	99.94	0.00	Yes
NCBI Assembly	GCA_001843335.1	NCBI: cellular organisms; Bacteria; Terrabacteria group; Actinobacteria; Actinomycetia; Corynebacteriales; Mycobacteriaceae; Mycobacterium; Mycobacterium tuberculosis complex; Mycobacterium tuberculosis;	NCBI: 1773	4381059	99.94	0.00	Yes
NCBI Assembly	GCA_001841715.1	NCBI: cellular organisms; Bacteria; Terrabacteria group; Actinobacteria; Actinomycetia; Corynebacteriales; Mycobacteriaceae; Mycobacterium; Mycobacterium tuberculosis complex; Mycobacterium tuberculosis;	NCBI: 1773	4397018	99.94	0.00	Yes
NCBI Assembly	GCA_001848345.1	NCBI: cellular organisms; Bacteria; Terrabacteria group; Actinobacteria; Actinomycetia; Corynebacteriales; Mycobacteriaceae; Mycobacterium; Mycobacterium tuberculosis complex; Mycobacterium tuberculosis;	NCBI: 1773	4380746	99.94	0.00	Yes
NCBI Assembly	GCA_001843645.1	NCBI: cellular organisms; Bacteria; Terrabacteria group; Actinobacteria; Actinomycetia; Corynebacteriales; Mycobacteriaceae; Mycobacterium; Mycobacterium tuberculosis complex; Mycobacterium tuberculosis;	NCBI: 1773	4429188	99.94	0.00	Yes
NCBI Assembly	GCA_001851125.1	NCBI: cellular organisms; Bacteria; Terrabacteria group; Actinobacteria; Actinomycetia; Corynebacteriales; Mycobacteriaceae; Mycobacterium; Mycobacterium tuberculosis complex; Mycobacterium tuberculosis;	NCBI: 1773	4395544	99.61	0.00	Yes
NCBI Assembly	GCA_001841075.1	NCBI: cellular organisms; Bacteria; Terrabacteria group; Actinobacteria; Actinomycetia; Corynebacteriales; Mycobacteriaceae; Mycobacterium; Mycobacterium tuberculosis complex; Mycobacterium tuberculosis;	NCBI: 1773	4398539	99.61	0.00	Yes
NCBI Assembly	GCA_001673085.1	NCBI: cellular organisms; Bacteria; Terrabacteria group; Actinobacteria; Actinomycetia; Corynebacteriales; Mycobacteriaceae; Mycobacterium; Mycobacterium avium complex (MAC); Mycobacterium colombiense;	NCBI: 339268	5667092	99.90	2.78	Yes
NCBI Assembly	GCA_001665335.1	NCBI: cellular organisms; Bacteria; Terrabacteria group; Actinobacteria; Actinomycetia; Corynebacteriales; Mycobacteriaceae; Mycolicibacterium; Mycolicibacterium conceptionense;	NCBI: 451644	6452644	100.00	0.00	Yes
NCBI Assembly	GCA_001840805.1	NCBI: cellular organisms; Bacteria; Terrabacteria group; Actinobacteria; Actinomycetia; Corynebacteriales; Mycobacteriaceae; Mycobacterium; Mycobacterium tuberculosis complex; Mycobacterium tuberculosis;	NCBI: 1773	4386186	99.61	0.00	Yes
NCBI Assembly	GCA_001853545.1	NCBI: cellular organisms; Bacteria; Terrabacteria group; Actinobacteria; Actinomycetia; Corynebacteriales; Mycobacteriaceae; Mycobacteroides; Mycobacteroides chelonae;	NCBI: 1774	5115523	99.50	0.03	Yes
NCBI Assembly	GCA_001839865.1	NCBI: cellular organisms; Bacteria; Terrabacteria group; Actinobacteria; Actinomycetia; Corynebacteriales; Mycobacteriaceae; Mycobacterium; Mycobacterium tuberculosis complex; Mycobacterium tuberculosis;	NCBI: 1773	4393178	99.94	0.00	Yes
NCBI Assembly	GCA_001842375.1	NCBI: cellular organisms; Bacteria; Terrabacteria group; Actinobacteria; Actinomycetia; Corynebacteriales; Mycobacteriaceae; Mycobacterium; Mycobacterium tuberculosis complex; Mycobacterium tuberculosis;	NCBI: 1773	4386601	99.94	0.00	Yes
NCBI Assembly	GCA_001843355.1	NCBI: cellular organisms; Bacteria; Terrabacteria group; Actinobacteria; Actinomycetia; Corynebacteriales; Mycobacteriaceae; Mycobacterium; Mycobacterium tuberculosis complex; Mycobacterium tuberculosis;	NCBI: 1773	4361443	99.94	0.00	Yes
NCBI Assembly	GCA_001848355.1	NCBI: cellular organisms; Bacteria; Terrabacteria group; Actinobacteria; Actinomycetia; Corynebacteriales; Mycobacteriaceae; Mycobacterium; Mycobacterium tuberculosis complex; Mycobacterium tuberculosis;	NCBI: 1773	4389882	99.94	0.00	Yes
NCBI Assembly	GCA_001841725.1	NCBI: cellular organisms; Bacteria; Terrabacteria group; Actinobacteria; Actinomycetia; Corynebacteriales; Mycobacteriaceae; Mycobacterium; Mycobacterium tuberculosis complex; Mycobacterium tuberculosis;	NCBI: 1773	4399710	99.94	0.00	Yes

NCBI Assembly	GCA_001851135.1	NCBI: cellular organisms; Bacteria; Terrabacteria group; Actinobacteria; Actinomycetia; Corynebacteriales; Mycobacteriaceae; Mycobacterium; Mycobacterium tuberculosis complex; Mycobacterium tuberculosis;	NCBI: 1773	4395696	99.94	0.00	Yes
NCBI Assembly	GCA_001843655.1	NCBI: cellular organisms; Bacteria; Terrabacteria group; Actinobacteria; Actinomycetia; Corynebacteriales; Mycobacteriaceae; Mycobacterium; Mycobacterium tuberculosis complex; Mycobacterium tuberculosis;	NCBI: 1773	4403678	99.94	0.00	Yes
NCBI Assembly	GCA_001841105.1	NCBI: cellular organisms; Bacteria; Terrabacteria group; Actinobacteria; Actinomycetia; Corynebacteriales; Mycobacteriaceae; Mycobacterium; Mycobacterium tuberculosis complex; Mycobacterium tuberculosis;	NCBI: 1773	4372594	99.61	0.00	Yes
NCBI Assembly	GCA_001673105.1	NCBI: cellular organisms; Bacteria; Terrabacteria group; Actinobacteria; Actinomycetia; Corynebacteriales; Mycobacteriaceae; Mycolicibacter; Mycolicibacter heraklionensis;	NCBI: 512402	4799517	100.00	0.61	Yes
NCBI Assembly	GCA_001665365.1	NCBI: cellular organisms; Bacteria; Terrabacteria group; Actinobacteria; Actinomycetia; Corynebacteriales; Mycobacteriaceae; Mycolicibacterium; Mycolicibacterium elephantis;	NCBI: 81858	5418725	99.55	3.14	Yes
NCBI Assembly	GCA_001840825.1	NCBI: cellular organisms; Bacteria; Terrabacteria group; Actinobacteria; Actinomycetia; Corynebacteriales; Mycobacteriaceae; Mycobacterium; Mycobacterium tuberculosis complex; Mycobacterium tuberculosis;	NCBI: 1773	4353176	99.94	0.00	Yes
NCBI Assembly	GCA_001839885.1	NCBI: cellular organisms; Bacteria; Terrabacteria group; Actinobacteria; Actinomycetia; Corynebacteriales; Mycobacteriaceae; Mycobacterium; Mycobacterium tuberculosis complex; Mycobacterium tuberculosis;	NCBI: 1773	4402354	99.94	0.00	Yes
NCBI Assembly	GCA_001853585.1	NCBI: cellular organisms; Bacteria; Terrabacteria group; Actinobacteria; Actinomycetia; Corynebacteriales; Mycobacteriaceae; Mycobacteroides; Mycobacteroides chelonae;	NCBI: 1774	5267678	99.44	0.03	Yes
NCBI Assembly	GCA_001843375.1	NCBI: cellular organisms; Bacteria; Terrabacteria group; Actinobacteria; Actinomycetia; Corynebacteriales; Mycobacteriaceae; Mycobacterium; Mycobacterium tuberculosis complex; Mycobacterium tuberculosis;	NCBI: 1773	4412715	99.94	0.00	Yes
NCBI Assembly	GCA_001842405.1	NCBI: cellular organisms; Bacteria; Terrabacteria group; Actinobacteria; Actinomycetia; Corynebacteriales; Mycobacteriaceae; Mycobacterium; Mycobacterium tuberculosis complex; Mycobacterium tuberculosis;	NCBI: 1773	4386493	99.94	0.00	Yes
NCBI Assembly	GCA_001841765.1	NCBI: cellular organisms; Bacteria; Terrabacteria group; Actinobacteria; Actinomycetia; Corynebacteriales; Mycobacteriaceae; Mycobacterium; Mycobacterium tuberculosis complex; Mycobacterium tuberculosis;	NCBI: 1773	4396148	99.94	0.00	Yes
NCBI Assembly	GCA_001848365.1	NCBI: cellular organisms; Bacteria; Terrabacteria group; Actinobacteria; Actinomycetia; Corynebacteriales; Mycobacteriaceae; Mycobacterium; Mycobacterium tuberculosis complex; Mycobacterium tuberculosis;	NCBI: 1773	4341498	99.94	0.00	Yes
NCBI Assembly	GCA_001841115.1	NCBI: cellular organisms; Bacteria; Terrabacteria group; Actinobacteria; Actinomycetia; Corynebacteriales; Mycobacteriaceae; Mycobacterium; Mycobacterium tuberculosis complex; Mycobacterium tuberculosis;	NCBI: 1773	4363656	99.61	0.00	Yes
NCBI Assembly	GCA_001851185.1	NCBI: cellular organisms; Bacteria; Terrabacteria group; Actinobacteria; Actinomycetia; Corynebacteriales; Mycobacteriaceae; Mycobacterium; Mycobacterium tuberculosis complex; Mycobacterium tuberculosis;	NCBI: 1773	4392239	99.94	0.00	Yes
NCBI Assembly	GCA_001843685.1	NCBI: cellular organisms; Bacteria; Terrabacteria group; Actinobacteria; Actinomycetia; Corynebacteriales; Mycobacteriaceae; Mycobacterium; Mycobacterium tuberculosis complex; Mycobacterium tuberculosis;	NCBI: 1773	4383009	99.94	0.00	Yes
NCBI Assembly	GCA_001673125.1	NCBI: cellular organisms; Bacteria; Terrabacteria group; Actinobacteria; Actinomycetia; Corynebacteriales; Mycobacteriaceae; Mycobacterium; Mycobacterium tuberculosis complex; Mycobacterium tuberculosis;	NCBI: 1773	4396097	99.50	1.28	Yes
NCBI Assembly	GCA_001665375.1	NCBI: cellular organisms; Bacteria; Terrabacteria group; Actinobacteria; Actinomycetia; Corynebacteriales; Mycobacteriaceae; Mycolicibacterium; Mycolicibacterium mucogenicum;	NCBI: 56689	6049775	99.43	1.70	Yes
NCBI Assembly	GCA_001840835.1	NCBI: cellular organisms; Bacteria; Terrabacteria group; Actinobacteria; Actinomycetia; Corynebacteriales; Mycobacteriaceae; Mycobacterium; Mycobacterium tuberculosis complex; Mycobacterium tuberculosis;	NCBI: 1773	4349452	99.94	0.00	Yes

NCBI Assembly	GCA_001853605.1	NCBI: cellular organisms; Bacteria; Terrabacteria group; Actinobacteria; Actinomycetia; Corynebacteriales; Mycobacteriaceae; Mycobacteroides; Mycobacteroides chelonae;	NCBI: 1774	5171588	99.50	0.03	Yes
NCBI Assembly	GCA_001842415.1	NCBI: cellular organisms; Bacteria; Terrabacteria group; Actinobacteria; Actinomycetia; Corynebacteriales; Mycobacteriaceae; Mycobacterium; Mycobacterium tuberculosis complex; Mycobacterium tuberculosis;	NCBI: 1773	4403104	99.94	0.00	Yes
NCBI Assembly	GCA_001843405.1	NCBI: cellular organisms; Bacteria; Terrabacteria group; Actinobacteria; Actinomycetia; Corynebacteriales; Mycobacteriaceae; Mycobacterium; Mycobacterium tuberculosis complex; Mycobacterium tuberculosis;	NCBI: 1773	4380463	99.61	0.00	Yes
NCBI Assembly	GCA_001841785.1	NCBI: cellular organisms; Bacteria; Terrabacteria group; Actinobacteria; Actinomycetia; Corynebacteriales; Mycobacteriaceae; Mycobacterium; Mycobacterium tuberculosis complex; Mycobacterium tuberculosis;	NCBI: 1773	4398429	99.94	0.00	Yes
NCBI Assembly	GCA_001839895.1	NCBI: cellular organisms; Bacteria; Terrabacteria group; Actinobacteria; Actinomycetia; Corynebacteriales; Mycobacteriaceae; Mycobacterium; Mycobacterium tuberculosis complex; Mycobacterium tuberculosis;	NCBI: 1773	4400151	99.94	0.00	Yes
NCBI Assembly	GCA_001848405.1	NCBI: cellular organisms; Bacteria; Terrabacteria group; Actinobacteria; Actinomycetia; Corynebacteriales; Mycobacteriaceae; Mycobacterium; Mycobacterium tuberculosis complex; Mycobacterium tuberculosis;	NCBI: 1773	4361791	99.94	0.00	Yes
NCBI Assembly	GCA_001841145.1	NCBI: cellular organisms; Bacteria; Terrabacteria group; Actinobacteria; Actinomycetia; Corynebacteriales; Mycobacteriaceae; Mycobacterium; Mycobacterium tuberculosis complex; Mycobacterium tuberculosis;	NCBI: 1773	4405512	99.94	0.00	Yes
NCBI Assembly	GCA_001851195.1	NCBI: cellular organisms; Bacteria; Terrabacteria group; Actinobacteria; Actinomycetia; Corynebacteriales; Mycobacteriaceae; Mycobacterium; Mycobacterium tuberculosis complex; Mycobacterium tuberculosis;	NCBI: 1773	4394312	99.94	0.00	Yes
NCBI Assembly	GCA_001843705.1	NCBI: cellular organisms; Bacteria; Terrabacteria group; Actinobacteria; Actinomycetia; Corynebacteriales; Mycobacteriaceae; Mycobacterium; Mycobacterium tuberculosis complex; Mycobacterium tuberculosis;	NCBI: 1773	4368414	99.94	0.00	Yes
NCBI Assembly	GCA_001673155.1	NCBI: cellular organisms; Bacteria; Terrabacteria group; Actinobacteria; Actinomycetia; Corynebacteriales; Mycobacteriaceae; Mycobacterium; unclassified Mycobacterium;	NCBI: 1856859	5351894	98.90	3.59	Yes
NCBI Assembly	GCA_001665395.1	NCBI: cellular organisms; Bacteria; Terrabacteria group; Actinobacteria; Actinomycetia; Corynebacteriales; Mycobacteriaceae; Mycobacterium; unclassified Mycobacterium;	NCBI: 1856861	5606096	100.00	0.78	Yes
NCBI Assembly	GCA_001840845.1	NCBI: cellular organisms; Bacteria; Terrabacteria group; Actinobacteria; Actinomycetia; Corynebacteriales; Mycobacteriaceae; Mycobacterium; Mycobacterium tuberculosis complex; Mycobacterium tuberculosis;	NCBI: 1773	4385675	99.94	0.00	Yes
NCBI Assembly	GCA_001841805.1	NCBI: cellular organisms; Bacteria; Terrabacteria group; Actinobacteria; Actinomycetia; Corynebacteriales; Mycobacteriaceae; Mycobacterium; Mycobacterium tuberculosis complex; Mycobacterium tuberculosis;	NCBI: 1773	4403290	99.94	0.00	Yes
NCBI Assembly	GCA_001853615.1	NCBI: cellular organisms; Bacteria; Terrabacteria group; Actinobacteria; Actinomycetia; Corynebacteriales; Mycobacteriaceae; Mycobacteroides; Mycobacteroides chelonae;	NCBI: 1774	5101196	99.50	0.03	Yes
NCBI Assembly	GCA_001839905.1	NCBI: cellular organisms; Bacteria; Terrabacteria group; Actinobacteria; Actinomycetia; Corynebacteriales; Mycobacteriaceae; Mycobacterium; Mycobacterium tuberculosis complex; Mycobacterium tuberculosis;	NCBI: 1773	4407139	99.94	0.00	Yes
NCBI Assembly	GCA_001843425.1	NCBI: cellular organisms; Bacteria; Terrabacteria group; Actinobacteria; Actinomycetia; Corynebacteriales; Mycobacteriaceae; Mycobacterium; Mycobacterium tuberculosis complex; Mycobacterium tuberculosis;	NCBI: 1773	4387316	99.61	0.00	Yes
NCBI Assembly	GCA_001848425.1	NCBI: cellular organisms; Bacteria; Terrabacteria group; Actinobacteria; Actinomycetia; Corynebacteriales; Mycobacteriaceae; Mycobacterium; Mycobacterium tuberculosis complex; Mycobacterium tuberculosis;	NCBI: 1773	4398940	99.94	0.00	Yes
NCBI Assembly	GCA_001842445.1	NCBI: cellular organisms; Bacteria; Terrabacteria group; Actinobacteria; Actinomycetia; Corynebacteriales; Mycobacteriaceae; Mycobacterium; Mycobacterium tuberculosis complex; Mycobacterium tuberculosis;	NCBI: 1773	4403842	99.94	0.00	Yes

NCBI Assembly	GCA_001851205.1	NCBI: cellular organisms; Bacteria; Terrabacteria group; Actinobacteria; Actinomycetia; Corynebacteriales; Mycobacteriaceae; Mycobacterium; Mycobacterium tuberculosis complex; Mycobacterium tuberculosis;	NCBI: 1773	4391489	99.94	0.00	Yes
NCBI Assembly	GCA_001841165.1	NCBI: cellular organisms; Bacteria; Terrabacteria group; Actinobacteria; Actinomycetia; Corynebacteriales; Mycobacteriaceae; Mycobacterium; Mycobacterium tuberculosis complex; Mycobacterium tuberculosis;	NCBI: 1773	4386664	99.61	0.07	Yes
NCBI Assembly	GCA_001843725.1	NCBI: cellular organisms; Bacteria; Terrabacteria group; Actinobacteria; Actinomycetia; Corynebacteriales; Mycobacteriaceae; Mycobacterium; Mycobacterium tuberculosis complex; Mycobacterium tuberculosis;	NCBI: 1773	4393192	99.94	0.00	Yes
NCBI Assembly	GCA_001673165.1	NCBI: cellular organisms; Bacteria; Terrabacteria group; Actinobacteria; Actinomycetia; Corynebacteriales; Mycobacteriaceae; Mycobacterium; Mycobacterium asiaticum;	NCBI: 1790	6138373	99.94	3.46	Yes
NCBI Assembly	GCA_001665415.1	NCBI: cellular organisms; Bacteria; Terrabacteria group; Actinobacteria; Actinomycetia; Corynebacteriales; Mycobacteriaceae; Mycolicibacterium; Mycolicibacterium fortuitum;	NCBI: 1766	6505563	99.69	0.50	Yes
NCBI Assembly	GCA_001853625.1	NCBI: cellular organisms; Bacteria; Terrabacteria group; Actinobacteria; Actinomycetia; Corynebacteriales; Mycobacteriaceae; Mycobacteroides; Mycobacteroides saopaulense;	NCBI: 1578165	5008608	99.50	0.86	Yes
NCBI Assembly	GCA_001841815.1	NCBI: cellular organisms; Bacteria; Terrabacteria group; Actinobacteria; Actinomycetia; Corynebacteriales; Mycobacteriaceae; Mycobacterium; Mycobacterium tuberculosis complex; Mycobacterium tuberculosis;	NCBI: 1773	4383019	99.94	0.00	Yes
NCBI Assembly	GCA_001840885.1	NCBI: cellular organisms; Bacteria; Terrabacteria group; Actinobacteria; Actinomycetia; Corynebacteriales; Mycobacteriaceae; Mycobacterium; Mycobacterium tuberculosis complex; Mycobacterium tuberculosis;	NCBI: 1773	4376323	99.61	0.00	Yes
NCBI Assembly	GCA_001839945.1	NCBI: cellular organisms; Bacteria; Terrabacteria group; Actinobacteria; Actinomycetia; Corynebacteriales; Mycobacteriaceae; Mycobacterium; Mycobacterium tuberculosis complex; Mycobacterium tuberculosis;	NCBI: 1773	4406701	99.61	0.00	Yes
NCBI Assembly	GCA_001842455.1	NCBI: cellular organisms; Bacteria; Terrabacteria group; Actinobacteria; Actinomycetia; Corynebacteriales; Mycobacteriaceae; Mycobacterium; Mycobacterium tuberculosis complex; Mycobacterium tuberculosis;	NCBI: 1773	4369317	99.94	0.00	Yes
NCBI Assembly	GCA_001843435.1	NCBI: cellular organisms; Bacteria; Terrabacteria group; Actinobacteria; Actinomycetia; Corynebacteriales; Mycobacteriaceae; Mycobacterium; Mycobacterium tuberculosis complex; Mycobacterium tuberculosis;	NCBI: 1773	4400867	99.94	0.00	Yes
NCBI Assembly	GCA_001848435.1	NCBI: cellular organisms; Bacteria; Terrabacteria group; Actinobacteria; Actinomycetia; Corynebacteriales; Mycobacteriaceae; Mycobacterium; Mycobacterium tuberculosis complex; Mycobacterium tuberculosis;	NCBI: 1773	4398075	99.94	0.00	Yes
NCBI Assembly	GCA_001673175.1	NCBI: cellular organisms; Bacteria; Terrabacteria group; Actinobacteria; Actinomycetia; Corynebacteriales; Mycobacteriaceae; Mycobacterium; Mycobacterium avium complex (MAC); Mycobacterium colombiense;	NCBI: 339268	5718497	99.61	1.36	Yes
NCBI Assembly	GCA_001841175.1	NCBI: cellular organisms; Bacteria; Terrabacteria group; Actinobacteria; Actinomycetia; Corynebacteriales; Mycobacteriaceae; Mycobacterium; Mycobacterium tuberculosis complex; Mycobacterium tuberculosis;	NCBI: 1773	4411524	99.94	0.00	Yes
NCBI Assembly	GCA_001843745.1	NCBI: cellular organisms; Bacteria; Terrabacteria group; Actinobacteria; Actinomycetia; Corynebacteriales; Mycobacteriaceae; Mycobacterium; Mycobacterium tuberculosis complex; Mycobacterium tuberculosis;	NCBI: 1773	4347835	99.94	0.00	Yes
NCBI Assembly	GCA_001851245.1	NCBI: cellular organisms; Bacteria; Terrabacteria group; Actinobacteria; Actinomycetia; Corynebacteriales; Mycobacteriaceae; Mycobacterium; Mycobacterium tuberculosis complex; Mycobacterium tuberculosis;	NCBI: 1773	4379104	99.94	0.00	Yes
NCBI Assembly	GCA_001665445.1	NCBI: cellular organisms; Bacteria; Terrabacteria group; Actinobacteria; Actinomycetia; Corynebacteriales; Mycobacteriaceae; Mycolicibacterium; Mycolicibacterium setense;	NCBI: 431269	6327085	99.06	1.32	Yes
NCBI Assembly	GCA_001841845.1	NCBI: cellular organisms; Bacteria; Terrabacteria group; Actinobacteria; Actinomycetia; Corynebacteriales; Mycobacteriaceae; Mycobacterium; Mycobacterium tuberculosis complex; Mycobacterium tuberculosis;	NCBI: 1773	4401239	99.94	0.00	Yes

NCBI Assembly	GCA_001853665.1	NCBI: cellular organisms; Bacteria; Terrabacteria group; Actinobacteria; Actinomycetia; Corynebacteriales; Mycobacteriaceae; Mycobacteroides; Mycobacteroides chelonae;	NCBI: 1774	5118876	99.50	0.03	Yes
NCBI Assembly	GCA_001839965.1	NCBI: cellular organisms; Bacteria; Terrabacteria group; Actinobacteria; Actinomycetia; Corynebacteriales; Mycobacteriaceae; Mycobacterium; Mycobacterium tuberculosis complex; Mycobacterium tuberculosis;	NCBI: 1773	4415407	99.94	0.00	Yes
NCBI Assembly	GCA_001840905.1	NCBI: cellular organisms; Bacteria; Terrabacteria group; Actinobacteria; Actinomycetia; Corynebacteriales; Mycobacteriaceae; Mycobacterium; Mycobacterium tuberculosis complex; Mycobacterium tuberculosis;	NCBI: 1773	4409384	99.61	0.00	Yes
NCBI Assembly	GCA_001842485.1	NCBI: cellular organisms; Bacteria; Terrabacteria group; Actinobacteria; Actinomycetia; Corynebacteriales; Mycobacteriaceae; Mycobacterium; Mycobacterium tuberculosis complex; Mycobacterium tuberculosis;	NCBI: 1773	4383769	99.94	0.00	Yes
NCBI Assembly	GCA_001848445.1	NCBI: cellular organisms; Bacteria; Terrabacteria group; Actinobacteria; Actinomycetia; Corynebacteriales; Mycobacteriaceae; Mycobacterium; Mycobacterium tuberculosis complex; Mycobacterium tuberculosis;	NCBI: 1773	4384193	99.94	0.00	Yes
NCBI Assembly	GCA_001673195.1	NCBI: cellular organisms; Bacteria; Terrabacteria group; Actinobacteria; Actinomycetia; Corynebacteriales; Mycobacteriaceae; Mycobacterium; Mycobacterium avium complex (MAC); Mycobacterium colombiense;	NCBI: 339268	5617729	99.50	1.16	Yes
NCBI Assembly	GCA_001843465.1	NCBI: cellular organisms; Bacteria; Terrabacteria group; Actinobacteria; Actinomycetia; Corynebacteriales; Mycobacteriaceae; Mycobacterium; Mycobacterium tuberculosis complex; Mycobacterium tuberculosis;	NCBI: 1773	4390908	99.94	0.00	Yes
NCBI Assembly	GCA_001841195.1	NCBI: cellular organisms; Bacteria; Terrabacteria group; Actinobacteria; Actinomycetia; Corynebacteriales; Mycobacteriaceae; Mycobacterium; Mycobacterium tuberculosis complex; Mycobacterium tuberculosis;	NCBI: 1773	4389976	99.61	0.00	Yes
NCBI Assembly	GCA_001843765.1	NCBI: cellular organisms; Bacteria; Terrabacteria group; Actinobacteria; Actinomycetia; Corynebacteriales; Mycobacteriaceae; Mycobacterium; Mycobacterium tuberculosis complex; Mycobacterium tuberculosis;	NCBI: 1773	4371446	99.94	0.00	Yes
NCBI Assembly	GCA_001851265.1	NCBI: cellular organisms; Bacteria; Terrabacteria group; Actinobacteria; Actinomycetia; Corynebacteriales; Mycobacteriaceae; Mycobacterium; Mycobacterium tuberculosis complex; Mycobacterium tuberculosis;	NCBI: 1773	4400519	99.61	0.00	Yes
NCBI Assembly	GCA_001665455.1	NCBI: cellular organisms; Bacteria; Terrabacteria group; Actinobacteria; Actinomycetia; Corynebacteriales; Mycobacteriaceae; Mycolicibacterium; Mycolicibacterium fortuitum;	NCBI: 1766	6358417	99.81	0.13	Yes
NCBI Assembly	GCA_001841865.1	NCBI: cellular organisms; Bacteria; Terrabacteria group; Actinobacteria; Actinomycetia; Corynebacteriales; Mycobacteriaceae; Mycobacterium; Mycobacterium tuberculosis complex; Mycobacterium tuberculosis;	NCBI: 1773	4382353	99.94	0.00	Yes
NCBI Assembly	GCA_001853685.1	NCBI: cellular organisms; Bacteria; Terrabacteria group; Actinobacteria; Actinomycetia; Corynebacteriales; Mycobacteriaceae; Mycobacteroides; Mycobacteroides chelonae;	NCBI: 1774	5142009	99.83	0.00	Yes
NCBI Assembly	GCA_001840925.1	NCBI: cellular organisms; Bacteria; Terrabacteria group; Actinobacteria; Actinomycetia; Corynebacteriales; Mycobacteriaceae; Mycobacterium; Mycobacterium tuberculosis complex; Mycobacterium tuberculosis;	NCBI: 1773	4363661	99.61	0.00	Yes
NCBI Assembly	GCA_001839975.1	NCBI: cellular organisms; Bacteria; Terrabacteria group; Actinobacteria; Actinomycetia; Corynebacteriales; Mycobacteriaceae; Mycobacterium; Mycobacterium tuberculosis complex; Mycobacterium tuberculosis;	NCBI: 1773	4390247	99.94	0.00	Yes
NCBI Assembly	GCA_001848475.1	NCBI: cellular organisms; Bacteria; Terrabacteria group; Actinobacteria; Actinomycetia; Corynebacteriales; Mycobacteriaceae; Mycobacterium; Mycobacterium tuberculosis complex; Mycobacterium tuberculosis;	NCBI: 1773	4393509	99.94	0.00	Yes
NCBI Assembly	GCA_001842505.1	NCBI: cellular organisms; Bacteria; Terrabacteria group; Actinobacteria; Actinomycetia; Corynebacteriales; Mycobacteriaceae; Mycobacterium; Mycobacterium tuberculosis complex; Mycobacterium tuberculosis;	NCBI: 1773	4402237	99.94	0.00	Yes
NCBI Assembly	GCA_001673235.1	NCBI: cellular organisms; Bacteria; Terrabacteria group; Actinobacteria; Actinomycetia; Corynebacteriales; Mycobacteriaceae; Mycobacterium; Mycobacterium avium complex (MAC); Mycobacterium marseillense;	NCBI: 701042	5255222	99.94	0.44	Yes

NCBI Assembly	GCA_001843475.1	NCBI: cellular organisms; Bacteria; Terrabacteria group; Actinobacteria; Actinomycetia; Corynebacteriales; Mycobacteriaceae; Mycobacterium; Mycobacterium tuberculosis complex; Mycobacterium tuberculosis;	NCBI: 1773	4406215	99.94	0.00	Yes
NCBI Assembly	GCA_001841205.1	NCBI: cellular organisms; Bacteria; Terrabacteria group; Actinobacteria; Actinomycetia; Corynebacteriales; Mycobacteriaceae; Mycobacterium; Mycobacterium tuberculosis complex; Mycobacterium tuberculosis;	NCBI: 1773	4393648	99.61	0.00	Yes
NCBI Assembly	GCA_001665535.1	NCBI: cellular organisms; Bacteria; Terrabacteria group; Actinobacteria; Actinomycetia; Corynebacteriales; Mycobacteriaceae; Mycobacterium; unclassified Mycobacterium;	NCBI: 1834105	5552786	100.00	1.36	Yes
NCBI Assembly	GCA_001843785.1	NCBI: cellular organisms; Bacteria; Terrabacteria group; Actinobacteria; Actinomycetia; Corynebacteriales; Mycobacteriaceae; Mycobacterium; Mycobacterium tuberculosis complex; Mycobacterium tuberculosis;	NCBI: 1773	4369579	99.94	0.00	Yes
NCBI Assembly	GCA_001851275.1	NCBI: cellular organisms; Bacteria; Terrabacteria group; Actinobacteria; Actinomycetia; Corynebacteriales; Mycobacteriaceae; Mycobacterium; Mycobacterium tuberculosis complex; Mycobacterium tuberculosis;	NCBI: 1773	4398306	99.61	0.00	Yes
NCBI Assembly	GCA_001841885.1	NCBI: cellular organisms; Bacteria; Terrabacteria group; Actinobacteria; Actinomycetia; Corynebacteriales; Mycobacteriaceae; Mycobacterium; Mycobacterium tuberculosis complex; Mycobacterium tuberculosis;	NCBI: 1773	4392509	99.94	0.00	Yes
NCBI Assembly	GCA_001673255.1	NCBI: cellular organisms; Bacteria; Terrabacteria group; Actinobacteria; Actinomycetia; Corynebacteriales; Mycobacteriaceae; Mycobacterium; Mycobacterium gordonae;	NCBI: 1778	6926928	100.00	2.44	Yes
NCBI Assembly	GCA_001839985.1	NCBI: cellular organisms; Bacteria; Terrabacteria group; Actinobacteria; Actinomycetia; Corynebacteriales; Mycobacteriaceae; Mycobacterium; Mycobacterium tuberculosis complex; Mycobacterium tuberculosis;	NCBI: 1773	4442782	99.94	0.00	Yes
NCBI Assembly	GCA_001848505.1	NCBI: cellular organisms; Bacteria; Terrabacteria group; Actinobacteria; Actinomycetia; Corynebacteriales; Mycobacteriaceae; Mycobacterium; Mycobacterium tuberculosis complex; Mycobacterium tuberculosis;	NCBI: 1773	4387724	99.94	0.00	Yes
NCBI Assembly	GCA_001842515.1	NCBI: cellular organisms; Bacteria; Terrabacteria group; Actinobacteria; Actinomycetia; Corynebacteriales; Mycobacteriaceae; Mycobacterium; Mycobacterium tuberculosis complex; Mycobacterium tuberculosis;	NCBI: 1773	4403817	99.94	0.00	Yes
NCBI Assembly	GCA_001840935.1	NCBI: cellular organisms; Bacteria; Terrabacteria group; Actinobacteria; Actinomycetia; Corynebacteriales; Mycobacteriaceae; Mycobacterium; Mycobacterium tuberculosis complex; Mycobacterium tuberculosis;	NCBI: 1773	4390482	99.28	0.00	Yes
NCBI Assembly	GCA_001853695.1	NCBI: cellular organisms; Bacteria; Terrabacteria group; Actinobacteria; Actinomycetia; Corynebacteriales; Mycobacteriaceae; Mycobacteroides; Mycobacteroides chelonae;	NCBI: 1774	4919524	99.50	0.03	Yes
NCBI Assembly	GCA_001665545.1	NCBI: cellular organisms; Bacteria; Terrabacteria group; Actinobacteria; Actinomycetia; Corynebacteriales; Mycobacteriaceae; Mycolicibacterium; Mycolicibacterium fortuitum;	NCBI: 1766	6686242	99.81	0.13	Yes
NCBI Assembly	GCA_001841245.1	NCBI: cellular organisms; Bacteria; Terrabacteria group; Actinobacteria; Actinomycetia; Corynebacteriales; Mycobacteriaceae; Mycobacterium; Mycobacterium tuberculosis complex; Mycobacterium tuberculosis;	NCBI: 1773	4377763	99.94	0.00	Yes
NCBI Assembly	GCA_001843505.1	NCBI: cellular organisms; Bacteria; Terrabacteria group; Actinobacteria; Actinomycetia; Corynebacteriales; Mycobacteriaceae; Mycobacterium; Mycobacterium tuberculosis complex; Mycobacterium tuberculosis;	NCBI: 1773	4400487	99.94	0.00	Yes
NCBI Assembly	GCA_001843805.1	NCBI: cellular organisms; Bacteria; Terrabacteria group; Actinobacteria; Actinomycetia; Corynebacteriales; Mycobacteriaceae; Mycobacterium; Mycobacterium tuberculosis complex; Mycobacterium tuberculosis;	NCBI: 1773	4395389	99.94	0.00	Yes
NCBI Assembly	GCA_001851295.1	NCBI: cellular organisms; Bacteria; Terrabacteria group; Actinobacteria; Actinomycetia; Corynebacteriales; Mycobacteriaceae; Mycobacterium; Mycobacterium tuberculosis complex; Mycobacterium tuberculosis;	NCBI: 1773	4380133	99.61	0.00	Yes
NCBI Assembly	GCA_001841895.1	NCBI: cellular organisms; Bacteria; Terrabacteria group; Actinobacteria; Actinomycetia; Corynebacteriales; Mycobacteriaceae; Mycobacterium; Mycobacterium tuberculosis complex; Mycobacterium tuberculosis;	NCBI: 1773	4390489	99.94	0.00	Yes

NCBI Assembly	GCA_001673265.1	NCBI: cellular organisms; Bacteria; Terrabacteria group; Actinobacteria; Actinomycetia; Corynebacteriales; Mycobacteriaceae; Mycolicibacterium; Mycolicibacterium conceptionense;	NCBI: 451644	6496279	99.94	0.00	Yes
NCBI Assembly	GCA_001840005.1	NCBI: cellular organisms; Bacteria; Terrabacteria group; Actinobacteria; Actinomycetia; Corynebacteriales; Mycobacteriaceae; Mycobacterium; Mycobacterium tuberculosis complex; Mycobacterium tuberculosis;	NCBI: 1773	4408389	99.94	0.00	Yes
NCBI Assembly	GCA_001842525.1	NCBI: cellular organisms; Bacteria; Terrabacteria group; Actinobacteria; Actinomycetia; Corynebacteriales; Mycobacteriaceae; Mycobacterium; Mycobacterium tuberculosis complex; Mycobacterium tuberculosis;	NCBI: 1773	4401408	99.94	0.00	Yes
NCBI Assembly	GCA_001853715.1	NCBI: cellular organisms; Bacteria; Terrabacteria group; Actinobacteria; Actinomycetia; Corynebacteriales; Mycobacteriaceae; Mycobacteroides; Mycobacteroides chelonae;	NCBI: 1774	5204027	99.83	0.00	Yes
NCBI Assembly	GCA_001665575.1	NCBI: cellular organisms; Bacteria; Terrabacteria group; Actinobacteria; Actinomycetia; Corynebacteriales; Mycobacteriaceae; Mycobacterium; unclassified Mycobacterium;	NCBI: 1834111	4763489	90.60	0.00	Yes
NCBI Assembly	GCA_001840945.1	NCBI: cellular organisms; Bacteria; Terrabacteria group; Actinobacteria; Actinomycetia; Corynebacteriales; Mycobacteriaceae; Mycobacterium; Mycobacterium tuberculosis complex; Mycobacterium tuberculosis;	NCBI: 1773	4393716	99.61	0.00	Yes
NCBI Assembly	GCA_001848515.1	NCBI: cellular organisms; Bacteria; Terrabacteria group; Actinobacteria; Actinomycetia; Corynebacteriales; Mycobacteriaceae; Mycobacterium; Mycobacterium tuberculosis complex; Mycobacterium tuberculosis;	NCBI: 1773	4388396	99.61	0.00	Yes
NCBI Assembly	GCA_001841265.1	NCBI: cellular organisms; Bacteria; Terrabacteria group; Actinobacteria; Actinomycetia; Corynebacteriales; Mycobacteriaceae; Mycobacterium; Mycobacterium tuberculosis complex; Mycobacterium tuberculosis;	NCBI: 1773	4390116	99.61	0.00	Yes
NCBI Assembly	GCA_001843815.1	NCBI: cellular organisms; Bacteria; Terrabacteria group; Actinobacteria; Actinomycetia; Corynebacteriales; Mycobacteriaceae; Mycobacterium; Mycobacterium tuberculosis complex; Mycobacterium tuberculosis;	NCBI: 1773	4381143	99.94	0.00	Yes
NCBI Assembly	GCA_001843515.1	NCBI: cellular organisms; Bacteria; Terrabacteria group; Actinobacteria; Actinomycetia; Corynebacteriales; Mycobacteriaceae; Mycobacterium; Mycobacterium tuberculosis complex; Mycobacterium tuberculosis;	NCBI: 1773	4406359	99.94	0.00	Yes
NCBI Assembly	GCA_001851325.1	NCBI: cellular organisms; Bacteria; Terrabacteria group; Actinobacteria; Actinomycetia; Corynebacteriales; Mycobacteriaceae; Mycobacterium; Mycobacterium tuberculosis complex; Mycobacterium tuberculosis;	NCBI: 1773	4381388	99.61	0.00	Yes
NCBI Assembly	GCA_001673275.1	NCBI: cellular organisms; Bacteria; Terrabacteria group; Actinobacteria; Actinomycetia; Corynebacteriales; Mycobacteriaceae; Mycolicibacterium; Mycolicibacterium fortuitum;	NCBI: 1766	6301194	99.81	0.57	Yes
NCBI Assembly	GCA_001840045.1	NCBI: cellular organisms; Bacteria; Terrabacteria group; Actinobacteria; Actinomycetia; Corynebacteriales; Mycobacteriaceae; Mycobacterium; Mycobacterium tuberculosis complex; Mycobacterium tuberculosis;	NCBI: 1773	4407246	99.94	0.00	Yes
NCBI Assembly	GCA_001841905.1	NCBI: cellular organisms; Bacteria; Terrabacteria group; Actinobacteria; Actinomycetia; Corynebacteriales; Mycobacteriaceae; Mycobacterium; Mycobacterium tuberculosis complex; Mycobacterium tuberculosis;	NCBI: 1773	4386998	99.94	0.00	Yes
NCBI Assembly	GCA_001853745.1	NCBI: cellular organisms; Bacteria; Terrabacteria group; Actinobacteria; Actinomycetia; Corynebacteriales; Mycobacteriaceae; Mycobacteroides; Mycobacteroides chelonae;	NCBI: 1774	5202317	99.83	0.00	Yes
NCBI Assembly	GCA_001665605.1	NCBI: cellular organisms; Bacteria; Terrabacteria group; Actinobacteria; Actinomycetia; Corynebacteriales; Mycobacteriaceae; Mycobacterium; unclassified Mycobacterium;	NCBI: 1834112	5382186	99.94	0.49	Yes
NCBI Assembly	GCA_001842565.1	NCBI: cellular organisms; Bacteria; Terrabacteria group; Actinobacteria; Actinomycetia; Corynebacteriales; Mycobacteriaceae; Mycobacterium; Mycobacterium tuberculosis complex; Mycobacterium tuberculosis;	NCBI: 1773	4396404	99.94	0.33	Yes
NCBI Assembly	GCA_001848545.1	NCBI: cellular organisms; Bacteria; Terrabacteria group; Actinobacteria; Actinomycetia; Corynebacteriales; Mycobacteriaceae; Mycobacterium; Mycobacterium tuberculosis complex; Mycobacterium tuberculosis;	NCBI: 1773	4371605	99.94	0.00	Yes

NCBI Assembly	GCA_001841275.1	NCBI: cellular organisms; Bacteria; Terrabacteria group; Actinobacteria; Actinomycetia; Corynebacteriales; Mycobacteriaceae; Mycobacterium; Mycobacterium tuberculosis complex; Mycobacterium tuberculosis;	NCBI: 1773	4405170	99.94	0.00	Yes
NCBI Assembly	GCA_001843845.1	NCBI: cellular organisms; Bacteria; Terrabacteria group; Actinobacteria; Actinomycetia; Corynebacteriales; Mycobacteriaceae; Mycobacterium; Mycobacterium tuberculosis complex; Mycobacterium tuberculosis;	NCBI: 1773	4375596	99.94	0.00	Yes
NCBI Assembly	GCA_001840985.1	NCBI: cellular organisms; Bacteria; Terrabacteria group; Actinobacteria; Actinomycetia; Corynebacteriales; Mycobacteriaceae; Mycobacterium; Mycobacterium tuberculosis complex; Mycobacterium tuberculosis;	NCBI: 1773	4372024	99.61	0.00	Yes
NCBI Assembly	GCA_001843535.1	NCBI: cellular organisms; Bacteria; Terrabacteria group; Actinobacteria; Actinomycetia; Corynebacteriales; Mycobacteriaceae; Mycobacterium; Mycobacterium tuberculosis complex; Mycobacterium tuberculosis;	NCBI: 1773	4374389	99.94	0.33	Yes
NCBI Assembly	GCA_001851345.1	NCBI: cellular organisms; Bacteria; Terrabacteria group; Actinobacteria; Actinomycetia; Corynebacteriales; Mycobacteriaceae; Mycobacterium; Mycobacterium tuberculosis complex; Mycobacterium tuberculosis;	NCBI: 1773	4358692	99.61	0.00	Yes
NCBI Assembly	GCA_001665615.1	NCBI: cellular organisms; Bacteria; Terrabacteria group; Actinobacteria; Actinomycetia; Corynebacteriales; Mycobacteriaceae; Mycobacterium; unclassified Mycobacterium;	NCBI: 1834115	5293011	100.00	0.45	Yes
NCBI Assembly	GCA_001673315.1	NCBI: cellular organisms; Bacteria; Terrabacteria group; Actinobacteria; Actinomycetia; Corynebacteriales; Mycobacteriaceae; Mycobacterium; Mycobacterium asiaticum;	NCBI: 1790	5432256	99.53	0.21	Yes
NCBI Assembly	GCA_001840055.1	NCBI: cellular organisms; Bacteria; Terrabacteria group; Actinobacteria; Actinomycetia; Corynebacteriales; Mycobacteriaceae; Mycobacterium; Mycobacterium tuberculosis complex; Mycobacterium tuberculosis;	NCBI: 1773	4402878	99.94	0.00	Yes
NCBI Assembly	GCA_001841945.1	NCBI: cellular organisms; Bacteria; Terrabacteria group; Actinobacteria; Actinomycetia; Corynebacteriales; Mycobacteriaceae; Mycobacterium; Mycobacterium tuberculosis complex; Mycobacterium tuberculosis;	NCBI: 1773	4389551	99.94	0.00	Yes
NCBI Assembly	GCA_001853765.1	NCBI: cellular organisms; Bacteria; Terrabacteria group; Actinobacteria; Actinomycetia; Corynebacteriales; Mycobacteriaceae; Mycobacteroides; Mycobacteroides saopaulense;	NCBI: 1578165	4997261	99.50	0.59	Yes
NCBI Assembly	GCA_001842585.1	NCBI: cellular organisms; Bacteria; Terrabacteria group; Actinobacteria; Actinomycetia; Corynebacteriales; Mycobacteriaceae; Mycobacterium; Mycobacterium tuberculosis complex; Mycobacterium tuberculosis;	NCBI: 1773	4409061	99.94	0.00	Yes
NCBI Assembly	GCA_001841005.1	NCBI: cellular organisms; Bacteria; Terrabacteria group; Actinobacteria; Actinomycetia; Corynebacteriales; Mycobacteriaceae; Mycobacterium; Mycobacterium tuberculosis complex; Mycobacterium tuberculosis;	NCBI: 1773	4390550	99.94	0.00	Yes
NCBI Assembly	GCA_001843855.1	NCBI: cellular organisms; Bacteria; Terrabacteria group; Actinobacteria; Actinomycetia; Corynebacteriales; Mycobacteriaceae; Mycobacterium; Mycobacterium tuberculosis complex; Mycobacterium tuberculosis;	NCBI: 1773	4393191	99.94	0.00	Yes
NCBI Assembly	GCA_001841285.1	NCBI: cellular organisms; Bacteria; Terrabacteria group; Actinobacteria; Actinomycetia; Corynebacteriales; Mycobacteriaceae; Mycobacterium; Mycobacterium tuberculosis complex; Mycobacterium tuberculosis;	NCBI: 1773	4389201	99.61	0.00	Yes
NCBI Assembly	GCA_001848555.1	NCBI: cellular organisms; Bacteria; Terrabacteria group; Actinobacteria; Actinomycetia; Corynebacteriales; Mycobacteriaceae; Mycobacterium; Mycobacterium tuberculosis complex; Mycobacterium tuberculosis;	NCBI: 1773	4398989	99.94	0.33	Yes
NCBI Assembly	GCA_001843565.1	NCBI: cellular organisms; Bacteria; Terrabacteria group; Actinobacteria; Actinomycetia; Corynebacteriales; Mycobacteriaceae; Mycobacterium; Mycobacterium tuberculosis complex; Mycobacterium tuberculosis;	NCBI: 1773	4400750	99.94	0.00	Yes
NCBI Assembly	GCA_001851365.1	NCBI: cellular organisms; Bacteria; Terrabacteria group; Actinobacteria; Actinomycetia; Corynebacteriales; Mycobacteriaceae; Mycobacterium; Mycobacterium tuberculosis complex; Mycobacterium tuberculosis;	NCBI: 1773	4391326	99.94	0.00	Yes
NCBI Assembly	GCA_001673325.1	NCBI: cellular organisms; Bacteria; Terrabacteria group; Actinobacteria; Actinomycetia; Corynebacteriales; Mycobacteriaceae; Mycolicibacterium; Mycolicibacterium fortuitum;	NCBI: 1766	6743562	99.81	0.88	Yes

NCBI Assembly	GCA_001667205.1	NCBI: cellular organisms; Bacteria; Terrabacteria group; Actinobacteria; Actinomycetia; Corynebacteriales; Mycobacteriaceae; Mycolicibacterium; Mycolicibacterium peregrinum;	NCBI: 43304	6536987	99.62	0.88	Yes
NCBI Assembly	GCA_001840065.1	NCBI: cellular organisms; Bacteria; Terrabacteria group; Actinobacteria; Actinomycetia; Corynebacteriales; Mycobacteriaceae; Mycobacterium; Mycobacterium tuberculosis complex; Mycobacterium tuberculosis;	NCBI: 1773	4353486	99.94	0.00	Yes
NCBI Assembly	GCA_001842595.1	NCBI: cellular organisms; Bacteria; Terrabacteria group; Actinobacteria; Actinomycetia; Corynebacteriales; Mycobacteriaceae; Mycobacterium; Mycobacterium tuberculosis complex; Mycobacterium tuberculosis;	NCBI: 1773	4405534	99.94	0.00	Yes
NCBI Assembly	GCA_001841965.1	NCBI: cellular organisms; Bacteria; Terrabacteria group; Actinobacteria; Actinomycetia; Corynebacteriales; Mycobacteriaceae; Mycobacterium; Mycobacterium tuberculosis complex; Mycobacterium tuberculosis;	NCBI: 1773	4396361	99.94	0.00	Yes
NCBI Assembly	GCA_001853775.1	NCBI: cellular organisms; Bacteria; Terrabacteria group; Actinobacteria; Actinomycetia; Corynebacteriales; Mycobacteriaceae; Mycobacteroides; Mycobacteroides chelonae;	NCBI: 1774	5252925	99.50	0.03	Yes
NCBI Assembly	GCA_001841345.1	NCBI: cellular organisms; Bacteria; Terrabacteria group; Actinobacteria; Actinomycetia; Corynebacteriales; Mycobacteriaceae; Mycobacterium; Mycobacterium tuberculosis complex; Mycobacterium tuberculosis;	NCBI: 1773	4403833	99.61	0.00	Yes
NCBI Assembly	GCA_001841015.1	NCBI: cellular organisms; Bacteria; Terrabacteria group; Actinobacteria; Actinomycetia; Corynebacteriales; Mycobacteriaceae; Mycobacterium; Mycobacterium tuberculosis complex; Mycobacterium tuberculosis;	NCBI: 1773	4404779	99.61	0.00	Yes
NCBI Assembly	GCA_001843885.1	NCBI: cellular organisms; Bacteria; Terrabacteria group; Actinobacteria; Actinomycetia; Corynebacteriales; Mycobacteriaceae; Mycobacterium; Mycobacterium tuberculosis complex; Mycobacterium tuberculosis;	NCBI: 1773	4394421	99.94	0.00	Yes
NCBI Assembly	GCA_001848585.1	NCBI: cellular organisms; Bacteria; Terrabacteria group; Actinobacteria; Actinomycetia; Corynebacteriales; Mycobacteriaceae; Mycobacterium; Mycobacterium tuberculosis complex; Mycobacterium tuberculosis;	NCBI: 1773	4400630	99.94	0.00	Yes
NCBI Assembly	GCA_001843585.1	NCBI: cellular organisms; Bacteria; Terrabacteria group; Actinobacteria; Actinomycetia; Corynebacteriales; Mycobacteriaceae; Mycobacterium; Mycobacterium tuberculosis complex; Mycobacterium tuberculosis;	NCBI: 1773	4391092	99.94	0.00	Yes
NCBI Assembly	GCA_001851385.1	NCBI: cellular organisms; Bacteria; Terrabacteria group; Actinobacteria; Actinomycetia; Corynebacteriales; Mycobacteriaceae; Mycobacterium; Mycobacterium tuberculosis complex; Mycobacterium tuberculosis;	NCBI: 1773	4396002	99.61	0.00	Yes
NCBI Assembly	GCA_001667925.1	NCBI: cellular organisms; Bacteria; Terrabacteria group; Actinobacteria; Actinomycetia; Corynebacteriales; Mycobacteriaceae; Mycobacterium; unclassified Mycobacterium;	NCBI: 1834134	5672622	99.94	1.67	Yes
NCBI Assembly	GCA_001667225.1	NCBI: cellular organisms; Bacteria; Terrabacteria group; Actinobacteria; Actinomycetia; Corynebacteriales; Mycobacteriaceae; Mycolicibacterium; Mycolicibacterium conceptionense;	NCBI: 451644	6386603	99.87	0.00	Yes
NCBI Assembly	GCA_001841975.1	NCBI: cellular organisms; Bacteria; Terrabacteria group; Actinobacteria; Actinomycetia; Corynebacteriales; Mycobacteriaceae; Mycobacterium; Mycobacterium tuberculosis complex; Mycobacterium tuberculosis;	NCBI: 1773	4352151	99.94	0.00	Yes
NCBI Assembly	GCA_001840075.1	NCBI: cellular organisms; Bacteria; Terrabacteria group; Actinobacteria; Actinomycetia; Corynebacteriales; Mycobacteriaceae; Mycobacterium; Mycobacterium tuberculosis complex; Mycobacterium tuberculosis;	NCBI: 1773	4419147	99.94	0.00	Yes
NCBI Assembly	GCA_001841355.1	NCBI: cellular organisms; Bacteria; Terrabacteria group; Actinobacteria; Actinomycetia; Corynebacteriales; Mycobacteriaceae; Mycobacterium; Mycobacterium tuberculosis complex; Mycobacterium tuberculosis;	NCBI: 1773	4393703	99.94	0.00	Yes
NCBI Assembly	GCA_001853795.1	NCBI: cellular organisms; Bacteria; Terrabacteria group; Actinobacteria; Actinomycetia; Corynebacteriales; Mycobacteriaceae; Mycobacteroides; Mycobacteroides franklinii;	NCBI: 948102	5023635	99.67	1.19	Yes
NCBI Assembly	GCA_001841045.1	NCBI: cellular organisms; Bacteria; Terrabacteria group; Actinobacteria; Actinomycetia; Corynebacteriales; Mycobacteriaceae; Mycobacterium; Mycobacterium tuberculosis complex; Mycobacterium tuberculosis;	NCBI: 1773	4407797	99.94	0.00	Yes

NCBI Assembly	GCA_001842605.1	NCBI: cellular organisms; Bacteria; Terrabacteria group; Actinobacteria; Actinomycetia; Corynebacteriales; Mycobacteriaceae; Mycobacterium; Mycobacterium tuberculosis complex; Mycobacterium tuberculosis;	NCBI: 1773	4411160	99.94	0.00	Yes
NCBI Assembly	GCA_001843895.1	NCBI: cellular organisms; Bacteria; Terrabacteria group; Actinobacteria; Actinomycetia; Corynebacteriales; Mycobacteriaceae; Mycobacterium; Mycobacterium tuberculosis complex; Mycobacterium tuberculosis;	NCBI: 1773	4387581	99.94	0.00	Yes
NCBI Assembly	GCA_001848595.1	NCBI: cellular organisms; Bacteria; Terrabacteria group; Actinobacteria; Actinomycetia; Corynebacteriales; Mycobacteriaceae; Mycobacterium; Mycobacterium tuberculosis complex; Mycobacterium tuberculosis;	NCBI: 1773	4401598	99.94	0.00	Yes
NCBI Assembly	GCA_001843605.1	NCBI: cellular organisms; Bacteria; Terrabacteria group; Actinobacteria; Actinomycetia; Corynebacteriales; Mycobacteriaceae; Mycobacterium; Mycobacterium tuberculosis complex; Mycobacterium tuberculosis;	NCBI: 1773	4378693	99.94	0.00	Yes
NCBI Assembly	GCA_001851395.1	NCBI: cellular organisms; Bacteria; Terrabacteria group; Actinobacteria; Actinomycetia; Corynebacteriales; Mycobacteriaceae; Mycobacterium; Mycobacterium tuberculosis complex; Mycobacterium tuberculosis;	NCBI: 1773	4383246	99.94	0.00	Yes
NCBI Assembly	GCA_001667965.1	NCBI: cellular organisms; Bacteria; Terrabacteria group; Actinobacteria; Actinomycetia; Corynebacteriales; Mycobacteriaceae; Mycolicibacterium; Mycolicibacterium conceptionense;	NCBI: 451644	6386103	100.00	0.00	Yes
NCBI Assembly	GCA_001667235.1	NCBI: cellular organisms; Bacteria; Terrabacteria group; Actinobacteria; Actinomycetia; Corynebacteriales; Mycobacteriaceae; Mycolicibacterium; Mycolicibacterium conceptionense;	NCBI: 451644	6392622	99.87	0.00	Yes
NCBI Assembly	GCA_001842005.1	NCBI: cellular organisms; Bacteria; Terrabacteria group; Actinobacteria; Actinomycetia; Corynebacteriales; Mycobacteriaceae; Mycobacterium; Mycobacterium tuberculosis complex; Mycobacterium tuberculosis;	NCBI: 1773	4408389	99.94	0.00	Yes
NCBI Assembly	GCA_001844545.1	NCBI: cellular organisms; Bacteria; Terrabacteria group; Actinobacteria; Actinomycetia; Corynebacteriales; Mycobacteriaceae; Mycobacterium; Mycobacterium tuberculosis complex; Mycobacterium tuberculosis;	NCBI: 1773	4377672	99.94	0.00	Yes
NCBI Assembly	GCA_001844245.1	NCBI: cellular organisms; Bacteria; Terrabacteria group; Actinobacteria; Actinomycetia; Corynebacteriales; Mycobacteriaceae; Mycobacterium; Mycobacterium tuberculosis complex; Mycobacterium tuberculosis;	NCBI: 1773	4402999	99.94	0.00	Yes
NCBI Assembly	GCA_001849235.1	NCBI: cellular organisms; Bacteria; Terrabacteria group; Actinobacteria; Actinomycetia; Corynebacteriales; Mycobacteriaceae; Mycobacterium; Mycobacterium tuberculosis complex; Mycobacterium tuberculosis;	NCBI: 1773	4383130	99.61	0.00	Yes
NCBI Assembly	GCA_001846425.1	NCBI: cellular organisms; Bacteria; Terrabacteria group; Actinobacteria; Actinomycetia; Corynebacteriales; Mycobacteriaceae; Mycobacterium; Mycobacterium tuberculosis complex; Mycobacterium tuberculosis;	NCBI: 1773	4378065	99.94	0.00	Yes
NCBI Assembly	GCA_001855985.1	NCBI: cellular organisms; Bacteria; Terrabacteria group; Actinobacteria; Actinomycetia; Corynebacteriales; Mycobacteriaceae; Mycobacterium; Mycobacterium tuberculosis complex; Mycobacterium tuberculosis;	NCBI: 1773	4411174	99.94	0.00	Yes
NCBI Assembly	GCA_001852685.1	NCBI: cellular organisms; Bacteria; Terrabacteria group; Actinobacteria; Actinomycetia; Corynebacteriales; Mycobacteriaceae; Mycobacterium; Mycobacterium tuberculosis complex; Mycobacterium tuberculosis;	NCBI: 1773	4388623	99.94	0.00	Yes
NCBI Assembly	GCA_001847675.1	NCBI: cellular organisms; Bacteria; Terrabacteria group; Actinobacteria; Actinomycetia; Corynebacteriales; Mycobacteriaceae; Mycobacterium; Mycobacterium tuberculosis complex; Mycobacterium tuberculosis;	NCBI: 1773	4389532	99.94	0.00	Yes
NCBI Assembly	GCA_001855365.1	NCBI: cellular organisms; Bacteria; Terrabacteria group; Actinobacteria; Actinomycetia; Corynebacteriales; Mycobacteriaceae; Mycobacterium; Mycobacterium avium complex (MAC); Mycobacterium avium; Mycobacterium avium subsp. paratuberculosis;	NCBI: 1770	4768495	99.28	0.33	Yes
NCBI Assembly	GCA_001667995.1	NCBI: cellular organisms; Bacteria; Terrabacteria group; Actinobacteria; Actinomycetia; Corynebacteriales; Mycobacteriaceae; Mycobacterium; Mycobacterium avium complex (MAC); Mycobacterium mantenii;	NCBI: 560555	5778092	99.71	1.13	Yes
NCBI Assembly	GCA_001852065.1	NCBI: cellular organisms; Bacteria; Terrabacteria group; Actinobacteria; Actinomycetia; Corynebacteriales; Mycobacteriaceae; Mycobacterium; Mycobacterium tuberculosis complex; Mycobacterium tuberculosis;	NCBI: 1773	4368587	99.94	0.00	Yes

NCBI Assembly	GCA_001667265.1	NCBI: cellular organisms; Bacteria; Terrabacteria group; Actinobacteria; Actinomycetia; Corynebacteriales; Mycobacteriaceae; Mycobacterium; unclassified Mycobacterium;	NCBI: 1834121	5755083	100.00	1.20	Yes
NCBI Assembly	GCA_001708265.1	NCBI: cellular organisms; Bacteria; Terrabacteria group; Actinobacteria; Actinomycetia; Corynebacteriales; Mycobacteriaceae; Mycobacterium; Mycobacterium tuberculosis complex; Mycobacterium tuberculosis;	NCBI: 1773	4439387	99.94	0.00	Yes
NCBI Assembly	GCA_001844585.1	NCBI: cellular organisms; Bacteria; Terrabacteria group; Actinobacteria; Actinomycetia; Corynebacteriales; Mycobacteriaceae; Mycobacterium; Mycobacterium tuberculosis complex; Mycobacterium tuberculosis;	NCBI: 1773	4402867	99.61	0.00	Yes
NCBI Assembly	GCA_001844265.1	NCBI: cellular organisms; Bacteria; Terrabacteria group; Actinobacteria; Actinomycetia; Corynebacteriales; Mycobacteriaceae; Mycobacterium; Mycobacterium tuberculosis complex; Mycobacterium tuberculosis;	NCBI: 1773	4389194	99.94	0.00	Yes
NCBI Assembly	GCA_001849265.1	NCBI: cellular organisms; Bacteria; Terrabacteria group; Actinobacteria; Actinomycetia; Corynebacteriales; Mycobacteriaceae; Mycobacterium; Mycobacterium tuberculosis complex; Mycobacterium tuberculosis;	NCBI: 1773	4348916	99.94	0.00	Yes
NCBI Assembly	GCA_001865165.1	NCBI: cellular organisms; Bacteria; Terrabacteria group; Actinobacteria; Actinomycetia; Corynebacteriales; Mycobacteriaceae; Mycolicibacterium; Mycolicibacterium llatzerense;	NCBI: 280871	6697820	99.53	1.07	Yes
NCBI Assembly	GCA_001846435.1	NCBI: cellular organisms; Bacteria; Terrabacteria group; Actinobacteria; Actinomycetia; Corynebacteriales; Mycobacteriaceae; Mycobacterium; Mycobacterium tuberculosis complex; Mycobacterium tuberculosis;	NCBI: 1773	4397216	99.94	0.00	Yes
NCBI Assembly	GCA_001852705.1	NCBI: cellular organisms; Bacteria; Terrabacteria group; Actinobacteria; Actinomycetia; Corynebacteriales; Mycobacteriaceae; Mycobacterium; Mycobacterium tuberculosis complex; Mycobacterium tuberculosis;	NCBI: 1773	4384992	99.61	0.00	Yes
NCBI Assembly	GCA_001847705.1	NCBI: cellular organisms; Bacteria; Terrabacteria group; Actinobacteria; Actinomycetia; Corynebacteriales; Mycobacteriaceae; Mycobacterium; Mycobacterium tuberculosis complex; Mycobacterium tuberculosis;	NCBI: 1773	4402004	99.94	0.00	Yes
NCBI Assembly	GCA_001855375.1	NCBI: cellular organisms; Bacteria; Terrabacteria group; Actinobacteria; Actinomycetia; Corynebacteriales; Mycobacteriaceae; Mycobacterium; Mycobacterium avium complex (MAC); Mycobacterium avium; Mycobacterium avium subsp. paratuberculosis;	NCBI: 1770	4771797	99.28	0.33	Yes
NCBI Assembly	GCA_001668575.1	NCBI: cellular organisms; Bacteria; Terrabacteria group; Actinobacteria; Actinomycetia; Corynebacteriales; Mycobacteriaceae; Mycobacterium; unclassified Mycobacterium;	NCBI: 1834069	5481330	100.00	0.55	Yes
NCBI Assembly	GCA_001667275.1	NCBI: cellular organisms; Bacteria; Terrabacteria group; Actinobacteria; Actinomycetia; Corynebacteriales; Mycobacteriaceae; Mycobacterium; unclassified Mycobacterium;	NCBI: 1834119	5155110	99.94	0.67	Yes
NCBI Assembly	GCA_001852085.1	NCBI: cellular organisms; Bacteria; Terrabacteria group; Actinobacteria; Actinomycetia; Corynebacteriales; Mycobacteriaceae; Mycobacterium; Mycobacterium tuberculosis complex; Mycobacterium tuberculosis;	NCBI: 1773	4390898	99.94	0.00	Yes
NCBI Assembly	GCA_001720305.1	NCBI: cellular organisms; Bacteria; Terrabacteria group; Actinobacteria; Actinomycetia; Corynebacteriales; Mycobacteriaceae; Mycobacterium; Mycobacterium tuberculosis complex; Mycobacterium tuberculosis;	NCBI: 1773	4370082	99.94	0.04	Yes
NCBI Assembly	GCA_001844605.1	NCBI: cellular organisms; Bacteria; Terrabacteria group; Actinobacteria; Actinomycetia; Corynebacteriales; Mycobacteriaceae; Mycobacterium; Mycobacterium tuberculosis complex; Mycobacterium tuberculosis;	NCBI: 1773	4402581	99.94	0.00	Yes
NCBI Assembly	GCA_001846465.1	NCBI: cellular organisms; Bacteria; Terrabacteria group; Actinobacteria; Actinomycetia; Corynebacteriales; Mycobacteriaceae; Mycobacterium; Mycobacterium tuberculosis complex; Mycobacterium tuberculosis;	NCBI: 1773	4413983	99.94	0.00	Yes
NCBI Assembly	GCA_001844275.1	NCBI: cellular organisms; Bacteria; Terrabacteria group; Actinobacteria; Actinomycetia; Corynebacteriales; Mycobacteriaceae; Mycobacterium; Mycobacterium tuberculosis complex; Mycobacterium tuberculosis;	NCBI: 1773	4413342	99.94	0.00	Yes
NCBI Assembly	GCA_001849285.1	NCBI: cellular organisms; Bacteria; Terrabacteria group; Actinobacteria; Actinomycetia; Corynebacteriales; Mycobacteriaceae; Mycobacterium; Mycobacterium tuberculosis complex; Mycobacterium tuberculosis;	NCBI: 1773	4370050	99.61	0.00	Yes

NCBI Assembly	GCA_001847725.1	NCBI: cellular organisms; Bacteria; Terrabacteria group; Actinobacteria; Actinomycetia; Corynebacteriales; Mycobacteriaceae; Mycobacterium; Mycobacterium tuberculosis complex; Mycobacterium tuberculosis;	NCBI: 1773	4393876	99.94	0.00	Yes
NCBI Assembly	GCA_001855435.1	NCBI: cellular organisms; Bacteria; Terrabacteria group; Actinobacteria; Actinomycetia; Corynebacteriales; Mycobacteriaceae; Mycobacterium; Mycobacterium avium complex (MAC); Mycobacterium avium; Mycobacterium avium subsp. paratuberculosis;	NCBI: 1770	4776830	99.28	0.33	Yes
NCBI Assembly	GCA_001852725.1	NCBI: cellular organisms; Bacteria; Terrabacteria group; Actinobacteria; Actinomycetia; Corynebacteriales; Mycobacteriaceae; Mycobacterium; Mycobacterium tuberculosis complex; Mycobacterium tuberculosis;	NCBI: 1773	4366557	99.61	0.00	Yes
NCBI Assembly	GCA_001668615.1	NCBI: cellular organisms; Bacteria; Terrabacteria group; Actinobacteria; Actinomycetia; Corynebacteriales; Mycobacteriaceae; Mycobacterium; unclassified Mycobacterium;	NCBI: 1834077	5078118	99.32	0.23	Yes
NCBI Assembly	GCA_001667305.1	NCBI: cellular organisms; Bacteria; Terrabacteria group; Actinobacteria; Actinomycetia; Corynebacteriales; Mycobacteriaceae; Mycolicibacterium; Mycolicibacterium conceptionense;	NCBI: 451644	6452502	100.00	0.00	Yes
NCBI Assembly	GCA_001852095.1	NCBI: cellular organisms; Bacteria; Terrabacteria group; Actinobacteria; Actinomycetia; Corynebacteriales; Mycobacteriaceae; Mycobacterium; Mycobacterium tuberculosis complex; Mycobacterium tuberculosis;	NCBI: 1773	4377323	99.90	0.00	Yes
NCBI Assembly	GCA_001720315.1	NCBI: cellular organisms; Bacteria; Terrabacteria group; Actinobacteria; Actinomycetia; Corynebacteriales; Mycobacteriaceae; Mycobacterium; Mycobacterium tuberculosis complex; Mycobacterium tuberculosis;	NCBI: 1773	4373806	99.83	0.04	Yes
NCBI Assembly	GCA_001844295.1	NCBI: cellular organisms; Bacteria; Terrabacteria group; Actinobacteria; Actinomycetia; Corynebacteriales; Mycobacteriaceae; Mycobacterium; Mycobacterium tuberculosis complex; Mycobacterium tuberculosis;	NCBI: 1773	4406824	99.94	0.00	Yes
NCBI Assembly	GCA_001846485.1	NCBI: cellular organisms; Bacteria; Terrabacteria group; Actinobacteria; Actinomycetia; Corynebacteriales; Mycobacteriaceae; Mycobacterium; Mycobacterium tuberculosis complex; Mycobacterium tuberculosis;	NCBI: 1773	4350744	99.94	0.00	Yes
NCBI Assembly	GCA_001844615.1	NCBI: cellular organisms; Bacteria; Terrabacteria group; Actinobacteria; Actinomycetia; Corynebacteriales; Mycobacteriaceae; Mycobacterium; Mycobacterium tuberculosis complex; Mycobacterium tuberculosis;	NCBI: 1773	4399714	99.94	0.00	Yes
NCBI Assembly	GCA_001849295.1	NCBI: cellular organisms; Bacteria; Terrabacteria group; Actinobacteria; Actinomycetia; Corynebacteriales; Mycobacteriaceae; Mycobacterium; Mycobacterium tuberculosis complex; Mycobacterium tuberculosis;	NCBI: 1773	4404099	99.94	0.00	Yes
NCBI Assembly	GCA_001847745.1	NCBI: cellular organisms; Bacteria; Terrabacteria group; Actinobacteria; Actinomycetia; Corynebacteriales; Mycobacteriaceae; Mycobacterium; Mycobacterium tuberculosis complex; Mycobacterium tuberculosis;	NCBI: 1773	4395670	99.94	0.00	Yes
NCBI Assembly	GCA_001852735.1	NCBI: cellular organisms; Bacteria; Terrabacteria group; Actinobacteria; Actinomycetia; Corynebacteriales; Mycobacteriaceae; Mycobacterium; Mycobacterium tuberculosis complex; Mycobacterium tuberculosis;	NCBI: 1773	4379870	99.28	0.00	Yes
NCBI Assembly	GCA_001855445.1	NCBI: cellular organisms; Bacteria; Terrabacteria group; Actinobacteria; Actinomycetia; Corynebacteriales; Mycobacteriaceae; Mycobacterium; Mycobacterium avium complex (MAC); Mycobacterium avium; Mycobacterium avium subsp. paratuberculosis;	NCBI: 1770	4772864	99.28	0.33	Yes
NCBI Assembly	GCA_001668625.1	NCBI: cellular organisms; Bacteria; Terrabacteria group; Actinobacteria; Actinomycetia; Corynebacteriales; Mycobacteriaceae; Mycobacterium; unclassified Mycobacterium;	NCBI: 1834076	3760301	100.00	0.15	Yes
NCBI Assembly	GCA_001667315.1	NCBI: cellular organisms; Bacteria; Terrabacteria group; Actinobacteria; Actinomycetia; Corynebacteriales; Mycobacteriaceae; Mycobacterium; unclassified Mycobacterium;	NCBI: 1834083	5254564	99.90	0.33	Yes
NCBI Assembly	GCA_001852125.1	NCBI: cellular organisms; Bacteria; Terrabacteria group; Actinobacteria; Actinomycetia; Corynebacteriales; Mycobacteriaceae; Mycobacterium; Mycobacterium tuberculosis complex; Mycobacterium tuberculosis;	NCBI: 1773	4390204	99.94	0.00	Yes
NCBI Assembly	GCA_001722325.1	NCBI: cellular organisms; Bacteria; Terrabacteria group; Actinobacteria; Actinomycetia; Corynebacteriales; Mycobacteriaceae; Mycobacterium; Mycobacterium simiae complex; Mycobacterium sherrisii;	NCBI: 243061	5686820	99.94	1.59	Yes

NCBI Assembly	GCA_001844325.1	NCBI: cellular organisms; Bacteria; Terrabacteria group; Actinobacteria; Actinomycetia; Corynebacteriales; Mycobacteriaceae; Mycobacterium; Mycobacterium tuberculosis complex; Mycobacterium tuberculosis;	NCBI: 1773	4390166	99.94	0.00	Yes
NCBI Assembly	GCA_001846495.1	NCBI: cellular organisms; Bacteria; Terrabacteria group; Actinobacteria; Actinomycetia; Corynebacteriales; Mycobacteriaceae; Mycobacterium; Mycobacterium tuberculosis complex; Mycobacterium tuberculosis;	NCBI: 1773	4396195	99.94	0.00	Yes
NCBI Assembly	GCA_001844625.1	NCBI: cellular organisms; Bacteria; Terrabacteria group; Actinobacteria; Actinomycetia; Corynebacteriales; Mycobacteriaceae; Mycobacterium; Mycobacterium tuberculosis complex; Mycobacterium tuberculosis;	NCBI: 1773	4375949	99.94	0.00	Yes
NCBI Assembly	GCA_001849325.1	NCBI: cellular organisms; Bacteria; Terrabacteria group; Actinobacteria; Actinomycetia; Corynebacteriales; Mycobacteriaceae; Mycobacterium; Mycobacterium tuberculosis complex; Mycobacterium tuberculosis;	NCBI: 1773	4385822	99.94	0.00	Yes
NCBI Assembly	GCA_001847755.1	NCBI: cellular organisms; Bacteria; Terrabacteria group; Actinobacteria; Actinomycetia; Corynebacteriales; Mycobacteriaceae; Mycobacterium; Mycobacterium tuberculosis complex; Mycobacterium tuberculosis;	NCBI: 1773	4391471	99.94	0.00	Yes
NCBI Assembly	GCA_001855455.1	NCBI: cellular organisms; Bacteria; Terrabacteria group; Actinobacteria; Actinomycetia; Corynebacteriales; Mycobacteriaceae; Mycobacterium; Mycobacterium avium complex (MAC); Mycobacterium avium; Mycobacterium avium subsp. paratuberculosis;	NCBI: 1770	4774273	99.28	0.33	Yes
NCBI Assembly	GCA_001852755.1	NCBI: cellular organisms; Bacteria; Terrabacteria group; Actinobacteria; Actinomycetia; Corynebacteriales; Mycobacteriaceae; Mycobacterium; Mycobacterium tuberculosis complex; Mycobacterium tuberculosis;	NCBI: 1773	4352588	99.94	0.00	Yes
NCBI Assembly	GCA_001668675.1	NCBI: cellular organisms; Bacteria; Terrabacteria group; Actinobacteria; Actinomycetia; Corynebacteriales; Mycobacteriaceae; Mycobacterium; Mycobacterium asiaticum;	NCBI: 1790	6035124	99.94	1.57	Yes
NCBI Assembly	GCA_001667345.1	NCBI: cellular organisms; Bacteria; Terrabacteria group; Actinobacteria; Actinomycetia; Corynebacteriales; Mycobacteriaceae; Mycolicibacterium; Mycolicibacterium fortuitum;	NCBI: 1766	6665038	99.43	0.13	Yes
NCBI Assembly	GCA_001852145.1	NCBI: cellular organisms; Bacteria; Terrabacteria group; Actinobacteria; Actinomycetia; Corynebacteriales; Mycobacteriaceae; Mycobacterium; Mycobacterium tuberculosis complex; Mycobacterium tuberculosis;	NCBI: 1773	4395670	99.94	0.00	Yes
NCBI Assembly	GCA_001722335.1	NCBI: cellular organisms; Bacteria; Terrabacteria group; Actinobacteria; Actinomycetia; Corynebacteriales; Mycobacteriaceae; Mycolicibacterium; Mycolicibacterium flavescens;	NCBI: 1776	5973421	100.00	0.00	Yes
NCBI Assembly	GCA_001846505.1	NCBI: cellular organisms; Bacteria; Terrabacteria group; Actinobacteria; Actinomycetia; Corynebacteriales; Mycobacteriaceae; Mycobacterium; Mycobacterium tuberculosis complex; Mycobacterium tuberculosis;	NCBI: 1773	4398040	99.94	0.00	Yes
NCBI Assembly	GCA_001844345.1	NCBI: cellular organisms; Bacteria; Terrabacteria group; Actinobacteria; Actinomycetia; Corynebacteriales; Mycobacteriaceae; Mycobacterium; Mycobacterium tuberculosis complex; Mycobacterium tuberculosis;	NCBI: 1773	4382452	99.94	0.00	Yes
NCBI Assembly	GCA_001844655.1	NCBI: cellular organisms; Bacteria; Terrabacteria group; Actinobacteria; Actinomycetia; Corynebacteriales; Mycobacteriaceae; Mycobacterium; Mycobacterium tuberculosis complex; Mycobacterium tuberculosis;	NCBI: 1773	4388552	99.94	0.00	Yes
NCBI Assembly	GCA_001849345.1	NCBI: cellular organisms; Bacteria; Terrabacteria group; Actinobacteria; Actinomycetia; Corynebacteriales; Mycobacteriaceae; Mycobacterium; Mycobacterium tuberculosis complex; Mycobacterium tuberculosis;	NCBI: 1773	4382742	99.61	0.00	Yes
NCBI Assembly	GCA_001847775.1	NCBI: cellular organisms; Bacteria; Terrabacteria group; Actinobacteria; Actinomycetia; Corynebacteriales; Mycobacteriaceae; Mycobacterium; Mycobacterium tuberculosis complex; Mycobacterium tuberculosis;	NCBI: 1773	4431065	99.94	0.33	Yes
NCBI Assembly	GCA_001855785.1	NCBI: cellular organisms; Bacteria; Terrabacteria group; Actinobacteria; Actinomycetia; Corynebacteriales; Mycobacteriaceae; Mycobacterium; Mycobacterium tuberculosis complex; Mycobacterium tuberculosis;	NCBI: 1773	4402204	99.94	0.00	Yes
NCBI Assembly	GCA_001668695.1	NCBI: cellular organisms; Bacteria; Terrabacteria group; Actinobacteria; Actinomycetia; Corynebacteriales; Mycobacteriaceae; Mycobacterium; unclassified Mycobacterium;	NCBI: 1834135	5656620	99.78	2.40	Yes

NCBI Assembly	GCA_001852785.1	NCBI: cellular organisms; Bacteria; Terrabacteria group; Actinobacteria; Actinomycetia; Corynebacteriales; Mycobacteriaceae; Mycobacterium; Mycobacterium tuberculosis complex; Mycobacterium tuberculosis;	NCBI: 1773	4394770	99.78	0.00	Yes
NCBI Assembly	GCA_001667355.1	NCBI: cellular organisms; Bacteria; Terrabacteria group; Actinobacteria; Actinomycetia; Corynebacteriales; Mycobacteriaceae; Mycobacterium; Mycobacterium avium complex (MAC); Mycobacterium intracellulare;	NCBI: 1767	5660297	99.94	0.33	Yes
NCBI Assembly	GCA_001852165.1	NCBI: cellular organisms; Bacteria; Terrabacteria group; Actinobacteria; Actinomycetia; Corynebacteriales; Mycobacteriaceae; Mycobacterium; Mycobacterium tuberculosis complex; Mycobacterium tuberculosis;	NCBI: 1773	4384102	99.94	0.07	Yes
NCBI Assembly	GCA_001844355.1	NCBI: cellular organisms; Bacteria; Terrabacteria group; Actinobacteria; Actinomycetia; Corynebacteriales; Mycobacteriaceae; Mycobacterium; Mycobacterium tuberculosis complex; Mycobacterium tuberculosis;	NCBI: 1773	4396782	99.94	0.00	Yes
NCBI Assembly	GCA_001846545.1	NCBI: cellular organisms; Bacteria; Terrabacteria group; Actinobacteria; Actinomycetia; Corynebacteriales; Mycobacteriaceae; Mycobacterium; Mycobacterium tuberculosis complex; Mycobacterium tuberculosis;	NCBI: 1773	4387410	99.94	0.00	Yes
NCBI Assembly	GCA_001722345.1	NCBI: cellular organisms; Bacteria; Terrabacteria group; Actinobacteria; Actinomycetia; Corynebacteriales; Mycobacteriaceae; Mycobacterium; Mycobacterium simiae complex; Mycobacterium intermedium;	NCBI: 28445	6855640	99.94	1.63	Yes
NCBI Assembly	GCA_001844685.1	NCBI: cellular organisms; Bacteria; Terrabacteria group; Actinobacteria; Actinomycetia; Corynebacteriales; Mycobacteriaceae; Mycobacterium; Mycobacterium tuberculosis complex; Mycobacterium tuberculosis;	NCBI: 1773	4413872	99.94	0.00	Yes
NCBI Assembly	GCA_001849365.1	NCBI: cellular organisms; Bacteria; Terrabacteria group; Actinobacteria; Actinomycetia; Corynebacteriales; Mycobacteriaceae; Mycobacterium; Mycobacterium tuberculosis complex; Mycobacterium tuberculosis;	NCBI: 1773	4395582	99.94	0.00	Yes
NCBI Assembly	GCA_001847805.1	NCBI: cellular organisms; Bacteria; Terrabacteria group; Actinobacteria; Actinomycetia; Corynebacteriales; Mycobacteriaceae; Mycobacterium; Mycobacterium tuberculosis complex; Mycobacterium tuberculosis;	NCBI: 1773	4397633	99.94	0.00	Yes
NCBI Assembly	GCA_001855795.1	NCBI: cellular organisms; Bacteria; Terrabacteria group; Actinobacteria; Actinomycetia; Corynebacteriales; Mycobacteriaceae; Mycobacterium; Mycobacterium tuberculosis complex; Mycobacterium tuberculosis;	NCBI: 1773	4405149	99.94	0.00	Yes
NCBI Assembly	GCA_001668705.1	NCBI: cellular organisms; Bacteria; Terrabacteria group; Actinobacteria; Actinomycetia; Corynebacteriales; Mycobacteriaceae; Mycolicibacterium; Mycolicibacterium mucogenicum;	NCBI: 56689	6242733	99.50	0.48	Yes
NCBI Assembly	GCA_001852795.1	NCBI: cellular organisms; Bacteria; Terrabacteria group; Actinobacteria; Actinomycetia; Corynebacteriales; Mycobacteriaceae; Mycobacterium; Mycobacterium tuberculosis complex; Mycobacterium tuberculosis;	NCBI: 1773	4385208	99.94	0.00	Yes
NCBI Assembly	GCA_001667425.1	NCBI: cellular organisms; Bacteria; Terrabacteria group; Actinobacteria; Actinomycetia; Corynebacteriales; Mycobacteriaceae; Mycobacterium; unclassified Mycobacterium;	NCBI: 1834104	5397251	99.94	1.44	Yes
NCBI Assembly	GCA_001852175.1	NCBI: cellular organisms; Bacteria; Terrabacteria group; Actinobacteria; Actinomycetia; Corynebacteriales; Mycobacteriaceae; Mycobacterium; Mycobacterium tuberculosis complex; Mycobacterium tuberculosis;	NCBI: 1773	4385416	99.94	0.00	Yes
NCBI Assembly	GCA_001844385.1	NCBI: cellular organisms; Bacteria; Terrabacteria group; Actinobacteria; Actinomycetia; Corynebacteriales; Mycobacteriaceae; Mycobacterium; Mycobacterium tuberculosis complex; Mycobacterium tuberculosis;	NCBI: 1773	4407065	99.61	0.00	Yes
NCBI Assembly	GCA_001846555.1	NCBI: cellular organisms; Bacteria; Terrabacteria group; Actinobacteria; Actinomycetia; Corynebacteriales; Mycobacteriaceae; Mycobacterium; Mycobacterium tuberculosis complex; Mycobacterium tuberculosis;	NCBI: 1773	4345505	99.94	0.00	Yes
NCBI Assembly	GCA_001722355.1	NCBI: cellular organisms; Bacteria; Terrabacteria group; Actinobacteria; Actinomycetia; Corynebacteriales; Mycobacteriaceae; Mycolicibacterium; Mycolicibacterium holsaticum;	NCBI: 152142	5748336	96.70	1.15	Yes
NCBI Assembly	GCA_001844695.1	NCBI: cellular organisms; Bacteria; Terrabacteria group; Actinobacteria; Actinomycetia; Corynebacteriales; Mycobacteriaceae; Mycobacterium; Mycobacterium tuberculosis complex; Mycobacterium tuberculosis;	NCBI: 1773	4399045	99.94	0.00	Yes

NCBI Assembly	GCA_001849385.1	NCBI: cellular organisms; Bacteria; Terrabacteria group; Actinobacteria; Actinomycetia; Corynebacteriales; Mycobacteriaceae; Mycobacterium; Mycobacterium tuberculosis complex; Mycobacterium tuberculosis;	NCBI: 1773	4396017	99.61	0.00	Yes
NCBI Assembly	GCA_001855805.1	NCBI: cellular organisms; Bacteria; Terrabacteria group; Actinobacteria; Actinomycetia; Corynebacteriales; Mycobacteriaceae; Mycobacterium; Mycobacterium tuberculosis complex; Mycobacterium tuberculosis;	NCBI: 1773	4406256	99.94	0.00	Yes
NCBI Assembly	GCA_001847815.1	NCBI: cellular organisms; Bacteria; Terrabacteria group; Actinobacteria; Actinomycetia; Corynebacteriales; Mycobacteriaceae; Mycobacterium; Mycobacterium tuberculosis complex; Mycobacterium tuberculosis;	NCBI: 1773	4345874	99.94	0.00	Yes
NCBI Assembly	GCA_001668725.1	NCBI: cellular organisms; Bacteria; Terrabacteria group; Actinobacteria; Actinomycetia; Corynebacteriales; Mycobacteriaceae; Mycobacterium; unclassified Mycobacterium;	NCBI: 1834150	5724741	99.83	2.25	Yes
NCBI Assembly	GCA_001870145.1	NCBI: cellular organisms; Bacteria; Terrabacteria group; Actinobacteria; Actinomycetia; Corynebacteriales; Mycobacteriaceae; Mycobacterium; Mycobacterium tuberculosis complex; Mycobacterium tuberculosis;	NCBI: 1773	4425860	99.94	0.17	Yes
NCBI Assembly	GCA_001667445.1	NCBI: cellular organisms; Bacteria; Terrabacteria group; Actinobacteria; Actinomycetia; Corynebacteriales; Mycobacteriaceae; Mycolicibacterium; Mycolicibacterium fortuitum;	NCBI: 1766	6926361	99.81	0.13	Yes
NCBI Assembly	GCA_001852815.1	NCBI: cellular organisms; Bacteria; Terrabacteria group; Actinobacteria; Actinomycetia; Corynebacteriales; Mycobacteriaceae; Mycobacterium; Mycobacterium tuberculosis complex; Mycobacterium tuberculosis;	NCBI: 1773	4379952	99.94	0.00	Yes
NCBI Assembly	GCA_001722405.1	NCBI: cellular organisms; Bacteria; Terrabacteria group; Actinobacteria; Actinomycetia; Corynebacteriales; Mycobacteriaceae; Mycobacterium; Mycobacterium gordonae;	NCBI: 1778	7293532	98.69	2.37	Yes
NCBI Assembly	GCA_001852205.1	NCBI: cellular organisms; Bacteria; Terrabacteria group; Actinobacteria; Actinomycetia; Corynebacteriales; Mycobacteriaceae; Mycobacterium; Mycobacterium tuberculosis complex; Mycobacterium tuberculosis;	NCBI: 1773	4382411	99.94	0.00	Yes
NCBI Assembly	GCA_001844395.1	NCBI: cellular organisms; Bacteria; Terrabacteria group; Actinobacteria; Actinomycetia; Corynebacteriales; Mycobacteriaceae; Mycobacterium; Mycobacterium tuberculosis complex; Mycobacterium tuberculosis;	NCBI: 1773	4410415	99.94	0.00	Yes
NCBI Assembly	GCA_001846575.1	NCBI: cellular organisms; Bacteria; Terrabacteria group; Actinobacteria; Actinomycetia; Corynebacteriales; Mycobacteriaceae; Mycobacterium; Mycobacterium tuberculosis complex; Mycobacterium tuberculosis;	NCBI: 1773	4384772	99.94	0.00	Yes
NCBI Assembly	GCA_001844725.1	NCBI: cellular organisms; Bacteria; Terrabacteria group; Actinobacteria; Actinomycetia; Corynebacteriales; Mycobacteriaceae; Mycobacterium; Mycobacterium tuberculosis complex; Mycobacterium tuberculosis;	NCBI: 1773	4376048	99.94	0.00	Yes
NCBI Assembly	GCA_001849395.1	NCBI: cellular organisms; Bacteria; Terrabacteria group; Actinobacteria; Actinomycetia; Corynebacteriales; Mycobacteriaceae; Mycobacterium; Mycobacterium tuberculosis complex; Mycobacterium tuberculosis;	NCBI: 1773	4395250	99.61	0.00	Yes
NCBI Assembly	GCA_001847825.1	NCBI: cellular organisms; Bacteria; Terrabacteria group; Actinobacteria; Actinomycetia; Corynebacteriales; Mycobacteriaceae; Mycobacterium; Mycobacterium tuberculosis complex; Mycobacterium tuberculosis;	NCBI: 1773	4384022	99.94	0.00	Yes
NCBI Assembly	GCA_001667455.1	NCBI: cellular organisms; Bacteria; Terrabacteria group; Actinobacteria; Actinomycetia; Corynebacteriales; Mycobacteriaceae; Mycobacterium; unclassified Mycobacterium;	NCBI: 1856866	7870802	99.32	1.32	Yes
NCBI Assembly	GCA_001669335.1	NCBI: cellular organisms; Bacteria; Terrabacteria group; Actinobacteria; Actinomycetia; Corynebacteriales; Mycobacteriaceae; Mycobacterium; unclassified Mycobacterium;	NCBI: 1856860	6147599	99.78	3.98	Yes
NCBI Assembly	GCA_001855815.1	NCBI: cellular organisms; Bacteria; Terrabacteria group; Actinobacteria; Actinomycetia; Corynebacteriales; Mycobacteriaceae; Mycobacterium; Mycobacterium tuberculosis complex; Mycobacterium tuberculosis;	NCBI: 1773	4417938	99.94	0.00	Yes
NCBI Assembly	GCA_001870245.1	NCBI: cellular organisms; Bacteria; Terrabacteria group; Actinobacteria; Actinomycetia; Corynebacteriales; Mycobacteriaceae; Mycobacterium; Mycobacterium tuberculosis complex; Mycobacterium tuberculosis;	NCBI: 1773	4404380	99.78	0.00	Yes

NCBI Assembly	GCA_001852825.1	NCBI: cellular organisms; Bacteria; Terrabacteria group; Actinobacteria; Actinomycetia; Corynebacteriales; Mycobacteriaceae; Mycobacterium; Mycobacterium tuberculosis complex; Mycobacterium tuberculosis;	NCBI: 1773	4371890	99.94	0.00	Yes
NCBI Assembly	GCA_001844425.1	NCBI: cellular organisms; Bacteria; Terrabacteria group; Actinobacteria; Actinomycetia; Corynebacteriales; Mycobacteriaceae; Mycobacterium; Mycobacterium tuberculosis complex; Mycobacterium tuberculosis;	NCBI: 1773	4406919	99.94	0.00	Yes
NCBI Assembly	GCA_001852225.1	NCBI: cellular organisms; Bacteria; Terrabacteria group; Actinobacteria; Actinomycetia; Corynebacteriales; Mycobacteriaceae; Mycobacterium; Mycobacterium tuberculosis complex; Mycobacterium tuberculosis;	NCBI: 1773	4412046	99.94	0.00	Yes
NCBI Assembly	GCA_001722415.1	NCBI: cellular organisms; Bacteria; Terrabacteria group; Actinobacteria; Actinomycetia; Corynebacteriales; Mycobacteriaceae; Mycolicibacillus; Mycolicibacillus koreensis;	NCBI: 1069220	3873226	100.00	0.00	Yes
NCBI Assembly	GCA_001846595.1	NCBI: cellular organisms; Bacteria; Terrabacteria group; Actinobacteria; Actinomycetia; Corynebacteriales; Mycobacteriaceae; Mycobacterium; Mycobacterium tuberculosis complex; Mycobacterium tuberculosis;	NCBI: 1773	4391581	99.94	0.00	Yes
NCBI Assembly	GCA_001844735.1	NCBI: cellular organisms; Bacteria; Terrabacteria group; Actinobacteria; Actinomycetia; Corynebacteriales; Mycobacteriaceae; Mycobacterium; Mycobacterium tuberculosis complex; Mycobacterium tuberculosis;	NCBI: 1773	4405351	99.94	0.00	Yes
NCBI Assembly	GCA_001849425.1	NCBI: cellular organisms; Bacteria; Terrabacteria group; Actinobacteria; Actinomycetia; Corynebacteriales; Mycobacteriaceae; Mycobacterium; Mycobacterium tuberculosis complex; Mycobacterium tuberculosis;	NCBI: 1773	4371558	99.61	0.00	Yes
NCBI Assembly	GCA_001847835.1	NCBI: cellular organisms; Bacteria; Terrabacteria group; Actinobacteria; Actinomycetia; Corynebacteriales; Mycobacteriaceae; Mycobacterium; Mycobacterium tuberculosis complex; Mycobacterium tuberculosis;	NCBI: 1773	4400230	99.94	0.00	Yes
NCBI Assembly	GCA_001669345.1	NCBI: cellular organisms; Bacteria; Terrabacteria group; Actinobacteria; Actinomycetia; Corynebacteriales; Mycobacteriaceae; Mycobacterium; Mycobacterium asiaticum;	NCBI: 1790	6208484	99.94	0.49	Yes
NCBI Assembly	GCA_001667465.1	NCBI: cellular organisms; Bacteria; Terrabacteria group; Actinobacteria; Actinomycetia; Corynebacteriales; Mycobacteriaceae; Mycolicibacterium; Mycolicibacterium monacense;	NCBI: 85693	5661695	99.81	0.00	Yes
NCBI Assembly	GCA_001855865.1	NCBI: cellular organisms; Bacteria; Terrabacteria group; Actinobacteria; Actinomycetia; Corynebacteriales; Mycobacteriaceae; Mycobacterium; Mycobacterium tuberculosis complex; Mycobacterium tuberculosis;	NCBI: 1773	4408715	99.94	0.00	Yes
NCBI Assembly	GCA_001870585.1	NCBI: cellular organisms; Bacteria; Terrabacteria group; Actinobacteria; Actinomycetia; Corynebacteriales; Mycobacteriaceae; Mycobacterium; Mycobacterium ulcerans;	NCBI: 1809	5399224	98.38	0.18	Yes
NCBI Assembly	GCA_001852865.1	NCBI: cellular organisms; Bacteria; Terrabacteria group; Actinobacteria; Actinomycetia; Corynebacteriales; Mycobacteriaceae; Mycobacterium; Mycobacterium tuberculosis complex; Mycobacterium tuberculosis;	NCBI: 1773	4403296	99.94	0.00	Yes
NCBI Assembly	GCA_001844435.1	NCBI: cellular organisms; Bacteria; Terrabacteria group; Actinobacteria; Actinomycetia; Corynebacteriales; Mycobacteriaceae; Mycobacterium; Mycobacterium tuberculosis complex; Mycobacterium tuberculosis;	NCBI: 1773	4399291	99.94	0.00	Yes
NCBI Assembly	GCA_001722425.1	NCBI: cellular organisms; Bacteria; Terrabacteria group; Actinobacteria; Actinomycetia; Corynebacteriales; Mycobacteriaceae; Mycolicibacterium; Mycolicibacterium porcinum;	NCBI: 39693	6930603	98.02	1.60	Yes
NCBI Assembly	GCA_001852245.1	NCBI: cellular organisms; Bacteria; Terrabacteria group; Actinobacteria; Actinomycetia; Corynebacteriales; Mycobacteriaceae; Mycobacterium; Mycobacterium tuberculosis complex; Mycobacterium tuberculosis;	NCBI: 1773	4387388	99.94	0.00	Yes
NCBI Assembly	GCA_001846625.1	NCBI: cellular organisms; Bacteria; Terrabacteria group; Actinobacteria; Actinomycetia; Corynebacteriales; Mycobacteriaceae; Mycobacterium; Mycobacterium tuberculosis complex; Mycobacterium tuberculosis;	NCBI: 1773	4385423	99.94	0.00	Yes
NCBI Assembly	GCA_001844765.1	NCBI: cellular organisms; Bacteria; Terrabacteria group; Actinobacteria; Actinomycetia; Corynebacteriales; Mycobacteriaceae; Mycobacterium; Mycobacterium tuberculosis complex; Mycobacterium tuberculosis;	NCBI: 1773	4352375	99.94	0.00	Yes

NCBI Assembly	GCA_001849435.1	NCBI: cellular organisms; Bacteria; Terrabacteria group; Actinobacteria; Actinomycetia; Corynebacteriales; Mycobacteriaceae; Mycobacterium; Mycobacterium tuberculosis complex; Mycobacterium tuberculosis;	NCBI: 1773	4381615	99.94	0.00	Yes
NCBI Assembly	GCA_001667505.1	NCBI: cellular organisms; Bacteria; Terrabacteria group; Actinobacteria; Actinomycetia; Corynebacteriales; Mycobacteriaceae; Mycobacterium; unclassified Mycobacterium;	NCBI: 1834117	6799733	100.00	1.06	Yes
NCBI Assembly	GCA_001672575.1	NCBI: cellular organisms; Bacteria; Terrabacteria group; Actinobacteria; Actinomycetia; Corynebacteriales; Mycobacteriaceae; Mycobacterium; Mycobacterium scrofulaceum;	NCBI: 1783	5650911	99.22	3.89	Yes
NCBI Assembly	GCA_001847885.1	NCBI: cellular organisms; Bacteria; Terrabacteria group; Actinobacteria; Actinomycetia; Corynebacteriales; Mycobacteriaceae; Mycobacterium; Mycobacterium tuberculosis complex; Mycobacterium tuberculosis;	NCBI: 1773	4394473	99.94	0.00	Yes
NCBI Assembly	GCA_001855875.1	NCBI: cellular organisms; Bacteria; Terrabacteria group; Actinobacteria; Actinomycetia; Corynebacteriales; Mycobacteriaceae; Mycobacterium; Mycobacterium tuberculosis complex; Mycobacterium tuberculosis;	NCBI: 1773	4397371	99.94	0.00	Yes
NCBI Assembly	GCA_001870845.1	NCBI: cellular organisms; Bacteria; Terrabacteria group; Actinobacteria; Actinomycetia; Corynebacteriales; Mycobacteriaceae; Mycobacterium; Mycobacterium malmoense;	NCBI: 1780	5665471	99.94	3.59	Yes
NCBI Assembly	GCA_001852885.1	NCBI: cellular organisms; Bacteria; Terrabacteria group; Actinobacteria; Actinomycetia; Corynebacteriales; Mycobacteriaceae; Mycobacterium; Mycobacterium tuberculosis complex; Mycobacterium tuberculosis;	NCBI: 1773	4404885	99.94	0.00	Yes
NCBI Assembly	GCA_001844465.1	NCBI: cellular organisms; Bacteria; Terrabacteria group; Actinobacteria; Actinomycetia; Corynebacteriales; Mycobacteriaceae; Mycobacterium; Mycobacterium tuberculosis complex; Mycobacterium tuberculosis;	NCBI: 1773	4432497	99.94	0.00	Yes
NCBI Assembly	GCA_001722445.1	NCBI: cellular organisms; Bacteria; Terrabacteria group; Actinobacteria; Actinomycetia; Corynebacteriales; Mycobacteriaceae; Mycobacterium; Mycobacterium shimoidei;	NCBI: 29313	4720739	99.55	0.45	Yes
NCBI Assembly	GCA_001846635.1	NCBI: cellular organisms; Bacteria; Terrabacteria group; Actinobacteria; Actinomycetia; Corynebacteriales; Mycobacteriaceae; Mycobacterium; Mycobacterium tuberculosis complex; Mycobacterium tuberculosis;	NCBI: 1773	4404462	99.94	0.00	Yes
NCBI Assembly	GCA_001852265.1	NCBI: cellular organisms; Bacteria; Terrabacteria group; Actinobacteria; Actinomycetia; Corynebacteriales; Mycobacteriaceae; Mycobacterium; Mycobacterium tuberculosis complex; Mycobacterium tuberculosis;	NCBI: 1773	4390152	99.94	0.00	Yes
NCBI Assembly	GCA_001667525.1	NCBI: cellular organisms; Bacteria; Terrabacteria group; Actinobacteria; Actinomycetia; Corynebacteriales; Mycobacteriaceae; Mycobacterium; Mycobacterium simiae complex; Mycobacterium kubicae;	NCBI: 120959	5618076	99.61	0.22	Yes
NCBI Assembly	GCA_001849465.1	NCBI: cellular organisms; Bacteria; Terrabacteria group; Actinobacteria; Actinomycetia; Corynebacteriales; Mycobacteriaceae; Mycobacterium; Mycobacterium tuberculosis complex; Mycobacterium tuberculosis;	NCBI: 1773	4401839	99.94	0.00	Yes
NCBI Assembly	GCA_001844775.1	NCBI: cellular organisms; Bacteria; Terrabacteria group; Actinobacteria; Actinomycetia; Corynebacteriales; Mycobacteriaceae; Mycobacterium; Mycobacterium tuberculosis complex; Mycobacterium tuberculosis;	NCBI: 1773	4398786	99.94	0.00	Yes
NCBI Assembly	GCA_001672655.1	NCBI: cellular organisms; Bacteria; Terrabacteria group; Actinobacteria; Actinomycetia; Corynebacteriales; Mycobacteriaceae; Mycolicibacter; Mycolicibacter heraklionensis;	NCBI: 512402	4574942	100.00	0.61	Yes
NCBI Assembly	GCA_001847905.1	NCBI: cellular organisms; Bacteria; Terrabacteria group; Actinobacteria; Actinomycetia; Corynebacteriales; Mycobacteriaceae; Mycobacterium; Mycobacterium tuberculosis complex; Mycobacterium tuberculosis;	NCBI: 1773	4418126	99.94	0.00	Yes
NCBI Assembly	GCA_001855905.1	NCBI: cellular organisms; Bacteria; Terrabacteria group; Actinobacteria; Actinomycetia; Corynebacteriales; Mycobacteriaceae; Mycobacterium; Mycobacterium tuberculosis complex; Mycobacterium tuberculosis;	NCBI: 1773	4344797	99.94	0.00	Yes
NCBI Assembly	GCA_001852895.1	NCBI: cellular organisms; Bacteria; Terrabacteria group; Actinobacteria; Actinomycetia; Corynebacteriales; Mycobacteriaceae; Mycobacterium; Mycobacterium tuberculosis complex; Mycobacterium tuberculosis;	NCBI: 1773	4396568	99.94	0.00	Yes

NCBI Assembly	GCA_001844475.1	NCBI: cellular organisms; Bacteria; Terrabacteria group; Actinobacteria; Actinomycetia; Corynebacteriales; Mycobacteriaceae; Mycobacterium; Mycobacterium tuberculosis complex; Mycobacterium tuberculosis;	NCBI: 1773	4403712	99.94	0.00	Yes
NCBI Assembly	GCA_001726085.1	NCBI: cellular organisms; Bacteria; Terrabacteria group; Actinobacteria; Actinomycetia; Corynebacteriales; Mycobacteriaceae; Mycobacterium; Mycobacterium tuberculosis complex; Mycobacterium tuberculosis; Mycobacterium tuberculosis variant bovis;	NCBI: 1765	4245279	98.11	0.17	Yes
NCBI Assembly	GCA_001846645.1	NCBI: cellular organisms; Bacteria; Terrabacteria group; Actinobacteria; Actinomycetia; Corynebacteriales; Mycobacteriaceae; Mycobacterium; Mycobacterium tuberculosis complex; Mycobacterium tuberculosis;	NCBI: 1773	4376226	99.94	0.00	Yes
NCBI Assembly	GCA_001852275.1	NCBI: cellular organisms; Bacteria; Terrabacteria group; Actinobacteria; Actinomycetia; Corynebacteriales; Mycobacteriaceae; Mycobacterium; Mycobacterium tuberculosis complex; Mycobacterium tuberculosis;	NCBI: 1773	4395321	99.94	0.00	Yes
NCBI Assembly	GCA_001672665.1	NCBI: cellular organisms; Bacteria; Terrabacteria group; Actinobacteria; Actinomycetia; Corynebacteriales; Mycobacteriaceae; Mycobacterium; unclassified Mycobacterium;	NCBI: 1834131	5382236	99.56	1.92	Yes
NCBI Assembly	GCA_001667535.1	NCBI: cellular organisms; Bacteria; Terrabacteria group; Actinobacteria; Actinomycetia; Corynebacteriales; Mycobacteriaceae; Mycolicibacterium; Mycolicibacterium elephantis;	NCBI: 81858	5223216	100.00	1.59	Yes
NCBI Assembly	GCA_001844805.1	NCBI: cellular organisms; Bacteria; Terrabacteria group; Actinobacteria; Actinomycetia; Corynebacteriales; Mycobacteriaceae; Mycobacterium; Mycobacterium tuberculosis complex; Mycobacterium tuberculosis;	NCBI: 1773	4400261	99.94	0.00	Yes
NCBI Assembly	GCA_001849485.1	NCBI: cellular organisms; Bacteria; Terrabacteria group; Actinobacteria; Actinomycetia; Corynebacteriales; Mycobacteriaceae; Mycobacterium; Mycobacterium tuberculosis complex; Mycobacterium tuberculosis;	NCBI: 1773	4398393	99.61	0.00	Yes
NCBI Assembly	GCA_001847915.1	NCBI: cellular organisms; Bacteria; Terrabacteria group; Actinobacteria; Actinomycetia; Corynebacteriales; Mycobacteriaceae; Mycobacterium; Mycobacterium tuberculosis complex; Mycobacterium tuberculosis;	NCBI: 1773	4406906	99.94	0.00	Yes
NCBI Assembly	GCA_001855915.1	NCBI: cellular organisms; Bacteria; Terrabacteria group; Actinobacteria; Actinomycetia; Corynebacteriales; Mycobacteriaceae; Mycobacterium; Mycobacterium tuberculosis complex; Mycobacterium tuberculosis;	NCBI: 1773	4374974	99.94	0.00	Yes
NCBI Assembly	GCA_001886515.1	NCBI: cellular organisms; Bacteria; Terrabacteria group; Actinobacteria; Actinomycetia; Corynebacteriales; Mycobacteriaceae; Mycobacterium; unclassified Mycobacterium;	NCBI: 1920667	6041408	100.00	0.15	Yes
NCBI Assembly	GCA_001852925.1	NCBI: cellular organisms; Bacteria; Terrabacteria group; Actinobacteria; Actinomycetia; Corynebacteriales; Mycobacteriaceae; Mycobacterium; Mycobacterium tuberculosis complex; Mycobacterium tuberculosis;	NCBI: 1773	4412091	99.94	0.00	Yes
NCBI Assembly	GCA_001844505.1	NCBI: cellular organisms; Bacteria; Terrabacteria group; Actinobacteria; Actinomycetia; Corynebacteriales; Mycobacteriaceae; Mycobacterium; Mycobacterium tuberculosis complex; Mycobacterium tuberculosis;	NCBI: 1773	4394203	99.94	0.00	Yes
NCBI Assembly	GCA_001750065.2	NCBI: cellular organisms; Bacteria; Terrabacteria group; Actinobacteria; Actinomycetia; Corynebacteriales; Mycobacteriaceae; Mycobacterium; Mycobacterium avium complex (MAC); Mycobacterium intracellulare; Mycobacterium intracellulare subsp. chimaera;	NCBI: 222805	6330036	99.88	2.26	Yes
NCBI Assembly	GCA_001672675.1	NCBI: cellular organisms; Bacteria; Terrabacteria group; Actinobacteria; Actinomycetia; Corynebacteriales; Mycobacteriaceae; Mycobacterium; unclassified Mycobacterium;	NCBI: 1834132	6221573	99.94	3.14	Yes
NCBI Assembly	GCA_001666875.1	NCBI: cellular organisms; Bacteria; Terrabacteria group; Actinobacteria; Actinomycetia; Corynebacteriales; Mycobacteriaceae; Mycobacterium; unclassified Mycobacterium;	NCBI: 1834152	5913732	99.69	0.05	Yes
NCBI Assembly	GCA_001846685.1	NCBI: cellular organisms; Bacteria; Terrabacteria group; Actinobacteria; Actinomycetia; Corynebacteriales; Mycobacteriaceae; Mycobacterium; Mycobacterium tuberculosis complex; Mycobacterium tuberculosis;	NCBI: 1773	4407589	99.94	0.00	Yes
NCBI Assembly	GCA_001852305.1	NCBI: cellular organisms; Bacteria; Terrabacteria group; Actinobacteria; Actinomycetia; Corynebacteriales; Mycobacteriaceae; Mycobacterium; Mycobacterium tuberculosis complex; Mycobacterium tuberculosis;	NCBI: 1773	4376936	99.94	0.00	Yes

NCBI Assembly	GCA_001844825.1	NCBI: cellular organisms; Bacteria; Terrabacteria group; Actinobacteria; Actinomycetia; Corynebacteriales; Mycobacteriaceae; Mycobacterium; Mycobacterium tuberculosis complex; Mycobacterium tuberculosis;	NCBI: 1773	4388100	99.94	0.00	Yes
NCBI Assembly	GCA_001849495.1	NCBI: cellular organisms; Bacteria; Terrabacteria group; Actinobacteria; Actinomycetia; Corynebacteriales; Mycobacteriaceae; Mycobacterium; Mycobacterium tuberculosis complex; Mycobacterium tuberculosis;	NCBI: 1773	4398658	99.94	0.00	Yes
NCBI Assembly	GCA_001847925.1	NCBI: cellular organisms; Bacteria; Terrabacteria group; Actinobacteria; Actinomycetia; Corynebacteriales; Mycobacteriaceae; Mycobacterium; Mycobacterium tuberculosis complex; Mycobacterium tuberculosis;	NCBI: 1773	4385474	99.94	0.00	Yes
NCBI Assembly	GCA_001855945.1	NCBI: cellular organisms; Bacteria; Terrabacteria group; Actinobacteria; Actinomycetia; Corynebacteriales; Mycobacteriaceae; Mycobacterium; Mycobacterium tuberculosis complex; Mycobacterium tuberculosis;	NCBI: 1773	4395702	99.94	0.00	Yes
NCBI Assembly	GCA_001889665.1	NCBI: cellular organisms; Bacteria; Terrabacteria group; Actinobacteria; Actinomycetia; Corynebacteriales; Mycobacteriaceae; Mycobacterium; Mycobacterium tuberculosis complex; Mycobacterium tuberculosis;	NCBI: 1773	4387523	99.94	0.00	Yes
NCBI Assembly	GCA_001852935.1	NCBI: cellular organisms; Bacteria; Terrabacteria group; Actinobacteria; Actinomycetia; Corynebacteriales; Mycobacteriaceae; Mycobacterium; Mycobacterium tuberculosis complex; Mycobacterium tuberculosis;	NCBI: 1773	4392093	99.94	0.00	Yes
NCBI Assembly	GCA_001750865.1	NCBI: cellular organisms; Bacteria; Terrabacteria group; Actinobacteria; Actinomycetia; Corynebacteriales; Mycobacteriaceae; Mycobacterium; Mycobacterium tuberculosis complex; Mycobacterium tuberculosis;	NCBI: 1773	4378588	99.94	0.00	Yes
NCBI Assembly	GCA_001667585.1	NCBI: cellular organisms; Bacteria; Terrabacteria group; Actinobacteria; Actinomycetia; Corynebacteriales; Mycobacteriaceae; Mycobacterium; unclassified Mycobacterium;	NCBI: 1834138	5708645	99.94	0.92	Yes
NCBI Assembly	GCA_001844525.1	NCBI: cellular organisms; Bacteria; Terrabacteria group; Actinobacteria; Actinomycetia; Corynebacteriales; Mycobacteriaceae; Mycobacterium; Mycobacterium tuberculosis complex; Mycobacterium tuberculosis;	NCBI: 1773	4382455	99.94	0.00	Yes
NCBI Assembly	GCA_001666895.1	NCBI: cellular organisms; Bacteria; Terrabacteria group; Actinobacteria; Actinomycetia; Corynebacteriales; Mycobacteriaceae; Mycobacterium; unclassified Mycobacterium;	NCBI: 1856865	5931488	99.88	6.98	No
NCBI Assembly	GCA_001846705.1	NCBI: cellular organisms; Bacteria; Terrabacteria group; Actinobacteria; Actinomycetia; Corynebacteriales; Mycobacteriaceae; Mycobacterium; Mycobacterium tuberculosis complex; Mycobacterium tuberculosis;	NCBI: 1773	4385650	99.94	0.00	Yes
NCBI Assembly	GCA_001844845.1	NCBI: cellular organisms; Bacteria; Terrabacteria group; Actinobacteria; Actinomycetia; Corynebacteriales; Mycobacteriaceae; Mycobacterium; Mycobacterium tuberculosis complex; Mycobacterium tuberculosis;	NCBI: 1773	4405710	99.94	0.00	Yes
NCBI Assembly	GCA_001852325.1	NCBI: cellular organisms; Bacteria; Terrabacteria group; Actinobacteria; Actinomycetia; Corynebacteriales; Mycobacteriaceae; Mycobacterium; Mycobacterium tuberculosis complex; Mycobacterium tuberculosis;	NCBI: 1773	4380976	99.94	0.00	Yes
NCBI Assembly	GCA_001849525.1	NCBI: cellular organisms; Bacteria; Terrabacteria group; Actinobacteria; Actinomycetia; Corynebacteriales; Mycobacteriaceae; Mycobacterium; Mycobacterium tuberculosis complex; Mycobacterium tuberculosis;	NCBI: 1773	4392412	99.94	0.00	Yes
NCBI Assembly	GCA_001847965.1	NCBI: cellular organisms; Bacteria; Terrabacteria group; Actinobacteria; Actinomycetia; Corynebacteriales; Mycobacteriaceae; Mycobacterium; Mycobacterium tuberculosis complex; Mycobacterium tuberculosis;	NCBI: 1773	4373785	99.61	0.00	Yes
NCBI Assembly	GCA_001855955.1	NCBI: cellular organisms; Bacteria; Terrabacteria group; Actinobacteria; Actinomycetia; Corynebacteriales; Mycobacteriaceae; Mycobacterium; Mycobacterium tuberculosis complex; Mycobacterium tuberculosis;	NCBI: 1773	4398895	99.94	0.00	Yes
NCBI Assembly	GCA_001889675.1	NCBI: cellular organisms; Bacteria; Terrabacteria group; Actinobacteria; Actinomycetia; Corynebacteriales; Mycobacteriaceae; Mycobacterium; Mycobacterium tuberculosis complex; Mycobacterium tuberculosis;	NCBI: 1773	4369097	99.61	0.00	Yes
NCBI Assembly	GCA_001852955.1	NCBI: cellular organisms; Bacteria; Terrabacteria group; Actinobacteria; Actinomycetia; Corynebacteriales; Mycobacteriaceae; Mycobacterium; Mycobacterium tuberculosis complex; Mycobacterium tuberculosis;	NCBI: 1773	4400330	99.94	0.00	Yes

NCBI Assembly	GCA_001667595.1	NCBI: cellular organisms; Bacteria; Terrabacteria group; Actinobacteria; Actinomycetia; Corynebacteriales; Mycobacteriaceae; Mycobacterium; unclassified Mycobacterium;	NCBI: 1834140	5276788	99.94	0.67	Yes
NCBI Assembly	GCA_001844535.1	NCBI: cellular organisms; Bacteria; Terrabacteria group; Actinobacteria; Actinomycetia; Corynebacteriales; Mycobacteriaceae; Mycobacterium; Mycobacterium tuberculosis complex; Mycobacterium tuberculosis;	NCBI: 1773	4381525	99.94	0.00	Yes
NCBI Assembly	GCA_001756795.1	NCBI: cellular organisms; Bacteria; Terrabacteria group; Actinobacteria; Actinomycetia; Corynebacteriales; Mycobacteriaceae; Mycolicibacterium; unclassified Mycolicibacterium;	NCBI: 1866905	5819532	91.95	0.00	Yes
NCBI Assembly	GCA_001666915.1	NCBI: cellular organisms; Bacteria; Terrabacteria group; Actinobacteria; Actinomycetia; Corynebacteriales; Mycobacteriaceae; Mycobacterium; unclassified Mycobacterium;	NCBI: 1834146	5307282	99.61	1.79	Yes
NCBI Assembly	GCA_001846725.1	NCBI: cellular organisms; Bacteria; Terrabacteria group; Actinobacteria; Actinomycetia; Corynebacteriales; Mycobacteriaceae; Mycobacterium; Mycobacterium tuberculosis complex; Mycobacterium tuberculosis;	NCBI: 1773	4400655	99.94	0.00	Yes
NCBI Assembly	GCA_001844855.1	NCBI: cellular organisms; Bacteria; Terrabacteria group; Actinobacteria; Actinomycetia; Corynebacteriales; Mycobacteriaceae; Mycobacterium; Mycobacterium tuberculosis complex; Mycobacterium tuberculosis;	NCBI: 1773	4406396	99.94	0.00	Yes
NCBI Assembly	GCA_001852335.1	NCBI: cellular organisms; Bacteria; Terrabacteria group; Actinobacteria; Actinomycetia; Corynebacteriales; Mycobacteriaceae; Mycobacterium; Mycobacterium tuberculosis complex; Mycobacterium tuberculosis;	NCBI: 1773	4354796	99.94	0.00	Yes
NCBI Assembly	GCA_001849535.1	NCBI: cellular organisms; Bacteria; Terrabacteria group; Actinobacteria; Actinomycetia; Corynebacteriales; Mycobacteriaceae; Mycobacterium; Mycobacterium tuberculosis complex; Mycobacterium tuberculosis;	NCBI: 1773	4396155	99.94	0.00	Yes
NCBI Assembly	GCA_001847985.1	NCBI: cellular organisms; Bacteria; Terrabacteria group; Actinobacteria; Actinomycetia; Corynebacteriales; Mycobacteriaceae; Mycobacterium; Mycobacterium tuberculosis complex; Mycobacterium tuberculosis;	NCBI: 1773	4392069	99.94	0.00	Yes
NCBI Assembly	GCA_001855975.1	NCBI: cellular organisms; Bacteria; Terrabacteria group; Actinobacteria; Actinomycetia; Corynebacteriales; Mycobacteriaceae; Mycobacterium; Mycobacterium tuberculosis complex; Mycobacterium tuberculosis;	NCBI: 1773	4404074	99.94	0.00	Yes
NCBI Assembly	GCA_001889725.1	NCBI: cellular organisms; Bacteria; Terrabacteria group; Actinobacteria; Actinomycetia; Corynebacteriales; Mycobacteriaceae; Mycobacterium; Mycobacterium tuberculosis complex; Mycobacterium tuberculosis;	NCBI: 1773	4384738	99.61	0.00	Yes
NCBI Assembly	GCA_001666935.1	NCBI: cellular organisms; Bacteria; Terrabacteria group; Actinobacteria; Actinomycetia; Corynebacteriales; Mycobacteriaceae; Mycobacterium; unclassified Mycobacterium;	NCBI: 1834130	5681106	99.78	1.22	Yes
NCBI Assembly	GCA_001852985.1	NCBI: cellular organisms; Bacteria; Terrabacteria group; Actinobacteria; Actinomycetia; Corynebacteriales; Mycobacteriaceae; Mycobacterium; Mycobacterium tuberculosis complex; Mycobacterium tuberculosis;	NCBI: 1773	4394312	99.94	0.00	Yes
NCBI Assembly	GCA_001667625.1	NCBI: cellular organisms; Bacteria; Terrabacteria group; Actinobacteria; Actinomycetia; Corynebacteriales; Mycobacteriaceae; Mycobacterium; unclassified Mycobacterium;	NCBI: 1856863	5640979	99.28	3.28	Yes
NCBI Assembly	GCA_001757405.1	NCBI: cellular organisms; Bacteria; Terrabacteria group; Actinobacteria; Actinomycetia; Corynebacteriales; Mycobacteriaceae; Mycobacterium; Mycobacterium tuberculosis complex; Mycobacterium tuberculosis;	NCBI: 1773	4319495	99.78	0.00	Yes
NCBI Assembly	GCA_001895845.1	NCBI: cellular organisms; Bacteria; Terrabacteria group; Actinobacteria; Actinomycetia; Corynebacteriales; Mycobacteriaceae; Mycobacterium; Mycobacterium tuberculosis complex; Mycobacterium tuberculosis;	NCBI: 1773	4385578	99.94	0.00	Yes
NCBI Assembly	GCA_001846115.1	NCBI: cellular organisms; Bacteria; Terrabacteria group; Actinobacteria; Actinomycetia; Corynebacteriales; Mycobacteriaceae; Mycobacterium; Mycobacterium tuberculosis complex; Mycobacterium tuberculosis;	NCBI: 1773	4355867	99.94	0.00	Yes
NCBI Assembly	GCA_001852365.1	NCBI: cellular organisms; Bacteria; Terrabacteria group; Actinobacteria; Actinomycetia; Corynebacteriales; Mycobacteriaceae; Mycobacterium; Mycobacterium tuberculosis complex; Mycobacterium tuberculosis;	NCBI: 1773	4399277	99.61	0.00	Yes

NCBI Assembly	GCA_001847385.1	NCBI: cellular organisms; Bacteria; Terrabacteria group; Actinobacteria; Actinomycetia; Corynebacteriales; Mycobacteriaceae; Mycobacterium; Mycobacterium tuberculosis complex; Mycobacterium tuberculosis;	NCBI: 1773	4376355	99.94	0.00	Yes
NCBI Assembly	GCA_001853805.1	NCBI: cellular organisms; Bacteria; Terrabacteria group; Actinobacteria; Actinomycetia; Corynebacteriales; Mycobacteriaceae; Mycobacteroides; Mycobacteroides chelonae;	NCBI: 1774	5357569	99.44	0.47	Yes
NCBI Assembly	GCA_001849565.1	NCBI: cellular organisms; Bacteria; Terrabacteria group; Actinobacteria; Actinomycetia; Corynebacteriales; Mycobacteriaceae; Mycobacterium; Mycobacterium tuberculosis complex; Mycobacterium tuberculosis;	NCBI: 1773	4398220	99.94	0.00	Yes
NCBI Assembly	GCA_001610465.1	NCBI: cellular organisms; Bacteria; Terrabacteria group; Actinobacteria; Actinomycetia; Corynebacteriales; Mycobacteriaceae; Mycobacterium; Mycobacterium avium complex (MAC); Mycobacterium avium; Mycobacterium avium subsp. hominissuis;	NCBI: 439334	5221056	98.89	2.19	Yes
NCBI Assembly	GCA_001895765.1	NCBI: cellular organisms; Bacteria; Terrabacteria group; Actinobacteria; Actinomycetia; Corynebacteriales; Mycobacteriaceae; Mycobacterium; Mycobacterium tuberculosis complex; Mycobacterium tuberculosis;	NCBI: 1773	4386132	99.94	0.00	Yes
NCBI Assembly	GCA_001666955.1	NCBI: cellular organisms; Bacteria; Terrabacteria group; Actinobacteria; Actinomycetia; Corynebacteriales; Mycobacteriaceae; Mycobacterium; unclassified Mycobacterium;	NCBI: 1834148	5661694	99.64	1.73	Yes
NCBI Assembly	GCA_001852375.1	NCBI: cellular organisms; Bacteria; Terrabacteria group; Actinobacteria; Actinomycetia; Corynebacteriales; Mycobacteriaceae; Mycobacterium; Mycobacterium tuberculosis complex; Mycobacterium tuberculosis;	NCBI: 1773	4348877	99.94	0.00	Yes
NCBI Assembly	GCA_001667655.1	NCBI: cellular organisms; Bacteria; Terrabacteria group; Actinobacteria; Actinomycetia; Corynebacteriales; Mycobacteriaceae; Mycobacterium; Mycobacterium avium complex (MAC); Mycobacterium intracellulare;	NCBI: 1767	5351573	99.90	1.03	Yes
NCBI Assembly	GCA_001757445.1	NCBI: cellular organisms; Bacteria; Terrabacteria group; Actinobacteria; Actinomycetia; Corynebacteriales; Mycobacteriaceae; Mycobacterium; Mycobacterium tuberculosis complex; Mycobacterium tuberculosis;	NCBI: 1773	4389402	99.61	0.00	Yes
NCBI Assembly	GCA_001895865.1	NCBI: cellular organisms; Bacteria; Terrabacteria group; Actinobacteria; Actinomycetia; Corynebacteriales; Mycobacteriaceae; Mycobacterium; Mycobacterium tuberculosis complex; Mycobacterium tuberculosis;	NCBI: 1773	4386447	99.94	0.00	Yes
NCBI Assembly	GCA_001846145.1	NCBI: cellular organisms; Bacteria; Terrabacteria group; Actinobacteria; Actinomycetia; Corynebacteriales; Mycobacteriaceae; Mycobacterium; Mycobacterium tuberculosis complex; Mycobacterium tuberculosis;	NCBI: 1773	4396858	99.94	0.00	Yes
NCBI Assembly	GCA_001851725.1	NCBI: cellular organisms; Bacteria; Terrabacteria group; Actinobacteria; Actinomycetia; Corynebacteriales; Mycobacteriaceae; Mycobacterium; Mycobacterium tuberculosis complex; Mycobacterium tuberculosis;	NCBI: 1773	4391458	99.94	0.00	Yes
NCBI Assembly	GCA_001847405.1	NCBI: cellular organisms; Bacteria; Terrabacteria group; Actinobacteria; Actinomycetia; Corynebacteriales; Mycobacteriaceae; Mycobacterium; Mycobacterium tuberculosis complex; Mycobacterium tuberculosis;	NCBI: 1773	4387971	99.94	0.00	Yes
NCBI Assembly	GCA_001853845.1	NCBI: cellular organisms; Bacteria; Terrabacteria group; Actinobacteria; Actinomycetia; Corynebacteriales; Mycobacteriaceae; Mycobacteroides; Mycobacteroides chelonae;	NCBI: 1774	4887531	99.50	0.33	Yes
NCBI Assembly	GCA_001849575.1	NCBI: cellular organisms; Bacteria; Terrabacteria group; Actinobacteria; Actinomycetia; Corynebacteriales; Mycobacteriaceae; Mycobacterium; Mycobacterium tuberculosis complex; Mycobacterium tuberculosis;	NCBI: 1773	4384807	99.94	0.00	Yes
NCBI Assembly	GCA_001610475.1	NCBI: cellular organisms; Bacteria; Terrabacteria group; Actinobacteria; Actinomycetia; Corynebacteriales; Mycobacteriaceae; Mycobacterium; Mycobacterium avium complex (MAC); Mycobacterium avium; Mycobacterium avium subsp. hominissuis;	NCBI: 439334	5934301	99.60	7.36	No
NCBI Assembly	GCA_001666985.1	NCBI: cellular organisms; Bacteria; Terrabacteria group; Actinobacteria; Actinomycetia; Corynebacteriales; Mycobacteriaceae; Mycolicibacterium; Mycolicibacterium fortuitum;	NCBI: 1766	6181898	99.81	0.88	Yes
NCBI Assembly	GCA_001895785.1	NCBI: cellular organisms; Bacteria; Terrabacteria group; Actinobacteria; Actinomycetia; Corynebacteriales; Mycobacteriaceae; Mycobacterium; Mycobacterium tuberculosis complex; Mycobacterium tuberculosis;	NCBI: 1773	4359825	99.94	0.00	Yes

NCBI Assembly	GCA_001667665.1	NCBI: cellular organisms; Bacteria; Terrabacteria group; Actinobacteria; Actinomycetia; Corynebacteriales; Mycobacteriaceae; Mycobacterium; unclassified Mycobacterium;	NCBI: 1834147	5842549	99.94	0.97	Yes
NCBI Assembly	GCA_001852405.1	NCBI: cellular organisms; Bacteria; Terrabacteria group; Actinobacteria; Actinomycetia; Corynebacteriales; Mycobacteriaceae; Mycobacterium; Mycobacterium tuberculosis complex; Mycobacterium tuberculosis;	NCBI: 1773	4379391	99.61	0.00	Yes
NCBI Assembly	GCA_001766635.1	NCBI: cellular organisms; Bacteria; Terrabacteria group; Actinobacteria; Actinomycetia; Corynebacteriales; Mycobacteriaceae; Mycobacterium; Mycobacterium grossiae;	NCBI: 1552759	6145628	99.89	0.94	Yes
NCBI Assembly	GCA_001901205.1	NCBI: cellular organisms; Bacteria; Terrabacteria group; Actinobacteria; Actinomycetia; Corynebacteriales; Mycobacteriaceae; Mycobacterium; Mycobacterium avium complex (MAC); Mycobacterium intracellulare; Mycobacterium intracellulare subsp. chimaera;	NCBI: 222805	6028403	99.94	1.15	Yes
NCBI Assembly	GCA_001846155.1	NCBI: cellular organisms; Bacteria; Terrabacteria group; Actinobacteria; Actinomycetia; Corynebacteriales; Mycobacteriaceae; Mycobacterium; Mycobacterium tuberculosis complex; Mycobacterium tuberculosis;	NCBI: 1773	4366347	99.94	0.00	Yes
NCBI Assembly	GCA_001851765.1	NCBI: cellular organisms; Bacteria; Terrabacteria group; Actinobacteria; Actinomycetia; Corynebacteriales; Mycobacteriaceae; Mycobacterium; Mycobacterium tuberculosis complex; Mycobacterium tuberculosis;	NCBI: 1773	4403833	99.61	0.00	Yes
NCBI Assembly	GCA_001847415.1	NCBI: cellular organisms; Bacteria; Terrabacteria group; Actinobacteria; Actinomycetia; Corynebacteriales; Mycobacteriaceae; Mycobacterium; Mycobacterium tuberculosis complex; Mycobacterium tuberculosis;	NCBI: 1773	4396913	99.94	0.00	Yes
NCBI Assembly	GCA_001849605.1	NCBI: cellular organisms; Bacteria; Terrabacteria group; Actinobacteria; Actinomycetia; Corynebacteriales; Mycobacteriaceae; Mycobacterium; Mycobacterium tuberculosis complex; Mycobacterium tuberculosis;	NCBI: 1773	4396236	99.94	0.00	Yes
NCBI Assembly	GCA_001853865.1	NCBI: cellular organisms; Bacteria; Terrabacteria group; Actinobacteria; Actinomycetia; Corynebacteriales; Mycobacteriaceae; Mycobacteroides; Mycobacteroides chelonae;	NCBI: 1774	4938961	99.39	0.00	Yes
NCBI Assembly	GCA_001666995.1	NCBI: cellular organisms; Bacteria; Terrabacteria group; Actinobacteria; Actinomycetia; Corynebacteriales; Mycobacteriaceae; Mycolicibacter; Mycolicibacter heraklionensis;	NCBI: 512402	4860770	100.00	1.29	Yes
NCBI Assembly	GCA_001610515.1	NCBI: cellular organisms; Bacteria; Terrabacteria group; Actinobacteria; Actinomycetia; Corynebacteriales; Mycobacteriaceae; Mycobacterium; Mycobacterium avium complex (MAC); Mycobacterium avium; Mycobacterium avium subsp. hominissuis;	NCBI: 439334	5479015	96.94	4.58	Yes
NCBI Assembly	GCA_001667695.1	NCBI: cellular organisms; Bacteria; Terrabacteria group; Actinobacteria; Actinomycetia; Corynebacteriales; Mycobacteriaceae; Mycobacterium; Mycobacterium avium complex (MAC); Mycobacterium mantenii;	NCBI: 560555	5836558	99.78	1.16	Yes
NCBI Assembly	GCA_001895805.1	NCBI: cellular organisms; Bacteria; Terrabacteria group; Actinobacteria; Actinomycetia; Corynebacteriales; Mycobacteriaceae; Mycobacterium; Mycobacterium tuberculosis complex; Mycobacterium tuberculosis;	NCBI: 1773	4365724	99.94	0.00	Yes
NCBI Assembly	GCA_001852425.1	NCBI: cellular organisms; Bacteria; Terrabacteria group; Actinobacteria; Actinomycetia; Corynebacteriales; Mycobacteriaceae; Mycobacterium; Mycobacterium tuberculosis complex; Mycobacterium tuberculosis;	NCBI: 1773	4383332	99.94	0.00	Yes
NCBI Assembly	GCA_001904575.1	NCBI: cellular organisms; Bacteria; Terrabacteria group; Actinobacteria; Actinomycetia; Corynebacteriales; Mycobacteriaceae; Mycobacterium; Mycobacterium avium complex (MAC); Mycobacterium avium; Mycobacterium avium subsp. paratuberculosis;	NCBI: 1770	4750273	99.61	0.50	Yes
NCBI Assembly	GCA_001846185.1	NCBI: cellular organisms; Bacteria; Terrabacteria group; Actinobacteria; Actinomycetia; Corynebacteriales; Mycobacteriaceae; Mycobacterium; Mycobacterium tuberculosis complex; Mycobacterium tuberculosis;	NCBI: 1773	4385152	99.94	0.00	Yes
NCBI Assembly	GCA_001851785.1	NCBI: cellular organisms; Bacteria; Terrabacteria group; Actinobacteria; Actinomycetia; Corynebacteriales; Mycobacteriaceae; Mycobacterium; Mycobacterium tuberculosis complex; Mycobacterium tuberculosis;	NCBI: 1773	4385183	99.61	0.07	Yes
NCBI Assembly	GCA_001667015.1	NCBI: cellular organisms; Bacteria; Terrabacteria group; Actinobacteria; Actinomycetia; Corynebacteriales; Mycobacteriaceae; Mycobacterium; unclassified Mycobacterium;	NCBI: 1834143	5805928	99.94	1.71	Yes

NCBI Assembly	GCA_001847445.1	NCBI: cellular organisms; Bacteria; Terrabacteria group; Actinobacteria; Actinomycetia; Corynebacteriales; Mycobacteriaceae; Mycobacterium; Mycobacterium tuberculosis complex; Mycobacterium tuberculosis;	NCBI: 1773	4374810	99.94	0.00	Yes
NCBI Assembly	GCA_001849615.1	NCBI: cellular organisms; Bacteria; Terrabacteria group; Actinobacteria; Actinomycetia; Corynebacteriales; Mycobacteriaceae; Mycobacterium; Mycobacterium tuberculosis complex; Mycobacterium tuberculosis;	NCBI: 1773	4351694	99.94	0.00	Yes
NCBI Assembly	GCA_001853885.1	NCBI: cellular organisms; Bacteria; Terrabacteria group; Actinobacteria; Actinomycetia; Corynebacteriales; Mycobacteriaceae; Mycobacteroides; Mycobacteroides chelonae;	NCBI: 1774	5201060	99.50	0.03	Yes
NCBI Assembly	GCA_001792615.1	NCBI: cellular organisms; Bacteria; Terrabacteria group; Actinobacteria; Actinomycetia; Corynebacteriales; Mycobacteriaceae; Mycobacteroides; Mycobacteroides abscessus; Mycobacteroides abscessus subsp. bolletii;	NCBI: 319705	4982977	99.67	0.33	Yes
NCBI Assembly	GCA_001610525.1	NCBI: cellular organisms; Bacteria; Terrabacteria group; Actinobacteria; Actinomycetia; Corynebacteriales; Mycobacteriaceae; Mycobacterium; Mycobacterium avium complex (MAC); Mycobacterium avium; Mycobacterium avium subsp. hominissuis;	NCBI: 439334	5122703	99.83	0.07	Yes
NCBI Assembly	GCA_001667705.1	NCBI: cellular organisms; Bacteria; Terrabacteria group; Actinobacteria; Actinomycetia; Corynebacteriales; Mycobacteriaceae; Mycobacterium; Mycobacterium avium complex (MAC); Mycobacterium intracellulare;	NCBI: 1767	5287387	99.61	0.38	Yes
NCBI Assembly	GCA_001895825.1	NCBI: cellular organisms; Bacteria; Terrabacteria group; Actinobacteria; Actinomycetia; Corynebacteriales; Mycobacteriaceae; Mycobacterium; Mycobacterium tuberculosis complex; Mycobacterium tuberculosis;	NCBI: 1773	4376067	99.94	0.00	Yes
NCBI Assembly	GCA_001852445.1	NCBI: cellular organisms; Bacteria; Terrabacteria group; Actinobacteria; Actinomycetia; Corynebacteriales; Mycobacteriaceae; Mycobacterium; Mycobacterium tuberculosis complex; Mycobacterium tuberculosis;	NCBI: 1773	4406212	99.61	0.00	Yes
NCBI Assembly	GCA_001904595.1	NCBI: cellular organisms; Bacteria; Terrabacteria group; Actinobacteria; Actinomycetia; Corynebacteriales; Mycobacteriaceae; Mycobacterium; Mycobacterium avium complex (MAC); Mycobacterium avium; Mycobacterium avium subsp. paratuberculosis;	NCBI: 1770	4727050	99.22	0.44	Yes
NCBI Assembly	GCA_001667035.1	NCBI: cellular organisms; Bacteria; Terrabacteria group; Actinobacteria; Actinomycetia; Corynebacteriales; Mycobacteriaceae; Mycobacterium; unclassified Mycobacterium;	NCBI: 1834094	5737937	99.00	1.59	Yes
NCBI Assembly	GCA_001846195.1	NCBI: cellular organisms; Bacteria; Terrabacteria group; Actinobacteria; Actinomycetia; Corynebacteriales; Mycobacteriaceae; Mycobacterium; Mycobacterium tuberculosis complex; Mycobacterium tuberculosis;	NCBI: 1773	4398969	99.94	0.00	Yes
NCBI Assembly	GCA_001610545.1	NCBI: cellular organisms; Bacteria; Terrabacteria group; Actinobacteria; Actinomycetia; Corynebacteriales; Mycobacteriaceae; Mycobacterium; Mycobacterium avium complex (MAC); Mycobacterium avium; Mycobacterium avium subsp. hominissuis;	NCBI: 439334	5111701	99.94	0.33	Yes
NCBI Assembly	GCA_001851795.1	NCBI: cellular organisms; Bacteria; Terrabacteria group; Actinobacteria; Actinomycetia; Corynebacteriales; Mycobacteriaceae; Mycobacterium; Mycobacterium tuberculosis complex; Mycobacterium tuberculosis;	NCBI: 1773	4402154	99.94	0.00	Yes
NCBI Assembly	GCA_001849645.1	NCBI: cellular organisms; Bacteria; Terrabacteria group; Actinobacteria; Actinomycetia; Corynebacteriales; Mycobacteriaceae; Mycobacterium; Mycobacterium tuberculosis complex; Mycobacterium tuberculosis;	NCBI: 1773	4412764	99.61	0.00	Yes
NCBI Assembly	GCA_001853895.1	NCBI: cellular organisms; Bacteria; Terrabacteria group; Actinobacteria; Actinomycetia; Corynebacteriales; Mycobacteriaceae; Mycobacteroides; Mycobacteroides chelonae;	NCBI: 1774	4984122	99.50	0.05	Yes
NCBI Assembly	GCA_001847465.1	NCBI: cellular organisms; Bacteria; Terrabacteria group; Actinobacteria; Actinomycetia; Corynebacteriales; Mycobacteriaceae; Mycobacterium; Mycobacterium tuberculosis complex; Mycobacterium tuberculosis;	NCBI: 1773	4400818	99.94	0.00	Yes
NCBI Assembly	GCA_001667735.1	NCBI: cellular organisms; Bacteria; Terrabacteria group; Actinobacteria; Actinomycetia; Corynebacteriales; Mycobacteriaceae; Mycobacterium; unclassified Mycobacterium;	NCBI: 1834129	5672219	99.72	1.38	Yes
NCBI Assembly	GCA_001610145.1	NCBI: cellular organisms; Bacteria; Terrabacteria group; Actinobacteria; Actinomycetia; Corynebacteriales; Mycobacteriaceae; Mycobacterium; Mycobacterium avium complex (MAC); Mycobacterium avium; Mycobacterium avium subsp. hominissuis;	NCBI: 439334	5274796	99.88	0.11	Yes

NCBI Assembly	GCA_001792625.1	NCBI: cellular organisms; Bacteria; Terrabacteria group; Actinobacteria; Actinomycetia; Corynebacteriales; Mycobacteriaceae; Mycobacteroides; Mycobacteroides abscessus; Mycobacteroides abscessus subsp. massiliense;	NCBI: 1962118	4890609	100.00	0.00	Yes
NCBI Assembly	GCA_001852455.1	NCBI: cellular organisms; Bacteria; Terrabacteria group; Actinobacteria; Actinomycetia; Corynebacteriales; Mycobacteriaceae; Mycobacterium; Mycobacterium tuberculosis complex; Mycobacterium tuberculosis;	NCBI: 1773	4404510	99.61	0.00	Yes
NCBI Assembly	GCA_001905305.1	NCBI: cellular organisms; Bacteria; Terrabacteria group; Actinobacteria; Actinomycetia; Corynebacteriales; Mycobacteriaceae; Mycobacterium; unclassified Mycobacterium;	NCBI: 1490483	6829398	98.85	1.28	Yes
NCBI Assembly	GCA_001667065.1	NCBI: cellular organisms; Bacteria; Terrabacteria group; Actinobacteria; Actinomycetia; Corynebacteriales; Mycobacteriaceae; Mycolicibacterium; Mycolicibacterium celeriflavum;	NCBI: 1249101	4927128	100.00	0.00	Yes
NCBI Assembly	GCA_001667745.1	NCBI: cellular organisms; Bacteria; Terrabacteria group; Actinobacteria; Actinomycetia; Corynebacteriales; Mycobacteriaceae; Mycobacterium; unclassified Mycobacterium;	NCBI: 1834124	5359520	99.88	2.40	Yes
NCBI Assembly	GCA_001610565.1	NCBI: cellular organisms; Bacteria; Terrabacteria group; Actinobacteria; Actinomycetia; Corynebacteriales; Mycobacteriaceae; Mycobacterium; Mycobacterium avium complex (MAC); Mycobacterium avium; Mycobacterium avium subsp. hominissuis;	NCBI: 439334	5271935	99.94	0.07	Yes
NCBI Assembly	GCA_001846225.1	NCBI: cellular organisms; Bacteria; Terrabacteria group; Actinobacteria; Actinomycetia; Corynebacteriales; Mycobacteriaceae; Mycobacterium; Mycobacterium tuberculosis complex; Mycobacterium tuberculosis;	NCBI: 1773	4369572	99.94	0.00	Yes
NCBI Assembly	GCA_001849665.1	NCBI: cellular organisms; Bacteria; Terrabacteria group; Actinobacteria; Actinomycetia; Corynebacteriales; Mycobacteriaceae; Mycobacterium; Mycobacterium tuberculosis complex; Mycobacterium tuberculosis;	NCBI: 1773	4378854	99.94	0.00	Yes
NCBI Assembly	GCA_001851825.1	NCBI: cellular organisms; Bacteria; Terrabacteria group; Actinobacteria; Actinomycetia; Corynebacteriales; Mycobacteriaceae; Mycobacterium; Mycobacterium tuberculosis complex; Mycobacterium tuberculosis;	NCBI: 1773	4397700	99.61	0.00	Yes
NCBI Assembly	GCA_001847485.1	NCBI: cellular organisms; Bacteria; Terrabacteria group; Actinobacteria; Actinomycetia; Corynebacteriales; Mycobacteriaceae; Mycobacterium; Mycobacterium tuberculosis complex; Mycobacterium tuberculosis;	NCBI: 1773	4377128	99.94	0.00	Yes
NCBI Assembly	GCA_001853925.1	NCBI: cellular organisms; Bacteria; Terrabacteria group; Actinobacteria; Actinomycetia; Corynebacteriales; Mycobacteriaceae; Mycobacteroides; Mycobacteroides chelonae;	NCBI: 1774	5331455	99.83	0.85	Yes
NCBI Assembly	GCA_001610165.1	NCBI: cellular organisms; Bacteria; Terrabacteria group; Actinobacteria; Actinomycetia; Corynebacteriales; Mycobacteriaceae; Mycobacterium; Mycobacterium avium complex (MAC); Mycobacterium avium; Mycobacterium avium subsp. hominissuis;	NCBI: 439334	5152325	99.94	0.32	Yes
NCBI Assembly	GCA_001792645.1	NCBI: cellular organisms; Bacteria; Terrabacteria group; Actinobacteria; Actinomycetia; Corynebacteriales; Mycobacteriaceae; Mycobacteroides; Mycobacteroides abscessus; Mycobacteroides abscessus subsp. bolletii;	NCBI: 319705	4925476	99.95	0.61	Yes
NCBI Assembly	GCA_001905565.1	NCBI: cellular organisms; Bacteria; Terrabacteria group; Actinobacteria; Actinomycetia; Corynebacteriales; Mycobacteriaceae; Mycobacterium; unclassified Mycobacterium;	NCBI: 1490485	5639487	98.67	2.43	Yes
NCBI Assembly	GCA_001667075.1	NCBI: cellular organisms; Bacteria; Terrabacteria group; Actinobacteria; Actinomycetia; Corynebacteriales; Mycobacteriaceae; Mycobacterium; unclassified Mycobacterium;	NCBI: 1834116	5422651	99.94	0.44	Yes
NCBI Assembly	GCA_001852485.1	NCBI: cellular organisms; Bacteria; Terrabacteria group; Actinobacteria; Actinomycetia; Corynebacteriales; Mycobacteriaceae; Mycobacterium; Mycobacterium tuberculosis complex; Mycobacterium tuberculosis;	NCBI: 1773	4401542	99.61	0.00	Yes
NCBI Assembly	GCA_001667775.1	NCBI: cellular organisms; Bacteria; Terrabacteria group; Actinobacteria; Actinomycetia; Corynebacteriales; Mycobacteriaceae; Mycobacterium; unclassified Mycobacterium;	NCBI: 1834128	5694145	99.28	2.84	Yes
NCBI Assembly	GCA_001611855.1	NCBI: cellular organisms; Bacteria; Terrabacteria group; Actinobacteria; Actinomycetia; Corynebacteriales; Mycobacteriaceae; Mycobacterium; unclassified Mycobacterium;	NCBI: 1561223	4855372	99.50	0.03	Yes

NCBI Assembly	GCA_001851845.1	NCBI: cellular organisms; Bacteria; Terrabacteria group; Actinobacteria; Actinomycetia; Corynebacteriales; Mycobacteriaceae; Mycobacterium; Mycobacterium tuberculosis complex; Mycobacterium tuberculosis;	NCBI: 1773	4411328	99.94	0.00	Yes
NCBI Assembly	GCA_001846235.1	NCBI: cellular organisms; Bacteria; Terrabacteria group; Actinobacteria; Actinomycetia; Corynebacteriales; Mycobacteriaceae; Mycobacterium; Mycobacterium tuberculosis complex; Mycobacterium tuberculosis;	NCBI: 1773	4394031	99.94	0.00	Yes
NCBI Assembly	GCA_001849675.1	NCBI: cellular organisms; Bacteria; Terrabacteria group; Actinobacteria; Actinomycetia; Corynebacteriales; Mycobacteriaceae; Mycobacterium; Mycobacterium tuberculosis complex; Mycobacterium tuberculosis;	NCBI: 1773	4340933	99.94	0.00	Yes
NCBI Assembly	GCA_001853945.1	NCBI: cellular organisms; Bacteria; Terrabacteria group; Actinobacteria; Actinomycetia; Corynebacteriales; Mycobacteriaceae; Mycobacteroides; Mycobacteroides chelonae;	NCBI: 1774	5815049	99.44	0.20	Yes
NCBI Assembly	GCA_001847495.1	NCBI: cellular organisms; Bacteria; Terrabacteria group; Actinobacteria; Actinomycetia; Corynebacteriales; Mycobacteriaceae; Mycobacterium; Mycobacterium tuberculosis complex; Mycobacterium tuberculosis;	NCBI: 1773	4372924	99.94	0.00	Yes
NCBI Assembly	GCA_001610185.1	NCBI: cellular organisms; Bacteria; Terrabacteria group; Actinobacteria; Actinomycetia; Corynebacteriales; Mycobacteriaceae; Mycobacterium; Mycobacterium avium complex (MAC); Mycobacterium avium; Mycobacterium avium subsp. hominissuis;	NCBI: 439334	5203244	99.94	0.00	Yes
NCBI Assembly	GCA_001816205.1	NCBI: cellular organisms; Bacteria; Terrabacteria group; Actinobacteria; Actinomycetia; Corynebacteriales; Mycobacteriaceae; Mycobacterium; Mycobacterium tuberculosis complex; Mycobacterium tuberculosis;	NCBI: 1773	4397833	99.61	0.00	Yes
NCBI Assembly	GCA_001667105.1	NCBI: cellular organisms; Bacteria; Terrabacteria group; Actinobacteria; Actinomycetia; Corynebacteriales; Mycobacteriaceae; Mycobacterium; unclassified Mycobacterium;	NCBI: 1834096	5309753	100.00	0.91	Yes
NCBI Assembly	GCA_001905655.1	NCBI: cellular organisms; Bacteria; Terrabacteria group; Actinobacteria; Actinomycetia; Corynebacteriales; Mycobacteriaceae; Mycobacterium; unclassified Mycobacterium;	NCBI: 1490484	5923116	99.06	1.07	Yes
NCBI Assembly	GCA_001852495.1	NCBI: cellular organisms; Bacteria; Terrabacteria group; Actinobacteria; Actinomycetia; Corynebacteriales; Mycobacteriaceae; Mycobacterium; Mycobacterium tuberculosis complex; Mycobacterium tuberculosis;	NCBI: 1773	4382526	99.61	0.00	Yes
NCBI Assembly	GCA_001667785.1	NCBI: cellular organisms; Bacteria; Terrabacteria group; Actinobacteria; Actinomycetia; Corynebacteriales; Mycobacteriaceae; Mycobacterium; unclassified Mycobacterium;	NCBI: 1856864	5955937	99.25	4.70	Yes
NCBI Assembly	GCA_001846265.1	NCBI: cellular organisms; Bacteria; Terrabacteria group; Actinobacteria; Actinomycetia; Corynebacteriales; Mycobacteriaceae; Mycobacterium; Mycobacterium tuberculosis complex; Mycobacterium tuberculosis;	NCBI: 1773	4393938	99.94	0.00	Yes
NCBI Assembly	GCA_001851855.1	NCBI: cellular organisms; Bacteria; Terrabacteria group; Actinobacteria; Actinomycetia; Corynebacteriales; Mycobacteriaceae; Mycobacterium; Mycobacterium tuberculosis complex; Mycobacterium tuberculosis;	NCBI: 1773	4379086	99.94	0.00	Yes
NCBI Assembly	GCA_001849705.1	NCBI: cellular organisms; Bacteria; Terrabacteria group; Actinobacteria; Actinomycetia; Corynebacteriales; Mycobacteriaceae; Mycobacterium; Mycobacterium tuberculosis complex; Mycobacterium tuberculosis;	NCBI: 1773	4385318	99.94	0.00	Yes
NCBI Assembly	GCA_001847505.1	NCBI: cellular organisms; Bacteria; Terrabacteria group; Actinobacteria; Actinomycetia; Corynebacteriales; Mycobacteriaceae; Mycobacterium; Mycobacterium tuberculosis complex; Mycobacterium tuberculosis;	NCBI: 1773	4403175	99.78	0.00	Yes
NCBI Assembly	GCA_001853965.1	NCBI: cellular organisms; Bacteria; Terrabacteria group; Actinobacteria; Actinomycetia; Corynebacteriales; Mycobacteriaceae; Mycobacteroides; Mycobacteroides chelonae;	NCBI: 1774	5015148	99.50	0.03	Yes
NCBI Assembly	GCA_001610215.1	NCBI: cellular organisms; Bacteria; Terrabacteria group; Actinobacteria; Actinomycetia; Corynebacteriales; Mycobacteriaceae; Mycobacterium; Mycobacterium avium complex (MAC); Mycobacterium avium; Mycobacterium avium subsp. hominissuis;	NCBI: 439334	5309782	99.94	0.00	Yes
NCBI Assembly	GCA_001667115.1	NCBI: cellular organisms; Bacteria; Terrabacteria group; Actinobacteria; Actinomycetia; Corynebacteriales; Mycobacteriaceae; Mycobacterium; unclassified Mycobacterium;	NCBI: 1834097	5529380	99.55	0.44	Yes

NCBI Assembly	GCA_001835505.1	NCBI: cellular organisms; Bacteria; Terrabacteria group; Actinobacteria; Actinomycetia; Corynebacteriales; Mycobacteriaceae; Mycobacterium; Mycobacterium tuberculosis complex; Mycobacterium tuberculosis;	NCBI: 1773	4399594	99.94	0.00	Yes
NCBI Assembly	GCA_001907615.1	NCBI: cellular organisms; Bacteria; Terrabacteria group; Actinobacteria; Actinomycetia; Corynebacteriales; Mycobacteriaceae; Mycobacterium; unclassified Mycobacterium;	NCBI: 1490482	8326240	98.21	2.10	Yes
NCBI Assembly	GCA_001667835.1	NCBI: cellular organisms; Bacteria; Terrabacteria group; Actinobacteria; Actinomycetia; Corynebacteriales; Mycobacteriaceae; Mycobacterium; Mycobacterium avium complex (MAC); Mycobacterium mantenii;	NCBI: 560555	5688292	99.61	1.28	Yes
NCBI Assembly	GCA_001852525.1	NCBI: cellular organisms; Bacteria; Terrabacteria group; Actinobacteria; Actinomycetia; Corynebacteriales; Mycobacteriaceae; Mycobacterium; Mycobacterium tuberculosis complex; Mycobacterium tuberculosis;	NCBI: 1773	4378819	99.61	0.00	Yes
NCBI Assembly	GCA_001632885.1	NCBI: cellular organisms; Bacteria; Terrabacteria group; Actinobacteria; Actinomycetia; Corynebacteriales; Mycobacteriaceae; Mycobacterium; Mycobacterium kansasii;	NCBI: 1768	5627124	99.67	0.17	Yes
NCBI Assembly	GCA_001846275.1	NCBI: cellular organisms; Bacteria; Terrabacteria group; Actinobacteria; Actinomycetia; Corynebacteriales; Mycobacteriaceae; Mycobacterium; Mycobacterium tuberculosis complex; Mycobacterium tuberculosis;	NCBI: 1773	4396440	99.94	0.00	Yes
NCBI Assembly	GCA_001853985.1	NCBI: cellular organisms; Bacteria; Terrabacteria group; Actinobacteria; Actinomycetia; Corynebacteriales; Mycobacteriaceae; Mycobacteroides; Mycobacteroides chelonae;	NCBI: 1774	5062740	99.50	0.41	Yes
NCBI Assembly	GCA_001851875.1	NCBI: cellular organisms; Bacteria; Terrabacteria group; Actinobacteria; Actinomycetia; Corynebacteriales; Mycobacteriaceae; Mycobacterium; Mycobacterium tuberculosis complex; Mycobacterium tuberculosis;	NCBI: 1773	4395665	99.94	0.00	Yes
NCBI Assembly	GCA_001849725.1	NCBI: cellular organisms; Bacteria; Terrabacteria group; Actinobacteria; Actinomycetia; Corynebacteriales; Mycobacteriaceae; Mycobacterium; Mycobacterium tuberculosis complex; Mycobacterium tuberculosis;	NCBI: 1773	4348691	99.94	0.00	Yes
NCBI Assembly	GCA_001847545.1	NCBI: cellular organisms; Bacteria; Terrabacteria group; Actinobacteria; Actinomycetia; Corynebacteriales; Mycobacteriaceae; Mycobacterium; Mycobacterium tuberculosis complex; Mycobacterium tuberculosis;	NCBI: 1773	4378202	99.94	0.00	Yes
NCBI Assembly	GCA_001610235.1	NCBI: cellular organisms; Bacteria; Terrabacteria group; Actinobacteria; Actinomycetia; Corynebacteriales; Mycobacteriaceae; Mycobacterium; Mycobacterium avium complex (MAC); Mycobacterium avium; Mycobacterium avium subsp. hominissuis;	NCBI: 439334	5349007	99.94	0.00	Yes
NCBI Assembly	GCA_001667145.1	NCBI: cellular organisms; Bacteria; Terrabacteria group; Actinobacteria; Actinomycetia; Corynebacteriales; Mycobacteriaceae; Mycobacterium; unclassified Mycobacterium;	NCBI: 1834106	5033627	99.94	0.33	Yes
NCBI Assembly	GCA_001907655.1	NCBI: cellular organisms; Bacteria; Terrabacteria group; Actinobacteria; Actinomycetia; Corynebacteriales; Mycobacteriaceae; Mycolicibacterium; Mycolicibacterium diernhoferi;	NCBI: 1801	5982379	99.34	0.19	Yes
NCBI Assembly	GCA_001835515.1	NCBI: cellular organisms; Bacteria; Terrabacteria group; Actinobacteria; Actinomycetia; Corynebacteriales; Mycobacteriaceae; Mycobacterium; Mycobacterium tuberculosis complex; Mycobacterium tuberculosis;	NCBI: 1773	4331816	99.94	0.00	Yes
NCBI Assembly	GCA_001667865.1	NCBI: cellular organisms; Bacteria; Terrabacteria group; Actinobacteria; Actinomycetia; Corynebacteriales; Mycobacteriaceae; Mycobacterium; Mycobacterium avium complex (MAC); Mycobacterium mantenii;	NCBI: 560555	5792343	99.94	0.50	Yes
NCBI Assembly	GCA_001852535.1	NCBI: cellular organisms; Bacteria; Terrabacteria group; Actinobacteria; Actinomycetia; Corynebacteriales; Mycobacteriaceae; Mycobacterium; Mycobacterium tuberculosis complex; Mycobacterium tuberculosis;	NCBI: 1773	4388430	99.94	0.00	Yes
NCBI Assembly	GCA_001846305.1	NCBI: cellular organisms; Bacteria; Terrabacteria group; Actinobacteria; Actinomycetia; Corynebacteriales; Mycobacteriaceae; Mycobacterium; Mycobacterium tuberculosis complex; Mycobacterium tuberculosis;	NCBI: 1773	4395988	99.94	0.00	Yes
NCBI Assembly	GCA_001849745.1	NCBI: cellular organisms; Bacteria; Terrabacteria group; Actinobacteria; Actinomycetia; Corynebacteriales; Mycobacteriaceae; Mycobacterium; Mycobacterium tuberculosis complex; Mycobacterium tuberculosis;	NCBI: 1773	4386764	99.61	0.07	Yes

NCBI Assembly	GCA_001853995.1	NCBI: cellular organisms; Bacteria; Terrabacteria group; Actinobacteria; Actinomycetia; Corynebacteriales; Mycobacteriaceae; Mycobacteroides; Mycobacteroides chelonae;	NCBI: 1774	5878106	99.44	3.53	Yes
NCBI Assembly	GCA_001851885.1	NCBI: cellular organisms; Bacteria; Terrabacteria group; Actinobacteria; Actinomycetia; Corynebacteriales; Mycobacteriaceae; Mycobacterium; Mycobacterium tuberculosis complex; Mycobacterium tuberculosis;	NCBI: 1773	4400054	99.94	0.00	Yes
NCBI Assembly	GCA_001847565.1	NCBI: cellular organisms; Bacteria; Terrabacteria group; Actinobacteria; Actinomycetia; Corynebacteriales; Mycobacteriaceae; Mycobacterium; Mycobacterium tuberculosis complex; Mycobacterium tuberculosis;	NCBI: 1773	4386830	99.94	0.00	Yes
NCBI Assembly	GCA_001610245.1	NCBI: cellular organisms; Bacteria; Terrabacteria group; Actinobacteria; Actinomycetia; Corynebacteriales; Mycobacteriaceae; Mycobacterium; Mycobacterium avium complex (MAC); Mycobacterium avium; Mycobacterium avium subsp. hominissuis;	NCBI: 439334	5090633	99.94	0.07	Yes
NCBI Assembly	GCA_001632895.1	NCBI: cellular organisms; Bacteria; Terrabacteria group; Actinobacteria; Actinomycetia; Corynebacteriales; Mycobacteriaceae; Mycobacterium; Mycobacterium ostraviense;	NCBI: 2738409	6027332	99.28	0.32	Yes
NCBI Assembly	GCA_001667155.1	NCBI: cellular organisms; Bacteria; Terrabacteria group; Actinobacteria; Actinomycetia; Corynebacteriales; Mycobacteriaceae; Mycobacterium; unclassified Mycobacterium;	NCBI: 1834107	5385761	99.78	0.68	Yes
NCBI Assembly	GCA_001667885.1	NCBI: cellular organisms; Bacteria; Terrabacteria group; Actinobacteria; Actinomycetia; Corynebacteriales; Mycobacteriaceae; Mycobacterium; Mycobacterium scrofulaceum;	NCBI: 1783	5536898	99.22	1.91	Yes
NCBI Assembly	GCA_001835525.1	NCBI: cellular organisms; Bacteria; Terrabacteria group; Actinobacteria; Actinomycetia; Corynebacteriales; Mycobacteriaceae; Mycobacterium; Mycobacterium tuberculosis complex; Mycobacterium tuberculosis;	NCBI: 1773	4348897	99.94	0.00	Yes
NCBI Assembly	GCA_001907675.1	NCBI: cellular organisms; Bacteria; Terrabacteria group; Actinobacteria; Actinomycetia; Corynebacteriales; Mycobacteriaceae; Mycobacterium; Mycobacterium paraffinicum;	NCBI: 53378	6475312	99.57	1.03	Yes
NCBI Assembly	GCA_001852555.1	NCBI: cellular organisms; Bacteria; Terrabacteria group; Actinobacteria; Actinomycetia; Corynebacteriales; Mycobacteriaceae; Mycobacterium; Mycobacterium tuberculosis complex; Mycobacterium tuberculosis;	NCBI: 1773	4407201	99.94	0.00	Yes
NCBI Assembly	GCA_001846315.1	NCBI: cellular organisms; Bacteria; Terrabacteria group; Actinobacteria; Actinomycetia; Corynebacteriales; Mycobacteriaceae; Mycobacterium; Mycobacterium tuberculosis complex; Mycobacterium tuberculosis;	NCBI: 1773	4358863	99.94	0.00	Yes
NCBI Assembly	GCA_001849755.1	NCBI: cellular organisms; Bacteria; Terrabacteria group; Actinobacteria; Actinomycetia; Corynebacteriales; Mycobacteriaceae; Mycobacterium; Mycobacterium tuberculosis complex; Mycobacterium tuberculosis;	NCBI: 1773	4403972	99.94	0.00	Yes
NCBI Assembly	GCA_001847585.1	NCBI: cellular organisms; Bacteria; Terrabacteria group; Actinobacteria; Actinomycetia; Corynebacteriales; Mycobacteriaceae; Mycobacterium; Mycobacterium tuberculosis complex; Mycobacterium tuberculosis;	NCBI: 1773	4376052	99.61	0.00	Yes
NCBI Assembly	GCA_001854015.1	NCBI: cellular organisms; Bacteria; Terrabacteria group; Actinobacteria; Actinomycetia; Corynebacteriales; Mycobacteriaceae; Mycobacteroides; Mycobacteroides chelonae;	NCBI: 1774	5004135	99.50	0.03	Yes
NCBI Assembly	GCA_001851925.1	NCBI: cellular organisms; Bacteria; Terrabacteria group; Actinobacteria; Actinomycetia; Corynebacteriales; Mycobacteriaceae; Mycobacterium; Mycobacterium tuberculosis complex; Mycobacterium tuberculosis;	NCBI: 1773	4380471	99.94	0.00	Yes
NCBI Assembly	GCA_001610265.1	NCBI: cellular organisms; Bacteria; Terrabacteria group; Actinobacteria; Actinomycetia; Corynebacteriales; Mycobacteriaceae; Mycobacterium; Mycobacterium avium complex (MAC); Mycobacterium avium; Mycobacterium avium subsp. hominissuis;	NCBI: 439334	5137984	99.61	0.90	Yes
NCBI Assembly	GCA_001667185.1	NCBI: cellular organisms; Bacteria; Terrabacteria group; Actinobacteria; Actinomycetia; Corynebacteriales; Mycobacteriaceae; Mycobacterium; unclassified Mycobacterium;	NCBI: 1834092	5520891	99.83	1.57	Yes
NCBI Assembly	GCA_001632905.1	NCBI: cellular organisms; Bacteria; Terrabacteria group; Actinobacteria; Actinomycetia; Corynebacteriales; Mycobacteriaceae; Mycobacterium; Mycobacterium kansasii;	NCBI: 1768	6171688	99.61	0.58	Yes

NCBI Assembly	GCA_001667905.1	NCBI: cellular organisms; Bacteria; Terrabacteria group; Actinobacteria; Actinomycetia; Corynebacteriales; Mycobacteriaceae; Mycobacterium; Mycobacterium avium complex (MAC); Mycobacterium colombiense;	NCBI: 339268	5813025	99.94	0.49	Yes
NCBI Assembly	GCA_001835535.1	NCBI: cellular organisms; Bacteria; Terrabacteria group; Actinobacteria; Actinomycetia; Corynebacteriales; Mycobacteriaceae; Mycobacterium; Mycobacterium tuberculosis complex; Mycobacterium tuberculosis;	NCBI: 1773	4357317	99.86	0.00	Yes
NCBI Assembly	GCA_001922485.1	NCBI: cellular organisms; Bacteria; Terrabacteria group; Actinobacteria; Actinomycetia; Corynebacteriales; Mycobacteriaceae; Mycobacterium; Mycobacterium tuberculosis complex; Mycobacterium tuberculosis;	NCBI: 1773	4411511	99.94	0.00	Yes
NCBI Assembly	GCA_001852575.1	NCBI: cellular organisms; Bacteria; Terrabacteria group; Actinobacteria; Actinomycetia; Corynebacteriales; Mycobacteriaceae; Mycobacterium; Mycobacterium tuberculosis complex; Mycobacterium tuberculosis;	NCBI: 1773	4396415	99.94	0.00	Yes
NCBI Assembly	GCA_001846345.1	NCBI: cellular organisms; Bacteria; Terrabacteria group; Actinobacteria; Actinomycetia; Corynebacteriales; Mycobacteriaceae; Mycobacterium; Mycobacterium tuberculosis complex; Mycobacterium tuberculosis;	NCBI: 1773	4400592	99.94	0.00	Yes
NCBI Assembly	GCA_001847595.1	NCBI: cellular organisms; Bacteria; Terrabacteria group; Actinobacteria; Actinomycetia; Corynebacteriales; Mycobacteriaceae; Mycobacterium; Mycobacterium tuberculosis complex; Mycobacterium tuberculosis;	NCBI: 1773	4411363	99.94	0.00	Yes
NCBI Assembly	GCA_001849785.1	NCBI: cellular organisms; Bacteria; Terrabacteria group; Actinobacteria; Actinomycetia; Corynebacteriales; Mycobacteriaceae; Mycobacterium; Mycobacterium tuberculosis complex; Mycobacterium tuberculosis;	NCBI: 1773	4385535	99.94	0.00	Yes
NCBI Assembly	GCA_001851935.1	NCBI: cellular organisms; Bacteria; Terrabacteria group; Actinobacteria; Actinomycetia; Corynebacteriales; Mycobacteriaceae; Mycobacterium; Mycobacterium tuberculosis complex; Mycobacterium tuberculosis;	NCBI: 1773	4385066	99.86	0.00	Yes
NCBI Assembly	GCA_001610285.1	NCBI: cellular organisms; Bacteria; Terrabacteria group; Actinobacteria; Actinomycetia; Corynebacteriales; Mycobacteriaceae; Mycobacterium; Mycobacterium avium complex (MAC); Mycobacterium avium; Mycobacterium avium subsp. hominissuis;	NCBI: 439334	5340230	99.94	0.21	Yes
NCBI Assembly	GCA_001854035.1	NCBI: cellular organisms; Bacteria; Terrabacteria group; Actinobacteria; Actinomycetia; Corynebacteriales; Mycobacteriaceae; Mycobacteroides; Mycobacteroides chelonae;	NCBI: 1774	5267463	99.50	0.37	Yes
NCBI Assembly	GCA_001632915.1	NCBI: cellular organisms; Bacteria; Terrabacteria group; Actinobacteria; Actinomycetia; Corynebacteriales; Mycobacteriaceae; Mycobacterium; Mycobacterium kansasii;	NCBI: 1768	6141835	99.28	1.01	Yes
NCBI Assembly	GCA_001850495.1	NCBI: cellular organisms; Bacteria; Terrabacteria group; Actinobacteria; Actinomycetia; Corynebacteriales; Mycobacteriaceae; Mycobacterium; Mycobacterium tuberculosis complex; Mycobacterium tuberculosis;	NCBI: 1773	4399291	99.61	0.00	Yes
NCBI Assembly	GCA_001640605.1	NCBI: cellular organisms; Bacteria; Terrabacteria group; Actinobacteria; Actinomycetia; Corynebacteriales; Mycobacteriaceae; Mycobacterium; Mycobacterium tuberculosis complex; Mycobacterium tuberculosis;	NCBI: 1773	4371105	99.94	0.00	Yes
NCBI Assembly	GCA_001839705.1	NCBI: cellular organisms; Bacteria; Terrabacteria group; Actinobacteria; Actinomycetia; Corynebacteriales; Mycobacteriaceae; Mycobacterium; Mycobacterium tuberculosis complex; Mycobacterium tuberculosis;	NCBI: 1773	4397285	99.94	0.00	Yes
NCBI Assembly	GCA_001936215.1	NCBI: cellular organisms; Bacteria; Terrabacteria group; Actinobacteria; Actinomycetia; Corynebacteriales; Mycobacteriaceae; Mycobacterium; Mycobacterium avium complex (MAC); Mycobacterium avium; Mycobacterium avium subsp. hominissuis;	NCBI: 439334	5626623	99.94	0.42	Yes
NCBI Assembly	GCA_001852605.1	NCBI: cellular organisms; Bacteria; Terrabacteria group; Actinobacteria; Actinomycetia; Corynebacteriales; Mycobacteriaceae; Mycobacterium; Mycobacterium tuberculosis complex; Mycobacterium tuberculosis;	NCBI: 1773	4402546	99.61	0.00	Yes
NCBI Assembly	GCA_001846355.1	NCBI: cellular organisms; Bacteria; Terrabacteria group; Actinobacteria; Actinomycetia; Corynebacteriales; Mycobacteriaceae; Mycobacterium; Mycobacterium tuberculosis complex; Mycobacterium tuberculosis;	NCBI: 1773	4403276	99.94	0.00	Yes
NCBI Assembly	GCA_001847615.1	NCBI: cellular organisms; Bacteria; Terrabacteria group; Actinobacteria; Actinomycetia; Corynebacteriales; Mycobacteriaceae; Mycobacterium; Mycobacterium tuberculosis complex; Mycobacterium tuberculosis;	NCBI: 1773	4381257	99.94	0.00	Yes

NCBI Assembly	GCA_001851945.1	NCBI: cellular organisms; Bacteria; Terrabacteria group; Actinobacteria; Actinomycetia; Corynebacteriales; Mycobacteriaceae; Mycobacterium; Mycobacterium tuberculosis complex; Mycobacterium tuberculosis;	NCBI: 1773	4400614	99.94	0.00	Yes
NCBI Assembly	GCA_001849805.1	NCBI: cellular organisms; Bacteria; Terrabacteria group; Actinobacteria; Actinomycetia; Corynebacteriales; Mycobacteriaceae; Mycobacterium; Mycobacterium tuberculosis complex; Mycobacterium tuberculosis;	NCBI: 1773	4388938	99.94	0.00	Yes
NCBI Assembly	GCA_001610315.1	NCBI: cellular organisms; Bacteria; Terrabacteria group; Actinobacteria; Actinomycetia; Corynebacteriales; Mycobacteriaceae; Mycobacterium; Mycobacterium avium complex (MAC); Mycobacterium avium; Mycobacterium avium subsp. hominissuis;	NCBI: 439334	5050135	99.94	0.00	Yes
NCBI Assembly	GCA_001854525.1	NCBI: cellular organisms; Bacteria; Terrabacteria group; Actinobacteria; Actinomycetia; Corynebacteriales; Mycobacteriaceae; Mycobacterium; Mycobacterium talmoniae;	NCBI: 1858794	5622166	99.66	1.21	Yes
NCBI Assembly	GCA_001632965.1	NCBI: cellular organisms; Bacteria; Terrabacteria group; Actinobacteria; Actinomycetia; Corynebacteriales; Mycobacteriaceae; Mycobacterium; Mycobacterium kansasii;	NCBI: 1768	6358301	99.61	0.38	Yes
NCBI Assembly	GCA_001850525.1	NCBI: cellular organisms; Bacteria; Terrabacteria group; Actinobacteria; Actinomycetia; Corynebacteriales; Mycobacteriaceae; Mycobacterium; Mycobacterium tuberculosis complex; Mycobacterium tuberculosis;	NCBI: 1773	4400947	99.61	0.00	Yes
NCBI Assembly	GCA_001640615.1	NCBI: cellular organisms; Bacteria; Terrabacteria group; Actinobacteria; Actinomycetia; Corynebacteriales; Mycobacteriaceae; Mycobacterium; Mycobacterium tuberculosis complex; Mycobacterium tuberculosis;	NCBI: 1773	4352163	99.94	0.00	Yes
NCBI Assembly	GCA_001839715.1	NCBI: cellular organisms; Bacteria; Terrabacteria group; Actinobacteria; Actinomycetia; Corynebacteriales; Mycobacteriaceae; Mycobacterium; Mycobacterium tuberculosis complex; Mycobacterium tuberculosis;	NCBI: 1773	4397401	99.94	0.00	Yes
NCBI Assembly	GCA_001938095.1	NCBI: cellular organisms; Bacteria; Terrabacteria group; Actinobacteria; Actinomycetia; Corynebacteriales; Mycobacteriaceae; Mycobacterium; Mycobacterium avium complex (MAC); Mycobacterium intracellulare; Mycobacterium intracellulare subsp. chimaera;	NCBI: 222805	6454529	99.94	1.04	Yes
NCBI Assembly	GCA_001846385.1	NCBI: cellular organisms; Bacteria; Terrabacteria group; Actinobacteria; Actinomycetia; Corynebacteriales; Mycobacteriaceae; Mycobacterium; Mycobacterium tuberculosis complex; Mycobacterium tuberculosis;	NCBI: 1773	4383428	99.94	0.00	Yes
NCBI Assembly	GCA_001610335.1	NCBI: cellular organisms; Bacteria; Terrabacteria group; Actinobacteria; Actinomycetia; Corynebacteriales; Mycobacteriaceae; Mycobacterium; Mycobacterium avium complex (MAC); Mycobacterium avium; Mycobacterium avium subsp. hominissuis;	NCBI: 439334	5057685	99.94	0.00	Yes
NCBI Assembly	GCA_001852625.1	NCBI: cellular organisms; Bacteria; Terrabacteria group; Actinobacteria; Actinomycetia; Corynebacteriales; Mycobacteriaceae; Mycobacterium; Mycobacterium tuberculosis complex; Mycobacterium tuberculosis;	NCBI: 1773	4365623	99.61	0.00	Yes
NCBI Assembly	GCA_001851955.1	NCBI: cellular organisms; Bacteria; Terrabacteria group; Actinobacteria; Actinomycetia; Corynebacteriales; Mycobacteriaceae; Mycobacterium; Mycobacterium tuberculosis complex; Mycobacterium tuberculosis;	NCBI: 1773	4347779	99.94	0.00	Yes
NCBI Assembly	GCA_001849825.1	NCBI: cellular organisms; Bacteria; Terrabacteria group; Actinobacteria; Actinomycetia; Corynebacteriales; Mycobacteriaceae; Mycobacterium; Mycobacterium tuberculosis complex; Mycobacterium tuberculosis;	NCBI: 1773	4361808	99.94	0.00	Yes
NCBI Assembly	GCA_001847645.1	NCBI: cellular organisms; Bacteria; Terrabacteria group; Actinobacteria; Actinomycetia; Corynebacteriales; Mycobacteriaceae; Mycobacterium; Mycobacterium tuberculosis complex; Mycobacterium tuberculosis;	NCBI: 1773	4403269	99.94	0.00	Yes
NCBI Assembly	GCA_001855255.1	NCBI: cellular organisms; Bacteria; Terrabacteria group; Actinobacteria; Actinomycetia; Corynebacteriales; Mycobacteriaceae; Mycobacterium; Mycobacterium tuberculosis complex; Mycobacterium tuberculosis;	NCBI: 1773	4402033	99.94	0.00	Yes
NCBI Assembly	GCA_001632975.1	NCBI: cellular organisms; Bacteria; Terrabacteria group; Actinobacteria; Actinomycetia; Corynebacteriales; Mycobacteriaceae; Mycobacterium; Mycobacterium persicum;	NCBI: 1487726	6266032	99.54	0.33	Yes
NCBI Assembly	GCA_001850535.1	NCBI: cellular organisms; Bacteria; Terrabacteria group; Actinobacteria; Actinomycetia; Corynebacteriales; Mycobacteriaceae; Mycobacterium; Mycobacterium tuberculosis complex; Mycobacterium tuberculosis;	NCBI: 1773	4369366	99.94	0.00	Yes

NCBI Assembly	GCA_001640645.1	NCBI: cellular organisms; Bacteria; Terrabacteria group; Actinobacteria; Actinomycetia; Corynebacteriales; Mycobacteriaceae; Mycobacterium; Mycobacterium tuberculosis complex; Mycobacterium tuberculosis;	NCBI: 1773	4356783	99.94	0.00	Yes
NCBI Assembly	GCA_001839725.1	NCBI: cellular organisms; Bacteria; Terrabacteria group; Actinobacteria; Actinomycetia; Corynebacteriales; Mycobacteriaceae; Mycobacterium; Mycobacterium tuberculosis complex; Mycobacterium tuberculosis;	NCBI: 1773	4394144	99.94	0.00	Yes
NCBI Assembly	GCA_001941665.1	NCBI: cellular organisms; Bacteria; Terrabacteria group; Actinobacteria; Actinomycetia; Corynebacteriales; Mycobacteriaceae; Mycobacterium; Mycobacterium tuberculosis complex; Mycobacterium tuberculosis; Mycobacterium tuberculosis variant caprae;	NCBI: 115862	4324961	99.78	0.00	Yes
NCBI Assembly	GCA_001846395.1	NCBI: cellular organisms; Bacteria; Terrabacteria group; Actinobacteria; Actinomycetia; Corynebacteriales; Mycobacteriaceae; Mycobacterium; Mycobacterium tuberculosis complex; Mycobacterium tuberculosis;	NCBI: 1773	4391687	99.94	0.00	Yes
NCBI Assembly	GCA_001610355.1	NCBI: cellular organisms; Bacteria; Terrabacteria group; Actinobacteria; Actinomycetia; Corynebacteriales; Mycobacteriaceae; Mycobacterium; Mycobacterium avium complex (MAC); Mycobacterium avium; Mycobacterium avium subsp. hominissuis;	NCBI: 439334	5145571	99.94	0.11	Yes
NCBI Assembly	GCA_001847665.1	NCBI: cellular organisms; Bacteria; Terrabacteria group; Actinobacteria; Actinomycetia; Corynebacteriales; Mycobacteriaceae; Mycobacterium; Mycobacterium tuberculosis complex; Mycobacterium tuberculosis;	NCBI: 1773	4375268	99.94	0.00	Yes
NCBI Assembly	GCA_001852635.1	NCBI: cellular organisms; Bacteria; Terrabacteria group; Actinobacteria; Actinomycetia; Corynebacteriales; Mycobacteriaceae; Mycobacterium; Mycobacterium tuberculosis complex; Mycobacterium tuberculosis;	NCBI: 1773	4393489	99.94	0.00	Yes
NCBI Assembly	GCA_001852005.1	NCBI: cellular organisms; Bacteria; Terrabacteria group; Actinobacteria; Actinomycetia; Corynebacteriales; Mycobacteriaceae; Mycobacterium; Mycobacterium tuberculosis complex; Mycobacterium tuberculosis;	NCBI: 1773	4379587	99.94	0.00	Yes
NCBI Assembly	GCA_001849845.1	NCBI: cellular organisms; Bacteria; Terrabacteria group; Actinobacteria; Actinomycetia; Corynebacteriales; Mycobacteriaceae; Mycobacterium; Mycobacterium tuberculosis complex; Mycobacterium tuberculosis;	NCBI: 1773	4375651	99.94	0.00	Yes
NCBI Assembly	GCA_001640575.1	NCBI: cellular organisms; Bacteria; Terrabacteria group; Actinobacteria; Actinomycetia; Corynebacteriales; Mycobacteriaceae; Mycobacterium; Mycobacterium tuberculosis complex; Mycobacterium tuberculosis;	NCBI: 1773	4392768	99.94	0.00	Yes
NCBI Assembly	GCA_001855355.1	NCBI: cellular organisms; Bacteria; Terrabacteria group; Actinobacteria; Actinomycetia; Corynebacteriales; Mycobacteriaceae; Mycobacterium; Mycobacterium avium complex (MAC); Mycobacterium avium; Mycobacterium avium subsp. paratuberculosis;	NCBI: 1770	4777365	99.61	0.33	Yes
NCBI Assembly	GCA_001850565.1	NCBI: cellular organisms; Bacteria; Terrabacteria group; Actinobacteria; Actinomycetia; Corynebacteriales; Mycobacteriaceae; Mycobacterium; Mycobacterium tuberculosis complex; Mycobacterium tuberculosis;	NCBI: 1773	4375377	99.94	0.00	Yes
NCBI Assembly	GCA_001640695.1	NCBI: cellular organisms; Bacteria; Terrabacteria group; Actinobacteria; Actinomycetia; Corynebacteriales; Mycobacteriaceae; Mycobacterium; Mycobacterium tuberculosis complex; Mycobacterium tuberculosis;	NCBI: 1773	4355856	99.94	0.00	Yes
NCBI Assembly	GCA_001839735.1	NCBI: cellular organisms; Bacteria; Terrabacteria group; Actinobacteria; Actinomycetia; Corynebacteriales; Mycobacteriaceae; Mycobacterium; Mycobacterium tuberculosis complex; Mycobacterium tuberculosis;	NCBI: 1773	4406500	99.94	0.00	Yes
NCBI Assembly	GCA_001610375.1	NCBI: cellular organisms; Bacteria; Terrabacteria group; Actinobacteria; Actinomycetia; Corynebacteriales; Mycobacteriaceae; Mycobacterium; Mycobacterium avium complex (MAC); Mycobacterium avium; Mycobacterium avium subsp. hominissuis;	NCBI: 439334	5036170	99.94	0.00	Yes
NCBI Assembly	GCA_001847065.1	NCBI: cellular organisms; Bacteria; Terrabacteria group; Actinobacteria; Actinomycetia; Corynebacteriales; Mycobacteriaceae; Mycobacterium; Mycobacterium tuberculosis complex; Mycobacterium tuberculosis;	NCBI: 1773	4392655	99.94	0.00	Yes
NCBI Assembly	GCA_001942045.1	NCBI: cellular organisms; Bacteria; Terrabacteria group; Actinobacteria; Actinomycetia; Corynebacteriales; Mycobacteriaceae; Mycolicibacterium; Mycolicibacterium porcinum;	NCBI: 39693	6240140	99.72	0.00	Yes
NCBI Assembly	GCA_001845495.1	NCBI: cellular organisms; Bacteria; Terrabacteria group; Actinobacteria; Actinomycetia; Corynebacteriales; Mycobacteriaceae; Mycobacterium; Mycobacterium tuberculosis complex; Mycobacterium tuberculosis;	NCBI: 1773	4391885	99.94	0.00	Yes

NCBI Assembly	GCA_001852665.1	NCBI: cellular organisms; Bacteria; Terrabacteria group; Actinobacteria; Actinomycetia; Corynebacteriales; Mycobacteriaceae; Mycobacterium; Mycobacterium tuberculosis complex; Mycobacterium tuberculosis;	NCBI: 1773	4411102	99.94	0.00	Yes
NCBI Assembly	GCA_001849865.1	NCBI: cellular organisms; Bacteria; Terrabacteria group; Actinobacteria; Actinomycetia; Corynebacteriales; Mycobacteriaceae; Mycobacterium; Mycobacterium tuberculosis complex; Mycobacterium tuberculosis;	NCBI: 1773	4401452	99.94	0.00	Yes
NCBI Assembly	GCA_001852025.1	NCBI: cellular organisms; Bacteria; Terrabacteria group; Actinobacteria; Actinomycetia; Corynebacteriales; Mycobacteriaceae; Mycobacterium; Mycobacterium tuberculosis complex; Mycobacterium tuberculosis;	NCBI: 1773	4407362	99.94	0.00	Yes
NCBI Assembly	GCA_001846735.1	NCBI: cellular organisms; Bacteria; Terrabacteria group; Actinobacteria; Actinomycetia; Corynebacteriales; Mycobacteriaceae; Mycobacterium; Mycobacterium tuberculosis complex; Mycobacterium tuberculosis;	NCBI: 1773	4398520	99.94	0.00	Yes
NCBI Assembly	GCA_001677135.1	NCBI: cellular organisms; Bacteria; Terrabacteria group; Actinobacteria; Actinomycetia; Corynebacteriales; Mycobacteriaceae; Mycobacteroides; Mycobacteroides immunogenum;	NCBI: 83262	5604845	99.61	2.15	Yes
NCBI Assembly	GCA_001850585.1	NCBI: cellular organisms; Bacteria; Terrabacteria group; Actinobacteria; Actinomycetia; Corynebacteriales; Mycobacteriaceae; Mycobacterium; Mycobacterium tuberculosis complex; Mycobacterium tuberculosis;	NCBI: 1773	4380881	99.94	0.00	Yes
NCBI Assembly	GCA_001640705.1	NCBI: cellular organisms; Bacteria; Terrabacteria group; Actinobacteria; Actinomycetia; Corynebacteriales; Mycobacteriaceae; Mycobacterium; Mycobacterium tuberculosis complex; Mycobacterium tuberculosis;	NCBI: 1773	4374121	99.94	0.00	Yes
NCBI Assembly	GCA_001605725.1	NCBI: cellular organisms; Bacteria; Terrabacteria group; Actinobacteria; Actinomycetia; Corynebacteriales; Mycobacteriaceae; Mycobacteroides; Mycobacteroides immunogenum;	NCBI: 83262	5573781	99.61	2.15	Yes
NCBI Assembly	GCA_001610395.1	NCBI: cellular organisms; Bacteria; Terrabacteria group; Actinobacteria; Actinomycetia; Corynebacteriales; Mycobacteriaceae; Mycobacterium; Mycobacterium avium complex (MAC); Mycobacterium avium; Mycobacterium avium subsp. hominissuis;	NCBI: 439334	5177134	99.94	0.00	Yes
NCBI Assembly	GCA_001847075.1	NCBI: cellular organisms; Bacteria; Terrabacteria group; Actinobacteria; Actinomycetia; Corynebacteriales; Mycobacteriaceae; Mycobacterium; Mycobacterium tuberculosis complex; Mycobacterium tuberculosis;	NCBI: 1773	4384444	99.94	0.00	Yes
NCBI Assembly	GCA_001839785.1	NCBI: cellular organisms; Bacteria; Terrabacteria group; Actinobacteria; Actinomycetia; Corynebacteriales; Mycobacteriaceae; Mycobacterium; Mycobacterium tuberculosis complex; Mycobacterium tuberculosis;	NCBI: 1773	4402788	99.94	0.00	Yes
NCBI Assembly	GCA_001846745.1	NCBI: cellular organisms; Bacteria; Terrabacteria group; Actinobacteria; Actinomycetia; Corynebacteriales; Mycobacteriaceae; Mycobacterium; Mycobacterium tuberculosis complex; Mycobacterium tuberculosis;	NCBI: 1773	4391067	99.94	0.00	Yes
NCBI Assembly	GCA_001845515.1	NCBI: cellular organisms; Bacteria; Terrabacteria group; Actinobacteria; Actinomycetia; Corynebacteriales; Mycobacteriaceae; Mycobacterium; Mycobacterium tuberculosis complex; Mycobacterium tuberculosis;	NCBI: 1773	4392363	99.94	0.00	Yes
NCBI Assembly	GCA_001845175.1	NCBI: cellular organisms; Bacteria; Terrabacteria group; Actinobacteria; Actinomycetia; Corynebacteriales; Mycobacteriaceae; Mycobacterium; Mycobacterium tuberculosis complex; Mycobacterium tuberculosis;	NCBI: 1773	4421807	99.94	0.00	Yes
NCBI Assembly	GCA_001679965.1	NCBI: cellular organisms; Bacteria; Terrabacteria group; Actinobacteria; Actinomycetia; Corynebacteriales; Mycobacteriaceae; Mycolicibacter; Mycolicibacter kumamotonensis;	NCBI: 354243	5330450	99.09	0.38	Yes
NCBI Assembly	GCA_001852045.1	NCBI: cellular organisms; Bacteria; Terrabacteria group; Actinobacteria; Actinomycetia; Corynebacteriales; Mycobacteriaceae; Mycobacterium; Mycobacterium tuberculosis complex; Mycobacterium tuberculosis;	NCBI: 1773	4394493	99.94	0.00	Yes
NCBI Assembly	GCA_001848925.1	NCBI: cellular organisms; Bacteria; Terrabacteria group; Actinobacteria; Actinomycetia; Corynebacteriales; Mycobacteriaceae; Mycobacterium; Mycobacterium tuberculosis complex; Mycobacterium tuberculosis;	NCBI: 1773	4390730	99.94	0.00	Yes
NCBI Assembly	GCA_001644575.1	NCBI: cellular organisms; Bacteria; Terrabacteria group; Actinobacteria; Actinomycetia; Corynebacteriales; Mycobacteriaceae; Mycobacterium; unclassified Mycobacterium;	NCBI: 1682113	6053985	99.95	0.62	Yes

NCBI Assembly	GCA_001850605.1	NCBI: cellular organisms; Bacteria; Terrabacteria group; Actinobacteria; Actinomycetia; Corynebacteriales; Mycobacteriaceae; Mycobacterium; Mycobacterium tuberculosis complex; Mycobacterium tuberculosis;	NCBI: 1773	4403975	99.94	0.00	Yes
NCBI Assembly	GCA_001609865.1	NCBI: cellular organisms; Bacteria; Terrabacteria group; Actinobacteria; Actinomycetia; Corynebacteriales; Mycobacteriaceae; Mycobacterium; Mycobacterium avium complex (MAC); Mycobacterium avium; Mycobacterium avium subsp. hominissuis;	NCBI: 439334	5342011	99.94	0.62	Yes
NCBI Assembly	GCA_001839805.1	NCBI: cellular organisms; Bacteria; Terrabacteria group; Actinobacteria; Actinomycetia; Corynebacteriales; Mycobacteriaceae; Mycobacterium; Mycobacterium tuberculosis complex; Mycobacterium tuberculosis;	NCBI: 1773	4399470	99.94	0.00	Yes
NCBI Assembly	GCA_001610405.1	NCBI: cellular organisms; Bacteria; Terrabacteria group; Actinobacteria; Actinomycetia; Corynebacteriales; Mycobacteriaceae; Mycobacterium; Mycobacterium avium complex (MAC); Mycobacterium avium; Mycobacterium avium subsp. hominissuis;	NCBI: 439334	5120580	99.78	0.00	Yes
NCBI Assembly	GCA_001847085.1	NCBI: cellular organisms; Bacteria; Terrabacteria group; Actinobacteria; Actinomycetia; Corynebacteriales; Mycobacteriaceae; Mycobacterium; Mycobacterium tuberculosis complex; Mycobacterium tuberculosis;	NCBI: 1773	4398631	99.94	0.00	Yes
NCBI Assembly	GCA_001845545.1	NCBI: cellular organisms; Bacteria; Terrabacteria group; Actinobacteria; Actinomycetia; Corynebacteriales; Mycobacteriaceae; Mycobacterium; Mycobacterium tuberculosis complex; Mycobacterium tuberculosis;	NCBI: 1773	4399099	99.94	0.00	Yes
NCBI Assembly	GCA_001845185.1	NCBI: cellular organisms; Bacteria; Terrabacteria group; Actinobacteria; Actinomycetia; Corynebacteriales; Mycobacteriaceae; Mycobacterium; Mycobacterium tuberculosis complex; Mycobacterium tuberculosis;	NCBI: 1773	4399454	99.94	0.00	Yes
NCBI Assembly	GCA_001846785.1	NCBI: cellular organisms; Bacteria; Terrabacteria group; Actinobacteria; Actinomycetia; Corynebacteriales; Mycobacteriaceae; Mycobacterium; Mycobacterium tuberculosis complex; Mycobacterium tuberculosis;	NCBI: 1773	4403704	99.94	0.00	Yes
NCBI Assembly	GCA_001683455.1	NCBI: cellular organisms; Bacteria; Terrabacteria group; Actinobacteria; Actinomycetia; Corynebacteriales; Mycobacteriaceae; Mycobacterium; Mycobacterium avium complex (MAC); Mycobacterium avium;	NCBI: 1764	4953610	99.94	0.00	Yes
NCBI Assembly	GCA_001844875.1	NCBI: cellular organisms; Bacteria; Terrabacteria group; Actinobacteria; Actinomycetia; Corynebacteriales; Mycobacteriaceae; Mycobacterium; Mycobacterium tuberculosis complex; Mycobacterium tuberculosis;	NCBI: 1773	4404398	99.78	0.00	Yes
NCBI Assembly	GCA_001645585.1	NCBI: cellular organisms; Bacteria; Terrabacteria group; Actinobacteria; Actinomycetia; Corynebacteriales; Mycobacteriaceae; Mycobacterium; Mycobacterium tuberculosis complex; Mycobacterium tuberculosis;	NCBI: 1773	4292219	99.53	0.00	Yes
NCBI Assembly	GCA_001848935.1	NCBI: cellular organisms; Bacteria; Terrabacteria group; Actinobacteria; Actinomycetia; Corynebacteriales; Mycobacteriaceae; Mycobacterium; Mycobacterium tuberculosis complex; Mycobacterium tuberculosis;	NCBI: 1773	4384510	99.94	0.00	Yes
NCBI Assembly	GCA_001850625.1	NCBI: cellular organisms; Bacteria; Terrabacteria group; Actinobacteria; Actinomycetia; Corynebacteriales; Mycobacteriaceae; Mycobacterium; Mycobacterium tuberculosis complex; Mycobacterium tuberculosis;	NCBI: 1773	4379168	99.61	0.00	Yes
NCBI Assembly	GCA_001609895.1	NCBI: cellular organisms; Bacteria; Terrabacteria group; Actinobacteria; Actinomycetia; Corynebacteriales; Mycobacteriaceae; Mycobacterium; Mycobacterium avium complex (MAC); Mycobacterium avium; Mycobacterium avium subsp. hominissuis;	NCBI: 439334	5284904	99.94	4.09	Yes
NCBI Assembly	GCA_001610435.1	NCBI: cellular organisms; Bacteria; Terrabacteria group; Actinobacteria; Actinomycetia; Corynebacteriales; Mycobacteriaceae; Mycobacterium; Mycobacterium avium complex (MAC); Mycobacterium avium; Mycobacterium avium subsp. hominissuis;	NCBI: 439334	5472951	99.83	0.44	Yes
NCBI Assembly	GCA_001847125.1	NCBI: cellular organisms; Bacteria; Terrabacteria group; Actinobacteria; Actinomycetia; Corynebacteriales; Mycobacteriaceae; Mycobacterium; Mycobacterium tuberculosis complex; Mycobacterium tuberculosis;	NCBI: 1773	4344755	99.94	0.00	Yes
NCBI Assembly	GCA_001845795.1	NCBI: cellular organisms; Bacteria; Terrabacteria group; Actinobacteria; Actinomycetia; Corynebacteriales; Mycobacteriaceae; Mycobacterium; Mycobacterium tuberculosis complex; Mycobacterium tuberculosis;	NCBI: 1773	4404185	99.94	0.00	Yes
NCBI Assembly	GCA_001845565.1	NCBI: cellular organisms; Bacteria; Terrabacteria group; Actinobacteria; Actinomycetia; Corynebacteriales; Mycobacteriaceae; Mycobacterium; Mycobacterium tuberculosis complex; Mycobacterium tuberculosis;	NCBI: 1773	4408680	99.94	0.00	Yes

NCBI Assembly	GCA_001846795.1	NCBI: cellular organisms; Bacteria; Terrabacteria group; Actinobacteria; Actinomycetia; Corynebacteriales; Mycobacteriaceae; Mycobacterium; Mycobacterium tuberculosis complex; Mycobacterium tuberculosis;	NCBI: 1773	4379828	99.94	0.00	Yes
NCBI Assembly	GCA_001845225.1	NCBI: cellular organisms; Bacteria; Terrabacteria group; Actinobacteria; Actinomycetia; Corynebacteriales; Mycobacteriaceae; Mycobacterium; Mycobacterium tuberculosis complex; Mycobacterium tuberculosis;	NCBI: 1773	4383116	99.94	0.00	Yes
NCBI Assembly	GCA_001645625.1	NCBI: cellular organisms; Bacteria; Terrabacteria group; Actinobacteria; Actinomycetia; Corynebacteriales; Mycobacteriaceae; Mycobacterium; Mycobacterium tuberculosis complex; Mycobacterium tuberculosis;	NCBI: 1773	4322170	99.94	0.00	Yes
NCBI Assembly	GCA_001686645.1	NCBI: cellular organisms; Bacteria; Terrabacteria group; Actinobacteria; Actinomycetia; Corynebacteriales; Mycobacteriaceae; Mycobacterium; Mycobacterium avium complex (MAC); Mycobacterium intracellulare; Mycobacterium intracellulare subsp. yongonense;	NCBI: 1203599	5842189	99.50	1.73	Yes
NCBI Assembly	GCA_001848965.1	NCBI: cellular organisms; Bacteria; Terrabacteria group; Actinobacteria; Actinomycetia; Corynebacteriales; Mycobacteriaceae; Mycobacterium; Mycobacterium tuberculosis complex; Mycobacterium tuberculosis;	NCBI: 1773	4399174	99.94	0.00	Yes
NCBI Assembly	GCA_001844905.1	NCBI: cellular organisms; Bacteria; Terrabacteria group; Actinobacteria; Actinomycetia; Corynebacteriales; Mycobacteriaceae; Mycobacterium; Mycobacterium tuberculosis complex; Mycobacterium tuberculosis;	NCBI: 1773	4402039	99.94	0.00	Yes
NCBI Assembly	GCA_001850645.1	NCBI: cellular organisms; Bacteria; Terrabacteria group; Actinobacteria; Actinomycetia; Corynebacteriales; Mycobacteriaceae; Mycobacterium; Mycobacterium tuberculosis complex; Mycobacterium tuberculosis;	NCBI: 1773	4387970	99.61	0.00	Yes
NCBI Assembly	GCA_001609905.1	NCBI: cellular organisms; Bacteria; Terrabacteria group; Actinobacteria; Actinomycetia; Corynebacteriales; Mycobacteriaceae; Mycobacterium; Mycobacterium avium complex (MAC); Mycobacterium avium; Mycobacterium avium subsp. hominissuis;	NCBI: 439334	5321014	99.94	0.86	Yes
NCBI Assembly	GCA_001845825.1	NCBI: cellular organisms; Bacteria; Terrabacteria group; Actinobacteria; Actinomycetia; Corynebacteriales; Mycobacteriaceae; Mycobacterium; Mycobacterium tuberculosis complex; Mycobacterium tuberculosis;	NCBI: 1773	4376929	99.94	0.00	Yes
NCBI Assembly	GCA_001610445.1	NCBI: cellular organisms; Bacteria; Terrabacteria group; Actinobacteria; Actinomycetia; Corynebacteriales; Mycobacteriaceae; Mycobacterium; Mycobacterium avium complex (MAC); Mycobacterium avium; Mycobacterium avium subsp. hominissuis;	NCBI: 439334	5399119	98.93	2.42	Yes
NCBI Assembly	GCA_001847135.1	NCBI: cellular organisms; Bacteria; Terrabacteria group; Actinobacteria; Actinomycetia; Corynebacteriales; Mycobacteriaceae; Mycobacterium; Mycobacterium tuberculosis complex; Mycobacterium tuberculosis;	NCBI: 1773	4348527	99.94	0.00	Yes
NCBI Assembly	GCA_001648835.1	NCBI: cellular organisms; Bacteria; Terrabacteria group; Actinobacteria; Actinomycetia; Corynebacteriales; Mycobacteriaceae; Mycobacterium; Mycobacterium leprae;	NCBI: 1297124	3266556	80.51	1.00	No
NCBI Assembly	GCA_001686655.1	NCBI: cellular organisms; Bacteria; Terrabacteria group; Actinobacteria; Actinomycetia; Corynebacteriales; Mycobacteriaceae; Mycobacterium; Mycobacterium avium complex (MAC); Mycobacterium intracellulare; Mycobacterium intracellulare subsp. yongonense;	NCBI: 1203599	5586164	99.43	1.18	Yes
NCBI Assembly	GCA_001845585.1	NCBI: cellular organisms; Bacteria; Terrabacteria group; Actinobacteria; Actinomycetia; Corynebacteriales; Mycobacteriaceae; Mycobacterium; Mycobacterium tuberculosis complex; Mycobacterium tuberculosis;	NCBI: 1773	4407504	99.94	0.00	Yes
NCBI Assembly	GCA_001846815.1	NCBI: cellular organisms; Bacteria; Terrabacteria group; Actinobacteria; Actinomycetia; Corynebacteriales; Mycobacteriaceae; Mycobacterium; Mycobacterium tuberculosis complex; Mycobacterium tuberculosis;	NCBI: 1773	4406141	99.94	0.00	Yes
NCBI Assembly	GCA_001845245.1	NCBI: cellular organisms; Bacteria; Terrabacteria group; Actinobacteria; Actinomycetia; Corynebacteriales; Mycobacteriaceae; Mycobacterium; Mycobacterium tuberculosis complex; Mycobacterium tuberculosis;	NCBI: 1773	4375282	99.61	0.00	Yes
NCBI Assembly	GCA_001844925.1	NCBI: cellular organisms; Bacteria; Terrabacteria group; Actinobacteria; Actinomycetia; Corynebacteriales; Mycobacteriaceae; Mycobacterium; Mycobacterium tuberculosis complex; Mycobacterium tuberculosis;	NCBI: 1773	4397970	99.94	0.00	Yes
NCBI Assembly	GCA_001848975.1	NCBI: cellular organisms; Bacteria; Terrabacteria group; Actinobacteria; Actinomycetia; Corynebacteriales; Mycobacteriaceae; Mycobacterium; Mycobacterium tuberculosis complex; Mycobacterium tuberculosis;	NCBI: 1773	4386811	99.94	0.00	Yes

NCBI Assembly	GCA_001850665.1	NCBI: cellular organisms; Bacteria; Terrabacteria group; Actinobacteria; Actinomycetia; Corynebacteriales; Mycobacteriaceae; Mycobacterium; Mycobacterium tuberculosis complex; Mycobacterium tuberculosis;	NCBI: 1773	4350826	99.94	0.00	Yes
NCBI Assembly	GCA_001609925.1	NCBI: cellular organisms; Bacteria; Terrabacteria group; Actinobacteria; Actinomycetia; Corynebacteriales; Mycobacteriaceae; Mycobacterium; Mycobacterium avium complex (MAC); Mycobacterium avium; Mycobacterium avium subsp. hominissuis;	NCBI: 439334	5294119	99.86	3.83	Yes
NCBI Assembly	GCA_001850185.1	NCBI: cellular organisms; Bacteria; Terrabacteria group; Actinobacteria; Actinomycetia; Corynebacteriales; Mycobacteriaceae; Mycobacterium; Mycobacterium tuberculosis complex; Mycobacterium tuberculosis;	NCBI: 1773	4391005	99.94	0.00	Yes
NCBI Assembly	GCA_001845835.1	NCBI: cellular organisms; Bacteria; Terrabacteria group; Actinobacteria; Actinomycetia; Corynebacteriales; Mycobacteriaceae; Mycobacterium; Mycobacterium tuberculosis complex; Mycobacterium tuberculosis;	NCBI: 1773	4384521	99.94	0.00	Yes
NCBI Assembly	GCA_001847155.1	NCBI: cellular organisms; Bacteria; Terrabacteria group; Actinobacteria; Actinomycetia; Corynebacteriales; Mycobacteriaceae; Mycobacterium; Mycobacterium tuberculosis complex; Mycobacterium tuberculosis;	NCBI: 1773	4400281	99.94	0.00	Yes
NCBI Assembly	GCA_001686665.1	NCBI: cellular organisms; Bacteria; Terrabacteria group; Actinobacteria; Actinomycetia; Corynebacteriales; Mycobacteriaceae; Mycobacterium; Mycobacterium malmoense;	NCBI: 1780	5807656	99.44	1.54	Yes
NCBI Assembly	GCA_001650495.1	NCBI: cellular organisms; Bacteria; Terrabacteria group; Actinobacteria; Actinomycetia; Corynebacteriales; Mycobacteriaceae; Mycolicibacterium; Mycolicibacterium iranicum;	NCBI: 912594	6484789	100.00	0.74	Yes
NCBI Assembly	GCA_001845595.1	NCBI: cellular organisms; Bacteria; Terrabacteria group; Actinobacteria; Actinomycetia; Corynebacteriales; Mycobacteriaceae; Mycobacterium; Mycobacterium tuberculosis complex; Mycobacterium tuberculosis;	NCBI: 1773	4410758	99.94	0.00	Yes
NCBI Assembly	GCA_001846825.1	NCBI: cellular organisms; Bacteria; Terrabacteria group; Actinobacteria; Actinomycetia; Corynebacteriales; Mycobacteriaceae; Mycobacterium; Mycobacterium tuberculosis complex; Mycobacterium tuberculosis;	NCBI: 1773	4409642	99.94	0.00	Yes
NCBI Assembly	GCA_001845265.1	NCBI: cellular organisms; Bacteria; Terrabacteria group; Actinobacteria; Actinomycetia; Corynebacteriales; Mycobacteriaceae; Mycobacterium; Mycobacterium tuberculosis complex; Mycobacterium tuberculosis;	NCBI: 1773	4427056	99.94	0.00	Yes
NCBI Assembly	GCA_001850685.1	NCBI: cellular organisms; Bacteria; Terrabacteria group; Actinobacteria; Actinomycetia; Corynebacteriales; Mycobacteriaceae; Mycobacterium; Mycobacterium tuberculosis complex; Mycobacterium tuberculosis;	NCBI: 1773	4353338	99.94	0.00	Yes
NCBI Assembly	GCA_001844945.1	NCBI: cellular organisms; Bacteria; Terrabacteria group; Actinobacteria; Actinomycetia; Corynebacteriales; Mycobacteriaceae; Mycobacterium; Mycobacterium tuberculosis complex; Mycobacterium tuberculosis;	NCBI: 1773	4398075	99.94	0.00	Yes
NCBI Assembly	GCA_001849005.1	NCBI: cellular organisms; Bacteria; Terrabacteria group; Actinobacteria; Actinomycetia; Corynebacteriales; Mycobacteriaceae; Mycobacterium; Mycobacterium tuberculosis complex; Mycobacterium tuberculosis;	NCBI: 1773	4411088	99.94	0.00	Yes
NCBI Assembly	GCA_001609945.1	NCBI: cellular organisms; Bacteria; Terrabacteria group; Actinobacteria; Actinomycetia; Corynebacteriales; Mycobacteriaceae; Mycobacterium; Mycobacterium avium complex (MAC); Mycobacterium avium; Mycobacterium avium subsp. hominissuis;	NCBI: 439334	5366801	99.86	3.08	Yes
NCBI Assembly	GCA_001850195.1	NCBI: cellular organisms; Bacteria; Terrabacteria group; Actinobacteria; Actinomycetia; Corynebacteriales; Mycobacteriaceae; Mycobacterium; Mycobacterium tuberculosis complex; Mycobacterium tuberculosis;	NCBI: 1773	4391589	99.94	0.00	Yes
NCBI Assembly	GCA_001845865.1	NCBI: cellular organisms; Bacteria; Terrabacteria group; Actinobacteria; Actinomycetia; Corynebacteriales; Mycobacteriaceae; Mycobacterium; Mycobacterium tuberculosis complex; Mycobacterium tuberculosis;	NCBI: 1773	4386220	99.94	0.00	Yes
NCBI Assembly	GCA_001847175.1	NCBI: cellular organisms; Bacteria; Terrabacteria group; Actinobacteria; Actinomycetia; Corynebacteriales; Mycobacteriaceae; Mycobacterium; Mycobacterium tuberculosis complex; Mycobacterium tuberculosis;	NCBI: 1773	4390483	99.94	0.00	Yes
NCBI Assembly	GCA_001686675.1	NCBI: cellular organisms; Bacteria; Terrabacteria group; Actinobacteria; Actinomycetia; Corynebacteriales; Mycobacteriaceae; Mycolicibacterium; Mycolicibacterium porcinum;	NCBI: 39693	6778270	99.81	0.75	Yes

NCBI Assembly	GCA_001652545.1	NCBI: cellular organisms; Bacteria; Terrabacteria group; Actinobacteria; Actinomycetia; Corynebacteriales; Mycobacteriaceae; Mycobacterium; Mycobacterium tuberculosis complex; Mycobacterium mungi;	NCBI: 1844474	4348519	99.61	0.38	Yes
NCBI Assembly	GCA_001846865.1	NCBI: cellular organisms; Bacteria; Terrabacteria group; Actinobacteria; Actinomycetia; Corynebacteriales; Mycobacteriaceae; Mycobacterium; Mycobacterium tuberculosis complex; Mycobacterium tuberculosis;	NCBI: 1773	4399997	99.94	0.00	Yes
NCBI Assembly	GCA_001845625.1	NCBI: cellular organisms; Bacteria; Terrabacteria group; Actinobacteria; Actinomycetia; Corynebacteriales; Mycobacteriaceae; Mycobacterium; Mycobacterium tuberculosis complex; Mycobacterium tuberculosis;	NCBI: 1773	4345078	99.94	0.00	Yes
NCBI Assembly	GCA_001845275.1	NCBI: cellular organisms; Bacteria; Terrabacteria group; Actinobacteria; Actinomycetia; Corynebacteriales; Mycobacteriaceae; Mycobacterium; Mycobacterium tuberculosis complex; Mycobacterium tuberculosis;	NCBI: 1773	4387370	99.94	0.00	Yes
NCBI Assembly	GCA_001849015.1	NCBI: cellular organisms; Bacteria; Terrabacteria group; Actinobacteria; Actinomycetia; Corynebacteriales; Mycobacteriaceae; Mycobacterium; Mycobacterium tuberculosis complex; Mycobacterium tuberculosis;	NCBI: 1773	4387803	99.94	0.00	Yes
NCBI Assembly	GCA_001844955.1	NCBI: cellular organisms; Bacteria; Terrabacteria group; Actinobacteria; Actinomycetia; Corynebacteriales; Mycobacteriaceae; Mycobacterium; Mycobacterium tuberculosis complex; Mycobacterium tuberculosis;	NCBI: 1773	4392257	99.94	0.00	Yes
NCBI Assembly	GCA_001850695.1	NCBI: cellular organisms; Bacteria; Terrabacteria group; Actinobacteria; Actinomycetia; Corynebacteriales; Mycobacteriaceae; Mycobacterium; Mycobacterium tuberculosis complex; Mycobacterium tuberculosis;	NCBI: 1773	4413469	99.61	0.00	Yes
NCBI Assembly	GCA_001609975.1	NCBI: cellular organisms; Bacteria; Terrabacteria group; Actinobacteria; Actinomycetia; Corynebacteriales; Mycobacteriaceae; Mycobacterium; Mycobacterium avium complex (MAC); Mycobacterium avium; Mycobacterium avium subsp. hominissuis;	NCBI: 439334	5283354	99.94	2.03	Yes
NCBI Assembly	GCA_001845875.1	NCBI: cellular organisms; Bacteria; Terrabacteria group; Actinobacteria; Actinomycetia; Corynebacteriales; Mycobacteriaceae; Mycobacterium; Mycobacterium tuberculosis complex; Mycobacterium tuberculosis;	NCBI: 1773	4388236	99.94	0.00	Yes
NCBI Assembly	GCA_001850225.1	NCBI: cellular organisms; Bacteria; Terrabacteria group; Actinobacteria; Actinomycetia; Corynebacteriales; Mycobacteriaceae; Mycobacterium; Mycobacterium tuberculosis complex; Mycobacterium tuberculosis;	NCBI: 1773	4381577	99.61	0.00	Yes
NCBI Assembly	GCA_001686725.1	NCBI: cellular organisms; Bacteria; Terrabacteria group; Actinobacteria; Actinomycetia; Corynebacteriales; Mycobacteriaceae; Mycobacterium; Mycobacterium malmoense;	NCBI: 1780	5872488	98.72	3.80	Yes
NCBI Assembly	GCA_001653355.1	NCBI: cellular organisms; Bacteria; Terrabacteria group; Actinobacteria; Actinomycetia; Corynebacteriales; Mycobacteriaceae; Mycobacterium; Mycobacterium avium complex (MAC); Mycobacterium avium; Mycobacterium avium subsp. paratuberculosis;	NCBI: 1770	4829781	99.61	0.33	Yes
NCBI Assembly	GCA_001847205.1	NCBI: cellular organisms; Bacteria; Terrabacteria group; Actinobacteria; Actinomycetia; Corynebacteriales; Mycobacteriaceae; Mycobacterium; Mycobacterium tuberculosis complex; Mycobacterium tuberculosis;	NCBI: 1773	4384827	99.94	0.00	Yes
NCBI Assembly	GCA_001845635.1	NCBI: cellular organisms; Bacteria; Terrabacteria group; Actinobacteria; Actinomycetia; Corynebacteriales; Mycobacteriaceae; Mycobacterium; Mycobacterium tuberculosis complex; Mycobacterium tuberculosis;	NCBI: 1773	4395214	99.94	0.00	Yes
NCBI Assembly	GCA_001846885.1	NCBI: cellular organisms; Bacteria; Terrabacteria group; Actinobacteria; Actinomycetia; Corynebacteriales; Mycobacteriaceae; Mycobacterium; Mycobacterium tuberculosis complex; Mycobacterium tuberculosis;	NCBI: 1773	4413490	99.94	0.00	Yes
NCBI Assembly	GCA_001844985.1	NCBI: cellular organisms; Bacteria; Terrabacteria group; Actinobacteria; Actinomycetia; Corynebacteriales; Mycobacteriaceae; Mycobacterium; Mycobacterium tuberculosis complex; Mycobacterium tuberculosis;	NCBI: 1773	4394479	99.94	0.00	Yes
NCBI Assembly	GCA_001845305.1	NCBI: cellular organisms; Bacteria; Terrabacteria group; Actinobacteria; Actinomycetia; Corynebacteriales; Mycobacteriaceae; Mycobacterium; Mycobacterium tuberculosis complex; Mycobacterium tuberculosis;	NCBI: 1773	4406888	99.94	0.00	Yes
NCBI Assembly	GCA_001849045.1	NCBI: cellular organisms; Bacteria; Terrabacteria group; Actinobacteria; Actinomycetia; Corynebacteriales; Mycobacteriaceae; Mycobacterium; Mycobacterium tuberculosis complex; Mycobacterium tuberculosis;	NCBI: 1773	4383860	99.94	0.00	Yes

NCBI Assembly	GCA_001850705.1	NCBI: cellular organisms; Bacteria; Terrabacteria group; Actinobacteria; Actinomycetia; Corynebacteriales; Mycobacteriaceae; Mycobacterium; Mycobacterium tuberculosis complex; Mycobacterium tuberculosis;	NCBI: 1773	4390256	99.94	0.07	Yes
NCBI Assembly	GCA_001609985.1	NCBI: cellular organisms; Bacteria; Terrabacteria group; Actinobacteria; Actinomycetia; Corynebacteriales; Mycobacteriaceae; Mycobacterium; Mycobacterium avium complex (MAC); Mycobacterium avium; Mycobacterium avium subsp. hominissuis;	NCBI: 439334	5561846	99.94	2.25	Yes
NCBI Assembly	GCA_001845895.1	NCBI: cellular organisms; Bacteria; Terrabacteria group; Actinobacteria; Actinomycetia; Corynebacteriales; Mycobacteriaceae; Mycobacterium; Mycobacterium tuberculosis complex; Mycobacterium tuberculosis;	NCBI: 1773	4382829	99.94	0.00	Yes
NCBI Assembly	GCA_001850245.1	NCBI: cellular organisms; Bacteria; Terrabacteria group; Actinobacteria; Actinomycetia; Corynebacteriales; Mycobacteriaceae; Mycobacterium; Mycobacterium tuberculosis complex; Mycobacterium tuberculosis;	NCBI: 1773	4386670	99.94	0.00	Yes
NCBI Assembly	GCA_001653495.1	NCBI: cellular organisms; Bacteria; Terrabacteria group; Actinobacteria; Actinomycetia; Corynebacteriales; Mycobacteriaceae; Mycobacterium; Mycobacterium leprae;	NCBI: 1297123	3266420	80.35	1.00	No
NCBI Assembly	GCA_001686735.1	NCBI: cellular organisms; Bacteria; Terrabacteria group; Actinobacteria; Actinomycetia; Corynebacteriales; Mycobacteriaceae; Mycobacterium; Mycobacterium malmoense;	NCBI: 1780	5596373	99.69	1.40	Yes
NCBI Assembly	GCA_001847225.1	NCBI: cellular organisms; Bacteria; Terrabacteria group; Actinobacteria; Actinomycetia; Corynebacteriales; Mycobacteriaceae; Mycobacterium; Mycobacterium tuberculosis complex; Mycobacterium tuberculosis;	NCBI: 1773	4394625	99.94	0.00	Yes
NCBI Assembly	GCA_001845665.1	NCBI: cellular organisms; Bacteria; Terrabacteria group; Actinobacteria; Actinomycetia; Corynebacteriales; Mycobacteriaceae; Mycobacterium; Mycobacterium tuberculosis complex; Mycobacterium tuberculosis;	NCBI: 1773	4369458	99.94	0.00	Yes
NCBI Assembly	GCA_001846895.1	NCBI: cellular organisms; Bacteria; Terrabacteria group; Actinobacteria; Actinomycetia; Corynebacteriales; Mycobacteriaceae; Mycobacterium; Mycobacterium tuberculosis complex; Mycobacterium tuberculosis;	NCBI: 1773	4354016	99.94	0.00	Yes
NCBI Assembly	GCA_001844995.1	NCBI: cellular organisms; Bacteria; Terrabacteria group; Actinobacteria; Actinomycetia; Corynebacteriales; Mycobacteriaceae; Mycobacterium; Mycobacterium tuberculosis complex; Mycobacterium tuberculosis;	NCBI: 1773	4410366	99.94	0.00	Yes
NCBI Assembly	GCA_001845325.1	NCBI: cellular organisms; Bacteria; Terrabacteria group; Actinobacteria; Actinomycetia; Corynebacteriales; Mycobacteriaceae; Mycobacterium; Mycobacterium tuberculosis complex; Mycobacterium tuberculosis;	NCBI: 1773	4392964	99.94	0.00	Yes
NCBI Assembly	GCA_001850745.1	NCBI: cellular organisms; Bacteria; Terrabacteria group; Actinobacteria; Actinomycetia; Corynebacteriales; Mycobacteriaceae; Mycobacterium; Mycobacterium tuberculosis complex; Mycobacterium tuberculosis;	NCBI: 1773	4389915	99.94	0.00	Yes
NCBI Assembly	GCA_001849055.1	NCBI: cellular organisms; Bacteria; Terrabacteria group; Actinobacteria; Actinomycetia; Corynebacteriales; Mycobacteriaceae; Mycobacterium; Mycobacterium tuberculosis complex; Mycobacterium tuberculosis;	NCBI: 1773	4394737	99.94	0.00	Yes
NCBI Assembly	GCA_001610005.1	NCBI: cellular organisms; Bacteria; Terrabacteria group; Actinobacteria; Actinomycetia; Corynebacteriales; Mycobacteriaceae; Mycobacterium; Mycobacterium avium complex (MAC); Mycobacterium avium; Mycobacterium avium subsp. hominissuis;	NCBI: 439334	5714075	99.86	0.83	Yes
NCBI Assembly	GCA_001845925.1	NCBI: cellular organisms; Bacteria; Terrabacteria group; Actinobacteria; Actinomycetia; Corynebacteriales; Mycobacteriaceae; Mycobacterium; Mycobacterium tuberculosis complex; Mycobacterium tuberculosis;	NCBI: 1773	4384772	99.94	0.00	Yes
NCBI Assembly	GCA_001686745.1	NCBI: cellular organisms; Bacteria; Terrabacteria group; Actinobacteria; Actinomycetia; Corynebacteriales; Mycobacteriaceae; Mycobacterium; Mycobacterium malmoense;	NCBI: 1780	5571999	99.44	2.53	Yes
NCBI Assembly	GCA_001655155.1	NCBI: cellular organisms; Bacteria; Terrabacteria group; Actinobacteria; Actinomycetia; Corynebacteriales; Mycobacteriaceae; Mycobacteroides; Mycobacteroides immunogenum;	NCBI: 83262	5272148	99.50	1.82	Yes
NCBI Assembly	GCA_001850265.1	NCBI: cellular organisms; Bacteria; Terrabacteria group; Actinobacteria; Actinomycetia; Corynebacteriales; Mycobacteriaceae; Mycobacterium; Mycobacterium tuberculosis complex; Mycobacterium tuberculosis;	NCBI: 1773	4350970	99.61	0.00	Yes

NCBI Assembly	GCA_001846905.1	NCBI: cellular organisms; Bacteria; Terrabacteria group; Actinobacteria; Actinomycetia; Corynebacteriales; Mycobacteriaceae; Mycobacterium; Mycobacterium tuberculosis complex; Mycobacterium tuberculosis;	NCBI: 1773	4391768	99.94	0.00	Yes
NCBI Assembly	GCA_001845675.1	NCBI: cellular organisms; Bacteria; Terrabacteria group; Actinobacteria; Actinomycetia; Corynebacteriales; Mycobacteriaceae; Mycobacterium; Mycobacterium tuberculosis complex; Mycobacterium tuberculosis;	NCBI: 1773	4398358	99.94	0.00	Yes
NCBI Assembly	GCA_001847235.1	NCBI: cellular organisms; Bacteria; Terrabacteria group; Actinobacteria; Actinomycetia; Corynebacteriales; Mycobacteriaceae; Mycobacterium; Mycobacterium tuberculosis complex; Mycobacterium tuberculosis;	NCBI: 1773	4386739	99.94	0.00	Yes
NCBI Assembly	GCA_001850765.1	NCBI: cellular organisms; Bacteria; Terrabacteria group; Actinobacteria; Actinomycetia; Corynebacteriales; Mycobacteriaceae; Mycobacterium; Mycobacterium tuberculosis complex; Mycobacterium tuberculosis;	NCBI: 1773	4389684	99.94	0.00	Yes
NCBI Assembly	GCA_001845005.1	NCBI: cellular organisms; Bacteria; Terrabacteria group; Actinobacteria; Actinomycetia; Corynebacteriales; Mycobacteriaceae; Mycobacterium; Mycobacterium tuberculosis complex; Mycobacterium tuberculosis;	NCBI: 1773	4403186	99.94	0.00	Yes
NCBI Assembly	GCA_001845335.1	NCBI: cellular organisms; Bacteria; Terrabacteria group; Actinobacteria; Actinomycetia; Corynebacteriales; Mycobacteriaceae; Mycobacterium; Mycobacterium tuberculosis complex; Mycobacterium tuberculosis;	NCBI: 1773	4401438	99.94	0.00	Yes
NCBI Assembly	GCA_001849085.1	NCBI: cellular organisms; Bacteria; Terrabacteria group; Actinobacteria; Actinomycetia; Corynebacteriales; Mycobacteriaceae; Mycobacterium; Mycobacterium tuberculosis complex; Mycobacterium tuberculosis;	NCBI: 1773	4394000	99.94	0.00	Yes
NCBI Assembly	GCA_001610035.1	NCBI: cellular organisms; Bacteria; Terrabacteria group; Actinobacteria; Actinomycetia; Corynebacteriales; Mycobacteriaceae; Mycobacterium; Mycobacterium avium complex (MAC); Mycobacterium avium; Mycobacterium avium subsp. hominissuis;	NCBI: 439334	5214573	99.94	1.19	Yes
NCBI Assembly	GCA_001845945.1	NCBI: cellular organisms; Bacteria; Terrabacteria group; Actinobacteria; Actinomycetia; Corynebacteriales; Mycobacteriaceae; Mycobacterium; Mycobacterium tuberculosis complex; Mycobacterium tuberculosis;	NCBI: 1773	4389654	99.94	0.00	Yes
NCBI Assembly	GCA_001686755.1	NCBI: cellular organisms; Bacteria; Terrabacteria group; Actinobacteria; Actinomycetia; Corynebacteriales; Mycobacteriaceae; Mycolicibacterium; Mycolicibacterium vulneris;	NCBI: 547163	7086979	99.37	0.75	Yes
NCBI Assembly	GCA_001850275.1	NCBI: cellular organisms; Bacteria; Terrabacteria group; Actinobacteria; Actinomycetia; Corynebacteriales; Mycobacteriaceae; Mycobacterium; Mycobacterium tuberculosis complex; Mycobacterium tuberculosis;	NCBI: 1773	4386428	99.94	0.00	Yes
NCBI Assembly	GCA_001846945.1	NCBI: cellular organisms; Bacteria; Terrabacteria group; Actinobacteria; Actinomycetia; Corynebacteriales; Mycobacteriaceae; Mycobacterium; Mycobacterium tuberculosis complex; Mycobacterium tuberculosis;	NCBI: 1773	4391013	99.94	0.00	Yes
NCBI Assembly	GCA_001850775.1	NCBI: cellular organisms; Bacteria; Terrabacteria group; Actinobacteria; Actinomycetia; Corynebacteriales; Mycobacteriaceae; Mycobacterium; Mycobacterium tuberculosis complex; Mycobacterium tuberculosis;	NCBI: 1773	4400506	99.94	0.00	Yes
NCBI Assembly	GCA_001845045.1	NCBI: cellular organisms; Bacteria; Terrabacteria group; Actinobacteria; Actinomycetia; Corynebacteriales; Mycobacteriaceae; Mycobacterium; Mycobacterium tuberculosis complex; Mycobacterium tuberculosis;	NCBI: 1773	4394807	99.61	0.00	Yes
NCBI Assembly	GCA_001845705.1	NCBI: cellular organisms; Bacteria; Terrabacteria group; Actinobacteria; Actinomycetia; Corynebacteriales; Mycobacteriaceae; Mycobacterium; Mycobacterium tuberculosis complex; Mycobacterium tuberculosis;	NCBI: 1773	4419831	99.78	0.00	Yes
NCBI Assembly	GCA_001847245.1	NCBI: cellular organisms; Bacteria; Terrabacteria group; Actinobacteria; Actinomycetia; Corynebacteriales; Mycobacteriaceae; Mycobacterium; Mycobacterium tuberculosis complex; Mycobacterium tuberculosis;	NCBI: 1773	4399159	99.94	0.00	Yes
NCBI Assembly	GCA_001845345.1	NCBI: cellular organisms; Bacteria; Terrabacteria group; Actinobacteria; Actinomycetia; Corynebacteriales; Mycobacteriaceae; Mycobacterium; Mycobacterium tuberculosis complex; Mycobacterium tuberculosis;	NCBI: 1773	4408039	99.61	0.00	Yes
NCBI Assembly	GCA_001849095.1	NCBI: cellular organisms; Bacteria; Terrabacteria group; Actinobacteria; Actinomycetia; Corynebacteriales; Mycobacteriaceae; Mycobacterium; Mycobacterium tuberculosis complex; Mycobacterium tuberculosis;	NCBI: 1773	4384846	99.61	0.00	Yes

NCBI Assembly	GCA_001610055.1	NCBI: cellular organisms; Bacteria; Terrabacteria group; Actinobacteria; Actinomycetia; Corynebacteriales; Mycobacteriaceae; Mycobacterium; Mycobacterium avium complex (MAC); Mycobacterium avium; Mycobacterium avium subsp. hominissuis;	NCBI: 439334	5697130	99.94	1.76	Yes
NCBI Assembly	GCA_001663055.1	NCBI: cellular organisms; Bacteria; Terrabacteria group; Actinobacteria; Actinomycetia; Corynebacteriales; Mycobacteriaceae; Mycobacterium; Mycobacterium tuberculosis complex; Mycobacterium tuberculosis;	NCBI: 1773	4235262	99.33	0.33	Yes
NCBI Assembly	GCA_001686825.1	NCBI: cellular organisms; Bacteria; Terrabacteria group; Actinobacteria; Actinomycetia; Corynebacteriales; Mycobacteriaceae; Mycobacterium; Mycobacterium malmoense;	NCBI: 1780	5686092	99.94	0.91	Yes
NCBI Assembly	GCA_001845965.1	NCBI: cellular organisms; Bacteria; Terrabacteria group; Actinobacteria; Actinomycetia; Corynebacteriales; Mycobacteriaceae; Mycobacterium; Mycobacterium tuberculosis complex; Mycobacterium tuberculosis;	NCBI: 1773	4396736	99.94	0.00	Yes
NCBI Assembly	GCA_001850285.1	NCBI: cellular organisms; Bacteria; Terrabacteria group; Actinobacteria; Actinomycetia; Corynebacteriales; Mycobacteriaceae; Mycobacterium; Mycobacterium tuberculosis complex; Mycobacterium tuberculosis;	NCBI: 1773	4395325	99.94	0.00	Yes
NCBI Assembly	GCA_001846965.1	NCBI: cellular organisms; Bacteria; Terrabacteria group; Actinobacteria; Actinomycetia; Corynebacteriales; Mycobacteriaceae; Mycobacterium; Mycobacterium tuberculosis complex; Mycobacterium tuberculosis;	NCBI: 1773	4391039	99.94	0.00	Yes
NCBI Assembly	GCA_001845715.1	NCBI: cellular organisms; Bacteria; Terrabacteria group; Actinobacteria; Actinomycetia; Corynebacteriales; Mycobacteriaceae; Mycobacterium; Mycobacterium tuberculosis complex; Mycobacterium tuberculosis;	NCBI: 1773	4393533	99.94	0.00	Yes
NCBI Assembly	GCA_001845065.1	NCBI: cellular organisms; Bacteria; Terrabacteria group; Actinobacteria; Actinomycetia; Corynebacteriales; Mycobacteriaceae; Mycobacterium; Mycobacterium tuberculosis complex; Mycobacterium tuberculosis;	NCBI: 1773	4391050	99.94	0.00	Yes
NCBI Assembly	GCA_001850785.1	NCBI: cellular organisms; Bacteria; Terrabacteria group; Actinobacteria; Actinomycetia; Corynebacteriales; Mycobacteriaceae; Mycobacterium; Mycobacterium tuberculosis complex; Mycobacterium tuberculosis;	NCBI: 1773	4386187	99.94	0.00	Yes
NCBI Assembly	GCA_001845375.1	NCBI: cellular organisms; Bacteria; Terrabacteria group; Actinobacteria; Actinomycetia; Corynebacteriales; Mycobacteriaceae; Mycobacterium; Mycobacterium tuberculosis complex; Mycobacterium tuberculosis;	NCBI: 1773	4394217	99.94	0.00	Yes
NCBI Assembly	GCA_001847285.1	NCBI: cellular organisms; Bacteria; Terrabacteria group; Actinobacteria; Actinomycetia; Corynebacteriales; Mycobacteriaceae; Mycobacterium; Mycobacterium tuberculosis complex; Mycobacterium tuberculosis;	NCBI: 1773	4338713	99.61	0.00	Yes
NCBI Assembly	GCA_001849125.1	NCBI: cellular organisms; Bacteria; Terrabacteria group; Actinobacteria; Actinomycetia; Corynebacteriales; Mycobacteriaceae; Mycobacterium; Mycobacterium tuberculosis complex; Mycobacterium tuberculosis;	NCBI: 1773	4395104	99.61	0.00	Yes
NCBI Assembly	GCA_001610065.1	NCBI: cellular organisms; Bacteria; Terrabacteria group; Actinobacteria; Actinomycetia; Corynebacteriales; Mycobacteriaceae; Mycobacterium; Mycobacterium avium complex (MAC); Mycobacterium avium; Mycobacterium avium subsp. hominissuis;	NCBI: 439334	5078892	99.88	0.57	Yes
NCBI Assembly	GCA_001849885.1	NCBI: cellular organisms; Bacteria; Terrabacteria group; Actinobacteria; Actinomycetia; Corynebacteriales; Mycobacteriaceae; Mycobacterium; Mycobacterium tuberculosis complex; Mycobacterium tuberculosis;	NCBI: 1773	4351828	99.94	0.00	Yes
NCBI Assembly	GCA_001686835.1	NCBI: cellular organisms; Bacteria; Terrabacteria group; Actinobacteria; Actinomycetia; Corynebacteriales; Mycobacteriaceae; Mycolicibacterium; Mycolicibacterium vulneris;	NCBI: 547163	6995549	99.81	0.75	Yes
NCBI Assembly	GCA_001850325.1	NCBI: cellular organisms; Bacteria; Terrabacteria group; Actinobacteria; Actinomycetia; Corynebacteriales; Mycobacteriaceae; Mycobacterium; Mycobacterium tuberculosis complex; Mycobacterium tuberculosis;	NCBI: 1773	4405274	99.61	0.00	Yes
NCBI Assembly	GCA_001845975.1	NCBI: cellular organisms; Bacteria; Terrabacteria group; Actinobacteria; Actinomycetia; Corynebacteriales; Mycobacteriaceae; Mycobacterium; Mycobacterium tuberculosis complex; Mycobacterium tuberculosis;	NCBI: 1773	4400357	99.94	0.00	Yes
NCBI Assembly	GCA_001845745.1	NCBI: cellular organisms; Bacteria; Terrabacteria group; Actinobacteria; Actinomycetia; Corynebacteriales; Mycobacteriaceae; Mycobacterium; Mycobacterium tuberculosis complex; Mycobacterium tuberculosis;	NCBI: 1773	4395946	99.94	0.00	Yes

NCBI Assembly	GCA_001846975.1	NCBI: cellular organisms; Bacteria; Terrabacteria group; Actinobacteria; Actinomycetia; Corynebacteriales; Mycobacteriaceae; Mycobacterium; Mycobacterium tuberculosis complex; Mycobacterium tuberculosis;	NCBI: 1773	4385936	99.94	0.00	Yes
NCBI Assembly	GCA_001845075.1	NCBI: cellular organisms; Bacteria; Terrabacteria group; Actinobacteria; Actinomycetia; Corynebacteriales; Mycobacteriaceae; Mycobacterium; Mycobacterium tuberculosis complex; Mycobacterium tuberculosis;	NCBI: 1773	4410012	99.94	0.00	Yes
NCBI Assembly	GCA_001845405.1	NCBI: cellular organisms; Bacteria; Terrabacteria group; Actinobacteria; Actinomycetia; Corynebacteriales; Mycobacteriaceae; Mycobacterium; Mycobacterium tuberculosis complex; Mycobacterium tuberculosis;	NCBI: 1773	4387035	99.94	0.00	Yes
NCBI Assembly	GCA_001850825.1	NCBI: cellular organisms; Bacteria; Terrabacteria group; Actinobacteria; Actinomycetia; Corynebacteriales; Mycobacteriaceae; Mycobacterium; Mycobacterium tuberculosis complex; Mycobacterium tuberculosis;	NCBI: 1773	4387725	99.94	0.00	Yes
NCBI Assembly	GCA_001847305.1	NCBI: cellular organisms; Bacteria; Terrabacteria group; Actinobacteria; Actinomycetia; Corynebacteriales; Mycobacteriaceae; Mycobacterium; Mycobacterium tuberculosis complex; Mycobacterium tuberculosis;	NCBI: 1773	4384169	99.94	0.00	Yes
NCBI Assembly	GCA_001849145.1	NCBI: cellular organisms; Bacteria; Terrabacteria group; Actinobacteria; Actinomycetia; Corynebacteriales; Mycobacteriaceae; Mycobacterium; Mycobacterium tuberculosis complex; Mycobacterium tuberculosis;	NCBI: 1773	4394043	99.61	0.00	Yes
NCBI Assembly	GCA_001610095.1	NCBI: cellular organisms; Bacteria; Terrabacteria group; Actinobacteria; Actinomycetia; Corynebacteriales; Mycobacteriaceae; Mycobacterium; Mycobacterium avium complex (MAC); Mycobacterium avium; Mycobacterium avium subsp. hominissuis;	NCBI: 439334	5513600	99.83	0.33	Yes
NCBI Assembly	GCA_001849905.1	NCBI: cellular organisms; Bacteria; Terrabacteria group; Actinobacteria; Actinomycetia; Corynebacteriales; Mycobacteriaceae; Mycobacterium; Mycobacterium tuberculosis complex; Mycobacterium tuberculosis;	NCBI: 1773	4389190	99.94	0.00	Yes
NCBI Assembly	GCA_001686845.1	NCBI: cellular organisms; Bacteria; Terrabacteria group; Actinobacteria; Actinomycetia; Corynebacteriales; Mycobacteriaceae; Mycolicibacterium; Mycolicibacterium vulneris;	NCBI: 547163	6997564	99.81	0.75	Yes
NCBI Assembly	GCA_001850345.1	NCBI: cellular organisms; Bacteria; Terrabacteria group; Actinobacteria; Actinomycetia; Corynebacteriales; Mycobacteriaceae; Mycobacterium; Mycobacterium tuberculosis complex; Mycobacterium tuberculosis;	NCBI: 1773	4406522	99.61	0.00	Yes
NCBI Assembly	GCA_001846005.1	NCBI: cellular organisms; Bacteria; Terrabacteria group; Actinobacteria; Actinomycetia; Corynebacteriales; Mycobacteriaceae; Mycobacterium; Mycobacterium tuberculosis complex; Mycobacterium tuberculosis;	NCBI: 1773	4384730	99.94	0.00	Yes
NCBI Assembly	GCA_001845755.1	NCBI: cellular organisms; Bacteria; Terrabacteria group; Actinobacteria; Actinomycetia; Corynebacteriales; Mycobacteriaceae; Mycobacterium; Mycobacterium tuberculosis complex; Mycobacterium tuberculosis;	NCBI: 1773	4406250	99.94	0.00	Yes
NCBI Assembly	GCA_001846995.1	NCBI: cellular organisms; Bacteria; Terrabacteria group; Actinobacteria; Actinomycetia; Corynebacteriales; Mycobacteriaceae; Mycobacterium; Mycobacterium tuberculosis complex; Mycobacterium tuberculosis;	NCBI: 1773	4396994	99.61	0.00	Yes
NCBI Assembly	GCA_001850845.1	NCBI: cellular organisms; Bacteria; Terrabacteria group; Actinobacteria; Actinomycetia; Corynebacteriales; Mycobacteriaceae; Mycobacterium; Mycobacterium tuberculosis complex; Mycobacterium tuberculosis;	NCBI: 1773	4400812	99.94	0.00	Yes
NCBI Assembly	GCA_001847315.1	NCBI: cellular organisms; Bacteria; Terrabacteria group; Actinobacteria; Actinomycetia; Corynebacteriales; Mycobacteriaceae; Mycobacterium; Mycobacterium tuberculosis complex; Mycobacterium tuberculosis;	NCBI: 1773	4390770	99.94	0.00	Yes
NCBI Assembly	GCA_001845085.1	NCBI: cellular organisms; Bacteria; Terrabacteria group; Actinobacteria; Actinomycetia; Corynebacteriales; Mycobacteriaceae; Mycobacterium; Mycobacterium tuberculosis complex; Mycobacterium tuberculosis;	NCBI: 1773	4395703	99.94	0.00	Yes
NCBI Assembly	GCA_001845415.1	NCBI: cellular organisms; Bacteria; Terrabacteria group; Actinobacteria; Actinomycetia; Corynebacteriales; Mycobacteriaceae; Mycobacterium; Mycobacterium tuberculosis complex; Mycobacterium tuberculosis;	NCBI: 1773	4406014	99.94	0.00	Yes
NCBI Assembly	GCA_001610105.1	NCBI: cellular organisms; Bacteria; Terrabacteria group; Actinobacteria; Actinomycetia; Corynebacteriales; Mycobacteriaceae; Mycobacterium; Mycobacterium avium complex (MAC); Mycobacterium avium; Mycobacterium avium subsp. hominissuis;	NCBI: 439334	5180892	99.94	0.44	Yes

NCBI Assembly	GCA_001849165.1	NCBI: cellular organisms; Bacteria; Terrabacteria group; Actinobacteria; Actinomycetia; Corynebacteriales; Mycobacteriaceae; Mycobacterium; Mycobacterium tuberculosis complex; Mycobacterium tuberculosis;	NCBI: 1773	4372264	99.94	0.00	Yes
NCBI Assembly	GCA_001695755.1	NCBI: cellular organisms; Bacteria; Terrabacteria group; Actinobacteria; Actinomycetia; Corynebacteriales; Mycobacteriaceae; Mycobacterium; unclassified Mycobacterium;	NCBI: 1879023	6579322	99.56	0.67	Yes
NCBI Assembly	GCA_001849915.1	NCBI: cellular organisms; Bacteria; Terrabacteria group; Actinobacteria; Actinomycetia; Corynebacteriales; Mycobacteriaceae; Mycobacterium; Mycobacterium tuberculosis complex; Mycobacterium tuberculosis;	NCBI: 1773	4383888	99.94	0.00	Yes
NCBI Assembly	GCA_001846015.1	NCBI: cellular organisms; Bacteria; Terrabacteria group; Actinobacteria; Actinomycetia; Corynebacteriales; Mycobacteriaceae; Mycobacterium; Mycobacterium tuberculosis complex; Mycobacterium tuberculosis;	NCBI: 1773	4401021	99.94	0.00	Yes
NCBI Assembly	GCA_001850365.1	NCBI: cellular organisms; Bacteria; Terrabacteria group; Actinobacteria; Actinomycetia; Corynebacteriales; Mycobacteriaceae; Mycobacterium; Mycobacterium tuberculosis complex; Mycobacterium tuberculosis;	NCBI: 1773	4405384	99.94	0.00	Yes
NCBI Assembly	GCA_001847025.1	NCBI: cellular organisms; Bacteria; Terrabacteria group; Actinobacteria; Actinomycetia; Corynebacteriales; Mycobacteriaceae; Mycobacterium; Mycobacterium tuberculosis complex; Mycobacterium tuberculosis;	NCBI: 1773	4402610	99.94	0.00	Yes
NCBI Assembly	GCA_001845785.1	NCBI: cellular organisms; Bacteria; Terrabacteria group; Actinobacteria; Actinomycetia; Corynebacteriales; Mycobacteriaceae; Mycobacterium; Mycobacterium tuberculosis complex; Mycobacterium tuberculosis;	NCBI: 1773	4407810	99.94	0.00	Yes
NCBI Assembly	GCA_001845105.1	NCBI: cellular organisms; Bacteria; Terrabacteria group; Actinobacteria; Actinomycetia; Corynebacteriales; Mycobacteriaceae; Mycobacterium; Mycobacterium tuberculosis complex; Mycobacterium tuberculosis;	NCBI: 1773	4388656	99.94	0.00	Yes
NCBI Assembly	GCA_001847335.1	NCBI: cellular organisms; Bacteria; Terrabacteria group; Actinobacteria; Actinomycetia; Corynebacteriales; Mycobacteriaceae; Mycobacterium; Mycobacterium tuberculosis complex; Mycobacterium tuberculosis;	NCBI: 1773	4399812	99.94	0.00	Yes
NCBI Assembly	GCA_001850855.1	NCBI: cellular organisms; Bacteria; Terrabacteria group; Actinobacteria; Actinomycetia; Corynebacteriales; Mycobacteriaceae; Mycobacterium; Mycobacterium tuberculosis complex; Mycobacterium tuberculosis;	NCBI: 1773	4394684	99.94	0.00	Yes
NCBI Assembly	GCA_001845435.1	NCBI: cellular organisms; Bacteria; Terrabacteria group; Actinobacteria; Actinomycetia; Corynebacteriales; Mycobacteriaceae; Mycobacterium; Mycobacterium tuberculosis complex; Mycobacterium tuberculosis;	NCBI: 1773	4391782	99.94	0.00	Yes
NCBI Assembly	GCA_001610135.1	NCBI: cellular organisms; Bacteria; Terrabacteria group; Actinobacteria; Actinomycetia; Corynebacteriales; Mycobacteriaceae; Mycobacterium; Mycobacterium avium complex (MAC); Mycobacterium avium; Mycobacterium avium subsp. hominissuis;	NCBI: 439334	5114531	99.94	0.36	Yes
NCBI Assembly	GCA_001702435.1	NCBI: cellular organisms; Bacteria; Terrabacteria group; Actinobacteria; Actinomycetia; Corynebacteriales; Mycobacteriaceae; Mycobacterium; Mycobacterium tuberculosis complex; Mycobacterium tuberculosis;	NCBI: 1773	4406628	99.94	0.00	Yes
NCBI Assembly	GCA_001849175.1	NCBI: cellular organisms; Bacteria; Terrabacteria group; Actinobacteria; Actinomycetia; Corynebacteriales; Mycobacteriaceae; Mycobacterium; Mycobacterium tuberculosis complex; Mycobacterium tuberculosis;	NCBI: 1773	4378116	99.61	0.00	Yes
NCBI Assembly	GCA_001846035.1	NCBI: cellular organisms; Bacteria; Terrabacteria group; Actinobacteria; Actinomycetia; Corynebacteriales; Mycobacteriaceae; Mycobacterium; Mycobacterium tuberculosis complex; Mycobacterium tuberculosis;	NCBI: 1773	4397179	99.94	0.00	Yes
NCBI Assembly	GCA_001849935.1	NCBI: cellular organisms; Bacteria; Terrabacteria group; Actinobacteria; Actinomycetia; Corynebacteriales; Mycobacteriaceae; Mycobacterium; Mycobacterium tuberculosis complex; Mycobacterium tuberculosis;	NCBI: 1773	4364139	99.94	0.00	Yes
NCBI Assembly	GCA_001850375.1	NCBI: cellular organisms; Bacteria; Terrabacteria group; Actinobacteria; Actinomycetia; Corynebacteriales; Mycobacteriaceae; Mycobacterium; Mycobacterium tuberculosis complex; Mycobacterium tuberculosis;	NCBI: 1773	4388522	99.61	0.00	Yes
NCBI Assembly	GCA_001673345.1	NCBI: cellular organisms; Bacteria; Terrabacteria group; Actinobacteria; Actinomycetia; Corynebacteriales; Mycobacteriaceae; Mycobacterium; Mycobacterium asiaticum;	NCBI: 1790	5910140	99.88	0.50	Yes

NCBI Assembly	GCA_001847045.1	NCBI: cellular organisms; Bacteria; Terrabacteria group; Actinobacteria; Actinomycetia; Corynebacteriales; Mycobacteriaceae; Mycobacterium; Mycobacterium tuberculosis complex; Mycobacterium tuberculosis;	NCBI: 1773	4395518	99.94	0.00	Yes
NCBI Assembly	GCA_001847365.1	NCBI: cellular organisms; Bacteria; Terrabacteria group; Actinobacteria; Actinomycetia; Corynebacteriales; Mycobacteriaceae; Mycobacterium; Mycobacterium tuberculosis complex; Mycobacterium tuberculosis;	NCBI: 1773	4387114	99.94	0.00	Yes
NCBI Assembly	GCA_001845145.1	NCBI: cellular organisms; Bacteria; Terrabacteria group; Actinobacteria; Actinomycetia; Corynebacteriales; Mycobacteriaceae; Mycobacterium; Mycobacterium tuberculosis complex; Mycobacterium tuberculosis;	NCBI: 1773	4404718	99.94	0.00	Yes
NCBI Assembly	GCA_001845455.1	NCBI: cellular organisms; Bacteria; Terrabacteria group; Actinobacteria; Actinomycetia; Corynebacteriales; Mycobacteriaceae; Mycobacterium; Mycobacterium tuberculosis complex; Mycobacterium tuberculosis;	NCBI: 1773	4417137	99.94	0.00	Yes
NCBI Assembly	GCA_001850875.1	NCBI: cellular organisms; Bacteria; Terrabacteria group; Actinobacteria; Actinomycetia; Corynebacteriales; Mycobacteriaceae; Mycobacterium; Mycobacterium tuberculosis complex; Mycobacterium tuberculosis;	NCBI: 1773	4394698	99.94	0.00	Yes
NCBI Assembly	GCA_001848625.1	NCBI: cellular organisms; Bacteria; Terrabacteria group; Actinobacteria; Actinomycetia; Corynebacteriales; Mycobacteriaceae; Mycobacterium; Mycobacterium tuberculosis complex; Mycobacterium tuberculosis;	NCBI: 1773	4355237	99.94	0.00	Yes
NCBI Assembly	GCA_001849205.1	NCBI: cellular organisms; Bacteria; Terrabacteria group; Actinobacteria; Actinomycetia; Corynebacteriales; Mycobacteriaceae; Mycobacterium; Mycobacterium tuberculosis complex; Mycobacterium tuberculosis;	NCBI: 1773	4395890	99.94	0.00	Yes
NCBI Assembly	GCA_001673365.1	NCBI: cellular organisms; Bacteria; Terrabacteria group; Actinobacteria; Actinomycetia; Corynebacteriales; Mycobacteriaceae; Mycobacterium; Mycobacterium asiaticum;	NCBI: 1790	5888063	99.00	0.53	Yes
NCBI Assembly	GCA_001846065.1	NCBI: cellular organisms; Bacteria; Terrabacteria group; Actinobacteria; Actinomycetia; Corynebacteriales; Mycobacteriaceae; Mycobacterium; Mycobacterium tuberculosis complex; Mycobacterium tuberculosis;	NCBI: 1773	4384872	99.94	0.00	Yes
NCBI Assembly	GCA_001849965.1	NCBI: cellular organisms; Bacteria; Terrabacteria group; Actinobacteria; Actinomycetia; Corynebacteriales; Mycobacteriaceae; Mycobacterium; Mycobacterium tuberculosis complex; Mycobacterium tuberculosis;	NCBI: 1773	4376529	99.94	0.00	Yes
NCBI Assembly	GCA_001850395.1	NCBI: cellular organisms; Bacteria; Terrabacteria group; Actinobacteria; Actinomycetia; Corynebacteriales; Mycobacteriaceae; Mycobacterium; Mycobacterium tuberculosis complex; Mycobacterium tuberculosis;	NCBI: 1773	4387307	99.61	0.00	Yes
NCBI Assembly	GCA_001845485.1	NCBI: cellular organisms; Bacteria; Terrabacteria group; Actinobacteria; Actinomycetia; Corynebacteriales; Mycobacteriaceae; Mycobacterium; Mycobacterium tuberculosis complex; Mycobacterium tuberculosis;	NCBI: 1773	4403289	99.94	0.00	Yes
NCBI Assembly	GCA_001845165.1	NCBI: cellular organisms; Bacteria; Terrabacteria group; Actinobacteria; Actinomycetia; Corynebacteriales; Mycobacteriaceae; Mycobacterium; Mycobacterium tuberculosis complex; Mycobacterium tuberculosis;	NCBI: 1773	4410534	99.94	0.00	Yes
NCBI Assembly	GCA_001850905.1	NCBI: cellular organisms; Bacteria; Terrabacteria group; Actinobacteria; Actinomycetia; Corynebacteriales; Mycobacteriaceae; Mycobacterium; Mycobacterium tuberculosis complex; Mycobacterium tuberculosis;	NCBI: 1773	4382900	99.28	0.00	Yes
NCBI Assembly	GCA_001848645.1	NCBI: cellular organisms; Bacteria; Terrabacteria group; Actinobacteria; Actinomycetia; Corynebacteriales; Mycobacteriaceae; Mycobacterium; Mycobacterium tuberculosis complex; Mycobacterium tuberculosis;	NCBI: 1773	4353155	99.94	0.00	Yes
NCBI Assembly	GCA_001849215.1	NCBI: cellular organisms; Bacteria; Terrabacteria group; Actinobacteria; Actinomycetia; Corynebacteriales; Mycobacteriaceae; Mycobacterium; Mycobacterium tuberculosis complex; Mycobacterium tuberculosis;	NCBI: 1773	4401409	99.94	0.00	Yes
NCBI Assembly	GCA_001673395.1	NCBI: cellular organisms; Bacteria; Terrabacteria group; Actinobacteria; Actinomycetia; Corynebacteriales; Mycobacteriaceae; Mycobacterium; Mycobacterium tuberculosis complex; Mycobacterium tuberculosis;	NCBI: 1773	4382524	99.86	1.31	Yes
NCBI Assembly	GCA_001846075.1	NCBI: cellular organisms; Bacteria; Terrabacteria group; Actinobacteria; Actinomycetia; Corynebacteriales; Mycobacteriaceae; Mycobacterium; Mycobacterium tuberculosis complex; Mycobacterium tuberculosis;	NCBI: 1773	4409219	99.94	0.00	Yes

NCBI Assembly	GCA_001849985.1	NCBI: cellular organisms; Bacteria; Terrabacteria group; Actinobacteria; Actinomycetia; Corynebacteriales; Mycobacteriaceae; Mycobacterium; Mycobacterium tuberculosis complex; Mycobacterium tuberculosis;	NCBI: 1773	4384389	99.94	0.00	Yes
NCBI Assembly	GCA_001850405.1	NCBI: cellular organisms; Bacteria; Terrabacteria group; Actinobacteria; Actinomycetia; Corynebacteriales; Mycobacteriaceae; Mycobacterium; Mycobacterium tuberculosis complex; Mycobacterium tuberculosis;	NCBI: 1773	4382748	99.61	0.00	Yes
NCBI Assembly	GCA_001850925.1	NCBI: cellular organisms; Bacteria; Terrabacteria group; Actinobacteria; Actinomycetia; Corynebacteriales; Mycobacteriaceae; Mycobacterium; Mycobacterium tuberculosis complex; Mycobacterium tuberculosis;	NCBI: 1773	4381277	99.94	0.00	Yes
NCBI Assembly	GCA_001848655.1	NCBI: cellular organisms; Bacteria; Terrabacteria group; Actinobacteria; Actinomycetia; Corynebacteriales; Mycobacteriaceae; Mycobacterium; Mycobacterium tuberculosis complex; Mycobacterium tuberculosis;	NCBI: 1773	4394267	99.90	0.00	Yes
NCBI Assembly	GCA_001673405.1	NCBI: cellular organisms; Bacteria; Terrabacteria group; Actinobacteria; Actinomycetia; Corynebacteriales; Mycobacteriaceae; Mycobacterium; unclassified Mycobacterium;	NCBI: 1834073	5624318	100.00	0.53	Yes
NCBI Assembly	GCA_001846095.1	NCBI: cellular organisms; Bacteria; Terrabacteria group; Actinobacteria; Actinomycetia; Corynebacteriales; Mycobacteriaceae; Mycobacterium; Mycobacterium tuberculosis complex; Mycobacterium tuberculosis;	NCBI: 1773	4406607	99.94	0.00	Yes
NCBI Assembly	GCA_001849995.1	NCBI: cellular organisms; Bacteria; Terrabacteria group; Actinobacteria; Actinomycetia; Corynebacteriales; Mycobacteriaceae; Mycobacterium; Mycobacterium tuberculosis complex; Mycobacterium tuberculosis;	NCBI: 1773	4387340	99.90	0.00	Yes
NCBI Assembly	GCA_001850935.1	NCBI: cellular organisms; Bacteria; Terrabacteria group; Actinobacteria; Actinomycetia; Corynebacteriales; Mycobacteriaceae; Mycobacterium; Mycobacterium tuberculosis complex; Mycobacterium tuberculosis;	NCBI: 1773	4384347	99.94	0.00	Yes
NCBI Assembly	GCA_001850445.1	NCBI: cellular organisms; Bacteria; Terrabacteria group; Actinobacteria; Actinomycetia; Corynebacteriales; Mycobacteriaceae; Mycobacterium; Mycobacterium tuberculosis complex; Mycobacterium tuberculosis;	NCBI: 1773	4374665	99.94	0.00	Yes
NCBI Assembly	GCA_001848685.1	NCBI: cellular organisms; Bacteria; Terrabacteria group; Actinobacteria; Actinomycetia; Corynebacteriales; Mycobacteriaceae; Mycobacterium; Mycobacterium tuberculosis complex; Mycobacterium tuberculosis;	NCBI: 1773	4402992	99.61	0.00	Yes
NCBI Assembly	GCA_001673415.1	NCBI: cellular organisms; Bacteria; Terrabacteria group; Actinobacteria; Actinomycetia; Corynebacteriales; Mycobacteriaceae; Mycobacterium; unclassified Mycobacterium;	NCBI: 1834071	5581711	99.11	0.54	Yes
NCBI Assembly	GCA_001850005.1	NCBI: cellular organisms; Bacteria; Terrabacteria group; Actinobacteria; Actinomycetia; Corynebacteriales; Mycobacteriaceae; Mycobacterium; Mycobacterium tuberculosis complex; Mycobacterium tuberculosis;	NCBI: 1773	4403778	99.61	0.00	Yes
NCBI Assembly	GCA_001850965.1	NCBI: cellular organisms; Bacteria; Terrabacteria group; Actinobacteria; Actinomycetia; Corynebacteriales; Mycobacteriaceae; Mycobacterium; Mycobacterium tuberculosis complex; Mycobacterium tuberculosis;	NCBI: 1773	4407230	99.61	0.00	Yes
NCBI Assembly	GCA_001850465.1	NCBI: cellular organisms; Bacteria; Terrabacteria group; Actinobacteria; Actinomycetia; Corynebacteriales; Mycobacteriaceae; Mycobacterium; Mycobacterium tuberculosis complex; Mycobacterium tuberculosis;	NCBI: 1773	4401980	99.61	0.00	Yes
NCBI Assembly	GCA_001848695.1	NCBI: cellular organisms; Bacteria; Terrabacteria group; Actinobacteria; Actinomycetia; Corynebacteriales; Mycobacteriaceae; Mycobacterium; Mycobacterium tuberculosis complex; Mycobacterium tuberculosis;	NCBI: 1773	4392416	99.61	0.00	Yes
NCBI Assembly	GCA_001673455.1	NCBI: cellular organisms; Bacteria; Terrabacteria group; Actinobacteria; Actinomycetia; Corynebacteriales; Mycobacteriaceae; Mycobacterium; Mycobacterium simiae complex; Mycobacterium kubicae;	NCBI: 120959	5534339	99.28	1.03	Yes
NCBI Assembly	GCA_001850045.1	NCBI: cellular organisms; Bacteria; Terrabacteria group; Actinobacteria; Actinomycetia; Corynebacteriales; Mycobacteriaceae; Mycobacterium; Mycobacterium tuberculosis complex; Mycobacterium tuberculosis;	NCBI: 1773	4349355	99.61	0.00	Yes
NCBI Assembly	GCA_001850975.1	NCBI: cellular organisms; Bacteria; Terrabacteria group; Actinobacteria; Actinomycetia; Corynebacteriales; Mycobacteriaceae; Mycobacterium; Mycobacterium tuberculosis complex; Mycobacterium tuberculosis;	NCBI: 1773	4420205	99.61	0.00	Yes

NCBI Assembly	GCA_001850475.1	NCBI: cellular organisms; Bacteria; Terrabacteria group; Actinobacteria; Actinomycetia; Corynebacteriales; Mycobacteriaceae; Mycobacterium; Mycobacterium tuberculosis complex; Mycobacterium tuberculosis;	NCBI: 1773	4384748	99.61	0.00	Yes
NCBI Assembly	GCA_001848725.1	NCBI: cellular organisms; Bacteria; Terrabacteria group; Actinobacteria; Actinomycetia; Corynebacteriales; Mycobacteriaceae; Mycobacterium; Mycobacterium tuberculosis complex; Mycobacterium tuberculosis;	NCBI: 1773	4391687	99.94	0.00	Yes
NCBI Assembly	GCA_001673475.1	NCBI: cellular organisms; Bacteria; Terrabacteria group; Actinobacteria; Actinomycetia; Corynebacteriales; Mycobacteriaceae; Mycobacterium; Mycobacterium gordonae;	NCBI: 1778	6746188	99.67	2.05	Yes
NCBI Assembly	GCA_001850055.1	NCBI: cellular organisms; Bacteria; Terrabacteria group; Actinobacteria; Actinomycetia; Corynebacteriales; Mycobacteriaceae; Mycobacterium; Mycobacterium tuberculosis complex; Mycobacterium tuberculosis;	NCBI: 1773	4392184	99.61	0.00	Yes
NCBI Assembly	GCA_001851005.1	NCBI: cellular organisms; Bacteria; Terrabacteria group; Actinobacteria; Actinomycetia; Corynebacteriales; Mycobacteriaceae; Mycobacterium; Mycobacterium tuberculosis complex; Mycobacterium tuberculosis;	NCBI: 1773	4349358	99.94	0.00	Yes
NCBI Assembly	GCA_001848745.1	NCBI: cellular organisms; Bacteria; Terrabacteria group; Actinobacteria; Actinomycetia; Corynebacteriales; Mycobacteriaceae; Mycobacterium; Mycobacterium tuberculosis complex; Mycobacterium tuberculosis;	NCBI: 1773	4409810	99.61	0.00	Yes
NCBI Assembly	GCA_001673485.1	NCBI: cellular organisms; Bacteria; Terrabacteria group; Actinobacteria; Actinomycetia; Corynebacteriales; Mycobacteriaceae; Mycolicibacterium; Mycolicibacterium fortuitum;	NCBI: 1766	6745646	99.43	2.75	Yes
NCBI Assembly	GCA_001850085.1	NCBI: cellular organisms; Bacteria; Terrabacteria group; Actinobacteria; Actinomycetia; Corynebacteriales; Mycobacteriaceae; Mycobacterium; Mycobacterium tuberculosis complex; Mycobacterium tuberculosis;	NCBI: 1773	4380312	99.94	0.00	Yes
NCBI Assembly	GCA_001851025.1	NCBI: cellular organisms; Bacteria; Terrabacteria group; Actinobacteria; Actinomycetia; Corynebacteriales; Mycobacteriaceae; Mycobacterium; Mycobacterium tuberculosis complex; Mycobacterium tuberculosis;	NCBI: 1773	4398456	99.94	0.00	Yes
NCBI Assembly	GCA_001848765.1	NCBI: cellular organisms; Bacteria; Terrabacteria group; Actinobacteria; Actinomycetia; Corynebacteriales; Mycobacteriaceae; Mycobacterium; Mycobacterium tuberculosis complex; Mycobacterium tuberculosis;	NCBI: 1773	4389514	99.61	0.00	Yes
NCBI Assembly	GCA_001673505.1	NCBI: cellular organisms; Bacteria; Terrabacteria group; Actinobacteria; Actinomycetia; Corynebacteriales; Mycobacteriaceae; Mycobacterium; Mycobacterium avium complex (MAC); Mycobacterium colombiense;	NCBI: 339268	5944999	99.28	0.98	Yes
NCBI Assembly	GCA_001850105.1	NCBI: cellular organisms; Bacteria; Terrabacteria group; Actinobacteria; Actinomycetia; Corynebacteriales; Mycobacteriaceae; Mycobacterium; Mycobacterium tuberculosis complex; Mycobacterium tuberculosis;	NCBI: 1773	4394319	99.61	0.00	Yes
NCBI Assembly	GCA_001851035.1	NCBI: cellular organisms; Bacteria; Terrabacteria group; Actinobacteria; Actinomycetia; Corynebacteriales; Mycobacteriaceae; Mycobacterium; Mycobacterium tuberculosis complex; Mycobacterium tuberculosis;	NCBI: 1773	4406223	99.61	0.00	Yes
NCBI Assembly	GCA_001673535.1	NCBI: cellular organisms; Bacteria; Terrabacteria group; Actinobacteria; Actinomycetia; Corynebacteriales; Mycobacteriaceae; Mycobacterium; unclassified Mycobacterium;	NCBI: 1834066	5613730	99.94	1.56	Yes
NCBI Assembly	GCA_001848785.1	NCBI: cellular organisms; Bacteria; Terrabacteria group; Actinobacteria; Actinomycetia; Corynebacteriales; Mycobacteriaceae; Mycobacterium; Mycobacterium tuberculosis complex; Mycobacterium tuberculosis;	NCBI: 1773	4401778	99.61	0.00	Yes
NCBI Assembly	GCA_001851045.1	NCBI: cellular organisms; Bacteria; Terrabacteria group; Actinobacteria; Actinomycetia; Corynebacteriales; Mycobacteriaceae; Mycobacterium; Mycobacterium tuberculosis complex; Mycobacterium tuberculosis;	NCBI: 1773	4381948	99.94	0.00	Yes
NCBI Assembly	GCA_001673555.1	NCBI: cellular organisms; Bacteria; Terrabacteria group; Actinobacteria; Actinomycetia; Corynebacteriales; Mycobacteriaceae; Mycobacterium; unclassified Mycobacterium;	NCBI: 1834070	5355294	99.17	4.45	Yes
NCBI Assembly	GCA_001850125.1	NCBI: cellular organisms; Bacteria; Terrabacteria group; Actinobacteria; Actinomycetia; Corynebacteriales; Mycobacteriaceae; Mycobacterium; Mycobacterium tuberculosis complex; Mycobacterium tuberculosis;	NCBI: 1773	4384867	99.94	0.00	Yes

NCBI Assembly	GCA_001848805.1	NCBI: cellular organisms; Bacteria; Terrabacteria group; Actinobacteria; Actinomycetia; Corynebacteriales; Mycobacteriaceae; Mycobacterium; Mycobacterium tuberculosis complex; Mycobacterium tuberculosis;	NCBI: 1773	4392175	99.94	0.00	Yes
NCBI Assembly	GCA_001673575.1	NCBI: cellular organisms; Bacteria; Terrabacteria group; Actinobacteria; Actinomycetia; Corynebacteriales; Mycobacteriaceae; Mycolicibacter; Mycolicibacter heraklionensis;	NCBI: 512402	4718971	100.00	1.63	Yes
NCBI Assembly	GCA_001851085.1	NCBI: cellular organisms; Bacteria; Terrabacteria group; Actinobacteria; Actinomycetia; Corynebacteriales; Mycobacteriaceae; Mycobacterium; Mycobacterium tuberculosis complex; Mycobacterium tuberculosis;	NCBI: 1773	4359603	99.61	0.00	Yes
NCBI Assembly	GCA_001850135.1	NCBI: cellular organisms; Bacteria; Terrabacteria group; Actinobacteria; Actinomycetia; Corynebacteriales; Mycobacteriaceae; Mycobacterium; Mycobacterium tuberculosis complex; Mycobacterium tuberculosis;	NCBI: 1773	4391494	99.94	0.33	Yes
NCBI Assembly	GCA_001848815.1	NCBI: cellular organisms; Bacteria; Terrabacteria group; Actinobacteria; Actinomycetia; Corynebacteriales; Mycobacteriaceae; Mycobacterium; Mycobacterium tuberculosis complex; Mycobacterium tuberculosis;	NCBI: 1773	4350752	99.94	0.00	Yes
NCBI Assembly	GCA_001673615.1	NCBI: cellular organisms; Bacteria; Terrabacteria group; Actinobacteria; Actinomycetia; Corynebacteriales; Mycobacteriaceae; Mycobacterium; unclassified Mycobacterium;	NCBI: 1834074	5652048	99.11	1.85	Yes
NCBI Assembly	GCA_001848845.1	NCBI: cellular organisms; Bacteria; Terrabacteria group; Actinobacteria; Actinomycetia; Corynebacteriales; Mycobacteriaceae; Mycobacterium; Mycobacterium tuberculosis complex; Mycobacterium tuberculosis;	NCBI: 1773	4387286	99.61	0.00	Yes
NCBI Assembly	GCA_001851105.1	NCBI: cellular organisms; Bacteria; Terrabacteria group; Actinobacteria; Actinomycetia; Corynebacteriales; Mycobacteriaceae; Mycobacterium; Mycobacterium tuberculosis complex; Mycobacterium tuberculosis;	NCBI: 1773	4392982	99.94	0.00	Yes
NCBI Assembly	GCA_001850155.1	NCBI: cellular organisms; Bacteria; Terrabacteria group; Actinobacteria; Actinomycetia; Corynebacteriales; Mycobacteriaceae; Mycobacterium; Mycobacterium tuberculosis complex; Mycobacterium tuberculosis;	NCBI: 1773	4401526	99.94	0.00	Yes
NCBI Assembly	GCA_001673635.1	NCBI: cellular organisms; Bacteria; Terrabacteria group; Actinobacteria; Actinomycetia; Corynebacteriales; Mycobacteriaceae; Mycobacterium; Mycobacterium asiaticum;	NCBI: 1790	5772654	99.94	0.62	Yes
NCBI Assembly	GCA_001848855.1	NCBI: cellular organisms; Bacteria; Terrabacteria group; Actinobacteria; Actinomycetia; Corynebacteriales; Mycobacteriaceae; Mycobacterium; Mycobacterium tuberculosis complex; Mycobacterium tuberculosis;	NCBI: 1773	4389520	99.94	0.00	Yes
NCBI Assembly	GCA_001675225.1	NCBI: cellular organisms; Bacteria; Terrabacteria group; Actinobacteria; Actinomycetia; Corynebacteriales; Mycobacteriaceae; Mycobacterium; Mycobacterium gordonae;	NCBI: 1778	7764157	99.96	4.32	Yes
NCBI Assembly	GCA_001848885.1	NCBI: cellular organisms; Bacteria; Terrabacteria group; Actinobacteria; Actinomycetia; Corynebacteriales; Mycobacteriaceae; Mycobacterium; Mycobacterium tuberculosis complex; Mycobacterium tuberculosis;	NCBI: 1773	4403443	99.94	0.00	Yes
NCBI Assembly	GCA_001848905.1	NCBI: cellular organisms; Bacteria; Terrabacteria group; Actinobacteria; Actinomycetia; Corynebacteriales; Mycobacteriaceae; Mycobacterium; Mycobacterium tuberculosis complex; Mycobacterium tuberculosis;	NCBI: 1773	4410064	99.61	0.00	Yes
NCBI Assembly	GCA_001954235.1	NCBI: cellular organisms; Bacteria; Terrabacteria group; Actinobacteria; Actinomycetia; Corynebacteriales; Mycobacteriaceae; Mycolicibacterium; Mycolicibacterium conceptionense;	NCBI: 451644	6443650	100.00	0.44	Yes
NCBI Assembly	GCA_001998075.1	NCBI: cellular organisms; Bacteria; Terrabacteria group; Actinobacteria; Actinomycetia; Corynebacteriales; Mycobacteriaceae; Mycobacterium; Mycobacterium tuberculosis complex; Mycobacterium tuberculosis;	NCBI: 1334051	4411532	99.94	0.00	Yes
NCBI Assembly	GCA_001945955.1	NCBI: cellular organisms; Bacteria; Terrabacteria group; Actinobacteria; Actinomycetia; Corynebacteriales; Mycobacteriaceae; Mycobacterium; Mycobacterium tuberculosis complex; Mycobacterium tuberculosis;	NCBI: 1773	4385691	99.94	0.00	Yes
NCBI Assembly	GCA_002130515.1	NCBI: cellular organisms; Bacteria; Terrabacteria group; Actinobacteria; Actinomycetia; Corynebacteriales; Mycobacteriaceae; Mycobacterium; Mycobacterium tuberculosis complex; Mycobacterium tuberculosis;	NCBI: 1773	4397046	99.94	0.00	Yes

NCBI Assembly	GCA_001949095.1	NCBI: cellular organisms; Bacteria; Terrabacteria group; Actinobacteria; Actinomycetia; Corynebacteriales; Mycobacteriaceae; Mycobacterium; Mycobacterium tuberculosis complex; Mycobacterium tuberculosis;	NCBI: 1773	4394484	99.94	0.00	Yes
NCBI Assembly	GCA_001948465.1	NCBI: cellular organisms; Bacteria; Terrabacteria group; Actinobacteria; Actinomycetia; Corynebacteriales; Mycobacteriaceae; Mycobacterium; Mycobacterium tuberculosis complex; Mycobacterium tuberculosis;	NCBI: 1773	4415182	99.94	0.00	Yes
NCBI Assembly	GCA_001944065.1	NCBI: cellular organisms; Bacteria; Terrabacteria group; Actinobacteria; Actinomycetia; Corynebacteriales; Mycobacteriaceae; Mycobacterium; Mycobacterium tuberculosis complex; Mycobacterium tuberculosis;	NCBI: 1773	4383687	99.94	0.00	Yes
NCBI Assembly	GCA_002128935.1	NCBI: cellular organisms; Bacteria; Terrabacteria group; Actinobacteria; Actinomycetia; Corynebacteriales; Mycobacteriaceae; Mycobacterium; Mycobacterium tuberculosis complex; Mycobacterium tuberculosis;	NCBI: 1773	4391284	99.94	0.00	Yes
NCBI Assembly	GCA_001947205.1	NCBI: cellular organisms; Bacteria; Terrabacteria group; Actinobacteria; Actinomycetia; Corynebacteriales; Mycobacteriaceae; Mycobacterium; Mycobacterium tuberculosis complex; Mycobacterium tuberculosis;	NCBI: 1773	4407237	99.94	0.00	Yes
NCBI Assembly	GCA_002447575.1	NCBI: cellular organisms; Bacteria; Terrabacteria group; Actinobacteria; Actinomycetia; Corynebacteriales; Mycobacteriaceae; Mycobacterium; Mycobacterium tuberculosis complex; Mycobacterium tuberculosis;	NCBI: 1773	4411365	99.94	0.00	Yes
NCBI Assembly	GCA_002086635.1	NCBI: cellular organisms; Bacteria; Terrabacteria group; Actinobacteria; Actinomycetia; Corynebacteriales; Mycobacteriaceae; Mycobacterium; Mycobacterium alsense;	NCBI: 324058	5692845	99.67	0.92	Yes
NCBI Assembly	GCA_002742165.1	NCBI: cellular organisms; Bacteria; Terrabacteria group; Actinobacteria; Actinomycetia; Corynebacteriales; Mycobacteriaceae; Mycobacterium; Mycobacterium celatum;	NCBI: 28045	4773526	100.00	0.55	Yes
NCBI Assembly	GCA_001954275.1	NCBI: cellular organisms; Bacteria; Terrabacteria group; Actinobacteria; Actinomycetia; Corynebacteriales; Mycobacteriaceae; Mycobacterium; unclassified Mycobacterium;	NCBI: 1834160	5000399	99.53	0.12	Yes
NCBI Assembly	GCA_001998105.1	NCBI: cellular organisms; Bacteria; Terrabacteria group; Actinobacteria; Actinomycetia; Corynebacteriales; Mycobacteriaceae; Mycobacterium; Mycobacterium tuberculosis complex; Mycobacterium tuberculosis;	NCBI: 1334069	4411532	99.94	0.00	Yes
NCBI Assembly	GCA_001946005.1	NCBI: cellular organisms; Bacteria; Terrabacteria group; Actinobacteria; Actinomycetia; Corynebacteriales; Mycobacteriaceae; Mycobacterium; Mycobacterium tuberculosis complex; Mycobacterium tuberculosis;	NCBI: 1773	4396379	99.94	0.00	Yes
NCBI Assembly	GCA_001953975.1	NCBI: cellular organisms; Bacteria; Terrabacteria group; Actinobacteria; Actinomycetia; Corynebacteriales; Mycobacteriaceae; Mycobacterium; unclassified Mycobacterium;	NCBI: 1834161	5560160	99.38	0.09	Yes
NCBI Assembly	GCA_001944075.1	NCBI: cellular organisms; Bacteria; Terrabacteria group; Actinobacteria; Actinomycetia; Corynebacteriales; Mycobacteriaceae; Mycobacterium; Mycobacterium tuberculosis complex; Mycobacterium tuberculosis;	NCBI: 1773	4377433	99.94	0.00	Yes
NCBI Assembly	GCA_001947215.1	NCBI: cellular organisms; Bacteria; Terrabacteria group; Actinobacteria; Actinomycetia; Corynebacteriales; Mycobacteriaceae; Mycobacterium; Mycobacterium tuberculosis complex; Mycobacterium tuberculosis;	NCBI: 1773	4384571	99.94	0.00	Yes
NCBI Assembly	GCA_002128945.1	NCBI: cellular organisms; Bacteria; Terrabacteria group; Actinobacteria; Actinomycetia; Corynebacteriales; Mycobacteriaceae; Mycobacterium; Mycobacterium tuberculosis complex; Mycobacterium tuberculosis;	NCBI: 1773	4409069	99.94	0.00	Yes
NCBI Assembly	GCA_002130525.1	NCBI: cellular organisms; Bacteria; Terrabacteria group; Actinobacteria; Actinomycetia; Corynebacteriales; Mycobacteriaceae; Mycobacterium; Mycobacterium tuberculosis complex; Mycobacterium tuberculosis;	NCBI: 1773	4396913	99.94	0.00	Yes
NCBI Assembly	GCA_001948475.1	NCBI: cellular organisms; Bacteria; Terrabacteria group; Actinobacteria; Actinomycetia; Corynebacteriales; Mycobacteriaceae; Mycobacterium; Mycobacterium tuberculosis complex; Mycobacterium tuberculosis;	NCBI: 1773	4395114	99.94	0.00	Yes
NCBI Assembly	GCA_002447595.1	NCBI: cellular organisms; Bacteria; Terrabacteria group; Actinobacteria; Actinomycetia; Corynebacteriales; Mycobacteriaceae; Mycobacterium; Mycobacterium tuberculosis complex; Mycobacterium tuberculosis;	NCBI: 1773	4411186	99.94	0.00	Yes

NCBI Assembly	GCA_002762075.1	NCBI: cellular organisms; Bacteria; Terrabacteria group; Actinobacteria; Actinomycetia; Corynebacteriales; Mycobacteriaceae; Mycobacterium; unclassified Mycobacterium;	NCBI: 2051551	5999427	100.00	0.00	Yes
NCBI Assembly	GCA_001998145.1	NCBI: cellular organisms; Bacteria; Terrabacteria group; Actinobacteria; Actinomycetia; Corynebacteriales; Mycobacteriaceae; Mycobacterium; Mycobacterium tuberculosis complex; Mycobacterium tuberculosis;	NCBI: 1334060	4411532	99.94	0.00	Yes
NCBI Assembly	GCA_001946015.1	NCBI: cellular organisms; Bacteria; Terrabacteria group; Actinobacteria; Actinomycetia; Corynebacteriales; Mycobacteriaceae; Mycobacterium; Mycobacterium tuberculosis complex; Mycobacterium tuberculosis;	NCBI: 1773	4381618	99.94	0.07	Yes
NCBI Assembly	GCA_001956335.1	NCBI: cellular organisms; Bacteria; Terrabacteria group; Actinobacteria; Actinomycetia; Corynebacteriales; Mycobacteriaceae; Mycobacterium; Mycobacterium avium complex (MAC); Mycobacterium avium; Mycobacterium avium subsp. hominissuis;	NCBI: 439334	5210894	99.36	0.73	Yes
NCBI Assembly	GCA_001953985.1	NCBI: cellular organisms; Bacteria; Terrabacteria group; Actinobacteria; Actinomycetia; Corynebacteriales; Mycobacteriaceae; Mycobacterium; Mycobacterium avium complex (MAC); Mycobacterium colombiense;	NCBI: 339268	5609019	99.78	0.61	Yes
NCBI Assembly	GCA_001944125.1	NCBI: cellular organisms; Bacteria; Terrabacteria group; Actinobacteria; Actinomycetia; Corynebacteriales; Mycobacteriaceae; Mycobacterium; Mycobacterium tuberculosis complex; Mycobacterium tuberculosis;	NCBI: 1773	4390403	99.94	0.00	Yes
NCBI Assembly	GCA_001947265.1	NCBI: cellular organisms; Bacteria; Terrabacteria group; Actinobacteria; Actinomycetia; Corynebacteriales; Mycobacteriaceae; Mycobacterium; Mycobacterium tuberculosis complex; Mycobacterium tuberculosis;	NCBI: 1773	4372450	99.94	0.00	Yes
NCBI Assembly	GCA_001948525.1	NCBI: cellular organisms; Bacteria; Terrabacteria group; Actinobacteria; Actinomycetia; Corynebacteriales; Mycobacteriaceae; Mycobacterium; Mycobacterium tuberculosis complex; Mycobacterium tuberculosis;	NCBI: 1773	4401631	99.94	0.00	Yes
NCBI Assembly	GCA_002128985.1	NCBI: cellular organisms; Bacteria; Terrabacteria group; Actinobacteria; Actinomycetia; Corynebacteriales; Mycobacteriaceae; Mycobacterium; Mycobacterium tuberculosis complex; Mycobacterium tuberculosis;	NCBI: 1773	4386421	99.94	0.00	Yes
NCBI Assembly	GCA_002130565.1	NCBI: cellular organisms; Bacteria; Terrabacteria group; Actinobacteria; Actinomycetia; Corynebacteriales; Mycobacteriaceae; Mycobacterium; Mycobacterium tuberculosis complex; Mycobacterium tuberculosis;	NCBI: 1773	4390438	99.94	0.00	Yes
NCBI Assembly	GCA_002447615.1	NCBI: cellular organisms; Bacteria; Terrabacteria group; Actinobacteria; Actinomycetia; Corynebacteriales; Mycobacteriaceae; Mycobacterium; Mycobacterium tuberculosis complex; Mycobacterium tuberculosis;	NCBI: 1773	4411340	99.94	0.00	Yes
NCBI Assembly	GCA_002086675.1	NCBI: cellular organisms; Bacteria; Terrabacteria group; Actinobacteria; Actinomycetia; Corynebacteriales; Mycobacteriaceae; Mycobacterium; Mycobacterium persicum;	NCBI: 1487726	6172209	99.83	0.67	Yes
NCBI Assembly	GCA_002798385.1	NCBI: cellular organisms; Bacteria; Terrabacteria group; Actinobacteria; Actinomycetia; Corynebacteriales; Mycobacteriaceae; Mycolicibacterium; Mycolicibacterium goodii;	NCBI: 134601	6621508	99.81	1.95	Yes
NCBI Assembly	GCA_001998175.1	NCBI: cellular organisms; Bacteria; Terrabacteria group; Actinobacteria; Actinomycetia; Corynebacteriales; Mycobacteriaceae; Mycobacterium; Mycobacterium tuberculosis complex; Mycobacterium tuberculosis;	NCBI: 1334058	4411532	99.94	0.00	Yes
NCBI Assembly	GCA_001946025.1	NCBI: cellular organisms; Bacteria; Terrabacteria group; Actinobacteria; Actinomycetia; Corynebacteriales; Mycobacteriaceae; Mycobacterium; Mycobacterium tuberculosis complex; Mycobacterium tuberculosis;	NCBI: 1773	4414632	99.94	0.00	Yes
NCBI Assembly	GCA_001947275.1	NCBI: cellular organisms; Bacteria; Terrabacteria group; Actinobacteria; Actinomycetia; Corynebacteriales; Mycobacteriaceae; Mycobacterium; Mycobacterium tuberculosis complex; Mycobacterium tuberculosis;	NCBI: 1773	4383583	99.94	0.00	Yes
NCBI Assembly	GCA_001944135.1	NCBI: cellular organisms; Bacteria; Terrabacteria group; Actinobacteria; Actinomycetia; Corynebacteriales; Mycobacteriaceae; Mycobacterium; Mycobacterium tuberculosis complex; Mycobacterium tuberculosis;	NCBI: 1773	4388808	99.94	0.00	Yes
NCBI Assembly	GCA_001953995.1	NCBI: cellular organisms; Bacteria; Terrabacteria group; Actinobacteria; Actinomycetia; Corynebacteriales; Mycobacteriaceae; Mycolicibacter; Mycolicibacter heraklionensis;	NCBI: 512402	4401465	100.00	0.15	Yes

NCBI Assembly	GCA_001969025.1	NCBI: cellular organisms; Bacteria; Terrabacteria group; Actinobacteria; Actinomycetia; Corynebacteriales; Mycobacteriaceae; Mycobacterium; Mycobacterium tuberculosis complex; Mycobacterium tuberculosis;	NCBI: 1773	4417418	99.94	0.00	Yes
NCBI Assembly	GCA_002130585.1	NCBI: cellular organisms; Bacteria; Terrabacteria group; Actinobacteria; Actinomycetia; Corynebacteriales; Mycobacteriaceae; Mycobacterium; Mycobacterium tuberculosis complex; Mycobacterium tuberculosis;	NCBI: 1773	4383961	99.94	0.00	Yes
NCBI Assembly	GCA_001948535.1	NCBI: cellular organisms; Bacteria; Terrabacteria group; Actinobacteria; Actinomycetia; Corynebacteriales; Mycobacteriaceae; Mycobacterium; Mycobacterium tuberculosis complex; Mycobacterium tuberculosis;	NCBI: 1773	4405574	99.94	0.00	Yes
NCBI Assembly	GCA_002129005.1	NCBI: cellular organisms; Bacteria; Terrabacteria group; Actinobacteria; Actinomycetia; Corynebacteriales; Mycobacteriaceae; Mycobacterium; Mycobacterium tuberculosis complex; Mycobacterium tuberculosis;	NCBI: 1773	4384970	99.94	0.00	Yes
NCBI Assembly	GCA_002447635.1	NCBI: cellular organisms; Bacteria; Terrabacteria group; Actinobacteria; Actinomycetia; Corynebacteriales; Mycobacteriaceae; Mycobacterium; Mycobacterium tuberculosis complex; Mycobacterium tuberculosis;	NCBI: 1773	4411321	99.94	0.00	Yes
NCBI Assembly	GCA_002086695.1	NCBI: cellular organisms; Bacteria; Terrabacteria group; Actinobacteria; Actinomycetia; Corynebacteriales; Mycobacteriaceae; Mycolicibacterium; Mycolicibacterium rhodesiae;	NCBI: 36814	6009003	100.00	0.44	Yes
NCBI Assembly	GCA_001998225.1	NCBI: cellular organisms; Bacteria; Terrabacteria group; Actinobacteria; Actinomycetia; Corynebacteriales; Mycobacteriaceae; Mycobacterium; Mycobacterium tuberculosis complex; Mycobacterium tuberculosis;	NCBI: 1334061	4411532	99.94	0.00	Yes
NCBI Assembly	GCA_002799625.1	NCBI: cellular organisms; Bacteria; Terrabacteria group; Actinobacteria; Actinomycetia; Corynebacteriales; Mycobacteriaceae; Mycobacteroides; Mycobacteroides abscessus; Mycobacteroides abscessus subsp. massiliense;	NCBI: 1962118	4777861	100.00	0.00	Yes
NCBI Assembly	GCA_001944145.1	NCBI: cellular organisms; Bacteria; Terrabacteria group; Actinobacteria; Actinomycetia; Corynebacteriales; Mycobacteriaceae; Mycobacterium; Mycobacterium tuberculosis complex; Mycobacterium tuberculosis;	NCBI: 1773	4381320	99.94	0.00	Yes
NCBI Assembly	GCA_001946035.1	NCBI: cellular organisms; Bacteria; Terrabacteria group; Actinobacteria; Actinomycetia; Corynebacteriales; Mycobacteriaceae; Mycobacterium; Mycobacterium tuberculosis complex; Mycobacterium tuberculosis;	NCBI: 1773	4383671	99.94	0.00	Yes
NCBI Assembly	GCA_001947285.1	NCBI: cellular organisms; Bacteria; Terrabacteria group; Actinobacteria; Actinomycetia; Corynebacteriales; Mycobacteriaceae; Mycobacterium; Mycobacterium tuberculosis complex; Mycobacterium tuberculosis;	NCBI: 1773	4393523	99.94	0.00	Yes
NCBI Assembly	GCA_001954035.1	NCBI: cellular organisms; Bacteria; Terrabacteria group; Actinobacteria; Actinomycetia; Corynebacteriales; Mycobacteriaceae; Mycolicibacterium; Mycolicibacterium fortuitum;	NCBI: 1766	6210935	99.81	0.13	Yes
NCBI Assembly	GCA_001948545.1	NCBI: cellular organisms; Bacteria; Terrabacteria group; Actinobacteria; Actinomycetia; Corynebacteriales; Mycobacteriaceae; Mycobacterium; Mycobacterium tuberculosis complex; Mycobacterium tuberculosis;	NCBI: 1773	4379835	99.94	0.00	Yes
NCBI Assembly	GCA_002130605.1	NCBI: cellular organisms; Bacteria; Terrabacteria group; Actinobacteria; Actinomycetia; Corynebacteriales; Mycobacteriaceae; Mycobacterium; Mycobacterium tuberculosis complex; Mycobacterium tuberculosis;	NCBI: 1773	4388056	99.94	0.00	Yes
NCBI Assembly	GCA_001969035.1	NCBI: cellular organisms; Bacteria; Terrabacteria group; Actinobacteria; Actinomycetia; Corynebacteriales; Mycobacteriaceae; Mycobacterium; Mycobacterium tuberculosis complex; Mycobacterium tuberculosis;	NCBI: 1773	4416025	99.94	0.00	Yes
NCBI Assembly	GCA_002129015.1	NCBI: cellular organisms; Bacteria; Terrabacteria group; Actinobacteria; Actinomycetia; Corynebacteriales; Mycobacteriaceae; Mycobacterium; Mycobacterium tuberculosis complex; Mycobacterium tuberculosis;	NCBI: 1773	4392832	99.94	0.00	Yes
NCBI Assembly	GCA_002086715.1	NCBI: cellular organisms; Bacteria; Terrabacteria group; Actinobacteria; Actinomycetia; Corynebacteriales; Mycobacteriaceae; Mycobacteroides; Mycobacteroides saopaulense;	NCBI: 1578165	5200319	99.39	0.08	Yes
NCBI Assembly	GCA_002447655.1	NCBI: cellular organisms; Bacteria; Terrabacteria group; Actinobacteria; Actinomycetia; Corynebacteriales; Mycobacteriaceae; Mycobacterium; Mycobacterium tuberculosis complex; Mycobacterium tuberculosis;	NCBI: 1773	4411331	99.94	0.00	Yes

NCBI Assembly	GCA_001998265.1	NCBI: cellular organisms; Bacteria; Terrabacteria group; Actinobacteria; Actinomycetia; Corynebacteriales; Mycobacteriaceae; Mycobacterium; Mycobacterium tuberculosis complex; Mycobacterium tuberculosis;	NCBI: 1334064	4411532	99.94	0.00	Yes
NCBI Assembly	GCA_002799645.1	NCBI: cellular organisms; Bacteria; Terrabacteria group; Actinobacteria; Actinomycetia; Corynebacteriales; Mycobacteriaceae; Mycobacteroides; Mycobacteroides abscessus; Mycobacteroides abscessus subsp. massiliense;	NCBI: 1962118	4899077	100.00	0.00	Yes
NCBI Assembly	GCA_001954045.1	NCBI: cellular organisms; Bacteria; Terrabacteria group; Actinobacteria; Actinomycetia; Corynebacteriales; Mycobacteriaceae; Mycobacterium; unclassified Mycobacterium;	NCBI: 1834162	5223609	99.53	3.15	Yes
NCBI Assembly	GCA_001946085.1	NCBI: cellular organisms; Bacteria; Terrabacteria group; Actinobacteria; Actinomycetia; Corynebacteriales; Mycobacteriaceae; Mycobacterium; Mycobacterium tuberculosis complex; Mycobacterium tuberculosis;	NCBI: 1773	4391742	99.94	0.07	Yes
NCBI Assembly	GCA_001944155.1	NCBI: cellular organisms; Bacteria; Terrabacteria group; Actinobacteria; Actinomycetia; Corynebacteriales; Mycobacteriaceae; Mycobacterium; Mycobacterium tuberculosis complex; Mycobacterium tuberculosis;	NCBI: 1773	4389720	99.94	0.00	Yes
NCBI Assembly	GCA_001984215.1	NCBI: cellular organisms; Bacteria; Terrabacteria group; Actinobacteria; Actinomycetia; Corynebacteriales; Mycobacteriaceae; Mycobacterium; unclassified Mycobacterium;	NCBI: 1936029	6829132	99.10	1.76	Yes
NCBI Assembly	GCA_001948565.1	NCBI: cellular organisms; Bacteria; Terrabacteria group; Actinobacteria; Actinomycetia; Corynebacteriales; Mycobacteriaceae; Mycobacterium; Mycobacterium tuberculosis complex; Mycobacterium tuberculosis;	NCBI: 1773	4393137	99.94	0.00	Yes
NCBI Assembly	GCA_001947305.1	NCBI: cellular organisms; Bacteria; Terrabacteria group; Actinobacteria; Actinomycetia; Corynebacteriales; Mycobacteriaceae; Mycobacterium; Mycobacterium tuberculosis complex; Mycobacterium tuberculosis;	NCBI: 1773	4387834	99.94	0.00	Yes
NCBI Assembly	GCA_002130615.1	NCBI: cellular organisms; Bacteria; Terrabacteria group; Actinobacteria; Actinomycetia; Corynebacteriales; Mycobacteriaceae; Mycobacterium; Mycobacterium tuberculosis complex; Mycobacterium tuberculosis;	NCBI: 1773	4386094	99.94	0.00	Yes
NCBI Assembly	GCA_002129025.1	NCBI: cellular organisms; Bacteria; Terrabacteria group; Actinobacteria; Actinomycetia; Corynebacteriales; Mycobacteriaceae; Mycobacterium; Mycobacterium tuberculosis complex; Mycobacterium tuberculosis;	NCBI: 1773	4356660	99.28	0.00	Yes
NCBI Assembly	GCA_001998365.1	NCBI: cellular organisms; Bacteria; Terrabacteria group; Actinobacteria; Actinomycetia; Corynebacteriales; Mycobacteriaceae; Mycobacterium; Mycobacterium tuberculosis complex; Mycobacterium tuberculosis;	NCBI: 1334048	4411532	99.94	0.00	Yes
NCBI Assembly	GCA_002086735.1	NCBI: cellular organisms; Bacteria; Terrabacteria group; Actinobacteria; Actinomycetia; Corynebacteriales; Mycobacteriaceae; Mycobacterium; Mycobacterium scrofulaceum;	NCBI: 1783	6170273	99.94	0.67	Yes
NCBI Assembly	GCA_002447675.1	NCBI: cellular organisms; Bacteria; Terrabacteria group; Actinobacteria; Actinomycetia; Corynebacteriales; Mycobacteriaceae; Mycobacterium; Mycobacterium tuberculosis complex; Mycobacterium tuberculosis;	NCBI: 1773	4411326	99.94	0.00	Yes
NCBI Assembly	GCA_001954055.1	NCBI: cellular organisms; Bacteria; Terrabacteria group; Actinobacteria; Actinomycetia; Corynebacteriales; Mycobacteriaceae; Mycobacterium; Mycobacterium avium complex (MAC); Mycobacterium colombiense;	NCBI: 339268	5553384	99.61	0.53	Yes
NCBI Assembly	GCA_001997425.1	NCBI: cellular organisms; Bacteria; Terrabacteria group; Actinobacteria; Actinomycetia; Corynebacteriales; Mycobacteriaceae; Mycobacterium; Mycobacterium tuberculosis complex; Mycobacterium tuberculosis;	NCBI: 1334055	4411532	99.94	0.00	Yes
NCBI Assembly	GCA_001944205.1	NCBI: cellular organisms; Bacteria; Terrabacteria group; Actinobacteria; Actinomycetia; Corynebacteriales; Mycobacteriaceae; Mycobacterium; Mycobacterium tuberculosis complex; Mycobacterium tuberculosis;	NCBI: 1773	4389465	99.94	0.00	Yes
NCBI Assembly	GCA_002799655.1	NCBI: cellular organisms; Bacteria; Terrabacteria group; Actinobacteria; Actinomycetia; Corynebacteriales; Mycobacteriaceae; Mycobacteroides; Mycobacteroides abscessus; Mycobacteroides abscessus subsp. massiliense;	NCBI: 1962118	4874447	100.00	0.00	Yes
NCBI Assembly	GCA_001946095.1	NCBI: cellular organisms; Bacteria; Terrabacteria group; Actinobacteria; Actinomycetia; Corynebacteriales; Mycobacteriaceae; Mycobacterium; Mycobacterium tuberculosis complex; Mycobacterium tuberculosis;	NCBI: 1773	4362662	99.94	0.00	Yes

NCBI Assembly	GCA_001947345.1	NCBI: cellular organisms; Bacteria; Terrabacteria group; Actinobacteria; Actinomycetia; Corynebacteriales; Mycobacteriaceae; Mycobacterium; Mycobacterium tuberculosis complex; Mycobacterium tuberculosis;	NCBI: 1773	4390690	99.94	0.00	Yes
NCBI Assembly	GCA_001948605.1	NCBI: cellular organisms; Bacteria; Terrabacteria group; Actinobacteria; Actinomycetia; Corynebacteriales; Mycobacteriaceae; Mycobacterium; Mycobacterium tuberculosis complex; Mycobacterium tuberculosis;	NCBI: 1773	4386981	99.94	0.00	Yes
NCBI Assembly	GCA_002130625.1	NCBI: cellular organisms; Bacteria; Terrabacteria group; Actinobacteria; Actinomycetia; Corynebacteriales; Mycobacteriaceae; Mycobacterium; Mycobacterium tuberculosis complex; Mycobacterium tuberculosis;	NCBI: 1773	4396003	99.94	0.00	Yes
NCBI Assembly	GCA_002129065.1	NCBI: cellular organisms; Bacteria; Terrabacteria group; Actinobacteria; Actinomycetia; Corynebacteriales; Mycobacteriaceae; Mycobacterium; Mycobacterium tuberculosis complex; Mycobacterium tuberculosis;	NCBI: 1773	4352860	99.94	0.00	Yes
NCBI Assembly	GCA_001998425.1	NCBI: cellular organisms; Bacteria; Terrabacteria group; Actinobacteria; Actinomycetia; Corynebacteriales; Mycobacteriaceae; Mycobacterium; Mycobacterium tuberculosis complex; Mycobacterium tuberculosis;	NCBI: 1334052	4411532	99.94	0.00	Yes
NCBI Assembly	GCA_002086755.1	NCBI: cellular organisms; Bacteria; Terrabacteria group; Actinobacteria; Actinomycetia; Corynebacteriales; Mycobacteriaceae; Mycobacterium; Mycobacterium shinjukuense;	NCBI: 398694	4409896	96.98	0.44	Yes
NCBI Assembly	GCA_002447695.1	NCBI: cellular organisms; Bacteria; Terrabacteria group; Actinobacteria; Actinomycetia; Corynebacteriales; Mycobacteriaceae; Mycobacterium; Mycobacterium tuberculosis complex; Mycobacterium tuberculosis;	NCBI: 1773	4411379	99.94	0.00	Yes
NCBI Assembly	GCA_001997465.1	NCBI: cellular organisms; Bacteria; Terrabacteria group; Actinobacteria; Actinomycetia; Corynebacteriales; Mycobacteriaceae; Mycobacterium; Mycobacterium tuberculosis complex; Mycobacterium tuberculosis;	NCBI: 1334076	4411532	99.94	0.00	Yes
NCBI Assembly	GCA_001954075.1	NCBI: cellular organisms; Bacteria; Terrabacteria group; Actinobacteria; Actinomycetia; Corynebacteriales; Mycobacteriaceae; Mycobacterium; Mycobacterium avium complex (MAC); Mycobacterium colombiense;	NCBI: 339268	5561014	99.28	0.90	Yes
NCBI Assembly	GCA_001944215.1	NCBI: cellular organisms; Bacteria; Terrabacteria group; Actinobacteria; Actinomycetia; Corynebacteriales; Mycobacteriaceae; Mycobacterium; Mycobacterium tuberculosis complex; Mycobacterium tuberculosis;	NCBI: 1773	4348149	99.94	0.00	Yes
NCBI Assembly	GCA_001947355.1	NCBI: cellular organisms; Bacteria; Terrabacteria group; Actinobacteria; Actinomycetia; Corynebacteriales; Mycobacteriaceae; Mycobacterium; Mycobacterium tuberculosis complex; Mycobacterium tuberculosis;	NCBI: 1773	4374340	99.94	0.00	Yes
NCBI Assembly	GCA_001946105.1	NCBI: cellular organisms; Bacteria; Terrabacteria group; Actinobacteria; Actinomycetia; Corynebacteriales; Mycobacteriaceae; Mycobacterium; Mycobacterium tuberculosis complex; Mycobacterium tuberculosis;	NCBI: 1773	4388534	99.94	0.00	Yes
NCBI Assembly	GCA_001948615.1	NCBI: cellular organisms; Bacteria; Terrabacteria group; Actinobacteria; Actinomycetia; Corynebacteriales; Mycobacteriaceae; Mycobacterium; Mycobacterium tuberculosis complex; Mycobacterium tuberculosis;	NCBI: 1773	4388727	99.94	0.00	Yes
NCBI Assembly	GCA_002130665.1	NCBI: cellular organisms; Bacteria; Terrabacteria group; Actinobacteria; Actinomycetia; Corynebacteriales; Mycobacteriaceae; Mycobacterium; Mycobacterium tuberculosis complex; Mycobacterium tuberculosis;	NCBI: 1773	4393887	99.94	0.00	Yes
NCBI Assembly	GCA_002799685.1	NCBI: cellular organisms; Bacteria; Terrabacteria group; Actinobacteria; Actinomycetia; Corynebacteriales; Mycobacteriaceae; Mycobacteroides; Mycobacteroides abscessus; Mycobacteroides abscessus subsp. massiliense;	NCBI: 1962118	5022810	100.00	0.11	Yes
NCBI Assembly	GCA_002129085.1	NCBI: cellular organisms; Bacteria; Terrabacteria group; Actinobacteria; Actinomycetia; Corynebacteriales; Mycobacteriaceae; Mycobacterium; Mycobacterium tuberculosis complex; Mycobacterium tuberculosis;	NCBI: 1773	4382808	99.78	0.00	Yes
NCBI Assembly	GCA_001998455.1	NCBI: cellular organisms; Bacteria; Terrabacteria group; Actinobacteria; Actinomycetia; Corynebacteriales; Mycobacteriaceae; Mycobacterium; Mycobacterium tuberculosis complex; Mycobacterium tuberculosis;	NCBI: 1334050	4411532	99.94	0.00	Yes
NCBI Assembly	GCA_002086775.1	NCBI: cellular organisms; Bacteria; Terrabacteria group; Actinobacteria; Actinomycetia; Corynebacteriales; Mycobacteriaceae; Mycobacterium; Mycobacterium avium complex (MAC); Mycobacterium timonense;	NCBI: 701043	6009989	99.17	0.50	Yes

NCBI Assembly	GCA_001997495.1	NCBI: cellular organisms; Bacteria; Terrabacteria group; Actinobacteria; Actinomycetia; Corynebacteriales; Mycobacteriaceae; Mycobacterium; Mycobacterium tuberculosis complex; Mycobacterium tuberculosis;	NCBI: 1334077	4411532	99.94	0.00	Yes
NCBI Assembly	GCA_001954115.1	NCBI: cellular organisms; Bacteria; Terrabacteria group; Actinobacteria; Actinomycetia; Corynebacteriales; Mycobacteriaceae; Mycobacterium; Mycobacterium avium complex (MAC); Mycobacterium colombiense;	NCBI: 339268	5780297	99.94	0.69	Yes
NCBI Assembly	GCA_001944225.1	NCBI: cellular organisms; Bacteria; Terrabacteria group; Actinobacteria; Actinomycetia; Corynebacteriales; Mycobacteriaceae; Mycobacterium; Mycobacterium tuberculosis complex; Mycobacterium tuberculosis;	NCBI: 1773	4379918	99.94	0.00	Yes
NCBI Assembly	GCA_002447715.1	NCBI: cellular organisms; Bacteria; Terrabacteria group; Actinobacteria; Actinomycetia; Corynebacteriales; Mycobacteriaceae; Mycobacterium; Mycobacterium tuberculosis complex; Mycobacterium tuberculosis;	NCBI: 1773	4411392	99.94	0.00	Yes
NCBI Assembly	GCA_001947365.1	NCBI: cellular organisms; Bacteria; Terrabacteria group; Actinobacteria; Actinomycetia; Corynebacteriales; Mycobacteriaceae; Mycobacterium; Mycobacterium tuberculosis complex; Mycobacterium tuberculosis;	NCBI: 1773	4383335	99.94	0.00	Yes
NCBI Assembly	GCA_001948625.1	NCBI: cellular organisms; Bacteria; Terrabacteria group; Actinobacteria; Actinomycetia; Corynebacteriales; Mycobacteriaceae; Mycobacterium; Mycobacterium tuberculosis complex; Mycobacterium tuberculosis;	NCBI: 1773	4387990	99.94	0.00	Yes
NCBI Assembly	GCA_001946115.1	NCBI: cellular organisms; Bacteria; Terrabacteria group; Actinobacteria; Actinomycetia; Corynebacteriales; Mycobacteriaceae; Mycobacterium; Mycobacterium tuberculosis complex; Mycobacterium tuberculosis;	NCBI: 1773	4390707	99.94	0.00	Yes
NCBI Assembly	GCA_002129095.1	NCBI: cellular organisms; Bacteria; Terrabacteria group; Actinobacteria; Actinomycetia; Corynebacteriales; Mycobacteriaceae; Mycobacterium; Mycobacterium tuberculosis complex; Mycobacterium tuberculosis;	NCBI: 1773	4381623	99.94	0.00	Yes
NCBI Assembly	GCA_002130685.1	NCBI: cellular organisms; Bacteria; Terrabacteria group; Actinobacteria; Actinomycetia; Corynebacteriales; Mycobacteriaceae; Mycobacterium; Mycobacterium tuberculosis complex; Mycobacterium tuberculosis;	NCBI: 1773	4392782	99.94	0.00	Yes
NCBI Assembly	GCA_001998485.1	NCBI: cellular organisms; Bacteria; Terrabacteria group; Actinobacteria; Actinomycetia; Corynebacteriales; Mycobacteriaceae; Mycobacterium; Mycobacterium tuberculosis complex; Mycobacterium tuberculosis;	NCBI: 1334074	4411532	99.94	0.00	Yes
NCBI Assembly	GCA_002799695.1	NCBI: cellular organisms; Bacteria; Terrabacteria group; Actinobacteria; Actinomycetia; Corynebacteriales; Mycobacteriaceae; Mycobacteroides; Mycobacteroides abscessus; Mycobacteroides abscessus subsp. massiliense;	NCBI: 1962118	4939897	100.00	0.00	Yes
NCBI Assembly	GCA_001997525.1	NCBI: cellular organisms; Bacteria; Terrabacteria group; Actinobacteria; Actinomycetia; Corynebacteriales; Mycobacteriaceae; Mycobacterium; Mycobacterium tuberculosis complex; Mycobacterium tuberculosis;	NCBI: 1334078	4411532	99.94	0.00	Yes
NCBI Assembly	GCA_002086795.1	NCBI: cellular organisms; Bacteria; Terrabacteria group; Actinobacteria; Actinomycetia; Corynebacteriales; Mycobacteriaceae; Mycolicibacterium; Mycolicibacterium tusciae;	NCBI: 75922	6162880	100.00	0.45	Yes
NCBI Assembly	GCA_001944235.1	NCBI: cellular organisms; Bacteria; Terrabacteria group; Actinobacteria; Actinomycetia; Corynebacteriales; Mycobacteriaceae; Mycobacterium; Mycobacterium tuberculosis complex; Mycobacterium tuberculosis;	NCBI: 1773	4390793	99.94	0.00	Yes
NCBI Assembly	GCA_001947385.1	NCBI: cellular organisms; Bacteria; Terrabacteria group; Actinobacteria; Actinomycetia; Corynebacteriales; Mycobacteriaceae; Mycobacterium; Mycobacterium tuberculosis complex; Mycobacterium tuberculosis;	NCBI: 1773	4378782	99.94	0.00	Yes
NCBI Assembly	GCA_001954125.1	NCBI: cellular organisms; Bacteria; Terrabacteria group; Actinobacteria; Actinomycetia; Corynebacteriales; Mycobacteriaceae; Mycolicibacterium; Mycolicibacterium conceptionense;	NCBI: 451644	6634698	99.94	0.00	Yes
NCBI Assembly	GCA_001948645.1	NCBI: cellular organisms; Bacteria; Terrabacteria group; Actinobacteria; Actinomycetia; Corynebacteriales; Mycobacteriaceae; Mycobacterium; Mycobacterium tuberculosis complex; Mycobacterium tuberculosis;	NCBI: 1773	4393083	99.94	0.00	Yes
NCBI Assembly	GCA_002447735.1	NCBI: cellular organisms; Bacteria; Terrabacteria group; Actinobacteria; Actinomycetia; Corynebacteriales; Mycobacteriaceae; Mycobacterium; Mycobacterium tuberculosis complex; Mycobacterium tuberculosis;	NCBI: 1773	4411494	99.94	0.00	Yes

NCBI Assembly	GCA_002130695.1	NCBI: cellular organisms; Bacteria; Terrabacteria group; Actinobacteria; Actinomycetia; Corynebacteriales; Mycobacteriaceae; Mycobacterium; Mycobacterium tuberculosis complex; Mycobacterium tuberculosis;	NCBI: 1773	4401029	99.94	0.33	Yes
NCBI Assembly	GCA_001946165.1	NCBI: cellular organisms; Bacteria; Terrabacteria group; Actinobacteria; Actinomycetia; Corynebacteriales; Mycobacteriaceae; Mycobacterium; Mycobacterium tuberculosis complex; Mycobacterium tuberculosis;	NCBI: 1773	4414337	99.94	0.00	Yes
NCBI Assembly	GCA_001998545.1	NCBI: cellular organisms; Bacteria; Terrabacteria group; Actinobacteria; Actinomycetia; Corynebacteriales; Mycobacteriaceae; Mycobacterium; Mycobacterium tuberculosis complex; Mycobacterium tuberculosis;	NCBI: 1334054	4411532	99.94	0.00	Yes
NCBI Assembly	GCA_002129105.1	NCBI: cellular organisms; Bacteria; Terrabacteria group; Actinobacteria; Actinomycetia; Corynebacteriales; Mycobacteriaceae; Mycobacterium; Mycobacterium tuberculosis complex; Mycobacterium tuberculosis;	NCBI: 1773	4414917	99.94	0.00	Yes
NCBI Assembly	GCA_002799715.1	NCBI: cellular organisms; Bacteria; Terrabacteria group; Actinobacteria; Actinomycetia; Corynebacteriales; Mycobacteriaceae; Mycobacteroides; Mycobacteroides abscessus; Mycobacteroides abscessus subsp. massiliense;	NCBI: 1962118	4740939	100.00	0.00	Yes
NCBI Assembly	GCA_001997545.1	NCBI: cellular organisms; Bacteria; Terrabacteria group; Actinobacteria; Actinomycetia; Corynebacteriales; Mycobacteriaceae; Mycobacterium; Mycobacterium tuberculosis complex; Mycobacterium tuberculosis;	NCBI: 1334079	4411532	99.94	0.00	Yes
NCBI Assembly	GCA_001944285.1	NCBI: cellular organisms; Bacteria; Terrabacteria group; Actinobacteria; Actinomycetia; Corynebacteriales; Mycobacteriaceae; Mycobacterium; Mycobacterium tuberculosis complex; Mycobacterium tuberculosis;	NCBI: 1773	4386928	99.61	0.00	Yes
NCBI Assembly	GCA_001954135.1	NCBI: cellular organisms; Bacteria; Terrabacteria group; Actinobacteria; Actinomycetia; Corynebacteriales; Mycobacteriaceae; Mycobacterium; unclassified Mycobacterium;	NCBI: 1834154	5857883	99.75	0.38	Yes
NCBI Assembly	GCA_001948685.1	NCBI: cellular organisms; Bacteria; Terrabacteria group; Actinobacteria; Actinomycetia; Corynebacteriales; Mycobacteriaceae; Mycobacterium; Mycobacterium tuberculosis complex; Mycobacterium tuberculosis;	NCBI: 1773	4367302	99.94	0.00	Yes
NCBI Assembly	GCA_001947425.1	NCBI: cellular organisms; Bacteria; Terrabacteria group; Actinobacteria; Actinomycetia; Corynebacteriales; Mycobacteriaceae; Mycobacterium; Mycobacterium tuberculosis complex; Mycobacterium tuberculosis;	NCBI: 1773	4346670	99.94	0.00	Yes
NCBI Assembly	GCA_002086815.1	NCBI: cellular organisms; Bacteria; Terrabacteria group; Actinobacteria; Actinomycetia; Corynebacteriales; Mycobacteriaceae; Mycolicibacterium; Mycolicibacterium parafortuitum;	NCBI: 39692	6136108	99.91	0.00	Yes
NCBI Assembly	GCA_001946175.1	NCBI: cellular organisms; Bacteria; Terrabacteria group; Actinobacteria; Actinomycetia; Corynebacteriales; Mycobacteriaceae; Mycobacterium; Mycobacterium tuberculosis complex; Mycobacterium tuberculosis;	NCBI: 1773	4400565	99.94	0.00	Yes
NCBI Assembly	GCA_001998585.1	NCBI: cellular organisms; Bacteria; Terrabacteria group; Actinobacteria; Actinomycetia; Corynebacteriales; Mycobacteriaceae; Mycobacterium; Mycobacterium tuberculosis complex; Mycobacterium tuberculosis;	NCBI: 1334057	4411532	99.61	0.00	Yes
NCBI Assembly	GCA_002447755.1	NCBI: cellular organisms; Bacteria; Terrabacteria group; Actinobacteria; Actinomycetia; Corynebacteriales; Mycobacteriaceae; Mycobacterium; Mycobacterium tuberculosis complex; Mycobacterium tuberculosis;	NCBI: 1773	4411335	99.94	0.00	Yes
NCBI Assembly	GCA_002130705.1	NCBI: cellular organisms; Bacteria; Terrabacteria group; Actinobacteria; Actinomycetia; Corynebacteriales; Mycobacteriaceae; Mycobacterium; Mycobacterium tuberculosis complex; Mycobacterium tuberculosis;	NCBI: 1773	4404039	99.94	0.00	Yes
NCBI Assembly	GCA_002129145.1	NCBI: cellular organisms; Bacteria; Terrabacteria group; Actinobacteria; Actinomycetia; Corynebacteriales; Mycobacteriaceae; Mycobacterium; Mycobacterium tuberculosis complex; Mycobacterium tuberculosis;	NCBI: 1773	4367770	99.94	0.00	Yes
NCBI Assembly	GCA_002799725.1	NCBI: cellular organisms; Bacteria; Terrabacteria group; Actinobacteria; Actinomycetia; Corynebacteriales; Mycobacteriaceae; Mycobacteroides; Mycobacteroides abscessus; Mycobacteroides abscessus subsp. massiliense;	NCBI: 1962118	5032717	100.00	0.00	Yes
NCBI Assembly	GCA_001997565.1	NCBI: cellular organisms; Bacteria; Terrabacteria group; Actinobacteria; Actinomycetia; Corynebacteriales; Mycobacteriaceae; Mycobacterium; Mycobacterium tuberculosis complex; Mycobacterium tuberculosis;	NCBI: 1334080	4411532	99.94	0.00	Yes

NCBI Assembly	GCA_001947435.1	NCBI: cellular organisms; Bacteria; Terrabacteria group; Actinobacteria; Actinomycetia; Corynebacteriales; Mycobacteriaceae; Mycobacterium; Mycobacterium tuberculosis complex; Mycobacterium tuberculosis;	NCBI: 1773	4373163	99.94	0.00	Yes
NCBI Assembly	GCA_001944295.1	NCBI: cellular organisms; Bacteria; Terrabacteria group; Actinobacteria; Actinomycetia; Corynebacteriales; Mycobacteriaceae; Mycobacterium; Mycobacterium tuberculosis complex; Mycobacterium tuberculosis;	NCBI: 1773	4390482	99.94	0.00	Yes
NCBI Assembly	GCA_001954145.1	NCBI: cellular organisms; Bacteria; Terrabacteria group; Actinobacteria; Actinomycetia; Corynebacteriales; Mycobacteriaceae; Mycolicibacterium; Mycolicibacterium fortuitum;	NCBI: 1766	6642199	99.81	0.72	Yes
NCBI Assembly	GCA_001948695.1	NCBI: cellular organisms; Bacteria; Terrabacteria group; Actinobacteria; Actinomycetia; Corynebacteriales; Mycobacteriaceae; Mycobacterium; Mycobacterium tuberculosis complex; Mycobacterium tuberculosis;	NCBI: 1773	4389051	99.94	0.00	Yes
NCBI Assembly	GCA_001998645.1	NCBI: cellular organisms; Bacteria; Terrabacteria group; Actinobacteria; Actinomycetia; Corynebacteriales; Mycobacteriaceae; Mycobacterium; Mycobacterium tuberculosis complex; Mycobacterium tuberculosis;	NCBI: 1334073	4411532	99.94	0.00	Yes
NCBI Assembly	GCA_001946185.1	NCBI: cellular organisms; Bacteria; Terrabacteria group; Actinobacteria; Actinomycetia; Corynebacteriales; Mycobacteriaceae; Mycobacterium; Mycobacterium tuberculosis complex; Mycobacterium tuberculosis;	NCBI: 1773	4376596	99.94	0.00	Yes
NCBI Assembly	GCA_002086835.1	NCBI: cellular organisms; Bacteria; Terrabacteria group; Actinobacteria; Actinomycetia; Corynebacteriales; Mycobacteriaceae; Mycolicibacterium; Mycolicibacterium porcinum;	NCBI: 39693	6960672	99.81	1.39	Yes
NCBI Assembly	GCA_002130745.1	NCBI: cellular organisms; Bacteria; Terrabacteria group; Actinobacteria; Actinomycetia; Corynebacteriales; Mycobacteriaceae; Mycobacterium; Mycobacterium tuberculosis complex; Mycobacterium tuberculosis;	NCBI: 1773	4406258	99.94	0.00	Yes
NCBI Assembly	GCA_002447775.1	NCBI: cellular organisms; Bacteria; Terrabacteria group; Actinobacteria; Actinomycetia; Corynebacteriales; Mycobacteriaceae; Mycobacterium; Mycobacterium tuberculosis complex; Mycobacterium tuberculosis;	NCBI: 1773	4411449	99.94	0.00	Yes
NCBI Assembly	GCA_002129165.1	NCBI: cellular organisms; Bacteria; Terrabacteria group; Actinobacteria; Actinomycetia; Corynebacteriales; Mycobacteriaceae; Mycobacterium; Mycobacterium tuberculosis complex; Mycobacterium tuberculosis;	NCBI: 1773	4385817	99.94	0.00	Yes
NCBI Assembly	GCA_001997585.1	NCBI: cellular organisms; Bacteria; Terrabacteria group; Actinobacteria; Actinomycetia; Corynebacteriales; Mycobacteriaceae; Mycobacterium; Mycobacterium tuberculosis complex; Mycobacterium tuberculosis;	NCBI: 1334081	4411532	99.78	0.00	Yes
NCBI Assembly	GCA_002799765.1	NCBI: cellular organisms; Bacteria; Terrabacteria group; Actinobacteria; Actinomycetia; Corynebacteriales; Mycobacteriaceae; Mycobacteroides; Mycobacteroides abscessus; Mycobacteroides abscessus subsp. massiliense;	NCBI: 1962118	4841633	100.00	0.00	Yes
NCBI Assembly	GCA_001954195.1	NCBI: cellular organisms; Bacteria; Terrabacteria group; Actinobacteria; Actinomycetia; Corynebacteriales; Mycobacteriaceae; Mycobacterium; unclassified Mycobacterium;	NCBI: 1834158	4922713	99.61	0.22	Yes
NCBI Assembly	GCA_001948705.1	NCBI: cellular organisms; Bacteria; Terrabacteria group; Actinobacteria; Actinomycetia; Corynebacteriales; Mycobacteriaceae; Mycobacterium; Mycobacterium tuberculosis complex; Mycobacterium tuberculosis;	NCBI: 1773	4388908	99.94	0.33	Yes
NCBI Assembly	GCA_001947445.1	NCBI: cellular organisms; Bacteria; Terrabacteria group; Actinobacteria; Actinomycetia; Corynebacteriales; Mycobacteriaceae; Mycobacterium; Mycobacterium tuberculosis complex; Mycobacterium tuberculosis;	NCBI: 1773	4347433	99.94	0.00	Yes
NCBI Assembly	GCA_001944325.1	NCBI: cellular organisms; Bacteria; Terrabacteria group; Actinobacteria; Actinomycetia; Corynebacteriales; Mycobacteriaceae; Mycobacterium; Mycobacterium tuberculosis complex; Mycobacterium tuberculosis;	NCBI: 1773	4360317	99.94	0.00	Yes
NCBI Assembly	GCA_001998705.1	NCBI: cellular organisms; Bacteria; Terrabacteria group; Actinobacteria; Actinomycetia; Corynebacteriales; Mycobacteriaceae; Mycobacterium; Mycobacterium tuberculosis complex; Mycobacterium tuberculosis;	NCBI: 1334070	4411532	99.94	0.00	Yes
NCBI Assembly	GCA_001946195.1	NCBI: cellular organisms; Bacteria; Terrabacteria group; Actinobacteria; Actinomycetia; Corynebacteriales; Mycobacteriaceae; Mycobacterium; Mycobacterium tuberculosis complex; Mycobacterium tuberculosis;	NCBI: 1773	4399202	99.94	0.00	Yes

NCBI Assembly	GCA_002086855.1	NCBI: cellular organisms; Bacteria; Terrabacteria group; Actinobacteria; Actinomycetia; Corynebacteriales; Mycobacteriaceae; Mycobacterium; Mycobacterium persicum;	NCBI: 1487726	6175840	99.92	0.67	Yes
NCBI Assembly	GCA_002130765.1	NCBI: cellular organisms; Bacteria; Terrabacteria group; Actinobacteria; Actinomycetia; Corynebacteriales; Mycobacteriaceae; Mycobacterium; Mycobacterium tuberculosis complex; Mycobacterium tuberculosis;	NCBI: 1773	4394512	99.94	0.00	Yes
NCBI Assembly	GCA_001997605.1	NCBI: cellular organisms; Bacteria; Terrabacteria group; Actinobacteria; Actinomycetia; Corynebacteriales; Mycobacteriaceae; Mycobacterium; Mycobacterium tuberculosis complex; Mycobacterium tuberculosis;	NCBI: 1334082	4411532	99.94	0.00	Yes
NCBI Assembly	GCA_002447795.1	NCBI: cellular organisms; Bacteria; Terrabacteria group; Actinobacteria; Actinomycetia; Corynebacteriales; Mycobacteriaceae; Mycobacterium; Mycobacterium tuberculosis complex; Mycobacterium tuberculosis;	NCBI: 1773	4411392	99.94	0.00	Yes
NCBI Assembly	GCA_002129175.1	NCBI: cellular organisms; Bacteria; Terrabacteria group; Actinobacteria; Actinomycetia; Corynebacteriales; Mycobacteriaceae; Mycobacterium; Mycobacterium tuberculosis complex; Mycobacterium tuberculosis;	NCBI: 1773	4384630	99.94	0.00	Yes
NCBI Assembly	GCA_001948725.1	NCBI: cellular organisms; Bacteria; Terrabacteria group; Actinobacteria; Actinomycetia; Corynebacteriales; Mycobacteriaceae; Mycobacterium; Mycobacterium tuberculosis complex; Mycobacterium tuberculosis;	NCBI: 1773	4353148	99.94	0.00	Yes
NCBI Assembly	GCA_001954205.1	NCBI: cellular organisms; Bacteria; Terrabacteria group; Actinobacteria; Actinomycetia; Corynebacteriales; Mycobacteriaceae; Mycolicibacterium; Mycolicibacterium conceptionense;	NCBI: 451644	6162534	100.00	0.00	Yes
NCBI Assembly	GCA_002799775.1	NCBI: cellular organisms; Bacteria; Terrabacteria group; Actinobacteria; Actinomycetia; Corynebacteriales; Mycobacteriaceae; Mycobacteroides; Mycobacteroides abscessus; Mycobacteroides abscessus subsp. massiliense;	NCBI: 1962118	4892361	100.00	0.00	Yes
NCBI Assembly	GCA_001998735.1	NCBI: cellular organisms; Bacteria; Terrabacteria group; Actinobacteria; Actinomycetia; Corynebacteriales; Mycobacteriaceae; Mycobacterium; Mycobacterium tuberculosis complex; Mycobacterium tuberculosis;	NCBI: 1334072	4411532	99.94	0.00	Yes
NCBI Assembly	GCA_001944335.1	NCBI: cellular organisms; Bacteria; Terrabacteria group; Actinobacteria; Actinomycetia; Corynebacteriales; Mycobacteriaceae; Mycobacterium; Mycobacterium tuberculosis complex; Mycobacterium tuberculosis;	NCBI: 1773	4384732	99.94	0.00	Yes
NCBI Assembly	GCA_001947455.1	NCBI: cellular organisms; Bacteria; Terrabacteria group; Actinobacteria; Actinomycetia; Corynebacteriales; Mycobacteriaceae; Mycobacterium; Mycobacterium tuberculosis complex; Mycobacterium tuberculosis;	NCBI: 1773	4341295	99.94	0.00	Yes
NCBI Assembly	GCA_001946245.1	NCBI: cellular organisms; Bacteria; Terrabacteria group; Actinobacteria; Actinomycetia; Corynebacteriales; Mycobacteriaceae; Mycobacterium; Mycobacterium tuberculosis complex; Mycobacterium tuberculosis;	NCBI: 1773	4394442	99.94	0.00	Yes
NCBI Assembly	GCA_002086865.1	NCBI: cellular organisms; Bacteria; Terrabacteria group; Actinobacteria; Actinomycetia; Corynebacteriales; Mycobacteriaceae; Mycobacterium; Mycobacterium kansasii;	NCBI: 1768	6514022	99.62	3.28	Yes
NCBI Assembly	GCA_002130775.1	NCBI: cellular organisms; Bacteria; Terrabacteria group; Actinobacteria; Actinomycetia; Corynebacteriales; Mycobacteriaceae; Mycobacterium; Mycobacterium tuberculosis complex; Mycobacterium tuberculosis;	NCBI: 1773	4396687	99.94	0.00	Yes
NCBI Assembly	GCA_001997625.1	NCBI: cellular organisms; Bacteria; Terrabacteria group; Actinobacteria; Actinomycetia; Corynebacteriales; Mycobacteriaceae; Mycobacterium; Mycobacterium tuberculosis complex; Mycobacterium tuberculosis;	NCBI: 1334083	4411532	99.94	0.00	Yes
NCBI Assembly	GCA_002129205.1	NCBI: cellular organisms; Bacteria; Terrabacteria group; Actinobacteria; Actinomycetia; Corynebacteriales; Mycobacteriaceae; Mycobacterium; Mycobacterium tuberculosis complex; Mycobacterium tuberculosis;	NCBI: 1773	4358725	99.94	0.00	Yes
NCBI Assembly	GCA_002447815.1	NCBI: cellular organisms; Bacteria; Terrabacteria group; Actinobacteria; Actinomycetia; Corynebacteriales; Mycobacteriaceae; Mycobacterium; Mycobacterium tuberculosis complex; Mycobacterium tuberculosis;	NCBI: 1773	4411299	99.94	0.07	Yes
NCBI Assembly	GCA_001954215.1	NCBI: cellular organisms; Bacteria; Terrabacteria group; Actinobacteria; Actinomycetia; Corynebacteriales; Mycobacteriaceae; Mycobacterium; unclassified Mycobacterium;	NCBI: 1834159	4871050	99.61	0.27	Yes

NCBI Assembly	GCA_001948765.1	NCBI: cellular organisms; Bacteria; Terrabacteria group; Actinobacteria; Actinomycetia; Corynebacteriales; Mycobacteriaceae; Mycobacterium; Mycobacterium tuberculosis complex; Mycobacterium tuberculosis;	NCBI: 1773	4416446	99.94	0.00	Yes
NCBI Assembly	GCA_001944365.1	NCBI: cellular organisms; Bacteria; Terrabacteria group; Actinobacteria; Actinomycetia; Corynebacteriales; Mycobacteriaceae; Mycobacterium; Mycobacterium tuberculosis complex; Mycobacterium tuberculosis;	NCBI: 1773	4379344	99.94	0.00	Yes
NCBI Assembly	GCA_001999445.1	NCBI: cellular organisms; Bacteria; Terrabacteria group; Actinobacteria; Actinomycetia; Corynebacteriales; Mycobacteriaceae; Mycobacterium; Mycobacterium tuberculosis complex; Mycobacterium tuberculosis;	NCBI: 1773	4280151	99.91	0.00	Yes
NCBI Assembly	GCA_001947505.1	NCBI: cellular organisms; Bacteria; Terrabacteria group; Actinobacteria; Actinomycetia; Corynebacteriales; Mycobacteriaceae; Mycobacterium; Mycobacterium tuberculosis complex; Mycobacterium tuberculosis;	NCBI: 1773	4385307	99.94	0.00	Yes
NCBI Assembly	GCA_002799795.1	NCBI: cellular organisms; Bacteria; Terrabacteria group; Actinobacteria; Actinomycetia; Corynebacteriales; Mycobacteriaceae; Mycobacteroides; Mycobacteroides abscessus; Mycobacteroides abscessus subsp. massiliense;	NCBI: 1962118	4939165	100.00	0.00	Yes
NCBI Assembly	GCA_001946255.1	NCBI: cellular organisms; Bacteria; Terrabacteria group; Actinobacteria; Actinomycetia; Corynebacteriales; Mycobacteriaceae; Mycobacterium; Mycobacterium tuberculosis complex; Mycobacterium tuberculosis;	NCBI: 1773	4389855	99.94	0.00	Yes
NCBI Assembly	GCA_002086895.1	NCBI: cellular organisms; Bacteria; Terrabacteria group; Actinobacteria; Actinomycetia; Corynebacteriales; Mycobacteriaceae; Mycobacterium; Mycobacterium kansasii;	NCBI: 1768	6687473	98.61	2.04	Yes
NCBI Assembly	GCA_002130785.1	NCBI: cellular organisms; Bacteria; Terrabacteria group; Actinobacteria; Actinomycetia; Corynebacteriales; Mycobacteriaceae; Mycobacterium; Mycobacterium tuberculosis complex; Mycobacterium tuberculosis;	NCBI: 1773	4383048	99.94	0.00	Yes
NCBI Assembly	GCA_001997665.1	NCBI: cellular organisms; Bacteria; Terrabacteria group; Actinobacteria; Actinomycetia; Corynebacteriales; Mycobacteriaceae; Mycobacterium; Mycobacterium tuberculosis complex; Mycobacterium tuberculosis;	NCBI: 1334084	4411532	99.94	0.00	Yes
NCBI Assembly	GCA_002129215.1	NCBI: cellular organisms; Bacteria; Terrabacteria group; Actinobacteria; Actinomycetia; Corynebacteriales; Mycobacteriaceae; Mycobacterium; Mycobacterium tuberculosis complex; Mycobacterium tuberculosis;	NCBI: 1773	4385699	99.94	0.00	Yes
NCBI Assembly	GCA_002447835.1	NCBI: cellular organisms; Bacteria; Terrabacteria group; Actinobacteria; Actinomycetia; Corynebacteriales; Mycobacteriaceae; Mycobacterium; Mycobacterium tuberculosis complex; Mycobacterium tuberculosis;	NCBI: 1773	4411327	99.94	0.00	Yes
NCBI Assembly	GCA_001944375.1	NCBI: cellular organisms; Bacteria; Terrabacteria group; Actinobacteria; Actinomycetia; Corynebacteriales; Mycobacteriaceae; Mycobacterium; Mycobacterium tuberculosis complex; Mycobacterium tuberculosis;	NCBI: 1773	4393899	99.94	0.00	Yes
NCBI Assembly	GCA_001946885.1	NCBI: cellular organisms; Bacteria; Terrabacteria group; Actinobacteria; Actinomycetia; Corynebacteriales; Mycobacteriaceae; Mycobacterium; Mycobacterium tuberculosis complex; Mycobacterium tuberculosis;	NCBI: 1773	4386429	99.94	0.00	Yes
NCBI Assembly	GCA_001948775.1	NCBI: cellular organisms; Bacteria; Terrabacteria group; Actinobacteria; Actinomycetia; Corynebacteriales; Mycobacteriaceae; Mycobacterium; Mycobacterium tuberculosis complex; Mycobacterium tuberculosis;	NCBI: 1773	4359937	99.58	0.00	Yes
NCBI Assembly	GCA_001997685.1	NCBI: cellular organisms; Bacteria; Terrabacteria group; Actinobacteria; Actinomycetia; Corynebacteriales; Mycobacteriaceae; Mycobacterium; Mycobacterium tuberculosis complex; Mycobacterium tuberculosis;	NCBI: 1773	4260025	99.94	0.11	Yes
NCBI Assembly	GCA_001947515.1	NCBI: cellular organisms; Bacteria; Terrabacteria group; Actinobacteria; Actinomycetia; Corynebacteriales; Mycobacteriaceae; Mycobacterium; Mycobacterium tuberculosis complex; Mycobacterium tuberculosis;	NCBI: 1773	4391128	99.61	0.00	Yes
NCBI Assembly	GCA_001946265.1	NCBI: cellular organisms; Bacteria; Terrabacteria group; Actinobacteria; Actinomycetia; Corynebacteriales; Mycobacteriaceae; Mycobacterium; Mycobacterium tuberculosis complex; Mycobacterium tuberculosis;	NCBI: 1773	4380705	99.94	0.00	Yes
NCBI Assembly	GCA_002799805.1	NCBI: cellular organisms; Bacteria; Terrabacteria group; Actinobacteria; Actinomycetia; Corynebacteriales; Mycobacteriaceae; Mycobacteroides; Mycobacteroides abscessus; Mycobacteroides abscessus subsp. massiliense;	NCBI: 1962118	4841633	100.00	0.00	Yes

NCBI Assembly	GCA_002086915.1	NCBI: cellular organisms; Bacteria; Terrabacteria group; Actinobacteria; Actinomycetia; Corynebacteriales; Mycobacteriaceae; Mycobacterium; Mycobacterium persicum;	NCBI: 1487726	6347217	99.37	1.00	Yes
NCBI Assembly	GCA_001945325.1	NCBI: cellular organisms; Bacteria; Terrabacteria group; Actinobacteria; Actinomycetia; Corynebacteriales; Mycobacteriaceae; Mycobacterium; Mycobacterium tuberculosis complex; Mycobacterium tuberculosis;	NCBI: 1773	4384114	99.61	0.00	Yes
NCBI Assembly	GCA_002130825.1	NCBI: cellular organisms; Bacteria; Terrabacteria group; Actinobacteria; Actinomycetia; Corynebacteriales; Mycobacteriaceae; Mycobacterium; Mycobacterium tuberculosis complex; Mycobacterium tuberculosis;	NCBI: 1773	4392910	99.94	0.00	Yes
NCBI Assembly	GCA_002129245.1	NCBI: cellular organisms; Bacteria; Terrabacteria group; Actinobacteria; Actinomycetia; Corynebacteriales; Mycobacteriaceae; Mycobacterium; Mycobacterium tuberculosis complex; Mycobacterium tuberculosis;	NCBI: 1773	4401572	99.94	0.00	Yes
NCBI Assembly	GCA_001997695.1	NCBI: cellular organisms; Bacteria; Terrabacteria group; Actinobacteria; Actinomycetia; Corynebacteriales; Mycobacteriaceae; Mycobacterium; Mycobacterium tuberculosis complex; Mycobacterium tuberculosis;	NCBI: 1334047	4411532	99.94	0.00	Yes
NCBI Assembly	GCA_001943755.1	NCBI: cellular organisms; Bacteria; Terrabacteria group; Actinobacteria; Actinomycetia; Corynebacteriales; Mycobacteriaceae; Mycobacterium; Mycobacterium tuberculosis complex; Mycobacterium tuberculosis;	NCBI: 1773	4390005	99.94	0.00	Yes
NCBI Assembly	GCA_001948785.1	NCBI: cellular organisms; Bacteria; Terrabacteria group; Actinobacteria; Actinomycetia; Corynebacteriales; Mycobacteriaceae; Mycobacterium; Mycobacterium tuberculosis complex; Mycobacterium tuberculosis;	NCBI: 1773	4384350	99.94	0.00	Yes
NCBI Assembly	GCA_001946895.1	NCBI: cellular organisms; Bacteria; Terrabacteria group; Actinobacteria; Actinomycetia; Corynebacteriales; Mycobacteriaceae; Mycobacterium; Mycobacterium tuberculosis complex; Mycobacterium tuberculosis;	NCBI: 1773	4409036	99.94	0.00	Yes
NCBI Assembly	GCA_002447855.1	NCBI: cellular organisms; Bacteria; Terrabacteria group; Actinobacteria; Actinomycetia; Corynebacteriales; Mycobacteriaceae; Mycobacterium; Mycobacterium tuberculosis complex; Mycobacterium tuberculosis;	NCBI: 1773	4411315	99.94	0.00	Yes
NCBI Assembly	GCA_001947525.1	NCBI: cellular organisms; Bacteria; Terrabacteria group; Actinobacteria; Actinomycetia; Corynebacteriales; Mycobacteriaceae; Mycobacterium; Mycobacterium tuberculosis complex; Mycobacterium tuberculosis;	NCBI: 1773	4379772	99.94	0.00	Yes
NCBI Assembly	GCA_001946275.1	NCBI: cellular organisms; Bacteria; Terrabacteria group; Actinobacteria; Actinomycetia; Corynebacteriales; Mycobacteriaceae; Mycobacterium; Mycobacterium tuberculosis complex; Mycobacterium tuberculosis;	NCBI: 1773	4365330	99.94	0.00	Yes
NCBI Assembly	GCA_002799845.1	NCBI: cellular organisms; Bacteria; Terrabacteria group; Actinobacteria; Actinomycetia; Corynebacteriales; Mycobacteriaceae; Mycobacteroides; Mycobacteroides abscessus; Mycobacteroides abscessus subsp. massiliense;	NCBI: 1962118	4899997	100.00	0.39	Yes
NCBI Assembly	GCA_001945685.1	NCBI: cellular organisms; Bacteria; Terrabacteria group; Actinobacteria; Actinomycetia; Corynebacteriales; Mycobacteriaceae; Mycobacterium; Mycobacterium tuberculosis complex; Mycobacterium tuberculosis;	NCBI: 1773	4377472	99.94	0.00	Yes
NCBI Assembly	GCA_002086925.1	NCBI: cellular organisms; Bacteria; Terrabacteria group; Actinobacteria; Actinomycetia; Corynebacteriales; Mycobacteriaceae; Mycobacterium; Mycobacterium persicum;	NCBI: 1487726	6527567	99.70	0.75	Yes
NCBI Assembly	GCA_002130845.1	NCBI: cellular organisms; Bacteria; Terrabacteria group; Actinobacteria; Actinomycetia; Corynebacteriales; Mycobacteriaceae; Mycobacterium; Mycobacterium tuberculosis complex; Mycobacterium tuberculosis;	NCBI: 1773	4406721	99.94	0.00	Yes
NCBI Assembly	GCA_002110345.1	NCBI: cellular organisms; Bacteria; Terrabacteria group; Actinobacteria; Actinomycetia; Corynebacteriales; Mycobacteriaceae; Mycobacterium; Mycobacterium tuberculosis complex; Mycobacterium tuberculosis;	NCBI: 1773	4409295	99.94	0.00	Yes
NCBI Assembly	GCA_001997725.1	NCBI: cellular organisms; Bacteria; Terrabacteria group; Actinobacteria; Actinomycetia; Corynebacteriales; Mycobacteriaceae; Mycobacterium; Mycobacterium tuberculosis complex; Mycobacterium tuberculosis;	NCBI: 1334062	4411532	99.94	0.00	Yes
NCBI Assembly	GCA_001943805.1	NCBI: cellular organisms; Bacteria; Terrabacteria group; Actinobacteria; Actinomycetia; Corynebacteriales; Mycobacteriaceae; Mycobacterium; Mycobacterium tuberculosis complex; Mycobacterium tuberculosis;	NCBI: 1773	4387456	99.94	0.00	Yes

NCBI Assembly	GCA_001946945.1	NCBI: cellular organisms; Bacteria; Terrabacteria group; Actinobacteria; Actinomycetia; Corynebacteriales; Mycobacteriaceae; Mycobacterium; Mycobacterium tuberculosis complex; Mycobacterium tuberculosis;	NCBI: 1773	4392051	99.94	0.00	Yes
NCBI Assembly	GCA_001948805.1	NCBI: cellular organisms; Bacteria; Terrabacteria group; Actinobacteria; Actinomycetia; Corynebacteriales; Mycobacteriaceae; Mycobacterium; Mycobacterium tuberculosis complex; Mycobacterium tuberculosis;	NCBI: 1773	4375492	99.94	0.00	Yes
NCBI Assembly	GCA_001947535.1	NCBI: cellular organisms; Bacteria; Terrabacteria group; Actinobacteria; Actinomycetia; Corynebacteriales; Mycobacteriaceae; Mycobacterium; Mycobacterium tuberculosis complex; Mycobacterium tuberculosis;	NCBI: 1773	4363923	99.28	0.00	Yes
NCBI Assembly	GCA_002447875.1	NCBI: cellular organisms; Bacteria; Terrabacteria group; Actinobacteria; Actinomycetia; Corynebacteriales; Mycobacteriaceae; Mycobacterium; Mycobacterium tuberculosis complex; Mycobacterium tuberculosis;	NCBI: 1773	4411290	99.94	0.00	Yes
NCBI Assembly	GCA_001946325.1	NCBI: cellular organisms; Bacteria; Terrabacteria group; Actinobacteria; Actinomycetia; Corynebacteriales; Mycobacteriaceae; Mycobacterium; Mycobacterium tuberculosis complex; Mycobacterium tuberculosis;	NCBI: 1773	4380456	99.94	0.00	Yes
NCBI Assembly	GCA_001945695.1	NCBI: cellular organisms; Bacteria; Terrabacteria group; Actinobacteria; Actinomycetia; Corynebacteriales; Mycobacteriaceae; Mycobacterium; Mycobacterium tuberculosis complex; Mycobacterium tuberculosis;	NCBI: 1773	4393053	99.94	0.00	Yes
NCBI Assembly	GCA_002130855.1	NCBI: cellular organisms; Bacteria; Terrabacteria group; Actinobacteria; Actinomycetia; Corynebacteriales; Mycobacteriaceae; Mycobacterium; Mycobacterium tuberculosis complex; Mycobacterium tuberculosis;	NCBI: 1773	4404043	99.94	0.00	Yes
NCBI Assembly	GCA_002799875.1	NCBI: cellular organisms; Bacteria; Terrabacteria group; Actinobacteria; Actinomycetia; Corynebacteriales; Mycobacteriaceae; Mycobacteroides; Mycobacteroides abscessus; Mycobacteroides abscessus subsp. massiliense;	NCBI: 1962118	4868095	100.00	0.00	Yes
NCBI Assembly	GCA_002086275.1	NCBI: cellular organisms; Bacteria; Terrabacteria group; Actinobacteria; Actinomycetia; Corynebacteriales; Mycobacteriaceae; Mycobacterium; Mycobacterium simiae complex; Mycobacterium intermedium;	NCBI: 28445	6817968	99.94	1.83	Yes
NCBI Assembly	GCA_002116635.1	NCBI: cellular organisms; Bacteria; Terrabacteria group; Actinobacteria; Actinomycetia; Corynebacteriales; Mycobacteriaceae; Mycobacterium; Mycobacterium szulgai;	NCBI: 1787	6672659	99.94	0.78	Yes
NCBI Assembly	GCA_001997745.1	NCBI: cellular organisms; Bacteria; Terrabacteria group; Actinobacteria; Actinomycetia; Corynebacteriales; Mycobacteriaceae; Mycobacterium; Mycobacterium tuberculosis complex; Mycobacterium tuberculosis;	NCBI: 1773	4262102	99.61	0.00	Yes
NCBI Assembly	GCA_001946955.1	NCBI: cellular organisms; Bacteria; Terrabacteria group; Actinobacteria; Actinomycetia; Corynebacteriales; Mycobacteriaceae; Mycobacterium; Mycobacterium tuberculosis complex; Mycobacterium tuberculosis;	NCBI: 1773	4388629	99.94	0.00	Yes
NCBI Assembly	GCA_001943815.1	NCBI: cellular organisms; Bacteria; Terrabacteria group; Actinobacteria; Actinomycetia; Corynebacteriales; Mycobacteriaceae; Mycobacterium; Mycobacterium tuberculosis complex; Mycobacterium tuberculosis;	NCBI: 1773	4364326	99.94	0.00	Yes
NCBI Assembly	GCA_001948845.1	NCBI: cellular organisms; Bacteria; Terrabacteria group; Actinobacteria; Actinomycetia; Corynebacteriales; Mycobacteriaceae; Mycobacterium; Mycobacterium tuberculosis complex; Mycobacterium tuberculosis;	NCBI: 1773	4378677	99.94	0.00	Yes
NCBI Assembly	GCA_001947585.1	NCBI: cellular organisms; Bacteria; Terrabacteria group; Actinobacteria; Actinomycetia; Corynebacteriales; Mycobacteriaceae; Mycobacterium; Mycobacterium tuberculosis complex; Mycobacterium tuberculosis;	NCBI: 1773	4413791	99.94	0.00	Yes
NCBI Assembly	GCA_002447275.1	NCBI: cellular organisms; Bacteria; Terrabacteria group; Actinobacteria; Actinomycetia; Corynebacteriales; Mycobacteriaceae; Mycobacterium; Mycobacterium tuberculosis complex; Mycobacterium tuberculosis;	NCBI: 1773	4411408	99.94	0.00	Yes
NCBI Assembly	GCA_001946335.1	NCBI: cellular organisms; Bacteria; Terrabacteria group; Actinobacteria; Actinomycetia; Corynebacteriales; Mycobacteriaceae; Mycobacterium; Mycobacterium tuberculosis complex; Mycobacterium tuberculosis;	NCBI: 1773	4345593	99.94	0.00	Yes
NCBI Assembly	GCA_001945705.1	NCBI: cellular organisms; Bacteria; Terrabacteria group; Actinobacteria; Actinomycetia; Corynebacteriales; Mycobacteriaceae; Mycobacterium; Mycobacterium tuberculosis complex; Mycobacterium tuberculosis;	NCBI: 1773	4390597	99.94	0.00	Yes

NCBI Assembly	GCA_002130865.1	NCBI: cellular organisms; Bacteria; Terrabacteria group; Actinobacteria; Actinomycetia; Corynebacteriales; Mycobacteriaceae; Mycobacterium; Mycobacterium tuberculosis complex; Mycobacterium tuberculosis;	NCBI: 1773	4398013	99.94	0.00	Yes
NCBI Assembly	GCA_002086285.1	NCBI: cellular organisms; Bacteria; Terrabacteria group; Actinobacteria; Actinomycetia; Corynebacteriales; Mycobacteriaceae; Mycolicibacter; Mycolicibacter kumamotonensis;	NCBI: 354243	4815853	99.55	0.15	Yes
NCBI Assembly	GCA_002799905.1	NCBI: cellular organisms; Bacteria; Terrabacteria group; Actinobacteria; Actinomycetia; Corynebacteriales; Mycobacteriaceae; Mycobacteroides; Mycobacteroides abscessus; Mycobacteroides abscessus subsp. massiliense;	NCBI: 1962118	4898505	99.89	0.00	Yes
NCBI Assembly	GCA_002116755.1	NCBI: cellular organisms; Bacteria; Terrabacteria group; Actinobacteria; Actinomycetia; Corynebacteriales; Mycobacteriaceae; Mycobacterium; Mycobacterium tuberculosis complex; Mycobacterium tuberculosis;	NCBI: 1773	4427062	99.94	0.33	Yes
NCBI Assembly	GCA_001997765.1	NCBI: cellular organisms; Bacteria; Terrabacteria group; Actinobacteria; Actinomycetia; Corynebacteriales; Mycobacteriaceae; Mycobacterium; Mycobacterium tuberculosis complex; Mycobacterium tuberculosis;	NCBI: 1773	4238058	99.42	0.00	Yes
NCBI Assembly	GCA_001946965.1	NCBI: cellular organisms; Bacteria; Terrabacteria group; Actinobacteria; Actinomycetia; Corynebacteriales; Mycobacteriaceae; Mycobacterium; Mycobacterium tuberculosis complex; Mycobacterium tuberculosis;	NCBI: 1773	4388641	99.94	0.00	Yes
NCBI Assembly	GCA_001948855.1	NCBI: cellular organisms; Bacteria; Terrabacteria group; Actinobacteria; Actinomycetia; Corynebacteriales; Mycobacteriaceae; Mycobacterium; Mycobacterium tuberculosis complex; Mycobacterium tuberculosis;	NCBI: 1773	4395795	99.94	0.00	Yes
NCBI Assembly	GCA_001947595.1	NCBI: cellular organisms; Bacteria; Terrabacteria group; Actinobacteria; Actinomycetia; Corynebacteriales; Mycobacteriaceae; Mycobacterium; Mycobacterium tuberculosis complex; Mycobacterium tuberculosis;	NCBI: 1773	4397541	99.94	0.00	Yes
NCBI Assembly	GCA_001943825.1	NCBI: cellular organisms; Bacteria; Terrabacteria group; Actinobacteria; Actinomycetia; Corynebacteriales; Mycobacteriaceae; Mycobacterium; Mycobacterium tuberculosis complex; Mycobacterium tuberculosis;	NCBI: 1773	4393079	99.61	0.00	Yes
NCBI Assembly	GCA_001946345.1	NCBI: cellular organisms; Bacteria; Terrabacteria group; Actinobacteria; Actinomycetia; Corynebacteriales; Mycobacteriaceae; Mycobacterium; Mycobacterium tuberculosis complex; Mycobacterium tuberculosis;	NCBI: 1773	4392481	99.94	0.00	Yes
NCBI Assembly	GCA_002447295.1	NCBI: cellular organisms; Bacteria; Terrabacteria group; Actinobacteria; Actinomycetia; Corynebacteriales; Mycobacteriaceae; Mycobacterium; Mycobacterium tuberculosis complex; Mycobacterium tuberculosis;	NCBI: 1773	4411306	99.94	0.00	Yes
NCBI Assembly	GCA_001945715.1	NCBI: cellular organisms; Bacteria; Terrabacteria group; Actinobacteria; Actinomycetia; Corynebacteriales; Mycobacteriaceae; Mycobacterium; Mycobacterium tuberculosis complex; Mycobacterium tuberculosis;	NCBI: 1773	4394210	99.94	0.00	Yes
NCBI Assembly	GCA_002130895.1	NCBI: cellular organisms; Bacteria; Terrabacteria group; Actinobacteria; Actinomycetia; Corynebacteriales; Mycobacteriaceae; Mycobacterium; Mycobacterium tuberculosis complex; Mycobacterium tuberculosis;	NCBI: 1773	4405527	99.94	0.00	Yes
NCBI Assembly	GCA_002086305.1	NCBI: cellular organisms; Bacteria; Terrabacteria group; Actinobacteria; Actinomycetia; Corynebacteriales; Mycobacteriaceae; Mycobacterium; Mycobacterium malmoense;	NCBI: 1780	5298816	99.61	1.22	Yes
NCBI Assembly	GCA_001997775.1	NCBI: cellular organisms; Bacteria; Terrabacteria group; Actinobacteria; Actinomycetia; Corynebacteriales; Mycobacteriaceae; Mycobacterium; Mycobacterium tuberculosis complex; Mycobacterium tuberculosis;	NCBI: 1334063	4411532	99.94	0.00	Yes
NCBI Assembly	GCA_002116775.1	NCBI: cellular organisms; Bacteria; Terrabacteria group; Actinobacteria; Actinomycetia; Corynebacteriales; Mycobacteriaceae; Mycobacterium; Mycobacterium tuberculosis complex; Mycobacterium tuberculosis;	NCBI: 1773	4441591	99.94	1.00	Yes
NCBI Assembly	GCA_002799915.1	NCBI: cellular organisms; Bacteria; Terrabacteria group; Actinobacteria; Actinomycetia; Corynebacteriales; Mycobacteriaceae; Mycobacteroides; Mycobacteroides abscessus; Mycobacteroides abscessus subsp. massiliense;	NCBI: 1962118	4785864	100.00	0.00	Yes
NCBI Assembly	GCA_001946995.1	NCBI: cellular organisms; Bacteria; Terrabacteria group; Actinobacteria; Actinomycetia; Corynebacteriales; Mycobacteriaceae; Mycobacterium; Mycobacterium tuberculosis complex; Mycobacterium tuberculosis;	NCBI: 1773	4386587	99.94	0.00	Yes

NCBI Assembly	GCA_001948865.1	NCBI: cellular organisms; Bacteria; Terrabacteria group; Actinobacteria; Actinomycetia; Corynebacteriales; Mycobacteriaceae; Mycobacterium; Mycobacterium tuberculosis complex; Mycobacterium tuberculosis;	NCBI: 1773	4390808	99.94	0.00	Yes
NCBI Assembly	GCA_001947605.1	NCBI: cellular organisms; Bacteria; Terrabacteria group; Actinobacteria; Actinomycetia; Corynebacteriales; Mycobacteriaceae; Mycobacterium; Mycobacterium tuberculosis complex; Mycobacterium tuberculosis;	NCBI: 1773	4396925	99.94	0.00	Yes
NCBI Assembly	GCA_001943835.1	NCBI: cellular organisms; Bacteria; Terrabacteria group; Actinobacteria; Actinomycetia; Corynebacteriales; Mycobacteriaceae; Mycobacterium; Mycobacterium tuberculosis complex; Mycobacterium tuberculosis;	NCBI: 1773	4352518	99.94	0.00	Yes
NCBI Assembly	GCA_001946355.1	NCBI: cellular organisms; Bacteria; Terrabacteria group; Actinobacteria; Actinomycetia; Corynebacteriales; Mycobacteriaceae; Mycobacterium; Mycobacterium tuberculosis complex; Mycobacterium tuberculosis;	NCBI: 1773	4389570	99.94	0.00	Yes
NCBI Assembly	GCA_002447315.1	NCBI: cellular organisms; Bacteria; Terrabacteria group; Actinobacteria; Actinomycetia; Corynebacteriales; Mycobacteriaceae; Mycobacterium; Mycobacterium tuberculosis complex; Mycobacterium tuberculosis;	NCBI: 1773	4411134	99.94	0.00	Yes
NCBI Assembly	GCA_001945765.1	NCBI: cellular organisms; Bacteria; Terrabacteria group; Actinobacteria; Actinomycetia; Corynebacteriales; Mycobacteriaceae; Mycobacterium; Mycobacterium tuberculosis complex; Mycobacterium tuberculosis;	NCBI: 1773	4381767	99.94	0.00	Yes
NCBI Assembly	GCA_002130925.1	NCBI: cellular organisms; Bacteria; Terrabacteria group; Actinobacteria; Actinomycetia; Corynebacteriales; Mycobacteriaceae; Mycobacterium; Mycobacterium tuberculosis complex; Mycobacterium tuberculosis;	NCBI: 1773	4384514	99.94	0.00	Yes
NCBI Assembly	GCA_002086335.1	NCBI: cellular organisms; Bacteria; Terrabacteria group; Actinobacteria; Actinomycetia; Corynebacteriales; Mycobacteriaceae; Mycobacterium; Mycobacterium avium complex (MAC); Mycobacterium mantenii;	NCBI: 560555	6121894	99.94	0.91	Yes
NCBI Assembly	GCA_001997805.1	NCBI: cellular organisms; Bacteria; Terrabacteria group; Actinobacteria; Actinomycetia; Corynebacteriales; Mycobacteriaceae; Mycobacterium; Mycobacterium tuberculosis complex; Mycobacterium tuberculosis;	NCBI: 1334066	4411532	99.94	0.00	Yes
NCBI Assembly	GCA_002116795.1	NCBI: cellular organisms; Bacteria; Terrabacteria group; Actinobacteria; Actinomycetia; Corynebacteriales; Mycobacteriaceae; Mycobacterium; Mycobacterium tuberculosis complex; Mycobacterium tuberculosis;	NCBI: 1773	4431885	99.94	0.67	Yes
NCBI Assembly	GCA_001947025.1	NCBI: cellular organisms; Bacteria; Terrabacteria group; Actinobacteria; Actinomycetia; Corynebacteriales; Mycobacteriaceae; Mycobacterium; Mycobacterium tuberculosis complex; Mycobacterium tuberculosis;	NCBI: 1773	4390106	99.94	0.00	Yes
NCBI Assembly	GCA_002799945.1	NCBI: cellular organisms; Bacteria; Terrabacteria group; Actinobacteria; Actinomycetia; Corynebacteriales; Mycobacteriaceae; Mycobacteroides; Mycobacteroides abscessus; Mycobacteroides abscessus subsp. massiliense;	NCBI: 1962118	5012583	100.00	0.00	Yes
NCBI Assembly	GCA_001948885.1	NCBI: cellular organisms; Bacteria; Terrabacteria group; Actinobacteria; Actinomycetia; Corynebacteriales; Mycobacteriaceae; Mycobacterium; Mycobacterium tuberculosis complex; Mycobacterium tuberculosis;	NCBI: 1773	4410040	99.94	0.00	Yes
NCBI Assembly	GCA_001943885.1	NCBI: cellular organisms; Bacteria; Terrabacteria group; Actinobacteria; Actinomycetia; Corynebacteriales; Mycobacteriaceae; Mycobacterium; Mycobacterium tuberculosis complex; Mycobacterium tuberculosis;	NCBI: 1773	4364760	99.61	0.00	Yes
NCBI Assembly	GCA_001947615.1	NCBI: cellular organisms; Bacteria; Terrabacteria group; Actinobacteria; Actinomycetia; Corynebacteriales; Mycobacteriaceae; Mycobacterium; Mycobacterium tuberculosis complex; Mycobacterium tuberculosis;	NCBI: 1773	4400344	99.94	0.00	Yes
NCBI Assembly	GCA_001946405.1	NCBI: cellular organisms; Bacteria; Terrabacteria group; Actinobacteria; Actinomycetia; Corynebacteriales; Mycobacteriaceae; Mycobacterium; Mycobacterium tuberculosis complex; Mycobacterium tuberculosis;	NCBI: 1773	4334873	99.94	0.00	Yes
NCBI Assembly	GCA_001945775.1	NCBI: cellular organisms; Bacteria; Terrabacteria group; Actinobacteria; Actinomycetia; Corynebacteriales; Mycobacteriaceae; Mycobacterium; Mycobacterium tuberculosis complex; Mycobacterium tuberculosis;	NCBI: 1773	4400654	99.94	0.00	Yes
NCBI Assembly	GCA_002447335.1	NCBI: cellular organisms; Bacteria; Terrabacteria group; Actinobacteria; Actinomycetia; Corynebacteriales; Mycobacteriaceae; Mycobacterium; Mycobacterium tuberculosis complex; Mycobacterium tuberculosis;	NCBI: 1773	4411337	99.94	0.00	Yes

NCBI Assembly	GCA_002086345.1	NCBI: cellular organisms; Bacteria; Terrabacteria group; Actinobacteria; Actinomycetia; Corynebacteriales; Mycobacteriaceae; Mycobacterium; Mycobacterium avium complex (MAC); Mycobacterium marseillense;	NCBI: 701042	5456846	99.94	0.05	Yes
NCBI Assembly	GCA_001997825.1	NCBI: cellular organisms; Bacteria; Terrabacteria group; Actinobacteria; Actinomycetia; Corynebacteriales; Mycobacteriaceae; Mycobacterium; Mycobacterium tuberculosis complex; Mycobacterium tuberculosis;	NCBI: 1773	4080864	94.94	1.50	Yes
NCBI Assembly	GCA_002130935.1	NCBI: cellular organisms; Bacteria; Terrabacteria group; Actinobacteria; Actinomycetia; Corynebacteriales; Mycobacteriaceae; Mycobacterium; Mycobacterium tuberculosis complex; Mycobacterium tuberculosis;	NCBI: 1773	4404874	99.94	0.00	Yes
NCBI Assembly	GCA_002116815.1	NCBI: cellular organisms; Bacteria; Terrabacteria group; Actinobacteria; Actinomycetia; Corynebacteriales; Mycobacteriaceae; Mycobacterium; Mycobacterium tuberculosis complex; Mycobacterium tuberculosis;	NCBI: 1773	4406925	99.94	0.00	Yes
NCBI Assembly	GCA_001947035.1	NCBI: cellular organisms; Bacteria; Terrabacteria group; Actinobacteria; Actinomycetia; Corynebacteriales; Mycobacteriaceae; Mycobacterium; Mycobacterium tuberculosis complex; Mycobacterium tuberculosis;	NCBI: 1773	4389305	99.94	0.00	Yes
NCBI Assembly	GCA_001948925.1	NCBI: cellular organisms; Bacteria; Terrabacteria group; Actinobacteria; Actinomycetia; Corynebacteriales; Mycobacteriaceae; Mycobacterium; Mycobacterium tuberculosis complex; Mycobacterium tuberculosis;	NCBI: 1773	4386406	99.90	0.00	Yes
NCBI Assembly	GCA_001943895.1	NCBI: cellular organisms; Bacteria; Terrabacteria group; Actinobacteria; Actinomycetia; Corynebacteriales; Mycobacteriaceae; Mycobacterium; Mycobacterium tuberculosis complex; Mycobacterium tuberculosis;	NCBI: 1773	4353432	99.94	0.00	Yes
NCBI Assembly	GCA_002799965.1	NCBI: cellular organisms; Bacteria; Terrabacteria group; Actinobacteria; Actinomycetia; Corynebacteriales; Mycobacteriaceae; Mycobacteroides; Mycobacteroides abscessus; Mycobacteroides abscessus subsp. massiliense;	NCBI: 1962118	4947450	99.93	0.00	Yes
NCBI Assembly	GCA_001947665.1	NCBI: cellular organisms; Bacteria; Terrabacteria group; Actinobacteria; Actinomycetia; Corynebacteriales; Mycobacteriaceae; Mycobacterium; Mycobacterium tuberculosis complex; Mycobacterium tuberculosis;	NCBI: 1773	4374342	99.94	0.00	Yes
NCBI Assembly	GCA_001946415.1	NCBI: cellular organisms; Bacteria; Terrabacteria group; Actinobacteria; Actinomycetia; Corynebacteriales; Mycobacteriaceae; Mycobacterium; Mycobacterium tuberculosis complex; Mycobacterium tuberculosis;	NCBI: 1773	4384905	99.94	0.00	Yes
NCBI Assembly	GCA_001945785.1	NCBI: cellular organisms; Bacteria; Terrabacteria group; Actinobacteria; Actinomycetia; Corynebacteriales; Mycobacteriaceae; Mycobacterium; Mycobacterium tuberculosis complex; Mycobacterium tuberculosis;	NCBI: 1773	4391876	99.94	0.00	Yes
NCBI Assembly	GCA_002086395.1	NCBI: cellular organisms; Bacteria; Terrabacteria group; Actinobacteria; Actinomycetia; Corynebacteriales; Mycobacteriaceae; Mycolicibacterium; Mycolicibacterium moriokaense;	NCBI: 39691	6217364	100.00	0.61	Yes
NCBI Assembly	GCA_002447355.1	NCBI: cellular organisms; Bacteria; Terrabacteria group; Actinobacteria; Actinomycetia; Corynebacteriales; Mycobacteriaceae; Mycobacterium; Mycobacterium tuberculosis complex; Mycobacterium tuberculosis;	NCBI: 1773	4411385	99.94	0.00	Yes
NCBI Assembly	GCA_001997855.1	NCBI: cellular organisms; Bacteria; Terrabacteria group; Actinobacteria; Actinomycetia; Corynebacteriales; Mycobacteriaceae; Mycobacterium; Mycobacterium tuberculosis complex; Mycobacterium tuberculosis;	NCBI: 1773	4240848	99.61	0.00	Yes
NCBI Assembly	GCA_001947045.1	NCBI: cellular organisms; Bacteria; Terrabacteria group; Actinobacteria; Actinomycetia; Corynebacteriales; Mycobacteriaceae; Mycobacterium; Mycobacterium tuberculosis complex; Mycobacterium tuberculosis;	NCBI: 1773	4392118	99.94	0.00	Yes
NCBI Assembly	GCA_002116835.1	NCBI: cellular organisms; Bacteria; Terrabacteria group; Actinobacteria; Actinomycetia; Corynebacteriales; Mycobacteriaceae; Mycobacterium; Mycobacterium tuberculosis complex; Mycobacterium tuberculosis;	NCBI: 1773	4444417	99.94	0.67	Yes
NCBI Assembly	GCA_002130945.1	NCBI: cellular organisms; Bacteria; Terrabacteria group; Actinobacteria; Actinomycetia; Corynebacteriales; Mycobacteriaceae; Mycobacterium; Mycobacterium tuberculosis complex; Mycobacterium tuberculosis;	NCBI: 1773	4399568	99.94	0.00	Yes
NCBI Assembly	GCA_001943905.1	NCBI: cellular organisms; Bacteria; Terrabacteria group; Actinobacteria; Actinomycetia; Corynebacteriales; Mycobacteriaceae; Mycobacterium; Mycobacterium tuberculosis complex; Mycobacterium tuberculosis;	NCBI: 1773	4389745	99.61	0.00	Yes

NCBI Assembly	GCA_001948935.1	NCBI: cellular organisms; Bacteria; Terrabacteria group; Actinobacteria; Actinomycetia; Corynebacteriales; Mycobacteriaceae; Mycobacterium; Mycobacterium tuberculosis complex; Mycobacterium tuberculosis;	NCBI: 1773	4396113	99.94	0.00	Yes
NCBI Assembly	GCA_001947675.1	NCBI: cellular organisms; Bacteria; Terrabacteria group; Actinobacteria; Actinomycetia; Corynebacteriales; Mycobacteriaceae; Mycobacterium; Mycobacterium tuberculosis complex; Mycobacterium tuberculosis;	NCBI: 1773	4346951	99.94	0.00	Yes
NCBI Assembly	GCA_001946425.1	NCBI: cellular organisms; Bacteria; Terrabacteria group; Actinobacteria; Actinomycetia; Corynebacteriales; Mycobacteriaceae; Mycobacterium; Mycobacterium tuberculosis complex; Mycobacterium tuberculosis;	NCBI: 1773	4405819	99.94	0.00	Yes
NCBI Assembly	GCA_002799985.1	NCBI: cellular organisms; Bacteria; Terrabacteria group; Actinobacteria; Actinomycetia; Corynebacteriales; Mycobacteriaceae; Mycobacteroides; Mycobacteroides abscessus; Mycobacteroides abscessus subsp. massiliense;	NCBI: 1962118	4712134	100.00	0.22	Yes
NCBI Assembly	GCA_001945795.1	NCBI: cellular organisms; Bacteria; Terrabacteria group; Actinobacteria; Actinomycetia; Corynebacteriales; Mycobacteriaceae; Mycobacterium; Mycobacterium tuberculosis complex; Mycobacterium tuberculosis;	NCBI: 1773	4392580	99.94	0.07	Yes
NCBI Assembly	GCA_001997885.1	NCBI: cellular organisms; Bacteria; Terrabacteria group; Actinobacteria; Actinomycetia; Corynebacteriales; Mycobacteriaceae; Mycobacterium; Mycobacterium tuberculosis complex; Mycobacterium tuberculosis;	NCBI: 1334059	4411532	99.94	0.00	Yes
NCBI Assembly	GCA_002447375.1	NCBI: cellular organisms; Bacteria; Terrabacteria group; Actinobacteria; Actinomycetia; Corynebacteriales; Mycobacteriaceae; Mycobacterium; Mycobacterium tuberculosis complex; Mycobacterium tuberculosis;	NCBI: 1773	4411394	99.94	0.00	Yes
NCBI Assembly	GCA_002086405.1	NCBI: cellular organisms; Bacteria; Terrabacteria group; Actinobacteria; Actinomycetia; Corynebacteriales; Mycobacteriaceae; Mycolicibacter; Mycolicibacter minnesotensis;	NCBI: 1118379	4187822	99.55	0.61	Yes
NCBI Assembly	GCA_001947065.1	NCBI: cellular organisms; Bacteria; Terrabacteria group; Actinobacteria; Actinomycetia; Corynebacteriales; Mycobacteriaceae; Mycobacterium; Mycobacterium tuberculosis complex; Mycobacterium tuberculosis;	NCBI: 1773	4347406	99.94	0.00	Yes
NCBI Assembly	GCA_002116855.1	NCBI: cellular organisms; Bacteria; Terrabacteria group; Actinobacteria; Actinomycetia; Corynebacteriales; Mycobacteriaceae; Mycobacterium; Mycobacterium tuberculosis complex; Mycobacterium tuberculosis;	NCBI: 1773	4380156	99.94	0.33	Yes
NCBI Assembly	GCA_001943915.1	NCBI: cellular organisms; Bacteria; Terrabacteria group; Actinobacteria; Actinomycetia; Corynebacteriales; Mycobacteriaceae; Mycobacterium; Mycobacterium tuberculosis complex; Mycobacterium tuberculosis;	NCBI: 1773	4385044	99.61	0.00	Yes
NCBI Assembly	GCA_001948945.1	NCBI: cellular organisms; Bacteria; Terrabacteria group; Actinobacteria; Actinomycetia; Corynebacteriales; Mycobacteriaceae; Mycobacterium; Mycobacterium tuberculosis complex; Mycobacterium tuberculosis;	NCBI: 1773	4350755	99.94	0.00	Yes
NCBI Assembly	GCA_002130955.1	NCBI: cellular organisms; Bacteria; Terrabacteria group; Actinobacteria; Actinomycetia; Corynebacteriales; Mycobacteriaceae; Mycobacterium; Mycobacterium tuberculosis complex; Mycobacterium tuberculosis;	NCBI: 1773	4395945	99.94	0.00	Yes
NCBI Assembly	GCA_001946435.1	NCBI: cellular organisms; Bacteria; Terrabacteria group; Actinobacteria; Actinomycetia; Corynebacteriales; Mycobacteriaceae; Mycobacterium; Mycobacterium tuberculosis complex; Mycobacterium tuberculosis;	NCBI: 1773	4392223	99.94	0.00	Yes
NCBI Assembly	GCA_001947685.1	NCBI: cellular organisms; Bacteria; Terrabacteria group; Actinobacteria; Actinomycetia; Corynebacteriales; Mycobacteriaceae; Mycobacterium; Mycobacterium tuberculosis complex; Mycobacterium tuberculosis;	NCBI: 1773	4366598	99.94	0.00	Yes
NCBI Assembly	GCA_001945845.1	NCBI: cellular organisms; Bacteria; Terrabacteria group; Actinobacteria; Actinomycetia; Corynebacteriales; Mycobacteriaceae; Mycobacterium; Mycobacterium tuberculosis complex; Mycobacterium tuberculosis;	NCBI: 1773	4379608	99.94	0.00	Yes
NCBI Assembly	GCA_002799995.1	NCBI: cellular organisms; Bacteria; Terrabacteria group; Actinobacteria; Actinomycetia; Corynebacteriales; Mycobacteriaceae; Mycobacteroides; Mycobacteroides abscessus; Mycobacteroides abscessus subsp. massiliense;	NCBI: 1962118	4938754	100.00	0.00	Yes
NCBI Assembly	GCA_001997905.1	NCBI: cellular organisms; Bacteria; Terrabacteria group; Actinobacteria; Actinomycetia; Corynebacteriales; Mycobacteriaceae; Mycobacterium; Mycobacterium tuberculosis complex; Mycobacterium tuberculosis;	NCBI: 1773	4275078	99.51	0.00	Yes

NCBI Assembly	GCA_002447395.1	NCBI: cellular organisms; Bacteria; Terrabacteria group; Actinobacteria; Actinomycetia; Corynebacteriales; Mycobacteriaceae; Mycobacterium; Mycobacterium tuberculosis complex; Mycobacterium tuberculosis;	NCBI: 1773	4411115	99.94	0.00	Yes
NCBI Assembly	GCA_001947105.1	NCBI: cellular organisms; Bacteria; Terrabacteria group; Actinobacteria; Actinomycetia; Corynebacteriales; Mycobacteriaceae; Mycobacterium; Mycobacterium tuberculosis complex; Mycobacterium tuberculosis;	NCBI: 1773	4378738	99.94	0.00	Yes
NCBI Assembly	GCA_002086415.1	NCBI: cellular organisms; Bacteria; Terrabacteria group; Actinobacteria; Actinomycetia; Corynebacteriales; Mycobacteriaceae; Mycobacterium; Mycobacterium noviomagense;	NCBI: 459858	4739740	99.55	0.00	Yes
NCBI Assembly	GCA_001948965.1	NCBI: cellular organisms; Bacteria; Terrabacteria group; Actinobacteria; Actinomycetia; Corynebacteriales; Mycobacteriaceae; Mycobacterium; Mycobacterium tuberculosis complex; Mycobacterium tuberculosis;	NCBI: 1773	4387718	99.94	0.07	Yes
NCBI Assembly	GCA_002128795.1	NCBI: cellular organisms; Bacteria; Terrabacteria group; Actinobacteria; Actinomycetia; Corynebacteriales; Mycobacteriaceae; Mycobacterium; Mycobacterium tuberculosis complex; Mycobacterium tuberculosis;	NCBI: 1773	4385818	99.94	0.00	Yes
NCBI Assembly	GCA_001943965.1	NCBI: cellular organisms; Bacteria; Terrabacteria group; Actinobacteria; Actinomycetia; Corynebacteriales; Mycobacteriaceae; Mycobacterium; Mycobacterium tuberculosis complex; Mycobacterium tuberculosis;	NCBI: 1773	4380569	99.94	0.00	Yes
NCBI Assembly	GCA_002131005.1	NCBI: cellular organisms; Bacteria; Terrabacteria group; Actinobacteria; Actinomycetia; Corynebacteriales; Mycobacteriaceae; Mycobacterium; Mycobacterium tuberculosis complex; Mycobacterium tuberculosis;	NCBI: 1773	4406964	99.94	0.00	Yes
NCBI Assembly	GCA_001946485.1	NCBI: cellular organisms; Bacteria; Terrabacteria group; Actinobacteria; Actinomycetia; Corynebacteriales; Mycobacteriaceae; Mycobacterium; Mycobacterium tuberculosis complex; Mycobacterium tuberculosis;	NCBI: 1773	4401843	99.94	0.00	Yes
NCBI Assembly	GCA_001947705.1	NCBI: cellular organisms; Bacteria; Terrabacteria group; Actinobacteria; Actinomycetia; Corynebacteriales; Mycobacteriaceae; Mycobacterium; Mycobacterium tuberculosis complex; Mycobacterium tuberculosis;	NCBI: 1773	4379739	99.94	0.00	Yes
NCBI Assembly	GCA_001945855.1	NCBI: cellular organisms; Bacteria; Terrabacteria group; Actinobacteria; Actinomycetia; Corynebacteriales; Mycobacteriaceae; Mycobacterium; Mycobacterium tuberculosis complex; Mycobacterium tuberculosis;	NCBI: 1773	4391206	99.94	0.00	Yes
NCBI Assembly	GCA_001997915.1	NCBI: cellular organisms; Bacteria; Terrabacteria group; Actinobacteria; Actinomycetia; Corynebacteriales; Mycobacteriaceae; Mycobacterium; Mycobacterium tuberculosis complex; Mycobacterium tuberculosis;	NCBI: 1773	4301039	99.94	0.00	Yes
NCBI Assembly	GCA_002800025.1	NCBI: cellular organisms; Bacteria; Terrabacteria group; Actinobacteria; Actinomycetia; Corynebacteriales; Mycobacteriaceae; Mycobacteroides; Mycobacteroides abscessus; Mycobacteroides abscessus subsp. massiliense;	NCBI: 1962118	4832720	100.00	0.00	Yes
NCBI Assembly	GCA_001949005.1	NCBI: cellular organisms; Bacteria; Terrabacteria group; Actinobacteria; Actinomycetia; Corynebacteriales; Mycobacteriaceae; Mycobacterium; Mycobacterium tuberculosis complex; Mycobacterium tuberculosis;	NCBI: 1773	4409603	99.94	0.00	Yes
NCBI Assembly	GCA_002086455.1	NCBI: cellular organisms; Bacteria; Terrabacteria group; Actinobacteria; Actinomycetia; Corynebacteriales; Mycobacteriaceae; Mycolicibacter; Mycolicibacter algericus;	NCBI: 723879	4619277	100.00	0.15	Yes
NCBI Assembly	GCA_002447415.1	NCBI: cellular organisms; Bacteria; Terrabacteria group; Actinobacteria; Actinomycetia; Corynebacteriales; Mycobacteriaceae; Mycobacterium; Mycobacterium tuberculosis complex; Mycobacterium tuberculosis;	NCBI: 1773	4411439	99.94	0.00	Yes
NCBI Assembly	GCA_001947115.1	NCBI: cellular organisms; Bacteria; Terrabacteria group; Actinobacteria; Actinomycetia; Corynebacteriales; Mycobacteriaceae; Mycobacterium; Mycobacterium tuberculosis complex; Mycobacterium tuberculosis;	NCBI: 1773	4392530	99.94	0.07	Yes
NCBI Assembly	GCA_002128825.1	NCBI: cellular organisms; Bacteria; Terrabacteria group; Actinobacteria; Actinomycetia; Corynebacteriales; Mycobacteriaceae; Mycobacterium; Mycobacterium tuberculosis complex; Mycobacterium tuberculosis;	NCBI: 1773	4393630	99.78	0.00	Yes
NCBI Assembly	GCA_001943975.1	NCBI: cellular organisms; Bacteria; Terrabacteria group; Actinobacteria; Actinomycetia; Corynebacteriales; Mycobacteriaceae; Mycobacterium; Mycobacterium tuberculosis complex; Mycobacterium tuberculosis;	NCBI: 1773	4393827	99.94	0.00	Yes

NCBI Assembly	GCA_001946495.1	NCBI: cellular organisms; Bacteria; Terrabacteria group; Actinobacteria; Actinomycetia; Corynebacteriales; Mycobacteriaceae; Mycobacterium; Mycobacterium tuberculosis complex; Mycobacterium tuberculosis;	NCBI: 1773	4389894	99.94	0.00	Yes
NCBI Assembly	GCA_001947745.1	NCBI: cellular organisms; Bacteria; Terrabacteria group; Actinobacteria; Actinomycetia; Corynebacteriales; Mycobacteriaceae; Mycobacterium; Mycobacterium tuberculosis complex; Mycobacterium tuberculosis;	NCBI: 1773	4385409	99.94	0.00	Yes
NCBI Assembly	GCA_002131015.1	NCBI: cellular organisms; Bacteria; Terrabacteria group; Actinobacteria; Actinomycetia; Corynebacteriales; Mycobacteriaceae; Mycobacterium; Mycobacterium tuberculosis complex; Mycobacterium tuberculosis;	NCBI: 1773	4395326	99.94	0.00	Yes
NCBI Assembly	GCA_001945865.1	NCBI: cellular organisms; Bacteria; Terrabacteria group; Actinobacteria; Actinomycetia; Corynebacteriales; Mycobacteriaceae; Mycobacterium; Mycobacterium tuberculosis complex; Mycobacterium tuberculosis;	NCBI: 1773	4410073	99.61	0.00	Yes
NCBI Assembly	GCA_001997935.1	NCBI: cellular organisms; Bacteria; Terrabacteria group; Actinobacteria; Actinomycetia; Corynebacteriales; Mycobacteriaceae; Mycobacterium; Mycobacterium tuberculosis complex; Mycobacterium tuberculosis;	NCBI: 1334067	4411532	99.94	0.00	Yes
NCBI Assembly	GCA_002800045.1	NCBI: cellular organisms; Bacteria; Terrabacteria group; Actinobacteria; Actinomycetia; Corynebacteriales; Mycobacteriaceae; Mycobacteroides; Mycobacteroides abscessus; Mycobacteroides abscessus subsp. massiliense;	NCBI: 1962118	4939423	100.00	0.00	Yes
NCBI Assembly	GCA_002086475.1	NCBI: cellular organisms; Bacteria; Terrabacteria group; Actinobacteria; Actinomycetia; Corynebacteriales; Mycobacteriaceae; Mycobacterium; Mycobacterium paraseoulense;	NCBI: 590652	6078492	99.94	0.97	Yes
NCBI Assembly	GCA_001947125.1	NCBI: cellular organisms; Bacteria; Terrabacteria group; Actinobacteria; Actinomycetia; Corynebacteriales; Mycobacteriaceae; Mycobacterium; Mycobacterium tuberculosis complex; Mycobacterium tuberculosis;	NCBI: 1773	4390143	99.94	0.00	Yes
NCBI Assembly	GCA_001949015.1	NCBI: cellular organisms; Bacteria; Terrabacteria group; Actinobacteria; Actinomycetia; Corynebacteriales; Mycobacteriaceae; Mycobacterium; Mycobacterium tuberculosis complex; Mycobacterium tuberculosis;	NCBI: 1773	4392541	99.94	0.00	Yes
NCBI Assembly	GCA_002447435.1	NCBI: cellular organisms; Bacteria; Terrabacteria group; Actinobacteria; Actinomycetia; Corynebacteriales; Mycobacteriaceae; Mycobacterium; Mycobacterium tuberculosis complex; Mycobacterium tuberculosis;	NCBI: 1773	4411230	99.94	0.00	Yes
NCBI Assembly	GCA_002128845.1	NCBI: cellular organisms; Bacteria; Terrabacteria group; Actinobacteria; Actinomycetia; Corynebacteriales; Mycobacteriaceae; Mycobacterium; Mycobacterium tuberculosis complex; Mycobacterium tuberculosis;	NCBI: 1773	4392745	99.78	0.00	Yes
NCBI Assembly	GCA_001946505.1	NCBI: cellular organisms; Bacteria; Terrabacteria group; Actinobacteria; Actinomycetia; Corynebacteriales; Mycobacteriaceae; Mycobacterium; Mycobacterium tuberculosis complex; Mycobacterium tuberculosis;	NCBI: 1773	4352930	99.94	0.00	Yes
NCBI Assembly	GCA_001943985.1	NCBI: cellular organisms; Bacteria; Terrabacteria group; Actinobacteria; Actinomycetia; Corynebacteriales; Mycobacteriaceae; Mycobacterium; Mycobacterium tuberculosis complex; Mycobacterium tuberculosis;	NCBI: 1773	4376270	99.94	0.00	Yes
NCBI Assembly	GCA_001947755.1	NCBI: cellular organisms; Bacteria; Terrabacteria group; Actinobacteria; Actinomycetia; Corynebacteriales; Mycobacteriaceae; Mycobacterium; Mycobacterium tuberculosis complex; Mycobacterium tuberculosis;	NCBI: 1773	4401445	99.94	0.00	Yes
NCBI Assembly	GCA_002131025.1	NCBI: cellular organisms; Bacteria; Terrabacteria group; Actinobacteria; Actinomycetia; Corynebacteriales; Mycobacteriaceae; Mycobacterium; Mycobacterium tuberculosis complex; Mycobacterium tuberculosis;	NCBI: 1773	4399321	99.94	0.00	Yes
NCBI Assembly	GCA_001945875.1	NCBI: cellular organisms; Bacteria; Terrabacteria group; Actinobacteria; Actinomycetia; Corynebacteriales; Mycobacteriaceae; Mycobacterium; Mycobacterium tuberculosis complex; Mycobacterium tuberculosis;	NCBI: 1773	4387371	99.94	0.00	Yes
NCBI Assembly	GCA_001997975.1	NCBI: cellular organisms; Bacteria; Terrabacteria group; Actinobacteria; Actinomycetia; Corynebacteriales; Mycobacteriaceae; Mycobacterium; Mycobacterium tuberculosis complex; Mycobacterium tuberculosis;	NCBI: 1334065	4411532	99.94	0.00	Yes
NCBI Assembly	GCA_002800055.1	NCBI: cellular organisms; Bacteria; Terrabacteria group; Actinobacteria; Actinomycetia; Corynebacteriales; Mycobacteriaceae; Mycobacteroides; Mycobacteroides abscessus; Mycobacteroides abscessus subsp. massiliense;	NCBI: 1962118	5039966	100.00	0.00	Yes

NCBI Assembly	GCA_001947135.1	NCBI: cellular organisms; Bacteria; Terrabacteria group; Actinobacteria; Actinomycetia; Corynebacteriales; Mycobacteriaceae; Mycobacterium; Mycobacterium tuberculosis complex; Mycobacterium tuberculosis;	NCBI: 1773	4390705	99.90	0.00	Yes
NCBI Assembly	GCA_001946515.1	NCBI: cellular organisms; Bacteria; Terrabacteria group; Actinobacteria; Actinomycetia; Corynebacteriales; Mycobacteriaceae; Mycobacterium; Mycobacterium tuberculosis complex; Mycobacterium tuberculosis;	NCBI: 1773	4383961	99.94	0.00	Yes
NCBI Assembly	GCA_001949025.1	NCBI: cellular organisms; Bacteria; Terrabacteria group; Actinobacteria; Actinomycetia; Corynebacteriales; Mycobacteriaceae; Mycobacterium; Mycobacterium tuberculosis complex; Mycobacterium tuberculosis;	NCBI: 1773	4391336	99.94	0.00	Yes
NCBI Assembly	GCA_002086485.1	NCBI: cellular organisms; Bacteria; Terrabacteria group; Actinobacteria; Actinomycetia; Corynebacteriales; Mycobacteriaceae; Mycobacterium; Mycobacterium aquaticum;	NCBI: 1927124	7927592	100.00	2.92	Yes
NCBI Assembly	GCA_002128855.1	NCBI: cellular organisms; Bacteria; Terrabacteria group; Actinobacteria; Actinomycetia; Corynebacteriales; Mycobacteriaceae; Mycobacterium; Mycobacterium tuberculosis complex; Mycobacterium tuberculosis;	NCBI: 1773	4374704	99.94	0.00	Yes
NCBI Assembly	GCA_002447455.1	NCBI: cellular organisms; Bacteria; Terrabacteria group; Actinobacteria; Actinomycetia; Corynebacteriales; Mycobacteriaceae; Mycobacterium; Mycobacterium tuberculosis complex; Mycobacterium tuberculosis;	NCBI: 1773	4411412	99.94	0.00	Yes
NCBI Assembly	GCA_001943995.1	NCBI: cellular organisms; Bacteria; Terrabacteria group; Actinobacteria; Actinomycetia; Corynebacteriales; Mycobacteriaceae; Mycobacterium; Mycobacterium tuberculosis complex; Mycobacterium tuberculosis;	NCBI: 1773	4384273	99.94	0.00	Yes
NCBI Assembly	GCA_001947765.1	NCBI: cellular organisms; Bacteria; Terrabacteria group; Actinobacteria; Actinomycetia; Corynebacteriales; Mycobacteriaceae; Mycobacterium; Mycobacterium tuberculosis complex; Mycobacterium tuberculosis;	NCBI: 1773	4398819	99.94	0.00	Yes
NCBI Assembly	GCA_002131055.1	NCBI: cellular organisms; Bacteria; Terrabacteria group; Actinobacteria; Actinomycetia; Corynebacteriales; Mycobacteriaceae; Mycobacterium; Mycobacterium tuberculosis complex; Mycobacterium tuberculosis;	NCBI: 1773	4371035	99.94	0.00	Yes
NCBI Assembly	GCA_001945925.1	NCBI: cellular organisms; Bacteria; Terrabacteria group; Actinobacteria; Actinomycetia; Corynebacteriales; Mycobacteriaceae; Mycobacterium; Mycobacterium tuberculosis complex; Mycobacterium tuberculosis;	NCBI: 1773	4402503	99.94	0.00	Yes
NCBI Assembly	GCA_001998025.1	NCBI: cellular organisms; Bacteria; Terrabacteria group; Actinobacteria; Actinomycetia; Corynebacteriales; Mycobacteriaceae; Mycobacterium; Mycobacterium tuberculosis complex; Mycobacterium tuberculosis;	NCBI: 1334053	4411532	99.94	0.00	Yes
NCBI Assembly	GCA_001947185.1	NCBI: cellular organisms; Bacteria; Terrabacteria group; Actinobacteria; Actinomycetia; Corynebacteriales; Mycobacteriaceae; Mycobacterium; Mycobacterium tuberculosis complex; Mycobacterium tuberculosis;	NCBI: 1773	4393975	99.94	0.00	Yes
NCBI Assembly	GCA_002800065.1	NCBI: cellular organisms; Bacteria; Terrabacteria group; Actinobacteria; Actinomycetia; Corynebacteriales; Mycobacteriaceae; Mycobacteroides; Mycobacteroides abscessus; Mycobacteroides abscessus subsp. massiliense;	NCBI: 1962118	4884609	100.00	0.00	Yes
NCBI Assembly	GCA_001946565.1	NCBI: cellular organisms; Bacteria; Terrabacteria group; Actinobacteria; Actinomycetia; Corynebacteriales; Mycobacteriaceae; Mycobacterium; Mycobacterium tuberculosis complex; Mycobacterium tuberculosis;	NCBI: 1773	4369262	99.94	0.00	Yes
NCBI Assembly	GCA_002128865.1	NCBI: cellular organisms; Bacteria; Terrabacteria group; Actinobacteria; Actinomycetia; Corynebacteriales; Mycobacteriaceae; Mycobacterium; Mycobacterium tuberculosis complex; Mycobacterium tuberculosis;	NCBI: 1773	4392860	99.94	0.00	Yes
NCBI Assembly	GCA_002086515.1	NCBI: cellular organisms; Bacteria; Terrabacteria group; Actinobacteria; Actinomycetia; Corynebacteriales; Mycobacteriaceae; Mycolicibacter; Mycolicibacter arupensis;	NCBI: 342002	4442590	99.55	0.61	Yes
NCBI Assembly	GCA_001949045.1	NCBI: cellular organisms; Bacteria; Terrabacteria group; Actinobacteria; Actinomycetia; Corynebacteriales; Mycobacteriaceae; Mycobacterium; Mycobacterium tuberculosis complex; Mycobacterium tuberculosis;	NCBI: 1773	4380390	99.94	0.07	Yes
NCBI Assembly	GCA_001944045.1	NCBI: cellular organisms; Bacteria; Terrabacteria group; Actinobacteria; Actinomycetia; Corynebacteriales; Mycobacteriaceae; Mycobacterium; Mycobacterium tuberculosis complex; Mycobacterium tuberculosis;	NCBI: 1773	4373366	99.94	0.00	Yes

NCBI Assembly	GCA_002447475.1	NCBI: cellular organisms; Bacteria; Terrabacteria group; Actinobacteria; Actinomycetia; Corynebacteriales; Mycobacteriaceae; Mycobacterium; Mycobacterium tuberculosis complex; Mycobacterium tuberculosis;	NCBI: 1773	4411415	99.94	0.00	Yes
NCBI Assembly	GCA_001945935.1	NCBI: cellular organisms; Bacteria; Terrabacteria group; Actinobacteria; Actinomycetia; Corynebacteriales; Mycobacteriaceae; Mycobacterium; Mycobacterium tuberculosis complex; Mycobacterium tuberculosis;	NCBI: 1773	4366361	99.94	0.00	Yes
NCBI Assembly	GCA_001947805.1	NCBI: cellular organisms; Bacteria; Terrabacteria group; Actinobacteria; Actinomycetia; Corynebacteriales; Mycobacteriaceae; Mycobacterium; Mycobacterium tuberculosis complex; Mycobacterium tuberculosis;	NCBI: 1773	4378712	99.94	0.00	Yes
NCBI Assembly	GCA_002131085.1	NCBI: cellular organisms; Bacteria; Terrabacteria group; Actinobacteria; Actinomycetia; Corynebacteriales; Mycobacteriaceae; Mycobacterium; Mycobacterium tuberculosis complex; Mycobacterium tuberculosis;	NCBI: 1773	4380449	99.94	0.00	Yes
NCBI Assembly	GCA_001998065.1	NCBI: cellular organisms; Bacteria; Terrabacteria group; Actinobacteria; Actinomycetia; Corynebacteriales; Mycobacteriaceae; Mycobacterium; Mycobacterium tuberculosis complex; Mycobacterium tuberculosis;	NCBI: 1773	4236478	99.86	0.00	Yes
NCBI Assembly	GCA_001947195.1	NCBI: cellular organisms; Bacteria; Terrabacteria group; Actinobacteria; Actinomycetia; Corynebacteriales; Mycobacteriaceae; Mycobacterium; Mycobacterium tuberculosis complex; Mycobacterium tuberculosis;	NCBI: 1773	4388091	99.94	0.00	Yes
NCBI Assembly	GCA_001946575.1	NCBI: cellular organisms; Bacteria; Terrabacteria group; Actinobacteria; Actinomycetia; Corynebacteriales; Mycobacteriaceae; Mycobacterium; Mycobacterium tuberculosis complex; Mycobacterium tuberculosis;	NCBI: 1773	4380547	99.94	0.00	Yes
NCBI Assembly	GCA_002128905.1	NCBI: cellular organisms; Bacteria; Terrabacteria group; Actinobacteria; Actinomycetia; Corynebacteriales; Mycobacteriaceae; Mycobacterium; Mycobacterium tuberculosis complex; Mycobacterium tuberculosis;	NCBI: 1773	4397670	99.94	0.00	Yes
NCBI Assembly	GCA_002086525.1	NCBI: cellular organisms; Bacteria; Terrabacteria group; Actinobacteria; Actinomycetia; Corynebacteriales; Mycobacteriaceae; Mycobacteroides; Mycobacteroides abscessus; Mycobacteroides abscessus subsp. bolletii;	NCBI: 319705	5061701	100.00	0.33	Yes
NCBI Assembly	GCA_002800135.1	NCBI: cellular organisms; Bacteria; Terrabacteria group; Actinobacteria; Actinomycetia; Corynebacteriales; Mycobacteriaceae; Mycobacteroides; Mycobacteroides abscessus; Mycobacteroides abscessus subsp. massiliense;	NCBI: 1962118	4965250	100.00	0.00	Yes
NCBI Assembly	GCA_001949085.1	NCBI: cellular organisms; Bacteria; Terrabacteria group; Actinobacteria; Actinomycetia; Corynebacteriales; Mycobacteriaceae; Mycobacterium; Mycobacterium tuberculosis complex; Mycobacterium tuberculosis;	NCBI: 1773	4411480	99.94	0.00	Yes
NCBI Assembly	GCA_001944055.1	NCBI: cellular organisms; Bacteria; Terrabacteria group; Actinobacteria; Actinomycetia; Corynebacteriales; Mycobacteriaceae; Mycobacterium; Mycobacterium tuberculosis complex; Mycobacterium tuberculosis;	NCBI: 1773	4375352	99.94	0.00	Yes
NCBI Assembly	GCA_001945945.1	NCBI: cellular organisms; Bacteria; Terrabacteria group; Actinobacteria; Actinomycetia; Corynebacteriales; Mycobacteriaceae; Mycobacterium; Mycobacterium tuberculosis complex; Mycobacterium tuberculosis;	NCBI: 1773	4394505	99.94	0.00	Yes
NCBI Assembly	GCA_002447495.1	NCBI: cellular organisms; Bacteria; Terrabacteria group; Actinobacteria; Actinomycetia; Corynebacteriales; Mycobacteriaceae; Mycobacterium; Mycobacterium tuberculosis complex; Mycobacterium tuberculosis;	NCBI: 1773	4411211	99.94	0.00	Yes
NCBI Assembly	GCA_001947815.1	NCBI: cellular organisms; Bacteria; Terrabacteria group; Actinobacteria; Actinomycetia; Corynebacteriales; Mycobacteriaceae; Mycobacterium; Mycobacterium tuberculosis complex; Mycobacterium tuberculosis;	NCBI: 1773	4397499	99.94	0.00	Yes
NCBI Assembly	GCA_002131105.1	NCBI: cellular organisms; Bacteria; Terrabacteria group; Actinobacteria; Actinomycetia; Corynebacteriales; Mycobacteriaceae; Mycobacterium; Mycobacterium tuberculosis complex; Mycobacterium tuberculosis;	NCBI: 1773	4408348	99.94	0.00	Yes
NCBI Assembly	GCA_002355775.1	NCBI: cellular organisms; Bacteria; Terrabacteria group; Actinobacteria; Actinomycetia; Corynebacteriales; Mycobacteriaceae; Mycobacterium; Mycobacterium ulcerans; Mycobacterium ulcerans subsp. shinshuense;	NCBI: 1124626	6066298	99.54	1.40	Yes
NCBI Assembly	GCA_001946585.1	NCBI: cellular organisms; Bacteria; Terrabacteria group; Actinobacteria; Actinomycetia; Corynebacteriales; Mycobacteriaceae; Mycobacterium; Mycobacterium tuberculosis complex; Mycobacterium tuberculosis;	NCBI: 1773	4365923	99.94	0.00	Yes

NCBI Assembly	GCA_002086575.1	NCBI: cellular organisms; Bacteria; Terrabacteria group; Actinobacteria; Actinomycetia; Corynebacteriales; Mycobacteriaceae; Mycobacterium; Mycobacterium branderi;	NCBI: 43348	5903509	100.00	0.61	Yes
NCBI Assembly	GCA_002448215.1	NCBI: cellular organisms; Bacteria; Terrabacteria group; Actinobacteria; Actinomycetia; Corynebacteriales; Mycobacteriaceae; Mycobacterium; Mycobacterium tuberculosis complex; Mycobacterium tuberculosis;	NCBI: 1773	4411414	99.94	0.00	Yes
NCBI Assembly	GCA_002128925.1	NCBI: cellular organisms; Bacteria; Terrabacteria group; Actinobacteria; Actinomycetia; Corynebacteriales; Mycobacteriaceae; Mycobacterium; Mycobacterium tuberculosis complex; Mycobacterium tuberculosis;	NCBI: 1773	4381183	99.94	0.00	Yes
NCBI Assembly	GCA_001944705.1	NCBI: cellular organisms; Bacteria; Terrabacteria group; Actinobacteria; Actinomycetia; Corynebacteriales; Mycobacteriaceae; Mycobacterium; Mycobacterium tuberculosis complex; Mycobacterium tuberculosis;	NCBI: 1773	4382800	99.94	0.00	Yes
NCBI Assembly	GCA_001948155.1	NCBI: cellular organisms; Bacteria; Terrabacteria group; Actinobacteria; Actinomycetia; Corynebacteriales; Mycobacteriaceae; Mycobacterium; Mycobacterium tuberculosis complex; Mycobacterium tuberculosis;	NCBI: 1773	4393709	99.94	0.00	Yes
NCBI Assembly	GCA_001945045.1	NCBI: cellular organisms; Bacteria; Terrabacteria group; Actinobacteria; Actinomycetia; Corynebacteriales; Mycobacteriaceae; Mycobacterium; Mycobacterium tuberculosis complex; Mycobacterium tuberculosis;	NCBI: 1773	4375691	99.94	0.00	Yes
NCBI Assembly	GCA_002800155.1	NCBI: cellular organisms; Bacteria; Terrabacteria group; Actinobacteria; Actinomycetia; Corynebacteriales; Mycobacteriaceae; Mycobacteroides; Mycobacteroides abscessus; Mycobacteroides abscessus subsp. massiliense;	NCBI: 1962118	5094009	100.00	0.08	Yes
NCBI Assembly	GCA_001947825.1	NCBI: cellular organisms; Bacteria; Terrabacteria group; Actinobacteria; Actinomycetia; Corynebacteriales; Mycobacteriaceae; Mycobacterium; Mycobacterium tuberculosis complex; Mycobacterium tuberculosis;	NCBI: 1773	4378622	99.94	0.00	Yes
NCBI Assembly	GCA_002447515.1	NCBI: cellular organisms; Bacteria; Terrabacteria group; Actinobacteria; Actinomycetia; Corynebacteriales; Mycobacteriaceae; Mycobacterium; Mycobacterium tuberculosis complex; Mycobacterium tuberculosis;	NCBI: 1773	4411475	99.94	0.00	Yes
NCBI Assembly	GCA_002131115.1	NCBI: cellular organisms; Bacteria; Terrabacteria group; Actinobacteria; Actinomycetia; Corynebacteriales; Mycobacteriaceae; Mycobacterium; Mycobacterium tuberculosis complex; Mycobacterium tuberculosis;	NCBI: 1773	4403173	99.94	0.00	Yes
NCBI Assembly	GCA_002356015.1	NCBI: cellular organisms; Bacteria; Terrabacteria group; Actinobacteria; Actinomycetia; Corynebacteriales; Mycobacteriaceae; Mycobacterium; Mycobacterium tuberculosis complex; Mycobacterium tuberculosis;	NCBI: 1773	4380602	99.94	0.00	Yes
NCBI Assembly	GCA_001946605.1	NCBI: cellular organisms; Bacteria; Terrabacteria group; Actinobacteria; Actinomycetia; Corynebacteriales; Mycobacteriaceae; Mycobacterium; Mycobacterium tuberculosis complex; Mycobacterium tuberculosis;	NCBI: 1773	4412441	99.94	0.00	Yes
NCBI Assembly	GCA_002086605.1	NCBI: cellular organisms; Bacteria; Terrabacteria group; Actinobacteria; Actinomycetia; Corynebacteriales; Mycobacteriaceae; Mycolicibacterium; Mycolicibacterium elephantis;	NCBI: 81858	5313551	100.00	1.14	Yes
NCBI Assembly	GCA_001944715.1	NCBI: cellular organisms; Bacteria; Terrabacteria group; Actinobacteria; Actinomycetia; Corynebacteriales; Mycobacteriaceae; Mycobacterium; Mycobacterium tuberculosis complex; Mycobacterium tuberculosis;	NCBI: 1773	4389417	99.94	0.00	Yes
NCBI Assembly	GCA_002129585.1	NCBI: cellular organisms; Bacteria; Terrabacteria group; Actinobacteria; Actinomycetia; Corynebacteriales; Mycobacteriaceae; Mycobacterium; Mycobacterium tuberculosis complex; Mycobacterium tuberculosis;	NCBI: 1773	4404704	99.94	0.00	Yes
NCBI Assembly	GCA_001948165.1	NCBI: cellular organisms; Bacteria; Terrabacteria group; Actinobacteria; Actinomycetia; Corynebacteriales; Mycobacteriaceae; Mycobacterium; Mycobacterium tuberculosis complex; Mycobacterium tuberculosis;	NCBI: 1773	4384052	99.94	0.00	Yes
NCBI Assembly	GCA_001945065.1	NCBI: cellular organisms; Bacteria; Terrabacteria group; Actinobacteria; Actinomycetia; Corynebacteriales; Mycobacteriaceae; Mycobacterium; Mycobacterium tuberculosis complex; Mycobacterium tuberculosis;	NCBI: 1773	4382398	99.94	0.00	Yes
NCBI Assembly	GCA_002800165.1	NCBI: cellular organisms; Bacteria; Terrabacteria group; Actinobacteria; Actinomycetia; Corynebacteriales; Mycobacteriaceae; Mycobacteroides; Mycobacteroides abscessus; Mycobacteroides abscessus subsp. abscessus;	NCBI: 1185650	5101392	99.92	0.25	Yes

NCBI Assembly	GCA_002447535.1	NCBI: cellular organisms; Bacteria; Terrabacteria group; Actinobacteria; Actinomycetia; Corynebacteriales; Mycobacteriaceae; Mycobacterium; Mycobacterium tuberculosis complex; Mycobacterium tuberculosis;	NCBI: 1773	4411243	99.94	0.00	Yes
NCBI Assembly	GCA_002101825.1	NCBI: cellular organisms; Bacteria; Terrabacteria group; Actinobacteria; Actinomycetia; Corynebacteriales; Mycobacteriaceae; Mycobacterium; Mycobacterium simiae complex; Mycobacterium paraense;	NCBI: 767916	5765665	99.94	1.73	Yes
NCBI Assembly	GCA_002130195.1	NCBI: cellular organisms; Bacteria; Terrabacteria group; Actinobacteria; Actinomycetia; Corynebacteriales; Mycobacteriaceae; Mycobacterium; Mycobacterium tuberculosis complex; Mycobacterium tuberculosis;	NCBI: 1773	4349402	99.94	0.00	Yes
NCBI Assembly	GCA_002356255.1	NCBI: cellular organisms; Bacteria; Terrabacteria group; Actinobacteria; Actinomycetia; Corynebacteriales; Mycobacteriaceae; Mycobacterium; Mycobacterium tuberculosis complex; Mycobacterium tuberculosis;	NCBI: 1773	4399916	99.94	0.00	Yes
NCBI Assembly	GCA_001946645.1	NCBI: cellular organisms; Bacteria; Terrabacteria group; Actinobacteria; Actinomycetia; Corynebacteriales; Mycobacteriaceae; Mycobacterium; Mycobacterium tuberculosis complex; Mycobacterium tuberculosis;	NCBI: 1773	4348033	99.94	0.00	Yes
NCBI Assembly	GCA_002129605.1	NCBI: cellular organisms; Bacteria; Terrabacteria group; Actinobacteria; Actinomycetia; Corynebacteriales; Mycobacteriaceae; Mycobacterium; Mycobacterium tuberculosis complex; Mycobacterium tuberculosis;	NCBI: 1773	4421358	99.94	0.00	Yes
NCBI Assembly	GCA_002089395.1	NCBI: cellular organisms; Bacteria; Terrabacteria group; Actinobacteria; Actinomycetia; Corynebacteriales; Mycobacteriaceae; Mycobacterium; Mycobacterium avium complex (MAC); Mycobacterium avium; Mycobacterium avium subsp. hominissuis;	NCBI: 439334	5752685	99.83	0.75	Yes
NCBI Assembly	GCA_001944765.1	NCBI: cellular organisms; Bacteria; Terrabacteria group; Actinobacteria; Actinomycetia; Corynebacteriales; Mycobacteriaceae; Mycobacterium; Mycobacterium tuberculosis complex; Mycobacterium tuberculosis;	NCBI: 1773	4343351	99.94	0.00	Yes
NCBI Assembly	GCA_001948205.1	NCBI: cellular organisms; Bacteria; Terrabacteria group; Actinobacteria; Actinomycetia; Corynebacteriales; Mycobacteriaceae; Mycobacterium; Mycobacterium tuberculosis complex; Mycobacterium tuberculosis;	NCBI: 1773	4400453	99.94	0.00	Yes
NCBI Assembly	GCA_001945075.1	NCBI: cellular organisms; Bacteria; Terrabacteria group; Actinobacteria; Actinomycetia; Corynebacteriales; Mycobacteriaceae; Mycobacterium; Mycobacterium tuberculosis complex; Mycobacterium tuberculosis;	NCBI: 1773	4392723	99.94	0.00	Yes
NCBI Assembly	GCA_002800205.1	NCBI: cellular organisms; Bacteria; Terrabacteria group; Actinobacteria; Actinomycetia; Corynebacteriales; Mycobacteriaceae; Mycobacteroides; Mycobacteroides abscessus; Mycobacteroides abscessus subsp. abscessus;	NCBI: 1185650	5073566	100.00	0.33	Yes
NCBI Assembly	GCA_002447555.1	NCBI: cellular organisms; Bacteria; Terrabacteria group; Actinobacteria; Actinomycetia; Corynebacteriales; Mycobacteriaceae; Mycobacterium; Mycobacterium tuberculosis complex; Mycobacterium tuberculosis;	NCBI: 1773	4411137	99.94	0.00	Yes
NCBI Assembly	GCA_002130215.1	NCBI: cellular organisms; Bacteria; Terrabacteria group; Actinobacteria; Actinomycetia; Corynebacteriales; Mycobacteriaceae; Mycobacterium; Mycobacterium tuberculosis complex; Mycobacterium tuberculosis;	NCBI: 1773	4387992	99.94	0.00	Yes
NCBI Assembly	GCA_002101845.1	NCBI: cellular organisms; Bacteria; Terrabacteria group; Actinobacteria; Actinomycetia; Corynebacteriales; Mycobacteriaceae; Mycobacterium; Mycobacterium riyadhense;	NCBI: 486698	6269850	99.94	0.53	Yes
NCBI Assembly	GCA_002356315.1	NCBI: cellular organisms; Bacteria; Terrabacteria group; Actinobacteria; Actinomycetia; Corynebacteriales; Mycobacteriaceae; Mycobacterium; Mycobacterium simiae complex; Mycobacterium shigaense;	NCBI: 722731	5232660	99.17	0.88	Yes
NCBI Assembly	GCA_001946655.1	NCBI: cellular organisms; Bacteria; Terrabacteria group; Actinobacteria; Actinomycetia; Corynebacteriales; Mycobacteriaceae; Mycobacterium; Mycobacterium tuberculosis complex; Mycobacterium tuberculosis;	NCBI: 1773	4409201	99.94	0.00	Yes
NCBI Assembly	GCA_002129625.1	NCBI: cellular organisms; Bacteria; Terrabacteria group; Actinobacteria; Actinomycetia; Corynebacteriales; Mycobacteriaceae; Mycobacterium; Mycobacterium tuberculosis complex; Mycobacterium tuberculosis;	NCBI: 1773	4393887	99.94	0.00	Yes
NCBI Assembly	GCA_001944775.1	NCBI: cellular organisms; Bacteria; Terrabacteria group; Actinobacteria; Actinomycetia; Corynebacteriales; Mycobacteriaceae; Mycobacterium; Mycobacterium tuberculosis complex; Mycobacterium tuberculosis;	NCBI: 1773	4342709	99.94	0.00	Yes

NCBI Assembly	GCA_001945085.1	NCBI: cellular organisms; Bacteria; Terrabacteria group; Actinobacteria; Actinomycetia; Corynebacteriales; Mycobacteriaceae; Mycobacterium; Mycobacterium tuberculosis complex; Mycobacterium tuberculosis;	NCBI: 1773	4375589	99.94	0.00	Yes
NCBI Assembly	GCA_001948215.1	NCBI: cellular organisms; Bacteria; Terrabacteria group; Actinobacteria; Actinomycetia; Corynebacteriales; Mycobacteriaceae; Mycobacterium; Mycobacterium tuberculosis complex; Mycobacterium tuberculosis;	NCBI: 1773	4377099	99.94	0.00	Yes
NCBI Assembly	GCA_002089415.1	NCBI: cellular organisms; Bacteria; Terrabacteria group; Actinobacteria; Actinomycetia; Corynebacteriales; Mycobacteriaceae; Mycobacterium; Mycobacterium avium complex (MAC); Mycobacterium avium; Mycobacterium avium subsp. hominissuis;	NCBI: 439334	5439676	99.06	0.00	Yes
NCBI Assembly	GCA_001947865.1	NCBI: cellular organisms; Bacteria; Terrabacteria group; Actinobacteria; Actinomycetia; Corynebacteriales; Mycobacteriaceae; Mycobacterium; Mycobacterium tuberculosis complex; Mycobacterium tuberculosis;	NCBI: 1773	4391558	99.94	0.00	Yes
NCBI Assembly	GCA_002800225.1	NCBI: cellular organisms; Bacteria; Terrabacteria group; Actinobacteria; Actinomycetia; Corynebacteriales; Mycobacteriaceae; Mycobacteroides; Mycobacteroides abscessus; Mycobacteroides abscessus subsp. abscessus;	NCBI: 1185650	5111127	100.00	0.00	Yes
NCBI Assembly	GCA_002130245.1	NCBI: cellular organisms; Bacteria; Terrabacteria group; Actinobacteria; Actinomycetia; Corynebacteriales; Mycobacteriaceae; Mycobacterium; Mycobacterium tuberculosis complex; Mycobacterium tuberculosis;	NCBI: 1773	4353715	99.94	0.00	Yes
NCBI Assembly	GCA_002356335.1	NCBI: cellular organisms; Bacteria; Terrabacteria group; Actinobacteria; Actinomycetia; Corynebacteriales; Mycobacteriaceae; Mycobacteroides; [Mycobacterium] stephanolepidis;	NCBI: 1520670	4994485	99.33	0.36	Yes
NCBI Assembly	GCA_001946675.1	NCBI: cellular organisms; Bacteria; Terrabacteria group; Actinobacteria; Actinomycetia; Corynebacteriales; Mycobacteriaceae; Mycobacterium; Mycobacterium tuberculosis complex; Mycobacterium tuberculosis;	NCBI: 1773	4401082	99.94	0.00	Yes
NCBI Assembly	GCA_001944795.1	NCBI: cellular organisms; Bacteria; Terrabacteria group; Actinobacteria; Actinomycetia; Corynebacteriales; Mycobacteriaceae; Mycobacterium; Mycobacterium tuberculosis complex; Mycobacterium tuberculosis;	NCBI: 1773	4383006	99.94	0.00	Yes
NCBI Assembly	GCA_002129635.1	NCBI: cellular organisms; Bacteria; Terrabacteria group; Actinobacteria; Actinomycetia; Corynebacteriales; Mycobacteriaceae; Mycobacterium; Mycobacterium tuberculosis complex; Mycobacterium tuberculosis;	NCBI: 1773	4406139	99.94	0.00	Yes
NCBI Assembly	GCA_002101875.1	NCBI: cellular organisms; Bacteria; Terrabacteria group; Actinobacteria; Actinomycetia; Corynebacteriales; Mycobacteriaceae; Mycobacterium; Mycobacterium simiae complex; Mycobacterium saskatchewanense;	NCBI: 220927	5930935	99.89	1.81	Yes
NCBI Assembly	GCA_001945125.1	NCBI: cellular organisms; Bacteria; Terrabacteria group; Actinobacteria; Actinomycetia; Corynebacteriales; Mycobacteriaceae; Mycobacterium; Mycobacterium tuberculosis complex; Mycobacterium tuberculosis;	NCBI: 1773	4392625	99.94	0.00	Yes
NCBI Assembly	GCA_002089435.1	NCBI: cellular organisms; Bacteria; Terrabacteria group; Actinobacteria; Actinomycetia; Corynebacteriales; Mycobacteriaceae; Mycobacterium; Mycobacterium avium complex (MAC); Mycobacterium avium; Mycobacterium avium subsp. hominissuis;	NCBI: 439334	5192099	99.67	0.00	Yes
NCBI Assembly	GCA_001948225.1	NCBI: cellular organisms; Bacteria; Terrabacteria group; Actinobacteria; Actinomycetia; Corynebacteriales; Mycobacteriaceae; Mycobacterium; Mycobacterium tuberculosis complex; Mycobacterium tuberculosis;	NCBI: 1773	4348820	99.94	0.00	Yes
NCBI Assembly	GCA_001947885.1	NCBI: cellular organisms; Bacteria; Terrabacteria group; Actinobacteria; Actinomycetia; Corynebacteriales; Mycobacteriaceae; Mycobacterium; Mycobacterium tuberculosis complex; Mycobacterium tuberculosis;	NCBI: 1773	4393211	99.94	0.00	Yes
NCBI Assembly	GCA_002705785.1	NCBI: cellular organisms; Bacteria; Terrabacteria group; Actinobacteria; Actinomycetia; Corynebacteriales; Mycobacteriaceae; Mycobacterium; Mycobacterium kansasii;	NCBI: 1768	6530604	99.90	0.71	Yes
NCBI Assembly	GCA_002130265.1	NCBI: cellular organisms; Bacteria; Terrabacteria group; Actinobacteria; Actinomycetia; Corynebacteriales; Mycobacteriaceae; Mycobacterium; Mycobacterium tuberculosis complex; Mycobacterium tuberculosis;	NCBI: 1773	4386523	99.94	0.00	Yes
NCBI Assembly	GCA_002357935.1	NCBI: cellular organisms; Bacteria; Terrabacteria group; Actinobacteria; Actinomycetia; Corynebacteriales; Mycobacteriaceae; Mycobacterium; Mycobacterium tuberculosis complex; Mycobacterium tuberculosis;	NCBI: 1773	4411033	99.94	0.00	Yes

NCBI Assembly	GCA_001946705.1	NCBI: cellular organisms; Bacteria; Terrabacteria group; Actinobacteria; Actinomycetia; Corynebacteriales; Mycobacteriaceae; Mycobacterium; Mycobacterium tuberculosis complex; Mycobacterium tuberculosis;	NCBI: 1773	4397141	99.94	0.00	Yes
NCBI Assembly	GCA_001944815.1	NCBI: cellular organisms; Bacteria; Terrabacteria group; Actinobacteria; Actinomycetia; Corynebacteriales; Mycobacteriaceae; Mycobacterium; Mycobacterium tuberculosis complex; Mycobacterium tuberculosis;	NCBI: 1773	4378810	99.61	0.00	Yes
NCBI Assembly	GCA_001945145.1	NCBI: cellular organisms; Bacteria; Terrabacteria group; Actinobacteria; Actinomycetia; Corynebacteriales; Mycobacteriaceae; Mycobacterium; Mycobacterium tuberculosis complex; Mycobacterium tuberculosis;	NCBI: 1773	4398123	99.94	0.00	Yes
NCBI Assembly	GCA_002101885.1	NCBI: cellular organisms; Bacteria; Terrabacteria group; Actinobacteria; Actinomycetia; Corynebacteriales; Mycobacteriaceae; Mycolicibacter; Mycolicibacter senuensis;	NCBI: 386913	4534628	100.00	0.15	Yes
NCBI Assembly	GCA_002129665.1	NCBI: cellular organisms; Bacteria; Terrabacteria group; Actinobacteria; Actinomycetia; Corynebacteriales; Mycobacteriaceae; Mycobacterium; Mycobacterium tuberculosis complex; Mycobacterium tuberculosis;	NCBI: 1773	4391229	99.94	0.00	Yes
NCBI Assembly	GCA_001948255.1	NCBI: cellular organisms; Bacteria; Terrabacteria group; Actinobacteria; Actinomycetia; Corynebacteriales; Mycobacteriaceae; Mycobacterium; Mycobacterium tuberculosis complex; Mycobacterium tuberculosis;	NCBI: 1773	4335946	99.94	0.00	Yes
NCBI Assembly	GCA_002089455.1	NCBI: cellular organisms; Bacteria; Terrabacteria group; Actinobacteria; Actinomycetia; Corynebacteriales; Mycobacteriaceae; Mycobacterium; Mycobacterium avium complex (MAC); Mycobacterium avium; Mycobacterium avium subsp. hominissuis;	NCBI: 439334	5384255	99.83	0.33	Yes
NCBI Assembly	GCA_001947895.1	NCBI: cellular organisms; Bacteria; Terrabacteria group; Actinobacteria; Actinomycetia; Corynebacteriales; Mycobacteriaceae; Mycobacterium; Mycobacterium tuberculosis complex; Mycobacterium tuberculosis;	NCBI: 1773	4387910	99.94	0.00	Yes
NCBI Assembly	GCA_002130275.1	NCBI: cellular organisms; Bacteria; Terrabacteria group; Actinobacteria; Actinomycetia; Corynebacteriales; Mycobacteriaceae; Mycobacterium; Mycobacterium tuberculosis complex; Mycobacterium tuberculosis;	NCBI: 1773	4388603	99.94	0.00	Yes
NCBI Assembly	GCA_002705825.1	NCBI: cellular organisms; Bacteria; Terrabacteria group; Actinobacteria; Actinomycetia; Corynebacteriales; Mycobacteriaceae; Mycobacterium; Mycobacterium kansasii;	NCBI: 1768	6679778	99.90	0.71	Yes
NCBI Assembly	GCA_001946725.1	NCBI: cellular organisms; Bacteria; Terrabacteria group; Actinobacteria; Actinomycetia; Corynebacteriales; Mycobacteriaceae; Mycobacterium; Mycobacterium tuberculosis complex; Mycobacterium tuberculosis;	NCBI: 1773	4364731	99.94	0.00	Yes
NCBI Assembly	GCA_002553495.1	NCBI: cellular organisms; Bacteria; Terrabacteria group; Actinobacteria; Actinomycetia; Corynebacteriales; Mycobacteriaceae; Mycolicibacterium; Mycolicibacterium diernhoferi;	NCBI: 1801	5976307	99.34	0.19	Yes
NCBI Assembly	GCA_002357955.1	NCBI: cellular organisms; Bacteria; Terrabacteria group; Actinobacteria; Actinomycetia; Corynebacteriales; Mycobacteriaceae; Mycobacterium; Mycobacterium tuberculosis complex; Mycobacterium tuberculosis;	NCBI: 1773	4421540	99.94	0.00	Yes
NCBI Assembly	GCA_001945155.1	NCBI: cellular organisms; Bacteria; Terrabacteria group; Actinobacteria; Actinomycetia; Corynebacteriales; Mycobacteriaceae; Mycobacterium; Mycobacterium tuberculosis complex; Mycobacterium tuberculosis;	NCBI: 1773	4390081	99.94	0.00	Yes
NCBI Assembly	GCA_001944845.1	NCBI: cellular organisms; Bacteria; Terrabacteria group; Actinobacteria; Actinomycetia; Corynebacteriales; Mycobacteriaceae; Mycobacterium; Mycobacterium tuberculosis complex; Mycobacterium tuberculosis;	NCBI: 1773	4382745	99.94	0.00	Yes
NCBI Assembly	GCA_002101905.1	NCBI: cellular organisms; Bacteria; Terrabacteria group; Actinobacteria; Actinomycetia; Corynebacteriales; Mycobacteriaceae; Mycobacterium; Mycobacterium shimoidei;	NCBI: 29313	4713740	99.55	0.45	Yes
NCBI Assembly	GCA_002129685.1	NCBI: cellular organisms; Bacteria; Terrabacteria group; Actinobacteria; Actinomycetia; Corynebacteriales; Mycobacteriaceae; Mycobacterium; Mycobacterium tuberculosis complex; Mycobacterium tuberculosis;	NCBI: 1773	4395984	99.94	0.00	Yes
NCBI Assembly	GCA_001948285.1	NCBI: cellular organisms; Bacteria; Terrabacteria group; Actinobacteria; Actinomycetia; Corynebacteriales; Mycobacteriaceae; Mycobacterium; Mycobacterium tuberculosis complex; Mycobacterium tuberculosis;	NCBI: 1773	4381435	99.94	0.00	Yes

NCBI Assembly	GCA_002089475.1	NCBI: cellular organisms; Bacteria; Terrabacteria group; Actinobacteria; Actinomycetia; Corynebacteriales; Mycobacteriaceae; Mycobacterium; Mycobacterium avium complex (MAC); Mycobacterium avium; Mycobacterium avium subsp. hominissuis;	NCBI: 439334	5358590	99.39	0.64	Yes
NCBI Assembly	GCA_001947905.1	NCBI: cellular organisms; Bacteria; Terrabacteria group; Actinobacteria; Actinomycetia; Corynebacteriales; Mycobacteriaceae; Mycobacterium; Mycobacterium tuberculosis complex; Mycobacterium tuberculosis;	NCBI: 1773	4356934	99.94	0.00	Yes
NCBI Assembly	GCA_002130305.1	NCBI: cellular organisms; Bacteria; Terrabacteria group; Actinobacteria; Actinomycetia; Corynebacteriales; Mycobacteriaceae; Mycobacterium; Mycobacterium tuberculosis complex; Mycobacterium tuberculosis;	NCBI: 1773	4379077	99.94	0.00	Yes
NCBI Assembly	GCA_001946745.1	NCBI: cellular organisms; Bacteria; Terrabacteria group; Actinobacteria; Actinomycetia; Corynebacteriales; Mycobacteriaceae; Mycobacterium; Mycobacterium tuberculosis complex; Mycobacterium tuberculosis;	NCBI: 1773	4379670	99.94	0.00	Yes
NCBI Assembly	GCA_002101935.1	NCBI: cellular organisms; Bacteria; Terrabacteria group; Actinobacteria; Actinomycetia; Corynebacteriales; Mycobacteriaceae; Mycobacterium; unclassified Mycobacterium;	NCBI: 1743230	6111348	99.94	4.55	Yes
NCBI Assembly	GCA_001944855.1	NCBI: cellular organisms; Bacteria; Terrabacteria group; Actinobacteria; Actinomycetia; Corynebacteriales; Mycobacteriaceae; Mycobacterium; Mycobacterium tuberculosis complex; Mycobacterium tuberculosis;	NCBI: 1773	4407412	99.94	0.00	Yes
NCBI Assembly	GCA_002357975.1	NCBI: cellular organisms; Bacteria; Terrabacteria group; Actinobacteria; Actinomycetia; Corynebacteriales; Mycobacteriaceae; Mycobacterium; Mycobacterium tuberculosis complex; Mycobacterium tuberculosis;	NCBI: 1773	4413362	99.94	0.00	Yes
NCBI Assembly	GCA_001945165.1	NCBI: cellular organisms; Bacteria; Terrabacteria group; Actinobacteria; Actinomycetia; Corynebacteriales; Mycobacteriaceae; Mycobacterium; Mycobacterium tuberculosis complex; Mycobacterium tuberculosis;	NCBI: 1773	4386443	99.61	0.00	Yes
NCBI Assembly	GCA_002553505.1	NCBI: cellular organisms; Bacteria; Terrabacteria group; Actinobacteria; Actinomycetia; Corynebacteriales; Mycobacteriaceae; Mycolicibacterium; Mycolicibacterium agri;	NCBI: 36811	6987256	100.00	1.36	Yes
NCBI Assembly	GCA_002705835.1	NCBI: cellular organisms; Bacteria; Terrabacteria group; Actinobacteria; Actinomycetia; Corynebacteriales; Mycobacteriaceae; Mycobacterium; Mycobacterium persicum;	NCBI: 1487726	6217645	99.94	0.33	Yes
NCBI Assembly	GCA_001948295.1	NCBI: cellular organisms; Bacteria; Terrabacteria group; Actinobacteria; Actinomycetia; Corynebacteriales; Mycobacteriaceae; Mycobacterium; Mycobacterium tuberculosis complex; Mycobacterium tuberculosis;	NCBI: 1773	4396585	99.94	0.00	Yes
NCBI Assembly	GCA_002129695.1	NCBI: cellular organisms; Bacteria; Terrabacteria group; Actinobacteria; Actinomycetia; Corynebacteriales; Mycobacteriaceae; Mycobacterium; Mycobacterium tuberculosis complex; Mycobacterium tuberculosis;	NCBI: 1773	4396405	99.94	0.00	Yes
NCBI Assembly	GCA_002089495.1	NCBI: cellular organisms; Bacteria; Terrabacteria group; Actinobacteria; Actinomycetia; Corynebacteriales; Mycobacteriaceae; Mycobacterium; Mycobacterium avium complex (MAC); Mycobacterium avium; Mycobacterium avium subsp. hominissuis;	NCBI: 439334	5089743	99.66	0.44	Yes
NCBI Assembly	GCA_001947915.1	NCBI: cellular organisms; Bacteria; Terrabacteria group; Actinobacteria; Actinomycetia; Corynebacteriales; Mycobacteriaceae; Mycobacterium; Mycobacterium tuberculosis complex; Mycobacterium tuberculosis;	NCBI: 1773	4414079	99.94	0.00	Yes
NCBI Assembly	GCA_001946755.1	NCBI: cellular organisms; Bacteria; Terrabacteria group; Actinobacteria; Actinomycetia; Corynebacteriales; Mycobacteriaceae; Mycobacterium; Mycobacterium tuberculosis complex; Mycobacterium tuberculosis;	NCBI: 1773	4396373	99.94	0.00	Yes
NCBI Assembly	GCA_002130315.1	NCBI: cellular organisms; Bacteria; Terrabacteria group; Actinobacteria; Actinomycetia; Corynebacteriales; Mycobacteriaceae; Mycobacterium; Mycobacterium tuberculosis complex; Mycobacterium tuberculosis;	NCBI: 1773	4358688	99.94	0.00	Yes
NCBI Assembly	GCA_001944865.1	NCBI: cellular organisms; Bacteria; Terrabacteria group; Actinobacteria; Actinomycetia; Corynebacteriales; Mycobacteriaceae; Mycobacterium; Mycobacterium tuberculosis complex; Mycobacterium tuberculosis;	NCBI: 1773	4378041	99.94	0.00	Yes
NCBI Assembly	GCA_001945205.1	NCBI: cellular organisms; Bacteria; Terrabacteria group; Actinobacteria; Actinomycetia; Corynebacteriales; Mycobacteriaceae; Mycobacterium; Mycobacterium tuberculosis complex; Mycobacterium tuberculosis;	NCBI: 1773	4388254	99.94	0.00	Yes

NCBI Assembly	GCA_002101955.1	NCBI: cellular organisms; Bacteria; Terrabacteria group; Actinobacteria; Actinomycetia; Corynebacteriales; Mycobacteriaceae; Mycolicibacter; Mycolicibacter terrae;	NCBI: 1788	4524815	99.55	0.15	Yes
NCBI Assembly	GCA_001948305.1	NCBI: cellular organisms; Bacteria; Terrabacteria group; Actinobacteria; Actinomycetia; Corynebacteriales; Mycobacteriaceae; Mycobacterium; Mycobacterium tuberculosis complex; Mycobacterium tuberculosis;	NCBI: 1773	4380729	99.94	0.00	Yes
NCBI Assembly	GCA_002129725.1	NCBI: cellular organisms; Bacteria; Terrabacteria group; Actinobacteria; Actinomycetia; Corynebacteriales; Mycobacteriaceae; Mycobacterium; Mycobacterium tuberculosis complex; Mycobacterium tuberculosis;	NCBI: 1773	4404169	99.94	0.00	Yes
NCBI Assembly	GCA_002553535.1	NCBI: cellular organisms; Bacteria; Terrabacteria group; Actinobacteria; Actinomycetia; Corynebacteriales; Mycobacteriaceae; Mycolicibacterium; Mycolicibacterium boenickei;	NCBI: 146017	6544811	99.81	0.50	Yes
NCBI Assembly	GCA_002406255.1	NCBI: cellular organisms; Bacteria; Terrabacteria group; Actinobacteria; Actinomycetia; Corynebacteriales; Mycobacteriaceae; Mycobacterium; Mycobacterium tuberculosis complex; Mycobacterium tuberculosis;	NCBI: 1773	4365322	99.58	0.66	Yes
NCBI Assembly	GCA_002089515.1	NCBI: cellular organisms; Bacteria; Terrabacteria group; Actinobacteria; Actinomycetia; Corynebacteriales; Mycobacteriaceae; Mycobacterium; Mycobacterium avium complex (MAC); Mycobacterium avium; Mycobacterium avium subsp. hominissuis;	NCBI: 439334	5397364	99.91	2.62	Yes
NCBI Assembly	GCA_002705865.1	NCBI: cellular organisms; Bacteria; Terrabacteria group; Actinobacteria; Actinomycetia; Corynebacteriales; Mycobacteriaceae; Mycobacterium; Mycobacterium kansasii;	NCBI: 1768	6667663	99.90	0.71	Yes
NCBI Assembly	GCA_001947965.1	NCBI: cellular organisms; Bacteria; Terrabacteria group; Actinobacteria; Actinomycetia; Corynebacteriales; Mycobacteriaceae; Mycobacterium; Mycobacterium tuberculosis complex; Mycobacterium tuberculosis;	NCBI: 1773	4380260	99.94	0.00	Yes
NCBI Assembly	GCA_001946785.1	NCBI: cellular organisms; Bacteria; Terrabacteria group; Actinobacteria; Actinomycetia; Corynebacteriales; Mycobacteriaceae; Mycobacterium; Mycobacterium tuberculosis complex; Mycobacterium tuberculosis;	NCBI: 1773	4342367	99.94	0.00	Yes
NCBI Assembly	GCA_002130345.1	NCBI: cellular organisms; Bacteria; Terrabacteria group; Actinobacteria; Actinomycetia; Corynebacteriales; Mycobacteriaceae; Mycobacterium; Mycobacterium tuberculosis complex; Mycobacterium tuberculosis;	NCBI: 1773	4391895	99.94	0.00	Yes
NCBI Assembly	GCA_001945225.1	NCBI: cellular organisms; Bacteria; Terrabacteria group; Actinobacteria; Actinomycetia; Corynebacteriales; Mycobacteriaceae; Mycobacterium; Mycobacterium tuberculosis complex; Mycobacterium tuberculosis;	NCBI: 1773	4382655	99.94	0.00	Yes
NCBI Assembly	GCA_001944895.1	NCBI: cellular organisms; Bacteria; Terrabacteria group; Actinobacteria; Actinomycetia; Corynebacteriales; Mycobacteriaceae; Mycobacterium; Mycobacterium tuberculosis complex; Mycobacterium tuberculosis;	NCBI: 1773	4374284	99.94	0.00	Yes
NCBI Assembly	GCA_001948315.1	NCBI: cellular organisms; Bacteria; Terrabacteria group; Actinobacteria; Actinomycetia; Corynebacteriales; Mycobacteriaceae; Mycobacterium; Mycobacterium tuberculosis complex; Mycobacterium tuberculosis;	NCBI: 1773	4392204	99.61	0.00	Yes
NCBI Assembly	GCA_002101965.1	NCBI: cellular organisms; Bacteria; Terrabacteria group; Actinobacteria; Actinomycetia; Corynebacteriales; Mycobacteriaceae; Mycolicibacterium; Mycolicibacterium wolinskyi;	NCBI: 59750	7516859	99.94	1.59	Yes
NCBI Assembly	GCA_002129745.1	NCBI: cellular organisms; Bacteria; Terrabacteria group; Actinobacteria; Actinomycetia; Corynebacteriales; Mycobacteriaceae; Mycobacterium; Mycobacterium tuberculosis complex; Mycobacterium tuberculosis;	NCBI: 1773	4400441	99.94	0.00	Yes
NCBI Assembly	GCA_002406295.1	NCBI: cellular organisms; Bacteria; Terrabacteria group; Actinobacteria; Actinomycetia; Corynebacteriales; Mycobacteriaceae; Mycobacterium; Mycobacterium tuberculosis complex; Mycobacterium tuberculosis;	NCBI: 1773	4362922	99.94	0.00	Yes
NCBI Assembly	GCA_002089535.1	NCBI: cellular organisms; Bacteria; Terrabacteria group; Actinobacteria; Actinomycetia; Corynebacteriales; Mycobacteriaceae; Mycobacterium; Mycobacterium avium complex (MAC); Mycobacterium avium; Mycobacterium avium subsp. hominissuis;	NCBI: 439334	5106249	99.13	1.25	Yes
NCBI Assembly	GCA_002553575.2	NCBI: cellular organisms; Bacteria; Terrabacteria group; Actinobacteria; Actinomycetia; Corynebacteriales; Mycobacteriaceae; Mycolicibacterium; Mycolicibacterium brumae;	NCBI: 85968	3878367	98.99	0.05	Yes

NCBI Assembly	GCA_002705885.1	NCBI: cellular organisms; Bacteria; Terrabacteria group; Actinobacteria; Actinomycetia; Corynebacteriales; Mycobacteriaceae; Mycobacterium; Mycobacterium kansasii;	NCBI: 1768	6371938	99.61	0.38	Yes
NCBI Assembly	GCA_001947975.1	NCBI: cellular organisms; Bacteria; Terrabacteria group; Actinobacteria; Actinomycetia; Corynebacteriales; Mycobacteriaceae; Mycobacterium; Mycobacterium tuberculosis complex; Mycobacterium tuberculosis;	NCBI: 1773	4366559	99.94	0.00	Yes
NCBI Assembly	GCA_001946795.1	NCBI: cellular organisms; Bacteria; Terrabacteria group; Actinobacteria; Actinomycetia; Corynebacteriales; Mycobacteriaceae; Mycobacterium; Mycobacterium tuberculosis complex; Mycobacterium tuberculosis;	NCBI: 1773	4386289	99.94	0.00	Yes
NCBI Assembly	GCA_002130355.1	NCBI: cellular organisms; Bacteria; Terrabacteria group; Actinobacteria; Actinomycetia; Corynebacteriales; Mycobacteriaceae; Mycobacterium; Mycobacterium tuberculosis complex; Mycobacterium tuberculosis;	NCBI: 1773	4370262	99.94	0.00	Yes
NCBI Assembly	GCA_001944925.1	NCBI: cellular organisms; Bacteria; Terrabacteria group; Actinobacteria; Actinomycetia; Corynebacteriales; Mycobacteriaceae; Mycobacterium; Mycobacterium tuberculosis complex; Mycobacterium tuberculosis;	NCBI: 1773	4376335	99.94	0.00	Yes
NCBI Assembly	GCA_001945235.1	NCBI: cellular organisms; Bacteria; Terrabacteria group; Actinobacteria; Actinomycetia; Corynebacteriales; Mycobacteriaceae; Mycobacterium; Mycobacterium tuberculosis complex; Mycobacterium tuberculosis;	NCBI: 1773	4372380	99.94	0.00	Yes
NCBI Assembly	GCA_001948365.1	NCBI: cellular organisms; Bacteria; Terrabacteria group; Actinobacteria; Actinomycetia; Corynebacteriales; Mycobacteriaceae; Mycobacterium; Mycobacterium tuberculosis complex; Mycobacterium tuberculosis;	NCBI: 1773	4392639	99.94	0.00	Yes
NCBI Assembly	GCA_002089555.1	NCBI: cellular organisms; Bacteria; Terrabacteria group; Actinobacteria; Actinomycetia; Corynebacteriales; Mycobacteriaceae; Mycobacterium; Mycobacterium avium complex (MAC); Mycobacterium avium; Mycobacterium avium subsp. hominissuis;	NCBI: 439334	5335072	99.46	1.00	Yes
NCBI Assembly	GCA_002101995.1	NCBI: cellular organisms; Bacteria; Terrabacteria group; Actinobacteria; Actinomycetia; Corynebacteriales; Mycobacteriaceae; Mycolicibacterium; Mycolicibacterium fallax;	NCBI: 1793	4232450	98.71	0.05	Yes
NCBI Assembly	GCA_002129755.1	NCBI: cellular organisms; Bacteria; Terrabacteria group; Actinobacteria; Actinomycetia; Corynebacteriales; Mycobacteriaceae; Mycobacterium; Mycobacterium tuberculosis complex; Mycobacterium tuberculosis;	NCBI: 1773	4406734	99.94	0.00	Yes
NCBI Assembly	GCA_002406455.1	NCBI: cellular organisms; Bacteria; Terrabacteria group; Actinobacteria; Actinomycetia; Corynebacteriales; Mycobacteriaceae; Mycobacterium; Mycobacterium tuberculosis complex; Mycobacterium tuberculosis;	NCBI: 1773	4235087	98.94	0.94	Yes
NCBI Assembly	GCA_002705895.1	NCBI: cellular organisms; Bacteria; Terrabacteria group; Actinobacteria; Actinomycetia; Corynebacteriales; Mycobacteriaceae; Mycobacterium; Mycobacterium persicum;	NCBI: 1487726	6238172	99.94	0.67	Yes
NCBI Assembly	GCA_001947985.1	NCBI: cellular organisms; Bacteria; Terrabacteria group; Actinobacteria; Actinomycetia; Corynebacteriales; Mycobacteriaceae; Mycobacterium; Mycobacterium tuberculosis complex; Mycobacterium tuberculosis;	NCBI: 1773	4390584	99.94	0.00	Yes
NCBI Assembly	GCA_001946815.1	NCBI: cellular organisms; Bacteria; Terrabacteria group; Actinobacteria; Actinomycetia; Corynebacteriales; Mycobacteriaceae; Mycobacterium; Mycobacterium tuberculosis complex; Mycobacterium tuberculosis;	NCBI: 1773	4378837	99.94	0.00	Yes
NCBI Assembly	GCA_002130375.1	NCBI: cellular organisms; Bacteria; Terrabacteria group; Actinobacteria; Actinomycetia; Corynebacteriales; Mycobacteriaceae; Mycobacterium; Mycobacterium tuberculosis complex; Mycobacterium tuberculosis;	NCBI: 1773	4395662	99.94	0.00	Yes
NCBI Assembly	GCA_001945245.1	NCBI: cellular organisms; Bacteria; Terrabacteria group; Actinobacteria; Actinomycetia; Corynebacteriales; Mycobacteriaceae; Mycobacterium; Mycobacterium tuberculosis complex; Mycobacterium tuberculosis;	NCBI: 1773	4375485	99.61	0.00	Yes
NCBI Assembly	GCA_001944935.1	NCBI: cellular organisms; Bacteria; Terrabacteria group; Actinobacteria; Actinomycetia; Corynebacteriales; Mycobacteriaceae; Mycobacterium; Mycobacterium tuberculosis complex; Mycobacterium tuberculosis;	NCBI: 1773	4376248	99.94	0.00	Yes
NCBI Assembly	GCA_002102015.1	NCBI: cellular organisms; Bacteria; Terrabacteria group; Actinobacteria; Actinomycetia; Corynebacteriales; Mycobacteriaceae; Mycobacterium; Mycobacterium xenopi;	NCBI: 1789	4933400	99.09	2.65	Yes

NCBI Assembly	GCA_001948375.1	NCBI: cellular organisms; Bacteria; Terrabacteria group; Actinobacteria; Actinomycetia; Corynebacteriales; Mycobacteriaceae; Mycobacterium; Mycobacterium tuberculosis complex; Mycobacterium tuberculosis;	NCBI: 1773	4406102	99.94	0.00	Yes
NCBI Assembly	GCA_002089575.1	NCBI: cellular organisms; Bacteria; Terrabacteria group; Actinobacteria; Actinomycetia; Corynebacteriales; Mycobacteriaceae; Mycobacterium; Mycobacterium avium complex (MAC); Mycobacterium avium; Mycobacterium avium subsp. hominissuis;	NCBI: 439334	5089718	99.09	1.04	Yes
NCBI Assembly	GCA_002129785.1	NCBI: cellular organisms; Bacteria; Terrabacteria group; Actinobacteria; Actinomycetia; Corynebacteriales; Mycobacteriaceae; Mycobacterium; Mycobacterium tuberculosis complex; Mycobacterium tuberculosis;	NCBI: 1773	4405750	99.94	0.00	Yes
NCBI Assembly	GCA_002446875.1	NCBI: cellular organisms; Bacteria; Terrabacteria group; Actinobacteria; Actinomycetia; Corynebacteriales; Mycobacteriaceae; Mycobacterium; Mycobacterium tuberculosis complex; Mycobacterium tuberculosis;	NCBI: 1773	4411288	99.94	0.00	Yes
NCBI Assembly	GCA_002705925.1	NCBI: cellular organisms; Bacteria; Terrabacteria group; Actinobacteria; Actinomycetia; Corynebacteriales; Mycobacteriaceae; Mycobacterium; Mycobacterium ostraviense;	NCBI: 2738409	6101088	99.61	0.99	Yes
NCBI Assembly	GCA_001947995.1	NCBI: cellular organisms; Bacteria; Terrabacteria group; Actinobacteria; Actinomycetia; Corynebacteriales; Mycobacteriaceae; Mycobacterium; Mycobacterium tuberculosis complex; Mycobacterium tuberculosis;	NCBI: 1773	4385254	99.94	0.00	Yes
NCBI Assembly	GCA_001946835.1	NCBI: cellular organisms; Bacteria; Terrabacteria group; Actinobacteria; Actinomycetia; Corynebacteriales; Mycobacteriaceae; Mycobacterium; Mycobacterium tuberculosis complex; Mycobacterium tuberculosis;	NCBI: 1773	4392995	99.94	0.00	Yes
NCBI Assembly	GCA_002588215.1	NCBI: cellular organisms; Bacteria; Terrabacteria group; Actinobacteria; Actinomycetia; Corynebacteriales; Mycobacteriaceae; Mycobacterium; Mycobacterium tuberculosis complex; Mycobacterium tuberculosis;	NCBI: 1773	4466604	99.94	0.00	Yes
NCBI Assembly	GCA_001945285.1	NCBI: cellular organisms; Bacteria; Terrabacteria group; Actinobacteria; Actinomycetia; Corynebacteriales; Mycobacteriaceae; Mycobacterium; Mycobacterium tuberculosis complex; Mycobacterium tuberculosis;	NCBI: 1773	4389808	99.94	0.00	Yes
NCBI Assembly	GCA_001944945.1	NCBI: cellular organisms; Bacteria; Terrabacteria group; Actinobacteria; Actinomycetia; Corynebacteriales; Mycobacteriaceae; Mycobacterium; Mycobacterium tuberculosis complex; Mycobacterium tuberculosis;	NCBI: 1773	4383228	99.94	0.00	Yes
NCBI Assembly	GCA_002102065.1	NCBI: cellular organisms; Bacteria; Terrabacteria group; Actinobacteria; Actinomycetia; Corynebacteriales; Mycobacteriaceae; Mycolicibacterium; Mycolicibacterium conceptionense;	NCBI: 451644	6468817	100.00	0.00	Yes
NCBI Assembly	GCA_002130395.1	NCBI: cellular organisms; Bacteria; Terrabacteria group; Actinobacteria; Actinomycetia; Corynebacteriales; Mycobacteriaceae; Mycobacterium; Mycobacterium tuberculosis complex; Mycobacterium tuberculosis;	NCBI: 1773	4400029	99.94	0.00	Yes
NCBI Assembly	GCA_001948385.1	NCBI: cellular organisms; Bacteria; Terrabacteria group; Actinobacteria; Actinomycetia; Corynebacteriales; Mycobacteriaceae; Mycobacterium; Mycobacterium tuberculosis complex; Mycobacterium tuberculosis;	NCBI: 1773	4374509	99.94	0.00	Yes
NCBI Assembly	GCA_002129805.1	NCBI: cellular organisms; Bacteria; Terrabacteria group; Actinobacteria; Actinomycetia; Corynebacteriales; Mycobacteriaceae; Mycobacterium; Mycobacterium tuberculosis complex; Mycobacterium tuberculosis;	NCBI: 1773	4405738	99.94	0.00	Yes
NCBI Assembly	GCA_002089595.1	NCBI: cellular organisms; Bacteria; Terrabacteria group; Actinobacteria; Actinomycetia; Corynebacteriales; Mycobacteriaceae; Mycobacterium; Mycobacterium avium complex (MAC); Mycobacterium avium; Mycobacterium avium subsp. hominissuis;	NCBI: 439334	5216878	99.78	0.05	Yes
NCBI Assembly	GCA_001948045.1	NCBI: cellular organisms; Bacteria; Terrabacteria group; Actinobacteria; Actinomycetia; Corynebacteriales; Mycobacteriaceae; Mycobacterium; Mycobacterium tuberculosis complex; Mycobacterium tuberculosis;	NCBI: 1773	4391292	99.94	0.00	Yes
NCBI Assembly	GCA_002446895.1	NCBI: cellular organisms; Bacteria; Terrabacteria group; Actinobacteria; Actinomycetia; Corynebacteriales; Mycobacteriaceae; Mycobacterium; Mycobacterium tuberculosis complex; Mycobacterium tuberculosis;	NCBI: 1773	4411271	99.94	0.00	Yes
NCBI Assembly	GCA_002705935.1	NCBI: cellular organisms; Bacteria; Terrabacteria group; Actinobacteria; Actinomycetia; Corynebacteriales; Mycobacteriaceae; Mycobacterium; Mycobacterium kansasii;	NCBI: 1768	6351519	99.61	0.23	Yes

NCBI Assembly	GCA_001946865.1	NCBI: cellular organisms; Bacteria; Terrabacteria group; Actinobacteria; Actinomycetia; Corynebacteriales; Mycobacteriaceae; Mycobacterium; Mycobacterium tuberculosis complex; Mycobacterium tuberculosis;	NCBI: 1773	4408243	99.94	0.00	Yes
NCBI Assembly	GCA_001945305.1	NCBI: cellular organisms; Bacteria; Terrabacteria group; Actinobacteria; Actinomycetia; Corynebacteriales; Mycobacteriaceae; Mycobacterium; Mycobacterium tuberculosis complex; Mycobacterium tuberculosis;	NCBI: 1773	4388470	99.94	0.00	Yes
NCBI Assembly	GCA_002591875.1	NCBI: cellular organisms; Bacteria; Terrabacteria group; Actinobacteria; Actinomycetia; Corynebacteriales; Mycobacteriaceae; Mycobacterium; Mycobacterium tuberculosis complex; Mycobacterium tuberculosis;	NCBI: 1773	4391906	99.94	0.00	Yes
NCBI Assembly	GCA_002102095.1	NCBI: cellular organisms; Bacteria; Terrabacteria group; Actinobacteria; Actinomycetia; Corynebacteriales; Mycobacteriaceae; Mycobacterium; Mycobacterium conspicuum;	NCBI: 44010	6200614	99.11	0.49	Yes
NCBI Assembly	GCA_001944955.1	NCBI: cellular organisms; Bacteria; Terrabacteria group; Actinobacteria; Actinomycetia; Corynebacteriales; Mycobacteriaceae; Mycobacterium; Mycobacterium tuberculosis complex; Mycobacterium tuberculosis;	NCBI: 1773	4389994	99.94	0.00	Yes
NCBI Assembly	GCA_002130425.1	NCBI: cellular organisms; Bacteria; Terrabacteria group; Actinobacteria; Actinomycetia; Corynebacteriales; Mycobacteriaceae; Mycobacterium; Mycobacterium tuberculosis complex; Mycobacterium tuberculosis;	NCBI: 1773	4390088	99.94	0.00	Yes
NCBI Assembly	GCA_001948405.1	NCBI: cellular organisms; Bacteria; Terrabacteria group; Actinobacteria; Actinomycetia; Corynebacteriales; Mycobacteriaceae; Mycobacterium; Mycobacterium tuberculosis complex; Mycobacterium tuberculosis;	NCBI: 1773	4378876	99.94	0.00	Yes
NCBI Assembly	GCA_002089615.1	NCBI: cellular organisms; Bacteria; Terrabacteria group; Actinobacteria; Actinomycetia; Corynebacteriales; Mycobacteriaceae; Mycobacterium; Mycobacterium avium complex (MAC); Mycobacterium avium; Mycobacterium avium subsp. hominissuis;	NCBI: 439334	5126590	99.83	0.51	Yes
NCBI Assembly	GCA_002129815.1	NCBI: cellular organisms; Bacteria; Terrabacteria group; Actinobacteria; Actinomycetia; Corynebacteriales; Mycobacteriaceae; Mycobacterium; Mycobacterium tuberculosis complex; Mycobacterium tuberculosis;	NCBI: 1773	4397866	99.94	0.00	Yes
NCBI Assembly	GCA_001948055.1	NCBI: cellular organisms; Bacteria; Terrabacteria group; Actinobacteria; Actinomycetia; Corynebacteriales; Mycobacteriaceae; Mycobacterium; Mycobacterium tuberculosis complex; Mycobacterium tuberculosis;	NCBI: 1773	4401323	99.94	0.00	Yes
NCBI Assembly	GCA_002446915.1	NCBI: cellular organisms; Bacteria; Terrabacteria group; Actinobacteria; Actinomycetia; Corynebacteriales; Mycobacteriaceae; Mycobacterium; Mycobacterium tuberculosis complex; Mycobacterium tuberculosis;	NCBI: 1773	4411312	99.94	0.00	Yes
NCBI Assembly	GCA_001946875.1	NCBI: cellular organisms; Bacteria; Terrabacteria group; Actinobacteria; Actinomycetia; Corynebacteriales; Mycobacteriaceae; Mycobacterium; Mycobacterium tuberculosis complex; Mycobacterium tuberculosis;	NCBI: 1773	4388059	99.94	0.00	Yes
NCBI Assembly	GCA_002705965.1	NCBI: cellular organisms; Bacteria; Terrabacteria group; Actinobacteria; Actinomycetia; Corynebacteriales; Mycobacteriaceae; Mycobacterium; Mycobacterium kansasii;	NCBI: 1768	6647820	99.94	4.61	Yes
NCBI Assembly	GCA_001945315.1	NCBI: cellular organisms; Bacteria; Terrabacteria group; Actinobacteria; Actinomycetia; Corynebacteriales; Mycobacteriaceae; Mycobacterium; Mycobacterium tuberculosis complex; Mycobacterium tuberculosis;	NCBI: 1773	4375417	99.94	0.00	Yes
NCBI Assembly	GCA_002102105.1	NCBI: cellular organisms; Bacteria; Terrabacteria group; Actinobacteria; Actinomycetia; Corynebacteriales; Mycobacteriaceae; Mycolicibacterium; Mycolicibacterium confluentis;	NCBI: 28047	5841691	100.00	0.06	Yes
NCBI Assembly	GCA_002591975.1	NCBI: cellular organisms; Bacteria; Terrabacteria group; Actinobacteria; Actinomycetia; Corynebacteriales; Mycobacteriaceae; Mycobacterium; Mycobacterium neglectum;	NCBI: 242737	6209311	100.00	0.15	Yes
NCBI Assembly	GCA_001945005.1	NCBI: cellular organisms; Bacteria; Terrabacteria group; Actinobacteria; Actinomycetia; Corynebacteriales; Mycobacteriaceae; Mycobacterium; Mycobacterium tuberculosis complex; Mycobacterium tuberculosis;	NCBI: 1773	4360235	99.94	0.00	Yes
NCBI Assembly	GCA_002130445.1	NCBI: cellular organisms; Bacteria; Terrabacteria group; Actinobacteria; Actinomycetia; Corynebacteriales; Mycobacteriaceae; Mycobacterium; Mycobacterium tuberculosis complex; Mycobacterium tuberculosis;	NCBI: 1773	4381652	99.94	0.00	Yes

NCBI Assembly	GCA_001948445.1	NCBI: cellular organisms; Bacteria; Terrabacteria group; Actinobacteria; Actinomycetia; Corynebacteriales; Mycobacteriaceae; Mycobacterium; Mycobacterium tuberculosis complex; Mycobacterium tuberculosis;	NCBI: 1773	4392713	99.94	0.00	Yes
NCBI Assembly	GCA_002129825.1	NCBI: cellular organisms; Bacteria; Terrabacteria group; Actinobacteria; Actinomycetia; Corynebacteriales; Mycobacteriaceae; Mycobacterium; Mycobacterium tuberculosis complex; Mycobacterium tuberculosis;	NCBI: 1773	4403523	99.94	0.00	Yes
NCBI Assembly	GCA_002089635.1	NCBI: cellular organisms; Bacteria; Terrabacteria group; Actinobacteria; Actinomycetia; Corynebacteriales; Mycobacteriaceae; Mycobacterium; Mycobacterium avium complex (MAC); Mycobacterium avium; Mycobacterium avium subsp. hominissuis;	NCBI: 439334	5389591	98.83	0.49	Yes
NCBI Assembly	GCA_001948065.1	NCBI: cellular organisms; Bacteria; Terrabacteria group; Actinobacteria; Actinomycetia; Corynebacteriales; Mycobacteriaceae; Mycobacterium; Mycobacterium tuberculosis complex; Mycobacterium tuberculosis;	NCBI: 1773	4418351	99.94	0.00	Yes
NCBI Assembly	GCA_002090015.1	NCBI: cellular organisms; Bacteria; Terrabacteria group; Actinobacteria; Actinomycetia; Corynebacteriales; Mycobacteriaceae; Mycobacterium; Mycobacterium avium complex (MAC); Mycobacterium avium; Mycobacterium avium subsp. hominissuis;	NCBI: 439334	5811642	99.22	0.89	Yes
NCBI Assembly	GCA_002446935.1	NCBI: cellular organisms; Bacteria; Terrabacteria group; Actinobacteria; Actinomycetia; Corynebacteriales; Mycobacteriaceae; Mycobacterium; Mycobacterium tuberculosis complex; Mycobacterium tuberculosis;	NCBI: 1773	4411180	99.94	0.00	Yes
NCBI Assembly	GCA_001945025.1	NCBI: cellular organisms; Bacteria; Terrabacteria group; Actinobacteria; Actinomycetia; Corynebacteriales; Mycobacteriaceae; Mycobacterium; Mycobacterium tuberculosis complex; Mycobacterium tuberculosis;	NCBI: 1773	4379979	99.94	0.00	Yes
NCBI Assembly	GCA_002447895.1	NCBI: cellular organisms; Bacteria; Terrabacteria group; Actinobacteria; Actinomycetia; Corynebacteriales; Mycobacteriaceae; Mycobacterium; Mycobacterium tuberculosis complex; Mycobacterium tuberculosis;	NCBI: 1773	4411338	99.94	0.00	Yes
NCBI Assembly	GCA_002102115.1	NCBI: cellular organisms; Bacteria; Terrabacteria group; Actinobacteria; Actinomycetia; Corynebacteriales; Mycobacteriaceae; Mycolicibacterium; Mycolicibacterium doricum;	NCBI: 126673	3952103	98.68	0.39	Yes
NCBI Assembly	GCA_002708555.1	NCBI: cellular organisms; Bacteria; Terrabacteria group; Actinobacteria; Actinomycetia; Corynebacteriales; Mycobacteriaceae; Mycobacterium; Mycobacterium tuberculosis complex; Mycobacterium tuberculosis;	NCBI: 1773	4280826	98.78	0.00	Yes
NCBI Assembly	GCA_002592005.1	NCBI: cellular organisms; Bacteria; Terrabacteria group; Actinobacteria; Actinomycetia; Corynebacteriales; Mycobacteriaceae; Mycobacterium; Mycobacterium palauense;	NCBI: 2034511	6230283	99.64	1.71	Yes
NCBI Assembly	GCA_002130455.1	NCBI: cellular organisms; Bacteria; Terrabacteria group; Actinobacteria; Actinomycetia; Corynebacteriales; Mycobacteriaceae; Mycobacterium; Mycobacterium tuberculosis complex; Mycobacterium tuberculosis;	NCBI: 1773	4391350	99.61	0.00	Yes
NCBI Assembly	GCA_001948455.1	NCBI: cellular organisms; Bacteria; Terrabacteria group; Actinobacteria; Actinomycetia; Corynebacteriales; Mycobacteriaceae; Mycobacterium; Mycobacterium tuberculosis complex; Mycobacterium tuberculosis;	NCBI: 1773	4388343	99.94	0.00	Yes
NCBI Assembly	GCA_002089655.1	NCBI: cellular organisms; Bacteria; Terrabacteria group; Actinobacteria; Actinomycetia; Corynebacteriales; Mycobacteriaceae; Mycobacterium; Mycobacterium avium complex (MAC); Mycobacterium avium; Mycobacterium avium subsp. hominissuis;	NCBI: 439334	5452988	99.44	0.81	Yes
NCBI Assembly	GCA_002129865.1	NCBI: cellular organisms; Bacteria; Terrabacteria group; Actinobacteria; Actinomycetia; Corynebacteriales; Mycobacteriaceae; Mycobacterium; Mycobacterium tuberculosis complex; Mycobacterium tuberculosis;	NCBI: 1773	4395550	99.94	0.00	Yes
NCBI Assembly	GCA_001948075.1	NCBI: cellular organisms; Bacteria; Terrabacteria group; Actinobacteria; Actinomycetia; Corynebacteriales; Mycobacteriaceae; Mycobacterium; Mycobacterium tuberculosis complex; Mycobacterium tuberculosis;	NCBI: 1773	4388293	99.94	0.00	Yes
NCBI Assembly	GCA_002090035.1	NCBI: cellular organisms; Bacteria; Terrabacteria group; Actinobacteria; Actinomycetia; Corynebacteriales; Mycobacteriaceae; Mycobacterium; Mycobacterium avium complex (MAC); Mycobacterium avium; Mycobacterium avium subsp. hominissuis;	NCBI: 439334	5463470	99.83	1.68	Yes
NCBI Assembly	GCA_002446955.1	NCBI: cellular organisms; Bacteria; Terrabacteria group; Actinobacteria; Actinomycetia; Corynebacteriales; Mycobacteriaceae; Mycobacterium; Mycobacterium tuberculosis complex; Mycobacterium tuberculosis;	NCBI: 1773	4411382	99.94	0.00	Yes

NCBI Assembly	GCA_001944405.1	NCBI: cellular organisms; Bacteria; Terrabacteria group; Actinobacteria; Actinomycetia; Corynebacteriales; Mycobacteriaceae; Mycobacterium; Mycobacterium tuberculosis complex; Mycobacterium tuberculosis;	NCBI: 1773	4391967	99.94	0.00	Yes
NCBI Assembly	GCA_002102155.1	NCBI: cellular organisms; Bacteria; Terrabacteria group; Actinobacteria; Actinomycetia; Corynebacteriales; Mycobacteriaceae; Mycobacterium; Mycobacterium simiae complex; Mycobacterium europaeum;	NCBI: 761804	5630674	99.94	0.50	Yes
NCBI Assembly	GCA_002447915.1	NCBI: cellular organisms; Bacteria; Terrabacteria group; Actinobacteria; Actinomycetia; Corynebacteriales; Mycobacteriaceae; Mycobacterium; Mycobacterium tuberculosis complex; Mycobacterium tuberculosis;	NCBI: 1773	4411432	99.94	0.00	Yes
NCBI Assembly	GCA_002708565.1	NCBI: cellular organisms; Bacteria; Terrabacteria group; Actinobacteria; Actinomycetia; Corynebacteriales; Mycobacteriaceae; Mycobacterium; Mycobacterium tuberculosis complex; Mycobacterium tuberculosis;	NCBI: 1773	4304863	99.08	0.00	Yes
NCBI Assembly	GCA_002130485.1	NCBI: cellular organisms; Bacteria; Terrabacteria group; Actinobacteria; Actinomycetia; Corynebacteriales; Mycobacteriaceae; Mycobacterium; Mycobacterium tuberculosis complex; Mycobacterium tuberculosis;	NCBI: 1773	4382457	99.94	0.00	Yes
NCBI Assembly	GCA_002131765.1	NCBI: cellular organisms; Bacteria; Terrabacteria group; Actinobacteria; Actinomycetia; Corynebacteriales; Mycobacteriaceae; Mycobacterium; Mycobacterium tuberculosis complex; Mycobacterium tuberculosis;	NCBI: 1773	4393572	99.94	0.00	Yes
NCBI Assembly	GCA_002703685.1	NCBI: cellular organisms; Bacteria; Terrabacteria group; Actinobacteria; Actinomycetia; Corynebacteriales; Mycobacteriaceae; Mycobacterium; Mycobacterium tuberculosis complex; Mycobacterium tuberculosis; Mycobacterium tuberculosis variant bovis;	NCBI: 1765	4373214	99.78	0.67	Yes
NCBI Assembly	GCA_002089675.1	NCBI: cellular organisms; Bacteria; Terrabacteria group; Actinobacteria; Actinomycetia; Corynebacteriales; Mycobacteriaceae; Mycobacterium; Mycobacterium avium complex (MAC); Mycobacterium avium; Mycobacterium avium subsp. hominissuis;	NCBI: 439334	5340288	99.32	0.48	Yes
NCBI Assembly	GCA_002129885.1	NCBI: cellular organisms; Bacteria; Terrabacteria group; Actinobacteria; Actinomycetia; Corynebacteriales; Mycobacteriaceae; Mycobacterium; Mycobacterium tuberculosis complex; Mycobacterium tuberculosis;	NCBI: 1773	4407321	99.94	0.00	Yes
NCBI Assembly	GCA_001948125.1	NCBI: cellular organisms; Bacteria; Terrabacteria group; Actinobacteria; Actinomycetia; Corynebacteriales; Mycobacteriaceae; Mycobacterium; Mycobacterium tuberculosis complex; Mycobacterium tuberculosis;	NCBI: 1773	4399884	99.94	0.00	Yes
NCBI Assembly	GCA_002093075.1	NCBI: cellular organisms; Bacteria; Terrabacteria group; Actinobacteria; Actinomycetia; Corynebacteriales; Mycobacteriaceae; Mycobacterium; Mycobacterium simiae complex; Mycobacterium simiae;	NCBI: 1784	6686819	99.53	2.78	Yes
NCBI Assembly	GCA_001944415.1	NCBI: cellular organisms; Bacteria; Terrabacteria group; Actinobacteria; Actinomycetia; Corynebacteriales; Mycobacteriaceae; Mycobacterium; Mycobacterium tuberculosis complex; Mycobacterium tuberculosis;	NCBI: 1773	4383305	99.94	0.00	Yes
NCBI Assembly	GCA_002102175.1	NCBI: cellular organisms; Bacteria; Terrabacteria group; Actinobacteria; Actinomycetia; Corynebacteriales; Mycobacteriaceae; Mycobacterium; Mycobacterium gastri;	NCBI: 1777	5816659	99.28	0.39	Yes
NCBI Assembly	GCA_002446975.1	NCBI: cellular organisms; Bacteria; Terrabacteria group; Actinobacteria; Actinomycetia; Corynebacteriales; Mycobacteriaceae; Mycobacterium; Mycobacterium tuberculosis complex; Mycobacterium tuberculosis;	NCBI: 1773	4411180	99.94	0.00	Yes
NCBI Assembly	GCA_002447935.1	NCBI: cellular organisms; Bacteria; Terrabacteria group; Actinobacteria; Actinomycetia; Corynebacteriales; Mycobacteriaceae; Mycobacterium; Mycobacterium tuberculosis complex; Mycobacterium tuberculosis;	NCBI: 1773	4411159	99.94	0.00	Yes
NCBI Assembly	GCA_002130505.1	NCBI: cellular organisms; Bacteria; Terrabacteria group; Actinobacteria; Actinomycetia; Corynebacteriales; Mycobacteriaceae; Mycobacterium; Mycobacterium tuberculosis complex; Mycobacterium tuberculosis;	NCBI: 1773	4388025	99.94	0.00	Yes
NCBI Assembly	GCA_002131785.1	NCBI: cellular organisms; Bacteria; Terrabacteria group; Actinobacteria; Actinomycetia; Corynebacteriales; Mycobacteriaceae; Mycobacterium; Mycobacterium tuberculosis complex; Mycobacterium tuberculosis;	NCBI: 1773	4395052	99.94	0.00	Yes
NCBI Assembly	GCA_002703695.1	NCBI: cellular organisms; Bacteria; Terrabacteria group; Actinobacteria; Actinomycetia; Corynebacteriales; Mycobacteriaceae; Mycobacterium; Mycobacterium tuberculosis complex; Mycobacterium tuberculosis; Mycobacterium tuberculosis variant bovis;	NCBI: 1765	4460806	99.78	2.11	Yes

NCBI Assembly	GCA_001948135.1	NCBI: cellular organisms; Bacteria; Terrabacteria group; Actinobacteria; Actinomycetia; Corynebacteriales; Mycobacteriaceae; Mycobacterium; Mycobacterium tuberculosis complex; Mycobacterium tuberculosis;	NCBI: 1773	4386009	99.94	0.00	Yes
NCBI Assembly	GCA_002129265.1	NCBI: cellular organisms; Bacteria; Terrabacteria group; Actinobacteria; Actinomycetia; Corynebacteriales; Mycobacteriaceae; Mycobacterium; Mycobacterium tuberculosis complex; Mycobacterium tuberculosis;	NCBI: 1773	4393542	99.94	0.00	Yes
NCBI Assembly	GCA_002089695.1	NCBI: cellular organisms; Bacteria; Terrabacteria group; Actinobacteria; Actinomycetia; Corynebacteriales; Mycobacteriaceae; Mycobacterium; Mycobacterium avium complex (MAC); Mycobacterium avium; Mycobacterium avium subsp. hominissuis;	NCBI: 439334	5482429	99.06	0.44	Yes
NCBI Assembly	GCA_002101555.1	NCBI: cellular organisms; Bacteria; Terrabacteria group; Actinobacteria; Actinomycetia; Corynebacteriales; Mycobacteriaceae; Mycolicibacterium; Mycolicibacterium canariasense;	NCBI: 228230	6755573	99.89	2.14	Yes
NCBI Assembly	GCA_002102185.1	NCBI: cellular organisms; Bacteria; Terrabacteria group; Actinobacteria; Actinomycetia; Corynebacteriales; Mycobacteriaceae; Mycobacterium; Mycobacterium fragae；	NCBI: 1260918	4731047	99.55	0.45	Yes
NCBI Assembly	GCA_001944445.1	NCBI: cellular organisms; Bacteria; Terrabacteria group; Actinobacteria; Actinomycetia; Corynebacteriales; Mycobacteriaceae; Mycobacterium; Mycobacterium tuberculosis complex; Mycobacterium tuberculosis;	NCBI: 1773	4384051	99.94	0.00	Yes
NCBI Assembly	GCA_002446995.1	NCBI: cellular organisms; Bacteria; Terrabacteria group; Actinobacteria; Actinomycetia; Corynebacteriales; Mycobacteriaceae; Mycobacterium; Mycobacterium tuberculosis complex; Mycobacterium tuberculosis;	NCBI: 1773	4411208	99.94	0.00	Yes
NCBI Assembly	GCA_002447955.1	NCBI: cellular organisms; Bacteria; Terrabacteria group; Actinobacteria; Actinomycetia; Corynebacteriales; Mycobacteriaceae; Mycobacterium; Mycobacterium tuberculosis complex; Mycobacterium tuberculosis;	NCBI: 1773	4411229	99.94	0.00	Yes
NCBI Assembly	GCA_002129905.1	NCBI: cellular organisms; Bacteria; Terrabacteria group; Actinobacteria; Actinomycetia; Corynebacteriales; Mycobacteriaceae; Mycobacterium; Mycobacterium tuberculosis complex; Mycobacterium tuberculosis;	NCBI: 1773	4399655	99.94	0.00	Yes
NCBI Assembly	GCA_002131805.1	NCBI: cellular organisms; Bacteria; Terrabacteria group; Actinobacteria; Actinomycetia; Corynebacteriales; Mycobacteriaceae; Mycobacterium; Mycobacterium tuberculosis complex; Mycobacterium tuberculosis;	NCBI: 1773	4418166	99.94	0.00	Yes
NCBI Assembly	GCA_002703725.1	NCBI: cellular organisms; Bacteria; Terrabacteria group; Actinobacteria; Actinomycetia; Corynebacteriales; Mycobacteriaceae; Mycobacterium; Mycobacterium tuberculosis complex; Mycobacterium tuberculosis; Mycobacterium tuberculosis variant bovis;	NCBI: 1765	4296237	98.11	1.00	Yes
NCBI Assembly	GCA_002089715.1	NCBI: cellular organisms; Bacteria; Terrabacteria group; Actinobacteria; Actinomycetia; Corynebacteriales; Mycobacteriaceae; Mycobacterium; Mycobacterium avium complex (MAC); Mycobacterium avium; Mycobacterium avium subsp. hominissuis;	NCBI: 439334	5249919	99.33	0.28	Yes
NCBI Assembly	GCA_002129275.1	NCBI: cellular organisms; Bacteria; Terrabacteria group; Actinobacteria; Actinomycetia; Corynebacteriales; Mycobacteriaceae; Mycobacterium; Mycobacterium tuberculosis complex; Mycobacterium tuberculosis;	NCBI: 1773	4404692	99.94	0.00	Yes
NCBI Assembly	GCA_002086935.1	NCBI: cellular organisms; Bacteria; Terrabacteria group; Actinobacteria; Actinomycetia; Corynebacteriales; Mycobacteriaceae; Mycobacterium; Mycobacterium persicum;	NCBI: 1487726	6267578	98.48	1.17	Yes
NCBI Assembly	GCA_002101585.1	NCBI: cellular organisms; Bacteria; Terrabacteria group; Actinobacteria; Actinomycetia; Corynebacteriales; Mycobacteriaceae; Mycolicibacter; Mycolicibacter engbaekii；	NCBI: 188915	4521435	100.00	0.15	Yes
NCBI Assembly	GCA_001944455.1	NCBI: cellular organisms; Bacteria; Terrabacteria group; Actinobacteria; Actinomycetia; Corynebacteriales; Mycobacteriaceae; Mycobacterium; Mycobacterium tuberculosis complex; Mycobacterium tuberculosis;	NCBI: 1773	4385779	99.94	0.00	Yes
NCBI Assembly	GCA_002102215.1	NCBI: cellular organisms; Bacteria; Terrabacteria group; Actinobacteria; Actinomycetia; Corynebacteriales; Mycobacteriaceae; Mycobacterium; Mycobacterium lacus；	NCBI: 169765	4905288	99.67	1.33	Yes
NCBI Assembly	GCA_002447015.1	NCBI: cellular organisms; Bacteria; Terrabacteria group; Actinobacteria; Actinomycetia; Corynebacteriales; Mycobacteriaceae; Mycobacterium; Mycobacterium tuberculosis complex; Mycobacterium tuberculosis;	NCBI: 1773	4411436	99.94	0.00	Yes

NCBI Assembly	GCA_002131815.1	NCBI: cellular organisms; Bacteria; Terrabacteria group; Actinobacteria; Actinomycetia; Corynebacteriales; Mycobacteriaceae; Mycobacterium; Mycobacterium tuberculosis complex; Mycobacterium tuberculosis;	NCBI: 1773	4383820	99.94	0.00	Yes
NCBI Assembly	GCA_002447975.1	NCBI: cellular organisms; Bacteria; Terrabacteria group; Actinobacteria; Actinomycetia; Corynebacteriales; Mycobacteriaceae; Mycobacterium; Mycobacterium tuberculosis complex; Mycobacterium tuberculosis;	NCBI: 1773	4411479	99.94	0.00	Yes
NCBI Assembly	GCA_002129925.1	NCBI: cellular organisms; Bacteria; Terrabacteria group; Actinobacteria; Actinomycetia; Corynebacteriales; Mycobacteriaceae; Mycobacterium; Mycobacterium tuberculosis complex; Mycobacterium tuberculosis;	NCBI: 1773	4400881	99.94	0.00	Yes
NCBI Assembly	GCA_002089735.1	NCBI: cellular organisms; Bacteria; Terrabacteria group; Actinobacteria; Actinomycetia; Corynebacteriales; Mycobacteriaceae; Mycobacterium; Mycobacterium avium complex (MAC); Mycobacterium avium; Mycobacterium avium subsp. hominissuis;	NCBI: 439334	5751252	99.39	0.08	Yes
NCBI Assembly	GCA_002703735.1	NCBI: cellular organisms; Bacteria; Terrabacteria group; Actinobacteria; Actinomycetia; Corynebacteriales; Mycobacteriaceae; Mycobacterium; Mycobacterium tuberculosis complex; Mycobacterium tuberculosis; Mycobacterium tuberculosis variant bovis;	NCBI: 1765	4377535	99.78	2.22	Yes
NCBI Assembly	GCA_002129305.1	NCBI: cellular organisms; Bacteria; Terrabacteria group; Actinobacteria; Actinomycetia; Corynebacteriales; Mycobacteriaceae; Mycobacterium; Mycobacterium tuberculosis complex; Mycobacterium tuberculosis;	NCBI: 1773	4376246	99.94	0.00	Yes
NCBI Assembly	GCA_002087305.1	NCBI: cellular organisms; Bacteria; Terrabacteria group; Actinobacteria; Actinomycetia; Corynebacteriales; Mycobacteriaceae; Mycobacterium; Mycobacterium tuberculosis complex; Mycobacterium tuberculosis;	NCBI: 1773	4356488	99.86	0.00	Yes
NCBI Assembly	GCA_002101595.1	NCBI: cellular organisms; Bacteria; Terrabacteria group; Actinobacteria; Actinomycetia; Corynebacteriales; Mycobacteriaceae; Mycobacterium; Mycobacterium celatum;	NCBI: 28045	4720680	100.00	0.55	Yes
NCBI Assembly	GCA_001944465.1	NCBI: cellular organisms; Bacteria; Terrabacteria group; Actinobacteria; Actinomycetia; Corynebacteriales; Mycobacteriaceae; Mycobacterium; Mycobacterium tuberculosis complex; Mycobacterium tuberculosis;	NCBI: 1773	4382185	99.94	0.00	Yes
NCBI Assembly	GCA_002102225.1	NCBI: cellular organisms; Bacteria; Terrabacteria group; Actinobacteria; Actinomycetia; Corynebacteriales; Mycobacteriaceae; Mycobacterium; Mycobacterium simiae complex; Mycobacterium interjectum;	NCBI: 33895	5903836	99.86	1.32	Yes
NCBI Assembly	GCA_002135065.1	NCBI: cellular organisms; Bacteria; Terrabacteria group; Actinobacteria; Actinomycetia; Corynebacteriales; Mycobacteriaceae; Mycobacterium; Mycobacterium tuberculosis complex; Mycobacterium tuberculosis;	NCBI: 1773	4386393	99.94	0.00	Yes
NCBI Assembly	GCA_002447035.1	NCBI: cellular organisms; Bacteria; Terrabacteria group; Actinobacteria; Actinomycetia; Corynebacteriales; Mycobacteriaceae; Mycobacterium; Mycobacterium tuberculosis complex; Mycobacterium tuberculosis;	NCBI: 1773	4411353	99.94	0.00	Yes
NCBI Assembly	GCA_002129945.1	NCBI: cellular organisms; Bacteria; Terrabacteria group; Actinobacteria; Actinomycetia; Corynebacteriales; Mycobacteriaceae; Mycobacterium; Mycobacterium tuberculosis complex; Mycobacterium tuberculosis;	NCBI: 1773	4392586	99.94	0.00	Yes
NCBI Assembly	GCA_002447995.1	NCBI: cellular organisms; Bacteria; Terrabacteria group; Actinobacteria; Actinomycetia; Corynebacteriales; Mycobacteriaceae; Mycobacterium; Mycobacterium tuberculosis complex; Mycobacterium tuberculosis;	NCBI: 1773	4411352	99.94	0.00	Yes
NCBI Assembly	GCA_002089755.1	NCBI: cellular organisms; Bacteria; Terrabacteria group; Actinobacteria; Actinomycetia; Corynebacteriales; Mycobacteriaceae; Mycobacterium; Mycobacterium avium complex (MAC); Mycobacterium avium; Mycobacterium avium subsp. hominissuis;	NCBI: 439334	5208636	99.83	0.40	Yes
NCBI Assembly	GCA_002129325.1	NCBI: cellular organisms; Bacteria; Terrabacteria group; Actinobacteria; Actinomycetia; Corynebacteriales; Mycobacteriaceae; Mycobacterium; Mycobacterium tuberculosis complex; Mycobacterium tuberculosis;	NCBI: 1773	4405257	99.94	0.00	Yes
NCBI Assembly	GCA_002087345.1	NCBI: cellular organisms; Bacteria; Terrabacteria group; Actinobacteria; Actinomycetia; Corynebacteriales; Mycobacteriaceae; Mycobacterium; Mycobacterium tuberculosis complex; Mycobacterium tuberculosis;	NCBI: 1773	4351699	99.86	0.00	Yes
NCBI Assembly	GCA_002703765.1	NCBI: cellular organisms; Bacteria; Terrabacteria group; Actinobacteria; Actinomycetia; Corynebacteriales; Mycobacteriaceae; Mycobacterium; Mycobacterium tuberculosis complex; Mycobacterium tuberculosis; Mycobacterium tuberculosis variant bovis;	NCBI: 1765	4503962	98.44	3.44	Yes

NCBI Assembly	GCA_002101635.1	NCBI: cellular organisms; Bacteria; Terrabacteria group; Actinobacteria; Actinomycetia; Corynebacteriales; Mycobacteriaceae; Mycobacterium; Mycobacterium simiae complex; Mycobacterium florentinum;	NCBI: 292462	6178353	99.61	1.50	Yes
NCBI Assembly	GCA_001944485.1	NCBI: cellular organisms; Bacteria; Terrabacteria group; Actinobacteria; Actinomycetia; Corynebacteriales; Mycobacteriaceae; Mycobacterium; Mycobacterium tuberculosis complex; Mycobacterium tuberculosis;	NCBI: 1773	4393926	99.94	0.00	Yes
NCBI Assembly	GCA_002102255.1	NCBI: cellular organisms; Bacteria; Terrabacteria group; Actinobacteria; Actinomycetia; Corynebacteriales; Mycobacteriaceae; Mycobacterium; Mycobacterium nebraskense;	NCBI: 244292	6727177	99.94	1.14	Yes
NCBI Assembly	GCA_002140035.1	NCBI: cellular organisms; Bacteria; Terrabacteria group; Actinobacteria; Actinomycetia; Corynebacteriales; Mycobacteriaceae; Mycobacteroides; Mycobacteroides abscessus; Mycobacteroides abscessus subsp. massiliense;	NCBI: 1962118	4936470	100.00	0.00	Yes
NCBI Assembly	GCA_002129955.1	NCBI: cellular organisms; Bacteria; Terrabacteria group; Actinobacteria; Actinomycetia; Corynebacteriales; Mycobacteriaceae; Mycobacterium; Mycobacterium tuberculosis complex; Mycobacterium tuberculosis;	NCBI: 1773	4402983	99.94	0.00	Yes
NCBI Assembly	GCA_002447055.1	NCBI: cellular organisms; Bacteria; Terrabacteria group; Actinobacteria; Actinomycetia; Corynebacteriales; Mycobacteriaceae; Mycobacterium; Mycobacterium tuberculosis complex; Mycobacterium tuberculosis;	NCBI: 1773	4411315	99.94	0.00	Yes
NCBI Assembly	GCA_002089775.1	NCBI: cellular organisms; Bacteria; Terrabacteria group; Actinobacteria; Actinomycetia; Corynebacteriales; Mycobacteriaceae; Mycobacterium; Mycobacterium avium complex (MAC); Mycobacterium avium; Mycobacterium avium subsp. hominissuis;	NCBI: 439334	5442059	99.61	0.07	Yes
NCBI Assembly	GCA_002129345.1	NCBI: cellular organisms; Bacteria; Terrabacteria group; Actinobacteria; Actinomycetia; Corynebacteriales; Mycobacteriaceae; Mycobacterium; Mycobacterium tuberculosis complex; Mycobacterium tuberculosis;	NCBI: 1773	4401586	99.94	0.00	Yes
NCBI Assembly	GCA_002448015.1	NCBI: cellular organisms; Bacteria; Terrabacteria group; Actinobacteria; Actinomycetia; Corynebacteriales; Mycobacteriaceae; Mycobacterium; Mycobacterium tuberculosis complex; Mycobacterium tuberculosis;	NCBI: 1773	4411454	99.94	0.00	Yes
NCBI Assembly	GCA_002089135.1	NCBI: cellular organisms; Bacteria; Terrabacteria group; Actinobacteria; Actinomycetia; Corynebacteriales; Mycobacteriaceae; Mycobacterium; Mycobacterium avium complex (MAC); Mycobacterium avium; Mycobacterium avium subsp. hominissuis;	NCBI: 439334	4981469	99.00	0.86	Yes
NCBI Assembly	GCA_002801025.1	NCBI: cellular organisms; Bacteria; Terrabacteria group; Actinobacteria; Actinomycetia; Corynebacteriales; Mycobacteriaceae; Mycobacteroides; Mycobacteroides abscessus; Mycobacteroides abscessus subsp. abscessus;	NCBI: 1185650	5368361	89.24	40.68	No
NCBI Assembly	GCA_002101655.1	NCBI: cellular organisms; Bacteria; Terrabacteria group; Actinobacteria; Actinomycetia; Corynebacteriales; Mycobacteriaceae; Mycolicibacter; Mycolicibacter hiberniae;	NCBI: 29314	4342192	99.55	0.15	Yes
NCBI Assembly	GCA_001944525.1	NCBI: cellular organisms; Bacteria; Terrabacteria group; Actinobacteria; Actinomycetia; Corynebacteriales; Mycobacteriaceae; Mycobacterium; Mycobacterium tuberculosis complex; Mycobacterium tuberculosis;	NCBI: 1773	4374083	99.94	0.00	Yes
NCBI Assembly	GCA_002102265.1	NCBI: cellular organisms; Bacteria; Terrabacteria group; Actinobacteria; Actinomycetia; Corynebacteriales; Mycobacteriaceae; Mycolicibacter; Mycolicibacter longobardus;	NCBI: 1108812	4812247	100.00	1.06	Yes
NCBI Assembly	GCA_002148175.1	NCBI: cellular organisms; Bacteria; Terrabacteria group; Actinobacteria; Actinomycetia; Corynebacteriales; Mycobacteriaceae; Mycobacterium; Mycobacterium avium complex (MAC); Mycobacterium intracellulare; Mycobacterium intracellulare subsp. yongonense;	NCBI: 1203599	5445538	99.94	0.07	Yes
NCBI Assembly	GCA_002129985.1	NCBI: cellular organisms; Bacteria; Terrabacteria group; Actinobacteria; Actinomycetia; Corynebacteriales; Mycobacteriaceae; Mycobacterium; Mycobacterium tuberculosis complex; Mycobacterium tuberculosis;	NCBI: 1773	4373292	99.94	0.00	Yes
NCBI Assembly	GCA_002089795.1	NCBI: cellular organisms; Bacteria; Terrabacteria group; Actinobacteria; Actinomycetia; Corynebacteriales; Mycobacteriaceae; Mycobacterium; Mycobacterium avium complex (MAC); Mycobacterium avium; Mycobacterium avium subsp. hominissuis;	NCBI: 439334	5514993	99.72	0.32	Yes
NCBI Assembly	GCA_002447075.1	NCBI: cellular organisms; Bacteria; Terrabacteria group; Actinobacteria; Actinomycetia; Corynebacteriales; Mycobacteriaceae; Mycobacterium; Mycobacterium tuberculosis complex; Mycobacterium tuberculosis;	NCBI: 1773	4411280	99.94	0.00	Yes

NCBI Assembly	GCA_002089155.1	NCBI: cellular organisms; Bacteria; Terrabacteria group; Actinobacteria; Actinomycetia; Corynebacteriales; Mycobacteriaceae; Mycobacterium; Mycobacterium avium complex (MAC); Mycobacterium avium; Mycobacterium avium subsp. hominissuis;	NCBI: 439334	5449121	99.94	0.17	Yes
NCBI Assembly	GCA_002129365.1	NCBI: cellular organisms; Bacteria; Terrabacteria group; Actinobacteria; Actinomycetia; Corynebacteriales; Mycobacteriaceae; Mycobacterium; Mycobacterium tuberculosis complex; Mycobacterium tuberculosis;	NCBI: 1773	4396280	99.94	0.00	Yes
NCBI Assembly	GCA_002448035.1	NCBI: cellular organisms; Bacteria; Terrabacteria group; Actinobacteria; Actinomycetia; Corynebacteriales; Mycobacteriaceae; Mycobacterium; Mycobacterium tuberculosis complex; Mycobacterium tuberculosis;	NCBI: 1773	4411157	99.94	0.00	Yes
NCBI Assembly	GCA_002801065.1	NCBI: cellular organisms; Bacteria; Terrabacteria group; Actinobacteria; Actinomycetia; Corynebacteriales; Mycobacteriaceae; Mycobacteroides; Mycobacteroides abscessus; Mycobacteroides abscessus subsp. abscessus;	NCBI: 1185650	5006611	100.00	0.00	Yes
NCBI Assembly	GCA_001944535.1	NCBI: cellular organisms; Bacteria; Terrabacteria group; Actinobacteria; Actinomycetia; Corynebacteriales; Mycobacteriaceae; Mycobacterium; Mycobacterium tuberculosis complex; Mycobacterium tuberculosis;	NCBI: 1773	4391116	99.78	0.00	Yes
NCBI Assembly	GCA_002102295.1	NCBI: cellular organisms; Bacteria; Terrabacteria group; Actinobacteria; Actinomycetia; Corynebacteriales; Mycobacteriaceae; Mycobacterium; Mycobacterium simiae complex; Mycobacterium paraense;	NCBI: 767916	5805377	99.94	2.32	Yes
NCBI Assembly	GCA_002101665.1	NCBI: cellular organisms; Bacteria; Terrabacteria group; Actinobacteria; Actinomycetia; Corynebacteriales; Mycobacteriaceae; Mycobacteroides; Mycobacteroides immunogenum;	NCBI: 83262	5546219	99.61	2.15	Yes
NCBI Assembly	GCA_002148195.1	NCBI: cellular organisms; Bacteria; Terrabacteria group; Actinobacteria; Actinomycetia; Corynebacteriales; Mycobacteriaceae; Mycobacterium; Mycobacterium avium complex (MAC); Mycobacterium intracellulare; Mycobacterium intracellulare subsp. yongonense;	NCBI: 1203599	5435152	99.94	0.07	Yes
NCBI Assembly	GCA_002129995.1	NCBI: cellular organisms; Bacteria; Terrabacteria group; Actinobacteria; Actinomycetia; Corynebacteriales; Mycobacteriaceae; Mycobacterium; Mycobacterium tuberculosis complex; Mycobacterium tuberculosis;	NCBI: 1773	4397495	99.94	0.00	Yes
NCBI Assembly	GCA_002089815.1	NCBI: cellular organisms; Bacteria; Terrabacteria group; Actinobacteria; Actinomycetia; Corynebacteriales; Mycobacteriaceae; Mycobacterium; Mycobacterium avium complex (MAC); Mycobacterium avium; Mycobacterium avium subsp. hominissuis;	NCBI: 439334	5279502	99.68	0.32	Yes
NCBI Assembly	GCA_002447095.1	NCBI: cellular organisms; Bacteria; Terrabacteria group; Actinobacteria; Actinomycetia; Corynebacteriales; Mycobacteriaceae; Mycobacterium; Mycobacterium tuberculosis complex; Mycobacterium tuberculosis;	NCBI: 1773	4411215	99.94	0.00	Yes
NCBI Assembly	GCA_002129385.1	NCBI: cellular organisms; Bacteria; Terrabacteria group; Actinobacteria; Actinomycetia; Corynebacteriales; Mycobacteriaceae; Mycobacterium; Mycobacterium tuberculosis complex; Mycobacterium tuberculosis;	NCBI: 1773	4395609	99.94	0.00	Yes
NCBI Assembly	GCA_002089175.1	NCBI: cellular organisms; Bacteria; Terrabacteria group; Actinobacteria; Actinomycetia; Corynebacteriales; Mycobacteriaceae; Mycobacterium; Mycobacterium avium complex (MAC); Mycobacterium avium; Mycobacterium avium subsp. hominissuis;	NCBI: 439334	5895354	99.83	1.60	Yes
NCBI Assembly	GCA_001944545.1	NCBI: cellular organisms; Bacteria; Terrabacteria group; Actinobacteria; Actinomycetia; Corynebacteriales; Mycobacteriaceae; Mycobacterium; Mycobacterium tuberculosis complex; Mycobacterium tuberculosis;	NCBI: 1773	4390036	99.94	0.00	Yes
NCBI Assembly	GCA_002448055.1	NCBI: cellular organisms; Bacteria; Terrabacteria group; Actinobacteria; Actinomycetia; Corynebacteriales; Mycobacteriaceae; Mycobacterium; Mycobacterium tuberculosis complex; Mycobacterium tuberculosis;	NCBI: 1773	4411316	99.94	0.00	Yes
NCBI Assembly	GCA_002801085.1	NCBI: cellular organisms; Bacteria; Terrabacteria group; Actinobacteria; Actinomycetia; Corynebacteriales; Mycobacteriaceae; Mycobacteroides; Mycobacteroides abscessus; Mycobacteroides abscessus subsp. abscessus;	NCBI: 1185650	5153314	100.00	0.67	Yes
NCBI Assembly	GCA_002101675.1	NCBI: cellular organisms; Bacteria; Terrabacteria group; Actinobacteria; Actinomycetia; Corynebacteriales; Mycobacteriaceae; Mycobacterium; Mycobacterium gordonae;	NCBI: 1778	7601632	99.94	3.08	Yes
NCBI Assembly	GCA_002102305.1	NCBI: cellular organisms; Bacteria; Terrabacteria group; Actinobacteria; Actinomycetia; Corynebacteriales; Mycobacteriaceae; Mycobacterium; Mycobacterium simiae complex; Mycobacterium paraense;	NCBI: 767916	5806129	99.94	2.32	Yes

NCBI Assembly	GCA_002157835.1	NCBI: cellular organisms; Bacteria; Terrabacteria group; Actinobacteria; Actinomycetia; Corynebacteriales; Mycobacteriaceae; Mycobacterium; Mycobacterium dioxanotrophicus;	NCBI: 482462	8080416	99.62	1.98	Yes
NCBI Assembly	GCA_002089835.1	NCBI: cellular organisms; Bacteria; Terrabacteria group; Actinobacteria; Actinomycetia; Corynebacteriales; Mycobacteriaceae; Mycobacterium; Mycobacterium avium complex (MAC); Mycobacterium avium; Mycobacterium avium subsp. hominissuis;	NCBI: 439334	5588265	99.94	0.36	Yes
NCBI Assembly	GCA_002130025.1	NCBI: cellular organisms; Bacteria; Terrabacteria group; Actinobacteria; Actinomycetia; Corynebacteriales; Mycobacteriaceae; Mycobacterium; Mycobacterium tuberculosis complex; Mycobacterium tuberculosis;	NCBI: 1773	4392930	99.94	0.00	Yes
NCBI Assembly	GCA_002129405.1	NCBI: cellular organisms; Bacteria; Terrabacteria group; Actinobacteria; Actinomycetia; Corynebacteriales; Mycobacteriaceae; Mycobacterium; Mycobacterium tuberculosis complex; Mycobacterium tuberculosis;	NCBI: 1773	4401160	99.94	0.00	Yes
NCBI Assembly	GCA_002089195.1	NCBI: cellular organisms; Bacteria; Terrabacteria group; Actinobacteria; Actinomycetia; Corynebacteriales; Mycobacteriaceae; Mycobacterium; Mycobacterium avium complex (MAC); Mycobacterium avium; Mycobacterium avium subsp. hominissuis;	NCBI: 439334	5076722	99.48	0.09	Yes
NCBI Assembly	GCA_002447115.1	NCBI: cellular organisms; Bacteria; Terrabacteria group; Actinobacteria; Actinomycetia; Corynebacteriales; Mycobacteriaceae; Mycobacterium; Mycobacterium tuberculosis complex; Mycobacterium tuberculosis;	NCBI: 1773	4411148	99.94	0.00	Yes
NCBI Assembly	GCA_001944555.1	NCBI: cellular organisms; Bacteria; Terrabacteria group; Actinobacteria; Actinomycetia; Corynebacteriales; Mycobacteriaceae; Mycobacterium; Mycobacterium tuberculosis complex; Mycobacterium tuberculosis;	NCBI: 1773	4384816	99.94	0.00	Yes
NCBI Assembly	GCA_002448075.1	NCBI: cellular organisms; Bacteria; Terrabacteria group; Actinobacteria; Actinomycetia; Corynebacteriales; Mycobacteriaceae; Mycobacterium; Mycobacterium tuberculosis complex; Mycobacterium tuberculosis;	NCBI: 1773	4411284	99.94	0.00	Yes
NCBI Assembly	GCA_002101705.1	NCBI: cellular organisms; Bacteria; Terrabacteria group; Actinobacteria; Actinomycetia; Corynebacteriales; Mycobacteriaceae; Mycolicibacterium; Mycolicibacterium iranicum;	NCBI: 912594	6335651	100.00	0.12	Yes
NCBI Assembly	GCA_002102335.1	NCBI: cellular organisms; Bacteria; Terrabacteria group; Actinobacteria; Actinomycetia; Corynebacteriales; Mycobacteriaceae; Mycobacterium; Mycobacterium simiae complex; Mycobacterium parmense;	NCBI: 185642	5891740	99.61	2.51	Yes
NCBI Assembly	GCA_002801095.1	NCBI: cellular organisms; Bacteria; Terrabacteria group; Actinobacteria; Actinomycetia; Corynebacteriales; Mycobacteriaceae; Mycobacteroides; Mycobacteroides abscessus; Mycobacteroides abscessus subsp. abscessus;	NCBI: 1185650	5071257	100.00	0.33	Yes
NCBI Assembly	GCA_002166795.1	NCBI: cellular organisms; Bacteria; Terrabacteria group; Actinobacteria; Actinomycetia; Corynebacteriales; Mycobacteriaceae; Mycobacterium; Mycobacterium avium complex (MAC); Mycobacterium intracellulare; Mycobacterium intracellulare subsp. chimaera;	NCBI: 222805	6247694	99.94	1.15	Yes
NCBI Assembly	GCA_002089855.1	NCBI: cellular organisms; Bacteria; Terrabacteria group; Actinobacteria; Actinomycetia; Corynebacteriales; Mycobacteriaceae; Mycobacterium; Mycobacterium avium complex (MAC); Mycobacterium avium; Mycobacterium avium subsp. hominissuis;	NCBI: 439334	5263728	99.67	0.25	Yes
NCBI Assembly	GCA_002130035.1	NCBI: cellular organisms; Bacteria; Terrabacteria group; Actinobacteria; Actinomycetia; Corynebacteriales; Mycobacteriaceae; Mycobacterium; Mycobacterium tuberculosis complex; Mycobacterium tuberculosis;	NCBI: 1773	4399691	99.94	0.00	Yes
NCBI Assembly	GCA_002089215.1	NCBI: cellular organisms; Bacteria; Terrabacteria group; Actinobacteria; Actinomycetia; Corynebacteriales; Mycobacteriaceae; Mycobacterium; Mycobacterium avium complex (MAC); Mycobacterium avium; Mycobacterium avium subsp. hominissuis;	NCBI: 439334	5129780	99.31	0.25	Yes
NCBI Assembly	GCA_002129425.1	NCBI: cellular organisms; Bacteria; Terrabacteria group; Actinobacteria; Actinomycetia; Corynebacteriales; Mycobacteriaceae; Mycobacterium; Mycobacterium tuberculosis complex; Mycobacterium tuberculosis;	NCBI: 1773	4392597	99.94	0.00	Yes
NCBI Assembly	GCA_001944605.1	NCBI: cellular organisms; Bacteria; Terrabacteria group; Actinobacteria; Actinomycetia; Corynebacteriales; Mycobacteriaceae; Mycobacterium; Mycobacterium tuberculosis complex; Mycobacterium tuberculosis;	NCBI: 1773	4364887	99.61	0.00	Yes
NCBI Assembly	GCA_002447135.1	NCBI: cellular organisms; Bacteria; Terrabacteria group; Actinobacteria; Actinomycetia; Corynebacteriales; Mycobacteriaceae; Mycobacterium; Mycobacterium tuberculosis complex; Mycobacterium tuberculosis;	NCBI: 1773	4411121	99.94	0.00	Yes

NCBI Assembly	GCA_002102345.1	NCBI: cellular organisms; Bacteria; Terrabacteria group; Actinobacteria; Actinomycetia; Corynebacteriales; Mycobacteriaceae; Mycolicibacterium; Mycolicibacterium peregrinum;	NCBI: 43304	7050152	100.00	0.50	Yes
NCBI Assembly	GCA_002448095.1	NCBI: cellular organisms; Bacteria; Terrabacteria group; Actinobacteria; Actinomycetia; Corynebacteriales; Mycobacteriaceae; Mycobacterium; Mycobacterium tuberculosis complex; Mycobacterium tuberculosis;	NCBI: 1773	4411314	99.94	0.00	Yes
NCBI Assembly	GCA_002101735.1	NCBI: cellular organisms; Bacteria; Terrabacteria group; Actinobacteria; Actinomycetia; Corynebacteriales; Mycobacteriaceae; Mycobacterium; Mycobacterium kyorinense;	NCBI: 487514	5606671	100.00	0.24	Yes
NCBI Assembly	GCA_002801105.1	NCBI: cellular organisms; Bacteria; Terrabacteria group; Actinobacteria; Actinomycetia; Corynebacteriales; Mycobacteriaceae; Mycobacteroides; Mycobacteroides abscessus; Mycobacteroides abscessus subsp. abscessus;	NCBI: 1185650	5012024	100.00	0.50	Yes
NCBI Assembly	GCA_002174295.1	NCBI: cellular organisms; Bacteria; Terrabacteria group; Actinobacteria; Actinomycetia; Corynebacteriales; Mycobacteriaceae; Mycobacterium; Mycobacterium avium complex (MAC); Mycobacterium avium; Mycobacterium avium subsp. paratuberculosis;	NCBI: 1770	4851414	98.50	0.67	Yes
NCBI Assembly	GCA_002089875.1	NCBI: cellular organisms; Bacteria; Terrabacteria group; Actinobacteria; Actinomycetia; Corynebacteriales; Mycobacteriaceae; Mycobacterium; Mycobacterium avium complex (MAC); Mycobacterium avium; Mycobacterium avium subsp. hominissuis;	NCBI: 439334	5155951	99.47	0.37	Yes
NCBI Assembly	GCA_002130065.1	NCBI: cellular organisms; Bacteria; Terrabacteria group; Actinobacteria; Actinomycetia; Corynebacteriales; Mycobacteriaceae; Mycobacterium; Mycobacterium tuberculosis complex; Mycobacterium tuberculosis;	NCBI: 1773	4385143	99.86	0.00	Yes
NCBI Assembly	GCA_002089235.1	NCBI: cellular organisms; Bacteria; Terrabacteria group; Actinobacteria; Actinomycetia; Corynebacteriales; Mycobacteriaceae; Mycobacterium; Mycobacterium avium complex (MAC); Mycobacterium avium; Mycobacterium avium subsp. hominissuis;	NCBI: 439334	5251565	99.79	0.13	Yes
NCBI Assembly	GCA_002129445.1	NCBI: cellular organisms; Bacteria; Terrabacteria group; Actinobacteria; Actinomycetia; Corynebacteriales; Mycobacteriaceae; Mycobacterium; Mycobacterium tuberculosis complex; Mycobacterium tuberculosis;	NCBI: 1773	4411751	99.94	0.00	Yes
NCBI Assembly	GCA_001944615.1	NCBI: cellular organisms; Bacteria; Terrabacteria group; Actinobacteria; Actinomycetia; Corynebacteriales; Mycobacteriaceae; Mycobacterium; Mycobacterium tuberculosis complex; Mycobacterium tuberculosis;	NCBI: 1773	4398018	99.94	0.00	Yes
NCBI Assembly	GCA_002102355.1	NCBI: cellular organisms; Bacteria; Terrabacteria group; Actinobacteria; Actinomycetia; Corynebacteriales; Mycobacteriaceae; Mycobacterium; Mycobacterium simiae complex; Mycobacterium sherrisii;	NCBI: 243061	5562166	99.94	1.53	Yes
NCBI Assembly	GCA_002447155.1	NCBI: cellular organisms; Bacteria; Terrabacteria group; Actinobacteria; Actinomycetia; Corynebacteriales; Mycobacteriaceae; Mycobacterium; Mycobacterium tuberculosis complex; Mycobacterium tuberculosis;	NCBI: 1773	4411217	99.94	0.00	Yes
NCBI Assembly	GCA_002448115.1	NCBI: cellular organisms; Bacteria; Terrabacteria group; Actinobacteria; Actinomycetia; Corynebacteriales; Mycobacteriaceae; Mycobacterium; Mycobacterium tuberculosis complex; Mycobacterium tuberculosis;	NCBI: 1773	4411369	99.94	0.00	Yes
NCBI Assembly	GCA_002101745.1	NCBI: cellular organisms; Bacteria; Terrabacteria group; Actinobacteria; Actinomycetia; Corynebacteriales; Mycobacteriaceae; Mycobacterium; Mycobacterium simiae complex; Mycobacterium kubicae;	NCBI: 120959	5832568	99.61	0.67	Yes
NCBI Assembly	GCA_002801115.1	NCBI: cellular organisms; Bacteria; Terrabacteria group; Actinobacteria; Actinomycetia; Corynebacteriales; Mycobacteriaceae; Mycobacteroides; Mycobacteroides abscessus; Mycobacteroides abscessus subsp. abscessus;	NCBI: 1185650	4839754	99.33	0.50	Yes
NCBI Assembly	GCA_002180005.1	NCBI: cellular organisms; Bacteria; Terrabacteria group; Actinobacteria; Actinomycetia; Corynebacteriales; Mycobacteriaceae; Mycobacterium; Mycobacterium avium complex (MAC); Mycobacterium avium; Mycobacterium avium subsp. paratuberculosis;	NCBI: 1770	4847731	98.38	0.33	Yes
NCBI Assembly	GCA_002089895.1	NCBI: cellular organisms; Bacteria; Terrabacteria group; Actinobacteria; Actinomycetia; Corynebacteriales; Mycobacteriaceae; Mycobacterium; Mycobacterium avium complex (MAC); Mycobacterium avium; Mycobacterium avium subsp. hominissuis;	NCBI: 439334	5398474	99.86	0.82	Yes
NCBI Assembly	GCA_002130075.1	NCBI: cellular organisms; Bacteria; Terrabacteria group; Actinobacteria; Actinomycetia; Corynebacteriales; Mycobacteriaceae; Mycobacterium; Mycobacterium tuberculosis complex; Mycobacterium tuberculosis;	NCBI: 1773	4352683	99.94	0.00	Yes

NCBI Assembly	GCA_002089255.1	NCBI: cellular organisms; Bacteria; Terrabacteria group; Actinobacteria; Actinomycetia; Corynebacteriales; Mycobacteriaceae; Mycobacterium; Mycobacterium avium complex (MAC); Mycobacterium avium; Mycobacterium avium subsp. hominissuis;	NCBI: 439334	5258000	99.68	0.41	Yes
NCBI Assembly	GCA_001944625.1	NCBI: cellular organisms; Bacteria; Terrabacteria group; Actinobacteria; Actinomycetia; Corynebacteriales; Mycobacteriaceae; Mycobacterium; Mycobacterium tuberculosis complex; Mycobacterium tuberculosis;	NCBI: 1773	4380006	99.94	0.00	Yes
NCBI Assembly	GCA_002129465.1	NCBI: cellular organisms; Bacteria; Terrabacteria group; Actinobacteria; Actinomycetia; Corynebacteriales; Mycobacteriaceae; Mycobacterium; Mycobacterium tuberculosis complex; Mycobacterium tuberculosis;	NCBI: 1773	4398729	99.94	0.00	Yes
NCBI Assembly	GCA_002102395.1	NCBI: cellular organisms; Bacteria; Terrabacteria group; Actinobacteria; Actinomycetia; Corynebacteriales; Mycobacteriaceae; Mycolicibacillus; Mycolicibacillus trivialis;	NCBI: 1798	3591083	100.00	0.15	Yes
NCBI Assembly	GCA_002447175.1	NCBI: cellular organisms; Bacteria; Terrabacteria group; Actinobacteria; Actinomycetia; Corynebacteriales; Mycobacteriaceae; Mycobacterium; Mycobacterium tuberculosis complex; Mycobacterium tuberculosis;	NCBI: 1773	4411143	99.94	0.00	Yes
NCBI Assembly	GCA_002101775.1	NCBI: cellular organisms; Bacteria; Terrabacteria group; Actinobacteria; Actinomycetia; Corynebacteriales; Mycobacteriaceae; Mycolicibacter; Mycolicibacter nonchromogenicus;	NCBI: 1782	4465329	99.55	0.15	Yes
NCBI Assembly	GCA_002448135.1	NCBI: cellular organisms; Bacteria; Terrabacteria group; Actinobacteria; Actinomycetia; Corynebacteriales; Mycobacteriaceae; Mycobacterium; Mycobacterium tuberculosis complex; Mycobacterium tuberculosis;	NCBI: 1773	4411457	99.94	0.00	Yes
NCBI Assembly	GCA_002089915.1	NCBI: cellular organisms; Bacteria; Terrabacteria group; Actinobacteria; Actinomycetia; Corynebacteriales; Mycobacteriaceae; Mycobacterium; Mycobacterium avium complex (MAC); Mycobacterium avium; Mycobacterium avium subsp. hominissuis;	NCBI: 439334	5741908	99.83	0.53	Yes
NCBI Assembly	GCA_002193045.1	NCBI: cellular organisms; Bacteria; Terrabacteria group; Actinobacteria; Actinomycetia; Corynebacteriales; Mycobacteriaceae; Mycobacterium; Mycobacterium tuberculosis complex; Mycobacterium tuberculosis;	NCBI: 1773	4311779	97.03	0.78	Yes
NCBI Assembly	GCA_002130105.1	NCBI: cellular organisms; Bacteria; Terrabacteria group; Actinobacteria; Actinomycetia; Corynebacteriales; Mycobacteriaceae; Mycobacterium; Mycobacterium tuberculosis complex; Mycobacterium tuberculosis;	NCBI: 1773	4403955	99.94	0.00	Yes
NCBI Assembly	GCA_002089275.1	NCBI: cellular organisms; Bacteria; Terrabacteria group; Actinobacteria; Actinomycetia; Corynebacteriales; Mycobacteriaceae; Mycobacterium; Mycobacterium avium complex (MAC); Mycobacterium avium; Mycobacterium avium subsp. hominissuis;	NCBI: 439334	5221809	99.61	0.37	Yes
NCBI Assembly	GCA_002801175.1	NCBI: cellular organisms; Bacteria; Terrabacteria group; Actinobacteria; Actinomycetia; Corynebacteriales; Mycobacteriaceae; Mycobacteroides; Mycobacteroides abscessus; Mycobacteroides abscessus subsp. abscessus;	NCBI: 1185650	5177049	100.00	0.00	Yes
NCBI Assembly	GCA_002104675.1	NCBI: cellular organisms; Bacteria; Terrabacteria group; Actinobacteria; Actinomycetia; Corynebacteriales; Mycobacteriaceae; Mycobacterium; Mycobacterium decipiens;	NCBI: 1430326	5225940	99.61	0.86	Yes
NCBI Assembly	GCA_002129485.1	NCBI: cellular organisms; Bacteria; Terrabacteria group; Actinobacteria; Actinomycetia; Corynebacteriales; Mycobacteriaceae; Mycobacterium; Mycobacterium tuberculosis complex; Mycobacterium tuberculosis;	NCBI: 1773	4408763	99.94	0.00	Yes
NCBI Assembly	GCA_001944635.1	NCBI: cellular organisms; Bacteria; Terrabacteria group; Actinobacteria; Actinomycetia; Corynebacteriales; Mycobacteriaceae; Mycobacterium; Mycobacterium tuberculosis complex; Mycobacterium tuberculosis;	NCBI: 1773	4376362	99.94	0.00	Yes
NCBI Assembly	GCA_002101785.1	NCBI: cellular organisms; Bacteria; Terrabacteria group; Actinobacteria; Actinomycetia; Corynebacteriales; Mycobacteriaceae; Mycobacterium; Mycobacterium simiae complex; Mycobacterium palustre;	NCBI: 153971	6037522	99.94	3.47	Yes
NCBI Assembly	GCA_002447195.1	NCBI: cellular organisms; Bacteria; Terrabacteria group; Actinobacteria; Actinomycetia; Corynebacteriales; Mycobacteriaceae; Mycobacterium; Mycobacterium tuberculosis complex; Mycobacterium tuberculosis;	NCBI: 1773	4411318	99.94	0.00	Yes
NCBI Assembly	GCA_002448155.1	NCBI: cellular organisms; Bacteria; Terrabacteria group; Actinobacteria; Actinomycetia; Corynebacteriales; Mycobacteriaceae; Mycobacterium; Mycobacterium tuberculosis complex; Mycobacterium tuberculosis;	NCBI: 1773	4411442	99.94	0.00	Yes

NCBI Assembly	GCA_002089935.1	NCBI: cellular organisms; Bacteria; Terrabacteria group; Actinobacteria; Actinomycetia; Corynebacteriales; Mycobacteriaceae; Mycobacterium; Mycobacterium avium complex (MAC); Mycobacterium avium; Mycobacterium avium subsp. hominissuis;	NCBI: 439334	5497861	99.68	0.75	Yes
NCBI Assembly	GCA_002193075.1	NCBI: cellular organisms; Bacteria; Terrabacteria group; Actinobacteria; Actinomycetia; Corynebacteriales; Mycobacteriaceae; Mycobacterium; Mycobacterium tuberculosis complex; Mycobacterium tuberculosis;	NCBI: 1773	4288394	96.96	0.00	Yes
NCBI Assembly	GCA_002089295.1	NCBI: cellular organisms; Bacteria; Terrabacteria group; Actinobacteria; Actinomycetia; Corynebacteriales; Mycobacteriaceae; Mycobacterium; Mycobacterium avium complex (MAC); Mycobacterium avium; Mycobacterium avium subsp. hominissuis;	NCBI: 439334	5121915	99.42	0.75	Yes
NCBI Assembly	GCA_002130115.1	NCBI: cellular organisms; Bacteria; Terrabacteria group; Actinobacteria; Actinomycetia; Corynebacteriales; Mycobacteriaceae; Mycobacterium; Mycobacterium tuberculosis complex; Mycobacterium tuberculosis;	NCBI: 1773	4395776	99.94	0.00	Yes
NCBI Assembly	GCA_001944685.1	NCBI: cellular organisms; Bacteria; Terrabacteria group; Actinobacteria; Actinomycetia; Corynebacteriales; Mycobacteriaceae; Mycobacterium; Mycobacterium tuberculosis complex; Mycobacterium tuberculosis;	NCBI: 1773	4370657	99.61	0.00	Yes
NCBI Assembly	GCA_002801205.1	NCBI: cellular organisms; Bacteria; Terrabacteria group; Actinobacteria; Actinomycetia; Corynebacteriales; Mycobacteriaceae; Mycobacteroides; Mycobacteroides abscessus; Mycobacteroides abscessus subsp. abscessus;	NCBI: 1185650	5315816	100.00	0.58	Yes
NCBI Assembly	GCA_002104695.1	NCBI: cellular organisms; Bacteria; Terrabacteria group; Actinobacteria; Actinomycetia; Corynebacteriales; Mycobacteriaceae; Mycobacterium; Mycobacterium tuberculosis complex; Mycobacterium tuberculosis;	NCBI: 1773	4201682	94.25	2.04	Yes
NCBI Assembly	GCA_002129505.1	NCBI: cellular organisms; Bacteria; Terrabacteria group; Actinobacteria; Actinomycetia; Corynebacteriales; Mycobacteriaceae; Mycobacterium; Mycobacterium tuberculosis complex; Mycobacterium tuberculosis;	NCBI: 1773	4409384	99.94	0.00	Yes
NCBI Assembly	GCA_002101815.1	NCBI: cellular organisms; Bacteria; Terrabacteria group; Actinobacteria; Actinomycetia; Corynebacteriales; Mycobacteriaceae; Mycobacterium; Mycobacterium simiae complex; Mycobacterium paraense;	NCBI: 767916	5619528	99.94	0.90	Yes
NCBI Assembly	GCA_002447215.1	NCBI: cellular organisms; Bacteria; Terrabacteria group; Actinobacteria; Actinomycetia; Corynebacteriales; Mycobacteriaceae; Mycobacterium; Mycobacterium tuberculosis complex; Mycobacterium tuberculosis;	NCBI: 1773	4411331	99.94	0.00	Yes
NCBI Assembly	GCA_002448175.1	NCBI: cellular organisms; Bacteria; Terrabacteria group; Actinobacteria; Actinomycetia; Corynebacteriales; Mycobacteriaceae; Mycobacterium; Mycobacterium tuberculosis complex; Mycobacterium tuberculosis;	NCBI: 1773	4411310	99.94	0.00	Yes
NCBI Assembly	GCA_002089955.1	NCBI: cellular organisms; Bacteria; Terrabacteria group; Actinobacteria; Actinomycetia; Corynebacteriales; Mycobacteriaceae; Mycobacterium; Mycobacterium avium complex (MAC); Mycobacterium avium; Mycobacterium avium subsp. hominissuis;	NCBI: 439334	5354245	99.18	0.31	Yes
NCBI Assembly	GCA_002198105.1	NCBI: cellular organisms; Bacteria; Terrabacteria group; Actinobacteria; Actinomycetia; Corynebacteriales; Mycobacteriaceae; Mycolicibacterium; Mycolicibacterium peregrinum;	NCBI: 43304	6931852	100.00	1.38	Yes
NCBI Assembly	GCA_002130135.1	NCBI: cellular organisms; Bacteria; Terrabacteria group; Actinobacteria; Actinomycetia; Corynebacteriales; Mycobacteriaceae; Mycobacterium; Mycobacterium tuberculosis complex; Mycobacterium tuberculosis;	NCBI: 1773	4376610	99.94	0.00	Yes
NCBI Assembly	GCA_002089315.1	NCBI: cellular organisms; Bacteria; Terrabacteria group; Actinobacteria; Actinomycetia; Corynebacteriales; Mycobacteriaceae; Mycobacterium; Mycobacterium avium complex (MAC); Mycobacterium avium; Mycobacterium avium subsp. hominissuis;	NCBI: 439334	5214580	99.72	0.00	Yes
NCBI Assembly	GCA_002723535.1	NCBI: cellular organisms; Bacteria; Terrabacteria group; Actinobacteria; Actinomycetia; Corynebacteriales; Mycobacteriaceae; Mycobacterium; Mycobacterium tuberculosis complex; Mycobacterium tuberculosis; Mycobacterium tuberculosis variant bovis;	NCBI: 1765	4301398	99.78	0.11	Yes
NCBI Assembly	GCA_001944695.1	NCBI: cellular organisms; Bacteria; Terrabacteria group; Actinobacteria; Actinomycetia; Corynebacteriales; Mycobacteriaceae; Mycobacterium; Mycobacterium tuberculosis complex; Mycobacterium tuberculosis;	NCBI: 1773	4395082	99.94	0.00	Yes
NCBI Assembly	GCA_002104795.1	NCBI: cellular organisms; Bacteria; Terrabacteria group; Actinobacteria; Actinomycetia; Corynebacteriales; Mycobacteriaceae; Mycolicibacillus; Mycolicibacillus koreensis;	NCBI: 1069220	4078902	100.00	0.55	Yes

NCBI Assembly	GCA_002801255.1	NCBI: cellular organisms; Bacteria; Terrabacteria group; Actinobacteria; Actinomycetia; Corynebacteriales; Mycobacteriaceae; Mycobacteroides; Mycobacteroides abscessus; Mycobacteroides abscessus subsp. abscessus;	NCBI: 1185650	4930183	100.00	0.00	Yes
NCBI Assembly	GCA_002129525.1	NCBI: cellular organisms; Bacteria; Terrabacteria group; Actinobacteria; Actinomycetia; Corynebacteriales; Mycobacteriaceae; Mycobacterium; Mycobacterium tuberculosis complex; Mycobacterium tuberculosis;	NCBI: 1773	4395965	99.94	0.00	Yes
NCBI Assembly	GCA_002447235.1	NCBI: cellular organisms; Bacteria; Terrabacteria group; Actinobacteria; Actinomycetia; Corynebacteriales; Mycobacteriaceae; Mycobacterium; Mycobacterium tuberculosis complex; Mycobacterium tuberculosis;	NCBI: 1773	4411398	99.94	0.00	Yes
NCBI Assembly	GCA_002703785.1	NCBI: cellular organisms; Bacteria; Terrabacteria group; Actinobacteria; Actinomycetia; Corynebacteriales; Mycobacteriaceae; Mycobacterium; Mycobacterium tuberculosis complex; Mycobacterium tuberculosis; Mycobacterium tuberculosis variant bovis;	NCBI: 1765	4330396	99.11	1.22	Yes
NCBI Assembly	GCA_002089975.1	NCBI: cellular organisms; Bacteria; Terrabacteria group; Actinobacteria; Actinomycetia; Corynebacteriales; Mycobacteriaceae; Mycobacterium; Mycobacterium avium complex (MAC); Mycobacterium avium; Mycobacterium avium subsp. hominissuis;	NCBI: 439334	5659277	99.25	0.38	Yes
NCBI Assembly	GCA_002448195.1	NCBI: cellular organisms; Bacteria; Terrabacteria group; Actinobacteria; Actinomycetia; Corynebacteriales; Mycobacteriaceae; Mycobacterium; Mycobacterium tuberculosis complex; Mycobacterium tuberculosis;	NCBI: 1773	4411173	99.94	0.00	Yes
NCBI Assembly	GCA_002201735.1	NCBI: cellular organisms; Bacteria; Terrabacteria group; Actinobacteria; Actinomycetia; Corynebacteriales; Mycobacteriaceae; Mycobacterium; Mycobacterium avium complex (MAC); Mycobacterium avium; Mycobacterium avium subsp. hominissuis;	NCBI: 439334	5448889	99.94	0.05	Yes
NCBI Assembly	GCA_002089335.1	NCBI: cellular organisms; Bacteria; Terrabacteria group; Actinobacteria; Actinomycetia; Corynebacteriales; Mycobacteriaceae; Mycobacterium; Mycobacterium avium complex (MAC); Mycobacterium avium; Mycobacterium avium subsp. hominissuis;	NCBI: 439334	5217578	99.78	0.83	Yes
NCBI Assembly	GCA_002130155.1	NCBI: cellular organisms; Bacteria; Terrabacteria group; Actinobacteria; Actinomycetia; Corynebacteriales; Mycobacteriaceae; Mycobacterium; Mycobacterium tuberculosis complex; Mycobacterium tuberculosis;	NCBI: 1773	4392600	99.94	0.00	Yes
NCBI Assembly	GCA_002723835.1	NCBI: cellular organisms; Bacteria; Terrabacteria group; Actinobacteria; Actinomycetia; Corynebacteriales; Mycobacteriaceae; Mycobacterium; unclassified Mycobacterium;	NCBI: 2039281	6533596	99.43	0.19	Yes
NCBI Assembly	GCA_001943135.1	NCBI: cellular organisms; Bacteria; Terrabacteria group; Actinobacteria; Actinomycetia; Corynebacteriales; Mycobacteriaceae; Mycobacterium; Mycobacterium tuberculosis complex; Mycobacterium tuberculosis;	NCBI: 1773	4394963	99.94	0.00	Yes
NCBI Assembly	GCA_002105755.1	NCBI: cellular organisms; Bacteria; Terrabacteria group; Actinobacteria; Actinomycetia; Corynebacteriales; Mycobacteriaceae; Mycobacterium; Mycobacterium avium complex (MAC); Mycobacterium colombiense;	NCBI: 1041522	5581643	98.77	0.00	Yes
NCBI Assembly	GCA_002129545.1	NCBI: cellular organisms; Bacteria; Terrabacteria group; Actinobacteria; Actinomycetia; Corynebacteriales; Mycobacteriaceae; Mycobacterium; Mycobacterium tuberculosis complex; Mycobacterium tuberculosis;	NCBI: 1773	4399910	99.94	0.00	Yes
NCBI Assembly	GCA_002801265.1	NCBI: cellular organisms; Bacteria; Terrabacteria group; Actinobacteria; Actinomycetia; Corynebacteriales; Mycobacteriaceae; Mycobacteroides; Mycobacteroides abscessus; Mycobacteroides abscessus subsp. abscessus;	NCBI: 1185650	4888874	100.00	0.00	Yes
NCBI Assembly	GCA_002447255.1	NCBI: cellular organisms; Bacteria; Terrabacteria group; Actinobacteria; Actinomycetia; Corynebacteriales; Mycobacteriaceae; Mycobacterium; Mycobacterium tuberculosis complex; Mycobacterium tuberculosis;	NCBI: 1773	4411342	99.94	0.00	Yes
NCBI Assembly	GCA_002703795.1	NCBI: cellular organisms; Bacteria; Terrabacteria group; Actinobacteria; Actinomycetia; Corynebacteriales; Mycobacteriaceae; Mycobacterium; Mycobacterium tuberculosis complex; Mycobacterium tuberculosis; Mycobacterium tuberculosis variant bovis;	NCBI: 1765	4211635	96.11	0.44	Yes
NCBI Assembly	GCA_002089995.1	NCBI: cellular organisms; Bacteria; Terrabacteria group; Actinobacteria; Actinomycetia; Corynebacteriales; Mycobacteriaceae; Mycobacterium; Mycobacterium avium complex (MAC); Mycobacterium avium; Mycobacterium avium subsp. hominissuis;	NCBI: 439334	5232109	99.86	0.00	Yes
NCBI Assembly	GCA_002013715.1	NCBI: cellular organisms; Bacteria; Terrabacteria group; Actinobacteria; Actinomycetia; Corynebacteriales; Mycobacteriaceae; Mycobacteroides; Mycobacteroides franklinii;	NCBI: 948102	5110168	99.56	1.72	Yes

NCBI Assembly	GCA_002208235.1	NCBI: cellular organisms; Bacteria; Terrabacteria group; Actinobacteria; Actinomycetia; Corynebacteriales; Mycobacteriaceae; Mycobacterium; Mycobacterium tuberculosis complex; Mycobacterium tuberculosis;	NCBI: 1773	4417716	99.94	0.00	Yes
NCBI Assembly	GCA_002013385.1	NCBI: cellular organisms; Bacteria; Terrabacteria group; Actinobacteria; Actinomycetia; Corynebacteriales; Mycobacteriaceae; Mycobacterium; unclassified Mycobacterium;	NCBI: 1855656	5212470	99.50	0.81	Yes
NCBI Assembly	GCA_002089355.1	NCBI: cellular organisms; Bacteria; Terrabacteria group; Actinobacteria; Actinomycetia; Corynebacteriales; Mycobacteriaceae; Mycobacterium; Mycobacterium avium complex (MAC); Mycobacterium avium; Mycobacterium avium subsp. hominissuis;	NCBI: 439334	5211422	99.42	0.52	Yes
NCBI Assembly	GCA_002130185.1	NCBI: cellular organisms; Bacteria; Terrabacteria group; Actinobacteria; Actinomycetia; Corynebacteriales; Mycobacteriaceae; Mycobacterium; Mycobacterium tuberculosis complex; Mycobacterium tuberculosis;	NCBI: 1773	4395292	99.94	0.00	Yes
NCBI Assembly	GCA_001943185.1	NCBI: cellular organisms; Bacteria; Terrabacteria group; Actinobacteria; Actinomycetia; Corynebacteriales; Mycobacteriaceae; Mycobacterium; Mycobacterium tuberculosis complex; Mycobacterium tuberculosis;	NCBI: 1773	4388266	99.94	0.00	Yes
NCBI Assembly	GCA_002129565.1	NCBI: cellular organisms; Bacteria; Terrabacteria group; Actinobacteria; Actinomycetia; Corynebacteriales; Mycobacteriaceae; Mycobacterium; Mycobacterium tuberculosis complex; Mycobacterium tuberculosis;	NCBI: 1773	4394793	99.94	0.00	Yes
NCBI Assembly	GCA_002800585.1	NCBI: cellular organisms; Bacteria; Terrabacteria group; Actinobacteria; Actinomycetia; Corynebacteriales; Mycobacteriaceae; Mycobacteroides; Mycobacteroides abscessus; Mycobacteroides abscessus subsp. abscessus;	NCBI: 1185650	4875652	99.67	0.00	Yes
NCBI Assembly	GCA_002801275.1	NCBI: cellular organisms; Bacteria; Terrabacteria group; Actinobacteria; Actinomycetia; Corynebacteriales; Mycobacteriaceae; Mycobacteroides; Mycobacteroides abscessus; Mycobacteroides abscessus subsp. abscessus;	NCBI: 1185650	4928089	100.00	0.00	Yes
NCBI Assembly	GCA_002292645.1	NCBI: cellular organisms; Bacteria; Terrabacteria group; Actinobacteria; Actinomycetia; Corynebacteriales; Mycobacteriaceae; Mycobacterium; Mycobacterium avium complex (MAC); Mycobacterium intracellulare; Mycobacterium intracellulare subsp. chimaera;	NCBI: 222805	5829603	99.94	0.23	Yes
NCBI Assembly	GCA_002013415.1	NCBI: cellular organisms; Bacteria; Terrabacteria group; Actinobacteria; Actinomycetia; Corynebacteriales; Mycobacteriaceae; Mycobacterium; unclassified Mycobacterium;	NCBI: 1855658	5165709	99.50	0.17	Yes
NCBI Assembly	GCA_002013725.1	NCBI: cellular organisms; Bacteria; Terrabacteria group; Actinobacteria; Actinomycetia; Corynebacteriales; Mycobacteriaceae; Mycobacteroides; Mycobacteroides chelonae;	NCBI: 1774	5087096	99.39	0.03	Yes
NCBI Assembly	GCA_002703825.1	NCBI: cellular organisms; Bacteria; Terrabacteria group; Actinobacteria; Actinomycetia; Corynebacteriales; Mycobacteriaceae; Mycobacterium; Mycobacterium tuberculosis complex; Mycobacterium tuberculosis; Mycobacterium tuberculosis variant bovis;	NCBI: 1765	4427272	99.78	1.00	Yes
NCBI Assembly	GCA_002208705.2	NCBI: cellular organisms; Bacteria; Terrabacteria group; Actinobacteria; Actinomycetia; Corynebacteriales; Mycobacteriaceae; Mycobacterium; Mycobacterium avium complex (MAC); Mycobacterium avium; Mycobacterium avium subsp. paratuberculosis;	NCBI: 1770	4832477	99.28	0.33	Yes
NCBI Assembly	GCA_002801485.1	NCBI: cellular organisms; Bacteria; Terrabacteria group; Actinobacteria; Actinomycetia; Corynebacteriales; Mycobacteriaceae; Mycobacteroides; Mycobacteroides abscessus; Mycobacteroides abscessus subsp. abscessus;	NCBI: 1185650	5093498	100.00	0.11	Yes
NCBI Assembly	GCA_002000185.1	NCBI: cellular organisms; Bacteria; Terrabacteria group; Actinobacteria; Actinomycetia; Corynebacteriales; Mycobacteriaceae; Mycobacterium; Mycobacterium tuberculosis complex; Mycobacterium tuberculosis;	NCBI: 1773	4310935	99.94	0.00	Yes
NCBI Assembly	GCA_002089375.1	NCBI: cellular organisms; Bacteria; Terrabacteria group; Actinobacteria; Actinomycetia; Corynebacteriales; Mycobacteriaceae; Mycobacterium; Mycobacterium avium complex (MAC); Mycobacterium avium; Mycobacterium avium subsp. hominissuis;	NCBI: 439334	5330540	99.94	0.08	Yes
NCBI Assembly	GCA_001943195.1	NCBI: cellular organisms; Bacteria; Terrabacteria group; Actinobacteria; Actinomycetia; Corynebacteriales; Mycobacteriaceae; Mycobacterium; Mycobacterium tuberculosis complex; Mycobacterium tuberculosis;	NCBI: 1773	4350617	99.94	0.00	Yes
NCBI Assembly	GCA_002043095.1	NCBI: cellular organisms; Bacteria; Terrabacteria group; Actinobacteria; Actinomycetia; Corynebacteriales; Mycobacteriaceae; Mycobacterium; unclassified Mycobacterium;	NCBI: 1961706	7072377	99.81	3.36	Yes

NCBI Assembly	GCA_002800605.1	NCBI: cellular organisms; Bacteria; Terrabacteria group; Actinobacteria; Actinomycetia; Corynebacteriales; Mycobacteriaceae; Mycobacteroides; Mycobacteroides abscessus; Mycobacteroides abscessus subsp. abscessus;	NCBI: 1185650	4965176	100.00	0.00	Yes
NCBI Assembly	GCA_002292655.1	NCBI: cellular organisms; Bacteria; Terrabacteria group; Actinobacteria; Actinomycetia; Corynebacteriales; Mycobacteriaceae; Mycobacterium; Mycobacterium avium complex (MAC); Mycobacterium avium;	NCBI: 1764	5357109	99.94	0.15	Yes
NCBI Assembly	GCA_002801345.1	NCBI: cellular organisms; Bacteria; Terrabacteria group; Actinobacteria; Actinomycetia; Corynebacteriales; Mycobacteriaceae; Mycobacteroides; Mycobacteroides abscessus; Mycobacteroides abscessus subsp. abscessus;	NCBI: 1185650	5252906	100.00	0.00	Yes
NCBI Assembly	GCA_002013425.1	NCBI: cellular organisms; Bacteria; Terrabacteria group; Actinobacteria; Actinomycetia; Corynebacteriales; Mycobacteriaceae; Mycobacterium; unclassified Mycobacterium;	NCBI: 1855661	5195636	99.50	0.81	Yes
NCBI Assembly	GCA_002013745.1	NCBI: cellular organisms; Bacteria; Terrabacteria group; Actinobacteria; Actinomycetia; Corynebacteriales; Mycobacteriaceae; Mycobacterium; unclassified Mycobacterium;	NCBI: 1855659	5017083	99.50	0.47	Yes
NCBI Assembly	GCA_002703835.1	NCBI: cellular organisms; Bacteria; Terrabacteria group; Actinobacteria; Actinomycetia; Corynebacteriales; Mycobacteriaceae; Mycobacterium; Mycobacterium tuberculosis complex; Mycobacterium tuberculosis; Mycobacterium tuberculosis variant bovis;	NCBI: 1765	4321108	99.78	0.33	Yes
NCBI Assembly	GCA_001942815.1	NCBI: cellular organisms; Bacteria; Terrabacteria group; Actinobacteria; Actinomycetia; Corynebacteriales; Mycobacteriaceae; Mycobacterium; Mycobacterium tuberculosis complex; Mycobacterium tuberculosis;	NCBI: 1773	4384051	99.94	0.00	Yes
NCBI Assembly	GCA_002211525.1	NCBI: cellular organisms; Bacteria; Terrabacteria group; Actinobacteria; Actinomycetia; Corynebacteriales; Mycobacteriaceae; Mycobacterium; Mycobacterium avium complex (MAC); Mycobacterium avium; Mycobacterium avium subsp. paratuberculosis;	NCBI: 1770	4829728	99.61	0.33	Yes
NCBI Assembly	GCA_002801495.1	NCBI: cellular organisms; Bacteria; Terrabacteria group; Actinobacteria; Actinomycetia; Corynebacteriales; Mycobacteriaceae; Mycobacteroides; Mycobacteroides abscessus; Mycobacteroides abscessus subsp. abscessus;	NCBI: 1185650	4971908	100.00	0.00	Yes
NCBI Assembly	GCA_002000215.1	NCBI: cellular organisms; Bacteria; Terrabacteria group; Actinobacteria; Actinomycetia; Corynebacteriales; Mycobacteriaceae; Mycobacterium; Mycobacterium tuberculosis complex; Mycobacterium tuberculosis;	NCBI: 1773	4297653	99.75	0.00	Yes
NCBI Assembly	GCA_001943205.1	NCBI: cellular organisms; Bacteria; Terrabacteria group; Actinobacteria; Actinomycetia; Corynebacteriales; Mycobacteriaceae; Mycobacterium; Mycobacterium tuberculosis complex; Mycobacterium tuberculosis;	NCBI: 1773	4387581	99.94	0.00	Yes
NCBI Assembly	GCA_002072635.1	NCBI: cellular organisms; Bacteria; Terrabacteria group; Actinobacteria; Actinomycetia; Corynebacteriales; Mycobacteriaceae; Mycobacteroides; Mycobacteroides abscessus; Mycobacteroides abscessus subsp. abscessus;	NCBI: 1185650	5118280	100.00	0.00	Yes
NCBI Assembly	GCA_002800625.1	NCBI: cellular organisms; Bacteria; Terrabacteria group; Actinobacteria; Actinomycetia; Corynebacteriales; Mycobacteriaceae; Mycobacteroides; Mycobacteroides abscessus; Mycobacteroides abscessus subsp. abscessus;	NCBI: 1185650	5226136	100.00	0.00	Yes
NCBI Assembly	GCA_002292705.1	NCBI: cellular organisms; Bacteria; Terrabacteria group; Actinobacteria; Actinomycetia; Corynebacteriales; Mycobacteriaceae; Mycobacterium; Mycobacterium avium complex (MAC); Mycobacterium avium;	NCBI: 1764	5335175	99.94	0.21	Yes
NCBI Assembly	GCA_002801355.1	NCBI: cellular organisms; Bacteria; Terrabacteria group; Actinobacteria; Actinomycetia; Corynebacteriales; Mycobacteriaceae; Mycobacteroides; Mycobacteroides abscessus; Mycobacteroides abscessus subsp. abscessus;	NCBI: 1185650	4956063	100.00	0.00	Yes
NCBI Assembly	GCA_002013455.1	NCBI: cellular organisms; Bacteria; Terrabacteria group; Actinobacteria; Actinomycetia; Corynebacteriales; Mycobacteriaceae; Mycobacteroides; Mycobacteroides chelonae;	NCBI: 1774	5152563	99.50	0.03	Yes
NCBI Assembly	GCA_002013775.1	NCBI: cellular organisms; Bacteria; Terrabacteria group; Actinobacteria; Actinomycetia; Corynebacteriales; Mycobacteriaceae; Mycobacterium; unclassified Mycobacterium;	NCBI: 1855663	4991856	99.31	0.18	Yes
NCBI Assembly	GCA_002000905.1	NCBI: cellular organisms; Bacteria; Terrabacteria group; Actinobacteria; Actinomycetia; Corynebacteriales; Mycobacteriaceae; Mycobacterium; Mycobacterium tuberculosis complex; Mycobacterium tuberculosis;	NCBI: 1334071	4411532	99.94	0.00	Yes

NCBI Assembly	GCA_001942865.1	NCBI: cellular organisms; Bacteria; Terrabacteria group; Actinobacteria; Actinomycetia; Corynebacteriales; Mycobacteriaceae; Mycobacterium; Mycobacterium tuberculosis complex; Mycobacterium tuberculosis;	NCBI: 1773	4404151	99.94	0.00	Yes
NCBI Assembly	GCA_002219245.1	NCBI: cellular organisms; Bacteria; Terrabacteria group; Actinobacteria; Actinomycetia; Corynebacteriales; Mycobacteriaceae; Mycobacterium; Mycobacterium avium complex (MAC); Mycobacterium intracellulare; Mycobacterium intracellulare subsp. chimaera;	NCBI: 222805	6533653	99.94	1.32	Yes
NCBI Assembly	GCA_002703865.1	NCBI: cellular organisms; Bacteria; Terrabacteria group; Actinobacteria; Actinomycetia; Corynebacteriales; Mycobacteriaceae; Mycobacterium; Mycobacterium tuberculosis complex; Mycobacterium tuberculosis; Mycobacterium tuberculosis variant bovis;	NCBI: 1765	3717838	87.33	0.33	No
NCBI Assembly	GCA_002801575.1	NCBI: cellular organisms; Bacteria; Terrabacteria group; Actinobacteria; Actinomycetia; Corynebacteriales; Mycobacteriaceae; Mycobacteroides; Mycobacteroides abscessus; Mycobacteroides abscessus subsp. abscessus;	NCBI: 1185650	5086684	100.00	0.00	Yes
NCBI Assembly	GCA_001943225.1	NCBI: cellular organisms; Bacteria; Terrabacteria group; Actinobacteria; Actinomycetia; Corynebacteriales; Mycobacteriaceae; Mycobacterium; Mycobacterium tuberculosis complex; Mycobacterium tuberculosis;	NCBI: 1773	4398678	99.94	0.00	Yes
NCBI Assembly	GCA_002072775.2	NCBI: cellular organisms; Bacteria; Terrabacteria group; Actinobacteria; Actinomycetia; Corynebacteriales; Mycobacteriaceae; Mycobacterium; Mycobacterium tuberculosis complex; Mycobacterium tuberculosis;	NCBI: 1773	4433542	99.94	0.42	Yes
NCBI Assembly	GCA_002292725.1	NCBI: cellular organisms; Bacteria; Terrabacteria group; Actinobacteria; Actinomycetia; Corynebacteriales; Mycobacteriaceae; Mycobacterium; Mycobacterium avium complex (MAC); Mycobacterium avium;	NCBI: 1764	5410350	99.94	0.15	Yes
NCBI Assembly	GCA_002013465.1	NCBI: cellular organisms; Bacteria; Terrabacteria group; Actinobacteria; Actinomycetia; Corynebacteriales; Mycobacteriaceae; Mycobacterium; unclassified Mycobacterium;	NCBI: 1855662	4807294	99.33	0.36	Yes
NCBI Assembly	GCA_002013785.1	NCBI: cellular organisms; Bacteria; Terrabacteria group; Actinobacteria; Actinomycetia; Corynebacteriales; Mycobacteriaceae; Mycobacterium; unclassified Mycobacterium;	NCBI: 1855660	4852331	99.17	0.33	Yes
NCBI Assembly	GCA_002800665.2	NCBI: cellular organisms; Bacteria; Terrabacteria group; Actinobacteria; Actinomycetia; Corynebacteriales; Mycobacteriaceae; Mycobacteroides; Mycobacteroides abscessus; Mycobacteroides abscessus subsp. abscessus;	NCBI: 1185650	5192777	100.00	0.00	Yes
NCBI Assembly	GCA_002000925.1	NCBI: cellular organisms; Bacteria; Terrabacteria group; Actinobacteria; Actinomycetia; Corynebacteriales; Mycobacteriaceae; Mycobacterium; Mycobacterium tuberculosis complex; Mycobacterium tuberculosis;	NCBI: 1334056	4411532	99.94	0.00	Yes
NCBI Assembly	GCA_001942875.1	NCBI: cellular organisms; Bacteria; Terrabacteria group; Actinobacteria; Actinomycetia; Corynebacteriales; Mycobacteriaceae; Mycobacterium; Mycobacterium tuberculosis complex; Mycobacterium tuberculosis;	NCBI: 1773	4390540	99.94	0.00	Yes
NCBI Assembly	GCA_002801365.1	NCBI: cellular organisms; Bacteria; Terrabacteria group; Actinobacteria; Actinomycetia; Corynebacteriales; Mycobacteriaceae; Mycobacteroides; Mycobacteroides abscessus; Mycobacteroides abscessus subsp. abscessus;	NCBI: 1185650	4640916	100.00	0.00	Yes
NCBI Assembly	GCA_002219265.1	NCBI: cellular organisms; Bacteria; Terrabacteria group; Actinobacteria; Actinomycetia; Corynebacteriales; Mycobacteriaceae; Mycobacterium; Mycobacterium avium complex (MAC); Mycobacterium intracellulare; Mycobacterium intracellulare subsp. chimaera;	NCBI: 222805	6379602	99.94	1.19	Yes
NCBI Assembly	GCA_001943265.1	NCBI: cellular organisms; Bacteria; Terrabacteria group; Actinobacteria; Actinomycetia; Corynebacteriales; Mycobacteriaceae; Mycobacterium; Mycobacterium tuberculosis complex; Mycobacterium tuberculosis;	NCBI: 1773	4383970	99.94	0.00	Yes
NCBI Assembly	GCA_002703875.1	NCBI: cellular organisms; Bacteria; Terrabacteria group; Actinobacteria; Actinomycetia; Corynebacteriales; Mycobacteriaceae; Mycobacterium; Mycobacterium tuberculosis complex; Mycobacterium tuberculosis; Mycobacterium tuberculosis variant bovis;	NCBI: 1765	4298793	99.78	0.00	Yes
NCBI Assembly	GCA_002085625.1	NCBI: cellular organisms; Bacteria; Terrabacteria group; Actinobacteria; Actinomycetia; Corynebacteriales; Mycobacteriaceae; Mycobacterium; Mycobacterium kansasii;	NCBI: 1768	6421275	99.61	0.38	Yes
NCBI Assembly	GCA_002801585.1	NCBI: cellular organisms; Bacteria; Terrabacteria group; Actinobacteria; Actinomycetia; Corynebacteriales; Mycobacteriaceae; Mycobacteroides; Mycobacteroides abscessus; Mycobacteroides abscessus subsp. abscessus;	NCBI: 1185650	4886723	99.67	0.03	Yes

NCBI Assembly	GCA_002000945.1	NCBI: cellular organisms; Bacteria; Terrabacteria group; Actinobacteria; Actinomycetia; Corynebacteriales; Mycobacteriaceae; Mycobacterium; Mycobacterium tuberculosis complex; Mycobacterium tuberculosis;	NCBI: 1334075	4411532	99.94	0.00	Yes
NCBI Assembly	GCA_002013485.1	NCBI: cellular organisms; Bacteria; Terrabacteria group; Actinobacteria; Actinomycetia; Corynebacteriales; Mycobacteriaceae; Mycobacteroides; Mycobacteroides chelonae;	NCBI: 1774	5124775	98.83	0.03	Yes
NCBI Assembly	GCA_002013815.1	NCBI: cellular organisms; Bacteria; Terrabacteria group; Actinobacteria; Actinomycetia; Corynebacteriales; Mycobacteriaceae; Mycobacteroides; Mycobacteroides chelonae;	NCBI: 1774	5454906	99.13	0.03	Yes
NCBI Assembly	GCA_002292735.1	NCBI: cellular organisms; Bacteria; Terrabacteria group; Actinobacteria; Actinomycetia; Corynebacteriales; Mycobacteriaceae; Mycobacterium; Mycobacterium avium complex (MAC); Mycobacterium avium;	NCBI: 1764	5347184	99.94	0.15	Yes
NCBI Assembly	GCA_001942885.1	NCBI: cellular organisms; Bacteria; Terrabacteria group; Actinobacteria; Actinomycetia; Corynebacteriales; Mycobacteriaceae; Mycobacterium; Mycobacterium tuberculosis complex; Mycobacterium tuberculosis;	NCBI: 1773	4371274	99.94	0.00	Yes
NCBI Assembly	GCA_002800685.1	NCBI: cellular organisms; Bacteria; Terrabacteria group; Actinobacteria; Actinomycetia; Corynebacteriales; Mycobacteriaceae; Mycobacteroides; Mycobacteroides abscessus; Mycobacteroides abscessus subsp. abscessus;	NCBI: 1185650	5012252	100.00	0.00	Yes
NCBI Assembly	GCA_002219285.1	NCBI: cellular organisms; Bacteria; Terrabacteria group; Actinobacteria; Actinomycetia; Corynebacteriales; Mycobacteriaceae; Mycobacterium; Mycobacterium avium complex (MAC); Mycobacterium intracellulare; Mycobacterium intracellulare subsp. chimaera;	NCBI: 222805	6133600	99.94	1.04	Yes
NCBI Assembly	GCA_001943275.1	NCBI: cellular organisms; Bacteria; Terrabacteria group; Actinobacteria; Actinomycetia; Corynebacteriales; Mycobacteriaceae; Mycobacterium; Mycobacterium tuberculosis complex; Mycobacterium tuberculosis;	NCBI: 1773	4392760	99.94	0.00	Yes
NCBI Assembly	GCA_002801435.1	NCBI: cellular organisms; Bacteria; Terrabacteria group; Actinobacteria; Actinomycetia; Corynebacteriales; Mycobacteriaceae; Mycobacteroides; Mycobacteroides abscessus; Mycobacteroides abscessus subsp. abscessus;	NCBI: 1185650	4754418	100.00	0.00	Yes
NCBI Assembly	GCA_002085645.1	NCBI: cellular organisms; Bacteria; Terrabacteria group; Actinobacteria; Actinomycetia; Corynebacteriales; Mycobacteriaceae; Mycobacterium; Mycobacterium kansasii;	NCBI: 1768	6440784	99.94	0.71	Yes
NCBI Assembly	GCA_002703885.1	NCBI: cellular organisms; Bacteria; Terrabacteria group; Actinobacteria; Actinomycetia; Corynebacteriales; Mycobacteriaceae; Mycobacterium; Mycobacterium tuberculosis complex; Mycobacterium tuberculosis; Mycobacterium tuberculosis variant bovis;	NCBI: 1765	4317983	99.78	0.00	Yes
NCBI Assembly	GCA_002801605.1	NCBI: cellular organisms; Bacteria; Terrabacteria group; Actinobacteria; Actinomycetia; Corynebacteriales; Mycobacteriaceae; Mycobacteroides; Mycobacteroides abscessus; Mycobacteroides abscessus subsp. abscessus;	NCBI: 1185650	5050407	100.00	0.00	Yes
NCBI Assembly	GCA_002000965.1	NCBI: cellular organisms; Bacteria; Terrabacteria group; Actinobacteria; Actinomycetia; Corynebacteriales; Mycobacteriaceae; Mycobacterium; Mycobacterium tuberculosis complex; Mycobacterium tuberculosis;	NCBI: 1334068	4411532	99.94	0.00	Yes
NCBI Assembly	GCA_002013515.1	NCBI: cellular organisms; Bacteria; Terrabacteria group; Actinobacteria; Actinomycetia; Corynebacteriales; Mycobacteriaceae; Mycobacteroides; Mycobacteroides chelonae;	NCBI: 1774	4988518	99.47	0.03	Yes
NCBI Assembly	GCA_002013825.1	NCBI: cellular organisms; Bacteria; Terrabacteria group; Actinobacteria; Actinomycetia; Corynebacteriales; Mycobacteriaceae; Mycobacteroides; Mycobacteroides chelonae;	NCBI: 1774	5122533	99.42	0.03	Yes
NCBI Assembly	GCA_002292745.1	NCBI: cellular organisms; Bacteria; Terrabacteria group; Actinobacteria; Actinomycetia; Corynebacteriales; Mycobacteriaceae; Mycobacterium; Mycobacterium avium complex (MAC); Mycobacterium avium;	NCBI: 1764	5614569	99.94	0.67	Yes
NCBI Assembly	GCA_001942905.1	NCBI: cellular organisms; Bacteria; Terrabacteria group; Actinobacteria; Actinomycetia; Corynebacteriales; Mycobacteriaceae; Mycobacterium; Mycobacterium tuberculosis complex; Mycobacterium tuberculosis;	NCBI: 1773	4376419	99.94	0.00	Yes
NCBI Assembly	GCA_001943285.1	NCBI: cellular organisms; Bacteria; Terrabacteria group; Actinobacteria; Actinomycetia; Corynebacteriales; Mycobacteriaceae; Mycobacterium; Mycobacterium tuberculosis complex; Mycobacterium tuberculosis;	NCBI: 1773	4391945	99.94	0.00	Yes

NCBI Assembly	GCA_002800705.1	NCBI: cellular organisms; Bacteria; Terrabacteria group; Actinobacteria; Actinomycetia; Corynebacteriales; Mycobacteriaceae; Mycobacteroides; Mycobacteroides abscessus; Mycobacteroides abscessus subsp. abscessus;	NCBI: 1185650	5259515	100.00	0.00	Yes
NCBI Assembly	GCA_002085775.1	NCBI: cellular organisms; Bacteria; Terrabacteria group; Actinobacteria; Actinomycetia; Corynebacteriales; Mycobacteriaceae; Mycobacterium; Mycobacterium kansasii;	NCBI: 1768	6462452	99.28	0.61	Yes
NCBI Assembly	GCA_002801465.1	NCBI: cellular organisms; Bacteria; Terrabacteria group; Actinobacteria; Actinomycetia; Corynebacteriales; Mycobacteriaceae; Mycobacteroides; Mycobacteroides abscessus; Mycobacteroides abscessus subsp. abscessus;	NCBI: 1185650	5237316	94.83	35.74	No
NCBI Assembly	GCA_002703895.1	NCBI: cellular organisms; Bacteria; Terrabacteria group; Actinobacteria; Actinomycetia; Corynebacteriales; Mycobacteriaceae; Mycobacterium; Mycobacterium tuberculosis complex; Mycobacterium tuberculosis; Mycobacterium tuberculosis variant bovis;	NCBI: 1765	4310131	95.11	1.94	Yes
NCBI Assembly	GCA_002801645.1	NCBI: cellular organisms; Bacteria; Terrabacteria group; Actinobacteria; Actinomycetia; Corynebacteriales; Mycobacteriaceae; Mycobacteroides; Mycobacteroides abscessus; Mycobacteroides abscessus subsp. abscessus;	NCBI: 1185650	5358242	100.00	0.06	Yes
NCBI Assembly	GCA_002013535.1	NCBI: cellular organisms; Bacteria; Terrabacteria group; Actinobacteria; Actinomycetia; Corynebacteriales; Mycobacteriaceae; Mycobacteroides; Mycobacteroides chelonae;	NCBI: 1774	5181067	99.50	0.20	Yes
NCBI Assembly	GCA_002003585.1	NCBI: cellular organisms; Bacteria; Terrabacteria group; Actinobacteria; Actinomycetia; Corynebacteriales; Mycobacteriaceae; Mycobacterium; Mycobacterium kansasii;	NCBI: 1768	6801699	90.01	1.71	Yes
NCBI Assembly	GCA_002013855.1	NCBI: cellular organisms; Bacteria; Terrabacteria group; Actinobacteria; Actinomycetia; Corynebacteriales; Mycobacteriaceae; Mycobacteroides; Mycobacteroides chelonae;	NCBI: 1774	5211550	99.45	0.03	Yes
NCBI Assembly	GCA_002292785.1	NCBI: cellular organisms; Bacteria; Terrabacteria group; Actinobacteria; Actinomycetia; Corynebacteriales; Mycobacteriaceae; Mycobacterium; Mycobacterium avium complex (MAC); Mycobacterium avium;	NCBI: 1764	5213473	99.94	0.00	Yes
NCBI Assembly	GCA_001942945.1	NCBI: cellular organisms; Bacteria; Terrabacteria group; Actinobacteria; Actinomycetia; Corynebacteriales; Mycobacteriaceae; Mycobacterium; Mycobacterium tuberculosis complex; Mycobacterium tuberculosis;	NCBI: 1773	4381829	99.94	0.00	Yes
NCBI Assembly	GCA_001943295.1	NCBI: cellular organisms; Bacteria; Terrabacteria group; Actinobacteria; Actinomycetia; Corynebacteriales; Mycobacteriaceae; Mycobacterium; Mycobacterium tuberculosis complex; Mycobacterium tuberculosis;	NCBI: 1773	4399304	99.94	0.00	Yes
NCBI Assembly	GCA_002085795.1	NCBI: cellular organisms; Bacteria; Terrabacteria group; Actinobacteria; Actinomycetia; Corynebacteriales; Mycobacteriaceae; Mycobacterium; Mycobacterium kansasii;	NCBI: 1768	6421364	99.86	0.38	Yes
NCBI Assembly	GCA_002131445.1	NCBI: cellular organisms; Bacteria; Terrabacteria group; Actinobacteria; Actinomycetia; Corynebacteriales; Mycobacteriaceae; Mycobacterium; Mycobacterium tuberculosis complex; Mycobacterium tuberculosis;	NCBI: 1773	4378188	99.94	0.00	Yes
NCBI Assembly	GCA_002800775.1	NCBI: cellular organisms; Bacteria; Terrabacteria group; Actinobacteria; Actinomycetia; Corynebacteriales; Mycobacteriaceae; Mycobacteroides; Mycobacteroides abscessus; Mycobacteroides abscessus subsp. abscessus;	NCBI: 1185650	5205518	100.00	0.00	Yes
NCBI Assembly	GCA_002703945.1	NCBI: cellular organisms; Bacteria; Terrabacteria group; Actinobacteria; Actinomycetia; Corynebacteriales; Mycobacteriaceae; Mycobacterium; Mycobacterium tuberculosis complex; Mycobacterium tuberculosis; Mycobacterium tuberculosis variant bovis;	NCBI: 1765	4310081	99.78	0.54	Yes
NCBI Assembly	GCA_002801665.1	NCBI: cellular organisms; Bacteria; Terrabacteria group; Actinobacteria; Actinomycetia; Corynebacteriales; Mycobacteriaceae; Mycobacteroides; Mycobacteroides abscessus; Mycobacteroides abscessus subsp. abscessus;	NCBI: 1185650	4703802	100.00	0.00	Yes
NCBI Assembly	GCA_002013545.1	NCBI: cellular organisms; Bacteria; Terrabacteria group; Actinobacteria; Actinomycetia; Corynebacteriales; Mycobacteriaceae; Mycobacterium; unclassified Mycobacterium;	NCBI: 1855664	4931340	99.50	0.47	Yes
NCBI Assembly	GCA_002003625.1	NCBI: cellular organisms; Bacteria; Terrabacteria group; Actinobacteria; Actinomycetia; Corynebacteriales; Mycobacteriaceae; Mycobacterium; Mycobacterium kansasii;	NCBI: 1768	6629039	90.13	2.04	Yes

NCBI Assembly	GCA_002013895.1	NCBI: cellular organisms; Bacteria; Terrabacteria group; Actinobacteria; Actinomycetia; Corynebacteriales; Mycobacteriaceae; Mycobacteroides; Mycobacteroides franklinii;	NCBI: 948102	5431726	99.67	0.20	Yes
NCBI Assembly	GCA_001943345.1	NCBI: cellular organisms; Bacteria; Terrabacteria group; Actinobacteria; Actinomycetia; Corynebacteriales; Mycobacteriaceae; Mycobacterium; Mycobacterium tuberculosis complex; Mycobacterium tuberculosis;	NCBI: 1773	4391588	99.94	0.00	Yes
NCBI Assembly	GCA_002300575.1	NCBI: cellular organisms; Bacteria; Terrabacteria group; Actinobacteria; Actinomycetia; Corynebacteriales; Mycobacteriaceae; Mycobacterium; Mycobacterium avium complex (MAC); Mycobacterium avium; Mycobacterium avium subsp. hominissuis;	NCBI: 439334	5266633	99.72	0.18	Yes
NCBI Assembly	GCA_001942955.1	NCBI: cellular organisms; Bacteria; Terrabacteria group; Actinobacteria; Actinomycetia; Corynebacteriales; Mycobacteriaceae; Mycobacterium; Mycobacterium tuberculosis complex; Mycobacterium tuberculosis;	NCBI: 1773	4392716	99.94	0.00	Yes
NCBI Assembly	GCA_002085815.1	NCBI: cellular organisms; Bacteria; Terrabacteria group; Actinobacteria; Actinomycetia; Corynebacteriales; Mycobacteriaceae; Mycobacterium; Mycobacterium kansasii;	NCBI: 1768	6434062	99.94	0.38	Yes
NCBI Assembly	GCA_002131455.1	NCBI: cellular organisms; Bacteria; Terrabacteria group; Actinobacteria; Actinomycetia; Corynebacteriales; Mycobacteriaceae; Mycobacterium; Mycobacterium tuberculosis complex; Mycobacterium tuberculosis;	NCBI: 1773	4365645	99.94	0.00	Yes
NCBI Assembly	GCA_002703955.1	NCBI: cellular organisms; Bacteria; Terrabacteria group; Actinobacteria; Actinomycetia; Corynebacteriales; Mycobacteriaceae; Mycobacterium; Mycobacterium tuberculosis complex; Mycobacterium tuberculosis; Mycobacterium tuberculosis variant bovis;	NCBI: 1765	4304820	99.78	0.00	Yes
NCBI Assembly	GCA_002800805.1	NCBI: cellular organisms; Bacteria; Terrabacteria group; Actinobacteria; Actinomycetia; Corynebacteriales; Mycobacteriaceae; Mycobacteroides; Mycobacteroides abscessus; Mycobacteroides abscessus subsp. abscessus;	NCBI: 1185650	4855277	100.00	0.00	Yes
NCBI Assembly	GCA_002013555.1	NCBI: cellular organisms; Bacteria; Terrabacteria group; Actinobacteria; Actinomycetia; Corynebacteriales; Mycobacteriaceae; Mycobacteroides; Mycobacteroides immunogenum;	NCBI: 83262	5542766	99.61	2.15	Yes
NCBI Assembly	GCA_002003715.1	NCBI: cellular organisms; Bacteria; Terrabacteria group; Actinobacteria; Actinomycetia; Corynebacteriales; Mycobacteriaceae; Mycobacterium; Mycobacterium tuberculosis complex; Mycobacterium tuberculosis;	NCBI: 1773	4322502	99.94	0.00	Yes
NCBI Assembly	GCA_002801725.1	NCBI: cellular organisms; Bacteria; Terrabacteria group; Actinobacteria; Actinomycetia; Corynebacteriales; Mycobacteriaceae; Mycobacteroides; Mycobacteroides abscessus; Mycobacteroides abscessus subsp. massiliense;	NCBI: 1962118	4786264	100.00	0.00	Yes
NCBI Assembly	GCA_002024965.1	NCBI: cellular organisms; Bacteria; Terrabacteria group; Actinobacteria; Actinomycetia; Corynebacteriales; Mycobacteriaceae; Mycobacterium; Mycobacterium simiae complex; Mycobacterium intermedium;	NCBI: 28445	6875104	99.38	1.63	Yes
NCBI Assembly	GCA_002304055.1	NCBI: cellular organisms; Bacteria; Terrabacteria group; Actinobacteria; Actinomycetia; Corynebacteriales; Mycobacteriaceae; Mycobacterium; Mycobacterium avium complex (MAC); Mycobacterium avium; Mycobacterium avium subsp. hominissuis;	NCBI: 439334	5058528	99.86	0.36	Yes
NCBI Assembly	GCA_001943355.1	NCBI: cellular organisms; Bacteria; Terrabacteria group; Actinobacteria; Actinomycetia; Corynebacteriales; Mycobacteriaceae; Mycobacterium; Mycobacterium tuberculosis complex; Mycobacterium tuberculosis;	NCBI: 1773	4406269	99.94	0.00	Yes
NCBI Assembly	GCA_001942965.1	NCBI: cellular organisms; Bacteria; Terrabacteria group; Actinobacteria; Actinomycetia; Corynebacteriales; Mycobacteriaceae; Mycobacterium; Mycobacterium tuberculosis complex; Mycobacterium tuberculosis;	NCBI: 1773	4348648	99.61	0.00	Yes
NCBI Assembly	GCA_002085835.1	NCBI: cellular organisms; Bacteria; Terrabacteria group; Actinobacteria; Actinomycetia; Corynebacteriales; Mycobacteriaceae; Mycobacterium; Mycobacterium kansasii;	NCBI: 1768	6463923	99.38	0.38	Yes
NCBI Assembly	GCA_002131485.1	NCBI: cellular organisms; Bacteria; Terrabacteria group; Actinobacteria; Actinomycetia; Corynebacteriales; Mycobacteriaceae; Mycobacterium; Mycobacterium tuberculosis complex; Mycobacterium tuberculosis;	NCBI: 1773	4384327	99.94	0.00	Yes
NCBI Assembly	GCA_002013595.1	NCBI: cellular organisms; Bacteria; Terrabacteria group; Actinobacteria; Actinomycetia; Corynebacteriales; Mycobacteriaceae; Mycobacteroides; Mycobacteroides chelonae;	NCBI: 1774	4996994	99.50	0.03	Yes

NCBI Assembly	GCA_002703985.1	NCBI: cellular organisms; Bacteria; Terrabacteria group; Actinobacteria; Actinomycetia; Corynebacteriales; Mycobacteriaceae; Mycobacterium; Mycobacterium tuberculosis complex; Mycobacterium tuberculosis; Mycobacterium tuberculosis variant bovis;	NCBI: 1765	4091885	93.61	1.00	Yes
NCBI Assembly	GCA_002800815.1	NCBI: cellular organisms; Bacteria; Terrabacteria group; Actinobacteria; Actinomycetia; Corynebacteriales; Mycobacteriaceae; Mycobacteroides; Mycobacteroides abscessus; Mycobacteroides abscessus subsp. abscessus;	NCBI: 1185650	4925761	100.00	0.00	Yes
NCBI Assembly	GCA_002024975.1	NCBI: cellular organisms; Bacteria; Terrabacteria group; Actinobacteria; Actinomycetia; Corynebacteriales; Mycobacteriaceae; Mycolicibacterium; Mycolicibacterium diernhoferi;	NCBI: 1801	5920839	98.00	1.69	Yes
NCBI Assembly	GCA_002003745.1	NCBI: cellular organisms; Bacteria; Terrabacteria group; Actinobacteria; Actinomycetia; Corynebacteriales; Mycobacteriaceae; Mycobacterium; Mycobacterium tuberculosis complex; Mycobacterium tuberculosis;	NCBI: 1773	4331900	99.86	0.00	Yes
NCBI Assembly	GCA_001943375.1	NCBI: cellular organisms; Bacteria; Terrabacteria group; Actinobacteria; Actinomycetia; Corynebacteriales; Mycobacteriaceae; Mycobacterium; Mycobacterium tuberculosis complex; Mycobacterium tuberculosis;	NCBI: 1773	4392093	99.94	0.00	Yes
NCBI Assembly	GCA_002801735.1	NCBI: cellular organisms; Bacteria; Terrabacteria group; Actinobacteria; Actinomycetia; Corynebacteriales; Mycobacteriaceae; Mycobacteroides; Mycobacteroides abscessus; Mycobacteroides abscessus subsp. massiliense;	NCBI: 1962118	4827942	100.00	0.00	Yes
NCBI Assembly	GCA_001942975.1	NCBI: cellular organisms; Bacteria; Terrabacteria group; Actinobacteria; Actinomycetia; Corynebacteriales; Mycobacteriaceae; Mycobacterium; Mycobacterium tuberculosis complex; Mycobacterium tuberculosis;	NCBI: 1773	4388128	99.61	0.00	Yes
NCBI Assembly	GCA_002086115.1	NCBI: cellular organisms; Bacteria; Terrabacteria group; Actinobacteria; Actinomycetia; Corynebacteriales; Mycobacteriaceae; Mycolicibacterium; Mycolicibacterium bacteremicum;	NCBI: 564198	5952041	99.76	1.01	Yes
NCBI Assembly	GCA_002131495.1	NCBI: cellular organisms; Bacteria; Terrabacteria group; Actinobacteria; Actinomycetia; Corynebacteriales; Mycobacteriaceae; Mycobacterium; Mycobacterium tuberculosis complex; Mycobacterium tuberculosis;	NCBI: 1773	4420055	99.94	0.00	Yes
NCBI Assembly	GCA_002240155.1	NCBI: cellular organisms; Bacteria; Terrabacteria group; Actinobacteria; Actinomycetia; Corynebacteriales; Mycobacteriaceae; Mycobacterium; Mycobacterium avium complex (MAC); Mycobacterium intracellulare; Mycobacterium intracellulare subsp. chimaera;	NCBI: 222805	5938632	99.94	0.53	Yes
NCBI Assembly	GCA_002013615.1	NCBI: cellular organisms; Bacteria; Terrabacteria group; Actinobacteria; Actinomycetia; Corynebacteriales; Mycobacteriaceae; Mycobacteroides; Mycobacteroides chelonae;	NCBI: 1774	2775019	58.53	0.00	No
NCBI Assembly	GCA_002026285.1	NCBI: cellular organisms; Bacteria; Terrabacteria group; Actinobacteria; Actinomycetia; Corynebacteriales; Mycobacteriaceae; Mycobacterium; Mycobacterium tuberculosis complex; Mycobacterium tuberculosis; Mycobacterium tuberculosis variant bovis;	NCBI: 1765	4295926	99.78	0.00	Yes
NCBI Assembly	GCA_002703995.1	NCBI: cellular organisms; Bacteria; Terrabacteria group; Actinobacteria; Actinomycetia; Corynebacteriales; Mycobacteriaceae; Mycobacterium; Mycobacterium tuberculosis complex; Mycobacterium tuberculosis; Mycobacterium tuberculosis variant bovis;	NCBI: 1765	4210032	98.78	0.33	Yes
NCBI Assembly	GCA_001943385.1	NCBI: cellular organisms; Bacteria; Terrabacteria group; Actinobacteria; Actinomycetia; Corynebacteriales; Mycobacteriaceae; Mycobacterium; Mycobacterium tuberculosis complex; Mycobacterium tuberculosis;	NCBI: 1773	4394542	99.94	0.00	Yes
NCBI Assembly	GCA_002800845.1	NCBI: cellular organisms; Bacteria; Terrabacteria group; Actinobacteria; Actinomycetia; Corynebacteriales; Mycobacteriaceae; Mycobacteroides; Mycobacteroides abscessus; Mycobacteroides abscessus subsp. abscessus;	NCBI: 1185650	4926198	100.00	0.00	Yes
NCBI Assembly	GCA_001943025.1	NCBI: cellular organisms; Bacteria; Terrabacteria group; Actinobacteria; Actinomycetia; Corynebacteriales; Mycobacteriaceae; Mycobacterium; Mycobacterium tuberculosis complex; Mycobacterium tuberculosis;	NCBI: 1773	4405158	99.94	0.00	Yes
NCBI Assembly	GCA_002007745.1	NCBI: cellular organisms; Bacteria; Terrabacteria group; Actinobacteria; Actinomycetia; Corynebacteriales; Mycobacteriaceae; Mycolicibacterium; Mycolicibacterium litorale;	NCBI: 758802	6103712	97.38	0.85	Yes
NCBI Assembly	GCA_002801765.1	NCBI: cellular organisms; Bacteria; Terrabacteria group; Actinobacteria; Actinomycetia; Corynebacteriales; Mycobacteriaceae; Mycobacteroides; Mycobacteroides abscessus; Mycobacteroides abscessus subsp. massiliense;	NCBI: 1962118	5039966	100.00	0.00	Yes

NCBI Assembly	GCA_002086125.1	NCBI: cellular organisms; Bacteria; Terrabacteria group; Actinobacteria; Actinomycetia; Corynebacteriales; Mycobacteriaceae; Mycobacterium; Mycobacterium avium complex (MAC); Mycobacterium arosiense;	NCBI: 1265311	5980206	99.94	1.46	Yes
NCBI Assembly	GCA_002131525.1	NCBI: cellular organisms; Bacteria; Terrabacteria group; Actinobacteria; Actinomycetia; Corynebacteriales; Mycobacteriaceae; Mycobacterium; Mycobacterium tuberculosis complex; Mycobacterium tuberculosis;	NCBI: 1773	4396771	99.94	0.00	Yes
NCBI Assembly	GCA_002245535.1	NCBI: cellular organisms; Bacteria; Terrabacteria group; Actinobacteria; Actinomycetia; Corynebacteriales; Mycobacteriaceae; Mycobacterium; Mycobacterium lehmannii;	NCBI: 2048550	5496052	100.00	0.61	Yes
NCBI Assembly	GCA_002013635.1	NCBI: cellular organisms; Bacteria; Terrabacteria group; Actinobacteria; Actinomycetia; Corynebacteriales; Mycobacteriaceae; Mycobacteroides; Mycobacteroides chelonae;	NCBI: 1774	5166650	98.72	0.03	Yes
NCBI Assembly	GCA_002026325.1	NCBI: cellular organisms; Bacteria; Terrabacteria group; Actinobacteria; Actinomycetia; Corynebacteriales; Mycobacteriaceae; Mycobacterium; Mycobacterium tuberculosis complex; Mycobacterium tuberculosis; Mycobacterium tuberculosis variant bovis;	NCBI: 1765	4308699	99.78	0.00	Yes
NCBI Assembly	GCA_002704025.1	NCBI: cellular organisms; Bacteria; Terrabacteria group; Actinobacteria; Actinomycetia; Corynebacteriales; Mycobacteriaceae; Mycobacterium; Mycobacterium tuberculosis complex; Mycobacterium tuberculosis; Mycobacterium tuberculosis variant bovis;	NCBI: 1765	4300842	99.78	0.00	Yes
NCBI Assembly	GCA_001943425.1	NCBI: cellular organisms; Bacteria; Terrabacteria group; Actinobacteria; Actinomycetia; Corynebacteriales; Mycobacteriaceae; Mycobacterium; Mycobacterium tuberculosis complex; Mycobacterium tuberculosis;	NCBI: 1773	4401353	99.94	0.00	Yes
NCBI Assembly	GCA_001943035.1	NCBI: cellular organisms; Bacteria; Terrabacteria group; Actinobacteria; Actinomycetia; Corynebacteriales; Mycobacteriaceae; Mycobacterium; Mycobacterium tuberculosis complex; Mycobacterium tuberculosis;	NCBI: 1773	4383808	99.94	0.00	Yes
NCBI Assembly	GCA_002800875.1	NCBI: cellular organisms; Bacteria; Terrabacteria group; Actinobacteria; Actinomycetia; Corynebacteriales; Mycobacteriaceae; Mycobacteroides; Mycobacteroides abscessus; Mycobacteroides abscessus subsp. abscessus;	NCBI: 1185650	5050413	100.00	0.33	Yes
NCBI Assembly	GCA_002013295.1	NCBI: cellular organisms; Bacteria; Terrabacteria group; Actinobacteria; Actinomycetia; Corynebacteriales; Mycobacteriaceae; Mycobacteroides; Mycobacteroides franklinii;	NCBI: 948102	5115012	99.61	1.55	Yes
NCBI Assembly	GCA_002086155.1	NCBI: cellular organisms; Bacteria; Terrabacteria group; Actinobacteria; Actinomycetia; Corynebacteriales; Mycobacteriaceae; Mycobacterium; Mycobacterium angelicum;	NCBI: 470074	6662911	99.94	1.68	Yes
NCBI Assembly	GCA_002801785.1	NCBI: cellular organisms; Bacteria; Terrabacteria group; Actinobacteria; Actinomycetia; Corynebacteriales; Mycobacteriaceae; Mycobacteroides; Mycobacteroides abscessus; Mycobacteroides abscessus subsp. massiliense;	NCBI: 1962118	4733699	100.00	0.00	Yes
NCBI Assembly	GCA_002131545.1	NCBI: cellular organisms; Bacteria; Terrabacteria group; Actinobacteria; Actinomycetia; Corynebacteriales; Mycobacteriaceae; Mycobacterium; Mycobacterium tuberculosis complex; Mycobacterium tuberculosis;	NCBI: 1773	4412316	99.94	0.00	Yes
NCBI Assembly	GCA_002245615.1	NCBI: cellular organisms; Bacteria; Terrabacteria group; Actinobacteria; Actinomycetia; Corynebacteriales; Mycobacteriaceae; Mycobacterium; Mycobacterium neumannii;	NCBI: 2048551	5376391	100.00	0.45	Yes
NCBI Assembly	GCA_002013645.1	NCBI: cellular organisms; Bacteria; Terrabacteria group; Actinobacteria; Actinomycetia; Corynebacteriales; Mycobacteriaceae; Mycobacteroides; Mycobacteroides salmoniphilum;	NCBI: 404941	4934017	99.44	0.03	Yes
NCBI Assembly	GCA_002026395.1	NCBI: cellular organisms; Bacteria; Terrabacteria group; Actinobacteria; Actinomycetia; Corynebacteriales; Mycobacteriaceae; Mycobacterium; Mycobacterium tuberculosis complex; Mycobacterium tuberculosis; Mycobacterium tuberculosis variant bovis;	NCBI: 1765	4294453	99.78	0.28	Yes
NCBI Assembly	GCA_002704035.1	NCBI: cellular organisms; Bacteria; Terrabacteria group; Actinobacteria; Actinomycetia; Corynebacteriales; Mycobacteriaceae; Mycobacterium; Mycobacterium tuberculosis complex; Mycobacterium tuberculosis; Mycobacterium tuberculosis variant bovis;	NCBI: 1765	4304820	99.78	0.00	Yes
NCBI Assembly	GCA_001943435.1	NCBI: cellular organisms; Bacteria; Terrabacteria group; Actinobacteria; Actinomycetia; Corynebacteriales; Mycobacteriaceae; Mycobacterium; Mycobacterium tuberculosis complex; Mycobacterium tuberculosis;	NCBI: 1773	4388153	99.94	0.00	Yes

NCBI Assembly	GCA_001943045.1	NCBI: cellular organisms; Bacteria; Terrabacteria group; Actinobacteria; Actinomycetia; Corynebacteriales; Mycobacteriaceae; Mycobacterium; Mycobacterium tuberculosis complex; Mycobacterium tuberculosis;	NCBI: 1773	4381895	99.28	0.00	Yes
NCBI Assembly	GCA_002086165.1	NCBI: cellular organisms; Bacteria; Terrabacteria group; Actinobacteria; Actinomycetia; Corynebacteriales; Mycobacteriaceae; Mycobacterium; Mycobacterium avium complex (MAC); Mycobacterium bouchedurhonense;	NCBI: 701041	5897311	99.94	0.75	Yes
NCBI Assembly	GCA_002800885.1	NCBI: cellular organisms; Bacteria; Terrabacteria group; Actinobacteria; Actinomycetia; Corynebacteriales; Mycobacteriaceae; Mycobacteroides; Mycobacteroides abscessus; Mycobacteroides abscessus subsp. abscessus;	NCBI: 1185650	4897060	100.00	0.00	Yes
NCBI Assembly	GCA_002013305.1	NCBI: cellular organisms; Bacteria; Terrabacteria group; Actinobacteria; Actinomycetia; Corynebacteriales; Mycobacteriaceae; Mycobacteroides; Mycobacteroides chelonae;	NCBI: 1774	4989234	99.50	0.03	Yes
NCBI Assembly	GCA_002801795.1	NCBI: cellular organisms; Bacteria; Terrabacteria group; Actinobacteria; Actinomycetia; Corynebacteriales; Mycobacteriaceae; Mycobacteroides; Mycobacteroides abscessus; Mycobacteroides abscessus subsp. massiliense;	NCBI: 1962118	5122576	99.93	1.07	Yes
NCBI Assembly	GCA_002131555.1	NCBI: cellular organisms; Bacteria; Terrabacteria group; Actinobacteria; Actinomycetia; Corynebacteriales; Mycobacteriaceae; Mycobacterium; Mycobacterium tuberculosis complex; Mycobacterium tuberculosis;	NCBI: 1773	4384711	99.94	0.00	Yes
NCBI Assembly	GCA_002249865.1	NCBI: cellular organisms; Bacteria; Terrabacteria group; Actinobacteria; Actinomycetia; Corynebacteriales; Mycobacteriaceae; Mycobacterium; Mycobacterium marinum;	NCBI: 1781	6254535	99.21	0.12	Yes
NCBI Assembly	GCA_002013675.1	NCBI: cellular organisms; Bacteria; Terrabacteria group; Actinobacteria; Actinomycetia; Corynebacteriales; Mycobacteriaceae; Mycobacteroides; Mycobacteroides chelonae;	NCBI: 1774	5132689	98.83	0.03	Yes
NCBI Assembly	GCA_002040725.1	NCBI: cellular organisms; Bacteria; Terrabacteria group; Actinobacteria; Actinomycetia; Corynebacteriales; Mycobacteriaceae; Mycobacterium; Mycobacterium tuberculosis complex; Mycobacterium tuberculosis;	NCBI: 1773	4355103	96.86	0.00	Yes
NCBI Assembly	GCA_001943445.1	NCBI: cellular organisms; Bacteria; Terrabacteria group; Actinobacteria; Actinomycetia; Corynebacteriales; Mycobacteriaceae; Mycobacterium; Mycobacterium tuberculosis complex; Mycobacterium tuberculosis;	NCBI: 1773	4387682	99.94	0.00	Yes
NCBI Assembly	GCA_001943055.1	NCBI: cellular organisms; Bacteria; Terrabacteria group; Actinobacteria; Actinomycetia; Corynebacteriales; Mycobacteriaceae; Mycobacterium; Mycobacterium tuberculosis complex; Mycobacterium tuberculosis;	NCBI: 1773	4393605	99.94	0.00	Yes
NCBI Assembly	GCA_002704055.1	NCBI: cellular organisms; Bacteria; Terrabacteria group; Actinobacteria; Actinomycetia; Corynebacteriales; Mycobacteriaceae; Mycobacterium; Mycobacterium tuberculosis complex; Mycobacterium tuberculosis; Mycobacterium tuberculosis variant bovis;	NCBI: 1765	4276322	98.28	0.33	Yes
NCBI Assembly	GCA_002086175.1	NCBI: cellular organisms; Bacteria; Terrabacteria group; Actinobacteria; Actinomycetia; Corynebacteriales; Mycobacteriaceae; Mycolicibacterium; Mycolicibacterium celeriflavum;	NCBI: 1249101	4949446	100.00	0.06	Yes
NCBI Assembly	GCA_002013315.1	NCBI: cellular organisms; Bacteria; Terrabacteria group; Actinobacteria; Actinomycetia; Corynebacteriales; Mycobacteriaceae; Mycobacteroides; Mycobacteroides chelonae;	NCBI: 1774	4939129	99.50	0.03	Yes
NCBI Assembly	GCA_002800925.1	NCBI: cellular organisms; Bacteria; Terrabacteria group; Actinobacteria; Actinomycetia; Corynebacteriales; Mycobacteriaceae; Mycobacteroides; Mycobacteroides abscessus; Mycobacteroides abscessus subsp. abscessus;	NCBI: 1185650	5097484	100.00	0.00	Yes
NCBI Assembly	GCA_002801825.1	NCBI: cellular organisms; Bacteria; Terrabacteria group; Actinobacteria; Actinomycetia; Corynebacteriales; Mycobacteriaceae; Mycobacteroides; Mycobacteroides abscessus; Mycobacteroides abscessus subsp. massiliense;	NCBI: 1962118	4843017	100.00	0.00	Yes
NCBI Assembly	GCA_002131585.1	NCBI: cellular organisms; Bacteria; Terrabacteria group; Actinobacteria; Actinomycetia; Corynebacteriales; Mycobacteriaceae; Mycobacterium; Mycobacterium tuberculosis complex; Mycobacterium tuberculosis;	NCBI: 1773	4391655	99.94	0.00	Yes
NCBI Assembly	GCA_002013685.1	NCBI: cellular organisms; Bacteria; Terrabacteria group; Actinobacteria; Actinomycetia; Corynebacteriales; Mycobacteriaceae; Mycobacteroides; Mycobacteroides salmoniphilum;	NCBI: 404941	4766268	99.00	0.36	Yes

NCBI Assembly	GCA_002131135.1	NCBI: cellular organisms; Bacteria; Terrabacteria group; Actinobacteria; Actinomycetia; Corynebacteriales; Mycobacteriaceae; Mycobacterium; Mycobacterium tuberculosis complex; Mycobacterium tuberculosis;	NCBI: 1773	4409813	99.94	0.00	Yes
NCBI Assembly	GCA_002250655.1	NCBI: cellular organisms; Bacteria; Terrabacteria group; Actinobacteria; Actinomycetia; Corynebacteriales; Mycobacteriaceae; Mycolicibacterium; Mycolicibacterium sphagni;	NCBI: 1786	6066978	100.00	0.56	Yes
NCBI Assembly	GCA_002304075.1	NCBI: cellular organisms; Bacteria; Terrabacteria group; Actinobacteria; Actinomycetia; Corynebacteriales; Mycobacteriaceae; Mycobacterium; Mycobacterium avium complex (MAC); Mycobacterium avium; Mycobacterium avium subsp. hominissuis;	NCBI: 439334	5248692	99.94	0.10	Yes
NCBI Assembly	GCA_001943485.1	NCBI: cellular organisms; Bacteria; Terrabacteria group; Actinobacteria; Actinomycetia; Corynebacteriales; Mycobacteriaceae; Mycobacterium; Mycobacterium tuberculosis complex; Mycobacterium tuberculosis;	NCBI: 1773	4381840	99.94	0.07	Yes
NCBI Assembly	GCA_001943105.1	NCBI: cellular organisms; Bacteria; Terrabacteria group; Actinobacteria; Actinomycetia; Corynebacteriales; Mycobacteriaceae; Mycobacterium; Mycobacterium tuberculosis complex; Mycobacterium tuberculosis;	NCBI: 1773	4351197	99.94	0.00	Yes
NCBI Assembly	GCA_002086215.1	NCBI: cellular organisms; Bacteria; Terrabacteria group; Actinobacteria; Actinomycetia; Corynebacteriales; Mycobacteriaceae; Mycobacterium; Mycobacterium simiae complex; Mycobacterium heidelbergense;	NCBI: 53376	4999105	99.25	0.48	Yes
NCBI Assembly	GCA_002013375.1	NCBI: cellular organisms; Bacteria; Terrabacteria group; Actinobacteria; Actinomycetia; Corynebacteriales; Mycobacteriaceae; Mycobacterium; unclassified Mycobacterium;	NCBI: 1855657	4903433	99.50	0.47	Yes
NCBI Assembly	GCA_002800965.1	NCBI: cellular organisms; Bacteria; Terrabacteria group; Actinobacteria; Actinomycetia; Corynebacteriales; Mycobacteriaceae; Mycobacteroides; Mycobacteroides abscessus; Mycobacteroides abscessus subsp. abscessus;	NCBI: 1185650	5000807	100.00	0.00	Yes
NCBI Assembly	GCA_002131595.1	NCBI: cellular organisms; Bacteria; Terrabacteria group; Actinobacteria; Actinomycetia; Corynebacteriales; Mycobacteriaceae; Mycobacterium; Mycobacterium tuberculosis complex; Mycobacterium tuberculosis;	NCBI: 1773	4387572	99.94	0.00	Yes
NCBI Assembly	GCA_002131155.1	NCBI: cellular organisms; Bacteria; Terrabacteria group; Actinobacteria; Actinomycetia; Corynebacteriales; Mycobacteriaceae; Mycobacterium; Mycobacterium tuberculosis complex; Mycobacterium tuberculosis;	NCBI: 1773	4390445	99.94	0.00	Yes
NCBI Assembly	GCA_002290145.1	NCBI: cellular organisms; Bacteria; Terrabacteria group; Actinobacteria; Actinomycetia; Corynebacteriales; Mycobacteriaceae; Mycobacterium; Mycobacterium tuberculosis complex; Mycobacterium tuberculosis;	NCBI: 1773	4411709	99.94	0.00	Yes
NCBI Assembly	GCA_002801845.1	NCBI: cellular organisms; Bacteria; Terrabacteria group; Actinobacteria; Actinomycetia; Corynebacteriales; Mycobacteriaceae; Mycobacteroides; Mycobacteroides abscessus; Mycobacteroides abscessus subsp. massiliense;	NCBI: 1962118	4964441	100.00	0.00	Yes
NCBI Assembly	GCA_002285675.1	NCBI: cellular organisms; Bacteria; Terrabacteria group; Actinobacteria; Actinomycetia; Corynebacteriales; Mycobacteriaceae; Mycobacterium; Mycobacterium avium complex (MAC); Mycobacterium intracellulare;	NCBI: 1767	5638643	99.94	0.09	Yes
NCBI Assembly	GCA_001943495.1	NCBI: cellular organisms; Bacteria; Terrabacteria group; Actinobacteria; Actinomycetia; Corynebacteriales; Mycobacteriaceae; Mycobacterium; Mycobacterium tuberculosis complex; Mycobacterium tuberculosis;	NCBI: 1773	4384957	99.94	0.00	Yes
NCBI Assembly	GCA_002304125.1	NCBI: cellular organisms; Bacteria; Terrabacteria group; Actinobacteria; Actinomycetia; Corynebacteriales; Mycobacteriaceae; Mycobacterium; Mycobacterium avium complex (MAC); Mycobacterium avium; Mycobacterium avium subsp. hominissuis;	NCBI: 439334	5121042	98.56	0.00	Yes
NCBI Assembly	GCA_001943115.1	NCBI: cellular organisms; Bacteria; Terrabacteria group; Actinobacteria; Actinomycetia; Corynebacteriales; Mycobacteriaceae; Mycobacterium; Mycobacterium tuberculosis complex; Mycobacterium tuberculosis;	NCBI: 1773	4366583	99.94	0.00	Yes
NCBI Assembly	GCA_001942555.1	NCBI: cellular organisms; Bacteria; Terrabacteria group; Actinobacteria; Actinomycetia; Corynebacteriales; Mycobacteriaceae; Mycobacteroides; Mycobacteroides chelonae;	NCBI: 1774	5099700	98.83	0.03	Yes
NCBI Assembly	GCA_002086255.1	NCBI: cellular organisms; Bacteria; Terrabacteria group; Actinobacteria; Actinomycetia; Corynebacteriales; Mycobacteriaceae; Mycolicibacterium; Mycolicibacterium insubricum;	NCBI: 444597	4553680	99.65	1.53	Yes

NCBI Assembly	GCA_002800235.1	NCBI: cellular organisms; Bacteria; Terrabacteria group; Actinobacteria; Actinomycetia; Corynebacteriales; Mycobacteriaceae; Mycobacteroides; Mycobacteroides abscessus; Mycobacteroides abscessus subsp. abscessus;	NCBI: 1185650	4992734	100.00	0.33	Yes
NCBI Assembly	GCA_002131625.1	NCBI: cellular organisms; Bacteria; Terrabacteria group; Actinobacteria; Actinomycetia; Corynebacteriales; Mycobacteriaceae; Mycobacterium; Mycobacterium tuberculosis complex; Mycobacterium tuberculosis;	NCBI: 1773	4389784	99.94	0.00	Yes
NCBI Assembly	GCA_002131185.1	NCBI: cellular organisms; Bacteria; Terrabacteria group; Actinobacteria; Actinomycetia; Corynebacteriales; Mycobacteriaceae; Mycobacterium; Mycobacterium tuberculosis complex; Mycobacterium tuberculosis;	NCBI: 1773	4388931	99.94	0.00	Yes
NCBI Assembly	GCA_002801005.1	NCBI: cellular organisms; Bacteria; Terrabacteria group; Actinobacteria; Actinomycetia; Corynebacteriales; Mycobacteriaceae; Mycobacteroides; Mycobacteroides abscessus; Mycobacteroides abscessus subsp. abscessus;	NCBI: 1185650	5311087	100.00	0.25	Yes
NCBI Assembly	GCA_002285695.1	NCBI: cellular organisms; Bacteria; Terrabacteria group; Actinobacteria; Actinomycetia; Corynebacteriales; Mycobacteriaceae; Mycobacterium; Mycobacterium avium complex (MAC); Mycobacterium intracellulare;	NCBI: 1767	5432063	99.94	0.05	Yes
NCBI Assembly	GCA_002290165.1	NCBI: cellular organisms; Bacteria; Terrabacteria group; Actinobacteria; Actinomycetia; Corynebacteriales; Mycobacteriaceae; Mycobacterium; Mycobacterium tuberculosis complex; Mycobacterium tuberculosis;	NCBI: 1773	4414325	99.94	0.00	Yes
NCBI Assembly	GCA_001943505.1	NCBI: cellular organisms; Bacteria; Terrabacteria group; Actinobacteria; Actinomycetia; Corynebacteriales; Mycobacteriaceae; Mycobacterium; Mycobacterium tuberculosis complex; Mycobacterium tuberculosis;	NCBI: 1773	4381381	99.94	0.07	Yes
NCBI Assembly	GCA_002801875.1	NCBI: cellular organisms; Bacteria; Terrabacteria group; Actinobacteria; Actinomycetia; Corynebacteriales; Mycobacteriaceae; Mycobacteroides; Mycobacteroides abscessus; Mycobacteroides abscessus subsp. massiliense;	NCBI: 1962118	4818167	100.00	0.00	Yes
NCBI Assembly	GCA_001943125.1	NCBI: cellular organisms; Bacteria; Terrabacteria group; Actinobacteria; Actinomycetia; Corynebacteriales; Mycobacteriaceae; Mycobacterium; Mycobacterium tuberculosis complex; Mycobacterium tuberculosis;	NCBI: 1773	4383674	99.94	0.00	Yes
NCBI Assembly	GCA_001942565.1	NCBI: cellular organisms; Bacteria; Terrabacteria group; Actinobacteria; Actinomycetia; Corynebacteriales; Mycobacteriaceae; Mycobacteroides; Mycobacteroides abscessus; Mycobacteroides abscessus subsp. abscessus;	NCBI: 1185650	5253175	100.00	0.00	Yes
NCBI Assembly	GCA_002304145.1	NCBI: cellular organisms; Bacteria; Terrabacteria group; Actinobacteria; Actinomycetia; Corynebacteriales; Mycobacteriaceae; Mycobacterium; Mycobacterium avium complex (MAC); Mycobacterium avium; Mycobacterium avium subsp. hominissuis;	NCBI: 439334	4990846	94.58	0.11	Yes
NCBI Assembly	GCA_002800255.1	NCBI: cellular organisms; Bacteria; Terrabacteria group; Actinobacteria; Actinomycetia; Corynebacteriales; Mycobacteriaceae; Mycobacteroides; Mycobacteroides abscessus; Mycobacteroides abscessus subsp. abscessus;	NCBI: 1185650	4960913	100.00	0.00	Yes
NCBI Assembly	GCA_002131195.1	NCBI: cellular organisms; Bacteria; Terrabacteria group; Actinobacteria; Actinomycetia; Corynebacteriales; Mycobacteriaceae; Mycobacterium; Mycobacterium tuberculosis complex; Mycobacterium tuberculosis;	NCBI: 1773	4361367	99.94	0.00	Yes
NCBI Assembly	GCA_002131645.1	NCBI: cellular organisms; Bacteria; Terrabacteria group; Actinobacteria; Actinomycetia; Corynebacteriales; Mycobacteriaceae; Mycobacterium; Mycobacterium tuberculosis complex; Mycobacterium tuberculosis;	NCBI: 1773	4384988	99.94	0.00	Yes
NCBI Assembly	GCA_002285715.1	NCBI: cellular organisms; Bacteria; Terrabacteria group; Actinobacteria; Actinomycetia; Corynebacteriales; Mycobacteriaceae; Mycobacterium; Mycobacterium avium complex (MAC); Mycobacterium marseillense;	NCBI: 701042	5285642	99.94	0.00	Yes
NCBI Assembly	GCA_002801015.1	NCBI: cellular organisms; Bacteria; Terrabacteria group; Actinobacteria; Actinomycetia; Corynebacteriales; Mycobacteriaceae; Mycobacteroides; Mycobacteroides abscessus; Mycobacteroides abscessus subsp. abscessus;	NCBI: 1185650	4758517	100.00	0.00	Yes
NCBI Assembly	GCA_001943515.1	NCBI: cellular organisms; Bacteria; Terrabacteria group; Actinobacteria; Actinomycetia; Corynebacteriales; Mycobacteriaceae; Mycobacterium; Mycobacterium tuberculosis complex; Mycobacterium tuberculosis;	NCBI: 1773	4373781	99.94	0.00	Yes
NCBI Assembly	GCA_001942575.1	NCBI: cellular organisms; Bacteria; Terrabacteria group; Actinobacteria; Actinomycetia; Corynebacteriales; Mycobacteriaceae; Mycobacteroides; Mycobacteroides chelonae;	NCBI: 1774	5114756	99.50	0.03	Yes

NCBI Assembly	GCA_002801945.1	NCBI: cellular organisms; Bacteria; Terrabacteria group; Actinobacteria; Actinomycetia; Corynebacteriales; Mycobacteriaceae; Mycobacteroides; Mycobacteroides abscessus; Mycobacteroides abscessus subsp. abscessus;	NCBI: 1185650	4905374	100.00	0.00	Yes
NCBI Assembly	GCA_002304155.1	NCBI: cellular organisms; Bacteria; Terrabacteria group; Actinobacteria; Actinomycetia; Corynebacteriales; Mycobacteriaceae; Mycobacterium; Mycobacterium avium complex (MAC); Mycobacterium avium; Mycobacterium avium subsp. hominissuis;	NCBI: 439334	5635307	98.44	0.10	Yes
NCBI Assembly	GCA_002131655.1	NCBI: cellular organisms; Bacteria; Terrabacteria group; Actinobacteria; Actinomycetia; Corynebacteriales; Mycobacteriaceae; Mycobacterium; Mycobacterium tuberculosis complex; Mycobacterium tuberculosis;	NCBI: 1773	4400505	99.94	0.00	Yes
NCBI Assembly	GCA_002800275.1	NCBI: cellular organisms; Bacteria; Terrabacteria group; Actinobacteria; Actinomycetia; Corynebacteriales; Mycobacteriaceae; Mycobacteroides; Mycobacteroides abscessus; Mycobacteroides abscessus subsp. abscessus;	NCBI: 1185650	4993537	100.00	0.33	Yes
NCBI Assembly	GCA_002287605.1	NCBI: cellular organisms; Bacteria; Terrabacteria group; Actinobacteria; Actinomycetia; Corynebacteriales; Mycobacteriaceae; Mycobacterium; Mycobacterium avium complex (MAC); Mycobacterium intracellulare; Mycobacterium intracellulare subsp. chimaera;	NCBI: 222805	5751541	99.94	0.23	Yes
NCBI Assembly	GCA_002131215.1	NCBI: cellular organisms; Bacteria; Terrabacteria group; Actinobacteria; Actinomycetia; Corynebacteriales; Mycobacteriaceae; Mycobacterium; Mycobacterium tuberculosis complex; Mycobacterium tuberculosis;	NCBI: 1773	4411774	99.94	0.00	Yes
NCBI Assembly	GCA_001943565.1	NCBI: cellular organisms; Bacteria; Terrabacteria group; Actinobacteria; Actinomycetia; Corynebacteriales; Mycobacteriaceae; Mycobacterium; Mycobacterium tuberculosis complex; Mycobacterium tuberculosis;	NCBI: 1773	4386795	99.94	0.00	Yes
NCBI Assembly	GCA_002292425.1	NCBI: cellular organisms; Bacteria; Terrabacteria group; Actinobacteria; Actinomycetia; Corynebacteriales; Mycobacteriaceae; Mycobacterium; Mycobacterium avium complex (MAC); Mycobacterium avium;	NCBI: 1764	5409790	99.94	0.95	Yes
NCBI Assembly	GCA_001942625.1	NCBI: cellular organisms; Bacteria; Terrabacteria group; Actinobacteria; Actinomycetia; Corynebacteriales; Mycobacteriaceae; Mycobacterium; Mycobacterium syngnathidarum;	NCBI: 1908205	6344489	99.81	0.00	Yes
NCBI Assembly	GCA_002304195.1	NCBI: cellular organisms; Bacteria; Terrabacteria group; Actinobacteria; Actinomycetia; Corynebacteriales; Mycobacteriaceae; Mycobacterium; Mycobacterium avium complex (MAC); Mycobacterium avium; Mycobacterium avium subsp. hominissuis;	NCBI: 439334	5811762	89.72	0.46	No
NCBI Assembly	GCA_002131685.1	NCBI: cellular organisms; Bacteria; Terrabacteria group; Actinobacteria; Actinomycetia; Corynebacteriales; Mycobacteriaceae; Mycobacterium; Mycobacterium tuberculosis complex; Mycobacterium tuberculosis;	NCBI: 1773	4410072	99.94	0.00	Yes
NCBI Assembly	GCA_002800305.1	NCBI: cellular organisms; Bacteria; Terrabacteria group; Actinobacteria; Actinomycetia; Corynebacteriales; Mycobacteriaceae; Mycobacteroides; Mycobacteroides abscessus; Mycobacteroides abscessus subsp. abscessus;	NCBI: 1185650	5001662	100.00	0.00	Yes
NCBI Assembly	GCA_001943575.1	NCBI: cellular organisms; Bacteria; Terrabacteria group; Actinobacteria; Actinomycetia; Corynebacteriales; Mycobacteriaceae; Mycobacterium; Mycobacterium tuberculosis complex; Mycobacterium tuberculosis;	NCBI: 1773	4381391	99.94	0.00	Yes
NCBI Assembly	GCA_002131245.1	NCBI: cellular organisms; Bacteria; Terrabacteria group; Actinobacteria; Actinomycetia; Corynebacteriales; Mycobacteriaceae; Mycobacterium; Mycobacterium tuberculosis complex; Mycobacterium tuberculosis;	NCBI: 1773	4395101	99.94	0.00	Yes
NCBI Assembly	GCA_002292435.1	NCBI: cellular organisms; Bacteria; Terrabacteria group; Actinobacteria; Actinomycetia; Corynebacteriales; Mycobacteriaceae; Mycobacterium; Mycobacterium avium complex (MAC); Mycobacterium avium;	NCBI: 1764	5605011	99.94	0.42	Yes
NCBI Assembly	GCA_001942635.1	NCBI: cellular organisms; Bacteria; Terrabacteria group; Actinobacteria; Actinomycetia; Corynebacteriales; Mycobacteriaceae; Mycobacterium; Mycobacterium tuberculosis complex; Mycobacterium tuberculosis;	NCBI: 1773	4399245	99.94	0.00	Yes
NCBI Assembly	GCA_002335685.1	NCBI: cellular organisms; Bacteria; Terrabacteria group; Actinobacteria; Actinomycetia; Corynebacteriales; Mycobacteriaceae; Mycobacterium; unclassified Mycobacterium;	NCBI: 2051552	6037017	100.00	0.38	Yes
NCBI Assembly	GCA_002131705.1	NCBI: cellular organisms; Bacteria; Terrabacteria group; Actinobacteria; Actinomycetia; Corynebacteriales; Mycobacteriaceae; Mycobacterium; Mycobacterium tuberculosis complex; Mycobacterium tuberculosis;	NCBI: 1773	4421068	99.94	0.00	Yes

NCBI Assembly	GCA_001943585.1	NCBI: cellular organisms; Bacteria; Terrabacteria group; Actinobacteria; Actinomycetia; Corynebacteriales; Mycobacteriaceae; Mycobacterium; Mycobacterium tuberculosis complex; Mycobacterium tuberculosis;	NCBI: 1773	4390580	99.94	0.00	Yes
NCBI Assembly	GCA_002131265.1	NCBI: cellular organisms; Bacteria; Terrabacteria group; Actinobacteria; Actinomycetia; Corynebacteriales; Mycobacteriaceae; Mycobacterium; Mycobacterium tuberculosis complex; Mycobacterium tuberculosis;	NCBI: 1773	4389750	99.94	0.00	Yes
NCBI Assembly	GCA_002292445.1	NCBI: cellular organisms; Bacteria; Terrabacteria group; Actinobacteria; Actinomycetia; Corynebacteriales; Mycobacteriaceae; Mycobacterium; Mycobacterium avium complex (MAC); Mycobacterium avium;	NCBI: 1764	5219426	99.94	0.05	Yes
NCBI Assembly	GCA_001942645.1	NCBI: cellular organisms; Bacteria; Terrabacteria group; Actinobacteria; Actinomycetia; Corynebacteriales; Mycobacteriaceae; Mycobacterium; Mycobacterium tuberculosis complex; Mycobacterium tuberculosis;	NCBI: 1773	4381743	99.94	0.00	Yes
NCBI Assembly	GCA_002800315.1	NCBI: cellular organisms; Bacteria; Terrabacteria group; Actinobacteria; Actinomycetia; Corynebacteriales; Mycobacteriaceae; Mycobacteroides; Mycobacteroides abscessus; Mycobacteroides abscessus subsp. abscessus;	NCBI: 1185650	5224078	100.00	0.17	Yes
NCBI Assembly	GCA_002131715.1	NCBI: cellular organisms; Bacteria; Terrabacteria group; Actinobacteria; Actinomycetia; Corynebacteriales; Mycobacteriaceae; Mycobacterium; Mycobacterium tuberculosis complex; Mycobacterium tuberculosis;	NCBI: 1773	4386367	99.94	0.00	Yes
NCBI Assembly	GCA_001943595.1	NCBI: cellular organisms; Bacteria; Terrabacteria group; Actinobacteria; Actinomycetia; Corynebacteriales; Mycobacteriaceae; Mycobacterium; Mycobacterium tuberculosis complex; Mycobacterium tuberculosis;	NCBI: 1773	4388712	99.94	0.00	Yes
NCBI Assembly	GCA_001942655.1	NCBI: cellular organisms; Bacteria; Terrabacteria group; Actinobacteria; Actinomycetia; Corynebacteriales; Mycobacteriaceae; Mycobacterium; Mycobacterium tuberculosis complex; Mycobacterium tuberculosis;	NCBI: 1773	4398130	99.94	0.00	Yes
NCBI Assembly	GCA_002131285.1	NCBI: cellular organisms; Bacteria; Terrabacteria group; Actinobacteria; Actinomycetia; Corynebacteriales; Mycobacteriaceae; Mycobacterium; Mycobacterium tuberculosis complex; Mycobacterium tuberculosis;	NCBI: 1773	4390293	99.94	0.00	Yes
NCBI Assembly	GCA_002800345.1	NCBI: cellular organisms; Bacteria; Terrabacteria group; Actinobacteria; Actinomycetia; Corynebacteriales; Mycobacteriaceae; Mycobacteroides; Mycobacteroides abscessus; Mycobacteroides abscessus subsp. abscessus;	NCBI: 1185650	5186076	100.00	0.08	Yes
NCBI Assembly	GCA_002292475.1	NCBI: cellular organisms; Bacteria; Terrabacteria group; Actinobacteria; Actinomycetia; Corynebacteriales; Mycobacteriaceae; Mycobacterium; Mycobacterium avium complex (MAC); Mycobacterium avium;	NCBI: 1764	5215748	99.94	0.00	Yes
NCBI Assembly	GCA_001943645.1	NCBI: cellular organisms; Bacteria; Terrabacteria group; Actinobacteria; Actinomycetia; Corynebacteriales; Mycobacteriaceae; Mycobacterium; Mycobacterium tuberculosis complex; Mycobacterium tuberculosis;	NCBI: 1773	4349376	99.94	0.00	Yes
NCBI Assembly	GCA_001942705.1	NCBI: cellular organisms; Bacteria; Terrabacteria group; Actinobacteria; Actinomycetia; Corynebacteriales; Mycobacteriaceae; Mycobacterium; Mycobacterium tuberculosis complex; Mycobacterium tuberculosis;	NCBI: 1773	4389129	99.94	0.00	Yes
NCBI Assembly	GCA_002131725.1	NCBI: cellular organisms; Bacteria; Terrabacteria group; Actinobacteria; Actinomycetia; Corynebacteriales; Mycobacteriaceae; Mycobacterium; Mycobacterium tuberculosis complex; Mycobacterium tuberculosis;	NCBI: 1773	4387577	99.94	0.00	Yes
NCBI Assembly	GCA_002131295.1	NCBI: cellular organisms; Bacteria; Terrabacteria group; Actinobacteria; Actinomycetia; Corynebacteriales; Mycobacteriaceae; Mycobacterium; Mycobacterium tuberculosis complex; Mycobacterium tuberculosis;	NCBI: 1773	4388174	99.94	0.00	Yes
NCBI Assembly	GCA_002292505.1	NCBI: cellular organisms; Bacteria; Terrabacteria group; Actinobacteria; Actinomycetia; Corynebacteriales; Mycobacteriaceae; Mycobacterium; Mycobacterium avium complex (MAC); Mycobacterium avium;	NCBI: 1764	5247172	99.94	0.38	Yes
NCBI Assembly	GCA_002800405.1	NCBI: cellular organisms; Bacteria; Terrabacteria group; Actinobacteria; Actinomycetia; Corynebacteriales; Mycobacteriaceae; Mycobacteroides; Mycobacteroides abscessus; Mycobacteroides abscessus subsp. abscessus;	NCBI: 1185650	5140399	100.00	0.00	Yes
NCBI Assembly	GCA_001943655.1	NCBI: cellular organisms; Bacteria; Terrabacteria group; Actinobacteria; Actinomycetia; Corynebacteriales; Mycobacteriaceae; Mycobacterium; Mycobacterium tuberculosis complex; Mycobacterium tuberculosis;	NCBI: 1773	4401542	99.94	0.00	Yes

NCBI Assembly	GCA_001942715.1	NCBI: cellular organisms; Bacteria; Terrabacteria group; Actinobacteria; Actinomycetia; Corynebacteriales; Mycobacteriaceae; Mycobacterium; Mycobacterium tuberculosis complex; Mycobacterium tuberculosis;	NCBI: 1773	4396181	99.94	0.00	Yes
NCBI Assembly	GCA_002131325.1	NCBI: cellular organisms; Bacteria; Terrabacteria group; Actinobacteria; Actinomycetia; Corynebacteriales; Mycobacteriaceae; Mycobacterium; Mycobacterium tuberculosis complex; Mycobacterium tuberculosis;	NCBI: 1773	4404937	99.94	0.00	Yes
NCBI Assembly	GCA_002292525.1	NCBI: cellular organisms; Bacteria; Terrabacteria group; Actinobacteria; Actinomycetia; Corynebacteriales; Mycobacteriaceae; Mycobacterium; Mycobacterium avium complex (MAC); Mycobacterium intracellulare;	NCBI: 1767	5655613	99.94	0.14	Yes
NCBI Assembly	GCA_002800415.1	NCBI: cellular organisms; Bacteria; Terrabacteria group; Actinobacteria; Actinomycetia; Corynebacteriales; Mycobacteriaceae; Mycobacteroides; Mycobacteroides abscessus; Mycobacteroides abscessus subsp. abscessus;	NCBI: 1185650	4927513	100.00	0.00	Yes
NCBI Assembly	GCA_001943665.1	NCBI: cellular organisms; Bacteria; Terrabacteria group; Actinobacteria; Actinomycetia; Corynebacteriales; Mycobacteriaceae; Mycobacterium; Mycobacterium tuberculosis complex; Mycobacterium tuberculosis;	NCBI: 1773	4386020	99.94	0.00	Yes
NCBI Assembly	GCA_001942725.1	NCBI: cellular organisms; Bacteria; Terrabacteria group; Actinobacteria; Actinomycetia; Corynebacteriales; Mycobacteriaceae; Mycobacterium; Mycobacterium tuberculosis complex; Mycobacterium tuberculosis;	NCBI: 1773	4379022	99.94	0.00	Yes
NCBI Assembly	GCA_002131335.1	NCBI: cellular organisms; Bacteria; Terrabacteria group; Actinobacteria; Actinomycetia; Corynebacteriales; Mycobacteriaceae; Mycobacterium; Mycobacterium tuberculosis complex; Mycobacterium tuberculosis;	NCBI: 1773	4356155	99.94	0.00	Yes
NCBI Assembly	GCA_002292535.1	NCBI: cellular organisms; Bacteria; Terrabacteria group; Actinobacteria; Actinomycetia; Corynebacteriales; Mycobacteriaceae; Mycobacterium; Mycobacterium avium complex (MAC); Mycobacterium intracellulare;	NCBI: 1767	5535378	99.94	0.23	Yes
NCBI Assembly	GCA_002800445.1	NCBI: cellular organisms; Bacteria; Terrabacteria group; Actinobacteria; Actinomycetia; Corynebacteriales; Mycobacteriaceae; Mycobacteroides; Mycobacteroides abscessus; Mycobacteroides abscessus subsp. abscessus;	NCBI: 1185650	5280104	100.00	0.00	Yes
NCBI Assembly	GCA_001943675.1	NCBI: cellular organisms; Bacteria; Terrabacteria group; Actinobacteria; Actinomycetia; Corynebacteriales; Mycobacteriaceae; Mycobacterium; Mycobacterium tuberculosis complex; Mycobacterium tuberculosis;	NCBI: 1773	4369909	99.94	0.00	Yes
NCBI Assembly	GCA_001942735.1	NCBI: cellular organisms; Bacteria; Terrabacteria group; Actinobacteria; Actinomycetia; Corynebacteriales; Mycobacteriaceae; Mycobacterium; Mycobacterium tuberculosis complex; Mycobacterium tuberculosis;	NCBI: 1773	4381378	99.94	0.00	Yes
NCBI Assembly	GCA_002292545.1	NCBI: cellular organisms; Bacteria; Terrabacteria group; Actinobacteria; Actinomycetia; Corynebacteriales; Mycobacteriaceae; Mycobacterium; Mycobacterium avium complex (MAC); Mycobacterium avium;	NCBI: 1764	5891371	99.94	0.15	Yes
NCBI Assembly	GCA_002131365.1	NCBI: cellular organisms; Bacteria; Terrabacteria group; Actinobacteria; Actinomycetia; Corynebacteriales; Mycobacteriaceae; Mycobacterium; Mycobacterium tuberculosis complex; Mycobacterium tuberculosis;	NCBI: 1773	4394604	99.94	0.00	Yes
NCBI Assembly	GCA_002800465.1	NCBI: cellular organisms; Bacteria; Terrabacteria group; Actinobacteria; Actinomycetia; Corynebacteriales; Mycobacteriaceae; Mycobacteroides; Mycobacteroides abscessus; Mycobacteroides abscessus subsp. abscessus;	NCBI: 1185650	5187555	100.00	0.00	Yes
NCBI Assembly	GCA_001943725.1	NCBI: cellular organisms; Bacteria; Terrabacteria group; Actinobacteria; Actinomycetia; Corynebacteriales; Mycobacteriaceae; Mycobacterium; Mycobacterium tuberculosis complex; Mycobacterium tuberculosis;	NCBI: 1773	4381950	99.94	0.00	Yes
NCBI Assembly	GCA_001942785.1	NCBI: cellular organisms; Bacteria; Terrabacteria group; Actinobacteria; Actinomycetia; Corynebacteriales; Mycobacteriaceae; Mycobacterium; Mycobacterium tuberculosis complex; Mycobacterium tuberculosis;	NCBI: 1773	4378211	99.94	0.00	Yes
NCBI Assembly	GCA_002131385.1	NCBI: cellular organisms; Bacteria; Terrabacteria group; Actinobacteria; Actinomycetia; Corynebacteriales; Mycobacteriaceae; Mycobacterium; Mycobacterium tuberculosis complex; Mycobacterium tuberculosis;	NCBI: 1773	4392433	99.94	0.00	Yes
NCBI Assembly	GCA_002292585.1	NCBI: cellular organisms; Bacteria; Terrabacteria group; Actinobacteria; Actinomycetia; Corynebacteriales; Mycobacteriaceae; Mycobacterium; Mycobacterium avium complex (MAC); Mycobacterium avium;	NCBI: 1764	5518049	99.94	0.15	Yes

NCBI Assembly	GCA_002800485.1	NCBI: cellular organisms; Bacteria; Terrabacteria group; Actinobacteria; Actinomycetia; Corynebacteriales; Mycobacteriaceae; Mycobacteroides; Mycobacteroides abscessus; Mycobacteroides abscessus subsp. abscessus;	NCBI: 1185650	4936600	100.00	0.33	Yes
NCBI Assembly	GCA_001943735.1	NCBI: cellular organisms; Bacteria; Terrabacteria group; Actinobacteria; Actinomycetia; Corynebacteriales; Mycobacteriaceae; Mycobacterium; Mycobacterium tuberculosis complex; Mycobacterium tuberculosis;	NCBI: 1773	4344512	99.94	0.00	Yes
NCBI Assembly	GCA_001942795.1	NCBI: cellular organisms; Bacteria; Terrabacteria group; Actinobacteria; Actinomycetia; Corynebacteriales; Mycobacteriaceae; Mycobacterium; Mycobacterium tuberculosis complex; Mycobacterium tuberculosis;	NCBI: 1773	4391087	99.94	0.00	Yes
NCBI Assembly	GCA_002131395.1	NCBI: cellular organisms; Bacteria; Terrabacteria group; Actinobacteria; Actinomycetia; Corynebacteriales; Mycobacteriaceae; Mycobacterium; Mycobacterium tuberculosis complex; Mycobacterium tuberculosis;	NCBI: 1773	4353086	99.94	0.00	Yes
NCBI Assembly	GCA_002292595.1	NCBI: cellular organisms; Bacteria; Terrabacteria group; Actinobacteria; Actinomycetia; Corynebacteriales; Mycobacteriaceae; Mycobacterium; Mycobacterium avium complex (MAC); Mycobacterium avium;	NCBI: 1764	5179834	99.94	0.07	Yes
NCBI Assembly	GCA_002800495.1	NCBI: cellular organisms; Bacteria; Terrabacteria group; Actinobacteria; Actinomycetia; Corynebacteriales; Mycobacteriaceae; Mycobacteroides; Mycobacteroides abscessus; Mycobacteroides abscessus subsp. abscessus;	NCBI: 1185650	5232465	100.00	0.00	Yes
NCBI Assembly	GCA_001943745.1	NCBI: cellular organisms; Bacteria; Terrabacteria group; Actinobacteria; Actinomycetia; Corynebacteriales; Mycobacteriaceae; Mycobacterium; Mycobacterium tuberculosis complex; Mycobacterium tuberculosis;	NCBI: 1773	4388959	99.94	0.00	Yes
NCBI Assembly	GCA_001942805.1	NCBI: cellular organisms; Bacteria; Terrabacteria group; Actinobacteria; Actinomycetia; Corynebacteriales; Mycobacteriaceae; Mycobacterium; Mycobacterium tuberculosis complex; Mycobacterium tuberculosis;	NCBI: 1773	4394380	99.94	0.00	Yes
NCBI Assembly	GCA_002131405.1	NCBI: cellular organisms; Bacteria; Terrabacteria group; Actinobacteria; Actinomycetia; Corynebacteriales; Mycobacteriaceae; Mycobacterium; Mycobacterium tuberculosis complex; Mycobacterium tuberculosis;	NCBI: 1773	4390167	99.94	0.00	Yes
NCBI Assembly	GCA_002292625.1	NCBI: cellular organisms; Bacteria; Terrabacteria group; Actinobacteria; Actinomycetia; Corynebacteriales; Mycobacteriaceae; Mycobacterium; Mycobacterium avium complex (MAC); Mycobacterium intracellulare; Mycobacterium intracellulare subsp. chimaera;	NCBI: 222805	5765580	99.94	0.07	Yes
NCBI Assembly	GCA_002800525.1	NCBI: cellular organisms; Bacteria; Terrabacteria group; Actinobacteria; Actinomycetia; Corynebacteriales; Mycobacteriaceae; Mycobacteroides; Mycobacteroides abscessus; Mycobacteroides abscessus subsp. abscessus;	NCBI: 1185650	5240952	100.00	0.00	Yes
NCBI Assembly	GCA_002292635.1	NCBI: cellular organisms; Bacteria; Terrabacteria group; Actinobacteria; Actinomycetia; Corynebacteriales; Mycobacteriaceae; Mycobacterium; Mycobacterium avium complex (MAC); Mycobacterium avium;	NCBI: 1764	5265231	99.94	0.15	Yes
NCBI Assembly	GCA_002800545.1	NCBI: cellular organisms; Bacteria; Terrabacteria group; Actinobacteria; Actinomycetia; Corynebacteriales; Mycobacteriaceae; Mycobacteroides; Mycobacteroides abscessus; Mycobacteroides abscessus subsp. abscessus;	NCBI: 1185650	5231696	99.67	0.00	Yes
NCBI Assembly	GCA_002800555.1	NCBI: cellular organisms; Bacteria; Terrabacteria group; Actinobacteria; Actinomycetia; Corynebacteriales; Mycobacteriaceae; Mycobacteroides; Mycobacteroides abscessus; Mycobacteroides abscessus subsp. abscessus;	NCBI: 1185650	4880871	100.00	0.33	Yes
NCBI Assembly	GCA_003326145.1	NCBI: cellular organisms; Bacteria; Terrabacteria group; Actinobacteria; Actinomycetia; Corynebacteriales; Mycobacteriaceae; Mycobacterium; Mycobacterium tuberculosis complex; Mycobacterium tuberculosis;	NCBI: 1773	4357245	99.94	0.00	Yes
NCBI Assembly	GCA_003396205.1	NCBI: cellular organisms; Bacteria; Terrabacteria group; Actinobacteria; Actinomycetia; Corynebacteriales; Mycobacteriaceae; Mycobacterium; Mycobacterium tuberculosis complex; Mycobacterium tuberculosis;	NCBI: 1773	4339181	99.86	0.00	Yes
NCBI Assembly	GCA_003398155.1	NCBI: cellular organisms; Bacteria; Terrabacteria group; Actinobacteria; Actinomycetia; Corynebacteriales; Mycobacteriaceae; Mycobacterium; Mycobacterium tuberculosis complex; Mycobacterium tuberculosis;	NCBI: 1773	4389551	99.86	0.67	Yes
NCBI Assembly	GCA_003394625.1	NCBI: cellular organisms; Bacteria; Terrabacteria group; Actinobacteria; Actinomycetia; Corynebacteriales; Mycobacteriaceae; Mycobacterium; Mycobacterium tuberculosis complex; Mycobacterium tuberculosis;	NCBI: 1773	4382881	99.86	0.00	Yes

NCBI Assembly	GCA_003394915.1	NCBI: cellular organisms; Bacteria; Terrabacteria group; Actinobacteria; Actinomycetia; Corynebacteriales; Mycobacteriaceae; Mycobacterium; Mycobacterium tuberculosis complex; Mycobacterium tuberculosis;	NCBI: 1773	4322532	99.86	0.00	Yes
NCBI Assembly	GCA_003396495.1	NCBI: cellular organisms; Bacteria; Terrabacteria group; Actinobacteria; Actinomycetia; Corynebacteriales; Mycobacteriaceae; Mycobacterium; Mycobacterium tuberculosis complex; Mycobacterium tuberculosis;	NCBI: 1773	4359867	99.86	0.00	Yes
NCBI Assembly	GCA_003393935.1	NCBI: cellular organisms; Bacteria; Terrabacteria group; Actinobacteria; Actinomycetia; Corynebacteriales; Mycobacteriaceae; Mycobacterium; Mycobacterium tuberculosis complex; Mycobacterium tuberculosis;	NCBI: 1773	4402973	99.86	0.33	Yes
NCBI Assembly	GCA_003395565.1	NCBI: cellular organisms; Bacteria; Terrabacteria group; Actinobacteria; Actinomycetia; Corynebacteriales; Mycobacteriaceae; Mycobacterium; Mycobacterium tuberculosis complex; Mycobacterium tuberculosis;	NCBI: 1773	4367109	99.86	0.00	Yes
NCBI Assembly	GCA_004106125.1	NCBI: cellular organisms; Bacteria; Terrabacteria group; Actinobacteria; Actinomycetia; Corynebacteriales; Mycobacteriaceae; Mycobacterium; Mycobacterium tuberculosis complex; Mycobacterium tuberculosis;	NCBI: 1773	4399212	99.94	0.00	Yes
NCBI Assembly	GCA_004109605.1	NCBI: cellular organisms; Bacteria; Terrabacteria group; Actinobacteria; Actinomycetia; Corynebacteriales; Mycobacteriaceae; Mycobacterium; Mycobacterium tuberculosis complex; Mycobacterium tuberculosis;	NCBI: 1773	4398070	99.61	0.00	Yes
NCBI Assembly	GCA_004025145.1	NCBI: cellular organisms; Bacteria; Terrabacteria group; Actinobacteria; Actinomycetia; Corynebacteriales; Mycobacteriaceae; Mycobacteroides; Mycobacteroides abscessus; Mycobacteroides abscessus subsp. abscessus;	NCBI: 1185650	5165259	100.00	0.33	Yes
NCBI Assembly	GCA_004111185.1	NCBI: cellular organisms; Bacteria; Terrabacteria group; Actinobacteria; Actinomycetia; Corynebacteriales; Mycobacteriaceae; Mycobacterium; Mycobacterium tuberculosis complex; Mycobacterium tuberculosis;	NCBI: 1773	4388465	99.94	0.00	Yes
NCBI Assembly	GCA_003347205.1	NCBI: cellular organisms; Bacteria; Terrabacteria group; Actinobacteria; Actinomycetia; Corynebacteriales; Mycobacteriaceae; Mycolicibacterium; Mycolicibacterium moriokaense;	NCBI: 39691	7123288	100.00	1.44	Yes
NCBI Assembly	GCA_003398185.1	NCBI: cellular organisms; Bacteria; Terrabacteria group; Actinobacteria; Actinomycetia; Corynebacteriales; Mycobacteriaceae; Mycobacterium; Mycobacterium tuberculosis complex; Mycobacterium tuberculosis;	NCBI: 1773	4371330	99.94	0.00	Yes
NCBI Assembly	GCA_003396215.1	NCBI: cellular organisms; Bacteria; Terrabacteria group; Actinobacteria; Actinomycetia; Corynebacteriales; Mycobacteriaceae; Mycobacterium; Mycobacterium tuberculosis complex; Mycobacterium tuberculosis;	NCBI: 1773	4241189	99.69	0.11	Yes
NCBI Assembly	GCA_003394645.1	NCBI: cellular organisms; Bacteria; Terrabacteria group; Actinobacteria; Actinomycetia; Corynebacteriales; Mycobacteriaceae; Mycobacterium; Mycobacterium tuberculosis complex; Mycobacterium tuberculosis;	NCBI: 1773	4318624	99.69	0.00	Yes
NCBI Assembly	GCA_003395575.1	NCBI: cellular organisms; Bacteria; Terrabacteria group; Actinobacteria; Actinomycetia; Corynebacteriales; Mycobacteriaceae; Mycobacterium; Mycobacterium tuberculosis complex; Mycobacterium tuberculosis;	NCBI: 1773	4378588	99.86	0.00	Yes
NCBI Assembly	GCA_003394925.1	NCBI: cellular organisms; Bacteria; Terrabacteria group; Actinobacteria; Actinomycetia; Corynebacteriales; Mycobacteriaceae; Mycobacterium; Mycobacterium tuberculosis complex; Mycobacterium tuberculosis;	NCBI: 1773	4326050	99.86	0.11	Yes
NCBI Assembly	GCA_003393955.1	NCBI: cellular organisms; Bacteria; Terrabacteria group; Actinobacteria; Actinomycetia; Corynebacteriales; Mycobacteriaceae; Mycobacterium; Mycobacterium tuberculosis complex; Mycobacterium tuberculosis;	NCBI: 1773	4336768	99.86	0.00	Yes
NCBI Assembly	GCA_003396545.1	NCBI: cellular organisms; Bacteria; Terrabacteria group; Actinobacteria; Actinomycetia; Corynebacteriales; Mycobacteriaceae; Mycobacterium; Mycobacterium tuberculosis complex; Mycobacterium tuberculosis;	NCBI: 1773	4323656	99.82	0.00	Yes
NCBI Assembly	GCA_004109615.1	NCBI: cellular organisms; Bacteria; Terrabacteria group; Actinobacteria; Actinomycetia; Corynebacteriales; Mycobacteriaceae; Mycobacterium; Mycobacterium tuberculosis complex; Mycobacterium tuberculosis;	NCBI: 1773	4382635	99.94	0.00	Yes
NCBI Assembly	GCA_004104565.1	NCBI: cellular organisms; Bacteria; Terrabacteria group; Actinobacteria; Actinomycetia; Corynebacteriales; Mycobacteriaceae; Mycobacterium; Mycobacterium tuberculosis complex; Mycobacterium tuberculosis;	NCBI: 1773	4386099	99.94	0.00	Yes

NCBI Assembly	GCA_004106135.1	NCBI: cellular organisms; Bacteria; Terrabacteria group; Actinobacteria; Actinomycetia; Corynebacteriales; Mycobacteriaceae; Mycobacterium; Mycobacterium tuberculosis complex; Mycobacterium tuberculosis;	NCBI: 1773	4410281	99.94	0.00	Yes
NCBI Assembly	GCA_004111225.1	NCBI: cellular organisms; Bacteria; Terrabacteria group; Actinobacteria; Actinomycetia; Corynebacteriales; Mycobacteriaceae; Mycobacterium; Mycobacterium tuberculosis complex; Mycobacterium tuberculosis;	NCBI: 1773	4380085	99.94	0.00	Yes
NCBI Assembly	GCA_003367335.1	NCBI: cellular organisms; Bacteria; Terrabacteria group; Actinobacteria; Actinomycetia; Corynebacteriales; Mycobacteriaceae; Mycolicibacterium; Mycolicibacterium neoaurum;	NCBI: 1795	5421383	99.62	1.44	Yes
NCBI Assembly	GCA_003398195.1	NCBI: cellular organisms; Bacteria; Terrabacteria group; Actinobacteria; Actinomycetia; Corynebacteriales; Mycobacteriaceae; Mycobacterium; Mycobacterium tuberculosis complex; Mycobacterium tuberculosis;	NCBI: 1773	4374774	99.86	0.00	Yes
NCBI Assembly	GCA_003396245.1	NCBI: cellular organisms; Bacteria; Terrabacteria group; Actinobacteria; Actinomycetia; Corynebacteriales; Mycobacteriaceae; Mycobacterium; Mycobacterium tuberculosis complex; Mycobacterium tuberculosis;	NCBI: 1773	4362339	99.86	0.17	Yes
NCBI Assembly	GCA_003395585.1	NCBI: cellular organisms; Bacteria; Terrabacteria group; Actinobacteria; Actinomycetia; Corynebacteriales; Mycobacteriaceae; Mycobacterium; Mycobacterium tuberculosis complex; Mycobacterium tuberculosis;	NCBI: 1773	4395253	99.86	0.00	Yes
NCBI Assembly	GCA_003393985.1	NCBI: cellular organisms; Bacteria; Terrabacteria group; Actinobacteria; Actinomycetia; Corynebacteriales; Mycobacteriaceae; Mycobacterium; Mycobacterium tuberculosis complex; Mycobacterium tuberculosis;	NCBI: 1773	4850740	100.00	16.22	No
NCBI Assembly	GCA_003394655.1	NCBI: cellular organisms; Bacteria; Terrabacteria group; Actinobacteria; Actinomycetia; Corynebacteriales; Mycobacteriaceae; Mycobacterium; Mycobacterium tuberculosis complex; Mycobacterium tuberculosis;	NCBI: 1773	4348961	99.86	0.06	Yes
NCBI Assembly	GCA_003394965.1	NCBI: cellular organisms; Bacteria; Terrabacteria group; Actinobacteria; Actinomycetia; Corynebacteriales; Mycobacteriaceae; Mycobacterium; Mycobacterium tuberculosis complex; Mycobacterium tuberculosis;	NCBI: 1773	4297608	99.86	0.00	Yes
NCBI Assembly	GCA_003396555.1	NCBI: cellular organisms; Bacteria; Terrabacteria group; Actinobacteria; Actinomycetia; Corynebacteriales; Mycobacteriaceae; Mycobacterium; Mycobacterium tuberculosis complex; Mycobacterium tuberculosis;	NCBI: 1773	4344355	99.69	0.11	Yes
NCBI Assembly	GCA_004104575.1	NCBI: cellular organisms; Bacteria; Terrabacteria group; Actinobacteria; Actinomycetia; Corynebacteriales; Mycobacteriaceae; Mycobacterium; Mycobacterium tuberculosis complex; Mycobacterium tuberculosis;	NCBI: 1773	4377311	99.94	0.00	Yes
NCBI Assembly	GCA_004109625.1	NCBI: cellular organisms; Bacteria; Terrabacteria group; Actinobacteria; Actinomycetia; Corynebacteriales; Mycobacteriaceae; Mycobacterium; Mycobacterium tuberculosis complex; Mycobacterium tuberculosis;	NCBI: 1773	4390516	99.94	0.00	Yes
NCBI Assembly	GCA_004111235.1	NCBI: cellular organisms; Bacteria; Terrabacteria group; Actinobacteria; Actinomycetia; Corynebacteriales; Mycobacteriaceae; Mycobacterium; Mycobacterium tuberculosis complex; Mycobacterium tuberculosis;	NCBI: 1773	4408332	99.94	0.00	Yes
NCBI Assembly	GCA_004106165.1	NCBI: cellular organisms; Bacteria; Terrabacteria group; Actinobacteria; Actinomycetia; Corynebacteriales; Mycobacteriaceae; Mycobacterium; Mycobacterium tuberculosis complex; Mycobacterium tuberculosis;	NCBI: 1773	4410515	99.61	0.00	Yes
NCBI Assembly	GCA_003383135.1	NCBI: cellular organisms; Bacteria; Terrabacteria group; Actinobacteria; Actinomycetia; Corynebacteriales; Mycobacteriaceae; Mycobacterium; Mycobacterium tuberculosis complex; Mycobacterium tuberculosis;	NCBI: 1773	4341817	99.94	0.00	Yes
NCBI Assembly	GCA_003398205.1	NCBI: cellular organisms; Bacteria; Terrabacteria group; Actinobacteria; Actinomycetia; Corynebacteriales; Mycobacteriaceae; Mycobacterium; Mycobacterium tuberculosis complex; Mycobacterium tuberculosis;	NCBI: 1773	4343613	99.86	0.44	Yes
NCBI Assembly	GCA_003396255.1	NCBI: cellular organisms; Bacteria; Terrabacteria group; Actinobacteria; Actinomycetia; Corynebacteriales; Mycobacteriaceae; Mycobacterium; Mycobacterium tuberculosis complex; Mycobacterium tuberculosis;	NCBI: 1773	4352256	99.86	0.00	Yes
NCBI Assembly	GCA_003393995.1	NCBI: cellular organisms; Bacteria; Terrabacteria group; Actinobacteria; Actinomycetia; Corynebacteriales; Mycobacteriaceae; Mycobacterium; Mycobacterium tuberculosis complex; Mycobacterium tuberculosis;	NCBI: 1773	5187470	100.00	38.39	No

NCBI Assembly	GCA_003394665.1	NCBI: cellular organisms; Bacteria; Terrabacteria group; Actinobacteria; Actinomycetia; Corynebacteriales; Mycobacteriaceae; Mycobacterium; Mycobacterium tuberculosis complex; Mycobacterium tuberculosis;	NCBI: 1773	4373745	99.86	0.00	Yes
NCBI Assembly	GCA_003395625.1	NCBI: cellular organisms; Bacteria; Terrabacteria group; Actinobacteria; Actinomycetia; Corynebacteriales; Mycobacteriaceae; Mycobacterium; Mycobacterium tuberculosis complex; Mycobacterium tuberculosis;	NCBI: 1773	4363160	99.86	0.00	Yes
NCBI Assembly	GCA_003396585.1	NCBI: cellular organisms; Bacteria; Terrabacteria group; Actinobacteria; Actinomycetia; Corynebacteriales; Mycobacteriaceae; Mycobacterium; Mycobacterium tuberculosis complex; Mycobacterium tuberculosis;	NCBI: 1773	4359331	99.86	0.00	Yes
NCBI Assembly	GCA_003394985.1	NCBI: cellular organisms; Bacteria; Terrabacteria group; Actinobacteria; Actinomycetia; Corynebacteriales; Mycobacteriaceae; Mycobacterium; Mycobacterium tuberculosis complex; Mycobacterium tuberculosis;	NCBI: 1773	4346101	99.86	0.08	Yes
NCBI Assembly	GCA_004104585.1	NCBI: cellular organisms; Bacteria; Terrabacteria group; Actinobacteria; Actinomycetia; Corynebacteriales; Mycobacteriaceae; Mycobacterium; Mycobacterium tuberculosis complex; Mycobacterium tuberculosis;	NCBI: 1773	4395451	99.94	0.00	Yes
NCBI Assembly	GCA_004109635.1	NCBI: cellular organisms; Bacteria; Terrabacteria group; Actinobacteria; Actinomycetia; Corynebacteriales; Mycobacteriaceae; Mycobacterium; Mycobacterium tuberculosis complex; Mycobacterium tuberculosis;	NCBI: 1773	4377670	99.94	0.00	Yes
NCBI Assembly	GCA_004111245.1	NCBI: cellular organisms; Bacteria; Terrabacteria group; Actinobacteria; Actinomycetia; Corynebacteriales; Mycobacteriaceae; Mycobacterium; Mycobacterium tuberculosis complex; Mycobacterium tuberculosis;	NCBI: 1773	4386804	99.61	0.00	Yes
NCBI Assembly	GCA_004106185.1	NCBI: cellular organisms; Bacteria; Terrabacteria group; Actinobacteria; Actinomycetia; Corynebacteriales; Mycobacteriaceae; Mycobacterium; Mycobacterium tuberculosis complex; Mycobacterium tuberculosis;	NCBI: 1773	4370197	99.94	0.00	Yes
NCBI Assembly	GCA_003383145.1	NCBI: cellular organisms; Bacteria; Terrabacteria group; Actinobacteria; Actinomycetia; Corynebacteriales; Mycobacteriaceae; Mycobacterium; Mycobacterium tuberculosis complex; Mycobacterium tuberculosis;	NCBI: 1773	4437279	99.94	0.00	Yes
NCBI Assembly	GCA_003396285.1	NCBI: cellular organisms; Bacteria; Terrabacteria group; Actinobacteria; Actinomycetia; Corynebacteriales; Mycobacteriaceae; Mycobacterium; Mycobacterium tuberculosis complex; Mycobacterium tuberculosis;	NCBI: 1773	4370280	99.86	0.00	Yes
NCBI Assembly	GCA_003394025.1	NCBI: cellular organisms; Bacteria; Terrabacteria group; Actinobacteria; Actinomycetia; Corynebacteriales; Mycobacteriaceae; Mycobacterium; Mycobacterium tuberculosis complex; Mycobacterium tuberculosis;	NCBI: 1773	4328048	99.86	0.33	Yes
NCBI Assembly	GCA_003398235.1	NCBI: cellular organisms; Bacteria; Terrabacteria group; Actinobacteria; Actinomycetia; Corynebacteriales; Mycobacteriaceae; Mycobacterium; Mycobacterium tuberculosis complex; Mycobacterium tuberculosis;	NCBI: 1773	4318501	99.86	0.00	Yes
NCBI Assembly	GCA_003396595.1	NCBI: cellular organisms; Bacteria; Terrabacteria group; Actinobacteria; Actinomycetia; Corynebacteriales; Mycobacteriaceae; Mycobacterium; Mycobacterium tuberculosis complex; Mycobacterium tuberculosis;	NCBI: 1773	4326229	99.86	0.00	Yes
NCBI Assembly	GCA_003394695.1	NCBI: cellular organisms; Bacteria; Terrabacteria group; Actinobacteria; Actinomycetia; Corynebacteriales; Mycobacteriaceae; Mycobacterium; Mycobacterium tuberculosis complex; Mycobacterium tuberculosis;	NCBI: 1773	4313490	99.86	0.00	Yes
NCBI Assembly	GCA_003395645.1	NCBI: cellular organisms; Bacteria; Terrabacteria group; Actinobacteria; Actinomycetia; Corynebacteriales; Mycobacteriaceae; Mycobacterium; Mycobacterium tuberculosis complex; Mycobacterium tuberculosis;	NCBI: 1773	4326588	99.89	0.11	Yes
NCBI Assembly	GCA_003394995.1	NCBI: cellular organisms; Bacteria; Terrabacteria group; Actinobacteria; Actinomycetia; Corynebacteriales; Mycobacteriaceae; Mycobacterium; Mycobacterium tuberculosis complex; Mycobacterium tuberculosis;	NCBI: 1773	4387312	99.86	0.00	Yes
NCBI Assembly	GCA_004104595.1	NCBI: cellular organisms; Bacteria; Terrabacteria group; Actinobacteria; Actinomycetia; Corynebacteriales; Mycobacteriaceae; Mycobacterium; Mycobacterium tuberculosis complex; Mycobacterium tuberculosis;	NCBI: 1773	4402579	99.94	0.00	Yes
NCBI Assembly	GCA_004109645.1	NCBI: cellular organisms; Bacteria; Terrabacteria group; Actinobacteria; Actinomycetia; Corynebacteriales; Mycobacteriaceae; Mycobacterium; Mycobacterium tuberculosis complex; Mycobacterium tuberculosis;	NCBI: 1773	4368398	99.94	0.00	Yes

NCBI Assembly	GCA_004111275.1	NCBI: cellular organisms; Bacteria; Terrabacteria group; Actinobacteria; Actinomycetia; Corynebacteriales; Mycobacteriaceae; Mycobacterium; Mycobacterium tuberculosis complex; Mycobacterium tuberculosis;	NCBI: 1773	4377131	99.94	0.00	Yes
NCBI Assembly	GCA_004106195.1	NCBI: cellular organisms; Bacteria; Terrabacteria group; Actinobacteria; Actinomycetia; Corynebacteriales; Mycobacteriaceae; Mycobacterium; Mycobacterium tuberculosis complex; Mycobacterium tuberculosis;	NCBI: 1773	4394579	99.94	0.00	Yes
NCBI Assembly	GCA_003383185.1	NCBI: cellular organisms; Bacteria; Terrabacteria group; Actinobacteria; Actinomycetia; Corynebacteriales; Mycobacteriaceae; Mycobacterium; Mycobacterium tuberculosis complex; Mycobacterium tuberculosis;	NCBI: 1773	4310676	99.61	0.00	Yes
NCBI Assembly	GCA_003396295.1	NCBI: cellular organisms; Bacteria; Terrabacteria group; Actinobacteria; Actinomycetia; Corynebacteriales; Mycobacteriaceae; Mycobacterium; Mycobacterium tuberculosis complex; Mycobacterium tuberculosis;	NCBI: 1773	4354303	99.94	0.00	Yes
NCBI Assembly	GCA_003394035.1	NCBI: cellular organisms; Bacteria; Terrabacteria group; Actinobacteria; Actinomycetia; Corynebacteriales; Mycobacteriaceae; Mycobacterium; Mycobacterium tuberculosis complex; Mycobacterium tuberculosis;	NCBI: 1773	4395559	99.86	0.33	Yes
NCBI Assembly	GCA_003398255.1	NCBI: cellular organisms; Bacteria; Terrabacteria group; Actinobacteria; Actinomycetia; Corynebacteriales; Mycobacteriaceae; Mycobacterium; Mycobacterium tuberculosis complex; Mycobacterium tuberculosis;	NCBI: 1773	4366097	99.86	0.00	Yes
NCBI Assembly	GCA_003396625.1	NCBI: cellular organisms; Bacteria; Terrabacteria group; Actinobacteria; Actinomycetia; Corynebacteriales; Mycobacteriaceae; Mycobacterium; Mycobacterium tuberculosis complex; Mycobacterium tuberculosis;	NCBI: 1773	4276663	98.63	1.72	Yes
NCBI Assembly	GCA_003395665.1	NCBI: cellular organisms; Bacteria; Terrabacteria group; Actinobacteria; Actinomycetia; Corynebacteriales; Mycobacteriaceae; Mycobacterium; Mycobacterium tuberculosis complex; Mycobacterium tuberculosis;	NCBI: 1773	4389514	99.94	0.00	Yes
NCBI Assembly	GCA_003394725.1	NCBI: cellular organisms; Bacteria; Terrabacteria group; Actinobacteria; Actinomycetia; Corynebacteriales; Mycobacteriaceae; Mycobacterium; Mycobacterium tuberculosis complex; Mycobacterium tuberculosis;	NCBI: 1773	4354507	99.86	0.17	Yes
NCBI Assembly	GCA_003395025.1	NCBI: cellular organisms; Bacteria; Terrabacteria group; Actinobacteria; Actinomycetia; Corynebacteriales; Mycobacteriaceae; Mycobacterium; Mycobacterium tuberculosis complex; Mycobacterium tuberculosis;	NCBI: 1773	4314955	99.69	0.28	Yes
NCBI Assembly	GCA_004104605.1	NCBI: cellular organisms; Bacteria; Terrabacteria group; Actinobacteria; Actinomycetia; Corynebacteriales; Mycobacteriaceae; Mycobacterium; Mycobacterium tuberculosis complex; Mycobacterium tuberculosis;	NCBI: 1773	4377762	99.61	0.00	Yes
NCBI Assembly	GCA_004109705.1	NCBI: cellular organisms; Bacteria; Terrabacteria group; Actinobacteria; Actinomycetia; Corynebacteriales; Mycobacteriaceae; Mycobacterium; Mycobacterium tuberculosis complex; Mycobacterium tuberculosis;	NCBI: 1773	4378557	99.94	0.00	Yes
NCBI Assembly	GCA_004111285.1	NCBI: cellular organisms; Bacteria; Terrabacteria group; Actinobacteria; Actinomycetia; Corynebacteriales; Mycobacteriaceae; Mycobacterium; Mycobacterium tuberculosis complex; Mycobacterium tuberculosis;	NCBI: 1773	4392627	99.94	0.00	Yes
NCBI Assembly	GCA_004106205.1	NCBI: cellular organisms; Bacteria; Terrabacteria group; Actinobacteria; Actinomycetia; Corynebacteriales; Mycobacteriaceae; Mycobacterium; Mycobacterium tuberculosis complex; Mycobacterium tuberculosis;	NCBI: 1773	4399468	99.61	0.00	Yes
NCBI Assembly	GCA_003383205.1	NCBI: cellular organisms; Bacteria; Terrabacteria group; Actinobacteria; Actinomycetia; Corynebacteriales; Mycobacteriaceae; Mycobacterium; Mycobacterium tuberculosis complex; Mycobacterium tuberculosis;	NCBI: 1773	4351252	99.94	0.00	Yes
NCBI Assembly	GCA_003396305.1	NCBI: cellular organisms; Bacteria; Terrabacteria group; Actinobacteria; Actinomycetia; Corynebacteriales; Mycobacteriaceae; Mycobacterium; Mycobacterium tuberculosis complex; Mycobacterium tuberculosis;	NCBI: 1773	4353625	99.86	0.00	Yes
NCBI Assembly	GCA_003394045.1	NCBI: cellular organisms; Bacteria; Terrabacteria group; Actinobacteria; Actinomycetia; Corynebacteriales; Mycobacteriaceae; Mycobacterium; Mycobacterium tuberculosis complex; Mycobacterium tuberculosis;	NCBI: 1773	4352034	99.86	0.44	Yes
NCBI Assembly	GCA_003395685.1	NCBI: cellular organisms; Bacteria; Terrabacteria group; Actinobacteria; Actinomycetia; Corynebacteriales; Mycobacteriaceae; Mycobacterium; Mycobacterium tuberculosis complex; Mycobacterium tuberculosis;	NCBI: 1773	4372810	99.53	0.11	Yes

NCBI Assembly	GCA_003398275.1	NCBI: cellular organisms; Bacteria; Terrabacteria group; Actinobacteria; Actinomycetia; Corynebacteriales; Mycobacteriaceae; Mycobacterium; Mycobacterium tuberculosis complex; Mycobacterium tuberculosis;	NCBI: 1773	4365825	99.69	0.00	Yes
NCBI Assembly	GCA_003396635.1	NCBI: cellular organisms; Bacteria; Terrabacteria group; Actinobacteria; Actinomycetia; Corynebacteriales; Mycobacteriaceae; Mycobacterium; Mycobacterium tuberculosis complex; Mycobacterium tuberculosis;	NCBI: 1773	4410876	99.86	0.33	Yes
NCBI Assembly	GCA_003394735.1	NCBI: cellular organisms; Bacteria; Terrabacteria group; Actinobacteria; Actinomycetia; Corynebacteriales; Mycobacteriaceae; Mycobacterium; Mycobacterium tuberculosis complex; Mycobacterium tuberculosis;	NCBI: 1773	4360903	99.86	0.00	Yes
NCBI Assembly	GCA_003395035.1	NCBI: cellular organisms; Bacteria; Terrabacteria group; Actinobacteria; Actinomycetia; Corynebacteriales; Mycobacteriaceae; Mycobacterium; Mycobacterium tuberculosis complex; Mycobacterium tuberculosis;	NCBI: 1773	4330585	99.86	0.00	Yes
NCBI Assembly	GCA_004104665.1	NCBI: cellular organisms; Bacteria; Terrabacteria group; Actinobacteria; Actinomycetia; Corynebacteriales; Mycobacteriaceae; Mycobacterium; Mycobacterium tuberculosis complex; Mycobacterium tuberculosis;	NCBI: 1773	4389501	99.94	0.00	Yes
NCBI Assembly	GCA_004109715.1	NCBI: cellular organisms; Bacteria; Terrabacteria group; Actinobacteria; Actinomycetia; Corynebacteriales; Mycobacteriaceae; Mycobacterium; Mycobacterium tuberculosis complex; Mycobacterium tuberculosis;	NCBI: 1773	4385550	99.94	0.00	Yes
NCBI Assembly	GCA_004111325.1	NCBI: cellular organisms; Bacteria; Terrabacteria group; Actinobacteria; Actinomycetia; Corynebacteriales; Mycobacteriaceae; Mycobacterium; Mycobacterium tuberculosis complex; Mycobacterium tuberculosis;	NCBI: 1773	4389248	99.28	0.00	Yes
NCBI Assembly	GCA_003383245.1	NCBI: cellular organisms; Bacteria; Terrabacteria group; Actinobacteria; Actinomycetia; Corynebacteriales; Mycobacteriaceae; Mycobacterium; Mycobacterium tuberculosis complex; Mycobacterium tuberculosis;	NCBI: 1773	4435455	99.94	0.00	Yes
NCBI Assembly	GCA_003394145.1	NCBI: cellular organisms; Bacteria; Terrabacteria group; Actinobacteria; Actinomycetia; Corynebacteriales; Mycobacteriaceae; Mycobacterium; Mycobacterium tuberculosis complex; Mycobacterium tuberculosis;	NCBI: 1773	4340107	99.53	0.75	Yes
NCBI Assembly	GCA_004106225.1	NCBI: cellular organisms; Bacteria; Terrabacteria group; Actinobacteria; Actinomycetia; Corynebacteriales; Mycobacteriaceae; Mycobacterium; Mycobacterium tuberculosis complex; Mycobacterium tuberculosis;	NCBI: 1773	4394900	99.94	0.00	Yes
NCBI Assembly	GCA_003396345.1	NCBI: cellular organisms; Bacteria; Terrabacteria group; Actinobacteria; Actinomycetia; Corynebacteriales; Mycobacteriaceae; Mycobacterium; Mycobacterium tuberculosis complex; Mycobacterium tuberculosis;	NCBI: 1773	4373002	99.86	0.00	Yes
NCBI Assembly	GCA_003398285.1	NCBI: cellular organisms; Bacteria; Terrabacteria group; Actinobacteria; Actinomycetia; Corynebacteriales; Mycobacteriaceae; Mycobacterium; Mycobacterium tuberculosis complex; Mycobacterium tuberculosis;	NCBI: 1773	4341742	99.69	0.00	Yes
NCBI Assembly	GCA_003395695.1	NCBI: cellular organisms; Bacteria; Terrabacteria group; Actinobacteria; Actinomycetia; Corynebacteriales; Mycobacteriaceae; Mycobacterium; Mycobacterium tuberculosis complex; Mycobacterium tuberculosis;	NCBI: 1773	4376428	99.86	0.00	Yes
NCBI Assembly	GCA_003394765.1	NCBI: cellular organisms; Bacteria; Terrabacteria group; Actinobacteria; Actinomycetia; Corynebacteriales; Mycobacteriaceae; Mycobacterium; Mycobacterium tuberculosis complex; Mycobacterium tuberculosis;	NCBI: 1773	4341716	99.86	0.00	Yes
NCBI Assembly	GCA_003396645.1	NCBI: cellular organisms; Bacteria; Terrabacteria group; Actinobacteria; Actinomycetia; Corynebacteriales; Mycobacteriaceae; Mycobacterium; Mycobacterium tuberculosis complex; Mycobacterium tuberculosis;	NCBI: 1773	4375121	99.69	0.00	Yes
NCBI Assembly	GCA_003395065.1	NCBI: cellular organisms; Bacteria; Terrabacteria group; Actinobacteria; Actinomycetia; Corynebacteriales; Mycobacteriaceae; Mycobacterium; Mycobacterium tuberculosis complex; Mycobacterium tuberculosis;	NCBI: 1773	4351336	99.86	0.83	Yes
NCBI Assembly	GCA_004104675.1	NCBI: cellular organisms; Bacteria; Terrabacteria group; Actinobacteria; Actinomycetia; Corynebacteriales; Mycobacteriaceae; Mycobacterium; Mycobacterium tuberculosis complex; Mycobacterium tuberculosis;	NCBI: 1773	4398981	99.94	0.00	Yes
NCBI Assembly	GCA_003383255.1	NCBI: cellular organisms; Bacteria; Terrabacteria group; Actinobacteria; Actinomycetia; Corynebacteriales; Mycobacteriaceae; Mycobacterium; Mycobacterium tuberculosis complex; Mycobacterium tuberculosis;	NCBI: 1773	4365549	99.90	0.00	Yes

NCBI Assembly	GCA_003396355.1	NCBI: cellular organisms; Bacteria; Terrabacteria group; Actinobacteria; Actinomycetia; Corynebacteriales; Mycobacteriaceae; Mycobacterium; Mycobacterium tuberculosis complex; Mycobacterium tuberculosis;	NCBI: 1773	4379831	99.86	0.00	Yes
NCBI Assembly	GCA_003394155.1	NCBI: cellular organisms; Bacteria; Terrabacteria group; Actinobacteria; Actinomycetia; Corynebacteriales; Mycobacteriaceae; Mycobacterium; Mycobacterium tuberculosis complex; Mycobacterium tuberculosis;	NCBI: 1773	4342209	99.86	0.00	Yes
NCBI Assembly	GCA_004109725.1	NCBI: cellular organisms; Bacteria; Terrabacteria group; Actinobacteria; Actinomycetia; Corynebacteriales; Mycobacteriaceae; Mycobacterium; Mycobacterium tuberculosis complex; Mycobacterium tuberculosis;	NCBI: 1773	4361175	99.94	0.00	Yes
NCBI Assembly	GCA_004111335.1	NCBI: cellular organisms; Bacteria; Terrabacteria group; Actinobacteria; Actinomycetia; Corynebacteriales; Mycobacteriaceae; Mycobacterium; Mycobacterium tuberculosis complex; Mycobacterium tuberculosis;	NCBI: 1773	4374394	99.94	0.00	Yes
NCBI Assembly	GCA_003398325.1	NCBI: cellular organisms; Bacteria; Terrabacteria group; Actinobacteria; Actinomycetia; Corynebacteriales; Mycobacteriaceae; Mycobacterium; Mycobacterium tuberculosis complex; Mycobacterium tuberculosis;	NCBI: 1773	4353810	99.94	0.00	Yes
NCBI Assembly	GCA_003395725.1	NCBI: cellular organisms; Bacteria; Terrabacteria group; Actinobacteria; Actinomycetia; Corynebacteriales; Mycobacteriaceae; Mycobacterium; Mycobacterium tuberculosis complex; Mycobacterium tuberculosis;	NCBI: 1773	4373242	99.94	0.00	Yes
NCBI Assembly	GCA_004106265.1	NCBI: cellular organisms; Bacteria; Terrabacteria group; Actinobacteria; Actinomycetia; Corynebacteriales; Mycobacteriaceae; Mycobacterium; Mycobacterium tuberculosis complex; Mycobacterium tuberculosis;	NCBI: 1773	4401927	99.61	0.00	Yes
NCBI Assembly	GCA_003394775.1	NCBI: cellular organisms; Bacteria; Terrabacteria group; Actinobacteria; Actinomycetia; Corynebacteriales; Mycobacteriaceae; Mycobacterium; Mycobacterium tuberculosis complex; Mycobacterium tuberculosis;	NCBI: 1773	4353722	99.86	0.00	Yes
NCBI Assembly	GCA_003395085.1	NCBI: cellular organisms; Bacteria; Terrabacteria group; Actinobacteria; Actinomycetia; Corynebacteriales; Mycobacteriaceae; Mycobacterium; Mycobacterium tuberculosis complex; Mycobacterium tuberculosis;	NCBI: 1773	4359927	99.86	0.00	Yes
NCBI Assembly	GCA_003396675.1	NCBI: cellular organisms; Bacteria; Terrabacteria group; Actinobacteria; Actinomycetia; Corynebacteriales; Mycobacteriaceae; Mycobacterium; Mycobacterium tuberculosis complex; Mycobacterium tuberculosis;	NCBI: 1773	4428578	99.86	0.33	Yes
NCBI Assembly	GCA_003383265.1	NCBI: cellular organisms; Bacteria; Terrabacteria group; Actinobacteria; Actinomycetia; Corynebacteriales; Mycobacteriaceae; Mycobacterium; Mycobacterium tuberculosis complex; Mycobacterium tuberculosis;	NCBI: 1773	4341535	99.83	0.00	Yes
NCBI Assembly	GCA_004104685.1	NCBI: cellular organisms; Bacteria; Terrabacteria group; Actinobacteria; Actinomycetia; Corynebacteriales; Mycobacteriaceae; Mycobacterium; Mycobacterium tuberculosis complex; Mycobacterium tuberculosis;	NCBI: 1773	4414862	99.61	0.00	Yes
NCBI Assembly	GCA_003396385.1	NCBI: cellular organisms; Bacteria; Terrabacteria group; Actinobacteria; Actinomycetia; Corynebacteriales; Mycobacteriaceae; Mycobacterium; Mycobacterium tuberculosis complex; Mycobacterium tuberculosis;	NCBI: 1773	4338878	99.86	0.00	Yes
NCBI Assembly	GCA_003394165.1	NCBI: cellular organisms; Bacteria; Terrabacteria group; Actinobacteria; Actinomycetia; Corynebacteriales; Mycobacteriaceae; Mycobacterium; Mycobacterium tuberculosis complex; Mycobacterium tuberculosis;	NCBI: 1773	4249646	99.58	1.79	Yes
NCBI Assembly	GCA_003398345.1	NCBI: cellular organisms; Bacteria; Terrabacteria group; Actinobacteria; Actinomycetia; Corynebacteriales; Mycobacteriaceae; Mycobacterium; Mycobacterium tuberculosis complex; Mycobacterium tuberculosis;	NCBI: 1773	4326648	99.86	0.11	Yes
NCBI Assembly	GCA_003395745.1	NCBI: cellular organisms; Bacteria; Terrabacteria group; Actinobacteria; Actinomycetia; Corynebacteriales; Mycobacteriaceae; Mycobacterium; Mycobacterium tuberculosis complex; Mycobacterium tuberculosis;	NCBI: 1773	4358864	99.86	0.00	Yes
NCBI Assembly	GCA_004109765.1	NCBI: cellular organisms; Bacteria; Terrabacteria group; Actinobacteria; Actinomycetia; Corynebacteriales; Mycobacteriaceae; Mycobacterium; Mycobacterium tuberculosis complex; Mycobacterium tuberculosis;	NCBI: 1773	4388210	99.61	0.00	Yes
NCBI Assembly	GCA_003395095.1	NCBI: cellular organisms; Bacteria; Terrabacteria group; Actinobacteria; Actinomycetia; Corynebacteriales; Mycobacteriaceae; Mycobacterium; Mycobacterium tuberculosis complex; Mycobacterium tuberculosis;	NCBI: 1773	4404216	99.86	0.06	Yes

NCBI Assembly	GCA_003394805.1	NCBI: cellular organisms; Bacteria; Terrabacteria group; Actinobacteria; Actinomycetia; Corynebacteriales; Mycobacteriaceae; Mycobacterium; Mycobacterium tuberculosis complex; Mycobacterium tuberculosis;	NCBI: 1773	4378170	99.86	0.78	Yes
NCBI Assembly	GCA_004111345.1	NCBI: cellular organisms; Bacteria; Terrabacteria group; Actinobacteria; Actinomycetia; Corynebacteriales; Mycobacteriaceae; Mycobacterium; Mycobacterium tuberculosis complex; Mycobacterium tuberculosis;	NCBI: 1773	4397956	99.61	0.00	Yes
NCBI Assembly	GCA_004106275.1	NCBI: cellular organisms; Bacteria; Terrabacteria group; Actinobacteria; Actinomycetia; Corynebacteriales; Mycobacteriaceae; Mycobacterium; Mycobacterium tuberculosis complex; Mycobacterium tuberculosis;	NCBI: 1773	4397616	99.94	0.00	Yes
NCBI Assembly	GCA_003396705.1	NCBI: cellular organisms; Bacteria; Terrabacteria group; Actinobacteria; Actinomycetia; Corynebacteriales; Mycobacteriaceae; Mycobacterium; Mycobacterium tuberculosis complex; Mycobacterium tuberculosis;	NCBI: 1773	4374224	99.86	0.17	Yes
NCBI Assembly	GCA_003383305.1	NCBI: cellular organisms; Bacteria; Terrabacteria group; Actinobacteria; Actinomycetia; Corynebacteriales; Mycobacteriaceae; Mycobacterium; Mycobacterium tuberculosis complex; Mycobacterium tuberculosis;	NCBI: 1773	4375712	99.94	0.00	Yes
NCBI Assembly	GCA_004104695.1	NCBI: cellular organisms; Bacteria; Terrabacteria group; Actinobacteria; Actinomycetia; Corynebacteriales; Mycobacteriaceae; Mycobacterium; Mycobacterium tuberculosis complex; Mycobacterium tuberculosis;	NCBI: 1773	4356662	99.94	0.00	Yes
NCBI Assembly	GCA_003394175.1	NCBI: cellular organisms; Bacteria; Terrabacteria group; Actinobacteria; Actinomycetia; Corynebacteriales; Mycobacteriaceae; Mycobacterium; Mycobacterium tuberculosis complex; Mycobacterium tuberculosis;	NCBI: 1773	4273606	99.66	0.33	Yes
NCBI Assembly	GCA_003396395.1	NCBI: cellular organisms; Bacteria; Terrabacteria group; Actinobacteria; Actinomycetia; Corynebacteriales; Mycobacteriaceae; Mycobacterium; Mycobacterium tuberculosis complex; Mycobacterium tuberculosis;	NCBI: 1773	4349654	99.86	0.00	Yes
NCBI Assembly	GCA_003395755.1	NCBI: cellular organisms; Bacteria; Terrabacteria group; Actinobacteria; Actinomycetia; Corynebacteriales; Mycobacteriaceae; Mycobacterium; Mycobacterium tuberculosis complex; Mycobacterium tuberculosis;	NCBI: 1773	4369781	99.94	0.04	Yes
NCBI Assembly	GCA_003398355.1	NCBI: cellular organisms; Bacteria; Terrabacteria group; Actinobacteria; Actinomycetia; Corynebacteriales; Mycobacteriaceae; Mycobacterium; Mycobacterium tuberculosis complex; Mycobacterium tuberculosis;	NCBI: 1773	4285492	99.86	0.00	Yes
NCBI Assembly	GCA_003395115.1	NCBI: cellular organisms; Bacteria; Terrabacteria group; Actinobacteria; Actinomycetia; Corynebacteriales; Mycobacteriaceae; Mycobacterium; Mycobacterium tuberculosis complex; Mycobacterium tuberculosis;	NCBI: 1773	4321371	99.86	0.11	Yes
NCBI Assembly	GCA_004109775.1	NCBI: cellular organisms; Bacteria; Terrabacteria group; Actinobacteria; Actinomycetia; Corynebacteriales; Mycobacteriaceae; Mycobacterium; Mycobacterium tuberculosis complex; Mycobacterium tuberculosis;	NCBI: 1773	4387793	99.61	0.00	Yes
NCBI Assembly	GCA_003394815.1	NCBI: cellular organisms; Bacteria; Terrabacteria group; Actinobacteria; Actinomycetia; Corynebacteriales; Mycobacteriaceae; Mycobacterium; Mycobacterium tuberculosis complex; Mycobacterium tuberculosis;	NCBI: 1773	4367912	99.86	0.00	Yes
NCBI Assembly	GCA_003396715.1	NCBI: cellular organisms; Bacteria; Terrabacteria group; Actinobacteria; Actinomycetia; Corynebacteriales; Mycobacteriaceae; Mycobacterium; Mycobacterium tuberculosis complex; Mycobacterium tuberculosis;	NCBI: 1773	4339630	99.86	0.11	Yes
NCBI Assembly	GCA_004106285.1	NCBI: cellular organisms; Bacteria; Terrabacteria group; Actinobacteria; Actinomycetia; Corynebacteriales; Mycobacteriaceae; Mycobacterium; Mycobacterium tuberculosis complex; Mycobacterium tuberculosis;	NCBI: 1773	4417114	99.94	0.00	Yes
NCBI Assembly	GCA_004111355.1	NCBI: cellular organisms; Bacteria; Terrabacteria group; Actinobacteria; Actinomycetia; Corynebacteriales; Mycobacteriaceae; Mycobacterium; Mycobacterium tuberculosis complex; Mycobacterium tuberculosis;	NCBI: 1773	4382593	99.94	0.00	Yes
NCBI Assembly	GCA_003383315.1	NCBI: cellular organisms; Bacteria; Terrabacteria group; Actinobacteria; Actinomycetia; Corynebacteriales; Mycobacteriaceae; Mycobacterium; Mycobacterium tuberculosis complex; Mycobacterium tuberculosis;	NCBI: 1773	4357370	99.94	0.17	Yes
NCBI Assembly	GCA_003395765.1	NCBI: cellular organisms; Bacteria; Terrabacteria group; Actinobacteria; Actinomycetia; Corynebacteriales; Mycobacteriaceae; Mycobacterium; Mycobacterium tuberculosis complex; Mycobacterium tuberculosis;	NCBI: 1773	4352286	99.86	0.00	Yes

NCBI Assembly	GCA_003396405.1	NCBI: cellular organisms; Bacteria; Terrabacteria group; Actinobacteria; Actinomycetia; Corynebacteriales; Mycobacteriaceae; Mycobacterium; Mycobacterium tuberculosis complex; Mycobacterium tuberculosis;	NCBI: 1773	4378779	99.86	0.00	Yes
NCBI Assembly	GCA_003395145.1	NCBI: cellular organisms; Bacteria; Terrabacteria group; Actinobacteria; Actinomycetia; Corynebacteriales; Mycobacteriaceae; Mycobacterium; Mycobacterium tuberculosis complex; Mycobacterium tuberculosis;	NCBI: 1773	4355502	99.86	0.00	Yes
NCBI Assembly	GCA_003398365.1	NCBI: cellular organisms; Bacteria; Terrabacteria group; Actinobacteria; Actinomycetia; Corynebacteriales; Mycobacteriaceae; Mycobacterium; Mycobacterium tuberculosis complex; Mycobacterium tuberculosis;	NCBI: 1773	4327845	99.86	0.00	Yes
NCBI Assembly	GCA_004104715.1	NCBI: cellular organisms; Bacteria; Terrabacteria group; Actinobacteria; Actinomycetia; Corynebacteriales; Mycobacteriaceae; Mycobacterium; Mycobacterium tuberculosis complex; Mycobacterium tuberculosis;	NCBI: 1773	4370113	99.61	0.00	Yes
NCBI Assembly	GCA_003394225.1	NCBI: cellular organisms; Bacteria; Terrabacteria group; Actinobacteria; Actinomycetia; Corynebacteriales; Mycobacteriaceae; Mycobacterium; Mycobacterium tuberculosis complex; Mycobacterium tuberculosis;	NCBI: 1773	4354822	99.86	0.00	Yes
NCBI Assembly	GCA_003396745.1	NCBI: cellular organisms; Bacteria; Terrabacteria group; Actinobacteria; Actinomycetia; Corynebacteriales; Mycobacteriaceae; Mycobacterium; Mycobacterium tuberculosis complex; Mycobacterium tuberculosis;	NCBI: 1773	4412305	99.86	0.33	Yes
NCBI Assembly	GCA_003394845.1	NCBI: cellular organisms; Bacteria; Terrabacteria group; Actinobacteria; Actinomycetia; Corynebacteriales; Mycobacteriaceae; Mycobacterium; Mycobacterium tuberculosis complex; Mycobacterium tuberculosis;	NCBI: 1773	4351542	99.86	0.00	Yes
NCBI Assembly	GCA_004109825.1	NCBI: cellular organisms; Bacteria; Terrabacteria group; Actinobacteria; Actinomycetia; Corynebacteriales; Mycobacteriaceae; Mycobacterium; Mycobacterium tuberculosis complex; Mycobacterium tuberculosis;	NCBI: 1773	4336625	99.94	0.00	Yes
NCBI Assembly	GCA_004106295.1	NCBI: cellular organisms; Bacteria; Terrabacteria group; Actinobacteria; Actinomycetia; Corynebacteriales; Mycobacteriaceae; Mycobacterium; Mycobacterium tuberculosis complex; Mycobacterium tuberculosis;	NCBI: 1773	4397412	99.94	0.00	Yes
NCBI Assembly	GCA_004111375.1	NCBI: cellular organisms; Bacteria; Terrabacteria group; Actinobacteria; Actinomycetia; Corynebacteriales; Mycobacteriaceae; Mycobacterium; Mycobacterium tuberculosis complex; Mycobacterium tuberculosis;	NCBI: 1773	4388146	99.94	0.00	Yes
NCBI Assembly	GCA_003383325.1	NCBI: cellular organisms; Bacteria; Terrabacteria group; Actinobacteria; Actinomycetia; Corynebacteriales; Mycobacteriaceae; Mycobacterium; Mycobacterium tuberculosis complex; Mycobacterium tuberculosis;	NCBI: 1773	4333274	99.94	0.00	Yes
NCBI Assembly	GCA_003395805.1	NCBI: cellular organisms; Bacteria; Terrabacteria group; Actinobacteria; Actinomycetia; Corynebacteriales; Mycobacteriaceae; Mycobacterium; Mycobacterium tuberculosis complex; Mycobacterium tuberculosis;	NCBI: 1773	4353638	99.94	0.00	Yes
NCBI Assembly	GCA_003395165.1	NCBI: cellular organisms; Bacteria; Terrabacteria group; Actinobacteria; Actinomycetia; Corynebacteriales; Mycobacteriaceae; Mycobacterium; Mycobacterium tuberculosis complex; Mycobacterium tuberculosis;	NCBI: 1773	4724294	99.86	9.33	No
NCBI Assembly	GCA_003396445.1	NCBI: cellular organisms; Bacteria; Terrabacteria group; Actinobacteria; Actinomycetia; Corynebacteriales; Mycobacteriaceae; Mycobacterium; Mycobacterium tuberculosis complex; Mycobacterium tuberculosis;	NCBI: 1773	4353730	99.69	0.00	Yes
NCBI Assembly	GCA_003398385.1	NCBI: cellular organisms; Bacteria; Terrabacteria group; Actinobacteria; Actinomycetia; Corynebacteriales; Mycobacteriaceae; Mycobacterium; Mycobacterium tuberculosis complex; Mycobacterium tuberculosis;	NCBI: 1773	4209993	99.03	0.81	Yes
NCBI Assembly	GCA_003394235.1	NCBI: cellular organisms; Bacteria; Terrabacteria group; Actinobacteria; Actinomycetia; Corynebacteriales; Mycobacteriaceae; Mycobacterium; Mycobacterium tuberculosis complex; Mycobacterium tuberculosis;	NCBI: 1773	4313468	99.86	0.00	Yes
NCBI Assembly	GCA_004104765.1	NCBI: cellular organisms; Bacteria; Terrabacteria group; Actinobacteria; Actinomycetia; Corynebacteriales; Mycobacteriaceae; Mycobacterium; Mycobacterium tuberculosis complex; Mycobacterium tuberculosis;	NCBI: 1773	4384559	99.94	0.00	Yes
NCBI Assembly	GCA_003396755.1	NCBI: cellular organisms; Bacteria; Terrabacteria group; Actinobacteria; Actinomycetia; Corynebacteriales; Mycobacteriaceae; Mycobacterium; Mycobacterium tuberculosis complex; Mycobacterium tuberculosis;	NCBI: 1773	4369650	99.69	0.00	Yes

NCBI Assembly	GCA_003394855.1	NCBI: cellular organisms; Bacteria; Terrabacteria group; Actinobacteria; Actinomycetia; Corynebacteriales; Mycobacteriaceae; Mycobacterium; Mycobacterium tuberculosis complex; Mycobacterium tuberculosis;	NCBI: 1773	4374103	99.94	0.00	Yes
NCBI Assembly	GCA_004109835.1	NCBI: cellular organisms; Bacteria; Terrabacteria group; Actinobacteria; Actinomycetia; Corynebacteriales; Mycobacteriaceae; Mycobacterium; Mycobacterium tuberculosis complex; Mycobacterium tuberculosis;	NCBI: 1773	4144755	94.61	0.00	Yes
NCBI Assembly	GCA_004106335.1	NCBI: cellular organisms; Bacteria; Terrabacteria group; Actinobacteria; Actinomycetia; Corynebacteriales; Mycobacteriaceae; Mycobacterium; Mycobacterium tuberculosis complex; Mycobacterium tuberculosis;	NCBI: 1773	4407746	99.61	0.00	Yes
NCBI Assembly	GCA_004111425.1	NCBI: cellular organisms; Bacteria; Terrabacteria group; Actinobacteria; Actinomycetia; Corynebacteriales; Mycobacteriaceae; Mycobacterium; Mycobacterium tuberculosis complex; Mycobacterium tuberculosis;	NCBI: 1773	4388920	99.94	0.00	Yes
NCBI Assembly	GCA_003383375.1	NCBI: cellular organisms; Bacteria; Terrabacteria group; Actinobacteria; Actinomycetia; Corynebacteriales; Mycobacteriaceae; Mycobacterium; Mycobacterium tuberculosis complex; Mycobacterium tuberculosis;	NCBI: 1773	4382789	99.94	0.00	Yes
NCBI Assembly	GCA_003395815.1	NCBI: cellular organisms; Bacteria; Terrabacteria group; Actinobacteria; Actinomycetia; Corynebacteriales; Mycobacteriaceae; Mycobacterium; Mycobacterium tuberculosis complex; Mycobacterium tuberculosis;	NCBI: 1773	4351039	99.86	0.00	Yes
NCBI Assembly	GCA_003395175.1	NCBI: cellular organisms; Bacteria; Terrabacteria group; Actinobacteria; Actinomycetia; Corynebacteriales; Mycobacteriaceae; Mycobacterium; Mycobacterium tuberculosis complex; Mycobacterium tuberculosis;	NCBI: 1773	4301538	99.86	0.00	Yes
NCBI Assembly	GCA_003396465.1	NCBI: cellular organisms; Bacteria; Terrabacteria group; Actinobacteria; Actinomycetia; Corynebacteriales; Mycobacteriaceae; Mycobacterium; Mycobacterium tuberculosis complex; Mycobacterium tuberculosis;	NCBI: 1773	4371532	99.94	0.00	Yes
NCBI Assembly	GCA_003398415.1	NCBI: cellular organisms; Bacteria; Terrabacteria group; Actinobacteria; Actinomycetia; Corynebacteriales; Mycobacteriaceae; Mycobacterium; Mycobacterium tuberculosis complex; Mycobacterium tuberculosis;	NCBI: 1773	4327531	99.86	0.00	Yes
NCBI Assembly	GCA_003394245.1	NCBI: cellular organisms; Bacteria; Terrabacteria group; Actinobacteria; Actinomycetia; Corynebacteriales; Mycobacteriaceae; Mycobacterium; Mycobacterium tuberculosis complex; Mycobacterium tuberculosis;	NCBI: 1773	4296337	99.50	0.00	Yes
NCBI Assembly	GCA_003394885.1	NCBI: cellular organisms; Bacteria; Terrabacteria group; Actinobacteria; Actinomycetia; Corynebacteriales; Mycobacteriaceae; Mycobacterium; Mycobacterium tuberculosis complex; Mycobacterium tuberculosis;	NCBI: 1773	4353591	99.86	0.00	Yes
NCBI Assembly	GCA_003396765.1	NCBI: cellular organisms; Bacteria; Terrabacteria group; Actinobacteria; Actinomycetia; Corynebacteriales; Mycobacteriaceae; Mycobacterium; Mycobacterium tuberculosis complex; Mycobacterium tuberculosis;	NCBI: 1773	4373373	99.94	0.00	Yes
NCBI Assembly	GCA_004104775.1	NCBI: cellular organisms; Bacteria; Terrabacteria group; Actinobacteria; Actinomycetia; Corynebacteriales; Mycobacteriaceae; Mycobacterium; Mycobacterium tuberculosis complex; Mycobacterium tuberculosis;	NCBI: 1773	4420876	99.94	0.00	Yes
NCBI Assembly	GCA_004106365.1	NCBI: cellular organisms; Bacteria; Terrabacteria group; Actinobacteria; Actinomycetia; Corynebacteriales; Mycobacteriaceae; Mycobacterium; Mycobacterium tuberculosis complex; Mycobacterium tuberculosis;	NCBI: 1773	4382991	99.61	0.00	Yes
NCBI Assembly	GCA_004109845.1	NCBI: cellular organisms; Bacteria; Terrabacteria group; Actinobacteria; Actinomycetia; Corynebacteriales; Mycobacteriaceae; Mycobacterium; Mycobacterium tuberculosis complex; Mycobacterium tuberculosis;	NCBI: 1773	4394635	99.61	0.00	Yes
NCBI Assembly	GCA_004111435.1	NCBI: cellular organisms; Bacteria; Terrabacteria group; Actinobacteria; Actinomycetia; Corynebacteriales; Mycobacteriaceae; Mycobacterium; Mycobacterium tuberculosis complex; Mycobacterium tuberculosis;	NCBI: 1773	4373917	99.94	0.00	Yes
NCBI Assembly	GCA_003383385.1	NCBI: cellular organisms; Bacteria; Terrabacteria group; Actinobacteria; Actinomycetia; Corynebacteriales; Mycobacteriaceae; Mycobacterium; Mycobacterium tuberculosis complex; Mycobacterium tuberculosis;	NCBI: 1773	4390527	99.94	0.00	Yes
NCBI Assembly	GCA_003395195.1	NCBI: cellular organisms; Bacteria; Terrabacteria group; Actinobacteria; Actinomycetia; Corynebacteriales; Mycobacteriaceae; Mycobacterium; Mycobacterium tuberculosis complex; Mycobacterium tuberculosis;	NCBI: 1773	4320517	99.69	0.00	Yes

NCBI Assembly	GCA_003395825.1	NCBI: cellular organisms; Bacteria; Terrabacteria group; Actinobacteria; Actinomycetia; Corynebacteriales; Mycobacteriaceae; Mycobacterium; Mycobacterium tuberculosis complex; Mycobacterium tuberculosis;	NCBI: 1773	4359567	99.94	0.67	Yes
NCBI Assembly	GCA_003396475.1	NCBI: cellular organisms; Bacteria; Terrabacteria group; Actinobacteria; Actinomycetia; Corynebacteriales; Mycobacteriaceae; Mycobacterium; Mycobacterium tuberculosis complex; Mycobacterium tuberculosis;	NCBI: 1773	4349970	99.69	0.00	Yes
NCBI Assembly	GCA_003398435.1	NCBI: cellular organisms; Bacteria; Terrabacteria group; Actinobacteria; Actinomycetia; Corynebacteriales; Mycobacteriaceae; Mycobacterium; Mycobacterium tuberculosis complex; Mycobacterium tuberculosis;	NCBI: 1773	4171981	97.85	1.91	Yes
NCBI Assembly	GCA_003394265.1	NCBI: cellular organisms; Bacteria; Terrabacteria group; Actinobacteria; Actinomycetia; Corynebacteriales; Mycobacteriaceae; Mycobacterium; Mycobacterium tuberculosis complex; Mycobacterium tuberculosis;	NCBI: 1773	4351320	99.83	0.09	Yes
NCBI Assembly	GCA_003394905.1	NCBI: cellular organisms; Bacteria; Terrabacteria group; Actinobacteria; Actinomycetia; Corynebacteriales; Mycobacteriaceae; Mycobacterium; Mycobacterium tuberculosis complex; Mycobacterium tuberculosis;	NCBI: 1773	4300642	99.86	0.07	Yes
NCBI Assembly	GCA_003396785.1	NCBI: cellular organisms; Bacteria; Terrabacteria group; Actinobacteria; Actinomycetia; Corynebacteriales; Mycobacteriaceae; Mycobacterium; Mycobacterium tuberculosis complex; Mycobacterium tuberculosis;	NCBI: 1773	4350477	99.86	0.00	Yes
NCBI Assembly	GCA_004104785.1	NCBI: cellular organisms; Bacteria; Terrabacteria group; Actinobacteria; Actinomycetia; Corynebacteriales; Mycobacteriaceae; Mycobacterium; Mycobacterium tuberculosis complex; Mycobacterium tuberculosis;	NCBI: 1773	4385503	99.94	0.00	Yes
NCBI Assembly	GCA_004109855.1	NCBI: cellular organisms; Bacteria; Terrabacteria group; Actinobacteria; Actinomycetia; Corynebacteriales; Mycobacteriaceae; Mycobacterium; Mycobacterium tuberculosis complex; Mycobacterium tuberculosis;	NCBI: 1773	4385818	99.94	0.00	Yes
NCBI Assembly	GCA_004106375.1	NCBI: cellular organisms; Bacteria; Terrabacteria group; Actinobacteria; Actinomycetia; Corynebacteriales; Mycobacteriaceae; Mycobacterium; Mycobacterium tuberculosis complex; Mycobacterium tuberculosis;	NCBI: 1773	4402342	99.94	0.00	Yes
NCBI Assembly	GCA_004111455.1	NCBI: cellular organisms; Bacteria; Terrabacteria group; Actinobacteria; Actinomycetia; Corynebacteriales; Mycobacteriaceae; Mycobacterium; Mycobacterium tuberculosis complex; Mycobacterium tuberculosis;	NCBI: 1773	4346595	99.94	0.00	Yes
NCBI Assembly	GCA_003383395.1	NCBI: cellular organisms; Bacteria; Terrabacteria group; Actinobacteria; Actinomycetia; Corynebacteriales; Mycobacteriaceae; Mycobacterium; Mycobacterium tuberculosis complex; Mycobacterium tuberculosis;	NCBI: 1773	4694425	99.94	0.51	Yes
NCBI Assembly	GCA_003395225.1	NCBI: cellular organisms; Bacteria; Terrabacteria group; Actinobacteria; Actinomycetia; Corynebacteriales; Mycobacteriaceae; Mycobacterium; Mycobacterium tuberculosis complex; Mycobacterium tuberculosis;	NCBI: 1773	4322644	99.86	0.11	Yes
NCBI Assembly	GCA_003394305.1	NCBI: cellular organisms; Bacteria; Terrabacteria group; Actinobacteria; Actinomycetia; Corynebacteriales; Mycobacteriaceae; Mycobacterium; Mycobacterium tuberculosis complex; Mycobacterium tuberculosis;	NCBI: 1773	4351844	99.86	0.00	Yes
NCBI Assembly	GCA_003395835.1	NCBI: cellular organisms; Bacteria; Terrabacteria group; Actinobacteria; Actinomycetia; Corynebacteriales; Mycobacteriaceae; Mycobacterium; Mycobacterium tuberculosis complex; Mycobacterium tuberculosis;	NCBI: 1773	4351499	99.78	0.00	Yes
NCBI Assembly	GCA_003396485.1	NCBI: cellular organisms; Bacteria; Terrabacteria group; Actinobacteria; Actinomycetia; Corynebacteriales; Mycobacteriaceae; Mycobacterium; Mycobacterium tuberculosis complex; Mycobacterium tuberculosis;	NCBI: 1773	4369520	99.94	0.00	Yes
NCBI Assembly	GCA_003398445.1	NCBI: cellular organisms; Bacteria; Terrabacteria group; Actinobacteria; Actinomycetia; Corynebacteriales; Mycobacteriaceae; Mycobacterium; Mycobacterium tuberculosis complex; Mycobacterium tuberculosis;	NCBI: 1773	4239087	99.61	0.04	Yes
NCBI Assembly	GCA_003396825.1	NCBI: cellular organisms; Bacteria; Terrabacteria group; Actinobacteria; Actinomycetia; Corynebacteriales; Mycobacteriaceae; Mycobacterium; Mycobacterium tuberculosis complex; Mycobacterium tuberculosis;	NCBI: 1773	4351149	99.86	0.00	Yes
NCBI Assembly	GCA_004108645.1	NCBI: cellular organisms; Bacteria; Terrabacteria group; Actinobacteria; Actinomycetia; Corynebacteriales; Mycobacteriaceae; Mycobacterium; Mycobacterium tuberculosis complex; Mycobacterium tuberculosis;	NCBI: 1773	4382633	99.94	0.00	Yes

NCBI Assembly	GCA_004104795.1	NCBI: cellular organisms; Bacteria; Terrabacteria group; Actinobacteria; Actinomycetia; Corynebacteriales; Mycobacteriaceae; Mycobacterium; Mycobacterium tuberculosis complex; Mycobacterium tuberculosis;	NCBI: 1773	4388010	99.94	0.00	Yes
NCBI Assembly	GCA_004109865.1	NCBI: cellular organisms; Bacteria; Terrabacteria group; Actinobacteria; Actinomycetia; Corynebacteriales; Mycobacteriaceae; Mycobacterium; Mycobacterium tuberculosis complex; Mycobacterium tuberculosis;	NCBI: 1773	4367994	99.94	0.00	Yes
NCBI Assembly	GCA_004106405.1	NCBI: cellular organisms; Bacteria; Terrabacteria group; Actinobacteria; Actinomycetia; Corynebacteriales; Mycobacteriaceae; Mycobacterium; Mycobacterium tuberculosis complex; Mycobacterium tuberculosis;	NCBI: 1773	4385155	99.61	0.00	Yes
NCBI Assembly	GCA_004111465.1	NCBI: cellular organisms; Bacteria; Terrabacteria group; Actinobacteria; Actinomycetia; Corynebacteriales; Mycobacteriaceae; Mycobacterium; Mycobacterium tuberculosis complex; Mycobacterium tuberculosis;	NCBI: 1773	4382934	99.94	0.00	Yes
NCBI Assembly	GCA_003383415.1	NCBI: cellular organisms; Bacteria; Terrabacteria group; Actinobacteria; Actinomycetia; Corynebacteriales; Mycobacteriaceae; Mycobacterium; Mycobacterium tuberculosis complex; Mycobacterium tuberculosis;	NCBI: 1773	4361524	99.94	0.00	Yes
NCBI Assembly	GCA_003395235.1	NCBI: cellular organisms; Bacteria; Terrabacteria group; Actinobacteria; Actinomycetia; Corynebacteriales; Mycobacteriaceae; Mycobacterium; Mycobacterium tuberculosis complex; Mycobacterium tuberculosis;	NCBI: 1773	4300546	99.86	0.00	Yes
NCBI Assembly	GCA_003394315.1	NCBI: cellular organisms; Bacteria; Terrabacteria group; Actinobacteria; Actinomycetia; Corynebacteriales; Mycobacteriaceae; Mycobacterium; Mycobacterium tuberculosis complex; Mycobacterium tuberculosis;	NCBI: 1773	4328079	99.86	0.00	Yes
NCBI Assembly	GCA_003395885.1	NCBI: cellular organisms; Bacteria; Terrabacteria group; Actinobacteria; Actinomycetia; Corynebacteriales; Mycobacteriaceae; Mycobacterium; Mycobacterium tuberculosis complex; Mycobacterium tuberculosis;	NCBI: 1773	4355721	99.86	0.67	Yes
NCBI Assembly	GCA_003397855.1	NCBI: cellular organisms; Bacteria; Terrabacteria group; Actinobacteria; Actinomycetia; Corynebacteriales; Mycobacteriaceae; Mycobacterium; Mycobacterium tuberculosis complex; Mycobacterium tuberculosis;	NCBI: 1773	4349227	99.86	0.00	Yes
NCBI Assembly	GCA_004112435.1	NCBI: cellular organisms; Bacteria; Terrabacteria group; Actinobacteria; Actinomycetia; Corynebacteriales; Mycobacteriaceae; Mycobacterium; Mycobacterium tuberculosis complex; Mycobacterium tuberculosis;	NCBI: 1773	4387812	99.94	0.00	Yes
NCBI Assembly	GCA_003396845.1	NCBI: cellular organisms; Bacteria; Terrabacteria group; Actinobacteria; Actinomycetia; Corynebacteriales; Mycobacteriaceae; Mycobacterium; Mycobacterium tuberculosis complex; Mycobacterium tuberculosis;	NCBI: 1773	4352958	99.69	0.00	Yes
NCBI Assembly	GCA_004108665.1	NCBI: cellular organisms; Bacteria; Terrabacteria group; Actinobacteria; Actinomycetia; Corynebacteriales; Mycobacteriaceae; Mycobacterium; Mycobacterium tuberculosis complex; Mycobacterium tuberculosis;	NCBI: 1773	4382100	99.61	0.00	Yes
NCBI Assembly	GCA_004104815.1	NCBI: cellular organisms; Bacteria; Terrabacteria group; Actinobacteria; Actinomycetia; Corynebacteriales; Mycobacteriaceae; Mycobacterium; Mycobacterium tuberculosis complex; Mycobacterium tuberculosis;	NCBI: 1773	4396257	99.94	0.00	Yes
NCBI Assembly	GCA_004106415.1	NCBI: cellular organisms; Bacteria; Terrabacteria group; Actinobacteria; Actinomycetia; Corynebacteriales; Mycobacteriaceae; Mycobacterium; Mycobacterium tuberculosis complex; Mycobacterium tuberculosis;	NCBI: 1773	4391977	99.94	0.00	Yes
NCBI Assembly	GCA_004111505.1	NCBI: cellular organisms; Bacteria; Terrabacteria group; Actinobacteria; Actinomycetia; Corynebacteriales; Mycobacteriaceae; Mycobacterium; Mycobacterium tuberculosis complex; Mycobacterium tuberculosis;	NCBI: 1773	4388554	99.94	0.00	Yes
NCBI Assembly	GCA_003383465.1	NCBI: cellular organisms; Bacteria; Terrabacteria group; Actinobacteria; Actinomycetia; Corynebacteriales; Mycobacteriaceae; Mycobacterium; Mycobacterium tuberculosis complex; Mycobacterium tuberculosis;	NCBI: 1773	4383602	99.94	0.00	Yes
NCBI Assembly	GCA_004109925.1	NCBI: cellular organisms; Bacteria; Terrabacteria group; Actinobacteria; Actinomycetia; Corynebacteriales; Mycobacteriaceae; Mycobacterium; Mycobacterium tuberculosis complex; Mycobacterium tuberculosis;	NCBI: 1773	4359768	99.94	0.00	Yes
NCBI Assembly	GCA_003394335.1	NCBI: cellular organisms; Bacteria; Terrabacteria group; Actinobacteria; Actinomycetia; Corynebacteriales; Mycobacteriaceae; Mycobacterium; Mycobacterium tuberculosis complex; Mycobacterium tuberculosis;	NCBI: 1773	4371335	99.53	0.00	Yes

NCBI Assembly	GCA_003395265.1	NCBI: cellular organisms; Bacteria; Terrabacteria group; Actinobacteria; Actinomycetia; Corynebacteriales; Mycobacteriaceae; Mycobacterium; Mycobacterium tuberculosis complex; Mycobacterium tuberculosis;	NCBI: 1773	4365284	99.86	0.11	Yes
NCBI Assembly	GCA_003395905.1	NCBI: cellular organisms; Bacteria; Terrabacteria group; Actinobacteria; Actinomycetia; Corynebacteriales; Mycobacteriaceae; Mycobacterium; Mycobacterium tuberculosis complex; Mycobacterium tuberculosis;	NCBI: 1773	4349408	99.86	0.00	Yes
NCBI Assembly	GCA_003397885.1	NCBI: cellular organisms; Bacteria; Terrabacteria group; Actinobacteria; Actinomycetia; Corynebacteriales; Mycobacteriaceae; Mycobacterium; Mycobacterium tuberculosis complex; Mycobacterium tuberculosis;	NCBI: 1773	4235015	99.53	0.33	Yes
NCBI Assembly	GCA_004112455.1	NCBI: cellular organisms; Bacteria; Terrabacteria group; Actinobacteria; Actinomycetia; Corynebacteriales; Mycobacteriaceae; Mycobacterium; Mycobacterium tuberculosis complex; Mycobacterium tuberculosis;	NCBI: 1773	4378752	99.94	0.00	Yes
NCBI Assembly	GCA_003396855.1	NCBI: cellular organisms; Bacteria; Terrabacteria group; Actinobacteria; Actinomycetia; Corynebacteriales; Mycobacteriaceae; Mycobacterium; Mycobacterium tuberculosis complex; Mycobacterium tuberculosis;	NCBI: 1773	4342923	99.86	0.11	Yes
NCBI Assembly	GCA_004108675.1	NCBI: cellular organisms; Bacteria; Terrabacteria group; Actinobacteria; Actinomycetia; Corynebacteriales; Mycobacteriaceae; Mycobacterium; Mycobacterium tuberculosis complex; Mycobacterium tuberculosis;	NCBI: 1773	4395100	99.94	0.00	Yes
NCBI Assembly	GCA_003782105.1	NCBI: cellular organisms; Bacteria; Terrabacteria group; Actinobacteria; Actinomycetia; Corynebacteriales; Mycobacteriaceae; Mycobacterium; Mycobacterium tuberculosis complex; Mycobacterium tuberculosis;	NCBI: 1773	3618156	97.87	0.00	Yes
NCBI Assembly	GCA_004111525.1	NCBI: cellular organisms; Bacteria; Terrabacteria group; Actinobacteria; Actinomycetia; Corynebacteriales; Mycobacteriaceae; Mycobacterium; Mycobacterium tuberculosis complex; Mycobacterium tuberculosis;	NCBI: 1773	4390462	99.94	0.00	Yes
NCBI Assembly	GCA_003383475.1	NCBI: cellular organisms; Bacteria; Terrabacteria group; Actinobacteria; Actinomycetia; Corynebacteriales; Mycobacteriaceae; Mycobacterium; Mycobacterium tuberculosis complex; Mycobacterium tuberculosis;	NCBI: 1773	4361703	99.94	0.00	Yes
NCBI Assembly	GCA_004106425.1	NCBI: cellular organisms; Bacteria; Terrabacteria group; Actinobacteria; Actinomycetia; Corynebacteriales; Mycobacteriaceae; Mycobacterium; Mycobacterium tuberculosis complex; Mycobacterium tuberculosis;	NCBI: 1773	4406101	99.94	0.00	Yes
NCBI Assembly	GCA_003395285.1	NCBI: cellular organisms; Bacteria; Terrabacteria group; Actinobacteria; Actinomycetia; Corynebacteriales; Mycobacteriaceae; Mycobacterium; Mycobacterium tuberculosis complex; Mycobacterium tuberculosis;	NCBI: 1773	4354476	99.86	0.00	Yes
NCBI Assembly	GCA_003394365.1	NCBI: cellular organisms; Bacteria; Terrabacteria group; Actinobacteria; Actinomycetia; Corynebacteriales; Mycobacteriaceae; Mycobacterium; Mycobacterium tuberculosis complex; Mycobacterium tuberculosis;	NCBI: 1773	4324183	99.94	0.00	Yes
NCBI Assembly	GCA_004109255.1	NCBI: cellular organisms; Bacteria; Terrabacteria group; Actinobacteria; Actinomycetia; Corynebacteriales; Mycobacteriaceae; Mycobacterium; Mycobacterium tuberculosis complex; Mycobacterium tuberculosis;	NCBI: 1773	4385547	99.61	0.00	Yes
NCBI Assembly	GCA_003395925.1	NCBI: cellular organisms; Bacteria; Terrabacteria group; Actinobacteria; Actinomycetia; Corynebacteriales; Mycobacteriaceae; Mycobacterium; Mycobacterium tuberculosis complex; Mycobacterium tuberculosis;	NCBI: 1773	4340679	99.86	0.04	Yes
NCBI Assembly	GCA_003397895.1	NCBI: cellular organisms; Bacteria; Terrabacteria group; Actinobacteria; Actinomycetia; Corynebacteriales; Mycobacteriaceae; Mycobacterium; Mycobacterium tuberculosis complex; Mycobacterium tuberculosis;	NCBI: 1773	4351730	99.86	0.06	Yes
NCBI Assembly	GCA_004112465.1	NCBI: cellular organisms; Bacteria; Terrabacteria group; Actinobacteria; Actinomycetia; Corynebacteriales; Mycobacteriaceae; Mycobacterium; Mycobacterium tuberculosis complex; Mycobacterium tuberculosis;	NCBI: 1773	4350474	99.94	0.00	Yes
NCBI Assembly	GCA_003396875.1	NCBI: cellular organisms; Bacteria; Terrabacteria group; Actinobacteria; Actinomycetia; Corynebacteriales; Mycobacteriaceae; Mycobacterium; Mycobacterium tuberculosis complex; Mycobacterium tuberculosis;	NCBI: 1773	4414791	99.86	0.44	Yes
NCBI Assembly	GCA_003782145.1	NCBI: cellular organisms; Bacteria; Terrabacteria group; Actinobacteria; Actinomycetia; Corynebacteriales; Mycobacteriaceae; Mycobacterium; Mycobacterium tuberculosis complex; Mycobacterium tuberculosis;	NCBI: 1773	3585501	97.98	0.00	Yes

NCBI Assembly	GCA_004108685.1	NCBI: cellular organisms; Bacteria; Terrabacteria group; Actinobacteria; Actinomycetia; Corynebacteriales; Mycobacteriaceae; Mycobacterium; Mycobacterium tuberculosis complex; Mycobacterium tuberculosis;	NCBI: 1773	4393461	99.94	0.00	Yes
NCBI Assembly	GCA_003383495.1	NCBI: cellular organisms; Bacteria; Terrabacteria group; Actinobacteria; Actinomycetia; Corynebacteriales; Mycobacteriaceae; Mycobacterium; Mycobacterium tuberculosis complex; Mycobacterium tuberculosis;	NCBI: 1773	4376826	99.94	0.33	Yes
NCBI Assembly	GCA_004111535.1	NCBI: cellular organisms; Bacteria; Terrabacteria group; Actinobacteria; Actinomycetia; Corynebacteriales; Mycobacteriaceae; Mycobacterium; Mycobacterium tuberculosis complex; Mycobacterium tuberculosis;	NCBI: 1773	4392895	99.94	0.00	Yes
NCBI Assembly	GCA_003395305.1	NCBI: cellular organisms; Bacteria; Terrabacteria group; Actinobacteria; Actinomycetia; Corynebacteriales; Mycobacteriaceae; Mycobacterium; Mycobacterium tuberculosis complex; Mycobacterium tuberculosis;	NCBI: 1773	4310901	99.69	0.00	Yes
NCBI Assembly	GCA_004106465.1	NCBI: cellular organisms; Bacteria; Terrabacteria group; Actinobacteria; Actinomycetia; Corynebacteriales; Mycobacteriaceae; Mycobacterium; Mycobacterium tuberculosis complex; Mycobacterium tuberculosis;	NCBI: 1773	4402840	99.61	0.00	Yes
NCBI Assembly	GCA_003394385.1	NCBI: cellular organisms; Bacteria; Terrabacteria group; Actinobacteria; Actinomycetia; Corynebacteriales; Mycobacteriaceae; Mycobacterium; Mycobacterium tuberculosis complex; Mycobacterium tuberculosis;	NCBI: 1773	4321834	99.86	0.11	Yes
NCBI Assembly	GCA_004109265.1	NCBI: cellular organisms; Bacteria; Terrabacteria group; Actinobacteria; Actinomycetia; Corynebacteriales; Mycobacteriaceae; Mycobacterium; Mycobacterium tuberculosis complex; Mycobacterium tuberculosis;	NCBI: 1773	4394559	99.94	0.00	Yes
NCBI Assembly	GCA_003395945.1	NCBI: cellular organisms; Bacteria; Terrabacteria group; Actinobacteria; Actinomycetia; Corynebacteriales; Mycobacteriaceae; Mycobacterium; Mycobacterium tuberculosis complex; Mycobacterium tuberculosis;	NCBI: 1773	4368411	99.86	0.00	Yes
NCBI Assembly	GCA_003397905.1	NCBI: cellular organisms; Bacteria; Terrabacteria group; Actinobacteria; Actinomycetia; Corynebacteriales; Mycobacteriaceae; Mycobacterium; Mycobacterium tuberculosis complex; Mycobacterium tuberculosis;	NCBI: 1773	4313302	99.83	0.00	Yes
NCBI Assembly	GCA_003396905.1	NCBI: cellular organisms; Bacteria; Terrabacteria group; Actinobacteria; Actinomycetia; Corynebacteriales; Mycobacteriaceae; Mycobacterium; Mycobacterium tuberculosis complex; Mycobacterium tuberculosis;	NCBI: 1773	4284662	99.36	0.87	Yes
NCBI Assembly	GCA_004112485.1	NCBI: cellular organisms; Bacteria; Terrabacteria group; Actinobacteria; Actinomycetia; Corynebacteriales; Mycobacteriaceae; Mycobacterium; Mycobacterium tuberculosis complex; Mycobacterium tuberculosis;	NCBI: 1773	4398348	99.94	0.00	Yes
NCBI Assembly	GCA_003782155.1	NCBI: cellular organisms; Bacteria; Terrabacteria group; Actinobacteria; Actinomycetia; Corynebacteriales; Mycobacteriaceae; Mycobacterium; Mycobacterium tuberculosis complex; Mycobacterium tuberculosis;	NCBI: 1773	3612264	98.04	0.00	Yes
NCBI Assembly	GCA_004108695.1	NCBI: cellular organisms; Bacteria; Terrabacteria group; Actinobacteria; Actinomycetia; Corynebacteriales; Mycobacteriaceae; Mycobacterium; Mycobacterium tuberculosis complex; Mycobacterium tuberculosis;	NCBI: 1773	4399230	99.94	0.00	Yes
NCBI Assembly	GCA_003383535.1	NCBI: cellular organisms; Bacteria; Terrabacteria group; Actinobacteria; Actinomycetia; Corynebacteriales; Mycobacteriaceae; Mycobacterium; Mycobacterium tuberculosis complex; Mycobacterium tuberculosis;	NCBI: 1773	4372932	99.94	0.33	Yes
NCBI Assembly	GCA_003395315.1	NCBI: cellular organisms; Bacteria; Terrabacteria group; Actinobacteria; Actinomycetia; Corynebacteriales; Mycobacteriaceae; Mycobacterium; Mycobacterium tuberculosis complex; Mycobacterium tuberculosis;	NCBI: 1773	4337164	99.86	0.00	Yes
NCBI Assembly	GCA_003394395.1	NCBI: cellular organisms; Bacteria; Terrabacteria group; Actinobacteria; Actinomycetia; Corynebacteriales; Mycobacteriaceae; Mycobacterium; Mycobacterium tuberculosis complex; Mycobacterium tuberculosis;	NCBI: 1773	4367010	99.86	0.11	Yes
NCBI Assembly	GCA_004111555.1	NCBI: cellular organisms; Bacteria; Terrabacteria group; Actinobacteria; Actinomycetia; Corynebacteriales; Mycobacteriaceae; Mycobacterium; Mycobacterium tuberculosis complex; Mycobacterium tuberculosis;	NCBI: 1773	4382368	99.94	0.00	Yes
NCBI Assembly	GCA_004106485.1	NCBI: cellular organisms; Bacteria; Terrabacteria group; Actinobacteria; Actinomycetia; Corynebacteriales; Mycobacteriaceae; Mycobacterium; Mycobacterium tuberculosis complex; Mycobacterium tuberculosis;	NCBI: 1773	4397190	99.94	0.00	Yes

NCBI Assembly	GCA_003395955.1	NCBI: cellular organisms; Bacteria; Terrabacteria group; Actinobacteria; Actinomycetia; Corynebacteriales; Mycobacteriaceae; Mycobacterium; Mycobacterium tuberculosis complex; Mycobacterium tuberculosis;	NCBI: 1773	4343116	99.86	0.00	Yes
NCBI Assembly	GCA_003397925.1	NCBI: cellular organisms; Bacteria; Terrabacteria group; Actinobacteria; Actinomycetia; Corynebacteriales; Mycobacteriaceae; Mycobacterium; Mycobacterium tuberculosis complex; Mycobacterium tuberculosis;	NCBI: 1773	4210626	98.56	1.08	Yes
NCBI Assembly	GCA_004109285.1	NCBI: cellular organisms; Bacteria; Terrabacteria group; Actinobacteria; Actinomycetia; Corynebacteriales; Mycobacteriaceae; Mycobacterium; Mycobacterium tuberculosis complex; Mycobacterium tuberculosis;	NCBI: 1773	4378987	99.94	0.00	Yes
NCBI Assembly	GCA_003396915.1	NCBI: cellular organisms; Bacteria; Terrabacteria group; Actinobacteria; Actinomycetia; Corynebacteriales; Mycobacteriaceae; Mycobacterium; Mycobacterium tuberculosis complex; Mycobacterium tuberculosis;	NCBI: 1773	4362372	99.69	0.33	Yes
NCBI Assembly	GCA_004112525.1	NCBI: cellular organisms; Bacteria; Terrabacteria group; Actinobacteria; Actinomycetia; Corynebacteriales; Mycobacteriaceae; Mycobacterium; Mycobacterium tuberculosis complex; Mycobacterium tuberculosis;	NCBI: 1773	4383720	99.94	0.07	Yes
NCBI Assembly	GCA_003815795.1	NCBI: cellular organisms; Bacteria; Terrabacteria group; Actinobacteria; Actinomycetia; Corynebacteriales; Mycobacteriaceae; Mycobacterium; Mycobacterium avium complex (MAC); Mycobacterium avium; Mycobacterium avium subsp. paratuberculosis;	NCBI: 1770	4836546	99.94	0.33	Yes
NCBI Assembly	GCA_003390495.1	NCBI: cellular organisms; Bacteria; Terrabacteria group; Actinobacteria; Actinomycetia; Corynebacteriales; Mycobacteriaceae; Mycobacteroides; Mycobacteroides chelonae; Mycobacteroides chelonae subsp. gwanakae;	NCBI: 2480908	5106636	99.50	0.36	Yes
NCBI Assembly	GCA_004108745.1	NCBI: cellular organisms; Bacteria; Terrabacteria group; Actinobacteria; Actinomycetia; Corynebacteriales; Mycobacteriaceae; Mycobacterium; Mycobacterium tuberculosis complex; Mycobacterium tuberculosis;	NCBI: 1773	4393370	99.94	0.00	Yes
NCBI Assembly	GCA_003395345.1	NCBI: cellular organisms; Bacteria; Terrabacteria group; Actinobacteria; Actinomycetia; Corynebacteriales; Mycobacteriaceae; Mycobacterium; Mycobacterium tuberculosis complex; Mycobacterium tuberculosis;	NCBI: 1773	4304243	99.86	0.11	Yes
NCBI Assembly	GCA_003394415.1	NCBI: cellular organisms; Bacteria; Terrabacteria group; Actinobacteria; Actinomycetia; Corynebacteriales; Mycobacteriaceae; Mycobacterium; Mycobacterium tuberculosis complex; Mycobacterium tuberculosis;	NCBI: 1773	4366888	99.86	0.00	Yes
NCBI Assembly	GCA_003395985.1	NCBI: cellular organisms; Bacteria; Terrabacteria group; Actinobacteria; Actinomycetia; Corynebacteriales; Mycobacteriaceae; Mycobacterium; Mycobacterium tuberculosis complex; Mycobacterium tuberculosis;	NCBI: 1773	4363605	99.86	0.00	Yes
NCBI Assembly	GCA_004106495.1	NCBI: cellular organisms; Bacteria; Terrabacteria group; Actinobacteria; Actinomycetia; Corynebacteriales; Mycobacteriaceae; Mycobacterium; Mycobacterium tuberculosis complex; Mycobacterium tuberculosis;	NCBI: 1773	4385845	99.94	0.00	Yes
NCBI Assembly	GCA_004111575.1	NCBI: cellular organisms; Bacteria; Terrabacteria group; Actinobacteria; Actinomycetia; Corynebacteriales; Mycobacteriaceae; Mycobacterium; Mycobacterium tuberculosis complex; Mycobacterium tuberculosis;	NCBI: 1773	4399596	99.94	0.00	Yes
NCBI Assembly	GCA_003397955.1	NCBI: cellular organisms; Bacteria; Terrabacteria group; Actinobacteria; Actinomycetia; Corynebacteriales; Mycobacteriaceae; Mycobacterium; Mycobacterium tuberculosis complex; Mycobacterium tuberculosis;	NCBI: 1773	4297974	99.53	0.00	Yes
NCBI Assembly	GCA_003396925.1	NCBI: cellular organisms; Bacteria; Terrabacteria group; Actinobacteria; Actinomycetia; Corynebacteriales; Mycobacteriaceae; Mycobacterium; Mycobacterium tuberculosis complex; Mycobacterium tuberculosis;	NCBI: 1773	4327632	99.86	1.00	Yes
NCBI Assembly	GCA_004109315.1	NCBI: cellular organisms; Bacteria; Terrabacteria group; Actinobacteria; Actinomycetia; Corynebacteriales; Mycobacteriaceae; Mycobacterium; Mycobacterium tuberculosis complex; Mycobacterium tuberculosis;	NCBI: 1773	4407740	99.94	0.00	Yes
NCBI Assembly	GCA_004112535.1	NCBI: cellular organisms; Bacteria; Terrabacteria group; Actinobacteria; Actinomycetia; Corynebacteriales; Mycobacteriaceae; Mycobacterium; Mycobacterium tuberculosis complex; Mycobacterium tuberculosis;	NCBI: 1773	4386096	99.94	0.00	Yes
NCBI Assembly	GCA_003391395.1	NCBI: cellular organisms; Bacteria; Terrabacteria group; Actinobacteria; Actinomycetia; Corynebacteriales; Mycobacteriaceae; Mycobacterium; Mycobacterium marinum;	NCBI: 1781	6453310	99.88	0.23	Yes

NCBI Assembly	GCA_003815815.1	NCBI: cellular organisms; Bacteria; Terrabacteria group; Actinobacteria; Actinomycetia; Corynebacteriales; Mycobacteriaceae; Mycobacterium; Mycobacterium avium complex (MAC); Mycobacterium avium; Mycobacterium avium subsp. paratuberculosis;	NCBI: 1770	4838766	99.94	0.33	Yes
NCBI Assembly	GCA_003395365.1	NCBI: cellular organisms; Bacteria; Terrabacteria group; Actinobacteria; Actinomycetia; Corynebacteriales; Mycobacteriaceae; Mycobacterium; Mycobacterium tuberculosis complex; Mycobacterium tuberculosis;	NCBI: 1773	4376400	99.86	0.00	Yes
NCBI Assembly	GCA_004108765.1	NCBI: cellular organisms; Bacteria; Terrabacteria group; Actinobacteria; Actinomycetia; Corynebacteriales; Mycobacteriaceae; Mycobacterium; Mycobacterium tuberculosis complex; Mycobacterium tuberculosis;	NCBI: 1773	4396257	99.94	0.00	Yes
NCBI Assembly	GCA_003394445.1	NCBI: cellular organisms; Bacteria; Terrabacteria group; Actinobacteria; Actinomycetia; Corynebacteriales; Mycobacteriaceae; Mycobacterium; Mycobacterium tuberculosis complex; Mycobacterium tuberculosis;	NCBI: 1773	4327870	99.86	0.00	Yes
NCBI Assembly	GCA_003395995.1	NCBI: cellular organisms; Bacteria; Terrabacteria group; Actinobacteria; Actinomycetia; Corynebacteriales; Mycobacteriaceae; Mycobacterium; Mycobacterium tuberculosis complex; Mycobacterium tuberculosis;	NCBI: 1773	4365582	99.86	0.00	Yes
NCBI Assembly	GCA_003397985.1	NCBI: cellular organisms; Bacteria; Terrabacteria group; Actinobacteria; Actinomycetia; Corynebacteriales; Mycobacteriaceae; Mycobacterium; Mycobacterium tuberculosis complex; Mycobacterium tuberculosis;	NCBI: 1773	4348651	99.86	0.00	Yes
NCBI Assembly	GCA_004106505.1	NCBI: cellular organisms; Bacteria; Terrabacteria group; Actinobacteria; Actinomycetia; Corynebacteriales; Mycobacteriaceae; Mycobacterium; Mycobacterium tuberculosis complex; Mycobacterium tuberculosis;	NCBI: 1773	4387316	99.94	0.00	Yes
NCBI Assembly	GCA_003396935.1	NCBI: cellular organisms; Bacteria; Terrabacteria group; Actinobacteria; Actinomycetia; Corynebacteriales; Mycobacteriaceae; Mycobacterium; Mycobacterium tuberculosis complex; Mycobacterium tuberculosis;	NCBI: 1773	4387888	99.86	0.33	Yes
NCBI Assembly	GCA_004111605.1	NCBI: cellular organisms; Bacteria; Terrabacteria group; Actinobacteria; Actinomycetia; Corynebacteriales; Mycobacteriaceae; Mycobacterium; Mycobacterium tuberculosis complex; Mycobacterium tuberculosis;	NCBI: 1773	4374211	99.94	0.00	Yes
NCBI Assembly	GCA_004109335.1	NCBI: cellular organisms; Bacteria; Terrabacteria group; Actinobacteria; Actinomycetia; Corynebacteriales; Mycobacteriaceae; Mycobacterium; Mycobacterium tuberculosis complex; Mycobacterium tuberculosis;	NCBI: 1773	4403379	99.94	0.00	Yes
NCBI Assembly	GCA_004112545.1	NCBI: cellular organisms; Bacteria; Terrabacteria group; Actinobacteria; Actinomycetia; Corynebacteriales; Mycobacteriaceae; Mycobacterium; Mycobacterium tuberculosis complex; Mycobacterium tuberculosis;	NCBI: 1773	4391419	99.94	0.00	Yes
NCBI Assembly	GCA_003391415.1	NCBI: cellular organisms; Bacteria; Terrabacteria group; Actinobacteria; Actinomycetia; Corynebacteriales; Mycobacteriaceae; Mycobacterium; Mycobacterium marinum;	NCBI: 1781	6467358	99.88	0.34	Yes
NCBI Assembly	GCA_003816035.1	NCBI: cellular organisms; Bacteria; Terrabacteria group; Actinobacteria; Actinomycetia; Corynebacteriales; Mycobacteriaceae; Mycobacterium; Mycobacterium avium complex (MAC); Mycobacterium avium; Mycobacterium avium subsp. paratuberculosis;	NCBI: 1770	4838649	99.94	0.33	Yes
NCBI Assembly	GCA_003395375.1	NCBI: cellular organisms; Bacteria; Terrabacteria group; Actinobacteria; Actinomycetia; Corynebacteriales; Mycobacteriaceae; Mycobacterium; Mycobacterium tuberculosis complex; Mycobacterium tuberculosis;	NCBI: 1773	4376617	99.86	0.00	Yes
NCBI Assembly	GCA_003394455.1	NCBI: cellular organisms; Bacteria; Terrabacteria group; Actinobacteria; Actinomycetia; Corynebacteriales; Mycobacteriaceae; Mycobacterium; Mycobacterium tuberculosis complex; Mycobacterium tuberculosis;	NCBI: 1773	4379171	99.94	0.00	Yes
NCBI Assembly	GCA_004108785.1	NCBI: cellular organisms; Bacteria; Terrabacteria group; Actinobacteria; Actinomycetia; Corynebacteriales; Mycobacteriaceae; Mycobacterium; Mycobacterium tuberculosis complex; Mycobacterium tuberculosis;	NCBI: 1773	4353993	99.94	0.00	Yes
NCBI Assembly	GCA_003396035.1	NCBI: cellular organisms; Bacteria; Terrabacteria group; Actinobacteria; Actinomycetia; Corynebacteriales; Mycobacteriaceae; Mycobacterium; Mycobacterium tuberculosis complex; Mycobacterium tuberculosis;	NCBI: 1773	4360182	99.86	0.00	Yes
NCBI Assembly	GCA_003397995.1	NCBI: cellular organisms; Bacteria; Terrabacteria group; Actinobacteria; Actinomycetia; Corynebacteriales; Mycobacteriaceae; Mycobacterium; Mycobacterium tuberculosis complex; Mycobacterium tuberculosis;	NCBI: 1773	4388674	99.86	0.00	Yes

NCBI Assembly	GCA_004106515.1	NCBI: cellular organisms; Bacteria; Terrabacteria group; Actinobacteria; Actinomycetia; Corynebacteriales; Mycobacteriaceae; Mycobacterium; Mycobacterium tuberculosis complex; Mycobacterium tuberculosis;	NCBI: 1773	4402016	99.94	0.00	Yes
NCBI Assembly	GCA_003396985.1	NCBI: cellular organisms; Bacteria; Terrabacteria group; Actinobacteria; Actinomycetia; Corynebacteriales; Mycobacteriaceae; Mycobacterium; Mycobacterium tuberculosis complex; Mycobacterium tuberculosis;	NCBI: 1773	4352574	99.94	0.00	Yes
NCBI Assembly	GCA_004111625.1	NCBI: cellular organisms; Bacteria; Terrabacteria group; Actinobacteria; Actinomycetia; Corynebacteriales; Mycobacteriaceae; Mycobacterium; Mycobacterium tuberculosis complex; Mycobacterium tuberculosis;	NCBI: 1773	4358923	99.61	0.00	Yes
NCBI Assembly	GCA_004109405.1	NCBI: cellular organisms; Bacteria; Terrabacteria group; Actinobacteria; Actinomycetia; Corynebacteriales; Mycobacteriaceae; Mycobacterium; Mycobacterium tuberculosis complex; Mycobacterium tuberculosis;	NCBI: 1773	4369879	99.94	0.00	Yes
NCBI Assembly	GCA_003391805.1	NCBI: cellular organisms; Bacteria; Terrabacteria group; Actinobacteria; Actinomycetia; Corynebacteriales; Mycobacteriaceae; Mycobacterium; Mycobacterium tuberculosis complex; Mycobacterium tuberculosis;	NCBI: 1773	4374478	99.86	0.00	Yes
NCBI Assembly	GCA_004112565.1	NCBI: cellular organisms; Bacteria; Terrabacteria group; Actinobacteria; Actinomycetia; Corynebacteriales; Mycobacteriaceae; Mycobacterium; Mycobacterium tuberculosis complex; Mycobacterium tuberculosis;	NCBI: 1773	4388770	99.94	0.00	Yes
NCBI Assembly	GCA_003395405.1	NCBI: cellular organisms; Bacteria; Terrabacteria group; Actinobacteria; Actinomycetia; Corynebacteriales; Mycobacteriaceae; Mycobacterium; Mycobacterium tuberculosis complex; Mycobacterium tuberculosis;	NCBI: 1773	4350210	99.86	0.11	Yes
NCBI Assembly	GCA_003851485.1	NCBI: cellular organisms; Bacteria; Terrabacteria group; Actinobacteria; Actinomycetia; Corynebacteriales; Mycobacteriaceae; Mycolicibacterium; unclassified Mycolicibacterium;	NCBI: 2185272	5773114	99.75	0.69	Yes
NCBI Assembly	GCA_003398025.1	NCBI: cellular organisms; Bacteria; Terrabacteria group; Actinobacteria; Actinomycetia; Corynebacteriales; Mycobacteriaceae; Mycobacterium; Mycobacterium tuberculosis complex; Mycobacterium tuberculosis;	NCBI: 1773	4257987	99.65	0.36	Yes
NCBI Assembly	GCA_003394465.1	NCBI: cellular organisms; Bacteria; Terrabacteria group; Actinobacteria; Actinomycetia; Corynebacteriales; Mycobacteriaceae; Mycobacterium; Mycobacterium tuberculosis complex; Mycobacterium tuberculosis;	NCBI: 1773	4351508	99.86	0.00	Yes
NCBI Assembly	GCA_004108795.1	NCBI: cellular organisms; Bacteria; Terrabacteria group; Actinobacteria; Actinomycetia; Corynebacteriales; Mycobacteriaceae; Mycobacterium; Mycobacterium tuberculosis complex; Mycobacterium tuberculosis;	NCBI: 1773	4388051	99.94	0.00	Yes
NCBI Assembly	GCA_003396995.1	NCBI: cellular organisms; Bacteria; Terrabacteria group; Actinobacteria; Actinomycetia; Corynebacteriales; Mycobacteriaceae; Mycobacterium; Mycobacterium tuberculosis complex; Mycobacterium tuberculosis;	NCBI: 1773	4427531	99.94	0.33	Yes
NCBI Assembly	GCA_003396065.1	NCBI: cellular organisms; Bacteria; Terrabacteria group; Actinobacteria; Actinomycetia; Corynebacteriales; Mycobacteriaceae; Mycobacterium; Mycobacterium tuberculosis complex; Mycobacterium tuberculosis;	NCBI: 1773	4376410	99.86	0.00	Yes
NCBI Assembly	GCA_004111635.1	NCBI: cellular organisms; Bacteria; Terrabacteria group; Actinobacteria; Actinomycetia; Corynebacteriales; Mycobacteriaceae; Mycobacterium; Mycobacterium tuberculosis complex; Mycobacterium tuberculosis;	NCBI: 1773	4354842	99.94	0.00	Yes
NCBI Assembly	GCA_004106565.1	NCBI: cellular organisms; Bacteria; Terrabacteria group; Actinobacteria; Actinomycetia; Corynebacteriales; Mycobacteriaceae; Mycobacterium; Mycobacterium tuberculosis complex; Mycobacterium tuberculosis;	NCBI: 1773	4398407	99.61	0.00	Yes
NCBI Assembly	GCA_004109415.1	NCBI: cellular organisms; Bacteria; Terrabacteria group; Actinobacteria; Actinomycetia; Corynebacteriales; Mycobacteriaceae; Mycobacterium; Mycobacterium tuberculosis complex; Mycobacterium tuberculosis;	NCBI: 1773	4375065	99.94	0.00	Yes
NCBI Assembly	GCA_003391945.1	NCBI: cellular organisms; Bacteria; Terrabacteria group; Actinobacteria; Actinomycetia; Corynebacteriales; Mycobacteriaceae; Mycobacterium; Mycobacterium tuberculosis complex; Mycobacterium tuberculosis;	NCBI: 1773	4320758	99.86	0.00	Yes
NCBI Assembly	GCA_004112575.1	NCBI: cellular organisms; Bacteria; Terrabacteria group; Actinobacteria; Actinomycetia; Corynebacteriales; Mycobacteriaceae; Mycobacterium; Mycobacterium tuberculosis complex; Mycobacterium tuberculosis;	NCBI: 1773	4392628	99.61	0.00	Yes

NCBI Assembly	GCA_003395425.1	NCBI: cellular organisms; Bacteria; Terrabacteria group; Actinobacteria; Actinomycetia; Corynebacteriales; Mycobacteriaceae; Mycobacterium; Mycobacterium tuberculosis complex; Mycobacterium tuberculosis;	NCBI: 1773	4360473	99.69	0.00	Yes
NCBI Assembly	GCA_003855255.1	NCBI: cellular organisms; Bacteria; Terrabacteria group; Actinobacteria; Actinomycetia; Corynebacteriales; Mycobacteriaceae; Mycolicibacterium; Mycolicibacterium nivoides;	NCBI: 2487344	6905961	99.81	1.36	Yes
NCBI Assembly	GCA_003394485.1	NCBI: cellular organisms; Bacteria; Terrabacteria group; Actinobacteria; Actinomycetia; Corynebacteriales; Mycobacteriaceae; Mycobacterium; Mycobacterium tuberculosis complex; Mycobacterium tuberculosis;	NCBI: 1773	4354367	99.86	0.00	Yes
NCBI Assembly	GCA_003398035.1	NCBI: cellular organisms; Bacteria; Terrabacteria group; Actinobacteria; Actinomycetia; Corynebacteriales; Mycobacteriaceae; Mycobacterium; Mycobacterium tuberculosis complex; Mycobacterium tuberculosis;	NCBI: 1773	4377519	99.94	0.00	Yes
NCBI Assembly	GCA_003397005.1	NCBI: cellular organisms; Bacteria; Terrabacteria group; Actinobacteria; Actinomycetia; Corynebacteriales; Mycobacteriaceae; Mycobacterium; Mycobacterium tuberculosis complex; Mycobacterium tuberculosis;	NCBI: 1773	4363441	99.94	0.00	Yes
NCBI Assembly	GCA_003396075.1	NCBI: cellular organisms; Bacteria; Terrabacteria group; Actinobacteria; Actinomycetia; Corynebacteriales; Mycobacteriaceae; Mycobacterium; Mycobacterium tuberculosis complex; Mycobacterium tuberculosis;	NCBI: 1773	4372188	99.94	0.00	Yes
NCBI Assembly	GCA_004111665.1	NCBI: cellular organisms; Bacteria; Terrabacteria group; Actinobacteria; Actinomycetia; Corynebacteriales; Mycobacteriaceae; Mycobacterium; Mycobacterium tuberculosis complex; Mycobacterium tuberculosis;	NCBI: 1773	4380996	99.94	0.00	Yes
NCBI Assembly	GCA_004108815.1	NCBI: cellular organisms; Bacteria; Terrabacteria group; Actinobacteria; Actinomycetia; Corynebacteriales; Mycobacteriaceae; Mycobacterium; Mycobacterium tuberculosis complex; Mycobacterium tuberculosis;	NCBI: 1773	4396553	99.94	0.00	Yes
NCBI Assembly	GCA_004106575.1	NCBI: cellular organisms; Bacteria; Terrabacteria group; Actinobacteria; Actinomycetia; Corynebacteriales; Mycobacteriaceae; Mycobacterium; Mycobacterium tuberculosis complex; Mycobacterium tuberculosis;	NCBI: 1773	4398498	99.94	0.00	Yes
NCBI Assembly	GCA_003391955.1	NCBI: cellular organisms; Bacteria; Terrabacteria group; Actinobacteria; Actinomycetia; Corynebacteriales; Mycobacteriaceae; Mycobacterium; Mycobacterium tuberculosis complex; Mycobacterium tuberculosis;	NCBI: 1773	4337446	99.86	0.11	Yes
NCBI Assembly	GCA_004109425.1	NCBI: cellular organisms; Bacteria; Terrabacteria group; Actinobacteria; Actinomycetia; Corynebacteriales; Mycobacteriaceae; Mycobacterium; Mycobacterium tuberculosis complex; Mycobacterium tuberculosis;	NCBI: 1773	4395462	99.94	0.00	Yes
NCBI Assembly	GCA_004112625.1	NCBI: cellular organisms; Bacteria; Terrabacteria group; Actinobacteria; Actinomycetia; Corynebacteriales; Mycobacteriaceae; Mycobacterium; Mycobacterium tuberculosis complex; Mycobacterium tuberculosis;	NCBI: 1773	4396880	99.94	0.00	Yes
NCBI Assembly	GCA_003395435.1	NCBI: cellular organisms; Bacteria; Terrabacteria group; Actinobacteria; Actinomycetia; Corynebacteriales; Mycobacteriaceae; Mycobacterium; Mycobacterium tuberculosis complex; Mycobacterium tuberculosis;	NCBI: 1773	4348607	99.86	0.00	Yes
NCBI Assembly	GCA_003394525.1	NCBI: cellular organisms; Bacteria; Terrabacteria group; Actinobacteria; Actinomycetia; Corynebacteriales; Mycobacteriaceae; Mycobacterium; Mycobacterium tuberculosis complex; Mycobacterium tuberculosis;	NCBI: 1773	4369316	99.86	0.00	Yes
NCBI Assembly	GCA_003861345.1	NCBI: cellular organisms; Bacteria; Terrabacteria group; Actinobacteria; Actinomycetia; Corynebacteriales; Mycobacteriaceae; Mycobacteroides; Mycobacteroides abscessus; Mycobacteroides abscessus subsp. massiliense;	NCBI: 1962118	4621353	100.00	0.33	Yes
NCBI Assembly	GCA_003396085.1	NCBI: cellular organisms; Bacteria; Terrabacteria group; Actinobacteria; Actinomycetia; Corynebacteriales; Mycobacteriaceae; Mycobacterium; Mycobacterium tuberculosis complex; Mycobacterium tuberculosis;	NCBI: 1773	4360827	99.64	0.11	Yes
NCBI Assembly	GCA_003398045.1	NCBI: cellular organisms; Bacteria; Terrabacteria group; Actinobacteria; Actinomycetia; Corynebacteriales; Mycobacteriaceae; Mycobacterium; Mycobacterium tuberculosis complex; Mycobacterium tuberculosis;	NCBI: 1773	4246110	99.13	0.67	Yes
NCBI Assembly	GCA_004106625.1	NCBI: cellular organisms; Bacteria; Terrabacteria group; Actinobacteria; Actinomycetia; Corynebacteriales; Mycobacteriaceae; Mycobacterium; Mycobacterium tuberculosis complex; Mycobacterium tuberculosis;	NCBI: 1773	4405979	99.94	0.00	Yes

NCBI Assembly	GCA_003397725.1	NCBI: cellular organisms; Bacteria; Terrabacteria group; Actinobacteria; Actinomycetia; Corynebacteriales; Mycobacteriaceae; Mycobacterium; Mycobacterium tuberculosis complex; Mycobacterium tuberculosis;	NCBI: 1773	4411914	99.86	0.00	Yes
NCBI Assembly	GCA_004111675.1	NCBI: cellular organisms; Bacteria; Terrabacteria group; Actinobacteria; Actinomycetia; Corynebacteriales; Mycobacteriaceae; Mycobacterium; Mycobacterium tuberculosis complex; Mycobacterium tuberculosis;	NCBI: 1773	4358003	99.94	0.00	Yes
NCBI Assembly	GCA_004108825.1	NCBI: cellular organisms; Bacteria; Terrabacteria group; Actinobacteria; Actinomycetia; Corynebacteriales; Mycobacteriaceae; Mycobacterium; Mycobacterium tuberculosis complex; Mycobacterium tuberculosis;	NCBI: 1773	4392228	99.61	0.00	Yes
NCBI Assembly	GCA_003391985.1	NCBI: cellular organisms; Bacteria; Terrabacteria group; Actinobacteria; Actinomycetia; Corynebacteriales; Mycobacteriaceae; Mycobacterium; Mycobacterium tuberculosis complex; Mycobacterium tuberculosis;	NCBI: 1773	4379314	99.86	0.00	Yes
NCBI Assembly	GCA_003395445.1	NCBI: cellular organisms; Bacteria; Terrabacteria group; Actinobacteria; Actinomycetia; Corynebacteriales; Mycobacteriaceae; Mycobacterium; Mycobacterium tuberculosis complex; Mycobacterium tuberculosis;	NCBI: 1773	4327415	99.86	0.00	Yes
NCBI Assembly	GCA_004109435.1	NCBI: cellular organisms; Bacteria; Terrabacteria group; Actinobacteria; Actinomycetia; Corynebacteriales; Mycobacteriaceae; Mycobacterium; Mycobacterium tuberculosis complex; Mycobacterium tuberculosis;	NCBI: 1773	4361726	99.94	0.00	Yes
NCBI Assembly	GCA_004112635.1	NCBI: cellular organisms; Bacteria; Terrabacteria group; Actinobacteria; Actinomycetia; Corynebacteriales; Mycobacteriaceae; Mycobacterium; Mycobacterium tuberculosis complex; Mycobacterium tuberculosis;	NCBI: 1773	4392746	99.90	0.00	Yes
NCBI Assembly	GCA_003394535.1	NCBI: cellular organisms; Bacteria; Terrabacteria group; Actinobacteria; Actinomycetia; Corynebacteriales; Mycobacteriaceae; Mycobacterium; Mycobacterium tuberculosis complex; Mycobacterium tuberculosis;	NCBI: 1773	4364412	99.86	0.00	Yes
NCBI Assembly	GCA_003933725.1	NCBI: cellular organisms; Bacteria; Terrabacteria group; Actinobacteria; Actinomycetia; Corynebacteriales; Mycobacteriaceae; Mycolicibacter; Mycolicibacter terrae;	NCBI: 1788	4465658	99.55	0.15	Yes
NCBI Assembly	GCA_003398055.1	NCBI: cellular organisms; Bacteria; Terrabacteria group; Actinobacteria; Actinomycetia; Corynebacteriales; Mycobacteriaceae; Mycobacterium; Mycobacterium tuberculosis complex; Mycobacterium tuberculosis;	NCBI: 1773	4398165	99.86	0.00	Yes
NCBI Assembly	GCA_003396105.1	NCBI: cellular organisms; Bacteria; Terrabacteria group; Actinobacteria; Actinomycetia; Corynebacteriales; Mycobacteriaceae; Mycobacterium; Mycobacterium tuberculosis complex; Mycobacterium tuberculosis;	NCBI: 1773	4307209	99.78	0.11	Yes
NCBI Assembly	GCA_004106635.1	NCBI: cellular organisms; Bacteria; Terrabacteria group; Actinobacteria; Actinomycetia; Corynebacteriales; Mycobacteriaceae; Mycobacterium; Mycobacterium tuberculosis complex; Mycobacterium tuberculosis;	NCBI: 1773	4405088	99.94	0.00	Yes
NCBI Assembly	GCA_003397785.1	NCBI: cellular organisms; Bacteria; Terrabacteria group; Actinobacteria; Actinomycetia; Corynebacteriales; Mycobacteriaceae; Mycobacterium; Mycobacterium tuberculosis complex; Mycobacterium tuberculosis;	NCBI: 1773	4346715	99.86	0.00	Yes
NCBI Assembly	GCA_004111685.1	NCBI: cellular organisms; Bacteria; Terrabacteria group; Actinobacteria; Actinomycetia; Corynebacteriales; Mycobacteriaceae; Mycobacterium; Mycobacterium tuberculosis complex; Mycobacterium tuberculosis;	NCBI: 1773	4388623	99.94	0.00	Yes
NCBI Assembly	GCA_004108845.1	NCBI: cellular organisms; Bacteria; Terrabacteria group; Actinobacteria; Actinomycetia; Corynebacteriales; Mycobacteriaceae; Mycobacterium; Mycobacterium tuberculosis complex; Mycobacterium tuberculosis;	NCBI: 1773	4393288	99.94	0.00	Yes
NCBI Assembly	GCA_003391995.1	NCBI: cellular organisms; Bacteria; Terrabacteria group; Actinobacteria; Actinomycetia; Corynebacteriales; Mycobacteriaceae; Mycobacterium; Mycobacterium tuberculosis complex; Mycobacterium tuberculosis;	NCBI: 1773	4367214	99.90	0.00	Yes
NCBI Assembly	GCA_003395485.1	NCBI: cellular organisms; Bacteria; Terrabacteria group; Actinobacteria; Actinomycetia; Corynebacteriales; Mycobacteriaceae; Mycobacterium; Mycobacterium tuberculosis complex; Mycobacterium tuberculosis;	NCBI: 1773	4350410	99.86	0.00	Yes
NCBI Assembly	GCA_004109445.1	NCBI: cellular organisms; Bacteria; Terrabacteria group; Actinobacteria; Actinomycetia; Corynebacteriales; Mycobacteriaceae; Mycobacterium; Mycobacterium tuberculosis complex; Mycobacterium tuberculosis;	NCBI: 1773	4374034	99.94	0.00	Yes

NCBI Assembly	GCA_003394565.1	NCBI: cellular organisms; Bacteria; Terrabacteria group; Actinobacteria; Actinomycetia; Corynebacteriales; Mycobacteriaceae; Mycobacterium; Mycobacterium tuberculosis complex; Mycobacterium tuberculosis;	NCBI: 1773	4349627	99.53	0.00	Yes
NCBI Assembly	GCA_004112665.1	NCBI: cellular organisms; Bacteria; Terrabacteria group; Actinobacteria; Actinomycetia; Corynebacteriales; Mycobacteriaceae; Mycobacterium; Mycobacterium tuberculosis complex; Mycobacterium tuberculosis;	NCBI: 1773	4375854	99.94	0.00	Yes
NCBI Assembly	GCA_003398105.1	NCBI: cellular organisms; Bacteria; Terrabacteria group; Actinobacteria; Actinomycetia; Corynebacteriales; Mycobacteriaceae; Mycobacterium; Mycobacterium tuberculosis complex; Mycobacterium tuberculosis;	NCBI: 1773	4304698	99.86	0.03	Yes
NCBI Assembly	GCA_003957335.1	NCBI: cellular organisms; Bacteria; Terrabacteria group; Actinobacteria; Actinomycetia; Corynebacteriales; Mycobacteriaceae; Mycobacterium; Mycobacterium avium complex (MAC); Mycobacterium avium; Mycobacterium avium subsp. paratuberculosis;	NCBI: 1770	4907428	99.53	0.33	Yes
NCBI Assembly	GCA_003396145.1	NCBI: cellular organisms; Bacteria; Terrabacteria group; Actinobacteria; Actinomycetia; Corynebacteriales; Mycobacteriaceae; Mycobacterium; Mycobacterium tuberculosis complex; Mycobacterium tuberculosis;	NCBI: 1773	4368483	99.86	0.00	Yes
NCBI Assembly	GCA_003397795.1	NCBI: cellular organisms; Bacteria; Terrabacteria group; Actinobacteria; Actinomycetia; Corynebacteriales; Mycobacteriaceae; Mycobacterium; Mycobacterium tuberculosis complex; Mycobacterium tuberculosis;	NCBI: 1773	4381649	99.86	0.00	Yes
NCBI Assembly	GCA_004106645.1	NCBI: cellular organisms; Bacteria; Terrabacteria group; Actinobacteria; Actinomycetia; Corynebacteriales; Mycobacteriaceae; Mycobacterium; Mycobacterium tuberculosis complex; Mycobacterium tuberculosis;	NCBI: 1773	4388351	99.61	0.00	Yes
NCBI Assembly	GCA_004111725.1	NCBI: cellular organisms; Bacteria; Terrabacteria group; Actinobacteria; Actinomycetia; Corynebacteriales; Mycobacteriaceae; Mycobacterium; Mycobacterium tuberculosis complex; Mycobacterium tuberculosis;	NCBI: 1773	4380073	99.94	0.00	Yes
NCBI Assembly	GCA_003392005.1	NCBI: cellular organisms; Bacteria; Terrabacteria group; Actinobacteria; Actinomycetia; Corynebacteriales; Mycobacteriaceae; Mycobacterium; Mycobacterium tuberculosis complex; Mycobacterium tuberculosis;	NCBI: 1773	4259823	99.53	0.32	Yes
NCBI Assembly	GCA_004108885.1	NCBI: cellular organisms; Bacteria; Terrabacteria group; Actinobacteria; Actinomycetia; Corynebacteriales; Mycobacteriaceae; Mycobacterium; Mycobacterium tuberculosis complex; Mycobacterium tuberculosis;	NCBI: 1773	4405411	99.61	0.00	Yes
NCBI Assembly	GCA_003394575.1	NCBI: cellular organisms; Bacteria; Terrabacteria group; Actinobacteria; Actinomycetia; Corynebacteriales; Mycobacteriaceae; Mycobacterium; Mycobacterium tuberculosis complex; Mycobacterium tuberculosis;	NCBI: 1773	4376598	99.86	0.11	Yes
NCBI Assembly	GCA_003395495.1	NCBI: cellular organisms; Bacteria; Terrabacteria group; Actinobacteria; Actinomycetia; Corynebacteriales; Mycobacteriaceae; Mycobacterium; Mycobacterium tuberculosis complex; Mycobacterium tuberculosis;	NCBI: 1773	4351673	99.86	0.11	Yes
NCBI Assembly	GCA_004109505.1	NCBI: cellular organisms; Bacteria; Terrabacteria group; Actinobacteria; Actinomycetia; Corynebacteriales; Mycobacteriaceae; Mycobacterium; Mycobacterium tuberculosis complex; Mycobacterium tuberculosis;	NCBI: 1773	4341291	99.94	0.00	Yes
NCBI Assembly	GCA_004112675.1	NCBI: cellular organisms; Bacteria; Terrabacteria group; Actinobacteria; Actinomycetia; Corynebacteriales; Mycobacteriaceae; Mycobacterium; Mycobacterium tuberculosis complex; Mycobacterium tuberculosis;	NCBI: 1773	4412649	99.94	0.00	Yes
NCBI Assembly	GCA_003398115.1	NCBI: cellular organisms; Bacteria; Terrabacteria group; Actinobacteria; Actinomycetia; Corynebacteriales; Mycobacteriaceae; Mycobacterium; Mycobacterium tuberculosis complex; Mycobacterium tuberculosis;	NCBI: 1773	4359245	99.86	0.00	Yes
NCBI Assembly	GCA_004014795.1	NCBI: cellular organisms; Bacteria; Terrabacteria group; Actinobacteria; Actinomycetia; Corynebacteriales; Mycobacteriaceae; Mycolicibacterium; Mycolicibacterium brumae;	NCBI: 1335619	3938050	98.99	0.05	Yes
NCBI Assembly	GCA_003396155.1	NCBI: cellular organisms; Bacteria; Terrabacteria group; Actinobacteria; Actinomycetia; Corynebacteriales; Mycobacteriaceae; Mycobacterium; Mycobacterium tuberculosis complex; Mycobacterium tuberculosis;	NCBI: 1773	4324960	99.86	0.00	Yes
NCBI Assembly	GCA_003397815.1	NCBI: cellular organisms; Bacteria; Terrabacteria group; Actinobacteria; Actinomycetia; Corynebacteriales; Mycobacteriaceae; Mycobacterium; Mycobacterium tuberculosis complex; Mycobacterium tuberculosis;	NCBI: 1773	4258525	99.61	0.74	Yes

NCBI Assembly	GCA_004106685.1	NCBI: cellular organisms; Bacteria; Terrabacteria group; Actinobacteria; Actinomycetia; Corynebacteriales; Mycobacteriaceae; Mycobacterium; Mycobacterium tuberculosis complex; Mycobacterium tuberculosis;	NCBI: 1773	4400581	99.94	0.00	Yes
NCBI Assembly	GCA_003392025.1	NCBI: cellular organisms; Bacteria; Terrabacteria group; Actinobacteria; Actinomycetia; Corynebacteriales; Mycobacteriaceae; Mycobacterium; Mycobacterium tuberculosis complex; Mycobacterium tuberculosis;	NCBI: 1773	4332423	99.86	0.00	Yes
NCBI Assembly	GCA_003394585.1	NCBI: cellular organisms; Bacteria; Terrabacteria group; Actinobacteria; Actinomycetia; Corynebacteriales; Mycobacteriaceae; Mycobacterium; Mycobacterium tuberculosis complex; Mycobacterium tuberculosis;	NCBI: 1773	4380719	99.86	0.00	Yes
NCBI Assembly	GCA_004111735.1	NCBI: cellular organisms; Bacteria; Terrabacteria group; Actinobacteria; Actinomycetia; Corynebacteriales; Mycobacteriaceae; Mycobacterium; Mycobacterium tuberculosis complex; Mycobacterium tuberculosis;	NCBI: 1773	4384222	99.94	0.00	Yes
NCBI Assembly	GCA_003395505.1	NCBI: cellular organisms; Bacteria; Terrabacteria group; Actinobacteria; Actinomycetia; Corynebacteriales; Mycobacteriaceae; Mycobacterium; Mycobacterium tuberculosis complex; Mycobacterium tuberculosis;	NCBI: 1773	4323061	99.86	0.00	Yes
NCBI Assembly	GCA_004108905.1	NCBI: cellular organisms; Bacteria; Terrabacteria group; Actinobacteria; Actinomycetia; Corynebacteriales; Mycobacteriaceae; Mycobacterium; Mycobacterium tuberculosis complex; Mycobacterium tuberculosis;	NCBI: 1773	4379601	99.94	0.00	Yes
NCBI Assembly	GCA_004109515.1	NCBI: cellular organisms; Bacteria; Terrabacteria group; Actinobacteria; Actinomycetia; Corynebacteriales; Mycobacteriaceae; Mycobacterium; Mycobacterium tuberculosis complex; Mycobacterium tuberculosis;	NCBI: 1773	4357422	99.94	0.00	Yes
NCBI Assembly	GCA_004112695.1	NCBI: cellular organisms; Bacteria; Terrabacteria group; Actinobacteria; Actinomycetia; Corynebacteriales; Mycobacteriaceae; Mycobacterium; Mycobacterium tuberculosis complex; Mycobacterium tuberculosis;	NCBI: 1773	4397762	99.61	0.00	Yes
NCBI Assembly	GCA_003398135.1	NCBI: cellular organisms; Bacteria; Terrabacteria group; Actinobacteria; Actinomycetia; Corynebacteriales; Mycobacteriaceae; Mycobacterium; Mycobacterium tuberculosis complex; Mycobacterium tuberculosis;	NCBI: 1773	4372008	99.86	0.10	Yes
NCBI Assembly	GCA_003396165.1	NCBI: cellular organisms; Bacteria; Terrabacteria group; Actinobacteria; Actinomycetia; Corynebacteriales; Mycobacteriaceae; Mycobacterium; Mycobacterium tuberculosis complex; Mycobacterium tuberculosis;	NCBI: 1773	4351503	99.86	0.00	Yes
NCBI Assembly	GCA_003397835.1	NCBI: cellular organisms; Bacteria; Terrabacteria group; Actinobacteria; Actinomycetia; Corynebacteriales; Mycobacteriaceae; Mycobacterium; Mycobacterium tuberculosis complex; Mycobacterium tuberculosis;	NCBI: 1773	4356126	99.94	0.00	Yes
NCBI Assembly	GCA_003122745.2	NCBI: cellular organisms; Bacteria; Terrabacteria group; Actinobacteria; Actinomycetia; Corynebacteriales; Mycobacteriaceae; Mycobacterium; Mycobacterium avium complex (MAC); Mycobacterium avium; Mycobacterium avium subsp. hominissuis;	NCBI: 439334	5131895	99.94	0.05	Yes
NCBI Assembly	GCA_003393625.1	NCBI: cellular organisms; Bacteria; Terrabacteria group; Actinobacteria; Actinomycetia; Corynebacteriales; Mycobacteriaceae; Mycobacterium; Mycobacterium tuberculosis complex; Mycobacterium tuberculosis;	NCBI: 1773	4400027	99.94	0.33	Yes
NCBI Assembly	GCA_003395515.1	NCBI: cellular organisms; Bacteria; Terrabacteria group; Actinobacteria; Actinomycetia; Corynebacteriales; Mycobacteriaceae; Mycobacterium; Mycobacterium tuberculosis complex; Mycobacterium tuberculosis;	NCBI: 1773	4277236	99.86	0.11	Yes
NCBI Assembly	GCA_004111755.1	NCBI: cellular organisms; Bacteria; Terrabacteria group; Actinobacteria; Actinomycetia; Corynebacteriales; Mycobacteriaceae; Mycobacterium; Mycobacterium tuberculosis complex; Mycobacterium tuberculosis;	NCBI: 1773	4382879	99.94	0.00	Yes
NCBI Assembly	GCA_004106695.1	NCBI: cellular organisms; Bacteria; Terrabacteria group; Actinobacteria; Actinomycetia; Corynebacteriales; Mycobacteriaceae; Mycobacterium; Mycobacterium tuberculosis complex; Mycobacterium tuberculosis;	NCBI: 1773	4388560	99.94	0.00	Yes
NCBI Assembly	GCA_004108915.1	NCBI: cellular organisms; Bacteria; Terrabacteria group; Actinobacteria; Actinomycetia; Corynebacteriales; Mycobacteriaceae; Mycobacterium; Mycobacterium tuberculosis complex; Mycobacterium tuberculosis;	NCBI: 1773	4388854	99.61	0.00	Yes
NCBI Assembly	GCA_004109525.1	NCBI: cellular organisms; Bacteria; Terrabacteria group; Actinobacteria; Actinomycetia; Corynebacteriales; Mycobacteriaceae; Mycobacterium; Mycobacterium tuberculosis complex; Mycobacterium tuberculosis;	NCBI: 1773	4390189	99.61	0.00	Yes

NCBI Assembly	GCA_004112725.1	NCBI: cellular organisms; Bacteria; Terrabacteria group; Actinobacteria; Actinomycetia; Corynebacteriales; Mycobacteriaceae; Mycobacterium; Mycobacterium tuberculosis complex; Mycobacterium tuberculosis;	NCBI: 1773	4391546	99.94	0.00	Yes
NCBI Assembly	GCA_003398805.1	NCBI: cellular organisms; Bacteria; Terrabacteria group; Actinobacteria; Actinomycetia; Corynebacteriales; Mycobacteriaceae; Mycobacterium; Mycobacterium tuberculosis complex; Mycobacterium tuberculosis;	NCBI: 1773	4261117	99.86	0.00	Yes
NCBI Assembly	GCA_003398485.1	NCBI: cellular organisms; Bacteria; Terrabacteria group; Actinobacteria; Actinomycetia; Corynebacteriales; Mycobacteriaceae; Mycobacterium; Mycobacterium tuberculosis complex; Mycobacterium tuberculosis;	NCBI: 1773	4299784	99.86	0.11	Yes
NCBI Assembly	GCA_004014805.1	NCBI: cellular organisms; Bacteria; Terrabacteria group; Actinobacteria; Actinomycetia; Corynebacteriales; Mycobacteriaceae; Mycolicibacterium; Mycolicibacterium elephantis;	NCBI: 1335622	5469939	100.00	1.68	Yes
NCBI Assembly	GCA_004110255.1	NCBI: cellular organisms; Bacteria; Terrabacteria group; Actinobacteria; Actinomycetia; Corynebacteriales; Mycobacteriaceae; Mycobacterium; Mycobacterium tuberculosis complex; Mycobacterium tuberculosis;	NCBI: 1773	4391500	99.94	0.00	Yes
NCBI Assembly	GCA_003129545.1	NCBI: cellular organisms; Bacteria; Terrabacteria group; Actinobacteria; Actinomycetia; Corynebacteriales; Mycobacteriaceae; Mycobacterium; Mycobacterium tuberculosis complex; Mycobacterium tuberculosis;	NCBI: 1773	4408432	99.61	0.00	Yes
NCBI Assembly	GCA_003393635.1	NCBI: cellular organisms; Bacteria; Terrabacteria group; Actinobacteria; Actinomycetia; Corynebacteriales; Mycobacteriaceae; Mycobacterium; Mycobacterium tuberculosis complex; Mycobacterium tuberculosis;	NCBI: 1773	4412079	99.86	1.00	Yes
NCBI Assembly	GCA_004109935.1	NCBI: cellular organisms; Bacteria; Terrabacteria group; Actinobacteria; Actinomycetia; Corynebacteriales; Mycobacteriaceae; Mycobacterium; Mycobacterium tuberculosis complex; Mycobacterium tuberculosis;	NCBI: 1773	4380815	99.94	0.00	Yes
NCBI Assembly	GCA_004106705.1	NCBI: cellular organisms; Bacteria; Terrabacteria group; Actinobacteria; Actinomycetia; Corynebacteriales; Mycobacteriaceae; Mycobacterium; Mycobacterium tuberculosis complex; Mycobacterium tuberculosis;	NCBI: 1773	4407797	99.94	0.00	Yes
NCBI Assembly	GCA_004111765.1	NCBI: cellular organisms; Bacteria; Terrabacteria group; Actinobacteria; Actinomycetia; Corynebacteriales; Mycobacteriaceae; Mycobacterium; Mycobacterium tuberculosis complex; Mycobacterium tuberculosis;	NCBI: 1773	4377353	99.94	0.00	Yes
NCBI Assembly	GCA_003398815.1	NCBI: cellular organisms; Bacteria; Terrabacteria group; Actinobacteria; Actinomycetia; Corynebacteriales; Mycobacteriaceae; Mycobacterium; Mycobacterium tuberculosis complex; Mycobacterium tuberculosis;	NCBI: 1773	4283154	99.86	0.33	Yes
NCBI Assembly	GCA_004108935.1	NCBI: cellular organisms; Bacteria; Terrabacteria group; Actinobacteria; Actinomycetia; Corynebacteriales; Mycobacteriaceae; Mycobacterium; Mycobacterium tuberculosis complex; Mycobacterium tuberculosis;	NCBI: 1773	4404455	99.94	0.00	Yes
NCBI Assembly	GCA_004109535.1	NCBI: cellular organisms; Bacteria; Terrabacteria group; Actinobacteria; Actinomycetia; Corynebacteriales; Mycobacteriaceae; Mycobacterium; Mycobacterium tuberculosis complex; Mycobacterium tuberculosis;	NCBI: 1773	4382426	99.94	0.00	Yes
NCBI Assembly	GCA_004112735.1	NCBI: cellular organisms; Bacteria; Terrabacteria group; Actinobacteria; Actinomycetia; Corynebacteriales; Mycobacteriaceae; Mycobacterium; Mycobacterium tuberculosis complex; Mycobacterium tuberculosis;	NCBI: 1773	4388569	99.94	0.00	Yes
NCBI Assembly	GCA_003398495.1	NCBI: cellular organisms; Bacteria; Terrabacteria group; Actinobacteria; Actinomycetia; Corynebacteriales; Mycobacteriaceae; Mycobacterium; Mycobacterium tuberculosis complex; Mycobacterium tuberculosis;	NCBI: 1773	4329165	99.86	0.00	Yes
NCBI Assembly	GCA_004022355.1	NCBI: cellular organisms; Bacteria; Terrabacteria group; Actinobacteria; Actinomycetia; Corynebacteriales; Mycobacteriaceae; Mycobacterium; Mycobacterium tuberculosis complex; Mycobacterium tuberculosis;	NCBI: 1773	4333226	99.91	0.00	Yes
NCBI Assembly	GCA_003201655.1	NCBI: cellular organisms; Bacteria; Terrabacteria group; Actinobacteria; Actinomycetia; Corynebacteriales; Mycobacteriaceae; Mycolicibacterium; Mycolicibacterium moriokaense;	NCBI: 39691	6421161	100.00	1.14	Yes
NCBI Assembly	GCA_004110265.1	NCBI: cellular organisms; Bacteria; Terrabacteria group; Actinobacteria; Actinomycetia; Corynebacteriales; Mycobacteriaceae; Mycobacterium; Mycobacterium tuberculosis complex; Mycobacterium tuberculosis;	NCBI: 1773	4384377	99.94	0.00	Yes

NCBI Assembly	GCA_003393645.1	NCBI: cellular organisms; Bacteria; Terrabacteria group; Actinobacteria; Actinomycetia; Corynebacteriales; Mycobacteriaceae; Mycobacterium; Mycobacterium tuberculosis complex; Mycobacterium tuberculosis;	NCBI: 1773	4364270	99.86	0.00	Yes
NCBI Assembly	GCA_004106725.1	NCBI: cellular organisms; Bacteria; Terrabacteria group; Actinobacteria; Actinomycetia; Corynebacteriales; Mycobacteriaceae; Mycobacterium; Mycobacterium tuberculosis complex; Mycobacterium tuberculosis;	NCBI: 1773	4393173	99.94	0.00	Yes
NCBI Assembly	GCA_004111775.1	NCBI: cellular organisms; Bacteria; Terrabacteria group; Actinobacteria; Actinomycetia; Corynebacteriales; Mycobacteriaceae; Mycobacterium; Mycobacterium tuberculosis complex; Mycobacterium tuberculosis;	NCBI: 1773	4387675	99.94	0.00	Yes
NCBI Assembly	GCA_004109945.1	NCBI: cellular organisms; Bacteria; Terrabacteria group; Actinobacteria; Actinomycetia; Corynebacteriales; Mycobacteriaceae; Mycobacterium; Mycobacterium tuberculosis complex; Mycobacterium tuberculosis;	NCBI: 1773	4373440	99.94	0.00	Yes
NCBI Assembly	GCA_004108945.1	NCBI: cellular organisms; Bacteria; Terrabacteria group; Actinobacteria; Actinomycetia; Corynebacteriales; Mycobacteriaceae; Mycobacterium; Mycobacterium tuberculosis complex; Mycobacterium tuberculosis;	NCBI: 1773	4391433	99.94	0.00	Yes
NCBI Assembly	GCA_003398825.1	NCBI: cellular organisms; Bacteria; Terrabacteria group; Actinobacteria; Actinomycetia; Corynebacteriales; Mycobacteriaceae; Mycobacterium; Mycobacterium tuberculosis complex; Mycobacterium tuberculosis;	NCBI: 1773	4271748	99.53	0.28	Yes
NCBI Assembly	GCA_004109565.1	NCBI: cellular organisms; Bacteria; Terrabacteria group; Actinobacteria; Actinomycetia; Corynebacteriales; Mycobacteriaceae; Mycobacterium; Mycobacterium tuberculosis complex; Mycobacterium tuberculosis;	NCBI: 1773	4382708	99.61	0.00	Yes
NCBI Assembly	GCA_004112755.1	NCBI: cellular organisms; Bacteria; Terrabacteria group; Actinobacteria; Actinomycetia; Corynebacteriales; Mycobacteriaceae; Mycobacterium; Mycobacterium tuberculosis complex; Mycobacterium tuberculosis;	NCBI: 1773	4386798	99.94	0.00	Yes
NCBI Assembly	GCA_003398505.1	NCBI: cellular organisms; Bacteria; Terrabacteria group; Actinobacteria; Actinomycetia; Corynebacteriales; Mycobacteriaceae; Mycobacterium; Mycobacterium tuberculosis complex; Mycobacterium tuberculosis;	NCBI: 1773	4303364	99.86	0.00	Yes
NCBI Assembly	GCA_004022405.1	NCBI: cellular organisms; Bacteria; Terrabacteria group; Actinobacteria; Actinomycetia; Corynebacteriales; Mycobacteriaceae; Mycobacterium; Mycobacterium tuberculosis complex; Mycobacterium tuberculosis;	NCBI: 1773	4446486	99.94	3.49	Yes
NCBI Assembly	GCA_003393685.1	NCBI: cellular organisms; Bacteria; Terrabacteria group; Actinobacteria; Actinomycetia; Corynebacteriales; Mycobacteriaceae; Mycobacterium; Mycobacterium tuberculosis complex; Mycobacterium tuberculosis;	NCBI: 1773	4251178	99.86	0.04	Yes
NCBI Assembly	GCA_004110305.1	NCBI: cellular organisms; Bacteria; Terrabacteria group; Actinobacteria; Actinomycetia; Corynebacteriales; Mycobacteriaceae; Mycobacterium; Mycobacterium tuberculosis complex; Mycobacterium tuberculosis;	NCBI: 1773	4393994	99.94	0.07	Yes
NCBI Assembly	GCA_004105795.1	NCBI: cellular organisms; Bacteria; Terrabacteria group; Actinobacteria; Actinomycetia; Corynebacteriales; Mycobacteriaceae; Mycobacterium; Mycobacterium tuberculosis complex; Mycobacterium tuberculosis;	NCBI: 1773	4381665	99.94	0.00	Yes
NCBI Assembly	GCA_004109975.1	NCBI: cellular organisms; Bacteria; Terrabacteria group; Actinobacteria; Actinomycetia; Corynebacteriales; Mycobacteriaceae; Mycobacterium; Mycobacterium tuberculosis complex; Mycobacterium tuberculosis;	NCBI: 1773	4364430	99.94	0.00	Yes
NCBI Assembly	GCA_004110855.1	NCBI: cellular organisms; Bacteria; Terrabacteria group; Actinobacteria; Actinomycetia; Corynebacteriales; Mycobacteriaceae; Mycobacterium; Mycobacterium tuberculosis complex; Mycobacterium tuberculosis;	NCBI: 1773	4361678	99.61	0.00	Yes
NCBI Assembly	GCA_003398835.1	NCBI: cellular organisms; Bacteria; Terrabacteria group; Actinobacteria; Actinomycetia; Corynebacteriales; Mycobacteriaceae; Mycobacterium; Mycobacterium tuberculosis complex; Mycobacterium tuberculosis;	NCBI: 1773	4372121	99.86	0.00	Yes
NCBI Assembly	GCA_004108955.1	NCBI: cellular organisms; Bacteria; Terrabacteria group; Actinobacteria; Actinomycetia; Corynebacteriales; Mycobacteriaceae; Mycobacterium; Mycobacterium tuberculosis complex; Mycobacterium tuberculosis;	NCBI: 1773	4407669	99.61	0.00	Yes
NCBI Assembly	GCA_004112765.1	NCBI: cellular organisms; Bacteria; Terrabacteria group; Actinobacteria; Actinomycetia; Corynebacteriales; Mycobacteriaceae; Mycobacterium; Mycobacterium tuberculosis complex; Mycobacterium tuberculosis;	NCBI: 1773	4385823	99.61	0.00	Yes

NCBI Assembly	GCA_004110565.1	NCBI: cellular organisms; Bacteria; Terrabacteria group; Actinobacteria; Actinomycetia; Corynebacteriales; Mycobacteriaceae; Mycobacterium; Mycobacterium tuberculosis complex; Mycobacterium tuberculosis;	NCBI: 1773	4364373	99.61	0.00	Yes
NCBI Assembly	GCA_003398545.1	NCBI: cellular organisms; Bacteria; Terrabacteria group; Actinobacteria; Actinomycetia; Corynebacteriales; Mycobacteriaceae; Mycobacterium; Mycobacterium tuberculosis complex; Mycobacterium tuberculosis;	NCBI: 1773	4329348	99.86	0.33	Yes
NCBI Assembly	GCA_003254575.1	NCBI: cellular organisms; Bacteria; Terrabacteria group; Actinobacteria; Actinomycetia; Corynebacteriales; Mycobacteriaceae; Mycobacterium; Mycobacterium kyogaense;	NCBI: 2212479	5759757	100.00	0.56	Yes
NCBI Assembly	GCA_004105175.1	NCBI: cellular organisms; Bacteria; Terrabacteria group; Actinobacteria; Actinomycetia; Corynebacteriales; Mycobacteriaceae; Mycobacterium; Mycobacterium tuberculosis complex; Mycobacterium tuberculosis;	NCBI: 1773	4392468	99.94	0.00	Yes
NCBI Assembly	GCA_003393695.1	NCBI: cellular organisms; Bacteria; Terrabacteria group; Actinobacteria; Actinomycetia; Corynebacteriales; Mycobacteriaceae; Mycobacterium; Mycobacterium tuberculosis complex; Mycobacterium tuberculosis;	NCBI: 1773	4342830	99.69	0.00	Yes
NCBI Assembly	GCA_004110325.1	NCBI: cellular organisms; Bacteria; Terrabacteria group; Actinobacteria; Actinomycetia; Corynebacteriales; Mycobacteriaceae; Mycobacterium; Mycobacterium tuberculosis complex; Mycobacterium tuberculosis;	NCBI: 1773	4366032	99.94	0.00	Yes
NCBI Assembly	GCA_004105805.1	NCBI: cellular organisms; Bacteria; Terrabacteria group; Actinobacteria; Actinomycetia; Corynebacteriales; Mycobacteriaceae; Mycobacterium; Mycobacterium tuberculosis complex; Mycobacterium tuberculosis;	NCBI: 1773	4399493	99.94	0.00	Yes
NCBI Assembly	GCA_003398885.1	NCBI: cellular organisms; Bacteria; Terrabacteria group; Actinobacteria; Actinomycetia; Corynebacteriales; Mycobacteriaceae; Mycobacterium; Mycobacterium tuberculosis complex; Mycobacterium tuberculosis;	NCBI: 1773	4373489	99.86	0.00	Yes
NCBI Assembly	GCA_004109985.1	NCBI: cellular organisms; Bacteria; Terrabacteria group; Actinobacteria; Actinomycetia; Corynebacteriales; Mycobacteriaceae; Mycobacterium; Mycobacterium tuberculosis complex; Mycobacterium tuberculosis;	NCBI: 1773	4363268	99.94	0.00	Yes
NCBI Assembly	GCA_004109005.1	NCBI: cellular organisms; Bacteria; Terrabacteria group; Actinobacteria; Actinomycetia; Corynebacteriales; Mycobacteriaceae; Mycobacterium; Mycobacterium tuberculosis complex; Mycobacterium tuberculosis;	NCBI: 1773	4391199	99.61	0.00	Yes
NCBI Assembly	GCA_004110865.1	NCBI: cellular organisms; Bacteria; Terrabacteria group; Actinobacteria; Actinomycetia; Corynebacteriales; Mycobacteriaceae; Mycobacterium; Mycobacterium tuberculosis complex; Mycobacterium tuberculosis;	NCBI: 1773	4371588	99.94	0.00	Yes
NCBI Assembly	GCA_004110575.1	NCBI: cellular organisms; Bacteria; Terrabacteria group; Actinobacteria; Actinomycetia; Corynebacteriales; Mycobacteriaceae; Mycobacterium; Mycobacterium tuberculosis complex; Mycobacterium tuberculosis;	NCBI: 1773	4389158	99.61	0.00	Yes
NCBI Assembly	GCA_004112785.1	NCBI: cellular organisms; Bacteria; Terrabacteria group; Actinobacteria; Actinomycetia; Corynebacteriales; Mycobacteriaceae; Mycobacterium; Mycobacterium tuberculosis complex; Mycobacterium tuberculosis;	NCBI: 1773	4387423	99.61	0.07	Yes
NCBI Assembly	GCA_003398565.1	NCBI: cellular organisms; Bacteria; Terrabacteria group; Actinobacteria; Actinomycetia; Corynebacteriales; Mycobacteriaceae; Mycobacterium; Mycobacterium tuberculosis complex; Mycobacterium tuberculosis;	NCBI: 1773	4333054	99.86	0.83	Yes
NCBI Assembly	GCA_003265005.1	NCBI: cellular organisms; Bacteria; Terrabacteria group; Actinobacteria; Actinomycetia; Corynebacteriales; Mycobacteriaceae; Mycobacterium; Mycobacterium tuberculosis complex; Mycobacterium tuberculosis;	NCBI: 1773	4418559	99.94	0.00	Yes
NCBI Assembly	GCA_003393705.1	NCBI: cellular organisms; Bacteria; Terrabacteria group; Actinobacteria; Actinomycetia; Corynebacteriales; Mycobacteriaceae; Mycobacterium; Mycobacterium tuberculosis complex; Mycobacterium tuberculosis;	NCBI: 1773	4300291	99.69	0.00	Yes
NCBI Assembly	GCA_004105185.1	NCBI: cellular organisms; Bacteria; Terrabacteria group; Actinobacteria; Actinomycetia; Corynebacteriales; Mycobacteriaceae; Mycobacterium; Mycobacterium tuberculosis complex; Mycobacterium tuberculosis;	NCBI: 1773	4389973	99.61	0.00	Yes
NCBI Assembly	GCA_004110335.1	NCBI: cellular organisms; Bacteria; Terrabacteria group; Actinobacteria; Actinomycetia; Corynebacteriales; Mycobacteriaceae; Mycobacterium; Mycobacterium tuberculosis complex; Mycobacterium tuberculosis;	NCBI: 1773	4378898	99.94	0.00	Yes

NCBI Assembly	GCA_004105815.1	NCBI: cellular organisms; Bacteria; Terrabacteria group; Actinobacteria; Actinomycetia; Corynebacteriales; Mycobacteriaceae; Mycobacterium; Mycobacterium tuberculosis complex; Mycobacterium tuberculosis;	NCBI: 1773	4412908	99.94	0.00	Yes
NCBI Assembly	GCA_003398905.1	NCBI: cellular organisms; Bacteria; Terrabacteria group; Actinobacteria; Actinomycetia; Corynebacteriales; Mycobacteriaceae; Mycobacterium; Mycobacterium tuberculosis complex; Mycobacterium tuberculosis;	NCBI: 1773	4346788	99.86	0.00	Yes
NCBI Assembly	GCA_004109025.1	NCBI: cellular organisms; Bacteria; Terrabacteria group; Actinobacteria; Actinomycetia; Corynebacteriales; Mycobacteriaceae; Mycobacterium; Mycobacterium tuberculosis complex; Mycobacterium tuberculosis;	NCBI: 1773	4400602	99.61	0.00	Yes
NCBI Assembly	GCA_004110025.1	NCBI: cellular organisms; Bacteria; Terrabacteria group; Actinobacteria; Actinomycetia; Corynebacteriales; Mycobacteriaceae; Mycobacterium; Mycobacterium tuberculosis complex; Mycobacterium tuberculosis;	NCBI: 1773	4373566	99.94	0.00	Yes
NCBI Assembly	GCA_004110925.1	NCBI: cellular organisms; Bacteria; Terrabacteria group; Actinobacteria; Actinomycetia; Corynebacteriales; Mycobacteriaceae; Mycobacterium; Mycobacterium tuberculosis complex; Mycobacterium tuberculosis;	NCBI: 1773	4381483	99.94	0.00	Yes
NCBI Assembly	GCA_004110625.1	NCBI: cellular organisms; Bacteria; Terrabacteria group; Actinobacteria; Actinomycetia; Corynebacteriales; Mycobacteriaceae; Mycobacterium; Mycobacterium tuberculosis complex; Mycobacterium tuberculosis;	NCBI: 1773	4395145	99.94	0.00	Yes
NCBI Assembly	GCA_004112825.1	NCBI: cellular organisms; Bacteria; Terrabacteria group; Actinobacteria; Actinomycetia; Corynebacteriales; Mycobacteriaceae; Mycobacterium; Mycobacterium tuberculosis complex; Mycobacterium tuberculosis;	NCBI: 1773	4359589	99.94	0.00	Yes
NCBI Assembly	GCA_003398575.1	NCBI: cellular organisms; Bacteria; Terrabacteria group; Actinobacteria; Actinomycetia; Corynebacteriales; Mycobacteriaceae; Mycobacterium; Mycobacterium tuberculosis complex; Mycobacterium tuberculosis;	NCBI: 1773	4368339	99.86	0.83	Yes
NCBI Assembly	GCA_003284925.1	NCBI: cellular organisms; Bacteria; Terrabacteria group; Actinobacteria; Actinomycetia; Corynebacteriales; Mycobacteriaceae; Mycolicibacter; Mycolicibacter senuensis;	NCBI: 386913	4792971	98.09	0.61	Yes
NCBI Assembly	GCA_003393715.1	NCBI: cellular organisms; Bacteria; Terrabacteria group; Actinobacteria; Actinomycetia; Corynebacteriales; Mycobacteriaceae; Mycobacterium; Mycobacterium tuberculosis complex; Mycobacterium tuberculosis;	NCBI: 1773	4331077	99.86	0.00	Yes
NCBI Assembly	GCA_004105205.1	NCBI: cellular organisms; Bacteria; Terrabacteria group; Actinobacteria; Actinomycetia; Corynebacteriales; Mycobacteriaceae; Mycobacterium; Mycobacterium tuberculosis complex; Mycobacterium tuberculosis;	NCBI: 1773	4405647	99.94	0.00	Yes
NCBI Assembly	GCA_004110345.1	NCBI: cellular organisms; Bacteria; Terrabacteria group; Actinobacteria; Actinomycetia; Corynebacteriales; Mycobacteriaceae; Mycobacterium; Mycobacterium tuberculosis complex; Mycobacterium tuberculosis;	NCBI: 1773	4388566	99.94	0.00	Yes
NCBI Assembly	GCA_003398915.1	NCBI: cellular organisms; Bacteria; Terrabacteria group; Actinobacteria; Actinomycetia; Corynebacteriales; Mycobacteriaceae; Mycobacterium; Mycobacterium tuberculosis complex; Mycobacterium tuberculosis;	NCBI: 1773	4368905	99.69	0.00	Yes
NCBI Assembly	GCA_004105865.1	NCBI: cellular organisms; Bacteria; Terrabacteria group; Actinobacteria; Actinomycetia; Corynebacteriales; Mycobacteriaceae; Mycobacterium; Mycobacterium tuberculosis complex; Mycobacterium tuberculosis;	NCBI: 1773	4400097	99.61	0.00	Yes
NCBI Assembly	GCA_004110035.1	NCBI: cellular organisms; Bacteria; Terrabacteria group; Actinobacteria; Actinomycetia; Corynebacteriales; Mycobacteriaceae; Mycobacterium; Mycobacterium tuberculosis complex; Mycobacterium tuberculosis;	NCBI: 1773	4371260	99.94	0.00	Yes
NCBI Assembly	GCA_004109035.1	NCBI: cellular organisms; Bacteria; Terrabacteria group; Actinobacteria; Actinomycetia; Corynebacteriales; Mycobacteriaceae; Mycobacterium; Mycobacterium tuberculosis complex; Mycobacterium tuberculosis;	NCBI: 1773	4370995	99.61	0.00	Yes
NCBI Assembly	GCA_004110935.1	NCBI: cellular organisms; Bacteria; Terrabacteria group; Actinobacteria; Actinomycetia; Corynebacteriales; Mycobacteriaceae; Mycobacterium; Mycobacterium tuberculosis complex; Mycobacterium tuberculosis;	NCBI: 1773	4374358	99.94	0.00	Yes
NCBI Assembly	GCA_004110635.1	NCBI: cellular organisms; Bacteria; Terrabacteria group; Actinobacteria; Actinomycetia; Corynebacteriales; Mycobacteriaceae; Mycobacterium; Mycobacterium tuberculosis complex; Mycobacterium tuberculosis;	NCBI: 1773	4366584	99.94	0.00	Yes

NCBI Assembly	GCA_003398585.1	NCBI: cellular organisms; Bacteria; Terrabacteria group; Actinobacteria; Actinomycetia; Corynebacteriales; Mycobacteriaceae; Mycobacterium; Mycobacterium tuberculosis complex; Mycobacterium tuberculosis;	NCBI: 1773	4380367	99.94	0.00	Yes
NCBI Assembly	GCA_003284935.1	NCBI: cellular organisms; Bacteria; Terrabacteria group; Actinobacteria; Actinomycetia; Corynebacteriales; Mycobacteriaceae; Mycobacterium; Mycobacterium avium complex (MAC); Mycobacterium colombiense;	NCBI: 339268	5473554	98.90	0.46	Yes
NCBI Assembly	GCA_004112835.1	NCBI: cellular organisms; Bacteria; Terrabacteria group; Actinobacteria; Actinomycetia; Corynebacteriales; Mycobacteriaceae; Mycobacterium; Mycobacterium tuberculosis complex; Mycobacterium tuberculosis;	NCBI: 1773	4382020	99.94	0.00	Yes
NCBI Assembly	GCA_003393765.1	NCBI: cellular organisms; Bacteria; Terrabacteria group; Actinobacteria; Actinomycetia; Corynebacteriales; Mycobacteriaceae; Mycobacterium; Mycobacterium tuberculosis complex; Mycobacterium tuberculosis;	NCBI: 1773	4284484	99.86	0.04	Yes
NCBI Assembly	GCA_004105235.1	NCBI: cellular organisms; Bacteria; Terrabacteria group; Actinobacteria; Actinomycetia; Corynebacteriales; Mycobacteriaceae; Mycobacterium; Mycobacterium tuberculosis complex; Mycobacterium tuberculosis;	NCBI: 1773	4382245	99.94	0.00	Yes
NCBI Assembly	GCA_004110365.1	NCBI: cellular organisms; Bacteria; Terrabacteria group; Actinobacteria; Actinomycetia; Corynebacteriales; Mycobacteriaceae; Mycobacterium; Mycobacterium tuberculosis complex; Mycobacterium tuberculosis;	NCBI: 1773	4398436	99.94	0.00	Yes
NCBI Assembly	GCA_003398925.1	NCBI: cellular organisms; Bacteria; Terrabacteria group; Actinobacteria; Actinomycetia; Corynebacteriales; Mycobacteriaceae; Mycobacterium; Mycobacterium tuberculosis complex; Mycobacterium tuberculosis;	NCBI: 1773	4349362	99.86	0.00	Yes
NCBI Assembly	GCA_004105875.1	NCBI: cellular organisms; Bacteria; Terrabacteria group; Actinobacteria; Actinomycetia; Corynebacteriales; Mycobacteriaceae; Mycobacterium; Mycobacterium tuberculosis complex; Mycobacterium tuberculosis;	NCBI: 1773	4377519	99.94	0.00	Yes
NCBI Assembly	GCA_004109045.1	NCBI: cellular organisms; Bacteria; Terrabacteria group; Actinobacteria; Actinomycetia; Corynebacteriales; Mycobacteriaceae; Mycobacterium; Mycobacterium tuberculosis complex; Mycobacterium tuberculosis;	NCBI: 1773	4393674	99.61	0.00	Yes
NCBI Assembly	GCA_004110945.1	NCBI: cellular organisms; Bacteria; Terrabacteria group; Actinobacteria; Actinomycetia; Corynebacteriales; Mycobacteriaceae; Mycobacterium; Mycobacterium tuberculosis complex; Mycobacterium tuberculosis;	NCBI: 1773	4371311	99.94	0.00	Yes
NCBI Assembly	GCA_004110045.1	NCBI: cellular organisms; Bacteria; Terrabacteria group; Actinobacteria; Actinomycetia; Corynebacteriales; Mycobacteriaceae; Mycobacterium; Mycobacterium tuberculosis complex; Mycobacterium tuberculosis;	NCBI: 1773	4390991	99.94	0.00	Yes
NCBI Assembly	GCA_003398625.1	NCBI: cellular organisms; Bacteria; Terrabacteria group; Actinobacteria; Actinomycetia; Corynebacteriales; Mycobacteriaceae; Mycobacterium; Mycobacterium tuberculosis complex; Mycobacterium tuberculosis;	NCBI: 1773	4388374	99.86	0.00	Yes
NCBI Assembly	GCA_003284965.1	NCBI: cellular organisms; Bacteria; Terrabacteria group; Actinobacteria; Actinomycetia; Corynebacteriales; Mycobacteriaceae; Mycolicibacterium; unclassified Mycolicibacterium;	NCBI: 2267251	5329052	100.00	0.45	Yes
NCBI Assembly	GCA_004110645.1	NCBI: cellular organisms; Bacteria; Terrabacteria group; Actinobacteria; Actinomycetia; Corynebacteriales; Mycobacteriaceae; Mycobacterium; Mycobacterium tuberculosis complex; Mycobacterium tuberculosis;	NCBI: 1773	4386082	99.61	0.00	Yes
NCBI Assembly	GCA_004112845.1	NCBI: cellular organisms; Bacteria; Terrabacteria group; Actinobacteria; Actinomycetia; Corynebacteriales; Mycobacteriaceae; Mycobacterium; Mycobacterium tuberculosis complex; Mycobacterium tuberculosis;	NCBI: 1773	4374679	99.94	0.00	Yes
NCBI Assembly	GCA_003393775.1	NCBI: cellular organisms; Bacteria; Terrabacteria group; Actinobacteria; Actinomycetia; Corynebacteriales; Mycobacteriaceae; Mycobacterium; Mycobacterium tuberculosis complex; Mycobacterium tuberculosis;	NCBI: 1773	4307095	99.86	0.00	Yes
NCBI Assembly	GCA_004105265.1	NCBI: cellular organisms; Bacteria; Terrabacteria group; Actinobacteria; Actinomycetia; Corynebacteriales; Mycobacteriaceae; Mycobacterium; Mycobacterium tuberculosis complex; Mycobacterium tuberculosis;	NCBI: 1773	4402363	99.61	0.00	Yes
NCBI Assembly	GCA_003398965.1	NCBI: cellular organisms; Bacteria; Terrabacteria group; Actinobacteria; Actinomycetia; Corynebacteriales; Mycobacteriaceae; Mycobacterium; Mycobacterium tuberculosis complex; Mycobacterium tuberculosis;	NCBI: 1773	4294464	99.36	0.05	Yes

NCBI Assembly	GCA_004110375.1	NCBI: cellular organisms; Bacteria; Terrabacteria group; Actinobacteria; Actinomycetia; Corynebacteriales; Mycobacteriaceae; Mycobacterium; Mycobacterium tuberculosis complex; Mycobacterium tuberculosis;	NCBI: 1773	4391842	99.94	0.00	Yes
NCBI Assembly	GCA_004105885.1	NCBI: cellular organisms; Bacteria; Terrabacteria group; Actinobacteria; Actinomycetia; Corynebacteriales; Mycobacteriaceae; Mycobacterium; Mycobacterium tuberculosis complex; Mycobacterium tuberculosis;	NCBI: 1773	4401211	99.94	0.00	Yes
NCBI Assembly	GCA_003284975.1	NCBI: cellular organisms; Bacteria; Terrabacteria group; Actinobacteria; Actinomycetia; Corynebacteriales; Mycobacteriaceae; Mycobacterium; Mycobacterium avium complex (MAC); Mycobacterium colombiense;	NCBI: 339268	5426817	97.53	0.44	Yes
NCBI Assembly	GCA_004110965.1	NCBI: cellular organisms; Bacteria; Terrabacteria group; Actinobacteria; Actinomycetia; Corynebacteriales; Mycobacteriaceae; Mycobacterium; Mycobacterium tuberculosis complex; Mycobacterium tuberculosis;	NCBI: 1773	4378450	99.94	0.00	Yes
NCBI Assembly	GCA_004109055.1	NCBI: cellular organisms; Bacteria; Terrabacteria group; Actinobacteria; Actinomycetia; Corynebacteriales; Mycobacteriaceae; Mycobacterium; Mycobacterium tuberculosis complex; Mycobacterium tuberculosis;	NCBI: 1773	4384195	99.90	0.00	Yes
NCBI Assembly	GCA_003398635.1	NCBI: cellular organisms; Bacteria; Terrabacteria group; Actinobacteria; Actinomycetia; Corynebacteriales; Mycobacteriaceae; Mycobacterium; Mycobacterium tuberculosis complex; Mycobacterium tuberculosis;	NCBI: 1773	4350494	99.86	0.00	Yes
NCBI Assembly	GCA_004112875.1	NCBI: cellular organisms; Bacteria; Terrabacteria group; Actinobacteria; Actinomycetia; Corynebacteriales; Mycobacteriaceae; Mycobacterium; Mycobacterium tuberculosis complex; Mycobacterium tuberculosis;	NCBI: 1773	4402420	99.94	0.00	Yes
NCBI Assembly	GCA_004110665.1	NCBI: cellular organisms; Bacteria; Terrabacteria group; Actinobacteria; Actinomycetia; Corynebacteriales; Mycobacteriaceae; Mycobacterium; Mycobacterium tuberculosis complex; Mycobacterium tuberculosis;	NCBI: 1773	4393384	99.94	0.00	Yes
NCBI Assembly	GCA_004110055.1	NCBI: cellular organisms; Bacteria; Terrabacteria group; Actinobacteria; Actinomycetia; Corynebacteriales; Mycobacteriaceae; Mycobacterium; Mycobacterium tuberculosis complex; Mycobacterium tuberculosis;	NCBI: 1773	4347530	99.94	0.00	Yes
NCBI Assembly	GCA_003393785.1	NCBI: cellular organisms; Bacteria; Terrabacteria group; Actinobacteria; Actinomycetia; Corynebacteriales; Mycobacteriaceae; Mycobacterium; Mycobacterium tuberculosis complex; Mycobacterium tuberculosis;	NCBI: 1773	4376136	99.94	0.33	Yes
NCBI Assembly	GCA_004105275.1	NCBI: cellular organisms; Bacteria; Terrabacteria group; Actinobacteria; Actinomycetia; Corynebacteriales; Mycobacteriaceae; Mycobacterium; Mycobacterium tuberculosis complex; Mycobacterium tuberculosis;	NCBI: 1773	4406781	99.94	0.00	Yes
NCBI Assembly	GCA_003398985.1	NCBI: cellular organisms; Bacteria; Terrabacteria group; Actinobacteria; Actinomycetia; Corynebacteriales; Mycobacteriaceae; Mycobacterium; Mycobacterium tuberculosis complex; Mycobacterium tuberculosis;	NCBI: 1773	4292223	99.94	0.17	Yes
NCBI Assembly	GCA_004110425.1	NCBI: cellular organisms; Bacteria; Terrabacteria group; Actinobacteria; Actinomycetia; Corynebacteriales; Mycobacteriaceae; Mycobacterium; Mycobacterium tuberculosis complex; Mycobacterium tuberculosis;	NCBI: 1773	4379520	99.61	0.00	Yes
NCBI Assembly	GCA_004105895.1	NCBI: cellular organisms; Bacteria; Terrabacteria group; Actinobacteria; Actinomycetia; Corynebacteriales; Mycobacteriaceae; Mycobacterium; Mycobacterium tuberculosis complex; Mycobacterium tuberculosis;	NCBI: 1773	4389739	99.94	0.00	Yes
NCBI Assembly	GCA_003287125.1	NCBI: cellular organisms; Bacteria; Terrabacteria group; Actinobacteria; Actinomycetia; Corynebacteriales; Mycobacteriaceae; Mycobacterium; Mycobacterium tuberculosis complex; Mycobacterium tuberculosis;	NCBI: 1773	4416938	99.94	0.00	Yes
NCBI Assembly	GCA_003398645.1	NCBI: cellular organisms; Bacteria; Terrabacteria group; Actinobacteria; Actinomycetia; Corynebacteriales; Mycobacteriaceae; Mycobacterium; Mycobacterium tuberculosis complex; Mycobacterium tuberculosis;	NCBI: 1773	4354507	99.86	0.17	Yes
NCBI Assembly	GCA_004110985.1	NCBI: cellular organisms; Bacteria; Terrabacteria group; Actinobacteria; Actinomycetia; Corynebacteriales; Mycobacteriaceae; Mycobacterium; Mycobacterium tuberculosis complex; Mycobacterium tuberculosis;	NCBI: 1773	4382967	99.94	0.00	Yes
NCBI Assembly	GCA_004109105.1	NCBI: cellular organisms; Bacteria; Terrabacteria group; Actinobacteria; Actinomycetia; Corynebacteriales; Mycobacteriaceae; Mycobacterium; Mycobacterium tuberculosis complex; Mycobacterium tuberculosis;	NCBI: 1773	4389704	99.94	0.00	Yes

NCBI Assembly	GCA_004110695.1	NCBI: cellular organisms; Bacteria; Terrabacteria group; Actinobacteria; Actinomycetia; Corynebacteriales; Mycobacteriaceae; Mycobacterium; Mycobacterium tuberculosis complex; Mycobacterium tuberculosis;	NCBI: 1773	4362865	99.94	0.00	Yes
NCBI Assembly	GCA_004110075.1	NCBI: cellular organisms; Bacteria; Terrabacteria group; Actinobacteria; Actinomycetia; Corynebacteriales; Mycobacteriaceae; Mycobacterium; Mycobacterium tuberculosis complex; Mycobacterium tuberculosis;	NCBI: 1773	4378543	99.94	0.00	Yes
NCBI Assembly	GCA_004112885.1	NCBI: cellular organisms; Bacteria; Terrabacteria group; Actinobacteria; Actinomycetia; Corynebacteriales; Mycobacteriaceae; Mycobacterium; Mycobacterium tuberculosis complex; Mycobacterium tuberculosis;	NCBI: 1773	4383744	99.94	0.00	Yes
NCBI Assembly	GCA_003393795.1	NCBI: cellular organisms; Bacteria; Terrabacteria group; Actinobacteria; Actinomycetia; Corynebacteriales; Mycobacteriaceae; Mycobacterium; Mycobacterium tuberculosis complex; Mycobacterium tuberculosis;	NCBI: 1773	4276201	99.79	0.17	Yes
NCBI Assembly	GCA_003399005.1	NCBI: cellular organisms; Bacteria; Terrabacteria group; Actinobacteria; Actinomycetia; Corynebacteriales; Mycobacteriaceae; Mycobacterium; Mycobacterium tuberculosis complex; Mycobacterium tuberculosis;	NCBI: 1773	4268390	99.53	0.76	Yes
NCBI Assembly	GCA_004105285.1	NCBI: cellular organisms; Bacteria; Terrabacteria group; Actinobacteria; Actinomycetia; Corynebacteriales; Mycobacteriaceae; Mycobacterium; Mycobacterium tuberculosis complex; Mycobacterium tuberculosis;	NCBI: 1773	4392970	99.94	0.00	Yes
NCBI Assembly	GCA_004110435.1	NCBI: cellular organisms; Bacteria; Terrabacteria group; Actinobacteria; Actinomycetia; Corynebacteriales; Mycobacteriaceae; Mycobacterium; Mycobacterium tuberculosis complex; Mycobacterium tuberculosis;	NCBI: 1773	4396229	99.94	0.00	Yes
NCBI Assembly	GCA_004105905.1	NCBI: cellular organisms; Bacteria; Terrabacteria group; Actinobacteria; Actinomycetia; Corynebacteriales; Mycobacteriaceae; Mycobacterium; Mycobacterium tuberculosis complex; Mycobacterium tuberculosis;	NCBI: 1773	4395127	99.94	0.00	Yes
NCBI Assembly	GCA_003398655.1	NCBI: cellular organisms; Bacteria; Terrabacteria group; Actinobacteria; Actinomycetia; Corynebacteriales; Mycobacteriaceae; Mycobacterium; Mycobacterium tuberculosis complex; Mycobacterium tuberculosis;	NCBI: 1773	4376059	99.86	0.00	Yes
NCBI Assembly	GCA_003287145.1	NCBI: cellular organisms; Bacteria; Terrabacteria group; Actinobacteria; Actinomycetia; Corynebacteriales; Mycobacteriaceae; Mycobacterium; Mycobacterium tuberculosis complex; Mycobacterium tuberculosis;	NCBI: 1773	4413214	99.94	0.00	Yes
NCBI Assembly	GCA_004111025.1	NCBI: cellular organisms; Bacteria; Terrabacteria group; Actinobacteria; Actinomycetia; Corynebacteriales; Mycobacteriaceae; Mycobacterium; Mycobacterium tuberculosis complex; Mycobacterium tuberculosis;	NCBI: 1773	4385716	99.94	0.00	Yes
NCBI Assembly	GCA_004109125.1	NCBI: cellular organisms; Bacteria; Terrabacteria group; Actinobacteria; Actinomycetia; Corynebacteriales; Mycobacteriaceae; Mycobacterium; Mycobacterium tuberculosis complex; Mycobacterium tuberculosis;	NCBI: 1773	4365143	99.28	0.00	Yes
NCBI Assembly	GCA_004110125.1	NCBI: cellular organisms; Bacteria; Terrabacteria group; Actinobacteria; Actinomycetia; Corynebacteriales; Mycobacteriaceae; Mycobacterium; Mycobacterium tuberculosis complex; Mycobacterium tuberculosis;	NCBI: 1773	4361820	99.94	0.00	Yes
NCBI Assembly	GCA_004110725.1	NCBI: cellular organisms; Bacteria; Terrabacteria group; Actinobacteria; Actinomycetia; Corynebacteriales; Mycobacteriaceae; Mycobacterium; Mycobacterium tuberculosis complex; Mycobacterium tuberculosis;	NCBI: 1773	4391788	99.94	0.00	Yes
NCBI Assembly	GCA_004112925.1	NCBI: cellular organisms; Bacteria; Terrabacteria group; Actinobacteria; Actinomycetia; Corynebacteriales; Mycobacteriaceae; Mycobacterium; Mycobacterium tuberculosis complex; Mycobacterium tuberculosis;	NCBI: 1773	4375494	99.61	0.00	Yes
NCBI Assembly	GCA_003393845.1	NCBI: cellular organisms; Bacteria; Terrabacteria group; Actinobacteria; Actinomycetia; Corynebacteriales; Mycobacteriaceae; Mycobacterium; Mycobacterium tuberculosis complex; Mycobacterium tuberculosis;	NCBI: 1773	4243563	99.42	0.17	Yes
NCBI Assembly	GCA_003399015.1	NCBI: cellular organisms; Bacteria; Terrabacteria group; Actinobacteria; Actinomycetia; Corynebacteriales; Mycobacteriaceae; Mycobacterium; Mycobacterium tuberculosis complex; Mycobacterium tuberculosis;	NCBI: 1773	4369427	99.94	0.00	Yes
NCBI Assembly	GCA_004105315.1	NCBI: cellular organisms; Bacteria; Terrabacteria group; Actinobacteria; Actinomycetia; Corynebacteriales; Mycobacteriaceae; Mycobacterium; Mycobacterium tuberculosis complex; Mycobacterium tuberculosis;	NCBI: 1773	4403514	99.94	0.00	Yes

NCBI Assembly	GCA_003398705.1	NCBI: cellular organisms; Bacteria; Terrabacteria group; Actinobacteria; Actinomycetia; Corynebacteriales; Mycobacteriaceae; Mycobacterium; Mycobacterium tuberculosis complex; Mycobacterium tuberculosis;	NCBI: 1773	4393966	99.86	0.00	Yes
NCBI Assembly	GCA_004105965.1	NCBI: cellular organisms; Bacteria; Terrabacteria group; Actinobacteria; Actinomycetia; Corynebacteriales; Mycobacteriaceae; Mycobacterium; Mycobacterium tuberculosis complex; Mycobacterium tuberculosis;	NCBI: 1773	4376819	99.61	0.00	Yes
NCBI Assembly	GCA_004110455.1	NCBI: cellular organisms; Bacteria; Terrabacteria group; Actinobacteria; Actinomycetia; Corynebacteriales; Mycobacteriaceae; Mycobacterium; Mycobacterium tuberculosis complex; Mycobacterium tuberculosis;	NCBI: 1773	4396332	99.94	0.00	Yes
NCBI Assembly	GCA_003287165.1	NCBI: cellular organisms; Bacteria; Terrabacteria group; Actinobacteria; Actinomycetia; Corynebacteriales; Mycobacteriaceae; Mycobacterium; Mycobacterium tuberculosis complex; Mycobacterium tuberculosis;	NCBI: 1773	4418796	99.94	0.00	Yes
NCBI Assembly	GCA_004109135.1	NCBI: cellular organisms; Bacteria; Terrabacteria group; Actinobacteria; Actinomycetia; Corynebacteriales; Mycobacteriaceae; Mycobacterium; Mycobacterium tuberculosis complex; Mycobacterium tuberculosis;	NCBI: 1773	4377933	99.94	0.00	Yes
NCBI Assembly	GCA_004111035.1	NCBI: cellular organisms; Bacteria; Terrabacteria group; Actinobacteria; Actinomycetia; Corynebacteriales; Mycobacteriaceae; Mycobacterium; Mycobacterium tuberculosis complex; Mycobacterium tuberculosis;	NCBI: 1773	4373685	99.94	0.00	Yes
NCBI Assembly	GCA_003393855.1	NCBI: cellular organisms; Bacteria; Terrabacteria group; Actinobacteria; Actinomycetia; Corynebacteriales; Mycobacteriaceae; Mycobacterium; Mycobacterium tuberculosis complex; Mycobacterium tuberculosis;	NCBI: 1773	4361243	99.86	0.00	Yes
NCBI Assembly	GCA_004110135.1	NCBI: cellular organisms; Bacteria; Terrabacteria group; Actinobacteria; Actinomycetia; Corynebacteriales; Mycobacteriaceae; Mycobacterium; Mycobacterium tuberculosis complex; Mycobacterium tuberculosis;	NCBI: 1773	4395721	99.94	0.00	Yes
NCBI Assembly	GCA_004112935.1	NCBI: cellular organisms; Bacteria; Terrabacteria group; Actinobacteria; Actinomycetia; Corynebacteriales; Mycobacteriaceae; Mycobacterium; Mycobacterium tuberculosis complex; Mycobacterium tuberculosis;	NCBI: 1773	4389154	99.94	0.00	Yes
NCBI Assembly	GCA_004110735.1	NCBI: cellular organisms; Bacteria; Terrabacteria group; Actinobacteria; Actinomycetia; Corynebacteriales; Mycobacteriaceae; Mycobacterium; Mycobacterium tuberculosis complex; Mycobacterium tuberculosis;	NCBI: 1773	4377661	99.94	0.00	Yes
NCBI Assembly	GCA_003399035.1	NCBI: cellular organisms; Bacteria; Terrabacteria group; Actinobacteria; Actinomycetia; Corynebacteriales; Mycobacteriaceae; Mycobacterium; Mycobacterium tuberculosis complex; Mycobacterium tuberculosis;	NCBI: 1773	4152548	98.10	1.40	Yes
NCBI Assembly	GCA_003398715.1	NCBI: cellular organisms; Bacteria; Terrabacteria group; Actinobacteria; Actinomycetia; Corynebacteriales; Mycobacteriaceae; Mycobacterium; Mycobacterium tuberculosis complex; Mycobacterium tuberculosis;	NCBI: 1773	4352054	99.86	0.00	Yes
NCBI Assembly	GCA_004105335.1	NCBI: cellular organisms; Bacteria; Terrabacteria group; Actinobacteria; Actinomycetia; Corynebacteriales; Mycobacteriaceae; Mycobacterium; Mycobacterium tuberculosis complex; Mycobacterium tuberculosis;	NCBI: 1773	4378027	99.94	0.00	Yes
NCBI Assembly	GCA_004110465.1	NCBI: cellular organisms; Bacteria; Terrabacteria group; Actinobacteria; Actinomycetia; Corynebacteriales; Mycobacteriaceae; Mycobacterium; Mycobacterium tuberculosis complex; Mycobacterium tuberculosis;	NCBI: 1773	4395242	99.94	0.00	Yes
NCBI Assembly	GCA_003287185.1	NCBI: cellular organisms; Bacteria; Terrabacteria group; Actinobacteria; Actinomycetia; Corynebacteriales; Mycobacteriaceae; Mycobacterium; Mycobacterium tuberculosis complex; Mycobacterium tuberculosis;	NCBI: 1773	4406346	99.94	0.00	Yes
NCBI Assembly	GCA_004105975.1	NCBI: cellular organisms; Bacteria; Terrabacteria group; Actinobacteria; Actinomycetia; Corynebacteriales; Mycobacteriaceae; Mycobacterium; Mycobacterium tuberculosis complex; Mycobacterium tuberculosis;	NCBI: 1773	4406447	99.61	0.00	Yes
NCBI Assembly	GCA_004111045.1	NCBI: cellular organisms; Bacteria; Terrabacteria group; Actinobacteria; Actinomycetia; Corynebacteriales; Mycobacteriaceae; Mycobacterium; Mycobacterium tuberculosis complex; Mycobacterium tuberculosis;	NCBI: 1773	4374996	99.94	0.00	Yes
NCBI Assembly	GCA_003393865.1	NCBI: cellular organisms; Bacteria; Terrabacteria group; Actinobacteria; Actinomycetia; Corynebacteriales; Mycobacteriaceae; Mycobacterium; Mycobacterium tuberculosis complex; Mycobacterium tuberculosis;	NCBI: 1773	4135238	94.69	3.94	Yes

NCBI Assembly	GCA_004109145.1	NCBI: cellular organisms; Bacteria; Terrabacteria group; Actinobacteria; Actinomycetia; Corynebacteriales; Mycobacteriaceae; Mycobacterium; Mycobacterium tuberculosis complex; Mycobacterium tuberculosis;	NCBI: 1773	4400687	99.94	0.00	Yes
NCBI Assembly	GCA_004110155.1	NCBI: cellular organisms; Bacteria; Terrabacteria group; Actinobacteria; Actinomycetia; Corynebacteriales; Mycobacteriaceae; Mycobacterium; Mycobacterium tuberculosis complex; Mycobacterium tuberculosis;	NCBI: 1773	4395339	99.94	0.00	Yes
NCBI Assembly	GCA_004112945.1	NCBI: cellular organisms; Bacteria; Terrabacteria group; Actinobacteria; Actinomycetia; Corynebacteriales; Mycobacteriaceae; Mycobacterium; Mycobacterium tuberculosis complex; Mycobacterium tuberculosis;	NCBI: 1773	4400052	99.94	0.00	Yes
NCBI Assembly	GCA_003399065.1	NCBI: cellular organisms; Bacteria; Terrabacteria group; Actinobacteria; Actinomycetia; Corynebacteriales; Mycobacteriaceae; Mycobacterium; Mycobacterium tuberculosis complex; Mycobacterium tuberculosis;	NCBI: 1773	4323042	99.86	0.00	Yes
NCBI Assembly	GCA_004110745.1	NCBI: cellular organisms; Bacteria; Terrabacteria group; Actinobacteria; Actinomycetia; Corynebacteriales; Mycobacteriaceae; Mycobacterium; Mycobacterium tuberculosis complex; Mycobacterium tuberculosis;	NCBI: 1773	4398002	99.94	0.00	Yes
NCBI Assembly	GCA_003398745.1	NCBI: cellular organisms; Bacteria; Terrabacteria group; Actinobacteria; Actinomycetia; Corynebacteriales; Mycobacteriaceae; Mycobacterium; Mycobacterium tuberculosis complex; Mycobacterium tuberculosis;	NCBI: 1773	4275774	99.53	0.07	Yes
NCBI Assembly	GCA_003293655.1	NCBI: cellular organisms; Bacteria; Terrabacteria group; Actinobacteria; Actinomycetia; Corynebacteriales; Mycobacteriaceae; Mycobacterium; Mycobacterium tuberculosis complex; Mycobacterium tuberculosis;	NCBI: 1773	4359659	99.94	0.00	Yes
NCBI Assembly	GCA_004105365.1	NCBI: cellular organisms; Bacteria; Terrabacteria group; Actinobacteria; Actinomycetia; Corynebacteriales; Mycobacteriaceae; Mycobacterium; Mycobacterium tuberculosis complex; Mycobacterium tuberculosis;	NCBI: 1773	4393953	99.94	0.00	Yes
NCBI Assembly	GCA_004110475.1	NCBI: cellular organisms; Bacteria; Terrabacteria group; Actinobacteria; Actinomycetia; Corynebacteriales; Mycobacteriaceae; Mycobacterium; Mycobacterium tuberculosis complex; Mycobacterium tuberculosis;	NCBI: 1773	4390045	99.61	0.00	Yes
NCBI Assembly	GCA_004105985.1	NCBI: cellular organisms; Bacteria; Terrabacteria group; Actinobacteria; Actinomycetia; Corynebacteriales; Mycobacteriaceae; Mycobacterium; Mycobacterium tuberculosis complex; Mycobacterium tuberculosis;	NCBI: 1773	4386298	99.94	0.00	Yes
NCBI Assembly	GCA_004111055.1	NCBI: cellular organisms; Bacteria; Terrabacteria group; Actinobacteria; Actinomycetia; Corynebacteriales; Mycobacteriaceae; Mycobacterium; Mycobacterium tuberculosis complex; Mycobacterium tuberculosis;	NCBI: 1773	4369277	99.94	0.00	Yes
NCBI Assembly	GCA_003393885.1	NCBI: cellular organisms; Bacteria; Terrabacteria group; Actinobacteria; Actinomycetia; Corynebacteriales; Mycobacteriaceae; Mycobacterium; Mycobacterium tuberculosis complex; Mycobacterium tuberculosis;	NCBI: 1773	4375429	99.86	0.00	Yes
NCBI Assembly	GCA_004110185.1	NCBI: cellular organisms; Bacteria; Terrabacteria group; Actinobacteria; Actinomycetia; Corynebacteriales; Mycobacteriaceae; Mycobacterium; Mycobacterium tuberculosis complex; Mycobacterium tuberculosis;	NCBI: 1773	4399814	99.94	0.00	Yes
NCBI Assembly	GCA_004109165.1	NCBI: cellular organisms; Bacteria; Terrabacteria group; Actinobacteria; Actinomycetia; Corynebacteriales; Mycobacteriaceae; Mycobacterium; Mycobacterium tuberculosis complex; Mycobacterium tuberculosis;	NCBI: 1773	4397778	99.94	0.00	Yes
NCBI Assembly	GCA_003399085.1	NCBI: cellular organisms; Bacteria; Terrabacteria group; Actinobacteria; Actinomycetia; Corynebacteriales; Mycobacteriaceae; Mycobacterium; Mycobacterium tuberculosis complex; Mycobacterium tuberculosis;	NCBI: 1773	4374761	99.86	0.00	Yes
NCBI Assembly	GCA_004112955.1	NCBI: cellular organisms; Bacteria; Terrabacteria group; Actinobacteria; Actinomycetia; Corynebacteriales; Mycobacteriaceae; Mycobacterium; Mycobacterium tuberculosis complex; Mycobacterium tuberculosis;	NCBI: 1773	4386163	99.28	0.00	Yes
NCBI Assembly	GCA_004110755.1	NCBI: cellular organisms; Bacteria; Terrabacteria group; Actinobacteria; Actinomycetia; Corynebacteriales; Mycobacteriaceae; Mycobacterium; Mycobacterium tuberculosis complex; Mycobacterium tuberculosis;	NCBI: 1773	4382996	99.94	0.00	Yes
NCBI Assembly	GCA_003398755.1	NCBI: cellular organisms; Bacteria; Terrabacteria group; Actinobacteria; Actinomycetia; Corynebacteriales; Mycobacteriaceae; Mycobacterium; Mycobacterium tuberculosis complex; Mycobacterium tuberculosis;	NCBI: 1773	4394824	99.86	1.17	Yes

NCBI Assembly	GCA_003311705.1	NCBI: cellular organisms; Bacteria; Terrabacteria group; Actinobacteria; Actinomycetia; Corynebacteriales; Mycobacteriaceae; Mycobacterium; Mycobacterium tuberculosis complex; Mycobacterium tuberculosis;	NCBI: 1773	4377155	99.94	0.00	Yes
NCBI Assembly	GCA_004105375.1	NCBI: cellular organisms; Bacteria; Terrabacteria group; Actinobacteria; Actinomycetia; Corynebacteriales; Mycobacteriaceae; Mycobacterium; Mycobacterium tuberculosis complex; Mycobacterium tuberculosis;	NCBI: 1773	4392787	99.94	0.00	Yes
NCBI Assembly	GCA_004110525.1	NCBI: cellular organisms; Bacteria; Terrabacteria group; Actinobacteria; Actinomycetia; Corynebacteriales; Mycobacteriaceae; Mycobacterium; Mycobacterium tuberculosis complex; Mycobacterium tuberculosis;	NCBI: 1773	4393811	99.94	0.00	Yes
NCBI Assembly	GCA_004105995.1	NCBI: cellular organisms; Bacteria; Terrabacteria group; Actinobacteria; Actinomycetia; Corynebacteriales; Mycobacteriaceae; Mycobacterium; Mycobacterium tuberculosis complex; Mycobacterium tuberculosis;	NCBI: 1773	4393504	99.94	0.00	Yes
NCBI Assembly	GCA_003393915.1	NCBI: cellular organisms; Bacteria; Terrabacteria group; Actinobacteria; Actinomycetia; Corynebacteriales; Mycobacteriaceae; Mycobacterium; Mycobacterium tuberculosis complex; Mycobacterium tuberculosis;	NCBI: 1773	4355134	99.86	0.67	Yes
NCBI Assembly	GCA_003399105.1	NCBI: cellular organisms; Bacteria; Terrabacteria group; Actinobacteria; Actinomycetia; Corynebacteriales; Mycobacteriaceae; Mycobacterium; Mycobacterium tuberculosis complex; Mycobacterium tuberculosis;	NCBI: 1773	4217695	99.36	1.17	Yes
NCBI Assembly	GCA_004111085.1	NCBI: cellular organisms; Bacteria; Terrabacteria group; Actinobacteria; Actinomycetia; Corynebacteriales; Mycobacteriaceae; Mycobacterium; Mycobacterium tuberculosis complex; Mycobacterium tuberculosis;	NCBI: 1773	4397117	99.94	0.00	Yes
NCBI Assembly	GCA_004110195.1	NCBI: cellular organisms; Bacteria; Terrabacteria group; Actinobacteria; Actinomycetia; Corynebacteriales; Mycobacteriaceae; Mycobacterium; Mycobacterium tuberculosis complex; Mycobacterium tuberculosis;	NCBI: 1773	4371568	99.94	0.00	Yes
NCBI Assembly	GCA_004113005.1	NCBI: cellular organisms; Bacteria; Terrabacteria group; Actinobacteria; Actinomycetia; Corynebacteriales; Mycobacteriaceae; Mycobacterium; Mycobacterium tuberculosis complex; Mycobacterium tuberculosis;	NCBI: 1773	4398446	99.94	0.00	Yes
NCBI Assembly	GCA_004109195.1	NCBI: cellular organisms; Bacteria; Terrabacteria group; Actinobacteria; Actinomycetia; Corynebacteriales; Mycobacteriaceae; Mycobacterium; Mycobacterium tuberculosis complex; Mycobacterium tuberculosis;	NCBI: 1773	4401689	99.61	0.00	Yes
NCBI Assembly	GCA_004110795.1	NCBI: cellular organisms; Bacteria; Terrabacteria group; Actinobacteria; Actinomycetia; Corynebacteriales; Mycobacteriaceae; Mycobacterium; Mycobacterium tuberculosis complex; Mycobacterium tuberculosis;	NCBI: 1773	4373540	99.61	0.00	Yes
NCBI Assembly	GCA_003044235.1	NCBI: cellular organisms; Bacteria; Terrabacteria group; Actinobacteria; Actinomycetia; Corynebacteriales; Mycobacteriaceae; Mycobacterium; Mycobacterium tuberculosis complex; Mycobacterium tuberculosis;	NCBI: 1773	4394577	98.94	0.00	Yes
NCBI Assembly	GCA_003398765.1	NCBI: cellular organisms; Bacteria; Terrabacteria group; Actinobacteria; Actinomycetia; Corynebacteriales; Mycobacteriaceae; Mycobacterium; Mycobacterium tuberculosis complex; Mycobacterium tuberculosis;	NCBI: 1773	4298633	99.86	0.11	Yes
NCBI Assembly	GCA_004110535.1	NCBI: cellular organisms; Bacteria; Terrabacteria group; Actinobacteria; Actinomycetia; Corynebacteriales; Mycobacteriaceae; Mycobacterium; Mycobacterium tuberculosis complex; Mycobacterium tuberculosis;	NCBI: 1773	4372587	99.94	0.00	Yes
NCBI Assembly	GCA_004105385.1	NCBI: cellular organisms; Bacteria; Terrabacteria group; Actinobacteria; Actinomycetia; Corynebacteriales; Mycobacteriaceae; Mycobacterium; Mycobacterium tuberculosis complex; Mycobacterium tuberculosis;	NCBI: 1773	4395323	99.94	0.00	Yes
NCBI Assembly	GCA_004106025.1	NCBI: cellular organisms; Bacteria; Terrabacteria group; Actinobacteria; Actinomycetia; Corynebacteriales; Mycobacteriaceae; Mycobacterium; Mycobacterium tuberculosis complex; Mycobacterium tuberculosis;	NCBI: 1773	4394730	99.94	0.00	Yes
NCBI Assembly	GCA_003393325.1	NCBI: cellular organisms; Bacteria; Terrabacteria group; Actinobacteria; Actinomycetia; Corynebacteriales; Mycobacteriaceae; Mycobacterium; Mycobacterium tuberculosis complex; Mycobacterium tuberculosis;	NCBI: 1773	4345281	99.86	0.00	Yes
NCBI Assembly	GCA_004111125.1	NCBI: cellular organisms; Bacteria; Terrabacteria group; Actinobacteria; Actinomycetia; Corynebacteriales; Mycobacteriaceae; Mycobacterium; Mycobacterium tuberculosis complex; Mycobacterium tuberculosis;	NCBI: 1773	4394052	99.61	0.00	Yes

NCBI Assembly	GCA_004108315.1	NCBI: cellular organisms; Bacteria; Terrabacteria group; Actinobacteria; Actinomycetia; Corynebacteriales; Mycobacteriaceae; Mycobacterium; Mycobacterium tuberculosis complex; Mycobacterium tuberculosis;	NCBI: 1773	4369480	99.94	0.00	Yes
NCBI Assembly	GCA_004110225.1	NCBI: cellular organisms; Bacteria; Terrabacteria group; Actinobacteria; Actinomycetia; Corynebacteriales; Mycobacteriaceae; Mycobacterium; Mycobacterium tuberculosis complex; Mycobacterium tuberculosis;	NCBI: 1773	4378151	99.94	0.00	Yes
NCBI Assembly	GCA_004109215.1	NCBI: cellular organisms; Bacteria; Terrabacteria group; Actinobacteria; Actinomycetia; Corynebacteriales; Mycobacteriaceae; Mycobacterium; Mycobacterium tuberculosis complex; Mycobacterium tuberculosis;	NCBI: 1773	4394006	99.94	0.00	Yes
NCBI Assembly	GCA_004113025.1	NCBI: cellular organisms; Bacteria; Terrabacteria group; Actinobacteria; Actinomycetia; Corynebacteriales; Mycobacteriaceae; Mycobacterium; Mycobacterium tuberculosis complex; Mycobacterium tuberculosis;	NCBI: 1773	4406946	99.61	0.00	Yes
NCBI Assembly	GCA_003049125.1	NCBI: cellular organisms; Bacteria; Terrabacteria group; Actinobacteria; Actinomycetia; Corynebacteriales; Mycobacteriaceae; Mycobacterium; Mycobacterium tuberculosis complex; Mycobacterium tuberculosis;	NCBI: 1773	4787190	99.86	7.25	No
NCBI Assembly	GCA_004110825.1	NCBI: cellular organisms; Bacteria; Terrabacteria group; Actinobacteria; Actinomycetia; Corynebacteriales; Mycobacteriaceae; Mycobacterium; Mycobacterium tuberculosis complex; Mycobacterium tuberculosis;	NCBI: 1773	4388874	99.94	0.00	Yes
NCBI Assembly	GCA_004112125.1	NCBI: cellular organisms; Bacteria; Terrabacteria group; Actinobacteria; Actinomycetia; Corynebacteriales; Mycobacteriaceae; Mycobacterium; Mycobacterium tuberculosis complex; Mycobacterium tuberculosis;	NCBI: 1773	4378117	99.94	0.00	Yes
NCBI Assembly	GCA_004105395.1	NCBI: cellular organisms; Bacteria; Terrabacteria group; Actinobacteria; Actinomycetia; Corynebacteriales; Mycobacteriaceae; Mycobacterium; Mycobacterium tuberculosis complex; Mycobacterium tuberculosis;	NCBI: 1773	4396430	99.94	0.00	Yes
NCBI Assembly	GCA_004110545.1	NCBI: cellular organisms; Bacteria; Terrabacteria group; Actinobacteria; Actinomycetia; Corynebacteriales; Mycobacteriaceae; Mycobacterium; Mycobacterium tuberculosis complex; Mycobacterium tuberculosis;	NCBI: 1773	4377543	99.94	0.00	Yes
NCBI Assembly	GCA_004106065.1	NCBI: cellular organisms; Bacteria; Terrabacteria group; Actinobacteria; Actinomycetia; Corynebacteriales; Mycobacteriaceae; Mycobacterium; Mycobacterium tuberculosis complex; Mycobacterium tuberculosis;	NCBI: 1773	4390719	99.94	0.00	Yes
NCBI Assembly	GCA_003393335.1	NCBI: cellular organisms; Bacteria; Terrabacteria group; Actinobacteria; Actinomycetia; Corynebacteriales; Mycobacteriaceae; Mycobacterium; Mycobacterium tuberculosis complex; Mycobacterium tuberculosis;	NCBI: 1773	4394401	99.86	0.33	Yes
NCBI Assembly	GCA_004110245.1	NCBI: cellular organisms; Bacteria; Terrabacteria group; Actinobacteria; Actinomycetia; Corynebacteriales; Mycobacteriaceae; Mycobacterium; Mycobacterium tuberculosis complex; Mycobacterium tuberculosis;	NCBI: 1773	4387904	99.94	0.00	Yes
NCBI Assembly	GCA_003049135.1	NCBI: cellular organisms; Bacteria; Terrabacteria group; Actinobacteria; Actinomycetia; Corynebacteriales; Mycobacteriaceae; Mycobacterium; Mycobacterium tuberculosis complex; Mycobacterium tuberculosis;	NCBI: 1773	5111390	99.92	15.68	No
NCBI Assembly	GCA_004111135.1	NCBI: cellular organisms; Bacteria; Terrabacteria group; Actinobacteria; Actinomycetia; Corynebacteriales; Mycobacteriaceae; Mycobacterium; Mycobacterium tuberculosis complex; Mycobacterium tuberculosis;	NCBI: 1773	4382253	99.94	0.00	Yes
NCBI Assembly	GCA_004108345.1	NCBI: cellular organisms; Bacteria; Terrabacteria group; Actinobacteria; Actinomycetia; Corynebacteriales; Mycobacteriaceae; Mycobacterium; Mycobacterium tuberculosis complex; Mycobacterium tuberculosis;	NCBI: 1773	4390089	99.94	0.00	Yes
NCBI Assembly	GCA_004109245.1	NCBI: cellular organisms; Bacteria; Terrabacteria group; Actinobacteria; Actinomycetia; Corynebacteriales; Mycobacteriaceae; Mycobacterium; Mycobacterium tuberculosis complex; Mycobacterium tuberculosis;	NCBI: 1773	4402456	99.61	0.00	Yes
NCBI Assembly	GCA_004113035.1	NCBI: cellular organisms; Bacteria; Terrabacteria group; Actinobacteria; Actinomycetia; Corynebacteriales; Mycobacteriaceae; Mycobacterium; Mycobacterium tuberculosis complex; Mycobacterium tuberculosis;	NCBI: 1773	4394758	99.61	0.00	Yes
NCBI Assembly	GCA_004110835.1	NCBI: cellular organisms; Bacteria; Terrabacteria group; Actinobacteria; Actinomycetia; Corynebacteriales; Mycobacteriaceae; Mycobacterium; Mycobacterium tuberculosis complex; Mycobacterium tuberculosis;	NCBI: 1773	4362386	99.94	0.00	Yes

NCBI Assembly	GCA_004112135.1	NCBI: cellular organisms; Bacteria; Terrabacteria group; Actinobacteria; Actinomycetia; Corynebacteriales; Mycobacteriaceae; Mycobacterium; Mycobacterium tuberculosis complex; Mycobacterium tuberculosis;	NCBI: 1773	4380313	99.94	0.00	Yes
NCBI Assembly	GCA_004105405.1	NCBI: cellular organisms; Bacteria; Terrabacteria group; Actinobacteria; Actinomycetia; Corynebacteriales; Mycobacteriaceae; Mycobacterium; Mycobacterium tuberculosis complex; Mycobacterium tuberculosis;	NCBI: 1773	4398634	99.94	0.00	Yes
NCBI Assembly	GCA_004107375.1	NCBI: cellular organisms; Bacteria; Terrabacteria group; Actinobacteria; Actinomycetia; Corynebacteriales; Mycobacteriaceae; Mycobacterium; Mycobacterium tuberculosis complex; Mycobacterium tuberculosis;	NCBI: 1773	4379862	99.94	0.00	Yes
NCBI Assembly	GCA_003393345.1	NCBI: cellular organisms; Bacteria; Terrabacteria group; Actinobacteria; Actinomycetia; Corynebacteriales; Mycobacteriaceae; Mycobacterium; Mycobacterium tuberculosis complex; Mycobacterium tuberculosis;	NCBI: 1773	4333723	99.86	0.00	Yes
NCBI Assembly	GCA_004106075.1	NCBI: cellular organisms; Bacteria; Terrabacteria group; Actinobacteria; Actinomycetia; Corynebacteriales; Mycobacteriaceae; Mycobacterium; Mycobacterium tuberculosis complex; Mycobacterium tuberculosis;	NCBI: 1773	4385296	99.94	0.00	Yes
NCBI Assembly	GCA_003049195.1	NCBI: cellular organisms; Bacteria; Terrabacteria group; Actinobacteria; Actinomycetia; Corynebacteriales; Mycobacteriaceae; Mycobacterium; Mycobacterium tuberculosis complex; Mycobacterium tuberculosis;	NCBI: 1773	4512388	99.69	0.00	Yes
NCBI Assembly	GCA_002920875.1	NCBI: cellular organisms; Bacteria; Terrabacteria group; Actinobacteria; Actinomycetia; Corynebacteriales; Mycobacteriaceae; Mycobacterium; Mycobacterium kansasii;	NCBI: 1768	6178862	99.61	0.46	Yes
NCBI Assembly	GCA_004111145.1	NCBI: cellular organisms; Bacteria; Terrabacteria group; Actinobacteria; Actinomycetia; Corynebacteriales; Mycobacteriaceae; Mycobacterium; Mycobacterium tuberculosis complex; Mycobacterium tuberculosis;	NCBI: 1773	4390187	99.94	0.00	Yes
NCBI Assembly	GCA_004105485.1	NCBI: cellular organisms; Bacteria; Terrabacteria group; Actinobacteria; Actinomycetia; Corynebacteriales; Mycobacteriaceae; Mycobacterium; Mycobacterium tuberculosis complex; Mycobacterium tuberculosis;	NCBI: 1773	4402269	99.94	0.00	Yes
NCBI Assembly	GCA_004108355.1	NCBI: cellular organisms; Bacteria; Terrabacteria group; Actinobacteria; Actinomycetia; Corynebacteriales; Mycobacteriaceae; Mycobacterium; Mycobacterium tuberculosis complex; Mycobacterium tuberculosis;	NCBI: 1773	4353252	99.94	0.00	Yes
NCBI Assembly	GCA_004113045.1	NCBI: cellular organisms; Bacteria; Terrabacteria group; Actinobacteria; Actinomycetia; Corynebacteriales; Mycobacteriaceae; Mycobacterium; Mycobacterium tuberculosis complex; Mycobacterium tuberculosis;	NCBI: 1773	4384824	99.61	0.00	Yes
NCBI Assembly	GCA_004112145.1	NCBI: cellular organisms; Bacteria; Terrabacteria group; Actinobacteria; Actinomycetia; Corynebacteriales; Mycobacteriaceae; Mycobacterium; Mycobacterium tuberculosis complex; Mycobacterium tuberculosis;	NCBI: 1773	4385559	99.94	0.00	Yes
NCBI Assembly	GCA_004110845.1	NCBI: cellular organisms; Bacteria; Terrabacteria group; Actinobacteria; Actinomycetia; Corynebacteriales; Mycobacteriaceae; Mycobacterium; Mycobacterium tuberculosis complex; Mycobacterium tuberculosis;	NCBI: 1773	4367416	99.94	0.00	Yes
NCBI Assembly	GCA_004105465.1	NCBI: cellular organisms; Bacteria; Terrabacteria group; Actinobacteria; Actinomycetia; Corynebacteriales; Mycobacteriaceae; Mycobacterium; Mycobacterium tuberculosis complex; Mycobacterium tuberculosis;	NCBI: 1773	4413980	99.94	0.00	Yes
NCBI Assembly	GCA_003393365.1	NCBI: cellular organisms; Bacteria; Terrabacteria group; Actinobacteria; Actinomycetia; Corynebacteriales; Mycobacteriaceae; Mycobacterium; Mycobacterium tuberculosis complex; Mycobacterium tuberculosis;	NCBI: 1773	4375774	99.86	1.00	Yes
NCBI Assembly	GCA_004107395.1	NCBI: cellular organisms; Bacteria; Terrabacteria group; Actinobacteria; Actinomycetia; Corynebacteriales; Mycobacteriaceae; Mycobacterium; Mycobacterium tuberculosis complex; Mycobacterium tuberculosis;	NCBI: 1773	4385881	99.61	0.00	Yes
NCBI Assembly	GCA_003049225.1	NCBI: cellular organisms; Bacteria; Terrabacteria group; Actinobacteria; Actinomycetia; Corynebacteriales; Mycobacteriaceae; Mycobacterium; Mycobacterium tuberculosis complex; Mycobacterium tuberculosis;	NCBI: 1773	4367756	99.86	0.47	Yes
NCBI Assembly	GCA_004106085.1	NCBI: cellular organisms; Bacteria; Terrabacteria group; Actinobacteria; Actinomycetia; Corynebacteriales; Mycobacteriaceae; Mycobacterium; Mycobacterium tuberculosis complex; Mycobacterium tuberculosis;	NCBI: 1773	4396360	99.94	0.00	Yes

NCBI Assembly	GCA_002927295.1	NCBI: cellular organisms; Bacteria; Terrabacteria group; Actinobacteria; Actinomycetia; Corynebacteriales; Mycobacteriaceae; Mycobacterium; Mycobacterium avium complex (MAC); Mycobacterium intracellulare; Mycobacterium intracellulare subsp. yongonense;	NCBI: 1203599	5528112	99.61	0.43	Yes
NCBI Assembly	GCA_002802775.1	NCBI: cellular organisms; Bacteria; Terrabacteria group; Actinobacteria; Actinomycetia; Corynebacteriales; Mycobacteriaceae; Mycobacteroides; Mycobacteroides abscessus; Mycobacteroides abscessus subsp. abscessus;	NCBI: 1185650	4843483	100.00	0.00	Yes
NCBI Assembly	GCA_004108365.1	NCBI: cellular organisms; Bacteria; Terrabacteria group; Actinobacteria; Actinomycetia; Corynebacteriales; Mycobacteriaceae; Mycobacterium; Mycobacterium tuberculosis complex; Mycobacterium tuberculosis;	NCBI: 1773	4384203	99.61	0.00	Yes
NCBI Assembly	GCA_004111165.1	NCBI: cellular organisms; Bacteria; Terrabacteria group; Actinobacteria; Actinomycetia; Corynebacteriales; Mycobacteriaceae; Mycobacterium; Mycobacterium tuberculosis complex; Mycobacterium tuberculosis;	NCBI: 1773	4396619	99.61	0.00	Yes
NCBI Assembly	GCA_004105495.1	NCBI: cellular organisms; Bacteria; Terrabacteria group; Actinobacteria; Actinomycetia; Corynebacteriales; Mycobacteriaceae; Mycobacterium; Mycobacterium tuberculosis complex; Mycobacterium tuberculosis;	NCBI: 1773	4387649	99.94	0.00	Yes
NCBI Assembly	GCA_002802405.1	NCBI: cellular organisms; Bacteria; Terrabacteria group; Actinobacteria; Actinomycetia; Corynebacteriales; Mycobacteriaceae; Mycobacteroides; Mycobacteroides abscessus; Mycobacteroides abscessus subsp. abscessus;	NCBI: 1185650	5261484	100.00	0.42	Yes
NCBI Assembly	GCA_004112155.1	NCBI: cellular organisms; Bacteria; Terrabacteria group; Actinobacteria; Actinomycetia; Corynebacteriales; Mycobacteriaceae; Mycobacterium; Mycobacterium tuberculosis complex; Mycobacterium tuberculosis;	NCBI: 1773	4395494	99.61	0.00	Yes
NCBI Assembly	GCA_003393405.1	NCBI: cellular organisms; Bacteria; Terrabacteria group; Actinobacteria; Actinomycetia; Corynebacteriales; Mycobacteriaceae; Mycobacterium; Mycobacterium tuberculosis complex; Mycobacterium tuberculosis;	NCBI: 1773	4323531	99.86	0.17	Yes
NCBI Assembly	GCA_003049245.1	NCBI: cellular organisms; Bacteria; Terrabacteria group; Actinobacteria; Actinomycetia; Corynebacteriales; Mycobacteriaceae; Mycobacterium; Mycobacterium tuberculosis complex; Mycobacterium tuberculosis;	NCBI: 1773	4327264	99.86	0.04	Yes
NCBI Assembly	GCA_004105475.1	NCBI: cellular organisms; Bacteria; Terrabacteria group; Actinobacteria; Actinomycetia; Corynebacteriales; Mycobacteriaceae; Mycobacterium; Mycobacterium tuberculosis complex; Mycobacterium tuberculosis;	NCBI: 1773	4388734	99.94	0.00	Yes
NCBI Assembly	GCA_004107405.1	NCBI: cellular organisms; Bacteria; Terrabacteria group; Actinobacteria; Actinomycetia; Corynebacteriales; Mycobacteriaceae; Mycobacterium; Mycobacterium tuberculosis complex; Mycobacterium tuberculosis;	NCBI: 1773	4394126	99.94	0.00	Yes
NCBI Assembly	GCA_002928215.1	NCBI: cellular organisms; Bacteria; Terrabacteria group; Actinobacteria; Actinomycetia; Corynebacteriales; Mycobacteriaceae; Mycobacterium; Mycobacterium avium complex (MAC); Mycobacterium avium; Mycobacterium avium subsp. hominissuis;	NCBI: 439334	5498075	99.42	11.87	No
NCBI Assembly	GCA_004108005.1	NCBI: cellular organisms; Bacteria; Terrabacteria group; Actinobacteria; Actinomycetia; Corynebacteriales; Mycobacteriaceae; Mycobacterium; Mycobacterium tuberculosis complex; Mycobacterium tuberculosis;	NCBI: 1773	4382643	99.94	0.00	Yes
NCBI Assembly	GCA_002802805.1	NCBI: cellular organisms; Bacteria; Terrabacteria group; Actinobacteria; Actinomycetia; Corynebacteriales; Mycobacteriaceae; Mycobacteroides; Mycobacteroides abscessus; Mycobacteroides abscessus subsp. abscessus;	NCBI: 1185650	5111730	100.00	0.06	Yes
NCBI Assembly	GCA_002886405.1	NCBI: cellular organisms; Bacteria; Terrabacteria group; Actinobacteria; Actinomycetia; Corynebacteriales; Mycobacteriaceae; Mycobacterium; Mycobacterium tuberculosis complex; Mycobacterium tuberculosis;	NCBI: 1773	4411413	99.94	0.00	Yes
NCBI Assembly	GCA_004108375.1	NCBI: cellular organisms; Bacteria; Terrabacteria group; Actinobacteria; Actinomycetia; Corynebacteriales; Mycobacteriaceae; Mycobacterium; Mycobacterium tuberculosis complex; Mycobacterium tuberculosis;	NCBI: 1773	4401295	99.94	0.00	Yes
NCBI Assembly	GCA_002802415.1	NCBI: cellular organisms; Bacteria; Terrabacteria group; Actinobacteria; Actinomycetia; Corynebacteriales; Mycobacteriaceae; Mycobacteroides; Mycobacteroides abscessus; Mycobacteroides abscessus subsp. abscessus;	NCBI: 1185650	5000498	100.00	0.03	Yes
NCBI Assembly	GCA_004105525.1	NCBI: cellular organisms; Bacteria; Terrabacteria group; Actinobacteria; Actinomycetia; Corynebacteriales; Mycobacteriaceae; Mycobacterium; Mycobacterium tuberculosis complex; Mycobacterium tuberculosis;	NCBI: 1773	4395637	99.94	0.00	Yes

NCBI Assembly	GCA_003393425.1	NCBI: cellular organisms; Bacteria; Terrabacteria group; Actinobacteria; Actinomycetia; Corynebacteriales; Mycobacteriaceae; Mycobacterium; Mycobacterium tuberculosis complex; Mycobacterium tuberculosis;	NCBI: 1773	4374993	99.86	0.00	Yes
NCBI Assembly	GCA_003049265.1	NCBI: cellular organisms; Bacteria; Terrabacteria group; Actinobacteria; Actinomycetia; Corynebacteriales; Mycobacteriaceae; Mycobacterium; Mycobacterium tuberculosis complex; Mycobacterium tuberculosis;	NCBI: 1773	4434838	99.53	0.40	Yes
NCBI Assembly	GCA_004112195.1	NCBI: cellular organisms; Bacteria; Terrabacteria group; Actinobacteria; Actinomycetia; Corynebacteriales; Mycobacteriaceae; Mycobacterium; Mycobacterium tuberculosis complex; Mycobacterium tuberculosis;	NCBI: 1773	4379565	99.94	0.00	Yes
NCBI Assembly	GCA_002928225.1	NCBI: cellular organisms; Bacteria; Terrabacteria group; Actinobacteria; Actinomycetia; Corynebacteriales; Mycobacteriaceae; Mycobacterium; Mycobacterium avium complex (MAC); Mycobacterium avium; Mycobacterium avium subsp. hominissuis;	NCBI: 439334	5147757	99.94	0.28	Yes
NCBI Assembly	GCA_004104865.1	NCBI: cellular organisms; Bacteria; Terrabacteria group; Actinobacteria; Actinomycetia; Corynebacteriales; Mycobacteriaceae; Mycobacterium; Mycobacterium tuberculosis complex; Mycobacterium tuberculosis;	NCBI: 1773	4384564	99.61	0.00	Yes
NCBI Assembly	GCA_002802815.1	NCBI: cellular organisms; Bacteria; Terrabacteria group; Actinobacteria; Actinomycetia; Corynebacteriales; Mycobacteriaceae; Mycobacteroides; Mycobacteroides abscessus; Mycobacteroides abscessus subsp. abscessus;	NCBI: 1185650	4918030	99.67	0.03	Yes
NCBI Assembly	GCA_004107445.1	NCBI: cellular organisms; Bacteria; Terrabacteria group; Actinobacteria; Actinomycetia; Corynebacteriales; Mycobacteriaceae; Mycobacterium; Mycobacterium tuberculosis complex; Mycobacterium tuberculosis;	NCBI: 1773	4392808	99.94	0.00	Yes
NCBI Assembly	GCA_004108045.1	NCBI: cellular organisms; Bacteria; Terrabacteria group; Actinobacteria; Actinomycetia; Corynebacteriales; Mycobacteriaceae; Mycobacterium; Mycobacterium tuberculosis complex; Mycobacterium tuberculosis;	NCBI: 1773	4395681	99.61	0.00	Yes
NCBI Assembly	GCA_002886505.1	NCBI: cellular organisms; Bacteria; Terrabacteria group; Actinobacteria; Actinomycetia; Corynebacteriales; Mycobacteriaceae; Mycobacterium; Mycobacterium tuberculosis complex; Mycobacterium tuberculosis;	NCBI: 1773	4411399	99.94	0.00	Yes
NCBI Assembly	GCA_002802425.1	NCBI: cellular organisms; Bacteria; Terrabacteria group; Actinobacteria; Actinomycetia; Corynebacteriales; Mycobacteriaceae; Mycobacteroides; Mycobacteroides abscessus; Mycobacteroides abscessus subsp. abscessus;	NCBI: 1185650	5008579	100.00	0.50	Yes
NCBI Assembly	GCA_004108415.1	NCBI: cellular organisms; Bacteria; Terrabacteria group; Actinobacteria; Actinomycetia; Corynebacteriales; Mycobacteriaceae; Mycobacterium; Mycobacterium tuberculosis complex; Mycobacterium tuberculosis;	NCBI: 1773	4401853	99.61	0.00	Yes
NCBI Assembly	GCA_004105565.1	NCBI: cellular organisms; Bacteria; Terrabacteria group; Actinobacteria; Actinomycetia; Corynebacteriales; Mycobacteriaceae; Mycobacterium; Mycobacterium tuberculosis complex; Mycobacterium tuberculosis;	NCBI: 1773	4373904	99.94	0.00	Yes
NCBI Assembly	GCA_003393435.1	NCBI: cellular organisms; Bacteria; Terrabacteria group; Actinobacteria; Actinomycetia; Corynebacteriales; Mycobacteriaceae; Mycobacterium; Mycobacterium tuberculosis complex; Mycobacterium tuberculosis;	NCBI: 1773	4398175	99.86	0.33	Yes
NCBI Assembly	GCA_003049275.1	NCBI: cellular organisms; Bacteria; Terrabacteria group; Actinobacteria; Actinomycetia; Corynebacteriales; Mycobacteriaceae; Mycobacterium; Mycobacterium tuberculosis complex; Mycobacterium tuberculosis;	NCBI: 1773	4658614	99.86	0.33	Yes
NCBI Assembly	GCA_002928255.1	NCBI: cellular organisms; Bacteria; Terrabacteria group; Actinobacteria; Actinomycetia; Corynebacteriales; Mycobacteriaceae; Mycobacterium; Mycobacterium avium complex (MAC); Mycobacterium avium; Mycobacterium avium subsp. hominissuis;	NCBI: 439334	5562260	99.94	1.50	Yes
NCBI Assembly	GCA_004112225.1	NCBI: cellular organisms; Bacteria; Terrabacteria group; Actinobacteria; Actinomycetia; Corynebacteriales; Mycobacteriaceae; Mycobacterium; Mycobacterium tuberculosis complex; Mycobacterium tuberculosis;	NCBI: 1773	4376151	99.94	0.00	Yes
NCBI Assembly	GCA_002802845.1	NCBI: cellular organisms; Bacteria; Terrabacteria group; Actinobacteria; Actinomycetia; Corynebacteriales; Mycobacteriaceae; Mycobacteroides; Mycobacteroides abscessus; Mycobacteroides abscessus subsp. abscessus;	NCBI: 1185650	5299007	100.00	0.11	Yes
NCBI Assembly	GCA_004104875.1	NCBI: cellular organisms; Bacteria; Terrabacteria group; Actinobacteria; Actinomycetia; Corynebacteriales; Mycobacteriaceae; Mycobacterium; Mycobacterium tuberculosis complex; Mycobacterium tuberculosis;	NCBI: 1773	4384969	99.94	0.00	Yes

NCBI Assembly	GCA_002886585.1	NCBI: cellular organisms; Bacteria; Terrabacteria group; Actinobacteria; Actinomycetia; Corynebacteriales; Mycobacteriaceae; Mycobacterium; Mycobacterium tuberculosis complex; Mycobacterium tuberculosis;	NCBI: 1773	4411519	99.94	0.00	Yes
NCBI Assembly	GCA_004107465.1	NCBI: cellular organisms; Bacteria; Terrabacteria group; Actinobacteria; Actinomycetia; Corynebacteriales; Mycobacteriaceae; Mycobacterium; Mycobacterium tuberculosis complex; Mycobacterium tuberculosis;	NCBI: 1773	4383750	99.94	0.00	Yes
NCBI Assembly	GCA_004108055.1	NCBI: cellular organisms; Bacteria; Terrabacteria group; Actinobacteria; Actinomycetia; Corynebacteriales; Mycobacteriaceae; Mycobacterium; Mycobacterium tuberculosis complex; Mycobacterium tuberculosis;	NCBI: 1773	4395185	99.61	0.00	Yes
NCBI Assembly	GCA_002802435.1	NCBI: cellular organisms; Bacteria; Terrabacteria group; Actinobacteria; Actinomycetia; Corynebacteriales; Mycobacteriaceae; Mycobacteroides; Mycobacteroides abscessus; Mycobacteroides abscessus subsp. abscessus;	NCBI: 1185650	5092232	100.00	0.08	Yes
NCBI Assembly	GCA_003049285.1	NCBI: cellular organisms; Bacteria; Terrabacteria group; Actinobacteria; Actinomycetia; Corynebacteriales; Mycobacteriaceae; Mycobacterium; Mycobacterium tuberculosis complex; Mycobacterium tuberculosis;	NCBI: 1773	4376991	99.86	0.92	Yes
NCBI Assembly	GCA_004108445.1	NCBI: cellular organisms; Bacteria; Terrabacteria group; Actinobacteria; Actinomycetia; Corynebacteriales; Mycobacteriaceae; Mycobacterium; Mycobacterium tuberculosis complex; Mycobacterium tuberculosis;	NCBI: 1773	4396381	99.61	0.00	Yes
NCBI Assembly	GCA_002928265.1	NCBI: cellular organisms; Bacteria; Terrabacteria group; Actinobacteria; Actinomycetia; Corynebacteriales; Mycobacteriaceae; Mycobacterium; Mycobacterium avium complex (MAC); Mycobacterium avium; Mycobacterium avium subsp. hominissuis;	NCBI: 439334	5363248	99.67	5.48	No
NCBI Assembly	GCA_003393445.1	NCBI: cellular organisms; Bacteria; Terrabacteria group; Actinobacteria; Actinomycetia; Corynebacteriales; Mycobacteriaceae; Mycobacterium; Mycobacterium tuberculosis complex; Mycobacterium tuberculosis;	NCBI: 1773	4330919	99.53	0.33	Yes
NCBI Assembly	GCA_004105575.1	NCBI: cellular organisms; Bacteria; Terrabacteria group; Actinobacteria; Actinomycetia; Corynebacteriales; Mycobacteriaceae; Mycobacterium; Mycobacterium tuberculosis complex; Mycobacterium tuberculosis;	NCBI: 1773	4385881	99.94	0.00	Yes
NCBI Assembly	GCA_002838065.1	NCBI: cellular organisms; Bacteria; Terrabacteria group; Actinobacteria; Actinomycetia; Corynebacteriales; Mycobacteriaceae; Mycobacterium; unclassified Mycobacterium;	NCBI: 1867256	5575795	100.00	0.67	Yes
NCBI Assembly	GCA_004112235.1	NCBI: cellular organisms; Bacteria; Terrabacteria group; Actinobacteria; Actinomycetia; Corynebacteriales; Mycobacteriaceae; Mycobacterium; Mycobacterium tuberculosis complex; Mycobacterium tuberculosis;	NCBI: 1773	4395599	99.61	0.00	Yes
NCBI Assembly	GCA_004104885.1	NCBI: cellular organisms; Bacteria; Terrabacteria group; Actinobacteria; Actinomycetia; Corynebacteriales; Mycobacteriaceae; Mycobacterium; Mycobacterium tuberculosis complex; Mycobacterium tuberculosis;	NCBI: 1773	4422151	99.61	0.00	Yes
NCBI Assembly	GCA_002886685.1	NCBI: cellular organisms; Bacteria; Terrabacteria group; Actinobacteria; Actinomycetia; Corynebacteriales; Mycobacteriaceae; Mycobacterium; Mycobacterium tuberculosis complex; Mycobacterium tuberculosis;	NCBI: 1773	4411526	99.94	0.00	Yes
NCBI Assembly	GCA_002802485.1	NCBI: cellular organisms; Bacteria; Terrabacteria group; Actinobacteria; Actinomycetia; Corynebacteriales; Mycobacteriaceae; Mycobacteroides; Mycobacteroides abscessus; Mycobacteroides abscessus subsp. abscessus;	NCBI: 1185650	4986376	100.00	0.00	Yes
NCBI Assembly	GCA_004107485.1	NCBI: cellular organisms; Bacteria; Terrabacteria group; Actinobacteria; Actinomycetia; Corynebacteriales; Mycobacteriaceae; Mycobacterium; Mycobacterium tuberculosis complex; Mycobacterium tuberculosis;	NCBI: 1773	4405354	99.61	0.00	Yes
NCBI Assembly	GCA_004108065.1	NCBI: cellular organisms; Bacteria; Terrabacteria group; Actinobacteria; Actinomycetia; Corynebacteriales; Mycobacteriaceae; Mycobacterium; Mycobacterium tuberculosis complex; Mycobacterium tuberculosis;	NCBI: 1773	4394452	99.94	0.00	Yes
NCBI Assembly	GCA_003049325.1	NCBI: cellular organisms; Bacteria; Terrabacteria group; Actinobacteria; Actinomycetia; Corynebacteriales; Mycobacteriaceae; Mycobacterium; Mycobacterium tuberculosis complex; Mycobacterium tuberculosis;	NCBI: 1773	4322707	99.86	0.00	Yes
NCBI Assembly	GCA_002939465.1	NCBI: cellular organisms; Bacteria; Terrabacteria group; Actinobacteria; Actinomycetia; Corynebacteriales; Mycobacteriaceae; Mycobacterium; Mycobacterium tuberculosis complex; Mycobacterium tuberculosis;	NCBI: 1773	4372076	99.94	0.00	Yes

NCBI Assembly	GCA_003393485.1	NCBI: cellular organisms; Bacteria; Terrabacteria group; Actinobacteria; Actinomycetia; Corynebacteriales; Mycobacteriaceae; Mycobacterium; Mycobacterium tuberculosis complex; Mycobacterium tuberculosis;	NCBI: 1773	4279263	99.86	0.21	Yes
NCBI Assembly	GCA_002843405.1	NCBI: cellular organisms; Bacteria; Terrabacteria group; Actinobacteria; Actinomycetia; Corynebacteriales; Mycobacteriaceae; Mycobacterium; unclassified Mycobacterium;	NCBI: 418031	4940239	99.50	0.41	Yes
NCBI Assembly	GCA_004108455.1	NCBI: cellular organisms; Bacteria; Terrabacteria group; Actinobacteria; Actinomycetia; Corynebacteriales; Mycobacteriaceae; Mycobacterium; Mycobacterium tuberculosis complex; Mycobacterium tuberculosis;	NCBI: 1773	4398007	99.61	0.00	Yes
NCBI Assembly	GCA_004105585.1	NCBI: cellular organisms; Bacteria; Terrabacteria group; Actinobacteria; Actinomycetia; Corynebacteriales; Mycobacteriaceae; Mycobacterium; Mycobacterium tuberculosis complex; Mycobacterium tuberculosis;	NCBI: 1773	4395755	99.94	0.00	Yes
NCBI Assembly	GCA_002886775.1	NCBI: cellular organisms; Bacteria; Terrabacteria group; Actinobacteria; Actinomycetia; Corynebacteriales; Mycobacteriaceae; Mycobacterium; Mycobacterium tuberculosis complex; Mycobacterium tuberculosis;	NCBI: 1773	4411510	99.94	0.00	Yes
NCBI Assembly	GCA_004104895.1	NCBI: cellular organisms; Bacteria; Terrabacteria group; Actinobacteria; Actinomycetia; Corynebacteriales; Mycobacteriaceae; Mycobacterium; Mycobacterium tuberculosis complex; Mycobacterium tuberculosis;	NCBI: 1773	4379282	99.94	0.00	Yes
NCBI Assembly	GCA_002802505.1	NCBI: cellular organisms; Bacteria; Terrabacteria group; Actinobacteria; Actinomycetia; Corynebacteriales; Mycobacteriaceae; Mycobacteroides; Mycobacteroides abscessus; Mycobacteroides abscessus subsp. abscessus;	NCBI: 1185650	5070818	100.00	0.03	Yes
NCBI Assembly	GCA_004112245.1	NCBI: cellular organisms; Bacteria; Terrabacteria group; Actinobacteria; Actinomycetia; Corynebacteriales; Mycobacteriaceae; Mycobacterium; Mycobacterium tuberculosis complex; Mycobacterium tuberculosis;	NCBI: 1773	4389684	99.94	0.00	Yes
NCBI Assembly	GCA_004107505.1	NCBI: cellular organisms; Bacteria; Terrabacteria group; Actinobacteria; Actinomycetia; Corynebacteriales; Mycobacteriaceae; Mycobacterium; Mycobacterium tuberculosis complex; Mycobacterium tuberculosis;	NCBI: 1773	4402783	99.94	0.00	Yes
NCBI Assembly	GCA_003049345.1	NCBI: cellular organisms; Bacteria; Terrabacteria group; Actinobacteria; Actinomycetia; Corynebacteriales; Mycobacteriaceae; Mycobacterium; Mycobacterium tuberculosis complex; Mycobacterium tuberculosis;	NCBI: 1773	4827425	100.00	17.08	No
NCBI Assembly	GCA_004108075.1	NCBI: cellular organisms; Bacteria; Terrabacteria group; Actinobacteria; Actinomycetia; Corynebacteriales; Mycobacteriaceae; Mycobacterium; Mycobacterium tuberculosis complex; Mycobacterium tuberculosis;	NCBI: 1773	4402409	99.94	0.00	Yes
NCBI Assembly	GCA_002939505.1	NCBI: cellular organisms; Bacteria; Terrabacteria group; Actinobacteria; Actinomycetia; Corynebacteriales; Mycobacteriaceae; Mycobacterium; Mycobacterium tuberculosis complex; Mycobacterium tuberculosis;	NCBI: 1773	4411005	99.94	0.00	Yes
NCBI Assembly	GCA_002851795.1	NCBI: cellular organisms; Bacteria; Terrabacteria group; Actinobacteria; Actinomycetia; Corynebacteriales; Mycobacteriaceae; Mycobacterium; Mycobacterium avium complex (MAC); Mycobacterium intracellulare; Mycobacterium intracellulare subsp. chimaera;	NCBI: 222805	6577953	99.94	1.19	Yes
NCBI Assembly	GCA_003393495.1	NCBI: cellular organisms; Bacteria; Terrabacteria group; Actinobacteria; Actinomycetia; Corynebacteriales; Mycobacteriaceae; Mycobacterium; Mycobacterium tuberculosis complex; Mycobacterium tuberculosis;	NCBI: 1773	4400483	99.86	0.33	Yes
NCBI Assembly	GCA_002886865.1	NCBI: cellular organisms; Bacteria; Terrabacteria group; Actinobacteria; Actinomycetia; Corynebacteriales; Mycobacteriaceae; Mycobacterium; Mycobacterium tuberculosis complex; Mycobacterium tuberculosis;	NCBI: 1773	4411478	99.94	0.00	Yes
NCBI Assembly	GCA_004105615.1	NCBI: cellular organisms; Bacteria; Terrabacteria group; Actinobacteria; Actinomycetia; Corynebacteriales; Mycobacteriaceae; Mycobacterium; Mycobacterium tuberculosis complex; Mycobacterium tuberculosis;	NCBI: 1773	4389828	99.61	0.00	Yes
NCBI Assembly	GCA_004108465.1	NCBI: cellular organisms; Bacteria; Terrabacteria group; Actinobacteria; Actinomycetia; Corynebacteriales; Mycobacteriaceae; Mycobacterium; Mycobacterium tuberculosis complex; Mycobacterium tuberculosis;	NCBI: 1773	4387571	99.94	0.00	Yes
NCBI Assembly	GCA_002802515.1	NCBI: cellular organisms; Bacteria; Terrabacteria group; Actinobacteria; Actinomycetia; Corynebacteriales; Mycobacteriaceae; Mycobacteroides; Mycobacteroides abscessus; Mycobacteroides abscessus subsp. abscessus;	NCBI: 1185650	5256603	100.00	0.42	Yes

NCBI Assembly	GCA_004104905.1	NCBI: cellular organisms; Bacteria; Terrabacteria group; Actinobacteria; Actinomycetia; Corynebacteriales; Mycobacteriaceae; Mycobacterium; Mycobacterium tuberculosis complex; Mycobacterium tuberculosis;	NCBI: 1773	4371589	99.94	0.00	Yes
NCBI Assembly	GCA_004112275.1	NCBI: cellular organisms; Bacteria; Terrabacteria group; Actinobacteria; Actinomycetia; Corynebacteriales; Mycobacteriaceae; Mycobacterium; Mycobacterium tuberculosis complex; Mycobacterium tuberculosis;	NCBI: 1773	4354066	99.94	0.00	Yes
NCBI Assembly	GCA_004107515.1	NCBI: cellular organisms; Bacteria; Terrabacteria group; Actinobacteria; Actinomycetia; Corynebacteriales; Mycobacteriaceae; Mycobacterium; Mycobacterium tuberculosis complex; Mycobacterium tuberculosis;	NCBI: 1773	4394477	99.61	0.00	Yes
NCBI Assembly	GCA_003049355.1	NCBI: cellular organisms; Bacteria; Terrabacteria group; Actinobacteria; Actinomycetia; Corynebacteriales; Mycobacteriaceae; Mycobacterium; Mycobacterium tuberculosis complex; Mycobacterium tuberculosis;	NCBI: 1773	4421334	99.81	1.00	Yes
NCBI Assembly	GCA_002851815.1	NCBI: cellular organisms; Bacteria; Terrabacteria group; Actinobacteria; Actinomycetia; Corynebacteriales; Mycobacteriaceae; Mycobacterium; Mycobacterium avium complex (MAC); Mycobacterium intracellulare; Mycobacterium intracellulare subsp. chimaera;	NCBI: 222805	6121676	99.94	1.15	Yes
NCBI Assembly	GCA_002939545.1	NCBI: cellular organisms; Bacteria; Terrabacteria group; Actinobacteria; Actinomycetia; Corynebacteriales; Mycobacteriaceae; Mycobacterium; Mycobacterium tuberculosis complex; Mycobacterium tuberculosis;	NCBI: 1773	4400560	99.94	0.00	Yes
NCBI Assembly	GCA_004108125.1	NCBI: cellular organisms; Bacteria; Terrabacteria group; Actinobacteria; Actinomycetia; Corynebacteriales; Mycobacteriaceae; Mycobacterium; Mycobacterium tuberculosis complex; Mycobacterium tuberculosis;	NCBI: 1773	4374938	99.94	0.00	Yes
NCBI Assembly	GCA_003393505.1	NCBI: cellular organisms; Bacteria; Terrabacteria group; Actinobacteria; Actinomycetia; Corynebacteriales; Mycobacteriaceae; Mycobacterium; Mycobacterium tuberculosis complex; Mycobacterium tuberculosis;	NCBI: 1773	4151716	96.73	3.57	Yes
NCBI Assembly	GCA_002886945.1	NCBI: cellular organisms; Bacteria; Terrabacteria group; Actinobacteria; Actinomycetia; Corynebacteriales; Mycobacteriaceae; Mycobacterium; Mycobacterium tuberculosis complex; Mycobacterium tuberculosis;	NCBI: 1773	4411443	99.94	0.00	Yes
NCBI Assembly	GCA_002802525.1	NCBI: cellular organisms; Bacteria; Terrabacteria group; Actinobacteria; Actinomycetia; Corynebacteriales; Mycobacteriaceae; Mycobacteroides; Mycobacteroides abscessus; Mycobacteroides abscessus subsp. abscessus;	NCBI: 1185650	4936890	99.33	0.33	Yes
NCBI Assembly	GCA_004105635.1	NCBI: cellular organisms; Bacteria; Terrabacteria group; Actinobacteria; Actinomycetia; Corynebacteriales; Mycobacteriaceae; Mycobacterium; Mycobacterium tuberculosis complex; Mycobacterium tuberculosis;	NCBI: 1773	4417997	99.61	0.00	Yes
NCBI Assembly	GCA_004108475.1	NCBI: cellular organisms; Bacteria; Terrabacteria group; Actinobacteria; Actinomycetia; Corynebacteriales; Mycobacteriaceae; Mycobacterium; Mycobacterium tuberculosis complex; Mycobacterium tuberculosis;	NCBI: 1773	4386981	99.94	0.00	Yes
NCBI Assembly	GCA_004112295.1	NCBI: cellular organisms; Bacteria; Terrabacteria group; Actinobacteria; Actinomycetia; Corynebacteriales; Mycobacteriaceae; Mycobacterium; Mycobacterium tuberculosis complex; Mycobacterium tuberculosis;	NCBI: 1773	4385422	99.94	0.00	Yes
NCBI Assembly	GCA_004104965.1	NCBI: cellular organisms; Bacteria; Terrabacteria group; Actinobacteria; Actinomycetia; Corynebacteriales; Mycobacteriaceae; Mycobacterium; Mycobacterium tuberculosis complex; Mycobacterium tuberculosis;	NCBI: 1773	4391916	99.61	0.00	Yes
NCBI Assembly	GCA_002851835.1	NCBI: cellular organisms; Bacteria; Terrabacteria group; Actinobacteria; Actinomycetia; Corynebacteriales; Mycobacteriaceae; Mycobacterium; Mycobacterium avium complex (MAC); Mycobacterium intracellulare; Mycobacterium intracellulare subsp. chimaera;	NCBI: 222805	6607049	99.94	1.32	Yes
NCBI Assembly	GCA_003049385.1	NCBI: cellular organisms; Bacteria; Terrabacteria group; Actinobacteria; Actinomycetia; Corynebacteriales; Mycobacteriaceae; Mycobacterium; Mycobacterium tuberculosis complex; Mycobacterium tuberculosis;	NCBI: 1773	5770594	100.00	53.62	No
NCBI Assembly	GCA_004107525.1	NCBI: cellular organisms; Bacteria; Terrabacteria group; Actinobacteria; Actinomycetia; Corynebacteriales; Mycobacteriaceae; Mycobacterium; Mycobacterium tuberculosis complex; Mycobacterium tuberculosis;	NCBI: 1773	4402648	99.94	0.00	Yes
NCBI Assembly	GCA_002939565.1	NCBI: cellular organisms; Bacteria; Terrabacteria group; Actinobacteria; Actinomycetia; Corynebacteriales; Mycobacteriaceae; Mycobacterium; Mycobacterium tuberculosis complex; Mycobacterium tuberculosis;	NCBI: 1773	4378402	99.94	0.00	Yes

NCBI Assembly	GCA_002887065.1	NCBI: cellular organisms; Bacteria; Terrabacteria group; Actinobacteria; Actinomycetia; Corynebacteriales; Mycobacteriaceae; Mycobacterium; Mycobacterium tuberculosis complex; Mycobacterium tuberculosis;	NCBI: 1773	4411469	99.94	0.00	Yes
NCBI Assembly	GCA_003393525.1	NCBI: cellular organisms; Bacteria; Terrabacteria group; Actinobacteria; Actinomycetia; Corynebacteriales; Mycobacteriaceae; Mycobacterium; Mycobacterium tuberculosis complex; Mycobacterium tuberculosis;	NCBI: 1773	4364982	99.86	0.00	Yes
NCBI Assembly	GCA_004108145.1	NCBI: cellular organisms; Bacteria; Terrabacteria group; Actinobacteria; Actinomycetia; Corynebacteriales; Mycobacteriaceae; Mycobacterium; Mycobacterium tuberculosis complex; Mycobacterium tuberculosis;	NCBI: 1773	4383687	99.94	0.00	Yes
NCBI Assembly	GCA_002802565.1	NCBI: cellular organisms; Bacteria; Terrabacteria group; Actinobacteria; Actinomycetia; Corynebacteriales; Mycobacteriaceae; Mycobacteroides; Mycobacteroides abscessus; Mycobacteroides abscessus subsp. abscessus;	NCBI: 1185650	5251515	100.00	0.25	Yes
NCBI Assembly	GCA_004105665.1	NCBI: cellular organisms; Bacteria; Terrabacteria group; Actinobacteria; Actinomycetia; Corynebacteriales; Mycobacteriaceae; Mycobacterium; Mycobacterium tuberculosis complex; Mycobacterium tuberculosis;	NCBI: 1773	4395224	99.94	0.00	Yes
NCBI Assembly	GCA_004108485.1	NCBI: cellular organisms; Bacteria; Terrabacteria group; Actinobacteria; Actinomycetia; Corynebacteriales; Mycobacteriaceae; Mycobacterium; Mycobacterium tuberculosis complex; Mycobacterium tuberculosis;	NCBI: 1773	4383374	99.61	0.00	Yes
NCBI Assembly	GCA_002865895.1	NCBI: cellular organisms; Bacteria; Terrabacteria group; Actinobacteria; Actinomycetia; Corynebacteriales; Mycobacteriaceae; Mycobacterium; Mycobacterium tuberculosis complex; Mycobacterium tuberculosis; Mycobacterium tuberculosis variant microti;	NCBI: 1427372	5740218	99.61	1.78	Yes
NCBI Assembly	GCA_004112325.1	NCBI: cellular organisms; Bacteria; Terrabacteria group; Actinobacteria; Actinomycetia; Corynebacteriales; Mycobacteriaceae; Mycobacterium; Mycobacterium tuberculosis complex; Mycobacterium tuberculosis;	NCBI: 1773	4390761	99.94	0.00	Yes
NCBI Assembly	GCA_002939585.1	NCBI: cellular organisms; Bacteria; Terrabacteria group; Actinobacteria; Actinomycetia; Corynebacteriales; Mycobacteriaceae; Mycobacterium; Mycobacterium tuberculosis complex; Mycobacterium tuberculosis;	NCBI: 1773	4374936	99.94	0.00	Yes
NCBI Assembly	GCA_004107565.1	NCBI: cellular organisms; Bacteria; Terrabacteria group; Actinobacteria; Actinomycetia; Corynebacteriales; Mycobacteriaceae; Mycobacterium; Mycobacterium tuberculosis complex; Mycobacterium tuberculosis;	NCBI: 1773	4390264	99.94	0.00	Yes
NCBI Assembly	GCA_004104975.1	NCBI: cellular organisms; Bacteria; Terrabacteria group; Actinobacteria; Actinomycetia; Corynebacteriales; Mycobacteriaceae; Mycobacterium; Mycobacterium tuberculosis complex; Mycobacterium tuberculosis;	NCBI: 1773	4398344	99.94	0.00	Yes
NCBI Assembly	GCA_002887145.1	NCBI: cellular organisms; Bacteria; Terrabacteria group; Actinobacteria; Actinomycetia; Corynebacteriales; Mycobacteriaceae; Mycobacterium; Mycobacterium tuberculosis complex; Mycobacterium tuberculosis;	NCBI: 1773	4411469	99.94	0.00	Yes
NCBI Assembly	GCA_003393555.1	NCBI: cellular organisms; Bacteria; Terrabacteria group; Actinobacteria; Actinomycetia; Corynebacteriales; Mycobacteriaceae; Mycobacterium; Mycobacterium tuberculosis complex; Mycobacterium tuberculosis;	NCBI: 1773	4344992	99.86	0.33	Yes
NCBI Assembly	GCA_002802585.1	NCBI: cellular organisms; Bacteria; Terrabacteria group; Actinobacteria; Actinomycetia; Corynebacteriales; Mycobacteriaceae; Mycobacteroides; Mycobacteroides abscessus; Mycobacteroides abscessus subsp. abscessus;	NCBI: 1185650	4880519	100.00	0.03	Yes
NCBI Assembly	GCA_004108155.1	NCBI: cellular organisms; Bacteria; Terrabacteria group; Actinobacteria; Actinomycetia; Corynebacteriales; Mycobacteriaceae; Mycobacterium; Mycobacterium tuberculosis complex; Mycobacterium tuberculosis;	NCBI: 1773	4398986	99.94	0.00	Yes
NCBI Assembly	GCA_004105675.1	NCBI: cellular organisms; Bacteria; Terrabacteria group; Actinobacteria; Actinomycetia; Corynebacteriales; Mycobacteriaceae; Mycobacterium; Mycobacterium tuberculosis complex; Mycobacterium tuberculosis;	NCBI: 1773	4404287	99.94	0.00	Yes
NCBI Assembly	GCA_002886145.1	NCBI: cellular organisms; Bacteria; Terrabacteria group; Actinobacteria; Actinomycetia; Corynebacteriales; Mycobacteriaceae; Mycobacterium; Mycobacterium tuberculosis complex; Mycobacterium tuberculosis;	NCBI: 1773	4411563	99.94	0.00	Yes
NCBI Assembly	GCA_004108545.1	NCBI: cellular organisms; Bacteria; Terrabacteria group; Actinobacteria; Actinomycetia; Corynebacteriales; Mycobacteriaceae; Mycobacterium; Mycobacterium tuberculosis complex; Mycobacterium tuberculosis;	NCBI: 1773	4397344	99.94	0.00	Yes

NCBI Assembly	GCA_002939605.1	NCBI: cellular organisms; Bacteria; Terrabacteria group; Actinobacteria; Actinomycetia; Corynebacteriales; Mycobacteriaceae; Mycobacterium; Mycobacterium tuberculosis complex; Mycobacterium tuberculosis;	NCBI: 1773	4372203	99.94	0.00	Yes
NCBI Assembly	GCA_002887255.1	NCBI: cellular organisms; Bacteria; Terrabacteria group; Actinobacteria; Actinomycetia; Corynebacteriales; Mycobacteriaceae; Mycobacterium; Mycobacterium tuberculosis complex; Mycobacterium tuberculosis;	NCBI: 1773	4411463	99.94	0.00	Yes
NCBI Assembly	GCA_004107585.1	NCBI: cellular organisms; Bacteria; Terrabacteria group; Actinobacteria; Actinomycetia; Corynebacteriales; Mycobacteriaceae; Mycobacterium; Mycobacterium tuberculosis complex; Mycobacterium tuberculosis;	NCBI: 1773	4390619	99.61	0.00	Yes
NCBI Assembly	GCA_004112335.1	NCBI: cellular organisms; Bacteria; Terrabacteria group; Actinobacteria; Actinomycetia; Corynebacteriales; Mycobacteriaceae; Mycobacterium; Mycobacterium tuberculosis complex; Mycobacterium tuberculosis;	NCBI: 1773	4398912	99.61	0.00	Yes
NCBI Assembly	GCA_002802595.1	NCBI: cellular organisms; Bacteria; Terrabacteria group; Actinobacteria; Actinomycetia; Corynebacteriales; Mycobacteriaceae; Mycobacteroides; Mycobacteroides abscessus; Mycobacteroides abscessus subsp. abscessus;	NCBI: 1185650	4731238	100.00	0.00	Yes
NCBI Assembly	GCA_003393565.1	NCBI: cellular organisms; Bacteria; Terrabacteria group; Actinobacteria; Actinomycetia; Corynebacteriales; Mycobacteriaceae; Mycobacterium; Mycobacterium tuberculosis complex; Mycobacterium tuberculosis;	NCBI: 1773	4383872	99.69	0.33	Yes
NCBI Assembly	GCA_004104985.1	NCBI: cellular organisms; Bacteria; Terrabacteria group; Actinobacteria; Actinomycetia; Corynebacteriales; Mycobacteriaceae; Mycobacterium; Mycobacterium tuberculosis complex; Mycobacterium tuberculosis;	NCBI: 1773	4408855	99.94	0.00	Yes
NCBI Assembly	GCA_004108165.1	NCBI: cellular organisms; Bacteria; Terrabacteria group; Actinobacteria; Actinomycetia; Corynebacteriales; Mycobacteriaceae; Mycobacterium; Mycobacterium tuberculosis complex; Mycobacterium tuberculosis;	NCBI: 1773	4394877	99.94	0.00	Yes
NCBI Assembly	GCA_004105685.1	NCBI: cellular organisms; Bacteria; Terrabacteria group; Actinobacteria; Actinomycetia; Corynebacteriales; Mycobacteriaceae; Mycobacterium; Mycobacterium tuberculosis complex; Mycobacterium tuberculosis;	NCBI: 1773	4384292	99.94	0.00	Yes
NCBI Assembly	GCA_002886165.1	NCBI: cellular organisms; Bacteria; Terrabacteria group; Actinobacteria; Actinomycetia; Corynebacteriales; Mycobacteriaceae; Mycobacterium; Mycobacterium tuberculosis complex; Mycobacterium tuberculosis;	NCBI: 1773	4411442	99.94	0.00	Yes
NCBI Assembly	GCA_002939625.1	NCBI: cellular organisms; Bacteria; Terrabacteria group; Actinobacteria; Actinomycetia; Corynebacteriales; Mycobacteriaceae; Mycobacterium; Mycobacterium tuberculosis complex; Mycobacterium tuberculosis;	NCBI: 1773	4395277	99.94	0.00	Yes
NCBI Assembly	GCA_004108565.1	NCBI: cellular organisms; Bacteria; Terrabacteria group; Actinobacteria; Actinomycetia; Corynebacteriales; Mycobacteriaceae; Mycobacterium; Mycobacterium tuberculosis complex; Mycobacterium tuberculosis;	NCBI: 1773	4383144	99.94	0.00	Yes
NCBI Assembly	GCA_002802655.1	NCBI: cellular organisms; Bacteria; Terrabacteria group; Actinobacteria; Actinomycetia; Corynebacteriales; Mycobacteriaceae; Mycobacteroides; Mycobacteroides abscessus; Mycobacteroides abscessus subsp. abscessus;	NCBI: 1185650	5034190	100.00	0.03	Yes
NCBI Assembly	GCA_002887335.1	NCBI: cellular organisms; Bacteria; Terrabacteria group; Actinobacteria; Actinomycetia; Corynebacteriales; Mycobacteriaceae; Mycobacterium; Mycobacterium tuberculosis complex; Mycobacterium tuberculosis;	NCBI: 1773	4411446	99.94	0.00	Yes
NCBI Assembly	GCA_004107595.1	NCBI: cellular organisms; Bacteria; Terrabacteria group; Actinobacteria; Actinomycetia; Corynebacteriales; Mycobacteriaceae; Mycobacterium; Mycobacterium tuberculosis complex; Mycobacterium tuberculosis;	NCBI: 1773	4380421	99.94	0.00	Yes
NCBI Assembly	GCA_003393575.1	NCBI: cellular organisms; Bacteria; Terrabacteria group; Actinobacteria; Actinomycetia; Corynebacteriales; Mycobacteriaceae; Mycobacterium; Mycobacterium tuberculosis complex; Mycobacterium tuberculosis;	NCBI: 1773	4434528	99.86	0.44	Yes
NCBI Assembly	GCA_004112345.1	NCBI: cellular organisms; Bacteria; Terrabacteria group; Actinobacteria; Actinomycetia; Corynebacteriales; Mycobacteriaceae; Mycobacterium; Mycobacterium tuberculosis complex; Mycobacterium tuberculosis;	NCBI: 1773	4405906	99.94	0.00	Yes
NCBI Assembly	GCA_004105015.1	NCBI: cellular organisms; Bacteria; Terrabacteria group; Actinobacteria; Actinomycetia; Corynebacteriales; Mycobacteriaceae; Mycobacterium; Mycobacterium tuberculosis complex; Mycobacterium tuberculosis;	NCBI: 1773	4389106	99.61	0.00	Yes

NCBI Assembly	GCA_004108185.1	NCBI: cellular organisms; Bacteria; Terrabacteria group; Actinobacteria; Actinomycetia; Corynebacteriales; Mycobacteriaceae; Mycobacterium; Mycobacterium tuberculosis complex; Mycobacterium tuberculosis;	NCBI: 1773	4391352	99.94	0.00	Yes
NCBI Assembly	GCA_002886195.1	NCBI: cellular organisms; Bacteria; Terrabacteria group; Actinobacteria; Actinomycetia; Corynebacteriales; Mycobacteriaceae; Mycobacterium; Mycobacterium tuberculosis complex; Mycobacterium tuberculosis;	NCBI: 1773	4411504	99.94	0.00	Yes
NCBI Assembly	GCA_004105695.1	NCBI: cellular organisms; Bacteria; Terrabacteria group; Actinobacteria; Actinomycetia; Corynebacteriales; Mycobacteriaceae; Mycobacterium; Mycobacterium tuberculosis complex; Mycobacterium tuberculosis;	NCBI: 1773	4406173	99.94	0.00	Yes
NCBI Assembly	GCA_002887815.1	NCBI: cellular organisms; Bacteria; Terrabacteria group; Actinobacteria; Actinomycetia; Corynebacteriales; Mycobacteriaceae; Mycobacterium; unclassified Mycobacterium;	NCBI: 1213407	6228710	100.00	0.12	Yes
NCBI Assembly	GCA_002939645.1	NCBI: cellular organisms; Bacteria; Terrabacteria group; Actinobacteria; Actinomycetia; Corynebacteriales; Mycobacteriaceae; Mycobacterium; Mycobacterium tuberculosis complex; Mycobacterium tuberculosis;	NCBI: 1773	4391694	99.94	0.00	Yes
NCBI Assembly	GCA_002802685.1	NCBI: cellular organisms; Bacteria; Terrabacteria group; Actinobacteria; Actinomycetia; Corynebacteriales; Mycobacteriaceae; Mycobacteroides; Mycobacteroides abscessus; Mycobacteroides abscessus subsp. abscessus;	NCBI: 1185650	4897021	100.00	0.47	Yes
NCBI Assembly	GCA_004108575.1	NCBI: cellular organisms; Bacteria; Terrabacteria group; Actinobacteria; Actinomycetia; Corynebacteriales; Mycobacteriaceae; Mycobacterium; Mycobacterium tuberculosis complex; Mycobacterium tuberculosis;	NCBI: 1773	4396581	99.94	0.00	Yes
NCBI Assembly	GCA_003392675.1	NCBI: cellular organisms; Bacteria; Terrabacteria group; Actinobacteria; Actinomycetia; Corynebacteriales; Mycobacteriaceae; Mycobacterium; Mycobacterium tuberculosis complex; Mycobacterium tuberculosis;	NCBI: 1773	4263293	99.69	0.40	Yes
NCBI Assembly	GCA_004107615.1	NCBI: cellular organisms; Bacteria; Terrabacteria group; Actinobacteria; Actinomycetia; Corynebacteriales; Mycobacteriaceae; Mycobacterium; Mycobacterium tuberculosis complex; Mycobacterium tuberculosis;	NCBI: 1773	4381546	99.94	0.00	Yes
NCBI Assembly	GCA_004112355.1	NCBI: cellular organisms; Bacteria; Terrabacteria group; Actinobacteria; Actinomycetia; Corynebacteriales; Mycobacteriaceae; Mycobacterium; Mycobacterium tuberculosis complex; Mycobacterium tuberculosis;	NCBI: 1773	4374551	99.94	0.00	Yes
NCBI Assembly	GCA_004105035.1	NCBI: cellular organisms; Bacteria; Terrabacteria group; Actinobacteria; Actinomycetia; Corynebacteriales; Mycobacteriaceae; Mycobacterium; Mycobacterium tuberculosis complex; Mycobacterium tuberculosis;	NCBI: 1773	4397320	99.94	0.00	Yes
NCBI Assembly	GCA_002886225.1	NCBI: cellular organisms; Bacteria; Terrabacteria group; Actinobacteria; Actinomycetia; Corynebacteriales; Mycobacteriaceae; Mycobacterium; Mycobacterium tuberculosis complex; Mycobacterium tuberculosis;	NCBI: 1773	4411508	99.94	0.00	Yes
NCBI Assembly	GCA_004108205.1	NCBI: cellular organisms; Bacteria; Terrabacteria group; Actinobacteria; Actinomycetia; Corynebacteriales; Mycobacteriaceae; Mycobacterium; Mycobacterium tuberculosis complex; Mycobacterium tuberculosis;	NCBI: 1773	4390562	99.61	0.00	Yes
NCBI Assembly	GCA_002802695.1	NCBI: cellular organisms; Bacteria; Terrabacteria group; Actinobacteria; Actinomycetia; Corynebacteriales; Mycobacteriaceae; Mycobacteroides; Mycobacteroides abscessus; Mycobacteroides abscessus subsp. abscessus;	NCBI: 1185650	4845292	100.00	0.00	Yes
NCBI Assembly	GCA_002890415.1	NCBI: cellular organisms; Bacteria; Terrabacteria group; Actinobacteria; Actinomycetia; Corynebacteriales; Mycobacteriaceae; Mycobacterium; Mycobacterium tuberculosis complex; Mycobacterium tuberculosis;	NCBI: 1773	4233499	99.44	0.04	Yes
NCBI Assembly	GCA_002939665.1	NCBI: cellular organisms; Bacteria; Terrabacteria group; Actinobacteria; Actinomycetia; Corynebacteriales; Mycobacteriaceae; Mycobacterium; Mycobacterium tuberculosis complex; Mycobacterium tuberculosis;	NCBI: 1773	4384898	99.94	0.00	Yes
NCBI Assembly	GCA_004105725.1	NCBI: cellular organisms; Bacteria; Terrabacteria group; Actinobacteria; Actinomycetia; Corynebacteriales; Mycobacteriaceae; Mycobacterium; Mycobacterium tuberculosis complex; Mycobacterium tuberculosis;	NCBI: 1773	4387053	99.94	0.00	Yes
NCBI Assembly	GCA_003392685.1	NCBI: cellular organisms; Bacteria; Terrabacteria group; Actinobacteria; Actinomycetia; Corynebacteriales; Mycobacteriaceae; Mycobacterium; Mycobacterium tuberculosis complex; Mycobacterium tuberculosis;	NCBI: 1773	4318716	99.86	0.00	Yes

NCBI Assembly	GCA_004108585.1	NCBI: cellular organisms; Bacteria; Terrabacteria group; Actinobacteria; Actinomycetia; Corynebacteriales; Mycobacteriaceae; Mycobacterium; Mycobacterium tuberculosis complex; Mycobacterium tuberculosis;	NCBI: 1773	4388776	99.94	0.00	Yes
NCBI Assembly	GCA_004107645.1	NCBI: cellular organisms; Bacteria; Terrabacteria group; Actinobacteria; Actinomycetia; Corynebacteriales; Mycobacteriaceae; Mycobacterium; Mycobacterium tuberculosis complex; Mycobacterium tuberculosis;	NCBI: 1773	4424190	99.61	0.00	Yes
NCBI Assembly	GCA_004112395.1	NCBI: cellular organisms; Bacteria; Terrabacteria group; Actinobacteria; Actinomycetia; Corynebacteriales; Mycobacteriaceae; Mycobacterium; Mycobacterium tuberculosis complex; Mycobacterium tuberculosis;	NCBI: 1773	4379160	99.94	0.00	Yes
NCBI Assembly	GCA_004105065.1	NCBI: cellular organisms; Bacteria; Terrabacteria group; Actinobacteria; Actinomycetia; Corynebacteriales; Mycobacteriaceae; Mycobacterium; Mycobacterium tuberculosis complex; Mycobacterium tuberculosis;	NCBI: 1773	4391846	99.61	0.00	Yes
NCBI Assembly	GCA_002886335.1	NCBI: cellular organisms; Bacteria; Terrabacteria group; Actinobacteria; Actinomycetia; Corynebacteriales; Mycobacteriaceae; Mycobacterium; Mycobacterium tuberculosis complex; Mycobacterium tuberculosis;	NCBI: 1773	4411602	99.94	0.00	Yes
NCBI Assembly	GCA_002894345.1	NCBI: cellular organisms; Bacteria; Terrabacteria group; Actinobacteria; Actinomycetia; Corynebacteriales; Mycobacteriaceae; Mycobacterium; Mycobacterium avium complex (MAC); Mycobacterium avium; Mycobacterium avium subsp. hominissuis;	NCBI: 439334	5544128	99.94	3.97	Yes
NCBI Assembly	GCA_004108245.1	NCBI: cellular organisms; Bacteria; Terrabacteria group; Actinobacteria; Actinomycetia; Corynebacteriales; Mycobacteriaceae; Mycobacterium; Mycobacterium tuberculosis complex; Mycobacterium tuberculosis;	NCBI: 1773	4384299	99.61	0.00	Yes
NCBI Assembly	GCA_002946335.1	NCBI: cellular organisms; Bacteria; Terrabacteria group; Actinobacteria; Actinomycetia; Corynebacteriales; Mycobacteriaceae; Mycobacterium; unclassified Mycobacterium;	NCBI: 2064767	6021153	99.72	0.00	Yes
NCBI Assembly	GCA_002802725.1	NCBI: cellular organisms; Bacteria; Terrabacteria group; Actinobacteria; Actinomycetia; Corynebacteriales; Mycobacteriaceae; Mycobacteroides; Mycobacteroides abscessus; Mycobacteroides abscessus subsp. abscessus;	NCBI: 1185650	4863208	100.00	0.33	Yes
NCBI Assembly	GCA_004105765.1	NCBI: cellular organisms; Bacteria; Terrabacteria group; Actinobacteria; Actinomycetia; Corynebacteriales; Mycobacteriaceae; Mycobacterium; Mycobacterium tuberculosis complex; Mycobacterium tuberculosis;	NCBI: 1773	4390052	99.94	0.00	Yes
NCBI Assembly	GCA_004107035.1	NCBI: cellular organisms; Bacteria; Terrabacteria group; Actinobacteria; Actinomycetia; Corynebacteriales; Mycobacteriaceae; Mycobacterium; Mycobacterium tuberculosis complex; Mycobacterium tuberculosis;	NCBI: 1773	4382305	99.94	0.00	Yes
NCBI Assembly	GCA_003392695.1	NCBI: cellular organisms; Bacteria; Terrabacteria group; Actinobacteria; Actinomycetia; Corynebacteriales; Mycobacteriaceae; Mycobacterium; Mycobacterium tuberculosis complex; Mycobacterium tuberculosis;	NCBI: 1773	4333766	99.86	0.00	Yes
NCBI Assembly	GCA_004108595.1	NCBI: cellular organisms; Bacteria; Terrabacteria group; Actinobacteria; Actinomycetia; Corynebacteriales; Mycobacteriaceae; Mycobacterium; Mycobacterium tuberculosis complex; Mycobacterium tuberculosis;	NCBI: 1773	4387119	99.94	0.00	Yes
NCBI Assembly	GCA_004107665.1	NCBI: cellular organisms; Bacteria; Terrabacteria group; Actinobacteria; Actinomycetia; Corynebacteriales; Mycobacteriaceae; Mycobacterium; Mycobacterium tuberculosis complex; Mycobacterium tuberculosis;	NCBI: 1773	4397729	99.94	0.00	Yes
NCBI Assembly	GCA_004112425.1	NCBI: cellular organisms; Bacteria; Terrabacteria group; Actinobacteria; Actinomycetia; Corynebacteriales; Mycobacteriaceae; Mycobacterium; Mycobacterium tuberculosis complex; Mycobacterium tuberculosis;	NCBI: 1773	4391395	99.94	0.00	Yes
NCBI Assembly	GCA_004105075.1	NCBI: cellular organisms; Bacteria; Terrabacteria group; Actinobacteria; Actinomycetia; Corynebacteriales; Mycobacteriaceae; Mycobacterium; Mycobacterium tuberculosis complex; Mycobacterium tuberculosis;	NCBI: 1773	4389139	99.61	0.00	Yes
NCBI Assembly	GCA_004111825.1	NCBI: cellular organisms; Bacteria; Terrabacteria group; Actinobacteria; Actinomycetia; Corynebacteriales; Mycobacteriaceae; Mycobacterium; Mycobacterium tuberculosis complex; Mycobacterium tuberculosis;	NCBI: 1773	4375736	99.94	0.00	Yes
NCBI Assembly	GCA_002894715.1	NCBI: cellular organisms; Bacteria; Terrabacteria group; Actinobacteria; Actinomycetia; Corynebacteriales; Mycobacteriaceae; Mycobacterium; Mycobacterium avium complex (MAC); Mycobacterium avium; Mycobacterium avium subsp. hominissuis;	NCBI: 439334	5221801	99.83	0.07	Yes

NCBI Assembly	GCA_002895065.1	NCBI: cellular organisms; Bacteria; Terrabacteria group; Actinobacteria; Actinomycetia; Corynebacteriales; Mycobacteriaceae; Mycobacterium; Mycobacterium tuberculosis complex; Mycobacterium tuberculosis;	NCBI: 1773	4411530	74.68	0.00	No
NCBI Assembly	GCA_002802735.1	NCBI: cellular organisms; Bacteria; Terrabacteria group; Actinobacteria; Actinomycetia; Corynebacteriales; Mycobacteriaceae; Mycobacteroides; Mycobacteroides abscessus; Mycobacteroides abscessus subsp. abscessus;	NCBI: 1185650	5062721	100.00	0.00	Yes
NCBI Assembly	GCA_004108255.1	NCBI: cellular organisms; Bacteria; Terrabacteria group; Actinobacteria; Actinomycetia; Corynebacteriales; Mycobacteriaceae; Mycobacterium; Mycobacterium tuberculosis complex; Mycobacterium tuberculosis;	NCBI: 1773	4380818	99.94	0.00	Yes
NCBI Assembly	GCA_004105775.1	NCBI: cellular organisms; Bacteria; Terrabacteria group; Actinobacteria; Actinomycetia; Corynebacteriales; Mycobacteriaceae; Mycobacterium; Mycobacterium tuberculosis complex; Mycobacterium tuberculosis;	NCBI: 1773	4388140	99.61	0.00	Yes
NCBI Assembly	GCA_004107085.1	NCBI: cellular organisms; Bacteria; Terrabacteria group; Actinobacteria; Actinomycetia; Corynebacteriales; Mycobacteriaceae; Mycobacterium; Mycobacterium tuberculosis complex; Mycobacterium tuberculosis;	NCBI: 1773	4433550	99.94	0.00	Yes
NCBI Assembly	GCA_003392745.1	NCBI: cellular organisms; Bacteria; Terrabacteria group; Actinobacteria; Actinomycetia; Corynebacteriales; Mycobacteriaceae; Mycobacterium; Mycobacterium tuberculosis complex; Mycobacterium tuberculosis;	NCBI: 1773	4366650	99.69	0.33	Yes
NCBI Assembly	GCA_003431775.1	NCBI: cellular organisms; Bacteria; Terrabacteria group; Actinobacteria; Actinomycetia; Corynebacteriales; Mycobacteriaceae; Mycobacterium; Mycobacterium marinum;	NCBI: 1781	5780552	99.88	0.40	Yes
NCBI Assembly	GCA_002894725.1	NCBI: cellular organisms; Bacteria; Terrabacteria group; Actinobacteria; Actinomycetia; Corynebacteriales; Mycobacteriaceae; Mycobacterium; Mycobacterium avium complex (MAC); Mycobacterium avium; Mycobacterium avium subsp. hominissuis;	NCBI: 439334	5105678	99.94	0.73	Yes
NCBI Assembly	GCA_004107675.1	NCBI: cellular organisms; Bacteria; Terrabacteria group; Actinobacteria; Actinomycetia; Corynebacteriales; Mycobacteriaceae; Mycobacterium; Mycobacterium tuberculosis complex; Mycobacterium tuberculosis;	NCBI: 1773	4386779	99.94	0.00	Yes
NCBI Assembly	GCA_002895185.2	NCBI: cellular organisms; Bacteria; Terrabacteria group; Actinobacteria; Actinomycetia; Corynebacteriales; Mycobacteriaceae; Mycobacterium; Mycobacterium tuberculosis complex; Mycobacterium tuberculosis;	NCBI: 1773	4404064	98.70	0.00	Yes
NCBI Assembly	GCA_004106755.1	NCBI: cellular organisms; Bacteria; Terrabacteria group; Actinobacteria; Actinomycetia; Corynebacteriales; Mycobacteriaceae; Mycobacterium; Mycobacterium tuberculosis complex; Mycobacterium tuberculosis;	NCBI: 1773	4389635	99.61	0.00	Yes
NCBI Assembly	GCA_004111835.1	NCBI: cellular organisms; Bacteria; Terrabacteria group; Actinobacteria; Actinomycetia; Corynebacteriales; Mycobacteriaceae; Mycobacterium; Mycobacterium tuberculosis complex; Mycobacterium tuberculosis;	NCBI: 1773	4396566	99.61	0.00	Yes
NCBI Assembly	GCA_002802025.1	NCBI: cellular organisms; Bacteria; Terrabacteria group; Actinobacteria; Actinomycetia; Corynebacteriales; Mycobacteriaceae; Mycobacteroides; Mycobacteroides abscessus; Mycobacteroides abscessus subsp. abscessus;	NCBI: 1185650	5343193	100.00	0.00	Yes
NCBI Assembly	GCA_004105085.1	NCBI: cellular organisms; Bacteria; Terrabacteria group; Actinobacteria; Actinomycetia; Corynebacteriales; Mycobacteriaceae; Mycobacterium; Mycobacterium tuberculosis complex; Mycobacterium tuberculosis;	NCBI: 1773	4384138	99.94	0.00	Yes
NCBI Assembly	GCA_004108265.1	NCBI: cellular organisms; Bacteria; Terrabacteria group; Actinobacteria; Actinomycetia; Corynebacteriales; Mycobacteriaceae; Mycobacterium; Mycobacterium tuberculosis complex; Mycobacterium tuberculosis;	NCBI: 1773	4391319	99.94	0.00	Yes
NCBI Assembly	GCA_003392345.1	NCBI: cellular organisms; Bacteria; Terrabacteria group; Actinobacteria; Actinomycetia; Corynebacteriales; Mycobacteriaceae; Mycobacterium; Mycobacterium tuberculosis complex; Mycobacterium tuberculosis;	NCBI: 1773	4418412	99.94	0.57	Yes
NCBI Assembly	GCA_003392765.1	NCBI: cellular organisms; Bacteria; Terrabacteria group; Actinobacteria; Actinomycetia; Corynebacteriales; Mycobacteriaceae; Mycobacterium; Mycobacterium tuberculosis complex; Mycobacterium tuberculosis;	NCBI: 1773	4300683	99.86	0.00	Yes
NCBI Assembly	GCA_003431805.1	NCBI: cellular organisms; Bacteria; Terrabacteria group; Actinobacteria; Actinomycetia; Corynebacteriales; Mycobacteriaceae; Mycobacterium; Mycobacterium marinum;	NCBI: 1781	6416700	99.88	0.40	Yes

NCBI Assembly	GCA_004107095.1	NCBI: cellular organisms; Bacteria; Terrabacteria group; Actinobacteria; Actinomycetia; Corynebacteriales; Mycobacteriaceae; Mycobacterium; Mycobacterium tuberculosis complex; Mycobacterium tuberculosis;	NCBI: 1773	4397972	99.94	0.00	Yes
NCBI Assembly	GCA_003392065.1	NCBI: cellular organisms; Bacteria; Terrabacteria group; Actinobacteria; Actinomycetia; Corynebacteriales; Mycobacteriaceae; Mycobacterium; Mycobacterium tuberculosis complex; Mycobacterium tuberculosis;	NCBI: 1773	4406118	99.86	0.00	Yes
NCBI Assembly	GCA_002911515.1	NCBI: cellular organisms; Bacteria; Terrabacteria group; Actinobacteria; Actinomycetia; Corynebacteriales; Mycobacteriaceae; Mycobacterium; Mycobacterium marinum;	NCBI: 1781	6404072	99.88	1.19	Yes
NCBI Assembly	GCA_002802045.1	NCBI: cellular organisms; Bacteria; Terrabacteria group; Actinobacteria; Actinomycetia; Corynebacteriales; Mycobacteriaceae; Mycobacteroides; Mycobacteroides abscessus; Mycobacteroides abscessus subsp. abscessus;	NCBI: 1185650	5166549	100.00	0.00	Yes
NCBI Assembly	GCA_004107695.1	NCBI: cellular organisms; Bacteria; Terrabacteria group; Actinobacteria; Actinomycetia; Corynebacteriales; Mycobacteriaceae; Mycobacterium; Mycobacterium tuberculosis complex; Mycobacterium tuberculosis;	NCBI: 1773	4396245	99.94	0.00	Yes
NCBI Assembly	GCA_004106785.1	NCBI: cellular organisms; Bacteria; Terrabacteria group; Actinobacteria; Actinomycetia; Corynebacteriales; Mycobacteriaceae; Mycobacterium; Mycobacterium tuberculosis complex; Mycobacterium tuberculosis;	NCBI: 1773	4406818	99.61	0.00	Yes
NCBI Assembly	GCA_004105095.1	NCBI: cellular organisms; Bacteria; Terrabacteria group; Actinobacteria; Actinomycetia; Corynebacteriales; Mycobacteriaceae; Mycobacterium; Mycobacterium tuberculosis complex; Mycobacterium tuberculosis;	NCBI: 1773	4394919	99.94	0.00	Yes
NCBI Assembly	GCA_004111845.1	NCBI: cellular organisms; Bacteria; Terrabacteria group; Actinobacteria; Actinomycetia; Corynebacteriales; Mycobacteriaceae; Mycobacterium; Mycobacterium tuberculosis complex; Mycobacterium tuberculosis;	NCBI: 1773	4384200	99.94	0.00	Yes
NCBI Assembly	GCA_004108305.1	NCBI: cellular organisms; Bacteria; Terrabacteria group; Actinobacteria; Actinomycetia; Corynebacteriales; Mycobacteriaceae; Mycobacterium; Mycobacterium tuberculosis complex; Mycobacterium tuberculosis;	NCBI: 1773	4400497	99.61	0.00	Yes
NCBI Assembly	GCA_003392355.1	NCBI: cellular organisms; Bacteria; Terrabacteria group; Actinobacteria; Actinomycetia; Corynebacteriales; Mycobacteriaceae; Mycobacterium; Mycobacterium tuberculosis complex; Mycobacterium tuberculosis;	NCBI: 1773	4271698	99.86	0.28	Yes
NCBI Assembly	GCA_003392775.1	NCBI: cellular organisms; Bacteria; Terrabacteria group; Actinobacteria; Actinomycetia; Corynebacteriales; Mycobacteriaceae; Mycobacterium; Mycobacterium tuberculosis complex; Mycobacterium tuberculosis;	NCBI: 1773	4289986	99.36	0.00	Yes
NCBI Assembly	GCA_003515645.1	NCBI: cellular organisms; Bacteria; Terrabacteria group; Actinobacteria; Actinomycetia; Corynebacteriales; Mycobacteriaceae; Mycobacterium; Mycobacterium simiae complex; Mycobacterium shigaense;	NCBI: 722731	5207883	99.33	0.88	Yes
NCBI Assembly	GCA_004107115.1	NCBI: cellular organisms; Bacteria; Terrabacteria group; Actinobacteria; Actinomycetia; Corynebacteriales; Mycobacteriaceae; Mycobacterium; Mycobacterium tuberculosis complex; Mycobacterium tuberculosis;	NCBI: 1773	4392451	99.94	0.00	Yes
NCBI Assembly	GCA_002802055.1	NCBI: cellular organisms; Bacteria; Terrabacteria group; Actinobacteria; Actinomycetia; Corynebacteriales; Mycobacteriaceae; Mycobacteroides; Mycobacteroides abscessus; Mycobacteroides abscessus subsp. abscessus;	NCBI: 1185650	5109395	100.00	0.00	Yes
NCBI Assembly	GCA_003392075.1	NCBI: cellular organisms; Bacteria; Terrabacteria group; Actinobacteria; Actinomycetia; Corynebacteriales; Mycobacteriaceae; Mycobacterium; Mycobacterium tuberculosis complex; Mycobacterium tuberculosis;	NCBI: 1773	4323569	99.86	0.00	Yes
NCBI Assembly	GCA_002915215.1	NCBI: cellular organisms; Bacteria; Terrabacteria group; Actinobacteria; Actinomycetia; Corynebacteriales; Mycobacteriaceae; Mycobacterium; Mycobacterium tuberculosis complex; Mycobacterium tuberculosis;	NCBI: 1773	4361703	99.94	0.00	Yes
NCBI Assembly	GCA_004107705.1	NCBI: cellular organisms; Bacteria; Terrabacteria group; Actinobacteria; Actinomycetia; Corynebacteriales; Mycobacteriaceae; Mycobacterium; Mycobacterium tuberculosis complex; Mycobacterium tuberculosis;	NCBI: 1773	4381470	99.61	0.00	Yes
NCBI Assembly	GCA_004106795.1	NCBI: cellular organisms; Bacteria; Terrabacteria group; Actinobacteria; Actinomycetia; Corynebacteriales; Mycobacteriaceae; Mycobacterium; Mycobacterium tuberculosis complex; Mycobacterium tuberculosis;	NCBI: 1773	4395420	99.94	0.00	Yes

NCBI Assembly	GCA_003393005.1	NCBI: cellular organisms; Bacteria; Terrabacteria group; Actinobacteria; Actinomycetia; Corynebacteriales; Mycobacteriaceae; Mycobacterium; Mycobacterium tuberculosis complex; Mycobacterium tuberculosis;	NCBI: 1773	4335557	99.53	0.44	Yes
NCBI Assembly	GCA_004111875.1	NCBI: cellular organisms; Bacteria; Terrabacteria group; Actinobacteria; Actinomycetia; Corynebacteriales; Mycobacteriaceae; Mycobacterium; Mycobacterium tuberculosis complex; Mycobacterium tuberculosis;	NCBI: 1773	4425018	99.94	0.00	Yes
NCBI Assembly	GCA_004105125.1	NCBI: cellular organisms; Bacteria; Terrabacteria group; Actinobacteria; Actinomycetia; Corynebacteriales; Mycobacteriaceae; Mycobacterium; Mycobacterium tuberculosis complex; Mycobacterium tuberculosis;	NCBI: 1773	4403980	99.61	0.00	Yes
NCBI Assembly	GCA_003392405.1	NCBI: cellular organisms; Bacteria; Terrabacteria group; Actinobacteria; Actinomycetia; Corynebacteriales; Mycobacteriaceae; Mycobacterium; Mycobacterium tuberculosis complex; Mycobacterium tuberculosis;	NCBI: 1773	4549390	99.86	2.86	Yes
NCBI Assembly	GCA_003392785.1	NCBI: cellular organisms; Bacteria; Terrabacteria group; Actinobacteria; Actinomycetia; Corynebacteriales; Mycobacteriaceae; Mycobacterium; Mycobacterium tuberculosis complex; Mycobacterium tuberculosis;	NCBI: 1773	4336350	99.86	0.00	Yes
NCBI Assembly	GCA_002802065.1	NCBI: cellular organisms; Bacteria; Terrabacteria group; Actinobacteria; Actinomycetia; Corynebacteriales; Mycobacteriaceae; Mycobacteroides; Mycobacteroides abscessus; Mycobacteroides abscessus subsp. abscessus;	NCBI: 1185650	5016141	100.00	0.00	Yes
NCBI Assembly	GCA_003545775.1	NCBI: cellular organisms; Bacteria; Terrabacteria group; Actinobacteria; Actinomycetia; Corynebacteriales; Mycobacteriaceae; Mycobacterium; Mycobacterium tuberculosis complex; Mycobacterium tuberculosis;	NCBI: 1773	4306835	99.61	0.00	Yes
NCBI Assembly	GCA_002920655.1	NCBI: cellular organisms; Bacteria; Terrabacteria group; Actinobacteria; Actinomycetia; Corynebacteriales; Mycobacteriaceae; Mycobacterium; Mycobacterium kansasii;	NCBI: 1768	6374282	99.94	0.54	Yes
NCBI Assembly	GCA_003392095.1	NCBI: cellular organisms; Bacteria; Terrabacteria group; Actinobacteria; Actinomycetia; Corynebacteriales; Mycobacteriaceae; Mycobacterium; Mycobacterium tuberculosis complex; Mycobacterium tuberculosis;	NCBI: 1773	4331643	99.86	0.00	Yes
NCBI Assembly	GCA_004107125.1	NCBI: cellular organisms; Bacteria; Terrabacteria group; Actinobacteria; Actinomycetia; Corynebacteriales; Mycobacteriaceae; Mycobacterium; Mycobacterium tuberculosis complex; Mycobacterium tuberculosis;	NCBI: 1773	4410345	99.94	0.00	Yes
NCBI Assembly	GCA_004107745.1	NCBI: cellular organisms; Bacteria; Terrabacteria group; Actinobacteria; Actinomycetia; Corynebacteriales; Mycobacteriaceae; Mycobacterium; Mycobacterium tuberculosis complex; Mycobacterium tuberculosis;	NCBI: 1773	4384355	99.94	0.00	Yes
NCBI Assembly	GCA_003393015.1	NCBI: cellular organisms; Bacteria; Terrabacteria group; Actinobacteria; Actinomycetia; Corynebacteriales; Mycobacteriaceae; Mycobacterium; Mycobacterium tuberculosis complex; Mycobacterium tuberculosis;	NCBI: 1773	4287531	99.36	0.39	Yes
NCBI Assembly	GCA_004106805.1	NCBI: cellular organisms; Bacteria; Terrabacteria group; Actinobacteria; Actinomycetia; Corynebacteriales; Mycobacteriaceae; Mycobacterium; Mycobacterium tuberculosis complex; Mycobacterium tuberculosis;	NCBI: 1773	4393246	99.94	0.00	Yes
NCBI Assembly	GCA_004111885.1	NCBI: cellular organisms; Bacteria; Terrabacteria group; Actinobacteria; Actinomycetia; Corynebacteriales; Mycobacteriaceae; Mycobacterium; Mycobacterium tuberculosis complex; Mycobacterium tuberculosis;	NCBI: 1773	4418471	99.94	0.00	Yes
NCBI Assembly	GCA_003392415.1	NCBI: cellular organisms; Bacteria; Terrabacteria group; Actinobacteria; Actinomycetia; Corynebacteriales; Mycobacteriaceae; Mycobacterium; Mycobacterium tuberculosis complex; Mycobacterium tuberculosis;	NCBI: 1773	4512694	99.86	2.32	Yes
NCBI Assembly	GCA_004105165.1	NCBI: cellular organisms; Bacteria; Terrabacteria group; Actinobacteria; Actinomycetia; Corynebacteriales; Mycobacteriaceae; Mycobacterium; Mycobacterium tuberculosis complex; Mycobacterium tuberculosis;	NCBI: 1773	4384680	99.94	0.00	Yes
NCBI Assembly	GCA_002802075.1	NCBI: cellular organisms; Bacteria; Terrabacteria group; Actinobacteria; Actinomycetia; Corynebacteriales; Mycobacteriaceae; Mycobacteroides; Mycobacteroides abscessus; Mycobacteroides abscessus subsp. abscessus;	NCBI: 1185650	4932758	100.00	0.00	Yes
NCBI Assembly	GCA_003392815.1	NCBI: cellular organisms; Bacteria; Terrabacteria group; Actinobacteria; Actinomycetia; Corynebacteriales; Mycobacteriaceae; Mycobacterium; Mycobacterium tuberculosis complex; Mycobacterium tuberculosis;	NCBI: 1773	4339435	99.86	0.00	Yes

NCBI Assembly	GCA_003546285.1	NCBI: cellular organisms; Bacteria; Terrabacteria group; Actinobacteria; Actinomycetia; Corynebacteriales; Mycobacteriaceae; Mycobacterium; Mycobacterium tuberculosis complex; Mycobacterium tuberculosis;	NCBI: 1773	4286102	99.61	0.00	Yes
NCBI Assembly	GCA_002920665.1	NCBI: cellular organisms; Bacteria; Terrabacteria group; Actinobacteria; Actinomycetia; Corynebacteriales; Mycobacteriaceae; Mycobacterium; Mycobacterium kansasii;	NCBI: 1768	6225838	99.94	0.79	Yes
NCBI Assembly	GCA_003392105.1	NCBI: cellular organisms; Bacteria; Terrabacteria group; Actinobacteria; Actinomycetia; Corynebacteriales; Mycobacteriaceae; Mycobacterium; Mycobacterium tuberculosis complex; Mycobacterium tuberculosis;	NCBI: 1773	4635877	99.69	3.10	Yes
NCBI Assembly	GCA_004107145.1	NCBI: cellular organisms; Bacteria; Terrabacteria group; Actinobacteria; Actinomycetia; Corynebacteriales; Mycobacteriaceae; Mycobacterium; Mycobacterium tuberculosis complex; Mycobacterium tuberculosis;	NCBI: 1773	4397112	99.94	0.00	Yes
NCBI Assembly	GCA_003393025.1	NCBI: cellular organisms; Bacteria; Terrabacteria group; Actinobacteria; Actinomycetia; Corynebacteriales; Mycobacteriaceae; Mycobacterium; Mycobacterium tuberculosis complex; Mycobacterium tuberculosis;	NCBI: 1773	4256721	99.25	0.33	Yes
NCBI Assembly	GCA_004107765.1	NCBI: cellular organisms; Bacteria; Terrabacteria group; Actinobacteria; Actinomycetia; Corynebacteriales; Mycobacteriaceae; Mycobacterium; Mycobacterium tuberculosis complex; Mycobacterium tuberculosis;	NCBI: 1773	4375217	99.94	0.00	Yes
NCBI Assembly	GCA_004106835.1	NCBI: cellular organisms; Bacteria; Terrabacteria group; Actinobacteria; Actinomycetia; Corynebacteriales; Mycobacteriaceae; Mycobacterium; Mycobacterium tuberculosis complex; Mycobacterium tuberculosis;	NCBI: 1773	4391507	99.94	0.00	Yes
NCBI Assembly	GCA_003392425.1	NCBI: cellular organisms; Bacteria; Terrabacteria group; Actinobacteria; Actinomycetia; Corynebacteriales; Mycobacteriaceae; Mycobacterium; Mycobacterium tuberculosis complex; Mycobacterium tuberculosis;	NCBI: 1773	4363658	99.86	0.00	Yes
NCBI Assembly	GCA_003399415.1	NCBI: cellular organisms; Bacteria; Terrabacteria group; Actinobacteria; Actinomycetia; Corynebacteriales; Mycobacteriaceae; Mycobacterium; Mycobacterium tuberculosis complex; Mycobacterium tuberculosis;	NCBI: 1773	4354482	99.86	0.00	Yes
NCBI Assembly	GCA_004111925.1	NCBI: cellular organisms; Bacteria; Terrabacteria group; Actinobacteria; Actinomycetia; Corynebacteriales; Mycobacteriaceae; Mycobacterium; Mycobacterium tuberculosis complex; Mycobacterium tuberculosis;	NCBI: 1773	4382871	99.61	0.00	Yes
NCBI Assembly	GCA_002802125.1	NCBI: cellular organisms; Bacteria; Terrabacteria group; Actinobacteria; Actinomycetia; Corynebacteriales; Mycobacteriaceae; Mycobacteroides; Mycobacteroides abscessus; Mycobacteroides abscessus subsp. abscessus;	NCBI: 1185650	5251430	100.00	0.00	Yes
NCBI Assembly	GCA_003392845.1	NCBI: cellular organisms; Bacteria; Terrabacteria group; Actinobacteria; Actinomycetia; Corynebacteriales; Mycobacteriaceae; Mycobacterium; Mycobacterium tuberculosis complex; Mycobacterium tuberculosis;	NCBI: 1773	4375394	99.86	0.00	Yes
NCBI Assembly	GCA_002920675.1	NCBI: cellular organisms; Bacteria; Terrabacteria group; Actinobacteria; Actinomycetia; Corynebacteriales; Mycobacteriaceae; Mycobacterium; Mycobacterium kansasii;	NCBI: 1768	6282326	99.94	0.46	Yes
NCBI Assembly	GCA_003546305.1	NCBI: cellular organisms; Bacteria; Terrabacteria group; Actinobacteria; Actinomycetia; Corynebacteriales; Mycobacteriaceae; Mycobacterium; Mycobacterium tuberculosis complex; Mycobacterium tuberculosis;	NCBI: 1773	4296142	99.61	0.00	Yes
NCBI Assembly	GCA_003392135.1	NCBI: cellular organisms; Bacteria; Terrabacteria group; Actinobacteria; Actinomycetia; Corynebacteriales; Mycobacteriaceae; Mycobacterium; Mycobacterium tuberculosis complex; Mycobacterium tuberculosis;	NCBI: 1773	4358466	99.86	0.00	Yes
NCBI Assembly	GCA_004107185.1	NCBI: cellular organisms; Bacteria; Terrabacteria group; Actinobacteria; Actinomycetia; Corynebacteriales; Mycobacteriaceae; Mycobacterium; Mycobacterium tuberculosis complex; Mycobacterium tuberculosis;	NCBI: 1773	4408731	99.94	0.00	Yes
NCBI Assembly	GCA_003393035.1	NCBI: cellular organisms; Bacteria; Terrabacteria group; Actinobacteria; Actinomycetia; Corynebacteriales; Mycobacteriaceae; Mycobacterium; Mycobacterium tuberculosis complex; Mycobacterium tuberculosis;	NCBI: 1773	4251109	99.79	0.86	Yes
NCBI Assembly	GCA_004107775.1	NCBI: cellular organisms; Bacteria; Terrabacteria group; Actinobacteria; Actinomycetia; Corynebacteriales; Mycobacteriaceae; Mycobacterium; Mycobacterium tuberculosis complex; Mycobacterium tuberculosis;	NCBI: 1773	4383572	99.94	0.00	Yes

NCBI Assembly	GCA_003392435.1	NCBI: cellular organisms; Bacteria; Terrabacteria group; Actinobacteria; Actinomycetia; Corynebacteriales; Mycobacteriaceae; Mycobacterium; Mycobacterium tuberculosis complex; Mycobacterium tuberculosis;	NCBI: 1773	4366083	99.86	0.00	Yes
NCBI Assembly	GCA_003401625.1	NCBI: cellular organisms; Bacteria; Terrabacteria group; Actinobacteria; Actinomycetia; Corynebacteriales; Mycobacteriaceae; Mycobacterium; Mycobacterium tuberculosis complex; Mycobacterium tuberculosis;	NCBI: 1773	4372455	99.86	0.00	Yes
NCBI Assembly	GCA_002802135.1	NCBI: cellular organisms; Bacteria; Terrabacteria group; Actinobacteria; Actinomycetia; Corynebacteriales; Mycobacteriaceae; Mycobacteroides; Mycobacteroides abscessus; Mycobacteroides abscessus subsp. abscessus;	NCBI: 1185650	5110690	100.00	0.00	Yes
NCBI Assembly	GCA_004106855.1	NCBI: cellular organisms; Bacteria; Terrabacteria group; Actinobacteria; Actinomycetia; Corynebacteriales; Mycobacteriaceae; Mycobacterium; Mycobacterium tuberculosis complex; Mycobacterium tuberculosis;	NCBI: 1773	4390062	99.94	0.00	Yes
NCBI Assembly	GCA_004111935.1	NCBI: cellular organisms; Bacteria; Terrabacteria group; Actinobacteria; Actinomycetia; Corynebacteriales; Mycobacteriaceae; Mycobacterium; Mycobacterium tuberculosis complex; Mycobacterium tuberculosis;	NCBI: 1773	4389224	99.61	0.00	Yes
NCBI Assembly	GCA_002920705.1	NCBI: cellular organisms; Bacteria; Terrabacteria group; Actinobacteria; Actinomycetia; Corynebacteriales; Mycobacteriaceae; Mycobacterium; Mycobacterium kansasii;	NCBI: 1768	6411468	99.94	0.96	Yes
NCBI Assembly	GCA_003392855.1	NCBI: cellular organisms; Bacteria; Terrabacteria group; Actinobacteria; Actinomycetia; Corynebacteriales; Mycobacteriaceae; Mycobacterium; Mycobacterium tuberculosis complex; Mycobacterium tuberculosis;	NCBI: 1773	4355880	99.86	0.00	Yes
NCBI Assembly	GCA_003546315.1	NCBI: cellular organisms; Bacteria; Terrabacteria group; Actinobacteria; Actinomycetia; Corynebacteriales; Mycobacteriaceae; Mycobacterium; Mycobacterium tuberculosis complex; Mycobacterium tuberculosis;	NCBI: 1773	4288285	99.61	0.39	Yes
NCBI Assembly	GCA_003392155.1	NCBI: cellular organisms; Bacteria; Terrabacteria group; Actinobacteria; Actinomycetia; Corynebacteriales; Mycobacteriaceae; Mycobacterium; Mycobacterium tuberculosis complex; Mycobacterium tuberculosis;	NCBI: 1773	4354153	99.86	0.44	Yes
NCBI Assembly	GCA_004107195.1	NCBI: cellular organisms; Bacteria; Terrabacteria group; Actinobacteria; Actinomycetia; Corynebacteriales; Mycobacteriaceae; Mycobacterium; Mycobacterium tuberculosis complex; Mycobacterium tuberculosis;	NCBI: 1773	4377870	99.94	0.00	Yes
NCBI Assembly	GCA_002802145.1	NCBI: cellular organisms; Bacteria; Terrabacteria group; Actinobacteria; Actinomycetia; Corynebacteriales; Mycobacteriaceae; Mycobacteroides; Mycobacteroides abscessus; Mycobacteroides abscessus subsp. abscessus;	NCBI: 1185650	5279956	100.00	0.00	Yes
NCBI Assembly	GCA_003393085.1	NCBI: cellular organisms; Bacteria; Terrabacteria group; Actinobacteria; Actinomycetia; Corynebacteriales; Mycobacteriaceae; Mycobacterium; Mycobacterium tuberculosis complex; Mycobacterium tuberculosis;	NCBI: 1773	4257532	99.53	0.33	Yes
NCBI Assembly	GCA_003392485.1	NCBI: cellular organisms; Bacteria; Terrabacteria group; Actinobacteria; Actinomycetia; Corynebacteriales; Mycobacteriaceae; Mycobacterium; Mycobacterium tuberculosis complex; Mycobacterium tuberculosis;	NCBI: 1773	4381819	99.86	0.00	Yes
NCBI Assembly	GCA_003402475.1	NCBI: cellular organisms; Bacteria; Terrabacteria group; Actinobacteria; Actinomycetia; Corynebacteriales; Mycobacteriaceae; Mycobacterium; unclassified Mycobacterium;	NCBI: 2049453	5006351	100.00	0.09	Yes
NCBI Assembly	GCA_004107785.1	NCBI: cellular organisms; Bacteria; Terrabacteria group; Actinobacteria; Actinomycetia; Corynebacteriales; Mycobacteriaceae; Mycobacterium; Mycobacterium tuberculosis complex; Mycobacterium tuberculosis;	NCBI: 1773	4383394	99.94	0.00	Yes
NCBI Assembly	GCA_002920735.1	NCBI: cellular organisms; Bacteria; Terrabacteria group; Actinobacteria; Actinomycetia; Corynebacteriales; Mycobacteriaceae; Mycobacterium; Mycobacterium kansasii;	NCBI: 1768	6464047	99.94	0.46	Yes
NCBI Assembly	GCA_004106885.1	NCBI: cellular organisms; Bacteria; Terrabacteria group; Actinobacteria; Actinomycetia; Corynebacteriales; Mycobacteriaceae; Mycobacterium; Mycobacterium tuberculosis complex; Mycobacterium tuberculosis;	NCBI: 1773	4388519	99.94	0.00	Yes
NCBI Assembly	GCA_004111945.1	NCBI: cellular organisms; Bacteria; Terrabacteria group; Actinobacteria; Actinomycetia; Corynebacteriales; Mycobacteriaceae; Mycobacterium; Mycobacterium tuberculosis complex; Mycobacterium tuberculosis;	NCBI: 1773	4396062	99.94	0.00	Yes

NCBI Assembly	GCA_003392865.1	NCBI: cellular organisms; Bacteria; Terrabacteria group; Actinobacteria; Actinomycetia; Corynebacteriales; Mycobacteriaceae; Mycobacterium; Mycobacterium tuberculosis complex; Mycobacterium tuberculosis;	NCBI: 1773	4297525	99.69	0.00	Yes
NCBI Assembly	GCA_003546345.1	NCBI: cellular organisms; Bacteria; Terrabacteria group; Actinobacteria; Actinomycetia; Corynebacteriales; Mycobacteriaceae; Mycobacterium; Mycobacterium tuberculosis complex; Mycobacterium tuberculosis;	NCBI: 1773	4306878	99.61	0.00	Yes
NCBI Assembly	GCA_003392165.1	NCBI: cellular organisms; Bacteria; Terrabacteria group; Actinobacteria; Actinomycetia; Corynebacteriales; Mycobacteriaceae; Mycobacterium; Mycobacterium tuberculosis complex; Mycobacterium tuberculosis;	NCBI: 1773	4320263	99.86	0.00	Yes
NCBI Assembly	GCA_002802165.1	NCBI: cellular organisms; Bacteria; Terrabacteria group; Actinobacteria; Actinomycetia; Corynebacteriales; Mycobacteriaceae; Mycobacteroides; Mycobacteroides abscessus; Mycobacteroides abscessus subsp. abscessus;	NCBI: 1185650	5139244	100.00	0.00	Yes
NCBI Assembly	GCA_004107205.1	NCBI: cellular organisms; Bacteria; Terrabacteria group; Actinobacteria; Actinomycetia; Corynebacteriales; Mycobacteriaceae; Mycobacterium; Mycobacterium tuberculosis complex; Mycobacterium tuberculosis;	NCBI: 1773	4403248	99.94	0.00	Yes
NCBI Assembly	GCA_003393095.1	NCBI: cellular organisms; Bacteria; Terrabacteria group; Actinobacteria; Actinomycetia; Corynebacteriales; Mycobacteriaceae; Mycobacterium; Mycobacterium tuberculosis complex; Mycobacterium tuberculosis;	NCBI: 1773	4444856	99.94	0.33	Yes
NCBI Assembly	GCA_003392495.1	NCBI: cellular organisms; Bacteria; Terrabacteria group; Actinobacteria; Actinomycetia; Corynebacteriales; Mycobacteriaceae; Mycobacterium; Mycobacterium tuberculosis complex; Mycobacterium tuberculosis;	NCBI: 1773	4282983	99.69	0.29	Yes
NCBI Assembly	GCA_003408535.1	NCBI: cellular organisms; Bacteria; Terrabacteria group; Actinobacteria; Actinomycetia; Corynebacteriales; Mycobacteriaceae; Mycobacterium; Mycobacterium avium complex (MAC); Mycobacterium avium; Mycobacterium avium subsp. hominissuis;	NCBI: 439334	5352499	99.94	0.00	Yes
NCBI Assembly	GCA_004107795.1	NCBI: cellular organisms; Bacteria; Terrabacteria group; Actinobacteria; Actinomycetia; Corynebacteriales; Mycobacteriaceae; Mycobacterium; Mycobacterium tuberculosis complex; Mycobacterium tuberculosis;	NCBI: 1773	4370099	99.94	0.00	Yes
NCBI Assembly	GCA_002920755.1	NCBI: cellular organisms; Bacteria; Terrabacteria group; Actinobacteria; Actinomycetia; Corynebacteriales; Mycobacteriaceae; Mycobacterium; Mycobacterium kansasii;	NCBI: 1768	6284879	99.83	0.71	Yes
NCBI Assembly	GCA_003392895.1	NCBI: cellular organisms; Bacteria; Terrabacteria group; Actinobacteria; Actinomycetia; Corynebacteriales; Mycobacteriaceae; Mycobacterium; Mycobacterium tuberculosis complex; Mycobacterium tuberculosis;	NCBI: 1773	4280188	99.86	0.11	Yes
NCBI Assembly	GCA_004106895.1	NCBI: cellular organisms; Bacteria; Terrabacteria group; Actinobacteria; Actinomycetia; Corynebacteriales; Mycobacteriaceae; Mycobacterium; Mycobacterium tuberculosis complex; Mycobacterium tuberculosis;	NCBI: 1773	4396245	99.94	0.00	Yes
NCBI Assembly	GCA_004111955.1	NCBI: cellular organisms; Bacteria; Terrabacteria group; Actinobacteria; Actinomycetia; Corynebacteriales; Mycobacteriaceae; Mycobacterium; Mycobacterium tuberculosis complex; Mycobacterium tuberculosis;	NCBI: 1773	4379951	99.61	0.00	Yes
NCBI Assembly	GCA_003546355.1	NCBI: cellular organisms; Bacteria; Terrabacteria group; Actinobacteria; Actinomycetia; Corynebacteriales; Mycobacteriaceae; Mycobacterium; Mycobacterium tuberculosis complex; Mycobacterium tuberculosis;	NCBI: 1773	4289463	99.61	0.33	Yes
NCBI Assembly	GCA_003392205.1	NCBI: cellular organisms; Bacteria; Terrabacteria group; Actinobacteria; Actinomycetia; Corynebacteriales; Mycobacteriaceae; Mycobacterium; Mycobacterium tuberculosis complex; Mycobacterium tuberculosis;	NCBI: 1773	4284320	99.86	0.00	Yes
NCBI Assembly	GCA_002802205.1	NCBI: cellular organisms; Bacteria; Terrabacteria group; Actinobacteria; Actinomycetia; Corynebacteriales; Mycobacteriaceae; Mycobacteroides; Mycobacteroides abscessus; Mycobacteroides abscessus subsp. abscessus;	NCBI: 1185650	5163058	100.00	1.26	Yes
NCBI Assembly	GCA_004107245.1	NCBI: cellular organisms; Bacteria; Terrabacteria group; Actinobacteria; Actinomycetia; Corynebacteriales; Mycobacteriaceae; Mycobacterium; Mycobacterium tuberculosis complex; Mycobacterium tuberculosis;	NCBI: 1773	4384733	99.94	0.00	Yes
NCBI Assembly	GCA_002920775.1	NCBI: cellular organisms; Bacteria; Terrabacteria group; Actinobacteria; Actinomycetia; Corynebacteriales; Mycobacteriaceae; Mycobacterium; Mycobacterium kansasii;	NCBI: 1768	6307487	99.94	0.54	Yes

NCBI Assembly	GCA_003393105.1	NCBI: cellular organisms; Bacteria; Terrabacteria group; Actinobacteria; Actinomycetia; Corynebacteriales; Mycobacteriaceae; Mycobacterium; Mycobacterium tuberculosis complex; Mycobacterium tuberculosis;	NCBI: 1773	4329024	99.86	0.33	Yes
NCBI Assembly	GCA_003392525.1	NCBI: cellular organisms; Bacteria; Terrabacteria group; Actinobacteria; Actinomycetia; Corynebacteriales; Mycobacteriaceae; Mycobacterium; Mycobacterium tuberculosis complex; Mycobacterium tuberculosis;	NCBI: 1773	4306555	99.53	0.33	Yes
NCBI Assembly	GCA_003408705.1	NCBI: cellular organisms; Bacteria; Terrabacteria group; Actinobacteria; Actinomycetia; Corynebacteriales; Mycobacteriaceae; Mycobacterium; Mycobacterium uberis;	NCBI: 2162698	3122721	89.37	0.23	No
NCBI Assembly	GCA_004107845.1	NCBI: cellular organisms; Bacteria; Terrabacteria group; Actinobacteria; Actinomycetia; Corynebacteriales; Mycobacteriaceae; Mycobacterium; Mycobacterium tuberculosis complex; Mycobacterium tuberculosis;	NCBI: 1773	4380949	99.94	0.00	Yes
NCBI Assembly	GCA_003392915.1	NCBI: cellular organisms; Bacteria; Terrabacteria group; Actinobacteria; Actinomycetia; Corynebacteriales; Mycobacteriaceae; Mycobacterium; Mycobacterium tuberculosis complex; Mycobacterium tuberculosis;	NCBI: 1773	4252094	99.69	1.03	Yes
NCBI Assembly	GCA_004106905.1	NCBI: cellular organisms; Bacteria; Terrabacteria group; Actinobacteria; Actinomycetia; Corynebacteriales; Mycobacteriaceae; Mycobacterium; Mycobacterium tuberculosis complex; Mycobacterium tuberculosis;	NCBI: 1773	4392902	99.61	0.00	Yes
NCBI Assembly	GCA_004111975.1	NCBI: cellular organisms; Bacteria; Terrabacteria group; Actinobacteria; Actinomycetia; Corynebacteriales; Mycobacteriaceae; Mycobacterium; Mycobacterium tuberculosis complex; Mycobacterium tuberculosis;	NCBI: 1773	4381947	99.94	0.00	Yes
NCBI Assembly	GCA_003546385.1	NCBI: cellular organisms; Bacteria; Terrabacteria group; Actinobacteria; Actinomycetia; Corynebacteriales; Mycobacteriaceae; Mycobacterium; Mycobacterium tuberculosis complex; Mycobacterium tuberculosis;	NCBI: 1773	4326765	99.61	0.00	Yes
NCBI Assembly	GCA_003392225.1	NCBI: cellular organisms; Bacteria; Terrabacteria group; Actinobacteria; Actinomycetia; Corynebacteriales; Mycobacteriaceae; Mycobacterium; Mycobacterium tuberculosis complex; Mycobacterium tuberculosis;	NCBI: 1773	4315600	99.86	0.11	Yes
NCBI Assembly	GCA_002802225.1	NCBI: cellular organisms; Bacteria; Terrabacteria group; Actinobacteria; Actinomycetia; Corynebacteriales; Mycobacteriaceae; Mycobacteroides; Mycobacteroides abscessus; Mycobacteroides abscessus subsp. abscessus;	NCBI: 1185650	4850386	100.00	0.00	Yes
NCBI Assembly	GCA_002920795.1	NCBI: cellular organisms; Bacteria; Terrabacteria group; Actinobacteria; Actinomycetia; Corynebacteriales; Mycobacteriaceae; Mycobacterium; Mycobacterium kansasii;	NCBI: 1768	6289619	99.94	0.46	Yes
NCBI Assembly	GCA_003393145.1	NCBI: cellular organisms; Bacteria; Terrabacteria group; Actinobacteria; Actinomycetia; Corynebacteriales; Mycobacteriaceae; Mycobacterium; Mycobacterium tuberculosis complex; Mycobacterium tuberculosis;	NCBI: 1773	4256704	99.86	0.17	Yes
NCBI Assembly	GCA_003392535.1	NCBI: cellular organisms; Bacteria; Terrabacteria group; Actinobacteria; Actinomycetia; Corynebacteriales; Mycobacteriaceae; Mycobacterium; Mycobacterium tuberculosis complex; Mycobacterium tuberculosis;	NCBI: 1773	4358757	99.86	0.50	Yes
NCBI Assembly	GCA_003431565.1	NCBI: cellular organisms; Bacteria; Terrabacteria group; Actinobacteria; Actinomycetia; Corynebacteriales; Mycobacteriaceae; Mycobacterium; Mycobacterium marinum;	NCBI: 1781	6395074	99.88	0.23	Yes
NCBI Assembly	GCA_004107265.1	NCBI: cellular organisms; Bacteria; Terrabacteria group; Actinobacteria; Actinomycetia; Corynebacteriales; Mycobacteriaceae; Mycobacterium; Mycobacterium tuberculosis complex; Mycobacterium tuberculosis;	NCBI: 1773	4351917	99.94	0.00	Yes
NCBI Assembly	GCA_004107865.1	NCBI: cellular organisms; Bacteria; Terrabacteria group; Actinobacteria; Actinomycetia; Corynebacteriales; Mycobacteriaceae; Mycobacterium; Mycobacterium tuberculosis complex; Mycobacterium tuberculosis;	NCBI: 1773	4381916	99.94	0.00	Yes
NCBI Assembly	GCA_003392925.1	NCBI: cellular organisms; Bacteria; Terrabacteria group; Actinobacteria; Actinomycetia; Corynebacteriales; Mycobacteriaceae; Mycobacterium; Mycobacterium tuberculosis complex; Mycobacterium tuberculosis;	NCBI: 1773	4238649	98.79	0.69	Yes
NCBI Assembly	GCA_004106915.1	NCBI: cellular organisms; Bacteria; Terrabacteria group; Actinobacteria; Actinomycetia; Corynebacteriales; Mycobacteriaceae; Mycobacterium; Mycobacterium tuberculosis complex; Mycobacterium tuberculosis;	NCBI: 1773	4398652	99.61	0.00	Yes

NCBI Assembly	GCA_003546395.1	NCBI: cellular organisms; Bacteria; Terrabacteria group; Actinobacteria; Actinomycetia; Corynebacteriales; Mycobacteriaceae; Mycobacterium; Mycobacterium tuberculosis complex; Mycobacterium tuberculosis;	NCBI: 1773	4329177	99.61	0.00	Yes
NCBI Assembly	GCA_004112025.1	NCBI: cellular organisms; Bacteria; Terrabacteria group; Actinobacteria; Actinomycetia; Corynebacteriales; Mycobacteriaceae; Mycobacterium; Mycobacterium tuberculosis complex; Mycobacterium tuberculosis;	NCBI: 1773	4392906	99.94	0.00	Yes
NCBI Assembly	GCA_003392245.1	NCBI: cellular organisms; Bacteria; Terrabacteria group; Actinobacteria; Actinomycetia; Corynebacteriales; Mycobacteriaceae; Mycobacterium; Mycobacterium tuberculosis complex; Mycobacterium tuberculosis;	NCBI: 1773	4315413	99.86	0.00	Yes
NCBI Assembly	GCA_002802285.1	NCBI: cellular organisms; Bacteria; Terrabacteria group; Actinobacteria; Actinomycetia; Corynebacteriales; Mycobacteriaceae; Mycobacteroides; Mycobacteroides abscessus; Mycobacteroides abscessus subsp. abscessus;	NCBI: 1185650	4850057	100.00	0.00	Yes
NCBI Assembly	GCA_002920805.1	NCBI: cellular organisms; Bacteria; Terrabacteria group; Actinobacteria; Actinomycetia; Corynebacteriales; Mycobacteriaceae; Mycobacterium; Mycobacterium kansasii;	NCBI: 1768	6213268	99.61	0.46	Yes
NCBI Assembly	GCA_003393155.1	NCBI: cellular organisms; Bacteria; Terrabacteria group; Actinobacteria; Actinomycetia; Corynebacteriales; Mycobacteriaceae; Mycobacterium; Mycobacterium tuberculosis complex; Mycobacterium tuberculosis;	NCBI: 1773	4359780	99.86	0.00	Yes
NCBI Assembly	GCA_003431575.1	NCBI: cellular organisms; Bacteria; Terrabacteria group; Actinobacteria; Actinomycetia; Corynebacteriales; Mycobacteriaceae; Mycobacterium; Mycobacterium marinum;	NCBI: 1781	6521082	99.88	0.10	Yes
NCBI Assembly	GCA_003392545.1	NCBI: cellular organisms; Bacteria; Terrabacteria group; Actinobacteria; Actinomycetia; Corynebacteriales; Mycobacteriaceae; Mycobacterium; Mycobacterium tuberculosis complex; Mycobacterium tuberculosis;	NCBI: 1773	4300430	99.86	0.11	Yes
NCBI Assembly	GCA_004107285.1	NCBI: cellular organisms; Bacteria; Terrabacteria group; Actinobacteria; Actinomycetia; Corynebacteriales; Mycobacteriaceae; Mycobacterium; Mycobacterium tuberculosis complex; Mycobacterium tuberculosis;	NCBI: 1773	4389164	99.94	0.00	Yes
NCBI Assembly	GCA_003392945.1	NCBI: cellular organisms; Bacteria; Terrabacteria group; Actinobacteria; Actinomycetia; Corynebacteriales; Mycobacteriaceae; Mycobacterium; Mycobacterium tuberculosis complex; Mycobacterium tuberculosis;	NCBI: 1773	4274644	99.53	0.08	Yes
NCBI Assembly	GCA_004107875.1	NCBI: cellular organisms; Bacteria; Terrabacteria group; Actinobacteria; Actinomycetia; Corynebacteriales; Mycobacteriaceae; Mycobacterium; Mycobacterium tuberculosis complex; Mycobacterium tuberculosis;	NCBI: 1773	4390263	99.94	0.00	Yes
NCBI Assembly	GCA_003392255.1	NCBI: cellular organisms; Bacteria; Terrabacteria group; Actinobacteria; Actinomycetia; Corynebacteriales; Mycobacteriaceae; Mycobacterium; Mycobacterium tuberculosis complex; Mycobacterium tuberculosis;	NCBI: 1773	4284905	99.69	0.00	Yes
NCBI Assembly	GCA_004106925.1	NCBI: cellular organisms; Bacteria; Terrabacteria group; Actinobacteria; Actinomycetia; Corynebacteriales; Mycobacteriaceae; Mycobacterium; Mycobacterium tuberculosis complex; Mycobacterium tuberculosis;	NCBI: 1773	4417710	99.61	0.00	Yes
NCBI Assembly	GCA_003546425.1	NCBI: cellular organisms; Bacteria; Terrabacteria group; Actinobacteria; Actinomycetia; Corynebacteriales; Mycobacteriaceae; Mycobacterium; Mycobacterium tuberculosis complex; Mycobacterium tuberculosis;	NCBI: 1773	4248907	99.61	0.00	Yes
NCBI Assembly	GCA_002802295.1	NCBI: cellular organisms; Bacteria; Terrabacteria group; Actinobacteria; Actinomycetia; Corynebacteriales; Mycobacteriaceae; Mycobacteroides; Mycobacteroides abscessus; Mycobacteroides abscessus subsp. abscessus;	NCBI: 1185650	5034122	100.00	0.00	Yes
NCBI Assembly	GCA_002920815.1	NCBI: cellular organisms; Bacteria; Terrabacteria group; Actinobacteria; Actinomycetia; Corynebacteriales; Mycobacteriaceae; Mycobacterium; Mycobacterium kansasii;	NCBI: 1768	6282923	99.67	1.10	Yes
NCBI Assembly	GCA_004112035.1	NCBI: cellular organisms; Bacteria; Terrabacteria group; Actinobacteria; Actinomycetia; Corynebacteriales; Mycobacteriaceae; Mycobacterium; Mycobacterium tuberculosis complex; Mycobacterium tuberculosis;	NCBI: 1773	4387522	99.94	0.00	Yes
NCBI Assembly	GCA_003393165.1	NCBI: cellular organisms; Bacteria; Terrabacteria group; Actinobacteria; Actinomycetia; Corynebacteriales; Mycobacteriaceae; Mycobacterium; Mycobacterium tuberculosis complex; Mycobacterium tuberculosis;	NCBI: 1773	4381938	99.86	0.00	Yes

NCBI Assembly	GCA_003431585.1	NCBI: cellular organisms; Bacteria; Terrabacteria group; Actinobacteria; Actinomycetia; Corynebacteriales; Mycobacteriaceae; Mycobacterium; Mycobacterium marinum;	NCBI: 1781	6324388	99.71	0.34	Yes
NCBI Assembly	GCA_003392585.1	NCBI: cellular organisms; Bacteria; Terrabacteria group; Actinobacteria; Actinomycetia; Corynebacteriales; Mycobacteriaceae; Mycobacterium; Mycobacterium tuberculosis complex; Mycobacterium tuberculosis;	NCBI: 1773	4408433	99.86	0.33	Yes
NCBI Assembly	GCA_003392965.1	NCBI: cellular organisms; Bacteria; Terrabacteria group; Actinobacteria; Actinomycetia; Corynebacteriales; Mycobacteriaceae; Mycobacterium; Mycobacterium tuberculosis complex; Mycobacterium tuberculosis;	NCBI: 1773	4326136	99.86	0.00	Yes
NCBI Assembly	GCA_004107295.1	NCBI: cellular organisms; Bacteria; Terrabacteria group; Actinobacteria; Actinomycetia; Corynebacteriales; Mycobacteriaceae; Mycobacterium; Mycobacterium tuberculosis complex; Mycobacterium tuberculosis;	NCBI: 1773	4392849	99.94	0.00	Yes
NCBI Assembly	GCA_003392275.1	NCBI: cellular organisms; Bacteria; Terrabacteria group; Actinobacteria; Actinomycetia; Corynebacteriales; Mycobacteriaceae; Mycobacterium; Mycobacterium tuberculosis complex; Mycobacterium tuberculosis;	NCBI: 1773	4326798	99.86	0.00	Yes
NCBI Assembly	GCA_002802345.1	NCBI: cellular organisms; Bacteria; Terrabacteria group; Actinobacteria; Actinomycetia; Corynebacteriales; Mycobacteriaceae; Mycobacteroides; Mycobacteroides abscessus; Mycobacteroides abscessus subsp. abscessus;	NCBI: 1185650	5015903	100.00	0.00	Yes
NCBI Assembly	GCA_004107885.1	NCBI: cellular organisms; Bacteria; Terrabacteria group; Actinobacteria; Actinomycetia; Corynebacteriales; Mycobacteriaceae; Mycobacterium; Mycobacterium tuberculosis complex; Mycobacterium tuberculosis;	NCBI: 1773	4387068	99.94	0.00	Yes
NCBI Assembly	GCA_003546445.1	NCBI: cellular organisms; Bacteria; Terrabacteria group; Actinobacteria; Actinomycetia; Corynebacteriales; Mycobacteriaceae; Mycobacterium; Mycobacterium tuberculosis complex; Mycobacterium tuberculosis;	NCBI: 1773	4304047	99.61	0.33	Yes
NCBI Assembly	GCA_002920845.1	NCBI: cellular organisms; Bacteria; Terrabacteria group; Actinobacteria; Actinomycetia; Corynebacteriales; Mycobacteriaceae; Mycobacterium; Mycobacterium kansasii;	NCBI: 1768	6228381	99.61	0.46	Yes
NCBI Assembly	GCA_004106985.1	NCBI: cellular organisms; Bacteria; Terrabacteria group; Actinobacteria; Actinomycetia; Corynebacteriales; Mycobacteriaceae; Mycobacterium; Mycobacterium tuberculosis complex; Mycobacterium tuberculosis;	NCBI: 1773	4392357	99.94	0.00	Yes
NCBI Assembly	GCA_004112055.1	NCBI: cellular organisms; Bacteria; Terrabacteria group; Actinobacteria; Actinomycetia; Corynebacteriales; Mycobacteriaceae; Mycobacterium; Mycobacterium tuberculosis complex; Mycobacterium tuberculosis;	NCBI: 1773	4383403	99.94	0.00	Yes
NCBI Assembly	GCA_003431605.1	NCBI: cellular organisms; Bacteria; Terrabacteria group; Actinobacteria; Actinomycetia; Corynebacteriales; Mycobacteriaceae; Mycobacterium; Mycobacterium marinum;	NCBI: 1781	6319947	99.88	0.23	Yes
NCBI Assembly	GCA_003392595.1	NCBI: cellular organisms; Bacteria; Terrabacteria group; Actinobacteria; Actinomycetia; Corynebacteriales; Mycobacteriaceae; Mycobacterium; Mycobacterium tuberculosis complex; Mycobacterium tuberculosis;	NCBI: 1773	4368880	99.94	0.00	Yes
NCBI Assembly	GCA_003393175.1	NCBI: cellular organisms; Bacteria; Terrabacteria group; Actinobacteria; Actinomycetia; Corynebacteriales; Mycobacteriaceae; Mycobacterium; Mycobacterium tuberculosis complex; Mycobacterium tuberculosis;	NCBI: 1773	4372354	99.86	0.00	Yes
NCBI Assembly	GCA_003609695.1	NCBI: cellular organisms; Bacteria; Terrabacteria group; Actinobacteria; Actinomycetia; Corynebacteriales; Mycobacteriaceae; Mycobacterium; Mycobacterium marinum;	NCBI: 1781	6579338	99.24	0.23	Yes
NCBI Assembly	GCA_002802385.1	NCBI: cellular organisms; Bacteria; Terrabacteria group; Actinobacteria; Actinomycetia; Corynebacteriales; Mycobacteriaceae; Mycobacteroides; Mycobacteroides abscessus; Mycobacteroides abscessus subsp. abscessus;	NCBI: 1185650	4941900	100.00	0.00	Yes
NCBI Assembly	GCA_004107305.1	NCBI: cellular organisms; Bacteria; Terrabacteria group; Actinobacteria; Actinomycetia; Corynebacteriales; Mycobacteriaceae; Mycobacterium; Mycobacterium tuberculosis complex; Mycobacterium tuberculosis;	NCBI: 1773	4393520	99.94	0.00	Yes
NCBI Assembly	GCA_003392285.1	NCBI: cellular organisms; Bacteria; Terrabacteria group; Actinobacteria; Actinomycetia; Corynebacteriales; Mycobacteriaceae; Mycobacterium; Mycobacterium tuberculosis complex; Mycobacterium tuberculosis;	NCBI: 1773	4348359	99.86	0.50	Yes

NCBI Assembly	GCA_002982285.1	NCBI: cellular organisms; Bacteria; Terrabacteria group; Actinobacteria; Actinomycetia; Corynebacteriales; Mycobacteriaceae; Mycobacterium; Mycobacterium tuberculosis complex; Mycobacterium tuberculosis; Mycobacterium tuberculosis variant bovis;	NCBI: 1765	4306490	98.28	1.21	Yes
NCBI Assembly	GCA_004107895.1	NCBI: cellular organisms; Bacteria; Terrabacteria group; Actinobacteria; Actinomycetia; Corynebacteriales; Mycobacteriaceae; Mycobacterium; Mycobacterium tuberculosis complex; Mycobacterium tuberculosis;	NCBI: 1773	4365216	99.61	0.00	Yes
NCBI Assembly	GCA_004106995.1	NCBI: cellular organisms; Bacteria; Terrabacteria group; Actinobacteria; Actinomycetia; Corynebacteriales; Mycobacteriaceae; Mycobacterium; Mycobacterium tuberculosis complex; Mycobacterium tuberculosis;	NCBI: 1773	4406510	99.94	0.00	Yes
NCBI Assembly	GCA_004112065.1	NCBI: cellular organisms; Bacteria; Terrabacteria group; Actinobacteria; Actinomycetia; Corynebacteriales; Mycobacteriaceae; Mycobacterium; Mycobacterium tuberculosis complex; Mycobacterium tuberculosis;	NCBI: 1773	4381453	99.94	0.00	Yes
NCBI Assembly	GCA_003431645.1	NCBI: cellular organisms; Bacteria; Terrabacteria group; Actinobacteria; Actinomycetia; Corynebacteriales; Mycobacteriaceae; Mycobacterium; Mycobacterium marinum;	NCBI: 1781	6304786	99.88	0.23	Yes
NCBI Assembly	GCA_003392605.1	NCBI: cellular organisms; Bacteria; Terrabacteria group; Actinobacteria; Actinomycetia; Corynebacteriales; Mycobacteriaceae; Mycobacterium; Mycobacterium tuberculosis complex; Mycobacterium tuberculosis;	NCBI: 1773	4406544	99.94	0.50	Yes
NCBI Assembly	GCA_003393225.1	NCBI: cellular organisms; Bacteria; Terrabacteria group; Actinobacteria; Actinomycetia; Corynebacteriales; Mycobacteriaceae; Mycobacterium; Mycobacterium tuberculosis complex; Mycobacterium tuberculosis;	NCBI: 1773	4377374	99.86	0.00	Yes
NCBI Assembly	GCA_003609715.1	NCBI: cellular organisms; Bacteria; Terrabacteria group; Actinobacteria; Actinomycetia; Corynebacteriales; Mycobacteriaceae; Mycobacteroides; Mycobacteroides abscessus; Mycobacteroides abscessus subsp. bolletii;	NCBI: 1091046	5080450	100.00	0.33	Yes
NCBI Assembly	GCA_002946495.1	NCBI: cellular organisms; Bacteria; Terrabacteria group; Actinobacteria; Actinomycetia; Corynebacteriales; Mycobacteriaceae; Mycobacterium; Mycobacterium tuberculosis complex; Mycobacterium tuberculosis;	NCBI: 1773	4399460	99.94	0.00	Yes
NCBI Assembly	GCA_002982335.1	NCBI: cellular organisms; Bacteria; Terrabacteria group; Actinobacteria; Actinomycetia; Corynebacteriales; Mycobacteriaceae; Mycobacterium; Mycobacterium tuberculosis complex; Mycobacterium tuberculosis; Mycobacterium tuberculosis variant africanum;	NCBI: 33894	4352625	99.83	0.00	Yes
NCBI Assembly	GCA_003392305.1	NCBI: cellular organisms; Bacteria; Terrabacteria group; Actinobacteria; Actinomycetia; Corynebacteriales; Mycobacteriaceae; Mycobacterium; Mycobacterium tuberculosis complex; Mycobacterium tuberculosis;	NCBI: 1773	4298609	97.94	0.57	Yes
NCBI Assembly	GCA_003584745.1	NCBI: cellular organisms; Bacteria; Terrabacteria group; Actinobacteria; Actinomycetia; Corynebacteriales; Mycobacteriaceae; Mycobacterium; Mycobacterium pseudoshottsii;	NCBI: 1136880	6061597	99.71	0.73	Yes
NCBI Assembly	GCA_004107345.1	NCBI: cellular organisms; Bacteria; Terrabacteria group; Actinobacteria; Actinomycetia; Corynebacteriales; Mycobacteriaceae; Mycobacterium; Mycobacterium tuberculosis complex; Mycobacterium tuberculosis;	NCBI: 1773	4371992	99.94	0.00	Yes
NCBI Assembly	GCA_004107945.1	NCBI: cellular organisms; Bacteria; Terrabacteria group; Actinobacteria; Actinomycetia; Corynebacteriales; Mycobacteriaceae; Mycobacterium; Mycobacterium tuberculosis complex; Mycobacterium tuberculosis;	NCBI: 1773	4392215	99.61	0.00	Yes
NCBI Assembly	GCA_004107005.1	NCBI: cellular organisms; Bacteria; Terrabacteria group; Actinobacteria; Actinomycetia; Corynebacteriales; Mycobacteriaceae; Mycobacterium; Mycobacterium tuberculosis complex; Mycobacterium tuberculosis;	NCBI: 1773	4395599	99.94	0.00	Yes
NCBI Assembly	GCA_003431655.1	NCBI: cellular organisms; Bacteria; Terrabacteria group; Actinobacteria; Actinomycetia; Corynebacteriales; Mycobacteriaceae; Mycobacterium; Mycobacterium marinum;	NCBI: 1781	6362177	99.88	0.23	Yes
NCBI Assembly	GCA_004112085.1	NCBI: cellular organisms; Bacteria; Terrabacteria group; Actinobacteria; Actinomycetia; Corynebacteriales; Mycobacteriaceae; Mycobacterium; Mycobacterium tuberculosis complex; Mycobacterium tuberculosis;	NCBI: 1773	4394099	99.94	0.00	Yes
NCBI Assembly	GCA_003392615.1	NCBI: cellular organisms; Bacteria; Terrabacteria group; Actinobacteria; Actinomycetia; Corynebacteriales; Mycobacteriaceae; Mycobacterium; Mycobacterium tuberculosis complex; Mycobacterium tuberculosis;	NCBI: 1773	4306065	99.83	0.00	Yes

NCBI Assembly	GCA_003393235.1	NCBI: cellular organisms; Bacteria; Terrabacteria group; Actinobacteria; Actinomycetia; Corynebacteriales; Mycobacteriaceae; Mycobacterium; Mycobacterium tuberculosis complex; Mycobacterium tuberculosis;	NCBI: 1773	4380225	99.86	0.00	Yes
NCBI Assembly	GCA_002983495.1	NCBI: cellular organisms; Bacteria; Terrabacteria group; Actinobacteria; Actinomycetia; Corynebacteriales; Mycobacteriaceae; Mycobacterium; Mycobacterium simiae complex; Mycobacterium shigaense;	NCBI: 722731	5198926	99.17	0.88	Yes
NCBI Assembly	GCA_003614435.1	NCBI: cellular organisms; Bacteria; Terrabacteria group; Actinobacteria; Actinomycetia; Corynebacteriales; Mycobacteriaceae; Mycobacterium; Mycobacterium paragordonae;	NCBI: 1389713	7224251	100.00	0.98	Yes
NCBI Assembly	GCA_002967005.1	NCBI: cellular organisms; Bacteria; Terrabacteria group; Actinobacteria; Actinomycetia; Corynebacteriales; Mycobacteriaceae; Mycobacterium; Mycobacterium talmoniae;	NCBI: 1858794	5125857	81.85	1.21	No
NCBI Assembly	GCA_003392335.1	NCBI: cellular organisms; Bacteria; Terrabacteria group; Actinobacteria; Actinomycetia; Corynebacteriales; Mycobacteriaceae; Mycobacterium; Mycobacterium tuberculosis complex; Mycobacterium tuberculosis;	NCBI: 1773	4356263	99.86	0.00	Yes
NCBI Assembly	GCA_003399115.1	NCBI: cellular organisms; Bacteria; Terrabacteria group; Actinobacteria; Actinomycetia; Corynebacteriales; Mycobacteriaceae; Mycobacterium; Mycobacterium tuberculosis complex; Mycobacterium tuberculosis;	NCBI: 1773	4172341	98.05	1.65	Yes
NCBI Assembly	GCA_004107365.1	NCBI: cellular organisms; Bacteria; Terrabacteria group; Actinobacteria; Actinomycetia; Corynebacteriales; Mycobacteriaceae; Mycobacterium; Mycobacterium tuberculosis complex; Mycobacterium tuberculosis;	NCBI: 1773	4391610	99.94	0.00	Yes
NCBI Assembly	GCA_004107965.1	NCBI: cellular organisms; Bacteria; Terrabacteria group; Actinobacteria; Actinomycetia; Corynebacteriales; Mycobacteriaceae; Mycobacterium; Mycobacterium tuberculosis complex; Mycobacterium tuberculosis;	NCBI: 1773	4398193	99.94	0.00	Yes
[truncated: 1,003,948 more chars]
